# Sex Differences in the Methylome and Transcriptome of the Human Liver and Circulating HDL-Cholesterol Levels

**DOI:** 10.1210/jc.2018-00423

**Published:** 2018-05-28

**Authors:** Sonia García-Calzón, Alexander Perfilyev, Vanessa D de Mello, Jussi Pihlajamäki, Charlotte Ling

**Affiliations:** 1 Epigenetics and Diabetes Unit, Department of Clinical Sciences, Lund University Diabetes Centre, Malmö, Sweden; 2 Institute of Public Health and Clinical Nutrition, University of Eastern Finland, Kuopio, Finland; 3 Clinical Nutrition and Obesity Center, Kuopio University Hospital, Kuopio, Finland

## Abstract

**Context:**

Epigenetics may contribute to sex-specific differences in human liver metabolism.

**Objective:**

To study the impact of sex on DNA methylation and gene expression in human liver.

**Design/Setting:**

Cross-sectional, Kuopio Obesity Surgery Study.

**Participants/Intervention:**

We analyzed DNA methylation with the Infinium HumanMethylation450 BeadChip in liver of an obese population (34 males, 61 females). Females had a higher high-density lipoprotein (HDL)–cholesterol levels compared with males. Gene expression was measured with the HumanHT-12 Expression BeadChip in a subset of 42 participants.

**Results:**

Females displayed higher average methylation in the X-chromosome, whereas males presented higher methylation in autosomes. We found 9455 CpG sites in the X-chromosome and 33,205 sites in autosomes with significant methylation differences in liver between sexes (*q* < 0.05). When comparing our findings with published studies, 95% of the sex-specific differences in liver methylation in the X-chromosome were also found in pancreatic islets and brain, and 26 autosomal sites showed sex-specific methylation differences in the liver as well as in other human tissues. Furthermore, this sex-specific methylation profile in liver was associated with hepatic gene expression changes between males and females. Notably, females showed higher HDL-cholesterol levels, which were associated with higher *KDM6A* expression and epigenetic differences in human liver. Accordingly, silencing of *KDM6A* in cultured liver cells reduced HDL-cholesterol levels and *APOA1* expression, which is a major component of HDL particles.

**Conclusions:**

Human liver has a sex-specific methylation profile in both the X-chromosome and autosomes, which associates with hepatic gene expression changes and HDL-cholesterol. We identified KDM6A as a novel target that regulates HDL-cholesterol levels.

## Teaser

Epigenetics is an important mechanism for sex-specific differences in the transcriptome in the human liver. KDM6A contributes to the differences in HDL-cholesterol between females and males.

It is well known that males and females are metabolically different. For instance, females have been found to be more insulin sensitive and have lower plasma low-density lipoprotein–cholesterol and triglycerides and higher high-density lipoprotein (HDL)–cholesterol levels compared with males (1, 2). In addition, sex has been reported to affect the risk, age at onset, and symptoms of disease (3). It is therefore important to identify molecular mechanisms that contribute to these differences between males and females. Sex disparities are due to not only sex chromosomes and hormones but also differential gene expression, metabolites, or epigenetic patterns (4, 5).

DNA methylation is an epigenetic mechanism essential for development and cell differentiation in mammals (6). It does also play a key role in parental imprinting (7) and X-chromosome inactivation where the extra X-chromosome in females is silenced to achieve dosage compensation (8). Interestingly, sex-specific differences in DNA methylation have been found in human pancreatic islets, brain, and blood using the Infinium HumanMethylation450 BeadChip (Illumina, San Diego, CA) (5, 9–11). However, the impact of sex on the DNA methylation pattern in human liver has not been studied.

Nevertheless, liver metabolism is sex dimorphic (12, 13), and the prevalence of liver diseases is different between males and females. For example, males have a higher risk of developing hepatocellular carcinoma and chronic hepatitis, whereas women have a higher prevalence of acute liver failure, primary biliary cirrhosis, and autoimmune hepatitis (14). Moreover, drug liver metabolism and tolerance to alcohol are also affected by sex (15, 16). Interestingly, sex differences in liver have been found at the transcriptome (17) and proteomic levels (13). A sex-specific DNA methylation pattern has also been observed in rodent liver (18), but it remains to be studied in human liver.

Therefore, our aim was to study the impact of sex on the genome-wide DNA methylation pattern in human liver using the Infinium HumanMethylation450 BeadChip (Illumina) and associate this to sex-specific differences in gene expression and metabolism. In addition, we assessed whether sex-specific methylation differences in human liver also could be found in other human tissues.

## Materials and Methods

### Liver donors

Liver biopsy specimens from 95 Finnish Caucasian patients (36% males) were collected after fasting in the morning of a Roux-en-Y gastric bypass surgery from the Kuopio Obesity Surgery Study (19, 20). The patients were middle age (49.5 ± 7.7 years) and obese [body mass index (BMI), 43.0 ± 5.7], 35% had diabetes, 27% had nonalcoholic steatohepatitis (NASH), and 30.5% were on lipid-lowering medication. More clinical characteristics of these patients according to sex are presented in Table 1. Histological assessment of liver biopsy samples was performed by one pathologist according to the standard criteria (19, 21). Written informed consent was obtained from all participants in accordance with the Declaration of Helsinki, and the study protocol was approved by the Northern Savo Hospital District ethics committee (54/2005, 104/2008, and 27/2010).

**Table 1.** Clinical Characteristics of the 95 Finnish Caucasian Patients From Kuopio Included in the Study

Phenotype	Males (n = 34)	Females (n = 61)	*P* Value^*a*^
Age, y	49.6 ± 7.7	49.4 ± 7.7	0.95
BMI, kg/m^2^	42.4 ± 5.1	43.4 ± 6.0	0.53
fP-glucose, mmol/L	6.8 ± 2.4	6.3 ± 1.9	0.14
fS-insulin, mU/L	35.3 ± 61.2	27.1 ± 71.7	0.077
Total cholesterol, mmol/L	4.1 ± 0.8	4.1 ± 1.1	0.82
LDL-cholesterol, mmol/L	2.4 ± 0.7	2.4 ± 0.9	0.85
HDL-cholesterol, mmol/L	0.9 ± 0.2	1.1 ± 0.2	0.002
Triglycerides, mmol/L	1.7 ± 0.6	1.6 ± 0.7	0.069
Lipid-lowering medication, %	35.3	27.9	0.451
Diabetes, %	50.0	18.0	0.047
Simple steatosis, %	67.6	60.7	0.498
NASH, %	38.2	21.3	0.076

Data are shown as mean ± SD or % as indicated.

Abbreviations: fP, fasting plasma; fS, fasting serum; LDL, low-density lipoprotein.

^a^ Refers to Mann-Whitney test or χ^2^ test for categorical variables.

All blood measurements were done in a single measurement. All were automatized and from reliable methods, and they were repeated only if the values were out of range or strange. Plasma glucose was measured by enzymatic hexokinase photometric assay (Konelab Systems Reagents; Thermo Fischer Scientific, Vantaa, Finland). Serum insulin was determined by immunoassay (ADVIA Centaur Insulin IRI, no 02230141; Siemens Medical Solutions Diagnostics, Tarrytown, NY). Cholesterol and triglycerides from serum and from lipoprotein fractions were assayed by an automated enzymatic method (Roche Diagnostics, Mannheim, Germany) (21).

### Genome-wide DNA methylation profiling of human liver

In total, 500 ng DNA from human liver was bisulfite converted with the EZ DNA methylation kit (Zymo Research, Orange, CA). The Infinium HumanMethylation450 BeadChip assay (Illumina) was used to analyze genome-wide DNA methylation (19, 20). Further details of the analyses of genome-wide DNA methylation are explained in the supplemental material.

### Validation of DNA methylation array results

Pyrosequencing was used to technically validate the DNA methylation results from the Infinium HumanMethylation450 BeadChip (Illumina) in a subset of our cohort. First, DNA from human liver of 36 donors (22 males and 14 females) was bisulfite converted with the EpiTect Bisulfite Kit (Qiagen, Hilden, Germany) and then amplified with the PyroMark PCR kit. Second, pyrosequencing was performed with the PyroMark ID Q96 and PyroMark Gold Q96 reagents (Qiagen), and the PyroMark Q96 2.5.8 software program was used to analyze the DNA methylation data. Predesigned pyrosequencing assays (PCR primers and sequencing primer) (Supplemental Table 1) from Qiagen were used for the two selected CpG sites (cg05688478 annotated to *APLN* and cg27483305 annotated to *NKAP*).

### Genome-wide mRNA expression analysis of human liver

RNA expression was assessed in a subsample of 42 patients because of the limited size of human liver biopsy specimens, available amounts of liver RNA, and resources. Clinical characteristics of these 42 participants are presented in Supplemental Table 2, which shows that this subcohort is a representative sample of the full cohort. RNA was extracted from human liver with the Qiagen miRNeasy minikit, and nucleic acid concentration and purity were determined using the NanoDrop 1000 spectrophotometer (NanoDrop Technologies, Wilmington, DE), whereas RNA quality was determined with the Agilent 2100 bioanalyzer (Agilent Technologies, Santa Clara, CA). For genome-wide mRNA expression analysis, the HumanHT-12 Expression BeadChip array (Illumina) was used, which covers 28,688 coding transcripts.

### Gene set enrichment analysis

Gene set enrichment analysis was performed to expression data using KEGG biological pathways (22). All gene symbols were ranked according to *t* statistics (estimate of the linear model/SEM) comparing males vs females. The analysis was run with highest occurrence for genes with multiple probes, and pathways with 1 to 500 transcripts were considered.

### Silencing of *KDM6A* and *APOL2* in hepatocytes

Huh-7 human hepatocellular carcinoma cells were cultured with DMEM and 1.0 g/L glucose plus 10% fetal bovine serum. Cells were transfected with ON-TARGET plus human small interfering RNA (siRNA) SMART pool (Dharmacon, Lafayette, CO) targeting *KDM6A* (L-014140-01-0005), *APOL2* (L-017407-00-0005), or a negative control (Nontargeting plus D-001810-10-05). siRNA, corresponding to a final concentration of 10 nM, was mixed with Opti-Mem reduced serum media (cat. 31985062; Thermo Fisher Scientific, Waltham, MA) and Lipofectamine RNAiMAX (13778075; Thermo Fisher Scientific) (7.5 μL/well) and incubated for 20 minutes at room temperature. Then, 500 μL siRNA/Lipofectamine was added to cells in 2-mL culture media. Cells were harvested 48 hours after transfection. RNA was extracted with the RNeasy Plus mini kit (Qiagen) and converted to cDNA with the QuantiTect Reverse Transcription kit (Qiagen). Knockdown was confirmed by quantitative PCR with predesigned TaqMan Gene Expression assays (Thermo Fisher Scientific): Hs00253500_m1 (*KDM6A*) and Hs01935263_s1 (*APOL2*) run in triplicates. mRNA expression for genes associated with HDL-cholesterol synthesis was also measured with quantitative PCR with predesigned TaqMan Gene Expression assays (Thermo Fisher Scientific): Hs00163641_m1 (*APOA1*), Hs01059137_m1 (*ABCA1*), and Hs00165106_m1 (*LIPC*). Expression levels were normalized to *PPIA* (4326316E-0901011; Thermo Fisher Scientific) and quantified using the ∆∆Ct method. HDL-cholesterol levels were measured in the cell lysate with the commercial HDL and LDL/VLDL Cholesterol Assay kit (ab6539; Abcam, Cambridge, United Kingdom) following the fluorometric method.

### Statistical analysis

To assess differences in DNA methylation and mRNA expression in the liver between males and females, a linear regression model was performed, including sex, age, BMI, degree of steatosis, and presence of type 2 diabetes and NASH as covariates. Variance inflation factors were calculated to evaluate multicollinearity of the studied phenotypes. A false discovery rate (FDR) was used to account for multiple testing, and a *q* value <0.05 (FDR <5%) was considered significant. Mann-Whitney tests were used when comparing means of clinical characteristics and DNA methylation. Statistical analyses were done using R statistical software (v.3.3.2; R Foundation for Statistical Computing, Vienna, Austria).

## Results

### Differential DNA methylation pattern between males and females in human liver

Genome-wide DNA methylation was analyzed in liver of 34 males and 61 females. Table 1 shows their clinical characteristics. Females had higher HDL levels compared with males.

**Figure 1.**
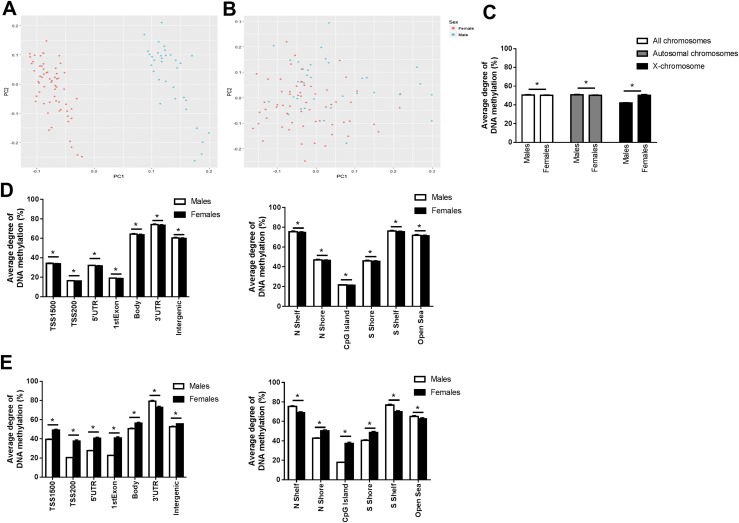
Impact of sex on DNA methylation in human liver from 34 male and 61 female donors. (A) PC analysis including genome-wide liver DNA methylation data of 466,246 sites after batch correction, quality control, and removing Y-chromosome data. (B) PC analysis including genome-wide liver DNA methylation data of only autosomes, including 455,526 sites after batch correction, quality control, and removing X- and Y-chromosome data. (C) Average degree of liver DNA methylation including DNA methylation on the autosomal chromosomes and the X-chromosome but excluding DNA methylation data on the Y-chromosome. (D) Average degree of liver DNA methylation levels of autosomal chromosomes for different functional genomic annotation and CpG island regions. (E) Average degree of liver DNA methylation levels of the X-chromosome in gene regions and CpG island regions. The 2-kb sequences, directly upstream and downstream of CpG islands are called the northern and southern shore (N shore, S shore), respectively. The 2-kb sequences directly adjacent to the shores are called the northern and southern shelves (N shelf, S shelf). DNA methylation sites outside the CpG island regions are annotated as “open sea.” Data are presented as mean and standard deviation, and all asterisks indicate FDR <0.05.

DNA methylation of 455,526 CpG sites located on autosomes and 10,720 located on the X-chromosome were included for the analyses. To elucidate if genome-wide DNA methylation data in human liver cluster by sex, a principal component (PC) analysis was carried out (Fig. 1A and 1B). The methylation data clearly clustered by sex when including data on both autosomes and X-chromosome (Fig. 1A). Moreover, sex correlated significantly with the top two PCs (*P* < 0.0001). However, when removing the X-chromosome methylation data, no clustering by sex was observed (Fig. 1B). Now, sex correlated significantly with PC2 and PC4, among the top five PCs (*P* < 0.007). Then, we evaluated if the average degree of DNA methylation was different between both sexes (Fig. 1C). Men displayed a small but significantly higher degree of methylation compared with women when analyzing DNA methylation in all chromosomes (males 50.5% ± 0.6% vs females 50.2% ± 0.6%, *P* = 0.011) and only in autosomes (males 50.7% ± 0.6% vs females 50.2% ± 0.6%, *P* = 1.2 × 10^−4^). However, when assessing the average degree of DNA methylation only in X-chromosome sites, females had significantly higher DNA methylation than males (males 41.9% ± 0.6% vs females 50.2% ± 1.1%, *P* = 2.5 × 10^−15^).

We further investigated the effect of sex on the average level of DNA methylation in human liver based on either the functional genome distribution [transcriptional start site (TSS) 1500, TSS200, 5′-untranslated region (5′-UTR), first exon, gene body, 3′-UTR or intergenic regions] or in relation to the CpG content (northern shelf, northern shore, CpG island, southern shore, southern shelf, or open sea regions) provided by the array annotation, in autosomes (Fig. 1D) and X-chromosome (Fig. 1E) separately. For the autosomes, males exhibited higher methylation compared with females in all genomic and CpG regions with FDR <5% (*q* < 0.05). For the X-chromosome, males had significantly lower DNA methylation levels than females in all regions except the 3′-UTR (*q* < 0.05). Moreover, although CpG islands and shores, which presented lower and intermediate methylation levels (17% to 50%), had lower average methylation levels in males compared with females, shelves and the open sea, which were hypermethylated (63% to 77%), had higher average DNA methylation levels in males than in females (*q* < 0.05).

### DNA methylation of individual CpG sites in human liver is affected by sex

**Figure 2.**
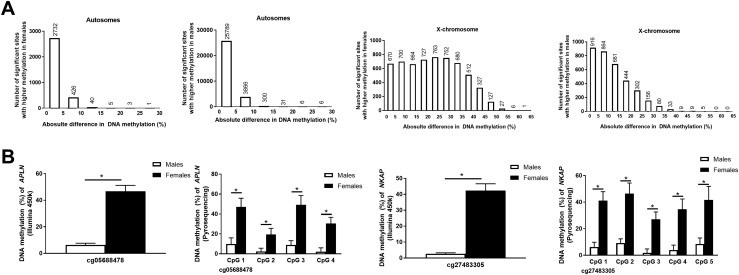
CpG sites with differential DNA methylation between males and females in human liver. (A) Absolute difference in DNA methylation of CpG sites with significantly (FDR <0.05) higher methylation levels in females than in males or higher methylation in males than in females in autosomes and the X-chromosome. (B) Technical validation of the Infinium HumanMethylation450 BeadChip data using pyrosequencing. We selected a CpG site (cg05688478) and surrounding CpG sites in the *APLN* gene, as well as a CpG site (cg27483305) and surrounding CpG sites in the *NKAP* gene. All CpG sites analyzed by pyrosequencing show differential DNA methylation between males and females in human liver (*P* < 0.00001). Selected CpG sites show sex-specific differences using both Infinium HumanMethylation450 BeadChip (*q* <0.05) and pyrosequencing. Data are represented by mean and standard deviation, and *P* values for pyrosequencing were calculated with the Mann-Whitney test. *P < 0.00001 when analyzing DNA methylation with pyrosequencing; *q < 0.05 when analyzing DNA methylation with the Illumina 450K array.

We next evaluated if sex also influences the level of DNA methylation of individual CpG sites in human liver for autosomes and X-chromosomes separately. In the autosomes, we identified 33,205 CpG sites that were differentially methylated between males and females (*q* < 0.05) (Supplemental Table 3). Most of them, 29,998 CpG sites, exhibited a higher degree of methylation in males than females (Fig. 2A). To increase the biological significance of our findings, we further filtered out the data, including significant CpG sites (*q* < 0.05) with absolute methylation differences >5% between males and females (5, 23, 24). We found 4684 autosomal CpG sites in human liver that had absolute differences in methylation >5% in males vs females. These sites had a fold change (DNA methylation in males/DNA methylation in females) ranging from 0.21 to 3.39. Out of these 4684 CpGs, 475 sites had higher methylation in females corresponding to 255 individual genes, and 4209 sites had higher methylation in males corresponding to 2016 individual genes (Supplemental Table 4). To test if the differentially methylated autosomal CpG sites between sexes contain androgen response elements, we used the coordinates of androgen response binding sites from the JASPAR database (25) using Easeq version 1.05 (26). Only 1 CpG site (cg00880562) out of the 33,205 autosomal CpG sites differentially methylated between females and males in the liver contained androgen response elements, suggesting that autosomal sex differences in DNA methylation in the liver may potentially be independent of testosterone.

Regarding the X-chromosome, 9455 CpG sites (corresponding to 88.2% of the sites analyzed on the X-chromosome) exhibited differential methylation between males and females (*q* < 0.05) (Supplemental Table 5), of which 5956 sites had higher methylation in females and 3499 sites were higher in males (Fig. 2A). Among these, 7869 CpG sites were found to have absolute differences in methylation >5% between the sexes (Supplemental Table 6). The fold change (DNA methylation in males/DNA methylation in females) of these sites ranged between 0.03 and 4.47, and females exhibited 5286 sites with higher methylation corresponding to 578 genes, whereas males had 2583 sites corresponding to 696 individual genes. Notably, among the genes located on the X-chromosome, 450 genes had some annotated CpG sites with higher methylation in both females and males.

The distribution of the significant CpG sites (*q* < 0.05 and absolute methylation differences >5%) according to sex, compared with all analyzed sites on the Illumina 450K array, is displayed in Supplemental Fig. 1. The distribution is shown based on functional genomic distribution or CpG content for autosomes and X-chromosome separately. CpG sites with differential methylation in the autosomes based on sex were enriched within the gene body, 3′-UTR, and intergenic regions. On the other hand, for the X-chromosome, we found that the significant CpG sites were enriched in the TSS200, 5′-UTR, and first exon. In relation to the CpG content and for the autosomes, the open sea and the southern shelf had the highest proportion of differentially methylated sites, and for the X-chromosome, the CpG island region had the highest proportion of significant CpG sites between males and females.

### Technical validation of DNA methylation in human liver

Two CpG sites that exhibited large differences in DNA methylation between males and females (∼40%) were selected for technical validation. These CpG sites were cg05688478 annotated to *APLN* and cg27483305 annotated to *NKAP* (Fig. 2B). These two genes have been previously shown not to escape X-chromosome inactivation (8), and sex differences in methylation of these two genes have been reported in other human tissues (5, 10). In agreement with the array, both sites showed differential DNA methylation in males compared with females using pyrosequencing with a similar difference between sexes (Fig. 2B). Notably, differences in DNA methylation between both sexes were also found in all adjacent CpG sites only covered by the pyrosequencing assays and not by the array.

### Overlapping between DNA methylation affected by sex and enrichment of histone modifications and enhancer elements in the human liver

To further integrate our DNA methylation data with regulatory regions and other epigenetic factors, we intersected the position of the 33,205 autosomal significant CpG sites and 9455 CpGs in the X-chromosome based on sex (*q* < 0.05) with published chromatin immunoprecipitation–sequencing data of histone modifications in human liver from the Roadmap Epigenomics Consortium (27) using Easeq version 1.05 (26). We included in the analysis chromatin immunoprecipitation–sequencing data of H3K4me1 and H3K27me3 that were available for the same four liver donors. Notably, 42% of the autosomal CpG sites (13,817 CpGs) and 27% of the X-chromosome sites (2601 CpGs) differentially methylated by sex overlapped with histone marks related to active chromatin and enhancer regions (H3K4me1), whereas 14% of the autosomal sites (4760 CpGs) and 11% of the X-chromosome sites overlapped with histone marks related to heterochromatin (H3K27me3). A permutation distribution test based on 10,000 permutations showed more autosomal and X-chromosome significant CpG sites due to sex overlapping with H3K4me1 and only more autosomal significant CpG sites due to sex overlapping with H3K27me3, compared with what it would have been expected by chance if all the sites in the array were analyzed (*P* < 0.05). The latter indicates an enrichment of histone modifications and enhancer elements at active chromatin regions in both autosomal and X-chromosome CpG sites differentially methylated by sex in the human liver.

### Differential mRNA expression in human liver between males and females

We also studied the impact of sex on gene expression in human liver. Gene set enrichment analysis generated significant gene sets with upregulated expression in liver from males compared with females (*q* < 0.05; Supplemental Table 7), including pathways involved in oxidative phosphorylation, the proteasome and the ribosome. No significant gene sets were yielded with upregulated expression in females compared with males (*q* > 0.05).

We next evaluated if the expression of individual genes was different in liver between males and females. Fifteen individual genes, 11 genes in the autosomes and 4 genes in the X-chromosome, were differentially expressed based on sex after FDR correction (*q* < 0.05) (Table 2). Eight of these genes were upregulated in males and seven genes were upregulated in females. Moreover, 844 individual genes, 795 genes in the autosomes and 49 genes in the X-chromosome, were differentially expressed between males and females with a nominal *P* value <0.05 without adjustment for multiple comparisons (Supplemental Table 8).

**Table 2.** Fifteen Genes Differentially Expressed Between Males and Females in Human Liver After FDR Correction (*q* < 0.05)

Symbol	Gene Name	Chromosome	mRNA Expression, Females	mRNA Expression, Males	Fold Change (Females/Males)	*P* Value	*q* Value
***XIST***	X inactive specific transcript (nonprotein coding)	X	8.32 ± 1.22	5.03 ± 1.05	1.66	1.71E-11	6.89E-08
***KDM6A***	Lysine (K)–specific demethylase 6A	X	5.51 ± 0.46	4.84 ± 0.25	1.14	3.04E-06	4.59E-03
***ARSE***	Arylsulfatase E (chondrodysplasia punctata 1)	X	6.86 ± 0.39	6.12 ± 0.53	1.12	2.74E-05	1.95E-02
***RPS4X***	Ribosomal protein S4, X-linked	X	9.64 ± 0.22	9.14 ± 0.22	1.05	7.55E-07	1.52E-03
***VWCE***	Von Willebrand factor C and EGF domains	11	4.78 ± 0.97	6.08 ± 0.57	0.79	4.04E-06	5.42E-03
***DGCR5***	DiGeorge syndrome critical region gene 5 (nonprotein coding)	22	5.17 ± 0.53	5.91 ± 0.53	0.88	6.03E-05	3.46E-02
*APOL2*	Apolipoprotein L, 2	22	5.75 ± 0.36	6.30 ± 0.38	0.91	1.63E-05	1.51E-02
***PITPNM1***	Phosphatidylinositol transfer protein, membrane-associated 1	11	4.81 ± 0.26	5.26 ± 0.29	0.92	2.15E-05	1.73E-02
*SDSL*	Serine dehydratase-like	12	7.53 ± 0.45	8.09 ± 0.36	0.93	5.18E-05	3.13E-02
*FAM210B*	Family with sequence similarity 210, member B	20	7.91 ± 0.29	8.36 ± 0.24	0.95	1.37E-05	1.50E-02
***SULT1A1***	Sulfotransferase family, cytosolic, 1A, phenol-preferring, member 1	16	8.72 ± 0.30	9.19 ± 0.40	0.95	9.07E-05	4.97E-02
***TTC39C***	Tetratricopeptide repeat domain 39C	18	8.73 ± 0.29	9.19 ± 0.25	0.95	2.45E-05	1.85E-02
*PKD2*	Polycystic kidney disease 2 (autosomal dominant)	4	5.11 ± 0.42	4.63 ± 0.44	1.10	4.56E-05	2.90E-02
***H19***	Imprinted maternally expressed transcript (nonprotein coding)	11	10.41 ± 0.87	8.87 ± 1.00	1.17	1.02E-06	1.75E-03
***PZP***	Pregnancy-zone protein	12	9.97 ± 1.37	7.82 ± 1.73	1.27	1.62E-05	1.51E-02

Means ± SDs are shown. Genes in bold also have differential DNA methylation between males and females (*q* < 0.05).

Abbreviation: EGF, epidermal growth factor.

### Overlap between methylation and expression in human liver

**Figure 3.**
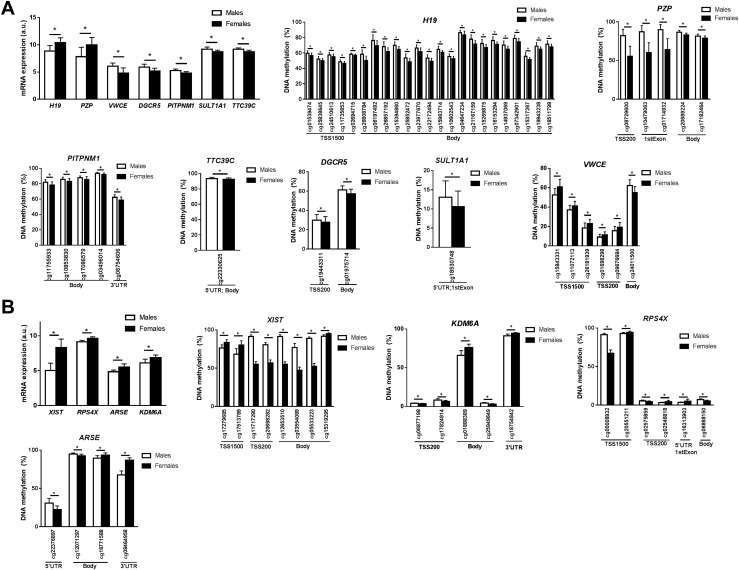
Genes with both differential mRNA expression (*q* < 0.05) and DNA methylation (*q* < 0.05) based on sex in (A) autosomes and (B) the X-chromosome. *q < 0.05.

Given that methylation may affect gene expression, we examined if any of the genes differentially methylated by sex in the autosomes (Supplemental Table 3) and X-chromosome (Supplemental Table 5) also exhibit differential mRNA expression based on sex in human liver. We found that seven genes on the autosomal chromosomes (Fig. 3A) and four genes on the X-chromosome (Fig. 3B) showed different levels of methylation (*q* < 0.05) and expression (*q* < 0.05) between males and females in human liver. Two genes in the autosomes had higher expression and lower methylation in females than in males: *H19*, an imprinted gene and a long noncoding RNA probably acting as a tumor suppressor (28, 29), and *PZP*, encoding a pregnancy-zone protein that is able to inhibit proteinases and shows higher circulating levels in females (30). Four genes in the autosomes had higher expression and higher methylation in males than in females: *PITPNM1*, which is necessary for maintaining the normal structure of the endoplasmic reticulum and the Golgi apparatus (31); *TTC39C*, which is involved in protein-protein interaction (32); *DGCR5*, which encodes a long noncoding RNA that has been reported as a biomarker for hepatocellular carcinoma (33); and *SULT1A1*, which encodes the most abundant sulfotransferase enzyme in liver, which catalyzes the sulfonation of many hormones and compounds (34). The last autosomal gene with differential methylation and expression between males and females is *VWCE*, which regulates stem cell pluripotency and cell fate decisions during development (35). Regarding the X-chromosome, all four genes had higher expression in liver from females compared with males and presented differential DNA methylation in males vs females. These genes encode proteins involved in X-inactivation (*XIST*) (36), demethylation of histones (*KDM6A*) (37, 38), protein synthesis (*RPS4X*) (39), and composition of bone and cartilage matrix that escape X-inactivation (*ARSE*) (40).

Moreover, 369 genes in the autosomes and 36 genes in the X-chromosome were both differentially methylated due to sex (*q* < 0.05) and differentially expressed between males and females with a nominal *P* value <0.05 without adjustment for multiple comparisons instead of a *q* value <0.05 (Supplemental Table 9). Thirty-six of these 369 autosomal genes encode proteins involved in metabolic pathways (adjusted *P* = 1.9 × 10^−11^) based on KEGG pathway analysis using WebGestalt (41). There was an enrichment of other seven pathways (adjusted *P* < 0.05), including insulin signaling pathway, galactose metabolism, neurotrophin signaling pathway, extracellular matrix-receptor interaction, and focal adhesion, among others.

### Correlations between liver gene expression and HDL-cholesterol serum levels

**Figure 4.**
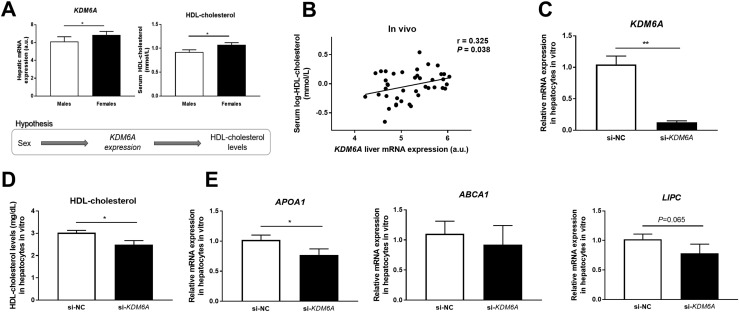
Impact of *KDM6A* on HDL-cholesterol levels *in vivo* and *in vitro*. (A) Association between sex, *KDM6A* mRNA expression in the liver, and serum HDL-cholesterol levels. (B) mRNA expression of *KDM6A* in human liver correlated positively with serum HDL-cholesterol levels in 42 patients. (C) Transfection of hepatocytes cultured *in vitro* with siRNA targeting *KDM6A* (si-*KDM6A*) resulted in decreased *KDM6A* mRNA expression compared with hepatocytes transfected with negative control siRNA (si-NC). (D) Decreased HDL-cholesterol levels in hepatocytes cultured *in vitro* deficient for *KDM6A* expression (si-*KDM6A*) compared with control cells transfected with negative control (si-NC). (E) mRNA expression of genes encoding proteins that contribute to HDL-cholesterol levels in hepatocytes cultured *in vitro* deficient for *KDM6A* expression (si-*KDM6A*) compared with control cells transfected with negative control (si-NC). Duplicate wells of cells from four different cell passages were used for all the *in vitro* analyses (n = 8). Data are presented as means ± SEs, and *P* values have been calculated using the Mann-Whitney test. *P < 0.05. **P < 0.001.

HDL-cholesterol levels are higher in females (Table 1), and we wondered if gene expression of the genes differentially expressed by sex presented in Fig. 3 and Table 2 could affect serum HDL levels. Our hypothesis was that the expression of these genes (*i.e.*, *KDM6A*) could potentially mediate the association between sex and HDL-cholesterol levels (Fig. 4A). Indeed, higher *KDM6A* expression was associated with higher HDL-cholesterol levels (Fig. 4B), whereas higher expression of *APOL2*, *PITPNM1*, and *SULT1A1* was associated with lower HDL-cholesterol levels (Supplemental Fig. 2) using Pearson correlations. However, we are aware that these associations could be confounded by sex.

Therefore, to elucidate if altered expression of some of these genes may directly affect HDL-cholesterol levels, we decided to silence two genes in hepatocytes cultured *in vitro* that showed the strongest association with HDL levels *in vivo*, *KDM6A* and *APOL2* (Fig. 4B and Supplemental Fig. 2). Silencing expression using siRNAs resulted in 87% reduction in mRNA levels in the liver cells (Fig. 4C). Interestingly, silencing of *KDM6A* reduced HDL-cholesterol levels in the cell lysate, supporting that *KDM6A* affects HDL levels (Fig. 4D). However, no differences in HDL-cholesterol levels were observed in hepatocytes deficient for *APOL2* expression (data not shown). *KDM6A* encodes a lysine demethylase that catalyzes demethylation of trimethylated/dimethylated histone H3, and altered expression of this gene may hence affect HDL levels by regulation of other genes. We therefore proceeded to analyze expression of important genes contributing to HDL-cholesterol levels (*e.g.*, *APOA1* encoding apolipoprotein A1, *ABCA1* encoding cholesterol efflux regulatory protein, and *LIPC* encoding hepatic triglyceride lipase) in hepatocytes deficient for *KDM6A*. Interestingly, silencing *KDM6A* resulted in lower expression levels of *APOA1* and *LIPC* (Fig. 4E).

### Comparison of sex-associated DNA methylation differences in liver with other studies assessing sex differences in methylation in pancreatic islets, brain, cord blood, and whole blood

We proceeded to test if any of our sex-associated differences in DNA methylation in human liver (FDR <0.05) have been previously identified in other human tissues such as pancreatic islets (FDR <0.05, absolute difference >5%) (5), brain (FDR <0.05) (10), cord blood (FDR <0.05) (11), and whole blood (Bonferroni <0.05) (9), based on published studies that also used the Illumina 450K array (Supplemental Table 10A). We looked for the overlap between FDR significant data in our study with what was considered significant in the other studies.

First, we compared the identified sex differences in liver DNA methylation with DNA methylation data in pancreatic islets from 87 Caucasian donors, including 61% males (5). Hall *et al.* (5) found 470 significant sites on the autosomes (FDR <0.05) with absolute methylation differences >5% in pancreatic islets between males and females, and among these, we also found 183 CpGs (38.9%) with sex differences in human liver, with all showing differences in the same direction (Supplemental Table 10B). With regard to the X-chromosome, 8140 sites were significant in islets (FDR <0.05) with an absolute methylation difference >5% between males and females, and we could replicate most of them, that is, 7620 CpG sites (93.6%) in the liver.

We then compared our findings in the liver with published DNA methylation data in human prefrontal cortex from 46 Caucasian adults, including 70% males, which identified 614 autosomal sites and 7743 sites on the X-chromosome to be different based on sex (10). In total, 220 autosomal sites (35.8%) (Supplemental Table 10C) and 7316 sites (94.5%) on the X-chromosome had differential DNA methylation in both the liver and the brain.

Two other studies have analyzed sex differences in the genome-wide DNA methylation pattern in autosomes in blood: one study assessed cord blood from 111 Mexican American newborns (48% males) and the other whole blood of 1799 patients (49% males) (9, 11). We found that 637 CpG sites out of 3031 significant autosomal sites found in cord blood (FDR <0.05) were also significant in our liver data with FDR<0.05 (21.0%) (Supplemental Table 10D). Moreover, we found that 1498 (13.6%) of the 11,010 significant CpG sites in the autosomes found in whole blood (Bonferroni <0.05) were also significant in our liver data (FDR <0.05) (Supplemental Table 10E). In addition, Singman *et al.* (9) replicated their results in whole blood in three other cohorts, which revealed 1184 CpG sites (Bonferroni <0.05). Among these, we were able to replicate sex differences in methylation in 189 CpG sites (16%) in human liver.

**Figure 5.**
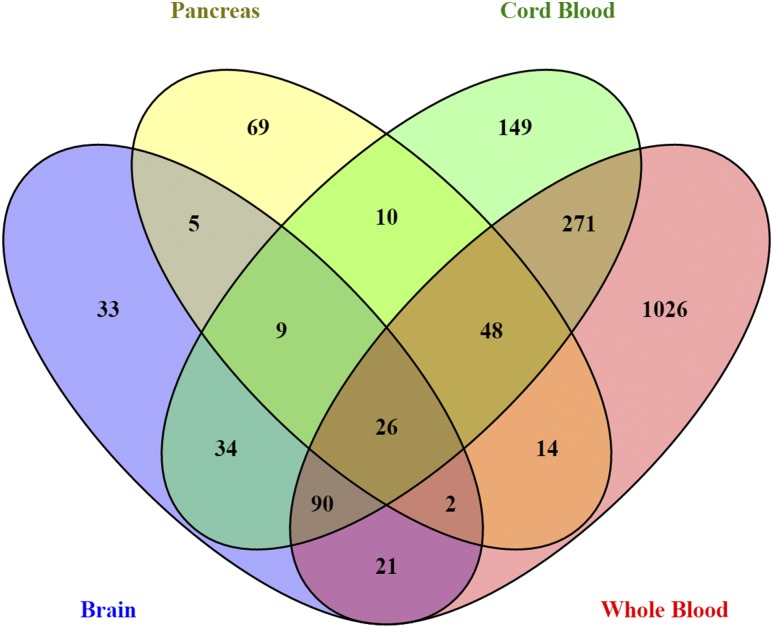
Venn diagram showing the overlap of autosomal CpG sites with sex-specific differences in DNA methylation in human liver and across four other human tissues. There are 26 common CpG sites in autosomes whose methylation is different due to sex in all studied human tissues, including liver, pancreas, brain, cord blood, and whole blood.

Taking into account sex differences in methylation in the autosomes in all four published studies (Supplemental Table 10A), we could replicate 1807 autosomal CpG sites in human liver (Fig. 5). Notably, 26 CpG sites were differentially methylated between males and females in liver, pancreatic islets, prefrontal cortex, cord blood, and whole blood (Fig. 5 and Supplemental Table 11). Among these 26 CpGs, 17 had higher methylation in males, and 9 showed higher methylation in females in all five tissues. Interestingly, we found that out of these 26 CpGs, there were 4 consecutive CpG sites in a CpG island in an intergenic region of chromosome 8, where males had higher methylation than females.

## Discussion

To our knowledge, this is the first epigenome-wide association study to provide a detailed map of differences in the human methylome in liver between males and females. We identified sex differences in DNA methylation in the X-chromosome and in autosomes, both chromosome-wide and in individual CpG sites. Notably, some of the autosomal sites with methylation sex differences in human liver were also differentially methylated between males and females in other human tissues (5, 9–11). Furthermore, we found sex differences in gene expression, and some of these genes were also differentially methylated between males and females. In addition, females had higher HDL-cholesterol levels, which were associated with higher *KDM6A* gene expression. In line, silencing *KDM6A* reduced HDL-cholesterol levels and *APOA1* gene expression—the major component of HDL particles—in hepatocytes cultured *in vitro*. All these data support a role for DNA methylation in liver sexual dimorphism.

It is well known that DNA methylation contributes to X-chromosome inactivation in females (8). In accordance, we found a higher average degree of X-chromosome methylation in females than in males with an absolute difference in methylation of 8.3%, which is in agreement with other published studies (5, 10). However, 37% of the significant sites on the X-chromosome (*q* < 0.05) had higher methylation in males, who also had higher average DNA methylation levels in the 3′-UTR gene region as well as in the shelves of CpG islands and in the open sea, which is in line with another previous study in pancreatic islets (5). Interestingly, most of the genes on the X-chromosome had annotated CpG sites with higher methylation in both females and males (5). Moreover, around 95% of the CpG sites in the X-chromosome that had differential DNA methylation in human liver between sexes also had different methylation levels between males and females in pancreatic islets and brain (5, 10), independently of the clinical characteristics of the population. These data demonstrate that DNA methylation in the X-chromosome in human liver mirrors the methylome in other human tissues.

In addition, we identified four genes on the X-chromosome with large differences in DNA methylation between males and females, and these genes were also more expressed in liver from females than males: *XIST*, *ARSE*, *RPS4X*, and *KDM6A.* Higher *ARSE* and *RPS4X* mRNA expression has also been found in pancreatic islets, and higher *XIST* and *RPS4X* mRNA expression was also found in brain from females compared with males (5, 10). These differences in gene expression in several tissues may explain some metabolic differences between males and females. All these four genes are known to escape X-chromosome inactivation (36–40). Of note, ∼25% of human genes escape X-chromosome inactivation, and they may do it tissue-specifically (8). Interestingly, a recent study suggested that this biallelic expression of X-chromosome genes could explain reduced cancer incidence in females compared with males across various tumor types (42). In particular, they found that *KDM6A*, a histone demethylase already described as a tumor suppressor gene, was more frequently mutated across all male cancers (43, 44). Because we found a higher expression of this gene in females’ liver and considering that males have an increased risk for hepatic cancer (14), *KDM6A* could possibly be involved in hepatic cancer protection in females. We also found that serum HDL-cholesterol levels were positively associated with *KDM6A* mRNA expression in human liver in addition to higher serum HDL-cholesterol levels and higher *KDM6A* expression in females. Interestingly, silencing *KDM6A* in hepatocytes resulted in lower HDL-cholesterol levels and lower expression of key genes encoding proteins that regulate HDL-cholesterol levels, supporting the direct contribution of KDM6A in the differences found in HDL-cholesterol levels between males and females. Although it is well established that females have higher HDL-cholesterol levels (1, 2) and some studies have found *KDM6A* to be more highly expressed in females in the liver and other human tissues (5, 45), to our knowledge, this is the first time that an association between liver *KDM6A* expression and HDL-cholesterol has been reported. *KDM6A* mediates demethylation of histone 3 trimethylated lysine 27 (H3K27me3), which results in activation of transcription of target genes (37, 46, 47).

We found a small but higher average degree of DNA methylation in autosomes in liver from males than females, which is in agreement with published data (48). Males exhibited higher methylation in liver compared with females in all autosomal genomic and CpG regions, contrary to what has been seen in pancreatic islets (5). The mechanisms involved in this sex difference remain unknown, but sex hormones are not considered major determinants in the regulation of global methylation (49). Moreover, there are 26 common CpG sites differentially methylated due to sex in the liver and also in the pancreas, brain, and blood (5, 9–11). Most of these sites are located in intergenic regions, for example, four consecutive CpG sites located in a CpG island with higher methylation in males compared with females. Given that DNA methylation in the intergenic region has been shown to maintain genomic integrity, may regulate transcription of distal genes, and can be located in enhancer regions (50), sex differences in DNA methylation of these CpG sites could affect gene expression. For example, we found that methylation in the *NAB1* gene was higher in males than in females in all investigated human tissues, and this gene had lower expression in liver from males compared with females. *NAB1* is known to act as a transcriptional repressor for zinc finger transcription factors *EGR1* and *EGR2*, which are involved in many biological processes, such as regulation of growth and differentiation (51). Thus, differential DNA methylation and gene expression of *NAB1* might affect metabolic pathways and could contribute to sex differences in liver metabolism.

Although we showed epigenetic similarities in different human tissues that may contribute to sex dimorphism, we also found liver-specific sex differences in DNA methylation in most autosomal CpG sites (31,398 sites), suggesting that DNA methylation in liver may contribute to tissue-specific differences between sexes. For example, one of the top genes was *PZP*, pregnancy-zone protein gene, which had higher DNA methylation in the promoter and first exon region and lower gene expression in liver from males. *PZP* is known to inhibit proteinases and is upregulated in inflammatory conditions where it is believed to exert cellular-protective effects (52). Most plasma *PZP* is synthesized by the liver (53), and in agreement with our results, other studies have found higher serum levels of this protein in females (30). *H19* is another gene that is highly expressed in females and highly methylated in males. It is a paternally imprinted gene that suppresses hepatocellular carcinoma (28, 29). Given that females have a lower risk of developing liver cancer (14) and that in this study, *H19* had a higher expression in females, *H19* could potentially suppress hepatic tumors in females. *VWCE* shows a higher expression and a lower promoter methylation in liver from males. It has been described as an oncogene that stimulates hepatocellular growth and survival (35), which agrees with the fact that males have a higher risk of developing liver cancer (14). Overall, sex differences in autosomal DNA methylation are associated with expression of several genes, which may give rise to sex differences in liver metabolism and in the prevalence of hepatic diseases.

We observed some overlap between sex differences in DNA methylation and gene expression in autosomes and in the X-chromosome. The association between DNA methylation and gene expression is quite complex. The impact of methylation on expression varies with genomic context, and methylation regulates not only mRNA expression but also other processes such as alternative splicing, genomic stability, or other epigenetic factors (54, 55). Indeed, we observed an enrichment of histone marks in our CpG sites differentially methylated by sex. Moreover, DNA methylation is a quite stable epigenetic mark, and gene expression is a more dynamic process that could change to adapt to the metabolism and different biological processes. Then, even if methylation has a modest effect on expression at a certain time point, it might still regulate gene expression under different physiological conditions and also play an important role for sex-specific traits in hepatic phenotype, physiology, and pathology. It should also be noticed that for the gene expression data, we could have lower statistical power because less people are included compared with the DNA methylation analyses.

To our knowledge, this is the first study to show genome-wide DNA methylation sex differences in human liver. Others strengths of this study include a large cohort of liver biopsy specimens, analysis of genome-wide DNA methylation and gene expression, technical validation of the results, use of multiple-adjusted models to minimize potential confounders that could affect the main findings and functional studies to further understand the *in vivo* data. However, this study also has few limitations. First, the HumanMethylation450 BeadChip array includes nonspecific probes that could cross-react with other sites of the genome, making more difficult to study sex differences in autosomal probes that could cross-react with X-chromosome probes. To solve this problem, we filtered away 14,548 probes to be cross-reactive with 49 and 50 base pairs. Second, DNA methylation of 2800 (8.4%) autosomal and 256 (2.7%) X-chromosome CpG sites associated with sex were also associated with NASH (20). NASH was included in the regression model as a covariate, but to make sure that we have separated the effect of sex from NASH on DNA methylation, we analyzed the residuals of some CpG sites without including sex in the model (age, BMI, degree of steatosis, presence of type 2 diabetes, and NASH). The residuals did not depend on NASH (*P* > 0.05), indicating that the model has been well corrected for NASH (data not shown). The effect of sex on DNA methylation was also independent of diabetes status because only DNA methylation of 39 autosomal and 3 X-chromosome CpG sites associated with sex were also associated with diabetes. In accordance, when we analyzed the residuals the same way as for NASH, they did not depend on diabetes (*P* > 0.05) (data not shown). Third, the published studies we considered to evaluate the consistency of our results in other human tissues used the same DNA methylation analysis platform as our study, but there are some differences in the clinical characteristics of the populations across all studies. For example, our study involved patients who are obese, whereas the pancreatic islet study included nonobese individuals and the whole-blood study comprised a very heterogeneous population from different cohorts. However, despite these differences, we still managed to detect an important overlap in sex-specific DNA methylation pattern across tissues. Fourth, we cannot completely rule out that sex hormones could play a role in the sex-specific DNA methylation differences we have observed in the liver. However, we have seen that no differentially methylated autosomal CpG sites between sexes, except one, contained androgen response elements, indicating that DNA methylation due to sex in the liver might be independent of testosterone. Moreover, sex hormones have been shown not to influence DNA methylation in human leukocytes or in mice liver (49, 56). Notably, Mayne *et al.* (45) reported that two-thirds of autosomal genes that were sex biased in the human liver were not under direct influence of sex hormones, which suggests there are other mechanisms driving hepatic sex differences, such as epigenetic factors. More studies are needed to properly address this matter. Fifth, our study includes only patients who are obese, and we cannot rule out that some differences may occur in the liver of normal-weight participants. Nevertheless, the fact that numerous sex-specific methylation differences also occur in other tissues (*e.g.*, pancreatic islets) of normal-weight people support a general sex-specific effect on DNA methylation independent of BMI. Moreover, we have adjusted for BMI in all statistical models.

Overall, we demonstrate a different genome-wide DNA methylation pattern in autosomes and X-chromosome between males and females in human liver. Sex-specific differences in liver expression and methylation of *KDM6A* may contribute to higher HDL-cholesterol levels in females. Our data support an important function of DNA methylation in liver sex dimorphism that could play a role in sex difference hepatic physiology and pathophysiology.

## Supplementary Material

Supplemental Data

## Abbreviations:

BMI: body mass index
FDR: false discovery rate
HDL: high-density lipoprotein
NASH: nonalcoholic steatohepatitis
PC: principal component
siRNA: small interfering RNA
TSS: transcriptional start site
UTR: untranslated region
