# Supplementary material for: Sex Differences in the Methylome and Transcriptome of the Human Liver and Circulating HDL-Cholesterol Levels
Source: J Clin Endocrinol Metab. 2018 May 28;103(12):4395–408. doi: 10.1210/jc.2018-00423 (PMC6212806; doi:10.1210/jc.2018-00423)
Supplement: Supplemental Data [file jc.2018-00423.sd1.pdf]

SUPPLEMANTAL EXPERIMENTAL PROCEDURES

Genome-wide DNA methylation profiling of human liver

DNA was extracted from human liver biopsies using Qiagen DNeasy blood & tissue kit. The nucleic concentration and purity were assessed using a Nano Drop 1000 spectrophotometer (NanoDrop Technologies, Wilmington, DE, USA). Next, 500 ng of DNA from human liver were bisulfite converted with the EZ DNA methylation kit (Zymo Research, Orange, CA, USA). The Infinium HumanMethylation450 BeadChip assay (Illumina, San Diego, CA, USA) was used to analyze DNA methylation genome-wide. This chip covers a total of 485,577 probes corresponding to 21,231 (99%) RefSeq genes. The bead chips were imaged using the Illumina iScan. All samples displayed a high-quality bisulfite conversion efficiency (intensity signal > 4,000), and passed GenomeStudio quality control steps. DNA methylation raw data were exported from GenomeStudio and analyzed using Bioconductor (1). Furthermore, the lumi package was used to convert β-values to M-values [$M=\log_2(\beta/(1-\beta))$] to make the data more homoscedastic (2). Then, the following probes were filtered away: 14,548 probes that are cross-reactive with 49 and 50 base pairs (3), 50 Y-chromosome probes and 2,973 non-CpG probes. In addition, 1,598 individual probes were filtered away based on Illumina detection *P*-value (mean *P*-value>0.01). Background correction and quantile normalization and the beta mixture quantile (BMIQ) normalization method were then performed (4). As 12 samples were analyzed on each chip, DNA methylation array data were batch-corrected using COMBAT (5). For easier interpretation and analysis of the results, M-values were reconverted to β-values, and methylation data was then separated on autosomes and X-chromosome for further analyses. There are no probes on the chip designed to target pseudoautosomal region on the X-chromosome because they would not be unique.

References

1. Gentleman RC, Carey VJ, Bates DM, Bolstad B, Dettling M, Dudoit S, et al. Bioconductor: open software development for computational biology and bioinformatics. Genome Biol 2004;5:R80.

2. Du P, Kibbe WA, Lin SM. lumi: a pipeline for processing Illumina microarray. Bioinformatics 2008;24:1547-1548.

3. Chen YA, Lemire M, Choufani S, Butcher DT, Grafodatskaya D, Zanke BW, et al. Discovery of cross-reactive probes and polymorphic CpGs in the Illumina Infinium HumanMethylation450 microarray. Epigenetics 2013;8:203-209.

4. Teschendorff AE, Marabita F, Lechner M, Bartlett T, Tegner J, Gomez-Cabrero D, et al. A beta-mixture quantile normalization method for correcting probe design bias in Illumina Infinium 450 k DNA methylation data. Bioinformatics 2013;29:189-196.

5. Johnson WE, Li C, Rabinovic A. Adjusting batch effects in microarray expression data using empirical Bayes methods. Biostatistics 2007;8:118-127.

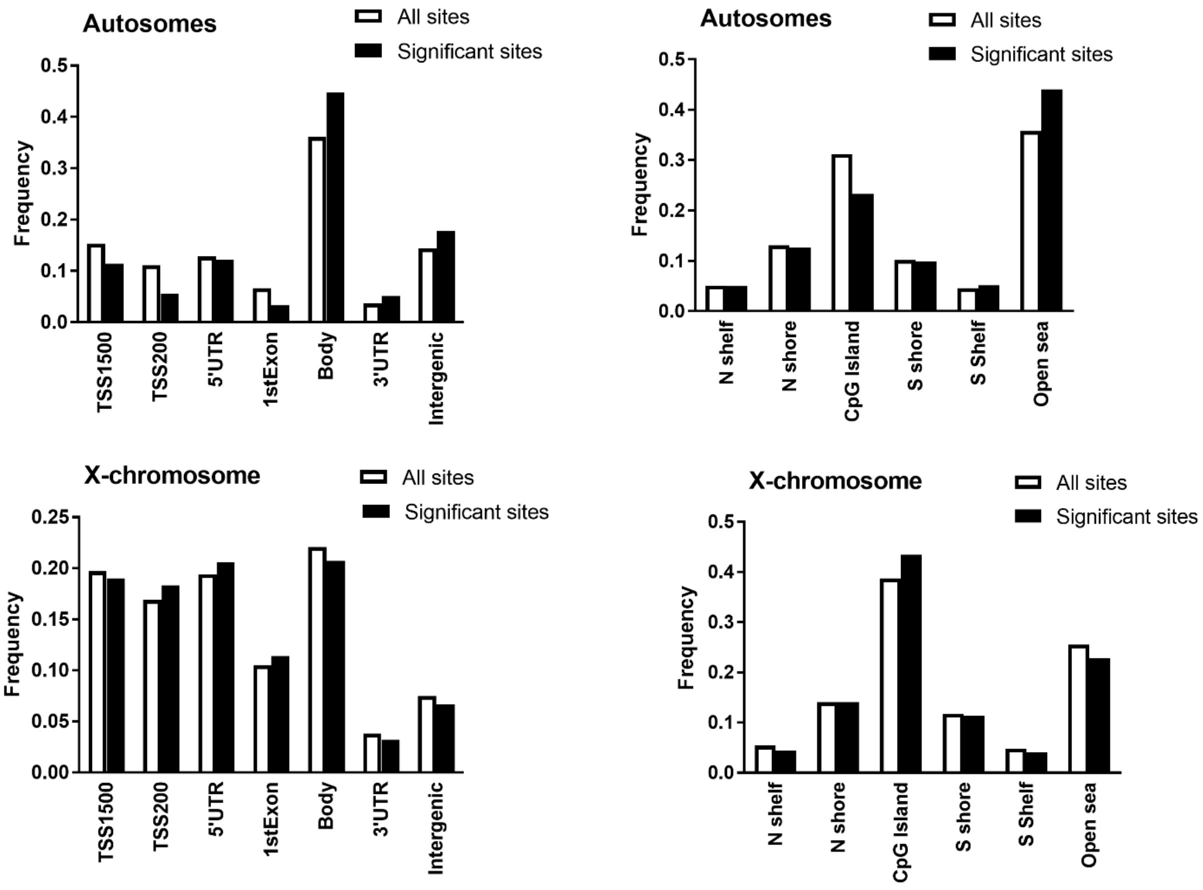

Supplemental Figure 1. Distribution of CpG sites with significant differences in DNA methylation in liver from males compared with females (FDR<0.05 and absolute differences >5%) versus all analyzed sites on the Infinium array in relation to genomic regions and CpG island regions on autosomes and on the X-chromosome. The distribution of significant CpG sites compared with all analyzed CpG sites is significantly different from what is expected by chance based on chi2 tests in all comparisons ($P < 2.2\text{x}10^{-16}$). Here, *P*-values have been corrected for multiple testing using Bonferroni correction.

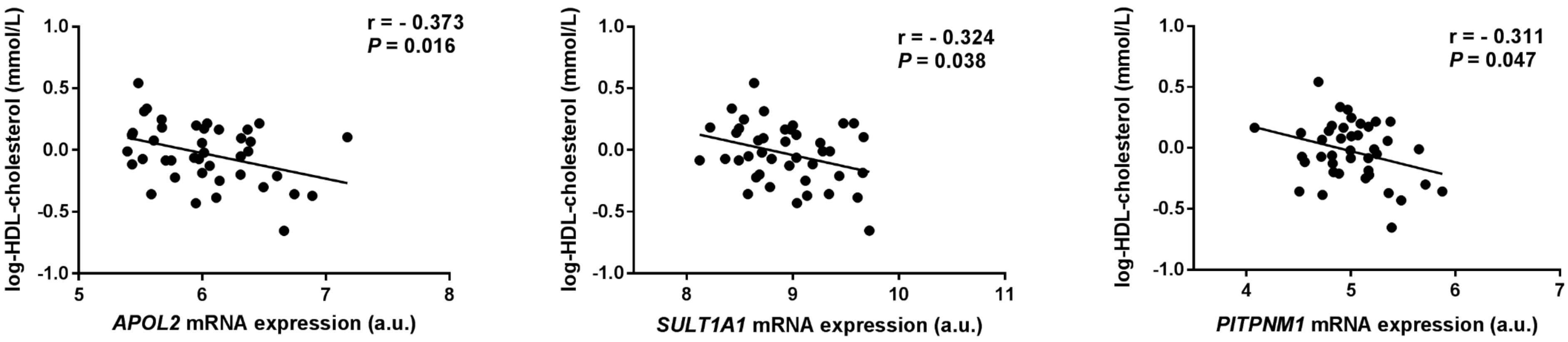

Supplemental Figure 2. Correlations between serum HDL-cholesterol levels and liver gene expression in human liver (*q*-value<0.05). mRNA expression of *APOL2, SULT1A1* and *PITPNM1* correlated significantly with HDL levels.

Supplemental Table 1. DNA sequences of the primers used for pyrosequencing

Gene	Illumina probe ID	Chr	Position	Pre-designed assay ID for pyrosequencing
APLN	cg05688478	X	128789164	Hs_CG05688478_01_PM_Q96
NKAP	cg27483305	X	119077757	Hs_CG27483305_01_PM_Q96

Supplemental Table 2. Clinical characteristics of 42 subjects included for the mRNA expression analyses

Phenotype	Males	Females	*P*-value[a]
n	19	23	
Age (years)	50.9 ± 7.7	51.9 ± 5.3	0.990
BMI (kg/m^2)	41.2 ± 5.8	43.9 ± 6.5	0.100
fP-glucose (mmol/L)	6.4 ± 1.5	6.1 ± 1.3	0.226
fS-insulin (mU/L)	21.4 ± 17.4	20.1 ± 14.9	0.822
Total cholesterol (mmol/L)	4.0 ± 0.8	4.2 ± 1.5	0.511
LDL-cholesterol (mmol/L)	2.3 ± 0.8	2.6 ± 1.0	0.464
HDL-cholesterol (mmol/L)	0.9 ± 0.2	1.1 ± 0.2	0.077
Triglycerides (mmol/L)	1.7 ± 0.5	1.6 ± 0.7	0.295

Abbreviations: fP, fasting glucose; fS, fasting serum; FFA: free fatty acids.
Data are shown as mean ± SD.
[a] Refers to Mann-Whitney test.

Table S3. Sex-significant autosomes DNA methylation data (q <0.05) in human liver.

					DNA methylation (%)						
Probe ID	Gene symbol	Chr	Gene region	CpG island region	Mean Females	SD Females	Mean Males	SD Males	Absolute difference (females-males)	P-value	q-value
cg04556210	GPR77	19	TSS1500	Open sea	49.02	10.57	76.58	3.94	-27.57	3.23E-27	2.65E-25
cg27121959	PGR	11	3'UTR	Open sea	45.69	8.24	72.75	3.56	-27.06	1.90E-34	1.79E-32
cg10479063	PZP	12	1stExon	Open sea	60.07	12.96	87.02	7.98	-26.95	3.09E-20	2.22E-18
cg08729600	PZP	12	TSS200	Open sea	55.34	13.14	82.06	8.26	-26.72	1.56E-18	1.09E-16
cg01714932	PZP	12	1stExon	Open sea	64.00	14.25	89.90	6.52	-25.90	5.77E-19	4.06E-17
cg21585138	CISH;CISH	3	Body;Body	N_Shelf	57.62	10.03	82.84	6.75	-25.22	4.06E-24	3.14E-22
cg10451565	GPR77	19	TSS1500	Open sea	48.88	9.38	72.60	4.85	-23.72	2.42E-25	1.92E-23
cg06416733		12	Intergenic	S_Shore	10.72	5.62	33.55	8.86	-22.82	4.56E-26	3.67E-24
cg07982208	GPR77	19	TSS200	Open sea	17.33	5.50	39.98	5.41	-22.65	2.44E-30	2.12E-28
cg04131969	MYADML	2	Body	N_Shore	39.84	34.03	62.34	33.09	-22.49	4.48E-03	4.93E-02
cg25593194	PADI2	1	3'UTR	Open sea	32.22	29.20	54.01	28.23	-21.79	1.37E-03	2.15E-02
cg01530482	MRPL23	11	Body	N_Shore	53.09	12.90	74.56	11.11	-21.47	4.35E-13	2.68E-11
cg13984330		17	Intergenic	Open sea	50.03	10.81	69.67	15.07	-19.64	3.13E-10	1.78E-08
cg20088245		8	Intergenic	Island	42.15	15.13	60.86	12.66	-18.71	1.36E-08	7.16E-07
cg12466610	MOSC2	1	Body	Open sea	21.28	28.83	39.36	31.23	-18.08	2.52E-03	3.32E-02
cg09203240	LRP5	11	Body	S_Shore	48.75	8.28	66.73	7.09	-17.99	5.26E-17	3.56E-15
cg22543924		12	Intergenic	N_Shore	54.10	27.70	72.07	22.27	-17.97	1.23E-03	2.00E-02
cg19548479	TMEM106A	17	TSS200	Island	16.76	9.84	34.59	12.07	-17.83	2.46E-11	1.44E-09
cg20704148	SLCO2B1;SLCO2B1	11	TSS1500;TSS1500	Open sea	34.10	33.50	51.55	28.95	-17.45	6.98E-04	1.31E-02
cg18222083	TMEM106A	17	5'UTR	Island	21.91	10.48	39.16	10.23	-17.25	4.35E-11	2.54E-09
cg01606800	SERPINA1;SERPINA1;SERPINA1;SERPII	14	TSS1500;5'UTR;5'UTR;5'UTR;5'UTR;5'UTR;	Open sea	66.70	10.93	83.88	7.16	-17.18	1.17E-12	7.11E-11
cg25735648		15	Intergenic	Open sea	38.02	9.49	55.19	7.72	-17.17	9.05E-14	5.68E-12
cg03128011		8	Intergenic	Island	41.21	14.61	58.08	12.71	-16.87	8.99E-08	4.50E-06
cg14283447	TFF3	21	1stExon	Open sea	45.56	8.89	62.37	5.66	-16.81	9.00E-16	5.93E-14
cg05334190		13	Intergenic	Open sea	38.41	6.17	55.14	5.78	-16.72	1.57E-21	1.16E-19
cg23180841		2	Intergenic	Open sea	63.66	8.49	80.34	7.77	-16.67	1.26E-15	8.28E-14
cg10341310	MTFR1;MTFR1;MTFR1	8	Body;Body;1stExon	Open sea	43.23	3.98	59.82	3.85	-16.59	8.25E-33	7.51E-31
cg13032057		6	Intergenic	N_Shore	60.53	11.89	77.05	4.55	-16.52	8.22E-13	5.04E-11
cg08377924	NBR1;TMEM106A;NBR1;NBR1	17	3'UTR;TSS1500;3'UTR;3'UTR	N_Shore	14.41	6.39	30.85	11.67	-16.45	2.24E-15	1.46E-13
cg12637676	GATA4	8	Body	Open sea	37.18	7.72	53.58	6.20	-16.39	3.12E-17	2.12E-15
cg10206882	STXBP6	14	Body	Open sea	64.52	10.48	80.87	4.97	-16.34	4.11E-14	2.60E-12
cg06408043		16	Intergenic	Open sea	57.52	7.53	73.85	9.89	-16.33	1.26E-12	7.67E-11
cg19662846	ADAMTS10	19	Body	Island	66.02	10.61	82.21	7.37	-16.19	1.83E-11	1.08E-09
cg00796611	SRGAP2;SRGAP2;SRGAP2	1	Body;Body;Body	Open sea	49.97	8.18	66.10	8.33	-16.13	2.16E-14	1.38E-12
cg26053346	LRIG1	3	Body	Open sea	52.51	11.79	68.19	12.54	-15.69	4.86E-08	2.48E-06
cg07824888	MRPL23	11	Body	N_Shore	72.71	10.24	88.35	6.29	-15.64	4.63E-14	2.92E-12
cg23499779		12	Intergenic	Open sea	46.97	6.47	62.41	7.44	-15.45	2.93E-17	2.00E-15
cg05613273	NARF;NARF;NARF;NARF	17	5'UTR;Body;Body;Body	S_Shelf	57.33	7.20	72.77	5.21	-15.43	1.84E-18	1.29E-16
cg03360728	ABTB2	11	Body	Open sea	58.49	8.05	73.89	5.92	-15.41	1.59E-16	1.07E-14
cg20149780	EFHD2	1	Body	Open sea	67.74	9.64	83.07	4.49	-15.33	5.99E-15	3.86E-13
cg24910352	GATA4	8	Body	Open sea	30.58	7.87	45.88	5.00	-15.30	5.64E-15	3.64E-13
cg06112835	MRPL21;MRPL21	11	3'UTR;3'UTR	Open sea	45.02	9.43	60.27	6.34	-15.25	5.67E-13	3.49E-11
cg13608166	NFATC1;NFATC1;NFATC1	18	Body;5'UTR;Body	Island	49.74	11.07	64.86	12.98	-15.12	4.88E-08	2.49E-06
cg02798874	TRPV1;TRPV1;TRPV1;TRPV1	17	3'UTR;3'UTR;3'UTR;3'UTR	Open sea	64.70	3.67	79.64	2.63	-14.93	8.14E-38	8.26E-36
cg08887138		8	Intergenic	Open sea	48.68	10.15	63.61	11.41	-14.93	2.96E-09	1.62E-07
cg06900821	LLGL2;LLGL2;LLGL2	17	5'UTR;5'UTR;5'UTR	Open sea	64.98	7.32	79.78	5.52	-14.80	3.84E-19	2.71E-17
cg15011775	RARB;RARB	3	5'UTR;Body	Open sea	58.12	3.60	72.88	3.63	-14.76	1.47E-32	1.33E-30
cg24983858	ZMIZ1	10	5'UTR	Open sea	58.19	8.64	72.95	7.34	-14.76	2.58E-15	1.68E-13

cg22235258	EHF	11	Body	Open sea	42.23	6.08	56.88	9.15	-14.65	7.83E-15	5.04E-13
cg21862992		11	Intergenic	Open sea	56.43	8.90	71.08	5.00	-14.65	9.81E-14	6.15E-12
cg11114242	MAP7	6	Body	Open sea	69.17	9.02	83.80	4.73	-14.63	5.02E-15	3.25E-13
cg24940138	TMEM106A	17	TSS200	Island	15.94	7.89	30.54	10.90	-14.60	4.21E-11	2.46E-09
cg23520422	PHLDA1	12	3'UTR	N_Shore	38.55	10.00	53.13	8.77	-14.58	1.84E-11	1.08E-09
cg07381872		1	Intergenic	Open sea	53.32	6.13	67.81	5.30	-14.49	8.48E-20	6.06E-18
cg03981793	C15orf33;FGF7	15	Body;5'UTR	Open sea	42.98	7.70	57.45	6.67	-14.47	1.79E-14	1.15E-12
cg12691488		1	Intergenic	Island	16.93	3.26	31.38	4.69	-14.45	4.66E-29	3.95E-27
cg22861548	LYNX1;LYNX1;LYNX1;LYNX1	8	TSS200;TSS1500;TSS200;TSS1500	S_Shore	50.60	11.46	65.02	13.10	-14.42	5.17E-07	2.41E-05
cg00531137		16	Intergenic	Open sea	25.48	6.93	39.83	8.42	-14.35	3.43E-13	2.12E-11
cg25148758		1	Intergenic	Open sea	59.46	8.89	73.76	7.55	-14.30	1.54E-11	9.13E-10
cg14434242		17	Intergenic	Open sea	71.71	11.23	86.01	6.30	-14.29	3.27E-10	1.86E-08
cg05135252		16	Intergenic	Open sea	68.12	6.42	82.38	6.78	-14.26	9.02E-17	6.07E-15
cg03049782	TMEM106A	17	TSS200	Island	18.64	8.88	32.88	8.62	-14.24	1.69E-10	9.69E-09
cg17599586	PISD	22	5'UTR	Island	20.63	8.85	34.71	9.21	-14.08	3.78E-11	2.21E-09
cg21126344	SOX9	17	Body	Island	24.47	11.65	38.41	12.29	-13.94	9.00E-06	3.42E-04
cg02734358	GPRIN3	4	5'UTR	N_Shore	41.82	10.02	55.74	11.40	-13.93	8.83E-10	4.93E-08
cg06305422		3	Intergenic	Open sea	16.64	6.05	30.53	8.44	-13.90	2.27E-17	1.55E-15
cg16467811		4	Intergenic	Open sea	25.55	6.32	39.41	4.36	-13.86	4.38E-18	3.03E-16
cg01872593		14	Intergenic	Open sea	51.30	10.12	65.03	9.75	-13.73	4.56E-12	2.74E-10
cg15877520	RNF39;RNF39	6	Body;Body	Island	19.10	11.43	32.82	16.98	-13.73	1.35E-05	4.94E-04
cg21796170		6	Intergenic	Open sea	72.91	21.56	86.56	10.20	-13.66	3.52E-05	1.15E-03
cg22884130	PISD	22	TSS200	Island	16.84	8.60	30.40	10.10	-13.56	9.76E-10	5.45E-08
cg23930334	SLC22A20	11	Body	Island	16.15	11.57	29.68	15.72	-13.52	1.10E-06	4.92E-05
cg14373380	ZBTB17	1	3'UTR	N_Shore	61.19	8.90	74.67	7.63	-13.48	1.35E-10	7.79E-09
cg17886715		1	Intergenic	S_Shore	69.89	5.38	83.33	3.55	-13.44	2.32E-23	1.77E-21
cg08452338	WWTR1;WWTR1;WWTR1	3	Body;Body;Body	Open sea	29.44	6.18	42.80	10.58	-13.36	7.95E-11	4.61E-09
cg04218124	VIL1	2	5'UTR	Open sea	59.07	9.17	72.42	6.65	-13.35	5.52E-11	3.21E-09
cg27637303		2	Intergenic	N_Shore	45.52	9.84	58.85	9.10	-13.33	2.13E-08	1.11E-06
cg12301921		5	Intergenic	Open sea	70.34	10.87	83.65	6.97	-13.30	7.29E-09	3.91E-07
cg10174575	C1orf130	1	Body	Open sea	62.16	9.36	75.45	8.31	-13.29	8.58E-09	4.58E-07
cg02556954	ETF1	5	Body	Open sea	52.61	2.78	65.89	2.47	-13.28	3.31E-37	3.30E-35
cg13405775	RFPL2;RFPL2;RFPL2	22	TSS200;5'UTR;TSS1500	Open sea	48.84	11.09	62.12	10.87	-13.27	7.85E-09	4.20E-07
cg08827454		6	Intergenic	N_Shore	47.61	9.13	60.87	3.93	-13.26	2.28E-12	1.38E-10
cg14923398		2	Intergenic	Open sea	32.44	10.30	45.69	14.71	-13.25	1.71E-05	6.07E-04
cg04815959	ADAM19	5	Body	Open sea	45.19	7.60	58.42	8.07	-13.22	4.85E-12	2.91E-10
cg27525902	C15orf33;FGF7	15	Body;5'UTR	Open sea	49.69	7.39	62.87	6.95	-13.18	1.51E-13	9.44E-12
cg19876649	MYOM1;MYOM1	18	TSS200;TSS200	Open sea	50.83	9.75	64.00	11.10	-13.17	1.30E-08	6.86E-07
cg12704462	MIR1178;CIT	12	Body;Body	Open sea	61.46	6.98	74.62	5.06	-13.15	1.99E-16	1.33E-14
cg18621672	DYDC1	10	5'UTR	N_Shelf	54.99	24.02	68.05	17.98	-13.06	2.59E-03	3.39E-02
cg00151768	NFATC1;NFATC1;NFATC1	18	Body;5'UTR;Body	Island	62.82	11.54	75.88	10.25	-13.06	9.85E-07	4.43E-05
cg23452498		5	Intergenic	Open sea	63.14	9.31	76.19	7.36	-13.05	8.98E-11	5.20E-09
cg12633154	RNF39;RNF39	6	Body;Body	Island	34.46	11.46	47.48	14.62	-13.03	2.19E-06	9.34E-05
cg24680439		10	Intergenic	N_Shore	55.67	17.27	68.70	12.14	-13.02	3.47E-05	1.13E-03
cg24128316	MYOM1;MYOM1;MYOM1;MYOM1	18	5'UTR;1stExon;5'UTR;1stExon	Open sea	33.50	7.39	46.51	12.48	-13.01	1.49E-08	7.85E-07
cg25184502		5	Intergenic	S_Shelf	60.99	26.73	74.00	17.15	-13.01	3.01E-03	3.75E-02
cg24300607		7	Intergenic	Open sea	51.75	9.75	64.71	7.51	-12.97	9.69E-11	5.61E-09
cg02325951	FOXN3;FOXN3	14	Body;Body	N_Shelf	58.88	7.44	71.83	4.89	-12.96	2.70E-17	1.84E-15
cg15945235	ANKRD22;ANKRD22	10	1stExon;5'UTR	Open sea	52.00	10.90	64.95	7.00	-12.95	2.39E-10	1.36E-08
cg04946709	LOC644649	16	Body	Island	68.54	4.10	81.41	3.18	-12.86	1.73E-29	1.47E-27
cg08304084	SALL1;SALL1	16	Body;Body	S_Shore	38.11	12.52	50.87	14.87	-12.77	1.01E-04	2.82E-03
cg01678472	KCTD2	17	Body	Open sea	54.50	9.21	67.20	9.06	-12.70	8.77E-08	4.40E-06
cg08257212		12	Intergenic	Open sea	60.09	4.07	72.78	3.96	-12.69	3.93E-26	3.17E-24

cg19689876	NLRP2	19	TSS200	Open sea	56.52	16.75	69.19	12.46	-12.68	4.64E-04	9.57E-03
cg12773325	INS-IGF2;IGF2AS;IGF2;IGF2;IGF2AS;IG	11	Body;TSS1500;Body;5'UTR;TSS1500;TSS15	Island	37.21	10.40	49.88	8.24	-12.68	4.34E-07	2.04E-05
cg12614029	INS-IGF2;IGF2AS;IGF2;IGF2;IGF2AS;IG	11	Body;TSS1500;Body;5'UTR;TSS1500;TSS15	Island	42.49	10.02	55.16	7.22	-12.67	6.53E-08	3.31E-06
cg17760849	NLRP2	19	TSS200	Open sea	66.96	16.31	79.63	11.72	-12.66	1.04E-04	2.91E-03
cg13492331	FAM107B	10	Body	Open sea	43.19	5.22	55.83	6.89	-12.64	4.76E-17	3.23E-15
cg10298992	ZNRF1	16	Body	Open sea	50.37	6.61	62.96	5.84	-12.59	1.31E-15	8.60E-14
cg22542685		17	Intergenic	N_Shore	67.73	3.16	80.30	3.05	-12.57	2.52E-32	2.27E-30
cg05272888	ANUBL1;ANUBL1	10	TSS1500;TSS1500	S_Shore	64.94	7.23	77.50	5.60	-12.56	2.77E-14	1.76E-12
cg14608156	INS-IGF2;IGF2AS;IGF2;IGF2;IGF2AS;IG	11	Body;TSS1500;Body;5'UTR;TSS1500;TSS15	Island	39.18	9.94	51.73	8.13	-12.55	8.84E-08	4.42E-06
cg07687131	SOCS2	12	Body	S_Shore	28.10	8.73	40.65	9.81	-12.55	1.84E-09	1.02E-07
cg15644727		11	Intergenic	Open sea	54.14	11.30	66.68	10.14	-12.54	7.61E-07	3.47E-05
cg01649623	AFF3;AFF3	2	Body;Body	Open sea	53.96	7.72	66.46	7.28	-12.50	5.38E-11	3.13E-09
cg12906381	RFPL2;RFPL2;RFPL2	22	TSS200;5'UTR;TSS1500	Open sea	46.85	11.04	59.32	9.07	-12.47	7.99E-08	4.01E-06
cg07293584		11	Intergenic	Island	11.31	6.69	23.75	13.68	-12.44	3.81E-09	2.07E-07
cg24008544	TMEM106A;TMEM106A	17	5'UTR;1stExon	Island	16.71	7.52	29.15	9.10	-12.44	2.43E-10	1.39E-08
cg25791279	PISD	22	TSS200	S_Shore	23.58	7.15	35.98	8.34	-12.41	2.61E-13	1.62E-11
cg12377641	C10orf47	10	5'UTR	S_Shelf	52.71	10.22	65.11	7.84	-12.40	2.27E-07	1.10E-05
cg16423624	ALDH2	12	Body	Open sea	53.74	10.52	66.12	12.21	-12.38	2.61E-06	1.10E-04
cg08194369	MPP4	2	TSS1500	Open sea	67.83	9.10	80.18	5.40	-12.36	1.02E-11	6.05E-10
cg10012530	HS6ST1	2	Body	N_Shore	36.06	6.21	48.40	7.62	-12.34	2.83E-12	1.71E-10
cg02858826	RPS6KL1	14	TSS200	N_Shore	71.59	9.85	83.91	4.73	-12.33	1.44E-10	8.31E-09
cg18087943	INS-IGF2;IGF2AS;IGF2;IGF2;IGF2AS;IG	11	Body;TSS1500;Body;5'UTR;TSS1500;TSS15	Island	33.72	9.02	46.03	8.22	-12.31	6.01E-08	3.05E-06
cg05479462	RFESD;RFESD;RFESD	5	5'UTR;5'UTR;5'UTR	S_Shelf	75.05	7.55	87.34	4.77	-12.30	3.97E-15	2.57E-13
cg08371852	ARNT2	15	Body	Open sea	55.75	9.16	68.04	8.82	-12.29	5.18E-08	2.64E-06
cg10780002		14	Intergenic	Open sea	66.35	7.90	78.62	8.02	-12.28	2.71E-10	1.54E-08
cg12160586	ASCL1	12	TSS1500	N_Shore	18.69	5.13	30.95	4.75	-12.26	1.35E-17	9.28E-16
cg00862597		14	Intergenic	S_Shelf	43.68	5.79	55.94	5.20	-12.26	4.95E-17	3.35E-15
cg25937216		8	Intergenic	Island	67.16	10.05	79.40	8.43	-12.24	6.95E-10	3.90E-08
cg01338084	PISD	22	5'UTR	Island	14.62	5.81	26.86	8.55	-12.24	3.83E-14	2.42E-12
cg11871050	GNB5	15	5'UTR	Open sea	47.90	6.54	60.13	5.91	-12.23	1.28E-14	8.19E-13
cg01124132	RFPL2;RFPL2;RFPL2	22	TSS200;5'UTR;TSS1500	Open sea	51.32	12.22	63.53	9.90	-12.22	2.13E-07	1.04E-05
cg24380380	LRP5	11	Body	N_Shore	51.37	6.82	63.57	4.40	-12.20	1.04E-15	6.86E-14
cg17238412		11	Intergenic	Island	17.45	9.40	29.65	10.87	-12.20	7.17E-07	3.28E-05
cg24501177		15	Intergenic	Open sea	48.09	5.72	60.27	7.44	-12.18	7.61E-13	4.67E-11
cg24929938		7	Intergenic	Open sea	41.73	7.51	53.88	4.01	-12.15	1.69E-12	1.02E-10
cg14450910		9	Intergenic	Open sea	58.70	9.61	70.85	6.96	-12.15	5.39E-12	3.23E-10
cg01860753	RASSF5;RASSF5;RASSF5;RASSF5	1	Body;5'UTR;Body;1stExon	Island	20.43	6.96	32.57	7.88	-12.14	3.43E-12	2.07E-10
cg03036702	FOXP4;FOXP4;FOXP4	6	5'UTR;5'UTR;5'UTR	N_Shore	37.47	23.64	49.60	15.24	-12.13	2.14E-03	2.96E-02
cg22958403	C14orf49	14	Body	Open sea	45.45	6.80	57.57	5.80	-12.12	1.37E-13	8.56E-12
cg00815832		1	Intergenic	Island	56.15	7.45	68.26	9.68	-12.12	2.23E-09	1.23E-07
cg13401893	RNF39;RNF39	6	Body;Body	Island	38.65	11.30	50.72	12.17	-12.07	3.33E-06	1.37E-04
cg09803321	NT5C2;NT5C2	10	Body;Body	Open sea	43.56	9.83	55.60	7.41	-12.04	4.39E-08	2.25E-06
cg10143507		2	Intergenic	N_Shore	22.79	6.63	34.83	6.67	-12.04	1.96E-11	1.15E-09
cg06974135	PDE4D;PDE4D;PDE4D	5	Body;Body;Body	Open sea	75.39	8.92	87.42	3.44	-12.03	3.72E-13	2.30E-11
cg27589809	CISH;CISH	3	TSS1500;TSS1500	S_Shore	50.21	5.60	62.23	4.34	-12.03	8.88E-17	5.98E-15
cg25880347	GLI4	8	Body	N_Shelf	55.39	7.18	67.38	7.22	-11.99	7.48E-11	4.34E-09
cg03455316		15	Intergenic	Island	52.62	9.68	64.59	8.60	-11.97	2.88E-07	1.38E-05
cg10568066	RNF39;RNF39	6	Body;Body	Island	38.35	13.38	50.30	14.86	-11.96	1.24E-04	3.34E-03
cg26271110	RASSF5;RASSF5;RASSF5;RASSF5	1	Body;5'UTR;Body;1stExon	Island	23.47	8.27	35.43	6.48	-11.95	3.53E-10	2.01E-08
cg23096305		15	Intergenic	Open sea	54.40	8.02	66.34	7.32	-11.94	9.44E-10	5.27E-08
cg18949721		14	Intergenic	Open sea	34.41	14.14	46.35	23.75	-11.94	9.48E-04	1.65E-02
cg06686742	LASS4	19	TSS1500	N_Shore	33.66	10.00	45.58	10.21	-11.92	1.50E-06	6.55E-05
cg20249327	RNF39;RNF39	6	Body;Body	Island	19.53	8.98	31.41	14.94	-11.87	3.33E-06	1.37E-04

cg24985525	LRP5	11	Body	S_Shelf	45.79	7.90	57.65	7.72	-11.86	1.35E-10	7.79E-09
cg05541267	CISH;CISH	3	Body;Body	N_Shore	75.28	8.76	87.13	3.36	-11.84	7.64E-13	4.69E-11
cg08840441	GMIP	19	Body	Island	53.77	11.75	65.61	11.11	-11.84	7.03E-06	2.73E-04
cg14477471		9	Intergenic	Open sea	34.90	7.80	46.74	8.64	-11.84	1.85E-08	9.72E-07
cg16597993	EPS15L1	19	Body	N_Shelf	48.85	7.40	60.68	8.89	-11.83	1.71E-09	9.44E-08
cg16848717	NFATC1;NFATC1;NFATC1	18	Body;5'UTR;Body	Island	44.81	8.00	56.64	10.47	-11.83	5.03E-08	2.57E-06
cg27462519		16	Intergenic	Island	51.05	8.99	62.87	8.32	-11.83	2.78E-09	1.52E-07
cg08100687		13	Intergenic	Island	26.82	10.05	38.64	13.93	-11.83	9.19E-06	3.49E-04
cg01917016	RASSF5;RASSF5;RASSF5;RASSF5	1	Body;5'UTR;Body;1stExon	Island	13.37	6.24	25.18	7.40	-11.82	5.40E-13	3.33E-11
cg21385480	LRIG1	3	Body	Open sea	55.52	9.52	67.33	11.42	-11.81	1.38E-06	6.08E-05
cg09941713	CHD7	8	Body	Open sea	42.82	6.96	54.63	6.75	-11.81	5.98E-12	3.58E-10
cg09248054	AGRN	1	Body	Island	37.92	9.94	49.71	9.21	-11.79	2.78E-08	1.44E-06
cg10036013	FOXK1	7	Body	Open sea	23.82	7.73	35.60	10.69	-11.77	2.43E-08	1.27E-06
cg18076842	C11orf75	11	5'UTR	Open sea	64.02	7.81	75.79	3.98	-11.77	3.50E-15	2.27E-13
cg11240062		8	Intergenic	Island	31.20	8.84	42.97	8.88	-11.77	7.18E-07	3.29E-05
cg05819837	CUX1;CUX1;CUX1	7	Body;Body;Body	Open sea	76.60	7.89	88.37	2.27	-11.77	9.08E-17	6.11E-15
cg21258821	KBTBD6;KBTBD6	13	3'UTR;1stExon	N_Shelf	66.69	8.35	78.44	3.66	-11.75	4.25E-13	2.63E-11
cg21148594		14	Intergenic	Open sea	55.87	4.96	67.61	3.29	-11.74	7.25E-21	5.28E-19
cg21211480	TMEM106A	17	TSS200	Island	11.94	6.37	23.66	8.22	-11.72	5.00E-12	3.00E-10
cg03342674		9	Intergenic	N_Shore	42.81	7.56	54.52	7.56	-11.71	6.62E-10	3.72E-08
cg03144839		3	Intergenic	Open sea	65.00	10.21	76.71	9.81	-11.71	5.56E-09	3.00E-07
cg14665366	GPRIN3	4	5'UTR	N_Shore	55.45	9.80	67.15	9.63	-11.70	3.68E-08	1.90E-06
cg03618918		1	Intergenic	Open sea	71.57	2.85	83.24	1.91	-11.68	7.24E-38	7.36E-36
cg01346596	C10orf47	10	5'UTR	S_Shelf	36.27	10.86	47.94	10.60	-11.67	6.04E-06	2.38E-04
cg14310225		6	Intergenic	N_Shelf	75.77	9.56	87.44	5.21	-11.66	1.80E-10	1.03E-08
cg01472043	C10orf47	10	5'UTR	S_Shelf	56.89	11.18	68.51	7.54	-11.63	1.18E-05	4.37E-04
cg15897368	SMOC1;SMOC1	14	Body;Body	Open sea	65.16	9.10	76.76	6.79	-11.60	5.04E-08	2.57E-06
cg08621893	NUDT6;NUDT6	4	5'UTR;Body	N_Shore	49.98	8.10	61.57	9.11	-11.59	2.81E-08	1.46E-06
cg02570643	AP3B1	5	Body	Open sea	76.53	6.75	88.11	4.12	-11.59	4.82E-17	3.27E-15
cg11946666	PRDM16;PRDM16	1	Body;Body	Open sea	58.96	6.85	70.53	5.16	-11.57	6.17E-14	3.88E-12
cg09409484	LOC400752	1	Body	S_Shore	59.03	6.84	70.60	5.99	-11.57	1.25E-11	7.40E-10
cg07382347	RNF39;RNF39	6	Body;Body	Island	24.70	11.68	36.26	14.54	-11.57	3.18E-04	7.12E-03
cg18792528	LDLRAP1	1	3'UTR	Open sea	45.96	6.74	57.52	8.98	-11.57	3.00E-09	1.64E-07
cg04482110	TMEM106A	17	5'UTR	Island	26.58	8.13	38.13	7.39	-11.55	6.32E-10	3.55E-08
cg18535410	F10	13	Body	Open sea	40.75	7.03	52.30	6.88	-11.55	1.14E-11	6.74E-10
cg02568716		12	Intergenic	Open sea	59.84	6.51	71.37	5.27	-11.53	1.39E-14	8.87E-13
cg03956243	ATP5G2;ATP5G2	12	3'UTR;3'UTR	Open sea	53.84	3.85	65.37	4.77	-11.52	7.49E-21	5.46E-19
cg24452821	ZBTB16;ZBTB16	11	Body;Body	Island	28.91	7.11	40.39	9.07	-11.49	9.99E-09	5.32E-07
cg15912732	AKT1;AKT1;AKT1	14	Body;Body;Body	S_Shelf	49.80	6.45	61.25	7.81	-11.46	2.30E-10	1.31E-08
cg03154717		6	Intergenic	N_Shore	45.86	8.58	57.32	7.58	-11.45	2.39E-09	1.31E-07
cg15026277	TMEM106A	17	TSS200	Island	11.82	6.52	23.28	9.10	-11.45	7.66E-10	4.29E-08
cg02973693	ZFHX3	16	5'UTR	S_Shelf	39.97	6.10	51.42	9.80	-11.45	3.38E-10	1.92E-08
cg03217115		2	Intergenic	N_Shore	40.91	7.59	52.29	7.13	-11.38	1.47E-09	8.15E-08
cg02998240		1	Intergenic	Open sea	41.37	10.85	52.75	10.34	-11.38	8.94E-07	4.04E-05
cg02097433		10	Intergenic	Open sea	58.72	11.74	70.07	7.26	-11.34	9.23E-06	3.50E-04
cg00792688		22	Intergenic	Open sea	60.76	8.12	72.10	5.63	-11.33	3.50E-11	2.05E-09
cg08405910	NCK2;NCK2;NCK2	2	Body;Body;Body	S_Shelf	59.75	6.79	71.07	5.08	-11.33	2.64E-13	1.64E-11
cg12269535	SRF	6	Body	Island	24.87	5.14	36.20	5.83	-11.33	6.36E-15	4.10E-13
cg25987564		4	Intergenic	Island	35.67	9.91	46.98	10.12	-11.30	1.76E-06	7.62E-05
cg06243458	BANF2;BANF2;BANF2	20	TSS1500;TSS1500;Body	Open sea	31.47	7.49	42.76	5.62	-11.29	1.86E-13	1.16E-11
cg08043781		2	Intergenic	Open sea	63.32	8.41	74.61	5.31	-11.28	1.26E-13	7.88E-12
cg15054077	NFIX	19	Body	Island	25.11	6.79	36.39	7.88	-11.28	1.79E-11	1.05E-09
cg27624826		2	Intergenic	Island	26.64	8.30	37.91	9.97	-11.27	2.85E-07	1.37E-05

cg05021743	C2CD4D;LOC100132111	1	Body;TSS200	Island	12.99	7.94	24.25	13.20	-11.26	5.47E-07	2.54E-05
cg13491490	ERC2	3	3'UTR	Open sea	58.75	5.29	69.99	5.34	-11.24	1.51E-16	1.01E-14
cg26448406	PISD	22	5'UTR	Island	11.40	6.19	22.63	8.91	-11.22	1.38E-10	7.96E-09
cg18391770	BAHCC1	17	Body	S_Shore	47.29	5.88	58.46	4.91	-11.17	3.24E-14	2.06E-12
cg09645818		11	Intergenic	Open sea	72.10	8.82	83.26	4.39	-11.16	1.05E-10	6.05E-09
cg10781408	C2CD4D;LOC100132111	1	Body;TSS200	Island	16.89	9.14	28.05	13.69	-11.16	7.93E-06	3.05E-04
cg19021188	LYNX1;LYNX1;LYNX1;LYNX1	8	TSS200;TSS1500;TSS200;TSS1500	S_Shore	52.33	8.54	63.47	10.96	-11.14	5.91E-07	2.74E-05
cg05132077		22	Intergenic	S_Shore	37.25	6.35	48.38	6.72	-11.14	2.28E-11	1.34E-09
cg00412675	GATA4	8	Body	Open sea	68.52	8.16	79.66	4.69	-11.14	4.22E-11	2.46E-09
cg08075024	SSFA2;SSFA2	2	Body;Body	S_Shelf	57.78	6.00	68.91	6.83	-11.12	1.77E-11	1.04E-09
cg01561719	ANKRD22	10	TSS200	Open sea	75.73	9.63	86.84	3.30	-11.11	5.29E-12	3.17E-10
cg05009047	FAM100A	16	3'UTR	Island	48.98	8.69	60.08	11.64	-11.10	5.57E-06	2.21E-04
cg07554771	MGMT	10	Body	Open sea	63.13	10.56	74.23	11.23	-11.10	5.94E-05	1.80E-03
cg24872571	LTBP4;LTBP4;LTBP4	19	Body;Body;Body	Island	51.40	11.55	62.49	12.26	-11.10	2.64E-04	6.17E-03
cg24603926	MIR1178;CIT	12	TSS200;Body	Open sea	51.08	6.73	62.18	6.91	-11.09	7.57E-12	4.52E-10
cg06648780	ALCAM	3	Body	Open sea	52.13	6.92	63.21	5.32	-11.08	3.20E-12	1.93E-10
cg24411597	NUDT6;NUDT6	4	5'UTR;Body	N_Shore	64.93	7.32	75.99	5.56	-11.07	3.48E-10	1.97E-08
cg27323784		11	Intergenic	Island	19.10	9.18	30.15	12.92	-11.05	8.49E-06	3.25E-04
cg05133179		11	Intergenic	Island	24.28	8.92	35.30	10.27	-11.03	1.48E-06	6.46E-05
cg07798386	HOOK2;HOOK2	19	3'UTR;3'UTR	N_Shelf	59.49	8.34	70.51	8.84	-11.01	9.58E-08	4.78E-06
cg23299919	PTPRN2;PTPRN2;PTPRN2	7	Body;Body;Body	Island	25.32	9.55	36.33	11.70	-11.01	1.83E-05	6.46E-04
cg20016993		2	Intergenic	S_Shelf	53.90	6.50	64.88	8.40	-10.98	1.32E-09	7.36E-08
cg11014921	TRANK1	3	Body	Open sea	61.76	7.14	72.75	7.34	-10.98	2.16E-10	1.24E-08
cg08911543	TMEM120A	7	Body	N_Shelf	53.93	9.21	64.89	8.76	-10.97	5.81E-09	3.13E-07
cg13726166	TIAF1;MYO18A;MYO18A	17	TSS1500;Body;Body	N_Shelf	62.58	7.95	73.54	8.65	-10.96	1.68E-08	8.82E-07
cg14089103	HCCA2	11	Body	Open sea	56.24	15.96	67.19	16.22	-10.95	1.20E-03	1.96E-02
cg09367198	ACIN1;ACIN1;ACIN1;ACIN1;ACIN1	14	TSS1500;TSS1500;Body;Body;Body	Open sea	47.08	6.74	58.01	5.05	-10.93	2.42E-12	1.47E-10
cg03174507		10	Intergenic	Island	29.22	8.71	40.14	15.28	-10.92	3.56E-05	1.16E-03
cg25104397	C10orf26;C10orf26;C10orf26	10	5'UTR;Body;1stExon	Open sea	32.90	10.42	43.82	8.58	-10.92	9.65E-06	3.65E-04
cg14408412	DOCK4	7	Body	Open sea	74.51	6.72	85.42	4.05	-10.91	1.26E-14	8.08E-13
cg07953015	SEPW1	19	3'UTR	S_Shelf	31.86	7.78	42.75	10.31	-10.90	1.18E-06	5.22E-05
cg27366882		3	Intergenic	Open sea	51.13	14.99	62.01	13.71	-10.88	2.94E-04	6.70E-03
cg16594139	PLEKHA4;HSD17B14;PLEKHA4	19	3'UTR;TSS1500;Body	Island	21.17	9.90	32.04	15.25	-10.87	8.67E-05	2.48E-03
cg13516808		11	Intergenic	Open sea	54.42	8.53	65.29	8.11	-10.87	2.68E-08	1.39E-06
cg19948807	GRB10;GRB10;GRB10;GRB10	7	Body;Body;Body;Body	Open sea	64.64	7.78	75.50	7.08	-10.87	1.38E-09	7.66E-08
cg19342782	HHLA3;ANKRD13C;HHLA3;HHLA3;HHL	1	Body;TSS1500;Body;Body;Body	S_Shore	50.32	6.49	61.17	6.84	-10.85	4.89E-11	2.85E-09
cg21735741		3	Intergenic	Island	71.99	13.98	82.82	11.36	-10.82	9.80E-05	2.75E-03
cg07608565	EPS8L1;EPS8L1	19	Body;Body	Island	29.65	9.15	40.47	10.93	-10.82	5.60E-07	2.60E-05
cg24983752		11	Intergenic	Open sea	45.13	10.09	55.94	10.22	-10.81	1.83E-06	7.88E-05
cg12184120	ZNF607	19	3'UTR	Open sea	80.31	2.98	91.10	1.79	-10.79	6.89E-36	6.70E-34
cg01150641	AGRN	1	Body	Island	13.58	6.16	24.37	12.61	-10.79	8.72E-08	4.37E-06
cg25304146	WBP11P1	18	Body	Open sea	57.85	2.91	68.60	3.03	-10.75	1.99E-29	1.69E-27
cg01234546	PLXNB2	22	Body	N_Shore	65.51	22.59	76.26	15.37	-10.75	3.53E-03	4.19E-02
cg02321620	RAPGEF4;RAPGEF4	2	Body;Body	Open sea	79.93	7.97	90.65	4.23	-10.73	4.13E-15	2.68E-13
cg22034735		8	Intergenic	S_Shore	57.17	10.22	67.89	8.58	-10.72	4.34E-07	2.05E-05
cg14786652	TTLL10	1	TSS200	N_Shore	26.46	8.93	37.18	11.93	-10.72	9.03E-07	4.08E-05
cg17529386		6	Intergenic	Island	59.38	10.67	70.08	12.85	-10.70	3.31E-05	1.09E-03
cg16977706	INS-IGF2;IGF2;IGF2;IGF2;IGF2	11	Body;Body;5'UTR;5'UTR;1stExon	Island	34.13	5.83	44.83	5.88	-10.70	3.63E-12	2.19E-10
cg00208274	FOXK1	7	Body	Open sea	39.55	8.68	50.25	12.06	-10.70	2.48E-06	1.05E-04
cg04590718	ADAMTS10	19	Body	N_Shore	62.34	7.44	73.03	5.97	-10.68	4.43E-10	2.50E-08
cg10629165	MAST4;MAST4;MAST4	5	TSS200;Body;Body	Open sea	27.39	8.18	38.05	9.06	-10.66	6.54E-08	3.31E-06
cg26218047		1	Intergenic	Open sea	65.07	13.75	75.73	13.05	-10.65	2.18E-04	5.30E-03
cg15015892	C2CD4D;LOC100132111	1	Body;TSS200	Island	18.01	8.96	28.66	12.86	-10.65	9.08E-06	3.45E-04

cg04296699	C2CD4D;LOC100132111	1	Body;TSS200	Island	17.06	9.00	27.69	13.77	-10.63	2.18E-05	7.55E-04
cg09768983		4	Intergenic	Open sea	57.74	13.11	68.36	12.53	-10.63	5.62E-04	1.11E-02
cg00211115		11	Intergenic	N_Shore	43.77	9.10	54.36	7.39	-10.59	1.73E-07	8.48E-06
cg10677144	MYOM1;MYOM1	18	TSS200;TSS200	Open sea	60.27	7.28	70.86	10.39	-10.59	7.75E-08	3.90E-06
cg04160749		8	Intergenic	Island	60.80	10.10	71.38	7.97	-10.58	5.06E-09	2.74E-07
cg20453820		12	Intergenic	Open sea	64.09	9.05	74.67	5.91	-10.57	1.06E-07	5.30E-06
cg19323059	LOC285954;LOC285954	7	Body;Body	S_Shelf	68.96	7.61	79.52	4.94	-10.56	3.22E-12	1.94E-10
cg00443917	FBRS	16	Body	Open sea	55.03	6.37	65.57	8.36	-10.55	4.16E-10	2.36E-08
cg16887422	HDAC4	2	Body	S_Shore	53.21	7.19	63.75	5.39	-10.54	5.52E-10	3.11E-08
cg14483162	TTC1	5	3'UTR	Open sea	68.56	8.17	79.09	3.84	-10.53	2.20E-11	1.30E-09
cg06249604	RNF39;RNF39	6	Body;Body	Island	19.52	10.60	30.05	14.33	-10.53	2.42E-04	5.76E-03
cg02277646		1	Intergenic	Open sea	60.06	12.04	70.59	10.28	-10.53	1.87E-04	4.68E-03
cg21963583	MRPL21;MRPL21	11	Body;Body	Open sea	48.64	6.00	59.16	5.48	-10.52	1.33E-12	8.12E-11
cg00339331		19	Intergenic	Island	57.27	7.60	67.78	7.44	-10.51	3.91E-08	2.01E-06
cg18403754	RAD51L1;RAD51L1;RAD51L1	14	Body;Body;Body	Open sea	65.24	7.76	75.75	7.12	-10.51	3.17E-09	1.73E-07
cg13253055	C10orf26;C10orf26	10	3'UTR;3'UTR	Open sea	57.97	8.38	68.47	8.74	-10.49	1.61E-07	7.89E-06
cg19895185	PACS2;PACS2	14	Body;Body	Open sea	53.02	8.10	63.51	9.44	-10.49	2.91E-06	1.21E-04
cg00051662	CYP2C19	10	TSS1500	Open sea	64.45	9.61	74.94	8.87	-10.48	6.14E-06	2.41E-04
cg25924746	IRS2	13	Body	N_Shore	61.86	7.93	72.31	7.29	-10.44	1.64E-08	8.64E-07
cg12082025	ABCA7	19	Body	Island	66.84	12.66	77.27	10.77	-10.43	1.46E-04	3.82E-03
cg07241084	TNS3	7	5'UTR	Open sea	32.20	7.62	42.62	6.68	-10.42	6.99E-10	3.92E-08
cg20790798		5	Intergenic	Island	57.62	11.58	68.04	16.30	-10.42	1.51E-04	3.94E-03
cg11715474		2	Intergenic	N_Shelf	36.65	7.35	47.06	6.22	-10.41	1.79E-10	1.03E-08
cg12469381	CHN2;CHN2	7	TSS200;Body	Open sea	19.13	10.56	29.53	12.26	-10.40	1.89E-03	2.71E-02
cg17438211		5	Intergenic	Open sea	49.17	8.36	59.57	11.11	-10.40	5.64E-06	2.23E-04
cg00154902		8	Intergenic	Island	40.34	12.39	50.74	16.77	-10.40	1.18E-03	1.94E-02
cg12701632		6	Intergenic	Open sea	53.04	7.93	63.44	9.87	-10.40	2.02E-06	8.66E-05
cg01564640	LTBP4;LTBP4;LTBP4	19	Body;Body;Body	Island	49.02	10.52	59.38	11.44	-10.37	1.38E-04	3.66E-03
cg22110839		7	Intergenic	Open sea	37.79	8.14	48.15	8.04	-10.36	7.19E-08	3.62E-06
cg10390762		6	Intergenic	Open sea	49.17	6.57	59.52	9.95	-10.35	2.34E-07	1.13E-05
cg12427303	RFPL2;RFPL2;RFPL2	22	TSS200;5'UTR;TSS1500	Open sea	40.48	10.56	50.82	9.22	-10.35	8.79E-07	3.97E-05
cg07355189	ALOX5	10	Body	Island	30.30	7.54	40.63	10.09	-10.34	1.00E-07	5.00E-06
cg26505974	MRPL23	11	Body	N_Shore	59.93	6.39	70.25	7.04	-10.32	4.00E-10	2.26E-08
cg27360992		22	Intergenic	Open sea	80.29	9.85	90.60	4.11	-10.31	2.67E-08	1.39E-06
cg19978416		7	Intergenic	N_Shelf	42.27	6.08	52.58	6.95	-10.31	2.28E-12	1.38E-10
cg22429121	AHDC1	1	5'UTR	Island	40.65	6.39	50.94	5.95	-10.29	1.45E-10	8.37E-09
cg05203809	SNX29	16	Body	Open sea	63.94	6.99	74.22	6.84	-10.29	2.15E-10	1.23E-08
cg03210827	C10orf26;C10orf26	10	3'UTR;3'UTR	Open sea	53.84	9.68	64.13	11.05	-10.28	3.88E-05	1.25E-03
cg00399059	LYNX1;LYNX1;LYNX1;LYNX1	8	TSS200;TSS1500;TSS200;TSS1500	S_Shore	44.58	9.65	54.86	13.74	-10.28	9.34E-05	2.65E-03
cg04755386		2	Intergenic	Open sea	62.72	7.39	72.99	9.69	-10.27	5.66E-07	2.63E-05
cg22177308		17	Intergenic	Open sea	42.80	8.11	53.06	12.73	-10.26	1.89E-05	6.66E-04
cg13782388	CHCHD6	3	Body	Open sea	55.84	6.67	66.09	3.45	-10.25	9.70E-13	5.93E-11
cg18237047		7	Intergenic	Open sea	52.84	16.81	63.09	11.10	-10.25	2.85E-03	3.61E-02
cg17214479		13	Intergenic	Open sea	57.40	7.88	67.63	7.31	-10.23	3.47E-08	1.79E-06
cg10622174		6	Intergenic	Open sea	32.62	5.38	42.85	6.60	-10.23	2.41E-12	1.46E-10
cg20268992	ZBTB16;ZBTB16	11	Body;Body	Island	54.97	10.87	65.20	10.89	-10.23	7.14E-05	2.11E-03
cg05563515	RNF39;RNF39	6	Body;Body	Island	38.62	10.29	48.84	13.55	-10.21	8.03E-05	2.33E-03
cg12418739	LRP5	11	Body	Island	78.59	7.57	88.80	3.81	-10.21	2.08E-12	1.26E-10
cg12789068	AKT1;AKT1;AKT1	14	Body;Body;Body	S_Shelf	65.76	8.11	75.97	6.14	-10.21	4.84E-08	2.47E-06
cg09975620	PDX1	13	Body	Island	21.84	9.75	32.05	12.27	-10.21	1.34E-04	3.57E-03
cg14663984	AGRN	1	Body	Island	18.21	5.78	28.40	10.26	-10.20	3.31E-09	1.81E-07
cg12031346	CNDP1	18	Body	Open sea	62.55	7.52	72.74	9.39	-10.19	7.94E-07	3.62E-05
cg02531277	CUX1;CUX1;CUX1	7	Body;Body;Body	N_Shore	57.38	8.46	67.57	9.83	-10.19	2.01E-06	8.62E-05

cg19246018	HDAC4	2	Body	N_Shore	75.79	9.29	85.98	7.17	-10.18	1.99E-07	9.69E-06
cg15202848		11	Intergenic	N_Shelf	40.75	6.53	50.93	6.91	-10.18	1.45E-09	8.04E-08
cg21900495	PDX1	13	Body	N_Shore	13.22	8.26	23.40	15.90	-10.18	2.45E-04	5.82E-03
cg13876809	GPR88	1	3'UTR	S_Shore	67.14	12.40	77.30	9.82	-10.16	5.90E-04	1.15E-02
cg26480889		2	Intergenic	N_Shore	19.32	6.00	29.48	6.12	-10.16	7.73E-10	4.33E-08
cg10036892		2	Intergenic	Open sea	36.58	7.83	46.74	11.94	-10.16	2.25E-05	7.76E-04
cg02757998	PLXNC1	12	Body	S_Shelf	63.89	7.14	74.04	5.63	-10.16	2.94E-10	1.68E-08
cg21380389		10	Intergenic	Open sea	50.41	8.52	60.56	11.90	-10.15	4.87E-05	1.52E-03
cg12535715	HTRA4;HTRA4	8	1stExon;5'UTR	Island	40.92	7.30	51.07	11.69	-10.15	2.48E-07	1.20E-05
cg13631075		6	Intergenic	Open sea	54.22	7.39	64.37	7.03	-10.15	1.08E-11	6.40E-10
cg18621142		10	Intergenic	Island	44.95	9.46	55.05	13.43	-10.11	1.34E-04	3.55E-03
cg14227418	UBAP2	9	Body	Open sea	62.86	8.75	72.96	8.80	-10.10	3.25E-06	1.34E-04
cg23180489	LYNX1;LYNX1;LYNX1;LYNX1	8	TSS200;TSS1500;TSS200;TSS1500	S_Shore	53.60	11.42	63.70	12.48	-10.10	2.43E-04	5.76E-03
cg16318112	AGRN	1	Body	Island	43.34	9.72	53.42	9.14	-10.09	3.91E-07	1.85E-05
cg09279736	RNF39;RNF39	6	Body;Body	Island	33.78	9.38	43.87	11.01	-10.09	5.06E-06	2.02E-04
cg02072875	IQCE;IQCE	7	Body;Body	Open sea	46.79	7.35	56.87	11.17	-10.08	2.05E-06	8.76E-05
cg14964336		4	Intergenic	Island	24.87	8.41	34.95	12.98	-10.08	3.20E-05	1.06E-03
cg02032299		16	Intergenic	Open sea	22.29	7.21	32.36	9.08	-10.08	1.47E-07	7.25E-06
cg13185413	RNF39;RNF39	6	Body;Body	Island	29.57	10.07	39.65	13.44	-10.08	7.51E-05	2.20E-03
cg09421111	GPR56;GPR56;GPR56;GPR56;GPR56;G	16	5'UTR;TSS200;5'UTR;5'UTR;5'UTR;5'UTR;5	Open sea	28.68	8.29	38.75	13.28	-10.07	5.21E-05	1.61E-03
cg06687102	RARRES1;RARRES1	3	Body;Body	Open sea	62.57	6.35	72.63	6.17	-10.06	5.07E-11	2.95E-09
cg22816343	BNIP3L	8	Body	S_Shelf	44.17	6.50	54.23	5.67	-10.06	8.19E-12	4.88E-10
cg06341731	BCL11B;BCL11B	14	Body;Body	Island	62.47	12.36	72.52	11.10	-10.04	1.07E-03	1.79E-02
cg20995778	NLRP2	19	5'UTR	Open sea	55.76	10.32	65.80	7.29	-10.04	4.58E-06	1.85E-04
cg17033080	IGFBP2	2	Body	Open sea	58.98	7.87	69.02	5.22	-10.04	7.28E-10	4.08E-08
cg21265278	TOR3A	1	Body	Open sea	69.79	8.20	79.82	4.98	-10.04	4.31E-11	2.52E-09
cg08835755		4	Intergenic	Island	52.58	9.06	62.59	7.16	-10.02	6.45E-07	2.97E-05
cg21279207	FLOT1	6	Body	N_Shelf	63.23	5.51	73.24	5.21	-10.01	6.29E-14	3.95E-12
cg09185884	KCTD2	17	Body	Open sea	29.07	7.27	39.08	9.03	-10.01	4.84E-07	2.26E-05
cg27517345	C10orf26;C10orf26;C10orf26	10	5'UTR;Body;1stExon	Open sea	38.90	6.93	48.91	7.03	-10.01	2.47E-08	1.29E-06
cg10670376	ARHGAP26;ARHGAP26	5	Body;Body	Open sea	81.75	8.61	91.74	2.37	-10.00	4.35E-13	2.69E-11
cg26267310	PLEKHA4;HSD17B14;PLEKHA4	19	3'UTR;TSS1500;Body	Island	26.43	9.44	36.43	14.91	-10.00	1.56E-04	4.04E-03
cg17224613		10	Intergenic	Open sea	55.91	10.05	65.90	11.39	-9.99	9.08E-05	2.58E-03
cg13167664	INS-IGF2;IGF2;IGF2;IGF2	11	Body;Body;5'UTR;5'UTR	Island	40.86	8.40	50.85	5.54	-9.99	6.15E-07	2.84E-05
cg00179374	PLXNA4;PLXNA4;PLXNA4	7	5'UTR;5'UTR;5'UTR	N_Shore	49.62	7.72	59.60	8.95	-9.98	1.58E-06	6.87E-05
cg20396510	MAST4;MAST4;MAST4	5	TSS200;Body;Body	Open sea	16.39	6.53	26.37	8.84	-9.98	1.35E-08	7.15E-07
cg09389931		14	Intergenic	S_Shore	62.39	6.66	72.36	4.51	-9.98	1.36E-11	8.05E-10
cg24908097	DSE;DSE	6	Body;Body	Open sea	71.98	6.81	81.96	5.24	-9.97	3.14E-11	1.84E-09
cg24140801		8	Intergenic	S_Shelf	56.45	8.93	66.42	7.20	-9.96	7.03E-07	3.22E-05
cg22870373	MAD1L1;MAD1L1;MAD1L1	7	Body;Body;Body	Island	73.96	12.01	83.92	7.15	-9.96	2.86E-04	6.56E-03
cg04089703	PICK1;PICK1;PICK1	22	3'UTR;3'UTR;3'UTR	S_Shore	69.23	8.70	79.19	7.78	-9.95	6.06E-07	2.80E-05
cg00889398		13	Intergenic	Island	24.25	7.82	34.20	12.15	-9.95	1.23E-06	5.46E-05
cg11858213	CRTAP	3	Body	Open sea	56.63	7.52	66.56	5.23	-9.93	9.88E-10	5.51E-08
cg03471593	LPCAT2	16	TSS1500	N_Shore	61.77	5.49	71.69	4.44	-9.92	1.28E-15	8.41E-14
cg02518546	AFF3;AFF3	2	Body;Body	Open sea	27.63	4.41	37.56	6.15	-9.92	7.85E-13	4.81E-11
cg06570476		19	Intergenic	Island	52.90	8.57	62.81	8.84	-9.91	4.08E-06	1.66E-04
cg15920887	MSTN	2	TSS1500	Open sea	63.69	7.57	73.60	6.58	-9.91	1.92E-08	1.01E-06
cg03894796		8	Intergenic	Island	33.96	7.57	43.84	8.16	-9.88	6.92E-07	3.17E-05
cg06772309	PADI1	1	3'UTR	Open sea	42.99	9.59	52.88	7.96	-9.88	1.41E-06	6.20E-05
cg00351283	RBMXL1;CCBL2;CCBL2;RBMXL1	1	Body;Body;5'UTR;Body	Open sea	44.58	4.43	54.45	5.13	-9.87	1.81E-15	1.18E-13
cg23955161	C6orf145	6	Body	Open sea	65.60	6.08	75.48	5.36	-9.87	4.71E-11	2.75E-09
cg21400851	KCTD2	17	Body	Open sea	58.11	8.16	67.98	7.56	-9.87	9.24E-07	4.17E-05
cg13048147	ATP5H;ATP5H;KCTD2	17	TSS1500;TSS1500;Body	S_Shore	58.79	7.34	68.65	6.25	-9.86	2.11E-08	1.10E-06

cg10441070	ESR1;ESR1	6	5'UTR;TSS1500	N_Shelf	46.67	8.40	56.52	7.84	-9.85	3.35E-06	1.38E-04
cg04605980		12	Intergenic	Open sea	29.19	11.14	39.05	15.26	-9.85	1.39E-03	2.18E-02
cg04021544		7	Intergenic	S_Shelf	40.61	7.49	50.46	8.10	-9.85	5.56E-07	2.58E-05
cg01524174	SOX9	17	Body	Island	17.28	7.09	27.12	8.35	-9.84	1.30E-06	5.75E-05
cg15192736		22	Intergenic	Island	59.62	6.35	69.45	5.39	-9.83	1.07E-10	6.18E-09
cg19502359		11	Intergenic	Open sea	56.58	8.21	66.40	9.80	-9.82	2.32E-06	9.84E-05
cg00585790	LIMS1;LIMS1	2	1stExon;5'UTR	Open sea	28.37	6.27	38.18	5.61	-9.82	3.62E-10	2.05E-08
cg01783051	FH	1	Body	N_Shelf	54.64	4.96	64.45	4.51	-9.82	2.54E-15	1.65E-13
cg22729681	PBX1	1	Body	Open sea	27.41	4.85	37.22	7.44	-9.81	2.98E-12	1.80E-10
cg13630944		10	Intergenic	Open sea	67.46	3.34	77.26	2.92	-9.80	4.36E-24	3.38E-22
cg03310167	DLGAP2	8	Body	N_Shore	58.86	5.72	68.64	7.31	-9.78	6.95E-10	3.90E-08
cg05590818	TLK1;TLK1	2	Body;Body	Open sea	45.88	7.05	55.65	6.67	-9.78	5.29E-08	2.70E-06
cg13918754	RNF39;RNF39	6	Body;Body	Island	10.64	7.01	20.41	12.43	-9.78	3.30E-07	1.58E-05
cg02276807		12	Intergenic	Island	50.75	11.33	60.52	9.96	-9.77	4.05E-05	1.29E-03
cg01140247	C10orf11	10	Body	Open sea	39.93	7.78	49.70	8.23	-9.77	9.92E-07	4.46E-05
cg08401872	DIRC3	2	Body	Open sea	27.13	4.81	36.88	4.96	-9.76	5.41E-14	3.40E-12
cg25674938	MUM1;MUM1	19	Body;Body	S_Shore	53.07	7.09	62.80	6.78	-9.74	4.16E-09	2.26E-07
cg04142864	PHLDB1;PHLDB1;PHLDB1	11	5'UTR;5'UTR;5'UTR	Island	29.05	6.17	38.79	10.98	-9.74	3.48E-07	1.66E-05
cg11680055	GSTM1;GSTM1	1	TSS200;TSS200	Island	31.29	8.85	41.01	12.37	-9.72	1.59E-04	4.12E-03
cg04524477	NBR1;TMEM106A;NBR1;NBR1	17	3'UTR;TSS1500;3'UTR;3'UTR	N_Shore	21.34	5.95	31.06	9.45	-9.72	3.86E-09	2.10E-07
cg16399664	OAS2;OAS2;OAS2	12	Body;Body;Body	Open sea	25.95	10.95	35.64	12.51	-9.69	8.36E-04	1.50E-02
cg06168204		6	Intergenic	Open sea	45.33	9.63	55.02	10.68	-9.69	9.01E-05	2.57E-03
cg10801060	LOC153684	5	Body	S_Shore	52.81	8.45	62.50	11.92	-9.68	2.71E-05	9.15E-04
cg19098465	LTBP4;LTBP4;LTBP4	19	Body;Body;Body	Island	53.75	9.23	63.42	10.43	-9.68	2.92E-05	9.75E-04
cg26631397	GREB1;GREB1;GREB1;GREB1	2	5'UTR;5'UTR;1stExon;5'UTR	Open sea	83.33	11.14	93.00	3.21	-9.67	2.11E-07	1.03E-05
cg15663948	C13orf28;C13orf28	13	1stExon;5'UTR	Open sea	54.71	19.93	64.38	10.96	-9.67	1.44E-03	2.24E-02
cg19323951	SLC22A20	11	Body	N_Shore	12.97	8.08	22.63	12.32	-9.66	4.13E-06	1.68E-04
cg21815337	SETMAR;SETMAR	3	Body;Body	S_Shelf	48.56	6.20	58.21	6.63	-9.65	1.44E-09	8.01E-08
cg00666109	RPS6KC1;RPS6KC1	1	Body;Body	Open sea	48.48	7.29	58.13	7.28	-9.65	9.47E-09	5.05E-07
cg00641052	NOD2	16	Body	Open sea	67.74	9.14	77.38	7.25	-9.65	7.73E-07	3.52E-05
cg15167959		13	Intergenic	Island	24.48	8.47	34.11	12.45	-9.63	2.68E-05	9.05E-04
cg14834903	PAPLN	14	Body	Island	36.94	8.94	46.55	11.79	-9.62	1.64E-05	5.87E-04
cg05404865	GLDN	15	Body	Open sea	53.89	6.99	63.51	5.67	-9.61	2.21E-09	1.22E-07
cg01214012		14	Intergenic	Open sea	38.09	6.42	47.70	11.89	-9.61	3.49E-06	1.43E-04
cg11086982	PAPLN	14	Body	Open sea	17.06	9.67	26.67	13.36	-9.61	1.93E-04	4.80E-03
cg15506703		2	Intergenic	Island	48.28	12.72	57.88	11.55	-9.60	2.19E-04	5.31E-03
cg24432977	CASP5;CASP5;CASP5;CASP5;CA	11	Body;3'UTR;3'UTR;3'UTR;3'UTR;3'UTR	Open sea	58.24	8.56	67.82	9.36	-9.59	5.15E-06	2.06E-04
cg15782311		12	Intergenic	N_Shore	73.22	9.62	82.80	7.41	-9.58	4.45E-06	1.80E-04
cg26607620	DLGAP2	8	Body	Island	45.51	6.85	55.09	9.57	-9.58	9.08E-07	4.10E-05
cg05107535		16	Intergenic	Island	38.58	7.01	48.16	9.44	-9.58	3.49E-07	1.66E-05
cg11560119		3	Intergenic	Open sea	59.10	6.33	68.67	6.18	-9.57	2.41E-09	1.32E-07
cg25452172	METTL1;METTL1	12	3'UTR;3'UTR	N_Shelf	62.03	2.84	71.60	2.28	-9.57	3.22E-28	2.68E-26
cg04852443	PRDM16;PRDM16	1	Body;Body	Island	24.90	8.03	34.47	9.91	-9.57	2.77E-06	1.16E-04
cg05691833	ZCCHC24	10	3'UTR	N_Shelf	59.91	6.22	69.47	5.91	-9.56	9.91E-11	5.73E-09
cg12195314		19	Intergenic	S_Shore	38.19	5.68	47.74	7.81	-9.55	2.99E-11	1.75E-09
cg26993798		16	Intergenic	N_Shelf	53.02	7.82	62.57	7.42	-9.55	2.53E-07	1.22E-05
cg16660494		5	Intergenic	Open sea	53.16	4.96	62.69	6.06	-9.54	1.42E-11	8.39E-10
cg14360448		9	Intergenic	Open sea	60.07	7.51	69.60	7.93	-9.53	1.13E-06	5.04E-05
cg08713365	NRSN2;NRSN2	20	1stExon;5'UTR	Island	34.65	6.35	44.17	5.86	-9.52	9.19E-12	5.48E-10
cg22831238	RASSF3	12	Body	Open sea	65.63	9.55	75.15	8.15	-9.52	1.45E-06	6.37E-05
cg17128417	GDF7	2	TSS1500	N_Shore	23.03	7.04	32.55	8.16	-9.51	2.67E-07	1.28E-05
cg19776833	FOXP1	3	Body	Open sea	82.46	7.41	91.97	2.13	-9.51	9.24E-14	5.79E-12
cg05841263	SPTBN1;SPTBN1	2	Body;Body	S_Shore	56.78	5.36	66.27	5.88	-9.50	1.30E-12	7.93E-11

cg08356028		10	Intergenic	Open sea	43.66	10.81	53.16	14.45	-9.49	2.04E-04	5.02E-03
cg08747889	PTK7;PTK7;PTK7;PTK7	6	TSS1500;TSS1500;TSS1500;TSS1500	N_Shore	30.46	7.01	39.95	7.09	-9.48	1.24E-09	6.90E-08
cg06335597	ZNF467	7	3'UTR	Island	63.20	8.62	72.68	6.97	-9.48	7.20E-08	3.63E-06
cg07175288		18	Intergenic	Island	57.44	8.77	66.92	9.47	-9.48	4.51E-06	1.82E-04
cg00791868	C14orf53;C14orf53	14	Body;Body	Open sea	48.59	6.75	58.06	6.81	-9.48	1.51E-09	8.37E-08
cg14881054		7	Intergenic	Open sea	33.75	6.03	43.22	5.95	-9.47	1.85E-09	1.02E-07
cg17516837		12	Intergenic	Open sea	38.17	7.00	47.61	6.71	-9.44	3.77E-08	1.94E-06
cg15388641	WASF2	1	3'UTR	Open sea	72.09	8.62	81.53	8.24	-9.44	3.18E-06	1.32E-04
cg10774352		6	Intergenic	Open sea	43.90	7.42	53.33	6.24	-9.43	8.96E-09	4.78E-07
cg08879099	FLOT1	6	Body	N_Shelf	82.15	7.03	91.57	3.28	-9.43	5.02E-13	3.09E-11
cg21758446	TSPAN8	12	3'UTR	Open sea	41.37	8.41	50.79	8.83	-9.43	9.61E-06	3.64E-04
cg20227255	SRF	6	Body	Island	11.33	4.40	20.75	8.39	-9.42	1.11E-08	5.91E-07
cg08162473	INS-IGF2;IGF2AS;IGF2;IGF2;IGF2AS;IG	11	Body;TSS1500;Body;5'UTR;TSS1500;TSS15	Island	31.04	7.81	40.46	4.89	-9.42	3.41E-07	1.62E-05
cg11412853		12	Intergenic	N_Shore	40.54	8.70	49.95	7.10	-9.41	8.39E-07	3.81E-05
cg23549902		7	Intergenic	Island	30.84	9.87	40.24	13.56	-9.40	2.05E-03	2.88E-02
cg17335108		17	Intergenic	S_Shelf	42.07	6.14	51.47	5.53	-9.40	3.03E-10	1.73E-08
cg25002649	CUL7;CUL7;MRPL2	6	TSS1500;TSS1500;Body	S_Shore	49.02	8.45	58.41	8.43	-9.40	6.45E-06	2.52E-04
cg09994022	STK24;STK24	13	Body;Body	Island	58.36	9.16	67.76	10.18	-9.40	4.48E-05	1.41E-03
cg01099855	SRGAP1	12	Body	Open sea	58.29	7.76	67.69	7.04	-9.39	1.52E-09	8.39E-08
cg26179400	FMNL2	2	Body	Open sea	64.88	15.99	74.27	9.15	-9.39	3.02E-03	3.76E-02
cg13583535	MBP;MBP	18	Body;Body	N_Shelf	43.43	7.65	52.82	4.64	-9.39	2.89E-09	1.58E-07
cg07522644	SRF	6	Body	Island	11.27	4.47	20.66	8.17	-9.39	5.25E-09	2.84E-07
cg15848095		4	Intergenic	Open sea	62.77	6.55	72.15	8.17	-9.38	1.13E-09	6.29E-08
cg14527250	WNT5B;WNT5B	12	Body;Body	N_Shore	31.00	7.59	40.38	9.27	-9.38	2.55E-06	1.07E-04
cg24637044	RNF39;RNF39	6	Body;Body	Island	44.86	8.95	54.24	12.51	-9.38	1.88E-05	6.63E-04
cg25970575		4	Intergenic	S_Shore	37.46	9.17	46.83	9.37	-9.37	4.26E-06	1.73E-04
cg17182817		10	Intergenic	Open sea	31.12	8.06	40.49	8.31	-9.37	2.02E-06	8.67E-05
cg25551714		11	Intergenic	S_Shore	22.71	7.13	32.08	9.25	-9.37	1.84E-07	8.96E-06
cg02884928	AKT1;AKT1;AKT1	14	Body;Body;Body	Island	55.48	5.77	64.84	5.31	-9.37	1.44E-10	8.29E-09
cg03461110	FOXK1	7	Body	Open sea	18.90	5.75	28.24	8.89	-9.34	3.70E-08	1.91E-06
cg22396555		13	Intergenic	Island	25.08	8.77	34.41	12.91	-9.33	9.51E-05	2.69E-03
cg25075347	EYA4;EYA4;EYA4	6	Body;Body;Body	Open sea	41.66	11.05	50.99	13.90	-9.33	4.85E-04	9.89E-03
cg26762347	PRDM16;PRDM16	1	Body;Body	Island	19.79	9.23	29.12	11.18	-9.32	9.87E-05	2.77E-03
cg20766090	INS-IGF2;IGF2;IGF2;IGF2	11	Body;Body;5'UTR;5'UTR	Island	21.53	6.25	30.84	6.94	-9.32	1.19E-08	6.33E-07
cg11054774	PNPLA2	11	Body	Island	70.90	7.42	80.21	7.88	-9.31	3.33E-07	1.59E-05
cg03900241		5	Intergenic	Open sea	47.50	7.86	56.81	8.89	-9.31	6.02E-06	2.37E-04
cg09592903	RTN4;RTN4;RTN4;RTN4;RTN4	2	Body;Body;Body;Body;Body	Open sea	39.50	8.53	48.80	12.91	-9.30	2.91E-05	9.73E-04
cg06152526	PLLP	16	3'UTR	Open sea	70.38	4.49	79.67	3.21	-9.29	9.64E-19	6.76E-17
cg27258399	HTRA4	8	Body	Island	49.95	6.88	59.23	8.28	-9.29	3.85E-08	1.98E-06
cg25538627	USP2;USP2	11	Body;Body	Island	19.29	7.78	28.57	9.20	-9.28	1.36E-05	4.95E-04
cg16916914	ITGB4;ITGB4;ITGB4	17	Body;Body;Body	Island	12.90	7.65	22.18	12.40	-9.28	2.07E-05	7.21E-04
cg26204079	DGKD	2	TSS1500	Open sea	30.54	5.91	39.80	6.57	-9.26	2.59E-09	1.42E-07
cg05507514	SGEF	3	Body	S_Shelf	70.10	7.44	79.35	5.79	-9.25	3.28E-07	1.57E-05
cg17139085		1	Intergenic	Island	50.41	8.05	59.66	8.98	-9.24	2.95E-07	1.42E-05
cg20295353	ZBTB1;ZBTB1	14	Body;Body	Open sea	61.56	7.53	70.81	5.36	-9.24	6.92E-09	3.71E-07
cg22242857	SALL3	18	Body	Island	55.38	13.46	64.62	14.52	-9.24	3.61E-03	4.25E-02
cg10650127	IGF2;INS-IGF2;INS-IGF2	11	TSS200;Body;Body	Open sea	54.90	7.36	64.13	6.80	-9.23	2.87E-07	1.38E-05
cg08052226	NFIX	19	3'UTR	N_Shore	36.64	7.50	45.87	8.75	-9.23	2.15E-06	9.19E-05
cg15031253	NLRP2	19	TSS1500	Open sea	80.10	15.54	89.33	6.86	-9.23	5.05E-05	1.57E-03
cg09110274	CACNA2D4	12	TSS1500	Open sea	54.17	8.00	63.39	6.24	-9.22	4.69E-07	2.20E-05
cg01070987	PFN2;PFN2	3	Body;Body	N_Shore	43.91	9.41	53.13	8.29	-9.22	9.16E-05	2.60E-03
cg18177275		14	Intergenic	Open sea	36.48	7.12	45.70	8.04	-9.22	1.20E-06	5.34E-05
cg05132828	PITPNM2	12	5'UTR	Open sea	50.34	8.33	59.55	7.68	-9.22	1.73E-06	7.47E-05

cg18254356	LOC153684	5	Body	S_Shore	39.37	8.13	48.58	13.19	-9.21	2.14E-04	5.21E-03
cg12211856	SDCCAG8	1	Body	Island	13.51	6.20	22.72	12.08	-9.21	1.36E-06	5.99E-05
cg11153485	IRS2	13	Body	N_Shelf	43.18	8.76	52.38	9.44	-9.20	1.77E-05	6.27E-04
cg11807188	LEPR;LEPR;LEPR	1	5'UTR;5'UTR;5'UTR	Open sea	50.12	4.85	59.32	4.44	-9.20	4.33E-14	2.74E-12
cg17502312	C10orf18	10	Body	Open sea	66.84	6.99	76.04	8.84	-9.20	4.92E-07	2.30E-05
cg20810195		1	Intergenic	Open sea	60.66	5.95	69.86	3.62	-9.20	3.68E-13	2.28E-11
cg04231636	KDM1B	6	Body	Open sea	32.02	10.74	41.20	13.59	-9.18	3.85E-03	4.45E-02
cg14059464		2	Intergenic	N_Shelf	42.62	8.80	51.79	10.43	-9.17	4.75E-06	1.91E-04
cg06472166		7	Intergenic	Open sea	51.56	5.22	60.72	4.91	-9.15	7.12E-14	4.47E-12
cg07073999	HS3ST3B1	17	Body	S_Shore	28.56	5.73	37.70	7.76	-9.15	3.35E-08	1.73E-06
cg09050832		8	Intergenic	Island	57.58	12.14	66.72	11.37	-9.14	5.18E-04	1.04E-02
cg02254407	PLEKHB1;PLEKHB1;PLEKHB1;PLEKHB1	11	TSS200;TSS200;TSS1500;TSS1500	Open sea	32.53	8.22	41.67	11.41	-9.14	1.73E-05	6.14E-04
cg09784932	RTN4R	22	Body	Island	53.14	7.82	62.27	10.00	-9.13	5.76E-05	1.76E-03
cg23520347	RASSF5;RASSF5;RASSF5;RASSF5	1	Body;5'UTR;Body;1stExon	Island	59.25	7.50	68.38	5.07	-9.13	6.99E-11	4.06E-09
cg21497139		3	Intergenic	Open sea	63.83	7.16	72.93	7.67	-9.10	6.40E-07	2.95E-05
cg27654966	CUX1;CUX1;CUX1	7	Body;Body;Body	N_Shore	49.91	7.52	59.00	8.94	-9.10	4.85E-06	1.95E-04
cg09859040	ARNT2	15	Body	Open sea	37.56	6.50	46.65	9.59	-9.09	1.09E-06	4.86E-05
cg06359931	LDLRAP1	1	Body	Open sea	47.33	5.39	56.42	6.67	-9.09	2.59E-09	1.42E-07
cg06688733		5	Intergenic	Open sea	46.62	8.74	55.71	11.27	-9.08	8.89E-05	2.54E-03
cg24420574	MAD1L1;MAD1L1;MAD1L1	7	Body;Body;Body	N_Shelf	62.24	8.81	71.33	9.01	-9.08	1.53E-05	5.52E-04
cg18479711	HDAC4	2	Body	N_Shore	79.73	9.14	88.82	5.73	-9.08	5.07E-07	2.37E-05
cg16360836	HDAC4	2	Body	Open sea	37.12	5.51	46.19	6.74	-9.07	7.12E-10	3.99E-08
cg08643852	MED4	13	TSS1500	S_Shore	48.52	6.41	57.59	5.65	-9.07	5.09E-10	2.87E-08
cg12167284	SNORA11B;C14orf159;C14orf159;C14	14	Body;5'UTR;5'UTR;5'UTR;5'UTR;5'UTR	Open sea	70.76	8.78	79.82	5.33	-9.06	2.56E-06	1.08E-04
cg23994511	SMNDC1	10	Body	N_Shelf	65.48	7.59	74.53	7.08	-9.05	4.93E-06	1.98E-04
cg14562054	RIN1	11	TSS1500	S_Shelf	39.78	5.70	48.83	4.77	-9.05	8.14E-11	4.72E-09
cg14295611		9	Intergenic	N_Shore	70.23	11.03	79.28	6.00	-9.05	2.82E-06	1.18E-04
cg20564892	KCNIP2;KCNIP2;KCNIP2;KCNIP2;KCNIP	10	1stExon;5'UTR;1stExon;1stExon;1stExon;5	Island	19.10	7.13	28.15	12.12	-9.05	1.14E-04	3.12E-03
cg25010691	IQCE;IQCE	7	Body;Body	Open sea	48.23	8.82	57.26	10.35	-9.03	1.03E-04	2.86E-03
cg05289983		7	Intergenic	Open sea	50.88	6.08	59.90	6.77	-9.02	2.01E-10	1.15E-08
cg01921126	IGF2;INS-IGF2;INS-IGF2	11	TSS200;Body;Body	Open sea	47.37	7.94	56.39	7.76	-9.02	3.58E-07	1.70E-05
cg23435915	LOC153684	5	Body	S_Shore	39.36	9.16	48.37	13.46	-9.01	7.25E-04	1.35E-02
cg14400030	BCL7A;BCL7A	12	3'UTR;3'UTR	Open sea	51.27	9.34	60.28	6.79	-9.01	1.92E-05	6.74E-04
cg13521170	BCL2	18	3'UTR	Open sea	43.33	6.51	52.33	6.58	-9.00	4.00E-08	2.05E-06
cg23356017	SDC2	8	Body	S_Shelf	59.68	7.49	68.68	9.18	-9.00	6.95E-06	2.70E-04
cg11299991	PTX3;VEPH1;VEPH1;PTX3;VEPH1	3	5'UTR;Body;Body;1stExon;Body	N_Shore	33.17	7.37	42.17	9.54	-9.00	6.53E-06	2.55E-04
cg14530382	SH2D3A	19	Body	Island	18.21	7.46	27.20	10.73	-8.99	8.94E-06	3.40E-04
cg24442740	AHDC1	1	5'UTR	Island	16.64	6.19	25.62	7.22	-8.99	3.82E-07	1.81E-05
cg26565893	CASKIN2;CASKIN2	17	Body;Body	Island	61.47	6.56	70.45	5.88	-8.98	1.90E-08	9.98E-07
cg13690590		19	Intergenic	N_Shore	55.96	7.03	64.94	7.04	-8.98	3.06E-07	1.46E-05
cg19713109	BAT5	6	Body	N_Shore	64.56	6.37	73.54	5.63	-8.98	6.46E-10	3.63E-08
cg03684807		22	Intergenic	Island	37.56	7.41	46.53	11.43	-8.97	3.80E-05	1.22E-03
cg08400124	SHROOM3	4	Body	Island	23.50	6.95	32.46	8.95	-8.97	2.28E-07	1.10E-05
cg27338109		16	Intergenic	Open sea	60.42	6.66	69.38	5.94	-8.96	3.01E-08	1.56E-06
cg02360445		10	Intergenic	Island	60.69	9.19	69.65	12.25	-8.96	6.09E-04	1.18E-02
cg02395846	C1QL1	17	3'UTR	Island	32.21	7.86	41.16	11.83	-8.95	8.33E-05	2.40E-03
cg23719318	GDF7	2	TSS1500	Island	13.93	6.77	22.88	11.92	-8.95	3.07E-05	1.02E-03
cg21518268		8	Intergenic	Open sea	48.09	6.94	57.04	8.35	-8.95	9.14E-07	4.13E-05
cg23842109		2	Intergenic	Open sea	33.22	7.87	42.17	9.55	-8.95	1.13E-05	4.20E-04
cg11174255		4	Intergenic	N_Shore	83.24	12.11	92.19	4.09	-8.94	1.07E-04	2.96E-03
cg26004771		1	Intergenic	Island	45.81	8.53	54.75	10.22	-8.94	9.80E-06	3.70E-04
cg26352676		11	Intergenic	N_Shore	25.55	7.80	34.49	8.39	-8.94	1.86E-06	8.03E-05
cg22050253	SHROOM3	4	Body	Island	12.55	6.73	21.49	8.58	-8.94	4.63E-07	2.17E-05

cg00129651	RAPGEFL1	17	Body	Island	6.62	3.09	15.56	8.73	-8.93	1.98E-10	1.13E-08
cg13521620	YPEL2	17	5'UTR	S_Shore	34.92	5.67	43.85	8.56	-8.93	2.24E-07	1.09E-05
cg03338209	CARD10	22	Body	N_Shelf	52.03	7.90	60.94	7.40	-8.91	8.65E-07	3.92E-05
cg00216138	LOC145845	15	Body	N_Shore	40.29	7.29	49.20	8.43	-8.91	1.81E-07	8.85E-06
cg19387165		15	Intergenic	Open sea	62.38	7.08	71.28	9.77	-8.90	4.54E-06	1.83E-04
cg24809529	CLDN15	7	TSS200	Open sea	35.99	7.27	44.88	7.61	-8.90	4.52E-07	2.12E-05
cg19919217	THRA	17	Body	N_Shore	58.50	6.89	67.39	9.31	-8.90	2.30E-06	9.77E-05
cg00423364	TSPAN4;TSPAN4;TSPAN4;TSPAN4;TSP	11	5'UTR;Body;Body;Body;Body;Body;Body	Island	77.76	8.50	86.65	6.39	-8.89	1.57E-07	7.70E-06
cg02802072	SLC38A10;SLC38A10	17	Body;Body	Open sea	34.90	7.83	43.79	7.80	-8.89	5.99E-06	2.36E-04
cg08767686		5	Intergenic	Island	63.43	11.33	72.32	8.77	-8.89	3.43E-05	1.12E-03
cg04854226		15	Intergenic	N_Shore	44.28	8.59	53.17	10.76	-8.89	5.91E-05	1.80E-03
cg17341174	BAIAP2L1	7	Body	Open sea	43.11	7.47	51.99	8.71	-8.89	5.64E-06	2.23E-04
cg07244980	CECR7	22	TSS1500	N_Shore	58.24	13.66	67.12	5.56	-8.88	5.95E-04	1.16E-02
cg21046874		13	Intergenic	N_Shore	19.46	8.00	28.34	10.98	-8.88	8.30E-05	2.39E-03
cg05129568	FLOT1	6	Body	N_Shelf	79.74	6.48	88.61	4.14	-8.87	3.19E-12	1.92E-10
cg00253171	WWOX;WWOX	16	Body;Body	Open sea	69.89	6.85	78.76	6.58	-8.87	4.14E-09	2.25E-07
cg25322094	FSTL4	5	Body	Open sea	60.51	6.25	69.38	9.74	-8.87	6.01E-07	2.78E-05
cg17284236	ZNF490	19	Body	Island	49.47	10.51	58.33	14.36	-8.86	4.51E-03	4.95E-02
cg03716852	TRPM4	19	Body	Island	50.70	8.98	59.56	9.50	-8.86	1.91E-04	4.76E-03
cg00454862		1	Intergenic	Open sea	64.23	10.51	73.09	8.99	-8.86	1.78E-03	2.61E-02
cg04455759	TIMM8B;SDHD;TIMM8B	11	Body;TSS1500;Body	N_Shore	85.09	2.74	93.93	1.20	-8.85	6.81E-37	6.74E-35
cg24764793	ESR1;ESR1	6	5'UTR;TSS200	N_Shelf	44.82	7.34	53.67	7.99	-8.84	1.46E-06	6.38E-05
cg00179906	HS3ST3B1;MGC12916	17	Body;Body	S_Shore	61.29	6.39	70.13	4.97	-8.84	2.14E-10	1.23E-08
cg07679948	DGKA;DGKA;DGKA;DGKA	12	5'UTR;5'UTR;5'UTR;5'UTR	Island	34.22	6.72	43.06	9.19	-8.84	5.27E-07	2.45E-05
cg24848192	RFPL2;RFPL2	22	TSS1500;TSS200	Open sea	45.71	10.92	54.55	10.55	-8.84	9.97E-05	2.80E-03
cg17137457	CPT1B;CPT1B;CPT1B;CPT1B;CHKB-CPT	22	TSS200;TSS200;TSS1500;TSS1500;Body;TS	S_Shore	45.68	9.22	54.52	8.47	-8.84	4.94E-05	1.54E-03
cg20194314		13	Intergenic	Island	14.24	6.58	23.07	9.77	-8.84	2.29E-07	1.11E-05
cg22016731	TRIT1	1	Body	Open sea	78.28	5.93	87.12	3.16	-8.84	1.70E-15	1.12E-13
cg22629528	OPCML	11	Body	Island	51.62	10.51	60.45	12.00	-8.83	7.94E-04	1.44E-02
cg11801727		1	Intergenic	Island	68.71	10.83	77.54	9.36	-8.83	9.12E-05	2.59E-03
cg10880163		11	Intergenic	S_Shelf	43.62	8.64	52.44	9.51	-8.82	1.25E-05	4.61E-04
cg25513635	GDF1;LASS1	19	5'UTR;3'UTR	S_Shore	21.99	7.48	30.81	11.26	-8.82	8.62E-06	3.29E-04
cg14181271	MAML3	4	Body	Open sea	57.76	8.53	66.57	7.42	-8.81	1.51E-06	6.58E-05
cg23306063	TYK2	19	Body	Island	37.57	8.06	46.38	12.68	-8.81	4.24E-04	8.91E-03
cg16051352	C14orf73	14	Body	S_Shelf	53.28	7.23	62.09	8.58	-8.81	1.36E-06	5.99E-05
cg10636490	PFN2;PFN2	3	Body;Body	N_Shore	46.00	8.40	54.81	6.99	-8.81	1.78E-05	6.31E-04
cg04934382		17	Intergenic	Island	37.19	7.93	45.99	11.64	-8.81	9.16E-05	2.60E-03
cg10930308	RNF39;RNF39	6	Body;Body	Island	22.65	7.84	31.46	12.51	-8.80	4.72E-05	1.48E-03
cg03180953	IGFALS;IGFALS;IGFALS	16	Body;Body;Body	Island	63.53	8.95	72.33	10.89	-8.80	3.15E-04	7.07E-03
cg13163919	TLE3;TLE3;TLE3	15	Body;Body;Body	Island	46.95	7.90	55.74	6.40	-8.80	1.62E-07	7.92E-06
cg03553386	IGF2;INS-IGF2;INS-IGF2	11	TSS200;Body;Body	Open sea	50.49	6.21	59.29	6.88	-8.80	2.63E-08	1.37E-06
cg01630691	BMPR2	2	Body	Open sea	55.30	8.64	64.09	7.06	-8.79	3.78E-06	1.55E-04
cg22738440	CLU;CLU;CLU	8	Body;Body;Body	Open sea	71.69	7.15	80.48	8.03	-8.79	7.50E-07	3.43E-05
cg25123470	KCNIP2;KCNIP2;KCNIP2;KCNIP2;KCNIP	10	TSS200;TSS200;TSS200;TSS200;TSS200;TS	S_Shore	13.37	4.42	22.16	9.25	-8.79	2.08E-09	1.15E-07
cg25434864		8	Intergenic	Open sea	60.84	14.77	69.63	11.89	-8.79	1.02E-03	1.73E-02
cg25028308		6	Intergenic	Open sea	37.56	6.73	46.35	9.56	-8.79	8.15E-06	3.13E-04
cg24869272	TSPAN4;TSPAN4;TSPAN4;TSPAN4;TSP	11	5'UTR;5'UTR;5'UTR;5'UTR;5'UTR;5'UTR;5'U	Island	52.52	8.96	61.30	8.81	-8.78	1.52E-05	5.48E-04
cg00226822		4	Intergenic	N_Shelf	63.09	5.37	71.88	5.37	-8.78	3.00E-11	1.76E-09
cg18990313	HTRA4	8	TSS200	Island	24.14	6.74	32.92	11.09	-8.78	8.50E-06	3.25E-04
cg26504110	LTBP4;LTBP4;LTBP4	19	Body;Body;Body	Island	28.16	7.84	36.94	11.46	-8.78	2.00E-04	4.93E-03
cg02165670	GPC6	13	Body	Open sea	65.20	8.05	73.98	6.80	-8.77	2.98E-08	1.54E-06
cg27298324		1	Intergenic	S_Shelf	43.85	5.17	52.62	8.61	-8.77	7.02E-08	3.54E-06
cg09084320	NRP1;NRP1;NRP1	10	Body;Body;Body	Open sea	51.97	5.68	60.73	8.91	-8.76	4.03E-10	2.28E-08

cg10143960	TTLL10	1	5'UTR	Island	31.95	11.39	40.72	13.64	-8.76	3.65E-03	4.28E-02
cg01406506	HTR4;HTR4;HTR4;HTR4;HTR4;HTR4;HT	5	Body;Body;Body;Body;Body;Body;Body	Open sea	56.09	6.08	64.84	7.12	-8.75	4.93E-08	2.52E-06
cg21480173	TYK2	19	Body	Island	49.34	9.28	58.10	13.79	-8.75	2.20E-03	3.02E-02
cg18077387		15	Intergenic	Open sea	57.42	8.04	66.17	9.62	-8.75	1.10E-05	4.10E-04
cg12058490	A2M	12	1stExon	Open sea	39.02	9.69	47.77	9.85	-8.75	1.59E-04	4.10E-03
cg23725583	ENPP2;ENPP2;ENPP2	8	Body;Body;Body	Open sea	78.89	7.03	87.63	3.59	-8.74	5.18E-10	2.92E-08
cg14793137	TPO;TPO;TPO;TPO	2	Body;Body;Body;Body	Island	57.58	8.61	66.32	9.79	-8.74	3.36E-05	1.10E-03
cg01604138	NADSYN1	11	Body	Open sea	66.66	5.86	75.40	5.05	-8.73	7.36E-10	4.12E-08
cg21184369	HTRA4;PLEKHA2	8	TSS1500;3'UTR	N_Shore	45.67	9.71	54.40	12.03	-8.73	4.62E-04	9.53E-03
cg17573292	SOD3	4	Body	Island	21.79	6.83	30.52	9.63	-8.73	2.81E-06	1.17E-04
cg26499089		10	Intergenic	Open sea	52.00	7.51	60.73	9.61	-8.73	4.26E-05	1.35E-03
cg00481725		15	Intergenic	Open sea	46.71	7.85	55.42	10.75	-8.71	1.51E-04	3.94E-03
cg06309788	LHFP	13	Body	Open sea	59.01	9.75	67.72	6.27	-8.71	6.43E-07	2.96E-05
cg16262201	PRDM16;PRDM16	1	Body;Body	N_Shore	57.09	5.99	65.80	7.83	-8.71	2.70E-07	1.30E-05
cg06402330	PCOLCE;PCOLCE	7	1stExon;5'UTR	N_Shore	43.76	6.77	52.47	9.03	-8.70	4.03E-07	1.90E-05
cg19405883	RAI14;RAI14;RAI14;RAI14;RAI14;RAI1	5	TSS200;Body;5'UTR;Body;Body;Body	Open sea	48.68	5.32	57.38	7.24	-8.70	1.19E-08	6.29E-07
cg17200768		13	Intergenic	Island	12.62	7.44	21.32	11.66	-8.70	1.69E-05	6.02E-04
cg17280106		17	Intergenic	Island	37.08	7.64	45.78	10.29	-8.70	1.79E-05	6.34E-04
cg14939082	C10orf26;C10orf26;C10orf26	10	5'UTR;Body;1stExon	Open sea	23.02	7.81	31.72	9.21	-8.70	3.48E-05	1.13E-03
cg14252059	TECTA	11	TSS200	Open sea	80.16	8.15	88.86	2.79	-8.70	1.93E-10	1.11E-08
cg20557688	PID1;PID1	2	5'UTR;Body	N_Shelf	42.24	7.37	50.93	8.84	-8.69	1.83E-05	6.47E-04
cg20890325	CTBP1;CTBP1	4	Body;5'UTR	N_Shelf	79.18	10.42	87.87	5.22	-8.69	3.13E-05	1.04E-03
cg12344107		11	Intergenic	Open sea	51.47	8.20	60.16	8.38	-8.69	3.73E-06	1.53E-04
cg10241812	DNAH6	2	Body	Open sea	58.31	6.51	66.99	7.03	-8.68	9.84E-08	4.91E-06
cg00947782	RNF39;RNF39	6	Body;Body	Island	16.83	8.99	25.51	11.71	-8.68	2.46E-04	5.83E-03
cg00716579	MIR181C;MIR181D	19	TSS1500;TSS1500	S_Shore	35.79	7.14	44.46	9.69	-8.68	2.49E-05	8.49E-04
cg09567180	FAM24B	10	Body	Open sea	69.84	6.66	78.51	5.10	-8.67	1.50E-08	7.93E-07
cg06381803	EML2	19	Body	Island	42.79	6.92	51.46	7.36	-8.67	1.20E-07	5.92E-06
cg12020396	EPS8L1;EPS8L1	19	Body;Body	Island	11.45	4.32	20.11	8.68	-8.66	2.90E-09	1.59E-07
cg20524889	KLF5	13	Body	Open sea	56.39	8.25	65.05	8.57	-8.66	1.19E-05	4.40E-04
cg10953436	C14orf49	14	Body	Open sea	74.07	7.37	82.73	4.41	-8.66	1.97E-08	1.03E-06
cg22152887	FCHSD2	11	Body	N_Shelf	64.19	7.06	72.85	7.69	-8.66	1.47E-06	6.43E-05
cg09423312	C7orf50;C7orf50;C7orf50	7	Body;Body;Body	Open sea	29.45	9.48	38.10	11.36	-8.65	8.48E-04	1.51E-02
cg17298275	ABR;ABR;ABR	17	Body;Body;Body	Island	44.75	7.65	53.40	10.82	-8.65	2.25E-04	5.43E-03
cg19086603	F7;F7	13	Body;Body	S_Shore	80.77	17.77	89.41	10.79	-8.63	1.89E-03	2.72E-02
cg10688297	IQCE;IQCE	7	Body;Body	Open sea	54.55	8.24	63.18	10.16	-8.63	1.23E-04	3.33E-03
cg22699026	SDCCAG8	1	Body	Island	9.58	5.36	18.21	11.69	-8.63	2.76E-06	1.16E-04
cg07537095		11	Intergenic	Open sea	11.58	5.22	20.21	7.93	-8.63	3.06E-08	1.58E-06
cg13629753	GBP2;GBP2	1	1stExon;5'UTR	Open sea	24.20	8.04	32.82	11.00	-8.62	1.32E-08	6.98E-07
cg08131184		19	Intergenic	Island	48.90	7.67	57.53	9.28	-8.62	1.95E-05	6.83E-04
cg10735582	TSC22D2	3	Body	Open sea	48.31	6.26	56.92	5.50	-8.61	1.09E-10	6.28E-09
cg00762003	AGPAT3;AGPAT3	21	Body;Body	Island	72.07	13.34	80.67	10.82	-8.61	1.34E-03	2.13E-02
cg12845177	ADAMTS10	19	Body	Island	86.29	8.95	94.90	3.68	-8.61	4.79E-09	2.59E-07
cg19989043	CD2AP	6	Body	Open sea	40.27	7.44	48.87	6.43	-8.60	3.06E-06	1.27E-04
cg16663419	PLEC1;PLEC1	8	Body;Body	Open sea	57.17	9.73	65.77	10.86	-8.60	2.75E-04	6.36E-03
cg05377512	PHF13	1	Body	N_Shelf	49.05	9.14	57.65	11.15	-8.60	1.35E-03	2.14E-02
cg04840051	RBM20	10	Body	Open sea	46.12	5.37	54.72	5.20	-8.60	3.92E-11	2.29E-09
cg23163573	SULT1C2;SULT1C2;SULT1C2;SULT1C2	2	5'UTR;1stExon;1stExon;5'UTR	Open sea	44.90	5.49	53.49	7.28	-8.59	2.41E-09	1.32E-07
cg13153708		14	Intergenic	Open sea	57.62	7.27	66.20	6.23	-8.58	3.13E-07	1.50E-05
cg10936995	SEC62	3	Body	S_Shelf	44.63	6.58	53.21	5.80	-8.58	7.05E-08	3.56E-06
cg08082389	C14orf135	14	5'UTR	S_Shelf	62.88	4.25	71.45	3.87	-8.57	1.07E-15	7.04E-14
cg00436969	RESP18	2	Body	S_Shore	52.33	9.94	60.89	9.80	-8.57	8.56E-05	2.46E-03
cg16302790	PDX1	13	Body	Island	13.24	7.50	21.80	12.61	-8.56	2.21E-04	5.36E-03

cg25558761	GPR132	14	5'UTR	Open sea	54.93	8.92	63.49	12.00	-8.56	7.14E-04	1.33E-02
cg03083695	ST6GALNAC6	9	Body	N_Shore	44.39	8.85	52.95	12.04	-8.56	1.77E-03	2.59E-02
cg04959572	MN1	22	3'UTR	Open sea	53.80	6.84	62.36	7.09	-8.56	3.64E-08	1.88E-06
cg03962421	UBE3C	7	Body	Open sea	42.76	9.29	51.31	10.41	-8.55	4.48E-04	9.31E-03
cg01550828	RNF168	3	Body	N_Shelf	46.61	11.34	55.16	12.16	-8.55	2.17E-03	2.99E-02
cg23602058	ITGA3;ITGA3	17	Body;Body	Open sea	37.89	8.58	46.44	11.10	-8.55	2.33E-04	5.59E-03
cg02245875	DSTN;DSTN	20	Body;5'UTR	S_Shore	59.56	6.71	68.10	6.42	-8.55	3.25E-08	1.68E-06
cg26135172		3	Intergenic	Open sea	27.67	6.10	36.21	6.60	-8.54	8.81E-10	4.92E-08
cg08091601	EIF3L	22	Body	S_Shelf	42.10	7.53	50.64	6.25	-8.54	8.05E-07	3.66E-05
cg07642499	ANO1;ANO1	11	Body;Body	Open sea	57.57	6.45	66.10	7.97	-8.54	8.61E-07	3.90E-05
cg12727374	MAP6D1	3	Body	Island	24.54	7.63	33.07	7.91	-8.53	4.61E-07	2.16E-05
cg09728102	MAST4;MAST4;MAST4	5	TSS200;Body;Body	Open sea	17.19	6.06	25.72	7.76	-8.53	5.88E-08	2.99E-06
cg03847896		1	Intergenic	Open sea	61.69	11.26	70.22	9.66	-8.53	8.18E-04	1.47E-02
cg03758241	COL27A1	9	Body	N_Shore	67.35	7.79	75.87	5.07	-8.53	2.38E-07	1.15E-05
cg27561099	MAP3K13	3	5'UTR	Open sea	35.97	7.98	44.48	8.94	-8.51	6.80E-05	2.02E-03
cg26283564	SGCE;SGCE;PEG10;SGCE;PEG10	7	Body;Body;TSS1500;Body;TSS1500	N_Shore	30.57	7.11	39.08	7.11	-8.51	1.52E-07	7.48E-06
cg12339131		15	Intergenic	N_Shelf	82.20	13.28	90.71	5.97	-8.50	5.58E-04	1.10E-02
cg21032008	GRM7;GRM7	3	Body;Body	Open sea	37.15	6.78	45.65	6.62	-8.50	1.50E-08	7.91E-07
cg02215171	HERC5	4	Body	S_Shore	32.13	5.66	40.63	6.38	-8.50	5.68E-09	3.06E-07
cg17021218	AGAP11	10	5'UTR	Island	22.97	8.51	31.46	8.30	-8.49	1.27E-05	4.67E-04
cg20728696	INS-IGF2;IGF2;IGF2;IGF2	11	Body;Body;5'UTR;5'UTR	N_Shore	14.25	5.60	22.74	8.13	-8.49	9.04E-07	4.09E-05
cg14913216	PLEC1;PLEC1	8	Body;Body	Open sea	58.51	7.10	67.00	9.15	-8.49	6.61E-06	2.58E-04
cg13213165	SGIP1	1	Body	Open sea	52.52	7.45	61.00	10.19	-8.49	6.42E-05	1.93E-03
cg14104148	DCPS	11	Body	Open sea	54.85	8.68	63.34	9.76	-8.49	2.08E-04	5.09E-03
cg10037072	POU6F2;POU6F2	7	Body;Body	Open sea	53.50	10.82	61.99	10.20	-8.48	8.62E-05	2.47E-03
cg09215588		12	Intergenic	Open sea	68.89	8.85	77.37	5.57	-8.48	1.28E-05	4.70E-04
cg26050906	GRB10;GRB10;GRB10	7	5'UTR;TSS200;TSS200	Open sea	56.15	7.17	64.63	7.69	-8.48	2.98E-06	1.24E-04
cg14467654	MIR219-2	9	TSS1500	Island	62.08	9.13	70.56	11.66	-8.48	2.98E-04	6.77E-03
cg06883496		13	Intergenic	Island	25.63	8.07	34.11	11.56	-8.48	3.48E-05	1.14E-03
cg12681948		3	Intergenic	Island	44.66	7.11	53.12	8.02	-8.47	1.60E-07	7.83E-06
cg13695535	MAST4;MAST4;MAST4;MAST4	5	1stExon;Body;Body;5'UTR	Open sea	19.24	6.00	27.71	7.01	-8.47	6.37E-08	3.23E-06
cg23192683		3	Intergenic	S_Shore	38.37	7.04	46.83	7.28	-8.47	1.20E-07	5.94E-06
cg13537646		3	Intergenic	Open sea	41.07	6.55	49.54	7.43	-8.46	2.42E-08	1.26E-06
cg21895319	MED12L	3	Body	Open sea	52.03	7.14	60.49	8.22	-8.46	2.54E-06	1.07E-04
cg02928928	MRPL23	11	Body	S_Shore	69.91	6.44	78.37	5.20	-8.45	3.73E-09	2.03E-07
cg26475911	KCTD2	17	Body	Open sea	27.83	7.84	36.27	8.71	-8.44	2.25E-05	7.76E-04
cg26095395		4	Intergenic	Island	48.86	6.44	57.31	6.45	-8.44	7.66E-08	3.85E-06
cg17589056		13	Intergenic	Open sea	41.22	7.03	49.66	7.25	-8.44	2.25E-06	9.56E-05
cg20366479	IGFBP2	2	Body	S_Shelf	55.35	11.35	63.79	8.57	-8.44	8.32E-04	1.49E-02
cg03538383	GIT2;GIT2;GIT2;GIT2;GIT2;GIT2	12	Body;Body;Body;Body;Body;Body	Open sea	65.74	9.06	74.17	6.01	-8.44	7.45E-06	2.88E-04
cg27544294		22	Intergenic	S_Shore	62.65	4.33	71.08	3.99	-8.43	2.14E-14	1.36E-12
cg01708977		2	Intergenic	Open sea	53.98	5.72	62.40	6.75	-8.42	8.61E-09	4.60E-07
cg06133897		12	Intergenic	Open sea	31.37	6.50	39.80	7.16	-8.42	2.05E-07	9.96E-06
cg09998597		7	Intergenic	Island	57.45	4.83	65.87	4.52	-8.42	1.09E-12	6.63E-11
cg14491362		14	Intergenic	S_Shelf	45.02	6.67	53.44	8.82	-8.42	4.09E-06	1.66E-04
cg01760090		8	Intergenic	S_Shore	40.59	12.28	49.01	12.98	-8.42	1.64E-03	2.45E-02
cg03361973	SEMA6D;SEMA6D;SEMA6D;SEMA6D;S	15	5'UTR;5'UTR;5'UTR;5'UTR;5'UTR;5'UTR	Open sea	55.31	9.89	63.72	8.98	-8.41	1.75E-04	4.45E-03
cg24904818	TRPM4	19	Body	N_Shore	47.86	7.55	56.27	13.14	-8.41	8.08E-04	1.46E-02
cg03565189	ANO1	11	1stExon	Island	10.60	7.82	19.01	11.12	-8.41	3.66E-05	1.19E-03
cg24439505	INS-IGF2;IGF2;INS-IGF2	11	Body;TSS1500;Body	Open sea	52.93	4.87	61.33	4.22	-8.40	6.45E-12	3.86E-10
cg20681688		13	Intergenic	Island	81.64	11.03	90.04	6.63	-8.40	2.02E-06	8.67E-05
cg00826537		1	Intergenic	Open sea	58.60	4.93	67.00	5.60	-8.40	9.68E-12	5.76E-10
cg10831725		19	Intergenic	Island	73.38	10.03	81.78	5.88	-8.40	1.64E-04	4.21E-03

cg01190109	PLEKHM3	2	3'UTR	Open sea	79.95	7.28	88.34	2.94	-8.40	6.13E-10	3.45E-08
cg08035479		8	Intergenic	Island	80.98	10.59	89.38	7.91	-8.39	4.19E-07	1.98E-05
cg24246628	KIF25;KIF25	6	Body;Body	Island	25.50	6.47	33.89	9.08	-8.39	6.75E-06	2.63E-04
cg17342709		2	Intergenic	Open sea	59.11	8.16	67.49	10.87	-8.38	2.26E-04	5.45E-03
cg02537309	FBRS	16	Body	Open sea	79.06	5.79	87.44	4.39	-8.38	1.23E-10	7.10E-09
cg18782201	FOXA3	19	3'UTR	N_Shelf	60.69	5.34	69.07	5.83	-8.38	2.39E-09	1.31E-07
cg11258452	SRRM4	12	Body	S_Shelf	73.28	7.18	81.65	8.31	-8.37	9.28E-07	4.19E-05
cg25418210		10	Intergenic	Open sea	53.67	7.35	62.04	9.44	-8.37	6.42E-05	1.93E-03
cg09160231	ELFN1	7	5'UTR	Open sea	46.49	9.80	54.86	7.68	-8.37	2.32E-05	7.97E-04
cg04423294	LPP;LPP;LPP	3	Body;Body;Body	Open sea	50.56	5.82	58.93	7.36	-8.37	1.79E-07	8.72E-06
cg13796936		1	Intergenic	Open sea	55.46	6.39	63.83	7.12	-8.37	1.81E-07	8.84E-06
cg01792430	CLIP4	2	Body	Open sea	52.43	6.62	60.79	7.87	-8.36	8.72E-07	3.94E-05
cg06106428	ARHGAP20	11	TSS1500	S_Shore	34.50	6.82	42.85	9.73	-8.35	5.39E-05	1.66E-03
cg07034329	MAST4;MAST4;MAST4	5	TSS1500;Body;Body	Open sea	15.96	5.29	24.31	6.92	-8.35	2.89E-09	1.58E-07
cg01200150		18	Intergenic	Island	52.41	9.76	60.76	9.61	-8.35	2.07E-04	5.08E-03
cg09109520	GPR56;GPR56;GPR56;GPR56;GPR56;G	16	5'UTR;5'UTR;5'UTR;5'UTR;5'UTR;1stExon;5	Open sea	28.35	5.92	36.69	10.06	-8.35	1.96E-06	8.41E-05
cg16353615		17	Intergenic	Island	47.04	7.79	55.39	9.84	-8.35	1.26E-05	4.63E-04
cg26730416		11	Intergenic	Open sea	23.86	6.31	32.20	9.24	-8.34	2.59E-06	1.09E-04
cg13425888	NLRP2	19	TSS1500	Open sea	73.00	15.23	81.34	8.58	-8.34	7.34E-04	1.36E-02
cg12736877	RNF39;RNF39	6	Body;Body	Island	46.56	8.08	54.90	10.46	-8.34	1.46E-05	5.28E-04
cg10984625	LRRFIP1;LRRFIP1;LRRFIP1;LRRFIP1;LRI	2	TSS1500;TSS1500;TSS1500;TSS1500;Body	Island	48.99	6.75	57.32	5.85	-8.33	3.13E-08	1.62E-06
cg15308737	ARSG	17	5'UTR	Open sea	76.11	8.42	84.44	7.71	-8.33	2.69E-05	9.08E-04
cg13155535		3	Intergenic	N_Shelf	73.28	7.36	81.61	2.83	-8.33	1.21E-09	6.72E-08
cg21443659		2	Intergenic	Open sea	59.22	6.62	67.55	5.33	-8.33	1.18E-08	6.25E-07
cg26390041		17	Intergenic	Open sea	16.89	7.40	25.22	13.03	-8.32	3.38E-04	7.47E-03
cg02293501		1	Intergenic	Open sea	69.65	18.46	77.97	8.68	-8.31	2.60E-03	3.39E-02
cg06623219	NFIX	19	3'UTR	Island	33.37	9.14	41.69	12.72	-8.31	1.12E-03	1.86E-02
cg09119043	SLC35F3	1	Body	Island	50.25	8.45	58.56	10.90	-8.31	8.09E-05	2.34E-03
cg15000222	TESC;TESC;TESC	12	Body;Body;Body	S_Shore	33.19	6.94	41.49	9.72	-8.31	1.62E-05	5.81E-04
cg08127021	LRP5	11	Body	Open sea	62.16	7.34	70.47	7.41	-8.30	7.86E-06	3.03E-04
cg01072388		16	Intergenic	Open sea	59.25	5.96	67.55	6.09	-8.30	1.03E-08	5.50E-07
cg16032894	MPPED2	11	5'UTR	N_Shore	52.73	7.06	61.03	8.83	-8.30	1.25E-06	5.53E-05
cg26299169	PDX1	13	3'UTR	Island	31.37	10.08	39.66	11.67	-8.29	2.52E-03	3.32E-02
cg08203715	ST3GAL4;ST3GAL4	11	1stExon;5'UTR	S_Shore	13.40	4.03	21.69	9.19	-8.29	7.35E-08	3.70E-06
cg05376982	GSTM5	1	TSS200	Open sea	55.15	8.40	63.43	9.95	-8.28	2.22E-04	5.38E-03
cg19347782	MICAL2	11	5'UTR	Open sea	61.18	14.83	69.47	11.11	-8.28	3.87E-03	4.47E-02
cg09288989	SETD1B	12	Body	Island	70.84	8.18	79.13	5.59	-8.28	4.69E-07	2.19E-05
cg19709146	FNBP4	11	Body	Open sea	50.18	7.96	58.47	10.63	-8.28	7.21E-05	2.12E-03
cg16078649	RNF39;RNF39	6	Body;Body	Island	33.38	7.33	41.66	10.85	-8.28	1.58E-05	5.68E-04
cg19563307	THADA;THADA	2	Body;Body	Open sea	53.98	5.70	62.26	4.73	-8.28	8.75E-10	4.89E-08
cg14493094		4	Intergenic	Island	51.07	11.64	59.35	10.50	-8.28	3.51E-03	4.18E-02
cg08151623		16	Intergenic	Island	30.50	6.02	38.78	8.53	-8.28	8.90E-07	4.02E-05
cg10970798	CAPZB	1	3'UTR	S_Shore	61.44	8.91	69.72	11.48	-8.27	1.63E-03	2.44E-02
cg27651238		12	Intergenic	Island	44.13	7.88	52.40	10.10	-8.27	2.43E-05	8.31E-04
cg24201034	SHROOM3	4	Body	Island	13.93	5.76	22.20	6.65	-8.27	3.94E-08	2.03E-06
cg14769786	RTN4RL1	17	Body	S_Shore	24.49	9.32	32.76	10.75	-8.26	4.27E-04	8.97E-03
cg07059925	EXPH5;EXPH5	11	TSS200;Body	Open sea	41.97	6.81	50.24	7.95	-8.26	1.08E-05	4.05E-04
cg09636214	PAPLN	14	Body	Island	37.34	6.09	45.60	8.31	-8.26	1.63E-07	8.00E-06
cg23220439	RIN1	11	TSS1500	S_Shelf	40.25	5.87	48.51	4.55	-8.26	2.77E-09	1.52E-07
cg06960164	C6orf132	6	Body	Island	22.90	7.27	31.16	10.16	-8.26	1.87E-05	6.57E-04
cg10825530	SOX6;SOX6;SOX6;SOX6	11	Body;Body;Body;Body	Open sea	50.24	9.39	58.49	6.17	-8.25	1.56E-06	6.81E-05
cg21033440	SIPA1;SIPA1	11	Body;Body	Island	37.36	6.10	45.60	8.22	-8.23	2.59E-06	1.09E-04
cg21475781	CFHR5	1	TSS1500	Open sea	48.85	10.11	57.08	12.18	-8.23	4.33E-03	4.82E-02

cg20368904	TNFAIP2	14	Body	N_Shore	53.92	6.79	62.15	10.08	-8.23	4.88E-05	1.52E-03
cg06173663	CUX1;CUX1;CUX1	7	Body;Body;Body	Open sea	27.76	6.88	35.99	9.49	-8.23	2.03E-05	7.08E-04
cg04113200	STX2;STX2	12	Body;Body	Island	30.63	6.87	38.85	6.96	-8.22	9.00E-09	4.81E-07
cg08352422		11	Intergenic	Open sea	48.25	9.41	56.47	9.93	-8.22	1.13E-04	3.10E-03
cg13494954	FBP1;FBP1	9	Body;Body	Open sea	71.22	8.56	79.44	8.62	-8.22	1.98E-04	4.91E-03
cg16614288	SLC27A1	19	Body	Island	60.05	8.22	68.26	10.25	-8.22	5.56E-05	1.70E-03
cg00589520		7	Intergenic	N_Shore	26.09	5.07	34.31	5.99	-8.22	2.01E-10	1.15E-08
cg16906964		1	Intergenic	Open sea	49.01	7.01	57.22	7.88	-8.21	9.06E-06	3.44E-04
cg08778316	DNASE1;DNASE1	16	5'UTR;1stExon	Open sea	74.21	6.91	82.42	4.27	-8.21	4.21E-08	2.16E-06
cg06746670	SALL1;SALL1	16	Body;Body	S_Shelf	51.22	9.00	59.43	12.66	-8.21	2.89E-03	3.65E-02
cg24202817	PFKFB3	10	Body	Open sea	44.93	10.51	53.13	9.74	-8.21	6.85E-04	1.29E-02
cg09426383	SKAP2	7	Body	Open sea	22.10	6.28	30.30	6.85	-8.20	6.32E-09	3.40E-07
cg03343571	RNF39;RNF39	6	Body;Body	Island	27.57	9.22	35.78	12.10	-8.20	4.42E-04	9.22E-03
cg13527735	C3orf26;C3orf26;FILIP1L;FILIP1L	3	TSS200;Body;TSS1500;TSS1500	Open sea	60.27	4.83	68.46	6.02	-8.20	2.17E-10	1.24E-08
cg07628073		19	Intergenic	N_Shore	46.71	7.21	54.91	8.78	-8.20	3.82E-05	1.23E-03
cg23124613	ABHD2;ABHD2	15	Body;Body	Open sea	51.40	7.33	59.59	10.34	-8.20	1.28E-04	3.44E-03
cg15102070	PPP2R2D;PPP2R2D;PPP2R2D	10	Body;Body;Body	Open sea	70.69	6.37	78.89	5.99	-8.19	8.52E-08	4.27E-06
cg10827488	ZBTB16;ZBTB16	11	Body;Body	Island	45.79	7.16	53.98	7.89	-8.19	3.11E-06	1.29E-04
cg13905081		9	Intergenic	S_Shore	46.64	4.70	54.83	5.84	-8.19	2.41E-10	1.38E-08
cg03821689	FXYD6;FXYD6;FXYD6;FXYD6;FXYD6	11	TSS1500;TSS1500;TSS1500;TSS1500;TSS20	S_Shore	42.10	7.77	50.29	11.74	-8.19	1.07E-03	1.80E-02
cg02224314	BCL11B;BCL11B	14	Body;Body	Island	54.22	7.56	62.40	8.50	-8.19	2.70E-05	9.11E-04
cg06996896	MAD1L1;MAD1L1;MAD1L1	7	Body;Body;Body	S_Shelf	71.43	8.31	79.60	6.66	-8.17	1.92E-05	6.73E-04
cg01657758	SORL1	11	Body	Open sea	59.90	6.60	68.07	6.76	-8.17	2.72E-07	1.31E-05
cg06717221	ATP8B3	19	Body	S_Shore	64.20	5.99	72.37	6.37	-8.17	2.18E-08	1.14E-06
cg06935979	KIAA1383	1	1stExon	Island	30.53	7.71	38.70	9.71	-8.17	7.56E-05	2.21E-03
cg16942644	COL18A1	21	Body	Open sea	48.31	10.20	56.47	13.03	-8.16	1.99E-03	2.81E-02
cg16766914	CUEDC1	17	Body	Island	50.21	6.97	58.37	8.64	-8.16	1.31E-05	4.80E-04
cg14656043	CREM;CREM;CREM;CREM;CREM;CREI	10	5'UTR;5'UTR;5'UTR;5'UTR;5'UTR;5'UTR	S_Shelf	41.26	5.80	49.42	6.00	-8.16	4.28E-09	2.32E-07
cg07997634	SLC25A13;SLC25A13;SLC25A13	7	Body;Body;Body	Open sea	58.23	7.83	66.39	7.40	-8.16	1.57E-06	6.84E-05
cg09057517		6	Intergenic	Open sea	25.55	7.89	33.71	10.27	-8.16	2.25E-04	5.43E-03
cg00491255	TEPP;TEPP	16	Body;Body	Island	49.55	8.19	57.71	12.52	-8.16	4.40E-04	9.17E-03
cg04589674	HTRA4	8	TSS200	N_Shore	27.71	8.79	35.86	12.09	-8.15	9.52E-04	1.65E-02
cg24468199	SIPA1;SIPA1	11	Body;Body	Island	59.36	6.05	67.51	5.96	-8.15	2.65E-08	1.38E-06
cg07931792	ZNF490	19	Body	Island	54.29	8.34	62.43	11.92	-8.15	1.03E-03	1.75E-02
cg13348458		6	Intergenic	Open sea	24.94	6.13	33.08	7.29	-8.15	6.13E-08	3.11E-06
cg13953978	USP20;USP20;USP20	9	5'UTR;5'UTR;5'UTR	S_Shelf	72.14	9.25	80.29	6.33	-8.14	2.25E-05	7.75E-04
cg06892548	TTBK1	6	Body	Island	44.49	7.33	52.63	8.83	-8.14	3.02E-05	1.01E-03
cg13465946	NFIX	19	3'UTR	Island	34.94	5.95	43.08	8.47	-8.14	1.79E-06	7.72E-05
cg21613693	CASKIN2;CASKIN2	17	Body;Body	Island	64.83	7.64	72.97	6.13	-8.14	1.33E-05	4.86E-04
cg02681842	PLEC1;PLEC1	8	Body;Body	Open sea	53.08	9.33	61.22	11.22	-8.13	8.49E-04	1.51E-02
cg20699780	ABR;ABR;ABR	17	Body;Body;Body	Island	36.89	6.51	45.02	10.87	-8.13	9.48E-05	2.68E-03
cg15227982	C10orf26;C10orf26	10	TSS200;Body	Open sea	40.54	7.98	48.67	6.98	-8.13	1.97E-05	6.91E-04
cg09775930		13	Intergenic	N_Shore	15.79	5.75	23.91	8.62	-8.13	4.89E-07	2.28E-05
cg17191109		10	Intergenic	Island	42.85	7.67	50.98	11.29	-8.12	2.10E-04	5.14E-03
cg25896644		11	Intergenic	Island	19.71	6.82	27.83	9.21	-8.12	3.56E-05	1.16E-03
cg20645966		12	Intergenic	Open sea	51.78	4.56	59.90	5.07	-8.12	1.90E-11	1.12E-09
cg24857560	CYP2C19	10	TSS1500	Open sea	67.61	9.65	75.73	8.58	-8.12	6.21E-05	1.88E-03
cg01437235	TMEM175	4	5'UTR	N_Shore	68.61	5.91	76.73	5.14	-8.12	2.56E-10	1.46E-08
cg19357999	P2RX4	12	Body	Open sea	62.48	9.98	70.59	10.61	-8.11	2.28E-03	3.10E-02
cg13038847		22	Intergenic	Island	54.96	8.88	63.07	11.57	-8.11	1.96E-03	2.79E-02
cg14646081	FXN;FXN;FXN	9	Body;3'UTR;3'UTR	Open sea	73.23	7.16	81.33	4.09	-8.11	2.40E-07	1.16E-05
cg06912922	ZNF490	19	Body	S_Shore	49.87	8.88	57.97	10.51	-8.11	1.10E-03	1.84E-02
cg14541209	DTX1	12	Body	N_Shore	37.26	6.12	45.36	6.81	-8.10	5.18E-08	2.64E-06

cg01710189	PDLIM2	8	3'UTR	N_Shore	36.04	5.99	44.14	6.32	-8.10	1.04E-09	5.78E-08
cg02069008		10	Intergenic	Open sea	54.56	8.14	62.65	10.04	-8.10	5.56E-04	1.10E-02
cg12614630	GPR182	12	TSS1500	Open sea	40.08	8.15	48.17	11.00	-8.09	4.90E-04	9.97E-03
cg09924366	ZFYVE21	14	Body	S_Shore	60.38	7.75	68.47	9.18	-8.09	2.06E-04	5.05E-03
cg20803293	GSTM5	1	TSS200	Open sea	37.57	7.71	45.65	10.96	-8.09	3.37E-04	7.46E-03
cg27353899	MUC4;MUC4;MUC4	3	Body;Body;Body	Island	26.67	9.22	34.76	11.35	-8.08	1.82E-03	2.65E-02
cg03373519		4	Intergenic	Open sea	74.75	17.39	82.83	8.68	-8.08	3.72E-03	4.34E-02
cg03795245	GPR162;CD4;GPR162	12	TSS1500;3'UTR;TSS1500	N_Shelf	64.76	10.74	72.84	9.50	-8.08	6.50E-04	1.24E-02
cg14181355	MAST1	19	Body	Island	62.28	9.36	70.35	8.77	-8.08	2.67E-04	6.22E-03
cg26472684		6	Intergenic	Open sea	49.25	5.69	57.32	6.62	-8.07	2.36E-07	1.14E-05
cg01048346	AMN1;AMN1	12	3'UTR;Body	Open sea	66.81	6.77	74.87	6.49	-8.07	5.53E-07	2.57E-05
cg15229747		19	Intergenic	N_Shore	28.05	6.08	36.12	7.74	-8.07	1.02E-08	5.41E-07
cg24147428	SIPA1;SIPA1	11	Body;Body	Island	43.29	5.42	51.36	8.51	-8.07	1.30E-06	5.75E-05
cg15101392	FXYD6;FXYD6;FXYD6;FXYD6;FXYD6;FX	11	TSS1500;1stExon;5'UTR;TSS1500;TSS1500;	S_Shore	43.20	8.83	51.27	10.90	-8.07	8.12E-04	1.46E-02
cg17082405	PLEC1;PLEC1	8	Body;Body	Open sea	61.02	9.36	69.08	11.21	-8.06	3.84E-04	8.24E-03
cg04286377	GABRA5;GABRA5	15	TSS1500;TSS1500	N_Shore	38.12	7.01	46.19	9.66	-8.06	1.03E-05	3.87E-04
cg26989646	FOSL2	2	Body	Open sea	72.61	4.87	80.67	4.19	-8.06	1.00E-13	6.28E-12
cg06147361	SOX5	12	5'UTR	Open sea	41.34	6.72	49.39	6.47	-8.05	2.30E-06	9.74E-05
cg03627300		2	Intergenic	Open sea	60.07	4.64	68.12	6.71	-8.05	6.47E-10	3.63E-08
cg05808246		1	Intergenic	Open sea	41.76	6.33	49.81	6.15	-8.05	1.18E-07	5.85E-06
cg18693051	SBNO2	19	5'UTR	N_Shore	57.25	9.48	65.30	11.35	-8.05	3.75E-03	4.36E-02
cg05791946	PRDM16;PRDM16	1	Body;Body	N_Shelf	43.41	7.45	51.46	10.06	-8.05	9.58E-05	2.70E-03
cg04329125	CRIP2	14	Body	Island	59.18	9.52	67.23	7.17	-8.05	2.17E-04	5.28E-03
cg10985430		13	Intergenic	Open sea	48.17	7.73	56.22	8.61	-8.05	4.72E-06	1.90E-04
cg27479455	ARHGEF18;ARHGEF18	19	Body;Body	N_Shore	66.55	7.16	74.60	7.70	-8.04	2.89E-05	9.64E-04
cg15682194	ANO1	11	Body	Island	19.41	7.14	27.45	7.60	-8.04	1.75E-07	8.56E-06
cg00783706	ZC3H3	8	Body	Island	49.72	8.45	57.76	12.29	-8.04	1.63E-03	2.44E-02
cg19855559		3	Intergenic	Open sea	38.40	6.77	46.44	7.64	-8.03	7.33E-06	2.84E-04
cg02192965	SLC3A1	2	1stExon	Open sea	69.34	11.11	77.38	7.47	-8.03	5.54E-04	1.10E-02
cg17921924	LOC145845	15	Body	S_Shore	55.34	6.60	63.37	7.00	-8.03	2.33E-07	1.13E-05
cg27118533	PTPRK;PTPRK	6	Body;Body	Open sea	75.61	10.74	83.64	9.59	-8.02	2.93E-03	3.69E-02
cg11378389	NOS1AP;NOS1AP	1	Body;Body	S_Shelf	51.66	5.90	59.69	5.75	-8.02	1.59E-08	8.38E-07
cg04077850	GRAMD3;GRAMD3;GRAMD3;GRAMD	5	Body;5'UTR;TSS1500;Body;Body	Open sea	66.85	6.16	74.88	4.00	-8.02	5.85E-10	3.29E-08
cg26043955		8	Intergenic	Open sea	53.94	6.31	61.96	6.14	-8.02	6.71E-07	3.08E-05
cg12180316	FN3KRP	17	Body	Open sea	69.25	9.41	77.27	6.37	-8.02	1.25E-04	3.37E-03
cg20680163	PLEC1;PLEC1	8	Body;Body	Open sea	65.38	8.37	73.40	10.45	-8.02	2.06E-04	5.05E-03
cg26808784	H19	11	Body	Island	50.22	5.76	58.24	5.76	-8.02	1.68E-08	8.83E-07
cg03991369	GOT1	10	Body	Open sea	54.20	5.28	62.21	6.59	-8.01	4.98E-08	2.54E-06
cg21621482	SLC38A10;SLC38A10	17	Body;Body	Open sea	37.82	6.11	45.83	5.56	-8.01	3.20E-07	1.53E-05
cg01203286		2	Intergenic	Open sea	60.04	6.06	68.04	5.10	-8.01	2.56E-08	1.33E-06
cg24318537	UNC119B	12	3'UTR	N_Shelf	60.28	8.87	68.29	12.02	-8.01	3.83E-03	4.43E-02
cg15615645	C10orf26;C10orf26	10	Body;Body	Open sea	27.33	7.30	35.33	10.42	-8.00	1.87E-04	4.69E-03
cg25612428	ANKRD33B	5	Body	Island	51.36	7.73	59.36	9.79	-8.00	9.36E-05	2.65E-03
cg06794355		1	Intergenic	N_Shelf	50.80	10.26	58.80	7.66	-8.00	2.71E-04	6.28E-03
cg19100988		5	Intergenic	Open sea	56.75	6.50	64.75	5.28	-7.99	6.49E-08	3.28E-06
cg08686462	INS-IGF2;IGF2;INS-IGF2	11	Body;TSS1500;Body	Open sea	40.99	6.26	48.98	5.84	-7.99	6.46E-08	3.27E-06
cg17782713	BMI1	10	5'UTR	N_Shore	67.27	6.95	75.26	4.56	-7.99	7.74E-08	3.89E-06
cg12955277		21	Intergenic	Island	61.93	9.39	69.92	8.30	-7.99	3.25E-04	7.25E-03
cg14484793	OLFM1;OLFM1	9	Body;Body	N_Shore	37.09	6.98	45.08	8.06	-7.99	4.39E-06	1.78E-04
cg03357650	KANK2;KANK2	19	Body;Body	S_Shore	43.55	6.16	51.53	9.86	-7.98	8.21E-05	2.37E-03
cg07170752	DNAH14	1	Body	Open sea	80.03	3.40	88.01	1.78	-7.98	2.48E-23	1.90E-21
cg01366985		6	Intergenic	Open sea	78.03	7.78	86.01	3.09	-7.98	1.92E-08	1.01E-06
cg13365436	AP2A2	11	Body	Open sea	63.03	7.32	71.00	7.24	-7.97	4.65E-06	1.87E-04

cg09947611	HCCA2	11	Body	Open sea	37.54	6.37	45.52	8.79	-7.97	1.56E-05	5.62E-04
cg01871581	ZNF490	19	Body	Island	52.28	9.49	60.25	11.16	-7.97	3.16E-03	3.88E-02
cg24467349	GSTM5	1	TSS200	Open sea	45.29	9.99	53.26	10.91	-7.97	4.41E-03	4.88E-02
cg22846149	HMGB1L1	20	TSS200	Open sea	53.62	6.65	61.59	5.26	-7.97	4.31E-08	2.21E-06
cg04640920		15	Intergenic	S_Shore	44.36	7.61	52.33	8.51	-7.97	4.60E-06	1.85E-04
cg26640901	C10orf26;C10orf26;C10orf26	10	5'UTR;Body;1stExon	Open sea	32.82	7.60	40.79	7.99	-7.97	4.03E-05	1.29E-03
cg26428069	NCKAP5;NCKAP5	2	TSS1500;TSS1500	Open sea	46.60	6.80	54.56	5.69	-7.96	4.29E-09	2.33E-07
cg11631271		19	Intergenic	Island	28.51	5.69	36.47	7.63	-7.96	7.52E-07	3.43E-05
cg07574163		14	Intergenic	N_Shore	45.86	6.16	53.82	6.54	-7.96	4.09E-07	1.93E-05
cg06693983	TMEM190	19	Body	Island	31.02	8.97	38.97	11.01	-7.96	2.47E-03	3.28E-02
cg10888337	WNT7B	22	Body	Island	12.35	5.63	20.31	10.29	-7.95	5.71E-05	1.74E-03
cg27517823	SDC2	8	Body	Open sea	51.92	8.58	59.87	10.26	-7.95	1.24E-03	2.01E-02
cg19712189	TDRD12;TDRD12	19	5'UTR;1stExon	Island	73.31	8.70	81.26	6.67	-7.95	1.00E-05	3.77E-04
cg11223622	ITGB4;ITGB4;ITGB4	17	Body;Body;Body	Island	20.66	8.40	28.60	11.41	-7.95	1.62E-03	2.44E-02
cg13502592	ELFN1	7	TSS200	Open sea	82.15	7.68	90.09	3.13	-7.94	7.96E-08	4.00E-06
cg05964487		13	Intergenic	S_Shore	65.88	6.42	73.82	5.93	-7.94	1.87E-09	1.03E-07
cg26861732		20	Intergenic	Island	55.30	7.75	63.24	8.64	-7.94	9.43E-06	3.57E-04
cg10692467	SEMA3B;SEMA3B	3	Body;Body	Island	17.96	6.38	25.89	10.43	-7.94	2.83E-05	9.49E-04
cg09286981	TSPAN9;TSPAN9	12	5'UTR;5'UTR	Open sea	57.75	8.52	65.68	12.17	-7.93	3.17E-03	3.89E-02
cg24932788	SOX9	17	Body	Island	11.39	4.63	19.32	6.52	-7.93	3.46E-09	1.89E-07
cg15249221		7	Intergenic	N_Shore	61.81	7.24	69.74	5.67	-7.93	2.38E-06	1.00E-04
cg16335762	CMTM3;CMTM3;CMTM3;CMTM3	16	TSS1500;TSS1500;TSS1500;TSS1500	N_Shore	28.76	8.13	36.69	10.20	-7.93	1.00E-04	2.80E-03
cg06789445	MITF;MITF;MITF;MITF	3	Body;Body;Body;Body	Open sea	69.56	7.24	77.49	7.97	-7.93	7.58E-06	2.93E-04
cg08906898	RBM39;RBM39	20	Body;Body	Open sea	84.82	3.19	92.75	1.82	-7.92	1.54E-26	1.25E-24
cg01685893	BCO2;BCO2	11	5'UTR;Body	Open sea	48.45	9.29	56.37	10.24	-7.92	8.21E-04	1.48E-02
cg18027831	PSEN2;PSEN2	1	3'UTR;3'UTR	Open sea	62.80	6.89	70.72	6.62	-7.92	2.23E-06	9.49E-05
cg20413392	MIR219-2	9	TSS200	Island	69.40	6.94	77.31	6.88	-7.91	1.18E-07	5.86E-06
cg05725263	PC;PC;PC	11	5'UTR;5'UTR;5'UTR	Open sea	59.91	7.32	67.82	10.16	-7.91	7.12E-05	2.10E-03
cg23756143	PC;PC;PC	11	5'UTR;5'UTR;5'UTR	Open sea	50.78	7.75	58.69	8.82	-7.91	7.28E-05	2.14E-03
cg09063683		5	Intergenic	Open sea	58.18	5.17	66.08	4.67	-7.90	1.24E-09	6.87E-08
cg20498962	NOS3	7	Body	Island	42.55	6.18	50.45	5.58	-7.90	8.71E-08	4.36E-06
cg26222311	AGRN	1	Body	Island	13.56	5.32	21.46	9.95	-7.90	5.19E-06	2.07E-04
cg12203206	PISD	22	TSS200	S_Shore	10.62	4.12	18.52	6.21	-7.90	1.62E-11	9.56E-10
cg26471058	CAPN13;CAPN13	2	1stExon;5'UTR	Open sea	39.68	5.10	47.58	4.43	-7.90	5.19E-12	3.11E-10
cg13436940		11	Intergenic	Open sea	15.55	8.28	23.43	10.47	-7.89	1.25E-04	3.37E-03
cg12569592	ATXN1;ATXN1	6	5'UTR;5'UTR	Open sea	52.18	6.98	60.06	7.01	-7.88	1.07E-05	4.00E-04
cg02915785		2	Intergenic	S_Shore	53.84	5.32	61.71	5.65	-7.88	1.77E-09	9.78E-08
cg23625715	AGRN	1	Body	Island	9.31	5.40	17.19	9.61	-7.88	1.94E-06	8.35E-05
cg13302869		2	Intergenic	Island	19.14	6.65	27.02	8.08	-7.88	4.18E-06	1.70E-04
cg01545079	CPNE6	14	5'UTR	Open sea	67.62	6.36	75.49	5.86	-7.87	7.35E-07	3.36E-05
cg08799350		6	Intergenic	Open sea	64.58	7.43	72.45	7.61	-7.87	1.01E-05	3.82E-04
cg07496545	SLC25A2	5	TSS200	Island	47.13	7.85	54.99	9.47	-7.87	5.60E-05	1.71E-03
cg24676346		6	Intergenic	S_Shore	51.80	8.51	59.66	12.63	-7.86	9.26E-04	1.62E-02
cg04265964		11	Intergenic	Open sea	57.46	7.07	65.32	7.49	-7.86	7.43E-07	3.40E-05
cg10023862	CUEDC1	17	Body	Island	64.76	7.17	72.61	8.85	-7.85	2.54E-05	8.64E-04
cg10389114	CNOT1;CNOT1	16	Body;Body	Open sea	37.07	4.87	44.92	6.05	-7.85	1.78E-09	9.83E-08
cg02692075	SCFD2	4	Body	Open sea	62.61	6.31	70.46	5.83	-7.85	6.47E-07	2.98E-05
cg02395396	RAB3B	1	Body	Open sea	56.35	5.82	64.20	5.87	-7.85	6.01E-10	3.38E-08
cg12385165	CLVS2	6	Body	S_Shelf	40.44	5.37	48.29	6.48	-7.85	1.87E-08	9.79E-07
cg05942111	RASSF3	12	Body	Open sea	46.40	9.88	54.24	9.15	-7.84	2.06E-05	7.17E-04
cg13058710	SOX9	17	Body	Island	30.63	7.04	38.47	9.22	-7.84	1.61E-04	4.15E-03
cg14486098	SLC38A10;SLC38A10	17	Body;Body	N_Shore	54.60	8.37	62.44	7.50	-7.84	2.57E-05	8.73E-04
cg03764193	ZCCHC24	10	Body	Open sea	70.63	6.84	78.47	7.01	-7.83	2.18E-06	9.31E-05

cg15721243	ZNF468;ZNF468	19	TSS1500;TSS1500	S_Shore	33.87	10.91	41.70	10.61	-7.83	4.59E-04	9.48E-03
cg07620573	FGF12	3	Body	Open sea	44.84	11.73	52.66	10.56	-7.83	8.48E-05	2.44E-03
cg19535073	LOC285954;LOC285954;INHBA	7	Body;Body;TSS1500	N_Shore	57.13	5.86	64.96	7.01	-7.82	2.89E-07	1.38E-05
cg08090164	LYNX1;LYNX1;LYNX1;LYNX1	8	TSS200;TSS1500;TSS200;TSS1500	S_Shore	67.64	10.74	75.46	10.32	-7.82	9.23E-04	1.61E-02
cg16554941	ZSWIM6	5	Body	Open sea	49.87	6.11	57.69	5.28	-7.82	7.93E-08	3.98E-06
cg10612452	NTN1	17	Body	Island	28.32	6.95	36.14	7.59	-7.82	4.20E-08	2.15E-06
cg25399818	ARRB2;ARRB2	17	3'UTR;3'UTR	Open sea	78.21	3.93	86.03	2.98	-7.82	3.71E-17	2.52E-15
cg24176731	PLEKHB1;PLEKHB1;PLEKHB1;PLEKHB1	11	TSS200;TSS200;TSS1500;TSS1500	Open sea	46.62	7.53	54.44	11.47	-7.82	1.74E-04	4.43E-03
cg19533679	CDRT4	17	Body	Open sea	75.45	4.76	83.26	5.11	-7.82	1.96E-10	1.12E-08
cg13180105		13	Intergenic	S_Shelf	83.95	7.81	91.77	3.34	-7.81	5.98E-07	2.77E-05
cg09993699	BST2	19	TSS1500	Open sea	35.44	10.17	43.25	11.40	-7.81	1.17E-03	1.92E-02
cg11717597	MEX3A	1	Body	Island	34.76	6.95	42.57	9.18	-7.81	3.30E-05	1.08E-03
cg07234199	LOC388796;LOC388796;SNORA71C	20	Body;Body;TSS1500	N_Shelf	68.50	9.72	76.31	6.79	-7.81	7.47E-05	2.19E-03
cg12917058	C1GALT1	7	5'UTR	Open sea	69.17	7.21	76.98	3.83	-7.81	1.09E-07	5.43E-06
cg14916108	CACNA2D4	12	TSS200	Open sea	55.48	9.07	63.28	7.34	-7.80	1.14E-04	3.11E-03
cg14281592	ANGPTL2;RALGPS1	9	5'UTR;Body	Open sea	39.20	7.84	47.00	10.59	-7.80	7.63E-04	1.40E-02
cg00580562		10	Intergenic	Island	9.96	4.56	17.75	9.46	-7.80	6.34E-09	3.41E-07
cg01886634	CEP78;CEP78	9	TSS1500;TSS1500	N_Shore	59.92	5.52	67.72	5.01	-7.79	4.84E-10	2.73E-08
cg24301724	GPR133	12	Body	Open sea	23.94	8.49	31.73	8.13	-7.79	1.92E-04	4.78E-03
cg08330031	C10orf32;C10orf32	10	3'UTR;3'UTR	Open sea	36.95	6.46	44.74	7.88	-7.79	6.70E-08	3.39E-06
cg11558551	BST2	19	TSS200	Open sea	19.96	9.30	27.75	12.45	-7.79	3.32E-03	4.02E-02
cg09025324	DSE;DSE	6	TSS1500;5'UTR	N_Shore	42.63	9.74	50.42	8.21	-7.79	6.98E-04	1.31E-02
cg16767867	SORBS2;SORBS2;SORBS2;SORBS2;SOF	4	5'UTR;5'UTR;5'UTR;5'UTR;5'UTR;5'UTR	Open sea	46.95	7.52	54.73	10.21	-7.79	2.47E-04	5.85E-03
cg19634315	THSD4	15	Body	Open sea	46.34	8.10	54.12	7.94	-7.78	1.27E-05	4.68E-04
cg14601038	TPO;TPO;TPO;TPO	2	Body;Body;Body;Body	Island	48.00	5.12	55.78	6.58	-7.78	7.38E-08	3.72E-06
cg12782933		11	Intergenic	Island	18.96	7.26	26.74	7.96	-7.78	8.42E-06	3.22E-04
cg25998680	PTK7;PTK7;PTK7;PTK7	6	1stExon;1stExon;1stExon;1stExon	Island	32.90	7.03	40.67	5.85	-7.77	4.44E-07	2.09E-05
cg14240646	ACBD5;ACBD5	10	TSS1500;TSS1500	S_Shore	30.89	14.92	38.66	15.52	-7.77	3.31E-03	4.01E-02
cg16460342	P2RX4	12	Body	Open sea	62.32	6.49	70.08	4.32	-7.77	2.45E-07	1.18E-05
cg17386911	FGFR4;FGFR4;FGFR4	5	Body;Body;Body	S_Shelf	57.01	7.22	64.77	7.78	-7.77	1.67E-05	5.94E-04
cg15411554	ADAMTS16	5	Body	Open sea	34.66	10.67	42.43	11.37	-7.77	1.63E-03	2.44E-02
cg02705910		16	Intergenic	Open sea	40.81	6.87	48.58	10.23	-7.76	1.65E-04	4.22E-03
cg26916916	NTN1	17	Body	S_Shelf	65.73	6.90	73.49	6.20	-7.76	1.96E-09	1.08E-07
cg17706972	ESR1;ESR1	6	5'UTR;TSS1500	N_Shelf	73.01	6.89	80.77	6.33	-7.76	8.29E-07	3.76E-05
cg13580107	NFATC1;NFATC1;NFATC1;NFATC1;NF/	18	Body;5'UTR;Body;Body;Body	N_Shore	74.73	8.05	82.49	7.51	-7.76	2.35E-06	9.94E-05
cg26883434	C5orf13;C5orf13;C5orf13;C5orf13;C5c	5	5'UTR;5'UTR;5'UTR;5'UTR;Body;1stExon;B	N_Shore	81.28	5.29	89.04	2.47	-7.75	4.57E-14	2.88E-12
cg02276826		10	Intergenic	N_Shore	57.51	10.38	65.27	8.69	-7.75	7.39E-05	2.17E-03
cg23712018	RNF39;RNF39	6	Body;Body	Island	14.54	7.23	22.29	9.53	-7.75	7.37E-05	2.16E-03
cg05061886	MN1	22	1stExon	Island	52.66	8.17	60.41	10.31	-7.75	1.12E-04	3.08E-03
cg06279274	FAM24B	10	5'UTR	N_Shelf	48.42	6.09	56.17	6.89	-7.75	7.93E-07	3.61E-05
cg09884635	CCNI2	5	Body	S_Shore	73.05	6.39	80.80	6.18	-7.75	1.14E-07	5.68E-06
cg16739178		16	Intergenic	Open sea	67.73	7.24	75.48	5.43	-7.75	8.42E-08	4.22E-06
cg07861373	CASKIN2;CASKIN2	17	Body;Body	S_Shore	61.91	4.98	69.66	5.47	-7.74	4.87E-09	2.64E-07
cg24815973	BPHL;BPHL;BPHL;BPHL	6	Body;Body;Body;Body	S_Shore	78.49	6.25	86.22	3.72	-7.74	1.24E-09	6.88E-08
cg19107511		12	Intergenic	S_Shelf	34.25	5.13	41.98	6.16	-7.74	1.62E-08	8.54E-07
cg27255454	IGFALS;IGFALS;IGFALS	16	Body;Body;Body	Island	76.22	11.67	83.96	11.93	-7.73	3.50E-03	4.17E-02
cg18892132	VAMP8	2	TSS1500	Open sea	51.11	6.76	58.84	7.04	-7.73	5.58E-06	2.21E-04
cg10297792		12	Intergenic	N_Shore	61.57	7.47	69.30	6.49	-7.73	5.02E-07	2.34E-05
cg23088810		12	Intergenic	Open sea	54.44	7.18	62.17	7.50	-7.73	1.08E-05	4.02E-04
cg10034176	SGMS1	10	5'UTR	Open sea	33.82	7.92	41.55	7.43	-7.73	1.98E-04	4.91E-03
cg17362434		11	Intergenic	Island	11.55	4.46	19.28	9.76	-7.73	9.81E-08	4.89E-06
cg22413388	WNT7B	22	Body	Island	9.13	4.69	16.86	9.82	-7.72	1.03E-06	4.60E-05
cg01201138	TRPV4;TRPV4	12	3'UTR;3'UTR	Open sea	60.12	4.48	67.84	4.23	-7.72	2.08E-12	1.26E-10

cg16258229	NPHP4	1	Body	Open sea	45.65	7.03	53.37	9.55	-7.72	9.25E-05	2.62E-03
cg12672189		6	Intergenic	Open sea	49.96	12.14	57.68	13.52	-7.72	9.15E-04	1.60E-02
cg14489801	KCNIP2;KCNIP2;KCNIP2;KCNIP2;KCNIP	10	TSS200;TSS200;TSS200;TSS200;TSS200;TSS	S_Shore	18.66	4.85	26.38	8.27	-7.72	6.29E-08	3.19E-06
cg21550372	WDR25;WDR25	14	Body;Body	Open sea	67.23	6.58	74.95	4.69	-7.72	5.46E-09	2.95E-07
cg00406319		10	Intergenic	Island	54.26	8.88	61.98	11.47	-7.72	1.72E-03	2.54E-02
cg14140118	EXPH5;EXPH5	11	TSS200;Body	Open sea	64.21	7.22	71.93	5.29	-7.72	2.27E-06	9.64E-05
cg13143330		14	Intergenic	Island	23.87	9.14	31.59	10.30	-7.72	3.74E-04	8.08E-03
cg00936935	SGMS1	10	5'UTR	Open sea	66.78	7.31	74.50	7.30	-7.72	2.87E-08	1.49E-06
cg10353108	USP2;USP2	11	3'UTR;3'UTR	Island	24.21	7.49	31.92	9.09	-7.71	2.29E-04	5.51E-03
cg22776386	ITIH5;ITIH5	10	Body;Body	Open sea	58.48	5.28	66.19	4.38	-7.70	3.28E-10	1.86E-08
cg18990050	C7orf50;C7orf50;C7orf50	7	Body;Body;Body	Open sea	34.86	7.39	42.57	9.94	-7.70	2.54E-04	5.97E-03
cg26643476	GPR81	12	TSS1500	Open sea	51.70	8.38	59.40	9.31	-7.70	3.64E-04	7.92E-03
cg13809915		2	Intergenic	Open sea	65.38	6.44	73.08	6.93	-7.70	5.59E-07	2.60E-05
cg19463193		15	Intergenic	Open sea	53.02	9.16	60.72	8.99	-7.69	7.08E-05	2.10E-03
cg10061342	C1orf21	1	5'UTR	S_Shore	21.17	6.72	28.87	7.39	-7.69	1.82E-06	7.84E-05
cg08898775	ADAM10	15	TSS1500	S_Shore	39.60	6.67	47.29	10.71	-7.69	4.38E-04	9.15E-03
cg10794257	HOXA3;HOXA3;HOXA3	7	5'UTR;TSS1500;5'UTR	N_Shore	36.44	10.57	44.13	11.64	-7.69	2.62E-03	3.41E-02
cg15548613	MAFF;MAFF;MAFF;MAFF	22	Body;Body;Body;Body	Island	23.56	6.52	31.25	9.14	-7.69	2.40E-05	8.21E-04
cg09388489		2	Intergenic	Open sea	79.46	5.50	87.15	2.64	-7.69	6.91E-14	4.34E-12
cg22861286	SSBP3;SSBP3;SSBP3	1	Body;Body;Body	Open sea	59.07	6.88	66.75	7.98	-7.69	2.66E-05	8.99E-04
cg05783449	SERTAD2	2	5'UTR	Open sea	64.28	6.94	71.96	6.39	-7.68	5.59E-06	2.22E-04
cg17575906	C5orf13;C5orf13;C5orf13;C5orf13;C5o	5	Body;Body;Body;Body;Body;Body;Body;Bo	N_Shelf	71.06	7.39	78.74	5.15	-7.68	1.04E-06	4.66E-05
cg01965462	ERBB4;ERBB4	2	Body;Body	N_Shore	34.45	7.64	42.13	11.34	-7.68	7.90E-04	1.43E-02
cg18593660	NAV2	11	Body	Open sea	37.43	6.34	45.11	4.98	-7.68	6.64E-08	3.36E-06
cg03799283		15	Intergenic	Island	29.01	5.47	36.69	9.11	-7.68	4.39E-06	1.78E-04
cg25997722	SYTL3	6	Body	Open sea	71.34	9.61	79.02	6.24	-7.68	3.72E-05	1.20E-03
cg18159180	CUL7;CUL7;MRPL2	6	TSS1500;TSS1500;Body	S_Shore	43.13	6.80	50.80	7.46	-7.67	1.77E-05	6.27E-04
cg11583981	HS3ST3B1	17	Body	S_Shore	75.01	6.02	82.67	4.82	-7.67	2.69E-09	1.48E-07
cg16271663	CSF3;CSF3;CSF3	17	TSS1500;TSS1500;TSS1500	Open sea	46.61	5.03	54.27	5.90	-7.66	6.49E-09	3.49E-07
cg27065717		16	Intergenic	Open sea	46.83	5.78	54.49	6.59	-7.66	7.97E-07	3.63E-05
cg26052377	KALRN;KALRN	3	Body;Body	Open sea	77.58	11.15	85.24	10.40	-7.66	1.42E-03	2.21E-02
cg03818395	ANKRD22	10	TSS1500	Open sea	49.00	5.58	56.66	5.97	-7.66	1.33E-07	6.59E-06
cg19896129		1	Intergenic	S_Shelf	57.24	5.83	64.89	5.99	-7.66	3.23E-09	1.76E-07
cg27320005	SLC25A2	5	TSS200	Island	52.58	5.76	60.23	6.91	-7.65	6.98E-08	3.52E-06
cg06481660		6	Intergenic	Open sea	49.46	5.14	57.12	5.35	-7.65	4.82E-09	2.61E-07
cg22995176	UPK3B;UPK3B	7	TSS1500;TSS1500	Open sea	32.73	6.96	40.39	7.20	-7.65	1.54E-06	6.71E-05
cg06926314	ANKRD33B	5	3'UTR	Island	44.53	8.10	52.18	11.36	-7.65	6.53E-04	1.24E-02
cg21238257	DCUN1D2	13	Body	Open sea	71.87	10.37	79.52	8.69	-7.65	2.80E-03	3.57E-02
cg14651518	SEMA3B;SEMA3B	3	Body;Body	Island	22.79	8.83	30.43	12.58	-7.65	3.28E-03	3.99E-02
cg15817705		1	Intergenic	S_Shore	58.43	5.49	66.08	4.34	-7.65	4.81E-10	2.71E-08
cg04566512		22	Intergenic	Island	36.17	6.30	43.81	9.98	-7.65	6.54E-05	1.96E-03
cg18494742		15	Intergenic	Open sea	77.15	5.07	84.80	3.69	-7.65	3.55E-11	2.08E-09
cg20821713	C7orf50;C7orf50;C7orf50	7	Body;Body;Body	N_Shelf	61.37	9.44	69.02	7.77	-7.64	7.96E-05	2.31E-03
cg24341759	SSBP3;SSBP3;SSBP3	1	Body;Body;Body	Open sea	71.41	8.02	79.05	6.52	-7.64	2.50E-05	8.52E-04
cg12052203	B3GNT1;B3GNT1	11	1stExon;5'UTR	Island	3.20	0.71	10.84	2.23	-7.64	1.59E-45	1.93E-43
cg21504064	NBR1;TMEM106A;NBR1;NBR1	17	3'UTR;TSS1500;3'UTR;3'UTR	N_Shore	18.79	4.59	26.42	6.29	-7.64	1.48E-11	8.74E-10
cg15284457		15	Intergenic	Open sea	58.95	5.98	66.58	5.03	-7.64	6.17E-08	3.13E-06
cg18335931	MAST3	19	Body	Island	36.27	6.32	43.91	7.27	-7.64	1.72E-06	7.47E-05
cg10627839	CFHR4	1	Body	Open sea	22.70	6.51	30.34	9.59	-7.64	4.81E-06	1.93E-04
cg26034919	KRTCAP3;KRTCAP3	2	Body;Body	S_Shore	51.76	7.49	59.39	8.42	-7.64	9.80E-05	2.75E-03
cg02447227	NFIA;NFIA;NFIA;NFIA	1	Body;Body;Body;Body	Open sea	65.97	6.30	73.60	5.83	-7.63	3.08E-07	1.48E-05
cg10531055	RREB1;RREB1;RREB1;RREB1	6	3'UTR;3'UTR;3'UTR;3'UTR	S_Shelf	65.98	5.70	73.61	6.85	-7.63	3.97E-08	2.04E-06
cg14826797	TARS	5	Body	S_Shelf	48.78	6.46	56.41	6.10	-7.63	1.03E-06	4.60E-05

cg20309703	PHLDB1;PHLDB1;PHLDB1	11	5'UTR;5'UTR;5'UTR	Island	11.59	6.82	19.21	10.46	-7.63	2.52E-05	8.58E-04
cg14134732	CCDC64B	16	Body	Island	20.28	7.08	27.90	11.24	-7.62	4.26E-04	8.95E-03
cg23769730	DHTKD1	10	Body	S_Shelf	53.18	9.22	60.79	9.76	-7.62	2.07E-03	2.90E-02
cg23535449	MIR1250;AATK	17	TSS200;Body	N_Shelf	69.47	8.61	77.08	6.08	-7.61	7.77E-06	2.99E-04
cg19988354		11	Intergenic	Open sea	83.29	6.62	90.90	2.55	-7.61	3.21E-11	1.88E-09
cg06720722	TP63;TP63;TP63	3	TSS200;TSS200;TSS200	Open sea	50.31	10.92	57.92	10.02	-7.61	1.36E-03	2.15E-02
cg00409844	OCLN	5	5'UTR	Open sea	55.85	4.52	63.47	5.76	-7.61	2.24E-10	1.28E-08
cg02837536	AJAP1;AJAP1	1	Body;Body	Island	20.39	6.46	28.00	9.82	-7.61	4.73E-05	1.48E-03
cg03220354	C8orf73	8	Body	N_Shore	58.19	8.28	65.79	8.51	-7.60	3.60E-04	7.85E-03
cg19120782	NUMA1	11	Body	Open sea	50.48	5.73	58.07	7.97	-7.60	9.39E-06	3.56E-04
cg17400609	LRP5	11	Body	Island	61.74	8.52	69.34	7.61	-7.60	5.47E-04	1.08E-02
cg08721112		1	Intergenic	S_Shore	58.15	3.94	65.74	4.34	-7.59	2.44E-16	1.63E-14
cg25887619	CUX1;CUX1;CUX1	7	Body;Body;Body	N_Shore	58.59	7.32	66.18	7.87	-7.59	2.55E-05	8.66E-04
cg21245816	LOC153684	5	Body	S_Shore	39.08	8.00	46.67	10.69	-7.59	5.94E-04	1.16E-02
cg03013946	SPINT3	20	Body	Open sea	42.66	7.94	50.25	9.77	-7.59	4.32E-04	9.05E-03
cg00143527	MESDC1	15	TSS1500	N_Shore	22.86	7.14	30.45	8.77	-7.59	7.12E-05	2.10E-03
cg11395698		1	Intergenic	Open sea	63.48	10.53	71.06	11.06	-7.59	2.03E-03	2.85E-02
cg16329197		12	Intergenic	Island	29.52	10.29	37.10	7.80	-7.58	2.04E-04	5.02E-03
cg18449739	DTX1;DTX1	12	1stExon;5'UTR	S_Shore	47.86	8.37	55.44	9.21	-7.58	7.84E-04	1.43E-02
cg24149364	RPL36AL	14	3'UTR	N_Shore	70.38	14.46	77.96	10.54	-7.58	2.94E-03	3.69E-02
cg14232218		9	Intergenic	Island	52.20	9.55	59.78	8.42	-7.58	1.10E-05	4.10E-04
cg05639679	KIAA1522	1	Body	Island	18.20	4.64	25.78	6.67	-7.58	3.81E-10	2.16E-08
cg13794565	HUS1	7	TSS1500	S_Shore	52.65	8.77	60.22	7.47	-7.57	4.38E-05	1.39E-03
cg01443285	SNTB1	8	Body	Open sea	50.91	6.16	58.49	5.94	-7.57	1.59E-07	7.80E-06
cg15837308		6	Intergenic	Open sea	47.61	12.83	55.18	13.19	-7.57	2.66E-03	3.45E-02
cg27418164	ZC3H4	19	Body	Island	43.94	8.04	51.51	9.42	-7.57	5.71E-07	2.65E-05
cg08982827	PC;PC;PC	11	5'UTR;5'UTR;5'UTR	Open sea	42.24	6.12	49.79	8.54	-7.55	4.83E-05	1.51E-03
cg09096671	PTDSS1	8	Body	Open sea	45.34	5.69	52.89	7.02	-7.55	1.04E-06	4.66E-05
cg11526665	CNIH3	1	Body	Open sea	53.20	4.00	60.75	5.63	-7.55	2.44E-10	1.39E-08
cg04209595	SCFD2	4	Body	Open sea	54.37	4.48	61.92	4.11	-7.55	1.05E-12	6.40E-11
cg04432597	AMIGO3;RNF123	3	TSS200;Body	Island	22.30	7.57	29.85	8.05	-7.54	1.09E-04	3.01E-03
cg03127349		10	Intergenic	Open sea	30.43	7.75	37.97	3.98	-7.54	1.11E-07	5.54E-06
cg15579587	LRRFIP1;LRRFIP1;LRRFIP1;LRRFIP1;LRI	2	TSS1500;TSS1500;TSS1500;TSS1500;Body	Island	44.11	7.24	51.65	5.88	-7.54	4.42E-06	1.79E-04
cg01047476	PNLIPRP2	10	TSS200	Open sea	48.08	5.05	55.62	5.81	-7.54	1.85E-08	9.70E-07
cg08388507	TG	8	Body	Open sea	63.16	7.20	70.70	8.85	-7.54	3.96E-05	1.27E-03
cg12587985		7	Intergenic	Open sea	80.10	8.68	87.64	3.58	-7.53	2.92E-07	1.40E-05
cg14651210		1	Intergenic	S_Shore	74.55	5.65	82.08	6.62	-7.53	2.02E-08	1.06E-06
cg13763783	LGI1	10	TSS200	Open sea	34.46	6.84	41.98	7.70	-7.53	1.20E-06	5.32E-05
cg19316407	NFATC1;NFATC1;NFATC1;NFATC1	18	Body;Body;Body;Body	Island	45.66	5.11	53.19	5.64	-7.52	1.44E-08	7.62E-07
cg00983697		1	Intergenic	S_Shelf	23.46	8.30	30.98	12.93	-7.52	2.76E-03	3.53E-02
cg11728738	FLJ22536	6	Body	Open sea	55.15	7.57	62.67	8.93	-7.52	1.45E-04	3.81E-03
cg21051815	HCCA2	11	Body	Open sea	32.26	6.86	39.77	8.50	-7.52	5.80E-05	1.77E-03
cg03272195	HS3ST3B1	17	Body	N_Shore	51.62	7.79	59.14	9.58	-7.52	2.52E-04	5.95E-03
cg00088797	IQSEC1;IQSEC1	3	Body;Body	N_Shore	59.99	4.38	67.50	4.65	-7.52	6.11E-11	3.55E-09
cg09509144	KCNQ1;KCNQ1	11	Body;Body	Open sea	62.56	7.27	70.07	9.68	-7.52	1.07E-04	2.97E-03
cg00626515	PMEPA1;PMEPA1;PMEPA1	20	TSS1500;TSS1500;TSS1500	S_Shore	58.55	6.23	66.06	8.05	-7.52	1.18E-05	4.36E-04
cg18620571	TNFAIP2	14	Body	Island	37.00	8.01	44.52	10.42	-7.51	9.97E-04	1.71E-02
cg06208270	MEX3A	1	3'UTR	Island	24.97	6.90	32.48	10.71	-7.51	2.96E-04	6.74E-03
cg17280346	ZIC1	3	TSS1500	N_Shore	30.82	5.87	38.33	8.40	-7.51	4.69E-07	2.19E-05
cg24801230	MAPT;MAPT;MAPT;MAPT;MAPT;MAI	17	5'UTR;5'UTR;5'UTR;5'UTR;5'UTR;5'UTR	S_Shelf	79.45	16.14	86.95	11.97	-7.50	2.23E-03	3.05E-02
cg00881448		10	Intergenic	Open sea	60.66	8.02	68.16	10.64	-7.50	5.45E-04	1.08E-02
cg05511872	SHANK2	11	Body	Open sea	37.42	7.79	44.92	7.13	-7.50	4.89E-05	1.53E-03
cg07674304	WWOX	16	Body	Open sea	34.09	4.98	41.58	6.98	-7.49	2.55E-08	1.32E-06

cg16848306		3	Intergenic	N_Shelf	39.34	6.75	46.83	8.31	-7.49	2.37E-05	8.13E-04
cg03586564	NFATC1;NFATC1;NFATC1	18	Body;5'UTR;Body	Island	78.27	9.18	85.76	7.07	-7.49	8.56E-05	2.46E-03
cg01363734	TESC;TESC;TESC	12	Body;Body;Body	N_Shore	37.29	7.53	44.78	11.44	-7.49	6.53E-04	1.24E-02
cg18671984	TUBB4;MIR220B	19	Body;TSS200	Island	77.76	2.63	85.24	2.98	-7.49	1.02E-20	7.42E-19
cg02909320	LZTFL1	3	Body	N_Shelf	48.40	7.94	55.88	9.95	-7.48	1.16E-03	1.91E-02
cg01833234	DNHD1	11	Body	Island	64.84	11.81	72.31	12.11	-7.48	2.51E-03	3.31E-02
cg05019807	NOTCH1	9	Body	N_Shore	66.83	8.91	74.30	8.86	-7.48	8.85E-04	1.56E-02
cg18896979		2	Intergenic	Island	58.44	10.56	65.92	10.22	-7.48	5.69E-04	1.12E-02
cg03352332	FETUB	3	Body	Open sea	77.10	7.18	84.58	5.51	-7.48	2.54E-06	1.07E-04
cg01515543	LOC100129066	9	TSS200	Open sea	63.40	5.91	70.87	3.75	-7.48	4.13E-09	2.25E-07
cg16119128	C15orf52	15	Body	Open sea	19.29	4.37	26.77	4.67	-7.47	1.86E-10	1.07E-08
cg23723818	PPAP2B;PPAP2B	1	Body;Body	Open sea	58.20	5.41	65.67	5.16	-7.47	8.63E-09	4.61E-07
cg17506621		6	Intergenic	Open sea	54.45	8.10	61.92	8.15	-7.47	2.18E-05	7.54E-04
cg13083776	RAB3B	1	Body	Open sea	45.19	4.27	52.67	4.97	-7.47	1.59E-11	9.43E-10
cg09068031	GALNTL4	11	Body	Open sea	70.62	7.97	78.09	9.20	-7.47	2.13E-04	5.19E-03
cg27624471	NFATC1;NFATC1;NFATC1;NFATC1	18	Body;Body;Body;Body	Island	52.13	6.40	59.60	7.06	-7.47	2.47E-06	1.04E-04
cg20893956	ESR1;ESR1	6	5'UTR;TSS200	N_Shelf	32.43	6.64	39.90	7.32	-7.47	4.10E-06	1.67E-04
cg10002888	BAT2	6	Body	S_Shelf	66.90	5.85	74.36	5.54	-7.47	1.72E-07	8.42E-06
cg16268707	PC;PC;PC	11	5'UTR;5'UTR;5'UTR	Open sea	68.44	6.26	75.91	5.94	-7.46	1.13E-06	5.04E-05
cg07015749	KCNAB2;KCNAB2	1	TSS1500;TSS1500	N_Shore	32.73	8.63	40.19	11.77	-7.46	1.36E-03	2.15E-02
cg27582323	EPHA2	1	Body	N_Shelf	45.58	10.22	53.04	9.74	-7.46	1.99E-03	2.82E-02
cg12807275	AVPR1A	12	Body	N_Shore	50.15	6.33	57.61	9.02	-7.46	4.11E-05	1.31E-03
cg22404125	TSPAN5	4	Body	Open sea	55.94	8.48	63.40	8.15	-7.46	3.99E-04	8.51E-03
cg03485909	FAM3C;FAM3C	7	5'UTR;5'UTR	N_Shore	66.07	5.93	73.52	6.39	-7.46	1.67E-07	8.17E-06
cg12140851	SKAP2	7	Body	Open sea	32.27	6.93	39.73	7.86	-7.45	1.17E-07	5.79E-06
cg19255477	LOC100132111;C2CD4D	1	TSS1500;Body	Island	15.69	6.86	23.14	10.63	-7.45	8.76E-05	2.51E-03
cg15408080		12	Intergenic	Open sea	33.51	6.11	40.96	5.38	-7.45	3.94E-08	2.02E-06
cg14417784		2	Intergenic	Island	24.66	7.05	32.11	10.24	-7.45	1.12E-04	3.07E-03
cg14825413	GLI4	8	Body	Island	16.53	7.91	23.98	7.00	-7.45	1.07E-06	4.79E-05
cg26921566	REEP6	19	3'UTR	S_Shore	51.74	5.33	59.19	7.04	-7.45	1.10E-06	4.92E-05
cg06928246		1	Intergenic	Island	51.72	7.06	59.17	6.08	-7.45	1.98E-06	8.50E-05
cg23880736		4	Intergenic	Island	54.69	8.99	62.14	7.99	-7.45	5.15E-04	1.04E-02
cg19563158	MAPKAPK5;MAPKAPK5	12	Body;Body	S_Shelf	52.37	5.73	59.82	5.99	-7.44	4.35E-07	2.05E-05
cg16955074	P2RX4	12	3'UTR	Open sea	78.58	7.30	86.02	5.94	-7.44	9.61E-06	3.64E-04
cg26470309	FBXO21;FBXO21	12	Body;Body	N_Shore	38.21	7.86	45.65	9.21	-7.44	9.62E-04	1.67E-02
cg19238349	CAB39L;CAB39L	13	Body;Body	Open sea	42.93	8.39	50.36	9.11	-7.44	1.05E-06	4.71E-05
cg01511231	TTLL10	1	5'UTR	Island	32.61	8.77	40.05	11.32	-7.44	1.58E-03	2.39E-02
cg03574306		16	Intergenic	Open sea	55.62	7.68	63.05	5.92	-7.43	2.11E-06	9.01E-05
cg10872209	SFXN2;ARL3	10	5'UTR;TSS1500	S_Shore	33.65	6.47	41.08	6.59	-7.43	3.48E-06	1.43E-04
cg07868599	CCDC64B	16	Body	Island	20.11	6.38	27.54	10.11	-7.43	1.25E-04	3.36E-03
cg20655099	TNRC18	7	Body	N_Shore	55.05	5.31	62.47	4.39	-7.42	1.42E-09	7.89E-08
cg10746278		11	Intergenic	Open sea	65.65	6.65	73.07	7.45	-7.42	9.16E-07	4.13E-05
cg05237469		11	Intergenic	Open sea	50.05	10.06	57.47	10.65	-7.42	5.29E-04	1.06E-02
cg05980111	KALRN;KALRN	3	Body;Body	Open sea	40.94	6.86	48.36	8.61	-7.42	9.73E-05	2.74E-03
cg07332757	ZFPM1	16	Body	S_Shelf	44.01	6.35	51.42	4.66	-7.41	4.80E-07	2.25E-05
cg14709479	TTLL10	1	TSS1500	N_Shore	23.62	8.12	31.03	10.37	-7.41	7.88E-04	1.43E-02
cg03417191	MAP6D1	3	Body	Island	29.40	6.62	36.81	5.66	-7.41	5.84E-08	2.97E-06
cg07119434		1	Intergenic	N_Shore	34.45	7.69	41.86	8.16	-7.41	5.89E-07	2.73E-05
cg07891179	WIPF3	7	Body	Island	40.13	6.09	47.54	7.30	-7.41	3.54E-07	1.68E-05
cg14151317	ABCA2;ABCA2	9	Body;Body	N_Shore	53.64	7.58	61.05	9.37	-7.41	2.70E-04	6.27E-03
cg10271819	HIPK2;HIPK2	7	Body;Body	Open sea	50.03	4.31	57.43	4.47	-7.41	4.26E-11	2.49E-09
cg17953716		8	Intergenic	Open sea	34.44	9.90	41.85	10.68	-7.40	2.71E-03	3.50E-02
cg02548777		6	Intergenic	Island	62.86	7.08	70.26	8.71	-7.40	5.61E-06	2.22E-04

cg00103806	ANKRD22	10	TSS1500	Open sea	56.33	6.25	63.73	7.95	-7.40	2.09E-05	7.26E-04
cg26355737	TFDP1;TFDP1	13	Body;Body	N_Shore	83.12	4.46	90.51	3.81	-7.39	1.62E-14	1.04E-12
cg01826444	FNDC7	1	TSS200	Open sea	57.95	8.29	65.34	10.43	-7.39	1.28E-03	2.06E-02
cg19025272	SPATS2L;SPATS2L;SPATS2L;SPATS2L	2	5'UTR;5'UTR;5'UTR;5'UTR	Open sea	73.66	5.36	81.05	2.77	-7.39	1.91E-12	1.16E-10
cg00213281	KIF5C;MIR1978	2	Body;TSS1500	Open sea	38.60	5.45	45.99	7.48	-7.39	4.63E-07	2.17E-05
cg06515235		13	Intergenic	N_Shore	15.22	6.38	22.60	9.84	-7.38	2.28E-05	7.86E-04
cg06051411	KCTD2	17	Body	S_Shore	78.31	5.83	85.69	3.83	-7.38	2.56E-09	1.41E-07
cg26665904	A2M	12	Body	Open sea	41.32	6.55	48.70	6.10	-7.38	1.49E-05	5.37E-04
cg23003807		8	Intergenic	Island	65.93	8.64	73.31	7.32	-7.38	1.68E-05	5.98E-04
cg06226156	LBX2	2	Body	Island	19.13	5.80	26.51	7.16	-7.38	2.55E-06	1.07E-04
cg04143909	SLC35F3	1	Body	Island	65.73	9.41	73.10	9.21	-7.37	3.06E-04	6.90E-03
cg15954675	SYNPR;SYNPR	3	Body;Body	Open sea	44.70	8.23	52.07	10.86	-7.37	1.58E-03	2.39E-02
cg13691961	DUSP6;DUSP6	12	Body;Body	N_Shore	35.45	6.21	42.82	7.58	-7.37	2.15E-06	9.18E-05
cg11586249	EXT1	8	Body	Open sea	57.23	7.76	64.59	8.62	-7.37	1.16E-05	4.30E-04
cg12374353		3	Intergenic	Open sea	67.43	5.92	74.79	4.40	-7.36	3.71E-09	2.02E-07
cg22695986		7	Intergenic	Island	14.52	8.45	21.88	9.09	-7.36	7.29E-05	2.15E-03
cg00654637	MIR219-2	9	TSS1500	S_Shore	60.78	7.32	68.13	9.30	-7.36	2.60E-04	6.08E-03
cg19645639	SLC16A1	1	TSS1500	S_Shore	33.10	7.54	40.46	7.86	-7.35	2.76E-05	9.27E-04
cg19707359	FAM188A	10	TSS1500	S_Shore	58.71	5.40	66.06	5.66	-7.35	5.61E-08	2.85E-06
cg15659713	TACC1;TACC1	8	1stExon;5'UTR	Open sea	17.94	8.06	25.30	11.44	-7.35	2.30E-03	3.12E-02
cg22579218	SRI;SRI	7	Body;Body	N_Shore	22.88	10.71	30.23	11.90	-7.35	3.13E-03	3.85E-02
cg07075085	ADAMTS9	3	Body	Open sea	35.76	5.81	43.11	8.86	-7.35	5.34E-05	1.64E-03
cg03001143	METTL11B	1	TSS1500	Open sea	41.90	6.80	49.25	9.02	-7.35	4.90E-05	1.53E-03
cg23090529		1	Intergenic	N_Shore	45.86	5.69	53.21	7.71	-7.35	1.95E-06	8.37E-05
cg08819417	FAM100A	16	3'UTR	Island	74.81	9.26	82.16	8.07	-7.35	1.65E-03	2.46E-02
cg00839579	MEOX2	7	TSS1500	Open sea	27.02	5.84	34.36	6.61	-7.34	1.13E-08	6.01E-07
cg22032709	CAMKK2;CAMKK2;CAMKK2;CAMKK2;(	12	3'UTR;3'UTR;3'UTR;3'UTR;3'UTR	Open sea	23.06	4.64	30.40	6.43	-7.34	7.40E-08	3.73E-06
cg16785000		6	Intergenic	Open sea	77.52	6.82	84.86	4.16	-7.34	1.03E-06	4.62E-05
cg16324314		14	Intergenic	Open sea	49.25	7.12	56.59	6.34	-7.34	3.62E-07	1.72E-05
cg05955210	OSMR;OSMR	5	5'UTR;5'UTR	S_Shore	31.36	5.68	38.70	7.22	-7.34	2.33E-06	9.88E-05
cg18043888	RHOD	11	3'UTR	Island	22.35	6.75	29.69	10.86	-7.34	4.27E-04	8.97E-03
cg27571374		19	Intergenic	S_Shore	60.46	7.75	67.80	8.45	-7.34	5.40E-05	1.66E-03
cg11235543	MGMT	10	Body	Open sea	50.14	6.09	57.47	7.49	-7.33	1.46E-05	5.30E-04
cg18376497	INPP4B;INPP4B	4	5'UTR;5'UTR	Open sea	61.18	7.99	68.51	6.21	-7.33	1.17E-06	5.20E-05
cg03829559		2	Intergenic	N_Shore	46.58	7.61	53.91	7.48	-7.33	2.63E-04	6.14E-03
cg16929139	RNF125	18	Body	S_Shelf	61.24	6.12	68.58	5.95	-7.33	1.33E-06	5.88E-05
cg23766996	GRM3	7	TSS1500	Open sea	40.18	8.30	47.51	10.44	-7.33	2.63E-03	3.42E-02
cg04547244	OSBPL3;OSBPL3;OSBPL3;OSBPL3	7	5'UTR;5'UTR;5'UTR;5'UTR	Island	15.52	7.23	22.85	8.34	-7.33	2.06E-06	8.83E-05
cg27017251	TOP3B	22	5'UTR	N_Shelf	69.91	7.41	77.23	6.66	-7.32	8.75E-05	2.50E-03
cg21817750	MRPS27	5	Body	Open sea	61.55	9.02	68.87	7.86	-7.32	1.60E-03	2.41E-02
cg02953007	ZCCHC24	10	Body	Open sea	70.71	7.42	78.03	7.00	-7.32	4.07E-05	1.30E-03
cg01297721	HAPLN4;HAPLN4	19	5'UTR;1stExon	S_Shore	26.72	6.29	34.03	6.75	-7.31	3.70E-08	1.91E-06
cg07645761	TMPRSS8	16	Body	N_Shore	47.51	7.51	54.82	8.30	-7.31	1.21E-04	3.27E-03
cg13997765		8	Intergenic	S_Shore	50.85	6.11	58.16	6.45	-7.31	6.70E-06	2.61E-04
cg24593832		1	Intergenic	Open sea	43.22	9.46	50.53	7.22	-7.31	2.38E-04	5.68E-03
cg25857695		3	Intergenic	Open sea	57.74	5.63	65.05	6.55	-7.31	1.30E-08	6.89E-07
cg22567585		15	Intergenic	S_Shore	24.71	5.89	32.02	7.64	-7.30	9.25E-07	4.18E-05
cg08806558	IGF1;IGF1;IGF1	12	TSS1500;TSS1500;TSS1500	Open sea	55.30	9.21	62.61	9.44	-7.30	2.18E-03	3.00E-02
cg26915952		1	Intergenic	Island	49.08	6.55	56.39	8.74	-7.30	3.38E-05	1.11E-03
cg24693760		1	Intergenic	Open sea	48.14	11.94	55.44	12.18	-7.30	1.23E-03	2.00E-02
cg13849419	TJP2;TJP2;TJP2;TJP2	9	Body;5'UTR;Body;Body	Open sea	46.56	6.32	53.85	4.74	-7.30	2.68E-07	1.29E-05
cg22336669		12	Intergenic	N_Shore	36.76	7.27	44.05	10.01	-7.30	4.23E-04	8.90E-03
cg15423872	FAM110B	8	TSS1500	N_Shore	27.00	5.70	34.30	5.92	-7.29	3.16E-07	1.51E-05

cg24956533	LOC145845	15	Body	S_Shore	47.63	7.59	54.92	8.80	-7.29	6.16E-05	1.86E-03
cg05135499		10	Intergenic	Island	46.42	7.64	53.71	11.53	-7.29	1.78E-03	2.61E-02
cg18113560		4	Intergenic	S_Shore	59.97	9.47	67.25	7.89	-7.28	2.27E-04	5.47E-03
cg26433333		5	Intergenic	Open sea	68.85	8.02	76.13	8.05	-7.28	1.85E-04	4.64E-03
cg06471596	CUX1;CUX1;CUX1	7	Body;Body;Body	Open sea	29.28	6.68	36.56	9.31	-7.28	2.25E-04	5.44E-03
cg12708994	DSE;DSE	6	TSS1500;5'UTR	N_Shore	59.54	9.33	66.82	6.13	-7.28	5.90E-04	1.15E-02
cg21389743	CLDN11	3	TSS200	Island	7.30	4.18	14.57	8.21	-7.28	3.81E-07	1.81E-05
cg01766505		17	Intergenic	N_Shore	51.62	7.33	58.89	6.32	-7.27	2.81E-05	9.42E-04
cg00905220	ABR;ABR;ABR	17	Body;Body;Body	S_Shore	60.34	9.09	67.61	11.37	-7.27	4.52E-03	4.96E-02
cg18087143		7	Intergenic	Open sea	33.62	6.89	40.89	7.37	-7.27	4.13E-05	1.32E-03
cg03356172	MAB21L1;MIR548F5;NBEA	13	1stExon;Body;Body	N_Shore	29.23	5.81	36.50	9.71	-7.27	7.27E-06	2.82E-04
cg02311794	KLHL25	15	3'UTR	S_Shore	66.64	6.17	73.91	5.39	-7.27	6.12E-08	3.10E-06
cg06630241	SOCS2	12	Body	S_Shore	61.25	7.45	68.52	6.04	-7.27	9.85E-07	4.43E-05
cg01446287	ANKRD33B	5	Body	Island	68.70	7.45	75.96	8.75	-7.26	1.77E-04	4.48E-03
cg25544075	TTLL10	1	5'UTR	Island	16.35	6.55	23.61	10.14	-7.26	1.34E-04	3.56E-03
cg14836313	MRPL23	11	Body	N_Shore	39.34	5.43	46.60	6.00	-7.26	1.62E-07	7.92E-06
cg19594052		7	Intergenic	N_Shore	58.29	7.51	65.55	9.14	-7.26	2.79E-04	6.43E-03
cg25433316	CYS1	2	3'UTR	Open sea	41.52	6.26	48.78	6.13	-7.26	5.77E-07	2.67E-05
cg12934382	GRM2;GRM2;GRM2;GRM2	3	1stExon;5'UTR;1stExon;5'UTR	Island	31.43	7.65	38.68	6.69	-7.25	1.28E-06	5.64E-05
cg01113530		10	Intergenic	N_Shelf	63.20	7.99	70.45	7.28	-7.25	1.66E-04	4.25E-03
cg05237374	GPHN;GPHN	14	Body;Body	S_Shelf	39.62	6.27	46.87	6.02	-7.25	2.95E-06	1.23E-04
cg26985203	ALKBH3	11	5'UTR	S_Shore	50.82	6.35	58.07	6.39	-7.24	3.52E-06	1.45E-04
cg17815035		18	Intergenic	S_Shore	47.44	5.60	54.68	6.51	-7.24	9.41E-07	4.24E-05
cg24011500	VWCE	11	Body	Open sea	55.06	6.13	62.30	5.94	-7.24	5.57E-08	2.83E-06
cg01194674	COL11A2;COL11A2;COL11A2	6	Body;Body;Body	Open sea	51.06	4.66	58.30	6.45	-7.24	8.90E-09	4.75E-07
cg07156115	AHR	7	Body	Open sea	79.37	6.63	86.60	4.81	-7.24	1.53E-06	6.69E-05
cg15046062	HTRA4	8	1stExon	Island	38.01	6.82	45.25	8.97	-7.24	4.43E-05	1.40E-03
cg21004358		4	Intergenic	Island	68.35	9.24	75.59	7.73	-7.24	1.23E-04	3.33E-03
cg20474425		12	Intergenic	Open sea	37.24	6.11	44.47	7.46	-7.23	3.88E-06	1.58E-04
cg12217625	VSIG8;C1orf204	1	Body;TSS1500	S_Shore	43.25	6.23	50.48	8.49	-7.23	3.38E-05	1.11E-03
cg05208056	NCK2;NCK2;NCK2	2	Body;Body;Body	Open sea	40.20	5.22	47.43	5.92	-7.23	5.16E-08	2.63E-06
cg23666945	TRIM71	3	Body	Island	43.82	5.37	51.05	6.72	-7.23	6.24E-07	2.88E-05
cg21146184		22	Intergenic	Island	24.33	5.13	31.56	8.30	-7.23	4.42E-07	2.08E-05
cg00437102		2	Intergenic	Open sea	35.50	6.72	42.72	10.39	-7.23	4.44E-04	9.24E-03
cg15168497	AHRR	5	Body	Open sea	75.14	11.52	82.37	6.87	-7.22	4.15E-03	4.68E-02
cg06841262	HS3ST3B1;MGC12916	17	Body;Body	S_Shore	67.46	6.26	74.68	7.20	-7.22	1.00E-06	4.49E-05
cg16727519	LOC440926;H3F3A	1	Body;Body	S_Shore	42.82	3.08	50.04	4.33	-7.22	3.32E-13	2.06E-11
cg05053327	CNTNAP1	17	Body	Island	49.10	5.84	56.32	6.86	-7.22	5.70E-06	2.26E-04
cg25999827	FAM190B	10	5'UTR	Open sea	71.25	6.46	78.47	5.86	-7.22	1.26E-07	6.24E-06
cg18886436	ZNF385A;ZNF385A	12	TSS200;TSS200	Island	36.62	8.31	43.84	8.08	-7.22	6.38E-06	2.50E-04
cg04145765	TRIM55;TRIM55;TRIM55;TRIM55;TRIN	8	5'UTR;1stExon;5'UTR;1stExon;5'UTR;1stEx	Open sea	49.94	7.66	57.16	10.58	-7.22	2.13E-03	2.96E-02
cg00672802		1	Intergenic	N_Shore	38.98	6.26	46.20	6.10	-7.21	1.86E-06	8.02E-05
cg09128751	CBS	21	Body	Island	73.22	6.69	80.43	7.19	-7.21	1.14E-04	3.13E-03
cg25416125	DGKA;DGKA;DGKA;DGKA	12	5'UTR;5'UTR;5'UTR;5'UTR	Island	38.26	5.19	45.46	7.06	-7.21	3.02E-07	1.45E-05
cg02977575	MEX3A	1	Body	Island	32.64	6.72	39.85	9.01	-7.21	2.55E-04	6.01E-03
cg04582294	GRID1	10	Body	Island	25.27	8.22	32.47	9.50	-7.21	2.02E-04	4.98E-03
cg18459618	RNF135;RNF135	17	TSS1500;TSS1500	N_Shore	43.86	6.25	51.06	7.55	-7.20	5.60E-06	2.22E-04
cg20722088	DUSP6;DUSP6	12	3'UTR;3'UTR	N_Shelf	31.98	5.62	39.18	7.61	-7.20	1.99E-06	8.54E-05
cg15701111	KRT81	12	TSS200	S_Shore	51.39	7.68	58.60	11.16	-7.20	1.59E-03	2.40E-02
cg11265958		15	Intergenic	Island	46.46	5.36	53.66	6.92	-7.20	1.21E-07	5.97E-06
cg09002919		12	Intergenic	Open sea	36.52	5.66	43.71	6.01	-7.19	2.16E-07	1.05E-05
cg04189838	CYP2C19	10	Body	Open sea	78.93	10.67	86.13	7.55	-7.19	1.82E-03	2.65E-02
cg06226386	FLJ14107;BIN3	8	TSS1500;Body	Open sea	65.62	5.81	72.81	5.56	-7.19	4.36E-07	2.05E-05

cg03268942		2	Intergenic	Open sea	41.24	5.34	48.44	7.00	-7.19	3.00E-07	1.44E-05
cg13879776	CLDN11	3	TSS1500	Island	36.54	6.55	43.74	7.69	-7.19	5.72E-06	2.26E-04
cg12289801	SFRS14;SFRS14	19	Body;Body	Open sea	49.28	5.44	56.47	4.09	-7.19	7.31E-10	4.09E-08
cg09133706	SIRT5;SIRT5	6	Body;Body	Open sea	78.73	5.37	85.92	2.83	-7.19	2.72E-10	1.55E-08
cg03356595		16	Intergenic	Island	36.41	8.23	43.60	9.41	-7.19	7.52E-04	1.38E-02
cg12664749		1	Intergenic	Open sea	50.10	8.13	57.29	7.86	-7.18	1.26E-03	2.04E-02
cg20431191	DDAH1;DDAH1	1	1stExon;5'UTR	S_Shore	14.42	5.36	21.61	8.50	-7.18	1.03E-06	4.62E-05
cg22821220	MIR563	3	Body	Open sea	64.29	8.70	71.48	7.61	-7.18	4.44E-04	9.24E-03
cg18110104	FNDC3B;FNDC3B	3	3'UTR;3'UTR	Open sea	76.50	6.79	83.69	3.60	-7.18	2.16E-08	1.13E-06
cg19150842	GK5	3	3'UTR	Open sea	63.52	6.69	70.71	5.58	-7.18	7.77E-07	3.54E-05
cg24127414	PCDHB11	5	1stExon	N_Shore	59.16	9.38	66.34	8.78	-7.18	4.78E-04	9.78E-03
cg24865623	EHD2	19	Body	S_Shelf	26.84	5.77	34.02	9.08	-7.18	5.02E-06	2.01E-04
cg27069566	PCOLCE;PCOLCE	7	1stExon;5'UTR	N_Shore	40.76	6.45	47.93	9.52	-7.17	1.40E-04	3.70E-03
cg10501093	TNFAIP2	14	Body	Island	34.83	7.56	42.01	10.03	-7.17	9.91E-04	1.70E-02
cg26326607	MRPL23	11	Body	N_Shore	75.61	6.42	82.78	4.66	-7.17	1.58E-07	7.74E-06
cg01723606	SH2B2	7	Body	Island	47.20	7.14	54.38	9.09	-7.17	1.62E-04	4.17E-03
cg21586152	ELANE	19	Body	N_Shore	53.49	6.85	60.66	7.68	-7.17	4.03E-05	1.29E-03
cg06847828	BBOX1	11	Body	Open sea	60.91	5.85	68.08	3.29	-7.17	1.36E-09	7.53E-08
cg07698121	KIAA0182;KIAA0182	16	5'UTR;Body	S_Shore	30.14	4.50	37.31	7.04	-7.17	1.31E-07	6.48E-06
cg19405088	AFF3;AFF3	2	Body;Body	Open sea	82.70	6.65	89.87	3.02	-7.17	5.66E-10	3.19E-08
cg22158462	PLXNA2	1	Body	Open sea	46.71	6.87	53.88	7.31	-7.17	2.26E-08	1.18E-06
cg00036976		16	Intergenic	Open sea	54.44	8.43	61.60	8.49	-7.17	3.04E-04	6.87E-03
cg23878906		18	Intergenic	S_Shore	47.63	7.23	54.79	8.68	-7.17	8.85E-04	1.56E-02
cg00251405	KIAA1522	1	Body	N_Shore	23.27	7.51	30.44	9.22	-7.17	1.94E-05	6.81E-04
cg12244668	NXNL1	19	3'UTR	N_Shore	51.85	6.92	59.01	7.14	-7.16	4.95E-06	1.98E-04
cg10981580	GPR137B	1	Body	S_Shore	15.92	7.66	23.08	7.40	-7.16	2.77E-06	1.16E-04
cg01032851	TMCO7	16	Body	Open sea	61.78	7.51	68.94	7.80	-7.16	4.13E-04	8.74E-03
cg10420609		6	Intergenic	N_Shelf	68.28	5.84	75.44	5.62	-7.16	1.24E-07	6.13E-06
cg26382697	CD81	11	Body	Island	46.82	7.82	53.98	6.72	-7.16	1.72E-04	4.38E-03
cg02091100	GUCA1A	6	TSS200	Open sea	53.87	6.77	61.02	6.40	-7.15	7.98E-07	3.63E-05
cg15661753		12	Intergenic	N_Shore	28.31	4.78	35.46	6.33	-7.15	2.89E-07	1.39E-05
cg04193820	RNF135;RNF135	17	TSS1500;TSS1500	N_Shore	60.44	6.98	67.59	5.95	-7.15	4.86E-06	1.95E-04
cg14219938	C9orf103	9	TSS1500	N_Shore	53.61	5.25	60.76	4.47	-7.15	3.28E-08	1.69E-06
cg10364249	AHDC1	1	5'UTR	Island	54.23	6.41	61.38	5.61	-7.15	3.41E-07	1.62E-05
cg05791356	ATG7;ATG7;ATG7	3	Body;Body;Body	Open sea	72.75	6.41	79.90	5.74	-7.15	1.48E-07	7.30E-06
cg16170614	SOD3	4	3'UTR	S_Shore	40.95	4.96	48.09	7.02	-7.14	3.43E-08	1.77E-06
cg07127888	GINS4	8	3'UTR	Open sea	25.89	6.10	33.03	7.75	-7.14	3.71E-05	1.20E-03
cg08110488		1	Intergenic	Open sea	32.24	8.56	39.38	9.97	-7.14	2.04E-04	5.02E-03
cg04507915	TRIM71	3	Body	Island	41.99	6.82	49.13	6.67	-7.13	3.40E-05	1.11E-03
cg14916924	LMX1B	9	Body	Island	26.27	5.47	33.40	6.52	-7.13	2.98E-07	1.43E-05
cg15164782	COL8A1;MIR548G;COL8A1	3	5'UTR;Body;5'UTR	Open sea	50.91	6.98	58.04	6.75	-7.13	1.89E-05	6.64E-04
cg00461685		8	Intergenic	Island	57.49	7.41	64.62	7.62	-7.13	2.20E-05	7.62E-04
cg16236228		12	Intergenic	N_Shelf	52.27	4.95	59.40	4.46	-7.13	1.64E-09	9.04E-08
cg27558820	SOLH	16	5'UTR	Island	67.99	5.84	75.12	7.54	-7.13	2.81E-06	1.17E-04
cg24768135	CHRNE;C17orf107	17	Body;3'UTR	Island	56.22	10.79	63.34	8.65	-7.13	2.60E-03	3.39E-02
cg11282828		5	Intergenic	Open sea	64.85	12.19	71.98	5.37	-7.12	2.29E-03	3.11E-02
cg13707943	FAM102A;FAM102A	9	Body;Body	N_Shore	56.02	5.41	63.14	5.67	-7.12	5.51E-08	2.80E-06
cg03656877		6	Intergenic	N_Shore	74.82	9.23	81.94	6.90	-7.12	2.88E-04	6.60E-03
cg20852364		3	Intergenic	Open sea	77.30	7.44	84.42	5.28	-7.12	1.86E-06	8.01E-05
cg07306604	RTN4R	22	Body	Island	52.36	5.14	59.48	7.61	-7.12	8.41E-06	3.22E-04
cg20638675		10	Intergenic	Open sea	21.79	8.42	28.91	10.73	-7.12	3.51E-03	4.18E-02
cg01833485	ESRRG;ESRRG;ESRRG;ESRRG;ESRRG	1	5'UTR;Body;5'UTR;5'UTR;Body	Open sea	32.37	7.26	39.49	11.77	-7.12	8.32E-04	1.49E-02
cg27460531	RPTOR;RPTOR	17	Body;Body	S_Shore	50.69	5.90	57.80	5.72	-7.11	2.01E-06	8.64E-05

cg07401067		5	Intergenic	Island	37.57	7.09	44.68	10.00	-7.11	7.84E-04	1.43E-02
cg24227371	RAB30	11	5'UTR	Open sea	60.80	8.05	67.91	8.57	-7.11	3.45E-04	7.59E-03
cg03989270		1	Intergenic	Open sea	58.43	5.60	65.53	5.75	-7.10	7.21E-07	3.30E-05
cg18838701	TNNI3	19	Body	S_Shore	23.80	5.94	30.91	7.07	-7.10	2.51E-06	1.06E-04
cg12749132	LOC100132111;C2CD4D	1	Body;Body	Island	10.43	6.40	17.53	10.79	-7.10	1.47E-04	3.85E-03
cg02793451	TOX3;TOX3	16	TSS1500;TSS1500	S_Shore	40.77	8.18	47.88	9.30	-7.10	1.11E-04	3.04E-03
cg04315771	NUP62;NUP62;IL4I1;ATF5;NUP62;NUF	19	TSS1500;TSS1500;TSS1500;5'UTR;TSS1500	S_Shore	27.03	5.74	34.13	8.34	-7.10	2.62E-05	8.89E-04
cg11729019	C1R	12	Body	Open sea	58.23	5.66	65.33	8.19	-7.10	3.88E-05	1.25E-03
cg09282201	PRDM16;PRDM16	1	Body;Body	Island	60.43	8.08	67.53	11.93	-7.10	2.42E-03	3.23E-02
cg12858902	GSTM5	1	TSS200	Open sea	34.77	5.58	41.87	8.55	-7.10	4.16E-05	1.32E-03
cg23717186	ITPKB	1	Body	N_Shore	39.99	6.03	47.08	8.11	-7.10	2.22E-05	7.68E-04
cg03980224	RAI14;RAI14;RAI14;RAI14;RAI14;RAI1	5	TSS1500;5'UTR;5'UTR;5'UTR;5'UTR;5'UTR	Open sea	46.75	5.66	53.84	5.05	-7.10	2.66E-08	1.38E-06
cg06375292	SLC35B1	17	TSS1500	Open sea	54.42	6.84	61.51	5.05	-7.09	1.42E-05	5.17E-04
cg01616529	DRD4	11	Body	Island	56.95	7.87	64.04	8.43	-7.09	8.40E-05	2.42E-03
cg19296405	KIAA1683;KIAA1683;KIAA1683	19	Body;Body;Body	Island	69.06	6.71	76.15	6.18	-7.09	6.01E-06	2.36E-04
cg07382129	MEF2A;MEF2A;MEF2A;MEF2A	15	Body;Body;Body;Body	Open sea	63.80	5.44	70.89	4.04	-7.09	2.04E-10	1.17E-08
cg16351002	FAM24B	10	5'UTR	Open sea	62.32	5.41	69.40	5.71	-7.09	1.42E-07	7.01E-06
cg13814654	TBC1D8	2	Body	Open sea	63.72	7.50	70.80	8.70	-7.09	1.23E-03	2.00E-02
cg16820616	MPPED2	11	5'UTR	Island	44.57	7.09	51.66	6.15	-7.08	1.79E-06	7.74E-05
cg19408572	DGKQ;DGKQ	4	5'UTR;1stExon	Island	11.54	10.08	18.63	12.94	-7.08	6.52E-04	1.24E-02
cg23994043	SATB2	2	TSS1500	Island	20.86	4.35	27.94	6.73	-7.08	3.43E-08	1.77E-06
cg16405432		14	Intergenic	Open sea	37.83	6.29	44.91	7.10	-7.08	2.30E-05	7.91E-04
cg10507304		6	Intergenic	Open sea	29.46	6.30	36.54	7.44	-7.08	1.60E-05	5.73E-04
cg04930463	SLC38A10;SLC38A10	17	Body;Body	N_Shore	75.74	8.88	82.81	6.76	-7.07	6.06E-04	1.18E-02
cg13667739	CRIP2	14	Body	Island	43.11	7.53	50.18	6.66	-7.07	2.70E-05	9.09E-04
cg06197751	SGEF	3	Body	Open sea	55.34	6.35	62.41	6.40	-7.07	9.96E-07	4.47E-05
cg01878308	KDM3A;KDM3A	2	3'UTR;3'UTR	Open sea	59.74	6.80	66.81	5.40	-7.07	6.06E-06	2.38E-04
cg06832339	STK31;STK31;STK31	7	Body;Body;Body	Open sea	65.61	5.70	72.68	4.57	-7.07	5.41E-10	3.05E-08
cg24237576	DNASE1L2	16	TSS1500	N_Shore	49.68	5.30	56.75	5.08	-7.07	5.90E-08	3.00E-06
cg07115626		14	Intergenic	S_Shelf	28.43	5.85	35.50	7.59	-7.07	3.19E-06	1.32E-04
cg15657936	GPR78	4	1stExon	Island	67.21	6.67	74.28	6.54	-7.07	2.71E-06	1.13E-04
cg01616225	GPR56;GPR56;GPR56;GPR56;GPR56;G	16	TSS200;TSS200;TSS200;5'UTR;TSS200;5'U1	Open sea	23.38	6.32	30.44	9.65	-7.06	1.12E-04	3.07E-03
cg09833911		16	Intergenic	Island	37.48	5.15	44.55	5.51	-7.06	7.37E-09	3.95E-07
cg25015733	MGAT4A	2	5'UTR	N_Shelf	50.61	7.27	57.67	9.26	-7.06	7.86E-04	1.43E-02
cg13812731	NRGN;NRGN	11	Body;Body	N_Shore	52.86	7.29	59.92	10.13	-7.06	1.08E-03	1.82E-02
cg20059832	SDCCAG8;AKT3	1	Body;3'UTR	S_Shore	70.10	7.92	77.16	7.32	-7.06	3.72E-04	8.05E-03
cg25294185	RNASEH2C	11	Body	Island	11.98	4.94	19.04	3.33	-7.06	1.13E-10	6.53E-09
cg22581974	FAM83F	22	3'UTR	Open sea	77.26	5.84	84.32	5.26	-7.06	1.52E-07	7.47E-06
cg18775702	C15orf39	15	Body	S_Shore	74.47	6.63	81.52	4.31	-7.06	6.02E-07	2.79E-05
cg09683562	RNF135;RNF135	17	TSS1500;TSS1500	N_Shore	23.46	4.36	30.52	6.34	-7.06	4.35E-08	2.23E-06
cg01286685	RNF39;RNF39	6	Body;Body	Island	36.14	7.09	43.19	9.33	-7.06	4.55E-05	1.43E-03
cg15654897	DCAF15	19	Body	S_Shore	71.67	5.79	78.72	4.43	-7.05	3.66E-08	1.89E-06
cg00014194	OLFM1;OLFM1	9	Body;Body	N_Shore	30.68	6.78	37.73	5.30	-7.05	2.36E-06	9.99E-05
cg02512860	CLDN15	7	1stExon	Open sea	51.18	7.54	58.23	6.92	-7.05	9.79E-05	2.75E-03
cg10116893	NTN1	17	Body	Island	41.80	7.14	48.85	7.51	-7.05	6.04E-07	2.79E-05
cg09462956	SLC9A3R2;SLC9A3R2	16	Body;Body	N_Shelf	31.80	7.32	38.85	7.24	-7.05	2.08E-04	5.10E-03
cg07133994	PRKD1	14	Body	Open sea	77.87	7.30	84.91	5.73	-7.05	5.05E-07	2.36E-05
cg26800786	KBTBD3;KBTBD3	11	3'UTR;3'UTR	Open sea	57.39	6.29	64.43	4.59	-7.04	3.54E-07	1.68E-05
cg17894755	C10orf26;C10orf26	10	TSS200;Body	Open sea	36.19	6.32	43.23	6.06	-7.04	6.40E-06	2.50E-04
cg23635789	SRC;SRC	20	Body;Body	Island	30.13	7.15	37.17	10.14	-7.04	8.20E-04	1.47E-02
cg20132775	TRPC1	3	Body	S_Shore	45.23	6.90	52.27	8.77	-7.04	1.90E-04	4.74E-03
cg04439374	MAD1L1;MAD1L1;MAD1L1	7	Body;Body;Body	Island	74.95	11.32	81.98	8.91	-7.04	4.28E-03	4.78E-02
cg01310875	C8orf80	8	5'UTR	Open sea	71.94	6.95	78.97	5.04	-7.04	1.66E-06	7.20E-05

cg03014829		7	Intergenic	Island	66.70	8.73	73.73	6.06	-7.03	2.29E-04	5.51E-03
cg22243298	MIR1276;KLHL25	15	TSS200;5'UTR	S_Shore	60.38	7.96	67.41	8.23	-7.03	7.43E-05	2.18E-03
cg06634460	VPS37B	12	TSS200	Island	28.36	4.71	35.39	5.29	-7.03	9.42E-10	5.26E-08
cg17853216	ZMIZ1	10	Body	Island	39.32	6.15	46.35	6.50	-7.03	3.04E-07	1.45E-05
cg02610813		7	Intergenic	N_Shelf	65.73	7.04	72.77	5.81	-7.03	4.89E-06	1.96E-04
cg00883212	RAPGEF4;RAPGEF4	2	Body;Body	Open sea	67.40	6.90	74.43	5.46	-7.03	3.07E-06	1.27E-04
cg03779973	DCLK2;DCLK2	4	Body;Body	S_Shore	64.82	7.03	71.85	7.07	-7.03	1.98E-05	6.92E-04
cg02043105	SH2B1;SH2B1;SH2B1;SH2B1;SH2B1	16	Body;Body;Body;Body;1stExon	S_Shelf	63.31	4.89	70.33	4.24	-7.03	5.50E-10	3.10E-08
cg21676617	ADAMTS10	19	Body	Island	85.29	13.48	92.31	8.22	-7.02	5.95E-04	1.16E-02
cg00509968		4	Intergenic	Open sea	48.15	7.97	55.17	10.64	-7.02	3.77E-03	4.38E-02
cg12560128	OAS2;OAS2;OAS2	12	TSS1500;TSS1500;TSS1500	Open sea	13.22	5.91	20.24	8.47	-7.02	2.32E-05	7.97E-04
cg17641252	PLEK2	14	Body	Island	41.30	6.71	48.32	5.13	-7.02	3.51E-06	1.44E-04
cg07914250	WDR66	12	Body	Open sea	50.13	7.55	57.15	7.18	-7.02	1.48E-06	6.48E-05
cg04439557	NADK	1	Body	N_Shelf	33.06	7.59	40.07	7.13	-7.01	5.86E-05	1.78E-03
cg10970349	MIB2;MIB2;MIB2;MIB2;MIB2;MIB2	1	Body;Body;Body;5'UTR;Body;Body	N_Shelf	42.96	6.81	49.97	9.25	-7.01	6.99E-04	1.31E-02
cg16525246		11	Intergenic	N_Shore	36.41	7.01	43.42	8.63	-7.01	5.90E-05	1.79E-03
cg25484790	MCAM	11	Body	N_Shore	76.09	6.40	83.10	5.80	-7.01	1.47E-06	6.45E-05
cg23038594	LOC284688	1	TSS1500	Open sea	41.22	6.29	48.23	7.99	-7.01	3.59E-05	1.17E-03
cg06766427	RICH2	17	Body	Island	14.19	7.18	21.20	11.52	-7.01	2.24E-03	3.06E-02
cg03147592		12	Intergenic	S_Shore	70.55	7.64	77.55	6.72	-7.00	6.66E-06	2.60E-04
cg04294971	RFNG	17	Body	N_Shore	29.77	5.35	36.77	9.14	-7.00	6.90E-05	2.05E-03
cg02132051	RGS6	14	Body	Open sea	54.56	7.22	61.56	6.50	-7.00	4.96E-05	1.54E-03
cg25193073	MAD1L1;MAD1L1;MAD1L1	7	Body;Body;Body	N_Shore	73.11	10.45	80.11	5.87	-7.00	2.94E-03	3.69E-02
cg14560110	CCDC144NL	17	TSS200	Island	45.59	11.69	52.59	12.28	-7.00	4.24E-03	4.75E-02
cg25279922		1	Intergenic	Open sea	66.53	9.44	73.53	9.98	-7.00	1.93E-03	2.76E-02
cg18875551		1	Intergenic	N_Shore	77.55	9.04	84.54	6.76	-7.00	1.88E-03	2.71E-02
cg00193613	C1QTNF1;C1QTNF1;C1QTNF1	17	Body;Body;Body	N_Shore	29.24	6.62	36.23	10.21	-7.00	6.50E-04	1.24E-02
cg13683194	C9orf125	9	3'UTR	Open sea	63.57	6.39	70.57	5.75	-6.99	5.28E-07	2.46E-05
cg03163783	SATB2	2	TSS1500	Island	29.11	6.46	36.10	8.49	-6.99	7.17E-05	2.12E-03
cg07835424		2	Intergenic	Island	11.40	4.50	18.38	8.58	-6.99	3.45E-06	1.42E-04
cg23004527	NFIC;NFIC	19	Body;Body	S_Shelf	50.34	5.60	57.33	6.66	-6.99	3.30E-06	1.36E-04
cg03171003		2	Intergenic	Island	40.20	5.73	47.19	6.18	-6.98	4.06E-07	1.92E-05
cg24397007	FOSL2	2	Body	S_Shelf	46.50	7.29	53.49	6.94	-6.98	1.07E-07	5.34E-06
cg19704074	DOCK7;ANGPTL3	1	Body;TSS1500	Open sea	41.31	4.86	48.29	7.34	-6.98	3.80E-06	1.55E-04
cg13315838		21	Intergenic	N_Shelf	61.91	8.47	68.89	9.96	-6.98	3.66E-03	4.29E-02
cg23631538	DENND2D	1	TSS1500	N_Shelf	26.36	5.96	33.34	7.56	-6.98	1.07E-06	4.80E-05
cg18269457	DPYD;DPYD	1	Body;Body	N_Shelf	66.76	5.85	73.74	5.33	-6.98	2.79E-07	1.34E-05
cg22753548	TNNI3	19	Body	Island	22.01	7.11	28.98	7.55	-6.97	2.22E-05	7.68E-04
cg17870959		18	Intergenic	Island	63.15	7.61	70.13	8.21	-6.97	1.88E-04	4.70E-03
cg00159289	MOGAT3	7	1stExon	N_Shore	61.01	7.71	67.98	7.30	-6.97	3.42E-07	1.63E-05
cg02366931	CMYA5	5	Body	Open sea	37.69	7.18	44.66	10.24	-6.97	1.28E-03	2.06E-02
cg07631407	TTBK1	6	Body	Island	22.40	7.25	29.37	9.89	-6.97	1.19E-03	1.95E-02
cg09663343		11	Intergenic	Open sea	60.33	5.20	67.31	5.51	-6.97	6.74E-08	3.41E-06
cg06295987		3	Intergenic	N_Shore	75.95	10.50	82.92	8.64	-6.97	1.96E-03	2.79E-02
cg13726644	DPF3	14	Body	Open sea	79.21	6.99	86.18	4.41	-6.97	4.44E-07	2.08E-05
cg01277438	NFATC1;NFATC1;NFATC1	18	Body;5'UTR;Body	Island	43.14	6.65	50.11	6.84	-6.96	2.60E-05	8.83E-04
cg25111130		20	Intergenic	S_Shelf	72.85	8.30	79.81	5.32	-6.96	1.92E-05	6.73E-04
cg21739934		11	Intergenic	Island	38.42	7.51	45.38	7.05	-6.96	8.79E-05	2.51E-03
cg04951476	FAM50B	6	TSS1500	N_Shore	53.50	5.39	60.46	4.83	-6.96	3.05E-08	1.58E-06
cg11178148		11	Intergenic	S_Shore	32.21	6.83	39.17	6.80	-6.96	5.91E-06	2.33E-04
cg26954519	MB;MB;MB	22	TSS1500;TSS1500;5'UTR	Open sea	51.41	6.46	58.37	6.96	-6.96	9.29E-05	2.63E-03
cg08234878	PAPLN	14	Body	Open sea	27.16	7.14	34.12	9.74	-6.95	7.33E-04	1.36E-02
cg03161190	C10orf26;C10orf26	10	TSS200;Body	Open sea	31.42	5.52	38.38	5.46	-6.95	1.15E-06	5.10E-05

cg14523948	AGT	1	Body	Open sea	47.48	5.22	54.44	5.20	-6.95	1.83E-07	8.92E-06
cg16892553	PRR15L;PRR15L	17	1stExon;5'UTR	Open sea	57.75	4.61	64.70	4.88	-6.95	2.33E-09	1.28E-07
cg06197492	H19	11	Body	N_Shore	69.33	6.89	76.28	7.41	-6.95	6.66E-06	2.60E-04
cg22543026		2	Intergenic	Open sea	17.59	5.08	24.54	7.95	-6.95	2.01E-06	8.62E-05
cg09578155	LRP5	11	Body	Open sea	25.94	4.78	32.89	6.95	-6.95	1.15E-06	5.13E-05
cg10483871		1	Intergenic	N_Shore	41.53	7.94	48.48	6.90	-6.95	2.60E-04	6.09E-03
cg07609780		16	Intergenic	Open sea	34.93	5.34	41.88	5.95	-6.94	1.08E-06	4.83E-05
cg02828785	GPR19	12	5'UTR	N_Shelf	59.56	5.33	66.51	4.53	-6.94	7.03E-09	3.77E-07
cg16745930	HPSE2;HPSE2;HPSE2;HPSE2	10	3'UTR;Body;Body;Body	Open sea	52.70	9.76	59.65	9.25	-6.94	4.06E-07	1.92E-05
cg10185917		19	Intergenic	N_Shelf	68.26	5.05	75.21	4.42	-6.94	3.07E-10	1.75E-08
cg12982374	FGFR4;FGFR4;FGFR4	5	Body;Body;Body	S_Shelf	43.52	4.81	50.46	7.63	-6.94	2.29E-06	9.70E-05
cg02492567	RAPGEF4;RAPGEF4	2	Body;Body	Open sea	79.13	7.92	86.07	7.40	-6.94	5.34E-06	2.12E-04
cg16177732		18	Intergenic	Island	28.28	5.89	35.22	9.54	-6.94	1.16E-04	3.15E-03
cg19255333	PEMT;PEMT;PEMT	17	Body;Body;Body	Open sea	79.23	7.47	86.17	5.58	-6.94	3.76E-06	1.54E-04
cg21699894	TOX2;TOX2;TOX2	20	5'UTR;Body;5'UTR	S_Shore	33.86	5.56	40.80	9.54	-6.94	1.17E-04	3.19E-03
cg06267075	TMCO7	16	Body	Open sea	57.11	5.64	64.05	5.78	-6.94	1.19E-06	5.31E-05
cg00228796	ECE1	1	Body	N_Shore	58.88	5.57	65.82	7.10	-6.94	4.51E-06	1.82E-04
cg20240243	MEF2A;MEF2A;MEF2A	15	5'UTR;5'UTR;5'UTR	Open sea	71.86	5.70	78.80	5.00	-6.93	2.86E-07	1.37E-05
cg25386151	ATF3	1	5'UTR	N_Shore	17.18	4.07	24.12	4.83	-6.93	1.62E-10	9.34E-09
cg05691004	SPATS2L;SPATS2L;SPATS2L;SPATS2L	2	TSS200;TSS1500;TSS1500;TSS1500	N_Shore	37.71	5.91	44.65	8.01	-6.93	8.67E-06	3.31E-04
cg07262395	MXI1	10	TSS1500	N_Shore	48.62	8.41	55.55	8.12	-6.93	3.53E-04	7.73E-03
cg27070372		19	Intergenic	S_Shore	54.06	6.61	60.99	8.39	-6.93	2.00E-04	4.95E-03
cg07786995	NOS1AP;NOS1AP	1	Body;Body	Open sea	47.82	5.94	54.75	7.31	-6.93	6.04E-06	2.38E-04
cg12273319		19	Intergenic	Island	51.34	7.70	58.26	9.68	-6.93	1.39E-04	3.66E-03
cg14912034	LYZL2	10	TSS1500	Open sea	62.89	5.90	69.81	5.76	-6.93	6.84E-07	3.14E-05
cg26348684		17	Intergenic	S_Shelf	54.71	3.37	61.63	3.42	-6.92	4.05E-14	2.56E-12
cg19484299	MSL3L2	2	Body	N_Shore	76.93	5.16	83.86	4.36	-6.92	1.91E-08	1.00E-06
cg00510507	ANK3;ANK3;ANK3	10	Body;5'UTR;1stExon	Open sea	63.70	7.23	70.62	6.73	-6.92	1.27E-05	4.65E-04
cg03149320		8	Intergenic	S_Shelf	61.36	6.05	68.28	5.98	-6.92	6.05E-07	2.79E-05
cg11003530	LVRN	5	Body	S_Shore	36.36	6.39	43.28	6.48	-6.92	1.85E-05	6.52E-04
cg04824378	TRPM7	15	3'UTR	Open sea	59.20	5.62	66.12	5.78	-6.92	8.39E-07	3.81E-05
cg20754465		4	Intergenic	Open sea	45.27	5.83	52.19	8.82	-6.92	2.98E-05	9.91E-04
cg09234582	HLA-DPB1	6	Body	N_Shore	28.27	5.73	35.19	5.54	-6.92	1.71E-06	7.40E-05
cg15597696	SGEF	3	Body	Open sea	75.88	6.66	82.80	5.09	-6.92	3.12E-06	1.29E-04
cg20078879		4	Intergenic	S_Shelf	66.86	4.53	73.78	3.97	-6.92	2.14E-10	1.23E-08
cg27196158	LOC100133545	11	TSS200	N_Shelf	66.24	7.19	73.15	4.86	-6.92	4.40E-06	1.78E-04
cg23206463		5	Intergenic	N_Shore	68.59	8.68	75.50	7.33	-6.91	2.76E-04	6.37E-03
cg06012872		10	Intergenic	Open sea	30.10	8.59	37.01	10.42	-6.91	2.19E-03	3.02E-02
cg25548942	OSBPL3;OSBPL3;OSBPL3;OSBPL3	7	5'UTR;5'UTR;5'UTR;5'UTR	Open sea	56.78	7.48	63.69	6.53	-6.91	2.48E-06	1.04E-04
cg06952534	CFHR5	1	TSS1500	Open sea	58.93	7.79	65.84	8.98	-6.91	1.27E-03	2.04E-02
cg20312390	ATP2B4;ATP2B4	1	5'UTR;5'UTR	Open sea	26.24	4.98	33.16	5.81	-6.91	1.32E-07	6.53E-06
cg17631424	TMPRSS11E	4	TSS1500	Open sea	51.04	6.19	57.95	6.86	-6.91	2.31E-06	9.80E-05
cg09782034	RORA	15	TSS1500	S_Shore	39.03	6.67	45.94	8.92	-6.91	1.52E-04	3.95E-03
cg15547143		10	Intergenic	N_Shelf	60.01	6.62	66.92	5.65	-6.91	5.02E-06	2.01E-04
cg14380217		14	Intergenic	Open sea	46.81	7.07	53.71	7.12	-6.90	1.29E-06	5.68E-05
cg01452189	LCMT1;LCMT1	16	3'UTR;3'UTR	Open sea	83.04	7.44	89.95	3.26	-6.90	7.85E-09	4.20E-07
cg14995181	TRIM39;TRIM39	6	Body;Body	Open sea	66.55	8.01	73.45	9.70	-6.90	1.66E-03	2.47E-02
cg27648858	PIK3R2	19	Body	Island	67.68	9.78	74.59	10.08	-6.90	3.87E-03	4.46E-02
cg03758047	C6orf145	6	Body	Open sea	50.43	4.56	57.32	5.05	-6.90	2.09E-08	1.09E-06
cg18930910	RNF39;RNF39	6	Body;Body	Island	18.58	7.64	25.48	10.43	-6.90	1.69E-03	2.51E-02
cg22455450	ZNF808	19	5'UTR	N_Shore	38.57	6.02	45.46	7.02	-6.90	7.68E-07	3.50E-05
cg25450819	WDR70	5	Body	Open sea	58.69	7.27	65.58	6.47	-6.90	1.57E-05	5.64E-04
cg16085649	AKAP13;AKAP13	15	Body;Body	Open sea	44.39	7.25	51.29	7.92	-6.89	4.94E-04	1.00E-02

cg07822928	HCN2	19	Body	Island	30.70	6.81	37.59	6.34	-6.89	2.45E-06	1.03E-04
cg11864574	SPAG6;SPAG6	10	Body;Body	S_Shore	51.53	5.57	58.42	6.18	-6.89	3.39E-07	1.61E-05
cg03151810		8	Intergenic	Island	69.90	6.88	76.79	6.63	-6.89	2.24E-05	7.72E-04
cg14256968	C11orf95	11	3'UTR	Island	32.61	6.52	39.50	10.16	-6.89	4.73E-04	9.70E-03
cg04193923	CSF3;CSF3;CSF3	17	TSS1500;TSS1500;TSS1500	Open sea	43.58	5.02	50.47	5.06	-6.89	6.64E-09	3.57E-07
cg04362711	ZFPM1	16	Body	Island	52.73	5.10	59.62	6.11	-6.89	1.37E-06	6.02E-05
cg23295683	SEL1L	14	Body	N_Shore	64.59	6.46	71.48	6.48	-6.89	2.04E-06	8.74E-05
cg14268226	HAUS6	9	Body	Open sea	65.91	5.08	72.80	4.43	-6.89	1.15E-09	6.40E-08
cg08055994		2	Intergenic	Open sea	59.40	5.08	66.29	5.44	-6.89	3.75E-07	1.78E-05
cg26896748	KCNQ1;KCNQ1	11	Body;Body	Open sea	59.05	8.18	65.93	11.41	-6.89	2.13E-03	2.95E-02
cg02976588		1	Intergenic	Open sea	48.52	7.87	55.41	7.55	-6.89	1.81E-05	6.39E-04
cg00099766	RRAGD	6	Body	N_Shore	36.48	6.96	43.37	8.24	-6.89	4.81E-04	9.82E-03
cg00431813	C7orf50;C7orf50;C7orf50	7	Body;Body;Body	N_Shore	42.35	5.63	49.24	8.38	-6.88	6.98E-05	2.07E-03
cg22250605	LEPREL1;LEPREL1	3	5'UTR;Body	Open sea	54.35	7.36	61.24	10.50	-6.88	8.15E-04	1.47E-02
cg08116408	TRIM40	6	Body	Open sea	22.88	4.24	29.77	6.27	-6.88	1.95E-08	1.02E-06
cg27092752	USP2;USP2	11	3'UTR;3'UTR	Island	14.66	5.14	21.55	7.11	-6.88	7.55E-06	2.92E-04
cg05313129	C8orf71	8	Body	Island	53.06	7.75	59.94	8.92	-6.88	1.75E-04	4.44E-03
cg11734112	PLOD2;PLOD2	3	Body;Body	Open sea	59.04	6.15	65.92	6.68	-6.88	3.69E-06	1.51E-04
cg10448227	TMPRSS8	16	TSS200	Island	22.18	4.84	29.06	5.78	-6.88	4.55E-08	2.33E-06
cg03641529	SREBF1;SREBF1	17	Body;Body	Island	43.71	7.05	50.59	9.12	-6.88	6.88E-04	1.29E-02
cg23213327	RSAD2	2	TSS1500	Open sea	60.05	6.53	66.93	6.66	-6.88	2.62E-05	8.86E-04
cg05491166	NAV2;NAV2;NAV2	11	Body;Body;Body	Open sea	68.05	6.38	74.92	5.19	-6.88	3.60E-07	1.71E-05
cg13419792	C19orf66	19	Body	S_Shore	55.87	6.83	62.75	6.90	-6.88	1.50E-04	3.91E-03
cg27174822	PRSS22	16	3'UTR	Island	44.25	8.31	51.13	9.50	-6.87	1.93E-03	2.76E-02
cg00963786	WWP2;WWP2	16	Body;Body	Open sea	45.02	6.70	51.90	9.05	-6.87	6.30E-04	1.21E-02
cg08774009		8	Intergenic	Island	39.83	5.97	46.70	5.94	-6.87	1.38E-06	6.05E-05
cg10469980	KIAA0182;KIAA0182	16	5'UTR;Body	S_Shore	47.47	5.32	54.34	6.10	-6.87	2.04E-06	8.74E-05
cg05618934		4	Intergenic	Island	28.51	5.68	35.37	6.85	-6.87	9.64E-06	3.65E-04
cg11562901	WIBG;WIBG	12	Body;Body	N_Shelf	60.11	6.85	66.97	6.41	-6.86	3.51E-06	1.44E-04
cg18285813	MET;MET	7	Body;Body	Open sea	66.32	6.51	73.18	7.31	-6.86	3.50E-05	1.14E-03
cg07685601	MAD1L1;MAD1L1;MAD1L1	7	Body;Body;Body	S_Shore	77.47	10.50	84.33	6.16	-6.86	4.54E-03	4.97E-02
cg12815918	PDX1	13	Body	Island	14.18	6.75	21.04	10.27	-6.86	1.20E-03	1.96E-02
cg18326719	MDFIC;MDFIC	7	Body;Body	Open sea	71.07	6.47	77.92	4.99	-6.85	2.96E-06	1.23E-04
cg27436940	C1QL1	17	TSS1500	N_Shore	44.02	5.95	50.87	7.33	-6.85	1.30E-05	4.76E-04
cg20320200	ESRRG;ESRRG;ESRRG	1	5'UTR;5'UTR;5'UTR	Open sea	57.74	6.23	64.59	9.40	-6.85	1.07E-04	2.96E-03
cg03379797	NHSL1	6	1stExon	Open sea	55.05	8.92	61.90	7.85	-6.84	1.65E-05	5.88E-04
cg11336382		1	Intergenic	N_Shore	44.67	7.86	51.51	8.60	-6.84	2.74E-04	6.34E-03
cg15072619	SLC39A12;SLC39A12	10	Body;Body	Open sea	39.13	6.46	45.97	7.59	-6.84	1.02E-04	2.85E-03
cg04464062	ZFPM1	16	Body	Island	40.15	4.59	46.99	6.72	-6.84	6.62E-07	3.05E-05
cg05640320	PC;PC;PC	11	5'UTR;5'UTR;5'UTR	Open sea	51.19	6.23	58.04	8.66	-6.84	4.34E-04	9.08E-03
cg08076018	ANGPTL2;RALGPS1	9	TSS200;Body	Open sea	20.32	6.38	27.15	9.00	-6.84	2.14E-04	5.22E-03
cg15510249	MCF2L2;B3GNT5	3	Body;TSS200	N_Shore	10.25	5.07	17.08	7.09	-6.84	1.83E-08	9.62E-07
cg01617285		9	Intergenic	Island	29.83	9.82	36.66	9.59	-6.84	6.95E-04	1.30E-02
cg26863534		12	Intergenic	Open sea	43.55	7.10	50.38	6.84	-6.83	6.11E-05	1.85E-03
cg05023691	RGS13;RGS13	1	TSS200;TSS200	Open sea	71.96	4.94	78.79	4.99	-6.83	1.08E-10	6.24E-09
cg00375025	KCNAB1;KCNAB1;KCNAB1	3	1stExon;5'UTR;Body	Open sea	70.68	5.37	77.51	4.85	-6.83	3.92E-08	2.02E-06
cg22610645	GEFT;GEFT	12	TSS1500;TSS200	Island	38.62	5.39	45.44	7.37	-6.83	5.73E-06	2.27E-04
cg01843034		6	Intergenic	Island	15.90	7.50	22.73	9.27	-6.83	6.26E-04	1.20E-02
cg03364486		8	Intergenic	Open sea	47.31	6.72	54.14	8.34	-6.83	3.62E-05	1.17E-03
cg12968041	ZNRF3	22	5'UTR	S_Shore	55.30	6.29	62.13	9.01	-6.83	5.06E-05	1.57E-03
cg18448977		2	Intergenic	Open sea	47.98	5.87	54.81	6.47	-6.83	1.86E-06	8.01E-05
cg12727138		2	Intergenic	S_Shore	75.36	7.10	82.19	4.78	-6.83	5.13E-06	2.05E-04
cg19869037	DSE;DSE	6	Body;Body	Open sea	55.28	7.13	62.11	4.88	-6.83	4.79E-06	1.93E-04

cg18810542	AHR	7	Body	Open sea	73.32	6.06	80.15	5.05	-6.83	1.94E-06	8.32E-05
cg12868970	TP63;TP63;TP63;TP63;TP63;TP63	3	Body;Body;Body;Body;Body;Body	Open sea	63.48	6.55	70.31	5.34	-6.83	4.00E-07	1.89E-05
cg02336762	KSR2	12	Body	Open sea	26.91	5.89	33.74	6.13	-6.82	2.20E-06	9.38E-05
cg11126762	OSMR;OSMR	5	5'UTR;5'UTR	S_Shore	29.71	5.34	36.53	6.22	-6.82	1.04E-06	4.65E-05
cg01972843	CECR1;CECR1	22	TSS200;Body	Open sea	46.74	10.44	53.57	10.56	-6.82	1.68E-03	2.50E-02
cg11517269	ZSCAN12L1	6	TSS200	Open sea	38.81	5.23	45.63	6.66	-6.82	1.41E-06	6.20E-05
cg01379412	DIXDC1	11	Body	S_Shelf	47.39	6.68	54.20	7.26	-6.82	9.91E-05	2.78E-03
cg12542255	FOSB;FOSB	19	Body;Body	Island	16.03	6.32	22.85	10.20	-6.81	5.87E-04	1.15E-02
cg20707786	BAIAP2;BAIAP2;BAIAP2;BAIAP2	17	Body;Body;Body;Body	Open sea	68.50	7.17	75.31	8.79	-6.81	5.71E-04	1.12E-02
cg15706621	SPTBN1;SPTBN1	2	Body;Body	Open sea	48.63	5.95	55.44	5.65	-6.81	1.56E-07	7.67E-06
cg12106976		6	Intergenic	Open sea	55.54	6.51	62.35	6.00	-6.81	9.34E-06	3.54E-04
cg04733744	C2orf54;C2orf54	2	1stExon;5'UTR	Open sea	73.78	12.35	80.58	10.64	-6.80	3.59E-03	4.24E-02
cg05895376		15	Intergenic	N_Shelf	32.83	7.27	39.63	7.07	-6.80	1.88E-06	8.08E-05
cg03092825		2	Intergenic	Open sea	52.56	6.71	59.36	10.25	-6.80	3.23E-04	7.21E-03
cg02528008	MCC;MCC	5	Body;Body	Open sea	70.62	5.07	77.43	3.78	-6.80	4.42E-09	2.40E-07
cg15292765	PARD3	10	Body	Open sea	18.68	3.85	25.48	5.09	-6.80	1.76E-10	1.01E-08
cg20480642	PXDNL	8	TSS200	Open sea	62.44	5.88	69.24	6.39	-6.80	2.50E-07	1.21E-05
cg04431508		3	Intergenic	Open sea	47.27	4.58	54.06	3.65	-6.80	9.77E-10	5.45E-08
cg22365119		12	Intergenic	Open sea	52.89	7.35	59.68	7.30	-6.79	8.00E-05	2.32E-03
cg13960660	FAM125B;FAM125B	9	Body;Body	N_Shelf	70.98	6.24	77.77	4.04	-6.79	1.72E-06	7.46E-05
cg12584100		6	Intergenic	Open sea	43.26	12.84	50.05	9.23	-6.79	1.77E-03	2.60E-02
cg11512608	PLEKHB1;PLEKHB1;PLEKHB1;PLEKHB1	11	5'UTR;1stExon;5'UTR;TSS1500;TSS1500;1s	Open sea	51.05	7.33	57.84	9.03	-6.79	3.25E-04	7.24E-03
cg15089217	CCDC14	3	3'UTR	Open sea	76.03	5.79	82.82	5.29	-6.79	2.92E-08	1.51E-06
cg10886747	ZCCHC24	10	Body	Open sea	69.11	6.22	75.89	6.27	-6.79	1.43E-05	5.18E-04
cg24048283	TXNRD2	22	Body	N_Shelf	46.17	7.86	52.95	9.37	-6.79	3.00E-03	3.74E-02
cg11798758	NPHP4	1	Body	Open sea	70.42	7.95	77.21	9.28	-6.79	2.62E-03	3.41E-02
cg24029082	TNXB	6	Body	S_Shore	80.72	6.50	87.51	4.63	-6.78	5.30E-09	2.86E-07
cg03775991		6	Intergenic	Island	44.02	7.63	50.80	7.82	-6.78	1.72E-04	4.38E-03
cg25045113	MFSD6L	17	1stExon	N_Shore	50.68	5.82	57.46	9.88	-6.78	1.75E-04	4.44E-03
cg07307142	HSD17B2	16	Body	Open sea	35.43	7.41	42.21	6.76	-6.78	1.93E-05	6.77E-04
cg24432768	CD1D	1	5'UTR	N_Shore	55.59	6.75	62.37	7.10	-6.78	8.23E-05	2.38E-03
cg08231710	MMP23A;MMP23B	1	TSS1500;TSS1500	Island	35.17	6.10	41.94	6.83	-6.78	2.35E-07	1.14E-05
cg17928920	AGRN	1	Body	Island	20.19	4.33	26.96	6.14	-6.78	1.48E-07	7.28E-06
cg03073218	COLEC10	8	Body	Open sea	28.74	5.56	35.52	5.63	-6.77	7.04E-07	3.23E-05
cg26511108	COMMD3;BMI1	10	3'UTR;TSS1500	S_Shelf	29.93	6.53	36.71	9.06	-6.77	6.83E-04	1.29E-02
cg26583598	ZSWIM6	5	Body	Open sea	60.50	7.53	67.28	10.75	-6.77	3.28E-03	3.98E-02
cg00431894		4	Intergenic	Open sea	67.99	10.72	74.76	10.48	-6.77	1.16E-03	1.91E-02
cg12948524		3	Intergenic	Open sea	52.38	6.01	59.14	9.40	-6.77	9.12E-05	2.59E-03
cg15800776	DOCK6	19	Body	Island	64.49	7.21	71.25	8.23	-6.77	3.10E-04	6.98E-03
cg09119854	AXIN2	17	Body	Open sea	28.93	5.77	35.69	8.90	-6.76	7.93E-05	2.30E-03
cg23500537		5	Intergenic	Open sea	47.54	7.53	54.30	5.22	-6.76	1.48E-05	5.34E-04
cg03035746	LBX2	2	Body	S_Shore	71.60	8.30	78.36	8.39	-6.76	2.09E-04	5.12E-03
cg20227044		11	Intergenic	S_Shelf	62.69	9.31	69.45	7.38	-6.76	3.11E-04	6.99E-03
cg01622304	PDX1	13	Body	N_Shore	24.73	7.63	31.49	10.00	-6.76	2.39E-03	3.20E-02
cg08326019	LPPR2;LPPR2	19	Body;Body	Island	45.16	5.85	51.91	6.60	-6.76	6.71E-06	2.62E-04
cg21727359	FAM46A	6	3'UTR	N_Shelf	33.79	6.32	40.54	7.47	-6.76	1.99E-05	6.96E-04
cg26214026	ATXN2	12	Body	Open sea	65.56	6.06	72.31	5.61	-6.76	6.08E-06	2.39E-04
cg04036049	MBD6	12	3'UTR	Open sea	56.86	5.59	63.62	5.68	-6.76	1.46E-06	6.41E-05
cg25333460	DGKD;DGKD	2	Body;Body	Island	57.67	7.73	64.43	7.39	-6.75	7.18E-04	1.34E-02
cg18187198		6	Intergenic	Open sea	23.51	5.91	30.26	7.17	-6.75	1.85E-05	6.52E-04
cg13231302	CDC42BPB	14	Body	Open sea	44.87	5.75	51.62	7.30	-6.75	5.69E-05	1.74E-03
cg07405121	SLC9A3R2;SLC9A3R2	16	Body;Body	N_Shelf	42.72	6.31	49.47	5.91	-6.75	1.41E-05	5.13E-04
cg07940714	EID2B	19	TSS1500	S_Shore	60.39	6.65	67.14	5.83	-6.75	6.30E-06	2.47E-04

cg07638450	CUX1;CUX1;CUX1	7	Body;Body;Body	Open sea	71.55	8.80	78.31	7.86	-6.75	6.46E-04	1.23E-02
cg16566899	LRP1	12	Body	Island	75.65	6.55	82.40	5.51	-6.75	9.16E-06	3.48E-04
cg13516362	KANK2	19	TSS1500	S_Shore	58.22	7.55	64.97	7.64	-6.75	2.21E-04	5.34E-03
cg15915602		2	Intergenic	Island	56.33	7.05	63.07	8.40	-6.74	1.52E-04	3.96E-03
cg20116828	NFIX	19	3'UTR	Island	46.87	7.77	53.61	8.33	-6.74	3.01E-04	6.82E-03
cg13399638	SUGT1P1;AQP7	9	Body;Body	Open sea	64.20	7.94	70.94	7.95	-6.74	2.13E-03	2.96E-02
cg20735041	MUSTN1	3	TSS200	Open sea	30.90	5.81	37.64	6.73	-6.74	5.38E-06	2.14E-04
cg09556515		16	Intergenic	Open sea	70.64	8.69	77.38	4.41	-6.74	5.28E-05	1.63E-03
cg27125439		10	Intergenic	Open sea	70.10	5.91	76.84	7.97	-6.74	1.68E-05	5.99E-04
cg22871559	RASSF5;RASSF5;RASSF5	1	Body;TSS1500;Body	N_Shore	14.59	4.07	21.33	5.18	-6.74	9.06E-11	5.24E-09
cg14361895	SPATS2L;SPATS2L;SPATS2L;SPATS2L	2	5'UTR;5'UTR;5'UTR;5'UTR	Open sea	54.47	6.06	61.20	5.67	-6.73	2.07E-06	8.85E-05
cg00478479	PRX;PRX	19	Body;Body	Island	48.96	6.98	55.69	8.14	-6.73	3.07E-05	1.02E-03
cg04147049	KIAA1522	1	Body	Island	27.21	7.18	33.94	9.08	-6.73	7.48E-05	2.19E-03
cg09273012	SRBD1	2	Body	Open sea	72.63	7.62	79.36	6.36	-6.73	2.56E-05	8.68E-04
cg06792134	WDR25;WDR25	14	Body;Body	Open sea	60.02	7.55	66.75	7.69	-6.73	3.96E-04	8.46E-03
cg01721555	PARP14	3	Body	S_Shore	76.89	5.58	83.62	4.56	-6.73	2.47E-08	1.29E-06
cg17554647	IQSEC1;IQSEC1	3	Body;Body	Open sea	56.49	6.08	63.22	6.59	-6.73	1.59E-05	5.71E-04
cg24927174	SHC1;SHC1;SHC1;SHC1	1	Body;1stExon;Body;1stExon	N_Shelf	57.12	4.51	63.85	4.05	-6.73	5.70E-10	3.21E-08
cg05385260	SLC22A18AS;SLC22A18;SLC22A18	11	5'UTR;5'UTR;TSS1500	N_Shore	70.23	7.69	76.96	6.03	-6.73	1.14E-04	3.12E-03
cg03292206	BUB1	2	Body	Open sea	43.06	5.86	49.78	7.11	-6.73	3.86E-05	1.24E-03
cg26971042	TLE3;TLE3;TLE3	15	Body;Body;Body	N_Shore	36.33	6.72	43.06	6.24	-6.73	4.23E-07	1.99E-05
cg01973483	PITPNM2	12	5'UTR	Open sea	44.22	5.37	50.94	7.17	-6.73	1.57E-05	5.64E-04
cg14492337	P4HB	17	Body	Open sea	48.62	5.81	55.34	6.49	-6.72	9.06E-06	3.44E-04
cg15604182		2	Intergenic	S_Shelf	65.52	8.31	72.24	6.32	-6.72	1.08E-04	2.98E-03
cg24892628	ADAMTSL5	19	Body	N_Shore	37.55	6.32	44.27	7.05	-6.72	1.88E-05	6.62E-04
cg18557837		3	Intergenic	N_Shelf	12.13	12.06	18.85	14.68	-6.72	1.64E-03	2.45E-02
cg02891082	LRP5	11	Body	S_Shelf	60.65	6.37	67.37	5.07	-6.72	1.65E-06	7.15E-05
cg16398581	FGGY;FGGY	1	Body;Body	Open sea	62.12	8.25	68.84	5.82	-6.72	4.41E-04	9.19E-03
cg23155965		10	Intergenic	N_Shore	78.06	10.81	84.78	6.81	-6.72	3.38E-04	7.47E-03
cg00023919	RHOD	11	3'UTR	Island	27.01	6.65	33.72	9.66	-6.72	9.86E-04	1.69E-02
cg02999781	PRDM16;PRDM16	1	Body;Body	Island	49.48	5.94	56.20	6.69	-6.72	1.85E-05	6.51E-04
cg04147969		15	Intergenic	Open sea	49.48	6.70	56.20	5.38	-6.72	1.56E-06	6.82E-05
cg09938479		1	Intergenic	Island	40.36	5.59	47.08	7.38	-6.71	1.16E-05	4.29E-04
cg11749792	ANXA13;ANXA13	8	Body;Body	Open sea	64.58	7.51	71.30	7.78	-6.71	6.06E-04	1.17E-02
cg19930203	COMT;COMT;COMT;COMT	22	3'UTR;3'UTR;3'UTR;3'UTR	Open sea	44.44	8.10	51.16	9.30	-6.71	2.21E-03	3.03E-02
cg11364273	MACROD1	11	Body	Island	10.65	4.10	17.36	7.50	-6.71	4.12E-07	1.94E-05
cg25299895	PDX1	13	Body	N_Shore	14.51	6.28	21.22	10.11	-6.71	4.47E-04	9.30E-03
cg11165765	LY6G6D	6	Body	Open sea	55.83	5.01	62.54	6.32	-6.71	4.20E-07	1.98E-05
cg15532942	NFATC1;NFATC1;NFATC1;NFATC1;NFA	18	Body;Body;Body;Body;Body	N_Shore	79.10	9.42	85.81	6.71	-6.71	8.15E-05	2.36E-03
cg19758142	NCEH1;NCEH1;NCEH1;NCEH1	3	5'UTR;Body;Body;5'UTR	Open sea	52.54	6.36	59.25	7.55	-6.71	1.97E-04	4.87E-03
cg21671248	LOC100130776;AGAP2;AGAP2	12	TSS1500;3'UTR;3'UTR	N_Shore	50.74	6.28	57.45	6.60	-6.71	2.22E-05	7.68E-04
cg05194426	CYP2E1	10	Body	S_Shore	26.44	8.81	33.14	10.05	-6.70	5.27E-04	1.05E-02
cg06171329		8	Intergenic	Open sea	55.56	5.93	62.26	7.41	-6.70	5.37E-05	1.65E-03
cg01267532		18	Intergenic	Island	54.73	7.88	61.44	8.47	-6.70	3.83E-04	8.24E-03
cg24595152	ABHD5	3	Body	S_Shore	66.47	5.05	73.16	4.71	-6.70	5.42E-09	2.93E-07
cg17939040	ACACA;ACACA;ACACA;ACACA;ACACA	17	Body;Body;Body;Body;Body	Open sea	79.45	5.08	86.15	2.31	-6.70	1.15E-11	6.81E-10
cg03496533	PRR15L	17	TSS200	Open sea	44.47	4.95	51.16	5.69	-6.70	1.90E-07	9.25E-06
cg13960999		13	Intergenic	Open sea	51.08	7.55	57.78	5.16	-6.69	1.37E-05	5.00E-04
cg05375686		12	Intergenic	Island	58.39	7.40	65.09	6.63	-6.69	4.22E-05	1.34E-03
cg01254505	BST2	19	TSS200	Open sea	16.18	7.97	22.88	11.20	-6.69	3.57E-03	4.22E-02
cg08064292		15	Intergenic	Island	37.77	9.97	44.46	9.36	-6.69	7.74E-04	1.41E-02
cg03735511	CLU;CLU;CLU	8	Body;Body;Body	Open sea	73.14	5.46	79.83	4.43	-6.69	1.06E-07	5.28E-06
cg20963865	TBC1D14;TBC1D14;TBC1D14	4	Body;Body;Body	Open sea	54.85	5.23	61.54	5.84	-6.69	4.39E-07	2.07E-05

cg13973180	C17orf101;C17orf101	17	Body;Body	N_Shore	63.24	6.97	69.93	7.59	-6.69	4.08E-05	1.30E-03
cg16263848	NTN1	17	Body	N_Shore	31.23	6.38	37.92	6.47	-6.69	2.61E-06	1.10E-04
cg00144673	MCCD1	6	Body	Open sea	61.38	5.92	68.07	5.02	-6.69	2.56E-07	1.24E-05
cg07719617	PER2	2	Body	Open sea	60.40	4.70	67.08	5.00	-6.69	7.20E-08	3.63E-06
cg11168104		5	Intergenic	Island	62.86	9.86	69.54	11.81	-6.69	1.78E-03	2.61E-02
cg24722112	AMIGO3;AMIGO3;RNF123	3	5'UTR;1stExon;Body	Island	31.00	10.08	37.68	9.45	-6.68	2.81E-03	3.58E-02
cg08289130		16	Intergenic	S_Shore	55.80	7.26	62.49	6.01	-6.68	4.58E-05	1.44E-03
cg07076076	UNKL	16	Body	Island	23.50	7.40	30.18	7.16	-6.68	2.70E-05	9.11E-04
cg21212034	LSG1	3	Body	N_Shelf	70.48	5.27	77.16	4.74	-6.68	5.22E-08	2.66E-06
cg24094897	MCC;MCC	5	Body;Body	Open sea	38.98	6.98	45.67	9.68	-6.68	2.50E-03	3.30E-02
cg23460285	FBRSL1	12	Body	S_Shelf	70.54	8.40	77.23	8.70	-6.68	1.07E-03	1.80E-02
cg17760391	MAST4;MAST4;MAST4	5	TSS200;Body;Body	Open sea	15.73	5.26	22.41	6.04	-6.68	3.26E-07	1.56E-05
cg05357108	CDC37	19	Body	N_Shore	70.66	4.93	77.33	3.92	-6.68	5.14E-09	2.78E-07
cg18184092	RERE;RERE;RERE	1	3'UTR;3'UTR;3'UTR	Open sea	48.84	5.56	55.51	6.07	-6.68	1.22E-06	5.41E-05
cg12210618	MCOLN3	1	TSS1500	S_Shore	36.54	4.82	43.22	7.51	-6.68	1.84E-06	7.93E-05
cg08449049	CDH15	16	Body	Island	14.33	4.19	21.00	9.12	-6.68	8.64E-05	2.48E-03
cg00022911	HAPLN4	19	Body	Island	28.12	7.32	34.79	8.24	-6.68	1.43E-05	5.20E-04
cg04799980		2	Intergenic	N_Shore	74.74	6.38	81.41	4.82	-6.67	2.04E-06	8.73E-05
cg02919615	EFNA2	19	Body	Island	38.13	5.16	44.80	5.48	-6.67	4.69E-08	2.39E-06
cg12944572	LATS2	13	Body	N_Shelf	75.73	4.76	82.40	4.10	-6.67	2.03E-11	1.20E-09
cg10539173	DUSP16	12	Body	Open sea	61.81	6.31	68.48	5.79	-6.67	8.01E-06	3.08E-04
cg19675103	ATP10D	4	5'UTR	Open sea	56.20	6.42	62.87	7.19	-6.67	2.72E-04	6.30E-03
cg02059214	HHLA2	3	TSS1500	Open sea	75.69	5.77	82.36	4.94	-6.67	1.59E-07	7.82E-06
cg10718395	TAF3	10	Body	Open sea	55.59	6.89	62.26	6.64	-6.67	2.68E-05	9.05E-04
cg23025804	SVIL;SVIL	10	Body;Body	Open sea	59.01	6.10	65.68	6.66	-6.67	5.46E-06	2.17E-04
cg20785796	SATB2	2	TSS1500	Island	27.85	4.81	34.52	6.47	-6.67	1.50E-07	7.38E-06
cg14041855		8	Intergenic	Open sea	37.53	5.54	44.20	6.33	-6.67	1.39E-06	6.12E-05
cg04714787		4	Intergenic	S_Shelf	18.88	6.43	25.53	8.64	-6.66	9.59E-05	2.70E-03
cg22812275	EZR;EZR	6	TSS1500;TSS1500	S_Shore	37.84	5.17	44.50	6.61	-6.66	7.79E-07	3.55E-05
cg01807241		4	Intergenic	Island	15.38	6.36	22.04	8.08	-6.66	7.84E-06	3.02E-04
cg02675946	SLC22A20	11	Body	N_Shore	8.90	4.30	15.56	9.07	-6.66	7.85E-07	3.57E-05
cg23429749		14	Intergenic	Island	66.79	6.67	73.44	7.62	-6.66	1.83E-05	6.47E-04
cg20559405	PTPRG	3	Body	Open sea	45.27	9.23	51.92	8.63	-6.65	1.34E-03	2.12E-02
cg23966361	RFNG	17	Body	N_Shore	77.94	6.70	84.59	5.49	-6.65	1.54E-05	5.53E-04
cg16496024	PCP4	21	TSS1500	Open sea	18.97	5.74	25.63	9.21	-6.65	1.04E-04	2.88E-03
cg12481170		5	Intergenic	Open sea	50.55	6.81	57.20	8.13	-6.65	3.75E-04	8.10E-03
cg26589665	THSD4	15	Body	Open sea	58.76	6.45	65.41	8.81	-6.65	7.02E-04	1.31E-02
cg00485171		15	Intergenic	Open sea	75.54	6.30	82.19	3.72	-6.65	2.04E-07	9.91E-06
cg25099892	C13orf35	13	5'UTR	Open sea	60.25	8.23	66.90	8.22	-6.65	1.02E-05	3.82E-04
cg04962978	SALL1;SALL1	16	3'UTR;3'UTR	S_Shore	61.63	8.89	68.27	8.35	-6.64	2.71E-03	3.49E-02
cg01675158	CCDC85C	14	Body	Open sea	49.99	6.54	56.64	5.36	-6.64	4.78E-06	1.92E-04
cg06759085	NAB1	2	3'UTR	Open sea	71.01	4.22	77.65	3.88	-6.64	2.12E-13	1.32E-11
cg22855860	MIR548F5;NBEA;MAB21L1	13	Body;Body;TSS200	N_Shore	31.56	6.47	38.20	9.68	-6.64	5.02E-05	1.56E-03
cg23501773	SEBOX;SEBOX	17	Body;TSS1500	Open sea	55.18	6.35	61.82	7.21	-6.64	3.41E-04	7.52E-03
cg03604774		1	Intergenic	Open sea	63.67	6.29	70.31	5.25	-6.64	4.04E-07	1.91E-05
cg11438441		3	Intergenic	N_Shelf	64.12	4.72	70.75	2.81	-6.63	3.25E-10	1.85E-08
cg17003143	C14orf73	14	Body	S_Shelf	58.73	6.84	65.36	7.31	-6.63	6.66E-05	1.99E-03
cg11201398		15	Intergenic	Open sea	39.04	7.02	45.68	9.44	-6.63	5.42E-04	1.08E-02
cg01450803		4	Intergenic	S_Shelf	62.93	7.15	69.56	7.31	-6.63	3.60E-05	1.17E-03
cg10839385	VSIG8;C1orf204;C1orf204	1	Body;5'UTR;1stExon	S_Shore	29.99	4.97	36.62	5.69	-6.63	1.95E-07	9.49E-06
cg01961752	TTLL10	1	TSS1500	N_Shore	17.82	7.16	24.45	10.52	-6.63	5.06E-04	1.02E-02
cg00935887		2	Intergenic	Island	50.57	8.53	57.20	10.49	-6.63	6.36E-04	1.22E-02
cg01899676	RFX4	12	Body	Open sea	38.94	6.24	45.56	7.73	-6.62	1.23E-05	4.52E-04

cg03731896	GYS1;GYS1;GYS1	19	Body;Body;Body	S_Shore	20.89	4.19	27.52	5.57	-6.62	2.87E-09	1.57E-07
cg21412629	PRDM16;PRDM16	1	Body;Body	N_Shore	51.76	6.24	58.38	10.10	-6.62	4.09E-04	8.66E-03
cg07791897		5	Intergenic	Open sea	67.99	5.70	74.61	5.10	-6.62	8.45E-07	3.83E-05
cg03663674	CAPN10;CAPN10;CAPN10;CAPN10	2	Body;Body;Body;Body	N_Shore	67.21	6.55	73.83	6.35	-6.62	9.48E-05	2.68E-03
cg22735105	ZBTB46	20	5'UTR	S_Shore	63.67	7.34	70.29	7.44	-6.62	4.47E-04	9.29E-03
cg08197509	NAPRT1	8	Body	N_Shore	49.84	6.47	56.46	8.42	-6.62	5.15E-04	1.04E-02
cg13242092	PTTG1IP	21	3'UTR	S_Shelf	60.05	5.89	66.66	5.33	-6.62	4.84E-07	2.26E-05
cg10428938	ARPC1B	7	Body	Island	17.63	5.37	24.25	7.39	-6.61	1.13E-05	4.19E-04
cg21536086	MIR190B	1	TSS1500	Open sea	51.86	6.19	58.47	5.20	-6.61	6.43E-07	2.96E-05
cg16932285	C1R	12	Body	Open sea	58.25	6.41	64.86	8.23	-6.61	5.20E-04	1.04E-02
cg26650383	SLC46A3;SLC46A3	13	Body;Body	N_Shelf	29.04	6.39	35.65	8.28	-6.61	2.50E-04	5.91E-03
cg11200222	KCNK2;KCNK2;KCNK2	1	TSS1500;TSS1500;Body	N_Shore	14.87	4.58	21.47	9.24	-6.60	7.05E-05	2.09E-03
cg24503449	PIK3R2	19	Body	Island	74.89	7.22	81.49	5.90	-6.60	9.54E-05	2.69E-03
cg21489266	LRRC2;TDGF1	3	5'UTR;TSS200	S_Shore	69.78	2.94	76.38	3.52	-6.60	2.10E-15	1.37E-13
cg23127986		6	Intergenic	Open sea	32.87	5.98	39.47	6.88	-6.60	6.37E-05	1.92E-03
cg01620165	DTX1	12	Body	Island	44.26	7.42	50.86	6.56	-6.60	2.76E-05	9.30E-04
cg27535211	PLXNA4;PLXNA4	7	Body;Body	Open sea	46.13	6.44	52.73	7.24	-6.60	1.08E-04	2.99E-03
cg11123545	APBB3;APBB3;APBB3;SRA1;APBB3	5	Body;Body;Body;TSS1500;Body	S_Shore	58.69	5.61	65.28	5.41	-6.60	2.35E-06	9.95E-05
cg11598897	C5orf42	5	Body	Island	39.92	2.41	46.51	2.88	-6.59	1.98E-17	1.35E-15
cg21251970	EXD3	9	5'UTR	Island	54.97	7.21	61.57	6.01	-6.59	8.32E-06	3.19E-04
cg09727934		5	Intergenic	Open sea	77.26	4.79	83.85	4.09	-6.59	5.60E-10	3.15E-08
cg13322954		12	Intergenic	N_Shelf	51.02	6.17	57.62	9.65	-6.59	4.17E-04	8.80E-03
cg25927215		6	Intergenic	Open sea	30.94	6.98	37.53	5.44	-6.59	3.26E-05	1.07E-03
cg22715764	PEX14	1	Body	Open sea	68.79	6.44	75.38	5.86	-6.59	1.51E-05	5.46E-04
cg12000995	KRTCAP3;KRTCAP3	2	TSS200;TSS200	Island	27.64	7.71	34.23	8.84	-6.59	2.75E-03	3.53E-02
cg22709202	ANKRD55;ANKRD55;ANKRD55	5	1stExon;Body;5'UTR	Open sea	28.84	6.76	35.43	9.59	-6.59	1.39E-03	2.18E-02
cg04612030	CCDC154	16	Body	N_Shore	87.59	12.84	94.18	1.49	-6.59	2.80E-05	9.39E-04
cg21464220	HDLBP	2	5'UTR	Open sea	60.53	8.21	67.12	5.05	-6.59	1.86E-04	4.67E-03
cg12252069	SKAP2	7	Body	Open sea	34.60	7.37	41.19	7.52	-6.59	2.05E-06	8.77E-05
cg14259208	COL15A1	9	Body	Open sea	51.23	6.34	57.82	7.04	-6.59	4.87E-06	1.95E-04
cg01509330		6	Intergenic	Open sea	70.99	8.30	77.58	7.38	-6.59	1.37E-04	3.62E-03
cg10144598	ATP2B4;ATP2B4	1	5'UTR;5'UTR	Open sea	26.78	6.45	33.36	6.41	-6.59	1.65E-07	8.08E-06
cg11520439	LOC286367	9	TSS200	Open sea	47.18	6.53	53.77	6.34	-6.58	8.63E-05	2.47E-03
cg10501602	NOS3	7	Body	Island	27.93	4.60	34.51	4.49	-6.58	4.11E-09	2.23E-07
cg26436583	PSTPIP2	18	Body	N_Shelf	57.27	4.92	63.85	6.15	-6.58	6.86E-08	3.46E-06
cg25190573	ACSL1;SLED1	4	Body;TSS1500	N_Shelf	67.61	7.55	74.19	7.52	-6.58	9.27E-04	1.62E-02
cg00047843	TMTC2	12	Body	Open sea	58.98	4.92	65.56	3.33	-6.58	2.43E-09	1.34E-07
cg16473915	HTRA4;HTRA4	8	1stExon;5'UTR	Island	28.78	6.03	35.36	8.55	-6.58	3.12E-05	1.03E-03
cg04428163	SDCBP2;SDCBP2	20	1stExon;Body	Island	43.04	5.54	49.62	6.45	-6.58	8.34E-07	3.78E-05
cg02377053	IL1RN;IL1RN;IL1RN;IL1RN	2	Body;Body;Body;Body	Open sea	45.24	5.62	51.81	7.51	-6.58	3.60E-05	1.17E-03
cg22997113	HOXA4	7	1stExon	Island	30.79	5.96	37.37	5.48	-6.58	1.48E-06	6.48E-05
cg05041795		8	Intergenic	S_Shore	69.31	7.41	75.89	7.25	-6.58	4.81E-05	1.50E-03
cg23985641	FAM50B	6	TSS1500	N_Shore	36.19	5.57	42.77	4.80	-6.58	9.74E-08	4.86E-06
cg02452639		1	Intergenic	Island	38.04	6.09	44.61	7.10	-6.58	3.52E-05	1.15E-03
cg10573643	PRDM16;PRDM16	1	Body;Body	N_Shore	45.91	6.58	52.48	8.30	-6.57	3.84E-04	8.25E-03
cg22588546	MYH10	17	Body	S_Shelf	70.79	7.08	77.37	4.80	-6.57	1.06E-05	3.98E-04
cg25597535	NEUROD6	7	5'UTR	S_Shelf	67.58	7.26	74.16	6.89	-6.57	1.12E-05	4.18E-04
cg23278418	VPS13D;VPS13D	1	Body;Body	Open sea	23.15	6.84	29.73	9.11	-6.57	6.70E-04	1.27E-02
cg22157025		11	Intergenic	S_Shelf	60.34	7.38	66.91	7.35	-6.57	3.07E-04	6.92E-03
cg08535058	RNASEH2A	19	Body	S_Shelf	56.86	4.83	63.44	4.52	-6.57	2.78E-08	1.44E-06
cg27307693		15	Intergenic	N_Shelf	44.96	4.40	51.53	7.03	-6.57	9.46E-07	4.26E-05
cg02601489	TDH	8	Body	N_Shore	54.78	7.57	61.35	7.90	-6.57	9.12E-05	2.59E-03
cg02399788	C16orf45	16	Body	Open sea	62.16	5.30	68.73	5.73	-6.57	4.17E-08	2.14E-06

cg01219135		7	Intergenic	Island	47.84	9.77	54.40	11.17	-6.57	3.88E-03	4.47E-02
cg04787579		14	Intergenic	N_Shelf	41.39	5.42	47.96	4.67	-6.57	3.07E-08	1.59E-06
cg03300078	HCCA2;LOC402778	11	Body;TSS200	Island	36.87	8.25	43.43	8.00	-6.56	5.27E-04	1.05E-02
cg27028479	HAGH;HAGH	16	Body;Body	N_Shelf	53.11	6.98	59.68	9.15	-6.56	9.59E-04	1.66E-02
cg26705720	JAG1	20	Body	Island	12.25	5.25	18.82	7.03	-6.56	2.19E-07	1.06E-05
cg20249071	UBASH3B	11	3'UTR	Open sea	44.90	7.03	51.46	9.31	-6.56	1.04E-03	1.76E-02
cg05430021		8	Intergenic	Open sea	46.05	5.65	52.61	4.91	-6.56	8.87E-08	4.44E-06
cg10587183	MAB21L1;MIR548F5;NBEA;MAB21L1	13	1stExon;Body;Body;3'UTR	N_Shore	52.09	6.39	58.65	9.23	-6.56	2.10E-04	5.13E-03
cg17897699		8	Intergenic	Open sea	72.31	4.32	78.87	3.84	-6.56	5.88E-10	3.31E-08
cg21201357	FRMPD1	9	3'UTR	Open sea	66.76	5.45	73.32	5.62	-6.56	1.31E-07	6.47E-06
cg11590772	ADAMTS10	19	Body	Island	52.55	5.30	59.10	5.68	-6.56	5.88E-08	2.99E-06
cg02057755	AGBL4	1	3'UTR	Open sea	58.60	7.16	65.15	6.27	-6.56	3.69E-05	1.19E-03
cg13893427		17	Intergenic	N_Shelf	61.09	6.55	67.65	6.43	-6.56	1.23E-04	3.31E-03
cg23931323	PLEKHB1;PLEKHB1;PLEKHB1;PLEKHB1	11	Body;Body;Body;Body;Body	Island	11.18	5.74	17.73	10.21	-6.55	1.07E-03	1.80E-02
cg01935405	ZFPM1	16	Body	N_Shore	72.90	8.51	79.45	6.17	-6.55	2.59E-04	6.07E-03
cg19668234	PRSS50	3	TSS200	Island	30.23	7.60	36.78	7.66	-6.55	2.90E-04	6.63E-03
cg18827332		12	Intergenic	Island	12.17	3.55	18.72	3.98	-6.55	9.80E-13	5.99E-11
cg24899767	CASKIN2;CASKIN2	17	Body;Body	N_Shore	59.49	4.73	66.04	3.84	-6.55	1.04E-08	5.52E-07
cg12654412		16	Intergenic	Island	36.47	7.57	43.02	9.52	-6.55	1.12E-03	1.86E-02
cg23247968	C10orf26;C10orf26;C10orf26	10	5'UTR;Body;1stExon	Open sea	24.90	5.95	31.46	6.43	-6.55	4.91E-05	1.53E-03
cg16863617		4	Intergenic	Open sea	28.47	5.33	35.02	6.88	-6.55	1.04E-06	4.66E-05
cg21158975	VAV2;VAV2	9	Body;Body	N_Shelf	63.70	7.82	70.25	7.05	-6.55	8.80E-04	1.56E-02
cg18221226	NCOR2;NCOR2	12	5'UTR;5'UTR	Open sea	40.97	8.19	47.52	6.74	-6.55	7.37E-06	2.85E-04
cg25900150	ZC3H3	8	Body	Island	46.96	6.64	53.50	10.31	-6.55	2.26E-03	3.07E-02
cg19563525	FRS2;FRS2	12	5'UTR;5'UTR	Open sea	52.47	6.37	59.02	5.83	-6.55	2.67E-06	1.12E-04
cg08657492	HOXA4	7	TSS1500	S_Shore	39.11	3.87	45.65	4.21	-6.54	3.14E-13	1.95E-11
cg03860037	PDX1	13	Body	N_Shore	23.12	7.49	29.66	9.47	-6.54	1.55E-03	2.36E-02
cg14377720		15	Intergenic	Open sea	46.20	6.83	52.74	8.97	-6.54	1.00E-03	1.71E-02
cg14118850	HPCAL1;HPCAL1	2	5'UTR;5'UTR	S_Shelf	41.14	6.11	47.68	6.16	-6.54	1.06E-05	3.98E-04
cg22778903	MX2	21	5'UTR	Open sea	37.97	8.96	44.51	10.58	-6.54	1.20E-03	1.97E-02
cg10210397		8	Intergenic	Open sea	31.70	6.31	38.24	6.73	-6.54	7.85E-05	2.28E-03
cg00514241	MN1	22	1stExon	Island	49.02	5.44	55.56	6.08	-6.54	2.18E-06	9.28E-05
cg14860397	WNT11	11	TSS1500	N_Shore	23.99	7.16	30.53	8.47	-6.54	7.51E-05	2.20E-03
cg25534244	HLF	17	TSS1500	N_Shore	28.49	5.27	35.03	8.25	-6.54	3.24E-05	1.07E-03
cg22547480	TMIGD1	17	TSS200	Open sea	53.87	5.52	60.41	6.06	-6.54	1.60E-06	6.97E-05
cg05131266	C14orf159;C14orf159;C14orf159;SNO	14	5'UTR;5'UTR;5'UTR;TSS1500;5'UTR;5'UTR	Open sea	47.75	5.35	54.29	6.83	-6.54	5.55E-06	2.20E-04
cg21167502	ZFYVE21	14	Body	S_Shore	74.11	6.64	80.64	5.72	-6.54	7.34E-05	2.16E-03
cg08765100	PRICKLE2	3	TSS1500	Open sea	37.95	6.20	44.48	6.54	-6.54	6.02E-06	2.37E-04
cg00320094	GPR177;GPR177	1	Body;Body	N_Shore	60.90	5.82	67.44	5.23	-6.54	5.05E-07	2.36E-05
cg01299774		5	Intergenic	Island	42.20	6.26	48.74	6.05	-6.53	1.40E-05	5.09E-04
cg00842076	FOXN3;FOXN3	14	5'UTR;5'UTR	N_Shore	42.27	5.79	48.80	8.35	-6.53	1.04E-04	2.89E-03
cg02347904	RFPL2;RFPL2;RFPL2	22	TSS200;5'UTR;TSS1500	Open sea	73.47	7.47	80.00	5.55	-6.53	3.93E-06	1.60E-04
cg17337874	TTYH3	7	3'UTR	Island	69.77	9.21	76.30	7.70	-6.53	1.39E-03	2.18E-02
cg02073887		15	Intergenic	Open sea	49.40	6.12	55.93	5.48	-6.53	4.22E-07	1.99E-05
cg01594949	NRARP	9	TSS1500	S_Shore	61.55	5.83	68.08	5.32	-6.53	9.66E-07	4.35E-05
cg06678594	THADA;THADA	2	Body;Body	Open sea	56.68	5.41	63.21	6.29	-6.53	6.66E-06	2.60E-04
cg06499778		11	Intergenic	Open sea	34.25	4.96	40.77	7.62	-6.53	4.69E-06	1.89E-04
cg09037813	LRRFIP1;LRRFIP1;LRRFIP1;LRRFIP1;LRI	2	TSS1500;TSS1500;TSS1500;TSS1500;Body	N_Shore	60.02	6.54	66.55	4.99	-6.53	2.34E-06	9.90E-05
cg01195214	UBE2I;UBE2I;UBE2I;UBE2I	16	5'UTR;5'UTR;5'UTR;5'UTR	N_Shore	30.04	5.81	36.57	7.02	-6.53	6.26E-05	1.89E-03
cg26348226	ECE1;ECE1;ECE1	1	TSS1500;TSS1500;Body	S_Shore	42.90	5.93	49.42	6.83	-6.53	1.61E-05	5.78E-04
cg02269083	ALPK2	18	TSS1500	Open sea	35.57	5.73	42.10	5.33	-6.53	8.68E-07	3.93E-05
cg12129839		17	Intergenic	Open sea	44.10	5.83	50.63	9.26	-6.52	3.35E-04	7.42E-03
cg02260774	SH3TC1	4	Body	Open sea	66.18	6.60	72.70	4.21	-6.52	3.48E-06	1.43E-04

cg02991571		13	Intergenic	N_Shore	16.59	5.39	23.12	8.91	-6.52	1.06E-05	3.97E-04
cg27408683	NACC2	9	Body	Island	66.35	6.99	72.87	5.97	-6.52	2.16E-05	7.49E-04
cg19022525	ATP6V0A1;ATP6V0A1;ATP6V0A1	17	Body;Body;Body	Open sea	66.34	6.11	72.86	4.70	-6.52	6.46E-07	2.97E-05
cg10402804	GPR132	14	5'UTR	Open sea	51.72	7.08	58.24	9.46	-6.52	1.49E-03	2.29E-02
cg16231923	NPTN;NPTN;NPTN;NPTN	15	Body;Body;Body;Body	N_Shore	26.95	5.48	33.47	5.70	-6.52	1.17E-07	5.80E-06
cg22470465	LOC729991-MEF2B;LOC729991-MEF2	19	Body;5'UTR;5'UTR;Body	N_Shelf	50.86	5.77	57.38	6.45	-6.52	8.25E-06	3.16E-04
cg14811105	NR4A2	2	Body	Island	56.21	6.06	62.73	5.37	-6.52	4.16E-08	2.13E-06
cg14029170	GCLC	6	Body	Open sea	61.23	6.51	67.75	6.50	-6.52	4.54E-05	1.43E-03
cg14985891	CASQ2	1	Body	Open sea	46.83	5.89	53.35	5.95	-6.52	1.53E-08	8.05E-07
cg01432520	ESRRG;ESRRG;ESRRG	1	5'UTR;5'UTR;5'UTR	Open sea	52.38	5.03	58.90	7.72	-6.52	5.70E-06	2.25E-04
cg03880033	CES1;CES1;CES1	16	Body;Body;Body	Island	32.05	6.07	38.57	4.78	-6.52	5.81E-06	2.30E-04
cg01695406	TMEM190	19	Body	Island	46.75	6.79	53.27	8.45	-6.52	2.92E-04	6.66E-03
cg08651538	C5orf13;C5orf13;C5orf13;C5orf13;C5c	5	Body;Body;Body;Body;Body;Body;Body;Bc	Open sea	48.46	5.28	54.98	5.35	-6.52	4.07E-07	1.92E-05
cg05025392		17	Intergenic	Open sea	57.25	10.62	63.76	6.33	-6.52	7.85E-04	1.43E-02
cg24556395	MYO10	5	Body	Open sea	49.65	6.30	56.16	9.56	-6.51	1.01E-03	1.73E-02
cg22258315		8	Intergenic	N_Shore	44.42	6.75	50.93	7.15	-6.51	5.26E-05	1.62E-03
cg11617964	PRRT1	6	Body	Island	56.43	5.31	62.94	5.27	-6.51	6.26E-07	2.89E-05
cg04235837	IQSEC1	3	TSS1500	Island	27.82	5.10	34.32	5.36	-6.51	1.04E-08	5.52E-07
cg21917740		2	Intergenic	S_Shore	39.15	9.14	45.66	8.12	-6.51	6.95E-05	2.06E-03
cg02606018		12	Intergenic	Open sea	18.76	2.42	25.27	2.92	-6.51	7.69E-18	5.30E-16
cg02530860		8	Intergenic	Island	68.23	6.64	74.74	7.76	-6.51	1.27E-04	3.40E-03
cg16167624	CLDN18;CLDN18	3	TSS200;Body	N_Shore	53.04	7.90	59.54	10.82	-6.51	2.93E-03	3.68E-02
cg08269974	ELANE	19	Body	Island	47.86	7.13	54.36	7.57	-6.50	8.67E-05	2.48E-03
cg00446697	HCCA2	11	Body	Open sea	62.76	7.28	69.27	6.56	-6.50	8.17E-05	2.36E-03
cg10002185	TRPM4	19	Body	S_Shore	68.31	8.66	74.81	9.05	-6.50	4.52E-03	4.96E-02
cg01739725		14	Intergenic	S_Shelf	18.49	5.49	24.99	8.77	-6.50	1.14E-04	3.11E-03
cg08499840	CCL11	17	TSS1500	Open sea	60.90	5.66	67.40	8.09	-6.50	1.92E-05	6.75E-04
cg00531745		6	Intergenic	N_Shore	66.05	4.84	72.55	4.28	-6.50	7.28E-09	3.91E-07
cg00827893	PDS5B	13	5'UTR	S_Shore	39.59	5.41	46.08	6.10	-6.50	3.06E-06	1.27E-04
cg00339695	SLC5A11	16	TSS200	Open sea	49.11	10.70	55.61	9.43	-6.50	9.06E-04	1.59E-02
cg15107724	TFAP2E	1	Body	Open sea	47.73	4.80	54.22	6.45	-6.50	1.98E-07	9.64E-06
cg05193538	MAN1C1	1	Body	Island	37.97	7.90	44.46	8.26	-6.49	3.67E-03	4.31E-02
cg08573038	GIT2;GIT2;GIT2;GIT2;GIT2;GIT2	12	Body;Body;Body;Body;Body;Body	Open sea	51.39	6.61	57.89	6.25	-6.49	4.56E-05	1.44E-03
cg03450107	HIVEP3;HIVEP3	1	Body;Body	N_Shore	55.08	6.53	61.57	7.46	-6.49	2.36E-06	9.97E-05
cg06521646	HCCA2	11	Body	S_Shelf	77.91	9.22	84.40	6.38	-6.49	2.73E-03	3.51E-02
cg00577847		1	Intergenic	Open sea	47.02	5.74	53.51	6.77	-6.49	9.49E-07	4.28E-05
cg27320518	MBL1P	10	TSS1500	Open sea	50.86	5.49	57.34	5.35	-6.49	2.16E-07	1.05E-05
cg20881888	ZMYND10;ZMYND10	3	1stExon;5'UTR	Island	24.10	6.44	30.59	6.87	-6.49	7.18E-06	2.79E-04
cg23551605	AGAP1;AGAP1	2	Body;Body	N_Shore	75.53	5.71	82.01	4.24	-6.48	7.08E-07	3.25E-05
cg18244286	SERPINB9	6	TSS1500	S_Shore	49.09	6.19	55.58	5.34	-6.48	8.56E-06	3.27E-04
cg12975892	ERICH1	8	Body	S_Shore	51.89	7.06	58.37	7.03	-6.48	2.43E-04	5.77E-03
cg07782795	BAIAP2;BAIAP2;BAIAP2;BAIAP2	17	Body;Body;Body;Body	Open sea	74.05	6.61	80.53	5.42	-6.48	8.14E-06	3.12E-04
cg22130585	ANO1	11	TSS200	Island	11.21	5.21	17.69	8.34	-6.48	1.08E-05	4.03E-04
cg04497389		16	Intergenic	Island	42.47	8.21	48.95	9.02	-6.48	2.74E-04	6.35E-03
cg11621464	LTBP4;LTBP4;LTBP4	19	Body;Body;Body	Island	24.60	6.52	31.08	7.75	-6.48	7.61E-05	2.22E-03
cg06749789	THAP4;THAP4	2	Body;Body	Island	16.39	4.48	22.87	6.18	-6.48	4.62E-08	2.36E-06
cg22302111		7	Intergenic	Open sea	55.40	7.32	61.88	8.33	-6.48	1.27E-03	2.04E-02
cg17191567		10	Intergenic	Open sea	68.30	8.47	74.78	6.88	-6.48	1.02E-03	1.73E-02
cg10179330		6	Intergenic	Open sea	39.93	5.70	46.40	6.23	-6.48	5.49E-07	2.55E-05
cg12618624	TMEM212	3	TSS1500	Open sea	68.09	8.57	74.57	9.36	-6.47	4.14E-03	4.67E-02
cg23530064	BACH1;BACH1;BACH1	21	Body;Body;Body	Open sea	65.41	6.34	71.88	4.75	-6.47	1.09E-05	4.06E-04
cg14042396		2	Intergenic	N_Shore	53.17	7.01	59.64	6.51	-6.47	5.32E-06	2.12E-04
cg19111408		15	Intergenic	Open sea	30.22	4.36	36.69	4.24	-6.47	9.05E-11	5.24E-09

cg10420159	WDR81;WDR81;WDR81;WDR81	17	3'UTR;3'UTR;3'UTR;3'UTR	Open sea	68.75	8.48	75.22	6.20	-6.47	6.93E-04	1.30E-02
cg24854681	MCM7;MCM7;MIR25	7	Body;Body;TSS200	Open sea	52.15	6.21	58.62	5.42	-6.47	2.64E-05	8.93E-04
cg07013148		8	Intergenic	Open sea	58.26	6.90	64.73	6.33	-6.47	1.79E-04	4.52E-03
cg16541222	EPB41L4A	5	Body	Open sea	42.76	7.19	49.23	10.69	-6.46	3.47E-03	4.14E-02
cg04579966	SPATA13;SPATA13	13	Body;Body	Open sea	22.28	7.76	28.74	9.35	-6.46	1.04E-04	2.90E-03
cg11241541	TACC1;TACC1	8	1stExon;5'UTR	Open sea	12.26	7.01	18.72	9.43	-6.46	4.89E-04	9.95E-03
cg21944491	LTBP4;LTBP4;LTBP4	19	Body;Body;Body	Island	17.78	6.97	24.24	7.72	-6.46	6.06E-04	1.17E-02
cg07724304	CASKIN2	17	5'UTR	N_Shore	45.60	5.55	52.06	6.24	-6.46	9.19E-06	3.49E-04
cg08294986	USP2;USP2	11	3'UTR;3'UTR	N_Shore	18.16	5.49	24.62	7.14	-6.46	2.61E-05	8.85E-04
cg07385778		3	Intergenic	Open sea	45.27	8.24	51.73	7.04	-6.46	4.15E-04	8.77E-03
cg24167841	GPR182	12	Body	Open sea	65.44	7.69	71.90	8.41	-6.46	1.07E-03	1.80E-02
cg23881458		6	Intergenic	S_Shelf	55.16	5.88	61.62	5.78	-6.46	9.16E-06	3.48E-04
cg20624886	DMPK;DMPK;DMPK;DMPK	19	Body;Body;Body;Body	S_Shore	48.58	6.79	55.04	8.36	-6.46	5.10E-04	1.03E-02
cg19256094	GDF1;LASS1	19	Body;3'UTR	Island	32.54	5.20	39.00	7.65	-6.46	5.91E-06	2.33E-04
cg24593324	VCAM1;VCAM1	1	Body;Body	Open sea	59.44	6.85	65.90	8.35	-6.46	1.97E-03	2.79E-02
cg24457743		10	Intergenic	Open sea	54.36	5.63	60.81	6.83	-6.46	1.32E-05	4.83E-04
cg13096007	SOD3	4	3'UTR	S_Shore	10.64	3.05	17.09	7.29	-6.45	3.40E-10	1.93E-08
cg21858961	MCM3	6	Body	N_Shore	79.52	4.41	85.97	3.66	-6.45	1.27E-11	7.51E-10
cg11388673		8	Intergenic	Island	75.85	6.74	82.29	5.57	-6.45	1.88E-05	6.62E-04
cg25718467	LOC145845	15	Body	Island	20.87	6.03	27.32	8.40	-6.45	3.33E-05	1.09E-03
cg00579502		1	Intergenic	Open sea	81.15	9.89	87.60	7.67	-6.45	1.59E-03	2.40E-02
cg05181941		12	Intergenic	N_Shore	61.96	6.67	68.41	7.57	-6.45	5.43E-05	1.67E-03
cg21167159	H19;MIR675	11	Body;TSS200	Island	71.38	5.01	77.83	5.85	-6.44	2.22E-08	1.16E-06
cg01608400	WWOX	16	Body	Open sea	39.65	7.71	46.09	9.45	-6.44	2.33E-03	3.14E-02
cg15065854	CFD	19	Body	Island	48.25	5.16	54.69	5.93	-6.44	2.34E-06	9.92E-05
cg14091258	PTPRN2;PTPRN2;PTPRN2	7	Body;Body;Body	Island	61.49	7.11	67.93	6.85	-6.44	2.44E-05	8.34E-04
cg16113156		18	Intergenic	Island	38.45	7.47	44.89	8.79	-6.44	9.19E-04	1.61E-02
cg14434755		12	Intergenic	N_Shore	29.51	6.11	35.95	8.29	-6.44	1.61E-04	4.16E-03
cg04714110		10	Intergenic	Island	29.48	5.16	35.92	8.26	-6.44	3.14E-05	1.04E-03
cg15725639	DHX34	19	Body	S_Shelf	65.27	6.27	71.71	4.39	-6.44	1.05E-05	3.93E-04
cg05729827	CSPG4	15	Body	Open sea	61.80	7.14	68.24	7.39	-6.44	1.17E-04	3.17E-03
cg12013713	PARP12	7	Body	N_Shore	51.87	6.12	58.31	3.98	-6.44	1.32E-06	5.83E-05
cg02100011	TMEM184B	22	Body	Open sea	41.01	8.47	47.44	7.76	-6.44	2.84E-03	3.61E-02
cg19230501		10	Intergenic	N_Shelf	45.80	6.35	52.24	6.63	-6.43	1.22E-04	3.31E-03
cg09171931		11	Intergenic	Open sea	53.08	5.87	59.52	6.50	-6.43	3.08E-05	1.02E-03
cg23260993	EFEMP1;EFEMP1;EFEMP1	2	Body;Body;Body	N_Shore	33.17	7.78	39.60	11.17	-6.43	2.68E-03	3.46E-02
cg18075315	RFX4	12	Body	Open sea	30.99	6.41	37.43	9.25	-6.43	5.78E-04	1.13E-02
cg25966908		7	Intergenic	N_Shore	46.79	7.36	53.22	7.39	-6.43	7.27E-05	2.14E-03
cg18678121	SEC61A2;SEC61A2;SEC61A2;SEC61A2;	10	TSS1500;TSS1500;TSS1500;TSS1500;TSS15	N_Shore	67.60	7.65	74.03	6.49	-6.43	7.49E-04	1.38E-02
cg05663453	ZBTB40;ZBTB40	1	5'UTR;5'UTR	S_Shelf	69.76	6.78	76.19	5.63	-6.43	2.94E-05	9.79E-04
cg16621790	ZMYND10	3	TSS1500	S_Shore	34.16	6.57	40.59	6.70	-6.43	5.60E-06	2.22E-04
cg12871835	SGK1	6	TSS1500	S_Shore	30.64	5.61	37.07	6.19	-6.43	3.34E-07	1.59E-05
cg21238619	GAA;GAA;GAA	17	Body;Body;Body	Island	36.34	4.68	42.77	4.51	-6.43	5.43E-08	2.76E-06
cg00091064		6	Intergenic	Island	43.48	7.02	49.91	10.73	-6.42	1.98E-03	2.81E-02
cg19265913	ZCCHC24	10	Body	Open sea	63.45	5.68	69.88	6.36	-6.42	2.60E-05	8.81E-04
cg23752752	FOXK1	7	Body	Open sea	19.10	5.04	25.52	7.11	-6.42	2.23E-06	9.48E-05
cg17962090		2	Intergenic	N_Shore	48.05	4.62	54.47	6.72	-6.42	2.75E-06	1.15E-04
cg14359435	USP2;USP2	11	Body;Body	Island	17.40	5.97	23.82	7.17	-6.42	1.37E-04	3.63E-03
cg23166773	SDCCAG8;AKT3	1	Body;3'UTR	Open sea	44.33	7.38	50.75	8.70	-6.42	2.44E-03	3.25E-02
cg07875121	PFDN1	5	Body	Open sea	58.22	4.70	64.64	3.68	-6.42	1.20E-09	6.70E-08
cg13040392		9	Intergenic	Island	56.04	8.44	62.46	6.63	-6.42	3.96E-04	8.45E-03
cg16676413	STX1A;STX1A	7	Body;Body	N_Shore	21.98	3.92	28.40	5.64	-6.42	3.09E-08	1.60E-06
cg01010221		2	Intergenic	Open sea	34.07	4.00	40.49	5.03	-6.42	1.17E-09	6.51E-08

cg26857192	H19	11	Body	Island	61.80	4.88	68.22	6.52	-6.42	9.33E-07	4.21E-05
cg07504200		19	Intergenic	Island	69.05	9.96	75.46	9.37	-6.42	2.44E-03	3.25E-02
cg20176388	VSNL1	2	5'UTR	S_Shelf	58.28	7.03	64.70	9.40	-6.42	1.01E-03	1.72E-02
cg10775173	PRKAG2	7	Body	Open sea	51.43	5.91	57.85	7.14	-6.42	2.59E-05	8.78E-04
cg08658895	BAIAP2;BAIAP2;BAIAP2;BAIAP2	17	Body;Body;Body;Body	Open sea	65.85	7.47	72.27	6.48	-6.41	2.04E-04	5.01E-03
cg25836232	TUBB6	18	TSS1500	N_Shore	34.26	7.48	40.68	9.96	-6.41	2.62E-03	3.41E-02
cg04429994		16	Intergenic	Open sea	63.69	7.33	70.11	5.20	-6.41	1.46E-04	3.82E-03
cg04664309		6	Intergenic	Open sea	48.94	5.95	55.35	9.78	-6.41	2.63E-04	6.15E-03
cg24594504	GFOD1	6	Body	Open sea	47.88	6.55	54.29	7.31	-6.41	4.66E-07	2.18E-05
cg20542619	NFATC1;NFATC1;NFATC1;NFATC1	18	3'UTR;3'UTR;3'UTR;3'UTR	Island	53.16	8.72	59.57	9.37	-6.41	4.25E-03	4.76E-02
cg06433816	EXD3	9	5'UTR	Island	75.51	8.65	81.92	6.18	-6.41	1.92E-04	4.78E-03
cg14065590	PCDHB11	5	TSS200	N_Shore	64.34	7.37	70.75	6.85	-6.41	3.44E-04	7.58E-03
cg05635340	C11orf34	11	Body	Open sea	65.70	9.91	72.11	8.40	-6.41	1.30E-03	2.08E-02
cg11418177	IL15;IL15	4	5'UTR;5'UTR	Open sea	34.31	5.99	40.72	7.04	-6.41	3.42E-05	1.12E-03
cg12747469	CCDC108;CCDC108	2	TSS1500;TSS1500	S_Shore	62.37	8.84	68.78	10.31	-6.41	1.55E-03	2.36E-02
cg00687306		15	Intergenic	Open sea	28.65	5.92	35.06	6.22	-6.41	1.14E-06	5.09E-05
cg14585054	CACNB1;CACNB1;CACNB1	17	Body;Body;Body	N_Shelf	48.77	6.31	55.17	8.36	-6.41	4.41E-04	9.20E-03
cg19221545	KCNIP2;KCNIP2;KCNIP2;KCNIP2;KCNIP	10	TSS200;TSS200;TSS200;TSS200;TSS200;TS	S_Shore	17.63	4.11	24.04	7.40	-6.41	2.35E-07	1.14E-05
cg26404226		10	Intergenic	Open sea	29.75	5.40	36.16	6.84	-6.41	3.61E-05	1.17E-03
cg04002794	FNDC3B;FNDC3B	3	5'UTR;5'UTR	Open sea	41.61	5.90	48.02	5.70	-6.41	4.71E-08	2.41E-06
cg13338783	PNMAL2	19	1stExon	Island	9.19	2.40	15.59	6.80	-6.40	2.88E-11	1.69E-09
cg01338139	C15orf53	15	TSS1500	Open sea	39.27	10.48	45.67	8.66	-6.40	7.12E-04	1.33E-02
cg25995955	LRBA	4	Body	N_Shore	51.63	7.82	58.03	6.32	-6.40	7.83E-05	2.28E-03
cg22507154		1	Intergenic	Island	51.14	6.70	57.54	5.84	-6.40	1.04E-05	3.90E-04
cg15467834		21	Intergenic	Island	72.97	7.02	79.37	6.30	-6.40	2.52E-05	8.58E-04
cg22396755	RAP1GAP;RAP1GAP	1	5'UTR;5'UTR	Island	14.97	7.06	21.37	8.04	-6.40	2.97E-05	9.90E-04
cg02555243	SOX5;SOX5	12	5'UTR;Body	Open sea	72.94	8.36	79.34	8.00	-6.40	1.43E-03	2.22E-02
cg17611045	FGF12	3	Body	Open sea	33.30	8.71	39.69	7.98	-6.40	6.31E-05	1.90E-03
cg20747577	SKAP2	7	Body	Open sea	19.95	5.52	26.35	7.20	-6.40	1.87E-08	9.79E-07
cg00562969	AJAP1;AJAP1	1	Body;Body	Open sea	44.89	5.14	51.29	6.17	-6.40	9.34E-07	4.21E-05
cg07137845	CLDN11	3	TSS200	Island	15.74	4.22	22.14	7.54	-6.40	9.13E-07	4.12E-05
cg07820783	FLJ32810	11	Body	Open sea	74.75	5.50	81.14	5.26	-6.40	2.33E-08	1.22E-06
cg20017590	SPTBN1;SPTBN1	2	Body;Body	N_Shelf	40.86	8.21	47.26	7.85	-6.39	3.95E-03	4.53E-02
cg02217035	C14orf73	14	Body	S_Shelf	53.44	5.50	59.83	7.02	-6.39	3.65E-05	1.18E-03
cg05132118		13	Intergenic	Open sea	80.00	5.18	86.39	5.16	-6.39	1.93E-07	9.41E-06
cg04056576	PPM1L	3	Body	S_Shore	32.45	7.41	38.84	8.73	-6.39	1.51E-04	3.94E-03
cg01329005	BST2	19	TSS1500	Open sea	17.44	8.08	23.83	10.82	-6.39	4.04E-03	4.60E-02
cg18308535	FKBP8	19	Body	N_Shore	79.70	6.32	86.08	3.88	-6.39	8.14E-07	3.70E-05
cg18388270		1	Intergenic	Open sea	24.70	8.32	31.09	9.71	-6.39	1.73E-03	2.55E-02
cg26200585	PRX;PRX;PRX;PRX	19	1stExon;5'UTR;1stExon;5'UTR	Open sea	55.79	5.28	62.17	6.83	-6.39	2.57E-06	1.08E-04
cg01438467	SLC43A2	17	Body	Island	60.45	4.65	66.84	5.74	-6.39	1.25E-08	6.60E-07
cg09146183	MAFF;MAFF;MAFF;MAFF	22	Body;Body;Body;5'UTR	Island	23.40	4.64	29.78	7.13	-6.38	3.22E-06	1.33E-04
cg00128386	KIAA0182;KIAA0182	16	5'UTR;Body	N_Shore	64.42	6.81	70.80	6.24	-6.38	1.54E-04	4.00E-03
cg19577080	INPP4B;INPP4B	4	5'UTR;5'UTR	Open sea	64.82	5.15	71.20	6.21	-6.38	3.61E-08	1.86E-06
cg27259271	SDCCAG8	1	Body	Island	6.56	4.18	12.95	10.05	-6.38	2.77E-05	9.30E-04
cg10146929	HIST1H1A	6	1stExon	N_Shelf	20.96	5.07	27.34	7.31	-6.38	3.23E-05	1.06E-03
cg11153256		7	Intergenic	Open sea	47.12	6.99	53.51	8.20	-6.38	5.16E-04	1.04E-02
cg25207447	PTK7;PTK7;PTK7;PTK7	6	TSS200;TSS200;TSS200;TSS200	N_Shore	21.03	5.27	27.41	6.27	-6.38	9.75E-07	4.38E-05
cg16190225	C7orf50;C7orf50;C7orf50	7	Body;Body;Body	Open sea	40.10	6.52	46.49	10.18	-6.38	2.78E-03	3.56E-02
cg12188416	TP63;TP63;TP63	3	TSS1500;TSS1500;TSS1500	Open sea	35.45	8.35	41.83	8.24	-6.38	5.01E-04	1.01E-02
cg11431957	VILL	3	Body	Island	16.63	4.22	23.01	5.69	-6.38	2.95E-08	1.53E-06
cg19771781	RAPSN;RAPSN	11	TSS200;TSS200	Open sea	65.08	7.96	71.46	7.69	-6.38	6.16E-04	1.19E-02
cg18910243	MARCH8;MARCH8;MARCH8	10	Body;Body;Body	Open sea	55.67	6.68	62.05	7.16	-6.38	4.35E-04	9.10E-03

cg17484699	SHROOM3	4	Body	S_Shore	54.90	6.68	61.28	5.77	-6.38	3.10E-06	1.29E-04
cg02147316	ACVR2B	3	Body	S_Shelf	56.77	7.04	63.15	8.57	-6.38	9.58E-04	1.66E-02
cg21849770	DEAF1	11	Body	Open sea	76.93	4.88	83.31	4.71	-6.38	1.79E-08	9.41E-07
cg00712857	MACROD1	11	Body	Island	32.84	5.97	39.22	5.67	-6.38	2.66E-07	1.28E-05
cg06470855		13	Intergenic	Island	54.50	7.47	60.88	7.54	-6.38	2.53E-04	5.95E-03
cg10162914	MFAP3L	4	5'UTR	N_Shelf	53.25	6.97	59.62	6.25	-6.37	2.34E-04	5.61E-03
cg14041452		14	Intergenic	S_Shelf	24.90	5.28	31.27	7.08	-6.37	3.16E-06	1.31E-04
cg04453501		7	Intergenic	Island	74.33	9.89	80.70	9.01	-6.37	4.07E-03	4.62E-02
cg22873167	FGFRL1;FGFRL1;FGFRL1	4	3'UTR;3'UTR;3'UTR	N_Shore	58.02	5.16	64.39	6.18	-6.37	1.18E-05	4.37E-04
cg19572487	RARA;RARA;RARA	17	5'UTR;5'UTR;5'UTR	S_Shore	36.07	7.03	42.44	7.17	-6.37	2.32E-06	9.82E-05
cg05012676	ZFPM1	16	Body	Island	40.83	5.01	47.20	5.95	-6.37	3.95E-06	1.61E-04
cg26978379	BACH2;BACH2	6	Body;Body	Open sea	58.61	6.49	64.98	6.60	-6.37	7.18E-05	2.12E-03
cg01674147	FHDC1	4	Body	Open sea	44.46	4.13	50.83	5.23	-6.37	3.89E-09	2.12E-07
cg22336337	ST18	8	5'UTR	Open sea	45.33	6.63	51.70	8.57	-6.37	3.62E-04	7.89E-03
cg27399125		6	Intergenic	N_Shore	57.93	8.31	64.30	8.07	-6.36	1.33E-03	2.11E-02
cg05239811	C6orf27	6	Body	Open sea	54.30	6.36	60.66	5.63	-6.36	7.31E-06	2.83E-04
cg19171161		17	Intergenic	N_Shelf	59.19	6.81	65.55	8.17	-6.36	2.43E-04	5.77E-03
cg18272307	RNF157	17	Body	Open sea	77.79	3.81	84.15	3.50	-6.36	2.00E-13	1.24E-11
cg23587980	KRT86	12	TSS200	N_Shore	29.21	6.96	35.57	8.25	-6.36	1.60E-04	4.14E-03
cg13049992	GRAMD3;GRAMD3;GRAMD3;GRAMD	5	Body;5'UTR;TSS200;Body;Body	Open sea	79.34	5.82	85.69	2.68	-6.36	5.93E-09	3.19E-07
cg02647835	ASPSCR1	17	Body	Island	68.17	5.68	74.52	6.84	-6.36	6.21E-05	1.88E-03
cg27244773	ARHGAP27;ARHGAP27;ARHGAP27	17	1stExon;5'UTR;5'UTR	Island	59.87	7.56	66.23	8.94	-6.36	8.23E-04	1.48E-02
cg11616411	MIR372;MIR371;MIR373	19	TSS1500;TSS200;TSS1500	Open sea	64.69	8.29	71.04	8.25	-6.35	7.43E-04	1.37E-02
cg13927135	PRR12	19	3'UTR	Open sea	76.34	4.55	82.69	3.58	-6.35	2.80E-10	1.60E-08
cg10717869	SLC41A1	1	5'UTR	N_Shore	31.08	7.04	37.43	7.22	-6.35	1.77E-04	4.49E-03
cg11672390	PRDM16;PRDM16	1	Body;Body	Island	41.97	5.82	48.32	6.77	-6.35	6.89E-05	2.05E-03
cg12627354	LRP4	11	TSS1500	S_Shore	39.26	8.16	45.61	7.62	-6.35	1.01E-03	1.72E-02
cg14065153	RARA;RARA;RARA;RARA	17	Body;Body;Body;Body	Open sea	50.09	5.07	56.44	4.97	-6.35	4.68E-07	2.19E-05
cg10827767	DRG1	22	TSS1500	N_Shore	55.34	6.51	61.68	6.71	-6.35	8.13E-05	2.35E-03
cg07665923	C7orf50;C7orf50;C7orf50	7	5'UTR;5'UTR;5'UTR	N_Shelf	70.02	8.45	76.37	7.68	-6.34	2.05E-03	2.87E-02
cg12695685	LMO2;LMO2;LMO2	11	Body;Body;Body	N_Shore	29.11	7.32	35.45	9.91	-6.34	2.27E-03	3.08E-02
cg26777475	PCOLCE;PCOLCE	7	1stExon;5'UTR	N_Shore	35.26	6.22	41.61	9.02	-6.34	2.77E-04	6.40E-03
cg16459412	LATS2	13	5'UTR	Open sea	78.23	6.77	84.57	5.69	-6.34	3.71E-05	1.20E-03
cg11930274	NUDT13	10	Body	Open sea	69.85	6.76	76.19	5.68	-6.34	8.70E-05	2.49E-03
cg25223634	C10orf26;C10orf26	10	Body;Body	Open sea	49.41	6.62	55.75	5.48	-6.34	1.78E-05	6.30E-04
cg14758757	SLC38A10;SLC38A10	17	Body;Body	N_Shelf	67.82	7.41	74.16	8.26	-6.34	2.10E-03	2.93E-02
cg08802933	VIL1	2	TSS1500	Open sea	60.24	4.45	66.58	4.32	-6.34	1.18E-08	6.24E-07
cg24492886	PTAFR;PTAFR;PTAFR;PTAFR	1	3'UTR;3'UTR;3'UTR;3'UTR	Open sea	44.85	4.88	51.18	4.01	-6.34	1.29E-09	7.16E-08
cg23085167	PRDM16;PRDM16	1	Body;Body	N_Shelf	38.27	5.36	44.60	8.26	-6.34	2.67E-05	9.01E-04
cg21958090		11	Intergenic	Open sea	38.66	6.27	45.00	7.26	-6.34	4.08E-05	1.30E-03
cg09141303	NHEDC2	4	TSS200	S_Shore	56.86	8.02	63.19	8.24	-6.33	1.36E-03	2.15E-02
cg15655495		12	Intergenic	Island	15.29	3.20	21.62	4.66	-6.33	8.03E-11	4.65E-09
cg05181279		11	Intergenic	Open sea	75.71	5.62	82.05	3.49	-6.33	1.72E-08	9.02E-07
cg05314394		15	Intergenic	N_Shelf	23.06	5.61	29.39	9.78	-6.33	4.01E-04	8.53E-03
cg01964525	EML6	2	Body	Open sea	46.62	6.92	52.95	7.81	-6.33	8.59E-04	1.53E-02
cg15123187		1	Intergenic	Island	78.23	7.38	84.56	5.12	-6.33	1.11E-04	3.05E-03
cg05341848	PTPLA	10	3'UTR	Open sea	65.82	7.14	72.15	7.26	-6.33	5.91E-05	1.80E-03
cg10317815		11	Intergenic	Open sea	70.70	7.22	77.04	7.20	-6.33	2.64E-06	1.11E-04
cg10984962	AGAP1;AGAP1	2	Body;Body	Open sea	66.35	6.96	72.68	4.73	-6.33	1.68E-05	6.00E-04
cg02113429	OVOL2	20	Body	N_Shore	10.48	5.32	16.81	9.93	-6.33	2.56E-04	6.01E-03
cg22454564		20	Intergenic	N_Shore	33.22	4.94	39.56	3.95	-6.33	6.28E-08	3.18E-06
cg27262236	RBM38;RBM38	20	TSS1500;TSS1500	Island	40.14	5.54	46.47	5.43	-6.33	3.88E-07	1.83E-05
cg00493341	MAST3	19	Body	Island	8.88	2.68	15.21	7.55	-6.33	1.29E-07	6.37E-06

cg01171885	GGH	8	Body	N_Shore	56.45	7.04	62.77	8.94	-6.33	2.86E-03	3.62E-02
cg10334703	MATN3	2	TSS1500	S_Shore	37.08	7.37	43.40	9.12	-6.33	4.12E-04	8.73E-03
cg11092487	MYH7B	20	Body	Island	55.64	5.53	61.97	4.71	-6.33	9.09E-07	4.11E-05
cg13962153	MAP7D1	1	Body	Island	55.90	6.12	62.23	9.57	-6.32	1.51E-03	2.31E-02
cg26128121	CACNB1	17	Body	Island	47.10	6.64	53.43	9.09	-6.32	1.36E-03	2.14E-02
cg01485157	VASN;CORO7	16	Body;Body	Open sea	63.03	7.52	69.35	8.52	-6.32	3.51E-03	4.17E-02
cg02370832	IFIT3;IFIT3	10	Body;Body	Open sea	54.78	6.20	61.10	6.28	-6.32	1.47E-06	6.45E-05
cg22191981		6	Intergenic	N_Shore	16.26	5.15	22.58	9.18	-6.32	1.74E-04	4.43E-03
cg06002288		18	Intergenic	S_Shelf	52.82	6.65	59.14	7.31	-6.32	2.64E-04	6.16E-03
cg01696784	SH3BP4	2	5'UTR	Open sea	62.00	8.62	68.32	6.63	-6.32	1.15E-04	3.14E-03
cg17583504	TRPM4	19	Body	Island	77.59	8.88	83.91	7.52	-6.32	2.21E-03	3.03E-02
cg07752120		5	Intergenic	Open sea	61.91	6.80	68.23	7.39	-6.32	3.16E-05	1.04E-03
cg07963046	GON4L;GON4L	1	Body;Body	Open sea	58.44	5.75	64.76	5.15	-6.32	1.64E-06	7.11E-05
cg18446744		6	Intergenic	S_Shelf	43.10	6.20	49.42	4.95	-6.31	2.36E-06	9.99E-05
cg26926765	C6orf142	6	Body	Open sea	61.18	6.52	67.50	4.49	-6.31	2.84E-06	1.18E-04
cg10537193	PISD	22	TSS200	S_Shore	29.26	7.03	35.57	7.56	-6.31	6.28E-05	1.89E-03
cg10171357		15	Intergenic	N_Shore	37.22	6.12	43.53	9.68	-6.31	3.29E-04	7.31E-03
cg03306486	APC2	19	Body	Island	14.36	6.50	20.67	9.73	-6.31	1.29E-04	3.45E-03
cg26672098		2	Intergenic	N_Shelf	38.43	4.09	44.74	4.48	-6.31	1.11E-08	5.92E-07
cg07904475	LRBA	4	Body	Island	32.17	5.97	38.49	5.46	-6.31	2.11E-06	9.01E-05
cg15039603	ITGB2;ITGB2	21	Body;Body	S_Shore	67.71	6.59	74.02	5.86	-6.31	4.31E-05	1.37E-03
cg20489228		12	Intergenic	S_Shore	33.98	4.96	40.29	4.30	-6.31	3.60E-09	1.96E-07
cg03714676	KIAA0146	8	Body	S_Shore	58.93	8.31	65.23	7.26	-6.31	3.60E-03	4.24E-02
cg02876925	11-Sep	4	TSS1500	Island	57.68	6.53	63.98	6.20	-6.31	3.95E-05	1.27E-03
cg17501774	ZNF697	1	5'UTR	Open sea	68.25	7.27	74.56	5.90	-6.31	4.92E-06	1.97E-04
cg04226002	ZBTB16;ZBTB16	11	Body;Body	N_Shore	66.48	8.37	72.78	7.36	-6.31	6.73E-04	1.27E-02
cg00034559	TMEM178;TMEM178	2	TSS1500;TSS1500	N_Shore	66.70	6.28	73.01	7.25	-6.31	1.80E-05	6.37E-04
cg08641881	PHYHIPL;PHYHIPL	10	Body;Body	S_Shore	69.90	6.21	76.20	5.93	-6.30	2.31E-06	9.82E-05
cg07540634	PLEK2	14	Body	N_Shore	65.89	6.13	72.20	6.22	-6.30	6.25E-05	1.89E-03
cg04635334		16	Intergenic	S_Shore	28.84	6.53	35.14	6.86	-6.30	2.83E-05	9.47E-04
cg15768901	LTBP4;LTBP4;LTBP4	19	Body;Body;Body	Island	26.72	6.58	33.02	7.62	-6.30	2.85E-04	6.54E-03
cg22879834	PER2	2	Body	Open sea	56.40	5.36	62.70	6.78	-6.30	5.64E-05	1.72E-03
cg23200634		11	Intergenic	Open sea	78.36	10.52	84.66	3.11	-6.30	3.10E-05	1.03E-03
cg01577029		12	Intergenic	S_Shore	48.00	7.71	54.30	5.93	-6.30	4.18E-04	8.82E-03
cg19698668	ZNF783	7	Body	Island	24.49	5.96	30.79	6.60	-6.30	2.49E-07	1.20E-05
cg14548858		2	Intergenic	Open sea	72.01	4.90	78.31	4.25	-6.30	2.90E-09	1.59E-07
cg09814127	TNFAIP2	14	Body	Island	41.48	6.75	47.77	9.64	-6.30	2.79E-03	3.56E-02
cg15276477		8	Intergenic	Open sea	87.69	14.71	93.99	1.87	-6.30	2.49E-03	3.29E-02
cg02117535		11	Intergenic	Open sea	66.43	6.20	72.73	4.97	-6.29	5.76E-09	3.10E-07
cg03464229	C7orf58;C7orf58	7	Body;Body	Open sea	39.58	5.27	45.88	4.97	-6.29	6.23E-07	2.87E-05
cg26107890	SLC12A8	3	Body	Island	40.19	13.06	46.48	10.71	-6.29	3.74E-03	4.36E-02
cg22515654	PEX14	1	Body	Open sea	35.10	6.46	41.39	6.61	-6.29	1.10E-05	4.11E-04
cg06689259	STAMBPL1	10	5'UTR	Open sea	76.62	4.53	82.91	4.81	-6.29	7.02E-08	3.54E-06
cg06360820		2	Intergenic	Island	83.85	13.24	90.14	10.05	-6.29	2.07E-03	2.89E-02
cg22613769	SMYD2	1	Body	Open sea	69.27	6.48	75.56	5.95	-6.29	3.05E-05	1.01E-03
cg02566259		4	Intergenic	Island	39.87	9.48	46.16	10.04	-6.29	3.34E-03	4.03E-02
cg02380585	SLFN13	17	TSS1500	Island	28.27	5.02	34.56	7.17	-6.29	2.26E-06	9.59E-05
cg00252032	PTK7;PTK7;PTK7;PTK7	6	TSS1500;TSS1500;TSS1500;TSS1500	N_Shore	19.66	5.79	25.95	5.98	-6.29	9.33E-07	4.20E-05
cg03846408	HCCA2;LOC402778	11	Body;TSS200	Island	37.65	7.14	43.94	7.24	-6.29	3.20E-04	7.16E-03
cg23282964	RIOK1;RIOK1	6	3'UTR;3'UTR	Open sea	64.40	7.40	70.69	7.55	-6.29	2.40E-03	3.21E-02
cg02059176		2	Intergenic	Open sea	63.06	8.64	69.34	5.84	-6.29	1.47E-05	5.33E-04
cg00808403	LOC100129354	3	TSS1500	S_Shore	68.84	5.06	75.13	5.64	-6.29	1.36E-06	5.98E-05
cg17876497	AGAP11	10	5'UTR	Island	18.65	5.53	24.93	8.52	-6.28	1.71E-04	4.36E-03

cg26318206	BRF1	14	Body	N_Shelf	73.98	6.81	80.26	5.77	-6.28	6.92E-05	2.05E-03
cg04771100	HTRA4	8	Body	Island	58.04	7.58	64.32	6.28	-6.28	5.89E-05	1.79E-03
cg25616829		1	Intergenic	S_Shore	80.86	7.41	87.14	5.54	-6.28	4.21E-05	1.34E-03
cg26211800	FAM50B	6	TSS1500	N_Shore	56.43	6.96	62.71	7.67	-6.28	8.14E-04	1.47E-02
cg09832911	ALOX5	10	Body	S_Shore	62.28	6.20	68.55	6.31	-6.28	1.70E-06	7.39E-05
cg02776664	MAST1	19	Body	Island	20.51	6.63	26.79	6.30	-6.28	1.50E-06	6.55E-05
cg16568727	TTBK1	6	Body	Island	15.23	6.36	21.50	8.17	-6.28	2.56E-04	6.02E-03
cg11525980	PC;PC;PC	11	5'UTR;5'UTR;5'UTR	Open sea	62.77	7.58	69.04	7.26	-6.28	6.73E-05	2.01E-03
cg18709166		6	Intergenic	N_Shelf	64.69	5.75	70.96	5.19	-6.28	2.04E-08	1.07E-06
cg27275698	CHRM5	15	Body	Open sea	72.49	4.84	78.76	5.41	-6.28	4.46E-07	2.10E-05
cg16784745	MIR148B;COPZ1	12	Body;Body	Open sea	57.25	7.10	63.52	5.87	-6.27	1.93E-07	9.39E-06
cg04006133	HSDL1;HSDL1	16	5'UTR;5'UTR	Open sea	66.36	4.92	72.63	5.46	-6.27	9.66E-09	5.15E-07
cg09785984		1	Intergenic	Island	36.26	5.16	42.53	6.40	-6.27	2.70E-06	1.13E-04
cg05395403	PDX1	13	Body	N_Shore	14.93	6.79	21.20	9.41	-6.27	1.43E-03	2.23E-02
cg07842386	IL23R	1	TSS200	Open sea	66.69	5.58	72.96	4.63	-6.27	2.01E-08	1.05E-06
cg19892525	ATM;ATM	11	Body;TSS1500	Open sea	54.42	6.43	60.69	6.20	-6.27	6.43E-06	2.51E-04
cg19460792		15	Intergenic	Open sea	49.38	7.93	55.65	6.45	-6.27	2.78E-04	6.41E-03
cg22801980	HNRNPH1	5	3'UTR	Open sea	51.21	6.09	57.47	5.00	-6.27	1.99E-05	6.95E-04
cg08721338	ELMO3	16	Body	S_Shore	58.77	4.91	65.03	5.02	-6.26	3.51E-07	1.67E-05
cg24510518	GLI4	8	Body	Island	20.13	6.97	26.39	7.08	-6.26	1.81E-05	6.40E-04
cg01812599		10	Intergenic	Island	31.27	5.38	37.54	7.62	-6.26	2.81E-05	9.43E-04
cg17468569	NOS1AP;NOS1AP	1	Body;Body	S_Shore	60.21	5.35	66.47	6.44	-6.26	2.02E-05	7.04E-04
cg26902557	DUSP8;HCCA2	11	Body;Body	Open sea	55.16	6.11	61.42	6.06	-6.26	6.13E-07	2.83E-05
cg13499717	C3orf55;C3orf55	3	Body;Body	Open sea	59.60	6.11	65.86	7.12	-6.26	7.85E-06	3.02E-04
cg23903129	GPR133	12	Body	Open sea	11.32	4.20	17.58	6.64	-6.26	6.74E-07	3.10E-05
cg02208302		14	Intergenic	Open sea	62.62	6.19	68.88	8.49	-6.26	2.46E-04	5.82E-03
cg12807187	EIF4E3;EIF4E3;EIF4E3	3	TSS200;5'UTR;5'UTR	S_Shelf	55.35	6.46	61.60	6.87	-6.26	1.86E-04	4.66E-03
cg01381829	TNRC6B;TNRC6B;TNRC6B	22	Body;TSS1500;TSS1500	Open sea	55.63	5.57	61.88	5.54	-6.26	9.13E-09	4.87E-07
cg05620261	WTIP	19	Body	S_Shelf	45.55	5.93	51.80	7.51	-6.26	2.35E-04	5.61E-03
cg15490703	EFNA2	19	Body	Island	29.89	4.27	36.14	5.65	-6.25	3.14E-08	1.62E-06
cg14663278	BGLAP	1	TSS1500	N_Shelf	71.39	5.60	77.65	3.96	-6.25	3.31E-07	1.58E-05
cg02797569	PCOLCE;PCOLCE	7	1stExon;5'UTR	N_Shore	27.32	5.18	33.57	9.04	-6.25	1.05E-04	2.92E-03
cg03872623	AHSG	3	3'UTR	Open sea	77.28	5.28	83.53	4.96	-6.25	1.25E-06	5.52E-05
cg00598553		16	Intergenic	Open sea	53.84	6.57	60.09	7.51	-6.25	1.79E-04	4.51E-03
cg00445518	PPP1R13L;PPP1R13L	19	Body;Body	Island	41.29	5.28	47.54	6.43	-6.25	7.82E-06	3.01E-04
cg03804240	PHLDB1;PHLDB1;PHLDB1	11	5'UTR;5'UTR;5'UTR	Island	12.07	5.40	18.31	9.54	-6.25	6.07E-04	1.18E-02
cg14666972	SEMA4B;SEMA4B	15	Body;Body	Island	38.18	7.51	44.43	8.64	-6.25	1.59E-04	4.11E-03
cg05511196	PDE7B	6	Body	Open sea	45.35	6.69	51.59	6.48	-6.24	6.92E-06	2.69E-04
cg00951395	KIAA1383	1	1stExon	S_Shore	15.76	4.10	22.00	5.67	-6.24	3.47E-07	1.65E-05
cg17782974	TRIM8	10	Body	S_Shelf	49.90	8.04	56.14	6.55	-6.24	1.70E-04	4.34E-03
cg15506894	ACSF2;CHAD	17	Body;1stExon	Island	66.70	8.04	72.94	9.05	-6.24	1.06E-03	1.79E-02
cg20031717	CYP2C19	10	Body	Open sea	64.48	9.31	70.72	7.59	-6.24	1.30E-03	2.08E-02
cg18628449	SHROOM3	4	Body	S_Shore	14.57	5.11	20.81	6.88	-6.24	3.13E-06	1.30E-04
cg15048099	C10orf93	10	Body	N_Shelf	29.60	5.94	35.83	10.78	-6.24	3.12E-03	3.85E-02
cg13004792		8	Intergenic	Open sea	50.69	5.28	56.93	6.16	-6.24	3.69E-06	1.51E-04
cg10095777	C6orf27	6	Body	Open sea	64.57	7.25	70.81	5.89	-6.24	4.24E-05	1.35E-03
cg23281527	KLHDC7A	1	1stExon	Island	86.09	9.03	92.33	5.20	-6.24	1.11E-03	1.85E-02
cg27261050	PIWIL1	12	TSS1500	N_Shore	39.63	6.04	45.87	6.94	-6.24	3.80E-05	1.23E-03
cg08216304	DPYSL2	8	Body	Open sea	51.00	5.55	57.23	5.17	-6.24	7.72E-07	3.52E-05
cg01176329	GABBR1;GABBR1;GABBR1	6	Body;Body;Body	N_Shelf	58.30	7.52	64.53	8.65	-6.23	1.80E-03	2.62E-02
cg19917989		6	Intergenic	Open sea	30.50	4.82	36.73	7.85	-6.23	7.10E-05	2.10E-03
cg05424970	FSCN3	7	TSS200	Open sea	47.93	5.22	54.16	6.06	-6.23	1.60E-05	5.74E-04
cg16732787	DUSP5P	1	Body	Island	51.91	7.06	58.14	8.53	-6.23	5.91E-04	1.15E-02

cg00863469	FAM83E	19	1stExon	Island	71.83	8.33	78.06	6.80	-6.23	3.16E-03	3.88E-02
cg18065174	SIRT6	19	Body	S_Shore	62.21	5.59	68.44	5.67	-6.23	1.64E-06	7.14E-05
cg05297739	CBR1	21	Body	S_Shore	63.76	7.99	69.99	7.85	-6.23	1.99E-03	2.81E-02
cg14301764	EIF2C2;EIF2C2	8	Body;Body	Open sea	69.14	5.69	75.37	4.97	-6.23	3.34E-06	1.38E-04
cg20698113	PIM3	22	3'UTR	Island	40.61	6.32	46.84	8.67	-6.23	8.54E-04	1.52E-02
cg07472953	CSF3;CSF3;CSF3	17	TSS1500;TSS1500;TSS1500	Open sea	47.37	5.76	53.59	6.23	-6.23	8.78E-06	3.35E-04
cg17852224	MAPK8IP2;MAPK8IP2	22	Body;Body	Island	26.14	4.59	32.36	6.30	-6.23	5.38E-07	2.50E-05
cg21620968	COPS7B	2	3'UTR	Open sea	76.67	6.69	82.89	5.63	-6.23	2.26E-06	9.60E-05
cg07793207	LGI1	10	TSS200	Open sea	41.14	7.38	47.36	8.55	-6.23	2.08E-04	5.09E-03
cg06566627		2	Intergenic	S_Shore	70.08	4.66	76.31	3.60	-6.23	9.46E-09	5.05E-07
cg20668644	RAPGEFL1	17	Body	Island	3.84	2.55	10.07	7.46	-6.23	1.15E-07	5.69E-06
cg02579509	LOC286002;SLC26A4	7	TSS1500;Body	S_Shore	43.86	5.39	50.08	7.33	-6.22	1.89E-06	8.14E-05
cg19657214	DEFA4	8	5'UTR	Open sea	61.36	5.20	67.58	5.15	-6.22	6.88E-07	3.16E-05
cg17612569	GABPA;ATP5J;ATP5J;ATP5J;ATP5J;ATF	21	TSS200;5'UTR;5'UTR;1stExon;Body;5'UTR;	Island	4.16	0.73	10.38	1.86	-6.22	2.44E-42	2.74E-40
cg00348389	TRIM72	16	Body	Island	48.81	5.04	55.03	6.78	-6.22	2.57E-06	1.08E-04
cg00685622		16	Intergenic	Open sea	69.87	6.92	76.09	5.85	-6.22	3.12E-05	1.03E-03
cg24206827		2	Intergenic	Open sea	45.30	6.31	51.52	7.49	-6.22	5.87E-05	1.79E-03
cg27552857	SEMA6B	19	3'UTR	N_Shore	46.70	4.74	52.92	6.22	-6.22	2.44E-06	1.03E-04
cg06818605	ZNF783	7	Body	Island	12.30	3.65	18.52	6.13	-6.22	2.41E-10	1.37E-08
cg04370442	TEPP;TEPP	16	Body;Body	Island	59.89	6.71	66.11	10.07	-6.22	4.47E-04	9.29E-03
cg07465899		4	Intergenic	N_Shore	50.39	4.71	56.61	5.60	-6.22	1.11E-06	4.95E-05
cg00870778	MGC72080	7	Body	N_Shelf	72.11	6.36	78.33	4.95	-6.22	2.23E-05	7.69E-04
cg01682111	UNKL	16	TSS1500	Island	32.94	6.54	39.16	8.56	-6.22	5.08E-04	1.03E-02
cg07475178	LDHD;LDHD	16	Body;Body	S_Shore	53.86	6.44	60.08	10.32	-6.21	3.20E-03	3.92E-02
cg11873311	UGGT1;UGGT1	2	Body;3'UTR	Open sea	34.09	6.09	40.30	6.94	-6.21	7.79E-05	2.27E-03
cg08926365	FYN;FYN;FYN	6	Body;Body;Body	Open sea	61.48	7.83	67.69	9.75	-6.21	3.88E-03	4.47E-02
cg12242450	GCNT2;GCNT2	6	TSS200;Body	Open sea	39.47	5.01	45.68	5.98	-6.21	7.02E-06	2.73E-04
cg25666660	FLJ25363	3	TSS200	Open sea	82.78	2.98	88.99	2.49	-6.21	2.11E-17	1.44E-15
cg05890620	ICAM4;ICAM4;ICAM4	19	TSS200;TSS200;TSS200	Island	28.61	6.34	34.82	5.93	-6.21	7.10E-07	3.25E-05
cg02927346	RASL10B	17	5'UTR	S_Shore	31.12	4.73	37.32	4.86	-6.21	2.36E-07	1.14E-05
cg21157690	ESR1;ESR1;ESR1	6	5'UTR;1stExon;5'UTR	N_Shore	55.53	6.36	61.73	6.86	-6.20	2.59E-05	8.79E-04
cg21023770	C20orf165	20	TSS1500	N_Shore	67.17	3.92	73.37	3.87	-6.20	1.63E-10	9.38E-09
cg26961824	SGOL2;SGOL2	2	3'UTR;3'UTR	N_Shore	72.44	7.03	78.64	5.95	-6.20	4.67E-05	1.47E-03
cg16300556		5	Intergenic	S_Shelf	56.89	9.59	63.10	9.39	-6.20	2.52E-03	3.32E-02
cg05129325	KCNK2;KCNK2;KCNK2	1	TSS1500;TSS1500;Body	N_Shore	16.49	4.44	22.69	7.27	-6.20	7.94E-06	3.05E-04
cg16477492	C11orf95	11	3'UTR	Island	30.24	6.28	36.44	9.50	-6.20	1.72E-03	2.53E-02
cg00723907	STK25	2	3'UTR	Open sea	60.15	5.13	66.35	5.78	-6.20	4.67E-07	2.19E-05
cg11876177	SLC17A3;SLC17A3	6	5'UTR;5'UTR	Open sea	74.77	7.41	80.97	5.92	-6.20	2.38E-04	5.68E-03
cg03479065		3	Intergenic	N_Shore	37.86	6.36	44.06	7.92	-6.20	2.10E-04	5.12E-03
cg10792987	ELL	19	Body	S_Shelf	28.80	5.69	34.99	7.23	-6.20	1.08E-04	3.00E-03
cg10819560		15	Intergenic	Open sea	63.41	5.01	69.61	4.67	-6.20	3.28E-07	1.56E-05
cg17493885	NSD1;NSD1	5	TSS1500;TSS1500	Island	39.12	7.33	45.32	8.76	-6.20	4.42E-04	9.21E-03
cg09732335	MAP3K5	6	Body	Open sea	52.02	7.80	58.21	8.35	-6.19	2.03E-03	2.86E-02
cg25013978	MIR580;LMBRD2	5	TSS1500;5'UTR	N_Shelf	68.92	5.41	75.12	5.19	-6.19	3.27E-08	1.69E-06
cg26326372	CRIP2	14	3'UTR	S_Shore	40.36	3.88	46.55	4.38	-6.19	2.17E-09	1.20E-07
cg06420129	TNRC6A	16	Body	Open sea	59.29	6.05	65.48	6.80	-6.19	4.56E-05	1.44E-03
cg08761909	PLCE1	10	Body	Open sea	64.62	6.61	70.81	5.83	-6.19	3.80E-05	1.23E-03
cg18595639	H6PD	1	Body	Island	64.61	6.25	70.80	6.74	-6.19	3.61E-04	7.86E-03
cg03358636	KIAA0226	3	5'UTR	N_Shelf	34.36	7.08	40.55	8.10	-6.19	1.85E-03	2.68E-02
cg26493224	THSD1;THSD1	13	TSS200;TSS200	S_Shore	28.05	5.85	34.24	6.04	-6.19	1.24E-05	4.56E-04
cg14430151	ACOX3;ACOX3	4	5'UTR;5'UTR	N_Shore	76.15	7.55	82.34	5.27	-6.19	2.18E-05	7.55E-04
cg26331226		12	Intergenic	Open sea	62.61	7.25	68.80	7.44	-6.19	1.30E-03	2.08E-02
cg08549332	CUX1;CUX1;CUX1	7	Body;Body;Body	N_Shore	42.75	4.81	48.94	6.45	-6.19	4.17E-06	1.70E-04

cg03701266	ANKRD33B	5	Body	Island	47.48	5.04	53.67	6.36	-6.19	3.17E-06	1.31E-04
cg02188185	RNF39;RNF39	6	Body;Body	S_Shore	35.19	5.57	41.37	7.55	-6.19	4.74E-05	1.48E-03
cg22930808	PARP9;PARP9;DTX3L;PARP9;PARP9;P/	3	5'UTR;5'UTR;TSS1500;5'UTR;5'UTR;5'UTR;	N_Shore	45.32	9.20	51.51	8.89	-6.19	4.10E-03	4.64E-02
cg14942906		11	Intergenic	S_Shore	53.74	7.12	59.92	6.24	-6.19	5.65E-04	1.11E-02
cg08808720	KIF5C;MIR1978	2	Body;TSS1500	Open sea	40.40	5.37	46.59	7.84	-6.19	4.29E-05	1.36E-03
cg08562004	AOC3	17	3'UTR	Open sea	59.32	6.36	65.51	7.16	-6.19	1.01E-05	3.82E-04
cg15477500	ZDHHC3;ZDHHC3;ZDHHC3	3	3'UTR;3'UTR;3'UTR	Open sea	39.62	4.42	45.80	4.25	-6.19	2.51E-08	1.31E-06
cg19571293	CNN2;CNN2	19	Body;Body	Island	23.24	8.43	29.42	9.10	-6.19	8.21E-04	1.47E-02
cg01851632	AAMP	2	Body	N_Shelf	51.87	3.61	58.05	5.71	-6.19	5.91E-08	3.00E-06
cg09065714	UCN	2	TSS1500	S_Shore	61.55	7.14	67.74	6.84	-6.19	1.30E-04	3.46E-03
cg03813964		2	Intergenic	N_Shelf	75.69	5.03	81.88	4.41	-6.19	3.55E-09	1.93E-07
cg03713186		7	Intergenic	N_Shelf	74.17	4.96	80.36	3.56	-6.19	2.83E-08	1.47E-06
cg00167841		6	Intergenic	S_Shore	16.67	5.20	22.86	10.05	-6.18	1.07E-03	1.80E-02
cg12668649		3	Intergenic	Open sea	59.04	6.08	65.22	5.98	-6.18	1.33E-05	4.88E-04
cg05221370	LRRN3;IMMP2L;LRRN3;LRRN3	7	5'UTR;Body;5'UTR;5'UTR	Open sea	31.50	6.92	37.68	6.65	-6.18	8.93E-05	2.55E-03
cg18246610	SEMA3A	7	Body	Open sea	85.26	11.46	91.44	8.77	-6.18	1.84E-03	2.67E-02
cg04562491	DTX1	12	TSS1500	Island	37.49	6.18	43.67	6.60	-6.18	1.33E-04	3.54E-03
cg02519751	ZIC1	3	TSS1500	N_Shore	24.10	6.07	30.27	7.40	-6.18	1.56E-05	5.60E-04
cg00582897		5	Intergenic	N_Shore	58.39	10.97	64.56	7.93	-6.17	1.11E-03	1.85E-02
cg22793067		5	Intergenic	Open sea	74.37	9.79	80.54	7.30	-6.17	9.39E-04	1.63E-02
cg06874403		10	Intergenic	Island	45.64	5.48	51.82	8.47	-6.17	1.16E-04	3.16E-03
cg04203508	ATF3	1	5'UTR	N_Shore	22.56	6.62	28.73	8.29	-6.17	1.89E-05	6.64E-04
cg00802237	SERPINA1;SERPINA1;SERPINA1;SERPII	14	TSS1500;TSS1500;TSS1500;TSS1500;TSS15	Open sea	55.40	4.63	61.57	3.21	-6.17	2.68E-08	1.39E-06
cg12599168	EPHB6	7	5'UTR	S_Shore	53.92	6.16	60.09	5.92	-6.17	1.89E-05	6.63E-04
cg18201070	OCA2	15	Body	Island	73.72	7.00	79.88	6.45	-6.17	1.73E-04	4.41E-03
cg01293346	TAS2R20;TAS2R20;PRR4;PRH1	12	3'UTR;1stExon;Body;5'UTR	Open sea	65.27	9.48	71.44	8.31	-6.17	1.64E-03	2.45E-02
cg11965311	LOC145845	15	TSS200	N_Shore	66.24	7.12	72.40	5.79	-6.17	3.64E-05	1.18E-03
cg14201387		1	Intergenic	Open sea	17.76	4.88	23.92	8.46	-6.16	1.11E-05	4.13E-04
cg12818699	FAM65B;FAM65B;FAM65B	6	5'UTR;1stExon;5'UTR	Open sea	54.05	6.17	60.21	10.01	-6.16	1.22E-03	1.99E-02
cg11586189	SHISA4;SHISA4	1	TSS1500;TSS1500	Open sea	33.95	10.27	40.12	11.71	-6.16	4.24E-03	4.74E-02
cg13250285	GNAT1;GNAT1	3	TSS200;TSS200	N_Shore	46.01	5.32	52.17	7.23	-6.16	7.31E-05	2.15E-03
cg22857604	RASSF5;RASSF5;RASSF5;RASSF5	1	Body;5'UTR;Body;1stExon	Island	29.58	6.89	35.74	8.00	-6.16	5.51E-05	1.69E-03
cg01006802	TMPRSS8	16	TSS200	Island	31.87	8.16	38.03	9.35	-6.16	2.62E-03	3.41E-02
cg16929959	TNFSF13B;TNFSF13B	13	Body;Body	Open sea	62.65	4.87	68.81	5.09	-6.16	4.59E-07	2.15E-05
cg06494642	ELMO3	16	Body	Island	67.28	5.73	73.44	5.55	-6.16	2.25E-06	9.56E-05
cg10956857	PRDM16;PRDM16	1	Body;Body	N_Shore	63.86	7.71	70.02	6.96	-6.16	2.37E-03	3.19E-02
cg20672549	IKZF4	12	3'UTR	N_Shelf	57.10	5.86	63.26	5.32	-6.16	1.54E-05	5.53E-04
cg10628617	SCUBE1	22	Body	S_Shelf	12.41	4.29	18.57	7.57	-6.16	1.57E-06	6.82E-05
cg08282037	EPS8L1;EPS8L1	19	Body;Body	N_Shore	62.41	7.32	68.56	8.15	-6.16	8.76E-04	1.55E-02
cg26186549		6	Intergenic	S_Shore	58.83	9.90	64.99	9.79	-6.16	3.98E-03	4.55E-02
cg20970205		17	Intergenic	Island	30.18	5.74	36.33	6.85	-6.15	1.42E-04	3.75E-03
cg27475923	C11orf95	11	Body	Island	47.54	5.65	53.69	8.07	-6.15	1.81E-04	4.57E-03
cg22594071	PLSCR5;PLSCR5	3	5'UTR;1stExon	Open sea	46.02	5.05	52.18	5.16	-6.15	8.43E-07	3.82E-05
cg18373855		6	Intergenic	N_Shore	47.06	7.20	53.21	7.14	-6.15	4.53E-04	9.39E-03
cg20223237	HLA-DPB1	6	Body	Island	42.79	7.27	48.94	6.63	-6.15	4.95E-04	1.00E-02
cg02675527	PAX8;PAX8;PAX8;PAX8;PAX8;LOC4408	2	Body;Body;Body;Body;Body;Body	N_Shelf	39.81	11.50	45.97	12.20	-6.15	2.73E-03	3.51E-02
cg12011522	OSBPL6;OSBPL6	2	Body;Body	Open sea	30.07	5.53	36.22	7.16	-6.15	1.11E-04	3.06E-03
cg17143291	CACNA1E	1	Body	S_Shelf	43.80	9.37	49.95	7.51	-6.15	1.75E-03	2.57E-02
cg13766864	KCNE3	11	5'UTR	Open sea	67.69	6.73	73.84	6.53	-6.15	5.08E-05	1.58E-03
cg09579413	RUSC1;FDPS;FDPS;RUSC1;FDPS	1	TSS1500;Body;Body;TSS1500;Body	N_Shore	72.71	2.96	78.86	2.70	-6.15	8.57E-16	5.64E-14
cg12427469	KCNQ1;KCNQ1	11	Body;Body	N_Shelf	75.88	7.48	82.03	6.10	-6.15	1.63E-04	4.19E-03
cg18571640		8	Intergenic	Open sea	48.78	6.99	54.92	5.13	-6.15	7.32E-06	2.83E-04
cg16535667	SIPA1;SIPA1	11	Body;Body	Island	70.49	5.76	76.63	5.09	-6.15	5.04E-06	2.01E-04

cg14871138	NTSR1	20	1stExon	Island	60.58	6.33	66.73	6.07	-6.15	5.35E-05	1.65E-03
cg22312494	WNT11	11	TSS1500	N_Shore	29.95	7.91	36.10	9.21	-6.15	3.56E-04	7.78E-03
cg23530266	SOX6;SOX6;SOX6;SOX6	11	Body;Body;Body;Body	Open sea	35.97	7.94	42.11	9.81	-6.15	1.35E-03	2.14E-02
cg02468966	PMEPA1	20	TSS1500	S_Shore	50.53	6.88	56.67	7.71	-6.14	1.08E-03	1.81E-02
cg15961466	PDX1	13	3'UTR	Island	11.93	5.32	18.08	9.23	-6.14	9.94E-04	1.70E-02
cg11376198	AKR7L;AKR7L	1	TSS200;TSS200	Island	14.48	6.55	20.62	9.38	-6.14	3.11E-03	3.84E-02
cg16386697	CPT1B;CPT1B;CPT1B;CPT1B;CHKB-CPT	22	TSS200;TSS200;TSS1500;TSS1500;Body;TS	S_Shore	44.23	6.54	50.37	7.18	-6.14	1.39E-04	3.67E-03
cg14053590	EPO	7	3'UTR	S_Shelf	59.39	4.75	65.53	4.84	-6.14	3.61E-07	1.71E-05
cg20473420		12	Intergenic	Open sea	29.19	4.68	35.33	5.04	-6.14	3.09E-07	1.48E-05
cg06878787	CTNS;CTNS	17	3'UTR;3'UTR	Open sea	66.38	8.05	72.52	7.79	-6.14	1.70E-03	2.51E-02
cg05197667	SRGAP1	12	Body	Open sea	75.97	8.14	82.11	5.71	-6.14	9.50E-06	3.60E-04
cg16725305		2	Intergenic	Open sea	37.85	6.97	43.99	6.18	-6.14	1.29E-04	3.46E-03
cg07637741	SYTL3	6	Body	Open sea	68.02	5.55	74.15	5.78	-6.13	3.16E-06	1.31E-04
cg01636599	FGF22	19	TSS1500	Island	40.28	6.77	46.41	7.14	-6.13	1.23E-04	3.33E-03
cg04359828	ARHGAP12	10	5'UTR	N_Shore	36.08	6.89	42.22	6.88	-6.13	1.40E-05	5.09E-04
cg09847368	GALNT2	1	Body	Open sea	76.61	7.66	82.74	6.38	-6.13	2.36E-04	5.63E-03
cg19782746	SCAPER;SCAPER	15	Body;Body	Open sea	77.12	6.34	83.25	5.42	-6.13	5.84E-06	2.31E-04
cg14908875	ALKBH3	11	TSS1500	N_Shore	68.16	7.40	74.28	5.70	-6.13	3.00E-04	6.81E-03
cg03409272	CCL14-CCL15;CCL15;CCL14-CCL15	17	TSS1500;TSS1500;TSS1500	Open sea	74.80	6.18	80.92	6.57	-6.12	1.01E-04	2.82E-03
cg15644756		12	Intergenic	N_Shelf	31.07	4.47	37.19	8.83	-6.12	1.90E-04	4.74E-03
cg23684316	EXOC6B	2	Body	Open sea	42.35	5.60	48.48	5.71	-6.12	3.69E-08	1.90E-06
cg02294870		17	Intergenic	S_Shelf	48.11	6.72	54.23	8.74	-6.12	3.19E-03	3.91E-02
cg01078582	DSCAM	21	Body	N_Shore	41.78	7.14	47.90	8.11	-6.12	1.07E-03	1.80E-02
cg25601879		10	Intergenic	Open sea	68.61	7.39	74.73	6.33	-6.12	5.21E-05	1.61E-03
cg05687149	IL18	11	TSS1500	Open sea	40.22	7.49	46.33	6.04	-6.12	2.15E-04	5.23E-03
cg03169041	A2BP1;A2BP1	16	5'UTR;5'UTR	Island	16.61	7.69	22.72	10.58	-6.11	2.01E-03	2.84E-02
cg19619909	ALDH1A2;ALDH1A2;ALDH1A2	15	Body;Body;TSS1500	Open sea	60.63	7.69	66.74	7.85	-6.11	3.47E-04	7.62E-03
cg09476997	SLC9A3R2;SLC9A3R2	16	Body;Body	N_Shore	29.34	6.14	35.45	8.63	-6.11	1.31E-03	2.09E-02
cg22375856	TBC1D5;TBC1D5;TBC1D5	3	Body;Body;Body	Open sea	78.19	7.99	84.31	5.36	-6.11	1.51E-03	2.31E-02
cg23924370		2	Intergenic	Island	50.24	6.30	56.35	7.34	-6.11	2.33E-04	5.58E-03
cg27519622	CCDC64B	16	Body	Island	29.74	7.01	35.85	10.85	-6.11	3.56E-03	4.22E-02
cg00786237		3	Intergenic	S_Shelf	48.64	4.89	54.75	5.24	-6.11	6.39E-09	3.44E-07
cg25960090		5	Intergenic	Open sea	55.85	5.23	61.96	6.83	-6.11	3.56E-06	1.46E-04
cg11237362	ABCD3;ABCD3	1	Body;Body	S_Shelf	62.86	7.14	68.97	7.07	-6.11	1.29E-04	3.44E-03
cg27179622	LEFTY2	1	Body	Island	41.51	8.22	47.62	7.62	-6.11	3.21E-04	7.17E-03
cg21648823	ING5	2	3'UTR	S_Shelf	64.17	6.34	70.28	5.19	-6.11	2.23E-05	7.70E-04
cg12581627	PCDH20	13	Body	Open sea	21.80	4.85	27.91	7.00	-6.11	8.33E-08	4.18E-06
cg01188509	IGF2R	6	Body	N_Shelf	50.74	7.48	56.85	6.72	-6.11	8.05E-04	1.45E-02
cg02287371	SLC6A18	5	3'UTR	Island	15.00	6.36	21.11	7.70	-6.11	3.91E-04	8.37E-03
cg19494818	MPP4	2	5'UTR	Open sea	58.52	5.48	64.62	4.48	-6.11	3.48E-06	1.43E-04
cg01069965	B4GALT5	20	TSS1500	S_Shore	35.27	6.46	41.38	6.45	-6.11	4.25E-05	1.35E-03
cg04143876		15	Intergenic	Island	17.95	4.13	24.06	5.73	-6.11	1.32E-08	6.97E-07
cg12419334	WWC2	4	Body	Open sea	50.94	5.34	57.05	4.79	-6.11	1.17E-06	5.21E-05
cg20156793		12	Intergenic	Open sea	59.70	6.40	65.80	5.66	-6.11	1.81E-05	6.39E-04
cg03547581	NUCKS1	1	3'UTR	Open sea	44.54	5.48	50.65	6.29	-6.11	4.92E-05	1.53E-03
cg26472636	SDHAP3	5	Body	Island	22.59	7.13	28.69	9.59	-6.11	6.86E-04	1.29E-02
cg00789793	RASA2	3	Body	Open sea	76.13	7.43	82.23	6.15	-6.10	1.27E-04	3.40E-03
cg27452341	CLCN1	7	Body	Island	14.47	5.31	20.57	8.11	-6.10	1.03E-06	4.60E-05
cg19502207	GCH1;GCH1;GCH1;GCH1	14	Body;Body;Body;Body	Open sea	50.49	7.83	56.59	8.11	-6.10	1.46E-04	3.82E-03
cg26799250	PITPNM3;PITPNM3	17	3'UTR;3'UTR	Island	54.83	5.95	60.94	8.29	-6.10	1.90E-04	4.74E-03
cg06578561	FAM135B	8	5'UTR	N_Shore	48.71	6.01	54.81	5.17	-6.10	2.58E-06	1.08E-04
cg26592076	INPP4B;INPP4B	4	Body;Body	Open sea	64.39	6.01	70.49	7.20	-6.10	3.98E-05	1.27E-03
cg12075202	ACOX2	3	Body	Open sea	48.41	5.64	54.51	8.00	-6.10	2.66E-04	6.20E-03

cg09267427	NCOR2;NCOR2	12	Body;Body	S_Shelf	31.21	5.04	37.31	6.41	-6.10	4.24E-06	1.72E-04
cg08351085	GRTP1	13	Body	S_Shelf	74.98	7.15	81.08	5.73	-6.10	1.55E-04	4.03E-03
cg22991936	AKT1;AKT1;AKT1	14	Body;Body;Body	S_Shelf	47.40	4.74	53.50	5.74	-6.10	4.53E-06	1.83E-04
cg09972836	COL19A1	6	Body	Open sea	75.25	9.00	81.35	7.46	-6.10	1.32E-03	2.10E-02
cg07938252	NTSR1	20	1stExon	Island	53.99	5.33	60.09	7.18	-6.10	6.68E-05	1.99E-03
cg06005876	VAMP5	2	Body	Open sea	44.61	6.06	50.71	6.92	-6.10	9.99E-07	4.49E-05
cg19888017		6	Intergenic	Island	43.51	6.10	49.61	5.57	-6.10	2.06E-05	7.16E-04
cg27597392		12	Intergenic	Open sea	30.74	3.62	36.83	5.33	-6.10	2.69E-08	1.40E-06
cg05354971		2	Intergenic	Island	37.76	6.45	43.85	7.37	-6.10	1.87E-04	4.69E-03
cg01114163		5	Intergenic	N_Shore	73.63	11.50	79.73	12.84	-6.09	3.99E-03	4.56E-02
cg11552025	SLC22A23;SLC22A23	6	Body;Body	Open sea	47.77	6.80	53.86	8.90	-6.09	1.83E-03	2.66E-02
cg24636244	PFKFB3	10	Body	Open sea	58.80	7.81	64.89	8.17	-6.09	1.64E-03	2.45E-02
cg17330093		6	Intergenic	Open sea	57.16	5.56	63.25	5.85	-6.09	2.75E-07	1.32E-05
cg19459332		15	Intergenic	Island	25.49	8.64	31.58	8.14	-6.09	5.03E-04	1.02E-02
cg00212786		2	Intergenic	N_Shore	72.02	4.18	78.11	3.95	-6.09	1.92E-09	1.06E-07
cg01347228	RNF135;RNF135	17	TSS1500;TSS1500	N_Shore	46.94	6.27	53.03	8.12	-6.09	1.10E-04	3.02E-03
cg25094972	ARFGAP1;ARFGAP1	20	3'UTR;3'UTR	S_Shore	57.79	4.81	63.88	5.65	-6.09	2.92E-06	1.22E-04
cg00832924	CTTNBP2	7	Body	Open sea	38.65	7.65	44.74	8.23	-6.09	7.49E-04	1.38E-02
cg02534363	NBEAL2	3	3'UTR	Island	11.43	4.07	17.52	8.83	-6.09	1.50E-04	3.91E-03
cg07584981	CSF3;CSF3;CSF3	17	Body;Body;Body	Open sea	81.18	5.38	87.26	3.22	-6.09	2.67E-08	1.39E-06
cg06302295	GOT2	16	Body	N_Shelf	49.47	7.22	55.55	7.61	-6.09	1.92E-03	2.74E-02
cg17502267	BNIP2	15	Body	Island	24.16	4.44	30.24	5.97	-6.08	1.05E-06	4.72E-05
cg13973436	ZDHHC1;TPPP3;TPPP3	16	Body;TSS1500;TSS1500	Island	39.56	4.95	45.64	6.24	-6.08	7.03E-06	2.73E-04
cg22117498		4	Intergenic	Open sea	41.93	5.36	48.01	5.54	-6.08	1.13E-05	4.20E-04
cg01327602	SHISA7	19	Body	Island	43.54	5.96	49.62	5.16	-6.08	1.12E-06	5.00E-05
cg04956801		10	Intergenic	Island	45.31	6.74	51.39	9.53	-6.08	2.63E-03	3.42E-02
cg02484774		19	Intergenic	Island	62.67	6.89	68.75	8.85	-6.08	1.23E-03	2.00E-02
cg22429911	RAI14;RAI14;RAI14;RAI14;RAI14;RAI1	5	TSS1500;5'UTR;5'UTR;5'UTR;5'UTR;5'UTR	Open sea	80.94	5.46	87.02	4.42	-6.08	2.46E-07	1.19E-05
cg16900964	NFATC1;NFATC1;NFATC1;NFATC1	18	Body;Body;Body;Body	Island	45.00	4.73	51.07	5.89	-6.08	3.36E-06	1.38E-04
cg04122019	HR;HR	8	Body;Body	N_Shelf	66.95	9.16	73.02	8.19	-6.08	3.97E-03	4.54E-02
cg25832305	COL18A1	21	Body	S_Shore	34.69	7.47	40.76	7.88	-6.08	2.25E-03	3.07E-02
cg05875302		2	Intergenic	Island	52.17	8.51	58.25	8.29	-6.07	4.51E-04	9.36E-03
cg03598731		8	Intergenic	Island	50.75	6.63	56.82	9.52	-6.07	2.60E-03	3.39E-02
cg00260201	PRR15L	17	3'UTR	Open sea	45.91	6.14	51.98	6.67	-6.07	6.20E-05	1.87E-03
cg11418663	AGMAT	1	Body	N_Shore	52.45	7.99	58.52	7.32	-6.07	2.23E-03	3.05E-02
cg00027570	CD2	1	Body	Open sea	41.11	5.36	47.19	6.36	-6.07	1.22E-06	5.39E-05
cg01546793	SIK3	11	Body	Open sea	62.31	5.94	68.38	6.00	-6.07	1.93E-05	6.76E-04
cg18028470	PIGV	1	5'UTR	S_Shelf	49.68	5.16	55.75	5.14	-6.07	5.90E-06	2.33E-04
cg16109731	DUSP10;DUSP10;DUSP10	1	5'UTR;Body;5'UTR	Open sea	71.59	5.19	77.66	7.07	-6.07	4.03E-05	1.29E-03
cg26478582	TOX2;TOX2;TOX2;TOX2	20	5'UTR;TSS1500;Body;5'UTR	Open sea	74.95	6.19	81.02	5.46	-6.07	3.20E-05	1.05E-03
cg05738470	SOLH	16	3'UTR	N_Shore	75.30	5.41	81.37	4.39	-6.07	1.18E-06	5.25E-05
cg21551271		16	Intergenic	Island	47.70	7.38	53.77	7.25	-6.07	5.55E-04	1.10E-02
cg06495763	SLC22A18;SLC22A18;SLC22A18AS	11	Body;Body;TSS1500	S_Shore	59.86	9.24	65.93	7.89	-6.07	3.21E-04	7.17E-03
cg03886898	DIO1;DIO1;DIO1;DIO1	1	TSS1500;TSS1500;TSS1500;TSS1500	S_Shelf	62.40	6.54	68.47	8.21	-6.07	1.28E-03	2.05E-02
cg12009398	VIPR2	7	Body	Island	69.54	5.81	75.61	5.44	-6.07	9.07E-06	3.45E-04
cg10532980	MAD1L1;MAD1L1;MAD1L1	7	Body;Body;Body	S_Shore	72.23	6.18	78.30	5.00	-6.07	1.06E-05	3.98E-04
cg03046812	SLC41A1	1	5'UTR	N_Shore	23.83	8.79	29.89	8.28	-6.06	7.41E-04	1.37E-02
cg04050941	DLGAP2	8	5'UTR	Open sea	45.12	6.11	51.19	5.92	-6.06	5.51E-06	2.18E-04
cg24188163	IDO1	8	Body	Open sea	35.40	5.18	41.46	5.57	-6.06	3.74E-06	1.53E-04
cg14750551	PARP14	3	Body	S_Shore	78.37	5.96	84.44	3.90	-6.06	2.22E-06	9.47E-05
cg05975881		16	Intergenic	Open sea	50.25	5.94	56.31	5.71	-6.06	3.06E-05	1.02E-03
cg01820273	C6orf127	6	Body	Island	51.99	8.21	58.05	7.43	-6.06	4.40E-04	9.17E-03
cg21452471	TTYH3	7	3'UTR	Island	75.91	9.53	81.97	7.79	-6.06	2.17E-03	2.99E-02

cg11736544	HDAC5;HDAC5	17	5'UTR;5'UTR	N_Shelf	54.19	5.24	60.26	4.46	-6.06	1.16E-06	5.15E-05
cg26432869		8	Intergenic	Open sea	45.53	6.05	51.59	5.30	-6.06	8.78E-06	3.35E-04
cg06271720		2	Intergenic	Open sea	65.33	7.11	71.38	7.32	-6.06	1.57E-04	4.06E-03
cg00781795	NEDD4L;NEDD4L;NEDD4L;NEDD4L;NE	18	5'UTR;TSS1500;TSS1500;Body;TSS1500;Bo	Open sea	49.04	4.78	55.10	4.70	-6.06	5.53E-07	2.57E-05
cg17718377	DTX1	12	TSS200	S_Shore	39.27	5.73	45.33	7.62	-6.06	4.96E-04	1.01E-02
cg12081325	EIF2B3;EIF2B3	1	Body;Body	Open sea	45.67	7.15	51.72	6.32	-6.06	1.41E-04	3.72E-03
cg02304353		17	Intergenic	S_Shelf	70.85	4.42	76.91	4.20	-6.06	3.15E-09	1.72E-07
cg23246769	ZNF664	12	5'UTR	S_Shelf	54.24	5.40	60.29	7.00	-6.06	1.15E-04	3.14E-03
cg25588725	CLIC1	6	Body	Open sea	24.55	8.53	30.60	9.23	-6.06	1.57E-04	4.07E-03
cg01511480	SCNN1A;SCNN1A;SCNN1A	12	Body;Body;Body	N_Shore	58.45	6.17	64.51	7.00	-6.06	1.21E-05	4.48E-04
cg26137103		4	Intergenic	Open sea	53.97	5.42	60.02	6.42	-6.05	2.33E-05	8.01E-04
cg26805720		5	Intergenic	Island	42.44	6.77	48.50	7.82	-6.05	5.70E-04	1.12E-02
cg13347910	GCNT2;GCNT2	6	TSS200;Body	Open sea	40.90	5.32	46.95	6.09	-6.05	3.70E-05	1.20E-03
cg09180239	KIAA0182;KIAA0182	16	5'UTR;Body	Island	61.18	6.03	67.23	6.37	-6.05	1.26E-04	3.38E-03
cg04203646	APC2	19	Body	Island	39.07	7.80	45.13	7.35	-6.05	7.18E-05	2.12E-03
cg17759252	CRTC1;CRTC1	19	Body;Body	Island	28.24	8.99	34.29	9.75	-6.05	3.87E-03	4.46E-02
cg21532636		22	Intergenic	Open sea	40.32	8.46	46.37	8.01	-6.05	3.22E-04	7.19E-03
cg18298927		18	Intergenic	Island	31.33	7.40	37.38	7.30	-6.05	3.45E-04	7.59E-03
cg13134634	LMO7	13	Body	N_Shelf	38.17	6.10	44.22	5.04	-6.05	1.15E-07	5.70E-06
cg07437546		6	Intergenic	Open sea	55.79	5.70	61.84	5.11	-6.05	6.81E-06	2.65E-04
cg03405260		17	Intergenic	Island	14.97	4.14	21.02	7.92	-6.05	6.95E-06	2.70E-04
cg12587615	LPPR2;LPPR2	19	Body;Body	N_Shore	35.71	5.03	41.76	6.16	-6.05	1.25E-06	5.56E-05
cg25485435	RAMP1	2	TSS1500	N_Shore	47.99	6.36	54.04	7.08	-6.05	1.08E-04	2.99E-03
cg27202913	CDH15	16	Body	Island	13.39	4.66	19.44	7.08	-6.05	1.37E-05	5.01E-04
cg00984009		7	Intergenic	Open sea	56.38	5.75	62.43	6.32	-6.05	4.25E-05	1.35E-03
cg01797704	CMIP;CMIP	16	Body;Body	Open sea	38.82	5.80	44.87	6.52	-6.05	3.36E-05	1.10E-03
cg06358131	PIP5K1C	19	Body	Island	29.57	5.10	35.61	5.49	-6.05	8.21E-07	3.73E-05
cg04208329		14	Intergenic	N_Shelf	75.35	4.56	81.39	5.25	-6.04	1.24E-07	6.15E-06
cg04471375	GOT2	16	Body	Open sea	52.61	7.43	58.65	8.40	-6.04	3.44E-03	4.12E-02
cg06716730	DUSP14	17	5'UTR	S_Shore	55.73	7.25	61.78	6.17	-6.04	2.23E-05	7.69E-04
cg02032558		4	Intergenic	N_Shore	51.73	6.17	57.78	6.57	-6.04	4.75E-05	1.49E-03
cg18537454		10	Intergenic	N_Shore	41.69	6.85	47.73	8.65	-6.04	1.09E-03	1.83E-02
cg24913168	SERP2	13	3'UTR	Open sea	52.86	7.61	58.90	8.40	-6.04	1.82E-03	2.65E-02
cg09829869	TECPR1	7	Body	Open sea	62.82	5.08	68.87	4.31	-6.04	1.12E-07	5.56E-06
cg22486834	GSTA4	6	5'UTR	N_Shore	51.87	8.43	57.91	7.72	-6.04	3.52E-04	7.71E-03
cg15201545	MYL5	4	Body	Island	12.08	7.21	18.12	10.67	-6.04	5.04E-04	1.02E-02
cg17192381	BCL2	18	3'UTR	Open sea	56.75	5.87	62.79	6.69	-6.04	5.30E-05	1.63E-03
cg15765817	ODZ4	11	Body	Island	54.31	8.85	60.35	7.18	-6.04	8.90E-04	1.57E-02
cg05552874	IFIT1	10	Body	Open sea	22.38	5.01	28.42	6.77	-6.04	7.30E-06	2.83E-04
cg07781995		1	Intergenic	Open sea	56.25	7.16	62.28	8.94	-6.04	1.37E-03	2.16E-02
cg11132223	SELE	1	Body	Open sea	56.11	6.32	62.15	6.85	-6.04	3.90E-04	8.36E-03
cg13142949		14	Intergenic	Open sea	73.54	5.34	79.58	3.83	-6.04	4.42E-07	2.08E-05
cg14062002		6	Intergenic	Island	36.89	4.84	42.92	5.24	-6.03	4.34E-08	2.22E-06
cg22828110	F2RL2;IQGAP2	5	TSS200;Body	Open sea	24.11	5.83	30.14	7.03	-6.03	4.51E-04	9.36E-03
cg19628739		22	Intergenic	N_Shelf	41.51	8.63	47.55	8.14	-6.03	3.35E-06	1.38E-04
cg12050497	FAM84A	2	5'UTR	Island	27.70	4.49	33.73	5.82	-6.03	4.87E-08	2.49E-06
cg12861034	RAPGEFL1	17	Body	S_Shore	7.57	3.05	13.61	6.68	-6.03	2.23E-07	1.08E-05
cg01351300	ARSJ	4	Body	Open sea	37.02	5.30	43.06	8.06	-6.03	2.80E-04	6.45E-03
cg08637874		12	Intergenic	Open sea	43.93	6.81	49.97	7.62	-6.03	1.23E-03	2.00E-02
cg06895913	PDE4D;PDE4D	5	Body;Body	Open sea	41.55	6.67	47.58	8.46	-6.03	3.06E-05	1.02E-03
cg07414525	CHL1	3	Body	Open sea	34.88	7.06	40.91	8.93	-6.03	2.34E-03	3.16E-02
cg21549285	MX1;MX1	21	5'UTR;5'UTR	S_Shore	61.39	7.75	67.42	7.53	-6.03	4.01E-04	8.53E-03
cg12499872	TEPP;TEPP	16	Body;Body	Island	65.49	6.88	71.52	8.49	-6.03	2.70E-04	6.27E-03

cg04127765	SHROOM3	4	Body	S_Shore	17.30	4.69	23.32	6.58	-6.03	2.67E-06	1.12E-04
cg02262866	LRRK1	15	Body	Island	80.53	8.27	86.55	5.09	-6.03	4.53E-04	9.39E-03
cg10369896	ELFN1	7	TSS200	Open sea	52.07	5.65	58.09	5.63	-6.03	2.25E-05	7.75E-04
cg10512089	MIR130A	11	Body	Open sea	34.19	4.88	40.21	4.78	-6.02	3.01E-06	1.25E-04
cg01432087		11	Intergenic	Open sea	61.81	5.25	67.83	5.42	-6.02	1.13E-05	4.19E-04
cg11841231	PARD3B;PARD3B;PARD3B	2	Body;Body;Body	Open sea	85.70	4.56	91.73	2.06	-6.02	4.80E-13	2.96E-11
cg16179720	SPATS2L;SPATS2L;SPATS2L;SPATS2L	2	5'UTR;5'UTR;5'UTR;5'UTR	S_Shelf	52.88	4.93	58.90	3.82	-6.02	9.00E-09	4.80E-07
cg08187736		8	Intergenic	Open sea	58.14	5.60	64.16	4.66	-6.02	2.28E-06	9.68E-05
cg21506509	MN1	22	1stExon	Island	18.57	4.43	24.59	6.82	-6.02	1.68E-06	7.27E-05
cg03124146		7	Intergenic	Island	12.66	7.01	18.68	8.22	-6.02	1.00E-04	2.81E-03
cg16437184		6	Intergenic	Open sea	64.07	5.60	70.09	4.65	-6.02	2.60E-07	1.25E-05
cg24110050	TCTEX1D1	1	TSS1500	N_Shore	28.01	5.95	34.03	6.51	-6.01	1.34E-06	5.89E-05
cg05056133	SLC4A3;SLC4A3	2	TSS1500;TSS1500	N_Shore	57.59	6.51	63.61	7.54	-6.01	4.46E-04	9.28E-03
cg10403394	TPM1;TPM1;TPM1;TPM1;TPM1;TPM1	15	Body;Body;Body;Body;Body;Body;Body	Open sea	41.32	5.76	47.34	5.70	-6.01	8.68E-06	3.31E-04
cg18666075		3	Intergenic	Open sea	46.50	4.82	52.51	4.06	-6.01	8.03E-09	4.30E-07
cg12289926	LRP5	11	Body	Island	70.71	7.54	76.72	5.52	-6.01	2.04E-03	2.87E-02
cg26717554	ELANE	19	Body	Island	25.30	6.75	31.31	7.81	-6.01	4.45E-04	9.25E-03
cg02245794	TACC1;TACC1	8	1stExon;5'UTR	Open sea	7.96	4.56	13.97	9.21	-6.01	1.66E-04	4.25E-03
cg21367907		10	Intergenic	Open sea	37.92	4.85	43.93	5.37	-6.01	1.21E-07	6.01E-06
cg02854536	USP2;USP2	11	Body;Body	S_Shore	35.09	6.59	41.10	6.96	-6.01	4.77E-04	9.76E-03
cg05364072	MAP7D1	1	Body	Island	77.28	6.83	83.29	6.28	-6.01	4.91E-04	9.98E-03
cg15552370	NEURL1B	5	3'UTR	S_Shelf	57.72	4.78	63.73	3.37	-6.01	5.79E-08	2.94E-06
cg24434387		17	Intergenic	N_Shelf	55.57	7.11	61.58	6.18	-6.01	8.64E-04	1.54E-02
cg19024700	PLEKHA4;HSD17B14;PLEKHA4	19	3'UTR;TSS1500;Body	Island	50.40	7.68	56.40	9.98	-6.01	2.48E-03	3.28E-02
cg05588508		3	Intergenic	S_Shelf	71.41	4.73	77.41	4.61	-6.01	3.06E-08	1.58E-06
cg11374732	INPP5D;INPP5D	2	Body;Body	Island	63.64	7.57	69.65	7.55	-6.00	3.02E-03	3.76E-02
cg10782668	PDX1	13	Body	Island	23.31	7.80	29.31	9.65	-6.00	3.85E-03	4.44E-02
cg07024786	WIPF3	7	Body	Open sea	53.94	6.88	59.94	6.66	-6.00	2.64E-05	8.94E-04
cg19623012		11	Intergenic	Island	32.09	7.47	38.10	7.81	-6.00	2.05E-03	2.88E-02
cg06038472	RAB43	3	Body	N_Shelf	59.40	5.27	65.40	6.08	-6.00	3.08E-05	1.02E-03
cg21213853	GRM2;GRM2	3	5'UTR;5'UTR	S_Shore	62.50	7.28	68.50	5.63	-6.00	4.32E-05	1.37E-03
cg21708058	TACC1	8	TSS200	Open sea	32.64	6.45	38.64	8.02	-6.00	1.03E-03	1.75E-02
cg05251952	TMEM192	4	3'UTR	Open sea	49.10	6.70	55.10	8.03	-6.00	8.91E-04	1.57E-02
cg11746813	HOXC4;HOXC4	12	1stExon;Body	Island	30.19	5.10	36.19	5.09	-6.00	9.31E-07	4.20E-05
cg18079499	CALM1;CALM1	14	Body;Body	S_Shelf	49.08	5.70	55.08	5.38	-6.00	9.80E-07	4.41E-05
cg08104146		15	Intergenic	Open sea	23.35	4.82	29.35	7.09	-6.00	1.12E-04	3.07E-03
cg15690400	TNFRSF8;TNFRSF8	1	5'UTR;Body	Open sea	53.87	6.60	59.86	9.12	-6.00	1.31E-03	2.09E-02
cg04103250	SVIL;SVIL	10	Body;Body	Open sea	74.89	4.82	80.88	4.37	-6.00	4.19E-08	2.15E-06
cg04743654	RASSF1;RASSF1;RASSF1;RASSF1	3	5'UTR;1stExon;1stExon;5'UTR	Island	27.49	5.69	33.48	6.72	-6.00	8.84E-06	3.37E-04
cg25677709	NDST1	5	5'UTR	Open sea	48.14	4.46	54.14	5.66	-6.00	1.61E-06	7.00E-05
cg03434624	VPS37B	12	TSS200	Island	17.17	4.05	23.16	5.42	-6.00	2.94E-09	1.61E-07
cg11253957	MYO18A;MYO18A	17	Body;Body	Open sea	68.55	6.47	74.54	7.15	-6.00	1.21E-03	1.97E-02
cg11663667		4	Intergenic	N_Shore	30.84	5.43	36.84	7.27	-6.00	1.38E-05	5.04E-04
cg04665867	SLC22A18AS;SLC22A18;SLC22A18	11	5'UTR;5'UTR;TSS1500	N_Shore	67.06	6.43	73.06	4.76	-6.00	2.51E-05	8.55E-04
cg07593390	IL20RB	3	Body	Open sea	41.90	6.28	47.90	4.89	-5.99	1.07E-05	3.99E-04
cg11209521	GNG12	1	5'UTR	Open sea	79.85	6.05	85.84	3.42	-5.99	5.68E-08	2.89E-06
cg08159989	KLHDC8A	1	TSS200	Open sea	64.85	5.81	70.85	4.28	-5.99	5.99E-06	2.36E-04
cg03239925	NFATC1;NFATC1;NFATC1;NFATC1	18	Body;Body;Body;Body	Island	80.32	8.18	86.31	5.40	-5.99	4.04E-05	1.29E-03
cg23135546	ASPSCR1	17	Body	Island	65.89	6.41	71.89	7.15	-5.99	9.31E-04	1.62E-02
cg06936779	PIP5K1A;PIP5K1A;PIP5K1A;PIP5K1A;PI	1	5'UTR;1stExon;5'UTR;1stExon;5'UTR;1stEx	Island	28.67	4.66	34.66	5.16	-5.99	2.99E-07	1.43E-05
cg25468928	TBC1D5;TBC1D5;TBC1D5	3	5'UTR;5'UTR;5'UTR	Open sea	82.66	11.95	88.66	3.12	-5.99	5.65E-04	1.11E-02
cg06855137	FAM38A	16	Body	S_Shore	66.43	4.78	72.42	4.71	-5.99	1.49E-07	7.33E-06
cg00700487	NOS3	7	Body	Island	37.09	5.17	43.08	5.66	-5.99	8.23E-06	3.16E-04

cg20703122	OCA2	15	5'UTR	N_Shore	57.76	7.43	63.75	8.29	-5.99	5.64E-04	1.11E-02
cg17206772		11	Intergenic	Open sea	42.17	6.02	48.16	7.41	-5.99	7.71E-05	2.25E-03
cg15680620	ANKRD45	1	TSS200	S_Shore	44.99	7.66	50.97	8.30	-5.99	1.93E-05	6.78E-04
cg20993754	RTN4;RTN4;RTN4;RTN4;RTN4	2	Body;Body;Body;Body;Body	Open sea	64.07	6.58	70.06	7.53	-5.99	9.25E-04	1.62E-02
cg04853583		19	Intergenic	Island	72.75	8.06	78.74	6.62	-5.99	1.82E-03	2.64E-02
cg18850728	MRO;MRO;MRO;MRO	18	TSS1500;TSS1500;5'UTR;TSS1500	Open sea	57.61	7.28	63.59	7.87	-5.98	1.58E-03	2.39E-02
cg00718336	C3orf26;FILIP1L;FILIP1L	3	Body;5'UTR;5'UTR	Open sea	57.40	5.61	63.38	4.33	-5.98	1.61E-06	7.02E-05
cg24336808		12	Intergenic	Open sea	48.58	8.01	54.56	7.38	-5.98	1.02E-04	2.84E-03
cg04492287	HIST1H2BD	6	3'UTR	N_Shelf	72.24	7.71	78.22	6.68	-5.98	1.04E-04	2.91E-03
cg22857963	GLI4	8	Body	Island	16.50	6.28	22.48	6.03	-5.98	1.01E-06	4.55E-05
cg14802609	PLXDC2	10	Body	Open sea	80.68	11.45	86.66	4.71	-5.98	3.63E-03	4.27E-02
cg00502926	RNASE7	14	TSS1500	Open sea	36.39	5.50	42.37	6.05	-5.98	2.80E-06	1.17E-04
cg27066319	SUOX;SUOX;SUOX	12	5'UTR;5'UTR;5'UTR	Open sea	54.58	7.20	60.56	6.82	-5.98	4.28E-04	8.99E-03
cg17944003	NDUFAF2	5	Body	Open sea	52.56	7.16	58.54	7.59	-5.98	1.18E-03	1.93E-02
cg09279566	TMEM175	4	5'UTR	N_Shore	72.43	5.39	78.40	4.26	-5.98	9.12E-07	4.12E-05
cg01962750		2	Intergenic	Open sea	73.88	6.35	79.85	5.68	-5.98	1.77E-04	4.48E-03
cg24869232		2	Intergenic	S_Shelf	54.45	5.65	60.43	5.25	-5.98	4.09E-06	1.67E-04
cg04780086	ACOXL	2	3'UTR	Island	21.79	6.40	27.77	7.95	-5.97	1.47E-04	3.85E-03
cg11825675	CAND1	12	Body	S_Shelf	73.91	7.34	79.88	4.86	-5.97	1.32E-04	3.51E-03
cg15052447	MXRA7;MXRA7;MXRA7	17	Body;Body;Body	Open sea	40.00	6.25	45.97	6.35	-5.97	1.05E-05	3.95E-04
cg27380499		2	Intergenic	S_Shore	29.69	4.03	35.67	4.26	-5.97	1.06E-08	5.61E-07
cg18960216	TIAF1;MYO18A;MYO18A;TIAF1	17	5'UTR;Body;Body;1stExon	Open sea	75.47	4.88	81.44	3.37	-5.97	7.55E-08	3.80E-06
cg21474897	CLDN11	3	TSS200	Island	7.32	3.23	13.29	7.17	-5.97	3.65E-07	1.73E-05
cg17907457	HS2ST1;HS2ST1	1	Body;Body	Open sea	75.90	6.68	81.87	3.83	-5.97	2.98E-05	9.93E-04
cg05991220	ITIH1;ITIH1;ITIH1;ITIH1	3	TSS200;TSS200;Body;Body	Open sea	51.72	6.19	57.69	8.27	-5.97	6.68E-04	1.27E-02
cg02652891	PACRG;PACRG;PACRG	6	Body;Body;Body	S_Shelf	45.62	4.92	51.59	4.46	-5.97	1.23E-07	6.07E-06
cg14184078	CBLN4;CBLN4	20	5'UTR;1stExon	Island	25.59	5.36	31.56	5.39	-5.97	1.29E-07	6.39E-06
cg22266749	COL25A1;COL25A1	4	TSS200;TSS200	Island	6.90	1.27	12.87	1.77	-5.97	5.15E-32	4.60E-30
cg05514909		12	Intergenic	Island	58.77	9.96	64.74	7.50	-5.97	1.09E-03	1.83E-02
cg16726947		2	Intergenic	Open sea	69.45	6.31	75.41	6.12	-5.97	7.04E-05	2.09E-03
cg20457145	KTN1;KTN1;KTN1;KTN1	14	5'UTR;5'UTR;5'UTR;5'UTR	Open sea	67.17	6.44	73.14	7.22	-5.97	2.47E-04	5.85E-03
cg09630142	C6orf27	6	Body	Open sea	72.02	6.29	77.99	4.37	-5.97	4.41E-06	1.79E-04
cg21207028	NRSN2;NRSN2	20	1stExon;5'UTR	Island	23.83	5.50	29.80	6.19	-5.97	2.74E-06	1.15E-04
cg09642378	ARHGAP12	10	5'UTR	N_Shore	40.41	6.38	46.37	6.78	-5.96	8.82E-06	3.36E-04
cg01525538	DUSP5P	1	Body	Island	45.20	7.48	51.16	8.83	-5.96	1.03E-03	1.76E-02
cg18205244	CUX1;CUX1;CUX1	7	Body;Body;Body	Open sea	54.84	6.18	60.80	5.60	-5.96	5.82E-05	1.77E-03
cg10185424	CD180	5	3'UTR	Open sea	37.59	5.76	43.55	5.42	-5.96	3.56E-06	1.46E-04
cg10404717	MIR219-2	9	Body	Island	80.59	6.84	86.55	6.35	-5.96	5.67E-05	1.73E-03
cg19812944	PRKAR1B;HEATR2;PRKAR1B;PRKAR1B	7	TSS1500;Body;TSS1500;TSS1500	S_Shore	41.25	5.93	47.21	6.47	-5.96	2.19E-05	7.57E-04
cg26957951		5	Intergenic	Open sea	37.77	5.54	43.73	5.81	-5.96	8.50E-06	3.25E-04
cg06223162	GPR88	1	TSS200	N_Shore	75.81	7.84	81.77	6.81	-5.96	8.58E-04	1.53E-02
cg25140444	PKN2	1	Body	Open sea	54.29	6.02	60.24	6.47	-5.96	4.60E-05	1.45E-03
cg02742676	PCDHA7;PCDHA12;PCDHA6;PCDHA10	5	Body;Body;Body;Body;Body;Body;Body;Bo	Open sea	66.27	7.57	72.22	6.23	-5.96	2.14E-03	2.97E-02
cg14527108		7	Intergenic	N_Shore	51.69	6.33	57.64	7.71	-5.95	3.02E-04	6.85E-03
cg21800284		12	Intergenic	S_Shore	31.81	6.42	37.76	7.65	-5.95	4.49E-04	9.33E-03
cg03414932	FGFRL1;FGFRL1;FGFRL1	4	Body;Body;Body	S_Shore	67.57	5.65	73.52	6.58	-5.95	3.64E-05	1.18E-03
cg07514343	MGA;MIR626;MGA	15	Body;TSS1500;Body	Open sea	30.99	3.87	36.94	4.69	-5.95	1.21E-08	6.40E-07
cg26217572		13	Intergenic	Open sea	28.89	3.94	34.84	5.19	-5.95	6.26E-07	2.89E-05
cg10186456	TMPRSS8	16	TSS200	S_Shore	49.02	5.78	54.97	6.67	-5.95	4.76E-06	1.92E-04
cg20469139	RNF135;RNF135	17	TSS1500;TSS1500	N_Shore	50.54	6.62	56.49	6.19	-5.95	3.61E-05	1.17E-03
cg00525683	ADAM19	5	Body	Open sea	45.26	7.85	51.21	7.07	-5.95	1.44E-03	2.24E-02
cg04255230	LBX2	2	Body	Island	45.98	7.05	51.93	7.80	-5.95	8.60E-04	1.53E-02
cg13684113	TMED7-TICAM2;TMED7-TICAM2	5	Body;Body	Open sea	72.09	7.35	78.04	5.92	-5.95	2.22E-04	5.37E-03

cg26565660		12	Intergenic	Open sea	52.41	5.04	58.36	4.71	-5.95	1.67E-07	8.20E-06
cg02773436		10	Intergenic	Open sea	60.59	6.56	66.54	6.39	-5.95	1.43E-04	3.77E-03
cg12203072		2	Intergenic	Island	50.95	5.49	56.90	5.51	-5.95	2.85E-06	1.19E-04
cg01643090	CAMTA1	1	Body	N_Shore	49.02	5.22	54.97	6.75	-5.95	1.64E-05	5.84E-04
cg10550182	C2orf62	2	TSS1500	Open sea	60.67	7.97	66.61	8.80	-5.95	2.08E-03	2.91E-02
cg01278873	ZNF233;ZNF233	19	1stExon;5'UTR	Island	29.63	6.17	35.57	6.03	-5.95	1.11E-08	5.87E-07
cg01407332	GPR133	12	Body	Open sea	23.16	6.99	29.11	7.09	-5.94	2.62E-04	6.13E-03
cg10247840	SNORA55;PABPC4;PABPC4;PABPC4	1	TSS1500;Body;Body;Body	Open sea	62.31	6.24	68.25	7.27	-5.94	4.89E-04	9.96E-03
cg06555959		8	Intergenic	Island	32.41	7.21	38.36	7.21	-5.94	1.50E-03	2.30E-02
cg00874055	GPR137B	1	Body	Island	24.33	8.04	30.27	6.56	-5.94	8.93E-05	2.55E-03
cg27638815	CYP4F22	19	3'UTR	S_Shore	36.68	6.01	42.62	7.44	-5.94	1.25E-04	3.35E-03
cg00014830		17	Intergenic	Island	28.22	4.26	34.16	5.68	-5.94	5.20E-07	2.42E-05
cg00864945		1	Intergenic	S_Shore	45.63	7.29	51.57	8.16	-5.94	4.06E-04	8.61E-03
cg23157419	PTPN9	15	Body	Open sea	66.82	5.01	72.76	5.28	-5.94	1.32E-06	5.83E-05
cg16820973	FGD4	12	5'UTR	Open sea	50.42	5.57	56.36	7.73	-5.94	4.52E-04	9.38E-03
cg23223933	KCNIP2;KCNIP2;KCNIP2;KCNIP2;KCNIP	10	1stExon;1stExon;1stExon;1stExon;1stExon	Island	52.68	5.29	58.62	5.82	-5.94	6.53E-07	3.01E-05
cg17375585		8	Intergenic	Open sea	36.88	6.61	42.82	6.26	-5.94	4.92E-05	1.53E-03
cg13490235	ESRRG;ESRRG;ESRRG	1	5'UTR;5'UTR;5'UTR	Open sea	55.57	6.74	61.51	10.02	-5.94	2.15E-03	2.97E-02
cg18470427	SLFN12L	17	Body	Open sea	34.81	8.34	40.75	10.37	-5.94	3.33E-03	4.03E-02
cg05106804	ENOPH1	4	3'UTR	Open sea	78.77	4.56	84.71	4.24	-5.94	9.56E-09	5.10E-07
cg04271991	PTGIS	20	3'UTR	Open sea	71.77	5.68	77.71	4.85	-5.93	4.02E-06	1.64E-04
cg00965168		1	Intergenic	Island	56.77	5.23	62.70	5.12	-5.93	6.40E-06	2.50E-04
cg07416364		2	Intergenic	Open sea	70.73	5.07	76.66	5.19	-5.93	7.11E-08	3.58E-06
cg09278695		7	Intergenic	Open sea	37.43	8.83	43.37	7.33	-5.93	1.50E-03	2.30E-02
cg06836102		15	Intergenic	Open sea	34.24	5.52	40.18	8.67	-5.93	9.68E-04	1.67E-02
cg24939819	GPN2;GPATCH3	1	TSS1500;3'UTR	S_Shore	46.23	3.83	52.17	5.42	-5.93	4.40E-08	2.25E-06
cg16524733	TAGLN;TAGLN;TAGLN;TAGLN	11	5'UTR;1stExon;5'UTR;1stExon	Open sea	38.44	8.46	44.38	8.79	-5.93	3.03E-03	3.77E-02
cg20077773		12	Intergenic	Open sea	74.17	6.14	80.10	5.85	-5.93	8.00E-05	2.32E-03
cg01720186	MCF2L2;B3GNT5	3	Body;TSS200	N_Shore	7.27	2.80	13.20	5.61	-5.93	2.16E-10	1.24E-08
cg08560074	FOXI1;FOXI1	5	TSS1500;TSS1500	N_Shore	71.80	6.39	77.73	6.41	-5.93	2.64E-05	8.94E-04
cg14519997		9	Intergenic	N_Shore	19.25	4.62	25.18	6.82	-5.93	5.33E-06	2.12E-04
cg13959241	NACC2	9	Body	S_Shore	64.79	5.69	70.72	4.32	-5.93	5.74E-06	2.27E-04
cg26589351	SPOP;SPOP;SPOP;SPOP;SPOP;SPOP	17	5'UTR;5'UTR;5'UTR;5'UTR;5'UTR;5'UTR	Open sea	57.22	5.91	63.15	6.68	-5.93	1.67E-04	4.29E-03
cg01199928	RUVBL1	3	Body	N_Shore	50.27	6.71	56.20	7.83	-5.93	6.81E-04	1.28E-02
cg27149073	SDHAP3	5	Body	Island	13.84	6.48	19.77	10.98	-5.93	2.36E-03	3.18E-02
cg03957885		10	Intergenic	Open sea	43.96	5.16	49.88	5.54	-5.93	6.06E-06	2.38E-04
cg07336016	AMFR	16	Body	Open sea	72.35	7.57	78.28	5.62	-5.92	5.72E-06	2.26E-04
cg24654266	DPP10	2	Body	Island	22.44	7.56	28.37	9.03	-5.92	1.57E-03	2.37E-02
cg13373343		1	Intergenic	Open sea	34.09	7.79	40.01	8.18	-5.92	6.42E-06	2.51E-04
cg17345276	ABHD1	2	Body	S_Shelf	44.90	7.03	50.83	6.95	-5.92	1.13E-03	1.88E-02
cg13822849	OLFM1	9	Body	S_Shelf	32.88	6.26	38.80	8.66	-5.92	7.53E-04	1.39E-02
cg01977473	GRAMD3;GRAMD3;GRAMD3;GRAMD	5	Body;5'UTR;5'UTR;Body;Body	Open sea	87.41	6.57	93.33	1.89	-5.92	2.88E-08	1.49E-06
cg22295864		11	Intergenic	Open sea	59.48	5.41	65.40	5.78	-5.92	8.40E-08	4.21E-06
cg13463897	NCAM1;NCAM1;NCAM1	11	Body;Body;Body	Open sea	43.73	5.94	49.65	7.76	-5.92	2.60E-04	6.08E-03
cg08826040	HAGH;HAGH	16	Body;Body	N_Shelf	49.50	6.87	55.41	8.60	-5.92	1.89E-03	2.72E-02
cg02805890		6	Intergenic	S_Shore	25.13	5.56	31.04	9.47	-5.92	2.12E-04	5.18E-03
cg18708502	LATS2	13	Body	Open sea	43.04	5.35	48.96	6.44	-5.92	3.61E-05	1.17E-03
cg14428590	FSIP1	15	TSS1500	S_Shore	34.99	4.15	40.91	4.13	-5.92	2.61E-08	1.36E-06
cg05142445	TTLL5	14	Body	Open sea	51.64	7.07	57.55	6.05	-5.91	2.83E-04	6.50E-03
cg08944029	MCC;MCC	5	Body;Body	Open sea	37.09	5.92	43.00	7.30	-5.91	3.81E-04	8.20E-03
cg06296169	PLXNA4;PLXNA4;PLXNA4	7	5'UTR;5'UTR;5'UTR	Open sea	78.18	5.59	84.09	4.64	-5.91	2.20E-06	9.36E-05
cg21901577		4	Intergenic	Open sea	61.14	5.56	67.05	6.15	-5.91	1.30E-05	4.76E-04
cg22293823		11	Intergenic	Open sea	67.27	7.90	73.18	8.16	-5.91	6.49E-04	1.24E-02

cg06271561	ALPK3	15	TSS200	N_Shore	47.75	4.92	53.66	6.51	-5.91	3.15E-05	1.04E-03
cg18101028	SMR3A	4	TSS1500	Open sea	73.81	5.24	79.73	4.04	-5.91	2.60E-08	1.35E-06
cg05554936	RAB3D;RAB3D	19	5'UTR;1stExon	Island	10.64	4.64	16.55	5.81	-5.91	1.14E-08	6.03E-07
cg03517919	KIF1B	1	Body	Open sea	52.98	5.46	58.89	5.58	-5.91	2.22E-06	9.46E-05
cg23562479	OR52K2	11	TSS1500	Open sea	38.50	4.79	44.41	6.26	-5.91	7.14E-07	3.27E-05
cg06310589		6	Intergenic	N_Shelf	50.83	5.70	56.73	5.44	-5.91	1.79E-06	7.75E-05
cg04677123		5	Intergenic	Open sea	76.92	4.17	82.83	4.03	-5.91	1.06E-09	5.93E-08
cg02805025	ASB4;ASB4	7	Body;Body	N_Shelf	56.20	6.99	62.11	4.55	-5.91	1.37E-05	5.01E-04
cg18431910	ODZ4	11	Body	Island	59.47	6.66	65.38	6.58	-5.91	3.14E-05	1.04E-03
cg19443750		13	Intergenic	N_Shore	56.06	7.30	61.96	7.76	-5.90	1.05E-03	1.77E-02
cg00397764	TDRD1	10	5'UTR	S_Shore	59.57	5.51	65.47	6.53	-5.90	7.74E-06	2.98E-04
cg05690644	GDF6	8	Body	Island	27.12	6.53	33.03	9.25	-5.90	2.07E-03	2.90E-02
cg12159023	HCN2	19	Body	Island	74.03	8.79	79.94	9.38	-5.90	1.67E-03	2.48E-02
cg03310838	ZFPM1	16	Body	Open sea	43.45	4.79	49.35	5.84	-5.90	9.64E-06	3.65E-04
cg20334738	MAB21L2;LRBA	4	TSS200;Body	N_Shore	53.56	6.98	59.46	6.39	-5.90	4.38E-05	1.39E-03
cg13313226	C11orf61	11	Body	N_Shelf	71.81	4.89	77.71	3.55	-5.90	7.01E-08	3.54E-06
cg01730970	MAP3K6	1	Body	Island	31.00	6.54	36.90	7.99	-5.90	9.81E-05	2.75E-03
cg22833478	SLC22A18;SLC22A18;SLC22A18AS	11	Body;Body;TSS1500	S_Shore	45.49	5.82	51.39	6.81	-5.90	5.99E-05	1.82E-03
cg24102622	NFIX	19	Body	Island	49.53	4.78	55.43	5.26	-5.90	9.89E-08	4.93E-06
cg20064452	DAAM1	14	Body	Open sea	67.66	5.65	73.56	4.65	-5.90	7.10E-07	3.25E-05
cg00311768	TSTA3	8	Body	N_Shore	37.71	4.26	43.61	4.83	-5.90	1.35E-07	6.66E-06
cg06639267	FBXL16	16	3'UTR	N_Shore	61.53	4.16	67.42	6.01	-5.90	2.58E-07	1.25E-05
cg00907810		7	Intergenic	Open sea	80.75	7.01	86.65	2.67	-5.90	1.67E-07	8.16E-06
cg18898632		2	Intergenic	Island	79.46	9.83	85.35	9.21	-5.89	2.68E-04	6.23E-03
cg18535611		13	Intergenic	S_Shelf	62.14	7.02	68.04	6.52	-5.89	2.22E-04	5.37E-03
cg02621287		12	Intergenic	Island	32.65	7.56	38.54	9.03	-5.89	9.36E-04	1.63E-02
cg16167052	PNMAL2;PNMAL2	19	1stExon;3'UTR	Island	22.20	5.07	28.10	8.55	-5.89	1.22E-04	3.30E-03
cg20185691		6	Intergenic	Open sea	66.01	5.35	71.90	4.76	-5.89	1.02E-06	4.58E-05
cg03650119	GJC2	1	Body	Island	44.63	7.05	50.52	7.70	-5.89	1.46E-03	2.26E-02
cg13917273		9	Intergenic	S_Shore	43.34	5.84	49.24	7.10	-5.89	1.07E-04	2.97E-03
cg16277843		6	Intergenic	Open sea	62.44	5.41	68.33	6.20	-5.89	1.43E-05	5.19E-04
cg06579481		7	Intergenic	N_Shelf	60.51	6.29	66.39	5.38	-5.89	1.69E-05	6.02E-04
cg03476791		7	Intergenic	N_Shelf	50.58	7.31	56.46	5.50	-5.88	4.15E-04	8.77E-03
cg27137258	TMPRSS8	16	TSS200	Island	36.85	7.95	42.73	7.17	-5.88	2.19E-03	3.01E-02
cg18988110	PRR15L	17	TSS1500	Open sea	43.89	4.21	49.77	4.51	-5.88	6.05E-08	3.07E-06
cg02727219		16	Intergenic	Open sea	68.80	7.39	74.68	4.79	-5.88	9.68E-05	2.72E-03
cg23223330		8	Intergenic	Open sea	63.37	5.91	69.25	4.66	-5.88	2.01E-07	9.77E-06
cg03276022	TAGLN;TAGLN	11	TSS200;TSS200	Open sea	40.25	9.14	46.13	8.34	-5.88	3.35E-03	4.04E-02
cg27517968		2	Intergenic	Island	26.40	7.59	32.28	8.33	-5.88	1.91E-03	2.74E-02
cg27015047	DMBT1;DMBT1;DMBT1	10	Body;Body;Body	Open sea	72.38	6.12	78.26	6.49	-5.88	3.37E-04	7.45E-03
cg18098187	PEMT;PEMT;PEMT	17	Body;Body;Body	Open sea	68.40	5.33	74.27	5.56	-5.87	1.49E-05	5.37E-04
cg22014491		4	Intergenic	Open sea	63.34	5.41	69.22	4.86	-5.87	6.75E-08	3.41E-06
cg11748730	ANKS1B	12	Body	Open sea	41.20	8.56	47.07	7.65	-5.87	1.51E-03	2.32E-02
cg17292384	COX8A	11	3'UTR	S_Shore	69.81	4.35	75.68	3.67	-5.87	6.95E-10	3.90E-08
cg06698795	CHST13	3	Body	Island	40.22	5.97	46.09	6.10	-5.87	2.85E-05	9.52E-04
cg18587137	TNFAIP2	14	Body	Island	39.26	6.68	45.13	7.82	-5.87	1.19E-03	1.95E-02
cg06883206		4	Intergenic	Open sea	40.29	4.80	46.16	7.62	-5.87	1.25E-04	3.37E-03
cg25688583	TCF12;TCF12;TCF12;TCF12;TCF12	15	TSS1500;Body;Body;Body;Body	Open sea	66.27	5.97	72.14	5.50	-5.87	5.90E-06	2.33E-04
cg10431989	HHLA2	3	TSS200	Open sea	75.81	5.90	81.68	5.19	-5.87	1.50E-06	6.54E-05
cg16348962	FUK	16	5'UTR	S_Shelf	51.18	4.51	57.05	5.65	-5.87	2.96E-06	1.23E-04
cg03337430	EPHX1;EPHX1	1	TSS1500;5'UTR	Open sea	57.16	6.46	63.03	6.13	-5.87	2.60E-04	6.10E-03
cg21377071	ADAMTSL3	15	5'UTR	S_Shore	38.84	5.40	44.71	7.62	-5.87	9.13E-04	1.60E-02
cg07739758	NCRNA00181;A1BG	19	Body;TSS1500	N_Shelf	60.32	6.40	66.18	6.25	-5.87	8.24E-05	2.38E-03

cg21251563	CHN2;CHN2;CHN2	7	5'UTR;Body;1stExon	Open sea	60.15	6.68	66.01	6.05	-5.87	5.60E-05	1.71E-03
cg13472192		1	Intergenic	Island	16.76	4.96	22.62	5.59	-5.86	4.62E-06	1.86E-04
cg07722333	CCDC79	16	Body	S_Shelf	69.36	5.71	75.22	5.67	-5.86	3.14E-05	1.04E-03
cg20419291	ROBO4	11	TSS200	Open sea	51.33	4.92	57.19	5.58	-5.86	6.08E-07	2.81E-05
cg17966192	SULT1C4	2	TSS1500	Open sea	27.42	5.19	33.28	6.28	-5.86	1.54E-06	6.73E-05
cg19504001	LRP1	12	Body	Open sea	59.18	5.13	65.05	4.92	-5.86	6.39E-06	2.50E-04
cg19203575	ZNF323;ZKSCAN3;ZNF323	6	5'UTR;5'UTR;TSS200	Open sea	35.29	5.31	41.15	5.59	-5.86	1.51E-05	5.45E-04
cg06933824	NEURL1B	5	Body	Island	49.85	5.32	55.71	6.70	-5.86	3.45E-05	1.13E-03
cg03271533	UGGT1;UGGT1	2	Body;Body	Open sea	47.50	7.11	53.35	6.63	-5.86	2.73E-04	6.32E-03
cg06916011	EPS8L1;EPS8L1	19	Body;Body	Island	26.06	6.61	31.92	7.16	-5.86	1.68E-04	4.30E-03
cg20756245	LOC100130872;LOC100130872-SPON2	4	Body;Body	N_Shore	59.64	6.71	65.49	4.43	-5.86	6.48E-06	2.53E-04
cg21705505	C21orf33;C21orf33	21	Body;Body	N_Shelf	47.20	4.19	53.05	4.96	-5.86	6.56E-07	3.02E-05
cg27160920	TEKT5	16	Body	Open sea	63.01	6.40	68.86	5.83	-5.86	1.29E-04	3.46E-03
cg03711802	ANKRD44	2	Body	S_Shore	53.45	5.75	59.31	5.07	-5.85	4.56E-06	1.84E-04
cg06091086		6	Intergenic	Open sea	54.05	8.07	59.90	7.03	-5.85	6.63E-04	1.26E-02
cg15279364	TMEM130;TMEM130;TMEM130	7	Body;Body;Body	N_Shore	29.45	6.07	35.31	5.74	-5.85	3.69E-05	1.19E-03
cg00904622		6	Intergenic	Open sea	66.59	5.85	72.44	4.53	-5.85	6.30E-07	2.90E-05
cg07152925	S100B	21	5'UTR	Open sea	50.47	7.04	56.32	6.26	-5.85	3.85E-04	8.27E-03
cg06652011		16	Intergenic	Open sea	79.14	11.89	84.99	8.55	-5.85	9.60E-04	1.66E-02
cg18127481		5	Intergenic	Open sea	59.92	6.09	65.77	7.47	-5.85	9.66E-05	2.72E-03
cg18162908	PDLIM3;PDLIM3	4	Body;Body	Open sea	48.25	7.00	54.10	8.12	-5.85	2.21E-03	3.03E-02
cg13874654	NTN1	17	Body	S_Shore	33.57	5.24	39.42	6.50	-5.85	8.48E-07	3.84E-05
cg16146501		15	Intergenic	Open sea	36.12	5.51	41.97	7.02	-5.85	2.02E-04	4.98E-03
cg02411275	AJAP1;AJAP1	1	Body;Body	N_Shore	54.78	7.21	60.62	7.10	-5.85	6.45E-04	1.23E-02
cg15405121		5	Intergenic	Open sea	69.68	6.43	75.53	5.35	-5.85	1.00E-05	3.78E-04
cg07908654		13	Intergenic	N_Shelf	47.14	6.65	52.98	7.40	-5.84	2.42E-04	5.75E-03
cg05931423		5	Intergenic	Island	40.35	5.25	46.19	5.15	-5.84	2.75E-06	1.15E-04
cg22504523		7	Intergenic	Open sea	58.32	5.84	64.16	5.60	-5.84	2.62E-05	8.87E-04
cg05303559		7	Intergenic	Open sea	69.64	6.50	75.48	4.96	-5.84	4.02E-05	1.29E-03
cg04181327		2	Intergenic	N_Shore	53.57	4.22	59.42	4.88	-5.84	6.42E-07	2.96E-05
cg03542797	CLMN	14	3'UTR	Open sea	58.95	7.08	64.79	6.02	-5.84	3.27E-04	7.28E-03
cg14268780	FTCD;FTCD	21	3'UTR;3'UTR	Open sea	67.34	6.59	73.18	7.63	-5.84	6.21E-04	1.20E-02
cg07091758	PKD1L2;PKD1L2	16	TSS1500;TSS1500	Open sea	53.59	5.99	59.43	7.16	-5.84	1.05E-04	2.92E-03
cg23596199	DVL1	1	Body	N_Shelf	47.68	5.70	53.52	6.16	-5.84	5.79E-05	1.76E-03
cg24063912		4	Intergenic	S_Shelf	43.36	7.23	49.20	8.01	-5.84	6.15E-04	1.19E-02
cg03689146		7	Intergenic	Island	53.02	6.96	58.86	8.21	-5.84	9.93E-04	1.70E-02
cg13490422		12	Intergenic	Open sea	39.18	5.01	45.02	5.11	-5.84	3.55E-07	1.69E-05
cg08691159		16	Intergenic	Open sea	58.63	5.54	64.46	5.83	-5.84	8.55E-05	2.46E-03
cg13405222	CDKAL1	6	Body	Open sea	57.02	6.62	62.85	6.09	-5.84	2.83E-04	6.50E-03
cg19823504	SEMA6B	19	Body	N_Shore	71.78	6.36	77.61	5.47	-5.84	5.05E-04	1.02E-02
cg25612297		2	Intergenic	Open sea	69.43	6.32	75.26	5.08	-5.84	1.88E-06	8.10E-05
cg20034792		6	Intergenic	Island	36.22	8.87	42.05	7.49	-5.83	4.38E-03	4.86E-02
cg16407069	PARVA	11	Body	Open sea	64.92	5.77	70.75	5.36	-5.83	9.24E-06	3.51E-04
cg16861031	ALDH1L2;ALDH1L2	12	Body;Body	N_Shore	31.19	5.59	37.03	4.82	-5.83	3.70E-06	1.51E-04
cg08555866		16	Intergenic	Island	49.03	5.95	54.86	7.13	-5.83	1.70E-04	4.33E-03
cg22369318	CALD1;CALD1;CALD1;CALD1;CALD1	7	Body;Body;Body;Body;Body	Open sea	69.11	6.12	74.94	4.39	-5.83	1.87E-06	8.05E-05
cg18765906	NID1	1	Body	Open sea	20.62	6.00	26.45	8.11	-5.83	7.95E-05	2.31E-03
cg09416908	ME3;ME3;ME3	11	TSS1500;TSS1500;TSS1500	S_Shore	41.56	5.15	47.39	6.87	-5.83	3.30E-05	1.08E-03
cg27568165	SLC45A3	1	Body	Island	66.55	5.51	72.37	5.88	-5.83	6.94E-05	2.06E-03
cg25321212	FAM196A;DOCK1	10	5'UTR;Body	N_Shore	64.90	5.62	70.73	4.55	-5.83	1.12E-05	4.17E-04
cg19500479	GDF7	2	TSS1500	Island	8.81	3.76	14.63	8.74	-5.83	2.77E-05	9.31E-04
cg25887394		15	Intergenic	Open sea	64.27	6.88	70.09	5.67	-5.82	1.16E-04	3.16E-03
cg07415388	MIR33B;SREBF1;SREBF1	17	TSS200;Body;Body	S_Shore	69.71	7.83	75.53	6.38	-5.82	3.03E-03	3.77E-02

cg13641185		6	Intergenic	Island	26.31	7.04	32.14	6.99	-5.82	1.85E-04	4.65E-03
cg17327492	FSHR;FSHR	2	TSS1500;TSS1500	Open sea	58.56	7.19	64.38	7.16	-5.82	1.98E-03	2.81E-02
cg16055106	FER	5	Body	Open sea	59.23	5.45	65.05	6.34	-5.82	7.68E-05	2.24E-03
cg26291600	ALOX5	10	Body	S_Shore	62.09	6.37	67.91	6.86	-5.82	1.63E-04	4.18E-03
cg18252039		14	Intergenic	S_Shelf	31.73	4.78	37.55	6.83	-5.82	1.54E-05	5.54E-04
cg12629991	PBX1	1	Body	Open sea	74.73	5.66	80.55	4.52	-5.82	3.69E-08	1.90E-06
cg01557221	APOL3;APOL3;APOL3;APOL3;APOL3;A	22	5'UTR;TSS1500;TSS1500;TSS1500;TSS1500	Open sea	50.42	7.53	56.24	7.70	-5.82	1.92E-03	2.75E-02
cg12069151	PPP1R10	6	5'UTR	Open sea	43.61	5.29	49.43	6.35	-5.82	1.66E-05	5.92E-04
cg20082196	FAM83E	19	TSS200	S_Shore	64.88	6.21	70.70	5.44	-5.82	9.38E-05	2.66E-03
cg20931951	VPS13D;VPS13D	1	Body;Body	Open sea	66.75	6.09	72.56	7.59	-5.82	4.66E-04	9.60E-03
cg16160493	FOXN3;FOXN3	14	Body;Body	Open sea	66.58	8.22	72.40	8.25	-5.82	3.83E-03	4.43E-02
cg06448070	DOCK6	19	Body	Island	65.47	6.70	71.29	8.03	-5.82	1.21E-03	1.98E-02
cg16435561	RFX8	2	5'UTR	Island	24.67	9.08	30.49	9.42	-5.81	1.03E-03	1.75E-02
cg19163395	HDAC5;HDAC5	17	Body;Body	Open sea	37.41	5.88	43.22	7.43	-5.81	6.06E-06	2.38E-04
cg22626747	C19orf39	19	3'UTR	S_Shore	59.54	6.69	65.35	5.74	-5.81	6.55E-05	1.96E-03
cg25204764	SRRM1	1	Body	Open sea	72.90	5.24	78.71	4.29	-5.81	1.98E-07	9.66E-06
cg12625437		5	Intergenic	Open sea	63.01	6.87	68.82	7.86	-5.81	2.20E-03	3.02E-02
cg01729262		1	Intergenic	N_Shore	49.19	6.39	55.01	8.35	-5.81	2.18E-03	3.00E-02
cg12942690	RILPL1	12	3'UTR	Open sea	51.75	6.21	57.56	7.97	-5.81	2.44E-03	3.25E-02
cg05593615	KCTD9	8	Body	Open sea	65.79	7.67	71.60	7.50	-5.81	5.00E-04	1.01E-02
cg13451127	NBEAL2	3	Body	Island	15.73	5.07	21.54	9.31	-5.81	3.54E-03	4.20E-02
cg05261349	ST3GAL2	16	TSS1500	S_Shore	59.53	5.22	65.34	6.59	-5.81	6.02E-05	1.82E-03
cg10207460		1	Intergenic	Open sea	76.72	2.39	82.53	1.84	-5.81	6.63E-20	4.75E-18
cg11630226	LY6K;LY6K;LY6K;LY6K;LY6K;LY6K	8	1stExon;1stExon;5'UTR;1stExon;5'UTR;5'U	Island	26.67	6.26	32.47	6.86	-5.81	9.57E-05	2.70E-03
cg27543538	AHDC1	1	5'UTR	Island	19.81	4.12	25.62	5.17	-5.81	8.74E-07	3.95E-05
cg25786525	C10orf11	10	Body	Open sea	32.49	4.00	38.29	5.02	-5.80	5.72E-09	3.08E-07
cg13484329	TCHP;TCHP	12	Body;Body	Open sea	73.18	5.60	78.99	3.80	-5.80	7.82E-06	3.01E-04
cg22796860	MACROD1	11	Body	Open sea	23.64	7.17	29.44	9.01	-5.80	9.48E-05	2.68E-03
cg11265725	VMAC	19	3'UTR	S_Shelf	64.41	7.49	70.22	5.62	-5.80	1.46E-03	2.26E-02
cg26574395	SULT2A1	19	TSS1500	Open sea	51.82	6.52	57.63	7.48	-5.80	1.99E-04	4.93E-03
cg21153040	MIR181D	19	Body	S_Shore	46.80	6.18	52.60	6.56	-5.80	1.07E-04	2.96E-03
cg13402847	RHOBTB2	8	TSS200	Open sea	54.16	5.55	59.96	5.16	-5.80	2.74E-07	1.32E-05
cg07809176	REP15;REP15	12	5'UTR;1stExon	Open sea	60.56	6.31	66.36	6.98	-5.80	3.23E-04	7.21E-03
cg07532405		14	Intergenic	Open sea	47.59	6.25	53.39	8.88	-5.80	1.65E-03	2.46E-02
cg14586373	KCTD17	22	TSS1500	N_Shore	29.21	6.02	35.01	7.54	-5.80	1.76E-04	4.47E-03
cg03712476	SHROOM3	4	Body	S_Shore	19.11	6.42	24.91	7.89	-5.80	1.75E-05	6.22E-04
cg17187167		8	Intergenic	Island	61.84	8.11	67.64	8.35	-5.80	2.30E-03	3.12E-02
cg02006915	PCDH24	5	5'UTR	Open sea	62.69	5.71	68.48	4.88	-5.80	6.14E-07	2.84E-05
cg22616933	KIAA1324L;KIAA1324L;KIAA1324L	7	Body;Body;Body	Open sea	53.44	6.12	59.24	6.86	-5.80	2.83E-04	6.50E-03
cg10035583	DAAM2	6	3'UTR	S_Shore	31.19	6.04	36.98	8.47	-5.80	1.18E-03	1.94E-02
cg18500085	SLC35C1;SLC35C1;SLC35C1	11	Body;Body;1stExon	S_Shore	40.33	5.20	46.12	9.36	-5.80	1.32E-03	2.11E-02
cg01804036	TESC;TESC;TESC	12	Body;Body;Body	N_Shore	18.47	5.60	24.27	7.69	-5.79	1.06E-04	2.95E-03
cg04439776	TYSND1;TYSND1	10	TSS1500;TSS1500	S_Shore	41.20	5.53	47.00	6.51	-5.79	2.78E-05	9.34E-04
cg19183395		2	Intergenic	Island	39.23	8.07	45.03	7.33	-5.79	1.80E-03	2.63E-02
cg01394847		16	Intergenic	S_Shore	40.68	6.16	46.47	6.95	-5.79	1.01E-04	2.83E-03
cg19002337	INS-IGF2;IGF2AS;IGF2;IGF2;IGF2AS;IG	11	Body;TSS1500;Body;5'UTR;TSS1500;TSS15	Island	43.95	5.45	49.74	5.37	-5.79	8.86E-07	4.01E-05
cg21908235		1	Intergenic	Island	23.11	6.84	28.89	7.14	-5.79	2.10E-05	7.31E-04
cg14036069	NEK6;NEK6;NEK6;NEK6	9	5'UTR;5'UTR;Body;5'UTR	Open sea	31.39	4.35	37.18	5.86	-5.79	2.92E-06	1.22E-04
cg10551856	NOL10	2	Body	Open sea	58.46	6.83	64.24	7.28	-5.79	4.80E-04	9.81E-03
cg01686177		7	Intergenic	Open sea	65.40	4.22	71.18	4.45	-5.79	1.66E-08	8.72E-07
cg12293170	PLXNA4;PLXNA4;PLXNA4	7	5'UTR;5'UTR;5'UTR	N_Shelf	37.91	6.63	43.70	6.97	-5.79	5.45E-04	1.08E-02
cg13670316	PCSK5	9	Body	Island	25.30	5.60	31.09	7.48	-5.79	1.61E-05	5.75E-04
cg09984479	TMEM184B	22	Body	Open sea	31.35	7.00	37.13	7.48	-5.78	1.31E-03	2.10E-02

cg21868798		1	Intergenic	Open sea	49.11	5.94	54.89	5.82	-5.78	2.68E-05	9.05E-04
cg09422291	MCOLN1	19	TSS1500	N_Shore	38.37	7.07	44.15	7.43	-5.78	1.63E-05	5.84E-04
cg20623506	P4HB	17	Body	Open sea	39.84	5.50	45.62	5.21	-5.78	3.46E-05	1.13E-03
cg25332918		7	Intergenic	N_Shore	72.26	9.17	78.04	8.68	-5.78	7.10E-04	1.32E-02
cg20803796		8	Intergenic	Island	43.34	7.30	49.12	8.35	-5.78	7.55E-04	1.39E-02
cg07322898	SFRS12IP1	5	Body	Open sea	52.51	5.49	58.28	5.87	-5.78	2.43E-05	8.30E-04
cg04154027	CMYA5	5	TSS200	Open sea	28.37	5.82	34.15	6.49	-5.78	2.51E-04	5.93E-03
cg14905634	TRHDE	12	3'UTR	Open sea	47.01	8.32	52.78	7.92	-5.78	5.63E-04	1.11E-02
cg00152878	PRDM16;PRDM16	1	Body;Body	Open sea	77.06	9.23	82.83	6.70	-5.78	1.28E-03	2.06E-02
cg23983887	VPS13D;VPS13D	1	Body;Body	Open sea	50.29	6.54	56.06	6.94	-5.78	5.30E-06	2.11E-04
cg01790464	SLC7A8;SLC7A8	14	Body;TSS1500	Open sea	37.11	4.21	42.88	5.09	-5.77	1.06E-07	5.27E-06
cg08197287	AFF1;AFF1	4	Body;Body	Open sea	56.18	6.56	61.95	4.86	-5.77	1.46E-05	5.28E-04
cg17795240	GATA4	8	Body	S_Shore	42.20	5.26	47.98	4.14	-5.77	1.58E-07	7.75E-06
cg06691520	LGI1;LGI1	10	5'UTR;1stExon	Open sea	50.25	6.77	56.02	7.02	-5.77	3.21E-04	7.18E-03
cg11838224	TNK1	17	Body	Island	34.50	5.84	40.28	6.45	-5.77	1.40E-06	6.14E-05
cg07683388	ANKRD44	2	Body	Island	40.61	4.40	46.38	5.11	-5.77	1.25E-07	6.19E-06
cg02283485	ZMIZ1	10	Body	Island	38.22	4.40	43.99	4.18	-5.77	2.46E-08	1.28E-06
cg21618730		13	Intergenic	N_Shore	49.09	6.59	54.86	6.49	-5.77	8.29E-07	3.76E-05
cg02019988	SEMA3B;SEMA3B	3	Body;Body	Island	22.58	5.78	28.35	7.13	-5.77	1.89E-04	4.73E-03
cg01212284	MCCC1	3	Body	N_Shore	73.33	6.27	79.10	6.17	-5.77	2.68E-04	6.23E-03
cg15341350	SRRM4	12	Body	Open sea	40.65	7.72	46.42	8.46	-5.77	2.13E-04	5.20E-03
cg02731193	GCLC	6	Body	N_Shore	57.35	7.06	63.12	6.86	-5.77	2.61E-03	3.40E-02
cg06637618	ATP2B4;ATP2B4	1	TSS1500;TSS1500	N_Shelf	32.99	3.97	38.75	3.67	-5.77	4.12E-10	2.33E-08
cg16054280	DIRC2	3	Body	S_Shelf	49.50	5.30	55.27	6.43	-5.76	1.97E-04	4.88E-03
cg14051973	MRPL23	11	Body	N_Shore	45.39	6.03	51.15	7.00	-5.76	5.23E-04	1.05E-02
cg00338996	SLC35C1;SLC35C1;SLC35C1	11	Body;Body;Body	S_Shore	45.66	8.76	51.42	8.54	-5.76	3.78E-03	4.39E-02
cg05049638		1	Intergenic	N_Shelf	72.89	4.90	78.65	5.82	-5.76	8.03E-07	3.65E-05
cg00454305	UNKL	16	TSS1500	Island	53.42	5.48	59.18	6.33	-5.76	6.88E-05	2.04E-03
cg24859722	RASSF1;RASSF1	3	Body;Body	Island	52.60	4.29	58.36	5.18	-5.76	1.97E-08	1.03E-06
cg04850976	CREBBP;CREBBP	16	Body;Body	S_Shore	50.10	5.26	55.86	5.01	-5.76	4.72E-06	1.90E-04
cg03188000		12	Intergenic	Open sea	24.73	9.00	30.49	9.22	-5.76	3.09E-03	3.82E-02
cg22681709	PDE11A;PDE11A;PDE11A;PDE11A	2	Body;Body;Body;Body	Open sea	59.41	9.42	65.17	8.12	-5.76	1.46E-03	2.26E-02
cg06100639		7	Intergenic	S_Shelf	46.35	6.39	52.11	6.72	-5.76	2.85E-04	6.54E-03
cg22327312	DLG2;DLG2;DLG2	11	TSS1500;Body;Body	Open sea	74.02	4.89	79.78	3.45	-5.76	8.19E-09	4.38E-07
cg16757984	NR1D1	17	TSS1500	S_Shore	60.49	6.27	66.25	6.96	-5.76	2.98E-04	6.76E-03
cg13458651	WNT7B	22	Body	Island	10.50	3.56	16.26	8.33	-5.76	2.94E-05	9.80E-04
cg26823666		1	Intergenic	N_Shore	29.38	7.67	35.14	6.93	-5.75	8.23E-04	1.48E-02
cg19993680		10	Intergenic	Open sea	47.04	6.33	52.79	6.60	-5.75	5.31E-04	1.06E-02
cg25012529	F10	13	Body	Open sea	39.88	6.89	45.63	5.19	-5.75	2.54E-05	8.65E-04
cg00796569	GABRA5;GABRA5	15	TSS1500;TSS1500	N_Shore	38.38	5.14	44.13	7.54	-5.75	1.68E-04	4.29E-03
cg10077239	PRKD1	14	TSS1500	S_Shore	34.68	4.82	40.43	7.56	-5.75	9.46E-05	2.67E-03
cg23415219	LOC285768;LOC285768	6	Body;Body	Open sea	66.53	6.21	72.28	7.77	-5.75	3.25E-04	7.24E-03
cg18157012	SLIT3	5	Body	Open sea	56.93	7.46	62.68	8.95	-5.75	4.03E-03	4.59E-02
cg10933494		6	Intergenic	N_Shore	45.82	6.13	51.57	6.24	-5.75	3.51E-05	1.14E-03
cg26310429	SLC39A11;SLC39A11	17	Body;Body	Open sea	64.52	5.70	70.26	6.75	-5.75	1.29E-04	3.45E-03
cg21970261	TFF3	21	TSS200	Open sea	63.01	8.49	68.76	6.35	-5.75	2.62E-04	6.13E-03
cg00287096		7	Intergenic	Island	36.39	6.61	42.14	8.26	-5.75	4.92E-04	9.99E-03
cg16786756		4	Intergenic	Island	66.60	8.49	72.34	7.89	-5.75	1.58E-03	2.39E-02
cg23968286		17	Intergenic	Open sea	72.66	4.39	78.41	3.21	-5.75	2.88E-10	1.64E-08
cg19199204	HCG9	6	Body	N_Shore	39.80	8.77	45.54	6.98	-5.75	1.31E-03	2.09E-02
cg16016281		5	Intergenic	Open sea	32.08	6.68	37.82	7.78	-5.74	4.12E-03	4.66E-02
cg02301920	CES1;CES1;CES1	16	Body;Body;Body	Island	33.36	8.64	39.10	8.12	-5.74	1.91E-03	2.74E-02
cg03453431		7	Intergenic	Open sea	33.13	7.16	38.87	9.05	-5.74	4.45E-03	4.91E-02

cg22854448	SPRED2	2	Body	Open sea	66.99	4.99	72.74	3.59	-5.74	4.13E-08	2.12E-06
cg24533804	LOC144571;A2M	12	Body;3'UTR	S_Shelf	57.56	5.19	63.30	5.32	-5.74	2.34E-06	9.91E-05
cg00555538	SSH2	17	Body	Island	20.51	6.74	26.25	8.42	-5.74	3.24E-05	1.07E-03
cg10972932		11	Intergenic	Open sea	59.66	5.06	65.40	4.66	-5.74	1.73E-06	7.50E-05
cg03357999		7	Intergenic	N_Shore	53.41	6.73	59.15	7.01	-5.74	3.13E-04	7.04E-03
cg16718710		4	Intergenic	Open sea	73.97	4.85	79.71	4.96	-5.74	5.19E-07	2.42E-05
cg23526676	C6orf27	6	Body	Open sea	36.94	4.46	42.68	5.83	-5.74	3.74E-06	1.53E-04
cg24160421		8	Intergenic	N_Shore	30.89	6.37	36.63	7.92	-5.74	3.61E-04	7.87E-03
cg21560076	EXPH5;EXPH5	11	TSS200;Body	Open sea	60.47	5.22	66.21	5.39	-5.74	1.42E-05	5.15E-04
cg25873875	NCOR1	17	Body	Open sea	61.15	6.28	66.89	8.29	-5.74	9.10E-04	1.60E-02
cg11256607	DAB1	1	TSS200	S_Shore	24.95	7.41	30.69	5.78	-5.74	8.48E-04	1.51E-02
cg12507643		12	Intergenic	Open sea	31.73	5.80	37.46	8.59	-5.74	1.40E-03	2.19E-02
cg11074933	GABRB3;GABRB3	15	Body;Body	Island	43.81	7.57	49.55	8.31	-5.73	3.60E-03	4.24E-02
cg02147980		3	Intergenic	N_Shelf	76.40	5.37	82.14	5.02	-5.73	6.07E-06	2.39E-04
cg07134316	NAB2	12	Body	Open sea	35.08	6.64	40.82	5.43	-5.73	3.52E-05	1.15E-03
cg07654982	EFHC1	6	TSS1500	N_Shore	36.17	5.04	41.90	5.82	-5.73	4.75E-07	2.22E-05
cg20671649	PRDM16;PRDM16	1	Body;Body	N_Shore	20.46	5.98	26.19	9.67	-5.73	1.91E-03	2.74E-02
cg01738406	VSIG2	11	Body	S_Shelf	43.79	6.62	49.52	8.79	-5.73	1.25E-03	2.02E-02
cg03729251	LRBA	4	Body	Island	33.74	7.69	39.47	5.81	-5.73	3.69E-04	8.00E-03
cg01755541	TPPP3;TPPP3;ZDHHC1	16	TSS1500;TSS1500;3'UTR	Island	46.14	6.15	51.87	8.90	-5.73	1.22E-03	1.99E-02
cg15111638		11	Intergenic	N_Shore	42.04	6.67	47.77	9.59	-5.73	2.31E-03	3.13E-02
cg11940526		10	Intergenic	Open sea	42.98	4.69	48.71	6.03	-5.73	7.67E-06	2.96E-04
cg19898448	ANKRD50;ANKRD50	4	5'UTR;Body	N_Shore	62.24	6.61	67.97	4.90	-5.73	1.13E-04	3.10E-03
cg24724917	SLC22A18;SLC22A18;SLC22A18AS	11	Body;Body;TSS1500	S_Shore	57.92	8.38	63.64	8.17	-5.73	4.82E-04	9.84E-03
cg14270293	SH3D19;SH3D19	4	TSS1500;TSS1500	Open sea	56.54	6.00	62.26	4.92	-5.72	9.65E-06	3.65E-04
cg21288685	TTLL10	1	TSS200	N_Shore	11.66	5.35	17.38	8.96	-5.72	3.04E-04	6.88E-03
cg05275229	OLFM2	19	Body	Island	33.34	7.28	39.07	6.46	-5.72	4.19E-04	8.84E-03
cg03989617	GPR56;GPR56;GPR56;GPR56;GPR56;G	16	5'UTR;1stExon;5'UTR;1stExon;1stExon;5'U	Open sea	35.56	5.20	41.28	6.82	-5.72	2.52E-05	8.57E-04
cg19935951	LRBA	4	Body	Open sea	65.27	6.04	70.99	5.14	-5.72	2.16E-05	7.48E-04
cg11642412	MACROD1	11	Body	Island	38.92	5.15	44.65	5.14	-5.72	1.00E-06	4.50E-05
cg19827780	RAPGEFL1	17	Body	Island	5.64	3.34	11.36	6.95	-5.72	4.04E-06	1.64E-04
cg12069309	SEMA3B;SEMA3B	3	Body;Body	Island	10.13	4.58	15.85	6.60	-5.72	3.58E-06	1.47E-04
cg21942770		13	Intergenic	Island	23.89	5.46	29.62	8.92	-5.72	5.40E-04	1.08E-02
cg09161579	CSF3;CSF3;CSF3	17	3'UTR;3'UTR;3'UTR	Open sea	40.27	4.68	45.99	4.60	-5.72	1.41E-06	6.19E-05
cg27530576	SYNGAP1	6	Body	Open sea	43.30	5.50	49.02	5.63	-5.72	1.77E-05	6.28E-04
cg14413584		9	Intergenic	S_Shore	34.05	5.65	39.77	7.19	-5.72	2.99E-04	6.78E-03
cg08237971		1	Intergenic	S_Shelf	48.91	7.45	54.63	9.35	-5.72	3.03E-03	3.77E-02
cg26263664	CASKIN2;CASKIN2	17	Body;Body	Island	65.18	4.96	70.90	3.20	-5.72	8.63E-08	4.32E-06
cg17867243	PLA2G4D	15	Body	Island	67.55	7.00	73.27	7.41	-5.72	9.30E-04	1.62E-02
cg07592254	NKAIN3	8	Body	Open sea	35.75	5.42	41.47	5.33	-5.72	4.29E-05	1.36E-03
cg18700428		16	Intergenic	Island	56.10	7.19	61.81	7.17	-5.72	1.75E-03	2.57E-02
cg12406992	MEX3A	1	Body	S_Shore	57.25	6.68	62.97	6.63	-5.72	1.04E-03	1.76E-02
cg18991862	MAD1L1;MAD1L1;MAD1L1	7	Body;Body;Body	S_Shelf	75.56	6.57	81.28	5.85	-5.72	1.75E-04	4.45E-03
cg15449049	MITF;MITF	3	TSS1500;Body	Open sea	63.46	5.53	69.18	5.69	-5.72	3.29E-06	1.36E-04
cg23620279	RPS24;RPS24;RPS24;RPS24;RPS24;RPS	10	TSS200;TSS200;TSS200;TSS200;TSS200;TSS	Island	23.11	4.93	28.83	6.93	-5.72	2.65E-05	8.97E-04
cg00507945		5	Intergenic	N_Shore	25.41	4.50	31.12	6.15	-5.72	3.78E-07	1.79E-05
cg02038233	DUOX1;DUOX1	15	Body;Body	N_Shelf	69.10	6.31	74.82	8.32	-5.72	1.36E-03	2.15E-02
cg13334650	APBB1;APBB1	11	5'UTR;5'UTR	Island	28.33	7.36	34.05	9.80	-5.72	1.62E-04	4.17E-03
cg17035899	MYL5	4	Body	Island	15.37	5.93	21.09	7.57	-5.72	2.69E-04	6.26E-03
cg01811815	RASA3	13	Body	S_Shore	42.23	6.67	47.94	6.10	-5.72	3.76E-05	1.21E-03
cg26099837	AKT1;AKT1;AKT1	14	Body;Body;Body	S_Shelf	38.40	6.64	44.11	8.54	-5.71	4.09E-03	4.63E-02
cg04709306	IQGAP2	5	Body	S_Shelf	71.03	6.34	76.75	5.46	-5.71	9.40E-05	2.66E-03
cg09550793	SLC12A4;SLC12A4;SLC12A4;SLC12A4;S	16	Body;Body;TSS1500;Body;Body	N_Shelf	59.07	5.42	64.78	5.57	-5.71	1.16E-05	4.31E-04

cg04044120	DNAJB13	11	Body	Open sea	64.89	8.67	70.60	8.80	-5.71	3.46E-03	4.14E-02
cg19373545	C1orf113;C1orf113	1	TSS1500;TSS200	N_Shore	58.36	5.89	64.07	5.65	-5.71	1.79E-04	4.51E-03
cg04563946	PLIN4	19	Body	Open sea	48.74	5.51	54.45	6.66	-5.71	1.14E-04	3.13E-03
cg15695155	KDM2B;KDM2B	12	Body;Body	N_Shore	23.66	5.16	29.37	5.73	-5.71	6.37E-05	1.92E-03
cg14843030	LCK	1	TSS1500	S_Shore	49.99	4.03	55.70	5.26	-5.71	8.98E-08	4.49E-06
cg07694621		2	Intergenic	Open sea	61.66	7.50	67.37	5.90	-5.71	4.99E-05	1.55E-03
cg08838842	PHLDB2;PHLDB2;PHLDB2;PHLDB2	3	TSS1500;Body;TSS1500;TSS1500	N_Shore	41.52	6.79	47.22	8.46	-5.71	4.41E-03	4.88E-02
cg24960291	IGF2BP2;IGF2BP2	3	Body;Body	N_Shelf	57.00	5.32	62.70	6.42	-5.70	5.02E-05	1.56E-03
cg01475325	TSKU	11	5'UTR	S_Shelf	53.09	6.89	58.80	6.67	-5.70	4.84E-04	9.87E-03
cg23484655		15	Intergenic	Island	78.57	5.04	84.27	4.06	-5.70	9.23E-07	4.17E-05
cg03138364	PBX1	1	Body	Open sea	80.78	5.10	86.49	3.63	-5.70	2.84E-09	1.56E-07
cg07974511	OTX1	2	Body	Island	28.28	6.64	33.98	6.89	-5.70	2.55E-04	6.01E-03
cg07704331	ZNF341	20	3'UTR	S_Shore	50.61	5.57	56.31	5.46	-5.70	7.25E-06	2.81E-04
cg09503045	EPS8L1;EPS8L1	19	Body;Body	Island	19.07	4.05	24.77	6.64	-5.70	4.95E-07	2.31E-05
cg11656175	PPFIA4	1	Body	N_Shelf	58.66	5.80	64.36	6.40	-5.70	2.22E-05	7.65E-04
cg25435917		3	Intergenic	Island	32.48	4.23	38.18	5.28	-5.70	1.21E-09	6.75E-08
cg00727310	IRS1	2	1stExon	Island	36.31	6.22	42.01	8.10	-5.70	2.03E-03	2.86E-02
cg21787063	ASB4;ASB4	7	TSS200;TSS200	Open sea	75.73	6.26	81.43	5.55	-5.70	3.58E-05	1.17E-03
cg06121226	SLC4A4;SLC4A4	4	Body;Body	Open sea	43.67	6.80	49.37	6.62	-5.69	2.31E-04	5.55E-03
cg02884773	SMAD1;SMAD1	4	Body;Body	Open sea	69.38	5.53	75.08	5.52	-5.69	4.94E-06	1.98E-04
cg10951117		8	Intergenic	Open sea	62.65	6.03	68.34	5.66	-5.69	3.26E-05	1.07E-03
cg03631065	UGGT2	13	Body	Open sea	53.25	5.15	58.94	4.85	-5.69	1.27E-05	4.65E-04
cg20947514		1	Intergenic	S_Shelf	63.95	6.83	69.64	4.71	-5.69	2.58E-04	6.06E-03
cg15236483		22	Intergenic	Island	30.24	6.09	35.93	6.73	-5.69	1.15E-03	1.90E-02
cg15247974		19	Intergenic	Island	48.23	6.03	53.92	7.69	-5.69	1.99E-03	2.82E-02
cg02594426		15	Intergenic	S_Shelf	56.77	7.37	62.46	7.33	-5.69	1.54E-03	2.35E-02
cg08952306	SH2B2	7	3'UTR	Island	52.46	6.36	58.15	6.80	-5.69	1.97E-04	4.89E-03
cg22389949		6	Intergenic	Open sea	25.76	4.69	31.45	5.64	-5.69	1.79E-05	6.35E-04
cg19493250	ELL2	5	Body	Open sea	82.52	5.19	88.21	4.34	-5.69	8.07E-07	3.67E-05
cg04240491	LCLAT1;LCLAT1	2	Body;Body	Open sea	60.59	6.69	66.28	6.50	-5.69	1.17E-03	1.93E-02
cg25034424		4	Intergenic	N_Shore	79.34	8.57	85.03	3.14	-5.69	6.15E-04	1.19E-02
cg17517737	ZNF468;ZNF468	19	5'UTR;5'UTR	N_Shore	67.89	6.62	73.57	5.98	-5.69	6.14E-05	1.85E-03
cg23046919	IGF1;IGF1;IGF1	12	3'UTR;3'UTR;3'UTR	Open sea	47.76	5.56	53.45	5.80	-5.69	3.02E-06	1.25E-04
cg20798604	COL11A2;COL11A2;COL11A2	6	Body;Body;Body	Open sea	44.91	4.98	50.60	6.57	-5.69	6.89E-06	2.68E-04
cg00920789		7	Intergenic	Island	56.21	3.74	61.90	3.86	-5.69	4.72E-10	2.67E-08
cg01215442	GDF1;LASS1	19	Body;3'UTR	Island	22.44	5.15	28.12	9.60	-5.69	1.63E-03	2.44E-02
cg24549659		4	Intergenic	Island	35.67	5.77	41.35	7.11	-5.69	1.79E-04	4.52E-03
cg08698605	LASS4	19	TSS1500	Island	24.10	4.41	29.78	4.57	-5.69	5.32E-08	2.71E-06
cg17485838		7	Intergenic	S_Shore	27.78	5.51	33.46	6.01	-5.69	4.11E-05	1.31E-03
cg07085177	WDR81;WDR81;WDR81;WDR81	17	Body;1stExon;5'UTR;5'UTR	S_Shore	51.52	6.17	57.21	7.72	-5.68	1.36E-03	2.15E-02
cg03157283	MRPL23	11	Body	S_Shore	62.48	5.22	68.16	3.57	-5.68	7.44E-07	3.40E-05
cg13753488	RALGDS	9	Body	Open sea	16.02	5.64	21.70	5.94	-5.68	9.32E-05	2.64E-03
cg01858089		4	Intergenic	Open sea	46.35	5.25	52.03	7.31	-5.68	2.09E-04	5.12E-03
cg12262070		11	Intergenic	N_Shore	71.43	5.29	77.11	5.05	-5.68	7.78E-07	3.55E-05
cg24082871	SEZ6;SEZ6	17	TSS1500;TSS1500	S_Shore	17.86	4.71	23.54	6.46	-5.68	5.19E-07	2.42E-05
cg06098530	MYST4	10	Body	Open sea	20.08	4.52	25.76	5.41	-5.68	4.39E-06	1.78E-04
cg01057552	OBSCN;OBSCN	1	Body;Body	Open sea	30.53	5.10	36.21	7.97	-5.68	2.39E-04	5.69E-03
cg15459822	PARD3B;PARD3B;PARD3B	2	Body;Body;Body	Open sea	58.97	6.75	64.65	7.31	-5.68	4.08E-05	1.30E-03
cg19427345	MFSD7	4	Body	Island	47.39	5.70	53.07	8.16	-5.68	1.22E-03	1.99E-02
cg04382108	COL27A1	9	Body	Island	79.66	5.66	85.33	4.05	-5.68	2.37E-06	1.00E-04
cg04799778	EIF5A2	3	3'UTR	Open sea	77.74	6.80	83.42	6.18	-5.68	7.45E-05	2.18E-03
cg22771960	MIR637;DAPK3	19	TSS200;Body	S_Shore	41.22	4.53	46.90	5.14	-5.68	5.44E-07	2.53E-05
cg02177444		2	Intergenic	Open sea	66.59	6.05	72.26	6.89	-5.68	5.73E-04	1.13E-02

cg17264271	ESR1;ESR1;ESR1	6	5'UTR;1stExon;5'UTR	N_Shore	32.67	5.22	38.35	5.62	-5.68	1.76E-05	6.25E-04
cg09075844	SLC6A18	5	Body	Island	40.89	5.84	46.56	6.49	-5.67	1.78E-04	4.50E-03
cg20652899	FXYD6;FXYD6;FXYD6;FXYD6;FXYD6	11	5'UTR;TSS200;TSS200;TSS200;TSS200	S_Shore	39.84	5.23	45.52	8.15	-5.67	4.37E-04	9.14E-03
cg15157024	KCTD6	3	5'UTR	S_Shelf	55.64	6.79	61.32	7.02	-5.67	6.64E-04	1.26E-02
cg14123607	APBA1	9	5'UTR	Open sea	26.36	6.44	32.03	6.68	-5.67	2.78E-05	9.34E-04
cg13798384	PCDH9;PCDH9	13	Body;Body	Open sea	57.96	6.19	63.63	6.17	-5.67	7.72E-05	2.25E-03
cg02914827	MMP21	10	Body	Island	55.08	5.28	60.75	5.33	-5.67	4.58E-06	1.85E-04
cg04844207	LDLRAD3	11	Body	Open sea	72.46	4.87	78.13	5.05	-5.67	5.60E-07	2.60E-05
cg17780731		5	Intergenic	Open sea	25.81	6.10	31.48	6.81	-5.67	7.06E-05	2.09E-03
cg14281039	TNNI3	19	TSS200	S_Shore	64.70	6.07	70.38	6.39	-5.67	6.92E-05	2.05E-03
cg10169241	APC2	19	Body	Island	36.61	8.00	42.27	7.31	-5.67	7.50E-04	1.38E-02
cg00377915	STOX1;STOX1;STOX1;STOX1;STOX1	10	5'UTR;Body;Body;Body;Body	Open sea	46.62	4.22	52.28	4.77	-5.67	8.38E-08	4.20E-06
cg08305551	MAST1	19	Body	Island	21.83	6.84	27.50	7.99	-5.67	2.66E-05	8.99E-04
cg12977346		8	Intergenic	Open sea	53.52	7.96	59.18	8.18	-5.67	3.31E-04	7.35E-03
cg01575601		11	Intergenic	Open sea	32.77	4.95	38.43	7.20	-5.67	1.54E-05	5.53E-04
cg11027879	HSD17B4	5	Body	Open sea	68.23	6.44	73.89	7.03	-5.67	1.41E-03	2.21E-02
cg15580075	NCRNA00167;PRDM10;PRDM10	11	TSS1500;5'UTR;5'UTR	N_Shore	43.86	6.98	49.53	8.12	-5.67	1.78E-03	2.61E-02
cg22488259	NAV1;NAV1	1	TSS1500;Body	N_Shore	44.36	5.67	50.02	6.98	-5.66	1.90E-04	4.74E-03
cg23630124		10	Intergenic	Open sea	57.89	5.62	63.55	5.92	-5.66	7.55E-05	2.21E-03
cg13580196	PDE8A;PDE8A	15	Body;Body	S_Shelf	63.68	6.87	69.34	6.35	-5.66	5.09E-04	1.03E-02
cg11749828	SCN1B;HPN;HPN	19	3'UTR;TSS1500;TSS1500	N_Shore	31.32	4.16	36.98	5.20	-5.66	3.07E-07	1.47E-05
cg10562586	MAP2;MAP2;MAP2;MAP2	2	TSS1500;TSS1500;TSS1500;5'UTR	Open sea	52.79	7.10	58.45	7.92	-5.66	2.94E-04	6.69E-03
cg12548022	C2orf72	2	3'UTR	N_Shelf	61.58	5.08	67.24	7.16	-5.66	8.27E-05	2.39E-03
cg10135260		4	Intergenic	Island	17.11	5.54	22.77	8.44	-5.66	2.24E-04	5.42E-03
cg08995609	RIN1	11	TSS200	S_Shore	36.76	6.33	42.42	5.75	-5.66	7.58E-06	2.93E-04
cg17888219	DEFB127	20	Body	Open sea	43.47	6.96	49.13	8.09	-5.66	2.59E-04	6.07E-03
cg13674486		7	Intergenic	Open sea	36.10	5.06	41.76	7.15	-5.66	5.37E-05	1.65E-03
cg10857427	ERP27	12	TSS200	Open sea	47.36	6.58	53.01	9.10	-5.66	4.15E-03	4.68E-02
cg14014955	HOXA5	7	TSS1500	Island	35.58	6.48	41.24	5.27	-5.66	8.86E-05	2.53E-03
cg22898362	KLK9;KLK8;KLK8;KLK8;KLK8	19	3'UTR;TSS1500;TSS1500;TSS1500;TSS1500	Island	73.13	8.59	78.78	7.00	-5.66	6.29E-04	1.21E-02
cg21399717		8	Intergenic	Open sea	46.60	4.07	52.25	4.44	-5.66	9.02E-08	4.51E-06
cg09206294	MAPKBP1;MAPKBP1	15	Body;Body	Open sea	50.15	5.27	55.81	5.74	-5.66	1.01E-05	3.82E-04
cg06163274	CCDC68	18	TSS1500	S_Shore	50.47	5.14	56.12	5.62	-5.65	1.15E-05	4.28E-04
cg17089062		4	Intergenic	N_Shore	32.08	7.18	37.73	9.45	-5.65	2.61E-04	6.10E-03
cg25134328		7	Intergenic	Open sea	24.85	4.30	30.50	4.25	-5.65	8.86E-09	4.73E-07
cg25393494	PLD6	17	TSS1500	Island	21.21	5.59	26.86	8.01	-5.65	6.65E-04	1.26E-02
cg04918350	TG	8	Body	Open sea	58.89	6.88	64.54	8.35	-5.65	2.54E-03	3.34E-02
cg27514038	NIPA2;NIPA2;NIPA2;NIPA2	15	TSS1500;TSS1500;TSS1500;TSS1500	S_Shore	74.52	8.11	80.17	7.80	-5.65	4.31E-04	9.03E-03
cg15964523	SPTBN2	11	Body	S_Shore	69.86	5.21	75.51	4.58	-5.65	4.21E-06	1.71E-04
cg01335127	ACPL2;ACPL2	3	5'UTR;5'UTR	Open sea	74.85	5.06	80.50	5.88	-5.65	3.86E-05	1.24E-03
cg18034299	ERGIC1	5	Body	Open sea	38.64	4.29	44.30	4.63	-5.65	7.06E-08	3.56E-06
cg13042575	RASSF3	12	Body	Open sea	29.57	8.26	35.22	7.61	-5.65	2.24E-05	7.74E-04
cg00318436	PARD6A;ACD;ACD;PARD6A;ACD	16	Body;TSS1500;TSS1500;Body;TSS1500	S_Shore	47.57	5.47	53.22	7.49	-5.65	8.54E-04	1.52E-02
cg12619347	SLC43A1	11	Body	N_Shore	48.54	5.11	54.19	6.05	-5.65	1.11E-05	4.15E-04
cg04705659	PLEKHA6	1	TSS200	Open sea	29.46	4.75	35.11	4.73	-5.65	6.22E-06	2.44E-04
cg27313776	ZNF827	4	Body	Open sea	56.59	4.92	62.24	5.43	-5.65	5.64E-06	2.23E-04
cg21302480	AGBL4	1	Body	N_Shelf	44.96	5.40	50.60	7.33	-5.65	3.74E-04	8.08E-03
cg07959490	CCDC57	17	Body	Open sea	54.91	6.42	60.55	7.89	-5.65	1.17E-03	1.93E-02
cg21865657	PIP5K1C	19	Body	N_Shore	63.87	4.72	69.51	4.82	-5.64	1.53E-09	8.47E-08
cg01236063		11	Intergenic	N_Shore	43.15	6.19	48.79	7.52	-5.64	7.63E-05	2.23E-03
cg15622891	QKI;QKI;QKI;QKI	6	Body;Body;Body;Body	Open sea	54.35	5.70	60.00	5.81	-5.64	1.78E-04	4.49E-03
cg11957261		8	Intergenic	Open sea	57.08	5.82	62.72	6.14	-5.64	2.34E-05	8.04E-04
cg01303236	ENC1	5	5'UTR	N_Shore	46.16	6.72	51.80	6.69	-5.64	1.20E-03	1.96E-02

cg12093443	CSMD3;CSMD3;CSMD3	8	Body;Body;Body	Open sea	55.29	4.80	60.93	4.18	-5.64	9.55E-07	4.30E-05
cg20668096	LRBA	4	Body	N_Shore	45.32	5.39	50.96	4.75	-5.64	1.43E-05	5.21E-04
cg01243513	DTX3;GEFT	12	3'UTR;TSS1500	N_Shore	35.93	4.46	41.57	7.63	-5.64	5.31E-05	1.64E-03
cg11703729	FRMD4A	10	Body	Open sea	44.70	6.69	50.33	8.13	-5.64	1.55E-03	2.36E-02
cg20784841	CFHR4	1	Body	Open sea	42.43	6.75	48.07	8.56	-5.64	2.87E-04	6.56E-03
cg09422614	CACNG8;MIR935	19	Body;TSS1500	N_Shore	62.75	5.96	68.39	5.60	-5.64	1.41E-04	3.71E-03
cg11248999	EPHB2;EPHB2	1	Body;Body	Open sea	56.47	6.36	62.11	7.69	-5.64	8.56E-04	1.52E-02
cg23287992	C6orf27	6	Body	Open sea	55.42	5.10	61.06	4.41	-5.64	4.43E-06	1.79E-04
cg16592301	PRKD1	14	Body	Open sea	81.09	6.96	86.73	4.86	-5.64	1.38E-06	6.07E-05
cg26317318		15	Intergenic	S_Shelf	60.50	4.71	66.14	4.72	-5.64	1.33E-06	5.88E-05
cg24030370	TRIM13;TRIM13;TRIM13;TRIM13;DLE	13	TSS1500;TSS1500;TSS1500;TSS1500;Body	N_Shore	21.70	7.41	27.34	8.46	-5.64	4.84E-04	9.87E-03
cg12432580	CNTNAP1	17	Body	N_Shore	61.78	5.75	67.41	5.66	-5.64	8.98E-05	2.56E-03
cg16523839	COL5A3	19	Body	Island	37.91	9.54	43.54	8.66	-5.63	2.77E-03	3.54E-02
cg10649706	EIF2C2;EIF2C2	8	Body;Body	Open sea	60.62	4.04	66.25	5.01	-5.63	1.12E-07	5.56E-06
cg18970338	VIL1	2	TSS200	Open sea	39.26	5.21	44.89	4.72	-5.63	2.78E-06	1.16E-04
cg27428304	SLC22A20	11	Body	Island	6.95	4.45	12.58	8.29	-5.63	1.14E-05	4.24E-04
cg21613754	AAMP;PNKD;PNKD	2	Body;TSS1500;TSS1500	N_Shore	48.04	5.73	53.68	6.66	-5.63	3.52E-04	7.72E-03
cg06098368		7	Intergenic	Island	14.59	6.40	20.22	7.35	-5.63	1.74E-04	4.43E-03
cg23907926	STX2;STX2	12	Body;Body	Island	15.89	4.78	21.52	5.98	-5.63	4.11E-07	1.94E-05
cg22352772		7	Intergenic	Island	10.13	4.07	15.76	7.69	-5.63	1.13E-05	4.22E-04
cg10562786	MICAL2	11	Body	Open sea	74.41	7.67	80.04	4.99	-5.63	7.72E-04	1.41E-02
cg27166581		1	Intergenic	S_Shore	33.99	6.28	39.62	9.15	-5.63	3.20E-03	3.92E-02
cg19664013		6	Intergenic	S_Shelf	32.10	6.20	37.73	5.99	-5.63	8.10E-06	3.11E-04
cg18315060		6	Intergenic	Island	36.14	6.27	41.76	9.42	-5.63	2.44E-03	3.25E-02
cg24161925		6	Intergenic	Island	35.74	4.55	41.37	4.59	-5.63	1.93E-07	9.40E-06
cg27287944	MIDN	19	Body	Island	58.69	6.89	64.31	6.49	-5.62	1.81E-03	2.63E-02
cg27630771	ADAMTSL4	1	3'UTR	N_Shore	53.64	3.70	59.27	4.44	-5.62	3.89E-08	2.00E-06
cg22259536	OSBPL10	3	Body	Open sea	66.96	6.01	72.58	5.73	-5.62	1.45E-05	5.25E-04
cg07460992	ZCCHC14	16	Body	Open sea	31.24	5.97	36.86	7.44	-5.62	1.46E-04	3.82E-03
cg13549497		6	Intergenic	Island	70.69	6.64	76.32	7.74	-5.62	1.19E-03	1.95E-02
cg08271745	HDGF2;HDGF2	19	Body;Body	N_Shelf	72.33	7.36	77.96	6.38	-5.62	3.17E-03	3.89E-02
cg04785213	LYZ	12	Body	Open sea	71.56	5.97	77.18	6.71	-5.62	1.41E-05	5.12E-04
cg01422274	TSPAN4;TSPAN4;TSPAN4;TSPAN4;TSP	11	5'UTR;5'UTR;5'UTR;5'UTR;5'UTR;5'UTR;5'U	Island	45.58	6.98	51.20	7.08	-5.62	6.31E-04	1.21E-02
cg24513387		8	Intergenic	Open sea	32.11	5.43	37.73	5.96	-5.62	1.66E-05	5.93E-04
cg13988338		13	Intergenic	S_Shore	42.30	7.49	47.92	6.83	-5.62	1.96E-04	4.85E-03
cg06365623		2	Intergenic	Open sea	35.80	5.41	41.42	5.16	-5.62	1.03E-04	2.87E-03
cg19893494	NSMCE2	8	Body	Open sea	62.39	5.95	68.01	4.30	-5.62	3.02E-06	1.26E-04
cg06947614	SLCO1B3	12	5'UTR	Open sea	81.47	6.81	87.09	5.48	-5.62	6.66E-05	1.99E-03
cg13600201		9	Intergenic	N_Shore	46.94	5.78	52.55	6.58	-5.62	2.22E-04	5.38E-03
cg21682210		1	Intergenic	N_Shelf	72.30	5.40	77.92	6.30	-5.62	1.12E-04	3.08E-03
cg05193369	CYP26C1	10	Body	Island	22.05	3.74	27.66	5.67	-5.62	1.02E-08	5.44E-07
cg03704667	CHTF8;CHTF8;CHTF8;HAS3;CHTF8	16	3'UTR;3'UTR;Body;3'UTR;3'UTR	Open sea	89.34	2.25	94.95	1.03	-5.62	5.78E-32	5.17E-30
cg11843623		6	Intergenic	Open sea	51.88	4.84	57.50	5.51	-5.62	7.43E-07	3.40E-05
cg14906909	ACSS3	12	Body	Open sea	66.49	5.73	72.10	3.39	-5.61	5.89E-06	2.32E-04
cg08144675		16	Intergenic	Island	47.29	6.43	52.90	6.02	-5.61	2.13E-04	5.19E-03
cg23445321	SLC35F3	1	Body	Island	70.70	8.58	76.31	7.93	-5.61	2.91E-03	3.66E-02
cg07600533	KLHDC7B	22	TSS1500	Island	50.86	8.87	56.47	7.22	-5.61	1.06E-03	1.78E-02
cg04899225		7	Intergenic	Open sea	37.84	4.50	43.45	7.79	-5.61	4.62E-04	9.53E-03
cg05129050	CAMTA1	1	Body	Open sea	51.75	5.17	57.36	4.94	-5.61	4.53E-07	2.12E-05
cg03861427	HLA-H	6	TSS200	Island	25.18	7.25	30.79	8.11	-5.61	2.05E-03	2.88E-02
cg07969684		2	Intergenic	Open sea	57.67	7.87	63.28	10.05	-5.61	3.43E-03	4.11E-02
cg00604454		16	Intergenic	Island	42.32	4.68	47.93	5.35	-5.61	2.42E-06	1.02E-04
cg18279094	FOXD3	1	1stExon	Island	24.17	5.95	29.78	6.78	-5.61	2.69E-06	1.13E-04

cg08325191	C10orf93	10	TSS1500	Island	20.55	8.22	26.16	9.90	-5.61	8.86E-04	1.56E-02
cg05863683	MAD1L1;MAD1L1;MAD1L1	7	Body;Body;Body	N_Shore	42.33	5.64	47.94	7.04	-5.61	3.37E-04	7.46E-03
cg06131630		18	Intergenic	S_Shelf	40.75	6.80	46.35	7.54	-5.61	7.71E-04	1.41E-02
cg17449088	MCC;MCC	5	Body;Body	Open sea	21.31	3.40	26.91	6.49	-5.60	2.94E-06	1.22E-04
cg25230284	ASPA	17	TSS200	S_Shelf	43.52	7.26	49.12	6.04	-5.60	2.96E-03	3.71E-02
cg09238677	C3AR1;C3AR1	12	1stExon;5'UTR	Open sea	45.38	6.32	50.98	6.39	-5.60	2.73E-06	1.14E-04
cg00176638	KDM5B	1	TSS1500	N_Shore	66.06	5.91	71.66	6.78	-5.60	1.32E-05	4.84E-04
cg07840332		17	Intergenic	Open sea	73.79	5.58	79.39	3.77	-5.60	3.79E-07	1.80E-05
cg06213635		11	Intergenic	S_Shore	49.05	8.36	54.65	7.78	-5.60	3.12E-03	3.85E-02
cg10593028	EXPH5;EXPH5;EXPH5	11	1stExon;5'UTR;Body	Open sea	76.30	6.42	81.90	4.53	-5.60	1.29E-04	3.46E-03
cg09167931		17	Intergenic	S_Shelf	47.56	5.61	53.16	7.88	-5.60	8.66E-04	1.54E-02
cg06909503	PBX1	1	Body	Open sea	86.27	4.67	91.87	2.31	-5.60	5.26E-11	3.06E-09
cg04971534	SEZ6;SEZ6	17	TSS1500;TSS1500	S_Shore	25.80	4.23	31.39	5.43	-5.60	1.47E-07	7.24E-06
cg15521572	MBP;MBP	18	Body;Body	N_Shore	54.31	3.51	59.91	2.91	-5.60	7.09E-10	3.97E-08
cg27331871	INS-IGF2;IGF2;INS-IGF2	11	Body;TSS1500;Body	Open sea	61.22	6.58	66.81	5.70	-5.60	4.72E-05	1.48E-03
cg09929374	DPYS	8	TSS1500	S_Shore	53.80	5.96	59.40	6.57	-5.60	2.07E-04	5.07E-03
cg16909733	ANKMY1;ANKMY1	2	Body;Body	Island	14.79	3.57	20.38	6.08	-5.60	4.13E-08	2.12E-06
cg03823431	SLC38A10;SLC38A10	17	Body;Body	Open sea	54.31	8.12	59.90	6.94	-5.60	7.40E-04	1.37E-02
cg04090794	HSP90B3P	1	TSS1500	Open sea	51.46	6.33	57.05	5.39	-5.60	7.31E-05	2.15E-03
cg10805856		19	Intergenic	Island	47.05	4.14	52.64	5.24	-5.60	1.11E-06	4.95E-05
cg11441533	SRGAP1	12	Body	Open sea	74.38	7.28	79.98	6.41	-5.59	2.12E-06	9.07E-05
cg01062942	RASAL3	19	Body	Island	24.92	6.48	30.52	8.57	-5.59	1.43E-03	2.22E-02
cg01436551	ZNF148	3	5'UTR	Open sea	75.65	8.08	81.24	7.39	-5.59	9.86E-04	1.69E-02
cg24616382	GAL3ST4	7	TSS1500	N_Shore	55.37	6.02	60.97	7.88	-5.59	5.89E-04	1.15E-02
cg10298187	TPPP3;TPPP3	16	TSS1500;TSS1500	Island	43.46	5.76	49.05	7.89	-5.59	3.69E-04	8.01E-03
cg27116510	IPO5	13	TSS200	Open sea	60.48	5.44	66.07	4.91	-5.59	1.47E-05	5.31E-04
cg00334498	AKAP9;AKAP9	7	Body;Body	Open sea	68.38	4.67	73.98	6.24	-5.59	1.18E-05	4.35E-04
cg10792982	BRF1	14	Body	Open sea	59.00	6.93	64.59	7.42	-5.59	1.63E-03	2.44E-02
cg15983605		11	Intergenic	Open sea	64.99	5.40	70.58	4.00	-5.59	1.52E-07	7.45E-06
cg26242531	ZFYVE21	14	Body	N_Shelf	27.08	4.98	32.67	5.36	-5.59	4.21E-05	1.34E-03
cg23279355	CMYA5	5	TSS200	Open sea	19.77	5.00	25.36	6.61	-5.59	1.04E-04	2.89E-03
cg20668546	GFAP;GFAP	17	Body;Body	S_Shore	59.96	6.35	65.54	6.55	-5.58	1.53E-03	2.34E-02
cg08476511	KIF25;KIF25	6	Body;Body	Island	38.37	4.77	43.95	7.71	-5.58	6.56E-05	1.96E-03
cg05721476	MARCH8;MARCH8	10	5'UTR;5'UTR	Open sea	38.07	6.48	43.66	6.32	-5.58	1.06E-03	1.78E-02
cg24199724		3	Intergenic	Open sea	51.08	5.87	56.66	5.10	-5.58	1.64E-04	4.22E-03
cg02805814		19	Intergenic	Island	64.61	8.26	70.19	7.68	-5.58	3.69E-03	4.32E-02
cg07629965	SEMA3B;SEMA3B	3	Body;Body	N_Shore	11.69	4.99	17.27	8.51	-5.58	3.76E-04	8.12E-03
cg16379091		11	Intergenic	Island	51.48	8.95	57.06	7.57	-5.58	4.40E-03	4.87E-02
cg27010159	SRRM4	12	Body	N_Shore	40.87	7.39	46.45	8.93	-5.58	2.66E-03	3.44E-02
cg06489569		16	Intergenic	Open sea	38.12	6.18	43.70	7.40	-5.58	7.72E-04	1.41E-02
cg00151922	JAG1	20	Body	N_Shore	31.39	6.32	36.97	5.38	-5.58	2.81E-05	9.41E-04
cg02241055	CLDN11;CLDN11	3	1stExon;5'UTR	Island	25.02	4.75	30.60	5.96	-5.58	3.18E-07	1.52E-05
cg22906397	RAPGEF2	4	Body	Open sea	65.42	6.59	71.00	8.58	-5.58	1.39E-03	2.18E-02
cg26280976		14	Intergenic	S_Shore	65.75	5.98	71.33	6.87	-5.58	2.60E-04	6.09E-03
cg03259564		16	Intergenic	S_Shore	35.43	5.91	41.01	7.29	-5.58	2.79E-04	6.42E-03
cg19007908	TRPM4	19	Body	Island	49.14	6.74	54.71	7.57	-5.58	1.60E-03	2.41E-02
cg08198265	BST1	4	Body	S_Shelf	54.90	4.90	60.47	5.24	-5.57	1.04E-05	3.91E-04
cg06964475		5	Intergenic	Island	81.50	8.42	87.07	5.10	-5.57	1.83E-03	2.66E-02
cg05127821	CYP26C1	10	Body	Island	7.97	2.98	13.54	7.47	-5.57	1.50E-06	6.56E-05
cg13151707	RRAGD	6	Body	N_Shelf	66.55	5.60	72.12	5.79	-5.57	6.32E-05	1.90E-03
cg20764575	DFNA5;DFNA5;DFNA5	7	TSS1500;TSS1500;TSS1500	S_Shore	35.22	5.94	40.79	7.74	-5.57	1.91E-03	2.74E-02
cg24107619		5	Intergenic	Open sea	61.01	6.52	66.58	8.33	-5.57	3.85E-03	4.44E-02
cg19392504	SLMAP	3	Body	Open sea	72.72	6.02	78.29	4.42	-5.57	1.99E-05	6.95E-04

cg14675404	BRUNOL5	19	Body	N_Shore	37.95	6.45	43.52	7.85	-5.57	1.35E-03	2.14E-02
cg03177729	SEC31A;SEC31A;SEC31A;SEC31A;SEC3	4	5'UTR;5'UTR;5'UTR;5'UTR;5'UTR	Open sea	45.36	6.42	50.93	5.48	-5.57	3.10E-04	6.97E-03
cg02212009	TEAD1	11	Body	Open sea	41.13	5.11	46.70	5.40	-5.57	2.65E-05	8.95E-04
cg14718065		2	Intergenic	Island	44.59	5.47	50.16	6.94	-5.57	1.57E-04	4.06E-03
cg08945756		6	Intergenic	Open sea	55.93	5.85	61.50	5.15	-5.57	4.12E-07	1.94E-05
cg09198782	SLC22A18AS;SLC22A18;SLC22A18	11	5'UTR;5'UTR;TSS1500	N_Shore	75.40	6.77	80.97	5.54	-5.57	1.04E-04	2.90E-03
cg25121292		2	Intergenic	Island	44.21	4.56	49.77	5.93	-5.57	2.13E-06	9.10E-05
cg01310345	BAIAP2;BAIAP2;BAIAP2;BAIAP2	17	Body;Body;Body;Body	Open sea	76.93	7.70	82.50	6.92	-5.57	7.29E-04	1.35E-02
cg01896432		4	Intergenic	Open sea	60.73	8.04	66.29	7.69	-5.57	1.67E-03	2.49E-02
cg13651986	WIPF1	2	TSS1500	S_Shore	29.59	6.34	35.15	6.90	-5.57	2.71E-04	6.28E-03
cg14611258	C17orf39	17	Body	S_Shelf	61.93	6.07	67.49	4.72	-5.57	1.69E-06	7.31E-05
cg03116035		4	Intergenic	Open sea	59.16	6.90	64.72	6.91	-5.57	2.00E-03	2.83E-02
cg04431935	UNKL	16	3'UTR	N_Shore	51.55	5.73	57.11	8.19	-5.56	2.34E-03	3.16E-02
cg20906753	SLC35C1;SLC35C1;SLC35C1	11	Body;Body;Body	S_Shelf	76.56	9.24	82.13	6.06	-5.56	2.24E-03	3.06E-02
cg18237545		7	Intergenic	Island	24.45	6.72	30.02	6.75	-5.56	6.02E-05	1.83E-03
cg20102877	KRTCAP3;KRTCAP3	2	Body;Body	Island	56.02	6.32	61.58	7.61	-5.56	2.03E-03	2.86E-02
cg00995065	SLCO1B1	12	TSS1500	Open sea	37.46	5.92	43.02	7.11	-5.56	6.47E-04	1.24E-02
cg14771070	KIAA1875	8	TSS200	N_Shore	70.62	7.29	76.18	6.93	-5.56	2.54E-03	3.34E-02
cg09990962	PRDM16;PRDM16	1	Body;Body	Island	12.52	3.42	18.08	6.19	-5.56	6.13E-08	3.11E-06
cg19216792	SKAP2	7	Body	Open sea	28.12	5.89	33.68	5.45	-5.56	1.37E-06	6.04E-05
cg00020799	PLXNC1	12	Body	Open sea	51.36	5.86	56.92	6.78	-5.56	7.60E-04	1.39E-02
cg09258877	TRIP12	2	Body	Open sea	70.13	5.05	75.69	5.59	-5.56	6.53E-05	1.95E-03
cg08790890		2	Intergenic	Open sea	16.43	4.97	21.99	5.35	-5.56	7.48E-06	2.89E-04
cg04237997	ESAM	11	Body	Island	38.12	5.28	43.68	7.76	-5.56	1.74E-04	4.42E-03
cg24903006	RAPGEFL1	17	Body	S_Shore	13.19	2.31	18.75	6.96	-5.56	2.32E-07	1.13E-05
cg13845133	PBX1	1	Body	N_Shore	79.34	4.56	84.90	4.14	-5.56	5.31E-09	2.87E-07
cg00176043		1	Intergenic	S_Shelf	52.74	5.98	58.29	5.44	-5.56	2.45E-05	8.36E-04
cg16466870		14	Intergenic	S_Shore	62.24	4.88	67.80	4.32	-5.56	5.86E-08	2.98E-06
cg16627854		6	Intergenic	Open sea	48.22	5.20	53.78	4.83	-5.56	9.29E-07	4.19E-05
cg27494470		6	Intergenic	Open sea	73.60	4.78	79.15	4.88	-5.56	4.96E-07	2.32E-05
cg19696891	ZNF331;ZNF331;ZNF331	19	5'UTR;5'UTR;TSS1500	Island	25.47	4.54	31.03	4.83	-5.56	1.05E-06	4.69E-05
cg25955565	SNORD56B	14	TSS1500	Open sea	48.35	5.76	53.91	7.32	-5.55	3.35E-04	7.43E-03
cg23928165	PNMAL2;PNMAL2	19	1stExon;3'UTR	Island	3.74	2.29	9.29	7.15	-5.55	1.35E-07	6.64E-06
cg08111922	MGC23270	14	TSS200	Open sea	47.79	6.82	53.35	6.24	-5.55	7.61E-04	1.40E-02
cg05258689	C16orf79	16	TSS200	N_Shelf	50.76	5.74	56.31	6.53	-5.55	4.01E-05	1.28E-03
cg13293535	GATA4	8	Body	Open sea	45.48	7.63	51.03	6.29	-5.55	3.37E-04	7.45E-03
cg16029623		14	Intergenic	N_Shelf	47.02	7.29	52.58	7.40	-5.55	6.69E-04	1.27E-02
cg10822665	ABR;ABR	17	Body;Body	N_Shore	63.71	7.71	69.27	8.26	-5.55	4.11E-03	4.65E-02
cg04573398	CCDC81;CCDC81;CCDC81;CCDC81	11	1stExon;5'UTR;1stExon;5'UTR	Island	60.67	6.76	66.22	5.24	-5.55	1.61E-04	4.15E-03
cg21814995	TGFBR2;TGFBR2	3	Body;Body	Open sea	70.96	4.70	76.51	4.29	-5.55	4.42E-07	2.08E-05
cg20963047		1	Intergenic	N_Shelf	66.08	5.23	71.63	3.89	-5.55	1.78E-07	8.69E-06
cg25712005	ELN;ELN;ELN;ELN;ELN	7	Body;Body;Body;Body;Body	Open sea	28.04	3.14	33.59	4.15	-5.55	3.09E-09	1.69E-07
cg20303331	PIP4K2B	17	Body	Open sea	41.73	4.78	47.28	4.78	-5.55	1.91E-07	9.29E-06
cg25649765	GREB1	2	TSS1500	Open sea	76.79	5.90	82.34	4.41	-5.55	5.09E-08	2.59E-06
cg26290155		14	Intergenic	S_Shelf	58.96	5.62	64.51	6.35	-5.55	1.96E-05	6.87E-04
cg02372702	CASC1;CASC1;CASC1	12	Body;Body;Body	Open sea	52.07	5.49	57.62	4.20	-5.55	2.17E-07	1.06E-05
cg18188326	CLRN2	4	Body	Open sea	33.71	5.09	39.26	6.58	-5.55	5.78E-05	1.76E-03
cg25391820	MATR3;SNHG4	5	TSS1500;TSS1500	N_Shore	53.58	6.50	59.13	7.84	-5.55	5.66E-06	2.24E-04
cg17356056		5	Intergenic	Island	59.84	5.46	65.39	5.88	-5.55	1.91E-06	8.23E-05
cg11165391		1	Intergenic	Open sea	34.82	5.59	40.37	5.07	-5.55	3.23E-05	1.06E-03
cg26026615		12	Intergenic	S_Shore	19.93	6.06	25.48	6.26	-5.55	2.10E-05	7.30E-04
cg16078836	MAP3K13	3	5'UTR	Open sea	35.76	5.94	41.31	7.31	-5.55	9.11E-06	3.46E-04
cg18836745	C1QTNF5;MFRP	11	5'UTR;3'UTR	S_Shore	25.10	4.29	30.65	5.85	-5.55	1.97E-06	8.44E-05

cg26675664		4	Intergenic	Island	73.17	5.26	78.72	4.98	-5.55	2.83E-05	9.48E-04
cg05492075		10	Intergenic	Open sea	73.12	6.45	78.67	6.73	-5.55	6.24E-04	1.20E-02
cg01078446	TBX3;TBX3	12	Body;Body	S_Shore	49.77	7.25	55.32	7.10	-5.55	2.62E-03	3.41E-02
cg01474011	CUL3	2	Body	Open sea	55.10	5.58	60.64	5.90	-5.55	1.40E-05	5.09E-04
cg04850059		1	Intergenic	Open sea	57.05	6.86	62.60	6.51	-5.54	1.75E-03	2.57E-02
cg27440002	LEAP2	5	TSS1500	Open sea	74.56	6.73	80.10	4.66	-5.54	7.42E-06	2.87E-04
cg26581504	BCO2;BCO2	11	TSS200;Body	Open sea	34.37	4.58	39.91	7.50	-5.54	3.10E-04	6.98E-03
cg23111488		5	Intergenic	Open sea	25.51	6.14	31.05	8.84	-5.54	2.08E-03	2.90E-02
cg09774289	CPPED1;CPPED1	16	Body;Body	N_Shelf	69.67	6.01	75.21	6.28	-5.54	4.11E-04	8.71E-03
cg17477090		1	Intergenic	Island	53.98	8.17	59.53	6.53	-5.54	1.19E-03	1.95E-02
cg19800032		7	Intergenic	Open sea	52.25	6.11	57.79	8.80	-5.54	1.65E-03	2.46E-02
cg06838283	C5orf13;C5orf13;C5orf13;C5orf13;C5c	5	Body;Body;Body;Body;Body;Body;Body;Bc	N_Shelf	45.32	5.26	50.86	5.18	-5.54	1.27E-05	4.68E-04
cg25615926		11	Intergenic	Open sea	66.77	7.35	72.31	5.85	-5.54	1.90E-04	4.75E-03
cg24626554	LOC389458	7	TSS200	Island	28.61	7.61	34.15	9.23	-5.54	2.00E-03	2.83E-02
cg21785384	TMCC2	1	Body	Open sea	39.59	5.99	45.13	6.10	-5.54	4.43E-05	1.40E-03
cg20403644	NXN	17	Body	N_Shore	51.92	7.38	57.47	8.67	-5.54	3.95E-04	8.43E-03
cg17861455	TRPV3	17	Body	Island	12.84	7.65	18.38	9.74	-5.54	4.29E-03	4.78E-02
cg01452110	HIST1H3A	6	TSS1500	N_Shore	76.50	4.86	82.04	4.46	-5.54	5.53E-07	2.57E-05
cg01016850		16	Intergenic	N_Shore	53.90	6.21	59.44	8.25	-5.54	2.79E-03	3.56E-02
cg00989002		16	Intergenic	Open sea	32.35	4.68	37.89	6.22	-5.54	2.66E-05	8.99E-04
cg06377226		4	Intergenic	Open sea	36.84	7.13	42.38	7.92	-5.54	2.81E-03	3.58E-02
cg00833344	MMADHC	2	3'UTR	Open sea	67.97	6.73	73.50	5.79	-5.54	3.81E-05	1.23E-03
cg19543317	JDP2;JDP2;JDP2;JDP2	14	Body;Body;Body;Body	Open sea	47.13	8.43	52.66	11.25	-5.54	2.59E-03	3.38E-02
cg13412754	MAPKAP1;MAPKAP1;MAPKAP1;MAPK	9	Body;Body;Body;Body;Body;Body	Open sea	43.96	6.42	49.50	5.96	-5.54	1.17E-03	1.92E-02
cg17314160	GIT2;GIT2;GIT2;GIT2;GIT2;GIT2	12	Body;Body;Body;Body;Body;Body	Open sea	72.33	7.01	77.86	6.19	-5.53	3.16E-04	7.09E-03
cg13306921	SSBP3;SSBP3;SSBP3	1	Body;Body;Body	Open sea	36.82	5.07	42.35	5.18	-5.53	4.05E-05	1.30E-03
cg21571602	ZC3H6	2	Body	S_Shelf	42.68	6.26	48.22	6.07	-5.53	4.44E-04	9.24E-03
cg24829409	C8orf71	8	Body	Island	45.88	5.80	51.41	6.53	-5.53	6.92E-05	2.05E-03
cg06336912		16	Intergenic	S_Shore	44.85	5.48	50.38	5.06	-5.53	4.93E-06	1.98E-04
cg03968618	SORBS2	4	5'UTR	Open sea	59.51	5.69	65.04	5.70	-5.53	2.05E-05	7.15E-04
cg27006914	RNF216;RNF216	7	Body;Body	Open sea	59.48	6.08	65.01	4.72	-5.53	1.21E-04	3.27E-03
cg27660720	STK39	2	Body	Open sea	64.23	5.82	69.76	7.43	-5.53	8.04E-05	2.33E-03
cg09777237	ELN;ELN;ELN;ELN;ELN	7	Body;Body;Body;Body;Body	Open sea	36.06	4.95	41.59	7.25	-5.53	2.81E-04	6.47E-03
cg00841760	PTPRN2;PTPRN2;PTPRN2	7	Body;Body;Body	Island	45.73	6.99	51.26	7.84	-5.53	1.27E-04	3.42E-03
cg24807547		6	Intergenic	S_Shore	28.49	6.05	34.02	7.33	-5.53	3.64E-04	7.92E-03
cg16284279		6	Intergenic	Open sea	64.93	6.95	70.46	5.02	-5.53	2.82E-06	1.18E-04
cg06882058	SDCCAG8	1	Body	Island	25.68	5.60	31.21	8.24	-5.53	5.53E-04	1.09E-02
cg23996800	TSKU	11	Body	N_Shelf	77.69	8.81	83.22	6.42	-5.53	1.85E-03	2.68E-02
cg25353990	EBF1	5	Body	Open sea	57.33	5.85	62.85	6.27	-5.53	3.32E-05	1.09E-03
cg22455720	TBCD	17	Body	Open sea	33.78	6.29	39.31	8.04	-5.53	3.83E-03	4.43E-02
cg01558212		2	Intergenic	Island	12.65	3.45	18.17	6.16	-5.53	3.55E-07	1.69E-05
cg06048910	SLC22A18;SLC22A18;SLC22A18AS	11	Body;Body;TSS1500	S_Shore	63.22	9.45	68.74	8.43	-5.53	1.11E-03	1.85E-02
cg24035484		12	Intergenic	Open sea	44.95	6.45	50.48	6.06	-5.53	1.81E-04	4.57E-03
cg18432877	HELZ	17	Body	Open sea	55.77	7.69	61.30	8.27	-5.53	3.63E-03	4.27E-02
cg03262295	FGFR3;FGFR3;FGFR3	4	Body;Body;Body	Island	82.10	5.28	87.63	4.21	-5.52	8.49E-07	3.85E-05
cg17701886	MCF2L2;B3GNT5	3	Body;TSS200	N_Shore	10.50	3.79	16.03	6.21	-5.52	1.42E-07	6.99E-06
cg17059388		4	Intergenic	Open sea	38.48	7.04	44.00	7.59	-5.52	2.46E-03	3.27E-02
cg15490715		6	Intergenic	Island	27.96	6.76	33.48	4.91	-5.52	1.49E-04	3.90E-03
cg01214458	MN1	22	1stExon	Island	21.67	4.03	27.19	5.51	-5.52	2.46E-06	1.04E-04
cg21312412	VSNL1	2	5'UTR	Open sea	48.51	4.38	54.04	6.90	-5.52	3.70E-05	1.20E-03
cg15231749	RGL2;RGL2	6	Body;Body	N_Shore	64.06	5.59	69.58	7.22	-5.52	2.84E-05	9.50E-04
cg05823218	LBX2	2	Body	Island	17.62	5.88	23.14	7.24	-5.52	4.94E-05	1.54E-03
cg02627599		6	Intergenic	N_Shore	53.09	6.53	58.61	6.97	-5.52	2.62E-03	3.41E-02

cg24126849		4	Intergenic	N_Shore	69.72	6.93	75.24	5.59	-5.52	2.95E-04	6.71E-03
cg24090399		4	Intergenic	Island	55.41	7.98	60.93	7.94	-5.52	3.42E-03	4.11E-02
cg04909257	PGD	1	Body	S_Shelf	70.53	5.41	76.04	5.57	-5.52	1.83E-04	4.60E-03
cg19743522	DTX1	12	TSS200	S_Shore	49.14	5.00	54.66	5.71	-5.52	9.45E-05	2.67E-03
cg24438354		12	Intergenic	Open sea	44.35	5.34	49.87	6.02	-5.52	1.04E-06	4.67E-05
cg16967626	GABRA5;GABRA5	15	TSS1500;TSS1500	N_Shore	27.33	5.10	32.85	7.10	-5.52	5.67E-05	1.73E-03
cg10353705	CCDC153	11	Body	Open sea	52.84	5.39	58.36	5.80	-5.52	1.24E-05	4.57E-04
cg02849507		16	Intergenic	Open sea	36.68	9.84	42.20	12.17	-5.52	2.64E-05	8.94E-04
cg05877788	TP53I13	17	Body	Island	27.61	5.68	33.13	6.05	-5.52	2.02E-04	4.98E-03
cg20673721	KSR1	17	5'UTR	Open sea	78.01	7.22	83.53	5.39	-5.52	4.30E-04	9.02E-03
cg01973920		5	Intergenic	Open sea	40.64	5.88	46.16	7.34	-5.51	3.18E-04	7.12E-03
cg03691530	PVT1	8	Body	Open sea	61.98	6.66	67.49	6.64	-5.51	1.69E-04	4.31E-03
cg05489292	MAPKAPK5;MAPKAPK5	12	3'UTR;3'UTR	Open sea	53.05	7.20	58.56	6.56	-5.51	1.17E-03	1.92E-02
cg11990296	ADAMTS10	19	Body	S_Shore	80.40	5.10	85.92	3.78	-5.51	2.25E-07	1.09E-05
cg22215453	SUV420H2	19	5'UTR	S_Shore	26.64	8.07	32.15	8.65	-5.51	1.32E-04	3.52E-03
cg18086445		3	Intergenic	Open sea	70.38	5.49	75.89	4.97	-5.51	1.21E-05	4.45E-04
cg05185021	METT10D	17	Body	Open sea	69.78	5.26	75.29	5.07	-5.51	1.26E-06	5.58E-05
cg10632773	VAMP8	2	TSS1500	Open sea	53.82	6.62	59.33	7.15	-5.51	1.84E-03	2.67E-02
cg02935024	RAB6B	3	Body	Open sea	51.35	3.45	56.86	4.22	-5.51	2.45E-10	1.40E-08
cg18808929		8	Intergenic	S_Shelf	50.42	5.97	55.93	6.36	-5.51	3.29E-04	7.31E-03
cg27531571		17	Intergenic	S_Shore	34.08	5.59	39.59	7.71	-5.51	3.88E-04	8.32E-03
cg13753613		9	Intergenic	N_Shore	77.08	7.45	82.59	4.96	-5.51	2.43E-05	8.32E-04
cg08561732		15	Intergenic	N_Shore	38.10	4.81	43.61	7.72	-5.51	3.16E-05	1.05E-03
cg12623302	ZSCAN12L1	6	TSS200	Open sea	53.99	5.42	59.50	5.74	-5.51	1.81E-05	6.39E-04
cg25083763	IQGAP2	5	Body	Open sea	59.99	4.76	65.50	5.36	-5.51	2.14E-05	7.43E-04
cg06898214	MOV10L1;MOV10L1;MOV10L1;MOV1	22	TSS200;Body;Body;Body	Open sea	37.70	5.75	43.21	8.91	-5.51	2.04E-03	2.87E-02
cg18313094	USP29	19	5'UTR	S_Shelf	38.27	7.88	43.78	9.62	-5.50	4.16E-03	4.69E-02
cg24456002		15	Intergenic	N_Shore	33.21	3.78	38.71	4.86	-5.50	5.78E-09	3.12E-07
cg19461383	MIR548H4;NOX5	15	Body;Body	Island	75.69	9.06	81.19	7.73	-5.50	4.18E-03	4.70E-02
cg09061207	BAT1;BAT1;SNORD117	6	Body;Body;Body	Open sea	58.23	5.09	63.73	4.49	-5.50	1.23E-05	4.55E-04
cg20415947		1	Intergenic	Open sea	43.80	6.80	49.30	7.45	-5.50	6.90E-04	1.30E-02
cg13851744	C9orf75;C9orf75	9	Body;Body	N_Shore	47.08	3.86	52.59	5.15	-5.50	6.12E-07	2.83E-05
cg19414598	DMC1	22	5'UTR	Island	12.33	3.12	17.83	6.65	-5.50	1.07E-06	4.80E-05
cg16340422	PLD6	17	TSS1500	Island	18.04	3.69	23.54	7.20	-5.50	2.59E-05	8.78E-04
cg23867673	CDH23;CDH23	10	Body;Body	Open sea	48.90	5.09	54.40	4.89	-5.50	1.94E-06	8.35E-05
cg26734350	GPR56;GPR56;GPR56;GPR56;GPR56;G	16	TSS1500;TSS1500;TSS1500;TSS1500;5'UTR	Open sea	22.33	5.59	27.83	7.81	-5.50	6.04E-04	1.17E-02
cg26615794		1	Intergenic	S_Shelf	73.24	3.74	78.74	4.11	-5.50	1.44E-08	7.60E-07
cg25810938	ANKMY1;ANKMY1	2	5'UTR;5'UTR	Island	45.79	7.26	51.29	5.85	-5.50	1.03E-04	2.87E-03
cg07906484	HNF4A;HNF4A;HNF4A;HNF4A;HNF4A;	20	Body;Body;Body;Body;Body;Body	Open sea	63.93	5.38	69.42	7.09	-5.50	6.86E-04	1.29E-02
cg15909056	LGR5	12	Body	S_Shelf	51.49	6.74	56.99	7.01	-5.50	1.05E-04	2.91E-03
cg26383454	HERV-FRD;LOC221710	6	Body;Body	Open sea	52.78	2.96	58.28	2.87	-5.50	1.02E-12	6.25E-11
cg21698185	KBTBD11	8	Body	Island	15.11	7.54	20.60	8.55	-5.50	1.35E-03	2.14E-02
cg04154281	UBTF;UBTF;UBTF	17	Body;Body;Body	S_Shore	57.44	4.68	62.94	4.40	-5.50	2.80E-06	1.17E-04
cg26347162		8	Intergenic	Open sea	87.83	9.77	93.33	4.73	-5.50	1.61E-03	2.42E-02
cg24912415	MYH7B	20	Body	Island	59.77	4.81	65.27	4.43	-5.50	1.88E-06	8.10E-05
cg23785859		6	Intergenic	Open sea	63.36	7.47	68.85	6.30	-5.50	1.86E-04	4.68E-03
cg04217082	EIF4EBP1	8	Body	S_Shore	55.00	6.30	60.50	7.41	-5.50	1.94E-03	2.77E-02
cg24342013	RB1	13	Body	Island	76.81	5.63	82.31	4.91	-5.50	1.30E-05	4.76E-04
cg17338258	SHBG;SHBG;SHBG;SHBG;SHBG;SHBG	17	1stExon;Body;1stExon;Body;1stExon;1stEx	S_Shelf	54.30	4.85	59.79	3.69	-5.49	2.03E-06	8.70E-05
cg09610578	TMCC1;TMCC1;TMCC1	3	TSS200;Body;Body	Open sea	34.55	6.72	40.04	7.61	-5.49	3.64E-03	4.28E-02
cg15575433	UFSP1;ACHE;ACHE	7	TSS1500;Body;Body	N_Shore	31.11	6.18	36.61	6.07	-5.49	1.87E-04	4.69E-03
cg04918402	LIMD2	17	TSS1500	Island	24.51	4.64	30.00	5.32	-5.49	7.33E-07	3.36E-05
cg05486035		16	Intergenic	Island	50.03	7.72	55.53	9.53	-5.49	1.81E-04	4.57E-03

cg10747785	MYH9	22	Body	Open sea	31.82	3.89	37.31	6.60	-5.49	1.08E-05	4.05E-04
cg07753510	TNIK;TNIK;TNIK;TNIK;TNIK;TNIK;TNIK;T	3	Body;Body;Body;Body;Body;Body;Body;Bo	Open sea	26.04	5.18	31.53	5.11	-5.49	2.59E-05	8.80E-04
cg08343644	GPR56;GPR56;GPR56;GPR56;GPR56;G	16	TSS1500;TSS1500;TSS1500;TSS1500;5'UTR	Open sea	46.18	5.54	51.67	7.72	-5.49	6.69E-04	1.27E-02
cg05583014	TREX1;TREX1;TREX1	3	3'UTR;1stExon;3'UTR	Open sea	57.83	5.46	63.32	7.91	-5.49	4.02E-04	8.55E-03
cg24688837	MIR1250;AATK	17	TSS200;Body	N_Shelf	79.09	5.30	84.57	4.21	-5.49	1.44E-08	7.61E-07
cg00403498	PRRT1;PPT2	6	TSS1500;TSS1500	N_Shore	39.13	4.77	44.62	5.85	-5.49	3.64E-06	1.49E-04
cg10146203	GABRA5;GABRA5	15	TSS1500;TSS1500	N_Shore	22.47	5.32	27.95	7.64	-5.49	7.40E-04	1.37E-02
cg09926562	FANCI;FANCI	15	Body;Body	Open sea	71.83	7.97	77.32	7.80	-5.49	1.74E-04	4.42E-03
cg17640744		6	Intergenic	S_Shore	66.59	4.77	72.08	3.31	-5.49	1.37E-07	6.76E-06
cg16570507	MIR346;GRID1	10	TSS200;Body	S_Shore	52.77	7.33	58.25	8.64	-5.48	1.83E-04	4.60E-03
cg26578072	TTLL10	1	TSS1500	N_Shore	54.06	3.79	59.54	4.36	-5.48	1.74E-07	8.51E-06
cg12418043		5	Intergenic	Open sea	80.00	5.15	85.49	4.03	-5.48	2.04E-07	9.93E-06
cg22694191	HAPLN4;HAPLN4	19	5'UTR;1stExon	S_Shore	20.00	5.18	25.48	6.75	-5.48	5.83E-06	2.30E-04
cg26707709	SNED1	2	Body	Island	36.84	5.96	42.32	6.27	-5.48	1.81E-05	6.38E-04
cg13706582	ZNF385D	3	TSS1500	Open sea	66.62	6.61	72.10	6.01	-5.48	1.47E-04	3.85E-03
cg12550597	FAM129C	19	3'UTR	N_Shore	62.91	5.70	68.39	4.95	-5.48	1.19E-05	4.40E-04
cg27160701	MUCL1	12	Body	Open sea	72.75	6.35	78.23	5.62	-5.48	3.29E-06	1.36E-04
cg20838586	RBMXL2;RBMXL2	11	5'UTR;1stExon	Island	53.24	6.74	58.72	7.76	-5.48	1.15E-03	1.90E-02
cg04851742		13	Intergenic	S_Shore	13.56	5.50	19.04	9.06	-5.48	2.48E-03	3.28E-02
cg20693607		3	Intergenic	Island	49.31	7.01	54.79	7.58	-5.48	4.19E-03	4.70E-02
cg06906869	NEK3;NEK3;NEK3;NEK3	13	TSS1500;TSS200;TSS200;TSS200	S_Shore	63.94	4.50	69.42	4.37	-5.48	7.00E-07	3.21E-05
cg21841632		3	Intergenic	Open sea	40.67	4.46	46.15	5.58	-5.48	5.38E-06	2.14E-04
cg26170683	TRIM26	6	5'UTR	Open sea	62.14	4.97	67.62	4.78	-5.48	2.54E-07	1.23E-05
cg27248571	LY6G6D	6	Body	Open sea	65.35	5.78	70.83	5.76	-5.48	1.42E-05	5.15E-04
cg24029819	PTPRN2;PTPRN2;PTPRN2	7	Body;Body;Body	Island	51.99	5.69	57.46	6.83	-5.48	2.75E-05	9.24E-04
cg10364881	DKK3;DKK3;DKK3	11	Body;Body;Body	Open sea	73.64	10.44	79.12	8.67	-5.47	3.14E-04	7.06E-03
cg13900773	PIWIL1	12	5'UTR	Island	22.74	8.11	28.22	10.14	-5.47	3.00E-03	3.74E-02
cg03874542		10	Intergenic	Open sea	48.49	5.81	53.96	7.24	-5.47	7.00E-04	1.31E-02
cg10092251		13	Intergenic	Open sea	20.23	6.08	25.70	9.07	-5.47	3.78E-03	4.39E-02
cg25035059	CHD7	8	Body	Open sea	45.79	5.92	51.27	4.94	-5.47	4.14E-06	1.68E-04
cg01044025		3	Intergenic	Open sea	61.20	5.03	66.67	6.35	-5.47	1.63E-06	7.08E-05
cg26390660		3	Intergenic	Open sea	60.31	5.87	65.78	3.77	-5.47	7.94E-05	2.31E-03
cg10274108	TBC1D1	4	Body	Open sea	63.05	5.46	68.52	4.56	-5.47	2.00E-05	7.00E-04
cg23619910	AMPD3;AMPD3;AMPD3	11	TSS200;5'UTR;Body	Open sea	21.64	6.20	27.11	7.88	-5.47	7.28E-04	1.35E-02
cg26401673	ANO10	3	Body	Open sea	20.19	6.12	25.66	6.65	-5.47	2.01E-04	4.97E-03
cg01331275		2	Intergenic	Open sea	70.62	5.49	76.09	4.24	-5.47	2.14E-06	9.14E-05
cg26707887		5	Intergenic	Open sea	44.81	5.86	50.28	6.64	-5.47	4.13E-04	8.74E-03
cg03217471		6	Intergenic	Open sea	55.54	6.33	61.00	7.44	-5.47	6.40E-04	1.23E-02
cg16992627		6	Intergenic	Open sea	66.01	4.96	71.48	4.85	-5.47	2.41E-07	1.16E-05
cg26867545	C10orf140	10	3'UTR	N_Shore	43.49	6.13	48.96	8.24	-5.47	1.43E-03	2.23E-02
cg09208961	LOC91948;LOC91948	15	TSS1500;TSS1500	Open sea	43.88	5.62	49.34	7.86	-5.47	6.93E-04	1.30E-02
cg17278864	RICH2	17	Body	Island	11.16	5.15	16.63	9.22	-5.47	1.88E-03	2.71E-02
cg12434681	HOXA3	7	TSS1500	N_Shelf	46.91	6.35	52.38	8.67	-5.47	2.20E-03	3.02E-02
cg20119745	RNF39;RNF39	6	Body;Body	Island	43.36	4.98	48.82	4.87	-5.47	2.51E-06	1.06E-04
cg06070970	GLI4	8	3'UTR	Island	36.38	6.83	41.84	6.95	-5.47	3.33E-04	7.38E-03
cg00672712	C7orf43	7	3'UTR	N_Shelf	51.13	3.60	56.60	4.00	-5.47	1.00E-08	5.33E-07
cg12972064	FNDC3B;FNDC3B	3	Body;Body	Open sea	39.72	5.82	45.18	7.16	-5.46	1.60E-05	5.73E-04
cg25781936	NHSL1;NHSL1	6	Body;Body	Open sea	43.23	5.30	48.69	6.96	-5.46	2.16E-04	5.25E-03
cg26893699		17	Intergenic	Open sea	36.96	5.63	42.43	7.24	-5.46	5.70E-04	1.12E-02
cg14073057	RPTOR;RPTOR	17	Body;Body	Island	87.35	5.51	92.81	3.48	-5.46	4.88E-07	2.28E-05
cg13160874	THAP4;THAP4	2	Body;Body	Island	20.27	5.07	25.74	5.30	-5.46	5.59E-07	2.59E-05
cg05377576	SEZ6L2;SEZ6L2;SEZ6L2;SEZ6L2	16	Body;Body;Body;Body	N_Shore	21.35	5.10	26.81	8.04	-5.46	7.11E-05	2.10E-03
cg22641093	FAM125A	19	3'UTR	Open sea	37.95	4.05	43.41	4.92	-5.46	3.80E-07	1.80E-05

cg24709285	TADA2B	4	Body	S_Shore	31.88	4.94	37.34	5.66	-5.46	4.81E-05	1.50E-03
cg13469268		7	Intergenic	Open sea	60.81	5.34	66.27	7.00	-5.46	2.10E-04	5.14E-03
cg27058244	CLCN6	1	Body	Island	62.23	4.45	67.68	5.01	-5.46	2.15E-06	9.19E-05
cg26250400		19	Intergenic	S_Shelf	44.93	5.14	50.39	6.28	-5.46	1.16E-05	4.31E-04
cg22860137	MREG	2	TSS200	S_Shore	11.92	4.92	17.38	8.24	-5.46	4.66E-06	1.88E-04
cg02933431		6	Intergenic	Open sea	55.85	5.49	61.31	4.38	-5.46	2.20E-05	7.62E-04
cg23645768	ANKMY1;ANKMY1	2	Body;Body	N_Shelf	74.26	5.47	79.72	4.60	-5.46	2.13E-06	9.09E-05
cg20092531		18	Intergenic	Island	32.57	5.56	38.03	7.09	-5.46	8.39E-05	2.42E-03
cg21840806	CREB5;CREB5;CREB5;CREB5	7	Body;Body;Body;Body	Open sea	55.22	7.39	60.67	8.21	-5.46	3.56E-03	4.22E-02
cg25840094	CFHR5	1	TSS1500	Open sea	33.84	5.88	39.30	7.71	-5.46	3.18E-03	3.90E-02
cg27362718	ZFPM1	16	Body	Open sea	50.39	6.47	55.84	6.20	-5.46	8.72E-05	2.50E-03
cg01612660	SRD5A3	4	TSS1500	N_Shore	66.69	5.33	72.14	5.98	-5.46	2.79E-05	9.37E-04
cg25431463	SRC;SRC	20	Body;Body	Island	20.70	5.14	26.16	8.55	-5.46	6.78E-04	1.28E-02
cg10776057	RASAL2	1	Body	Open sea	67.12	8.83	72.58	7.01	-5.45	2.55E-03	3.35E-02
cg10708739	YPEL2	17	Body	Open sea	73.77	5.17	79.23	3.85	-5.45	4.71E-07	2.21E-05
cg26926221	UTRN	6	Body	Open sea	57.00	5.59	62.46	5.94	-5.45	5.04E-06	2.01E-04
cg15179515	FAM35B2	10	Body	S_Shore	31.34	8.41	36.79	9.90	-5.45	3.92E-03	4.51E-02
cg15269875	H19;MIR675	11	Body;Body	Island	66.91	4.05	72.36	5.57	-5.45	2.95E-06	1.23E-04
cg14270346	SHB	9	Body	Open sea	51.91	7.74	57.36	7.49	-5.45	2.82E-04	6.49E-03
cg12229364	WWC2	4	Body	Open sea	55.05	6.15	60.50	6.76	-5.45	6.66E-05	1.99E-03
cg23882862	C11orf92;C11orf93	11	5'UTR;TSS200	Island	12.05	5.78	17.50	6.36	-5.45	2.17E-05	7.53E-04
cg17605604	C5orf30	5	3'UTR	Open sea	41.21	9.02	46.66	9.89	-5.45	3.44E-03	4.12E-02
cg08815110	ARSG	17	5'UTR	S_Shelf	46.91	5.80	52.36	4.64	-5.45	2.58E-05	8.75E-04
cg08939275	CPSF3L	1	Body	N_Shelf	72.69	6.84	78.14	5.31	-5.45	1.23E-03	2.00E-02
cg05208396		19	Intergenic	N_Shore	44.90	5.75	50.34	8.74	-5.44	2.28E-03	3.10E-02
cg25539074		4	Intergenic	Open sea	55.89	6.11	61.33	5.80	-5.44	2.60E-06	1.09E-04
cg02916147	AQP8	16	TSS1500	Open sea	54.72	6.04	60.16	6.21	-5.44	2.50E-04	5.91E-03
cg17261676		6	Intergenic	N_Shelf	24.61	5.47	30.05	8.39	-5.44	9.82E-04	1.69E-02
cg04277232	ATP6V1G2;ATP6V1G2	6	3'UTR;3'UTR	S_Shelf	59.64	4.54	65.09	4.22	-5.44	1.29E-06	5.71E-05
cg02992588	SERPINA6	14	TSS1500	Open sea	60.41	6.03	65.85	6.74	-5.44	5.13E-04	1.03E-02
cg01432609	RPTOR;RPTOR	17	Body;Body	Open sea	34.37	4.95	39.81	6.48	-5.44	1.59E-04	4.12E-03
cg27485108	KCNK3	2	Body	Island	24.55	4.62	29.99	5.71	-5.44	1.94E-05	6.81E-04
cg03915559	LGI1	10	Body	Open sea	70.19	5.78	75.63	4.32	-5.44	1.18E-05	4.37E-04
cg25692621	ASB4;ASB4	7	TSS200;TSS200	Open sea	74.40	7.09	79.84	5.93	-5.44	7.42E-04	1.37E-02
cg08259168		1	Intergenic	N_Shelf	57.30	4.90	62.74	5.22	-5.44	8.24E-06	3.16E-04
cg08893109	CYTH3	7	TSS1500	S_Shore	53.64	5.73	59.08	5.71	-5.44	2.03E-04	5.00E-03
cg18250736		3	Intergenic	Open sea	68.06	6.37	73.50	5.88	-5.44	6.96E-05	2.07E-03
cg01484487	LBX2	2	Body	Island	30.81	5.77	36.25	6.88	-5.44	4.06E-04	8.62E-03
cg15180869	ADAMTS2;ADAMTS2	5	Body;Body	Open sea	78.90	11.65	84.33	7.83	-5.44	3.77E-03	4.38E-02
cg10131465		11	Intergenic	N_Shelf	52.36	5.67	57.79	4.89	-5.44	3.63E-05	1.18E-03
cg04439622	CRIP2	14	Body	Island	67.22	9.11	72.66	6.24	-5.44	4.34E-03	4.82E-02
cg21952119	LY6G6D	6	Body	Open sea	69.04	5.63	74.47	6.06	-5.44	7.22E-05	2.13E-03
cg01858517	KCNMA1;KCNMA1;KCNMA1;KCNMA1	10	Body;Body;Body;Body	Island	36.66	6.75	42.10	6.33	-5.44	4.65E-05	1.46E-03
cg25727520	QPCT	2	Body	Open sea	73.65	6.20	79.09	5.19	-5.44	2.67E-05	9.01E-04
cg14079473		9	Intergenic	Open sea	68.87	2.39	74.30	2.48	-5.44	2.11E-15	1.37E-13
cg01376469	TRABD	22	Body	N_Shore	69.93	6.55	75.36	5.86	-5.44	2.98E-04	6.76E-03
cg10180281	SKAP2	7	Body	Open sea	33.36	5.38	38.80	6.14	-5.44	1.84E-06	7.93E-05
cg22976533	PACS2;PACS2	14	Body;Body	S_Shelf	44.01	7.79	49.45	5.41	-5.43	2.33E-04	5.58E-03
cg19841005	PIP5K1C	19	Body	Island	48.07	4.21	53.50	3.90	-5.43	1.49E-08	7.88E-07
cg20449692	CLDN11	3	1stExon	Island	16.69	4.10	22.13	6.26	-5.43	2.14E-06	9.12E-05
cg00297876	NAPSA	19	3'UTR	Island	31.18	7.17	36.61	8.50	-5.43	2.53E-03	3.33E-02
cg19838506		7	Intergenic	Open sea	72.39	5.77	77.82	5.63	-5.43	1.05E-04	2.92E-03
cg26023960	AKT1;AKT1;AKT1	14	Body;Body;Body	S_Shore	65.84	6.98	71.28	5.90	-5.43	1.91E-03	2.74E-02

cg07598199	MYOZ2	4	TSS200	Open sea	58.08	5.76	63.51	5.67	-5.43	5.68E-06	2.25E-04
cg01561869	PCDHGA2;PCDHGA3;PCDHGA3;PCDH(	5	Body;1stExon;1stExon;Body	Island	36.69	6.41	42.12	7.03	-5.43	3.85E-04	8.27E-03
cg04525852	ANKRD50;ANKRD50	4	5'UTR;5'UTR	N_Shore	17.81	5.30	23.25	5.54	-5.43	8.58E-05	2.46E-03
cg16644344	PRKCZ;PRKCZ;PRKCZ	1	Body;Body;Body	Open sea	65.31	6.45	70.74	6.58	-5.43	1.72E-03	2.54E-02
cg07589192	IGFALS	16	TSS1500	S_Shelf	87.14	8.01	92.57	3.07	-5.43	3.38E-05	1.11E-03
cg11921736	WDR52;WDR52	3	TSS200;TSS200	Island	37.14	4.43	42.57	5.49	-5.43	6.16E-08	3.12E-06
cg00031105	RXRB	6	Body	N_Shore	33.68	3.55	39.11	5.12	-5.43	1.02E-07	5.08E-06
cg03815147	PGAP3	17	Body	Open sea	55.96	5.07	61.39	4.08	-5.43	4.87E-07	2.28E-05
cg15095581		3	Intergenic	Open sea	56.95	4.87	62.37	4.81	-5.43	6.60E-07	3.03E-05
cg05399330	BSND	1	TSS200	S_Shore	39.80	6.57	45.22	6.40	-5.43	8.12E-04	1.46E-02
cg11415739	TRAF5;TRAF5	1	5'UTR;5'UTR	Open sea	52.59	4.05	58.01	3.82	-5.43	6.21E-08	3.15E-06
cg14417760	GABRA5;GABRA5	15	TSS1500;TSS200	N_Shore	30.22	5.12	35.65	6.98	-5.43	9.49E-05	2.68E-03
cg09496748	ANKRD44	2	Body	Island	68.17	5.59	73.60	4.35	-5.43	5.11E-07	2.38E-05
cg18733548		17	Intergenic	Island	51.43	5.98	56.85	6.34	-5.43	6.85E-05	2.04E-03
cg01942751	HDAC4	2	Body	N_Shore	83.87	6.03	89.29	4.17	-5.43	1.21E-05	4.48E-04
cg27599211	TESC;TESC;TESC	12	Body;Body;Body	N_Shore	32.02	6.33	37.45	7.71	-5.42	1.44E-03	2.24E-02
cg06715661	KCNN1	19	Body	N_Shelf	52.48	5.27	57.90	5.14	-5.42	1.47E-06	6.44E-05
cg17132030	SLC27A1	19	Body	S_Shore	37.37	7.78	42.79	7.43	-5.42	1.98E-04	4.91E-03
cg00664406	GRM2;GRM2	3	TSS1500;TSS1500	Island	37.50	5.75	42.93	5.01	-5.42	3.28E-06	1.35E-04
cg27425146		1	Intergenic	Open sea	21.03	5.09	26.45	7.60	-5.42	6.32E-04	1.21E-02
cg15732107		12	Intergenic	Island	70.02	7.80	75.45	9.45	-5.42	2.11E-03	2.93E-02
cg19821124	C14orf49	14	Body	Open sea	68.22	5.93	73.64	3.56	-5.42	4.60E-05	1.45E-03
cg04071270		5	Intergenic	Open sea	48.53	6.56	53.95	7.16	-5.42	1.09E-03	1.83E-02
cg01642461	SPTB;SPTB	14	1stExon;1stExon	Open sea	57.78	4.84	63.20	5.39	-5.42	1.82E-05	6.44E-04
cg19183879		15	Intergenic	Open sea	49.47	7.00	54.89	6.67	-5.42	1.68E-03	2.50E-02
cg06488655		15	Intergenic	Island	11.06	4.97	16.48	7.62	-5.42	5.25E-04	1.05E-02
cg13321105		1	Intergenic	S_Shelf	11.11	5.86	16.53	9.45	-5.42	7.98E-04	1.45E-02
cg03379552	CRTAC1	10	Body	Open sea	30.75	7.83	36.17	7.43	-5.42	1.61E-03	2.42E-02
cg09406760	PLEC1;PLEC1;PLEC1;PLEC1;PLEC1;PLE(	8	Body;Body;TSS1500;Body;TSS1500;Body	Island	77.43	7.26	82.84	7.45	-5.42	2.87E-03	3.62E-02
cg00566187	GALNT2	1	Body	Open sea	83.97	7.39	89.38	4.79	-5.42	7.88E-04	1.43E-02
cg26038465	MAFF;MAFF;MAFF;MAFF	22	Body;Body;Body;Body	Island	39.58	4.74	45.00	6.55	-5.42	2.05E-05	7.13E-04
cg25055120		2	Intergenic	Open sea	78.90	6.85	84.32	5.12	-5.42	8.26E-05	2.38E-03
cg19094530		10	Intergenic	N_Shore	41.81	5.53	47.22	6.86	-5.42	7.18E-05	2.12E-03
cg26689203	MYOZ2	4	Body	Open sea	28.59	5.03	34.01	4.87	-5.42	3.06E-09	1.67E-07
cg00822030	KCNAB2;KCNAB2	1	TSS1500;TSS1500	N_Shore	40.78	6.37	46.20	6.45	-5.41	3.67E-04	7.96E-03
cg03340701		11	Intergenic	N_Shore	59.44	7.00	64.85	7.38	-5.41	1.55E-03	2.36E-02
cg27292835	RASSF3	12	Body	Open sea	66.02	9.27	71.43	6.79	-5.41	2.24E-04	5.41E-03
cg03577128		3	Intergenic	Open sea	62.50	5.53	67.91	6.64	-5.41	1.27E-04	3.42E-03
cg27542767		6	Intergenic	Open sea	21.26	4.76	26.67	7.38	-5.41	4.73E-04	9.70E-03
cg09736440		6	Intergenic	N_Shore	39.80	3.30	45.22	2.93	-5.41	4.11E-10	2.33E-08
cg27504991	CUL7;CUL7;MRPL2	6	TSS1500;TSS1500;Body	S_Shore	66.84	6.06	72.25	5.25	-5.41	4.41E-04	9.20E-03
cg25030018	STATH;STATH	4	3'UTR;3'UTR	Open sea	52.89	7.31	58.30	7.85	-5.41	5.63E-04	1.11E-02
cg18079128	JAG1	20	Body	Island	13.95	3.81	19.36	5.25	-5.41	4.12E-09	2.24E-07
cg21841803		2	Intergenic	Open sea	77.44	6.12	82.85	6.73	-5.41	8.46E-05	2.43E-03
cg00342530	GARNL3;GARNL3	9	1stExon;5'UTR	Open sea	33.48	5.06	38.90	4.45	-5.41	1.24E-05	4.57E-04
cg20999932	PAPLN	14	Body	Island	35.22	6.11	40.63	8.09	-5.41	1.09E-03	1.82E-02
cg26542856		1	Intergenic	Open sea	59.46	6.38	64.87	6.85	-5.41	8.02E-04	1.45E-02
cg21964564	NFASC;NFASC;NFASC;NFASC;NFASC;N	1	Body;Body;Body;Body;Body;Body	Open sea	46.60	5.28	52.01	4.29	-5.41	2.87E-06	1.20E-04
cg25800328	AGBL2	11	Body	N_Shore	39.65	7.60	45.06	6.12	-5.41	1.78E-03	2.60E-02
cg27311590		4	Intergenic	Island	70.01	8.44	75.42	6.94	-5.41	2.61E-03	3.40E-02
cg14376607	FLOT1	6	Body	N_Shelf	49.86	3.62	55.27	4.49	-5.41	1.57E-07	7.72E-06
cg14445366		17	Intergenic	Island	31.51	5.51	36.91	5.67	-5.40	9.46E-05	2.67E-03
cg14026106	MARCH8;MARCH8;MARCH8	10	Body;Body;Body	Open sea	52.85	6.37	58.25	5.19	-5.40	7.37E-07	3.37E-05

cg14414415	FAM50B	6	TSS1500	N_Shore	51.69	6.42	57.10	7.62	-5.40	2.41E-03	3.23E-02
cg10924875		8	Intergenic	N_Shore	64.83	7.32	70.24	7.78	-5.40	1.47E-03	2.27E-02
cg04865442	FSTL1	3	Body	N_Shore	27.14	6.07	32.54	5.99	-5.40	3.05E-04	6.90E-03
cg09191731	HS3ST2	16	TSS1500	Island	31.80	5.11	37.20	7.24	-5.40	5.86E-05	1.78E-03
cg19371097	RCC2;RCC2	1	3'UTR;3'UTR	S_Shore	83.69	2.97	89.09	1.78	-5.40	6.72E-16	4.44E-14
cg16907934	FAM50B	6	TSS1500	N_Shore	47.07	5.25	52.47	5.83	-5.40	1.58E-04	4.09E-03
cg10713515	COMMD3	10	TSS1500	N_Shore	35.94	4.89	41.34	5.93	-5.40	7.72E-05	2.25E-03
cg08894020		5	Intergenic	Open sea	40.33	4.75	45.73	5.64	-5.40	1.38E-06	6.07E-05
cg14407987	RAPSN;RAPSN	11	TSS200;TSS200	Open sea	59.44	7.05	64.84	8.00	-5.40	1.66E-03	2.48E-02
cg05575639	CHD7	8	5'UTR	S_Shore	53.79	7.30	59.18	7.27	-5.40	3.24E-03	3.95E-02
cg03176917		19	Intergenic	N_Shelf	24.11	9.12	29.51	9.59	-5.40	3.58E-03	4.23E-02
cg07089660	DEFA4	8	5'UTR	Open sea	60.05	7.01	65.45	8.42	-5.40	3.88E-03	4.47E-02
cg20717075	GPC6	13	Body	Open sea	75.73	7.90	81.13	3.34	-5.40	4.87E-05	1.52E-03
cg24352349	C22orf9;C22orf9;MIR1249	22	Body;Body;TSS1500	N_Shore	69.00	6.52	74.39	4.23	-5.40	8.55E-04	1.52E-02
cg02238549		10	Intergenic	S_Shore	44.24	5.85	49.64	7.35	-5.39	5.79E-05	1.77E-03
cg13260377	BAT2	6	Body	S_Shelf	74.83	5.80	80.22	4.57	-5.39	3.91E-05	1.25E-03
cg13333304	CLDN11	3	TSS1500	N_Shore	16.01	3.62	21.40	6.15	-5.39	1.58E-06	6.86E-05
cg05000446	C9orf131;C9orf131;C9orf131;C9orf13	9	1stExon;Body;Body;Body	Open sea	37.25	5.37	42.64	6.59	-5.39	2.29E-04	5.51E-03
cg17023856	PRKCZ;PRKCZ;PRKCZ	1	5'UTR;Body;5'UTR	N_Shelf	70.99	4.55	76.38	4.88	-5.39	1.57E-06	6.84E-05
cg20859933		11	Intergenic	S_Shelf	41.43	5.09	46.82	6.16	-5.39	1.43E-04	3.77E-03
cg22659676		5	Intergenic	Island	42.98	4.22	48.38	3.66	-5.39	5.67E-09	3.06E-07
cg22486630	LPPR2;LPPR2	19	Body;Body	Island	56.01	5.28	61.41	5.92	-5.39	7.42E-05	2.18E-03
cg27424995	AHDC1	1	5'UTR	Island	11.31	4.90	16.71	6.00	-5.39	5.53E-05	1.70E-03
cg01893695		14	Intergenic	Open sea	64.43	5.65	69.82	4.11	-5.39	2.02E-06	8.67E-05
cg21475097	PRDM16;PRDM16	1	Body;Body	Island	10.61	3.91	16.00	7.55	-5.39	5.61E-06	2.22E-04
cg18494902	BMF;BMF;BMF;BMF;BMF	15	TSS1500;5'UTR;TSS1500;5'UTR;1stExon	N_Shelf	45.66	7.68	51.05	8.63	-5.39	9.27E-04	1.62E-02
cg05576949	IGF2BP2;IGF2BP2	3	Body;Body	N_Shore	41.49	6.61	46.88	7.28	-5.39	1.60E-03	2.41E-02
cg07601536	RREB1;RREB1;RREB1;RREB1	6	Body;Body;Body;Body	S_Shore	47.03	4.41	52.41	6.91	-5.39	2.14E-04	5.21E-03
cg03206681		3	Intergenic	Open sea	40.03	4.48	45.42	5.36	-5.39	5.69E-07	2.64E-05
cg14703002	HIST1H1A	6	TSS200	N_Shelf	12.66	4.67	18.05	7.47	-5.39	3.45E-04	7.60E-03
cg05279761	ACAD11	3	Body	Open sea	59.56	6.48	64.95	5.57	-5.39	2.15E-04	5.23E-03
cg17204557	LOC84931	2	Body	Open sea	62.79	4.94	68.17	4.40	-5.39	2.31E-06	9.79E-05
cg07054641	WDR52;WDR52	3	TSS200;TSS200	Island	33.09	5.85	38.47	6.68	-5.39	2.03E-05	7.09E-04
cg00674706		6	Intergenic	Island	37.39	6.99	42.78	6.15	-5.38	1.54E-04	4.00E-03
cg20734030		12	Intergenic	Open sea	56.86	7.63	62.24	7.33	-5.38	5.16E-04	1.04E-02
cg02043329	FNDC3A;FNDC3A	13	Body;Body	Open sea	71.83	5.69	77.22	4.33	-5.38	1.39E-05	5.07E-04
cg01678701		17	Intergenic	Open sea	40.33	5.84	45.72	6.16	-5.38	2.43E-05	8.31E-04
cg19837815	C7orf29;LRRC61;LRRC61	7	TSS200;5'UTR;5'UTR	Open sea	52.13	6.30	57.51	7.55	-5.38	1.09E-03	1.82E-02
cg21209091	DKK4	8	TSS1500	Open sea	36.55	4.53	41.93	4.56	-5.38	1.29E-08	6.82E-07
cg00059737	VPS13D;VPS13D	1	Body;Body	Open sea	37.21	6.55	42.59	6.94	-5.38	2.36E-03	3.18E-02
cg12368105		11	Intergenic	Open sea	51.96	5.34	57.34	6.82	-5.38	2.30E-04	5.53E-03
cg11849703	FGFR4;FGFR4;FGFR4	5	Body;Body;Body	S_Shelf	46.42	5.05	51.80	6.97	-5.38	2.30E-04	5.53E-03
cg01727419	SH3PXD2A	10	TSS1500	S_Shore	40.34	6.25	45.72	6.75	-5.38	2.28E-03	3.10E-02
cg06522515	THPO	3	Body	Open sea	75.07	4.67	80.45	4.53	-5.38	4.56E-06	1.84E-04
cg19798832	PARP12	7	Body	N_Shelf	35.34	4.34	40.72	3.88	-5.38	4.27E-07	2.01E-05
cg14202325	IQSEC1	3	TSS1500	Island	31.20	4.57	36.57	5.62	-5.38	3.87E-07	1.83E-05
cg07138686		14	Intergenic	Open sea	63.92	5.35	69.29	6.10	-5.38	9.12E-05	2.59E-03
cg08927631	C5orf33;C5orf33	5	5'UTR;Body	N_Shelf	54.83	6.12	60.20	6.81	-5.37	1.59E-03	2.40E-02
cg17825770	TRPS1	8	Body	Open sea	67.09	7.88	72.46	6.43	-5.37	3.21E-04	7.17E-03
cg00135888	MIR130A	11	TSS1500	Open sea	23.13	5.31	28.50	6.23	-5.37	1.47E-04	3.86E-03
cg18626035	NHEDC2	4	TSS200	S_Shore	63.12	6.86	68.50	7.21	-5.37	1.24E-03	2.01E-02
cg09374293	PRMT2;PRMT2	21	Body;Body	Island	28.02	6.91	33.39	7.89	-5.37	1.95E-03	2.78E-02
cg21247948	PRKD3	2	TSS1500	Open sea	57.25	6.28	62.62	5.89	-5.37	2.09E-04	5.11E-03

cg09183146	UNKL	16	TSS200	Island	39.72	5.61	45.09	7.25	-5.37	6.01E-04	1.17E-02
cg17129188	NDRG1;NDRG1	8	5'UTR;5'UTR	N_Shore	38.01	5.82	43.38	6.05	-5.37	1.41E-04	3.72E-03
cg10852698		6	Intergenic	Open sea	58.27	5.72	63.64	4.87	-5.37	1.19E-05	4.40E-04
cg17202086	PAK1;PAK1	11	5'UTR;5'UTR	Island	47.85	4.26	53.22	4.35	-5.37	6.29E-08	3.19E-06
cg11790074		14	Intergenic	Open sea	66.37	4.34	71.74	4.12	-5.37	5.18E-07	2.41E-05
cg05825053		16	Intergenic	Open sea	63.76	6.71	69.13	7.18	-5.37	5.94E-04	1.16E-02
cg20948091	BSN	3	Body	Open sea	49.39	4.27	54.76	5.19	-5.37	5.77E-07	2.67E-05
cg01081346	CPT1B;CPT1B;CPT1B;CPT1B;CHKB-CPT	22	TSS200;TSS200;TSS1500;TSS1500;Body;TS	S_Shore	52.66	6.78	58.03	6.45	-5.37	1.43E-03	2.22E-02
cg21350349	LOC440839;PSD4	2	Body;5'UTR	Open sea	36.96	3.93	42.33	4.28	-5.37	2.59E-07	1.25E-05
cg26216323	RAB11FIP1;RAB11FIP1;RAB11FIP1	8	Body;Body;Body	Open sea	36.81	4.47	42.18	5.21	-5.36	9.49E-06	3.59E-04
cg11737172	KBTBD11	8	5'UTR	S_Shore	36.76	6.46	42.13	7.18	-5.36	4.31E-04	9.04E-03
cg27095820	TMCO3	13	Body	N_Shore	60.22	5.06	65.59	4.28	-5.36	1.04E-05	3.89E-04
cg22473846	OSMR;OSMR	5	5'UTR;5'UTR	S_Shore	13.88	4.23	19.24	5.18	-5.36	3.10E-07	1.48E-05
cg15026634	OTX1	2	5'UTR	N_Shore	47.14	4.57	52.51	6.07	-5.36	2.23E-06	9.49E-05
cg21027282	RAD51L1;RAD51L1;RAD51L1	14	Body;Body;Body	Open sea	70.66	5.80	76.03	5.92	-5.36	1.74E-04	4.42E-03
cg26870936	RECQL5;LOC643008;LOC643008	17	Body;TSS1500;5'UTR	S_Shelf	59.20	4.04	64.56	4.74	-5.36	1.53E-07	7.53E-06
cg13222036		1	Intergenic	Open sea	25.39	5.66	30.75	5.66	-5.36	4.47E-05	1.41E-03
cg19150916	MRPL23	11	Body	N_Shore	82.31	5.25	87.67	3.59	-5.36	2.40E-06	1.02E-04
cg27534957		2	Intergenic	Open sea	21.96	6.95	27.33	7.87	-5.36	5.83E-04	1.14E-02
cg10904972	USP2;USP2	11	3'UTR;3'UTR	Island	33.20	7.06	38.56	7.20	-5.36	3.47E-03	4.14E-02
cg27268835		16	Intergenic	Open sea	70.87	5.45	76.23	4.57	-5.36	7.36E-06	2.85E-04
cg11904906	LOC151174;LOC643387;LOC151174	2	Body;TSS1500;Body	N_Shore	15.81	4.72	21.17	6.90	-5.36	3.62E-06	1.49E-04
cg09233156		15	Intergenic	Open sea	48.32	5.12	53.68	5.64	-5.36	4.90E-06	1.96E-04
cg05008070	DACT1;DACT1	14	Body;Body	S_Shore	57.96	6.81	63.32	5.46	-5.36	2.55E-04	6.00E-03
cg24706115	TRPV4;TRPV4	12	5'UTR;5'UTR	Open sea	49.77	6.42	55.14	5.95	-5.36	1.32E-04	3.52E-03
cg00514407	SERPINE2;SERPINE2;SERPINE2	2	TSS1500;TSS1500;TSS1500	Island	16.32	3.88	21.68	7.09	-5.36	7.93E-06	3.05E-04
cg09511724	CASZ1;CASZ1	1	5'UTR;5'UTR	Open sea	75.22	6.36	80.58	6.27	-5.36	1.18E-04	3.21E-03
cg02043791	FGFR1;FGFR1;FGFR1;FGFR1;FGFR1;FG	8	5'UTR;5'UTR;5'UTR;5'UTR;5'UTR;5'UTR;5'U	N_Shore	67.76	4.40	73.12	4.92	-5.36	3.10E-06	1.29E-04
cg01409163	ERICH1	8	Body	Open sea	75.55	4.96	80.91	5.09	-5.36	2.81E-05	9.41E-04
cg18729186	SNORD126;MIR1201;CCNB1IP1;CCNB	14	TSS1500;TSS1500;5'UTR;5'UTR;5'UTR;5'UT	Open sea	51.16	5.79	56.52	6.86	-5.36	4.71E-04	9.68E-03
cg22538871		19	Intergenic	Island	59.81	5.98	65.17	7.24	-5.36	4.78E-04	9.77E-03
cg02360514	FOSB;FOSB	19	Body;Body	Island	15.82	5.03	21.18	8.75	-5.36	2.31E-03	3.13E-02
cg00594099	AHDC1	1	5'UTR	N_Shore	60.06	4.69	65.42	4.11	-5.36	2.43E-06	1.02E-04
cg19951107	TMPRSS7;TMPRSS7	3	5'UTR;Body	Open sea	60.13	4.45	65.49	4.78	-5.36	1.93E-07	9.42E-06
cg10280048	MVD	16	Body	S_Shore	73.31	6.24	78.67	5.07	-5.36	1.48E-04	3.86E-03
cg05999324	PDCD4;PDCD4	10	Body;Body	Open sea	70.78	6.01	76.14	4.72	-5.36	2.62E-04	6.13E-03
cg20519373	SAMD12;SAMD12	8	Body;Body	Open sea	42.28	5.17	47.63	5.71	-5.36	1.18E-05	4.36E-04
cg22105158	C19orf77	19	TSS200	N_Shore	22.01	4.09	27.37	5.50	-5.35	8.69E-06	3.32E-04
cg24010531		19	Intergenic	Island	76.18	7.45	81.53	5.07	-5.35	2.19E-03	3.01E-02
cg16585333	TLR5	1	5'UTR	Open sea	17.70	5.52	23.05	7.48	-5.35	8.55E-05	2.46E-03
cg08295111	PHF15	5	5'UTR	S_Shelf	61.99	5.45	67.34	4.52	-5.35	5.74E-05	1.75E-03
cg22622123	SLC37A4;SLC37A4;SLC37A4;SLC37A4	11	TSS1500;TSS1500;TSS1500;TSS1500	S_Shore	56.46	5.15	61.81	4.59	-5.35	2.99E-06	1.24E-04
cg06087185		6	Intergenic	Island	39.48	7.53	44.83	6.14	-5.35	1.03E-03	1.75E-02
cg24330456	RNF39;RNF39	6	Body;Body	Island	55.18	6.13	60.53	8.62	-5.35	1.35E-03	2.13E-02
cg27438690	AKT3;AKT3	1	Body;Body	Open sea	64.30	6.73	69.65	5.12	-5.35	4.19E-04	8.83E-03
cg22592160		5	Intergenic	Open sea	38.85	5.22	44.20	4.76	-5.35	6.15E-06	2.41E-04
cg04448709	GAN	16	Body	S_Shore	69.64	5.68	74.99	4.77	-5.35	4.91E-06	1.97E-04
cg12463325	TCF7L1	2	Body	Open sea	48.07	6.90	53.42	6.24	-5.35	1.31E-03	2.08E-02
cg25650185	ZNF431	19	TSS200	Open sea	32.44	6.51	37.79	7.35	-5.35	5.44E-04	1.08E-02
cg02229135	MIR548F5;NBEA	13	Body;Body	Island	33.65	5.98	39.00	9.58	-5.35	3.16E-03	3.88E-02
cg25866552		7	Intergenic	Island	55.81	6.97	61.16	6.13	-5.35	5.45E-04	1.08E-02
cg20031900	FAM193A	4	3'UTR	Island	60.83	4.52	66.18	5.11	-5.35	4.52E-06	1.83E-04
cg01487661		7	Intergenic	Island	43.01	6.94	48.36	8.41	-5.35	1.47E-03	2.27E-02

cg15834355	HOXC4	12	5'UTR	S_Shore	50.74	6.70	56.09	6.78	-5.35	3.32E-04	7.36E-03
cg16286281	EXT1	8	Body	N_Shelf	67.40	7.47	72.75	7.34	-5.35	9.56E-05	2.70E-03
cg07603484	NUP62;NUP62;IL4I1;ATF5;NUP62;NUF	19	TSS1500;TSS1500;TSS1500;Body;TSS1500;	S_Shore	39.76	6.26	45.10	7.95	-5.34	4.33E-03	4.82E-02
cg05276978	NEDD4L;NEDD4L;NEDD4L;NEDD4L;NE	18	5'UTR;TSS1500;TSS1500;Body;TSS1500;Bo	Open sea	32.78	4.05	38.12	4.65	-5.34	1.82E-06	7.84E-05
cg06476685	ANKMY1;ANKMY1	2	Body;Body	Island	52.63	6.26	57.98	5.98	-5.34	4.64E-05	1.46E-03
cg09168808		12	Intergenic	N_Shore	41.33	5.24	46.67	4.89	-5.34	2.49E-06	1.05E-04
cg15791248	FGFR1;FGFR1;FGFR1;FGFR1;FGFR1;FC	8	TSS1500;TSS1500;TSS1500;TSS1500;TSS15	S_Shore	67.59	4.85	72.94	4.72	-5.34	9.69E-06	3.66E-04
cg27265617	ATP2B2;ATP2B2	3	Body;Body	S_Shore	63.39	4.65	68.73	4.45	-5.34	6.15E-07	2.84E-05
cg13900763		9	Intergenic	Open sea	63.38	7.47	68.72	5.85	-5.34	1.42E-03	2.22E-02
cg13858974	FRMD3	9	Body	N_Shelf	65.66	6.09	71.00	5.27	-5.34	2.07E-05	7.19E-04
cg01567051	CMIP	16	Body	Open sea	65.87	6.80	71.21	5.71	-5.34	1.37E-04	3.63E-03
cg19246197	USP2;USP2	11	Body;Body	N_Shelf	71.66	5.40	76.99	4.61	-5.34	2.63E-07	1.27E-05
cg23733845	SH2B2	7	Body	Island	33.49	5.10	38.82	5.87	-5.34	2.33E-04	5.58E-03
cg09571082		6	Intergenic	Open sea	64.38	5.23	69.71	5.38	-5.34	2.79E-05	9.37E-04
cg11785537	SCNN1A;SCNN1A;SCNN1A;SCNN1A	12	Body;TSS1500;1stExon;5'UTR	Open sea	64.39	7.08	69.73	8.18	-5.34	5.41E-04	1.08E-02
cg13249244		18	Intergenic	N_Shore	60.51	8.30	65.85	8.89	-5.34	2.15E-03	2.97E-02
cg08354681	CDYL	6	Body	N_Shore	42.22	2.95	47.56	4.46	-5.34	8.26E-09	4.42E-07
cg10588705	USP22	17	3'UTR	Open sea	55.86	5.88	61.20	4.43	-5.33	1.17E-04	3.19E-03
cg05427381	AHNAK;AHNAK	11	5'UTR;5'UTR	N_Shelf	61.26	9.33	66.59	7.39	-5.33	1.88E-03	2.71E-02
cg18588589		6	Intergenic	Island	22.59	5.82	27.92	7.27	-5.33	3.98E-04	8.48E-03
cg23194926	STX2;STX2	12	Body;Body	Island	39.07	8.41	44.40	8.48	-5.33	7.39E-05	2.17E-03
cg11694519	CRYAB;HSPB2	11	TSS1500;1stExon	Open sea	34.35	5.38	39.68	7.36	-5.33	8.07E-05	2.34E-03
cg00803758		4	Intergenic	Open sea	47.13	6.31	52.46	6.46	-5.33	9.05E-04	1.59E-02
cg10330056		2	Intergenic	Open sea	17.96	4.43	23.29	5.76	-5.33	2.87E-06	1.20E-04
cg15291243	LHX1	17	Body	Island	33.22	5.24	38.56	5.02	-5.33	3.32E-07	1.58E-05
cg03769116	KIAA0355	19	Body	Open sea	45.63	5.27	50.96	6.81	-5.33	3.41E-04	7.53E-03
cg23378546		17	Intergenic	N_Shelf	47.75	7.68	53.08	7.67	-5.33	2.42E-03	3.23E-02
cg19753675		2	Intergenic	N_Shore	33.24	4.45	38.57	5.67	-5.33	9.28E-07	4.19E-05
cg10024100	TRIT1	1	TSS1500	S_Shore	69.30	5.06	74.63	4.50	-5.33	2.54E-09	1.39E-07
cg24718903	RRAS	19	3'UTR	Open sea	54.37	4.04	59.70	4.09	-5.33	5.58E-08	2.84E-06
cg10357060	DPYSL5	2	Body	Open sea	63.59	5.36	68.92	6.79	-5.33	3.32E-04	7.38E-03
cg19213703		3	Intergenic	Open sea	71.37	5.89	76.70	4.88	-5.33	6.24E-05	1.88E-03
cg10120764		1	Intergenic	Open sea	42.71	5.74	48.03	7.46	-5.33	4.01E-04	8.53E-03
cg19769521	IQSEC1	3	Body	S_Shelf	64.35	4.89	69.68	4.18	-5.33	2.82E-06	1.18E-04
cg02913568		3	Intergenic	Open sea	75.86	4.88	81.18	5.72	-5.33	1.54E-05	5.54E-04
cg04176122	BCL9L	11	Body	N_Shore	47.62	4.76	52.95	4.31	-5.33	3.08E-06	1.28E-04
cg05038152	LIPJ	10	TSS1500	S_Shelf	64.18	5.12	69.51	4.49	-5.33	3.47E-06	1.43E-04
cg00782690	GORASP2	2	Body	Open sea	59.91	5.96	65.23	6.58	-5.32	1.26E-03	2.03E-02
cg18465615		15	Intergenic	Open sea	71.87	4.95	77.19	4.87	-5.32	1.37E-05	4.98E-04
cg23624957	HDAC2	6	3'UTR	Open sea	41.33	5.83	46.65	7.39	-5.32	6.53E-04	1.24E-02
cg25928521	PDE3B	11	3'UTR	Open sea	73.15	7.05	78.48	6.96	-5.32	2.26E-04	5.44E-03
cg09459529		5	Intergenic	N_Shelf	33.14	5.90	38.46	6.19	-5.32	5.91E-04	1.15E-02
cg22535103	C8orf71	8	Body	Island	62.40	8.30	67.72	8.22	-5.32	1.56E-03	2.36E-02
cg19228683		11	Intergenic	S_Shelf	60.51	7.02	65.84	7.11	-5.32	8.22E-04	1.48E-02
cg07480653	GAST	17	TSS1500	Open sea	64.55	6.93	69.88	4.67	-5.32	8.78E-05	2.51E-03
cg12855851	PGC;PGC	6	TSS200;TSS200	Open sea	68.52	6.12	73.84	6.48	-5.32	4.17E-04	8.81E-03
cg13137458	PAK1;PAK1	11	5'UTR;5'UTR	Open sea	54.74	4.90	60.06	5.42	-5.32	6.63E-07	3.05E-05
cg09895166	LRBA	4	Body	Open sea	54.85	6.36	60.18	5.73	-5.32	1.05E-03	1.77E-02
cg15742496	TRIM6-TRIM34;TRIM6;TRIM6	11	Body;Body;5'UTR	Open sea	54.84	7.12	60.16	7.73	-5.32	3.87E-03	4.46E-02
cg00341522		2	Intergenic	Open sea	63.69	5.88	69.01	4.65	-5.32	1.21E-04	3.28E-03
cg22294063	NUDT18	8	3'UTR	N_Shore	72.91	5.25	78.24	5.77	-5.32	3.46E-06	1.42E-04
cg20075683	CCNI2	5	Body	Island	74.14	5.54	79.46	6.32	-5.32	6.44E-05	1.93E-03
cg09574499	HOXA4	7	Body	N_Shore	45.24	6.42	50.56	8.55	-5.32	1.95E-03	2.77E-02

cg26697065	SEPHS2	16	1stExon	N_Shore	43.65	7.25	48.97	5.73	-5.32	1.62E-03	2.43E-02
cg00190206		2	Intergenic	N_Shore	51.16	4.89	56.48	5.86	-5.32	1.11E-05	4.13E-04
cg19768143	DAAM1	14	5'UTR	Open sea	69.62	5.22	74.94	5.34	-5.32	1.93E-05	6.76E-04
cg12050434		12	Intergenic	Open sea	28.73	5.00	34.05	7.53	-5.32	2.18E-04	5.30E-03
cg11200568	FAM20C	7	Body	S_Shore	72.81	5.83	78.12	4.15	-5.32	3.93E-05	1.26E-03
cg16317516	MADD;MADD;MADD;MADD;MADD;N	11	5'UTR;5'UTR;5'UTR;5'UTR;5'UTR;5'UTR;5'L	S_Shelf	62.51	5.77	67.83	6.18	-5.32	1.87E-06	8.05E-05
cg07265444	PION	7	Body	Open sea	35.57	6.35	40.89	6.07	-5.32	5.22E-05	1.61E-03
cg26289712		8	Intergenic	N_Shore	56.76	2.89	62.07	3.10	-5.32	5.48E-13	3.38E-11
cg06720768		2	Intergenic	Open sea	73.63	8.06	78.95	8.53	-5.32	3.37E-03	4.06E-02
cg13057055		17	Intergenic	N_Shelf	29.12	3.76	34.43	3.38	-5.32	3.64E-10	2.06E-08
cg20845639		8	Intergenic	S_Shore	61.37	6.54	66.68	7.24	-5.31	1.40E-03	2.20E-02
cg20459035	ISOC1	5	Body	S_Shelf	74.93	6.64	80.25	6.21	-5.31	7.11E-04	1.33E-02
cg02022102	HOXA4	7	3'UTR	N_Shore	68.03	5.92	73.34	6.94	-5.31	5.86E-04	1.14E-02
cg01039763		9	Intergenic	Island	78.49	6.69	83.80	5.23	-5.31	1.08E-04	3.00E-03
cg14337614		17	Intergenic	Open sea	36.12	6.34	41.43	8.43	-5.31	1.69E-03	2.51E-02
cg20564496		7	Intergenic	Island	28.66	7.99	33.97	7.85	-5.31	7.86E-04	1.43E-02
cg03509898	ZFYVE21	14	Body	N_Shelf	70.39	6.05	75.71	4.06	-5.31	5.09E-05	1.58E-03
cg15394860	H19	11	Body	N_Shore	64.52	3.82	69.83	4.51	-5.31	6.52E-08	3.30E-06
cg01190484	TRIM40;TRIM40	6	1stExon;5'UTR	Open sea	50.36	5.54	55.67	7.13	-5.31	2.80E-05	9.38E-04
cg19334350	PTPRN2;PTPRN2;PTPRN2	7	Body;Body;Body	S_Shore	34.80	5.44	40.11	7.49	-5.31	1.64E-04	4.22E-03
cg01986682	ALDOA;ALDOA;ALDOA	16	TSS200;TSS1500;5'UTR	N_Shore	16.51	4.41	21.82	6.94	-5.31	4.48E-05	1.41E-03
cg25827309	PPAP2C;PPAP2C;PPAP2C	19	Body;Body;Body	Open sea	57.94	5.69	63.25	6.02	-5.31	1.02E-04	2.85E-03
cg05269440		2	Intergenic	Island	63.12	6.75	68.43	6.64	-5.31	1.64E-04	4.22E-03
cg11872743		17	Intergenic	Island	57.89	5.80	63.20	3.90	-5.31	1.88E-05	6.62E-04
cg12003941	KIF25;KIF25	6	Body;Body	Island	15.41	6.08	20.71	7.69	-5.31	7.76E-04	1.42E-02
cg08065122	SLC25A22	11	Body	N_Shelf	65.38	7.21	70.69	7.22	-5.31	2.06E-03	2.88E-02
cg21581312	LOC723972	15	TSS200	Open sea	33.25	5.29	38.55	6.43	-5.31	1.17E-04	3.19E-03
cg03972071	ZADH2	18	Body	Island	50.99	7.46	56.30	7.33	-5.31	5.95E-04	1.16E-02
cg00064778		5	Intergenic	N_Shore	32.40	5.22	37.71	6.90	-5.31	9.33E-05	2.64E-03
cg03910557	HEY2	6	Body	S_Shelf	34.34	5.90	39.65	6.15	-5.31	4.20E-04	8.86E-03
cg03762366	HOXC11	12	TSS1500	N_Shore	51.16	5.87	56.46	8.07	-5.31	9.64E-04	1.67E-02
cg12073911	HNRNPD;HNRNPD;HNRNPD;HNRNPD	4	Body;Body;Body;Body	Open sea	61.48	4.40	66.79	5.01	-5.31	2.27E-07	1.10E-05
cg20675702		10	Intergenic	N_Shelf	61.70	6.96	67.00	5.52	-5.30	4.73E-04	9.70E-03
cg21850735	NPTN;NPTN;NPTN;NPTN	15	Body;Body;Body;Body	Open sea	71.78	6.82	77.09	7.01	-5.30	7.23E-05	2.13E-03
cg06664254		16	Intergenic	Open sea	65.02	4.68	70.33	4.26	-5.30	1.11E-06	4.96E-05
cg11312087	PCCA;PCCA	13	Body;Body	Open sea	79.00	3.84	84.30	2.92	-5.30	1.87E-10	1.07E-08
cg07570470		8	Intergenic	Island	39.34	7.20	44.64	7.88	-5.30	5.60E-04	1.10E-02
cg21516302		2	Intergenic	Open sea	80.86	7.20	86.17	5.95	-5.30	4.58E-04	9.46E-03
cg08362102	ANP32A	15	Body	Open sea	22.02	4.48	27.33	5.09	-5.30	2.88E-07	1.38E-05
cg07733481	SEMA5B	3	5'UTR	Open sea	43.82	4.59	49.13	6.06	-5.30	4.34E-06	1.76E-04
cg22226904		1	Intergenic	S_Shelf	41.31	6.05	46.62	6.65	-5.30	1.46E-03	2.26E-02
cg17176894	LRRC8C	1	Body	Open sea	57.59	4.50	62.89	5.66	-5.30	6.28E-06	2.46E-04
cg08750432		8	Intergenic	N_Shore	51.37	6.05	56.67	6.28	-5.30	1.16E-04	3.17E-03
cg09608949	FXYD6;FXYD6;FXYD6;FXYD6;FXYD6	11	TSS1500;TSS1500;TSS1500;TSS1500;TSS20	S_Shore	43.54	5.53	48.84	7.34	-5.30	8.61E-04	1.53E-02
cg23960022	SCAF1	19	3'UTR	Open sea	58.58	3.83	63.88	3.98	-5.30	4.43E-08	2.27E-06
cg19791606	RIN2	20	TSS1500	Open sea	42.16	6.34	47.46	5.52	-5.30	4.59E-05	1.44E-03
cg27057275		13	Intergenic	Open sea	69.79	4.68	75.08	5.15	-5.30	2.26E-06	9.60E-05
cg18365406	LPPR2;LPPR2	19	Body;Body	Island	29.96	3.89	35.25	4.96	-5.30	6.97E-07	3.19E-05
cg03347444	QSER1	11	5'UTR	Open sea	43.10	6.11	48.40	6.57	-5.30	3.15E-04	7.07E-03
cg19311431	TANK	2	Body	N_Shelf	86.76	9.96	92.05	3.12	-5.30	7.55E-04	1.39E-02
cg11548404	FBRSL1	12	Body	S_Shelf	77.55	6.21	82.85	6.44	-5.29	2.02E-04	4.98E-03
cg01269191	KRAS;KRAS	12	TSS1500;TSS1500	Open sea	25.71	4.99	31.00	6.46	-5.29	1.77E-04	4.48E-03
cg02873541		11	Intergenic	Open sea	75.66	5.15	80.95	3.93	-5.29	5.18E-06	2.07E-04

cg02800817	TRIM26	6	3'UTR	Open sea	77.78	5.26	83.07	4.84	-5.29	3.12E-05	1.03E-03
cg13270163		10	Intergenic	Open sea	62.51	7.46	67.81	7.96	-5.29	3.88E-03	4.47E-02
cg03085377	RB1	13	Body	Island	45.88	5.24	51.18	7.60	-5.29	7.91E-04	1.44E-02
cg02937671	ZFYVE28	4	Body	Island	61.92	8.48	67.22	7.07	-5.29	8.68E-04	1.54E-02
cg18119192	GATA4	8	Body	S_Shore	19.66	5.22	24.95	7.06	-5.29	5.94E-04	1.16E-02
cg09934381		12	Intergenic	Open sea	33.07	4.73	38.36	5.09	-5.29	5.09E-06	2.03E-04
cg01267709	SLC7A8;SLC7A8	14	Body;TSS200	Open sea	44.63	5.06	49.92	4.44	-5.29	2.81E-06	1.17E-04
cg01706943	PCOLCE	7	TSS200	N_Shore	30.27	5.34	35.56	9.12	-5.29	4.14E-03	4.67E-02
cg12684958	PPBPL2	4	TSS1500	Open sea	76.49	5.93	81.78	4.84	-5.29	3.69E-07	1.75E-05
cg08951273	C6orf15	6	3'UTR	Open sea	69.19	7.81	74.48	7.79	-5.29	3.67E-03	4.31E-02
cg16153294	H19;MIR675	11	Body;TSS200	Island	71.04	3.33	76.33	4.75	-5.29	2.05E-08	1.07E-06
cg26235748	FLRT2	14	5'UTR	Open sea	59.63	5.80	64.92	5.58	-5.29	6.45E-06	2.52E-04
cg00899855	ZCCHC14	16	Body	Open sea	52.80	5.90	58.09	5.60	-5.29	4.05E-05	1.29E-03
cg10316764	TAOK2;TAOK2	16	TSS1500;TSS1500	Island	45.72	6.11	51.01	6.15	-5.29	5.24E-04	1.05E-02
cg11352369		19	Intergenic	Island	49.21	6.34	54.49	6.83	-5.29	1.63E-03	2.44E-02
cg27534671	THSD1;THSD1	13	5'UTR;5'UTR	N_Shore	26.74	5.73	32.03	4.37	-5.29	1.97E-08	1.03E-06
cg05785481	POU3F1;POU3F1	1	3'UTR;1stExon	N_Shore	43.26	5.24	48.55	5.84	-5.29	2.93E-07	1.40E-05
cg11331717		4	Intergenic	Open sea	40.52	7.02	45.80	6.73	-5.29	1.46E-03	2.26E-02
cg02820072	TDRD12	19	TSS200	Island	82.42	5.94	87.71	4.33	-5.29	2.33E-06	9.89E-05
cg09320277		14	Intergenic	Open sea	67.77	5.20	73.06	4.68	-5.29	3.01E-06	1.25E-04
cg16622613		13	Intergenic	Open sea	46.34	7.72	51.62	7.56	-5.28	1.36E-03	2.15E-02
cg23928763	EXD3	9	5'UTR	Island	76.46	7.06	81.74	5.45	-5.28	7.20E-05	2.12E-03
cg01393632		12	Intergenic	S_Shore	5.71	1.79	10.99	3.77	-5.28	1.51E-16	1.01E-14
cg26120813		12	Intergenic	S_Shore	44.84	6.72	50.12	6.79	-5.28	1.77E-03	2.60E-02
cg14003853	AKAP13;AKAP13;AKAP13	15	Body;Body;Body	Open sea	41.84	5.07	47.12	6.20	-5.28	1.98E-05	6.93E-04
cg02703145		9	Intergenic	Island	62.62	8.12	67.90	7.84	-5.28	9.06E-04	1.59E-02
cg22800233		10	Intergenic	N_Shore	48.21	5.05	53.49	4.81	-5.28	4.43E-07	2.08E-05
cg17118391		4	Intergenic	S_Shore	45.85	6.77	51.13	7.51	-5.28	1.63E-03	2.44E-02
cg11015251	HOXA4	7	TSS200	Island	30.45	4.53	35.73	5.60	-5.28	1.96E-06	8.41E-05
cg09632136	NNMT	11	TSS1500	Open sea	26.21	5.97	31.49	6.57	-5.28	1.78E-03	2.60E-02
cg08567941		2	Intergenic	Open sea	49.37	5.65	54.65	6.64	-5.28	3.49E-04	7.66E-03
cg03110993	MPG;MPG;MPG	16	TSS200;TSS1500;TSS1500	N_Shore	22.42	6.94	27.70	7.14	-5.28	2.54E-05	8.64E-04
cg06550232		2	Intergenic	Island	35.97	3.95	41.25	5.25	-5.28	5.76E-07	2.67E-05
cg12600530	RASA1;RASA1	5	3'UTR;3'UTR	Open sea	53.12	4.91	58.40	6.93	-5.28	1.12E-04	3.08E-03
cg10848724	VPS37B	12	TSS200	Island	21.59	4.44	26.87	6.30	-5.28	2.13E-06	9.10E-05
cg04758026		2	Intergenic	Open sea	52.73	5.50	58.01	5.16	-5.28	4.44E-07	2.08E-05
cg07072391	MLPH;MLPH	2	Body;Body	Open sea	38.93	4.20	44.21	6.65	-5.28	7.39E-05	2.17E-03
cg08802358	FGFR4;FGFR4;FGFR4	5	3'UTR;3'UTR;3'UTR	Open sea	39.29	4.18	44.57	6.43	-5.28	3.70E-05	1.20E-03
cg05035508	PRDM16;PRDM16	1	Body;Body	Island	39.44	4.61	44.71	6.46	-5.28	1.88E-05	6.60E-04
cg17399143		7	Intergenic	Open sea	59.35	4.55	64.62	4.34	-5.28	3.28E-08	1.70E-06
cg14565151	USP44	12	TSS200	S_Shelf	39.72	5.22	45.00	6.91	-5.28	1.87E-04	4.69E-03
cg21406075		15	Intergenic	S_Shore	52.46	7.52	57.74	6.73	-5.28	2.34E-04	5.60E-03
cg02886589	TMEM178;TMEM178	2	TSS1500;TSS200	N_Shore	20.47	4.03	25.74	5.96	-5.27	1.28E-05	4.69E-04
cg14306366		12	Intergenic	S_Shore	74.69	8.04	79.96	5.20	-5.27	1.64E-03	2.45E-02
cg08424184	TFAP2E	1	Body	S_Shore	27.79	5.36	33.06	6.71	-5.27	3.84E-05	1.24E-03
cg12479444		7	Intergenic	Island	13.79	7.07	19.07	7.63	-5.27	7.80E-04	1.42E-02
cg16639692		5	Intergenic	S_Shore	52.52	6.14	57.79	8.45	-5.27	1.90E-03	2.72E-02
cg21554552	RASSF1;RASSF1	3	TSS200;TSS200	Island	28.12	4.51	33.39	5.22	-5.27	1.12E-06	5.00E-05
cg26878836	LRP5	11	Body	S_Shelf	44.81	5.42	50.08	7.10	-5.27	9.44E-04	1.64E-02
cg00447018	LOC100133469	14	TSS200	Island	66.51	7.74	71.78	9.40	-5.27	3.41E-03	4.09E-02
cg07213414	USP31	16	Body	Open sea	48.37	6.42	53.64	6.95	-5.27	2.41E-04	5.74E-03
cg05702774	SUCNR1	3	TSS1500	Open sea	65.92	8.28	71.19	7.26	-5.27	3.26E-03	3.96E-02
cg04482817	PPIH	1	TSS1500	N_Shore	40.28	5.26	45.54	6.93	-5.27	6.05E-04	1.17E-02

cg04132186		3	Intergenic	S_Shore	25.99	5.39	31.26	8.01	-5.27	2.34E-04	5.59E-03
cg27385729	DDX6	11	3'UTR	Open sea	57.21	7.08	62.48	6.62	-5.27	3.23E-03	3.94E-02
cg17908719		3	Intergenic	Open sea	49.59	3.04	54.86	3.75	-5.27	3.04E-09	1.66E-07
cg13294447	DIP2B	12	Body	Open sea	31.14	6.92	36.41	6.18	-5.27	2.32E-03	3.14E-02
cg10192779		20	Intergenic	S_Shelf	55.86	5.38	61.13	5.75	-5.27	2.31E-04	5.54E-03
cg17425818	NRP1;NRP1;NRP1	10	Body;Body;Body	Open sea	44.98	5.51	50.25	4.90	-5.27	7.80E-07	3.55E-05
cg02049217	DLC1;DLC1	8	Body;Body	Open sea	56.21	4.42	61.47	3.63	-5.27	2.64E-07	1.27E-05
cg23821329	VIM	10	TSS1500	N_Shore	32.96	6.03	38.22	9.19	-5.27	1.68E-03	2.49E-02
cg08349093		10	Intergenic	Open sea	44.40	5.42	49.67	8.13	-5.26	1.39E-03	2.19E-02
cg10801331	SDCCAG8;AKT3	1	Body;3'UTR	Open sea	72.70	7.28	77.96	5.02	-5.26	1.86E-03	2.69E-02
cg15822982	LGI1;LGI1	10	5'UTR;1stExon	Open sea	32.08	5.92	37.34	6.61	-5.26	1.24E-04	3.34E-03
cg20434856	MYO10	5	Body	Open sea	47.94	5.35	53.21	6.25	-5.26	2.16E-05	7.48E-04
cg03636880	HLA-DPB1	6	Body	Island	34.54	5.51	39.80	6.30	-5.26	4.62E-04	9.54E-03
cg03667968	APC;APC;APC	5	TSS200;TSS200;5'UTR	Open sea	25.18	8.02	30.44	8.99	-5.26	4.28E-05	1.36E-03
cg06294376	FSCN3	7	TSS200	Open sea	40.11	4.00	45.37	4.00	-5.26	1.04E-06	4.67E-05
cg14937069	H19;MIR675	11	Body;TSS200	Island	64.96	4.04	70.22	5.40	-5.26	1.24E-06	5.48E-05
cg23660197	MICB	6	3'UTR	Open sea	51.74	6.48	57.00	5.96	-5.26	3.15E-04	7.06E-03
cg27219972		16	Intergenic	Open sea	78.92	6.37	84.18	3.40	-5.26	1.40E-04	3.70E-03
cg15014458	LYPD3;LYPD3	19	1stExon;5'UTR	S_Shore	36.68	5.62	41.94	6.35	-5.26	3.15E-05	1.04E-03
cg14109551	C6orf204;C6orf204	6	Body;Body	Open sea	69.10	6.03	74.36	5.24	-5.26	5.51E-05	1.69E-03
cg16456087	OXNAD1	3	Body	Open sea	26.87	5.58	32.13	6.34	-5.26	9.67E-04	1.67E-02
cg25293806	TMEM190	19	Body	Island	26.57	4.72	31.83	6.69	-5.26	2.52E-04	5.94E-03
cg04988514	GRIN2D;GRWD1	19	3'UTR;TSS1500	Island	30.21	4.41	35.46	4.91	-5.26	5.43E-06	2.16E-04
cg10152624	KIAA1530	4	Body	Open sea	86.27	5.60	91.52	3.21	-5.26	1.31E-05	4.81E-04
cg01730258	SNX32	11	Body	Open sea	43.62	4.78	48.88	4.96	-5.26	3.20E-05	1.05E-03
cg24161018	PLXNA4	7	Body	Open sea	74.48	6.28	79.74	6.01	-5.26	3.84E-04	8.25E-03
cg05337607	CRAMP1L	16	Body	N_Shelf	45.49	4.46	50.74	4.20	-5.26	5.15E-07	2.40E-05
cg25649039	PRKCA	17	Body	Open sea	44.70	6.77	49.96	7.03	-5.26	3.49E-03	4.15E-02
cg20016631	FAM114A1	4	5'UTR	S_Shore	34.71	5.45	39.97	6.34	-5.26	5.08E-04	1.02E-02
cg04864441	PTPRN2;PTPRN2;PTPRN2	7	Body;Body;Body	S_Shore	68.06	10.27	73.32	5.22	-5.26	2.68E-03	3.46E-02
cg01571001		3	Intergenic	Island	22.43	5.26	27.69	6.11	-5.26	4.38E-04	9.15E-03
cg08128444		1	Intergenic	S_Shelf	38.62	4.90	43.88	5.18	-5.26	3.70E-05	1.20E-03
cg16365807	BACE2;BACE2;BACE2	21	Body;Body;Body	Open sea	58.37	5.88	63.62	6.72	-5.25	3.84E-04	8.25E-03
cg27663389		19	Intergenic	Island	77.13	7.32	82.39	4.56	-5.25	1.14E-04	3.13E-03
cg26719056		5	Intergenic	N_Shelf	60.90	4.18	66.16	3.87	-5.25	8.52E-08	4.27E-06
cg21339822	MYBPC1;MYBPC1;MYBPC1;MYBPC1;N	12	5'UTR;1stExon;1stExon;5'UTR;1stExon;5'U	Open sea	35.66	5.12	40.92	6.05	-5.25	1.30E-04	3.48E-03
cg15282632	CACNB3	12	Body	S_Shore	66.20	5.50	71.45	5.69	-5.25	4.71E-05	1.48E-03
cg00661855	BPHL;BPHL;BPHL;BPHL	6	Body;Body;Body;Body	N_Shore	54.75	6.25	60.00	6.16	-5.25	1.12E-03	1.86E-02
cg02610600	STX1A;STX1A	7	Body;Body	Island	44.96	4.90	50.21	5.15	-5.25	3.10E-06	1.29E-04
cg09189889	DCT;DCT	13	TSS1500;TSS1500	Open sea	50.22	5.27	55.47	6.06	-5.25	3.20E-05	1.05E-03
cg26735503		20	Intergenic	N_Shelf	40.48	4.54	45.73	4.21	-5.25	2.48E-07	1.20E-05
cg26220203		1	Intergenic	Open sea	63.84	5.02	69.09	5.34	-5.25	7.58E-05	2.22E-03
cg20629183	MPP2	17	TSS1500	S_Shore	32.00	5.50	37.25	6.19	-5.25	2.02E-05	7.05E-04
cg09690145	IPO5	13	TSS200	Open sea	69.91	4.19	75.16	4.30	-5.25	6.66E-07	3.06E-05
cg26087524		6	Intergenic	Open sea	58.34	5.68	63.59	5.61	-5.25	9.84E-05	2.76E-03
cg20029140	NUDT5	10	5'UTR	N_Shelf	61.80	7.23	67.05	7.36	-5.25	3.47E-03	4.14E-02
cg21748207	FAM59B	2	TSS1500	N_Shore	29.36	5.30	34.61	4.87	-5.25	9.56E-06	3.62E-04
cg25786785	TMPRSS6	22	TSS1500	Open sea	62.24	4.88	67.48	4.26	-5.25	3.28E-06	1.36E-04
cg04308938	ZNF217	20	3'UTR	Open sea	71.63	6.99	76.87	8.26	-5.25	4.81E-04	9.83E-03
cg00848392	C6orf27	6	Body	Open sea	34.47	5.69	39.71	6.09	-5.25	1.44E-04	3.78E-03
cg27074041	NR4A2	2	Body	Island	67.89	4.59	73.13	3.82	-5.25	4.63E-07	2.17E-05
cg10606923	CYFIP2;CYFIP2;CYFIP2	5	Body;Body;Body	Open sea	71.26	4.91	76.51	4.95	-5.25	5.39E-07	2.51E-05
cg06621480		22	Intergenic	Island	68.39	7.67	73.64	6.47	-5.25	1.97E-03	2.80E-02

cg04272613	DAAM1	14	5'UTR	Open sea	50.56	5.18	55.80	4.72	-5.25	3.50E-06	1.44E-04
cg00880562	SGEF	3	TSS1500	N_Shore	62.51	6.34	67.76	6.74	-5.25	1.77E-03	2.60E-02
cg12368583	C10orf11	10	Body	Open sea	21.45	5.25	26.70	4.61	-5.24	5.88E-06	2.32E-04
cg05129081	TP63;TP63;TP63	3	TSS1500;TSS1500;TSS1500	Open sea	19.80	4.59	25.04	5.71	-5.24	3.77E-06	1.54E-04
cg20705829	CBR4	4	Body	N_Shelf	69.91	4.37	75.15	5.38	-5.24	8.04E-06	3.09E-04
cg10760339	COL9A3	20	TSS1500	N_Shore	62.04	5.17	67.28	5.94	-5.24	2.04E-04	5.02E-03
cg10474881	MARCKS	6	Body	Island	25.81	4.13	31.05	6.54	-5.24	6.30E-05	1.90E-03
cg13932473	DVL1	1	Body	N_Shore	69.95	7.25	75.19	5.22	-5.24	1.96E-03	2.78E-02
cg17273683	C1orf130	1	5'UTR	Open sea	78.07	5.01	83.32	3.29	-5.24	1.17E-07	5.80E-06
cg13172432	SLC26A9;SLC26A9	1	TSS1500;TSS1500	Open sea	49.76	6.63	54.99	7.06	-5.24	1.39E-03	2.18E-02
cg01413005	BAT2L2	1	3'UTR	Open sea	74.13	3.79	79.36	3.21	-5.24	1.62E-09	8.96E-08
cg18240463	PRDM16;PRDM16	1	Body;Body	Island	10.93	3.05	16.17	8.04	-5.24	1.00E-05	3.78E-04
cg16353350	C10orf55	10	TSS1500	Open sea	62.39	6.00	67.63	5.25	-5.24	3.83E-04	8.24E-03
cg24494102	CNGA1	4	TSS1500	N_Shelf	38.10	6.77	43.34	6.61	-5.24	2.72E-03	3.50E-02
cg08549390		7	Intergenic	Open sea	64.70	6.48	69.94	4.95	-5.24	2.95E-05	9.84E-04
cg13594711	SRCRB4D;SRCRB4D;ZP3	7	1stExon;5'UTR;5'UTR	Open sea	68.53	6.78	73.77	4.96	-5.24	2.92E-04	6.67E-03
cg19268976	LTBP4;LTBP4;LTBP4	19	Body;Body;Body	N_Shore	46.42	5.31	51.65	7.73	-5.23	1.18E-03	1.93E-02
cg08364137		7	Intergenic	S_Shore	29.21	8.95	34.45	7.18	-5.23	4.56E-03	4.99E-02
cg03022926	TC2N;TC2N;TC2N	14	3'UTR;3'UTR;3'UTR	Open sea	66.78	5.36	72.01	5.27	-5.23	6.69E-05	2.00E-03
cg06865629	MIR1-2;MIB1	18	TSS200;Body	Open sea	76.89	7.65	82.13	6.26	-5.23	1.91E-03	2.74E-02
cg19764370	ROBO4	11	TSS200	Open sea	47.70	5.34	52.93	6.11	-5.23	1.28E-05	4.70E-04
cg23095743	PITX3	10	5'UTR	Island	21.23	3.93	26.46	4.64	-5.23	7.26E-08	3.66E-06
cg21535253	MAN2B1	19	Body	Island	41.10	5.69	46.33	5.57	-5.23	1.62E-04	4.16E-03
cg20341089		10	Intergenic	Open sea	61.07	4.60	66.30	4.70	-5.23	6.60E-06	2.57E-04
cg00598235	LAP3	4	Body	S_Shore	65.15	5.88	70.38	5.71	-5.23	6.07E-05	1.84E-03
cg14911395	SEMA3B;SEMA3B	3	Body;Body	Island	24.93	5.58	30.16	8.72	-5.23	2.67E-03	3.46E-02
cg20222519	UBE2E2	3	5'UTR	S_Shore	27.41	5.64	32.64	6.29	-5.23	1.89E-04	4.73E-03
cg26454662	RAPSN;RAPSN	11	TSS200;TSS200	Open sea	56.99	7.10	62.23	9.00	-5.23	2.67E-03	3.46E-02
cg15978565	LPIN1	2	5'UTR	Open sea	30.76	8.40	35.99	8.75	-5.23	2.19E-04	5.32E-03
cg10045354	C11orf93;C11orf92	11	TSS1500;5'UTR	Island	56.73	7.18	61.96	5.54	-5.23	4.34E-04	9.09E-03
cg06846495	NCL	2	Body	N_Shore	32.20	5.67	37.43	5.39	-5.23	4.76E-07	2.23E-05
cg02824888		12	Intergenic	N_Shore	47.69	6.33	52.92	6.32	-5.23	7.81E-05	2.27E-03
cg05264101	FLJ45079	17	TSS200	N_Shelf	39.30	5.97	44.53	8.45	-5.23	2.89E-03	3.65E-02
cg02803914	APBB1IP	10	5'UTR	Island	7.61	3.76	12.84	6.89	-5.23	8.57E-06	3.28E-04
cg05698069	PDLIM2	8	TSS1500	N_Shore	24.87	4.37	30.10	3.93	-5.23	6.15E-08	3.12E-06
cg05917188	CD36;CD36;CD36;CD36;CD36;CD36	7	5'UTR;5'UTR;5'UTR;5'UTR;1stExon;5'UTR	Open sea	43.12	5.55	48.35	7.26	-5.23	9.14E-04	1.60E-02
cg10320997	SYNPO2;SYNPO2;SYNPO2	4	TSS1500;TSS1500;TSS1500	Open sea	64.02	5.36	69.25	4.05	-5.23	1.02E-05	3.82E-04
cg16991168		15	Intergenic	Open sea	65.34	6.43	70.57	7.53	-5.23	6.44E-04	1.23E-02
cg15868425	NUDT14	14	Body	N_Shelf	55.59	6.10	60.82	5.05	-5.23	1.75E-04	4.44E-03
cg19889856	C7orf49;C7orf49;C7orf49	7	TSS1500;TSS1500;TSS1500	S_Shore	25.51	4.97	30.74	7.61	-5.23	1.36E-03	2.14E-02
cg21541159	TCF7L1	2	Body	Open sea	32.09	5.29	37.31	5.44	-5.23	5.17E-05	1.60E-03
cg15093383		17	Intergenic	Open sea	64.14	5.51	69.37	4.92	-5.23	1.35E-05	4.95E-04
cg06882842	ZNF678;ZNF678	1	Body;Body	S_Shelf	62.64	5.17	67.86	5.24	-5.23	4.57E-06	1.84E-04
cg00353643	UNKL	16	Body	N_Shore	32.55	7.37	37.78	6.26	-5.23	2.17E-04	5.27E-03
cg02966404		7	Intergenic	Open sea	46.27	7.24	51.50	7.48	-5.23	3.89E-04	8.34E-03
cg08007925	PRDM16;PRDM16	1	Body;Body	Island	54.71	4.74	59.93	4.98	-5.23	1.61E-05	5.78E-04
cg16033151	SSH2	17	Body	Open sea	39.72	5.68	44.94	5.49	-5.23	3.60E-06	1.48E-04
cg16651126	HOXA4	7	TSS200	Island	22.52	4.48	27.74	5.51	-5.23	2.31E-06	9.79E-05
cg23053961	GLIS3	9	TSS1500	S_Shore	27.51	5.26	32.74	6.87	-5.22	4.31E-04	9.04E-03
cg17559894		7	Intergenic	Open sea	51.32	2.83	56.54	2.34	-5.22	1.05E-12	6.40E-11
cg25470197	CECR6;CECR6;CECR6	22	1stExon;3'UTR;3'UTR	N_Shelf	64.08	9.39	69.31	4.95	-5.22	9.89E-04	1.70E-02
cg26477549	CKAP4	12	TSS1500	S_Shore	26.54	5.04	31.76	6.59	-5.22	6.34E-05	1.91E-03
cg25245082	KIRREL3;KIRREL3	11	TSS1500;TSS1500	N_Shore	15.73	5.10	20.95	7.73	-5.22	9.42E-04	1.64E-02

cg10208609	LRRC8D;LRRC8D	1	5'UTR;5'UTR	Open sea	77.90	4.35	83.12	3.44	-5.22	4.81E-09	2.61E-07
cg15480962	TRIM31	6	Body	S_Shelf	47.91	6.05	53.14	5.53	-5.22	3.36E-04	7.44E-03
cg14228460	PRUNE2	9	Body	Open sea	55.51	5.01	60.73	6.14	-5.22	6.29E-05	1.89E-03
cg08501265		2	Intergenic	Island	39.53	4.16	44.75	4.68	-5.22	9.99E-07	4.48E-05
cg08750510		5	Intergenic	Open sea	69.19	5.02	74.41	4.67	-5.22	7.38E-07	3.38E-05
cg10609174		3	Intergenic	Open sea	67.62	6.19	72.84	8.89	-5.22	3.59E-03	4.24E-02
cg01574741	FZD5	2	3'UTR	N_Shelf	73.56	6.69	78.78	5.95	-5.22	1.40E-04	3.70E-03
cg07407499	RAPSN;RAPSN	11	TSS200;TSS200	Open sea	56.68	6.75	61.90	8.06	-5.22	1.48E-03	2.28E-02
cg20048109	MAGI2	7	Body	Open sea	69.15	5.94	74.37	5.04	-5.22	5.47E-06	2.17E-04
cg25097946	SORBS1;SORBS1;SORBS1;SORBS1;SOF	10	5'UTR;TSS200;TSS200;TSS200;5'UTR;TSS2(	Open sea	50.78	5.70	56.00	5.37	-5.22	2.60E-04	6.09E-03
cg05491587	KCNG2	18	Body	Island	70.34	7.13	75.56	7.71	-5.22	7.02E-04	1.31E-02
cg02254181		1	Intergenic	N_Shore	59.94	6.72	65.16	6.55	-5.22	2.45E-03	3.26E-02
cg17586764	KIAA1324L;KIAA1324L;KIAA1324L	7	Body;Body;Body	Open sea	63.03	7.83	68.25	6.83	-5.22	3.61E-03	4.25E-02
cg21953891	SLIT3	5	Body	Open sea	61.51	6.16	66.73	6.99	-5.22	1.08E-03	1.81E-02
cg15055553	FBXL14	12	Body	Open sea	68.11	5.02	73.33	4.36	-5.22	4.63E-07	2.17E-05
cg04654530	OTX1	2	Body	Island	31.37	4.72	36.58	4.37	-5.22	1.04E-06	4.66E-05
cg11335171	HSD17B8	6	Body	S_Shore	63.99	3.90	69.21	5.03	-5.22	2.01E-08	1.05E-06
cg25613478		10	Intergenic	Open sea	45.25	4.14	50.46	4.62	-5.22	7.43E-08	3.74E-06
cg11873854	LCN6	9	1stExon	S_Shore	57.40	6.32	62.62	5.06	-5.22	1.03E-04	2.87E-03
cg03072003	PPP2R2D;PPP2R2D;PPP2R2D	10	Body;Body;Body	Open sea	71.35	5.63	76.56	4.85	-5.21	2.61E-04	6.11E-03
cg00301239		8	Intergenic	Open sea	16.20	6.44	21.41	6.98	-5.21	1.16E-05	4.30E-04
cg06995068		11	Intergenic	Open sea	10.41	3.04	15.62	6.82	-5.21	1.85E-05	6.52E-04
cg01751047	CDH15	16	Body	Island	54.69	5.78	59.90	6.89	-5.21	5.80E-04	1.14E-02
cg10692614	ITIH3	3	TSS1500	Open sea	40.50	3.66	45.71	4.97	-5.21	5.42E-07	2.52E-05
cg09453076	ARL4C	2	TSS1500	Island	21.38	4.59	26.59	6.70	-5.21	1.09E-06	4.88E-05
cg15617591		3	Intergenic	Open sea	78.31	4.64	83.52	4.67	-5.21	1.76E-07	8.61E-06
cg08468913	SEMA5B	3	Body	Island	54.96	6.01	60.18	6.09	-5.21	1.44E-03	2.23E-02
cg03364874		8	Intergenic	Open sea	40.97	6.79	46.18	6.77	-5.21	2.85E-03	3.61E-02
cg17633431	SEMA5B	3	5'UTR	Open sea	64.44	5.38	69.65	6.31	-5.21	3.71E-04	8.03E-03
cg03409187		6	Intergenic	Island	39.65	8.32	44.86	7.37	-5.21	4.36E-03	4.84E-02
cg04464085		6	Intergenic	Island	10.83	3.63	16.04	7.19	-5.21	1.09E-05	4.06E-04
cg19139092		3	Intergenic	N_Shelf	58.47	7.02	63.68	7.21	-5.21	1.70E-03	2.52E-02
cg18885346	PKHD1;PKHD1	6	TSS1500;TSS1500	Open sea	42.31	5.30	47.52	4.77	-5.21	3.60E-05	1.17E-03
cg04185862		16	Intergenic	Island	58.55	6.08	63.76	4.95	-5.21	1.36E-04	3.60E-03
cg00659878		1	Intergenic	Open sea	73.44	5.88	78.65	4.88	-5.21	7.93E-05	2.30E-03
cg17293195	KRTAP5-7	11	TSS1500	S_Shelf	60.53	6.66	65.74	6.85	-5.21	1.53E-03	2.34E-02
cg10402121		10	Intergenic	N_Shelf	81.42	5.60	86.63	2.27	-5.21	1.24E-07	6.13E-06
cg01489256	TDH	8	Body	N_Shore	76.89	6.98	82.09	6.05	-5.21	3.93E-04	8.40E-03
cg21610439		2	Intergenic	S_Shore	69.48	6.96	74.68	7.44	-5.20	3.13E-03	3.86E-02
cg18014277	APBB1IP	10	3'UTR	S_Shore	62.99	5.16	68.19	6.52	-5.20	8.49E-05	2.44E-03
cg14684854		2	Intergenic	N_Shelf	21.35	5.61	26.56	6.33	-5.20	6.30E-05	1.90E-03
cg11267546	HOXA3	7	TSS1500	N_Shore	28.71	7.13	33.91	7.92	-5.20	2.34E-03	3.15E-02
cg08632388		16	Intergenic	Island	50.73	6.72	55.94	5.03	-5.20	1.59E-04	4.11E-03
cg26176204	AGAP1;AGAP1	2	TSS1500;TSS1500	N_Shore	36.41	5.78	41.61	5.37	-5.20	3.26E-06	1.35E-04
cg23415212	CACNB1	17	3'UTR	Island	13.39	4.99	18.59	7.33	-5.20	5.40E-04	1.07E-02
cg01963464	AGFG1;AGFG1;AGFG1;AGFG1	2	Body;Body;Body;Body	S_Shore	71.77	6.99	76.97	6.75	-5.20	3.28E-04	7.30E-03
cg03892838	LRRFIP1;LRRFIP1;LRRFIP1;LRRFIP1;LRI	2	TSS1500;TSS1500;TSS1500;TSS1500;Body	N_Shore	68.93	7.15	74.13	5.20	-5.20	2.77E-04	6.40E-03
cg06644892	IFT80	3	3'UTR	Open sea	55.24	5.77	60.44	5.71	-5.20	4.52E-04	9.37E-03
cg20484653	NFASC;NFASC;NFASC;NFASC	1	Body;Body;Body;Body	S_Shelf	71.95	4.71	77.15	4.87	-5.20	2.28E-06	9.66E-05
cg12134349		8	Intergenic	S_Shore	64.63	6.30	69.83	6.24	-5.20	1.14E-03	1.89E-02
cg04557423	LOC727677	8	TSS1500	Open sea	77.34	5.48	82.54	5.64	-5.20	1.17E-04	3.19E-03
cg04174538	PC;PC;PC	11	5'UTR;5'UTR;5'UTR	Open sea	47.23	4.93	52.42	4.08	-5.20	1.11E-05	4.15E-04
cg25727671	HOXA7	7	3'UTR	N_Shore	38.61	5.95	43.81	7.20	-5.20	6.70E-04	1.27E-02

cg14463292	FGF12	3	Body	Open sea	31.89	7.13	37.09	6.37	-5.20	5.97E-05	1.81E-03
cg01881549	SH3BP4	2	5'UTR	Open sea	45.55	6.57	50.74	7.65	-5.20	3.48E-03	4.15E-02
cg22488367	C22orf9;C22orf9	22	Body;TSS200	Open sea	50.10	4.70	55.29	4.22	-5.19	1.33E-06	5.87E-05
cg10821127		11	Intergenic	Open sea	62.18	5.24	67.37	5.25	-5.19	2.06E-06	8.81E-05
cg26023709	MRPL43;SEMA4G;MRPL43;MRPL43	10	Body;3'UTR;Body;Body	N_Shore	39.06	7.05	44.26	6.50	-5.19	3.08E-03	3.81E-02
cg22157525	NDST1	5	Body	Open sea	58.77	5.15	63.97	5.44	-5.19	6.36E-05	1.91E-03
cg10322876	CYP2B6	19	TSS1500	Open sea	48.38	4.62	53.57	4.77	-5.19	1.57E-05	5.63E-04
cg10943932	SLC22A18AS;SLC22A18;SLC22A18	11	5'UTR;5'UTR;TSS1500	N_Shore	81.42	8.11	86.61	6.25	-5.19	3.80E-03	4.40E-02
cg17180633		1	Intergenic	S_Shelf	44.69	9.73	49.88	7.85	-5.19	2.63E-03	3.42E-02
cg18743730	CA4	17	TSS1500	N_Shore	33.13	5.06	38.32	7.42	-5.19	5.29E-04	1.06E-02
cg23688719	LEPR;LEPR;LEPR	1	5'UTR;5'UTR;5'UTR	Open sea	28.63	5.63	33.82	6.28	-5.19	5.70E-04	1.12E-02
cg03568064	MAPK8IP1	11	Body	Island	31.88	5.99	37.07	6.51	-5.19	4.04E-05	1.29E-03
cg00901982		2	Intergenic	Open sea	35.31	6.01	40.50	6.08	-5.19	5.34E-05	1.65E-03
cg03277658		6	Intergenic	Open sea	65.56	6.25	70.75	8.12	-5.19	2.86E-03	3.62E-02
cg10832076	SLCO1A2;SLCO1A2	12	3'UTR;3'UTR	Open sea	36.20	4.69	41.39	5.87	-5.19	7.49E-06	2.90E-04
cg23251761	PIK3CD	1	5'UTR	Island	53.78	5.77	58.97	5.16	-5.19	1.57E-05	5.63E-04
cg04364261		2	Intergenic	S_Shore	58.08	5.50	63.27	4.97	-5.19	4.99E-06	2.00E-04
cg03357727	ULK4	3	Body	Open sea	67.86	4.70	73.04	4.66	-5.19	1.98E-06	8.48E-05
cg18583931		15	Intergenic	Open sea	60.15	5.80	65.33	6.93	-5.19	2.58E-04	6.05E-03
cg10089028		13	Intergenic	Open sea	58.55	6.25	63.73	7.07	-5.19	2.67E-03	3.46E-02
cg26604605	SNORD57;NOP56;NOP56;SNORD56	20	TSS1500;Body;Body;TSS200	S_Shelf	38.80	5.35	43.98	7.18	-5.19	1.69E-03	2.50E-02
cg14772639	SAP130;SAP130	2	Body;Body	Open sea	76.43	6.31	81.62	4.11	-5.19	1.70E-04	4.34E-03
cg04666465	GGA2	16	Body	N_Shore	43.69	6.61	48.88	6.20	-5.19	1.66E-04	4.25E-03
cg09099868		10	Intergenic	N_Shore	40.29	6.19	45.48	6.59	-5.19	3.77E-04	8.13E-03
cg19252741	GAL	11	Body	S_Shelf	34.01	5.93	39.19	7.36	-5.19	1.36E-03	2.14E-02
cg24793022		6	Intergenic	Open sea	78.72	6.77	83.91	6.86	-5.19	9.56E-04	1.66E-02
cg25582353	ERBB2;ERBB2	17	Body;Body	N_Shelf	52.53	5.60	57.72	5.43	-5.19	2.17E-04	5.27E-03
cg07012128	DLGAP2	8	TSS1500	Open sea	49.76	5.32	54.95	6.11	-5.19	1.94E-05	6.80E-04
cg09167779	MCC;MCC	5	Body;Body	Open sea	64.80	5.48	69.98	5.33	-5.18	1.79E-05	6.33E-04
cg08293242		13	Intergenic	Island	73.88	7.55	79.06	7.00	-5.18	1.71E-03	2.53E-02
cg05749728	CTBP2;CTBP2	10	5'UTR;5'UTR	Open sea	39.07	5.89	44.26	7.24	-5.18	1.01E-03	1.73E-02
cg15632659	PEX6	6	3'UTR	S_Shelf	60.29	5.59	65.48	3.41	-5.18	1.23E-05	4.52E-04
cg00819142	SOX13	1	5'UTR	Open sea	68.96	4.71	74.14	5.19	-5.18	1.65E-06	7.15E-05
cg05328667	OCA2	15	Body	Island	70.34	5.79	75.52	4.56	-5.18	1.02E-04	2.84E-03
cg08014661		6	Intergenic	Open sea	64.87	4.36	70.05	4.88	-5.18	1.59E-06	6.93E-05
cg22249612	CD63;CD63	12	Body;Body	N_Shore	24.28	5.66	29.47	5.06	-5.18	1.41E-05	5.13E-04
cg09908110	MACROD1	11	Body	Open sea	41.39	8.49	46.57	10.11	-5.18	3.47E-04	7.63E-03
cg00183684	DDAH1;DDAH1	1	1stExon;5'UTR	S_Shore	29.41	5.96	34.59	7.69	-5.18	6.21E-04	1.20E-02
cg10309776	SLC8A3;SLC8A3;SLC8A3;SLC8A3;SLC8/	14	Body;Body;Body;Body;Body;Body	S_Shore	69.57	6.91	74.75	6.50	-5.18	1.49E-03	2.29E-02
cg26839512	SPATA18	4	Body	S_Shore	17.65	6.52	22.83	5.20	-5.18	1.76E-05	6.25E-04
cg24838839		5	Intergenic	Open sea	61.94	6.93	67.12	6.94	-5.18	3.67E-04	7.97E-03
cg26055718	JMJD1C	10	Body	Open sea	57.27	5.17	62.45	6.02	-5.18	3.76E-04	8.11E-03
cg08962038	GRID1	10	Body	Island	18.55	6.12	23.73	7.17	-5.18	1.54E-04	4.00E-03
cg17377797	OR6C1	12	TSS200	Open sea	44.11	4.82	49.29	6.90	-5.18	6.43E-05	1.93E-03
cg07105499	CES7;CES7	16	TSS200;TSS200	Open sea	58.69	6.46	63.87	5.05	-5.18	3.20E-04	7.15E-03
cg08899442		5	Intergenic	Open sea	51.23	4.80	56.41	5.66	-5.18	2.23E-05	7.69E-04
cg27661104	PDE6D	2	Body	N_Shore	41.54	5.43	46.72	6.23	-5.18	1.23E-05	4.55E-04
cg27546977		6	Intergenic	Island	46.69	7.90	51.87	6.23	-5.18	1.99E-03	2.82E-02
cg21899500	GRM2;GRM2	3	TSS1500;TSS1500	Island	52.00	5.45	57.18	4.68	-5.18	2.80E-06	1.17E-04
cg13238479	MAB21L1;MIR548F5;NBEA	13	1stExon;Body;Body	Island	28.44	4.93	33.62	8.17	-5.18	4.88E-04	9.94E-03
cg04507945		1	Intergenic	Open sea	47.30	5.96	52.48	7.12	-5.18	9.98E-04	1.71E-02
cg20247486		1	Intergenic	Island	33.43	4.97	38.61	4.68	-5.18	6.28E-07	2.90E-05
cg11133524		8	Intergenic	Island	40.90	5.82	46.08	5.56	-5.18	1.77E-04	4.48E-03

cg26457569	KANK2;KANK2	19	Body;Body	Island	63.21	4.95	68.39	4.88	-5.18	1.61E-05	5.77E-04
cg22904048		4	Intergenic	Island	44.53	5.45	49.71	5.51	-5.18	4.98E-05	1.55E-03
cg02515553	SV2C	5	Body	S_Shelf	65.03	6.00	70.21	5.90	-5.18	2.31E-04	5.55E-03
cg19501543	ZMYM4	1	Body	Open sea	62.23	4.36	67.41	4.04	-5.18	5.57E-08	2.83E-06
cg07207490	PHLDB1;PHLDB1;PHLDB1	11	Body;Body;Body	Open sea	43.13	5.19	48.31	4.52	-5.18	8.10E-06	3.11E-04
cg17374364	OTP	5	Body	N_Shelf	60.73	6.26	65.90	6.42	-5.18	2.63E-04	6.15E-03
cg27539480	HOXA3;HOXA3;HOXA3	7	3'UTR;3'UTR;3'UTR	N_Shore	48.51	5.36	53.68	6.08	-5.18	1.87E-04	4.69E-03
cg06239923		20	Intergenic	N_Shelf	73.47	7.94	78.65	8.27	-5.18	3.44E-03	4.11E-02
cg12630147	TET1	10	5'UTR	S_Shore	39.89	8.32	45.06	7.62	-5.18	1.87E-04	4.68E-03
cg10868156	RAI1	17	3'UTR	Island	48.49	5.46	53.67	7.81	-5.18	2.75E-03	3.53E-02
cg23932332	DUSP10;DUSP10;DUSP10	1	TSS1500;Body;5'UTR	Open sea	62.02	5.57	67.20	7.60	-5.18	2.55E-03	3.35E-02
cg06206881		12	Intergenic	Open sea	24.47	5.92	29.65	6.01	-5.17	6.48E-05	1.94E-03
cg00083072	SIPA1;SIPA1	11	Body;Body	Island	37.38	5.38	42.56	5.06	-5.17	1.77E-04	4.49E-03
cg12045337	SP5	2	Body	Island	13.63	7.60	18.81	8.02	-5.17	8.83E-04	1.56E-02
cg25701710	STK24;STK24	13	Body;Body	Island	62.36	3.37	67.53	5.13	-5.17	1.57E-06	6.86E-05
cg13521229	JOSD2	19	Body	N_Shore	51.57	4.30	56.74	4.68	-5.17	4.62E-06	1.86E-04
cg06782204		12	Intergenic	Open sea	29.12	5.34	34.29	5.75	-5.17	2.23E-05	7.70E-04
cg03623288	OR2G6	1	TSS1500	Open sea	69.95	5.77	75.12	4.04	-5.17	1.64E-05	5.87E-04
cg25281849	SND1	7	Body	Open sea	53.05	7.83	58.23	6.95	-5.17	1.35E-03	2.14E-02
cg10740880	KBTBD10	2	TSS1500	Open sea	75.28	7.85	80.45	6.88	-5.17	5.15E-04	1.04E-02
cg27514336	NEK11;NEK11	3	3'UTR;3'UTR	Open sea	54.50	6.80	59.67	7.15	-5.17	1.34E-03	2.13E-02
cg27637521	SOCS3	17	5'UTR	Island	27.96	7.54	33.13	7.68	-5.17	2.99E-03	3.73E-02
cg27541454	AGRN	1	Body	Island	45.23	5.49	50.40	5.70	-5.17	2.22E-05	7.68E-04
cg00225623	SPATA13;SPATA13	13	Body;Body	Open sea	37.52	10.18	42.69	11.32	-5.17	1.68E-03	2.50E-02
cg22730830	PRSS21;PRSS21;PRSS21	16	Body;Body;Body	Island	49.18	7.77	54.35	7.02	-5.17	3.80E-03	4.41E-02
cg10155522		11	Intergenic	Open sea	61.31	6.34	66.47	4.52	-5.17	7.85E-05	2.28E-03
cg22094163	KDM3A;KDM3A	2	Body;Body	Open sea	41.51	7.14	46.68	7.12	-5.17	5.29E-04	1.06E-02
cg01115243	VPS4A	16	Body	N_Shore	36.29	5.14	41.46	6.95	-5.17	1.14E-03	1.89E-02
cg23829584		6	Intergenic	Open sea	44.37	4.78	49.54	7.11	-5.17	1.41E-04	3.71E-03
cg09728487		4	Intergenic	S_Shelf	55.25	5.93	60.42	4.91	-5.17	2.83E-04	6.50E-03
cg27032075	ITGBL1	13	Body	Open sea	71.01	5.20	76.18	4.07	-5.17	8.15E-06	3.13E-04
cg15357934	TSPAN31	12	TSS1500	Open sea	19.33	3.96	24.50	4.57	-5.17	2.13E-06	9.08E-05
cg00551398	RARS2;ORC3L;ORC3L	6	Body;TSS1500;TSS1500	N_Shore	62.84	5.26	68.01	5.18	-5.17	1.19E-04	3.22E-03
cg19010166		5	Intergenic	Open sea	77.47	6.71	82.64	4.35	-5.17	6.50E-05	1.95E-03
cg06345736	TYMP;TYMP;TYMP	22	Body;Body;Body	Island	73.55	5.18	78.71	5.25	-5.17	1.15E-04	3.14E-03
cg07686872		7	Intergenic	N_Shore	22.05	5.75	27.22	6.06	-5.17	4.64E-05	1.46E-03
cg15097489		19	Intergenic	S_Shelf	60.90	5.38	66.07	5.53	-5.17	2.42E-05	8.28E-04
cg19563365		11	Intergenic	N_Shelf	29.01	5.95	34.17	6.85	-5.17	1.85E-03	2.67E-02
cg00675878	BCR;BCR	22	TSS1500;TSS1500	N_Shore	40.12	3.82	45.29	4.25	-5.17	5.62E-07	2.61E-05
cg07242540		1	Intergenic	Island	39.89	5.51	45.05	6.82	-5.16	8.44E-04	1.51E-02
cg02405662	DAZAP1;DAZAP1	19	Body;Body	S_Shore	84.77	6.55	89.93	4.16	-5.16	1.31E-04	3.49E-03
cg09440270	CYP26C1	10	TSS1500	Island	37.92	5.49	43.08	6.85	-5.16	3.21E-04	7.18E-03
cg14244577	DDX19B;DDX19B;DDX19B	16	TSS200;TSS200;TSS200	Open sea	24.13	4.59	29.29	3.10	-5.16	1.47E-07	7.24E-06
cg27055759	STEAP1	7	Body	Open sea	74.69	6.74	79.85	6.28	-5.16	7.60E-04	1.39E-02
cg09543819		6	Intergenic	Open sea	71.89	6.22	77.05	4.20	-5.16	2.00E-04	4.94E-03
cg14420613	NBEAL1	2	3'UTR	Open sea	77.14	8.03	82.30	6.11	-5.16	6.95E-04	1.30E-02
cg02928345		1	Intergenic	Open sea	48.22	6.75	53.38	4.69	-5.16	5.11E-05	1.58E-03
cg00965795		2	Intergenic	N_Shore	65.33	7.77	70.49	5.26	-5.16	2.88E-03	3.64E-02
cg18210511	ZC3H3	8	Body	Island	41.90	5.32	47.06	7.86	-5.16	2.53E-03	3.33E-02
cg00655923		7	Intergenic	Open sea	62.28	3.19	67.44	3.34	-5.16	1.72E-09	9.52E-08
cg19502266		11	Intergenic	N_Shelf	51.12	4.28	56.28	4.43	-5.16	5.85E-07	2.71E-05
cg00325113	WFDC2	20	TSS1500	N_Shore	52.06	5.76	57.22	8.99	-5.16	3.75E-03	4.36E-02
cg00145904	CASZ1	1	3'UTR	N_Shore	70.27	4.67	75.43	5.69	-5.16	1.50E-05	5.42E-04

cg03985727	MEOX2	7	TSS1500	Open sea	16.75	4.55	21.91	5.22	-5.16	7.77E-07	3.54E-05
cg16294838	DOCK2	5	Body	Open sea	53.52	5.56	58.68	5.03	-5.16	5.29E-04	1.06E-02
cg23208326	MTHFD2L	4	Body	Open sea	72.33	6.85	77.49	6.74	-5.16	1.88E-04	4.69E-03
cg04777726	PLEKHA4;PLEKHA4;HSD17B14	19	3'UTR;3'UTR;TSS1500	Island	22.38	4.99	27.54	7.79	-5.16	8.04E-04	1.45E-02
cg12727256	C22orf45;ADORA2A;C22orf45	22	Body;3'UTR;Body	Open sea	63.16	4.92	68.32	4.10	-5.16	9.35E-06	3.54E-04
cg13908074	MBD5	2	Body	Open sea	41.17	5.42	46.32	5.36	-5.16	8.77E-05	2.51E-03
cg27326062		3	Intergenic	Open sea	64.72	6.30	69.88	5.27	-5.16	3.15E-04	7.07E-03
cg25342568		4	Intergenic	Island	42.46	5.10	47.61	4.63	-5.16	8.61E-07	3.90E-05
cg08202754		7	Intergenic	Island	48.65	7.71	53.81	6.01	-5.16	1.28E-03	2.06E-02
cg15646123	CDKAL1	6	Body	Open sea	79.92	5.32	85.07	4.79	-5.15	4.35E-06	1.76E-04
cg12538674	HOXA3;HOXA3;HOXA3	7	3'UTR;3'UTR;3'UTR	N_Shore	28.06	5.80	33.21	6.28	-5.15	1.50E-03	2.30E-02
cg00552892	SLC23A3;SLC23A3;SLC23A3	2	TSS1500;TSS1500;TSS1500	Open sea	47.86	6.32	53.01	5.57	-5.15	5.10E-05	1.58E-03
cg10059324	PER3	1	Body	N_Shelf	78.86	3.11	84.01	3.71	-5.15	4.04E-12	2.43E-10
cg00772991		2	Intergenic	Open sea	36.31	7.05	41.46	7.65	-5.15	7.03E-04	1.31E-02
cg11511084	ASPSCR1	17	Body	N_Shore	47.36	3.76	52.51	5.46	-5.15	4.65E-06	1.87E-04
cg20368818		11	Intergenic	S_Shelf	45.90	5.04	51.05	6.40	-5.15	1.40E-04	3.70E-03
cg13532505	NCBP1	9	Body	S_Shelf	56.58	6.85	61.73	7.12	-5.15	1.79E-03	2.61E-02
cg22425474		6	Intergenic	Island	23.66	6.01	28.81	5.83	-5.15	1.40E-05	5.08E-04
cg15999356	YAP1;YAP1	11	Body;Body	Open sea	47.22	6.27	52.37	5.37	-5.15	2.58E-04	6.05E-03
cg12809925	PARK7;PARK7	1	Body;Body	Open sea	21.41	4.86	26.55	5.85	-5.15	6.17E-05	1.86E-03
cg04526087		5	Intergenic	Open sea	45.73	5.80	50.88	5.93	-5.15	3.23E-05	1.06E-03
cg13558371	CRB2	9	Body	Island	21.20	4.75	26.35	6.07	-5.15	2.74E-05	9.21E-04
cg01524618	UTRN	6	Body	Open sea	52.65	6.87	57.79	5.15	-5.15	2.46E-05	8.39E-04
cg10273135	CMYA5	5	Body	Open sea	35.48	5.50	40.63	8.02	-5.15	3.43E-03	4.11E-02
cg16353006	LHX9	1	Body	S_Shore	39.68	5.87	44.83	6.43	-5.15	1.63E-05	5.82E-04
cg02814118	CMIP;CMIP	16	TSS200;Body	Open sea	51.64	7.23	56.79	4.96	-5.15	6.70E-04	1.27E-02
cg26424468		2	Intergenic	S_Shelf	69.06	6.28	74.21	5.29	-5.15	9.96E-05	2.79E-03
cg03574652		3	Intergenic	Open sea	59.71	6.13	64.86	4.80	-5.15	7.14E-05	2.11E-03
cg08101036	HOXA3;HOXA3;HOXA3	7	5'UTR;5'UTR;TSS200	N_Shore	27.04	6.21	32.18	6.34	-5.15	1.37E-03	2.16E-02
cg02046532	DEFB129	20	TSS200	Open sea	77.17	5.09	82.32	3.78	-5.15	3.30E-06	1.36E-04
cg16945633		12	Intergenic	S_Shore	66.96	6.75	72.11	6.57	-5.15	3.45E-04	7.58E-03
cg01266375		4	Intergenic	Open sea	61.62	6.78	66.77	6.11	-5.15	5.43E-05	1.67E-03
cg11959399	C20orf54	20	Body	Island	17.41	5.86	22.55	7.79	-5.15	2.63E-03	3.42E-02
cg20646500	UBR2	6	Body	S_Shelf	54.39	7.15	59.54	6.19	-5.15	1.29E-03	2.07E-02
cg02330683	ITPKA	15	Body	Island	29.01	4.87	34.15	6.54	-5.15	3.33E-05	1.09E-03
cg01878940		16	Intergenic	S_Shelf	51.80	6.50	56.95	5.77	-5.15	8.82E-04	1.56E-02
cg03991807	FLOT1	6	Body	N_Shelf	76.55	4.75	81.70	4.10	-5.15	4.61E-08	2.36E-06
cg17962756		5	Intergenic	Open sea	78.26	9.45	83.40	5.73	-5.15	4.91E-04	9.98E-03
cg03032607	LZTFL1	3	Body	N_Shore	65.90	7.22	71.05	7.36	-5.15	2.10E-03	2.93E-02
cg05734675	DIP2C	10	Body	Open sea	31.95	5.78	37.10	5.58	-5.15	6.52E-04	1.24E-02
cg01438868		3	Intergenic	Open sea	57.77	8.38	62.91	8.38	-5.15	3.69E-03	4.32E-02
cg18634709		4	Intergenic	N_Shelf	58.84	6.41	63.98	6.87	-5.14	4.11E-03	4.65E-02
cg27432085		1	Intergenic	Open sea	38.63	5.15	43.78	4.96	-5.14	3.53E-06	1.45E-04
cg18691800	RBM24;RBM24	6	TSS1500;TSS1500	Island	29.15	4.95	34.29	6.36	-5.14	6.08E-05	1.84E-03
cg19227031	PRRT1	6	Body	Island	50.70	6.29	55.85	6.17	-5.14	1.07E-03	1.80E-02
cg23703687		7	Intergenic	Open sea	50.97	5.42	56.12	4.64	-5.14	5.89E-05	1.79E-03
cg26347632	CDC42EP1	22	3'UTR	S_Shelf	40.80	4.56	45.95	4.29	-5.14	8.69E-06	3.31E-04
cg03693925	MICB	6	Body	Open sea	61.93	6.71	67.07	6.67	-5.14	2.40E-03	3.21E-02
cg24138433	ANO1	11	Body	S_Shore	31.48	4.77	36.63	4.75	-5.14	6.94E-07	3.18E-05
cg00733722		16	Intergenic	Open sea	56.69	6.38	61.83	7.48	-5.14	3.74E-03	4.36E-02
cg00033155	NPHP1;NPHP1;NPHP1;NPHP1	2	Body;Body;Body;Body	Open sea	64.93	5.13	70.07	7.39	-5.14	4.03E-04	8.56E-03
cg14807446	THADA;THADA	2	Body;Body	Open sea	83.56	4.59	88.70	3.26	-5.14	3.90E-09	2.12E-07
cg05337042	SLC7A9;SLC7A9	19	TSS1500;TSS1500	Open sea	58.58	4.72	63.72	5.60	-5.14	2.40E-05	8.22E-04

cg04955912	FARP2	2	TSS1500	N_Shore	19.01	5.18	24.15	6.25	-5.14	8.52E-05	2.45E-03
cg12510201	GFRA1;GFRA1;GFRA1	10	3'UTR;3'UTR;3'UTR	Open sea	79.42	5.36	84.56	4.48	-5.14	1.72E-05	6.11E-04
cg19350133	PARP11	12	Body	Island	50.55	3.83	55.69	3.46	-5.14	7.73E-09	4.14E-07
cg16073236	NRCAM;NRCAM;NRCAM	7	3'UTR;3'UTR;3'UTR	Open sea	75.57	4.97	80.71	5.73	-5.14	5.53E-07	2.57E-05
cg13525067	CYP4F22	19	3'UTR	Island	18.69	3.80	23.83	5.03	-5.14	4.96E-07	2.32E-05
cg16635196	GPR160	3	5'UTR	S_Shore	46.17	6.37	51.30	7.10	-5.14	3.76E-03	4.37E-02
cg00027650	TCF12;TCF12;TCF12;TCF12;TCF12	15	TSS1500;Body;Body;Body;Body	Open sea	62.93	5.55	68.06	4.25	-5.14	3.89E-06	1.59E-04
cg07537562		7	Intergenic	Open sea	31.57	6.89	36.71	8.22	-5.14	2.07E-03	2.89E-02
cg00491180	TMPRSS8	16	Body	Island	14.17	4.46	19.31	5.46	-5.14	5.64E-05	1.73E-03
cg10103036		8	Intergenic	N_Shelf	74.99	7.69	80.12	6.72	-5.14	1.25E-03	2.02E-02
cg06333233	CREG2	2	3'UTR	Open sea	54.56	4.92	59.70	4.37	-5.14	1.39E-06	6.12E-05
cg21392700	MAB21L1;MIR548F5;NBEA	13	1stExon;Body;Body	N_Shore	20.41	4.89	25.55	7.70	-5.14	3.34E-05	1.10E-03
cg24668728		12	Intergenic	N_Shore	78.03	6.44	83.17	5.44	-5.14	1.08E-04	2.98E-03
cg02627403	UNC13D	17	3'UTR	Open sea	58.29	6.36	63.43	5.05	-5.14	6.39E-04	1.22E-02
cg15680359		2	Intergenic	Open sea	54.03	5.56	59.17	5.53	-5.14	4.68E-05	1.47E-03
cg27431671	FCHSD2	11	Body	Open sea	66.60	5.34	71.73	5.87	-5.13	1.45E-05	5.24E-04
cg26453130	NCRNA00171	6	Body	Open sea	49.57	3.79	54.71	4.05	-5.13	5.70E-08	2.90E-06
cg04180823		6	Intergenic	Open sea	41.14	5.55	46.27	7.48	-5.13	1.56E-03	2.37E-02
cg01433654	ARL15	5	Body	N_Shore	27.60	4.49	32.73	4.32	-5.13	7.98E-06	3.07E-04
cg00053728	TTC15	2	Body	S_Shelf	81.93	5.19	87.06	4.78	-5.13	8.69E-06	3.31E-04
cg01055633	CACNA1H;CACNA1H	16	Body;Body	N_Shelf	75.55	4.93	80.68	3.92	-5.13	3.81E-07	1.80E-05
cg05703211	LRRC8D;LRRC8D	1	5'UTR;5'UTR	Open sea	81.63	4.67	86.76	4.17	-5.13	3.43E-08	1.77E-06
cg01847441	ACTN1;ACTN1;ACTN1	14	Body;Body;Body	Open sea	58.03	5.79	63.16	6.32	-5.13	1.06E-04	2.93E-03
cg15002362	LAMA2;LAMA2	6	Body;Body	Open sea	39.76	5.36	44.89	6.77	-5.13	7.59E-04	1.39E-02
cg19526455	PPP1R1B;PPP1R1B	17	Body;Body	S_Shelf	41.16	4.49	46.29	4.90	-5.13	1.49E-06	6.53E-05
cg12728621	EPB41L1;EPB41L1	20	5'UTR;5'UTR	Open sea	34.91	5.49	40.05	5.50	-5.13	1.66E-04	4.25E-03
cg05207620	VSIG2	11	Body	S_Shore	78.64	7.17	83.77	8.03	-5.13	3.15E-03	3.87E-02
cg16385330	LRBA;MAB21L2;MAB21L2	4	Body;1stExon;5'UTR	N_Shore	37.35	7.57	42.48	6.74	-5.13	3.78E-03	4.39E-02
cg06999856		10	Intergenic	Island	72.98	5.54	78.12	5.46	-5.13	5.09E-05	1.58E-03
cg18304704	MAST3	19	Body	S_Shore	18.02	3.94	23.15	5.12	-5.13	8.55E-07	3.87E-05
cg08500128	MAGOHB	12	Body	N_Shelf	76.67	4.26	81.80	4.25	-5.13	1.42E-08	7.49E-07
cg06068373	SEC22C;SEC22C	3	3'UTR;Body	Open sea	34.39	6.63	39.52	7.19	-5.13	4.56E-03	4.99E-02
cg02035242	KLRG2	7	TSS1500	S_Shore	58.96	4.86	64.09	4.28	-5.13	2.84E-06	1.19E-04
cg00444151	SLFN12L	17	Body	Open sea	38.32	5.66	43.45	5.93	-5.13	5.03E-04	1.02E-02
cg12178237	MAB21L1;MIR548F5;NBEA;MAB21L1	13	1stExon;Body;Body;5'UTR	S_Shore	16.28	5.87	21.41	9.26	-5.13	2.21E-03	3.03E-02
cg14323977		9	Intergenic	N_Shore	61.30	5.88	66.43	5.30	-5.13	6.12E-05	1.85E-03
cg17657707	ARFGEF2	20	Body	S_Shelf	41.19	6.34	46.31	6.51	-5.13	3.51E-04	7.69E-03
cg17324149		3	Intergenic	Open sea	50.65	5.20	55.78	4.19	-5.13	8.48E-06	3.24E-04
cg22550549	NFIX	19	Body	Island	40.56	4.46	45.69	4.49	-5.13	2.38E-06	1.01E-04
cg24030037	CLU;CLU;CLU	8	Body;Body;Body	Open sea	87.15	5.48	92.27	3.20	-5.13	1.27E-05	4.67E-04
cg03451670	AGAP1;AGAP1	2	Body;Body	Island	37.15	7.52	42.28	7.15	-5.12	4.83E-04	9.86E-03
cg20033652	NIN;NIN;NIN;NIN	14	5'UTR;5'UTR;TSS1500;5'UTR	Open sea	46.52	6.60	51.65	6.18	-5.12	2.34E-05	8.02E-04
cg24534743	AHDC1	1	5'UTR	Open sea	39.10	2.72	44.23	4.92	-5.12	5.67E-08	2.88E-06
cg18696900	FERMT2;FERMT2;FERMT2	14	TSS1500;TSS1500;TSS1500	S_Shore	35.17	5.70	40.29	5.92	-5.12	2.32E-05	7.96E-04
cg04890851		15	Intergenic	N_Shore	28.61	3.92	33.73	6.49	-5.12	7.11E-06	2.76E-04
cg01242977	C5orf13;C5orf13;C5orf13;C5orf13;C5c	5	Body;Body;Body;Body;Body;Body;Body;Bc	Open sea	79.15	4.80	84.27	3.73	-5.12	1.07E-06	4.80E-05
cg02961808	MIR138-2	16	TSS1500	Open sea	39.50	7.06	44.62	7.11	-5.12	3.81E-03	4.41E-02
cg24458315	CBX1;CBX1	17	3'UTR;3'UTR	Open sea	85.12	3.09	90.24	1.88	-5.12	8.56E-15	5.50E-13
cg02003612	TMEM114	16	TSS200	S_Shelf	59.18	3.83	64.30	3.63	-5.12	3.31E-09	1.81E-07
cg01303420	C3orf55;C3orf55;C3orf55	3	Body;Body;3'UTR	Open sea	49.77	5.67	54.89	7.09	-5.12	4.95E-04	1.00E-02
cg15607311	LOC90586	17	Body	Open sea	55.17	6.08	60.29	7.11	-5.12	4.55E-03	4.98E-02
cg24814707	KIAA1211	4	TSS1500	Open sea	39.13	7.38	44.25	6.85	-5.12	3.14E-03	3.87E-02
cg05029148		8	Intergenic	S_Shore	79.07	7.17	84.19	5.98	-5.12	1.04E-04	2.90E-03

cg17495087		8	Intergenic	Island	41.44	5.75	46.56	4.61	-5.12	2.86E-05	9.55E-04
cg04518342	PDLIM4;PDLIM4	5	TSS1500;TSS1500	Island	32.88	6.30	38.00	7.57	-5.12	1.91E-04	4.76E-03
cg11793499	CFHR4	1	TSS1500	Open sea	47.84	6.94	52.96	5.63	-5.12	4.72E-04	9.69E-03
cg12388707		5	Intergenic	Open sea	80.98	6.11	86.10	4.90	-5.12	6.65E-04	1.26E-02
cg16683073		8	Intergenic	Open sea	58.95	5.36	64.07	5.96	-5.12	4.51E-06	1.82E-04
cg13619277	FMN1	15	Body	Open sea	27.32	5.56	32.44	7.32	-5.12	1.19E-03	1.95E-02
cg15409796	CD4	12	Body	Open sea	86.50	9.32	91.61	5.77	-5.12	3.51E-03	4.18E-02
cg14171460	PRDM16;PRDM16	1	Body;Body	Island	63.66	5.68	68.78	5.78	-5.12	4.01E-04	8.53E-03
cg06697448	LMCD1	3	Body	S_Shore	65.97	7.12	71.09	6.60	-5.12	1.38E-04	3.65E-03
cg16993015		11	Intergenic	N_Shelf	51.88	4.01	56.99	5.28	-5.12	1.11E-06	4.96E-05
cg14284174	C14orf53;C14orf53	14	TSS200;TSS200	Open sea	77.85	4.92	82.97	3.15	-5.12	2.55E-07	1.23E-05
cg15833843	HLF	17	TSS1500	N_Shore	17.23	4.28	22.35	7.43	-5.12	5.97E-05	1.81E-03
cg17134770		1	Intergenic	S_Shelf	68.68	6.58	73.79	6.79	-5.12	1.98E-03	2.81E-02
cg11518839		5	Intergenic	Open sea	31.40	4.51	36.52	6.76	-5.12	1.04E-04	2.89E-03
cg24057615	TFF3	21	3'UTR	Open sea	75.43	5.86	80.55	4.56	-5.12	1.63E-05	5.81E-04
cg02289287		17	Intergenic	Open sea	80.06	6.16	85.18	6.25	-5.12	1.60E-04	4.14E-03
cg03049691	PROCA1	17	TSS200	S_Shore	39.52	4.18	44.63	5.36	-5.12	4.69E-06	1.89E-04
cg08531263	ZNF468;ZNF468	19	TSS1500;TSS1500	S_Shore	26.86	5.07	31.97	5.92	-5.12	2.13E-05	7.38E-04
cg12583572	MAD1L1;MAD1L1;MAD1L1	7	Body;Body;Body	Island	75.50	7.84	80.62	4.31	-5.12	1.37E-03	2.16E-02
cg27212541	DAG1;DAG1	3	TSS200;TSS200	Island	24.59	5.50	29.70	8.60	-5.11	9.91E-04	1.70E-02
cg01766718	MIR1276;KLHL25	15	TSS200;5'UTR	S_Shore	75.26	7.49	80.37	6.96	-5.11	2.14E-04	5.21E-03
cg07706352	COL5A3	19	Body	Island	32.73	6.36	37.84	7.53	-5.11	5.52E-04	1.09E-02
cg03378797	MYO18A;MYO18A	17	Body;Body	Island	81.28	5.54	86.39	4.35	-5.11	1.21E-05	4.48E-04
cg24399349		7	Intergenic	Island	39.52	4.52	44.63	3.72	-5.11	2.21E-06	9.42E-05
cg00442267	RSPH6A	19	1stExon	N_Shore	76.34	5.79	81.45	4.63	-5.11	3.81E-05	1.23E-03
cg22139166	ZNF608	5	Body	Open sea	25.10	3.64	30.21	5.45	-5.11	1.53E-06	6.68E-05
cg19688772	PPP3CA;PPP3CA;PPP3CA	4	Body;Body;Body	Open sea	45.44	5.90	50.55	5.73	-5.11	2.47E-04	5.85E-03
cg05783384		2	Intergenic	Island	33.72	5.60	38.83	5.13	-5.11	8.80E-04	1.56E-02
cg12042952	AFF3;AFF3	2	TSS1500;5'UTR	S_Shelf	64.42	4.44	69.53	4.09	-5.11	1.89E-06	8.13E-05
cg08145949	FGFR3;FGFR3;FGFR3	4	Body;Body;Body	N_Shore	46.73	4.43	51.83	6.26	-5.11	6.10E-05	1.85E-03
cg27379787		2	Intergenic	Open sea	59.85	6.71	64.96	6.49	-5.11	2.42E-03	3.23E-02
cg05259508		12	Intergenic	Island	32.89	6.64	38.00	5.56	-5.11	2.30E-05	7.89E-04
cg26091747	TRIM40	6	Body	Open sea	58.08	5.95	63.19	5.36	-5.11	4.71E-05	1.48E-03
cg03202034	MGC23284;MVD;MGC23284	16	Body;TSS1500;Body	S_Shore	30.01	5.41	35.12	7.63	-5.11	9.46E-05	2.67E-03
cg16909914	ANKRD46	8	5'UTR	Open sea	48.18	6.15	53.29	5.71	-5.11	2.56E-05	8.70E-04
cg07168204		5	Intergenic	N_Shore	40.50	4.62	45.61	5.70	-5.11	5.92E-05	1.80E-03
cg03780701	ADAM32	8	Body	Island	40.58	5.38	45.69	7.46	-5.11	2.10E-04	5.14E-03
cg03133987	ZNF598	16	Body	N_Shore	64.66	4.43	69.76	4.76	-5.11	1.00E-05	3.77E-04
cg18795569	C19orf77;C19orf77	19	5'UTR;1stExon	N_Shore	27.67	3.45	32.78	3.82	-5.11	1.17E-07	5.79E-06
cg00498211	SLC1A4;SLC1A4	2	Body;5'UTR	S_Shore	54.25	6.58	59.35	8.13	-5.11	2.78E-04	6.41E-03
cg19741148		5	Intergenic	Open sea	72.87	6.10	77.98	6.42	-5.11	9.52E-04	1.65E-02
cg08427067		11	Intergenic	Open sea	48.24	6.53	53.35	6.68	-5.11	1.41E-04	3.72E-03
cg05922723	GIGYF1	7	Body	S_Shore	59.48	4.03	64.58	3.67	-5.10	9.16E-09	4.89E-07
cg08451637	RDX	11	Body	Open sea	62.54	5.16	67.64	5.63	-5.10	1.22E-04	3.29E-03
cg14232793	NCOA2	8	5'UTR	Open sea	76.79	5.90	81.89	4.75	-5.10	7.01E-05	2.08E-03
cg18456640		3	Intergenic	Open sea	73.79	4.83	78.90	4.20	-5.10	1.16E-07	5.74E-06
cg23855121	TLR10;TLR10	4	TSS1500;TSS1500	Open sea	61.44	5.47	66.54	5.30	-5.10	1.93E-04	4.80E-03
cg16191009	CPNE9	3	TSS200	N_Shore	57.70	4.86	62.80	4.84	-5.10	4.01E-05	1.28E-03
cg25149251	UFSP1;ACHE;ACHE	7	TSS1500;Body;3'UTR	N_Shore	29.09	5.40	34.19	6.72	-5.10	1.17E-05	4.33E-04
cg15944367	PCGF2	17	5'UTR	Open sea	64.26	5.20	69.36	5.70	-5.10	1.56E-04	4.05E-03
cg08138586	PARD3B;PARD3B;PARD3B	2	Body;Body;Body	S_Shore	45.76	5.80	50.86	5.94	-5.10	5.61E-04	1.11E-02
cg19845127		8	Intergenic	N_Shore	45.23	4.76	50.33	5.31	-5.10	1.52E-05	5.48E-04
cg00130808	LOC145845	15	TSS200	N_Shore	75.04	4.27	80.14	3.42	-5.10	1.70E-08	8.92E-07

cg26896771		15	Intergenic	S_Shore	29.22	4.34	34.32	6.56	-5.10	1.22E-05	4.51E-04
cg00381541		17	Intergenic	Island	58.45	5.45	63.55	5.81	-5.10	1.79E-04	4.52E-03
cg26673396	ST20;C15orf37	15	5'UTR;TSS200	N_Shore	26.59	5.93	31.69	5.99	-5.10	8.01E-04	1.45E-02
cg02191862		10	Intergenic	Open sea	57.69	4.45	62.78	4.87	-5.10	1.96E-06	8.41E-05
cg10017653	THBS3	1	Body	N_Shelf	67.36	4.56	72.45	4.70	-5.10	1.54E-06	6.72E-05
cg15306012	EXOC3L	16	TSS200	Open sea	49.41	4.98	54.51	4.96	-5.10	2.03E-05	7.09E-04
cg01370449	HOXA5	7	TSS200	Island	25.66	5.06	30.76	5.27	-5.10	1.19E-04	3.22E-03
cg25685510	TECR	19	Body	Open sea	23.87	5.31	28.96	4.90	-5.10	3.07E-05	1.02E-03
cg11770323	NDFIP2;NDFIP2	13	Body;Body	Open sea	49.60	5.68	54.70	5.60	-5.10	2.57E-04	6.03E-03
cg16379999	SEL1L	14	Body	Open sea	62.42	4.76	67.51	3.83	-5.10	4.56E-07	2.14E-05
cg01541600	BANP;BANP	16	5'UTR;5'UTR	N_Shelf	54.87	4.91	59.96	5.08	-5.10	2.00E-05	7.00E-04
cg26574610	VPREB3	22	Body	S_Shelf	73.50	3.99	78.59	4.91	-5.09	1.09E-06	4.87E-05
cg07178008	MIB1	18	3'UTR	Open sea	71.44	7.04	76.53	6.31	-5.09	5.55E-04	1.10E-02
cg09447675		12	Intergenic	Open sea	56.82	7.70	61.91	6.11	-5.09	1.06E-03	1.79E-02
cg04860977	FAM155A	13	Body	Open sea	67.10	5.66	72.20	5.84	-5.09	4.97E-05	1.54E-03
cg13353733	PDDC1	11	Body	N_Shelf	53.77	4.47	58.87	5.24	-5.09	4.23E-06	1.72E-04
cg26381457	USP44;USP44	12	3'UTR;3'UTR	Open sea	66.39	6.16	71.48	6.00	-5.09	1.77E-03	2.59E-02
cg06421590		4	Intergenic	S_Shore	15.33	5.36	20.42	7.75	-5.09	3.41E-04	7.52E-03
cg04098547	ABCC12	16	Body	Open sea	60.33	6.49	65.42	7.32	-5.09	9.90E-04	1.70E-02
cg19040195	BCL11A;BCL11A;BCL11A	2	3'UTR;Body;Body	N_Shore	53.28	6.62	58.37	6.12	-5.09	6.11E-04	1.18E-02
cg00979931	ATG16L2	11	Body	Island	32.60	4.32	37.69	4.95	-5.09	1.66E-06	7.19E-05
cg19601636	ARVCF	22	5'UTR	N_Shelf	43.66	4.08	48.75	3.69	-5.09	5.41E-07	2.52E-05
cg27624162		2	Intergenic	Island	30.89	4.53	35.98	6.53	-5.09	4.71E-05	1.48E-03
cg23534142	ATF3	1	5'UTR	Open sea	53.01	4.45	58.10	4.85	-5.09	3.80E-06	1.55E-04
cg20018344	ITPA;ITPA	20	3'UTR;3'UTR	Open sea	66.44	5.43	71.53	5.45	-5.09	1.12E-06	4.99E-05
cg03942042	GABRB3;GABRB3	15	Body;Body	Open sea	51.18	6.22	56.27	7.46	-5.09	1.03E-03	1.75E-02
cg02039815	SERPINE2;SERPINE2;SERPINE2	2	TSS1500;TSS1500;TSS1500	Island	18.27	4.16	23.36	7.74	-5.09	4.50E-04	9.34E-03
cg19614919	TIAM2	6	Body	Open sea	45.09	6.09	50.18	7.33	-5.09	9.06E-04	1.59E-02
cg03421510		12	Intergenic	Open sea	77.51	4.46	82.60	3.92	-5.09	1.14E-06	5.08E-05
cg01178099		3	Intergenic	Open sea	62.38	8.35	67.47	6.43	-5.09	1.56E-03	2.37E-02
cg01359676	RXRB	6	Body	S_Shelf	81.86	3.93	86.95	3.14	-5.09	5.72E-10	3.22E-08
cg06903103		15	Intergenic	S_Shelf	56.09	6.27	61.18	4.23	-5.09	6.02E-05	1.83E-03
cg05160558		4	Intergenic	Open sea	21.36	5.31	26.45	7.47	-5.09	3.65E-04	7.94E-03
cg25232510	NDRG1;NDRG1	8	5'UTR;5'UTR	N_Shelf	84.99	6.23	90.07	4.68	-5.09	1.19E-05	4.42E-04
cg08338216	INPP5F;INPP5F	10	Body;TSS1500	Island	72.02	4.98	77.11	4.30	-5.09	2.25E-05	7.76E-04
cg10372421	CACNA1H;CACNA1H	16	Body;Body	N_Shore	56.52	4.05	61.61	3.74	-5.09	1.15E-06	5.12E-05
cg01815292		5	Intergenic	N_Shore	40.29	6.30	45.37	8.14	-5.09	5.29E-04	1.06E-02
cg04153838		12	Intergenic	S_Shore	61.50	6.09	66.59	4.51	-5.09	4.93E-06	1.97E-04
cg00811961		19	Intergenic	Island	71.54	8.41	76.63	7.95	-5.09	3.94E-03	4.52E-02
cg25977313		2	Intergenic	Open sea	68.88	5.59	73.97	6.51	-5.09	3.31E-04	7.36E-03
cg08071173		7	Intergenic	N_Shelf	62.37	5.26	67.46	5.60	-5.09	4.27E-05	1.35E-03
cg19046189	HSD17B14	19	Body	S_Shore	74.53	5.27	79.61	5.13	-5.09	6.32E-05	1.90E-03
cg13773570	NRN1	6	Body	Island	22.32	3.30	27.41	4.60	-5.09	3.03E-08	1.57E-06
cg08873673	FUT2;FUT2	19	TSS200;TSS200	N_Shore	69.07	5.78	74.16	4.85	-5.09	4.22E-07	1.99E-05
cg05179921	CD69	12	3'UTR	Open sea	60.56	7.04	65.64	6.62	-5.09	3.22E-04	7.20E-03
cg01371233	SEPHS2	16	1stExon	N_Shore	34.96	7.51	40.05	6.94	-5.09	3.43E-03	4.11E-02
cg08442052	DNAH5	5	Body	Open sea	43.34	6.97	48.42	6.65	-5.09	4.85E-04	9.89E-03
cg01978246	LRRC24;LRRC14	8	Body;3'UTR	N_Shore	49.77	5.51	54.86	5.34	-5.08	2.95E-04	6.71E-03
cg12626242		11	Intergenic	Open sea	56.73	5.37	61.81	6.20	-5.08	4.09E-08	2.10E-06
cg25256067	THSD7A	7	Body	Open sea	53.51	5.06	58.59	5.86	-5.08	1.80E-05	6.37E-04
cg06204755		17	Intergenic	Open sea	40.09	5.82	45.18	8.62	-5.08	3.41E-03	4.10E-02
cg27328673	PCDHB15	5	1stExon	N_Shore	50.74	7.54	55.82	7.40	-5.08	2.21E-03	3.03E-02
cg20723129		6	Intergenic	Island	41.51	7.85	46.59	5.76	-5.08	1.92E-03	2.75E-02

cg05208605		11	Intergenic	Island	25.43	5.37	30.51	5.72	-5.08	1.55E-05	5.58E-04
cg20731529	SATB2	2	Body	Open sea	38.75	4.90	43.83	5.78	-5.08	4.13E-06	1.68E-04
cg19273253	MRPL23	11	Body	Island	47.61	5.55	52.69	6.38	-5.08	8.94E-04	1.58E-02
cg04880737	LRRFIP1;LRRFIP1;LRRFIP1;LRRFIP1;LRI	2	Body;Body;Body;Body;Body	Open sea	78.65	5.30	83.73	5.06	-5.08	1.58E-05	5.66E-04
cg13156144	SLC38A10;SLC38A10	17	Body;Body	Open sea	70.12	5.32	75.20	5.54	-5.08	5.66E-05	1.73E-03
cg25886621	PLEKHG7	12	TSS200	Open sea	47.73	4.88	52.81	4.84	-5.08	1.46E-05	5.30E-04
cg12532266	GDF9;UQCRQ	5	TSS1500;TSS1500	N_Shore	39.73	4.70	44.81	7.20	-5.08	5.55E-04	1.10E-02
cg06772580	TP53I13	17	Body	Island	18.65	5.59	23.73	7.57	-5.08	2.59E-03	3.39E-02
cg04573550	SSTR1	14	3'UTR	Island	23.02	6.66	28.10	6.42	-5.08	1.13E-04	3.10E-03
cg22460204	POU6F2;POU6F2	7	Body;Body	Open sea	53.68	9.02	58.76	8.85	-5.08	3.34E-03	4.03E-02
cg25852472	H19	11	Body	Island	48.41	4.24	53.49	5.48	-5.08	1.69E-05	6.01E-04
cg13978902		6	Intergenic	N_Shelf	63.82	8.17	68.90	7.05	-5.08	6.41E-04	1.23E-02
cg21331386		17	Intergenic	N_Shelf	55.29	6.01	60.36	6.13	-5.08	2.82E-04	6.48E-03
cg10925829	OSBPL2;OSBPL2	20	Body;Body	Island	37.20	5.73	42.28	5.78	-5.08	1.46E-03	2.26E-02
cg13049997	SFT2D1	6	Body	Open sea	35.61	5.23	40.68	5.86	-5.08	3.25E-04	7.24E-03
cg00085486		7	Intergenic	N_Shelf	35.63	4.33	40.71	6.24	-5.08	7.58E-06	2.93E-04
cg13335567	NCOA2	8	Body	Open sea	36.07	4.01	41.15	4.44	-5.08	3.66E-07	1.74E-05
cg26322737	ZNF385D	3	Body	Open sea	76.74	6.13	81.82	4.09	-5.08	2.79E-05	9.38E-04
cg11100804	PCDHGA4;PCDHGA6;PCDHGA1;PCDH	5	Body;Body;Body;Body;Body;Body;Body;Bo	S_Shelf	36.61	6.00	41.68	7.32	-5.08	1.82E-03	2.65E-02
cg27619813	FBXL19	16	Body	N_Shore	81.04	5.98	86.11	3.21	-5.07	1.12E-05	4.18E-04
cg15343510	AIF1;AIF1	6	TSS1500;TSS1500	Open sea	73.28	5.83	78.36	3.93	-5.07	9.23E-07	4.17E-05
cg11005826	IGF2AS;INS-IGF2;IGF2;IGF2AS	11	Body;Body;5'UTR;Body	S_Shore	39.80	4.86	44.87	5.49	-5.07	3.61E-05	1.17E-03
cg24409876		1	Intergenic	Open sea	52.75	4.51	57.82	6.35	-5.07	9.50E-05	2.68E-03
cg08500510	PFKFB3	10	Body	Open sea	39.74	6.32	44.82	6.72	-5.07	2.77E-03	3.54E-02
cg03718416	EPS8	12	5'UTR	Open sea	71.66	6.43	76.73	4.94	-5.07	2.97E-04	6.74E-03
cg18543142		3	Intergenic	Open sea	54.24	6.12	59.31	6.51	-5.07	1.46E-03	2.26E-02
cg12852407		13	Intergenic	Open sea	47.27	4.67	52.35	4.42	-5.07	1.50E-06	6.55E-05
cg04150136		16	Intergenic	Island	35.34	4.83	40.41	4.34	-5.07	1.58E-06	6.89E-05
cg18708986		12	Intergenic	Island	37.07	4.27	42.14	5.14	-5.07	4.31E-06	1.75E-04
cg00303378	VSIG8;C1orf204	1	Body;TSS1500	S_Shore	52.51	5.67	57.58	8.40	-5.07	3.18E-03	3.90E-02
cg23590472		10	Intergenic	N_Shelf	64.46	6.02	69.53	4.20	-5.07	1.52E-04	3.96E-03
cg06905367	SPATS2L;SPATS2L;SPATS2L;SPATS2L	2	TSS1500;TSS1500;TSS1500;TSS1500	N_Shore	52.34	6.83	57.41	8.10	-5.07	1.56E-03	2.36E-02
cg15273575	UTRN	6	Body	Open sea	90.11	4.18	95.19	1.48	-5.07	1.77E-12	1.08E-10
cg27100916	USP44	12	TSS200	S_Shelf	62.16	7.85	67.23	7.02	-5.07	3.03E-03	3.77E-02
cg07206827	LINGO4	1	TSS1500	Open sea	31.77	5.23	36.84	6.97	-5.07	1.19E-03	1.95E-02
cg06186450	SHC1;SHC1;SHC1;SHC1	1	5'UTR;5'UTR;1stExon;1stExon	N_Shelf	48.76	5.28	53.83	4.79	-5.07	1.83E-05	6.46E-04
cg16151451	TSPAN4;TSPAN4;TSPAN4;TSPAN4;TSP	11	5'UTR;5'UTR;5'UTR;5'UTR;5'UTR;5'UTR;5'U	Island	49.61	6.54	54.67	8.54	-5.07	6.76E-04	1.28E-02
cg05026403	C10orf10;RASSF4	10	3'UTR;Body	S_Shore	41.62	6.82	46.68	6.25	-5.07	1.52E-03	2.33E-02
cg20587808		3	Intergenic	Island	33.14	4.08	38.21	4.86	-5.07	3.24E-06	1.34E-04
cg15001843		2	Intergenic	Open sea	51.87	5.09	56.93	6.90	-5.07	4.45E-04	9.26E-03
cg04300684		4	Intergenic	S_Shore	62.88	6.94	67.94	7.10	-5.07	7.02E-04	1.31E-02
cg15146515	IZUMO1	19	TSS1500	N_Shelf	14.53	4.24	19.59	5.27	-5.06	1.01E-06	4.52E-05
cg05476287	FGFR4;FGFR4;FGFR4	5	Body;Body;Body	S_Shore	67.95	6.34	73.02	6.49	-5.06	2.83E-03	3.60E-02
cg00344308		3	Intergenic	Open sea	63.64	6.94	68.71	6.94	-5.06	1.16E-03	1.91E-02
cg15409097	GEFT;GEFT	12	TSS1500;TSS200	Island	22.32	4.96	27.39	8.58	-5.06	3.48E-03	4.15E-02
cg16617366	KIAA0427;KIAA0427	18	TSS1500;TSS1500	N_Shore	53.81	4.89	58.87	3.85	-5.06	1.44E-06	6.31E-05
cg04255044	FBXO31;FBXO31	16	Body;Body	N_Shore	32.81	4.58	37.87	5.87	-5.06	5.69E-05	1.74E-03
cg16505204	THRSP	11	TSS1500	Open sea	36.21	6.21	41.27	6.07	-5.06	7.47E-04	1.38E-02
cg14094793	H6PD	1	Body	Open sea	72.91	6.27	77.97	6.92	-5.06	2.60E-03	3.39E-02
cg21776577	XPR1;XPR1	1	Body;Body	Open sea	63.88	5.62	68.94	4.77	-5.06	6.58E-05	1.97E-03
cg06330323	TSC2;TSC2;TSC2	16	Body;Body;Body	N_Shore	19.91	4.75	24.97	8.24	-5.06	2.08E-03	2.90E-02
cg09684233		2	Intergenic	Island	20.24	3.90	25.30	5.02	-5.06	4.49E-07	2.11E-05
cg13672200		12	Intergenic	N_Shore	39.85	6.59	44.91	5.79	-5.06	1.42E-03	2.21E-02

cg11747499	SSH1;SSH1	12	TSS1500;TSS1500	S_Shore	23.00	7.19	28.06	6.78	-5.06	5.48E-05	1.68E-03
cg26115667	TRAF3;TRAF3;TRAF3	14	5'UTR;5'UTR;5'UTR	Open sea	50.93	6.43	55.99	6.38	-5.06	5.98E-05	1.81E-03
cg21478967		18	Intergenic	Island	59.91	7.58	64.97	6.59	-5.06	2.50E-03	3.31E-02
cg19916794	SLC25A34	1	TSS1500	S_Shelf	49.38	4.80	54.44	6.42	-5.06	4.10E-04	8.68E-03
cg27599376	C2orf34	2	Body	Open sea	60.47	5.49	65.53	5.89	-5.06	3.82E-05	1.23E-03
cg19002149	MAP7D1	1	Body	Island	42.13	5.07	47.19	8.06	-5.06	2.46E-03	3.27E-02
cg05612574	RMND1	6	Body	Open sea	58.45	6.20	63.50	5.07	-5.06	9.43E-04	1.64E-02
cg19037007	C20orf54	20	Body	Island	18.80	5.47	23.86	6.91	-5.06	6.46E-04	1.23E-02
cg16153547	LBX2	2	Body	Island	17.09	5.43	22.14	6.31	-5.05	9.86E-05	2.77E-03
cg26132737	ANKRD44	2	Body	Island	58.70	5.25	63.76	3.65	-5.05	3.86E-06	1.58E-04
cg21696012	HMGCL;HMGCL	1	Body;Body	S_Shelf	40.17	4.91	45.22	6.79	-5.05	3.36E-04	7.44E-03
cg24722198		13	Intergenic	Open sea	61.48	4.54	66.53	3.82	-5.05	6.96E-08	3.51E-06
cg14437045		8	Intergenic	Open sea	32.47	4.37	37.53	4.85	-5.05	1.05E-05	3.92E-04
cg00225067	LIMD1	3	Body	Open sea	79.30	6.31	84.35	5.95	-5.05	4.06E-04	8.62E-03
cg16457413	TWF2	3	Body	N_Shelf	77.79	7.88	82.84	4.35	-5.05	9.30E-04	1.62E-02
cg01953658		15	Intergenic	N_Shore	45.90	7.88	50.95	7.69	-5.05	7.40E-04	1.37E-02
cg01143426	MPL	1	Body	N_Shore	60.97	4.47	66.02	4.23	-5.05	1.27E-06	5.62E-05
cg12788108	C17orf64	17	TSS1500	Island	41.19	7.29	46.24	7.04	-5.05	3.29E-04	7.31E-03
cg22165171	PARP12	7	TSS200	S_Shore	52.97	6.35	58.03	7.22	-5.05	3.75E-03	4.37E-02
cg01404163	TOX3;TOX3	16	TSS200;TSS1500	S_Shore	41.14	7.22	46.19	7.46	-5.05	2.67E-03	3.46E-02
cg06635351		1	Intergenic	Open sea	20.65	4.01	25.70	4.81	-5.05	4.46E-06	1.80E-04
cg15420720	STC2	5	Body	N_Shelf	31.28	7.65	36.33	5.58	-5.05	2.78E-04	6.41E-03
cg15706943	SHROOM3	4	Body	Open sea	69.13	5.06	74.18	5.92	-5.05	1.30E-04	3.47E-03
cg14741939	ZIC1	3	TSS200	Island	19.45	6.13	24.50	7.75	-5.05	1.05E-04	2.91E-03
cg05337753	SEPT9;SEPT9;SEPT9;SEPT9;SEPT9	17	5'UTR;5'UTR;Body;Body;Body	S_Shelf	41.43	5.22	46.48	7.87	-5.05	1.34E-03	2.13E-02
cg19575909	LRRC28	15	Body	Open sea	61.55	5.97	66.60	7.26	-5.05	4.10E-04	8.69E-03
cg10453550	PRDM5	4	Body	Open sea	46.17	5.12	51.21	6.26	-5.05	3.01E-04	6.82E-03
cg12151446	HMOX1	22	Body	S_Shelf	53.63	5.64	58.68	6.14	-5.05	3.19E-04	7.14E-03
cg26190233	PANX2;PANX2;PANX2	22	Body;Body;Body	Island	54.40	5.03	59.45	5.45	-5.05	9.80E-05	2.75E-03
cg11127249	IL2RA	10	TSS1500	Open sea	52.02	4.57	57.07	6.10	-5.05	2.26E-05	7.77E-04
cg01919401	MKLN1	7	Body	Open sea	58.05	5.37	63.10	4.57	-5.05	1.97E-05	6.90E-04
cg19144949	GCLC	6	Body	Open sea	54.83	5.52	59.88	5.96	-5.05	3.70E-04	8.01E-03
cg07179033	RNF39;RNF39	6	Body;Body	S_Shore	20.97	5.25	26.02	8.21	-5.04	2.33E-03	3.14E-02
cg22835435		19	Intergenic	Island	39.22	6.90	44.27	6.72	-5.04	1.42E-03	2.22E-02
cg11390690	FAM190B	10	5'UTR	Open sea	45.04	5.98	50.08	6.36	-5.04	1.02E-03	1.74E-02
cg03504005	CCDC43;CCDC43	17	3'UTR;3'UTR	Open sea	50.79	7.54	55.83	7.44	-5.04	4.04E-03	4.60E-02
cg06475902		6	Intergenic	Open sea	31.45	4.98	36.49	6.68	-5.04	2.53E-04	5.96E-03
cg08200770	TBCD	17	Body	Open sea	66.01	7.66	71.05	8.88	-5.04	3.00E-03	3.74E-02
cg13721589	SREBF1;SREBF1	17	Body;Body	Island	44.27	4.22	49.31	7.51	-5.04	6.87E-04	1.29E-02
cg05044764	SALL1;SALL1	16	Body;Body	Open sea	74.55	3.52	79.59	3.29	-5.04	5.26E-10	2.97E-08
cg01915885	NCRNA00171	6	Body	Open sea	55.36	5.60	60.40	4.03	-5.04	1.83E-05	6.45E-04
cg05715492	ARPC1B	7	Body	S_Shore	29.94	4.88	34.98	4.91	-5.04	1.41E-05	5.14E-04
cg25473910		7	Intergenic	Open sea	65.43	4.84	70.47	4.52	-5.04	6.34E-07	2.92E-05
cg25734463		13	Intergenic	Open sea	31.37	4.97	36.41	6.62	-5.04	2.64E-05	8.94E-04
cg27209741		7	Intergenic	Open sea	31.56	5.71	36.60	7.50	-5.04	1.48E-03	2.29E-02
cg04801430	PIP5K1C	19	Body	Island	49.13	4.81	54.17	6.56	-5.04	9.24E-04	1.61E-02
cg01227445	RPL13P5	12	TSS1500	Open sea	68.51	7.68	73.55	5.39	-5.04	2.77E-03	3.54E-02
cg06767766	TET1	10	5'UTR	S_Shore	35.38	7.90	40.41	7.43	-5.04	3.68E-04	7.98E-03
cg03261408		10	Intergenic	Open sea	29.73	5.82	34.76	5.80	-5.04	3.73E-04	8.06E-03
cg04966851		14	Intergenic	Open sea	61.12	5.80	66.15	4.54	-5.04	5.31E-06	2.12E-04
cg04094641	SAG	2	TSS1500	Open sea	62.00	4.83	67.04	4.66	-5.04	9.21E-06	3.49E-04
cg11416650		7	Intergenic	S_Shelf	56.41	5.20	61.44	4.68	-5.04	3.56E-06	1.46E-04
cg11928962	C8orf80	8	TSS1500	Open sea	65.15	3.99	70.19	4.02	-5.03	4.03E-07	1.90E-05

cg09029192	TNRC6C;TNRC6C	17	5'UTR;5'UTR	Open sea	69.29	4.25	74.33	4.35	-5.03	5.18E-06	2.07E-04
cg13520532	LOC619207	10	Body	Island	71.75	5.81	76.78	6.08	-5.03	1.02E-04	2.84E-03
cg23856267		7	Intergenic	Island	41.38	6.98	46.41	7.45	-5.03	1.39E-03	2.19E-02
cg00817731	PRSS50	3	TSS200	Island	25.62	7.67	30.65	7.69	-5.03	4.12E-03	4.66E-02
cg21693907	ATP2B4;ATP2B4	1	TSS1500;TSS1500	N_Shelf	41.15	4.14	46.19	4.28	-5.03	3.08E-07	1.48E-05
cg06895747	GNA12	7	Body	Open sea	67.01	4.98	72.04	4.65	-5.03	1.09E-05	4.09E-04
cg20808136		15	Intergenic	Open sea	86.27	4.01	91.30	2.13	-5.03	6.52E-12	3.90E-10
cg26754273		16	Intergenic	Open sea	68.25	4.92	73.28	3.81	-5.03	4.27E-06	1.73E-04
cg07223266		16	Intergenic	Island	45.05	7.76	50.08	5.39	-5.03	5.33E-04	1.06E-02
cg06883126		5	Intergenic	Open sea	18.68	3.83	23.71	4.17	-5.03	3.64E-07	1.73E-05
cg10318080		2	Intergenic	Open sea	52.06	3.64	57.08	3.68	-5.03	1.13E-07	5.61E-06
cg25953130	ARID5B	10	Body	Open sea	52.50	5.37	57.53	5.44	-5.03	2.51E-05	8.54E-04
cg04091325	SH3GL3;SH3GL3	15	TSS1500;TSS1500	N_Shore	46.35	5.97	51.37	7.24	-5.03	2.36E-03	3.17E-02
cg23232188	EAF2	3	Body	S_Shelf	87.22	7.25	92.25	2.86	-5.03	2.88E-04	6.60E-03
cg20427938	FH	1	Body	Open sea	84.01	5.75	89.04	5.03	-5.03	5.98E-05	1.81E-03
cg00713005		1	Intergenic	N_Shelf	71.16	4.95	76.19	5.47	-5.03	1.06E-04	2.95E-03
cg14042308	RIN1	11	TSS1500	S_Shelf	30.46	3.87	35.49	3.94	-5.03	1.04E-07	5.19E-06
cg22657772	SIK3	11	Body	Open sea	69.27	5.92	74.30	5.33	-5.03	9.33E-04	1.63E-02
cg16343302	ANKRD33B	5	Body	N_Shore	78.97	6.22	83.99	6.26	-5.03	1.48E-03	2.28E-02
cg09349604		4	Intergenic	N_Shore	27.95	7.85	32.98	10.02	-5.03	3.65E-03	4.29E-02
cg06959251	FAM124A	13	Body	Open sea	50.28	3.89	55.31	4.04	-5.03	6.90E-08	3.48E-06
cg25548984	TRAM1L1	4	TSS1500	S_Shore	51.46	6.96	56.48	5.65	-5.03	1.77E-03	2.59E-02
cg11628972		5	Intergenic	Island	27.11	5.68	32.13	6.23	-5.02	4.13E-04	8.74E-03
cg05457563	APC2	19	Body	Island	35.95	6.23	40.97	6.55	-5.02	8.45E-05	2.43E-03
cg19952454	IGF2BP2;IGF2BP2	3	Body;Body	Open sea	59.29	5.02	64.31	4.78	-5.02	2.57E-06	1.08E-04
cg08382926		11	Intergenic	Open sea	24.95	4.05	29.97	5.10	-5.02	1.56E-06	6.80E-05
cg16524049	LMX1A	1	Body	Island	28.45	4.13	33.48	5.80	-5.02	5.53E-06	2.19E-04
cg04881673	SCD5;SCD5	4	Body;3'UTR	Open sea	34.79	4.82	39.81	7.32	-5.02	1.05E-03	1.78E-02
cg09330145		10	Intergenic	Open sea	41.66	5.81	46.68	5.44	-5.02	8.69E-05	2.49E-03
cg14986464	BAT5	6	Body	N_Shore	27.73	4.72	32.75	5.26	-5.02	1.35E-05	4.92E-04
cg12242345		10	Intergenic	Open sea	50.08	4.25	55.11	5.10	-5.02	1.56E-05	5.62E-04
cg08321867		13	Intergenic	Open sea	74.18	6.01	79.20	3.47	-5.02	1.06E-05	3.97E-04
cg03466525		10	Intergenic	Open sea	54.20	6.46	59.22	6.19	-5.02	2.23E-03	3.05E-02
cg24102266	KIAA1522	1	Body	Island	6.86	3.02	11.88	6.16	-5.02	2.89E-07	1.39E-05
cg01454592	CCDC36;CCDC36	3	TSS200;5'UTR	Island	28.69	5.58	33.71	6.34	-5.02	1.53E-04	3.98E-03
cg26672159	PPP1R10	6	5'UTR	Open sea	58.60	5.82	63.62	6.29	-5.02	8.26E-04	1.48E-02
cg10170504	FLJ43663;FLJ43663	7	Body;Body	Open sea	65.19	5.24	70.21	5.02	-5.02	7.99E-05	2.32E-03
cg19975849		12	Intergenic	Open sea	38.50	5.74	43.52	6.50	-5.02	4.20E-04	8.85E-03
cg05451094		17	Intergenic	S_Shelf	53.13	5.32	58.14	7.18	-5.02	1.03E-04	2.88E-03
cg01802898	TMEM82	1	Body	N_Shore	50.36	4.21	55.37	6.98	-5.02	1.81E-04	4.56E-03
cg23468878	ZIC1	3	TSS1500	N_Shore	20.53	7.30	25.54	9.99	-5.02	2.02E-03	2.85E-02
cg00426720	NR4A2	2	5'UTR	N_Shore	71.24	5.27	76.25	4.65	-5.01	2.49E-05	8.48E-04
cg24137322	SHROOM3	4	Body	Open sea	52.06	5.90	57.07	6.62	-5.01	9.90E-04	1.70E-02
cg13906813	HLA-DPA1	6	Body	Open sea	43.28	5.57	48.29	5.70	-5.01	5.94E-06	2.34E-04
cg03096347	TUB	11	Body	Open sea	40.34	4.78	45.35	5.82	-5.01	4.06E-05	1.30E-03
cg03251454		7	Intergenic	Open sea	42.70	4.26	47.71	6.65	-5.01	2.67E-04	6.22E-03
cg10466011		3	Intergenic	Open sea	51.02	6.18	56.03	5.41	-5.01	1.45E-05	5.25E-04
cg13973641	ZNF462;MIR548Q	9	Body;Body	Open sea	68.13	6.68	73.15	6.85	-5.01	1.95E-03	2.78E-02
cg17186388	WIPF3	7	Body	N_Shore	45.11	5.04	50.12	6.09	-5.01	3.80E-05	1.22E-03
cg26558287	TPPP3;TPPP3	16	TSS1500;TSS1500	Island	34.61	4.82	39.63	6.53	-5.01	8.78E-05	2.51E-03
cg04225551	GRHL2	8	Body	Open sea	32.93	4.63	37.94	4.97	-5.01	8.97E-06	3.41E-04
cg03115444	CLIP1;CLIP1	12	Body;Body	Open sea	37.78	5.54	42.79	5.28	-5.01	4.87E-04	9.91E-03
cg23480619	KCNE1;KCNE1;KCNE1;KCNE1;KCNE1	21	5'UTR;5'UTR;1stExon;1stExon;5'UTR	Island	22.23	7.59	27.24	8.85	-5.01	2.64E-03	3.43E-02

cg26739510	C9orf172	9	1stExon	Island	45.81	4.40	50.82	6.31	-5.01	1.01E-04	2.84E-03
cg25031061	STK40	1	Body	S_Shore	38.57	4.24	43.58	5.65	-5.01	5.08E-05	1.57E-03
cg24868122	ARHGAP26;ARHGAP26	5	Body;Body	Open sea	81.68	4.18	86.68	4.23	-5.01	2.03E-07	9.90E-06
cg19368145	CROCC	1	TSS200	Open sea	32.34	5.81	37.35	5.10	-5.01	1.71E-05	6.09E-04
cg14068657	NCRNA00092	9	TSS1500	S_Shore	23.62	4.95	28.63	7.20	-5.01	1.26E-04	3.38E-03
cg14123409	RAP1GAP2;RAP1GAP2	17	Body;Body	Open sea	69.29	7.19	74.30	9.74	-5.01	3.00E-03	3.75E-02
cg21595233		2	Intergenic	Open sea	73.96	6.29	78.97	8.07	-5.01	6.96E-04	1.31E-02
cg05585475		5	Intergenic	N_Shelf	59.00	6.23	64.01	5.69	-5.01	4.72E-04	9.69E-03
cg20566840	GPR81	12	TSS200	Open sea	51.47	5.39	56.48	6.05	-5.01	1.49E-03	2.29E-02
cg14801692	HLA-DPB1	6	Body	Island	33.16	5.35	38.17	5.80	-5.01	4.14E-04	8.75E-03
cg17801295		2	Intergenic	Island	76.78	7.40	81.78	7.75	-5.00	3.75E-03	4.37E-02
cg04642669	WDR46;WDR46	6	Body;Body	N_Shore	64.75	5.05	69.75	4.61	-5.00	2.63E-05	8.92E-04
cg10000052	KDM4B	19	Body	Island	10.35	4.72	15.35	5.97	-5.00	1.07E-05	4.00E-04
cg01035261	CNGB1	16	Body	Island	56.26	4.95	61.26	4.67	-5.00	6.20E-06	2.43E-04
cg18493761		11	Intergenic	Open sea	51.14	4.94	56.14	5.87	-5.00	5.28E-05	1.63E-03
cg24559261	DCPS	11	Body	S_Shelf	66.44	5.62	71.44	4.28	-5.00	5.91E-05	1.80E-03
cg08683615	FAM101B	17	1stExon	S_Shelf	81.57	5.51	86.57	4.50	-5.00	6.35E-06	2.49E-04
cg09107055	C16orf87	16	Body	Open sea	47.82	6.01	52.82	6.13	-5.00	4.05E-04	8.61E-03
cg12417775	PPP2R5A	1	Body	S_Shelf	48.07	6.09	53.08	7.00	-5.00	3.02E-04	6.84E-03
cg03980370		6	Intergenic	S_Shore	57.56	4.02	62.56	3.61	-5.00	2.51E-08	1.31E-06
cg12640394	DYDC1;DYDC2	10	TSS200;5'UTR	Island	12.10	5.04	17.10	6.72	-5.00	2.41E-04	5.73E-03
cg26304147	ANO1	11	TSS1500	N_Shore	15.94	4.29	20.94	6.48	-5.00	6.32E-06	2.47E-04
cg05022688	CLDN11	3	Body	Island	27.35	6.02	32.35	6.99	-5.00	2.77E-04	6.40E-03
cg26776957	PLEKHB1;PLEKHB1;PLEKHB1;PLEKHB1	11	TSS200;TSS200;TSS1500;TSS1500	Open sea	31.23	5.45	36.23	7.39	-5.00	9.66E-04	1.67E-02
cg04513422	C13orf29	13	Body	Open sea	31.21	6.34	36.21	7.77	-5.00	1.78E-04	4.51E-03
cg14511866	MAP4K4;MAP4K4;MAP4K4	2	Body;Body;Body	Open sea	63.44	5.72	68.44	4.68	-5.00	2.63E-05	8.91E-04
cg20924286	CLDN11	3	TSS1500	N_Shore	19.47	4.28	24.47	5.69	-5.00	2.20E-05	7.59E-04
cg26167708	MUC20;MUC20	3	TSS1500;Body	N_Shore	62.09	6.27	67.09	6.11	-5.00	2.65E-04	6.18E-03
cg26698460	ZNF274;ZNF274;ZNF274	19	Body;Body;5'UTR	Island	51.93	6.11	56.93	6.99	-5.00	3.41E-04	7.52E-03
cg02618049	APBB3;APBB3;APBB3;APBB3	5	Body;Body;Body;Body	N_Shelf	69.44	4.29	74.44	3.97	-5.00	1.11E-06	4.96E-05
cg24367761	MED23;MED23	6	Body;3'UTR	Open sea	68.70	5.66	73.69	5.28	-5.00	1.04E-04	2.89E-03
cg11455444	LOC84931	2	TSS200	Open sea	35.00	4.11	40.00	3.83	-5.00	1.87E-06	8.04E-05
cg09162909	RBP7	1	3'UTR	Open sea	54.31	6.06	59.31	5.02	-5.00	3.54E-04	7.75E-03
cg02771744		7	Intergenic	Open sea	64.85	5.48	69.84	5.89	-5.00	3.55E-04	7.77E-03
cg22524980		12	Intergenic	S_Shelf	74.82	4.52	79.81	4.72	-5.00	2.13E-07	1.04E-05
cg07190921	IGFALS;IGFALS;IGFALS	16	TSS1500;TSS1500;TSS1500	S_Shelf	56.96	6.87	61.96	6.54	-5.00	1.07E-03	1.80E-02
cg22305167		6	Intergenic	Island	42.32	7.07	47.31	5.87	-4.99	2.43E-03	3.24E-02
cg07849811		3	Intergenic	Open sea	62.30	5.75	67.29	5.54	-4.99	1.60E-05	5.73E-04
cg25694915	NOSTRIN;NOSTRIN;NOSTRIN;NOSTRIN	2	5'UTR;TSS200;TSS200;TSS200	Open sea	42.55	4.93	47.55	4.92	-4.99	6.48E-07	2.98E-05
cg23627062	LOC285045	2	TSS200	Open sea	45.82	9.17	50.82	5.14	-4.99	2.92E-03	3.67E-02
cg13717446	LOC146880;LOC146880	17	Body;TSS200	Island	19.21	5.20	24.21	5.37	-4.99	4.15E-06	1.69E-04
cg23246095	DPP4	2	TSS1500	S_Shore	51.88	6.15	56.88	5.81	-4.99	1.50E-04	3.91E-03
cg17061004		12	Intergenic	Open sea	63.81	5.87	68.80	5.09	-4.99	4.20E-04	8.86E-03
cg26218269	LRBA;MAB21L2;MAB21L2	4	Body;1stExon;5'UTR	N_Shore	50.51	7.94	55.50	7.61	-4.99	3.23E-03	3.94E-02
cg02735058		6	Intergenic	Open sea	72.56	6.85	77.55	5.68	-4.99	7.48E-04	1.38E-02
cg02865033	TRIM72	16	Body	Island	60.53	4.96	65.52	6.43	-4.99	1.16E-04	3.15E-03
cg07981910	DAB2IP	9	Body	N_Shore	38.99	7.02	43.98	6.26	-4.99	2.41E-03	3.23E-02
cg05819221	FLOT1	6	Body	N_Shelf	69.17	4.95	74.16	5.32	-4.99	1.02E-05	3.85E-04
cg19955284	SLC44A4	6	Body	S_Shelf	62.53	5.54	67.52	5.54	-4.99	3.03E-04	6.85E-03
cg23967848		2	Intergenic	N_Shelf	46.49	6.10	51.48	6.56	-4.99	7.53E-04	1.38E-02
cg13046608	SLC43A3;SLC43A3;SLC43A3	11	TSS1500;TSS1500;TSS1500	S_Shore	30.67	5.29	35.66	4.96	-4.99	1.95E-04	4.84E-03
cg20492868	HIVEP3;HIVEP3	1	5'UTR;5'UTR	Open sea	70.35	4.61	75.34	4.99	-4.99	1.27E-06	5.60E-05
cg22528521	DAZAP1;DAZAP1	19	Body;Body	Island	64.63	6.22	69.62	8.63	-4.99	2.64E-03	3.42E-02

cg11142389	ZIC4;ZIC1	3	TSS1500;TSS1500	N_Shore	32.24	7.27	37.22	8.10	-4.99	3.34E-04	7.40E-03
cg06605704	HMOX2;HMOX2;HMOX2;HMOX2	16	5'UTR;5'UTR;5'UTR;5'UTR	Open sea	51.63	8.43	56.61	5.99	-4.99	1.23E-03	2.00E-02
cg15381259	LOC441204	7	Body	Open sea	74.33	6.45	79.32	5.97	-4.99	3.56E-04	7.78E-03
cg24100168	TRIM78P	11	Body	Open sea	54.34	4.96	59.33	5.18	-4.99	3.62E-05	1.18E-03
cg12046793	AGTRAP;AGTRAP;AGTRAP;AGTRAP;A(	1	Body;Body;Body;Body;Body	S_Shore	63.94	4.00	68.92	3.66	-4.99	2.12E-08	1.11E-06
cg13781408	AATK	17	Body	Island	60.78	6.96	65.77	6.40	-4.99	1.32E-03	2.10E-02
cg26456259		6	Intergenic	Open sea	63.70	6.40	68.68	6.85	-4.99	1.39E-05	5.06E-04
cg18396811	PRDM16;PRDM16	1	Body;Body	S_Shore	43.62	4.40	48.61	6.01	-4.99	5.37E-05	1.65E-03
cg00385620	UHRF1BP1	6	TSS1500	N_Shore	19.24	5.11	24.23	6.91	-4.99	1.62E-04	4.18E-03
cg04672750	SLC13A3;TP53RK	20	TSS1500;3'UTR	Open sea	59.16	7.60	64.14	4.97	-4.99	2.61E-03	3.40E-02
cg12497171	ZNF233	19	TSS1500	N_Shore	61.98	4.78	66.96	5.60	-4.98	6.75E-05	2.01E-03
cg13480898	C19orf66	19	TSS1500	N_Shore	61.91	6.87	66.89	5.43	-4.98	3.01E-03	3.76E-02
cg25019722		6	Intergenic	Island	46.79	6.45	51.77	7.10	-4.98	2.04E-03	2.87E-02
cg23898305	MYO7B	2	Body	N_Shelf	41.32	4.98	46.30	6.04	-4.98	3.61E-04	7.87E-03
cg02511456	MNX1;MNX1	7	Body;Body	Island	25.97	5.40	30.96	7.38	-4.98	1.97E-03	2.80E-02
cg16191307		12	Intergenic	Open sea	52.07	6.63	57.05	8.40	-4.98	2.49E-03	3.29E-02
cg08155625		21	Intergenic	Open sea	24.34	3.63	29.32	4.67	-4.98	3.66E-06	1.50E-04
cg13827179		1	Intergenic	Island	17.51	5.31	22.49	6.80	-4.98	4.50E-04	9.34E-03
cg01664241	ADAMTS3	4	TSS1500	S_Shore	29.47	5.30	34.45	6.35	-4.98	9.52E-05	2.69E-03
cg18473521	HOXC4;HOXC4	12	Body;Body	S_Shore	48.86	5.26	53.84	6.35	-4.98	4.00E-06	1.63E-04
cg26724232	RORA;RORA;RORA;RORA	15	Body;Body;TSS1500;Body	S_Shore	50.63	6.65	55.61	6.98	-4.98	4.06E-03	4.61E-02
cg18518074	EHD1	11	Body	N_Shelf	48.83	5.61	53.81	6.75	-4.98	2.30E-03	3.12E-02
cg14306161		15	Intergenic	Open sea	48.47	3.72	53.45	3.65	-4.98	1.65E-07	8.10E-06
cg17737681	DLX1;DLX1	2	Body;Body	Island	21.59	4.32	26.56	4.89	-4.98	6.55E-07	3.01E-05
cg06712559	AGRN	1	Body	Island	36.03	4.59	41.01	5.52	-4.98	1.79E-06	7.72E-05
cg10316357	LY9;LY9	1	Body;Body	N_Shelf	38.86	6.52	43.83	6.14	-4.98	1.08E-04	2.99E-03
cg19818750		17	Intergenic	N_Shore	59.21	6.03	64.18	7.17	-4.98	1.98E-03	2.81E-02
cg22877504	MREG	2	Body	Open sea	66.11	6.97	71.09	5.16	-4.98	2.54E-04	5.97E-03
cg03179500	PSMA2	7	Body	N_Shore	74.55	5.22	79.52	3.70	-4.98	6.28E-06	2.46E-04
cg17585528	LPP;LPP;LPP	3	5'UTR;5'UTR;5'UTR	Open sea	40.62	6.29	45.60	6.68	-4.98	4.53E-03	4.97E-02
cg11232448		19	Intergenic	N_Shore	64.50	6.06	69.47	5.16	-4.98	2.25E-04	5.43E-03
cg15985533	TYW3;TYW3;TYW3	1	3'UTR;3'UTR;Body	Open sea	48.08	7.07	53.06	7.34	-4.98	1.36E-03	2.15E-02
cg20008140	GNAS;GNAS;GNAS;GNAS	20	3'UTR;3'UTR;TSS1500;Body	N_Shore	39.09	5.96	44.06	6.27	-4.98	2.77E-04	6.40E-03
cg06436504	C3orf26;C3orf26;FILIP1L;FILIP1L	3	TSS200;Body;TSS1500;TSS1500	Open sea	47.62	4.90	52.60	6.27	-4.98	1.69E-04	4.32E-03
cg14986386		5	Intergenic	N_Shelf	64.03	5.68	69.00	4.94	-4.98	2.00E-04	4.95E-03
cg16172549	PCP4L1	1	Body	S_Shore	47.69	5.77	52.66	6.84	-4.97	6.07E-04	1.18E-02
cg02149189		6	Intergenic	Island	36.40	7.01	41.37	5.84	-4.97	4.89E-04	9.94E-03
cg10366878	ANXA3	4	3'UTR	Open sea	74.43	5.46	79.41	4.03	-4.97	1.38E-05	5.04E-04
cg11959156	MYT1	20	Body	Open sea	39.19	5.82	44.16	5.57	-4.97	8.30E-04	1.49E-02
cg18088442		18	Intergenic	Island	19.18	3.57	24.16	4.32	-4.97	1.85E-07	9.04E-06
cg04579507	SLCO1B1	12	Body	Open sea	22.16	4.22	27.13	4.38	-4.97	4.08E-08	2.09E-06
cg09523339	ZNF512B	20	Body	Island	83.12	4.94	88.09	3.61	-4.97	8.36E-06	3.20E-04
cg01218896		15	Intergenic	S_Shore	35.60	4.79	40.57	5.86	-4.97	2.49E-05	8.48E-04
cg22166728		1	Intergenic	Open sea	26.86	5.12	31.83	6.75	-4.97	7.32E-06	2.83E-04
cg17737621		20	Intergenic	S_Shore	43.07	4.58	48.04	5.58	-4.97	2.87E-06	1.20E-04
cg15006881	GDF6	8	Body	S_Shore	33.40	4.02	38.37	6.26	-4.97	4.70E-05	1.47E-03
cg11086415		2	Intergenic	Open sea	65.64	5.16	70.61	6.53	-4.97	3.53E-04	7.73E-03
cg08216899	KCNK6	19	3'UTR	Open sea	70.40	5.92	75.37	4.70	-4.97	8.63E-05	2.48E-03
cg24409539	PAX8;PAX8;PAX8;PAX8;PAX8;LOC440(	2	Body;Body;Body;Body;Body;Body	Island	13.78	5.03	18.75	7.77	-4.97	2.64E-04	6.16E-03
cg06943431	DOK7;DOK7	4	Body;Body	N_Shore	56.61	5.49	61.58	5.02	-4.97	1.06E-04	2.93E-03
cg09466190		4	Intergenic	Open sea	78.18	5.40	83.15	4.33	-4.97	1.73E-05	6.16E-04
cg13789423	GALC;GALC	14	Body;Body	N_Shelf	59.98	5.87	64.95	5.51	-4.97	8.68E-05	2.49E-03
cg25265764	DLGAP2	8	5'UTR	Island	72.61	6.22	77.58	7.39	-4.97	2.03E-04	5.00E-03

cg24864518		4	Intergenic	Open sea	54.99	5.78	59.96	4.09	-4.97	1.62E-04	4.17E-03
cg05225461	C5orf30	5	5'UTR	S_Shore	57.03	7.26	61.99	7.36	-4.97	1.74E-03	2.57E-02
cg10256477	KCNH3	12	Body	S_Shore	62.20	6.17	67.17	7.24	-4.97	3.97E-03	4.54E-02
cg08511651	HIST1H1A	6	1stExon	N_Shelf	20.52	4.50	25.49	6.92	-4.97	7.80E-05	2.27E-03
cg13716421	HSD17B4	5	TSS200	Island	19.27	3.32	24.23	5.28	-4.97	1.60E-07	7.86E-06
cg13289884	CDKN1B	12	3'UTR	S_Shore	27.54	5.38	32.51	7.62	-4.97	3.11E-03	3.84E-02
cg03732014	BACH1;BACH1;BACH1	21	5'UTR;TSS1500;5'UTR	Open sea	36.59	5.16	41.56	5.39	-4.97	4.57E-05	1.44E-03
cg24000814		3	Intergenic	Island	28.79	5.62	33.76	6.46	-4.97	5.95E-04	1.16E-02
cg07600998		1	Intergenic	S_Shelf	53.20	3.83	58.17	4.30	-4.97	5.75E-07	2.66E-05
cg09100338		8	Intergenic	N_Shore	36.46	7.35	41.43	7.26	-4.97	2.48E-03	3.29E-02
cg09449150		11	Intergenic	Open sea	39.39	8.72	44.36	7.83	-4.97	5.15E-04	1.04E-02
cg02867305		12	Intergenic	S_Shelf	70.28	5.85	75.24	5.08	-4.97	6.37E-04	1.22E-02
cg05138702	CTBP2;CTBP2	10	5'UTR;5'UTR	Open sea	46.70	6.52	51.66	7.05	-4.96	4.20E-03	4.71E-02
cg05670995	MMP21	10	Body	Island	67.44	7.97	72.40	7.44	-4.96	2.73E-03	3.51E-02
cg24035898	VSIG8;C1orf204	1	Body;TSS1500	S_Shore	51.81	5.15	56.77	7.08	-4.96	1.69E-03	2.50E-02
cg27171835	GPBP1L1	1	5'UTR	Open sea	43.66	5.97	48.63	5.61	-4.96	1.18E-04	3.22E-03
cg05211790	RAI14;RAI14;RAI14;RAI14;RAI14	5	5'UTR;5'UTR;5'UTR;TSS1500;TSS1500	Open sea	79.08	4.73	84.04	4.04	-4.96	1.19E-05	4.40E-04
cg09232430	ECE1;ECE1;ECE1	1	TSS200;Body;Body	Island	10.03	6.77	15.00	8.91	-4.96	1.41E-04	3.71E-03
cg08031967	NXN	17	Body	S_Shelf	50.11	5.92	55.08	6.34	-4.96	8.01E-05	2.32E-03
cg14571092	RTN4RL1	17	Body	Open sea	46.77	5.00	51.73	4.24	-4.96	2.63E-05	8.90E-04
cg09637757	RIMS2;RIMS2;RIMS2	8	1stExon;5'UTR;Body	Open sea	76.92	6.29	81.88	4.82	-4.96	2.30E-05	7.91E-04
cg19046783	CYP3A4	7	TSS1500	Open sea	61.95	5.62	66.92	5.55	-4.96	1.52E-04	3.95E-03
cg04136185		5	Intergenic	Island	54.53	5.04	59.49	5.09	-4.96	5.36E-05	1.65E-03
cg21315873	WDR69	2	Body	S_Shelf	39.73	5.90	44.69	7.06	-4.96	1.08E-03	1.82E-02
cg00116315	TULP1;TEAD3	6	Body;TSS1500	Island	42.77	5.22	47.74	4.72	-4.96	3.97E-05	1.27E-03
cg23412850	SOCS2	12	Body	Island	10.65	3.04	15.61	4.66	-4.96	8.93E-08	4.47E-06
cg03546977	CMYA5	5	TSS200	Open sea	25.14	5.65	30.10	6.44	-4.96	1.38E-03	2.17E-02
cg14681511	PCDHGA4;PCDHGA11;PCDHGA11;PCD	5	Body;Body;Body;Body;Body;TSS200;Body;	N_Shore	47.71	7.28	52.67	7.73	-4.96	2.99E-03	3.73E-02
cg06832677		2	Intergenic	Open sea	42.71	5.12	47.67	4.99	-4.96	9.27E-05	2.63E-03
cg05670461	VEPH1;VEPH1;PTX3;VEPH1	3	Body;Body;Body;Body	N_Shore	15.01	4.15	19.97	6.70	-4.96	3.35E-04	7.42E-03
cg23077820	PAX3;PAX3;PAX3;PAX3;PAX3;PAX3	2	Body;Body;Body;Body;Body;Body	N_Shore	54.74	5.20	59.70	5.16	-4.96	4.01E-06	1.63E-04
cg18241189	TAS2R13;PRR4;PRH1	12	TSS1500;Body;5'UTR	Open sea	65.73	7.90	70.69	8.10	-4.96	3.95E-03	4.53E-02
cg13508175		10	Intergenic	Island	44.91	7.59	49.87	7.86	-4.96	2.12E-03	2.95E-02
cg25262528	NT5E	6	TSS1500	N_Shore	34.50	5.93	39.46	6.38	-4.96	5.01E-06	2.01E-04
cg22254463	EXPH5;EXPH5	11	5'UTR;Body	Open sea	30.18	4.69	35.14	6.96	-4.96	9.69E-04	1.67E-02
cg09236126		17	Intergenic	Open sea	63.54	5.21	68.50	4.83	-4.96	4.09E-05	1.31E-03
cg07048348	LRRC15;LRRC15	3	TSS1500;TSS1500	Open sea	41.30	8.14	46.26	5.27	-4.96	1.35E-03	2.14E-02
cg25276892	TNRC6B;TNRC6B;TNRC6B	22	Body;TSS1500;TSS1500	Open sea	44.34	5.95	49.30	5.23	-4.96	1.05E-05	3.93E-04
cg21133153		6	Intergenic	Island	39.02	6.13	43.97	5.41	-4.96	1.99E-04	4.93E-03
cg14898151	ZFYVE26	14	Body	Open sea	70.79	5.24	75.75	4.30	-4.96	5.06E-05	1.57E-03
cg20802392	CTSK	1	5'UTR	Open sea	62.06	5.48	67.02	5.32	-4.96	8.19E-05	2.37E-03
cg07039560	SLC25A2	5	TSS200	Island	39.97	5.69	44.93	6.71	-4.96	8.16E-04	1.47E-02
cg00270764	IL27	16	Body	S_Shore	62.21	4.35	67.17	4.80	-4.96	1.49E-05	5.38E-04
cg09154959	ARMC2	6	Body	Open sea	49.14	6.47	54.09	6.01	-4.96	1.37E-03	2.16E-02
cg19600372		3	Intergenic	Open sea	50.67	4.87	55.63	4.72	-4.96	4.78E-05	1.49E-03
cg04992852		19	Intergenic	S_Shelf	57.13	6.44	62.09	7.36	-4.96	2.18E-03	3.00E-02
cg05839570	ALS2CL	3	5'UTR	N_Shelf	61.59	5.32	66.54	4.90	-4.95	2.95E-04	6.71E-03
cg06471402	CDH15	16	Body	Island	22.83	4.81	27.79	6.69	-4.95	4.90E-04	9.96E-03
cg01043616	ACCN2;ACCN2	12	Body;Body	Island	41.77	5.26	46.72	5.92	-4.95	2.40E-04	5.72E-03
cg01961153		1	Intergenic	N_Shore	46.06	5.05	51.01	5.82	-4.95	6.14E-05	1.86E-03
cg25184724		7	Intergenic	Open sea	47.32	5.36	52.27	6.17	-4.95	3.88E-04	8.32E-03
cg17568809	ARHGAP26;ARHGAP26	5	Body;Body	S_Shelf	44.86	6.67	49.81	5.94	-4.95	1.16E-03	1.91E-02
cg17796960	LOC619207	10	Body	Island	58.42	6.65	63.37	6.72	-4.95	1.26E-03	2.04E-02

cg06892898	ELMO1;ELMO1;ELMO1	7	Body;TSS200;5'UTR	Open sea	44.02	5.16	48.97	4.10	-4.95	3.25E-05	1.07E-03
cg13286698	TDH	8	Body	N_Shore	49.86	7.92	54.81	8.08	-4.95	3.25E-03	3.96E-02
cg06583549	IRF2BP1	19	1stExon	Island	62.94	5.93	67.89	6.70	-4.95	2.48E-03	3.28E-02
cg17584009	PDE4D;PDE4D;PDE4D	5	Body;Body;Body	Open sea	67.31	6.63	72.26	6.07	-4.95	7.80E-06	3.00E-04
cg03760191		10	Intergenic	N_Shore	68.54	6.06	73.49	7.73	-4.95	6.56E-04	1.25E-02
cg16126286	SDCCAG8	1	Body	N_Shore	41.53	4.03	46.48	5.27	-4.95	5.21E-07	2.43E-05
cg01758106	SNX29	16	Body	Open sea	73.37	4.20	78.32	2.88	-4.95	7.74E-08	3.89E-06
cg00766368	TRAPPC9;TRAPPC9	8	Body;Body	Island	77.85	4.42	82.80	3.97	-4.95	3.96E-07	1.87E-05
cg16342550	SLC22A17;SLC22A17	14	Body;Body	Island	72.47	5.62	77.41	5.81	-4.95	2.88E-04	6.59E-03
cg26954464	ATP6V0D1	16	Body	Open sea	83.92	6.64	88.87	4.92	-4.95	3.65E-04	7.94E-03
cg06349851	PRICKLE2	3	5'UTR	Open sea	33.68	5.00	38.63	6.38	-4.95	5.45E-04	1.08E-02
cg00911776	FLJ43663;FLJ43663	7	Body;Body	Open sea	73.50	5.62	78.45	4.33	-4.95	7.67E-06	2.96E-04
cg03896269	ACBD6	1	Body	Open sea	27.89	5.26	32.84	6.23	-4.95	9.92E-04	1.70E-02
cg14453935		18	Intergenic	Island	36.30	6.37	41.25	5.70	-4.95	8.45E-04	1.51E-02
cg27175943		16	Intergenic	Open sea	66.80	5.44	71.74	5.04	-4.95	2.12E-04	5.17E-03
cg11729679	BCL2L11;BCL2L11;BCL2L11	2	TSS1500;TSS1500;TSS1500	Island	22.03	4.57	26.97	6.79	-4.95	1.38E-04	3.64E-03
cg23777302		11	Intergenic	Island	40.88	3.92	45.83	4.27	-4.95	9.26E-07	4.18E-05
cg11829072	TRMT12	8	TSS200	Open sea	5.15	2.13	10.10	5.83	-4.95	7.02E-07	3.22E-05
cg10783149		14	Intergenic	Open sea	46.94	5.99	51.88	5.26	-4.95	1.46E-03	2.26E-02
cg13412545		5	Intergenic	Island	33.59	4.59	38.53	5.92	-4.94	1.89E-04	4.71E-03
cg18439861	PCDHGA4;PCDHGA11;PCDHGA11;PCD	5	Body;Body;Body;Body;Body;Body;Body;Bo	Island	57.93	6.38	62.87	5.50	-4.94	6.92E-04	1.30E-02
cg00496026	LZTS2	10	Body	Island	48.41	5.30	53.35	6.14	-4.94	1.19E-03	1.95E-02
cg20495370	SLC7A7;SLC7A7;SLC7A7	14	Body;Body;Body	Open sea	56.16	6.35	61.11	8.02	-4.94	1.66E-03	2.47E-02
cg23530543	HEPN1	11	TSS1500	N_Shelf	68.10	6.44	73.04	5.74	-4.94	1.42E-03	2.21E-02
cg14916079	NANOS1	10	TSS1500	Open sea	30.94	5.47	35.88	7.58	-4.94	3.88E-03	4.47E-02
cg21151899	CENPM;CENPM;CENPM	22	TSS1500;Body;Body	Open sea	56.14	4.30	61.09	4.42	-4.94	6.10E-06	2.39E-04
cg07894352	ZNF233	19	TSS200	Island	42.16	6.89	47.11	5.89	-4.94	5.58E-06	2.21E-04
cg05786314	ACOT7	1	Body	N_Shore	51.65	4.27	56.59	5.02	-4.94	1.97E-05	6.91E-04
cg26767593	DCTN6	8	Body	S_Shore	44.59	6.44	49.53	6.46	-4.94	3.38E-03	4.07E-02
cg02306798	TRIO	5	Body	Open sea	46.60	8.46	51.54	8.40	-4.94	2.86E-03	3.62E-02
cg01102232	DNAJC13	3	TSS1500	N_Shore	42.89	5.56	47.83	6.12	-4.94	1.70E-03	2.51E-02
cg18625630	SORBS2;SORBS2;SORBS2;SORBS2;SOF	4	Body;5'UTR;Body;Body;Body;5'UTR	Open sea	67.28	7.17	72.22	8.38	-4.94	2.66E-03	3.45E-02
cg14431850	ABCA1	9	5'UTR	Open sea	37.32	4.73	42.26	6.20	-4.94	2.29E-04	5.52E-03
cg03408354	SLC7A5	16	Body	Open sea	42.83	5.92	47.77	6.94	-4.94	2.80E-03	3.57E-02
cg13872831	RASSF1;RASSF1	3	1stExon;1stExon	Island	32.06	4.12	37.00	4.41	-4.94	1.82E-06	7.86E-05
cg06618356		16	Intergenic	S_Shore	56.92	6.59	61.86	5.94	-4.94	7.76E-04	1.42E-02
cg26868097	MBTPS1	16	Body	Open sea	49.67	5.35	54.60	6.32	-4.94	3.56E-05	1.16E-03
cg11404296		12	Intergenic	Open sea	66.92	7.08	71.86	6.42	-4.94	2.65E-03	3.44E-02
cg13578272	UBE2R2	9	Body	S_Shore	60.77	6.21	65.71	6.36	-4.94	1.19E-04	3.24E-03
cg25471478		19	Intergenic	Island	8.16	2.39	13.10	3.85	-4.94	7.17E-12	4.28E-10
cg19083626		7	Intergenic	N_Shore	52.45	6.13	57.38	7.60	-4.94	2.25E-03	3.07E-02
cg04534077	TMIE;TMIE	3	1stExon;5'UTR	Island	24.54	4.66	29.47	5.59	-4.94	1.02E-06	4.59E-05
cg18831134	ABR	17	5'UTR	S_Shore	43.80	5.60	48.73	7.15	-4.94	1.59E-03	2.40E-02
cg05895034	PRSS22	16	TSS1500	S_Shore	19.56	6.57	24.50	7.43	-4.94	1.49E-03	2.30E-02
cg14785609		1	Intergenic	Island	25.87	4.64	30.81	6.26	-4.94	1.37E-04	3.62E-03
cg01098320		8	Intergenic	N_Shore	26.89	5.68	31.82	5.46	-4.94	2.09E-04	5.11E-03
cg24730564	LOC100133469	14	TSS200	Island	63.95	5.96	68.88	7.62	-4.94	1.12E-03	1.86E-02
cg05531796	CCDC46	17	TSS1500	Island	39.08	5.16	44.01	4.71	-4.94	1.51E-06	6.58E-05
cg22268137	PDX1	13	Body	N_Shore	13.31	4.81	18.24	7.71	-4.94	1.97E-03	2.80E-02
cg01267797	SERINC5	5	Body	Open sea	35.28	6.56	40.22	4.40	-4.93	3.75E-05	1.21E-03
cg13807386	QSOX2	9	3'UTR	S_Shore	53.56	5.13	58.49	6.83	-4.93	7.31E-04	1.35E-02
cg15239888	TIMM44	19	Body	N_Shore	70.89	4.01	75.82	3.22	-4.93	2.08E-07	1.01E-05
cg24843609	BGLAP	1	TSS1500	N_Shelf	64.58	4.01	69.52	3.93	-4.93	3.41E-08	1.76E-06

cg16127349	PBX1	1	Body	Open sea	84.28	5.53	89.21	4.92	-4.93	1.73E-07	8.46E-06
cg17749454	SLC16A5	17	Body	N_Shelf	32.16	5.15	37.10	5.71	-4.93	2.95E-04	6.72E-03
cg23974473	PCDHB15	5	1stExon	N_Shore	50.95	7.56	55.88	8.23	-4.93	4.50E-03	4.95E-02
cg02123734		10	Intergenic	Open sea	75.21	5.23	80.14	4.85	-4.93	4.16E-05	1.33E-03
cg22610388	FLNB;FLNB;FLNB;FLNB	3	Body;Body;Body;Body	Open sea	44.39	5.88	49.33	5.61	-4.93	6.47E-04	1.24E-02
cg14694191	AIF1;AIF1;AIF1	6	TSS1500;TSS1500;TSS1500	Open sea	61.46	4.77	66.39	4.17	-4.93	9.74E-07	4.38E-05
cg10120657		17	Intergenic	Island	11.63	3.33	16.56	6.42	-4.93	9.31E-06	3.53E-04
cg02215611	NRN1L	16	Body	S_Shore	29.53	4.79	34.46	8.23	-4.93	3.72E-03	4.35E-02
cg14184524	TMC1	9	Body	Open sea	48.59	5.92	53.52	7.15	-4.93	1.59E-03	2.41E-02
cg14847626	NCOR2;NCOR2	12	Body;Body	N_Shore	54.05	6.01	58.98	6.31	-4.93	1.90E-04	4.74E-03
cg17209507	MYH9	22	Body	Island	86.80	4.76	91.73	2.54	-4.93	3.68E-08	1.90E-06
cg02804091	PCDHB17	5	Body	N_Shore	60.44	6.51	65.37	6.48	-4.93	5.23E-04	1.05E-02
cg07198887		6	Intergenic	Open sea	63.89	4.91	68.81	5.94	-4.93	1.18E-04	3.22E-03
cg11139127	A2M	12	Body	Open sea	58.53	5.58	63.45	5.93	-4.93	3.05E-04	6.89E-03
cg00088844		12	Intergenic	Open sea	35.62	4.37	40.55	5.35	-4.93	6.54E-06	2.55E-04
cg17815933	FOXJ2	12	5'UTR	Open sea	63.62	6.68	68.55	6.64	-4.93	7.12E-05	2.10E-03
cg17355329		5	Intergenic	S_Shore	33.80	3.81	38.73	4.31	-4.93	7.50E-07	3.42E-05
cg14009504	PCNX	14	Body	Open sea	65.19	6.33	70.12	5.77	-4.93	2.13E-04	5.18E-03
cg14717122	CYP2C8	10	3'UTR	Open sea	60.85	8.96	65.77	7.52	-4.93	1.37E-03	2.16E-02
cg20348765	CCDC85A	2	Body	S_Shelf	56.68	5.50	61.60	6.84	-4.93	8.52E-04	1.52E-02
cg08983011	BCL9L	11	Body	Open sea	72.21	4.23	77.13	3.34	-4.93	3.43E-07	1.63E-05
cg23465650	EBF1	5	Body	N_Shore	45.43	4.53	50.36	7.14	-4.93	2.16E-04	5.24E-03
cg04606087		11	Intergenic	N_Shelf	67.23	6.43	72.15	5.26	-4.92	4.82E-05	1.51E-03
cg17818435	SOAT2	12	TSS200	Open sea	65.46	6.05	70.39	5.26	-4.92	6.28E-05	1.89E-03
cg08241465	CNGB1	16	Body	Open sea	22.97	5.83	27.89	7.05	-4.92	1.68E-03	2.49E-02
cg27020966		6	Intergenic	Open sea	29.78	5.19	34.70	5.16	-4.92	3.28E-05	1.08E-03
cg26120971		8	Intergenic	Open sea	34.76	4.68	39.68	6.71	-4.92	8.97E-04	1.58E-02
cg00944181		14	Intergenic	S_Shelf	37.21	6.13	42.14	5.22	-4.92	6.93E-04	1.30E-02
cg06262098	TNRC18	7	Body	Island	61.13	4.55	66.05	3.49	-4.92	1.15E-06	5.10E-05
cg27398401	PPAP2B;PPAP2B	1	Body;Body	Open sea	33.33	6.10	38.26	6.91	-4.92	4.48E-03	4.93E-02
cg09375488	ASB4;ASB4	7	TSS1500;TSS1500	Open sea	68.58	7.08	73.50	6.99	-4.92	5.73E-04	1.12E-02
cg01383486	GINS2	16	Body	N_Shore	82.85	5.23	87.77	3.51	-4.92	3.93E-06	1.60E-04
cg09674468	SLC35F3	1	Body	Open sea	28.33	5.77	33.26	6.56	-4.92	1.54E-03	2.35E-02
cg19882784	C7orf49;C7orf49;C7orf49	7	TSS1500;TSS1500;TSS1500	S_Shore	39.54	5.56	44.46	7.24	-4.92	4.51E-04	9.36E-03
cg05914935	ATF7IP2	16	TSS1500	Open sea	70.58	5.77	75.50	5.77	-4.92	1.55E-04	4.02E-03
cg14825633		11	Intergenic	N_Shelf	29.11	4.79	34.04	6.52	-4.92	1.26E-04	3.39E-03
cg25500196		17	Intergenic	Island	55.67	4.00	60.59	4.29	-4.92	1.74E-06	7.53E-05
cg08597067	ELOVL5	6	Body	Open sea	66.85	5.55	71.77	4.80	-4.92	5.08E-05	1.58E-03
cg26924825	SLC12A4;SLC12A4;SLC12A4;LCAT;SLC1	16	3'UTR;3'UTR;3'UTR;1stExon;3'UTR;3'UTR	Open sea	33.79	4.69	38.71	5.77	-4.92	2.18E-04	5.29E-03
cg04245250	FAM166B;FAM166B;FAM166B;FAM1(	9	5'UTR;5'UTR;1stExon;1stExon	Open sea	23.18	5.05	28.10	5.66	-4.92	2.93E-05	9.77E-04
cg06271696		7	Intergenic	Open sea	77.33	6.69	82.25	7.16	-4.92	6.41E-04	1.23E-02
cg19251500	CD79A;CD79A	19	Body;Body	N_Shelf	77.57	4.43	82.49	3.89	-4.92	6.53E-07	3.00E-05
cg15672653		11	Intergenic	S_Shelf	25.91	4.33	30.83	6.09	-4.92	8.62E-05	2.47E-03
cg01861377	PPAP2C;PPAP2C;PPAP2C	19	Body;Body;Body	Open sea	48.29	4.65	53.21	5.89	-4.92	4.20E-05	1.34E-03
cg05867884	SLC6A18	5	3'UTR	Island	25.04	4.73	29.96	6.35	-4.92	1.83E-04	4.60E-03
cg17596731		5	Intergenic	Island	67.71	4.75	72.63	5.45	-4.92	8.56E-05	2.46E-03
cg04899500	KCNJ14;KCNJ14	19	Body;Body	Island	69.60	4.07	74.52	3.60	-4.92	2.95E-07	1.41E-05
cg02319818	PAK4;PAK4;PAK4;PAK4;PAK4	19	5'UTR;5'UTR;5'UTR;5'UTR;5'UTR	S_Shore	34.89	4.78	39.80	6.06	-4.92	1.52E-04	3.96E-03
cg05976623	ROS1	6	Body	Open sea	78.98	3.95	83.90	2.97	-4.92	1.00E-08	5.33E-07
cg18155407	TDRD12	19	TSS200	Island	82.95	7.86	87.87	6.68	-4.91	9.38E-04	1.63E-02
cg23108232		14	Intergenic	Open sea	36.38	5.05	41.29	5.69	-4.91	1.85E-05	6.52E-04
cg18900591	SALL4	20	TSS200	S_Shore	50.31	6.11	55.22	5.80	-4.91	1.58E-03	2.40E-02
cg17210546		19	Intergenic	Island	43.02	5.19	47.93	6.21	-4.91	1.44E-03	2.24E-02

cg17973661	MAP2K5;MAP2K5	15	Body;Body	Open sea	60.46	4.46	65.38	3.55	-4.91	1.48E-06	6.49E-05
cg03200571	SNX25	4	3'UTR	Open sea	41.46	6.31	46.37	5.65	-4.91	1.64E-03	2.45E-02
cg24957628	ODZ4	11	Body	Open sea	51.97	4.04	56.89	3.63	-4.91	2.32E-07	1.13E-05
cg19244312	FNDC3B;FNDC3B	3	Body;Body	Open sea	68.49	6.08	73.40	5.56	-4.91	1.28E-03	2.06E-02
cg24153754	NDST1	5	Body	Open sea	50.69	4.14	55.61	5.21	-4.91	3.77E-05	1.22E-03
cg18108716	RCN3	19	Body	Island	34.89	5.26	39.80	5.42	-4.91	1.39E-05	5.06E-04
cg01048203	RNASE9;RNASE9;RNASE9;RNASE9;RN/	14	5'UTR;5'UTR;5'UTR;5'UTR;5'UTR;5'UTR;5'U	Open sea	76.76	4.86	81.67	4.76	-4.91	4.80E-05	1.50E-03
cg25020820	TTBK1	6	Body	Island	59.92	4.57	64.83	4.90	-4.91	3.53E-05	1.15E-03
cg20027296	TLX3	5	Body	Island	27.86	4.39	32.77	4.99	-4.91	2.05E-05	7.14E-04
cg12852210		5	Intergenic	S_Shore	66.25	5.31	71.16	5.68	-4.91	2.92E-04	6.66E-03
cg19688761	PTPRE	10	5'UTR	Open sea	69.43	6.69	74.34	6.23	-4.91	1.57E-04	4.07E-03
cg10504436	DERL3;DERL3;DERL3	22	Body;Body;Body	Island	50.23	4.93	55.14	5.35	-4.91	4.31E-06	1.75E-04
cg00708486	GCH1;GCH1;GCH1;GCH1	14	Body;Body;Body;Body	Open sea	78.67	4.49	83.58	4.27	-4.91	3.05E-06	1.27E-04
cg21472639	FURIN	15	Body	N_Shelf	59.07	4.95	63.97	5.98	-4.91	7.37E-04	1.36E-02
cg10299976		10	Intergenic	Island	26.43	5.97	31.33	7.62	-4.91	4.17E-03	4.69E-02
cg18606375	PITX1	5	Body	N_Shore	28.78	5.27	33.69	5.26	-4.91	3.34E-04	7.41E-03
cg12179288	ANKH	5	Body	Open sea	45.44	3.68	50.35	4.83	-4.91	2.35E-06	9.95E-05
cg20215476	GYG1	3	TSS1500	N_Shore	47.24	8.11	52.15	7.82	-4.91	3.47E-03	4.14E-02
cg08591859	LRP5L;LRP5L	22	Body;1stExon	N_Shore	70.71	6.23	75.61	5.10	-4.91	3.80E-03	4.41E-02
cg27543607	CDK10;CDK10;CDK10;CDK10;CDK10;C	16	3'UTR;Body;3'UTR;Body;Body;3'UTR	S_Shore	47.07	3.79	51.98	4.34	-4.91	2.51E-06	1.06E-04
cg26604359	PGLYRP2	19	Body	S_Shelf	56.70	5.28	61.61	4.89	-4.91	1.70E-04	4.34E-03
cg02037307	PITX1	5	3'UTR	Island	35.69	5.39	40.59	4.59	-4.91	5.76E-06	2.28E-04
cg15144631	IQCE;IQCE	7	Body;Body	Open sea	31.73	3.49	36.64	5.70	-4.91	1.76E-05	6.25E-04
cg23717725		18	Intergenic	N_Shelf	53.59	5.68	58.49	4.79	-4.91	2.39E-04	5.70E-03
cg27351978	DLGAP2	8	3'UTR	S_Shore	46.02	5.61	50.93	7.28	-4.91	7.20E-04	1.34E-02
cg04678713	BDH1;BDH1;BDH1	3	3'UTR;3'UTR;3'UTR	Open sea	20.28	4.64	25.19	7.59	-4.91	1.73E-04	4.40E-03
cg14787880	BMPR2	2	Body	S_Shore	72.01	6.28	76.92	5.31	-4.91	1.81E-04	4.57E-03
cg24536827		2	Intergenic	Open sea	61.74	4.65	66.64	3.97	-4.91	6.49E-08	3.28E-06
cg17752112	RAB3D	19	TSS200	S_Shore	17.45	5.14	22.35	6.74	-4.90	1.14E-05	4.25E-04
cg05529152		12	Intergenic	N_Shelf	48.31	4.56	53.21	6.44	-4.90	7.14E-05	2.11E-03
cg02102533	CCDC102A	16	Body	Island	40.02	4.46	44.93	6.31	-4.90	2.77E-04	6.40E-03
cg06520300	MYCN;MYCNOS	2	5'UTR;Body	Island	17.52	6.42	22.42	7.34	-4.90	1.38E-04	3.65E-03
cg24875029	POU3F3	2	TSS1500	Island	32.89	4.37	37.80	4.54	-4.90	1.97E-06	8.47E-05
cg27314761	SLC39A10;SLC39A10	2	5'UTR;5'UTR	S_Shelf	69.38	5.02	74.28	4.62	-4.90	1.85E-04	4.65E-03
cg03190891	SORBS1;SORBS1;SORBS1;SORBS1;SOF	10	5'UTR;TSS1500;TSS1500;TSS1500;TSS1500	Open sea	71.70	5.57	76.60	6.17	-4.90	8.47E-09	4.53E-07
cg07948194	SLC30A10	1	Body	N_Shore	42.30	5.05	47.21	5.67	-4.90	1.74E-04	4.43E-03
cg08202399	MKL2	16	Body	Open sea	52.19	3.89	57.09	3.47	-4.90	2.36E-07	1.14E-05
cg23193613		8	Intergenic	Island	26.89	5.48	31.79	6.01	-4.90	4.01E-04	8.53E-03
cg12648201	KRTCAP3;KRTCAP3	2	TSS200;TSS200	Island	31.23	5.38	36.13	7.07	-4.90	1.82E-03	2.65E-02
cg10868668		4	Intergenic	Open sea	53.96	5.04	58.86	5.03	-4.90	1.86E-05	6.54E-04
cg26198700		5	Intergenic	Open sea	31.67	6.25	36.57	5.26	-4.90	3.29E-05	1.08E-03
cg13617248	DSCR3	21	TSS1500	Island	18.26	3.34	23.16	5.77	-4.90	3.12E-06	1.30E-04
cg20098710	YJEFN3	19	Body	Island	69.84	5.94	74.74	4.41	-4.90	2.92E-04	6.66E-03
cg16137498		10	Intergenic	N_Shore	50.37	5.27	55.27	4.68	-4.90	6.07E-05	1.84E-03
cg24836281	LTBP4	19	Body	N_Shelf	54.26	5.28	59.16	4.74	-4.90	5.82E-06	2.30E-04
cg13564428	PTPN3;PTPN3	9	5'UTR;5'UTR	N_Shelf	45.94	6.85	50.84	6.01	-4.90	1.65E-03	2.47E-02
cg08988420	PDLIM3;PDLIM3	4	Body;Body	Open sea	47.70	6.75	52.59	6.58	-4.90	2.03E-03	2.86E-02
cg23819092	MEX3A	1	Body	Island	67.49	6.00	72.39	5.70	-4.90	3.34E-04	7.40E-03
cg21006256		8	Intergenic	Open sea	25.66	4.42	30.56	5.15	-4.90	3.76E-06	1.54E-04
cg14468090		3	Intergenic	N_Shore	53.38	5.15	58.28	7.13	-4.90	9.75E-04	1.68E-02
cg26179590	SGCE;SGCE;PEG10;SGCE;PEG10	7	Body;Body;TSS1500;Body;TSS1500	N_Shore	13.90	3.91	18.80	4.35	-4.90	1.07E-08	5.68E-07
cg17819635	TCTEX1D1	1	TSS1500	N_Shore	37.51	5.87	42.40	5.45	-4.90	1.40E-05	5.09E-04
cg00691671		1	Intergenic	S_Shelf	41.29	4.94	46.19	5.23	-4.90	1.10E-05	4.10E-04

cg10433547	CCDC154		16	Body	N_Shore	65.13	6.64	70.03	5.13	-4.90	6.13E-04	1.19E-02
cg11532433			2	Intergenic	Open sea	65.19	5.42	70.09	4.30	-4.90	1.12E-05	4.17E-04
cg14976342	KIAA1026;KIAA1026;KIAA1026;KIAA1(		1	Body;TSS1500;Body;Body	S_Shelf	54.13	6.03	59.03	5.49	-4.90	5.39E-04	1.07E-02
cg13382567	SRRM4		12	Body	Open sea	39.31	6.56	44.20	6.58	-4.89	6.48E-05	1.94E-03
cg15874390	ATG2B		14	3'UTR	Open sea	20.05	4.11	24.95	5.72	-4.89	4.96E-06	1.99E-04
cg22550815	KANK2;KANK2		19	3'UTR;3'UTR	Open sea	79.37	7.14	84.26	6.21	-4.89	1.47E-03	2.27E-02
cg04948956			12	Intergenic	S_Shelf	40.57	4.27	45.47	4.13	-4.89	3.96E-06	1.61E-04
cg23155911		03-Mar	5	3'UTR	Open sea	39.10	5.48	43.99	7.37	-4.89	1.03E-03	1.75E-02
cg08394248			16	Intergenic	Open sea	49.76	5.58	54.65	5.59	-4.89	5.28E-05	1.63E-03
cg02400474	ZSCAN22		19	5'UTR	S_Shelf	85.98	6.02	90.87	2.21	-4.89	1.76E-06	7.60E-05
cg03349950			10	Intergenic	S_Shore	85.23	7.73	90.12	3.77	-4.89	8.07E-04	1.46E-02
cg11387088	CUEDC1		17	Body	Island	72.23	7.36	77.13	8.14	-4.89	4.50E-03	4.95E-02
cg20443451	MCCD1		6	Body	Open sea	52.40	5.94	57.29	5.99	-4.89	9.95E-04	1.70E-02
cg16824477			17	Intergenic	Island	67.03	5.72	71.92	5.31	-4.89	8.66E-05	2.48E-03
cg17270887			12	Intergenic	Open sea	71.35	6.36	76.24	5.09	-4.89	6.44E-04	1.23E-02
cg00809021			3	Intergenic	Open sea	72.38	4.51	77.27	4.33	-4.89	6.74E-08	3.41E-06
cg08944940	MACF1		1	Body	Open sea	62.69	5.89	67.58	5.15	-4.89	1.18E-04	3.21E-03
cg23389808			17	Intergenic	N_Shore	18.79	4.31	23.68	5.65	-4.89	2.99E-05	9.96E-04
cg14504259			9	Intergenic	Island	26.17	3.96	31.06	4.07	-4.89	7.16E-07	3.28E-05
cg18417245	PCDHB11		5	TSS200	N_Shore	32.34	6.52	37.23	6.90	-4.89	4.61E-04	9.52E-03
cg09409956	LY6G6D		6	Body	Open sea	63.37	6.30	68.25	5.71	-4.89	7.71E-04	1.41E-02
cg24973483	CCDC57		17	Body	Open sea	57.93	5.05	62.82	5.32	-4.89	1.41E-04	3.71E-03
cg26381352	B3GALT4		6	TSS200	Island	26.05	7.55	30.93	6.37	-4.89	7.45E-04	1.37E-02
cg15171911	TCF4;TCF4		18	Body;Body	Open sea	50.42	6.88	55.31	4.86	-4.89	3.63E-05	1.18E-03
cg25349034	WWTR1;WWTR1;WWTR1		3	Body;Body;Body	Open sea	32.63	5.00	37.52	6.84	-4.89	8.83E-04	1.56E-02
cg24391279			8	Intergenic	N_Shelf	29.91	5.53	34.80	6.35	-4.89	8.18E-04	1.47E-02
cg19261864			6	Intergenic	Open sea	78.08	2.40	82.96	1.93	-4.89	1.93E-15	1.26E-13
cg00860321	MAD1L1;MAD1L1;MAD1L1		7	Body;Body;Body	N_Shore	78.83	6.57	83.72	4.57	-4.88	3.40E-04	7.50E-03
cg14081251	ST6GALNAC4;ST6GALNAC4		9	5'UTR;Body	S_Shore	61.87	5.82	66.75	6.64	-4.88	2.66E-03	3.45E-02
cg21230162			4	Intergenic	N_Shore	49.39	7.01	54.27	7.16	-4.88	1.18E-03	1.94E-02
cg11451506	AFF3;AFF3		2	Body;Body	Island	77.42	8.13	82.30	7.09	-4.88	2.32E-03	3.14E-02
cg19147129			2	Intergenic	S_Shore	25.39	4.60	30.27	5.23	-4.88	6.90E-06	2.68E-04
cg07481360	DLG1;DLG1		3	Body;Body	Open sea	57.39	6.00	62.27	6.19	-4.88	2.56E-04	6.02E-03
cg04369341	TOX2;TOX2;TOX2		20	5'UTR;Body;5'UTR	Island	20.83	4.21	25.71	4.76	-4.88	3.49E-06	1.43E-04
cg24940583	KIAA0319L		1	5'UTR	N_Shore	49.06	5.17	53.94	4.71	-4.88	2.23E-06	9.50E-05
cg05088356	MAST4;MAST4;MAST4		5	TSS1500;Body;Body	Open sea	66.42	6.41	71.30	5.82	-4.88	7.37E-04	1.36E-02
cg21022868	SLAMF9;SLAMF9;SLAMF9		1	Body;Body;Body	Open sea	63.14	5.51	68.03	4.93	-4.88	1.37E-05	4.99E-04
cg16246488	HPCAL1;HPCAL1		2	5'UTR;5'UTR	S_Shore	43.84	4.92	48.72	5.37	-4.88	4.10E-05	1.31E-03
cg07884222			15	Intergenic	S_Shelf	46.09	5.96	50.98	6.17	-4.88	1.37E-04	3.63E-03
cg09652746	E2F4;EXOC3L		16	TSS1500;TSS1500	Open sea	55.98	4.75	60.86	4.37	-4.88	3.59E-07	1.70E-05
cg21079805	CDGAP		3	Body	Open sea	66.28	4.21	71.16	6.02	-4.88	1.84E-05	6.48E-04
cg09682796	ZMYND10		3	TSS200	Island	14.16	5.48	19.04	6.50	-4.88	5.11E-04	1.03E-02
cg17147638	AGAP1;AGAP1		2	Body;Body	S_Shelf	50.72	4.64	55.60	3.49	-4.88	7.15E-06	2.77E-04
cg15542924	QKI;QKI;QKI;QKI		6	Body;Body;Body;Body	Open sea	66.99	5.11	71.87	5.14	-4.88	5.26E-04	1.05E-02
cg17737409	GALNT2		1	Body	Open sea	59.68	5.72	64.56	5.55	-4.88	7.14E-04	1.33E-02
cg12558347	SLC22A5		5	Body	Open sea	44.68	4.35	49.56	5.75	-4.88	8.50E-05	2.44E-03
cg00474657	SPNS1;SPNS1;SPNS1;SPNS1;SPNS1		16	Body;Body;Body;Body;Body	S_Shelf	47.35	3.65	52.23	3.94	-4.88	4.60E-07	2.16E-05
cg15199987			6	Intergenic	Open sea	51.23	5.93	56.11	5.06	-4.88	7.32E-04	1.36E-02
cg05068636	SPG11;SPG11		15	Body;Body	Open sea	46.54	5.59	51.42	5.35	-4.88	6.47E-04	1.24E-02
cg22874255	ONECUT3		19	Body	S_Shelf	33.50	5.68	38.37	5.34	-4.88	6.90E-04	1.30E-02
cg22143274			5	Intergenic	Open sea	85.74	4.52	90.62	3.10	-4.88	2.42E-09	1.33E-07
cg20984972	NFIC;NFIC		19	Body;Body	S_Shore	34.28	4.36	39.16	6.50	-4.88	2.92E-04	6.67E-03
cg02520281	PCDHB17		5	Body	N_Shore	42.94	5.66	47.82	7.77	-4.88	8.88E-04	1.57E-02

cg09232805	GALNTL6	4	Body	Open sea	45.20	5.83	50.08	6.13	-4.88	3.83E-04	8.24E-03
cg08721802		4	Intergenic	N_Shore	19.97	6.18	24.85	7.11	-4.88	3.62E-03	4.26E-02
cg25264948	C6orf27	6	Body	Open sea	52.73	5.43	57.61	4.31	-4.88	6.35E-05	1.91E-03
cg06794253		1	Intergenic	Island	11.01	4.29	15.89	6.18	-4.88	2.25E-04	5.42E-03
cg04333867	PCK2;PCK2	14	Body;Body	S_Shelf	80.47	9.06	85.34	3.98	-4.88	1.71E-03	2.53E-02
cg04662056	CASKIN2;CASKIN2	17	Body;TSS200	Open sea	64.80	6.80	69.68	5.62	-4.88	9.59E-04	1.66E-02
cg25500028		3	Intergenic	Open sea	59.80	6.17	64.68	6.20	-4.88	3.52E-04	7.71E-03
cg06451900	LOC145845	15	Body	N_Shore	60.85	5.84	65.72	7.69	-4.88	1.03E-03	1.75E-02
cg24095120	EFNA5	5	Body	Open sea	72.98	5.30	77.86	4.95	-4.87	6.90E-05	2.05E-03
cg15644671	CYP26C1	10	TSS1500	N_Shore	75.71	6.93	80.58	6.65	-4.87	2.12E-03	2.95E-02
cg11898431		6	Intergenic	Open sea	62.33	4.51	67.20	5.68	-4.87	2.83E-05	9.48E-04
cg05912338		11	Intergenic	Open sea	63.32	6.22	68.19	5.02	-4.87	3.05E-05	1.01E-03
cg05158606	ABI2	2	Body	S_Shore	44.93	7.16	49.80	8.50	-4.87	1.17E-03	1.92E-02
cg04367107	GRM5;GRM5	11	3'UTR;3'UTR	N_Shelf	51.46	5.74	56.34	7.02	-4.87	1.77E-03	2.60E-02
cg23161318	C10orf50	10	TSS200	Open sea	80.26	9.76	85.13	6.44	-4.87	3.68E-03	4.31E-02
cg08928753	TIE1	1	Body	Island	54.80	5.98	59.67	6.64	-4.87	9.96E-04	1.71E-02
cg16256492	ZBTB7B	1	3'UTR	Island	18.14	5.20	23.01	6.65	-4.87	2.86E-04	6.56E-03
cg17245996		1	Intergenic	Open sea	80.12	4.82	84.99	3.83	-4.87	1.67E-06	7.23E-05
cg24527183	CDC42SE2;CDC42SE2	5	3'UTR;3'UTR	Open sea	67.03	5.72	71.90	5.50	-4.87	1.90E-04	4.75E-03
cg14057560		9	Intergenic	Open sea	47.77	4.32	52.64	4.51	-4.87	8.16E-07	3.71E-05
cg23798668		11	Intergenic	N_Shelf	36.39	4.75	41.26	7.63	-4.87	1.63E-03	2.44E-02
cg13789938	WDR11	10	Body	S_Shelf	72.21	6.19	77.08	4.33	-4.87	5.77E-05	1.76E-03
cg00229754		15	Intergenic	Island	20.39	4.65	25.26	5.15	-4.87	4.26E-05	1.35E-03
cg05479174	SFRS8	12	Body	Open sea	75.08	5.64	79.95	5.80	-4.87	2.85E-03	3.61E-02
cg08355345		7	Intergenic	Open sea	70.67	3.34	75.54	4.17	-4.87	6.34E-09	3.41E-07
cg11712835		1	Intergenic	Island	25.85	4.92	30.72	5.52	-4.87	5.22E-07	2.43E-05
cg06270545	LOC390595	15	3'UTR	Open sea	55.24	5.78	60.11	6.64	-4.87	1.65E-03	2.46E-02
cg13208333	INSC;INSC	11	Body;Body	Open sea	56.15	4.21	61.02	4.73	-4.87	5.04E-06	2.02E-04
cg18257996		4	Intergenic	Open sea	35.31	5.04	40.18	5.99	-4.87	2.29E-04	5.50E-03
cg10187692	VPS13B;VPS13B	8	Body;Body	Open sea	21.10	4.04	25.97	5.23	-4.87	3.74E-06	1.53E-04
cg11124260		10	Intergenic	Open sea	54.27	4.04	59.14	3.68	-4.87	2.08E-08	1.09E-06
cg06525453	CPT2	1	TSS1500	N_Shore	25.78	4.44	30.65	5.80	-4.87	1.21E-04	3.27E-03
cg08133755	NAV2;NAV2;NAV2	11	Body;Body;Body	Open sea	53.22	4.57	58.09	4.13	-4.87	5.43E-07	2.53E-05
cg18417408		6	Intergenic	Open sea	69.90	4.49	74.77	4.74	-4.87	3.77E-06	1.54E-04
cg01204606		11	Intergenic	Island	10.98	4.55	15.85	6.73	-4.87	2.31E-04	5.54E-03
cg05453885	ZFP1	16	Body	Open sea	75.61	4.69	80.48	4.33	-4.87	3.62E-06	1.48E-04
cg01250212	GNG7	19	5'UTR	Island	67.30	6.18	72.17	5.83	-4.87	2.87E-04	6.58E-03
cg00730561	SEC31B	10	TSS200	Island	16.15	4.81	21.02	5.78	-4.87	4.41E-04	9.19E-03
cg07191834	PACRG;PACRG;PACRG	6	Body;Body;Body	Open sea	37.83	5.49	42.69	6.02	-4.87	1.38E-03	2.18E-02
cg07252200		10	Intergenic	Island	41.96	6.89	46.82	6.74	-4.87	2.35E-03	3.17E-02
cg12034757	NAGLU	17	Body	Open sea	78.04	5.39	82.91	4.28	-4.86	6.68E-05	1.99E-03
cg26101485		8	Intergenic	S_Shore	54.52	5.51	59.38	6.90	-4.86	1.88E-03	2.71E-02
cg15685922	MEX3A	1	3'UTR	N_Shore	37.55	5.27	42.42	6.18	-4.86	5.29E-04	1.06E-02
cg20123153	NTN1	17	Body	Island	12.27	3.71	17.14	4.64	-4.86	5.02E-09	2.72E-07
cg08599085		8	Intergenic	Open sea	44.31	5.60	49.18	6.80	-4.86	8.35E-04	1.49E-02
cg05208483	CSPG4	15	Body	Island	78.44	4.89	83.31	4.25	-4.86	1.90E-05	6.68E-04
cg12289964		4	Intergenic	Open sea	59.11	7.09	63.97	6.52	-4.86	3.58E-04	7.82E-03
cg19100169	CCNJL	5	Body	Open sea	43.52	5.26	48.38	6.99	-4.86	2.19E-04	5.30E-03
cg18101333	LEMD2	6	TSS1500	S_Shore	60.92	5.14	65.77	5.56	-4.86	1.78E-04	4.51E-03
cg22097731	TPM1;TPM1;TPM1;TPM1;TPM1;TPM1	15	TSS1500;TSS1500;TSS1500;TSS1500;TSS15	Island	10.37	4.23	15.23	5.36	-4.86	4.59E-07	2.15E-05
cg22681021	RGS8;RGS8;RGS8;RGS8	1	5'UTR;1stExon;1stExon;5'UTR	Open sea	49.40	5.62	54.25	5.89	-4.86	4.90E-05	1.53E-03
cg17545418	MT1IP	16	Body	Island	27.60	5.20	32.46	5.60	-4.86	1.97E-06	8.44E-05
cg26171271		6	Intergenic	S_Shelf	74.36	7.50	79.21	5.44	-4.86	4.57E-04	9.45E-03

cg02668581	MREG;MREG	2	5'UTR;1stExon	Island	16.97	6.44	21.82	8.88	-4.86	6.23E-04	1.20E-02
cg10094238	ARHGAP27;ARHGAP27;ARHGAP27	17	1stExon;5'UTR;5'UTR	Island	49.84	5.44	54.69	6.97	-4.86	1.69E-03	2.50E-02
cg23373626	FCHSD1	5	TSS1500	S_Shore	31.57	4.96	36.42	5.27	-4.85	3.24E-06	1.34E-04
cg11210422	PRDM16;PRDM16	1	Body;Body	N_Shelf	71.30	5.18	76.15	4.71	-4.85	2.36E-04	5.64E-03
cg20927649	GUCA1A;GUCA1A	6	5'UTR;1stExon	Open sea	58.77	3.57	63.63	3.99	-4.85	3.18E-08	1.65E-06
cg09093485	RNF39;RNF39	6	3'UTR;3'UTR	Island	62.67	5.82	67.53	6.81	-4.85	8.64E-04	1.54E-02
cg08485259		3	Intergenic	N_Shelf	50.89	4.51	55.74	4.59	-4.85	9.68E-06	3.66E-04
cg20446143	MS4A8B	11	TSS1500	Open sea	39.70	5.23	44.56	5.61	-4.85	2.81E-04	6.46E-03
cg10313947	VSIG2	11	TSS1500	Open sea	49.94	4.24	54.80	4.53	-4.85	1.14E-06	5.09E-05
cg06125229	MLPH;MLPH	2	5'UTR;5'UTR	S_Shore	43.95	8.14	48.80	6.81	-4.85	2.83E-03	3.60E-02
cg27577554		17	Intergenic	Open sea	36.99	4.80	41.84	6.87	-4.85	2.56E-04	6.01E-03
cg24437535	PLEKHO1	1	TSS1500	N_Shore	39.01	4.25	43.87	4.59	-4.85	1.00E-06	4.50E-05
cg10462778	ERGIC1	5	Body	Open sea	31.91	5.03	36.76	5.57	-4.85	1.47E-04	3.84E-03
cg24385580	SSH2	17	Body	Island	11.17	4.66	16.02	6.76	-4.85	1.77E-05	6.26E-04
cg26001764	EXTL3	8	5'UTR	S_Shore	34.72	4.35	39.57	6.40	-4.85	1.26E-04	3.39E-03
cg20287029	OR10W1	11	TSS1500	Open sea	62.49	6.67	67.34	5.98	-4.85	2.00E-03	2.83E-02
cg21464565	HOXA2	7	Body	N_Shelf	28.55	4.56	33.40	6.36	-4.85	2.24E-04	5.41E-03
cg06877024		6	Intergenic	Open sea	20.48	2.42	25.33	2.24	-4.85	2.92E-14	1.85E-12
cg22799757	SCRN1;SCRN1;SCRN1;SCRN1	7	Body;Body;Body;5'UTR	Open sea	60.40	5.47	65.25	5.41	-4.85	3.06E-05	1.01E-03
cg06724402	ANGPTL5	11	5'UTR	N_Shelf	42.39	6.19	47.24	5.26	-4.85	2.46E-03	3.27E-02
cg19257832		11	Intergenic	Open sea	59.91	5.22	64.75	5.32	-4.85	3.60E-05	1.17E-03
cg07826255	SGCA;SGCA	17	Body;Body	Open sea	31.13	5.13	35.98	5.58	-4.85	7.33E-06	2.84E-04
cg08823985	IL20RA	6	TSS1500	S_Shore	43.47	4.32	48.32	3.39	-4.85	1.58E-06	6.88E-05
cg12524553	ITCH	20	5'UTR	S_Shore	26.94	6.02	31.79	7.44	-4.85	2.10E-03	2.92E-02
cg25568066	03-Mar	5	3'UTR	Open sea	30.57	4.61	35.41	6.36	-4.85	2.43E-04	5.77E-03
cg04557057		7	Intergenic	S_Shore	16.62	5.11	21.46	8.39	-4.85	2.47E-03	3.28E-02
cg14992219		5	Intergenic	Open sea	68.00	5.62	72.84	5.67	-4.85	1.01E-05	3.82E-04
cg22387528	LOC285830;LOC285830	6	Body;Body	Open sea	65.79	6.84	70.63	4.60	-4.85	8.60E-04	1.53E-02
cg11690979	C1orf230	1	Body	S_Shore	14.95	3.48	19.79	4.43	-4.85	4.08E-07	1.93E-05
cg27514550	TAF4B	18	3'UTR	Open sea	64.94	6.70	69.78	7.04	-4.84	2.06E-03	2.89E-02
cg04930816	GNAO1;GNAO1	16	Body;Body	Open sea	51.95	3.59	56.80	3.73	-4.84	2.39E-07	1.16E-05
cg22888967	SLC46A3;SLC46A3	13	TSS1500;TSS1500	S_Shore	64.26	5.19	69.10	4.43	-4.84	5.48E-05	1.68E-03
cg17561788	RANBP1	22	3'UTR	N_Shelf	78.25	4.30	83.10	3.36	-4.84	1.62E-08	8.53E-07
cg15671317		5	Intergenic	Open sea	43.77	6.63	48.61	9.33	-4.84	3.69E-03	4.32E-02
cg19757631	SRM	1	Body	N_Shore	61.10	6.12	65.94	5.38	-4.84	6.92E-04	1.30E-02
cg14142743	NFIL3	9	5'UTR	N_Shelf	66.75	5.77	71.59	5.03	-4.84	4.87E-05	1.52E-03
cg23259607	INPP4B;INPP4B	4	Body;Body	Open sea	70.05	4.45	74.89	3.28	-4.84	1.28E-08	6.77E-07
cg21778340	SGCE;PEG10;SGCE;PEG10;SGCE	7	TSS1500;5'UTR;TSS1500;5'UTR;TSS1500	Island	43.81	4.96	48.65	4.12	-4.84	3.78E-06	1.54E-04
cg24863255	TMEM212	3	TSS200	Open sea	49.36	4.79	54.20	6.57	-4.84	7.49E-04	1.38E-02
cg17154218	NCOR2;NCOR2	12	Body;Body	S_Shore	61.10	4.79	65.94	3.27	-4.84	1.04E-05	3.89E-04
cg02172058	C17orf64;C17orf64	17	1stExon;5'UTR	S_Shore	67.33	6.48	72.17	3.82	-4.84	5.08E-04	1.03E-02
cg05104581	ACSF3;ACSF3;ACSF3	16	Body;5'UTR;5'UTR	N_Shelf	55.18	7.00	60.02	5.51	-4.84	1.29E-03	2.06E-02
cg18817529		8	Intergenic	S_Shore	69.11	6.13	73.95	5.06	-4.84	1.65E-03	2.47E-02
cg26636010		2	Intergenic	Open sea	41.54	7.78	46.38	7.63	-4.84	2.24E-03	3.06E-02
cg17158414	KRTCAP3;KRTCAP3	2	1stExon;1stExon	Island	48.96	5.81	53.79	6.21	-4.84	2.91E-03	3.66E-02
cg15809217	BAT3;BAT3;BAT3;BAT3	6	Body;Body;Body;Body	Open sea	68.16	6.37	73.00	5.95	-4.84	3.41E-05	1.11E-03
cg21791252		6	Intergenic	Open sea	38.34	4.71	43.18	4.80	-4.84	4.20E-06	1.71E-04
cg24483493	TPM1;TPM1;TPM1;TPM1;TPM1;TPM1	15	TSS1500;TSS1500;TSS1500;TSS1500;TSS15	N_Shore	56.23	7.27	61.07	6.01	-4.84	1.10E-03	1.83E-02
cg16624124		5	Intergenic	Open sea	46.41	4.75	51.25	6.73	-4.84	1.30E-04	3.48E-03
cg02587648	PRKCZ;PRKCZ	1	Body;5'UTR	N_Shore	45.79	5.64	50.63	6.51	-4.84	4.99E-04	1.01E-02
cg02283949	AIG1	6	Body	Open sea	59.35	4.86	64.19	4.72	-4.84	3.33E-05	1.09E-03
cg04921315		16	Intergenic	Open sea	65.17	7.48	70.00	6.07	-4.84	1.29E-03	2.06E-02
cg25083618	CYP26C1	10	TSS200	Island	55.31	4.53	60.14	5.07	-4.84	3.67E-05	1.19E-03

cg01623008		15	Intergenic	Open sea	72.42	5.61	77.26	5.06	-4.84	4.54E-04	9.41E-03
cg12744722	CUX2	12	Body	N_Shore	22.21	8.46	27.05	9.41	-4.84	2.97E-03	3.72E-02
cg13356133	RICH2	17	Body	Island	19.07	4.63	23.90	8.82	-4.84	1.27E-03	2.05E-02
cg19904058	LOC619207	10	Body	Island	46.70	7.48	51.53	7.22	-4.83	1.80E-03	2.63E-02
cg17408087	BHMT2	5	Body	S_Shelf	57.55	5.31	62.38	6.07	-4.83	1.16E-03	1.92E-02
cg03930929	LRRN1	3	5'UTR	Open sea	69.22	5.79	74.05	4.94	-4.83	6.90E-06	2.68E-04
cg13845858	ABCA2;ABCA2	9	Body;Body	N_Shore	41.88	4.60	46.71	7.08	-4.83	1.11E-03	1.85E-02
cg19236559	UPB1	22	Body	S_Shelf	49.38	5.98	54.21	7.49	-4.83	3.09E-03	3.82E-02
cg06707765	GATA4	8	Body	N_Shelf	37.49	4.51	42.32	4.69	-4.83	1.91E-05	6.73E-04
cg03007010	SNX26	19	Body	S_Shore	15.74	5.46	20.57	5.43	-4.83	8.34E-05	2.40E-03
cg13532055	FXN;FXN;FXN	9	TSS1500;TSS1500;TSS1500	N_Shore	57.75	4.94	62.58	4.98	-4.83	1.93E-04	4.79E-03
cg22253078		2	Intergenic	Open sea	66.45	6.50	71.28	8.44	-4.83	4.17E-03	4.69E-02
cg01259782	CRYM;NCRNA00169	16	5'UTR;Body	S_Shore	62.66	5.79	67.49	3.93	-4.83	1.25E-04	3.37E-03
cg01612936	CEP250	20	Body	Open sea	35.77	5.58	40.60	6.14	-4.83	1.78E-03	2.61E-02
cg17977099		7	Intergenic	Open sea	62.84	6.21	67.67	6.50	-4.83	3.77E-03	4.38E-02
cg07107453	IFI44	1	TSS1500	Open sea	43.02	4.76	47.85	5.51	-4.83	2.56E-05	8.70E-04
cg16024937	ACOX1;ACOX1	17	3'UTR;3'UTR	S_Shelf	72.87	5.00	77.70	4.60	-4.83	1.14E-05	4.22E-04
cg18313661		7	Intergenic	Island	35.07	7.54	39.90	6.06	-4.83	2.83E-03	3.60E-02
cg00759329		1	Intergenic	Open sea	44.99	5.59	49.82	6.70	-4.83	1.01E-03	1.73E-02
cg00655184	CTAGE1	18	TSS200	Open sea	59.53	4.69	64.36	6.00	-4.83	1.30E-04	3.48E-03
cg14854517	RAPGEF3;RAPGEF3;RAPGEF3;RAPGEF	12	1stExon;5'UTR;TSS1500;TSS200	N_Shore	48.64	6.00	53.47	5.99	-4.83	4.15E-04	8.77E-03
cg22319016	FZD4	11	Body	N_Shelf	72.68	5.30	77.50	4.36	-4.83	1.61E-04	4.15E-03
cg07209071	PRDM16;PRDM16	1	Body;Body	N_Shelf	48.94	4.74	53.77	6.61	-4.83	5.81E-05	1.77E-03
cg26147098		8	Intergenic	Island	47.47	6.76	52.29	6.62	-4.83	1.71E-03	2.52E-02
cg18202741	AQP6	12	TSS200	Open sea	50.24	5.28	55.07	3.97	-4.82	6.64E-05	1.98E-03
cg17728125	SBNO2;SBNO2	19	Body;Body	S_Shore	30.96	4.20	35.78	5.96	-4.82	1.43E-04	3.76E-03
cg17562697		6	Intergenic	S_Shore	60.26	6.50	65.08	5.34	-4.82	1.04E-03	1.76E-02
cg20198611	RXRB	6	Body	S_Shelf	44.46	3.88	49.28	5.09	-4.82	2.83E-05	9.48E-04
cg09852187	MIR7-2	15	TSS200	Open sea	80.79	4.63	85.61	3.87	-4.82	6.24E-07	2.88E-05
cg19802981	ADAMTS10	19	Body	N_Shore	70.98	5.27	75.80	4.07	-4.82	2.69E-05	9.07E-04
cg00275741	PCDHA7;PCDHA12;PCDHA6;PCDHA10	5	Body;Body;Body;Body;Body;Body;Body;Bo	N_Shore	19.77	5.36	24.59	4.95	-4.82	6.70E-05	2.00E-03
cg09299592	SGSM3	22	TSS1500	N_Shore	68.53	4.55	73.36	4.15	-4.82	9.13E-07	4.12E-05
cg14311630	SERPING1	11	TSS1500	Open sea	54.41	5.78	59.23	6.61	-4.82	1.22E-03	1.99E-02
cg05513208	XAF1;XAF1;XAF1;XAF1	17	5'UTR;5'UTR;1stExon;1stExon	Open sea	10.70	4.63	15.52	6.90	-4.82	6.91E-04	1.30E-02
cg13644645	MRPL14	6	3'UTR	Open sea	75.45	5.25	80.27	4.07	-4.82	5.64E-05	1.72E-03
cg16539741	CHN2	7	Body	Open sea	43.77	7.25	48.59	6.41	-4.82	3.69E-03	4.32E-02
cg17822779		8	Intergenic	Open sea	33.33	4.12	38.15	4.59	-4.82	9.72E-06	3.67E-04
cg15252509	MALT1;MALT1	18	TSS1500;TSS1500	N_Shore	48.01	4.90	52.83	5.21	-4.82	1.02E-04	2.84E-03
cg21348565	SLC22A25	11	TSS200	Open sea	76.96	5.44	81.79	4.47	-4.82	1.28E-04	3.44E-03
cg09676630	FBRSL1	12	Body	N_Shore	56.01	3.77	60.83	4.36	-4.82	1.33E-09	7.39E-08
cg08438920	EIF5A2	3	Body	N_Shelf	70.09	7.04	74.91	6.09	-4.82	3.44E-03	4.12E-02
cg25252561	CAMK2G;CAMK2G;CAMK2G;CAMK2G	10	Body;Body;Body;Body;Body	Open sea	41.31	4.42	46.13	4.43	-4.82	6.91E-06	2.69E-04
cg10333402	MOGAT3	7	TSS1500	Island	18.88	5.17	23.70	5.04	-4.82	1.14E-05	4.24E-04
cg03315817	ALS2CR8;ALS2CR8	2	Body;Body	Open sea	76.59	7.03	81.41	4.66	-4.82	1.06E-03	1.79E-02
cg25077558	ADAM19	5	Body	Open sea	27.43	5.12	32.25	7.10	-4.82	1.66E-03	2.48E-02
cg00257800		2	Intergenic	Open sea	40.54	5.29	45.36	6.16	-4.82	1.12E-04	3.07E-03
cg16750112	BBS9;BBS9;BBS9;BBS9	7	Body;Body;Body;Body	Open sea	67.46	4.47	72.28	4.53	-4.82	5.56E-05	1.70E-03
cg23539633		11	Intergenic	Open sea	67.14	7.04	71.96	6.85	-4.82	3.46E-04	7.61E-03
cg14751552	RDH10	8	Body	S_Shore	48.54	4.11	53.36	5.63	-4.82	9.28E-05	2.63E-03
cg03415916	PCDHGA4;PCDHGA9;PCDHGA1;PCDH(	5	Body;Body;Body;Body;1stExon;1stExon;Bo	N_Shore	72.39	6.04	77.20	5.16	-4.82	2.47E-03	3.28E-02
cg24082440	MRAP;MRAP	21	Body;Body	N_Shore	70.48	8.07	75.30	6.72	-4.82	3.43E-03	4.11E-02
cg27305304	MREG;MREG	2	5'UTR;1stExon	Island	21.82	7.74	26.64	10.20	-4.82	1.22E-03	1.99E-02
cg22133378	GLYATL1	11	3'UTR	Open sea	55.38	5.53	60.20	6.50	-4.82	1.90E-03	2.72E-02

cg24051554	LOC100132111;C2CD4D	1	Body;5'UTR	Island	20.44	4.66	25.26	6.08	-4.82	3.73E-05	1.20E-03
cg26985149		17	Intergenic	Island	46.38	4.38	51.20	4.44	-4.82	7.88E-06	3.03E-04
cg06056884	MUC15;MUC15;MUC15;MUC15;MUC	11	1stExon;5'UTR;1stExon;1stExon;5'UTR;Boc	Open sea	41.81	4.60	46.63	6.59	-4.82	1.79E-04	4.53E-03
cg27389454	BRSK1	19	Body	N_Shore	48.70	5.31	53.51	7.11	-4.82	6.52E-04	1.24E-02
cg08884979	SCG3;SCG3	15	3'UTR;3'UTR	Open sea	73.62	5.84	78.44	6.60	-4.82	2.37E-05	8.12E-04
cg22940268	FAM73A	1	TSS1500	Open sea	72.94	4.78	77.75	4.73	-4.82	6.24E-06	2.45E-04
cg21304285	C1orf100	1	5'UTR	Open sea	53.04	6.42	57.85	4.97	-4.82	1.60E-03	2.41E-02
cg22606681	TRIM24;TRIM24	7	Body;Body	S_Shore	28.95	6.21	33.76	7.30	-4.81	2.43E-03	3.24E-02
cg01347702	SLC44A4	6	Body	Open sea	70.36	5.14	75.18	4.85	-4.81	7.97E-06	3.07E-04
cg03233876	BSG;BSG;BSG	19	Body;Body;Body	N_Shelf	37.32	5.20	42.13	6.20	-4.81	8.76E-04	1.55E-02
cg16274678	TPM3;TPM3;NUP210L;TPM3;TPM3;NI	1	3'UTR;3'UTR;TSS1500;3'UTR;3'UTR;TSS15(	Open sea	62.66	5.54	67.47	5.49	-4.81	1.68E-05	6.00E-04
cg16510793		9	Intergenic	Island	17.68	5.04	22.49	4.30	-4.81	4.80E-07	2.25E-05
cg09780231	NXN	17	Body	Open sea	51.17	8.88	55.98	10.79	-4.81	4.03E-03	4.59E-02
cg20060666	PRKAR1A;PRKAR1A;PRKAR1A	17	3'UTR;3'UTR;3'UTR	Open sea	67.03	5.90	71.85	6.03	-4.81	4.02E-03	4.58E-02
cg20595453	VPS52	6	Body	S_Shore	68.05	3.54	72.86	3.55	-4.81	5.34E-09	2.88E-07
cg13641591	UVRAG	11	Body	S_Shore	73.29	5.06	78.10	3.09	-4.81	1.57E-06	6.84E-05
cg03489186	FANCI;FANCI	15	5'UTR;5'UTR	S_Shelf	68.02	4.90	72.84	4.67	-4.81	8.07E-06	3.10E-04
cg19211853	RBM26	13	Body	Open sea	48.70	8.17	53.51	6.19	-4.81	2.29E-03	3.11E-02
cg11145461	CSRP1;CSRP1	1	5'UTR;5'UTR	N_Shore	23.81	5.18	28.63	6.32	-4.81	1.49E-03	2.30E-02
cg26705266	ANO1	11	TSS200	Island	8.34	4.17	13.15	6.95	-4.81	2.77E-05	9.32E-04
cg02874031	RFX4	12	Body	Open sea	47.56	6.55	52.37	6.22	-4.81	4.05E-04	8.60E-03
cg13282547	TBPL1	6	5'UTR	Open sea	73.22	6.78	78.03	4.66	-4.81	4.75E-04	9.74E-03
cg02311364	IQSEC1;IQSEC1	3	Body;Body	Open sea	65.06	5.23	69.87	4.24	-4.81	1.86E-05	6.55E-04
cg25749107		12	Intergenic	Open sea	53.77	4.95	58.58	6.08	-4.81	2.35E-06	9.95E-05
cg21300318	MIR7-3;C19orf30	19	TSS1500;Body	Open sea	47.35	7.04	52.16	6.91	-4.81	4.18E-03	4.70E-02
cg08962888	LGALS3BP	17	Body	Open sea	59.00	4.96	63.81	6.07	-4.81	8.30E-04	1.49E-02
cg21286701	C6orf174	6	Body	S_Shore	59.36	4.93	64.17	7.35	-4.81	1.33E-03	2.12E-02
cg18817459	SOBP	6	3'UTR	Open sea	63.29	5.22	68.10	5.12	-4.81	7.44E-06	2.88E-04
cg19368031	MYST4	10	Body	Open sea	38.99	5.34	43.80	6.06	-4.81	5.52E-04	1.09E-02
cg09975715	MYH10	17	3'UTR	N_Shore	73.49	5.34	78.29	4.71	-4.81	1.70E-04	4.34E-03
cg01851672	PPAP2A;PPAP2A;RNF138P1	5	Body;Body;Body	N_Shore	25.23	4.84	30.04	5.25	-4.81	1.93E-04	4.81E-03
cg13747435	AK1	9	Body	Open sea	72.51	5.21	77.32	4.90	-4.81	4.83E-05	1.51E-03
cg03954045		12	Intergenic	Open sea	55.87	6.13	60.67	8.00	-4.81	2.75E-03	3.52E-02
cg11018313	ALPK3;ALPK3	15	1stExon;5'UTR	N_Shore	47.89	5.41	52.70	6.60	-4.81	1.73E-03	2.55E-02
cg23930230		19	Intergenic	N_Shelf	64.62	4.41	69.43	4.15	-4.81	3.17E-06	1.31E-04
cg08404739	ABCC11;ABCC11	16	TSS1500;TSS1500	Open sea	43.85	5.29	48.65	7.04	-4.81	1.74E-03	2.56E-02
cg14777244	SPACA4;FAM83E;SPACA4	19	1stExon;Body;5'UTR	S_Shelf	58.94	4.67	63.74	4.53	-4.81	3.09E-06	1.28E-04
cg05739816	C6orf217	6	Body	Open sea	22.19	6.56	27.00	7.59	-4.81	2.24E-03	3.06E-02
cg25431535		22	Intergenic	N_Shelf	55.75	6.67	60.55	5.51	-4.81	2.33E-04	5.58E-03
cg16415646	LOC285954;LOC285954;INHBA	7	Body;Body;TSS1500	N_Shelf	47.90	5.85	52.71	6.81	-4.81	1.50E-03	2.30E-02
cg04414451	SULT1C4	2	TSS1500	Open sea	43.36	5.60	48.17	5.46	-4.80	2.55E-05	8.66E-04
cg27570661		16	Intergenic	S_Shore	31.47	5.28	36.27	4.93	-4.80	1.65E-04	4.24E-03
cg27405731	CUX1;CUX1;CUX1	7	Body;Body;Body	Open sea	24.50	4.84	29.31	5.83	-4.80	4.66E-04	9.59E-03
cg10294253		15	Intergenic	Open sea	51.73	6.74	56.53	5.74	-4.80	4.42E-04	9.22E-03
cg21164545	LOC100240726	20	TSS1500	Open sea	72.18	8.12	76.99	6.49	-4.80	3.55E-03	4.21E-02
cg06212670	ZFPM1	16	Body	S_Shelf	37.77	5.50	42.57	7.60	-4.80	4.09E-03	4.64E-02
cg18428234		12	Intergenic	Island	42.35	4.85	47.15	6.14	-4.80	7.33E-05	2.16E-03
cg17591595	HOXA4	7	TSS1500	S_Shore	33.18	3.47	37.98	4.13	-4.80	2.23E-08	1.16E-06
cg09481121	CMYA5	5	TSS1500	Open sea	37.21	4.88	42.01	7.02	-4.80	1.02E-03	1.74E-02
cg06989693	PLCXD3	5	Body	Open sea	33.83	4.47	38.63	5.54	-4.80	4.13E-05	1.32E-03
cg04493169	JAZF1	7	Body	Open sea	43.25	5.70	48.05	6.69	-4.80	3.95E-03	4.52E-02
cg00977978	HIPK2;HIPK2	7	Body;Body	Open sea	82.42	4.73	87.22	4.37	-4.80	7.42E-06	2.87E-04
cg07349815		3	Intergenic	N_Shore	32.42	5.47	37.22	5.68	-4.80	7.70E-04	1.41E-02

cg00936348	HECW1	7	Body	Open sea	67.09	7.03	71.89	5.24	-4.80	1.84E-04	4.63E-03
cg04291891		11	Intergenic	Open sea	48.84	4.40	53.64	4.84	-4.80	1.28E-06	5.65E-05
cg09987231	MTA1	14	Body	Open sea	49.20	4.50	54.00	6.64	-4.80	6.34E-04	1.22E-02
cg14184866		9	Intergenic	N_Shore	53.07	6.34	57.87	5.82	-4.80	3.76E-04	8.12E-03
cg17986992	VSIG8;C1orf204	1	Body;TSS1500	S_Shore	49.36	4.93	54.16	7.64	-4.80	2.65E-03	3.44E-02
cg09291217		8	Intergenic	S_Shelf	38.68	6.17	43.48	7.08	-4.80	4.02E-03	4.58E-02
cg14286208	KLHL33	14	5'UTR	Island	44.10	4.73	48.90	5.77	-4.80	1.68E-04	4.30E-03
cg12893780	HLA-DPB1	6	Body	Island	27.02	5.55	31.82	4.84	-4.80	3.55E-04	7.77E-03
cg27263998	IGF2;INS-IGF2;INS-IGF2	11	TSS200;Body;Body	Open sea	26.59	4.63	31.38	5.06	-4.80	3.33E-05	1.09E-03
cg20764033	RFC2;RFC2	7	Body;Body	Open sea	70.43	4.89	75.23	4.77	-4.80	1.70E-05	6.03E-04
cg13527921	NANS	9	Body	S_Shore	11.10	2.58	15.90	4.22	-4.80	2.47E-08	1.29E-06
cg27134944	COMMD6;COMMD6	13	Body;Body	N_Shore	47.02	5.91	51.81	5.95	-4.80	6.24E-06	2.45E-04
cg22762215		6	Intergenic	Island	38.24	5.88	43.03	4.85	-4.80	3.00E-04	6.80E-03
cg23095988	ACOT7;ACOT7;ACOT7	1	Body;1stExon;5'UTR	Open sea	65.29	4.26	70.09	4.22	-4.80	3.20E-06	1.32E-04
cg10897223	TRIM40	6	Body	Open sea	60.79	4.05	65.58	6.38	-4.79	1.27E-04	3.40E-03
cg03971661		2	Intergenic	N_Shelf	54.36	7.63	59.15	6.25	-4.79	2.16E-03	2.98E-02
cg03410667	ZCCHC24	10	3'UTR	Island	54.33	4.01	59.12	4.08	-4.79	1.93E-06	8.28E-05
cg24389054	HOXA7	7	3'UTR	N_Shore	37.66	5.74	42.45	6.03	-4.79	7.15E-04	1.33E-02
cg06121808	SLC20A1	2	Body	S_Shore	49.93	4.50	54.72	4.82	-4.79	1.93E-06	8.31E-05
cg00809267	NEDD4L;NEDD4L;NEDD4L;NEDD4L;NE	18	TSS200;5'UTR;TSS200;TSS200;Body;TSS20	Open sea	41.24	5.32	46.03	5.27	-4.79	2.01E-05	7.03E-04
cg09690483		2	Intergenic	Open sea	62.61	5.41	67.40	5.67	-4.79	3.34E-04	7.41E-03
cg08903465		1	Intergenic	Open sea	47.10	3.35	51.90	3.92	-4.79	1.54E-07	7.56E-06
cg04128746	FAM196A;DOCK1	10	TSS1500;Body	Island	28.39	4.85	33.18	4.28	-4.79	5.23E-06	2.09E-04
cg24707573	SH2B2	7	Body	Island	30.51	5.22	35.30	4.98	-4.79	5.47E-05	1.68E-03
cg00321824	SLC22A3	6	3'UTR	Open sea	65.79	6.61	70.58	5.80	-4.79	7.56E-04	1.39E-02
cg09461545	SEL1L3	4	Body	Open sea	38.67	4.21	43.46	4.22	-4.79	2.58E-06	1.08E-04
cg08390254	ATP1A2	1	TSS1500	Open sea	47.89	4.22	52.69	6.41	-4.79	7.02E-05	2.08E-03
cg22065514	COL7A1;UCN2	3	Body;TSS1500	Open sea	74.92	4.37	79.72	3.82	-4.79	7.90E-08	3.97E-06
cg25523633	SNORD57;NOP56;NOP56;SNORD56	20	TSS1500;Body;Body;TSS200	S_Shelf	55.45	5.82	60.24	5.24	-4.79	1.32E-03	2.10E-02
cg15738800	PRR15L	17	TSS200	Open sea	40.76	3.48	45.55	4.75	-4.79	4.59E-06	1.85E-04
cg16595288	RASGEF1A	10	Body	S_Shore	48.88	4.96	53.67	6.82	-4.79	1.70E-03	2.51E-02
cg22446399	CGREF1;CGREF1;CGREF1;CGREF1	2	Body;Body;Body;5'UTR	Open sea	47.46	4.86	52.25	6.36	-4.79	4.36E-04	9.12E-03
cg21831174	MASP1;MASP1;MASP1	3	TSS1500;TSS1500;TSS1500	Open sea	27.59	4.36	32.38	6.37	-4.79	3.07E-04	6.92E-03
cg02627216	IGFBP4	17	TSS1500	N_Shore	34.43	6.04	39.22	5.85	-4.79	1.76E-03	2.58E-02
cg02690013	DLGAP2	8	3'UTR	S_Shore	56.15	5.44	60.94	6.11	-4.79	2.96E-04	6.73E-03
cg09865015	LOC100130872;LOC100130872-SPON2	4	Body;Body	N_Shore	59.39	6.03	64.18	4.02	-4.79	9.85E-05	2.77E-03
cg21842967	DDHD1;DDHD1;DDHD1	14	3'UTR;3'UTR;3'UTR	Open sea	73.59	3.89	78.38	3.69	-4.79	2.22E-08	1.16E-06
cg00379559	ZFPM1	16	Body	S_Shore	35.47	4.43	40.26	5.99	-4.79	1.88E-04	4.70E-03
cg19368582	MMRN2	10	Body	Open sea	63.73	3.80	68.52	3.31	-4.79	1.58E-08	8.34E-07
cg17485265	FAM50B	6	TSS1500	N_Shore	52.54	5.50	57.33	4.97	-4.79	6.32E-04	1.21E-02
cg03421069	PAN3;PAN3	13	Body;Body	Open sea	39.24	4.83	44.03	5.44	-4.79	1.57E-04	4.07E-03
cg13529335	SMARCA2;SMARCA2	9	Body;Body	Open sea	18.39	5.48	23.18	7.52	-4.79	2.03E-04	4.99E-03
cg13621396	STIM2;STIM2;STIM2	4	TSS1500;TSS1500;TSS1500	N_Shore	26.20	4.73	30.99	4.94	-4.79	1.27E-04	3.40E-03
cg23250715	CD2AP	6	TSS1500	N_Shore	71.78	7.11	76.56	5.84	-4.79	3.22E-03	3.93E-02
cg00357273	POU5F1	6	TSS200	Open sea	65.02	6.24	69.81	6.22	-4.79	1.73E-03	2.55E-02
cg01521060		11	Intergenic	N_Shelf	30.13	4.48	34.92	6.94	-4.79	1.09E-03	1.82E-02
cg26665667	SGMS1	10	5'UTR	Open sea	59.40	5.90	64.19	6.09	-4.79	1.25E-03	2.02E-02
cg12550399	SLC47A1	17	3'UTR	N_Shore	63.18	6.33	67.97	7.15	-4.79	4.44E-03	4.90E-02
cg26890354	PCDHGA4;PCDHGC3;PCDHGA12;PCDH	5	Body;Body;Body;TSS1500;Body;Body;Body	N_Shore	29.12	4.40	33.91	5.48	-4.79	4.56E-05	1.44E-03
cg11075718	GOLSYN;GOLSYN	8	5'UTR;5'UTR	N_Shelf	47.91	6.22	52.70	6.91	-4.78	8.43E-04	1.51E-02
cg21871232	CLMN	14	Body	Open sea	79.19	5.81	83.98	4.07	-4.78	1.30E-05	4.76E-04
cg14518346	CLYBL	13	3'UTR	N_Shelf	77.47	5.48	82.25	4.74	-4.78	2.37E-04	5.67E-03
cg27260312		3	Intergenic	Open sea	72.50	5.45	77.29	5.46	-4.78	2.90E-04	6.62E-03

cg03228893		19	Intergenic	N_Shelf	28.20	6.34	32.98	6.57	-4.78	2.64E-04	6.16E-03
cg15854662	ANKMY1;ANKMY1	2	Body;Body	Open sea	74.95	6.87	79.74	5.38	-4.78	1.16E-04	3.16E-03
cg05451527	OSGIN1;OSGIN1;OSGIN1	16	3'UTR;3'UTR;3'UTR	S_Shore	63.77	5.93	68.56	5.55	-4.78	7.29E-04	1.35E-02
cg20569893	FLOT2	17	Body	N_Shelf	23.50	3.57	28.29	5.57	-4.78	6.92E-05	2.05E-03
cg20483374	C1QTNF5;MFRP	11	5'UTR;3'UTR	Island	49.07	5.90	53.85	5.73	-4.78	1.98E-04	4.91E-03
cg16241421	CUX2	12	Body	N_Shore	20.40	6.57	25.18	8.28	-4.78	3.99E-04	8.50E-03
cg02882785	CLDN22	4	TSS200	Open sea	75.45	5.12	80.23	3.65	-4.78	5.03E-05	1.56E-03
cg00526772	CASC2;CASC2;CASC2	10	Body;Body;Body	Open sea	59.76	5.42	64.54	5.91	-4.78	3.43E-04	7.55E-03
cg00994250	PLEKHG1	6	Body	Open sea	38.88	5.36	43.66	5.84	-4.78	1.14E-03	1.89E-02
cg19820921	ATP11B	3	Body	Open sea	79.59	6.83	84.37	6.41	-4.78	1.92E-03	2.75E-02
cg26441877	CHD7	8	5'UTR	N_Shore	46.95	6.62	51.73	5.76	-4.78	1.65E-03	2.46E-02
cg11270070	ZMYND10;ZMYND10	3	1stExon;5'UTR	Island	16.94	7.12	21.72	6.98	-4.78	1.77E-03	2.59E-02
cg23927469	TBC1D14	4	TSS1500	N_Shore	57.15	6.49	61.93	6.79	-4.78	3.55E-04	7.77E-03
cg24413781	LASS4	19	TSS1500	Island	12.27	4.19	17.05	4.97	-4.78	1.39E-06	6.09E-05
cg17069739	CLIC1	6	Body	Open sea	15.68	7.24	20.46	7.87	-4.78	1.62E-04	4.17E-03
cg24317742		1	Intergenic	Open sea	78.48	7.37	83.26	5.70	-4.78	3.30E-03	4.00E-02
cg05413282	PRSS3;PRSS3	9	Body;Body	Open sea	50.25	5.83	55.04	4.96	-4.78	4.28E-04	8.98E-03
cg18247055	SPAG6;SPAG6	10	TSS200;TSS200	Island	13.35	4.14	18.13	5.23	-4.78	9.09E-06	3.45E-04
cg19499085	ATP8B3	19	Body	Island	51.70	6.46	56.48	5.93	-4.78	8.91E-04	1.57E-02
cg22726349	PRDM16;PRDM16	1	Body;Body	Island	46.13	4.56	50.91	4.70	-4.78	6.91E-05	2.05E-03
cg18687753	BPTF;BPTF	17	Body;Body	Open sea	69.64	5.45	74.42	5.71	-4.78	6.15E-04	1.19E-02
cg19851487	HRC	19	Body	S_Shore	37.19	4.66	41.96	5.84	-4.78	1.93E-05	6.76E-04
cg16957569	IDO2	8	TSS1500	Open sea	73.22	4.82	77.99	3.19	-4.78	1.73E-06	7.47E-05
cg25614935	C10orf110;C10orf110;C10orf110;C10c	10	Body;Body;Body;Body	Open sea	77.92	7.63	82.70	5.04	-4.78	1.06E-03	1.79E-02
cg01660827	CNNM3;CNNM3	2	Body;Body	S_Shelf	74.36	5.74	79.13	5.90	-4.78	5.37E-04	1.07E-02
cg26635219	CFTR	7	TSS1500	Open sea	27.98	8.38	32.76	9.42	-4.78	4.15E-04	8.77E-03
cg01215651		4	Intergenic	Open sea	51.99	7.12	56.77	5.85	-4.78	1.91E-03	2.74E-02
cg22354646	FAM107A;FAM107A	3	3'UTR;3'UTR	Open sea	20.70	5.37	25.48	4.96	-4.78	4.86E-05	1.52E-03
cg13470969		1	Intergenic	S_Shelf	75.47	4.87	80.25	3.49	-4.77	5.75E-07	2.66E-05
cg17695537		2	Intergenic	Open sea	41.29	4.99	46.07	5.84	-4.77	2.40E-04	5.71E-03
cg03580107		1	Intergenic	S_Shelf	46.03	4.01	50.81	5.22	-4.77	3.71E-06	1.52E-04
cg14827832		17	Intergenic	Open sea	38.90	5.88	43.68	6.41	-4.77	8.36E-04	1.50E-02
cg05090785	PDE1C	7	Body	Open sea	30.22	5.05	35.00	7.45	-4.77	1.45E-03	2.25E-02
cg20699305	ACTL8	1	Body	Island	56.73	5.06	61.50	4.74	-4.77	1.62E-05	5.79E-04
cg07596467		19	Intergenic	N_Shelf	33.30	3.86	38.08	5.33	-4.77	2.86E-05	9.56E-04
cg15442888		4	Intergenic	Open sea	45.29	4.68	50.07	5.51	-4.77	2.89E-05	9.65E-04
cg26459819	C11orf93;C11orf92	11	TSS1500;5'UTR	Island	48.49	6.35	53.26	5.79	-4.77	2.53E-04	5.96E-03
cg05237640		16	Intergenic	Open sea	65.96	5.99	70.73	7.02	-4.77	1.91E-03	2.74E-02
cg17526175	WDR88	19	Body	N_Shore	59.32	7.17	64.09	5.02	-4.77	4.07E-04	8.63E-03
cg03602288	HOXB3	17	5'UTR	Island	25.88	4.86	30.65	4.92	-4.77	6.00E-05	1.82E-03
cg07515250	PTK2;PTK2	8	Body;Body	Open sea	77.05	4.85	81.82	3.81	-4.77	5.37E-07	2.50E-05
cg24398379	LOC650226	7	TSS200	S_Shore	39.37	7.53	44.13	5.58	-4.77	3.28E-03	3.98E-02
cg03793551	TEKT5	16	TSS1500	Open sea	40.80	4.60	45.57	4.13	-4.77	2.36E-06	9.99E-05
cg19280364	ADPGK;ADPGK;ADPGK	15	TSS1500;TSS1500;TSS1500	S_Shore	80.56	3.92	85.33	3.81	-4.77	1.09E-06	4.88E-05
cg00856997	ZFYVE28	4	Body	N_Shore	72.46	8.56	77.23	6.55	-4.77	3.17E-03	3.88E-02
cg01838004	KIAA1529	9	Body	Island	33.55	5.98	38.31	7.27	-4.77	3.99E-03	4.56E-02
cg00227578	RAI14;RAI14;RAI14	5	5'UTR;5'UTR;5'UTR	S_Shore	21.62	6.54	26.38	8.22	-4.77	2.25E-03	3.07E-02
cg22682811		20	Intergenic	Island	37.50	5.19	42.27	5.07	-4.77	1.86E-06	8.01E-05
cg02627227	AGPAT4	6	5'UTR	Open sea	59.89	5.26	64.65	6.15	-4.77	9.41E-05	2.66E-03
cg22141459	TP53I13	17	Body	N_Shore	31.33	4.34	36.09	6.62	-4.77	7.87E-04	1.43E-02
cg01416891	CYP26C1	10	TSS200	Island	17.92	5.04	22.68	7.73	-4.77	1.96E-03	2.79E-02
cg24374013	ATP2B2;ATP2B2	3	Body;Body	Open sea	59.64	4.10	64.41	3.67	-4.77	3.63E-07	1.72E-05
cg00194126	NR4A2	2	Body	Island	52.00	6.62	56.76	6.83	-4.77	1.88E-04	4.70E-03

cg07435215	LOC729082	15	TSS1500	N_Shore	38.99	5.85	43.75	6.38	-4.76	3.34E-03	4.04E-02
cg14796908	FAM198B;FAM198B;FAM198B	4	Body;Body;Body	Open sea	67.68	6.56	72.45	6.05	-4.76	1.03E-03	1.75E-02
cg06705122	PLEKHA4;PLEKHA4	19	5'UTR;5'UTR	Open sea	40.46	6.05	45.22	6.99	-4.76	1.95E-03	2.77E-02
cg26710209	VSIG2	11	3'UTR	S_Shore	72.17	6.31	76.93	7.23	-4.76	3.69E-03	4.32E-02
cg12602633	PRRT1;PRRT1	6	1stExon;5'UTR	S_Shore	35.34	5.60	40.10	5.89	-4.76	1.61E-04	4.15E-03
cg02004337	MARCKS	6	Body	Island	34.25	4.00	39.01	6.58	-4.76	1.63E-04	4.20E-03
cg09427311	ANGPTL2;RALGPS1	9	TSS1500;Body	Open sea	28.71	5.95	33.48	6.60	-4.76	2.00E-03	2.83E-02
cg27569446	RASSF1;RASSF1	3	TSS200;TSS200	Island	41.57	4.47	46.33	4.49	-4.76	3.58E-06	1.47E-04
cg01577475	PAX8;PAX8;PAX8;PAX8;PAX8;LOC440(	2	Body;Body;Body;Body;Body;Body	Island	33.35	7.04	38.12	7.63	-4.76	1.32E-03	2.10E-02
cg20045970	GLTP	12	TSS1500	S_Shore	51.27	5.38	56.04	4.58	-4.76	7.37E-05	2.16E-03
cg01149940	TRABD	22	Body	Island	62.71	5.48	67.47	5.05	-4.76	9.37E-04	1.63E-02
cg00425217	NFIX	19	3'UTR	N_Shore	48.68	4.35	53.44	3.92	-4.76	1.89E-06	8.12E-05
cg10347032	MAP6D1	3	TSS1500	Island	32.81	4.43	37.58	4.59	-4.76	9.69E-08	4.84E-06
cg18085435	ATP8B1	18	TSS1500	Open sea	65.07	5.03	69.83	3.77	-4.76	4.79E-06	1.92E-04
cg26956135	C17orf47;C17orf47	17	5'UTR;1stExon	Open sea	72.37	4.25	77.13	4.10	-4.76	1.15E-06	5.11E-05
cg08290728	CDK1;CDC2;CDK1;CDK1;CDK1	10	TSS1500;TSS1500;TSS1500;TSS1500;TSS15	N_Shore	69.73	6.49	74.50	5.91	-4.76	1.58E-03	2.39E-02
cg05129572	NEDD4;NEDD4	15	Body;Body	Open sea	57.49	7.51	62.25	7.34	-4.76	2.27E-03	3.09E-02
cg05595088		16	Intergenic	Island	74.81	6.74	79.57	6.37	-4.76	2.06E-03	2.89E-02
cg23756264	CPT1A;CPT1A	11	5'UTR;5'UTR	N_Shore	53.12	4.31	57.88	4.40	-4.76	4.53E-06	1.83E-04
cg16364495	APBB2;APBB2;APBB2;APBB2;APBB2;A	4	Body;TSS200;TSS200;Body;TSS200;Body;T	S_Shore	42.12	7.66	46.89	6.49	-4.76	1.49E-04	3.89E-03
cg04503600	PCDHGA4;PCDHGA9;PCDHGA1;PCDH(	5	Body;Body;Body;Body;1stExon;Body;Body	N_Shore	35.65	5.80	40.41	7.27	-4.76	1.51E-03	2.31E-02
cg11168614		14	Intergenic	Open sea	66.64	4.40	71.40	5.14	-4.76	6.22E-06	2.44E-04
cg01012082	NCOA2	8	3'UTR	Open sea	40.31	5.89	45.07	7.65	-4.76	3.73E-03	4.35E-02
cg24813588	SAMD10	20	3'UTR	S_Shore	59.43	4.83	64.19	4.83	-4.76	2.94E-05	9.79E-04
cg06573088	CAP2	6	Body	Open sea	36.19	5.36	40.95	5.69	-4.76	4.30E-04	9.02E-03
cg16694007		17	Intergenic	Island	54.03	4.33	58.79	4.26	-4.76	1.33E-05	4.86E-04
cg14131824	SLC43A2	17	Body	N_Shore	53.51	5.13	58.27	5.61	-4.76	1.49E-05	5.37E-04
cg18332838		8	Intergenic	Open sea	66.51	5.80	71.27	5.01	-4.76	1.36E-04	3.61E-03
cg07116631		16	Intergenic	Island	48.02	4.27	52.78	4.54	-4.76	1.52E-05	5.49E-04
cg09015774	CAMTA1	1	Body	Island	36.97	5.64	41.73	6.44	-4.76	1.04E-06	4.64E-05
cg00140335	GPR176	15	Body	Open sea	80.29	5.59	85.05	4.75	-4.76	4.59E-04	9.48E-03
cg21911007	POLH	6	5'UTR	Island	73.71	5.78	78.46	5.34	-4.76	9.30E-05	2.64E-03
cg11261691	KIF3B	20	Body	Open sea	46.66	5.38	51.42	6.63	-4.76	1.50E-03	2.30E-02
cg19287858	ZBTB44	11	5'UTR	N_Shelf	62.64	5.49	67.40	4.89	-4.76	4.23E-05	1.34E-03
cg10551809		13	Intergenic	Open sea	54.83	3.67	59.58	3.98	-4.76	1.09E-06	4.88E-05
cg18288967	PRDX1;PRDX1;PRDX1	1	TSS200;TSS200;TSS200	Island	37.99	4.58	42.75	4.19	-4.76	1.24E-05	4.58E-04
cg00222472	PBX1	1	Body	Island	54.84	5.70	59.60	5.01	-4.76	3.41E-04	7.53E-03
cg20513447	ST6GAL2;ST6GAL2;ST6GAL2	2	Body;Body;Body	Island	80.33	7.89	85.08	7.09	-4.75	7.84E-04	1.43E-02
cg09607679	PRR15L	17	TSS200	Open sea	43.21	4.18	47.96	6.52	-4.75	3.65E-04	7.93E-03
cg16618260	BCL2L12;BCL2L12	19	3'UTR;3'UTR	N_Shelf	73.01	4.34	77.76	4.12	-4.75	8.89E-06	3.39E-04
cg25693132	GRM2;GRM2	3	TSS1500;TSS1500	Island	43.03	5.28	47.78	5.47	-4.75	7.10E-05	2.10E-03
cg25972943	MAGI2	7	Body	Open sea	64.31	4.71	69.06	5.57	-4.75	4.24E-05	1.35E-03
cg25344672	C9orf171	9	TSS1500	N_Shore	47.68	4.55	52.43	4.95	-4.75	8.40E-06	3.22E-04
cg23245007		3	Intergenic	S_Shore	47.56	6.19	52.32	5.91	-4.75	1.21E-04	3.28E-03
cg26870865	TFDP1;TFDP1	13	Body;Body	S_Shore	77.04	3.94	81.79	2.89	-4.75	2.98E-08	1.54E-06
cg17239057		1	Intergenic	Open sea	60.26	5.85	65.01	5.80	-4.75	5.72E-04	1.12E-02
cg09217898		3	Intergenic	S_Shelf	46.55	7.77	51.30	5.89	-4.75	1.96E-03	2.79E-02
cg20322977	CYP26C1	10	1stExon	Island	37.23	5.04	41.98	6.43	-4.75	4.66E-04	9.59E-03
cg20219911	ITIH1;ITIH1;ITIH1;ITIH1	3	TSS1500;Body;TSS1500;Body	Open sea	46.00	4.26	50.75	5.09	-4.75	1.48E-04	3.87E-03
cg24354784		6	Intergenic	S_Shelf	67.50	4.95	72.25	5.54	-4.75	1.89E-05	6.64E-04
cg21272713		12	Intergenic	N_Shelf	59.02	5.84	63.76	5.68	-4.75	4.20E-04	8.85E-03
cg10122865	OTX1	2	3'UTR	Island	32.58	5.13	37.33	5.59	-4.75	1.07E-05	4.01E-04
cg07897069		5	Intergenic	N_Shore	60.52	5.68	65.27	6.42	-4.75	1.40E-03	2.19E-02

cg02203380	C7orf41	7	TSS1500	N_Shore	18.11	4.65	22.86	5.25	-4.75	1.13E-04	3.09E-03
cg25705486	LOC100192426;PTPRM;PTPRM	18	TSS1500;Body;Body	Island	11.99	4.17	16.74	6.01	-4.75	5.49E-06	2.18E-04
cg02457328		12	Intergenic	S_Shelf	11.57	3.72	16.32	3.33	-4.75	2.32E-09	1.28E-07
cg14128315	CTBP1;CTBP1	4	Body;Body	N_Shelf	43.61	3.44	48.36	4.49	-4.75	6.25E-07	2.88E-05
cg18184910	GPR111	6	TSS200	Open sea	64.88	6.14	69.63	6.80	-4.75	3.53E-03	4.19E-02
cg07102913	PCDHB13	5	TSS200	N_Shore	17.61	7.00	22.35	7.88	-4.75	4.43E-03	4.89E-02
cg07707891	MRPS6;SLC5A3	21	Body;3'UTR	Open sea	47.40	5.07	52.14	5.79	-4.75	7.46E-04	1.38E-02
cg20732881		4	Intergenic	Island	87.68	8.19	92.43	4.98	-4.75	5.90E-04	1.15E-02
cg11887733		3	Intergenic	Open sea	75.21	6.15	79.96	3.81	-4.75	4.12E-04	8.72E-03
cg04881472	MRPL23	11	Body	N_Shore	68.61	4.49	73.35	4.76	-4.75	4.53E-05	1.43E-03
cg00329052		4	Intergenic	S_Shelf	81.20	4.77	85.95	3.02	-4.75	2.92E-06	1.21E-04
cg04724319	ABR;ABR;ABR	17	Body;Body;Body	N_Shelf	39.35	5.59	44.10	6.02	-4.75	5.40E-04	1.08E-02
cg13655242	KCNIP4;KCNIP4;KCNIP4;KCNIP4	4	Body;Body;5'UTR;5'UTR	Open sea	40.60	5.72	45.35	5.76	-4.75	1.73E-04	4.40E-03
cg15828524	CACNB3	12	TSS1500	N_Shore	69.68	5.64	74.43	7.30	-4.75	1.05E-03	1.77E-02
cg10124710		1	Intergenic	Island	30.81	3.90	35.55	4.42	-4.74	1.28E-07	6.34E-06
cg07356534	KIRREL	1	Body	Open sea	44.59	4.20	49.33	5.83	-4.74	3.73E-05	1.20E-03
cg13401823	NFASC;NFASC;NFASC;NFASC;NFASC	1	5'UTR;5'UTR;5'UTR;5'UTR;5'UTR	Open sea	35.91	4.71	40.66	4.49	-4.74	2.64E-05	8.94E-04
cg03744383	CES1;CES1;CES1;CES1;CES1;CES1	16	1stExon;5'UTR;5'UTR;1stExon;1stExon;5'U	S_Shore	14.38	4.71	19.13	6.87	-4.74	8.83E-04	1.56E-02
cg00680551	NCALD;NCALD;NCALD;NCALD;NCALD;	8	5'UTR;5'UTR;5'UTR;5'UTR;5'UTR;5'UTR;5'U	N_Shore	22.94	5.48	27.68	5.86	-4.74	2.48E-04	5.87E-03
cg11420644		15	Intergenic	Open sea	84.43	3.89	89.17	2.58	-4.74	3.30E-09	1.80E-07
cg24795274		15	Intergenic	Open sea	55.73	5.35	60.47	6.60	-4.74	2.71E-04	6.28E-03
cg09187338		7	Intergenic	Open sea	38.98	6.53	43.73	8.14	-4.74	2.70E-04	6.27E-03
cg11587957		17	Intergenic	Open sea	62.02	5.56	66.77	6.92	-4.74	5.22E-04	1.05E-02
cg14552982	EXOSC2	9	TSS1500	N_Shore	50.07	6.19	54.82	6.91	-4.74	1.64E-03	2.45E-02
cg04124740	PRKCA	17	Body	Open sea	32.66	6.27	37.40	6.05	-4.74	1.37E-04	3.63E-03
cg25795258		17	Intergenic	Open sea	63.29	4.73	68.03	4.13	-4.74	8.92E-06	3.39E-04
cg01061391		13	Intergenic	N_Shore	55.63	6.49	60.38	4.76	-4.74	2.78E-04	6.42E-03
cg25592907	DNAJB13	11	Body	Open sea	44.55	5.77	49.29	7.26	-4.74	2.92E-03	3.68E-02
cg14601053	FAM129B;FAM129B	9	Body;Body	S_Shore	60.26	5.26	65.00	5.44	-4.74	3.59E-04	7.84E-03
cg14832378		14	Intergenic	Open sea	66.18	5.45	70.93	6.16	-4.74	2.74E-04	6.33E-03
cg13702235	FNIP2	4	TSS1500	N_Shore	50.05	4.99	54.79	6.58	-4.74	8.83E-04	1.56E-02
cg03780997	CCHCR1;CCHCR1;CCHCR1	6	3'UTR;3'UTR;3'UTR	Open sea	67.41	4.43	72.15	3.76	-4.74	6.02E-06	2.37E-04
cg19106893	PTPN23	3	Body	S_Shelf	75.60	4.18	80.34	4.08	-4.74	7.30E-07	3.34E-05
cg10872457		12	Intergenic	Open sea	75.47	5.69	80.21	4.71	-4.74	2.86E-04	6.55E-03
cg13615367	KCTD6	3	5'UTR	S_Shore	40.03	5.51	44.77	6.97	-4.74	1.86E-03	2.69E-02
cg22444116		7	Intergenic	Open sea	72.90	6.49	77.64	5.00	-4.74	6.40E-04	1.23E-02
cg20866121		7	Intergenic	Open sea	31.62	7.00	36.36	7.92	-4.74	3.45E-03	4.12E-02
cg03837903	GNAS;GNAS;GNAS;GNAS	20	3'UTR;TSS200;3'UTR;Body	Island	45.26	6.49	50.00	6.93	-4.74	7.06E-04	1.32E-02
cg03293507	RNF39;RNF39	6	Body;Body	Island	51.93	4.28	56.67	6.62	-4.74	7.59E-05	2.22E-03
cg06494844	ENGASE	17	Body	Open sea	77.03	4.17	81.77	3.41	-4.74	5.60E-07	2.60E-05
cg21821108	ARHGEF7;ARHGEF7;ARHGEF7;ARHGE	13	Body;Body;Body;Body;Body	Open sea	64.93	5.01	69.67	4.93	-4.74	1.52E-05	5.49E-04
cg11856487	LEPRE1;LEPRE1	1	Body;Body	Open sea	67.83	4.68	72.57	4.15	-4.74	1.72E-05	6.12E-04
cg06920037		12	Intergenic	S_Shore	19.95	5.54	24.69	5.93	-4.74	6.99E-05	2.07E-03
cg16475558	GNAO1	16	Body	Open sea	70.01	7.14	74.75	7.10	-4.74	2.84E-03	3.61E-02
cg16619991	ITGA1;PELO	5	Body;Body	S_Shore	48.46	8.72	53.19	7.79	-4.74	4.15E-03	4.68E-02
cg17590003		17	Intergenic	Island	48.42	6.02	53.16	8.06	-4.74	4.15E-04	8.77E-03
cg14766448		3	Intergenic	S_Shelf	68.85	5.91	73.59	6.04	-4.74	2.74E-04	6.33E-03
cg01099300		10	Intergenic	Open sea	74.17	5.73	78.91	4.21	-4.74	3.94E-06	1.61E-04
cg26576047	PLXNA4	7	Body	Open sea	67.92	5.09	72.65	5.49	-4.74	3.39E-05	1.11E-03
cg20411935		1	Intergenic	S_Shore	25.40	6.01	30.14	6.52	-4.74	1.19E-03	1.95E-02
cg00643392	RTBDN	19	TSS1500	S_Shore	32.17	4.55	36.91	5.15	-4.74	1.64E-05	5.86E-04
cg07208114	MLXIP	12	Body	Open sea	57.98	6.54	62.72	4.86	-4.74	1.52E-03	2.33E-02
cg03730533	SKAP2	7	Body	Open sea	21.84	4.82	26.57	5.17	-4.73	1.14E-05	4.22E-04

cg20731875	HS3ST3B1;MGC12916	17	Body;Body	Island	26.63	5.63	31.37	6.66	-4.73	3.56E-03	4.21E-02
cg08855938		1	Intergenic	Open sea	37.49	7.02	42.22	7.03	-4.73	7.66E-04	1.40E-02
cg14495033	EHMT1	9	Body	N_Shore	64.30	6.93	69.03	5.25	-4.73	5.56E-04	1.10E-02
cg23493867	SYNPO2;SYNPO2;SYNPO2	4	Body;Body;Body	Open sea	61.97	5.32	66.70	5.50	-4.73	2.42E-06	1.02E-04
cg18220920		12	Intergenic	S_Shore	41.68	6.00	46.41	4.79	-4.73	1.16E-04	3.16E-03
cg23444677		15	Intergenic	Open sea	67.19	5.87	71.93	4.91	-4.73	1.71E-06	7.40E-05
cg22849665	PRRT1	6	Body	Island	38.71	5.31	43.44	5.43	-4.73	8.30E-04	1.49E-02
cg25521481	TTBK1	6	5'UTR	Island	29.01	3.50	33.75	3.96	-4.73	1.49E-07	7.32E-06
cg07869830	NEURL	10	TSS1500	N_Shore	56.67	3.22	61.41	4.92	-4.73	1.13E-06	5.04E-05
cg17078326	STX18	4	Body	Open sea	65.78	4.83	70.51	3.25	-4.73	5.62E-07	2.61E-05
cg00593607		12	Intergenic	Open sea	36.86	5.50	41.59	5.80	-4.73	1.21E-03	1.98E-02
cg07582661	TMEM59	1	Body	N_Shelf	76.52	5.18	81.25	3.28	-4.73	2.19E-06	9.33E-05
cg00259472	FABP6;FABP6	5	5'UTR;TSS1500	Open sea	55.12	5.84	59.85	6.82	-4.73	4.24E-03	4.75E-02
cg13771161	SLC35D2	9	TSS1500	S_Shore	33.44	5.05	38.17	5.78	-4.73	4.36E-04	9.12E-03
cg10471574	SOX9	17	Body	Island	52.42	6.14	57.15	5.72	-4.73	6.87E-04	1.29E-02
cg14381712	VPS13D;VPS13D	1	Body;Body	Open sea	26.47	3.99	31.19	4.54	-4.73	3.99E-06	1.63E-04
cg24052731		12	Intergenic	Open sea	69.05	5.68	73.78	5.93	-4.73	2.71E-04	6.28E-03
cg11251498	IDO2	8	Body	Open sea	67.04	4.58	71.76	5.12	-4.73	1.18E-05	4.37E-04
cg12200716		11	Intergenic	Open sea	71.97	4.83	76.70	4.92	-4.73	2.18E-06	9.28E-05
cg12052789	EFNA1;EFNA1	1	Body;Body	N_Shelf	53.34	5.00	58.07	5.83	-4.73	3.71E-04	8.03E-03
cg14582550	C9orf3	9	Body	Open sea	24.79	5.54	29.51	6.74	-4.73	3.58E-03	4.23E-02
cg04400131	CACHD1	1	Body	Open sea	67.53	7.68	72.26	7.26	-4.73	9.30E-04	1.62E-02
cg05663480	NFASC;NFASC;NFASC;NFASC;NFASC	1	5'UTR;5'UTR;5'UTR;5'UTR;5'UTR	Open sea	55.64	5.88	60.36	5.99	-4.73	1.60E-03	2.41E-02
cg07581680		2	Intergenic	Open sea	49.35	5.71	54.08	7.20	-4.73	1.29E-03	2.07E-02
cg08276542	U2AF1;U2AF1;U2AF1	21	5'UTR;Body;Body	N_Shore	58.33	4.47	63.06	4.55	-4.72	1.45E-06	6.37E-05
cg26131286		6	Intergenic	Island	41.72	6.15	46.45	5.57	-4.72	7.22E-04	1.34E-02
cg13106472		14	Intergenic	Open sea	74.68	5.29	79.40	5.55	-4.72	8.30E-05	2.39E-03
cg00003014	BDH1;BDH1;BDH1	3	5'UTR;5'UTR;5'UTR	N_Shore	55.76	5.59	60.49	6.23	-4.72	3.92E-03	4.50E-02
cg18885766	CCDC85C	14	Body	Open sea	43.12	4.28	47.85	4.68	-4.72	2.05E-05	7.13E-04
cg26571535	SNX27	1	Body	Open sea	74.65	6.94	79.37	5.34	-4.72	2.01E-03	2.84E-02
cg00821400	TBPL2	14	Body	Open sea	73.22	4.93	77.95	4.31	-4.72	2.67E-06	1.12E-04
cg23550060	NAB2	12	Body	S_Shore	36.29	4.20	41.01	4.98	-4.72	8.34E-05	2.40E-03
cg22638766	MIR25;MCM7;MCM7;MIR93	7	TSS1500;Body;Body;TSS200	Open sea	56.90	5.32	61.62	4.59	-4.72	1.95E-04	4.84E-03
cg20885815	SLC30A3	2	Body	Open sea	40.83	4.57	45.56	5.31	-4.72	1.58E-04	4.08E-03
cg23019889		1	Intergenic	Island	64.31	6.75	69.03	4.73	-4.72	1.82E-04	4.58E-03
cg23173806	TRIM39;TRIM39	6	Body;Body	Open sea	70.02	6.15	74.75	5.86	-4.72	2.08E-03	2.91E-02
cg06547984		19	Intergenic	Island	36.66	4.23	41.39	4.79	-4.72	2.90E-05	9.67E-04
cg23441641	TMX4	20	3'UTR	Open sea	49.08	5.79	53.81	5.85	-4.72	1.53E-03	2.34E-02
cg26156279	FBXW4	10	Body	Open sea	75.53	3.89	80.25	3.57	-4.72	2.82E-07	1.36E-05
cg10902738		8	Intergenic	Open sea	55.26	6.09	59.98	6.36	-4.72	1.88E-04	4.70E-03
cg08649068	RARA;RARA;RARA;RARA	17	Body;Body;Body;Body	Open sea	44.41	4.14	49.13	4.37	-4.72	2.56E-05	8.70E-04
cg26853265	PGLYRP2	19	Body	Island	76.51	6.01	81.23	4.85	-4.72	2.08E-04	5.09E-03
cg01925738	PCDHB3	5	1stExon	N_Shore	29.01	5.90	33.73	7.35	-4.72	2.20E-03	3.02E-02
cg15487251	IGF2BP2;IGF2BP2	3	TSS1500;TSS1500	S_Shore	66.38	4.45	71.10	3.66	-4.72	9.44E-07	4.25E-05
cg09287629		2	Intergenic	Island	44.90	5.17	49.62	5.55	-4.72	2.93E-05	9.78E-04
cg22501143	HP1BP3	1	5'UTR	N_Shelf	53.26	6.26	57.98	6.27	-4.72	1.03E-03	1.75E-02
cg12619262		7	Intergenic	Open sea	59.74	5.93	64.46	5.87	-4.72	4.69E-04	9.64E-03
cg26512045	CCHCR1;CCHCR1;CCHCR1	6	Body;Body;Body	Open sea	50.16	4.77	54.88	4.86	-4.72	8.22E-05	2.37E-03
cg18118490	SAMD4A;SAMD4A;SAMD4A	14	Body;Body;Body	Open sea	29.59	5.41	34.31	5.36	-4.72	1.11E-05	4.12E-04
cg25372085		10	Intergenic	Open sea	54.19	7.93	58.91	5.69	-4.72	7.67E-04	1.40E-02
cg03502812	LRFN3	19	Body	Island	71.33	5.37	76.04	5.29	-4.72	3.57E-04	7.79E-03
cg16736889	ACSM5	16	5'UTR	Open sea	28.74	4.43	33.45	4.72	-4.72	2.40E-05	8.22E-04
cg04428346	SEC24D	4	Body	Open sea	72.30	5.68	77.01	3.89	-4.72	2.68E-04	6.23E-03

cg06623017		15	Intergenic	Open sea	41.44	4.96	46.15	4.22	-4.72	1.26E-05	4.63E-04
cg19707853		15	Intergenic	S_Shore	27.74	7.26	32.46	6.66	-4.72	5.80E-04	1.14E-02
cg22306173	WNT7B	22	Body	Island	10.53	3.32	15.24	7.04	-4.71	1.59E-04	4.11E-03
cg06094607	ZC3H3	8	Body	N_Shelf	70.07	3.92	74.78	3.31	-4.71	2.00E-07	9.72E-06
cg17533797	C7orf50;C7orf50;C7orf50	7	Body;Body;Body	Island	83.79	7.29	88.51	5.27	-4.71	1.72E-03	2.53E-02
cg14610403	SGIP1	1	TSS1500	Island	35.10	4.61	39.81	5.73	-4.71	2.04E-06	8.72E-05
cg04864807		2	Intergenic	Island	30.46	6.94	35.17	6.70	-4.71	1.26E-03	2.03E-02
cg05203213		2	Intergenic	Open sea	35.56	5.23	40.28	5.47	-4.71	3.72E-05	1.20E-03
cg19614811	GPR15	3	TSS200	Open sea	79.65	4.94	84.36	4.28	-4.71	1.31E-06	5.79E-05
cg03466326		1	Intergenic	Open sea	45.17	5.35	49.89	5.46	-4.71	1.44E-05	5.22E-04
cg12812834	OR51S1	11	1stExon	Open sea	68.27	7.11	72.98	5.58	-4.71	1.13E-03	1.87E-02
cg23367341	STK10	5	Body	Open sea	59.60	5.49	64.32	5.47	-4.71	1.47E-04	3.85E-03
cg04102163		4	Intergenic	Island	48.16	6.05	52.87	6.16	-4.71	1.20E-04	3.26E-03
cg11362513	EEF1D;EEF1D;EEF1D;EEF1D;EEF1D;EEF	8	Body;Body;Body;Body;Body;Body;Body	Open sea	79.72	5.62	84.44	5.82	-4.71	1.40E-04	3.69E-03
cg06531430		16	Intergenic	S_Shelf	59.91	7.35	64.63	7.10	-4.71	2.96E-03	3.71E-02
cg11019430		10	Intergenic	Open sea	11.27	3.93	15.98	5.89	-4.71	3.64E-05	1.18E-03
cg23646044	EFTUD2;EFTUD2	17	Body;Body	Open sea	77.22	4.85	81.93	3.91	-4.71	3.84E-05	1.24E-03
cg12861170	YJEFN3	19	TSS200	Island	61.86	6.70	66.57	4.94	-4.71	3.26E-03	3.96E-02
cg02564259	SLC12A4;SLC12A4;SLC12A4;SLC12A4;S	16	3'UTR;3'UTR;3'UTR;3'UTR;3'UTR;TSS1500	Open sea	41.96	4.14	46.67	5.38	-4.71	1.04E-04	2.90E-03
cg23273897	MME;MME;MME;MME	3	TSS1500;TSS1500;TSS1500;TSS1500	N_Shore	48.62	5.61	53.33	5.47	-4.71	2.01E-05	7.02E-04
cg12148647	PIP5K1C	19	Body	Island	45.68	5.56	50.39	4.12	-4.71	1.72E-05	6.10E-04
cg22293481	SEMA4B;SEMA4B	15	Body;Body	Island	24.64	6.31	29.35	7.42	-4.71	7.86E-04	1.43E-02
cg23145336	TNXB	6	Body	N_Shelf	29.43	4.38	34.14	6.02	-4.71	1.92E-04	4.79E-03
cg26624055		12	Intergenic	Open sea	88.38	6.05	93.09	1.59	-4.71	1.06E-06	4.75E-05
cg13622821	CLIC1	6	Body	Open sea	28.20	8.15	32.91	7.99	-4.71	3.78E-04	8.15E-03
cg15066489	SLC15A2;SLC15A2	3	1stExon;1stExon	Open sea	57.60	4.65	62.31	4.64	-4.71	6.18E-07	2.85E-05
cg00456892	ESAM	11	Body	Island	18.89	3.72	23.60	5.86	-4.71	6.58E-05	1.97E-03
cg25759916	AGRN	1	Body	Island	12.69	3.24	17.40	6.51	-4.71	3.25E-05	1.07E-03
cg06106484		8	Intergenic	Open sea	34.91	4.87	39.62	6.03	-4.71	3.67E-04	7.97E-03
cg23921459	YOD1	1	TSS200	N_Shore	20.45	5.97	25.16	7.71	-4.71	2.55E-03	3.35E-02
cg12368612		12	Intergenic	Island	17.61	4.39	22.32	4.15	-4.71	7.76E-08	3.90E-06
cg17178468		17	Intergenic	S_Shore	49.82	4.26	54.53	4.55	-4.71	1.02E-05	3.85E-04
cg12559119		17	Intergenic	Open sea	44.47	4.62	49.18	5.63	-4.71	2.16E-04	5.25E-03
cg21772509	NKX6-3	8	3'UTR	N_Shore	18.90	3.98	23.61	4.35	-4.71	1.15E-07	5.70E-06
cg11876912	SYNPR;SYNPR	3	3'UTR;3'UTR	Open sea	45.82	5.51	50.52	7.29	-4.71	2.69E-03	3.47E-02
cg06756436	CLUL1;CLUL1	18	TSS1500;5'UTR	Open sea	70.82	4.93	75.53	4.32	-4.71	1.19E-05	4.40E-04
cg21188610	FNBP1	9	Body	Open sea	47.93	4.48	52.63	4.70	-4.71	7.99E-06	3.07E-04
cg11012584	THAP4;THAP4	2	Body;Body	Island	14.58	3.96	19.29	4.80	-4.71	1.68E-07	8.21E-06
cg10110933	DUSP13;DUSP13;DUSP13	10	Body;Body;5'UTR	N_Shelf	54.48	4.09	59.19	4.28	-4.71	1.78E-05	6.29E-04
cg20592700	WIPI2;WIPI2;WIPI2;WIPI2	7	1stExon;1stExon;1stExon;1stExon	Island	17.78	4.01	22.48	4.15	-4.71	6.04E-06	2.38E-04
cg02045948		3	Intergenic	N_Shore	26.85	4.69	31.56	4.12	-4.71	2.17E-05	7.51E-04
cg22958047		15	Intergenic	N_Shore	31.54	4.77	36.24	7.67	-4.71	1.09E-03	1.82E-02
cg25268435	NFE2L2;NFE2L2;NFE2L2	2	Body;5'UTR;5'UTR	Open sea	59.98	5.78	64.68	5.76	-4.71	1.30E-03	2.08E-02
cg24441810	TMEM177;TMEM177;TMEM177	2	TSS1500;TSS1500;TSS1500	N_Shore	21.88	4.90	26.59	6.10	-4.70	1.52E-04	3.95E-03
cg10533069		14	Intergenic	Open sea	65.44	6.08	70.14	5.66	-4.70	1.01E-03	1.73E-02
cg24578857	PLD6	17	TSS1500	Island	27.52	5.13	32.23	7.57	-4.70	3.04E-03	3.78E-02
cg16869862	ACOX2	3	Body	Open sea	64.21	4.60	68.91	5.47	-4.70	6.73E-05	2.01E-03
cg06712204		16	Intergenic	Open sea	67.05	5.21	71.76	3.79	-4.70	2.58E-06	1.08E-04
cg10832281	PAMR1;PAMR1	11	Body;Body	Open sea	44.51	5.25	49.21	6.71	-4.70	9.77E-04	1.68E-02
cg03360992	TRIOBP	22	5'UTR	Open sea	37.33	5.89	42.03	5.84	-4.70	1.25E-04	3.37E-03
cg23977670	H19	11	Body	N_Shore	61.73	4.92	66.43	4.13	-4.70	4.42E-05	1.40E-03
cg04629424	LRRC23;LRRC23;LRRC23	12	Body;Body;Body	N_Shelf	68.10	4.54	72.80	4.70	-4.70	1.88E-07	9.19E-06
cg07836815	LIMD2	17	TSS1500	Island	40.52	3.92	45.22	3.87	-4.70	3.58E-07	1.70E-05

cg11209447	SCNN1A;SCNN1A;SCNN1A	12	Body;TSS200;5'UTR	Open sea	26.26	4.42	30.96	6.15	-4.70	1.68E-05	5.97E-04
cg22389654	PHGDH	1	Body	Open sea	63.87	4.92	68.57	6.48	-4.70	2.83E-06	1.18E-04
cg02844984	EIF3K	19	Body	S_Shelf	81.06	3.73	85.76	2.62	-4.70	9.63E-09	5.13E-07
cg26186239	EEF1A2	20	3'UTR	Island	16.81	3.84	21.51	7.77	-4.70	9.48E-04	1.65E-02
cg23334662	RFTN2;RFTN2	2	1stExon;5'UTR	Open sea	43.62	6.76	48.32	5.10	-4.70	7.04E-04	1.32E-02
cg13849647	DCAF12	9	Body	N_Shelf	50.88	6.18	55.58	6.71	-4.70	5.19E-04	1.04E-02
cg16658255		5	Intergenic	N_Shelf	48.45	5.73	53.15	7.31	-4.70	1.36E-03	2.14E-02
cg22857356	KANK3	19	Body	Island	50.76	4.53	55.46	4.27	-4.70	1.07E-05	4.01E-04
cg03032025	CPEB4	5	1stExon	S_Shore	32.43	5.25	37.13	6.54	-4.70	2.68E-03	3.46E-02
cg26654286	RARA;RARA	17	5'UTR;1stExon	Open sea	19.62	5.59	24.31	4.90	-4.70	3.15E-06	1.30E-04
cg23204968	HOXA5	7	TSS1500	Island	36.78	5.37	41.48	6.15	-4.70	1.18E-03	1.94E-02
cg03801871	LBH	2	TSS1500	N_Shore	41.65	5.32	46.35	6.30	-4.70	2.76E-04	6.38E-03
cg11063110	CPA5;CPA5;CPA5	7	5'UTR;5'UTR;5'UTR	Open sea	40.19	4.88	44.89	6.46	-4.70	1.88E-03	2.71E-02
cg04123507	KRT86	12	TSS1500	N_Shore	79.23	5.55	83.92	6.45	-4.70	7.13E-04	1.33E-02
cg01168822	LYPLA2P1	6	TSS1500	Open sea	56.07	5.72	60.77	6.28	-4.70	9.41E-04	1.64E-02
cg13472610		21	Intergenic	N_Shelf	72.51	4.16	77.21	4.82	-4.70	1.57E-06	6.86E-05
cg09838217	GFPT2	5	Body	Open sea	45.11	6.08	49.81	6.36	-4.70	7.18E-04	1.34E-02
cg13577194	IPPK	9	Body	Open sea	65.42	6.10	70.11	5.76	-4.70	2.95E-04	6.71E-03
cg26475094	MIR138-2	16	TSS1500	Open sea	61.28	5.40	65.98	5.41	-4.70	2.60E-04	6.08E-03
cg02502704		10	Intergenic	N_Shelf	34.58	4.58	39.28	6.28	-4.70	9.65E-04	1.67E-02
cg04085909		10	Intergenic	Island	74.68	7.66	79.38	6.36	-4.70	3.76E-03	4.37E-02
cg09430647		10	Intergenic	Open sea	55.72	7.14	60.42	7.00	-4.70	2.65E-03	3.44E-02
cg14417974	GSN;GSN;GSN;GSN;GSN;GSN;GSN	9	5'UTR;5'UTR;5'UTR;5'UTR;5'UTR;5'UTR;5'U	N_Shelf	48.15	5.07	52.85	5.56	-4.70	6.44E-04	1.23E-02
cg06070324	PPCDC	15	Body	Open sea	35.83	6.01	40.52	5.61	-4.69	1.25E-03	2.02E-02
cg26052885	PRKAA2	1	3'UTR	Open sea	71.72	5.05	76.42	5.45	-4.69	2.06E-04	5.05E-03
cg08857729	TCTEX1D1	1	TSS1500	N_Shore	43.92	4.93	48.61	4.69	-4.69	3.81E-06	1.56E-04
cg15587571	UBE2E3;UBE2E3	2	Body;Body	S_Shelf	71.89	4.58	76.59	4.21	-4.69	4.13E-06	1.68E-04
cg07283441	PVT1	8	Body	Open sea	36.22	6.39	40.91	5.27	-4.69	2.65E-04	6.18E-03
cg09406615	BCHE;BCHE	3	1stExon;5'UTR	Open sea	27.71	5.77	32.40	7.68	-4.69	1.64E-03	2.46E-02
cg14870156	HLA-DPB1	6	Body	Island	23.07	5.31	27.76	5.73	-4.69	4.81E-04	9.82E-03
cg11858733	DHH	12	TSS1500	S_Shore	73.67	4.97	78.36	4.50	-4.69	3.18E-05	1.05E-03
cg25382764		7	Intergenic	Open sea	62.46	5.15	67.16	5.09	-4.69	8.38E-05	2.41E-03
cg00178984	SLC16A7	12	5'UTR	Open sea	71.29	6.97	75.99	5.82	-4.69	6.39E-04	1.22E-02
cg19802639	CBX6	22	Body	Island	66.50	7.11	71.19	6.33	-4.69	3.72E-03	4.34E-02
cg05304405	FSTL3	19	TSS1500	Island	38.46	6.59	43.16	4.18	-4.69	1.46E-04	3.83E-03
cg27628340	TG	8	Body	Open sea	46.77	4.87	51.46	5.68	-4.69	2.94E-04	6.69E-03
cg01052294		19	Intergenic	Island	36.10	4.77	40.79	4.99	-4.69	8.35E-05	2.41E-03
cg24027742	SYVN1;SYVN1	11	3'UTR;3'UTR	Open sea	65.96	4.01	70.65	3.87	-4.69	9.60E-07	4.32E-05
cg00094595		12	Intergenic	Open sea	27.70	6.45	32.39	7.75	-4.69	3.67E-04	7.97E-03
cg06367788		11	Intergenic	N_Shore	38.50	5.26	43.19	5.25	-4.69	1.51E-04	3.94E-03
cg00731185		7	Intergenic	Island	26.23	4.88	30.92	8.51	-4.69	1.30E-04	3.48E-03
cg01810111		6	Intergenic	Open sea	33.07	6.53	37.76	7.36	-4.69	1.68E-05	5.98E-04
cg18009021	PDLIM5;PDLIM5;PDLIM5;PDLIM5;PDL	4	Body;Body;Body;Body;Body	S_Shelf	39.08	4.65	43.77	4.77	-4.69	7.17E-05	2.12E-03
cg24722070		1	Intergenic	Open sea	62.58	6.07	67.26	6.13	-4.69	8.01E-04	1.45E-02
cg06662140	KLHDC7A	1	1stExon	Island	91.07	6.13	95.76	3.06	-4.69	2.34E-05	8.04E-04
cg02859421		19	Intergenic	S_Shore	50.99	5.13	55.68	6.68	-4.69	2.32E-03	3.13E-02
cg19234495	C1orf210;C1orf210	1	Body;Body	Open sea	74.17	5.03	78.86	4.56	-4.69	3.63E-07	1.72E-05
cg06233202		2	Intergenic	Open sea	25.81	6.48	30.50	7.11	-4.69	7.46E-05	2.19E-03
cg23370883	MYL7	7	Body	Open sea	61.05	5.28	65.74	5.36	-4.69	2.18E-04	5.28E-03
cg01622718	PTPRN2;PTPRN2;PTPRN2	7	Body;Body;Body	S_Shore	71.87	6.75	76.56	4.33	-4.69	6.62E-04	1.26E-02
cg22791535	ELAVL4;ELAVL4;ELAVL4;ELAVL4;ELAVL	1	3'UTR;3'UTR;3'UTR;3'UTR;3'UTR	Open sea	66.21	6.13	70.89	5.53	-4.69	6.87E-04	1.29E-02
cg27182551	RB1	13	Body	N_Shore	63.39	5.29	68.07	6.39	-4.69	1.03E-03	1.75E-02
cg08559772	ATP5EP2	13	TSS1500	Open sea	45.63	4.89	50.32	6.16	-4.69	1.06E-04	2.94E-03

cg10878307	PRR15L	17	5'UTR	Open sea	50.39	4.07	55.08	2.99	-4.69	8.35E-07	3.79E-05
cg18586212	PPP2R2B;PPP2R2B;PPP2R2B;PPP2R2B	5	Body;Body;Body;Body;Body;Body;Body	Open sea	58.26	5.22	62.95	6.32	-4.69	4.31E-04	9.04E-03
cg03406106	PKD1L2;PKD1L2	16	TSS200;TSS200	Open sea	65.72	5.99	70.41	6.47	-4.69	2.15E-03	2.97E-02
cg17218813	PRRT1;PRRT1	6	1stExon;5'UTR	S_Shore	39.17	6.20	43.86	5.76	-4.68	1.36E-03	2.14E-02
cg26385286	GCNT2	6	5'UTR	Open sea	57.99	6.86	62.67	4.83	-4.68	1.20E-03	1.96E-02
cg13392861		9	Intergenic	Open sea	71.78	4.18	76.47	4.44	-4.68	4.25E-06	1.72E-04
cg17238496	SEC22B	1	Body	Open sea	65.55	4.80	70.24	4.74	-4.68	1.03E-04	2.87E-03
cg12727940	PLSCR4;PLSCR4;PLSCR4;PLSCR4	3	TSS1500;TSS1500;TSS1500;TSS1500	S_Shore	34.50	4.58	39.18	5.17	-4.68	7.42E-05	2.18E-03
cg26523264	ADAMTS10	19	Body	Island	84.31	5.32	88.99	2.79	-4.68	2.14E-06	9.13E-05
cg05405389	DLG2;DLG2	11	Body;Body	Open sea	44.65	4.42	49.34	3.95	-4.68	1.63E-07	8.00E-06
cg19706516		1	Intergenic	Island	19.48	6.00	24.16	6.78	-4.68	1.79E-04	4.52E-03
cg20322299	INSIG2	2	5'UTR	S_Shelf	74.94	6.25	79.62	5.57	-4.68	1.21E-04	3.27E-03
cg16345366		10	Intergenic	Open sea	77.56	7.40	82.24	5.86	-4.68	3.71E-04	8.04E-03
cg09092280		8	Intergenic	Open sea	24.28	5.94	28.96	6.09	-4.68	2.69E-03	3.47E-02
cg11416492		10	Intergenic	Open sea	64.54	6.57	69.21	5.96	-4.68	6.54E-04	1.25E-02
cg08920810	GABRG3	15	TSS1500	N_Shore	70.14	5.35	74.82	5.13	-4.68	2.45E-04	5.81E-03
cg15735157		7	Intergenic	Open sea	50.81	5.74	55.48	5.33	-4.68	2.29E-05	7.88E-04
cg19520372	KIAA1026;KIAA1026;KIAA1026;KIAA1(	1	Body;TSS200;Body;Body	Open sea	63.52	5.31	68.20	5.22	-4.68	3.35E-04	7.42E-03
cg14406859		17	Intergenic	S_Shelf	52.21	7.23	56.89	6.26	-4.68	1.27E-03	2.05E-02
cg12548341		10	Intergenic	S_Shelf	40.44	6.49	45.12	6.21	-4.68	8.00E-05	2.32E-03
cg11145160	CLDN11	3	TSS1500	Island	25.54	4.78	30.22	5.94	-4.68	9.43E-05	2.67E-03
cg00562468		6	Intergenic	Open sea	38.82	4.63	43.50	5.62	-4.68	1.06E-05	3.99E-04
cg14850477	DACH1;DACH1;DACH1	13	Body;Body;Body	Open sea	67.44	5.83	72.11	7.14	-4.68	1.20E-03	1.96E-02
cg21497480	LOC286135	8	Body	Open sea	57.54	4.59	62.21	6.17	-4.68	8.34E-05	2.40E-03
cg23515420		7	Intergenic	S_Shelf	33.11	5.70	37.79	5.36	-4.68	5.23E-04	1.05E-02
cg26037602		11	Intergenic	Open sea	56.33	5.91	61.01	4.54	-4.68	4.01E-05	1.28E-03
cg04319873		2	Intergenic	S_Shore	37.92	5.64	42.60	5.99	-4.68	5.18E-04	1.04E-02
cg16964673	KIAA0562;DFFB	1	TSS1500;Body	Island	57.99	4.61	62.67	5.52	-4.68	1.69E-04	4.32E-03
cg09775533		20	Intergenic	Island	28.29	6.33	32.97	8.51	-4.68	8.20E-04	1.47E-02
cg24940576	CDON	11	5'UTR	Open sea	25.06	5.18	29.74	7.50	-4.67	3.70E-03	4.33E-02
cg02291245	RAB11FIP2	10	Body	Open sea	73.36	5.08	78.04	4.64	-4.67	1.32E-04	3.52E-03
cg18057306	MIPEP	13	Body	Open sea	59.55	3.88	64.22	3.76	-4.67	7.52E-07	3.43E-05
cg08037774	SLC1A7	1	TSS1500	Open sea	76.24	6.01	80.92	4.97	-4.67	7.15E-04	1.33E-02
cg26311262	ARHGEF3	3	Body	Open sea	36.90	5.67	41.57	3.64	-4.67	9.01E-05	2.57E-03
cg06800231	C14orf165	14	Body	Open sea	50.50	6.47	55.17	6.31	-4.67	5.86E-05	1.78E-03
cg05876824		6	Intergenic	Open sea	69.40	4.56	74.08	4.51	-4.67	2.83E-07	1.36E-05
cg14962049	PRELID2;PRELID2;PRELID2	5	Body;Body;Body	Open sea	52.20	5.34	56.87	4.72	-4.67	3.54E-05	1.15E-03
cg04086391	DPP6;DPP6;DPP6	7	3'UTR;3'UTR;3'UTR	Island	74.77	5.80	79.44	5.75	-4.67	7.89E-04	1.43E-02
cg27111150	SORBS1;SORBS1;SORBS1;SORBS1;SOF	10	Body;Body;Body;Body;Body;Body;Body	Open sea	32.63	5.10	37.30	5.60	-4.67	5.16E-04	1.04E-02
cg00911881	GATA4	8	Body	N_Shore	65.16	4.82	69.83	3.25	-4.67	1.41E-05	5.11E-04
cg18740872	FYB;FYB	5	TSS1500;TSS1500	Open sea	59.49	6.10	64.16	4.73	-4.67	5.02E-04	1.02E-02
cg00389785		6	Intergenic	N_Shelf	53.60	5.37	58.28	6.14	-4.67	2.89E-04	6.61E-03
cg04368058	TBKBP1;TBKBP1	17	1stExon;5'UTR	Island	23.25	6.10	27.92	7.44	-4.67	1.01E-04	2.83E-03
cg12238015	PDX1	13	Body	N_Shore	10.74	5.25	15.41	7.26	-4.67	9.95E-04	1.70E-02
cg23132774	RASA3	13	Body	Island	54.23	4.28	58.90	4.88	-4.67	1.13E-05	4.19E-04
cg07732755	RUSC1;RUSC1;RUSC1;C1orf104;RUSC1	1	Body;Body;Body;TSS1500;Body	Island	19.35	4.86	24.02	6.35	-4.67	1.27E-03	2.05E-02
cg00645383		13	Intergenic	Island	78.33	5.64	83.00	4.87	-4.67	4.27E-05	1.36E-03
cg07209141	LRP5	11	Body	Open sea	42.55	4.58	47.22	4.60	-4.67	1.97E-04	4.88E-03
cg00438026	ATP6V1G2;NFKBIL1;NFKBIL1;ATP6V1G	6	Body;TSS1500;TSS1500;Body	S_Shelf	57.03	3.67	61.70	4.22	-4.67	5.58E-06	2.21E-04
cg18151487	SFXN2;ARL3	10	5'UTR;TSS1500	S_Shore	50.77	4.80	55.44	5.64	-4.67	1.00E-04	2.80E-03
cg07061728	RTN4RL1	17	Body	Island	26.63	5.05	31.30	7.72	-4.67	3.70E-03	4.33E-02
cg02388882	AGAP11	10	5'UTR	Island	35.86	5.82	40.53	5.87	-4.67	4.23E-04	8.91E-03
cg11963400	TRIM40	6	Body	Open sea	60.83	5.47	65.50	5.06	-4.67	3.89E-05	1.25E-03

cg04491064		8	Intergenic	S_Shelf	69.13	5.02	73.80	4.88	-4.67	2.03E-04	5.00E-03
cg10854423		11	Intergenic	Open sea	57.86	4.65	62.53	5.86	-4.67	4.92E-05	1.53E-03
cg15535683		7	Intergenic	Open sea	54.19	6.10	58.86	6.29	-4.67	3.06E-04	6.91E-03
cg03669268	CST9L;CST9L	20	1stExon;5'UTR	Open sea	53.12	5.07	57.79	7.02	-4.67	1.88E-03	2.71E-02
cg08111284	TNXB	6	Body	S_Shore	47.53	3.62	52.19	4.20	-4.67	2.33E-07	1.13E-05
cg03028061		8	Intergenic	S_Shore	46.91	5.72	51.57	7.55	-4.67	2.58E-03	3.37E-02
cg10708761	TMEM178;TMEM178	2	TSS1500;TSS200	N_Shore	40.71	5.43	45.38	7.26	-4.67	3.67E-03	4.30E-02
cg15660498	C19orf55	19	Body	S_Shore	48.67	6.37	53.34	6.27	-4.67	3.98E-03	4.55E-02
cg19211851	THADA;THADA	2	Body;Body	Open sea	43.81	4.57	48.47	6.04	-4.67	8.63E-04	1.53E-02
cg02710860	MAPKBP1;MAPKBP1	15	TSS1500;TSS1500	N_Shore	61.72	6.96	66.39	5.88	-4.67	3.92E-03	4.50E-02
cg12438576	NOX4	11	5'UTR	Open sea	68.93	5.96	73.59	5.51	-4.67	2.95E-04	6.71E-03
cg04072545	INS-IGF2;IGF2AS;IGF2;IGF2;IGF2AS;IG	11	Body;TSS1500;Body;5'UTR;TSS1500;TSS15	Island	49.46	5.84	54.12	6.00	-4.67	2.04E-05	7.12E-04
cg16736181		10	Intergenic	Open sea	65.76	3.29	70.43	2.42	-4.67	1.60E-09	8.86E-08
cg06537526		4	Intergenic	S_Shelf	72.14	6.97	76.81	5.78	-4.67	1.68E-03	2.50E-02
cg06638795	KCNG3;KCNG3	2	Body;Body	Island	36.76	6.79	41.43	5.40	-4.67	1.32E-03	2.10E-02
cg13691744	SLC44A4	6	Body	Open sea	44.60	4.44	49.26	4.65	-4.67	7.30E-07	3.34E-05
cg11335335	DRD4	11	Body	Island	46.35	7.48	51.01	8.00	-4.66	2.18E-03	3.00E-02
cg16259229		5	Intergenic	Open sea	20.43	6.31	25.09	6.23	-4.66	6.10E-04	1.18E-02
cg24177393		5	Intergenic	Island	37.87	5.63	42.53	6.64	-4.66	2.19E-03	3.02E-02
cg05667197	PIP	7	TSS1500	Open sea	51.66	5.48	56.33	5.65	-4.66	8.78E-06	3.35E-04
cg00780456	TMEM55A	8	Body	Open sea	73.60	4.99	78.26	5.52	-4.66	3.04E-04	6.87E-03
cg15164881	TSPAN4;TSPAN4;TSPAN4;TSPAN4;TSP	11	5'UTR;Body;Body;Body;Body;Body;Body	Island	70.83	6.84	75.49	6.50	-4.66	7.61E-04	1.40E-02
cg16735881	NBEA	13	Body	Island	16.30	4.48	20.97	7.26	-4.66	1.73E-03	2.55E-02
cg03324851	GNB2	7	Body	S_Shore	51.38	5.09	56.04	5.43	-4.66	6.43E-04	1.23E-02
cg24118850		6	Intergenic	S_Shore	75.79	5.31	80.45	2.83	-4.66	3.81E-06	1.56E-04
cg00257920		1	Intergenic	S_Shelf	64.89	7.14	69.55	8.04	-4.66	2.52E-03	3.32E-02
cg16540262		15	Intergenic	Open sea	41.53	7.31	46.19	6.13	-4.66	1.61E-03	2.42E-02
cg24039780	PBX1	1	Body	Open sea	76.18	4.70	80.84	2.76	-4.66	1.11E-06	4.94E-05
cg25919098	MAD1L1;MAD1L1;MAD1L1	7	Body;Body;Body	Open sea	85.18	6.34	89.84	4.85	-4.66	4.33E-04	9.07E-03
cg02228185	ASPA;ASPA	17	1stExon;Body	Open sea	28.61	4.72	33.27	6.53	-4.66	1.33E-03	2.12E-02
cg05079448	PIWIL2;PIWIL2	8	Body;Body	Open sea	25.81	4.85	30.47	5.70	-4.66	1.03E-03	1.76E-02
cg07341609	CDC42EP1	22	Body	Island	39.68	5.60	44.34	6.16	-4.66	2.35E-05	8.07E-04
cg09748946		7	Intergenic	Island	40.21	5.80	44.86	5.62	-4.66	2.94E-04	6.70E-03
cg16434190	EHD1	11	Body	N_Shelf	61.93	3.98	66.59	4.71	-4.66	1.75E-06	7.59E-05
cg21186576		17	Intergenic	Island	66.54	5.31	71.20	7.46	-4.66	7.97E-04	1.44E-02
cg08159945	HDAC10;HDAC10	22	Body;Body	N_Shelf	55.27	5.20	59.92	4.16	-4.66	1.46E-04	3.83E-03
cg14830002	OR2B11	1	TSS1500	S_Shore	20.84	6.03	25.50	7.03	-4.66	1.76E-03	2.58E-02
cg25833200	NSUN3	3	Body	S_Shelf	61.40	3.98	66.05	3.69	-4.66	6.07E-07	2.80E-05
cg13208003	LHFP	13	Body	Open sea	42.60	5.32	47.26	5.43	-4.66	3.30E-05	1.08E-03
cg20016023	RRP12;RRP12	10	Body;Body	N_Shore	30.03	5.69	34.69	5.01	-4.66	1.93E-04	4.79E-03
cg19991108		10	Intergenic	Open sea	40.07	5.22	44.73	5.56	-4.66	1.46E-04	3.82E-03
cg10935612	UBE2QL1	5	Body	S_Shore	30.39	5.28	35.05	6.00	-4.66	1.58E-05	5.66E-04
cg21447117	HOXA11;HOXA11AS	7	TSS200;TSS200	N_Shore	10.97	3.17	15.62	3.64	-4.66	9.98E-09	5.31E-07
cg25772723	GATA2	3	5'UTR	Island	40.70	3.57	45.35	4.33	-4.65	1.67E-08	8.77E-07
cg03241046	SCN1A;SCN1A;SCN1A	2	Body;Body;Body	Open sea	52.81	5.00	57.46	4.05	-4.65	1.89E-06	8.14E-05
cg19831386	AKT1;AKT1;AKT1	14	3'UTR;3'UTR;3'UTR	N_Shelf	66.74	4.45	71.39	4.63	-4.65	9.18E-06	3.49E-04
cg14171486		1	Intergenic	Open sea	46.18	4.87	50.83	4.24	-4.65	2.83E-05	9.49E-04
cg17278453		7	Intergenic	S_Shore	14.31	5.99	18.96	7.82	-4.65	1.22E-03	1.98E-02
cg27211576	CSK;CSK	15	Body;Body	N_Shelf	62.61	3.79	67.26	3.97	-4.65	1.63E-06	7.08E-05
cg26571870	GINS2	16	TSS1500	S_Shore	52.37	6.52	57.02	6.36	-4.65	1.41E-03	2.21E-02
cg26406074	NBEA	13	Body	N_Shore	28.31	4.81	32.97	7.57	-4.65	2.40E-03	3.21E-02
cg00497232		4	Intergenic	Island	11.41	3.47	16.06	4.69	-4.65	2.01E-06	8.64E-05
cg04768557	ANK3	10	Body	Open sea	68.69	7.29	73.35	5.40	-4.65	3.97E-04	8.47E-03

cg14228614	FAM110B	8	5'UTR	S_Shore	26.02	5.98	30.67	6.84	-4.65	1.55E-03	2.36E-02
cg11458498	SLIT3	5	Body	Open sea	46.35	5.29	51.00	7.43	-4.65	1.26E-03	2.04E-02
cg08009313	ATP2C1;ATP2C1;ATP2C1;ATP2C1	3	Body;Body;Body;Body	Open sea	37.37	3.37	42.02	2.90	-4.65	6.03E-09	3.25E-07
cg02629586	RDH16	12	3'UTR	Open sea	61.20	5.47	65.85	4.92	-4.65	2.41E-05	8.26E-04
cg13938187	OAZ2	15	3'UTR	Open sea	62.65	4.19	67.30	3.37	-4.65	1.48E-06	6.48E-05
cg06666025	CMTM2	16	TSS200	Island	22.10	4.80	26.75	5.72	-4.65	1.82E-04	4.59E-03
cg01807748	ABCA4	1	Body	Open sea	40.42	5.46	45.07	5.72	-4.65	9.54E-05	2.69E-03
cg03166236	RNF144A	2	5'UTR	S_Shelf	83.46	4.90	88.11	3.79	-4.65	8.63E-07	3.91E-05
cg12154261	TDRD9	14	TSS1500	Island	57.88	7.61	62.53	6.04	-4.65	4.23E-03	4.74E-02
cg03562117	RALYL;RALYL;RALYL;RALYL	8	Body;Body;Body;Body	Open sea	66.44	6.83	71.08	4.67	-4.65	1.46E-04	3.82E-03
cg19554281		15	Intergenic	Open sea	52.80	5.73	57.45	6.71	-4.65	4.74E-04	9.72E-03
cg26450228	PRKCA	17	Body	Open sea	43.64	3.34	48.29	5.22	-4.65	1.21E-05	4.46E-04
cg23810463		11	Intergenic	Open sea	65.16	6.00	69.81	4.76	-4.65	1.07E-04	2.97E-03
cg02573472		5	Intergenic	N_Shore	46.79	4.43	51.44	5.05	-4.65	3.23E-06	1.33E-04
cg04088433	DCN;DCN	12	1stExon;5'UTR	Open sea	54.55	5.85	59.19	5.35	-4.65	1.31E-04	3.50E-03
cg00964333		10	Intergenic	Open sea	82.79	9.13	87.44	6.97	-4.65	3.77E-03	4.38E-02
cg09852744	TULP4;TULP4	6	TSS1500;TSS1500	Open sea	49.97	4.02	54.61	3.96	-4.65	1.13E-06	5.04E-05
cg05565400		11	Intergenic	Island	69.87	6.19	74.52	6.04	-4.65	3.13E-03	3.85E-02
cg00385983		11	Intergenic	Open sea	56.71	6.36	61.36	6.33	-4.65	1.62E-03	2.44E-02
cg23191370	ATP6V1G2;NFKBIL1;NFKBIL1;ATP6V1G	6	Body;TSS1500;TSS1500;Body	S_Shelf	71.98	4.78	76.63	3.96	-4.65	4.71E-05	1.48E-03
cg18950481	LOC149134	1	TSS200	S_Shore	70.79	6.04	75.43	5.66	-4.65	5.40E-04	1.08E-02
cg14604470	FAM102A;FAM102A	9	TSS1500;Body	Open sea	47.87	6.88	52.51	7.47	-4.64	2.37E-03	3.19E-02
cg11572340	KCNIP2;KCNIP2;KCNIP2;KCNIP2;KCNIP	10	1stExon;5'UTR;1stExon;1stExon;1stExon;5	Island	9.84	3.17	14.49	6.99	-4.64	6.07E-04	1.18E-02
cg00651400		6	Intergenic	N_Shore	28.69	3.68	33.33	4.48	-4.64	1.80E-07	8.81E-06
cg17535278		2	Intergenic	Open sea	18.75	4.58	23.39	4.92	-4.64	9.14E-06	3.47E-04
cg07573342	C1QTNF6;C1QTNF6	22	Body;Body	S_Shelf	47.51	5.83	52.16	6.59	-4.64	9.58E-06	3.63E-04
cg09088720	LARP1	5	TSS1500	Open sea	58.84	5.78	63.48	5.40	-4.64	1.23E-05	4.52E-04
cg14223671	PRR25	16	Body	Island	12.05	7.33	16.69	8.36	-4.64	2.03E-03	2.86E-02
cg09933458		13	Intergenic	Island	16.24	4.79	20.88	6.25	-4.64	3.93E-04	8.40E-03
cg19138538	GPR44;GPR44	11	5'UTR;1stExon	S_Shelf	37.34	3.87	41.98	3.80	-4.64	9.75E-08	4.86E-06
cg14473416	SUGT1P1;AQP3	9	Body;Body	Open sea	53.55	4.80	58.19	5.72	-4.64	4.15E-04	8.77E-03
cg15544654	LHX9	1	Body	S_Shore	28.76	5.47	33.40	6.47	-4.64	9.61E-05	2.71E-03
cg18398550	PSPN	19	TSS1500	S_Shelf	49.00	4.18	53.64	4.64	-4.64	2.44E-05	8.33E-04
cg12343380		13	Intergenic	Open sea	53.61	6.35	58.25	6.30	-4.64	4.41E-03	4.88E-02
cg23363182		2	Intergenic	Open sea	77.84	7.56	82.48	5.28	-4.64	3.66E-03	4.30E-02
cg27195304	TIMM50	19	3'UTR	Open sea	71.64	4.30	76.28	3.97	-4.64	4.68E-06	1.88E-04
cg19453726	C2CD3	11	Body	Open sea	60.25	5.47	64.89	4.56	-4.64	6.97E-04	1.31E-02
cg25821437	USP2	11	5'UTR	N_Shelf	53.64	6.13	58.28	5.23	-4.64	1.10E-03	1.84E-02
cg07224291	EYA4;EYA4;EYA4	6	Body;Body;Body	Open sea	67.50	4.51	72.14	5.24	-4.64	9.65E-06	3.65E-04
cg24243629	ZNF532	18	5'UTR	S_Shore	49.27	4.77	53.90	5.15	-4.64	9.24E-05	2.62E-03
cg04857033	PHGDH	1	Body	S_Shelf	44.32	4.71	48.96	5.86	-4.64	1.62E-04	4.17E-03
cg18613031	MARCH8;MARCH8;MARCH8	10	Body;Body;Body	Open sea	52.38	5.84	57.02	6.51	-4.64	3.41E-03	4.10E-02
cg19500607	HTR4;HTR4;HTR4;HTR4;HTR4;HTR4;HT	5	TSS1500;TSS1500;TSS1500;TSS1500;TSS15	S_Shore	45.96	4.07	50.60	4.65	-4.64	3.19E-05	1.05E-03
cg19661705	TRPM1	15	Body	Open sea	39.24	6.47	43.87	6.50	-4.64	1.03E-04	2.88E-03
cg22670128	UBE2J2;UBE2J2;UBE2J2;UBE2J2	1	TSS1500;TSS1500;TSS1500;TSS1500	S_Shore	49.07	4.60	53.71	7.03	-4.64	7.79E-04	1.42E-02
cg08854599	NADSYN1	11	Body	Open sea	88.07	5.12	92.71	2.27	-4.64	8.86E-06	3.38E-04
cg17873645	PTPN9	15	Body	N_Shore	68.95	5.78	73.58	3.97	-4.64	1.00E-04	2.81E-03
cg06899799	ANKRD52	12	Body	N_Shore	29.36	3.67	34.00	3.57	-4.64	1.14E-06	5.08E-05
cg03310930		10	Intergenic	S_Shelf	57.97	5.93	62.61	6.68	-4.64	1.83E-03	2.65E-02
cg14620908	TNXB	6	TSS1500	Open sea	22.97	5.24	27.60	5.49	-4.64	1.13E-03	1.88E-02
cg08447934	ACVRL1;ACVRL1	12	Body;Body	S_Shelf	37.41	3.58	42.05	4.45	-4.64	1.71E-06	7.41E-05
cg01990593	ADAMTS12	5	Body	N_Shore	20.39	5.09	25.03	6.67	-4.64	3.40E-03	4.09E-02
cg04002235		11	Intergenic	Island	27.39	4.62	32.02	6.15	-4.64	8.03E-04	1.45E-02

cg11443506		3	Intergenic	Open sea	47.75	5.98	52.38	5.18	-4.64	1.44E-03	2.24E-02
cg27662832	MAP2;MAP2;MAP2;MAP2	2	TSS200;TSS200;TSS200;5'UTR	Open sea	30.26	4.98	34.89	7.33	-4.64	2.06E-03	2.88E-02
cg13938785	WDR85	9	Body	N_Shore	53.01	6.94	57.65	6.98	-4.63	4.45E-03	4.91E-02
cg06033488	FOXP1	3	Body	Open sea	87.31	5.03	91.95	2.26	-4.63	4.53E-08	2.32E-06
cg19601328	MAP3K14	17	5'UTR	Open sea	42.48	4.88	47.12	5.45	-4.63	5.09E-04	1.03E-02
cg24293507	APITD1;CORT	1	3'UTR;3'UTR	Open sea	22.58	4.35	27.22	4.30	-4.63	2.42E-05	8.28E-04
cg16636671	ZIC1	3	TSS1500	N_Shore	27.89	7.74	32.52	8.92	-4.63	1.04E-03	1.77E-02
cg27293357	RGS7	1	Body	Open sea	36.83	5.66	41.46	5.48	-4.63	7.32E-04	1.36E-02
cg19816811	HOXA6	7	TSS1500	N_Shore	43.60	4.69	48.23	5.56	-4.63	2.59E-04	6.07E-03
cg21167963	TJP2	9	5'UTR	N_Shore	59.14	6.62	63.77	6.09	-4.63	5.45E-04	1.08E-02
cg04451570	PLAC9	10	TSS200	N_Shore	24.08	4.19	28.71	5.95	-4.63	2.51E-04	5.93E-03
cg22731383		2	Intergenic	Open sea	54.88	6.01	59.51	6.96	-4.63	3.13E-03	3.85E-02
cg10776533	EIF4G1;EIF4G1;EIF4G1;EIF4G1;EIF4G1	3	Body;Body;TSS1500;5'UTR;Body	Open sea	37.16	5.39	41.79	6.61	-4.63	2.43E-03	3.24E-02
cg06594770	TRIOBP	22	TSS200	Open sea	29.51	6.74	34.15	7.11	-4.63	1.39E-04	3.67E-03
cg17001464	FLJ43663;FLJ43663	7	Body;Body	Open sea	52.81	5.62	57.44	5.37	-4.63	1.47E-03	2.27E-02
cg14080982	THSD4	15	Body	Open sea	56.46	6.28	61.09	5.84	-4.63	3.66E-03	4.29E-02
cg24938881		2	Intergenic	Island	32.12	3.81	36.75	4.90	-4.63	8.36E-06	3.20E-04
cg07565228	LDHD;LDHD	16	Body;Body	Island	77.33	4.09	81.96	2.50	-4.63	2.45E-07	1.18E-05
cg07641355		7	Intergenic	S_Shelf	54.45	4.65	59.08	3.66	-4.63	2.10E-06	8.96E-05
cg12075776	DMTF1;DMTF1;DMTF1;DMTF1;DMTF	7	5'UTR;5'UTR;Body;Body;5'UTR	Open sea	54.62	3.97	59.25	3.14	-4.63	6.84E-08	3.45E-06
cg23413567	FOXO1	13	Body	Open sea	57.54	6.11	62.16	5.64	-4.63	9.43E-05	2.67E-03
cg03607855		1	Intergenic	N_Shelf	51.11	5.79	55.74	5.31	-4.63	9.32E-04	1.63E-02
cg22865640		20	Intergenic	N_Shelf	62.22	4.93	66.85	4.85	-4.63	1.46E-04	3.83E-03
cg17090891		1	Intergenic	Open sea	77.29	5.44	81.92	6.81	-4.63	5.65E-04	1.11E-02
cg07147958		8	Intergenic	Open sea	73.42	6.83	78.04	5.19	-4.63	3.91E-04	8.37E-03
cg20725662	HSN2;HSN2;WNK1	12	1stExon;3'UTR;Body	Open sea	82.73	7.32	87.35	5.41	-4.63	5.27E-04	1.05E-02
cg26389950	PCDHB16	5	1stExon	N_Shore	36.73	6.47	41.36	7.66	-4.63	3.69E-03	4.32E-02
cg22219450	ABTB2	11	Body	Open sea	41.41	4.19	46.04	3.89	-4.63	1.73E-05	6.15E-04
cg00647232	SEC16B	1	5'UTR	Open sea	47.80	5.65	52.42	6.68	-4.62	1.13E-03	1.88E-02
cg24032691	CAPN13	2	TSS200	Open sea	17.26	3.65	21.88	4.41	-4.62	1.62E-07	7.92E-06
cg24535439	MMP23B	1	TSS1500	Island	25.53	3.99	30.16	5.47	-4.62	1.65E-06	7.17E-05
cg00834536	ADCY9	16	3'UTR	N_Shore	53.18	4.19	57.80	4.90	-4.62	1.08E-05	4.05E-04
cg18450319		7	Intergenic	S_Shore	61.83	4.04	66.45	3.49	-4.62	1.74E-06	7.52E-05
cg09374838		7	Intergenic	Open sea	51.81	5.66	56.44	5.38	-4.62	3.03E-04	6.86E-03
cg10979368	C2orf34	2	Body	Open sea	77.69	5.74	82.31	5.30	-4.62	6.77E-04	1.28E-02
cg22950598	GDF11	12	Body	S_Shelf	46.38	4.63	51.00	4.62	-4.62	2.37E-05	8.14E-04
cg22848598	ADAM32	8	TSS200	Island	15.30	3.60	19.92	7.48	-4.62	9.47E-05	2.68E-03
cg22986876	MAPK1IP1L	14	3'UTR	Open sea	77.08	6.08	81.70	4.91	-4.62	2.64E-04	6.17E-03
cg26847490	ASB4;ASB4	7	1stExon;1stExon	Open sea	72.02	6.04	76.64	6.04	-4.62	5.32E-04	1.06E-02
cg16096717		1	Intergenic	Open sea	37.82	5.17	42.44	6.43	-4.62	1.91E-03	2.73E-02
cg02102075	RAB11FIP3	16	TSS1500	N_Shore	25.53	5.12	30.15	5.86	-4.62	9.63E-04	1.67E-02
cg24711774		7	Intergenic	S_Shore	65.01	7.28	69.63	5.29	-4.62	1.83E-03	2.65E-02
cg13609017	MYPN	10	TSS1500	Open sea	64.72	6.51	69.34	5.74	-4.62	1.12E-03	1.87E-02
cg08490624	ADHFE1	8	Body	N_Shelf	44.96	4.92	49.58	5.62	-4.62	3.89E-04	8.34E-03
cg11411865	TTYH3	7	3'UTR	S_Shore	50.31	5.48	54.93	6.30	-4.62	1.12E-03	1.86E-02
cg21760006		5	Intergenic	Open sea	39.56	4.67	44.18	5.03	-4.62	8.33E-06	3.19E-04
cg11608005		8	Intergenic	Open sea	42.36	4.70	46.98	5.55	-4.62	1.40E-04	3.70E-03
cg26548883	CCL14-CCL15;CCL15;CCL14-CCL15	17	TSS1500;TSS1500;TSS1500	Open sea	74.21	4.66	78.83	4.60	-4.62	2.06E-05	7.18E-04
cg11963436	TNKS2	10	Body	Open sea	25.37	6.58	29.99	6.61	-4.62	1.79E-03	2.61E-02
cg22616430	VARS	6	Body	N_Shelf	65.36	5.23	69.98	4.39	-4.62	8.63E-06	3.30E-04
cg27137955	NKPD1	19	TSS200	Open sea	72.63	4.15	77.25	3.80	-4.62	2.45E-06	1.03E-04
cg04878146		11	Intergenic	Open sea	59.87	5.45	64.49	5.06	-4.62	1.72E-04	4.39E-03
cg20355301	C9orf3;MIR24-1	9	3'UTR;TSS200	N_Shore	76.79	5.38	81.40	5.12	-4.62	3.92E-04	8.38E-03

cg13918042	LCOR;LCOR;LCOR	10	5'UTR;5'UTR;5'UTR	S_Shelf	32.09	5.59	36.70	5.68	-4.62	3.72E-04	8.05E-03
cg01577414	NAALAD2	11	TSS200	Open sea	18.75	4.53	23.37	5.63	-4.62	4.53E-05	1.43E-03
cg21635307	ANKRD6	6	Body	Open sea	26.34	4.94	30.96	4.50	-4.62	5.78E-06	2.28E-04
cg18805612	MIR1178;CIT	12	TSS200;Body	Open sea	43.49	4.95	48.11	4.92	-4.62	2.96E-04	6.73E-03
cg08549241	ANKRD11	16	5'UTR	S_Shore	58.16	5.50	62.77	5.62	-4.62	3.07E-04	6.92E-03
cg02546607	WWOX	16	Body	Open sea	75.53	3.81	80.14	3.29	-4.62	4.80E-08	2.45E-06
cg01771096	AGPAT4	6	Body	Open sea	74.30	5.49	78.92	6.31	-4.62	2.28E-04	5.50E-03
cg13845561		9	Intergenic	S_Shelf	42.34	5.85	46.95	5.83	-4.61	1.95E-03	2.78E-02
cg04570910		12	Intergenic	Open sea	59.59	5.36	64.21	6.40	-4.61	2.16E-03	2.98E-02
cg07198752	CYBRD1;CYBRD1	2	Body;Body	S_Shelf	70.80	5.53	75.41	5.06	-4.61	7.33E-04	1.36E-02
cg05418106		11	Intergenic	Open sea	75.92	2.83	80.53	3.28	-4.61	2.76E-10	1.58E-08
cg03353995	LRRN4CL	11	Body	Island	26.51	3.30	31.13	4.84	-4.61	5.50E-06	2.18E-04
cg16733419	TMEM9B	11	Body	N_Shelf	56.87	4.26	61.49	4.96	-4.61	4.41E-06	1.78E-04
cg13041244		11	Intergenic	Open sea	53.28	4.96	57.89	5.13	-4.61	2.42E-04	5.75E-03
cg09354949	CDC42EP2	11	Body	Open sea	80.34	4.22	84.95	2.70	-4.61	9.58E-09	5.11E-07
cg00556029	MARCKSL1	1	TSS200	Island	37.04	5.29	41.65	5.51	-4.61	2.07E-05	7.20E-04
cg23162647	DKK3;DKK3;DKK3	11	Body;Body;Body	Open sea	39.25	6.71	43.87	6.49	-4.61	1.14E-03	1.89E-02
cg11697169	CLU;CLU;CLU	8	Body;Body;Body	Open sea	82.74	5.08	87.36	4.16	-4.61	1.12E-04	3.07E-03
cg04473569	CLDN15;CLDN15	7	1stExon;5'UTR	Open sea	20.62	5.41	25.23	6.39	-4.61	1.39E-03	2.18E-02
cg11219917	CPLX1	4	5'UTR	Island	23.60	5.34	28.21	6.04	-4.61	4.95E-06	1.98E-04
cg10124807		1	Intergenic	Island	17.66	3.99	22.27	3.85	-4.61	1.66E-05	5.94E-04
cg27528817	SHQ1	3	Body	Open sea	84.66	4.64	89.27	2.47	-4.61	2.24E-06	9.51E-05
cg25509871	PLD3;PLD3	19	5'UTR;5'UTR	N_Shore	34.79	6.31	39.40	7.96	-4.61	7.98E-04	1.44E-02
cg18905039	DYNC1H1	14	Body	Open sea	62.99	5.12	67.60	5.37	-4.61	1.51E-04	3.93E-03
cg02387843	SLC2A9;SLC2A9	4	Body;Body	Open sea	47.00	6.90	51.61	6.30	-4.61	1.99E-03	2.82E-02
cg13472369	DUXA	19	TSS200	Open sea	61.06	6.61	65.67	6.38	-4.61	1.42E-03	2.21E-02
cg06861519	KIAA1949;KIAA1949	6	Body;Body	Open sea	50.23	5.21	54.84	6.73	-4.61	3.15E-03	3.88E-02
cg22996783		11	Intergenic	Open sea	42.81	5.73	47.42	5.14	-4.61	4.38E-07	2.06E-05
cg00004608		6	Intergenic	N_Shore	40.62	4.30	45.23	5.18	-4.61	1.06E-04	2.95E-03
cg24459409	CCDC61	19	Body	S_Shore	49.73	6.65	54.34	7.63	-4.61	3.16E-04	7.08E-03
cg14518803		5	Intergenic	N_Shelf	59.30	3.89	63.90	3.58	-4.61	6.93E-07	3.18E-05
cg16028117	ATG7;ATG7;ATG7	3	Body;Body;Body	Open sea	65.21	6.82	69.82	6.14	-4.61	5.74E-04	1.13E-02
cg19109431	NFKBIE	6	TSS1500	S_Shore	70.05	4.87	74.66	4.13	-4.61	7.55E-05	2.21E-03
cg01043845		2	Intergenic	N_Shore	42.14	5.32	46.75	4.84	-4.61	4.81E-05	1.50E-03
cg14548527		8	Intergenic	N_Shore	49.10	5.45	53.71	6.27	-4.61	1.04E-03	1.76E-02
cg15408497	ITSN1;ITSN1	21	Body;Body	Open sea	26.11	4.63	30.72	6.46	-4.61	1.10E-03	1.84E-02
cg25375711	TRMT12;TRMT12	8	1stExon;5'UTR	Open sea	5.68	2.43	10.29	6.35	-4.61	9.67E-06	3.66E-04
cg18782491	GRM3	7	Body	Open sea	68.17	7.14	72.77	4.57	-4.61	1.19E-03	1.95E-02
cg11639130	STX2;STX2	12	Body;Body	Island	28.98	6.43	33.59	6.75	-4.61	2.91E-04	6.64E-03
cg10486974	PACSIN1	6	Body	Island	86.08	5.80	90.69	3.75	-4.61	3.84E-05	1.24E-03
cg19912197	NLK	17	3'UTR	Open sea	45.76	5.98	50.37	6.55	-4.60	3.01E-03	3.75E-02
cg20583930	PLCH1;PLCH1;PLCH1	3	5'UTR;TSS200;TSS200	Open sea	58.50	6.52	63.11	5.57	-4.60	1.80E-03	2.62E-02
cg27238887	PNPLA2	11	3'UTR	N_Shore	82.56	4.67	87.16	3.12	-4.60	1.04E-05	3.90E-04
cg01961092	HELQ	4	Body	N_Shelf	71.65	4.97	76.25	5.76	-4.60	2.38E-04	5.67E-03
cg23208152	ZMYND10;ZMYND10	3	1stExon;5'UTR	Island	9.86	4.56	14.46	5.24	-4.60	1.99E-05	6.95E-04
cg24769879	CCDC61	19	Body	N_Shore	23.79	4.31	28.39	5.55	-4.60	5.67E-05	1.73E-03
cg21229570		12	Intergenic	Island	18.44	4.72	23.05	4.44	-4.60	1.41E-06	6.18E-05
cg13992402	TSPAN4;TSPAN4;TSPAN4;TSPAN4;TSP	11	5'UTR;Body;Body;Body;Body;Body;Body	S_Shore	84.18	6.30	88.79	4.41	-4.60	2.97E-04	6.74E-03
cg01629993		12	Intergenic	Open sea	65.41	5.56	70.01	6.16	-4.60	4.87E-04	9.91E-03
cg00883190		16	Intergenic	S_Shelf	74.16	4.54	78.76	4.38	-4.60	1.39E-06	6.09E-05
cg19599045	SLC7A7	14	TSS1500	N_Shore	40.94	5.95	45.55	6.23	-4.60	3.89E-03	4.47E-02
cg05666820	FZD9	7	1stExon	Island	58.16	5.69	62.77	7.00	-4.60	1.06E-03	1.79E-02
cg25152584		2	Intergenic	Open sea	44.30	6.23	48.90	6.10	-4.60	3.46E-03	4.14E-02

cg02341670	TAS2R40	7	TSS200	Open sea	59.25	5.13	63.85	5.17	-4.60	1.37E-04	3.63E-03
cg20938231		3	Intergenic	Open sea	51.71	5.36	56.31	5.54	-4.60	1.10E-03	1.84E-02
cg04318619	ABR;ABR	17	1stExon;5'UTR	Open sea	47.71	5.03	52.31	6.21	-4.60	8.38E-04	1.50E-02
cg21606168		21	Intergenic	S_Shore	50.06	2.61	54.66	2.99	-4.60	1.97E-10	1.13E-08
cg01651250	TNXB	6	Body	N_Shelf	50.81	5.70	55.42	6.54	-4.60	6.13E-04	1.18E-02
cg04177826	KLHDC7B	22	TSS1500	Island	34.65	7.60	39.25	5.87	-4.60	1.20E-03	1.96E-02
cg02529909	F2RL2;IQGAP2	5	TSS1500;Body	Open sea	68.96	5.62	73.56	6.51	-4.60	3.38E-04	7.47E-03
cg21824343	WDR81;WDR81;WDR81;WDR81	17	Body;Body;Body;Body	N_Shelf	67.67	4.18	72.27	3.68	-4.60	6.23E-06	2.44E-04
cg25073998		2	Intergenic	Open sea	29.94	5.70	34.54	6.82	-4.60	2.74E-03	3.52E-02
cg00465247		13	Intergenic	N_Shore	49.61	5.37	54.21	7.89	-4.60	9.19E-04	1.61E-02
cg05627138		5	Intergenic	Open sea	20.50	4.96	25.10	7.50	-4.60	2.03E-03	2.85E-02
cg05270634	RND2	17	1stExon	Island	40.58	6.25	45.18	6.02	-4.60	4.13E-04	8.74E-03
cg20584841	HMHA1	19	Body	Island	38.35	5.03	42.95	4.76	-4.60	1.04E-04	2.90E-03
cg24640637	CEND1	11	TSS200	N_Shore	62.51	5.57	67.11	6.01	-4.60	1.46E-03	2.26E-02
cg22068093		5	Intergenic	Open sea	73.60	4.70	78.20	4.34	-4.60	4.17E-05	1.33E-03
cg02248486	HOXA5	7	1stExon	Island	17.92	3.84	22.52	4.93	-4.60	7.78E-05	2.26E-03
cg02633117	MYO9B;HAUS8;MYO9B;HAUS8	19	5'UTR;TSS1500;5'UTR;TSS1500	S_Shore	29.54	5.73	34.14	5.18	-4.60	4.81E-05	1.50E-03
cg22344162	CREG1	1	TSS1500	S_Shore	52.51	7.27	57.11	6.79	-4.60	3.37E-03	4.06E-02
cg10825234		4	Intergenic	N_Shelf	70.22	6.97	74.82	7.17	-4.60	4.45E-03	4.91E-02
cg08866129	HPR	16	TSS1500	Open sea	46.31	6.00	50.91	6.34	-4.60	1.76E-03	2.58E-02
cg23513183		19	Intergenic	Island	69.03	7.42	73.63	6.51	-4.60	4.28E-03	4.78E-02
cg22621867	GPR62	3	1stExon	Island	21.77	5.29	26.37	6.93	-4.60	1.12E-04	3.07E-03
cg16204618	ZNF320	19	TSS200	Open sea	50.07	4.89	54.66	6.17	-4.60	5.18E-04	1.04E-02
cg25965471	TTBK1;TTBK1	6	5'UTR;1stExon	Island	28.96	5.47	33.56	5.13	-4.60	2.53E-04	5.96E-03
cg03705366		5	Intergenic	Open sea	37.69	5.29	42.28	5.01	-4.60	8.43E-05	2.43E-03
cg15826444		2	Intergenic	Open sea	66.72	4.87	71.31	4.70	-4.60	7.94E-05	2.31E-03
cg26951614	LONP2	16	Body	S_Shelf	56.82	6.33	61.42	6.52	-4.60	2.86E-03	3.62E-02
cg06031041	FOXK1	7	3'UTR	S_Shelf	59.95	6.22	64.54	6.65	-4.60	1.69E-03	2.50E-02
cg24379599	OCLM;C1orf27;C1orf27;MIR548F1;C1(	1	TSS1500;Body;Body;Body;Body	Open sea	82.39	6.07	86.99	4.67	-4.60	3.57E-05	1.16E-03
cg00408285	KIAA0182;KIAA0182	16	5'UTR;Body	Island	56.63	5.35	61.23	6.33	-4.60	4.52E-03	4.96E-02
cg04026354	SMG6;SMG6	17	Body;Body	Open sea	57.17	7.26	61.77	5.92	-4.60	2.48E-04	5.86E-03
cg06143864	OSGIN1;OSGIN1	16	TSS1500;TSS1500	Open sea	38.49	5.77	43.08	5.89	-4.60	1.03E-03	1.75E-02
cg23288725		8	Intergenic	Island	69.28	5.99	73.87	6.75	-4.60	7.83E-04	1.43E-02
cg24040450	KCNT2	1	TSS1500	S_Shore	60.73	4.90	65.32	4.97	-4.60	6.42E-05	1.93E-03
cg13573346	MEG8	14	Body	Open sea	49.92	6.11	54.51	6.36	-4.59	8.85E-04	1.56E-02
cg05338009	ZNF331;ZNF331;ZNF331	19	1stExon;5'UTR;5'UTR	Island	32.59	6.46	37.19	5.58	-4.59	3.36E-03	4.05E-02
cg26015971	LY6G6D	6	Body	Open sea	62.26	5.27	66.85	4.79	-4.59	9.56E-05	2.70E-03
cg25367249	NTN4	12	Body	Open sea	71.76	5.51	76.36	4.36	-4.59	1.04E-04	2.89E-03
cg24482288	MIR181D;MIR181C	19	TSS200;Body	S_Shore	67.19	5.02	71.78	6.36	-4.59	5.40E-04	1.08E-02
cg10210094	TNNI1	1	3'UTR	Open sea	57.45	6.77	62.05	6.91	-4.59	1.60E-04	4.14E-03
cg13543473	RAPGEF1	9	Body	Open sea	62.67	4.68	67.26	4.62	-4.59	5.41E-05	1.66E-03
cg13508369	ANGPTL2;RALGPS1	9	5'UTR;Body	Open sea	57.04	5.37	61.63	5.76	-4.59	1.29E-03	2.06E-02
cg07130150		12	Intergenic	S_Shore	49.42	5.50	54.01	6.02	-4.59	2.01E-03	2.84E-02
cg27038428	RNF139	8	TSS1500	N_Shore	31.62	6.42	36.22	6.08	-4.59	1.06E-03	1.79E-02
cg18105612		14	Intergenic	S_Shore	39.51	5.75	44.11	6.84	-4.59	2.21E-04	5.34E-03
cg22892036	JAZF1	7	Body	Open sea	48.66	5.68	53.25	6.52	-4.59	2.91E-03	3.66E-02
cg13837721		11	Intergenic	Open sea	23.35	5.98	27.94	6.68	-4.59	1.46E-03	2.26E-02
cg19255783	PLAC8;PLAC8	4	TSS200;TSS200	Open sea	50.93	5.48	55.53	6.05	-4.59	4.06E-05	1.30E-03
cg16701914	PRSS22	16	Body	Island	35.74	5.27	40.33	5.80	-4.59	5.28E-04	1.06E-02
cg25060765		6	Intergenic	Open sea	50.77	3.96	55.36	5.18	-4.59	9.79E-05	2.75E-03
cg10586870	IQGAP2	5	Body	Open sea	33.80	4.96	38.39	6.60	-4.59	2.52E-04	5.94E-03
cg17017514		1	Intergenic	S_Shelf	39.66	4.00	44.25	4.92	-4.59	8.28E-05	2.39E-03
cg07546654	TOLLIP	11	Body	S_Shelf	28.03	4.86	32.62	4.73	-4.59	8.98E-06	3.42E-04

cg24047926	SOX13	1	3'UTR	Open sea	53.04	3.61	57.63	4.76	-4.59	5.37E-06	2.14E-04
cg06547203	RRN3	16	3'UTR	S_Shelf	79.44	6.07	84.03	5.81	-4.59	1.34E-04	3.55E-03
cg20878925		13	Intergenic	Open sea	78.53	6.42	83.12	4.26	-4.59	4.13E-04	8.74E-03
cg20264543		3	Intergenic	Open sea	17.66	4.23	22.25	5.12	-4.59	1.28E-05	4.71E-04
cg02118840		14	Intergenic	Open sea	70.17	4.45	74.76	4.25	-4.59	2.99E-06	1.24E-04
cg04873188	MXD1	2	Body	S_Shore	49.64	6.13	54.23	5.16	-4.59	2.61E-04	6.10E-03
cg08473330	OTX1	2	Body	Island	32.89	4.00	37.48	4.42	-4.59	8.78E-07	3.97E-05
cg02920150	ESRRG;ESRRG;ESRRG	1	5'UTR;5'UTR;5'UTR	Open sea	71.38	6.01	75.97	6.87	-4.59	1.44E-03	2.24E-02
cg22472290	ZNF577;ZNF577;ZNF577	19	TSS1500;TSS1500;TSS1500	S_Shore	25.31	4.83	29.89	5.30	-4.59	2.13E-04	5.19E-03
cg21472506	OTX1	2	3'UTR	Island	15.84	3.65	20.43	4.29	-4.59	4.63E-06	1.86E-04
cg06729723	SBF1	22	3'UTR	Island	62.58	5.05	67.16	5.41	-4.59	3.86E-04	8.28E-03
cg18638931	MOV10L1;MOV10L1;MOV10L1	22	TSS1500;TSS1500;TSS1500	N_Shore	63.66	6.90	68.25	7.48	-4.59	2.14E-03	2.96E-02
cg03239422	CRYL1	13	TSS1500	S_Shore	55.14	6.17	59.72	7.42	-4.59	1.85E-03	2.67E-02
cg11002923	CCDC36;CCDC36	3	5'UTR;5'UTR	Island	76.91	7.14	81.50	5.83	-4.59	3.56E-03	4.22E-02
cg15074033		12	Intergenic	Island	12.86	3.19	17.44	4.54	-4.59	8.78E-08	4.40E-06
cg04793338		13	Intergenic	Open sea	68.49	6.67	73.08	5.77	-4.59	1.36E-04	3.60E-03
cg27395342	DLC1;DLC1	8	Body;Body	Open sea	32.33	4.85	36.91	4.79	-4.59	8.65E-06	3.30E-04
cg09338148		16	Intergenic	Open sea	50.69	3.93	55.27	4.50	-4.59	1.63E-06	7.09E-05
cg14798390		5	Intergenic	S_Shelf	54.68	5.00	59.27	4.52	-4.59	8.56E-05	2.46E-03
cg11309217	FN1;FN1;FN1;FN1;FN1;FN1;FN1	2	Body;Body;Body;Body;Body;Body;Body	N_Shore	48.15	5.43	52.73	6.65	-4.59	3.48E-03	4.15E-02
cg25083732	S100P	4	TSS200	Open sea	45.83	5.93	50.41	6.12	-4.59	2.86E-03	3.62E-02
cg14039246	ECHDC2	1	Body	Open sea	26.34	5.49	30.93	6.35	-4.59	1.71E-03	2.53E-02
cg11418725		5	Intergenic	Open sea	55.08	5.71	59.67	5.48	-4.59	2.53E-04	5.95E-03
cg00240860	KIAA0430	16	Body	Open sea	30.91	5.74	35.50	6.32	-4.59	4.49E-03	4.94E-02
cg25772365		6	Intergenic	Open sea	37.30	6.81	41.88	6.27	-4.59	2.72E-03	3.50E-02
cg11524400	HCCA2;CTSD	11	Body;Body	S_Shelf	50.69	4.68	55.27	5.03	-4.59	3.86E-05	1.24E-03
cg11321190		3	Intergenic	Open sea	32.44	5.82	37.03	5.67	-4.59	2.18E-05	7.53E-04
cg22707705	CYP27A1	2	TSS1500	N_Shore	27.03	5.57	31.61	5.08	-4.58	5.23E-04	1.05E-02
cg21384729		2	Intergenic	S_Shelf	67.33	5.31	71.91	4.59	-4.58	4.08E-05	1.30E-03
cg13170891	PPP1R10	6	3'UTR	Open sea	69.76	4.69	74.35	5.07	-4.58	4.16E-04	8.78E-03
cg04420889	SGK2	20	TSS1500	Open sea	41.95	7.49	46.53	5.52	-4.58	4.30E-03	4.79E-02
cg11651614		1	Intergenic	Island	39.17	4.96	43.75	5.87	-4.58	3.34E-04	7.41E-03
cg20869658	TTC30A;TTC30A	2	1stExon;3'UTR	N_Shelf	67.92	6.81	72.50	4.20	-4.58	3.12E-04	7.00E-03
cg10803302	ZBTB41	1	Body	Open sea	54.43	5.75	59.02	7.00	-4.58	3.35E-03	4.04E-02
cg16024950	ACTL6B	7	TSS200	Island	20.72	4.38	25.31	4.75	-4.58	3.43E-05	1.12E-03
cg04550950	LRRC8D;LRRC8D	1	5'UTR;5'UTR	Open sea	50.36	4.96	54.94	5.02	-4.58	2.12E-04	5.17E-03
cg05219493	CYP26C1	10	TSS200	Island	66.62	5.88	71.21	6.15	-4.58	1.90E-03	2.73E-02
cg20475005	WASL	7	Body	Open sea	69.04	5.56	73.62	4.06	-4.58	5.62E-05	1.72E-03
cg08187814	ZEB2;ZEB2	2	Body;Body	Open sea	44.66	6.11	49.24	6.90	-4.58	2.71E-03	3.49E-02
cg07335376	MRPL15	8	TSS1500	N_Shore	43.40	6.29	47.98	7.84	-4.58	3.10E-03	3.83E-02
cg09502015		5	Intergenic	Open sea	23.43	5.01	28.01	5.29	-4.58	7.04E-05	2.08E-03
cg14163444		7	Intergenic	Island	10.52	2.38	15.10	6.17	-4.58	4.84E-06	1.94E-04
cg20852788	SEC24D	4	Body	Open sea	46.37	6.00	50.95	7.14	-4.58	4.15E-03	4.68E-02
cg08580224		5	Intergenic	N_Shelf	58.76	6.68	63.34	4.65	-4.58	9.69E-04	1.67E-02
cg04606020	GDF11	12	Body	S_Shore	20.63	4.41	25.21	4.70	-4.58	1.45E-05	5.24E-04
cg02374934	DLL4	15	Body	Island	55.35	5.75	59.93	5.69	-4.58	1.60E-03	2.42E-02
cg03611852	PLCD3	17	Body	N_Shelf	54.25	5.06	58.83	4.89	-4.58	8.97E-05	2.56E-03
cg00262621	MMP23B	1	TSS1500	Island	30.04	4.61	34.61	5.30	-4.58	2.79E-05	9.37E-04
cg04285666		5	Intergenic	N_Shore	40.73	4.18	45.31	4.21	-4.58	1.95E-06	8.39E-05
cg07342901	H19;MIR675	11	Body;TSS1500	N_Shore	74.35	4.63	78.93	4.40	-4.58	1.57E-05	5.63E-04
cg23443098	FRMD4A	10	Body	Open sea	67.95	4.18	72.53	4.27	-4.58	2.34E-06	9.91E-05
cg08783509		11	Intergenic	Island	3.15	2.00	7.73	6.15	-4.58	4.13E-08	2.12E-06
cg18011517		2	Intergenic	Open sea	31.02	4.25	35.60	5.93	-4.58	1.38E-04	3.65E-03

cg24389585	HOXA5	7	TSS1500	Island	59.72	4.67	64.30	4.98	-4.58	8.07E-05	2.34E-03
cg22609511		7	Intergenic	N_Shelf	46.93	6.14	51.51	7.60	-4.58	2.06E-03	2.89E-02
cg22054793	SLC26A4	7	Body	S_Shelf	76.10	4.65	80.68	4.65	-4.58	2.27E-05	7.80E-04
cg22653915	PXDNL	8	TSS200	Open sea	50.04	4.73	54.62	5.87	-4.58	6.20E-05	1.87E-03
cg20002504	ANKMY1;ANKMY1	2	TSS200;TSS200	Island	17.96	5.23	22.54	3.80	-4.58	3.36E-07	1.60E-05
cg22532194	NFATC1;NFATC1;NFATC1;NFATC1	18	Body;Body;Body;Body	N_Shore	82.94	5.85	87.52	4.36	-4.58	1.06E-04	2.95E-03
cg09814487		16	Intergenic	N_Shore	33.71	4.35	38.28	5.79	-4.58	1.22E-04	3.30E-03
cg10189962		2	Intergenic	N_Shore	35.36	3.65	39.94	3.84	-4.58	2.29E-07	1.11E-05
cg24126851	DCHS1	11	TSS1500	S_Shore	69.18	5.35	73.75	4.89	-4.58	8.69E-05	2.49E-03
cg19728577	GUCA2B	1	1stExon	Open sea	62.91	5.52	67.48	5.04	-4.58	4.83E-05	1.51E-03
cg17031543	SPATA18	4	Body	S_Shore	17.31	6.51	21.89	4.95	-4.58	3.81E-05	1.23E-03
cg06226516		7	Intergenic	Open sea	62.79	6.17	67.36	5.69	-4.58	4.18E-04	8.81E-03
cg05292788	GABBR1;GABBR1;GABBR1	6	Body;Body;Body	Open sea	75.28	5.80	79.86	4.90	-4.58	8.65E-05	2.48E-03
cg23245620	SIX3	2	3'UTR	S_Shore	42.05	6.00	46.63	7.80	-4.57	4.04E-03	4.59E-02
cg07955474	IRF8	16	5'UTR	S_Shelf	25.58	4.38	30.16	4.50	-4.57	1.62E-06	7.06E-05
cg23390920	RMST	12	TSS1500	Open sea	62.74	5.85	67.32	6.44	-4.57	2.65E-03	3.44E-02
cg10100424	PRDM16;PRDM16	1	Body;Body	Open sea	21.62	4.23	26.19	7.05	-4.57	9.68E-04	1.67E-02
cg06752157	MYST4	10	Body	Open sea	53.47	6.13	58.04	7.18	-4.57	3.75E-03	4.37E-02
cg21266330	NPFFR2	4	1stExon	Island	17.38	5.23	21.95	7.60	-4.57	1.41E-03	2.21E-02
cg05700079	ZIC1	3	TSS1500	N_Shore	34.27	7.18	38.84	7.46	-4.57	2.21E-03	3.04E-02
cg14677130	EGFLAM;EGFLAM;EGFLAM;EGFLAM	5	Body;5'UTR;Body;Body	Open sea	80.93	6.56	85.50	5.02	-4.57	8.21E-04	1.47E-02
cg11846580	ZFPM1	16	Body	Island	59.55	5.38	64.12	3.97	-4.57	1.80E-04	4.55E-03
cg15358846		5	Intergenic	Open sea	66.73	4.22	71.30	3.87	-4.57	4.38E-07	2.06E-05
cg13540639	IL17F	6	TSS1500	Open sea	77.26	4.72	81.83	4.34	-4.57	5.97E-05	1.81E-03
cg14223822		13	Intergenic	N_Shore	60.95	8.88	65.52	7.62	-4.57	3.18E-03	3.90E-02
cg19815596		1	Intergenic	Open sea	22.79	4.86	27.36	3.84	-4.57	6.26E-07	2.89E-05
cg18744668	OSR2;OSR2	8	Body;Body	S_Shore	66.68	5.10	71.25	4.78	-4.57	9.41E-05	2.66E-03
cg17783697	SEC31A;SEC31A;SEC31A;SEC31A;SEC3	4	Body;Body;Body;Body;Body	Open sea	68.16	5.98	72.73	5.26	-4.57	8.87E-04	1.57E-02
cg03402459		6	Intergenic	Island	44.72	6.06	49.29	4.29	-4.57	3.66E-04	7.95E-03
cg03267511		6	Intergenic	N_Shelf	47.99	5.12	52.55	6.81	-4.57	2.65E-03	3.44E-02
cg14120583	SHC2	19	Body	Island	73.40	7.12	77.97	6.32	-4.57	2.70E-04	6.27E-03
cg01556275		1	Intergenic	N_Shore	26.85	4.68	31.42	7.16	-4.57	4.49E-03	4.94E-02
cg01124523		4	Intergenic	Open sea	45.32	4.93	49.89	3.99	-4.57	2.81E-06	1.17E-04
cg09173325	SPRYD3	12	3'UTR	Open sea	64.03	4.10	68.60	4.26	-4.57	2.11E-05	7.31E-04
cg18371424	SYNE2;SYNE2	14	Body;Body	Open sea	71.77	6.12	76.33	5.27	-4.56	5.62E-04	1.11E-02
cg20543645		8	Intergenic	Open sea	55.65	4.92	60.22	4.69	-4.56	4.01E-05	1.29E-03
cg02709321	TOX3;TOX3	16	5'UTR;TSS1500	Island	30.96	8.87	35.52	8.34	-4.56	1.04E-03	1.76E-02
cg13971484	BAT2L1	9	Body	S_Shelf	55.54	6.04	60.11	5.97	-4.56	7.74E-04	1.41E-02
cg19475870	CDH9;CDH9	5	1stExon;5'UTR	Open sea	36.06	5.71	40.63	6.68	-4.56	3.60E-03	4.25E-02
cg11545887	RBMXL2;RBMXL2	11	5'UTR;1stExon	Island	59.51	5.86	64.07	6.10	-4.56	7.80E-04	1.42E-02
cg05944661		1	Intergenic	S_Shelf	57.93	4.59	62.50	4.00	-4.56	3.11E-07	1.49E-05
cg17723381		12	Intergenic	Open sea	34.16	4.12	38.72	5.80	-4.56	5.03E-05	1.56E-03
cg12037324	NXPH2	2	Body	Open sea	79.51	5.46	84.07	4.44	-4.56	4.47E-06	1.80E-04
cg07738730	IGF2BP1;IGF2BP1	17	Body;Body	S_Shore	49.65	4.71	54.21	5.64	-4.56	9.56E-05	2.70E-03
cg25910261	PTPRN2;PTPRN2;PTPRN2	7	Body;Body;Body	Island	25.79	3.84	30.35	5.88	-4.56	5.83E-05	1.78E-03
cg07636194	HIST1H2BK;HIST1H4I	6	3'UTR;1stExon	Island	38.98	5.98	43.54	6.10	-4.56	1.42E-04	3.74E-03
cg16029659		21	Intergenic	S_Shelf	66.55	5.74	71.11	4.56	-4.56	2.07E-04	5.07E-03
cg03347207	PTPRF;PTPRF	1	Body;Body	Open sea	16.98	5.79	21.54	8.55	-4.56	8.98E-04	1.58E-02
cg18613421	WDR72	15	5'UTR	N_Shore	43.29	5.17	47.85	5.41	-4.56	6.26E-05	1.89E-03
cg16196021	RFC1	4	Body	N_Shore	58.71	5.31	63.27	7.20	-4.56	2.46E-03	3.27E-02
cg12686273	CCDC64B	16	TSS1500	N_Shore	39.27	4.98	43.83	5.33	-4.56	6.31E-04	1.21E-02
cg25703567	MCM3	6	3'UTR	Open sea	27.81	4.14	32.37	5.30	-4.56	5.30E-05	1.63E-03
cg10459018		6	Intergenic	S_Shelf	56.02	4.95	60.58	6.60	-4.56	4.64E-04	9.57E-03

cg17832639	TNXB	6	TSS1500	Open sea	55.14	4.54	59.70	6.17	-4.56	1.62E-04	4.17E-03
cg17408972	RAI14;RAI14;RAI14;RAI14;RAI14;RAI1	5	1stExon;5'UTR;5'UTR;5'UTR;5'UTR;1stExor	Open sea	75.29	5.17	79.85	3.82	-4.56	2.23E-06	9.50E-05
cg08606748		19	Intergenic	Island	53.60	5.47	58.16	4.54	-4.56	2.09E-04	5.11E-03
cg12379720	TRIOBP	22	TSS200	Open sea	39.62	7.79	44.18	7.48	-4.56	4.74E-04	9.72E-03
cg15564000	TRIM33;TRIM33	1	Body;Body	Open sea	64.46	5.90	69.02	5.26	-4.56	4.14E-04	8.76E-03
cg12434982	LHX9;LHX9	1	Body;Body	Island	65.46	4.96	70.02	5.01	-4.56	1.13E-04	3.10E-03
cg05436939	LOC100130872;LOC100130872-SPON2	4	Body;Body	N_Shore	57.25	4.78	61.81	4.27	-4.56	4.96E-06	1.98E-04
cg14081737	BSPRY	9	TSS1500	N_Shore	74.07	5.19	78.63	5.56	-4.56	3.53E-04	7.72E-03
cg15402210	LOC100132215	2	TSS200	Island	36.36	3.85	40.91	5.26	-4.56	1.24E-06	5.48E-05
cg03576555	CTBP2;CTBP2	10	5'UTR;5'UTR	Open sea	76.74	3.94	81.29	4.00	-4.56	3.65E-07	1.73E-05
cg26671477		1	Intergenic	N_Shelf	39.87	5.47	44.43	5.86	-4.56	8.41E-04	1.50E-02
cg04974499	PRDM16;PRDM16	1	Body;Body	N_Shore	50.44	4.70	55.00	6.52	-4.56	1.90E-03	2.73E-02
cg23715407		14	Intergenic	Open sea	59.36	6.40	63.91	4.64	-4.56	8.18E-04	1.47E-02
cg10307662		4	Intergenic	Open sea	68.53	5.90	73.08	5.58	-4.56	4.93E-04	1.00E-02
cg09745688	PRSS22	16	TSS1500	S_Shore	19.99	7.72	24.55	8.76	-4.55	2.91E-03	3.67E-02
cg16934969	RAPGEF4	2	Body	Open sea	65.58	5.50	70.14	5.76	-4.55	5.24E-05	1.62E-03
cg20372886		3	Intergenic	Open sea	14.49	3.81	19.04	6.20	-4.55	1.23E-04	3.33E-03
cg04116306	PLXNB1;PLXNB1	3	Body;Body	Open sea	82.79	6.13	87.34	4.12	-4.55	6.32E-04	1.21E-02
cg01403010	MAFF;MAFF;MAFF;MAFF	22	5'UTR;5'UTR;5'UTR;5'UTR	N_Shore	68.65	5.03	73.21	4.37	-4.55	2.82E-05	9.45E-04
cg02985568	HSPA13	21	Body	N_Shore	49.19	5.88	53.75	6.37	-4.55	1.87E-03	2.70E-02
cg23957912		4	Intergenic	S_Shelf	67.57	6.71	72.13	6.96	-4.55	7.72E-04	1.41E-02
cg02340851	ANP32E;ANP32E;ANP32E	1	Body;5'UTR;Body	N_Shore	20.42	5.47	24.97	5.28	-4.55	2.32E-06	9.85E-05
cg24199400	WDR52;WDR52	3	TSS200;TSS200	Island	33.52	3.79	38.07	4.67	-4.55	2.80E-08	1.45E-06
cg11521797	LOC284798;LOC284798;LOC284798	20	Body;Body;Body	N_Shelf	39.99	6.02	44.54	6.43	-4.55	3.90E-04	8.36E-03
cg26064774		4	Intergenic	Island	22.43	4.51	26.98	5.43	-4.55	1.67E-05	5.96E-04
cg19197922		10	Intergenic	S_Shelf	60.73	6.07	65.28	5.80	-4.55	1.06E-04	2.94E-03
cg23510415	KIAA1467	12	Body	Open sea	36.22	5.80	40.77	5.59	-4.55	1.65E-04	4.23E-03
cg22597340	HTT	4	Body	Open sea	84.27	4.38	88.82	3.54	-4.55	5.40E-06	2.15E-04
cg13268590	MAGI1;MAGI1;MAGI1	3	Body;Body;Body	Open sea	28.33	5.25	32.87	6.38	-4.55	2.01E-03	2.84E-02
cg06618474	CRIP1	14	TSS1500	N_Shore	42.62	4.61	47.17	5.89	-4.55	5.24E-04	1.05E-02
cg21078198	USP42	7	Body	N_Shelf	62.55	4.57	67.10	4.57	-4.55	3.79E-05	1.22E-03
cg09730500	GPR56;GPR56;GPR56;GPR56;GPR56;G	16	5'UTR;1stExon;5'UTR;1stExon;1stExon;5'U	Open sea	32.14	4.68	36.69	7.22	-4.55	1.04E-03	1.76E-02
cg08728495		12	Intergenic	Open sea	64.51	5.54	69.06	4.59	-4.55	1.10E-04	3.02E-03
cg27182935	ZNF276;ZNF276	16	Body;Body	S_Shore	44.13	3.67	48.68	3.83	-4.55	2.84E-06	1.19E-04
cg00641210	BCL6B	17	Body	Island	28.58	5.15	33.13	5.83	-4.55	1.51E-04	3.92E-03
cg10994564	ADPRH	3	Body	Open sea	62.57	6.78	67.12	5.59	-4.55	2.11E-04	5.15E-03
cg15150064	KIF3C	2	Body	N_Shelf	60.98	4.63	65.53	5.20	-4.55	2.01E-05	7.01E-04
cg02150262	ROBO4	11	Body	Open sea	62.87	4.16	67.41	5.16	-4.55	2.50E-07	1.21E-05
cg02451831	KIAA0087	7	Body	Open sea	65.69	5.62	70.23	6.19	-4.55	1.30E-03	2.08E-02
cg23258940	NAT14;ZNF628	19	TSS1500;3'UTR	Island	66.04	5.28	70.58	4.70	-4.55	2.27E-04	5.48E-03
cg24747122	GNA13	17	TSS1500	S_Shore	27.02	6.03	31.56	6.90	-4.55	3.19E-03	3.91E-02
cg19092167	MASP1;MASP1;MASP1	3	Body;Body;Body	Open sea	38.65	4.42	43.20	6.19	-4.54	5.49E-04	1.09E-02
cg00973118	AXIN1;AXIN1	16	Body;Body	N_Shore	71.60	4.82	76.15	4.72	-4.54	3.41E-05	1.12E-03
cg01141438	LEPREL1;LEPREL1	3	5'UTR;Body	N_Shore	50.73	4.62	55.27	5.93	-4.54	6.42E-05	1.93E-03
cg00030420	SLC25A2	5	TSS200	Island	73.67	4.78	78.22	4.86	-4.54	2.19E-04	5.31E-03
cg21847393	ZMYND10	3	TSS1500	S_Shore	32.19	6.36	36.74	6.16	-4.54	8.80E-05	2.52E-03
cg01650137		4	Intergenic	Open sea	30.29	4.97	34.83	5.07	-4.54	1.62E-04	4.16E-03
cg18075666	CALD1;CALD1;CALD1	7	5'UTR;5'UTR;5'UTR	Open sea	47.91	6.61	52.45	5.61	-4.54	2.15E-04	5.23E-03
cg12399536	TEAD1	11	Body	Open sea	27.73	5.54	32.28	5.94	-4.54	7.94E-04	1.44E-02
cg25924807	CNTN4	3	Body	Open sea	48.54	7.41	53.09	5.31	-4.54	2.17E-03	2.99E-02
cg23885131	RNF41;RNF41;RNF41	12	Body;Body;Body	Open sea	69.45	5.13	73.99	4.90	-4.54	3.70E-04	8.02E-03
cg03348461	CAMTA1	1	Body	Island	36.42	5.04	40.96	5.19	-4.54	1.64E-06	7.13E-05
cg14320626		4	Intergenic	N_Shelf	50.63	4.64	55.17	6.52	-4.54	9.73E-04	1.68E-02

cg07312601	MRTO4	1	Body	Open sea	30.76	4.17	35.30	4.04	-4.54	2.82E-06	1.18E-04
cg07522913	HOXA3;HOXA3;HOXA3	7	5'UTR;5'UTR;5'UTR	Island	33.73	3.89	38.27	5.13	-4.54	2.82E-05	9.44E-04
cg17982038		15	Intergenic	Open sea	37.58	6.15	42.12	6.11	-4.54	3.78E-04	8.15E-03
cg03641033	FAM38A	16	Body	Open sea	61.01	5.12	65.55	4.82	-4.54	1.44E-04	3.79E-03
cg13902067	RNF183	9	TSS1500	Open sea	74.95	5.28	79.49	4.64	-4.54	7.63E-05	2.23E-03
cg14033170	CREB5;CREB5;CREB5	7	Body;Body;Body	Open sea	33.10	4.03	37.64	6.24	-4.54	1.90E-04	4.75E-03
cg12552104	CREB5;CREB5;CREB5	7	Body;Body;Body	Open sea	54.08	5.33	58.62	6.58	-4.54	1.61E-03	2.43E-02
cg26920673		8	Intergenic	Open sea	67.15	11.95	71.69	5.40	-4.54	4.19E-03	4.71E-02
cg09777657	RAI1	17	5'UTR	Open sea	58.05	5.90	62.59	6.32	-4.54	4.00E-05	1.28E-03
cg03330762	TRPM4	19	Body	Island	82.65	6.08	87.19	6.30	-4.54	1.56E-03	2.37E-02
cg26972479		17	Intergenic	N_Shelf	23.63	4.17	28.17	5.72	-4.54	6.24E-04	1.20E-02
cg09378425	REPIN1;REPIN1;REPIN1;REPIN1	7	5'UTR;Body;TSS1500;5'UTR	N_Shore	37.71	5.56	42.25	6.15	-4.54	2.79E-03	3.56E-02
cg04630639	FSCN1	7	Body	N_Shore	73.83	4.25	78.36	5.90	-4.54	1.08E-04	2.98E-03
cg17679619		4	Intergenic	Open sea	37.61	5.67	42.15	4.53	-4.54	6.14E-04	1.19E-02
cg23499846	KIAA0664	17	5'UTR	S_Shore	55.60	4.68	60.13	5.45	-4.54	3.09E-04	6.96E-03
cg03318924	C1QL4	12	Body	N_Shore	70.17	5.85	74.71	6.04	-4.54	4.19E-04	8.84E-03
cg12646029	OR2H1	6	5'UTR	Open sea	46.32	6.40	50.86	7.61	-4.54	3.27E-03	3.97E-02
cg03450635	FAM198B;FAM198B;FAM198B	4	TSS200;TSS1500;TSS200	Open sea	26.24	4.92	30.77	3.94	-4.53	2.66E-05	9.00E-04
cg12970937	CALCR;CALCR;CALCR	7	5'UTR;5'UTR;5'UTR	Open sea	66.57	6.94	71.11	5.88	-4.53	6.11E-04	1.18E-02
cg08715146	ATXN10;ATXN10	22	TSS1500;TSS1500	N_Shore	50.99	6.96	55.52	7.23	-4.53	3.02E-03	3.76E-02
cg11740416	FAM43A;FAM43A	3	3'UTR;1stExon	S_Shore	38.46	6.32	43.00	6.74	-4.53	4.03E-03	4.59E-02
cg14402591	SEMA6B	19	3'UTR	Island	50.53	5.68	55.06	6.43	-4.53	2.52E-03	3.32E-02
cg21222681	ANXA1	9	Body	Open sea	76.85	6.15	81.38	5.86	-4.53	4.64E-05	1.46E-03
cg19861427	CRTC1;CRTC1	19	Body;Body	Island	56.63	4.40	61.16	5.28	-4.53	3.81E-04	8.21E-03
cg12428514	UCN3;UCN3	10	1stExon;5'UTR	Open sea	69.26	7.53	73.80	6.27	-4.53	2.96E-03	3.71E-02
cg21945989	CSN2	4	1stExon	Open sea	74.60	4.90	79.14	5.36	-4.53	6.11E-06	2.40E-04
cg21323494	CAMKK1;CAMKK1;CAMKK1	17	Body;Body;Body	S_Shore	70.37	4.78	74.90	5.47	-4.53	6.28E-05	1.89E-03
cg04842161	SKAP2	7	Body	N_Shelf	73.55	6.39	78.09	6.34	-4.53	2.87E-05	9.59E-04
cg13480010	IGFALS;IGFALS;IGFALS	16	Body;Body;Body	Island	62.20	4.46	66.74	5.03	-4.53	1.27E-04	3.40E-03
cg19717060		17	Intergenic	Open sea	18.38	4.44	22.91	5.71	-4.53	3.92E-05	1.26E-03
cg09697510		7	Intergenic	Open sea	42.94	5.35	47.47	6.53	-4.53	6.81E-04	1.28E-02
cg13209335		1	Intergenic	N_Shelf	73.60	4.96	78.13	4.72	-4.53	2.24E-05	7.71E-04
cg03665818		16	Intergenic	Open sea	65.98	6.45	70.51	4.69	-4.53	1.92E-04	4.78E-03
cg12751565	PIGR	1	TSS200	Open sea	42.35	4.51	46.88	4.71	-4.53	5.29E-05	1.63E-03
cg01206418		12	Intergenic	Open sea	73.31	4.82	77.84	5.05	-4.53	1.36E-05	4.97E-04
cg02598441	LOC146880;LOC146880	17	Body;Body	Island	20.09	5.02	24.62	5.76	-4.53	5.47E-06	2.17E-04
cg06370057		10	Intergenic	Island	24.51	4.88	29.04	5.02	-4.53	6.02E-05	1.83E-03
cg03126765		16	Intergenic	Open sea	50.03	4.82	54.56	4.87	-4.53	6.31E-05	1.90E-03
cg25729545	BAT5	6	Body	Open sea	72.46	3.94	76.99	3.21	-4.53	8.51E-08	4.27E-06
cg21156684	ACOT7;ACOT7;ACOT7;ACOT7	1	Body;Body;Body;Body	Open sea	76.47	4.51	81.00	4.82	-4.53	5.11E-05	1.58E-03
cg14142817	GAB1;GAB1	4	Body;Body	Open sea	73.31	4.39	77.84	4.39	-4.53	2.34E-06	9.90E-05
cg21139485	PILRA;PILRA;PILRA	7	Body;Body;Body	Open sea	52.13	5.10	56.65	4.48	-4.53	1.19E-04	3.23E-03
cg23688465	NDRG2;NDRG2;NDRG2;NDRG2;NDRG	14	Body;Body;Body;Body;Body;Body;Body;Bo	Open sea	34.69	3.54	39.22	4.24	-4.53	5.05E-06	2.02E-04
cg09471407		2	Intergenic	Open sea	44.19	4.90	48.72	5.73	-4.53	2.06E-04	5.05E-03
cg09119776	SUFU	10	Body	Open sea	35.80	3.94	40.32	4.61	-4.53	8.56E-06	3.27E-04
cg14030674	ANK1;ANK1;ANK1;ANK1;ANK1	8	Body;Body;Body;Body;Body	N_Shore	65.45	4.71	69.97	5.76	-4.53	5.17E-05	1.60E-03
cg09242181	LRP4	11	TSS1500	S_Shore	48.22	5.38	52.75	5.32	-4.53	2.30E-04	5.52E-03
cg09699726		16	Intergenic	Open sea	74.37	3.32	78.90	2.90	-4.53	7.78E-09	4.17E-07
cg04955914	C2orf24	2	Body	N_Shore	43.67	4.70	48.20	4.16	-4.53	2.64E-06	1.11E-04
cg02536838	ANGPT1	8	TSS200	Open sea	57.13	5.55	61.66	5.57	-4.53	8.40E-04	1.50E-02
cg15583072	SLCO1A2;IAPP	12	5'UTR;TSS200	Open sea	62.06	5.79	66.59	5.27	-4.53	9.35E-05	2.65E-03
cg17142870	C20orf95	20	Body	S_Shore	40.64	5.63	45.17	5.80	-4.53	5.49E-04	1.09E-02
cg24368912	ADH1B	4	TSS1500	Open sea	62.29	6.58	66.82	5.39	-4.53	8.50E-04	1.52E-02

cg19727801	PCDHB19P	5	TSS1500	N_Shore	17.47	4.55	22.00	5.60	-4.53	4.29E-05	1.36E-03
cg13798842	MAN2A2	15	Body	S_Shelf	69.04	3.46	73.56	4.15	-4.53	4.30E-07	2.02E-05
cg01853216	ABHD8	19	Body	Island	42.83	5.38	47.36	5.80	-4.53	2.32E-03	3.14E-02
cg06225589	GNG13	16	TSS200	S_Shore	22.32	3.78	26.85	4.58	-4.53	4.59E-06	1.85E-04
cg12292205	C6orf41	6	Body	Open sea	77.67	5.86	82.20	3.79	-4.52	9.44E-05	2.67E-03
cg19670693		15	Intergenic	Open sea	54.15	9.43	58.68	9.83	-4.52	2.35E-03	3.17E-02
cg10495164	EHD1	11	Body	Open sea	26.12	3.52	30.64	5.28	-4.52	3.76E-05	1.21E-03
cg12116027	AGAP2;AGAP2	12	Body;Body	N_Shore	30.72	4.72	35.24	4.74	-4.52	1.75E-04	4.44E-03
cg11929898	TMPRSS7;TMPRSS7	3	5'UTR;Body	Open sea	69.61	6.15	74.14	5.53	-4.52	1.18E-03	1.93E-02
cg14753458	IP6K2;IP6K2;IP6K2;IP6K2;IP6K2;IP6K2;	3	5'UTR;5'UTR;5'UTR;5'UTR;Body;5'UTR;5'U	N_Shelf	71.21	5.00	75.73	4.16	-4.52	8.18E-05	2.37E-03
cg19913551	ZFR2	19	Body	S_Shore	51.80	5.45	56.32	7.00	-4.52	4.01E-04	8.53E-03
cg19523255		4	Intergenic	Open sea	51.92	6.40	56.44	5.11	-4.52	3.95E-04	8.44E-03
cg21142272	EXOC3L	16	TSS1500	Open sea	55.04	3.97	59.56	3.55	-4.52	2.74E-06	1.15E-04
cg02631126	ZSCAN12L1	6	TSS200	Open sea	43.81	5.26	48.34	5.40	-4.52	1.99E-04	4.92E-03
cg10012623		14	Intergenic	S_Shelf	70.46	5.62	74.98	4.71	-4.52	1.18E-04	3.22E-03
cg07519235	GPRC5B	16	5'UTR	Island	34.15	7.10	38.67	7.43	-4.52	1.09E-03	1.82E-02
cg23234811	PLEKHA6	1	Body	S_Shelf	68.91	5.34	73.43	5.78	-4.52	2.30E-04	5.52E-03
cg20751398	KIRREL3;KIRREL3	11	Body;Body	Open sea	61.98	6.02	66.50	6.66	-4.52	3.31E-03	4.01E-02
cg16692227	SAMD12;SAMD12	8	Body;Body	Open sea	66.45	6.50	70.97	6.77	-4.52	1.57E-03	2.38E-02
cg03287126	MSRA;MSRA;MSRA	8	Body;Body;Body	S_Shelf	53.14	6.78	57.66	7.17	-4.52	3.74E-03	4.35E-02
cg19974879	PHRF1	11	Body	Island	43.64	3.82	48.16	5.40	-4.52	1.58E-04	4.10E-03
cg07094915		7	Intergenic	Island	34.08	3.34	38.60	3.92	-4.52	6.43E-09	3.46E-07
cg00987031		5	Intergenic	Island	31.33	6.96	35.85	7.28	-4.52	3.42E-03	4.10E-02
cg26209005	PLXNA4;PLXNA4;PLXNA4	7	TSS1500;TSS1500;5'UTR	Island	20.01	4.44	24.53	4.96	-4.52	9.66E-06	3.65E-04
cg06833613	ABR;ABR	17	Body;5'UTR	Open sea	51.84	5.71	56.35	4.95	-4.52	1.24E-03	2.01E-02
cg26636054	FAM18A	16	TSS1500	S_Shore	69.17	5.69	73.69	4.02	-4.52	6.50E-04	1.24E-02
cg24040595	HOXA6	7	Body	Island	33.33	3.89	37.84	3.69	-4.52	6.39E-06	2.50E-04
cg16810559	PRICKLE2	3	Body	Open sea	65.51	4.50	70.02	4.33	-4.52	4.45E-06	1.80E-04
cg21523613		12	Intergenic	N_Shelf	49.79	6.06	54.31	6.63	-4.52	1.17E-03	1.93E-02
cg17250296	CACNB1	17	Body	Island	43.72	5.20	48.24	6.67	-4.52	2.89E-03	3.65E-02
cg27444062	SPA17	11	Body	S_Shelf	38.26	3.91	42.78	4.75	-4.52	1.62E-05	5.79E-04
cg23818888	LOC100128292;DLG5	10	Body;TSS1500	S_Shore	31.44	3.59	35.96	4.85	-4.52	4.11E-07	1.94E-05
cg27069921		5	Intergenic	Island	39.52	5.27	44.04	6.08	-4.52	1.22E-04	3.31E-03
cg00790126		2	Intergenic	N_Shore	71.61	5.11	76.13	4.37	-4.52	1.83E-06	7.91E-05
cg24317827	RNF220	1	Body	Open sea	75.75	5.46	80.26	4.33	-4.52	1.95E-04	4.84E-03
cg26563141	RGPD2;RGPD1	2	Body;Body	N_Shore	26.31	3.52	30.83	3.55	-4.52	1.53E-07	7.51E-06
cg23978242	DIP2C	10	Body	N_Shore	20.99	6.22	25.51	6.82	-4.52	1.62E-03	2.44E-02
cg23029526	ECE1;ECE1;ECE1	1	TSS1500;TSS200;Body	Island	7.94	4.87	12.46	6.12	-4.52	2.53E-06	1.06E-04
cg13003239	WDR6	3	TSS200	Island	41.19	4.31	45.71	5.13	-4.52	5.11E-05	1.58E-03
cg07521929	SLC4A9	5	Body	N_Shore	43.73	5.17	48.24	6.56	-4.51	6.02E-05	1.83E-03
cg05147143		6	Intergenic	Open sea	45.46	5.80	49.98	5.45	-4.51	1.35E-03	2.13E-02
cg17745210	PSPH	7	5'UTR	N_Shelf	49.30	4.38	53.81	5.63	-4.51	1.64E-04	4.22E-03
cg12211557		2	Intergenic	Open sea	73.45	5.17	77.96	4.89	-4.51	3.35E-04	7.43E-03
cg09182533	MACROD1	11	Body	N_Shore	27.06	5.39	31.58	7.52	-4.51	2.79E-05	9.36E-04
cg24676384	PBXIP1	1	TSS1500	Open sea	74.64	4.78	79.15	4.03	-4.51	1.14E-05	4.23E-04
cg04399988		14	Intergenic	N_Shore	65.44	4.50	69.95	3.73	-4.51	7.39E-06	2.86E-04
cg05764010		6	Intergenic	Open sea	70.89	3.32	75.40	3.62	-4.51	1.64E-09	9.06E-08
cg03538095	IFIH1	2	Body	Open sea	70.44	5.26	74.95	5.25	-4.51	1.00E-04	2.81E-03
cg13326508	MPP2	17	TSS1500	S_Shore	31.65	6.33	36.17	6.57	-4.51	1.60E-03	2.41E-02
cg17482963	CCDC25	8	3'UTR	Open sea	82.33	4.44	86.84	4.07	-4.51	5.01E-06	2.01E-04
cg25278175	NRXN1;NRXN1	2	Body;Body	Open sea	58.28	5.21	62.79	4.42	-4.51	1.84E-05	6.50E-04
cg08848774		17	Intergenic	Island	23.08	6.02	27.60	8.40	-4.51	1.07E-03	1.80E-02
cg11647944	NTF3;NTF3	12	TSS200;Body	N_Shore	63.58	4.70	68.09	4.62	-4.51	1.82E-05	6.42E-04

cg08996613	A2BP1;A2BP1	16	5'UTR;5'UTR	Open sea	74.67	5.41	79.19	4.19	-4.51	1.47E-05	5.32E-04
cg17284963		6	Intergenic	S_Shelf	60.91	5.17	65.42	5.70	-4.51	2.98E-05	9.92E-04
cg24751707		14	Intergenic	N_Shore	35.59	5.45	40.10	6.72	-4.51	2.85E-03	3.61E-02
cg18755531		5	Intergenic	Open sea	51.85	6.44	56.36	6.23	-4.51	6.31E-04	1.21E-02
cg25045942	HLA-DPB1	6	Body	N_Shore	43.53	6.59	48.04	5.68	-4.51	4.22E-03	4.73E-02
cg17670237		4	Intergenic	Open sea	32.73	6.13	37.24	6.03	-4.51	3.43E-04	7.55E-03
cg10965581		6	Intergenic	Open sea	65.11	6.64	69.62	6.03	-4.51	2.47E-04	5.85E-03
cg09954631	ROR2	9	Body	Island	62.75	7.29	67.26	6.69	-4.51	3.38E-03	4.06E-02
cg19063834	FAM134B;FAM134B	5	Body;TSS200	Open sea	42.47	5.57	46.98	6.40	-4.51	3.38E-03	4.07E-02
cg14567374	ANAPC2;SSNA1	9	TSS1500;Body	S_Shore	47.51	3.67	52.02	4.97	-4.51	2.10E-05	7.30E-04
cg01357135	PPM1L	3	Body	S_Shore	24.75	5.67	29.25	7.02	-4.51	2.01E-03	2.84E-02
cg07133749	ADAP2	17	Body	Island	15.34	4.51	19.84	4.68	-4.51	1.32E-05	4.84E-04
cg11847992		5	Intergenic	Open sea	27.35	6.99	31.86	6.02	-4.51	2.90E-04	6.62E-03
cg12386015	RAB20	13	TSS1500	S_Shore	42.58	6.78	47.08	6.49	-4.51	3.20E-03	3.92E-02
cg21777188	PCDHB17	5	Body	N_Shore	22.87	5.92	27.38	7.48	-4.51	4.41E-03	4.88E-02
cg07128416	WDR52;WDR52	3	TSS200;TSS200	Island	47.38	6.71	51.89	8.56	-4.51	7.80E-04	1.42E-02
cg08772163	ARL11	13	TSS200	Open sea	46.97	4.57	51.47	5.07	-4.51	3.41E-06	1.40E-04
cg07435282	MRPL23	11	Body	S_Shore	31.28	4.61	35.79	6.56	-4.51	1.90E-03	2.73E-02
cg12163871	PRKD2;PRKD2;PRKD2;PRKD2	19	Body;Body;Body;Body	Island	47.31	3.96	51.82	4.46	-4.51	2.99E-06	1.24E-04
cg11302401		6	Intergenic	Open sea	30.61	4.90	35.12	5.85	-4.51	3.20E-04	7.15E-03
cg21935113	C11orf95	11	Body	Island	54.56	4.25	59.06	7.67	-4.51	1.71E-03	2.53E-02
cg11773367	SYTL2	11	5'UTR	Open sea	66.20	4.86	70.71	5.55	-4.51	1.01E-04	2.82E-03
cg10266455		2	Intergenic	Open sea	65.93	7.10	70.43	6.15	-4.51	1.43E-03	2.23E-02
cg08722122	FGFR1;FGFR1;FGFR1;FGFR1;FGFR1;FG	8	TSS1500;TSS1500;TSS1500;TSS1500;TSS15	S_Shore	54.52	5.17	59.02	5.46	-4.51	5.45E-04	1.08E-02
cg25159159	NUDT18	8	3'UTR	N_Shelf	73.71	3.97	78.22	3.53	-4.51	2.75E-07	1.32E-05
cg27640302	TIMM10	11	3'UTR	Open sea	43.02	4.32	47.53	5.65	-4.51	2.27E-04	5.48E-03
cg00885918	MYLK2	20	TSS200	N_Shelf	46.88	4.30	51.39	4.92	-4.50	6.68E-05	1.99E-03
cg11644627	INTS1	7	Body	N_Shore	74.03	4.73	78.53	4.91	-4.50	2.96E-05	9.86E-04
cg08466034	TRPV2;TRPV2	17	1stExon;5'UTR	Open sea	35.08	7.91	39.59	7.82	-4.50	1.52E-03	2.33E-02
cg17264618	ENTPD3	3	5'UTR	Island	32.60	5.82	37.11	4.68	-4.50	5.95E-04	1.16E-02
cg00750518	DCTN2	12	Body	N_Shore	64.40	4.23	68.90	4.02	-4.50	4.34E-06	1.76E-04
cg15317267	H19;MIR675	11	Body;TSS200	Island	51.30	3.16	55.80	4.67	-4.50	4.01E-06	1.63E-04
cg14220634	ZNF169	9	TSS1500	N_Shore	58.57	3.53	63.07	3.96	-4.50	1.36E-07	6.70E-06
cg18430465	TRPS1	8	3'UTR	Open sea	57.10	7.20	61.61	7.10	-4.50	4.32E-04	9.05E-03
cg22946959	ENOX1;ENOX1	13	Body;Body	Open sea	78.04	5.92	82.55	5.25	-4.50	8.64E-04	1.53E-02
cg04310331	LOC100133545	11	TSS1500	N_Shore	41.21	4.37	45.71	5.49	-4.50	3.79E-05	1.22E-03
cg22132509	PPM1L	3	Body	S_Shore	38.01	5.65	42.51	7.20	-4.50	1.12E-03	1.87E-02
cg19341992	FAM90A1	12	TSS1500	Open sea	46.07	5.54	50.57	5.74	-4.50	4.37E-04	9.13E-03
cg01445052	LHX9;LHX9;LHX9	1	5'UTR;1stExon;Body	N_Shore	17.50	3.54	22.00	3.40	-4.50	6.78E-09	3.64E-07
cg09663608		15	Intergenic	Open sea	39.93	5.03	44.43	4.98	-4.50	3.09E-04	6.95E-03
cg03716388	MDFIC;MDFIC	7	Body;Body	Open sea	78.74	4.14	83.24	3.75	-4.50	4.13E-07	1.95E-05
cg12699599	PHGR1	15	TSS200	Open sea	38.39	5.86	42.89	5.50	-4.50	1.57E-04	4.06E-03
cg02350039	NHSL1;NHSL1	6	Body;Body	Open sea	76.10	4.21	80.60	3.37	-4.50	4.87E-06	1.95E-04
cg02194684	PDZD2	5	Body	N_Shore	49.88	5.16	54.38	6.87	-4.50	1.66E-03	2.48E-02
cg23172664	NT5E	6	Body	Open sea	68.41	6.20	72.91	4.73	-4.50	2.03E-04	5.00E-03
cg25706447	PIGV	1	Body	S_Shelf	80.73	4.28	85.23	2.93	-4.50	3.46E-08	1.79E-06
cg03539789	CCDC140	2	5'UTR	N_Shore	33.86	4.22	38.36	5.16	-4.50	3.65E-05	1.18E-03
cg02331769	SLFNL1;SLFNL1	1	TSS1500;TSS1500	Open sea	47.84	3.78	52.34	4.64	-4.50	1.76E-05	6.24E-04
cg03434509		8	Intergenic	Island	44.62	6.44	49.12	6.34	-4.50	1.51E-04	3.94E-03
cg23035602	ZNF761;LOC147804	19	TSS200;TSS200	Island	16.36	4.11	20.86	5.36	-4.50	6.96E-05	2.06E-03
cg07180197	TRIM55;TRIM55;TRIM55;TRIM55	8	TSS200;TSS200;TSS200;TSS200	Open sea	16.83	3.41	21.33	6.14	-4.50	2.58E-04	6.06E-03
cg01514356		8	Intergenic	N_Shelf	61.47	6.72	65.97	6.45	-4.50	8.86E-04	1.56E-02
cg03676569	BTAF1	10	Body	Open sea	70.35	5.53	74.84	4.64	-4.50	4.05E-04	8.60E-03

cg15393221	PRX;PRX	19	TSS1500;TSS1500	Open sea	46.30	5.15	50.80	4.39	-4.50	3.80E-04	8.19E-03
cg10378538		6	Intergenic	Open sea	70.40	4.22	74.89	5.05	-4.50	6.90E-06	2.68E-04
cg14839837	ARHGEF10	8	Body	S_Shelf	53.45	4.74	57.95	6.60	-4.50	4.04E-04	8.58E-03
cg09735579	FGF9	13	Body	N_Shore	29.50	4.56	33.99	5.00	-4.50	4.23E-06	1.72E-04
cg08737246	MBP;MBP	18	Body;Body	Island	54.44	4.30	58.94	4.93	-4.50	4.98E-05	1.55E-03
cg09588186		3	Intergenic	Open sea	38.14	4.44	42.64	6.36	-4.50	4.70E-04	9.65E-03
cg23299484		11	Intergenic	Open sea	41.48	4.71	45.97	3.50	-4.50	5.91E-05	1.80E-03
cg26347089	VWA3B	2	Body	Open sea	77.95	6.67	82.44	6.37	-4.49	1.04E-03	1.77E-02
cg01254991		4	Intergenic	S_Shelf	59.10	4.91	63.59	3.79	-4.49	1.25E-05	4.59E-04
cg07196426	TFAP2E	1	Body	Island	58.10	5.70	62.60	5.65	-4.49	1.47E-03	2.27E-02
cg22636056		11	Intergenic	N_Shelf	67.15	4.04	71.64	3.04	-4.49	1.13E-06	5.04E-05
cg12446613		5	Intergenic	Open sea	54.50	5.28	58.99	4.30	-4.49	1.69E-05	6.02E-04
cg11401293	COL6A1	21	Body	Island	65.06	8.04	69.55	8.02	-4.49	1.06E-03	1.79E-02
cg01747969	DLX6AS	7	Body	Open sea	54.91	5.24	59.40	5.53	-4.49	6.81E-06	2.65E-04
cg18846889	CCDC85C	14	Body	Open sea	42.51	5.21	47.01	4.26	-4.49	2.89E-04	6.61E-03
cg13800496	UTRN	6	Body	Open sea	59.21	4.97	63.70	4.64	-4.49	1.53E-05	5.53E-04
cg02327604	HEATR2	7	Body	Island	58.56	6.49	63.05	7.04	-4.49	7.02E-04	1.31E-02
cg11135080	NFYC;NFYC;NFYC;NFYC;NFYC;MIR30C	1	Body;Body;Body;Body;Body;TSS200	Open sea	48.18	5.60	52.67	5.80	-4.49	1.56E-03	2.37E-02
cg22296149	AMH;AMH	19	5'UTR;1stExon	N_Shore	55.77	4.71	60.26	4.56	-4.49	1.96E-04	4.85E-03
cg00549040		2	Intergenic	Open sea	51.15	3.40	55.64	3.47	-4.49	9.97E-08	4.97E-06
cg08055124		4	Intergenic	S_Shelf	60.06	6.23	64.55	5.48	-4.49	4.47E-04	9.29E-03
cg02119091		8	Intergenic	N_Shore	45.06	6.56	49.55	6.38	-4.49	4.28E-03	4.78E-02
cg03566787	CUGBP1;CUGBP1	11	TSS1500;5'UTR	Open sea	39.86	4.05	44.35	4.65	-4.49	3.08E-06	1.28E-04
cg24811864	ST8SIA3	18	TSS200	Island	38.14	4.70	42.63	5.88	-4.49	7.26E-06	2.81E-04
cg15378605	CHST15	10	1stExon	S_Shore	68.86	7.35	73.35	7.75	-4.49	1.02E-03	1.74E-02
cg16293656	FGF17;FGF17	8	5'UTR;1stExon	Open sea	25.39	4.37	29.88	4.70	-4.49	2.93E-06	1.22E-04
cg13582775		8	Intergenic	S_Shore	52.60	5.63	57.09	6.22	-4.49	3.07E-04	6.92E-03
cg03071098	OSR1	2	5'UTR	N_Shore	61.84	5.06	66.33	4.47	-4.49	1.40E-04	3.70E-03
cg26916862		7	Intergenic	S_Shore	14.75	6.85	19.24	8.18	-4.49	4.01E-04	8.53E-03
cg09225701		4	Intergenic	Open sea	41.66	4.62	46.15	4.93	-4.49	1.83E-04	4.61E-03
cg19958890	SNTN	3	TSS1500	Open sea	51.82	4.95	56.31	6.50	-4.49	5.21E-04	1.05E-02
cg27062571	FER1L6	8	5'UTR	Open sea	29.65	4.94	34.14	6.20	-4.49	8.62E-05	2.47E-03
cg03103023	CLIC4	1	Body	Open sea	68.43	3.40	72.92	3.65	-4.49	8.29E-07	3.76E-05
cg11801110	PIM3	22	Body	Island	25.98	3.20	30.47	4.34	-4.49	2.51E-06	1.06E-04
cg27304533	NFIX	19	Body	Island	55.30	3.97	59.79	4.68	-4.49	8.17E-06	3.14E-04
cg23971915	TMIE;TMIE	3	1stExon;5'UTR	Island	17.64	4.68	22.13	5.45	-4.49	7.73E-06	2.98E-04
cg03596034	RNF135;RNF135	17	TSS1500;TSS1500	N_Shore	19.29	5.20	23.77	5.55	-4.49	3.89E-04	8.33E-03
cg00460268	ZMYND10	3	TSS200	Island	20.35	4.37	24.83	4.87	-4.49	1.12E-05	4.16E-04
cg11193342		12	Intergenic	Open sea	18.73	3.81	23.22	5.91	-4.49	1.17E-04	3.19E-03
cg25476262	ARRDC2;ARRDC2	19	Body;Body	S_Shore	85.02	4.35	89.51	3.09	-4.49	6.40E-09	3.44E-07
cg17434634		19	Intergenic	Island	27.58	6.11	32.07	6.52	-4.49	1.73E-03	2.56E-02
cg03753942		8	Intergenic	Open sea	34.60	4.66	39.09	5.07	-4.49	1.11E-04	3.06E-03
cg06513139	UBXN6;UBXN6	19	Body;TSS1500	N_Shore	36.54	4.94	41.02	7.07	-4.49	3.32E-03	4.02E-02
cg01067216		14	Intergenic	Island	67.87	6.49	72.35	6.74	-4.49	1.81E-03	2.64E-02
cg02072400		12	Intergenic	Island	15.66	4.15	20.15	6.52	-4.49	5.48E-04	1.09E-02
cg04890036	VEZF1	17	Body	Open sea	63.13	7.32	67.61	6.40	-4.49	1.18E-03	1.93E-02
cg08827122	SNX8	7	Body	N_Shelf	73.57	4.97	78.05	4.62	-4.48	1.40E-04	3.70E-03
cg13702536	GPR81	12	TSS1500	Open sea	51.81	6.23	56.29	5.38	-4.48	1.09E-03	1.82E-02
cg22227345		15	Intergenic	N_Shore	23.13	3.07	27.62	5.36	-4.48	3.13E-06	1.30E-04
cg13305652		7	Intergenic	Open sea	72.14	3.07	76.63	3.23	-4.48	1.00E-08	5.32E-07
cg06090684	UTP20	12	TSS1500	N_Shore	44.71	4.70	49.20	5.93	-4.48	5.71E-04	1.12E-02
cg26394257	ADAM32	8	TSS200	N_Shore	37.57	5.36	42.05	7.42	-4.48	9.92E-04	1.70E-02
cg09168997	ATP1A2	1	TSS1500	Open sea	51.96	3.53	56.45	4.74	-4.48	6.74E-06	2.63E-04

cg21583440	GDPD5	11	Body	Open sea	57.34	5.14	61.82	5.10	-4.48	3.04E-05	1.01E-03
cg15041662		17	Intergenic	Open sea	55.46	6.23	59.94	6.04	-4.48	2.34E-03	3.16E-02
cg27660490	TULP3;TULP3	12	Body;3'UTR	Open sea	60.78	4.93	65.26	4.80	-4.48	9.60E-05	2.71E-03
cg03895540	OR52N2	11	TSS200	Open sea	53.82	6.66	58.30	6.53	-4.48	1.07E-03	1.81E-02
cg24199203	NFIC;NFIC	19	Body;Body	N_Shore	22.33	4.46	26.81	6.80	-4.48	1.04E-03	1.76E-02
cg12635848		8	Intergenic	Open sea	72.72	6.65	77.20	5.60	-4.48	1.38E-03	2.17E-02
cg04303509	ZNF792	19	Body	N_Shore	66.33	4.71	70.81	3.84	-4.48	3.31E-06	1.37E-04
cg10280342	PSPN	19	TSS1500	S_Shelf	49.23	3.78	53.71	3.99	-4.48	3.62E-06	1.49E-04
cg24866923	WDR72	15	5'UTR	N_Shore	50.62	5.76	55.10	6.89	-4.48	1.81E-03	2.64E-02
cg15630071	TRIM71	3	Body	Island	39.16	4.03	43.64	5.72	-4.48	1.92E-04	4.78E-03
cg18909389	CLIC1	6	Body	S_Shelf	40.99	5.96	45.47	6.49	-4.48	2.97E-03	3.71E-02
cg07795916		2	Intergenic	Open sea	72.89	6.38	77.37	5.77	-4.48	4.12E-04	8.73E-03
cg19732886		10	Intergenic	N_Shelf	75.34	4.58	79.82	4.00	-4.48	1.07E-05	3.99E-04
cg06782350	DNAJC17	15	Body	S_Shelf	80.67	4.61	85.15	3.56	-4.48	3.16E-06	1.31E-04
cg08842907	BSCL2;BSCL2;BSCL2	11	Body;Body;Body	N_Shelf	64.88	4.45	69.36	3.60	-4.48	5.60E-06	2.22E-04
cg11224783		19	Intergenic	N_Shelf	50.59	6.68	55.07	6.65	-4.48	1.12E-03	1.87E-02
cg10656016	PLCXD2	3	Body	Open sea	69.01	6.82	73.49	5.22	-4.48	2.61E-03	3.40E-02
cg05335876	TNFSF18	1	Body	Open sea	72.53	6.08	77.01	5.78	-4.48	3.49E-04	7.67E-03
cg09461494	VPS13B;VPS13B	8	Body;Body	Open sea	76.08	7.08	80.56	6.18	-4.48	5.60E-04	1.10E-02
cg04918831	CHML;CHML;OPN3	1	3'UTR;1stExon;Body	Open sea	48.54	5.37	53.01	6.47	-4.48	4.53E-03	4.97E-02
cg09513416		6	Intergenic	Open sea	63.69	5.43	68.17	4.61	-4.48	2.67E-04	6.22E-03
cg06235329	ANKS3	16	Body	S_Shelf	53.75	4.22	58.22	4.12	-4.48	1.77E-05	6.28E-04
cg12715235	PAG1	8	TSS1500	S_Shore	78.08	4.62	82.56	4.09	-4.48	1.61E-05	5.76E-04
cg06767637	UBE2O	17	Body	Open sea	57.25	4.95	61.73	4.03	-4.48	2.96E-04	6.72E-03
cg10266524		19	Intergenic	S_Shelf	66.71	6.39	71.19	5.95	-4.48	2.19E-04	5.30E-03
cg22445742	BCL11A;BCL11A	2	Body;Body	Open sea	57.60	5.48	62.07	6.37	-4.48	1.94E-03	2.76E-02
cg24795748	COL6A1	21	Body	Island	75.70	5.08	80.18	5.08	-4.48	3.19E-06	1.32E-04
cg01722963		16	Intergenic	Island	77.98	6.59	82.46	4.81	-4.48	2.14E-04	5.21E-03
cg00782828		1	Intergenic	Island	40.49	4.14	44.97	4.84	-4.48	1.14E-05	4.23E-04
cg08428292		16	Intergenic	N_Shore	29.66	4.85	34.14	6.44	-4.48	7.55E-04	1.39E-02
cg18264932		7	Intergenic	Open sea	68.06	5.00	72.54	6.55	-4.48	8.20E-04	1.47E-02
cg20690774	PRDM11	11	Body	Island	20.60	5.83	25.07	6.00	-4.48	6.90E-05	2.05E-03
cg21549437	PIAS1	15	Body	Open sea	83.60	5.01	88.07	3.73	-4.47	6.42E-05	1.93E-03
cg08834922	AOC3;AOC3	17	1stExon;5'UTR	Open sea	39.29	5.85	43.76	5.60	-4.47	2.73E-04	6.33E-03
cg18306861	PLEK2	14	TSS1500	Island	22.78	3.83	27.25	4.49	-4.47	2.50E-06	1.05E-04
cg12244052	IGFBP3;IGFBP3	7	TSS1500;TSS1500	S_Shore	23.18	4.74	27.66	5.50	-4.47	1.84E-05	6.50E-04
cg18777299	STIM2;STIM2;STIM2	4	Body;Body;Body	Open sea	27.30	5.52	31.77	6.43	-4.47	2.44E-03	3.25E-02
cg24734281		14	Intergenic	Open sea	74.92	5.22	79.40	5.27	-4.47	1.60E-03	2.41E-02
cg12798157	AKR7L;AKR7L	1	TSS200;TSS200	Island	7.30	3.37	11.77	6.71	-4.47	1.47E-03	2.27E-02
cg22077553	MYOC	1	1stExon	Open sea	56.46	5.27	60.93	5.17	-4.47	4.69E-04	9.65E-03
cg07953201	KIF26B	1	Body	Island	24.57	4.70	29.04	7.63	-4.47	4.40E-03	4.87E-02
cg09994356	GDAP1L1	20	TSS200	Island	19.27	5.06	23.74	5.65	-4.47	8.80E-05	2.52E-03
cg25602543	ZSWIM3	20	3'UTR	Open sea	74.41	5.34	78.88	4.33	-4.47	5.40E-04	1.07E-02
cg21887097	LAMB1	7	Body	Open sea	41.63	7.82	46.10	6.56	-4.47	2.27E-03	3.09E-02
cg06088032	ZMYND10	3	TSS200	Island	14.76	4.46	19.23	4.96	-4.47	6.73E-06	2.62E-04
cg15482717	FADD	11	3'UTR	S_Shelf	71.29	4.12	75.76	3.46	-4.47	9.15E-06	3.47E-04
cg26010182	CHD1L	1	TSS1500	N_Shore	68.15	6.32	72.62	5.48	-4.47	3.46E-03	4.13E-02
cg00091760		10	Intergenic	N_Shelf	57.15	5.56	61.62	3.62	-4.47	2.17E-05	7.52E-04
cg19607845	FBXO9;FBXO9;FBXO9	6	Body;5'UTR;TSS1500	S_Shelf	32.33	5.50	36.80	6.32	-4.47	1.53E-03	2.34E-02
cg15431659	C3orf21	3	Body	Open sea	46.61	4.86	51.07	5.09	-4.47	2.21E-04	5.34E-03
cg06791107	CLRN2	4	TSS200	S_Shelf	37.57	4.77	42.04	6.11	-4.47	6.06E-04	1.18E-02
cg01314555	TMPRSS11E	4	Body	Open sea	75.33	7.15	79.80	7.03	-4.47	1.30E-03	2.08E-02
cg17667035		10	Intergenic	Open sea	52.06	5.49	56.53	5.91	-4.47	1.34E-03	2.13E-02

cg01816714	TEX2	17	Body	S_Shelf	60.03	6.29	64.49	6.67	-4.47	2.67E-03	3.45E-02
cg01174441	C18orf26	18	Body	Open sea	46.71	5.58	51.18	5.87	-4.47	1.56E-03	2.37E-02
cg18181229	PBX1	1	Body	Island	60.56	6.12	65.03	6.06	-4.47	1.66E-03	2.48E-02
cg15313859	LMOD2	7	TSS1500	Open sea	41.72	5.55	46.19	5.67	-4.47	6.44E-04	1.23E-02
cg13681468	GPRIN3	4	5'UTR	N_Shore	69.38	6.68	73.85	5.70	-4.47	4.15E-04	8.77E-03
cg26478079	ATP6V1G2;NFKBIL1;NFKBIL1;ATP6V1G	6	Body;TSS1500;TSS1500;Body	S_Shelf	66.90	4.81	71.37	3.89	-4.47	6.25E-05	1.89E-03
cg01782709	HLA-DMA	6	Body	Open sea	66.86	6.00	71.32	7.19	-4.47	3.76E-03	4.37E-02
cg05720491		17	Intergenic	Open sea	51.29	5.60	55.76	6.19	-4.47	2.49E-03	3.29E-02
cg26635346		5	Intergenic	Open sea	76.18	7.36	80.64	7.81	-4.47	1.92E-03	2.74E-02
cg11182199	LRP11	6	Body	Open sea	38.40	7.25	42.87	6.58	-4.47	2.73E-03	3.51E-02
cg19426522		3	Intergenic	S_Shore	29.60	5.59	34.07	6.20	-4.47	2.54E-03	3.33E-02
cg17803965	SPOCD1	1	TSS1500	Open sea	40.55	4.62	45.02	4.23	-4.47	4.09E-05	1.31E-03
cg16665383	C8orf37	8	3'UTR	Open sea	67.12	5.46	71.59	4.58	-4.47	1.10E-04	3.03E-03
cg09484541	FAM169A	5	5'UTR	N_Shore	55.09	6.75	59.56	5.76	-4.46	2.99E-04	6.78E-03
cg23020600	KIAA1026;KIAA1026;KIAA1026;KIAA10	1	Body;TSS200;Body;Body	Open sea	51.74	4.97	56.20	5.15	-4.46	3.98E-04	8.49E-03
cg11017947		22	Intergenic	N_Shelf	72.48	6.47	76.94	5.12	-4.46	4.63E-05	1.46E-03
cg03290131	DUSP5	10	Body	Open sea	32.50	5.44	36.97	5.90	-4.46	2.01E-04	4.95E-03
cg07450633		5	Intergenic	N_Shore	54.76	4.06	59.22	4.60	-4.46	1.25E-05	4.59E-04
cg27344587	LOC100132215	2	TSS1500	Island	28.78	4.26	33.24	4.90	-4.46	1.90E-05	6.68E-04
cg25793628	ABCA5;ABCA5	17	TSS200;5'UTR	Open sea	51.83	4.10	56.30	4.06	-4.46	8.85E-06	3.37E-04
cg21780711		5	Intergenic	Open sea	47.33	5.96	51.79	6.73	-4.46	3.70E-03	4.33E-02
cg17297238	PCDHGA4;PCDHGC3;PCDHGA12;PCDH	5	Body;Body;Body;Body;Body;Body;Body;Bo	Island	33.46	5.16	37.92	5.00	-4.46	1.28E-04	3.42E-03
cg03739955		6	Intergenic	Island	19.72	5.02	24.18	6.69	-4.46	1.06E-03	1.79E-02
cg10451566	DLL4	15	Body	Island	77.96	4.17	82.42	3.81	-4.46	3.73E-06	1.53E-04
cg18035534	SLC19A1	21	5'UTR	S_Shore	83.82	7.28	88.28	6.06	-4.46	3.69E-04	8.00E-03
cg03954858	FAM83F	22	Body	Open sea	52.25	4.58	56.71	4.19	-4.46	1.22E-06	5.43E-05
cg01081263	SCUBE2;SCUBE2	11	Body;Body	Island	46.67	4.87	51.14	5.55	-4.46	3.29E-04	7.31E-03
cg17653886	SLFN13	17	TSS1500	N_Shore	30.88	4.89	35.34	6.16	-4.46	4.65E-04	9.59E-03
cg00407820	LRP11	6	Body	N_Shelf	54.02	5.38	58.48	5.47	-4.46	1.14E-03	1.89E-02
cg11267065	PISD	22	TSS200	S_Shore	8.41	2.23	12.87	3.73	-4.46	1.36E-11	8.07E-10
cg15819471	RFX4;RFX4	12	Body;Body	Open sea	64.40	5.01	68.87	4.98	-4.46	6.97E-05	2.07E-03
cg27208169	RAI1	17	5'UTR	Island	64.74	6.41	69.20	4.91	-4.46	8.73E-04	1.55E-02
cg03437675	TRPV4;TRPV4	12	TSS1500;TSS1500	S_Shore	57.22	5.17	61.69	5.27	-4.46	1.39E-04	3.68E-03
cg11699265	ARPC1B	7	Body	Island	39.71	5.10	44.17	4.83	-4.46	1.96E-04	4.86E-03
cg06711298	SEMA4C	2	Body	Island	72.89	4.00	77.35	4.00	-4.46	1.20E-05	4.42E-04
cg15690853	MBNL2;MBNL2	13	5'UTR;5'UTR	Open sea	51.36	4.86	55.82	5.95	-4.46	2.46E-04	5.83E-03
cg23042148	DNMT3A;DNMT3A	2	Body;Body	S_Shore	50.34	4.74	54.80	4.86	-4.46	1.63E-05	5.83E-04
cg03107393	HOXB8	17	TSS1500	S_Shore	19.70	3.72	24.16	3.88	-4.46	7.96E-07	3.62E-05
cg01101782	KDM5B	1	TSS1500	N_Shore	60.36	6.89	64.82	4.99	-4.46	1.11E-03	1.85E-02
cg02958263	RABGAP1L	1	Body	Open sea	41.35	8.49	45.80	9.92	-4.46	2.94E-03	3.69E-02
cg14064774	FRYL	4	Body	Open sea	74.56	6.57	79.02	5.89	-4.46	8.66E-04	1.54E-02
cg02481669	MCCD1	6	TSS200	Open sea	49.10	4.39	53.55	6.02	-4.46	7.57E-04	1.39E-02
cg19646028	MIR7-3;C19orf30	19	TSS1500;Body	Open sea	21.73	4.32	26.19	4.57	-4.46	2.62E-06	1.10E-04
cg15150550	DISP1	1	Body	Open sea	77.61	5.66	82.07	4.08	-4.46	2.52E-04	5.95E-03
cg19050152		5	Intergenic	Open sea	59.58	5.97	64.04	7.29	-4.46	8.98E-04	1.58E-02
cg03788230		10	Intergenic	Island	75.74	4.23	80.20	4.24	-4.46	2.07E-06	8.86E-05
cg01574788	SNX29	16	Body	Open sea	63.92	6.13	68.38	4.90	-4.46	2.76E-04	6.38E-03
cg07184658		9	Intergenic	Island	25.13	4.61	29.59	4.78	-4.46	4.39E-05	1.39E-03
cg26494441	RFPL2	22	TSS1500	Open sea	54.91	5.89	59.37	5.65	-4.46	4.25E-04	8.94E-03
cg03146625	HOXC4;HOXC4	12	Body;Body	S_Shore	42.40	5.65	46.86	5.03	-4.46	6.67E-04	1.26E-02
cg25216821	RREB1;RREB1;RREB1;RREB1	6	Body;Body;Body;Body	S_Shore	87.81	4.62	92.26	2.07	-4.46	2.59E-06	1.09E-04
cg15771416		10	Intergenic	Open sea	60.67	5.22	65.13	5.39	-4.46	8.15E-04	1.47E-02
cg21367811	HOXA4	7	Body	N_Shore	26.04	4.19	30.50	5.34	-4.46	3.87E-05	1.25E-03

cg07143805	PMEPA1;PMEPA1;PMEPA1	20	TSS1500;TSS1500;TSS200	S_Shore	58.42	4.87	62.88	5.36	-4.46	6.91E-04	1.30E-02
cg14459158		9	Intergenic	N_Shore	64.46	6.90	68.92	7.04	-4.46	3.41E-03	4.10E-02
cg12769599		11	Intergenic	Open sea	23.54	4.84	27.99	7.32	-4.45	2.02E-03	2.85E-02
cg06955998	KLC1;KLC1;KLC1	14	Body;Body;Body	Open sea	68.18	4.68	72.63	4.45	-4.45	2.79E-05	9.37E-04
cg01040719		17	Intergenic	Open sea	51.10	6.50	55.56	5.61	-4.45	3.55E-03	4.21E-02
cg00101940		16	Intergenic	Open sea	82.27	5.50	86.72	3.19	-4.45	6.98E-05	2.07E-03
cg19695266	ACAP3	1	Body	N_Shore	29.13	4.81	33.58	3.89	-4.45	1.38E-05	5.02E-04
cg08297985		16	Intergenic	Open sea	65.84	5.61	70.29	6.19	-4.45	8.90E-05	2.54E-03
cg02727079	KIF13A;KIF13A;KIF13A;KIF13A	6	Body;Body;Body;Body	N_Shore	28.60	4.37	33.06	6.00	-4.45	2.13E-04	5.20E-03
cg08993283		7	Intergenic	Open sea	38.67	5.69	43.12	5.04	-4.45	3.76E-04	8.13E-03
cg00505001	MAB21L1;MIR548F5;NBEA	13	1stExon;Body;Body	Island	17.00	3.94	21.45	7.80	-4.45	8.44E-04	1.51E-02
cg15925478	BCAR3	1	Body	Open sea	73.08	7.63	77.53	6.22	-4.45	8.28E-04	1.49E-02
cg13044052	TRIM40	6	TSS1500	Open sea	29.33	5.76	33.78	6.78	-4.45	1.02E-03	1.74E-02
cg14570307	ZNF783	7	Body	Island	11.82	3.06	16.27	4.88	-4.45	1.67E-07	8.16E-06
cg22379573	DENND1B;DENND1B	1	Body;Body	Open sea	77.71	4.88	82.16	3.88	-4.45	7.70E-06	2.97E-04
cg15630458		8	Intergenic	S_Shore	32.28	5.57	36.73	5.92	-4.45	2.72E-03	3.50E-02
cg09062708	NFIA;NFIA;NFIA;NFIA	1	Body;Body;Body;Body	Open sea	25.44	4.72	29.89	5.92	-4.45	1.45E-03	2.25E-02
cg16844539	CUL3	2	Body	Open sea	83.80	5.60	88.25	3.58	-4.45	2.83E-05	9.48E-04
cg00405484	EIF2C2;EIF2C2	8	Body;Body	N_Shore	70.72	4.14	75.16	3.21	-4.45	1.44E-06	6.32E-05
cg10403161		10	Intergenic	S_Shore	52.99	4.31	57.44	4.19	-4.45	9.88E-06	3.72E-04
cg10682957		11	Intergenic	Open sea	33.08	4.91	37.53	6.80	-4.45	1.95E-03	2.78E-02
cg08368055	DHX38;TXNL4B	16	5'UTR;TSS1500	S_Shore	61.99	4.37	66.44	3.46	-4.45	4.41E-06	1.79E-04
cg06907356		21	Intergenic	S_Shore	44.61	12.26	49.06	10.04	-4.45	3.89E-03	4.48E-02
cg08662766	PLIN5	19	TSS1500	S_Shore	45.98	6.20	50.43	5.62	-4.45	2.89E-03	3.64E-02
cg01831160	IQSEC1	3	Body	N_Shore	67.33	4.57	71.78	4.52	-4.45	8.57E-06	3.28E-04
cg14974749	HOXA5	7	Body	N_Shore	42.77	4.74	47.22	6.11	-4.45	1.31E-03	2.10E-02
cg25820703	GGN	19	Body	Island	52.26	6.29	56.71	6.88	-4.45	5.83E-04	1.14E-02
cg26939946	RNF112	17	TSS200	Open sea	44.60	5.08	49.04	5.17	-4.45	3.81E-04	8.20E-03
cg10257332	DHX30;DHX30	3	Body;Body	S_Shelf	33.92	4.16	38.37	4.60	-4.45	1.79E-05	6.32E-04
cg13733615		9	Intergenic	S_Shore	51.87	4.74	56.31	6.63	-4.45	7.82E-04	1.42E-02
cg09887589	CLIC1	6	Body	Open sea	50.78	6.48	55.23	6.81	-4.45	5.17E-05	1.60E-03
cg04621521	CYP3A7;CYP3A7	7	5'UTR;1stExon	Open sea	77.17	8.31	81.62	6.35	-4.45	2.34E-03	3.16E-02
cg19837124	MRPL23	11	Body	Island	37.95	5.06	42.40	6.74	-4.45	2.93E-03	3.68E-02
cg07841173	GATA2	3	5'UTR	Island	49.09	4.58	53.54	4.44	-4.45	9.25E-06	3.51E-04
cg17379828	NAT14;SSC5D	19	3'UTR;TSS1500	N_Shore	59.49	3.87	63.94	4.45	-4.45	1.75E-05	6.22E-04
cg23918610	EXT1	8	Body	Open sea	53.23	5.95	57.68	6.53	-4.45	3.87E-03	4.47E-02
cg16340030	STX3	11	Body	Open sea	41.04	4.66	45.49	5.92	-4.45	3.34E-05	1.09E-03
cg21606696	BCL3	19	Body	N_Shore	38.23	4.66	42.68	4.98	-4.45	1.01E-04	2.83E-03
cg18295744	ZMIZ1	10	5'UTR	Open sea	71.17	4.82	75.61	4.46	-4.45	2.20E-06	9.37E-05
cg13081498	HSPB7	1	TSS200	Open sea	58.93	6.90	63.38	4.94	-4.45	2.40E-04	5.71E-03
cg09953520	EBF2	8	Body	Open sea	44.56	5.90	49.01	5.32	-4.44	3.13E-04	7.02E-03
cg18905668	GTF2H1;GTF2H1	11	3'UTR;3'UTR	Open sea	60.55	4.83	64.99	3.95	-4.44	9.43E-06	3.57E-04
cg27359566		16	Intergenic	N_Shore	26.44	5.12	30.88	6.40	-4.44	1.53E-03	2.34E-02
cg02004044	MIR1250;AATK	17	TSS200;Body	N_Shelf	78.13	6.26	82.57	4.70	-4.44	1.87E-04	4.69E-03
cg20615117	SORL1	11	Body	Open sea	74.62	4.51	79.06	3.15	-4.44	2.70E-06	1.13E-04
cg22304799		2	Intergenic	Open sea	35.41	5.29	39.85	5.19	-4.44	8.44E-04	1.51E-02
cg08719712	KLF14	7	TSS1500	S_Shore	38.08	5.60	42.53	6.48	-4.44	9.03E-05	2.57E-03
cg18385190		14	Intergenic	Island	50.38	5.86	54.83	6.32	-4.44	3.58E-03	4.23E-02
cg00018184	S100A3	1	3'UTR	Open sea	62.04	4.92	66.49	6.40	-4.44	1.32E-03	2.10E-02
cg14130664	FRMD3	9	Body	N_Shore	17.04	4.91	21.49	7.09	-4.44	1.52E-03	2.32E-02
cg22154659	HOXA1;HOXA1	7	3'UTR;Body	N_Shore	41.59	5.51	46.03	4.99	-4.44	1.08E-03	1.81E-02
cg03011822	C10orf55;C10orf55	10	1stExon;5'UTR	Open sea	79.75	5.36	84.19	4.08	-4.44	7.37E-06	2.85E-04
cg00950265		14	Intergenic	N_Shore	22.27	3.67	26.71	3.83	-4.44	2.29E-07	1.11E-05

cg14243616		9	Intergenic	S_Shelf	62.27	5.58	66.71	4.52	-4.44	5.88E-04	1.15E-02
cg13711144	BCAP29;DUS4L;BCAP29	7	TSS1500;3'UTR;TSS1500	N_Shore	76.18	6.56	80.62	6.41	-4.44	3.08E-03	3.81E-02
cg25214900	SYT1;SYT1;SYT1	12	Body;Body;Body	Open sea	42.47	5.64	46.91	5.54	-4.44	2.21E-03	3.03E-02
cg01631277	STYX;STYX	14	Body;Body	Open sea	51.04	6.47	55.48	6.27	-4.44	3.45E-03	4.12E-02
cg00968267		13	Intergenic	Open sea	73.78	5.63	78.22	5.85	-4.44	1.06E-03	1.79E-02
cg02876221		4	Intergenic	Open sea	49.77	5.49	54.21	6.89	-4.44	3.37E-03	4.06E-02
cg19539826	PDE4D;PDE4D;PDE4D	5	Body;Body;Body	Open sea	47.75	6.29	52.19	6.43	-4.44	4.18E-03	4.70E-02
cg24261333	PLEKHB1;PLEKHB1	11	TSS1500;TSS1500	Open sea	18.45	4.09	22.89	6.89	-4.44	5.15E-04	1.04E-02
cg27382405	KCNH3	12	Body	Island	22.28	5.69	26.72	6.81	-4.44	3.54E-03	4.20E-02
cg06225294	ZBTB20;ZBTB20;ZBTB20;ZBTB20;ZBTB	3	5'UTR;1stExon;5'UTR;5'UTR;5'UTR;1stExor	Open sea	28.68	4.85	33.11	5.61	-4.44	1.82E-03	2.65E-02
cg15899743		14	Intergenic	Open sea	71.94	4.21	76.38	4.38	-4.44	2.94E-06	1.22E-04
cg12198769	COX5B	2	TSS200	N_Shore	16.02	3.40	20.45	4.66	-4.44	1.15E-05	4.28E-04
cg16272084	HIP1	7	Body	Open sea	72.32	6.32	76.75	6.38	-4.44	6.64E-05	1.98E-03
cg02895447	SYT7	11	Body	S_Shore	63.44	5.64	67.88	4.74	-4.44	3.68E-04	7.98E-03
cg11994390		7	Intergenic	Open sea	69.89	6.17	74.33	5.04	-4.44	9.48E-04	1.65E-02
cg10551804	C3orf45	3	3'UTR	Open sea	68.83	3.88	73.27	4.24	-4.44	6.46E-06	2.52E-04
cg08967764		3	Intergenic	Open sea	41.62	7.39	46.06	7.31	-4.44	2.76E-03	3.53E-02
cg13990129	FSD1L	9	3'UTR	Open sea	75.15	7.84	79.58	8.29	-4.44	4.38E-03	4.85E-02
cg26426745	LOC100132111;C2CD4D	1	TSS1500;Body	Island	27.96	5.09	32.40	7.70	-4.44	4.38E-03	4.86E-02
cg13467672		17	Intergenic	Island	25.94	4.98	30.38	5.15	-4.44	3.02E-04	6.83E-03
cg00377070	PLXNA4	7	3'UTR	Open sea	80.94	4.27	85.37	3.93	-4.44	1.26E-06	5.56E-05
cg09750210		6	Intergenic	Open sea	51.81	6.43	56.25	5.44	-4.44	2.18E-05	7.55E-04
cg23994112	SEPT9;SEPT9;SEPT9;SEPT9;SEPT9	17	5'UTR;5'UTR;Body;Body;Body	S_Shelf	27.04	4.79	31.47	6.59	-4.44	1.10E-03	1.83E-02
cg02324920	NEURL	10	TSS1500	N_Shore	55.23	3.22	59.66	4.22	-4.44	4.82E-07	2.25E-05
cg14042889	HOXA4	7	Body	N_Shore	28.69	5.37	33.13	6.15	-4.44	9.83E-04	1.69E-02
cg21786191	ZADH2	18	Body	N_Shore	43.38	5.50	47.82	6.43	-4.44	2.84E-03	3.61E-02
cg06799163		5	Intergenic	N_Shelf	42.55	5.22	46.99	5.46	-4.43	2.84E-04	6.52E-03
cg16586518	NBEAL2	3	Body	Open sea	36.95	4.71	41.39	4.63	-4.43	6.04E-05	1.83E-03
cg25209111	GBF1	10	5'UTR	S_Shelf	49.95	5.19	54.39	4.72	-4.43	6.93E-05	2.06E-03
cg18206567	MBP;MBP	18	5'UTR;5'UTR	N_Shelf	70.37	6.40	74.80	6.38	-4.43	4.08E-03	4.62E-02
cg17091456	ELOVL5	6	5'UTR	Open sea	56.79	5.84	61.22	5.83	-4.43	1.99E-03	2.82E-02
cg07592361	ANKMY1;ANKMY1	2	5'UTR;5'UTR	Island	50.84	5.51	55.27	3.87	-4.43	9.23E-05	2.62E-03
cg11945372	MICAL3;MICAL3;MICAL3	22	TSS1500;TSS1500;TSS1500	S_Shore	32.09	6.33	36.52	7.14	-4.43	3.05E-03	3.79E-02
cg01256846	C15orf38	15	TSS1500	S_Shore	65.04	6.01	69.47	5.21	-4.43	1.17E-03	1.93E-02
cg21534309	RSF1	11	Body	Open sea	66.18	6.40	70.62	5.74	-4.43	2.72E-03	3.50E-02
cg16546503	TUBB6	18	TSS1500	N_Shore	8.95	3.22	13.38	6.42	-4.43	5.56E-05	1.70E-03
cg10640333	OTX1	2	Body	N_Shore	46.47	5.46	50.90	6.82	-4.43	7.06E-04	1.32E-02
cg16923137	FAM171A1	10	Body	N_Shelf	73.27	7.11	77.70	5.41	-4.43	1.29E-03	2.07E-02
cg11578064		12	Intergenic	Open sea	23.68	4.50	28.11	5.32	-4.43	3.72E-04	8.06E-03
cg14599961	UBE3A	15	TSS1500	S_Shore	86.47	5.42	90.90	2.36	-4.43	6.56E-05	1.96E-03
cg07555437	SLC13A3;SLC13A3	20	Body;5'UTR	Island	36.01	4.50	40.44	3.62	-4.43	6.36E-06	2.49E-04
cg08129505	PGLYRP2	19	Body	Island	48.69	4.63	53.12	6.68	-4.43	4.13E-04	8.74E-03
cg26187962	EPHX1;EPHX1	1	TSS1500;5'UTR	Open sea	33.05	4.57	37.48	4.71	-4.43	1.45E-04	3.80E-03
cg20049415	NKX2-4	20	1stExon	Island	20.31	4.52	24.74	5.83	-4.43	3.96E-06	1.62E-04
cg06528559	LOC100132215	2	TSS1500	Island	24.86	5.80	29.29	6.13	-4.43	1.31E-03	2.09E-02
cg14517355		3	Intergenic	S_Shelf	33.32	5.05	37.75	6.43	-4.43	3.60E-03	4.25E-02
cg26521766		11	Intergenic	Open sea	65.65	5.50	70.08	4.56	-4.43	8.91E-06	3.39E-04
cg16620390		12	Intergenic	Island	22.22	3.42	26.65	4.84	-4.43	4.94E-06	1.98E-04
cg20025630	BAT5	6	Body	N_Shore	64.48	4.90	68.91	5.74	-4.43	8.08E-04	1.46E-02
cg25025879		12	Intergenic	Island	24.96	6.63	29.39	6.38	-4.43	8.83E-04	1.56E-02
cg11986861	ZNRF3	22	5'UTR	S_Shelf	53.04	4.98	57.47	5.55	-4.43	1.21E-03	1.97E-02
cg25824978	ZNF830;CCT6B;ZNF830	17	1stExon;TSS1500;3'UTR	S_Shore	63.17	6.24	67.60	6.35	-4.43	3.62E-04	7.88E-03
cg14851608	ZBTB40;ZBTB40	1	TSS1500;TSS1500	N_Shore	64.63	3.50	69.06	3.27	-4.43	4.89E-08	2.49E-06

cg18477569	BACH2;BACH2	6	5'UTR;5'UTR	Open sea	53.21	5.47	57.64	5.34	-4.43	8.57E-04	1.53E-02
cg26681123	SLC34A3	9	TSS200	N_Shelf	56.28	4.36	60.70	4.76	-4.43	1.00E-04	2.80E-03
cg14989252	GAPT	5	TSS1500	Open sea	58.11	5.52	62.54	5.20	-4.43	2.07E-04	5.08E-03
cg03532673	NAPEPLD;NAPEPLD	7	5'UTR;5'UTR	N_Shore	29.16	5.46	33.59	5.73	-4.43	1.75E-03	2.57E-02
cg10000952		11	Intergenic	Island	12.51	3.93	16.93	4.55	-4.42	6.53E-07	3.01E-05
cg21453840		8	Intergenic	N_Shelf	56.54	6.25	60.97	5.01	-4.42	1.65E-03	2.47E-02
cg16611584	AKAP10	17	3'UTR	Open sea	57.25	5.88	61.68	4.60	-4.42	2.59E-04	6.07E-03
cg08091771	PTPRG	3	Body	Open sea	61.38	4.22	65.80	4.18	-4.42	4.62E-06	1.86E-04
cg16266763		5	Intergenic	Open sea	74.16	5.84	78.59	5.62	-4.42	2.48E-03	3.29E-02
cg12659933	ST7L;ST7L;ST7L;ST7L	1	Body;Body;Body;Body	Open sea	80.38	6.22	84.80	6.16	-4.42	5.36E-04	1.07E-02
cg13169221	RNF39;RNF39	6	TSS1500;TSS1500	S_Shore	51.81	3.98	56.23	3.78	-4.42	9.08E-07	4.10E-05
cg09433588	FGF17	8	TSS1500	Open sea	36.46	5.71	40.89	4.80	-4.42	1.29E-04	3.44E-03
cg10823473	ISL2	15	Body	Island	26.31	3.21	30.73	3.62	-4.42	1.86E-08	9.77E-07
cg19685521	PCOLCE	7	TSS200	N_Shore	26.06	4.93	30.48	7.47	-4.42	3.90E-03	4.49E-02
cg04303382		17	Intergenic	Island	30.22	4.33	34.64	5.54	-4.42	1.97E-05	6.89E-04
cg19211931	DPP10	2	Body	N_Shore	26.87	5.83	31.29	7.19	-4.42	1.22E-03	1.98E-02
cg11229663	PRKG1;PRKG1	10	Body;Body	Open sea	47.70	4.72	52.12	6.49	-4.42	9.01E-04	1.59E-02
cg04611437	SEC31B	10	TSS200	Island	16.58	5.38	21.00	6.16	-4.42	4.41E-03	4.88E-02
cg01164099	MPST;MPST;MPST;MPST	22	3'UTR;3'UTR;3'UTR;Body	Open sea	76.11	6.29	80.53	5.34	-4.42	3.68E-03	4.31E-02
cg17329886	RP1L1	8	TSS1500	Open sea	63.55	6.30	67.97	5.29	-4.42	3.14E-04	7.05E-03
cg25449315		12	Intergenic	N_Shelf	38.43	4.46	42.85	5.14	-4.42	3.05E-04	6.88E-03
cg14270193	ASGR1	17	Body	S_Shore	61.48	3.85	65.90	3.60	-4.42	8.14E-06	3.12E-04
cg24604988	CHMP4B	20	Body	Open sea	48.18	5.14	52.60	7.46	-4.42	2.51E-03	3.31E-02
cg22436336	LHX4	1	Body	S_Shore	24.74	4.36	29.16	5.47	-4.42	4.33E-04	9.07E-03
cg13547127		6	Intergenic	Open sea	35.08	5.23	39.50	4.18	-4.42	7.83E-06	3.02E-04
cg11971423	HOXB4	17	3'UTR	N_Shore	32.70	3.51	37.11	4.67	-4.42	1.61E-05	5.76E-04
cg03111394	BAT3;BAT3;BAT3;BAT3	6	Body;Body;Body;Body	Open sea	81.69	5.32	86.11	3.92	-4.42	1.89E-04	4.73E-03
cg17573447		2	Intergenic	Island	19.11	4.36	23.52	3.69	-4.42	1.38E-05	5.02E-04
cg03978143		2	Intergenic	Open sea	79.21	5.08	83.62	4.65	-4.42	3.07E-05	1.02E-03
cg16273051	C3orf25	3	Body	Open sea	64.56	6.13	68.97	6.48	-4.42	5.47E-06	2.17E-04
cg15818008	C11orf34	11	Body	Open sea	60.15	6.04	64.57	5.59	-4.42	1.69E-03	2.51E-02
cg13657785	ZNF876P	4	TSS1500	N_Shore	71.71	5.91	76.13	5.68	-4.42	1.13E-03	1.87E-02
cg00879497	VMAC	19	Body	S_Shelf	51.92	3.90	56.34	4.91	-4.42	5.10E-05	1.58E-03
cg20441135	CPEB3	10	Body	Island	27.86	5.49	32.28	5.90	-4.42	2.97E-03	3.72E-02
cg12688234	FAM196B;DOCK2;FAM196B	5	5'UTR;Body;1stExon	Open sea	24.29	4.98	28.70	6.09	-4.42	1.88E-03	2.71E-02
cg18320766	EPS8L1;EPS8L1	19	Body;Body	Island	8.98	3.05	13.39	7.51	-4.42	5.42E-04	1.08E-02
cg18879590	GPR137B	1	Body	Island	33.23	6.18	37.65	4.46	-4.41	1.09E-04	3.01E-03
cg27354635	IQGAP2	5	Body	Open sea	41.32	5.97	45.73	5.44	-4.41	2.62E-03	3.41E-02
cg07158747		1	Intergenic	S_Shelf	28.75	4.74	33.16	4.47	-4.41	2.83E-06	1.18E-04
cg25958098	SLC12A6;SLC12A6;SLC12A6;SLC12A6;S	15	Body;Body;Body;TSS1500;Body;Body	Open sea	66.50	6.63	70.92	7.26	-4.41	3.86E-03	4.45E-02
cg21416237	FKBP10	17	Body	S_Shore	67.76	4.65	72.18	5.33	-4.41	1.12E-04	3.09E-03
cg24536249		2	Intergenic	N_Shore	60.37	3.59	64.78	3.74	-4.41	8.46E-07	3.84E-05
cg04839706	GPT2;GPT2	16	Body;5'UTR	S_Shore	42.29	5.20	46.71	5.86	-4.41	1.51E-03	2.32E-02
cg06268398		3	Intergenic	Open sea	51.17	4.44	55.58	5.98	-4.41	4.46E-04	9.28E-03
cg16659470	RBM12B	8	3'UTR	Open sea	31.40	5.06	35.81	5.56	-4.41	9.83E-04	1.69E-02
cg12333338		6	Intergenic	Open sea	67.71	6.22	72.12	8.24	-4.41	3.72E-03	4.34E-02
cg04812351	ADAMTS3	4	Body	Open sea	36.44	6.73	40.85	5.22	-4.41	4.38E-04	9.14E-03
cg07527324	C14orf159;C14orf159;C14orf159;SNO	14	5'UTR;5'UTR;5'UTR;TSS1500;5'UTR;5'UTR	Open sea	83.62	5.25	88.03	3.67	-4.41	3.27E-05	1.08E-03
cg04477789	FBXL19;NCRNA00095	16	TSS200;TSS1500	S_Shore	54.01	4.59	58.42	5.40	-4.41	6.02E-06	2.37E-04
cg14009759		7	Intergenic	Open sea	70.61	3.44	75.02	3.17	-4.41	3.94E-10	2.23E-08
cg18568534	OXA1L	14	Body	S_Shelf	71.78	4.53	76.19	3.87	-4.41	2.66E-05	8.99E-04
cg03758467	PCDHA2;PCDHA1;PCDHA1;PCDHA6;PC	5	Body;Body;Body;TSS200;Body;TSS200;Boc	N_Shore	28.26	4.99	32.67	5.69	-4.41	6.50E-05	1.95E-03
cg11567001	SMARCD3;SMARCD3;SMARCD3	7	Body;Body;Body	Island	9.60	3.52	14.01	7.10	-4.41	8.77E-04	1.55E-02

cg13501951		7	Intergenic	Island	30.96	5.91	35.37	6.12	-4.41	2.57E-03	3.36E-02
cg25005374	CLIC4	1	Body	Open sea	60.04	4.39	64.45	6.08	-4.41	5.27E-04	1.05E-02
cg24730892	KCNAB2;KCNAB2	1	5'UTR;5'UTR	Open sea	51.53	5.16	55.94	5.34	-4.41	5.06E-04	1.02E-02
cg07345999		11	Intergenic	Island	38.28	4.71	42.69	5.08	-4.41	7.68E-05	2.24E-03
cg06371583	GNG7	19	5'UTR	S_Shore	42.51	4.62	46.92	3.99	-4.41	2.71E-05	9.12E-04
cg07209298	CFHR1	1	Body	Open sea	63.01	6.16	67.42	5.27	-4.41	3.60E-03	4.25E-02
cg12772086		6	Intergenic	S_Shelf	64.38	5.03	68.78	5.10	-4.41	5.52E-04	1.09E-02
cg00423030	TNRC6C;TNRC6C	17	5'UTR;5'UTR	Island	48.74	6.23	53.15	7.30	-4.41	4.14E-03	4.67E-02
cg12528229		2	Intergenic	Open sea	42.61	4.14	47.01	7.62	-4.41	1.45E-03	2.24E-02
cg01216388		18	Intergenic	N_Shelf	73.96	4.82	78.36	3.90	-4.41	2.08E-05	7.24E-04
cg20885179	VILL	3	Body	Island	30.38	4.61	34.79	3.64	-4.41	2.84E-06	1.18E-04
cg26697583		15	Intergenic	Open sea	53.50	5.17	57.91	5.02	-4.41	2.12E-04	5.18E-03
cg01160692	HIC1;HIC1	17	1stExon;5'UTR	Island	61.36	4.68	65.76	4.71	-4.41	4.63E-06	1.87E-04
cg04797323	SOCS2	12	Body	Island	10.29	3.19	14.69	4.31	-4.41	5.13E-07	2.39E-05
cg07895499	TIPARP	3	Body	Open sea	54.22	6.16	58.63	5.38	-4.41	2.92E-04	6.65E-03
cg17199018	ZNF395	8	Body	Open sea	58.56	5.22	62.96	5.65	-4.41	9.69E-04	1.67E-02
cg11584098		5	Intergenic	S_Shelf	46.88	5.57	51.28	6.46	-4.41	2.86E-03	3.62E-02
cg14031054	PBX1	1	Body	Open sea	16.26	5.00	20.67	5.47	-4.40	7.55E-04	1.39E-02
cg05245346		11	Intergenic	Open sea	62.63	5.46	67.04	5.71	-4.40	1.34E-04	3.57E-03
cg10802509	SGIP1	1	Body	Open sea	58.18	5.15	62.58	5.24	-4.40	4.55E-04	9.42E-03
cg03492469	MPG;MPG;MPG	16	TSS200;TSS1500;TSS1500	N_Shore	34.72	6.49	39.12	5.22	-4.40	6.90E-04	1.30E-02
cg00328935	FLJ43860	8	TSS200	Open sea	31.42	5.40	35.82	4.92	-4.40	1.31E-04	3.49E-03
cg03419948	CDC42EP1	22	5'UTR	N_Shore	28.54	3.36	32.94	3.70	-4.40	8.35E-07	3.79E-05
cg10969006	IBSP	4	TSS200	Open sea	49.78	5.49	54.18	5.19	-4.40	1.02E-04	2.84E-03
cg01672894	FBXO31;FBXO31	16	3'UTR;Body	N_Shelf	69.57	5.68	73.97	4.28	-4.40	1.44E-03	2.23E-02
cg25152411		6	Intergenic	N_Shore	84.10	5.70	88.50	3.24	-4.40	6.99E-05	2.07E-03
cg24051786	SCAP	3	5'UTR	N_Shelf	49.40	5.51	53.81	7.00	-4.40	3.51E-03	4.18E-02
cg27215768	PIK3AP1	10	Body	Open sea	73.32	4.27	77.73	4.21	-4.40	6.30E-05	1.90E-03
cg10205045	ANP32A	15	Body	Open sea	23.52	4.77	27.92	5.60	-4.40	6.38E-05	1.92E-03
cg10362113	PAPOLA	14	Body	Open sea	53.58	6.74	57.98	6.70	-4.40	2.19E-03	3.01E-02
cg09940032		8	Intergenic	Island	15.45	4.51	19.85	7.30	-4.40	3.77E-03	4.38E-02
cg13393830	ZNF577;ZNF577;ZNF577	19	TSS1500;TSS1500;TSS1500	S_Shore	35.48	6.61	39.89	6.41	-4.40	1.77E-03	2.59E-02
cg13266496	DDO;DDO	6	Body;Body	N_Shore	52.74	5.01	57.15	5.14	-4.40	1.51E-04	3.93E-03
cg18702971	THOC5;THOC5;THOC5;THOC5	22	Body;Body;Body;Body	Open sea	37.73	5.66	42.13	5.64	-4.40	1.59E-03	2.40E-02
cg11924806		4	Intergenic	S_Shore	45.67	6.21	50.07	6.83	-4.40	1.70E-03	2.52E-02
cg06107104	MYO15B	17	Body	Open sea	74.70	4.85	79.10	3.43	-4.40	2.16E-05	7.48E-04
cg14518178		17	Intergenic	N_Shelf	38.94	3.33	43.34	4.33	-4.40	2.16E-06	9.20E-05
cg12819781	MOGS;MOGS	2	TSS1500;TSS1500	S_Shore	28.39	3.36	32.79	2.55	-4.40	1.21E-08	6.42E-07
cg06346505	AFAP1L2;AFAP1L2	10	TSS200;TSS200	S_Shore	37.02	4.23	41.42	5.16	-4.40	2.14E-05	7.41E-04
cg17583946	LOC100132215	2	TSS200	Island	36.39	4.24	40.79	5.61	-4.40	1.45E-04	3.80E-03
cg14691499	GTDC1;GTDC1;GTDC1	2	Body;Body;Body	Open sea	58.79	3.73	63.19	3.75	-4.40	4.58E-07	2.15E-05
cg10375559		13	Intergenic	Open sea	31.03	4.81	35.43	6.52	-4.40	1.32E-03	2.11E-02
cg16505233	EDEM1	3	Body	Open sea	65.05	5.88	69.44	4.68	-4.40	1.11E-04	3.06E-03
cg04117076		5	Intergenic	Open sea	30.87	5.24	35.27	3.90	-4.40	1.14E-05	4.23E-04
cg00812578	APOM	6	Body	N_Shelf	50.83	4.04	55.23	3.94	-4.40	8.34E-06	3.19E-04
cg22163535	L3MBTL4	18	5'UTR	N_Shelf	51.43	5.11	55.83	5.48	-4.40	1.44E-04	3.78E-03
cg14487854	NXN	17	Body	S_Shore	80.61	5.86	85.01	5.88	-4.40	1.18E-04	3.21E-03
cg15991405	HOXD9	2	Body	N_Shore	29.64	3.99	34.03	4.24	-4.40	3.93E-07	1.86E-05
cg17695791		11	Intergenic	Open sea	67.15	4.16	71.55	5.00	-4.40	6.21E-07	2.87E-05
cg04975920	TSPAN1	1	5'UTR	Open sea	42.65	3.94	47.04	4.65	-4.39	7.78E-06	3.00E-04
cg14377923	MTA1	14	Body	Open sea	34.29	4.82	38.68	5.16	-4.39	4.78E-04	9.78E-03
cg07028396		10	Intergenic	Island	47.39	5.28	51.78	4.61	-4.39	2.53E-04	5.95E-03
cg27099262		12	Intergenic	Open sea	72.67	5.52	77.07	3.70	-4.39	6.76E-05	2.01E-03

cg12604435	NPR1	1	Body	S_Shore	56.33	5.58	60.72	5.83	-4.39	3.71E-03	4.33E-02
cg25416153		18	Intergenic	Island	21.05	4.98	25.44	4.93	-4.39	1.69E-04	4.32E-03
cg16678426	TNXB	6	Body	Open sea	46.50	5.15	50.89	4.51	-4.39	1.04E-05	3.91E-04
cg02446871	EPB41;EPB41;EPB41;EPB41;EPB41;EPI	1	5'UTR;5'UTR;5'UTR;5'UTR;5'UTR;5'UTR	Island	18.95	4.94	23.34	5.97	-4.39	1.76E-05	6.24E-04
cg13676759	IPO11;IPO11	5	Body;Body	Open sea	75.69	8.51	80.09	8.17	-4.39	2.32E-03	3.13E-02
cg10718130	DGAT1	8	3'UTR	Open sea	31.42	4.77	35.82	5.54	-4.39	2.39E-03	3.20E-02
cg09095792	KIAA1033	12	Body	S_Shelf	58.54	6.14	62.93	5.38	-4.39	1.69E-03	2.51E-02
cg01410415	ASPSCR1	17	Body	S_Shore	50.91	4.68	55.30	4.88	-4.39	3.43E-04	7.55E-03
cg14902773		14	Intergenic	Open sea	55.73	5.71	60.12	4.62	-4.39	8.99E-04	1.58E-02
cg06491573	KLF15	3	Body	Island	86.95	5.09	91.34	4.83	-4.39	2.71E-05	9.12E-04
cg01464748	ALOX5	10	Body	Island	69.35	6.04	73.75	6.37	-4.39	3.82E-03	4.42E-02
cg02458554		18	Intergenic	Island	41.23	4.89	45.62	5.20	-4.39	3.15E-04	7.06E-03
cg06734406	AQP2	12	Body	Island	47.24	5.26	51.63	6.17	-4.39	1.37E-03	2.16E-02
cg13661519	HOXA2	7	Body	N_Shelf	30.62	3.60	35.01	4.47	-4.39	1.83E-05	6.44E-04
cg27118929	MIR548H4;NOX5	15	Body;Body	N_Shore	68.35	6.60	72.74	6.26	-4.39	4.24E-03	4.75E-02
cg14705155		6	Intergenic	Open sea	83.40	4.82	87.79	3.37	-4.39	4.53E-06	1.83E-04
cg27019158		14	Intergenic	Open sea	70.82	5.45	75.20	3.84	-4.39	2.38E-05	8.14E-04
cg00146864	MAP3K14	17	5'UTR	N_Shore	33.47	3.35	37.86	4.98	-4.39	2.39E-07	1.15E-05
cg07912161	PI16	6	Body	Island	75.06	6.76	79.45	5.22	-4.39	2.26E-03	3.08E-02
cg03840289	MXD4	4	Body	N_Shore	47.93	4.01	52.32	3.09	-4.39	6.65E-06	2.59E-04
cg17849569	ZSCAN12L1	6	TSS200	Open sea	35.41	5.25	39.80	6.20	-4.39	1.25E-03	2.02E-02
cg01986648		6	Intergenic	Open sea	44.41	5.42	48.80	5.26	-4.39	1.45E-03	2.25E-02
cg02106279		6	Intergenic	Open sea	71.67	5.48	76.06	4.77	-4.39	8.93E-05	2.55E-03
cg08884395	ESR1;ESR1;ESR1;ESR1	6	TSS1500;5'UTR;TSS1500;5'UTR	N_Shore	49.84	4.72	54.23	5.87	-4.39	7.56E-04	1.39E-02
cg15798385	EVX1	7	TSS1500	N_Shore	27.23	4.58	31.62	4.39	-4.39	4.34E-06	1.76E-04
cg23061121		13	Intergenic	Open sea	81.00	4.54	85.39	3.91	-4.39	1.53E-06	6.67E-05
cg12832204		7	Intergenic	Open sea	41.98	6.44	46.37	6.61	-4.39	3.64E-03	4.28E-02
cg27281030	NLRP12;NLRP12	19	Body;TSS1500	Open sea	78.01	6.28	82.40	5.99	-4.39	2.75E-03	3.52E-02
cg18673409	AANAT;UBE2O	17	TSS1500;Body	N_Shore	42.34	4.00	46.73	5.22	-4.39	1.67E-06	7.23E-05
cg24402990	MIR548B;FAM184A;FAM184A	6	Body;5'UTR;Body	Open sea	50.28	4.64	54.67	4.66	-4.39	5.92E-05	1.80E-03
cg04815577		5	Intergenic	Island	63.99	7.16	68.38	6.76	-4.39	2.95E-03	3.70E-02
cg14881470	CLYBL	13	Body	S_Shore	19.46	6.13	23.85	6.12	-4.39	1.65E-03	2.46E-02
cg20655792	CD97;CD97;CD97	19	Body;Body;Body	Island	28.58	5.34	32.97	6.58	-4.39	4.77E-04	9.77E-03
cg02489438		19	Intergenic	Island	22.89	3.92	27.28	4.52	-4.39	1.33E-06	5.86E-05
cg05458220	ADNP2	18	TSS1500	Open sea	23.95	4.89	28.33	5.23	-4.39	1.74E-03	2.56E-02
cg18263455	MIR548H4;TMEM84	15	Body;Body	Open sea	66.69	6.17	71.08	4.47	-4.38	6.51E-04	1.24E-02
cg10836540		10	Intergenic	S_Shelf	62.23	5.64	66.62	6.01	-4.38	1.12E-04	3.08E-03
cg19808205	TCTEX1D1	1	TSS200	N_Shore	37.24	4.91	41.62	4.44	-4.38	1.54E-05	5.55E-04
cg19674178	FGF7;C15orf33	15	Body;Body	Open sea	70.65	4.79	75.04	4.51	-4.38	4.42E-05	1.40E-03
cg25407085		6	Intergenic	S_Shore	62.34	5.40	66.72	4.26	-4.38	3.42E-04	7.53E-03
cg01152052	KIAA1984	9	Body	N_Shelf	40.46	4.52	44.84	4.34	-4.38	5.42E-05	1.66E-03
cg10542223	ZNF815	7	TSS200	N_Shore	48.80	5.27	53.18	5.57	-4.38	4.12E-04	8.72E-03
cg02219197	PROC	2	TSS1500	S_Shore	24.21	4.51	28.59	6.03	-4.38	1.45E-03	2.25E-02
cg14240223	NOTCH1	9	Body	S_Shore	68.46	4.47	72.84	2.96	-4.38	1.33E-06	5.85E-05
cg23260484	EFCAB2;EFCAB2;EFCAB2;EFCAB2;EFC/	1	TSS1500;TSS1500;TSS1500;TSS1500;TSS15	N_Shore	27.01	6.34	31.39	6.67	-4.38	4.19E-03	4.71E-02
cg00528056		5	Intergenic	Open sea	67.53	5.02	71.91	3.92	-4.38	4.31E-05	1.37E-03
cg07547054		2	Intergenic	Open sea	61.55	5.88	65.93	7.00	-4.38	2.85E-03	3.61E-02
cg10395772	FGF14	13	Body	S_Shore	15.85	3.07	20.23	4.86	-4.38	4.55E-06	1.84E-04
cg09012091	GPLD1	6	Body	Open sea	74.78	5.26	79.16	4.19	-4.38	4.09E-06	1.66E-04
cg21610090	CDKL4	2	Body	Open sea	51.50	4.97	55.88	4.86	-4.38	2.66E-04	6.21E-03
cg23300486	TRAF3;TRAF3;TRAF3	14	5'UTR;5'UTR;5'UTR	Open sea	74.21	5.69	78.59	3.99	-4.38	5.54E-05	1.70E-03
cg19164761	CNN3	1	Body	Open sea	46.83	4.52	51.21	4.66	-4.38	6.09E-05	1.84E-03
cg17965019	HIST1H3J	6	1stExon	Island	25.41	5.08	29.79	6.46	-4.38	1.90E-05	6.68E-04

cg19314470	RBPMS;RBPMS;RBPMS;RBPMS	8	Body;Body;Body;Body	Open sea	46.62	6.10	51.00	7.47	-4.38	4.13E-03	4.66E-02
cg06649282	C16orf57	16	3'UTR	N_Shelf	46.75	5.08	51.13	5.37	-4.38	2.73E-03	3.50E-02
cg01434649	TP73;WDR8	1	TSS1500;TSS1500	Island	34.83	3.50	39.21	4.36	-4.38	2.27E-07	1.10E-05
cg04581018	CCDC36;CCDC36	3	TSS200;5'UTR	Island	24.95	4.19	29.33	6.15	-4.38	1.82E-04	4.59E-03
cg07723251	CA10;CA10	17	TSS1500;TSS1500	S_Shore	37.16	6.23	41.54	5.91	-4.38	2.36E-03	3.18E-02
cg17457701	MIR1276;KLHL25	15	TSS200;5'UTR	S_Shore	65.63	6.12	70.01	6.26	-4.38	4.91E-04	9.98E-03
cg15827779	CDC42BPB	14	TSS1500	S_Shore	26.89	5.71	31.27	7.07	-4.38	3.97E-03	4.54E-02
cg07236114	DLG2;DLG2	11	Body;Body	Open sea	28.95	4.50	33.33	5.97	-4.38	3.20E-04	7.16E-03
cg05954147	GRM8;GRM8;GRM8	7	Body;Body;Body	Open sea	81.80	5.60	86.18	5.43	-4.38	7.74E-05	2.26E-03
cg04946910		16	Intergenic	Island	63.50	5.99	67.88	6.08	-4.38	4.04E-03	4.59E-02
cg23430771	SLC45A2;SLC45A2	5	TSS200;TSS200	Open sea	78.12	6.15	82.50	5.14	-4.38	2.43E-03	3.24E-02
cg18994015		2	Intergenic	N_Shelf	56.63	5.69	61.00	6.19	-4.38	1.04E-03	1.76E-02
cg21645554	C11orf93;C11orf92	11	TSS1500;5'UTR	Island	43.81	6.20	48.19	4.35	-4.38	2.17E-04	5.27E-03
cg05492904	CYP19A1;CYP19A1	15	5'UTR;5'UTR	Open sea	51.94	4.68	56.31	6.33	-4.38	3.93E-04	8.39E-03
cg21494776	ICAM4;ICAM4;ICAM4	19	1stExon;1stExon;1stExon	Island	24.24	5.23	28.62	4.75	-4.38	1.72E-04	4.38E-03
cg22970003	PTPRN2;PTPRN2;PTPRN2	7	Body;Body;Body	Island	19.38	4.56	23.76	6.33	-4.38	1.12E-03	1.86E-02
cg05894734	APBB2;APBB2;APBB2;APBB2;APBB2;A	4	Body;TSS200;TSS200;Body;TSS200;Body;T	S_Shore	29.30	6.51	33.68	5.99	-4.38	7.74E-05	2.26E-03
cg06637550	C1QTNF6;C1QTNF6	22	TSS1500;TSS1500	Open sea	74.48	6.11	78.85	2.85	-4.38	1.14E-04	3.11E-03
cg13466409	AATK;MIR1250	17	Body;Body	N_Shelf	75.12	5.99	79.49	4.41	-4.38	1.67E-05	5.96E-04
cg11147155	ENSA;ENSA;ENSA;ENSA	1	3'UTR;3'UTR;3'UTR;3'UTR	Open sea	48.57	6.41	52.95	5.17	-4.38	2.82E-03	3.59E-02
cg08056229	ZBTB40;ZBTB40	1	5'UTR;5'UTR	Open sea	66.40	5.16	70.77	5.13	-4.38	1.22E-03	1.98E-02
cg26345888	DAB1	1	TSS200	S_Shore	19.98	4.90	24.35	4.60	-4.38	2.81E-04	6.47E-03
cg15501219		3	Intergenic	N_Shelf	80.36	2.64	84.74	2.67	-4.38	1.42E-12	8.64E-11
cg16163419	ABI3BP;ABI3BP	3	5'UTR;1stExon	Open sea	19.64	3.41	24.02	4.09	-4.38	2.84E-06	1.19E-04
cg08448479	ANK2;ANK2;ANK2	4	Body;Body;Body	Open sea	68.72	5.58	73.09	4.04	-4.38	1.55E-04	4.01E-03
cg01716296	DCLK2;DCLK2	4	Body;Body	Open sea	59.09	4.94	63.46	6.55	-4.37	4.42E-04	9.21E-03
cg24974737		17	Intergenic	Island	28.78	3.90	33.15	6.68	-4.37	4.68E-04	9.63E-03
cg12807924	SRC;SRC	20	Body;Body	S_Shore	37.83	4.07	42.20	6.62	-4.37	1.10E-03	1.84E-02
cg03655049		12	Intergenic	Open sea	80.56	5.81	84.94	4.88	-4.37	3.41E-07	1.62E-05
cg10322504		20	Intergenic	Island	22.40	4.53	26.77	6.32	-4.37	9.77E-05	2.75E-03
cg06870266		13	Intergenic	Open sea	69.71	5.20	74.08	4.49	-4.37	4.21E-04	8.87E-03
cg12861771	C15orf27	15	Body	Open sea	62.94	5.37	67.32	4.97	-4.37	2.19E-03	3.01E-02
cg20720680	INPPL1	11	Body	N_Shelf	72.20	4.91	76.57	4.52	-4.37	4.80E-05	1.50E-03
cg26430059	FOXN3;FOXN3	14	Body;Body	Open sea	46.10	6.04	50.47	5.08	-4.37	1.14E-04	3.11E-03
cg09232069	SEC14L1;SEC14L1;SEC14L1;SEC14L1	17	Body;Body;Body;Body	Open sea	20.32	4.63	24.69	6.80	-4.37	4.11E-03	4.65E-02
cg04275847	OAT	10	5'UTR	N_Shore	41.01	5.77	45.38	5.86	-4.37	2.35E-03	3.17E-02
cg12374682	GPHA2	11	TSS200	Open sea	52.20	5.23	56.57	4.56	-4.37	6.19E-04	1.19E-02
cg08733739	GFOD1	6	Body	Open sea	73.96	5.35	78.33	4.91	-4.37	8.56E-04	1.52E-02
cg01966732		7	Intergenic	Open sea	75.58	5.43	79.95	4.10	-4.37	5.79E-04	1.13E-02
cg08577693	NUTF2	16	Body	N_Shelf	56.21	3.41	60.58	2.85	-4.37	5.63E-08	2.86E-06
cg27045264		16	Intergenic	S_Shelf	49.90	5.24	54.27	5.71	-4.37	2.58E-04	6.06E-03
cg16635108	B3GAT1;B3GAT1	11	5'UTR;5'UTR	N_Shore	12.95	3.49	17.32	4.57	-4.37	5.94E-06	2.34E-04
cg19015511	ITGAE	17	Body	Open sea	69.16	4.92	73.53	3.88	-4.37	7.05E-06	2.74E-04
cg03037620		10	Intergenic	Open sea	51.38	3.15	55.75	3.75	-4.37	1.45E-07	7.14E-06
cg14456683	ZIC1	3	TSS200	Island	27.21	4.44	31.57	5.01	-4.37	1.71E-04	4.37E-03
cg21984645	FAM20C	7	Body	N_Shelf	39.95	5.09	44.32	6.13	-4.37	1.55E-03	2.35E-02
cg18449964	ZADH2	18	Body	Island	17.37	4.99	21.74	5.20	-4.37	4.78E-04	9.77E-03
cg18145810	REG3G;REG3G	2	TSS200;TSS200	Open sea	65.00	5.60	69.37	3.51	-4.37	4.66E-05	1.46E-03
cg03493123	B4GALT7	5	Body	N_Shore	58.01	3.70	62.38	4.13	-4.37	6.84E-06	2.66E-04
cg13981184		13	Intergenic	Open sea	57.67	6.68	62.03	5.31	-4.37	1.42E-03	2.22E-02
cg01900413	ETS1	11	Body	Island	47.25	5.60	51.61	6.42	-4.36	4.30E-04	9.03E-03
cg01246855	NIPSNAP1	22	Body	S_Shelf	76.63	7.36	81.00	5.44	-4.36	4.55E-03	4.98E-02
cg03277153		10	Intergenic	Open sea	44.50	4.40	48.87	5.45	-4.36	1.74E-04	4.42E-03

cg20927567	RPL23A;SNORD42A;SNORD4B;SNORD	17	Body;TSS1500;TSS1500;Body	N_Shelf	47.54	3.80	51.90	4.07	-4.36	1.39E-06	6.13E-05
cg25001190	NFIA;NFIA;NFIA;NFIA	1	Body;Body;Body;Body	Open sea	39.40	4.68	43.77	5.37	-4.36	6.93E-04	1.30E-02
cg05127548	ADC	1	Body	Open sea	61.78	4.83	66.15	5.22	-4.36	2.25E-04	5.43E-03
cg22136749		12	Intergenic	Open sea	48.85	7.18	53.22	5.94	-4.36	7.08E-04	1.32E-02
cg20282550	AKR1E2	10	TSS200	Island	40.02	6.17	44.38	5.72	-4.36	2.03E-03	2.86E-02
cg15150652	XRCC4;XRCC4;XRCC4	5	Body;Body;Body	Open sea	82.57	4.59	86.93	3.64	-4.36	5.48E-07	2.55E-05
cg17204129	CFTR	7	TSS1500	Open sea	25.11	6.70	29.47	8.44	-4.36	2.48E-04	5.87E-03
cg03222868	KCNH3	12	Body	Island	63.34	5.24	67.70	5.19	-4.36	3.69E-04	8.00E-03
cg06690085	KALRN;KALRN	3	Body;Body	Open sea	31.42	8.54	35.78	9.79	-4.36	5.05E-04	1.02E-02
cg17919186	CCDC88C	14	Body	Island	35.52	8.23	39.88	7.37	-4.36	9.42E-04	1.64E-02
cg07987148	TP53RK	20	TSS1500	S_Shore	22.26	4.53	26.63	5.23	-4.36	9.71E-05	2.73E-03
cg16243243		12	Intergenic	N_Shore	22.69	4.87	27.05	6.28	-4.36	1.19E-03	1.95E-02
cg09642740		8	Intergenic	Island	57.53	4.59	61.90	6.67	-4.36	1.61E-03	2.42E-02
cg11418508		19	Intergenic	Island	75.18	7.60	79.54	7.11	-4.36	1.20E-03	1.96E-02
cg06600725		12	Intergenic	Open sea	33.06	5.35	37.42	7.10	-4.36	3.62E-03	4.26E-02
cg11654562		6	Intergenic	Open sea	62.22	6.49	66.58	5.08	-4.36	1.26E-03	2.03E-02
cg14975237	SEPHS1	10	3'UTR	Open sea	77.34	3.98	81.70	4.37	-4.36	5.23E-06	2.09E-04
cg00712965		3	Intergenic	N_Shelf	69.90	4.75	74.26	5.68	-4.36	3.42E-04	7.54E-03
cg13937817	APBA1	9	5'UTR	S_Shelf	39.89	5.23	44.25	6.78	-4.36	3.11E-03	3.84E-02
cg21609024	LRP8;LRP8;LRP8;LRP8	1	TSS1500;TSS1500;TSS1500;TSS1500	S_Shore	47.72	4.03	52.08	5.30	-4.36	1.96E-04	4.86E-03
cg18287222	MAP3K5	6	Body	Open sea	48.04	4.93	52.40	7.32	-4.36	4.18E-03	4.70E-02
cg14016166	POR	7	TSS1500	N_Shore	77.88	5.17	82.24	5.15	-4.36	2.54E-05	8.64E-04
cg25199254		1	Intergenic	Open sea	38.79	3.91	43.15	3.97	-4.36	3.37E-06	1.39E-04
cg02362309	NCRNA00171	6	Body	Open sea	61.41	4.11	65.77	4.63	-4.36	3.34E-05	1.09E-03
cg24901474	RGS5	1	TSS200	Open sea	52.04	5.65	56.40	4.82	-4.36	2.08E-04	5.10E-03
cg14477619	NPC1L1;NPC1L1;NPC1L1;NPC1L1	7	1stExon;5'UTR;1stExon;5'UTR	Open sea	43.15	4.58	47.51	6.33	-4.36	1.07E-03	1.79E-02
cg20850981	ZNF323;ZKSCAN3;ZNF323	6	5'UTR;5'UTR;TSS200	Open sea	75.50	3.94	79.86	3.38	-4.36	4.62E-06	1.86E-04
cg21801428		3	Intergenic	Open sea	50.76	4.84	55.11	6.76	-4.36	8.21E-04	1.48E-02
cg06810231		14	Intergenic	Open sea	63.34	6.34	67.70	4.75	-4.36	8.59E-04	1.53E-02
cg04659396		5	Intergenic	Open sea	76.38	5.50	80.74	5.53	-4.36	6.76E-04	1.28E-02
cg18213979	RNF135;RNF135	17	TSS200;TSS200	N_Shore	25.14	4.53	29.49	5.72	-4.36	7.87E-06	3.03E-04
cg11445542	SNX33	15	Body	S_Shelf	21.93	4.11	26.29	3.92	-4.36	1.19E-05	4.39E-04
cg25967418	PCDHA7;PCDHA12;PCDHA6;PCDHA10	5	Body;Body;Body;Body;Body;Body;Bo	N_Shore	28.76	4.29	33.12	4.38	-4.36	2.79E-05	9.35E-04
cg06437417	DLAT	11	TSS1500	N_Shore	49.25	6.69	53.61	6.93	-4.36	1.21E-04	3.27E-03
cg06911238	FAAH	1	TSS1500	N_Shore	61.32	6.76	65.67	7.81	-4.36	1.91E-04	4.76E-03
cg11088672		4	Intergenic	Open sea	57.42	6.36	61.77	5.92	-4.35	1.01E-03	1.73E-02
cg02513485	SPTBN1	2	TSS1500	N_Shore	10.44	5.01	14.79	5.36	-4.35	6.26E-05	1.89E-03
cg10949007	GLRX;GLRX	5	TSS1500;TSS1500	Open sea	45.31	6.31	49.67	6.12	-4.35	3.08E-04	6.93E-03
cg19954602	FILIP1	6	TSS200	Open sea	37.01	5.53	41.36	4.94	-4.35	8.77E-04	1.55E-02
cg03355286	SLCO1B3	12	TSS1500	Open sea	53.87	5.47	58.23	3.87	-4.35	1.45E-04	3.81E-03
cg26020826	LOC100133612	1	Body	N_Shelf	61.75	5.04	66.11	5.74	-4.35	3.12E-05	1.03E-03
cg03854077		4	Intergenic	Island	67.71	5.52	72.07	5.44	-4.35	7.61E-04	1.40E-02
cg26063721	PACSIN1	6	Body	Island	80.97	4.85	85.32	3.67	-4.35	7.61E-06	2.94E-04
cg24685239	ALCAM	3	Body	Open sea	39.26	5.18	43.61	6.21	-4.35	1.61E-03	2.42E-02
cg18073906	SEMA5B	3	Body	Island	29.70	4.66	34.05	5.61	-4.35	4.73E-04	9.71E-03
cg03026080		10	Intergenic	S_Shore	44.02	5.93	48.38	6.12	-4.35	1.11E-03	1.85E-02
cg15617640	SH2D1B	1	Body	Open sea	21.37	3.67	25.72	4.16	-4.35	6.57E-07	3.02E-05
cg06045968	CD80	3	Body	Open sea	57.19	4.79	61.54	3.98	-4.35	1.43E-05	5.20E-04
cg00997280		2	Intergenic	Island	33.51	4.05	37.86	5.59	-4.35	7.57E-05	2.21E-03
cg09640686	ANKRD11	16	5'UTR	N_Shelf	54.52	4.86	58.88	6.07	-4.35	1.89E-03	2.71E-02
cg12496975	RAPGEF3;RAPGEF3;RAPGEF3	12	Body;Body;Body	N_Shelf	60.23	4.92	64.58	4.39	-4.35	1.24E-05	4.57E-04
cg00295744	TNRC4	1	TSS1500	Open sea	26.26	5.08	30.61	4.50	-4.35	1.36E-05	4.96E-04
cg25813936	GNE	9	Body	Open sea	32.87	5.35	37.22	6.26	-4.35	2.61E-03	3.40E-02

cg09784335		4	Intergenic	N_Shore	42.64	6.55	46.99	7.76	-4.35	2.02E-04	4.97E-03
cg12239557	TACC2;TACC2	10	5'UTR;5'UTR	Open sea	46.86	8.50	51.21	6.98	-4.35	2.05E-03	2.88E-02
cg18194887	BET1	7	Body	N_Shelf	67.83	7.93	72.18	6.90	-4.35	4.29E-03	4.78E-02
cg17302896		5	Intergenic	N_Shelf	30.93	5.46	35.28	5.59	-4.35	5.05E-04	1.02E-02
cg18339718	MAN2B1	19	Body	Island	27.11	5.46	31.46	5.98	-4.35	1.85E-03	2.67E-02
cg03337057	TSKS	19	Body	Island	56.61	5.66	60.96	6.44	-4.35	3.85E-03	4.44E-02
cg11187807		15	Intergenic	Open sea	55.12	5.73	59.47	5.44	-4.35	5.66E-04	1.11E-02
cg02821998	LETM1	4	Body	N_Shore	75.31	5.01	79.65	3.86	-4.35	2.03E-04	5.01E-03
cg05629774	EP400	12	Body	Open sea	84.34	5.23	88.69	3.42	-4.35	1.09E-05	4.07E-04
cg06827015		3	Intergenic	S_Shore	45.52	6.11	49.87	6.47	-4.35	3.45E-03	4.13E-02
cg07697597	SCUBE1	22	TSS1500	Island	32.39	5.49	36.73	4.84	-4.35	1.63E-04	4.18E-03
cg12377111	CHD3;CHD3;CHD3;SCARNA21	17	Body;Body;Body;TSS1500	Open sea	80.24	4.43	84.59	3.36	-4.35	2.44E-06	1.03E-04
cg11433980	CBR3	21	Body	S_Shelf	67.20	6.28	71.55	4.54	-4.35	6.52E-04	1.24E-02
cg13036381	LOC401097	3	TSS1500	N_Shore	20.38	4.49	24.73	7.33	-4.35	2.29E-03	3.11E-02
cg03463153		7	Intergenic	N_Shelf	64.59	6.80	68.94	4.52	-4.35	1.91E-03	2.74E-02
cg08211301		16	Intergenic	S_Shelf	61.23	4.71	65.58	5.34	-4.35	4.29E-04	9.00E-03
cg06070625	MITF;MITF	3	Body;TSS200	Open sea	79.04	4.99	83.39	5.21	-4.35	2.26E-04	5.45E-03
cg00811930	AK7	14	Body	S_Shelf	67.37	5.65	71.72	5.76	-4.35	5.78E-04	1.13E-02
cg23093692	SLC35F3	1	Body	Island	69.51	6.34	73.86	5.45	-4.35	4.31E-04	9.03E-03
cg13113737		1	Intergenic	Open sea	52.36	4.47	56.71	6.15	-4.35	1.04E-03	1.76E-02
cg08987995	ITPR1;ITPR1;ITPR1	3	Body;Body;Body	Open sea	47.21	5.12	51.55	6.67	-4.35	2.94E-03	3.69E-02
cg15431934	BAHD1	15	3'UTR	N_Shelf	53.10	5.52	57.44	5.89	-4.35	6.99E-05	2.07E-03
cg08533336	USP2;USP2	11	Body;Body	Island	49.99	4.93	54.33	5.37	-4.35	7.45E-04	1.37E-02
cg25985013	EPB49;EPB49;EPB49;EPB49;EPB49;EPI	8	Body;Body;Body;Body;Body;Body	S_Shore	51.70	5.03	56.05	5.89	-4.35	1.69E-04	4.32E-03
cg11721591		10	Intergenic	Open sea	41.80	5.38	46.15	6.75	-4.35	3.43E-03	4.11E-02
cg12776061	RXRB	6	Body	S_Shelf	53.60	5.96	57.95	6.65	-4.34	4.08E-03	4.62E-02
cg24071057	EZH1	17	Body	Open sea	23.03	4.12	27.37	4.84	-4.34	6.10E-05	1.84E-03
cg07541160	FGD2	6	Body	Open sea	59.52	4.52	63.87	3.93	-4.34	6.13E-06	2.41E-04
cg04380118	ZSWIM5	1	Body	N_Shore	56.31	5.58	60.66	5.88	-4.34	1.72E-04	4.39E-03
cg24379995	CATSPER2;CATSPER2;CATSPER2	15	Body;Body;Body	N_Shelf	57.12	5.35	61.47	4.33	-4.34	2.77E-04	6.39E-03
cg18815340		6	Intergenic	Open sea	61.86	5.88	66.20	6.14	-4.34	8.71E-07	3.94E-05
cg07969095	ACVR2A	2	Body	S_Shelf	74.57	6.79	78.92	6.73	-4.34	1.46E-03	2.26E-02
cg00043444		6	Intergenic	Open sea	77.57	6.04	81.91	5.01	-4.34	3.59E-03	4.24E-02
cg02578559		3	Intergenic	N_Shore	62.13	5.16	66.47	4.94	-4.34	8.82E-05	2.52E-03
cg13011331	EEPD1	7	Body	Open sea	69.97	3.99	74.31	3.49	-4.34	8.81E-07	3.98E-05
cg13774825	AKT1;AKT1	14	TSS1500;TSS1500	S_Shore	54.15	5.18	58.49	5.25	-4.34	5.02E-04	1.02E-02
cg17106157	MIR1-2;MIB1	18	TSS1500;Body	Open sea	76.85	5.29	81.19	4.75	-4.34	3.69E-04	8.00E-03
cg05772125	AHSP	16	TSS200	Open sea	24.88	5.46	29.22	6.02	-4.34	2.70E-03	3.48E-02
cg07944051		15	Intergenic	Open sea	68.07	6.35	72.41	4.59	-4.34	9.83E-04	1.69E-02
cg25649188	CASKIN2;CASKIN2	17	Body;Body	N_Shore	62.10	6.81	66.44	6.76	-4.34	3.76E-03	4.38E-02
cg03647861	THADA;THADA	2	Body;Body	Open sea	76.39	4.95	80.73	3.84	-4.34	3.12E-05	1.03E-03
cg20427331	INTS6;INTS6	13	Body;Body	Open sea	78.30	5.28	82.64	4.82	-4.34	4.74E-04	9.72E-03
cg18950952	MALL	2	Body	N_Shore	51.09	5.30	55.43	6.35	-4.34	4.53E-03	4.97E-02
cg16691158		5	Intergenic	S_Shore	34.37	3.90	38.71	4.37	-4.34	2.75E-07	1.32E-05
cg26244738	APBB2;APBB2;APBB2;APBB2;APBB2;A	4	Body;TSS200;TSS200;Body;TSS200;Body;T	S_Shore	19.31	4.09	23.65	5.28	-4.34	4.37E-06	1.77E-04
cg03746015	CLEC16A	16	Body	Open sea	75.35	6.42	79.69	6.22	-4.34	3.36E-04	7.44E-03
cg12007362	C2orf24	2	Body	N_Shelf	78.87	4.03	83.21	3.40	-4.34	4.41E-07	2.08E-05
cg22797884		19	Intergenic	N_Shelf	14.64	3.85	18.98	6.21	-4.34	3.04E-04	6.88E-03
cg06901307		13	Intergenic	Open sea	60.18	6.27	64.52	6.94	-4.34	1.36E-03	2.15E-02
cg21773820		8	Intergenic	Open sea	59.93	6.13	64.27	7.39	-4.34	3.29E-03	3.99E-02
cg15197065	CMYA5	5	TSS200	Open sea	27.76	5.50	32.09	5.96	-4.34	3.72E-03	4.34E-02
cg10413089	CYFIP1	15	5'UTR	Open sea	73.94	5.55	78.27	4.63	-4.34	2.12E-04	5.17E-03
cg03436956		8	Intergenic	Open sea	82.63	3.03	86.97	2.67	-4.34	6.57E-10	3.69E-08

cg26551092	ARID1B;ARID1B;ARID1B	6	Body;Body;Body	Open sea	19.79	4.89	24.13	5.01	-4.34	8.10E-04	1.46E-02
cg10805676	TMEM8A;MRPL28	16	Body;TSS1500	N_Shore	73.04	5.16	77.38	3.84	-4.34	1.61E-04	4.15E-03
cg02005600	HOXA5	7	TSS1500	Island	27.23	4.34	31.57	4.40	-4.34	5.13E-05	1.59E-03
cg00610021	SMC1B;RIBC2	22	Body;TSS1500	Island	27.48	5.51	31.82	5.23	-4.34	9.42E-04	1.64E-02
cg10227863	PDGFRA	4	5'UTR	Open sea	65.17	5.28	69.51	6.22	-4.34	6.20E-04	1.20E-02
cg15329642	PTCH2;PTCH2	1	Body;Body	N_Shore	56.70	5.33	61.04	5.26	-4.34	1.65E-03	2.47E-02
cg27171704	TBKBP1	17	Body	N_Shore	64.41	5.57	68.75	5.70	-4.34	1.00E-03	1.71E-02
cg12991609		8	Intergenic	N_Shelf	72.13	6.15	76.47	5.65	-4.34	2.88E-03	3.64E-02
cg20432960	TFAP2B	6	Body	Open sea	36.55	4.90	40.89	6.27	-4.34	9.18E-06	3.48E-04
cg04682969	WNT5B;WNT5B	12	Body;Body	N_Shelf	49.70	5.14	54.03	5.84	-4.34	3.31E-03	4.01E-02
cg15925365	GSTA4	6	Body	N_Shore	60.37	7.35	64.71	6.52	-4.33	8.51E-04	1.52E-02
cg04753078	TNXB	6	Body	Island	46.87	4.67	51.20	4.37	-4.33	4.57E-06	1.84E-04
cg15986668	NFYC;NFYC;NFYC;LOC100130557;NFY(	1	TSS1500;TSS1500;TSS1500;Body;TSS1500;	N_Shore	49.45	6.05	53.79	5.98	-4.33	1.31E-03	2.09E-02
cg01284448	FLVCR2	14	3'UTR	Open sea	59.68	5.04	64.01	6.10	-4.33	1.04E-03	1.76E-02
cg20401130		2	Intergenic	N_Shelf	73.94	5.15	78.27	4.34	-4.33	6.82E-05	2.03E-03
cg26205751	FSCN3	7	TSS200	Open sea	43.70	4.54	48.03	5.17	-4.33	8.92E-04	1.57E-02
cg12536527	CACNA2D1	7	Body	Open sea	69.57	5.79	73.90	5.33	-4.33	6.97E-04	1.31E-02
cg14419393	ASPSCR1	17	Body	Island	78.96	5.43	83.29	5.08	-4.33	3.42E-03	4.10E-02
cg01845111	EFCAB8	20	5'UTR	Open sea	58.88	5.23	63.21	4.92	-4.33	2.26E-04	5.45E-03
cg08810072		17	Intergenic	Open sea	45.89	5.51	50.22	5.27	-4.33	1.62E-03	2.43E-02
cg08897150	CCDC81;CCDC81	11	TSS1500;TSS1500	N_Shore	42.22	6.87	46.55	6.90	-4.33	1.02E-03	1.74E-02
cg20445402	IRS2	13	Body	N_Shelf	84.65	5.23	88.98	2.82	-4.33	6.89E-05	2.05E-03
cg12252090	SNAI2	8	Body	N_Shelf	15.27	2.95	19.60	5.06	-4.33	1.10E-06	4.91E-05
cg10584024		1	Intergenic	Open sea	36.74	5.51	41.07	5.55	-4.33	1.84E-03	2.67E-02
cg23986495		15	Intergenic	Open sea	73.53	6.36	77.86	3.85	-4.33	2.19E-03	3.01E-02
cg19911116	STEAP3;STEAP3;STEAP3	2	Body;Body;Body	N_Shelf	72.86	4.83	77.19	5.45	-4.33	9.69E-06	3.66E-04
cg02902313	NOTCH4	6	Body	S_Shore	55.35	4.93	59.68	4.76	-4.33	8.41E-04	1.50E-02
cg21333861	B3GALT4;B3GALT4	6	1stExon;5'UTR	Island	49.47	7.43	53.79	3.51	-4.33	1.56E-03	2.36E-02
cg16165651	RASGRP3;RASGRP3;RASGRP3	2	Body;Body;Body	Open sea	70.32	6.54	74.65	5.43	-4.33	1.59E-03	2.40E-02
cg05338187	LGALS8;LGALS8;LGALS8;LGALS8	1	TSS200;TSS200;5'UTR;TSS1500	N_Shore	17.22	3.83	21.55	5.92	-4.33	1.08E-03	1.81E-02
cg09249877	DOCK4	7	Body	Open sea	82.31	4.90	86.63	4.30	-4.33	2.33E-04	5.58E-03
cg06454157		6	Intergenic	Open sea	70.72	4.74	75.05	3.63	-4.33	5.55E-06	2.20E-04
cg14152268	MGC23270	14	TSS200	Open sea	53.82	5.39	58.15	5.73	-4.33	8.15E-04	1.47E-02
cg05739419	CSAD	12	Body	N_Shelf	45.39	4.83	49.72	4.16	-4.33	1.46E-04	3.83E-03
cg17449759		1	Intergenic	Open sea	75.97	5.13	80.29	5.17	-4.33	7.36E-04	1.36E-02
cg20720918	GORASP2	2	TSS1500	N_Shore	31.36	5.75	35.69	7.64	-4.33	1.93E-03	2.76E-02
cg00370022	CYP1A1	15	Body	N_Shelf	73.79	7.32	78.11	6.32	-4.33	1.63E-03	2.45E-02
cg19513876		17	Intergenic	N_Shelf	73.72	6.23	78.05	5.76	-4.32	4.16E-03	4.69E-02
cg25416334		5	Intergenic	Island	46.52	3.87	50.84	5.57	-4.32	1.51E-04	3.93E-03
cg09259595	ARHGEF10	8	5'UTR	N_Shore	60.49	4.02	64.82	3.69	-4.32	2.40E-05	8.22E-04
cg01108445	C16orf59	16	TSS1500	N_Shore	59.60	5.40	63.92	6.02	-4.32	6.03E-04	1.17E-02
cg00928816	ARID5B	10	Body	Open sea	19.21	3.37	23.54	3.36	-4.32	2.21E-07	1.07E-05
cg23321397		6	Intergenic	Open sea	39.35	6.44	43.67	5.92	-4.32	1.12E-03	1.86E-02
cg08360009	GIMAP5	7	TSS1500	Open sea	57.79	5.86	62.12	4.44	-4.32	9.34E-05	2.65E-03
cg06231385	FOXK1	7	Body	Open sea	71.34	5.52	75.66	3.90	-4.32	1.01E-04	2.83E-03
cg04095339	IRX4	5	Body	Island	43.11	4.19	47.44	5.27	-4.32	1.70E-05	6.05E-04
cg12961733		22	Intergenic	Island	62.68	5.56	67.00	6.57	-4.32	8.15E-04	1.47E-02
cg13522891	ARHGEF10	8	3'UTR	Open sea	23.50	4.19	27.83	5.36	-4.32	2.95E-04	6.72E-03
cg25049733	GMDS	6	Body	Open sea	44.10	4.98	48.42	4.11	-4.32	1.31E-04	3.49E-03
cg03405593	MLLT1	19	3'UTR	Island	65.75	4.49	70.07	4.75	-4.32	1.34E-05	4.90E-04
cg09390414	KCNQ1;KCNQ1	11	Body;Body	Open sea	60.70	4.43	65.02	5.56	-4.32	5.40E-04	1.07E-02
cg02721602	C6orf168	6	Body	Open sea	50.66	4.77	54.98	6.36	-4.32	4.30E-04	9.02E-03
cg01761362	ACLY;ACLY	17	Body;Body	Open sea	50.84	5.52	55.16	6.04	-4.32	2.25E-03	3.07E-02

cg22932677	MAST1	19	Body	N_Shore	39.48	6.51	43.81	6.41	-4.32	3.66E-03	4.30E-02
cg23993337	FANK1	10	Body	Open sea	72.36	5.46	76.68	4.44	-4.32	8.19E-04	1.47E-02
cg20127899	NRG1;NRG1;NRG1;NRG1;NRG1;NRG1	8	Body;Body;Body;Body;Body;Body;Body;TS	Open sea	71.35	5.02	75.67	5.09	-4.32	5.97E-07	2.76E-05
cg02159913	JAG2;JAG2	14	TSS1500;TSS1500	Open sea	27.33	3.70	31.65	5.66	-4.32	1.09E-04	3.00E-03
cg14235701	FAM69B	9	TSS1500	N_Shore	37.29	5.64	41.61	6.37	-4.32	4.83E-04	9.85E-03
cg12905571		1	Intergenic	Open sea	54.86	5.75	59.18	6.13	-4.32	7.96E-04	1.44E-02
cg04731384	DBNDD1;DBNDD1	16	Body;TSS1500	Open sea	72.08	5.54	76.40	4.27	-4.32	1.32E-04	3.51E-03
cg07918174	GHRHR;GHRHR	7	TSS200;TSS200	Open sea	68.73	4.92	73.04	5.18	-4.32	1.08E-04	2.99E-03
cg02006647	EXOC3L2	19	TSS1500	Island	29.93	6.07	34.24	6.71	-4.32	4.08E-03	4.63E-02
cg22107147		2	Intergenic	Open sea	43.10	5.10	47.41	6.23	-4.32	7.49E-04	1.38E-02
cg18217706	TFF3	21	Body	Open sea	53.36	5.67	57.68	4.00	-4.32	1.27E-04	3.42E-03
cg10809485	FAM150A	8	Body	Open sea	19.36	4.46	23.67	6.59	-4.32	1.23E-03	2.00E-02
cg08657654		7	Intergenic	S_Shelf	29.13	6.09	33.45	4.54	-4.32	1.52E-03	2.33E-02
cg26289155	LLGL2;LLGL2;LLGL2	17	Body;Body;Body	Open sea	81.30	7.98	85.62	4.03	-4.32	1.16E-03	1.92E-02
cg03015672	ARHGAP12	10	5'UTR	N_Shore	24.51	4.95	28.82	5.95	-4.32	6.49E-04	1.24E-02
cg05350224	AES;AES;AES	19	3'UTR;3'UTR;3'UTR	Open sea	64.35	4.20	68.66	4.02	-4.32	3.32E-05	1.09E-03
cg12619509	DNASE1L2	16	5'UTR	Island	31.57	4.55	35.88	5.96	-4.32	1.37E-03	2.15E-02
cg08557649		15	Intergenic	Island	26.01	4.36	30.32	4.93	-4.32	6.45E-06	2.52E-04
cg07215003	PPP2CA	5	3'UTR	Open sea	74.18	4.83	78.50	3.27	-4.32	3.96E-05	1.27E-03
cg11671400		11	Intergenic	Open sea	38.75	7.62	43.07	7.65	-4.32	2.84E-03	3.61E-02
cg12349181	BAIAP2;BAIAP2;BAIAP2;BAIAP2	17	Body;Body;Body;Body	Open sea	36.19	5.56	40.51	6.45	-4.32	4.50E-03	4.95E-02
cg01629240	HOXB3	17	5'UTR	Island	21.44	5.21	25.76	4.98	-4.32	8.27E-04	1.48E-02
cg16190266	SSTR1	14	3'UTR	Island	36.22	5.84	40.53	5.90	-4.32	3.67E-05	1.19E-03
cg19843891		8	Intergenic	N_Shore	25.45	5.46	29.76	6.73	-4.32	3.03E-03	3.77E-02
cg02896970	ZCCHC14	16	Body	Open sea	62.89	5.34	67.20	5.76	-4.32	7.63E-04	1.40E-02
cg01233722	NFATC4;NFATC4	14	Body;Body	Island	32.91	7.00	37.23	7.90	-4.31	2.59E-03	3.39E-02
cg01776278	SSPO	7	TSS1500	S_Shore	53.68	4.04	58.00	4.31	-4.31	3.89E-05	1.25E-03
cg04060571	BCAR1;BCAR1;BCAR1;BCAR1;BCAR1;E	16	Body;Body;Body;Body;Body;Body;Body;Bc	N_Shore	29.56	3.63	33.87	4.14	-4.31	1.06E-05	3.96E-04
cg14659814	PRDM16;PRDM16	1	Body;Body	Open sea	37.88	4.74	42.19	6.55	-4.31	2.73E-03	3.51E-02
cg10911354		22	Intergenic	Open sea	55.04	5.46	59.35	5.05	-4.31	9.11E-04	1.60E-02
cg22242216	GRIN2D;GRWD1	19	3'UTR;TSS1500	Island	26.36	4.09	30.68	4.74	-4.31	9.42E-05	2.66E-03
cg14846617		14	Intergenic	Open sea	44.50	6.22	48.81	4.97	-4.31	2.51E-04	5.93E-03
cg01684233	ALDH1L1	3	5'UTR	N_Shelf	62.43	4.50	66.74	4.27	-4.31	7.27E-06	2.82E-04
cg02938601	TNFRSF10C	8	Body	S_Shore	29.10	4.86	33.41	6.16	-4.31	1.57E-04	4.06E-03
cg13080927	ABCF2	7	3'UTR	S_Shelf	55.07	4.09	59.38	4.45	-4.31	3.75E-05	1.21E-03
cg23499115	PEX5L	3	Body	Open sea	36.52	6.08	40.83	6.10	-4.31	7.84E-04	1.43E-02
cg18149394	STK25	2	Body	N_Shore	37.39	3.61	41.70	3.79	-4.31	1.33E-05	4.87E-04
cg26501049	FAM184A	6	TSS1500	Open sea	53.46	5.23	57.77	4.68	-4.31	9.35E-05	2.65E-03
cg07174843	SOX5	12	5'UTR	Open sea	64.84	6.15	69.15	5.62	-4.31	2.37E-03	3.19E-02
cg01634458		16	Intergenic	Island	46.48	5.99	50.79	5.16	-4.31	9.57E-04	1.66E-02
cg06756164	CLCC1;CLCC1	1	Body;Body	Open sea	60.07	5.82	64.38	5.12	-4.31	3.62E-04	7.89E-03
cg08855551	CSN1S2B	4	TSS1500	Open sea	60.56	7.36	64.87	7.68	-4.31	2.75E-03	3.53E-02
cg16281322	SH3D20	17	TSS200	S_Shore	56.19	3.50	60.50	3.92	-4.31	4.74E-07	2.22E-05
cg23730696	VSIG2	11	TSS1500	Open sea	63.20	6.45	67.51	5.32	-4.31	3.95E-03	4.53E-02
cg04007936	CARHSP1;CARHSP1	16	5'UTR;5'UTR	Open sea	45.30	4.55	49.61	4.32	-4.31	4.64E-05	1.46E-03
cg18973939	FLJ37453	1	Body	Island	14.79	3.94	19.10	7.04	-4.31	6.59E-04	1.25E-02
cg18813599	TMEM66	8	Body	N_Shelf	56.20	4.39	60.51	3.56	-4.31	1.38E-05	5.03E-04
cg04558553	UGT2B7	4	TSS1500	Open sea	42.07	5.25	46.38	5.04	-4.31	1.17E-03	1.92E-02
cg14760176		2	Intergenic	Open sea	54.23	5.43	58.54	6.07	-4.31	9.89E-04	1.70E-02
cg11289591	CKAP5;CKAP5	11	Body;Body	Open sea	55.80	6.51	60.11	5.00	-4.31	7.96E-04	1.44E-02
cg07441278		3	Intergenic	Open sea	53.27	4.05	57.58	5.05	-4.31	4.87E-06	1.95E-04
cg25811526	SLC4A5	2	TSS200	Open sea	57.09	4.38	61.40	4.74	-4.31	5.30E-06	2.11E-04
cg12078738	CGA	6	TSS200	Open sea	47.69	6.14	51.99	6.40	-4.31	3.96E-03	4.54E-02

cg04494791		16	Intergenic	Open sea	63.79	3.32	68.10	3.25	-4.31	2.06E-08	1.08E-06
cg02814525	ZBTB42	14	Body	Island	81.96	5.04	86.27	3.71	-4.31	2.12E-05	7.34E-04
cg03146733	TEKT5	16	Body	Open sea	79.75	6.18	84.06	5.00	-4.31	4.00E-04	8.52E-03
cg17408637	LMO2;LMO2;LMO2	11	Body;Body;Body	Open sea	63.08	3.64	67.38	3.08	-4.31	4.21E-07	1.99E-05
cg12811575		8	Intergenic	Open sea	40.81	4.94	45.12	4.71	-4.31	1.61E-04	4.15E-03
cg25560924	SLC4A4;SLC4A4;SLC4A4	4	Body;Body;Body	Open sea	71.94	4.34	76.25	4.44	-4.31	5.80E-06	2.29E-04
cg27042584		16	Intergenic	Island	35.52	5.78	39.83	6.23	-4.31	1.46E-03	2.26E-02
cg03178042		16	Intergenic	Island	47.36	4.45	51.66	4.12	-4.31	7.34E-05	2.16E-03
cg13996593	DNER	2	Body	N_Shelf	73.34	5.47	77.65	4.78	-4.31	2.66E-06	1.12E-04
cg06733281	INPP4B;INPP4B	4	Body;Body	Open sea	73.18	4.66	77.49	6.08	-4.31	8.10E-05	2.35E-03
cg27524531	WDR90	16	Body	N_Shore	49.27	5.07	53.58	5.63	-4.31	8.92E-04	1.57E-02
cg24996979	C14orf43;C14orf43	14	5'UTR;5'UTR	N_Shelf	20.87	3.92	25.18	3.40	-4.31	5.29E-07	2.46E-05
cg03069098	TIAM2	6	Body	Open sea	78.11	6.08	82.42	3.76	-4.31	6.96E-04	1.30E-02
cg15936385	CD81	11	Body	N_Shore	51.98	6.10	56.28	5.57	-4.31	3.08E-03	3.82E-02
cg20164964	TSPAN9;TSPAN9	12	5'UTR;5'UTR	Open sea	41.84	6.33	46.14	5.42	-4.31	3.11E-03	3.84E-02
cg06357743	RNF39;RNF39	6	Body;Body	N_Shore	66.00	4.88	70.31	6.84	-4.31	1.67E-03	2.48E-02
cg08972843	PGBD1	6	TSS1500	N_Shore	35.20	5.12	39.50	3.95	-4.30	1.20E-04	3.25E-03
cg07211239	SSU72	1	Body	S_Shore	34.86	4.52	39.16	6.23	-4.30	2.47E-03	3.28E-02
cg09082287	DNAJC6;DNAJC6	1	5'UTR;1stExon	N_Shore	14.59	4.65	18.89	5.95	-4.30	1.30E-04	3.48E-03
cg18335796	CALB1	8	Body	Open sea	35.33	4.63	39.64	6.17	-4.30	1.27E-03	2.05E-02
cg15841511		7	Intergenic	Open sea	71.76	6.41	76.06	5.98	-4.30	2.69E-03	3.47E-02
cg08406486	CUX2	12	Body	N_Shore	39.69	6.44	44.00	5.43	-4.30	2.29E-04	5.50E-03
cg11736167	PHLDB1;PHLDB1;PHLDB1	11	Body;Body;Body	Open sea	65.59	4.77	69.90	6.06	-4.30	7.05E-05	2.09E-03
cg14182890	C9orf50	9	3'UTR	S_Shore	57.18	5.96	61.48	5.43	-4.30	2.99E-04	6.78E-03
cg15818307		6	Intergenic	Island	37.55	5.66	41.85	4.53	-4.30	1.85E-04	4.64E-03
cg00352417	FAM43A;FAM43A	3	3'UTR;1stExon	Island	21.08	4.17	25.39	6.42	-4.30	5.95E-04	1.16E-02
cg20164601		7	Intergenic	Island	37.60	5.72	41.90	6.69	-4.30	2.30E-03	3.12E-02
cg27655716	FBRSL1	12	Body	N_Shore	41.29	2.82	45.59	2.51	-4.30	6.30E-09	3.39E-07
cg18450254	PRICKLE2	3	5'UTR	Open sea	53.48	8.02	57.78	8.12	-4.30	7.56E-04	1.39E-02
cg14878988	PP14571;GPC1	2	Body;Body	Island	28.13	4.35	32.43	4.21	-4.30	2.54E-05	8.63E-04
cg05609218	ITGA8	10	Body	N_Shore	30.45	5.43	34.75	5.75	-4.30	4.30E-04	9.03E-03
cg01127300		22	Intergenic	S_Shelf	28.76	7.30	33.06	7.36	-4.30	7.11E-04	1.33E-02
cg17915676	VWA3B	2	3'UTR	Open sea	46.48	5.48	50.78	5.64	-4.30	2.12E-03	2.94E-02
cg23752985	VAMP8	2	TSS1500	Open sea	69.37	4.15	73.67	3.72	-4.30	3.27E-05	1.08E-03
cg20961940	SLC44A4	6	Body	S_Shore	53.24	4.52	57.54	4.41	-4.30	4.70E-06	1.89E-04
cg26056498		8	Intergenic	Open sea	23.23	3.73	27.53	5.39	-4.30	5.26E-05	1.62E-03
cg11150353	H6PD	1	Body	Open sea	47.24	4.47	51.54	5.72	-4.30	1.35E-03	2.14E-02
cg12231088	BRD3	9	Body	N_Shore	79.83	5.29	84.12	4.65	-4.30	1.20E-03	1.96E-02
cg04453050	GRM2;GRM2	3	TSS200;TSS200	Island	33.94	4.11	38.24	3.74	-4.30	7.53E-07	3.44E-05
cg05778494		1	Intergenic	S_Shore	56.77	6.48	61.07	7.06	-4.30	2.94E-03	3.70E-02
cg02834909	PRSS50	3	TSS200	Island	28.07	6.16	32.37	7.12	-4.30	4.04E-03	4.60E-02
cg25756406		10	Intergenic	Island	44.61	4.57	48.91	4.81	-4.30	3.97E-05	1.27E-03
cg27409514	SBNO2	19	5'UTR	Island	30.60	4.97	34.90	5.75	-4.30	2.26E-03	3.08E-02
cg01807735		11	Intergenic	Open sea	52.08	5.74	56.37	4.99	-4.30	6.40E-04	1.23E-02
cg00333828	LRRK1	15	Body	Open sea	66.95	5.53	71.25	7.67	-4.30	2.17E-03	2.99E-02
cg07609862	MTNR1B	11	1stExon	Island	15.25	5.10	19.55	6.02	-4.30	4.07E-04	8.63E-03
cg05691152	TRIOBP	22	TSS200	Open sea	41.15	7.88	45.45	7.21	-4.30	3.43E-04	7.55E-03
cg27295742	RNMTL1	17	Body	Open sea	75.68	4.71	79.98	3.79	-4.30	2.88E-05	9.62E-04
cg20287434	ASPSCR1	17	Body	S_Shore	48.35	4.66	52.65	5.89	-4.30	1.01E-03	1.72E-02
cg09889948		17	Intergenic	Island	51.44	6.67	55.73	5.39	-4.30	3.32E-03	4.02E-02
cg19282250	PRUNE2	9	Body	Open sea	35.18	4.19	39.47	5.08	-4.30	1.52E-04	3.95E-03
cg03834116		2	Intergenic	Open sea	36.86	5.99	41.15	6.55	-4.30	3.87E-03	4.46E-02
cg17819119	SEMA4C	2	Body	N_Shore	74.27	5.43	78.57	4.21	-4.30	1.69E-03	2.51E-02

cg05152653	RTCD1;RTCD1	1	Body;Body	S_Shelf	52.95	6.16	57.25	5.19	-4.30	3.07E-03	3.81E-02
cg06994692	LOC389458	7	TSS200	Island	24.91	5.75	29.20	6.46	-4.30	4.26E-04	8.96E-03
cg02065148	SPON2;SPON2	4	Body;Body	N_Shore	70.31	5.93	74.61	6.61	-4.30	3.22E-03	3.93E-02
cg27438980	IGF1R	15	Body	S_Shore	36.60	4.20	40.89	6.60	-4.29	1.47E-03	2.27E-02
cg20216139		11	Intergenic	Island	24.22	4.28	28.51	4.86	-4.29	9.22E-05	2.62E-03
cg04395788	EXT1	8	Body	Open sea	54.97	6.51	59.26	6.89	-4.29	6.28E-04	1.21E-02
cg11093939	WDR25;WDR25	14	Body;Body	Open sea	72.53	5.48	76.82	5.03	-4.29	7.09E-04	1.32E-02
cg01383287	FBXO41	2	Body	N_Shore	42.12	5.51	46.42	5.53	-4.29	2.97E-03	3.72E-02
cg07020001	KDM2B;KDM2B	12	TSS200;TSS1500	S_Shore	43.43	6.93	47.72	6.55	-4.29	2.11E-04	5.15E-03
cg12688609	TTC39A	1	TSS1500	S_Shore	52.80	4.44	57.09	3.62	-4.29	1.57E-05	5.64E-04
cg23676042	CSMD3;CSMD3;CSMD3	8	Body;Body;TSS200	Open sea	79.84	5.17	84.13	3.88	-4.29	1.53E-05	5.52E-04
cg22202689		19	Intergenic	N_Shore	50.35	5.73	54.65	5.92	-4.29	1.50E-04	3.91E-03
cg01397449	EXOC3L	16	Body	S_Shore	48.49	4.62	52.78	4.59	-4.29	9.19E-05	2.61E-03
cg24928161	SHBG;SHBG;SHBG;SHBG;SHBG;SHBG	17	TSS1500;Body;TSS1500;Body;TSS1500;TSS	S_Shore	88.06	4.68	92.35	1.45	-4.29	1.28E-06	5.67E-05
cg19093370	PLD6	17	TSS1500	Island	29.91	4.12	34.20	6.00	-4.29	5.13E-04	1.03E-02
cg24867468	TMC6;TMC6	17	3'UTR;3'UTR	Open sea	70.17	4.63	74.46	5.57	-4.29	5.23E-04	1.05E-02
cg25674613		8	Intergenic	Open sea	49.41	6.13	53.70	6.50	-4.29	2.81E-03	3.58E-02
cg20273670		17	Intergenic	Island	26.18	3.74	30.47	7.16	-4.29	3.19E-04	7.14E-03
cg02216481	ALOX15B;ALOX15B;ALOX15B	17	TSS1500;TSS1500;TSS1500	Open sea	59.73	6.20	64.02	5.23	-4.29	2.27E-04	5.46E-03
cg02975081		16	Intergenic	Island	60.03	5.93	64.33	6.23	-4.29	4.16E-04	8.79E-03
cg12298445	ARAP1	11	5'UTR	S_Shore	57.47	3.92	61.76	3.69	-4.29	1.45E-06	6.34E-05
cg02235848	PDE10A;PDE10A	6	Body;Body	Open sea	66.43	4.96	70.72	4.28	-4.29	6.23E-05	1.88E-03
cg00184461	NOTCH1	9	Body	Island	38.00	3.65	42.29	5.07	-4.29	2.20E-04	5.34E-03
cg00017362		8	Intergenic	Open sea	77.57	5.13	81.86	4.94	-4.29	2.53E-04	5.97E-03
cg08285589	RAG1	11	3'UTR	Open sea	74.19	5.94	78.48	6.24	-4.29	2.41E-04	5.73E-03
cg26059790	PRKD1	14	Body	Open sea	65.04	5.77	69.33	5.26	-4.29	6.75E-05	2.01E-03
cg08142821		13	Intergenic	Open sea	77.96	5.81	82.25	5.83	-4.29	1.37E-03	2.16E-02
cg20628734		8	Intergenic	Open sea	67.81	4.30	72.10	5.03	-4.29	1.47E-05	5.31E-04
cg03807883		11	Intergenic	S_Shelf	29.47	4.32	33.76	3.84	-4.29	1.27E-05	4.67E-04
cg11166600	C3orf58;C3orf58	3	Body;Body	S_Shelf	74.60	6.99	78.89	5.63	-4.29	3.85E-03	4.45E-02
cg19379541		2	Intergenic	Open sea	70.30	3.34	74.59	3.49	-4.29	3.55E-08	1.83E-06
cg04138181	PLK1	16	Body	S_Shore	41.63	6.52	45.92	8.26	-4.29	3.00E-03	3.75E-02
cg04387073		6	Intergenic	Open sea	55.58	2.81	59.87	3.69	-4.29	1.69E-08	8.89E-07
cg15896978	APOA1	11	TSS1500	S_Shelf	56.35	4.90	60.64	5.25	-4.29	1.26E-04	3.38E-03
cg01648999	KBTBD11	8	5'UTR	S_Shore	62.58	4.28	66.86	4.77	-4.29	4.58E-07	2.15E-05
cg02343602		11	Intergenic	Open sea	53.62	5.15	57.91	5.47	-4.29	8.84E-04	1.56E-02
cg03953232	C7orf31	7	Body	N_Shelf	79.00	5.26	83.28	3.87	-4.29	8.68E-05	2.49E-03
cg02127234	SGK3;SGK3;SGK3	8	5'UTR;5'UTR;5'UTR	S_Shore	66.83	5.29	71.12	4.71	-4.29	4.09E-04	8.66E-03
cg24179445		10	Intergenic	N_Shore	45.65	5.03	49.93	5.24	-4.29	8.09E-04	1.46E-02
cg21002957	RASAL3	19	Body	Island	36.64	6.16	40.93	6.17	-4.29	2.50E-03	3.30E-02
cg05508408	DCUN1D2	13	Body	Open sea	85.08	5.86	89.37	4.00	-4.29	1.74E-03	2.56E-02
cg04352272		17	Intergenic	Island	16.59	5.23	20.88	7.01	-4.29	3.60E-03	4.25E-02
cg12768447	CTNNA3;CTNNA3	10	TSS1500;5'UTR	Open sea	61.77	6.30	66.05	6.41	-4.29	5.20E-04	1.04E-02
cg01973695		11	Intergenic	Island	25.60	4.63	29.88	5.87	-4.29	1.07E-03	1.80E-02
cg23701314	UBR1	15	Body	Open sea	60.18	7.15	64.47	5.98	-4.28	6.40E-04	1.23E-02
cg26550872	CPSF6	12	Body	Open sea	69.88	6.01	74.17	6.66	-4.28	2.93E-04	6.67E-03
cg00062070	C15orf41;C15orf41	15	Body;TSS1500	Open sea	64.84	6.37	69.12	4.82	-4.28	1.26E-03	2.04E-02
cg11736657	CALML3	10	TSS200	N_Shore	49.93	4.24	54.21	5.42	-4.28	4.18E-04	8.81E-03
cg18672053		5	Intergenic	Open sea	62.99	5.42	67.27	7.55	-4.28	4.06E-03	4.61E-02
cg05504729	ANKRD11	16	5'UTR	Open sea	39.54	5.25	43.82	4.26	-4.28	1.07E-05	4.01E-04
cg02992048	DLG2	11	Body	Open sea	76.22	4.89	80.51	4.65	-4.28	7.91E-05	2.30E-03
cg24650267		10	Intergenic	S_Shore	53.11	5.90	57.39	6.43	-4.28	2.51E-03	3.31E-02
cg16499677	C14orf37	14	Body	Open sea	71.87	4.98	76.16	4.88	-4.28	1.91E-04	4.77E-03

cg08461352	FAM110B	8	5'UTR	S_Shelf	43.65	6.69	47.94	5.50	-4.28	2.10E-04	5.13E-03
cg18987026	LOC286002	7	Body	N_Shelf	80.96	5.59	85.24	5.61	-4.28	2.38E-04	5.69E-03
cg18266783	MEF2D	1	5'UTR	N_Shelf	28.02	4.33	32.30	5.51	-4.28	4.47E-04	9.30E-03
cg01534316	ADORA3	1	Body	S_Shelf	49.63	5.95	53.92	5.98	-4.28	4.04E-03	4.59E-02
cg10314133	C1orf86;LOC100128003;C1orf86	1	Body;TSS200;Body	N_Shore	57.80	5.28	62.08	5.92	-4.28	2.09E-03	2.91E-02
cg03239638		21	Intergenic	Island	15.74	2.55	20.02	3.78	-4.28	1.63E-08	8.56E-07
cg26732544	ZNF438;ZNF438;ZNF438;ZNF438;ZNF4	10	5'UTR;5'UTR;5'UTR;5'UTR;5'UTR;5'UTR;Bo	Open sea	64.88	4.69	69.16	4.45	-4.28	7.20E-07	3.30E-05
cg00755423	RGS8;RGS8	1	TSS200;TSS200	Open sea	78.74	4.26	83.02	5.46	-4.28	1.05E-05	3.92E-04
cg17457379	PDE4DIP	1	Body	Open sea	74.52	3.59	78.79	3.45	-4.28	7.39E-07	3.38E-05
cg12787836	DOPEY2	21	TSS200	Open sea	32.05	4.33	36.33	4.95	-4.28	2.33E-04	5.59E-03
cg27268279	COL13A1;COL13A1;COL13A1;COL13A1	10	Body;Body;Body;Body;Body;Body;Body;Bo	Open sea	56.58	5.58	60.86	4.57	-4.28	7.19E-04	1.34E-02
cg26185079	SLMO1;SLMO1	18	Body;Body	S_Shelf	69.76	5.59	74.04	5.35	-4.28	1.03E-03	1.75E-02
cg04845466	KRTCAP3;NRBP1;KRTCAP3	2	TSS200;3'UTR;TSS200	Island	58.10	5.04	62.38	5.41	-4.28	8.68E-04	1.54E-02
cg05390356	KIF1B;KIF1B	1	5'UTR;5'UTR	Open sea	53.71	5.66	57.99	5.69	-4.28	1.55E-03	2.36E-02
cg16735509	PRKCZ;PRKCZ	1	Body;5'UTR	N_Shelf	60.46	5.80	64.74	5.59	-4.28	2.78E-03	3.56E-02
cg17153133		6	Intergenic	Open sea	76.83	5.19	81.11	3.69	-4.28	3.67E-04	7.96E-03
cg08860136	DLG4;DLG4	17	Body;Body	S_Shelf	35.73	3.43	40.00	3.94	-4.28	1.80E-06	7.76E-05
cg09753006	CFHR2	1	Body	Open sea	74.38	4.66	78.66	4.09	-4.28	1.89E-05	6.64E-04
cg23017573	PIP4K2A	10	Body	Open sea	60.04	5.11	64.32	4.38	-4.28	4.72E-04	9.69E-03
cg25753473		6	Intergenic	S_Shelf	39.62	6.27	43.89	7.12	-4.28	1.80E-03	2.63E-02
cg22018083	NAPSA	19	3'UTR	Island	30.07	6.33	34.35	6.27	-4.28	3.53E-03	4.19E-02
cg02952379	PLCZ1	12	Body	Open sea	80.84	4.65	85.11	2.87	-4.28	4.15E-06	1.69E-04
cg02305377		12	Intergenic	N_Shelf	21.34	4.79	25.61	5.08	-4.28	4.24E-05	1.35E-03
cg21786372	PRDM16;PRDM16	1	Body;Body	N_Shore	55.45	6.17	59.73	5.68	-4.28	2.76E-03	3.54E-02
cg06666376	C19orf77	19	TSS200	N_Shore	47.91	5.61	52.18	4.91	-4.28	1.25E-03	2.02E-02
cg01172872		7	Intergenic	N_Shore	66.53	5.20	70.81	3.96	-4.28	8.72E-05	2.50E-03
cg03054080		14	Intergenic	Open sea	60.73	5.38	65.00	5.00	-4.28	1.11E-03	1.85E-02
cg12479098	PRKCDBP	11	TSS1500	S_Shore	39.92	4.30	44.20	5.09	-4.27	1.45E-05	5.27E-04
cg03238709	FCGBP	19	Body	N_Shelf	60.92	6.19	65.19	5.32	-4.27	1.75E-03	2.57E-02
cg14974938	LPHN3	4	Body	Open sea	40.85	4.21	45.12	4.36	-4.27	1.23E-05	4.53E-04
cg00301660		8	Intergenic	Island	44.00	4.23	48.27	4.92	-4.27	1.17E-04	3.20E-03
cg20606255	KIAA1755	20	3'UTR	Open sea	25.98	4.64	30.26	4.48	-4.27	4.82E-05	1.51E-03
cg10012448	FARS2	6	Body	Open sea	72.95	4.55	77.22	3.96	-4.27	1.84E-05	6.50E-04
cg02002551	NEFM;NEFM	8	5'UTR;Body	S_Shore	18.26	3.58	22.53	4.44	-4.27	6.05E-06	2.38E-04
cg17576603	DAB2	5	5'UTR	Open sea	71.30	7.07	75.57	5.96	-4.27	4.25E-04	8.94E-03
cg27499185	ACSBG2	19	5'UTR	Open sea	44.05	5.23	48.32	5.90	-4.27	7.37E-05	2.16E-03
cg13228934	CCDC109A	10	Body	Open sea	70.51	5.11	74.78	5.17	-4.27	2.55E-04	5.99E-03
cg01447828	PRX;PRX	19	TSS200;TSS200	Open sea	54.88	4.70	59.15	3.89	-4.27	3.31E-04	7.36E-03
cg18023263	DLG2;DLG2	11	Body;Body	Open sea	59.44	5.14	63.71	4.89	-4.27	3.01E-05	1.00E-03
cg26654807	ZMIZ1	10	Body	Island	44.92	4.55	49.19	4.90	-4.27	4.32E-05	1.37E-03
cg27247563		10	Intergenic	Open sea	67.26	7.26	71.54	5.60	-4.27	1.62E-03	2.43E-02
cg01641930	PSMD9	12	TSS1500	N_Shore	75.11	5.91	79.38	5.31	-4.27	5.51E-04	1.09E-02
cg15726557	POLRMT	19	3'UTR	Island	83.02	3.40	87.29	2.93	-4.27	4.36E-09	2.36E-07
cg07138768	C7orf20	7	Body	Island	33.46	3.77	37.73	4.35	-4.27	1.68E-05	6.00E-04
cg23580725	BCL11B;BCL11B	14	Body;Body	Open sea	51.38	6.05	55.65	7.24	-4.27	2.63E-05	8.90E-04
cg00354381	SLC43A3;SLC43A3;SLC43A3	11	TSS1500;TSS200;TSS1500	S_Shore	32.92	5.56	37.19	5.13	-4.27	7.46E-04	1.37E-02
cg15904523	ZNF233	19	TSS200	Island	13.49	3.79	17.76	3.48	-4.27	3.34E-09	1.82E-07
cg04891611		15	Intergenic	Island	71.86	6.17	76.13	4.17	-4.27	2.27E-03	3.09E-02
cg16206364	CPT1C;CPT1C	19	Body;Body	S_Shore	67.60	6.44	71.87	4.93	-4.27	3.47E-03	4.14E-02
cg06618629	SPNS2	17	Body	N_Shelf	50.20	7.13	54.47	5.82	-4.27	2.29E-04	5.50E-03
cg25070565	CYTSB;CYTSB	17	Body;Body	Open sea	67.62	5.10	71.89	4.82	-4.27	1.09E-04	3.01E-03
cg12061236	AKAP12	6	TSS1500	Island	32.60	5.23	36.87	6.25	-4.27	1.53E-03	2.33E-02
cg25987417	PPEF2;PPEF2	4	1stExon;5'UTR	Open sea	81.70	3.95	85.96	3.10	-4.27	4.91E-07	2.30E-05

cg17137063	PLEKHG5;PLEKHG5	1	Body;Body	N_Shelf	68.21	4.75	72.48	5.04	-4.27	9.12E-05	2.59E-03
cg10021122	TTF2	1	Body	Open sea	20.94	4.14	25.21	5.93	-4.27	1.28E-03	2.06E-02
cg13655635	NR4A3;NR4A3;NR4A3;NR4A3	9	5'UTR;5'UTR;5'UTR;TSS1500	S_Shore	60.06	5.07	64.33	5.03	-4.27	6.41E-05	1.93E-03
cg17400285		15	Intergenic	Open sea	62.31	4.29	66.58	4.71	-4.27	1.23E-04	3.33E-03
cg24125525	AGAP3;AGAP3	7	Body;Body	N_Shore	82.12	5.04	86.38	2.70	-4.27	5.71E-05	1.74E-03
cg16528345		6	Intergenic	Open sea	78.62	5.01	82.89	4.95	-4.27	3.33E-04	7.38E-03
cg03736099		17	Intergenic	S_Shore	57.52	4.82	61.78	4.35	-4.27	4.80E-05	1.50E-03
cg06206086	EIF2C3;EIF2C3	1	5'UTR;Body	Open sea	80.53	6.55	84.79	5.59	-4.27	5.49E-04	1.09E-02
cg12690575	C11orf9;C11orf9	11	Body;Body	Open sea	47.71	4.43	51.98	5.75	-4.27	1.35E-04	3.59E-03
cg24499462	CSNK1A1P;C15orf41;C15orf41	15	Body;Body;Body	Open sea	59.03	5.47	63.29	6.29	-4.26	3.53E-03	4.19E-02
cg00997338	TRIM71	3	Body	N_Shore	77.35	4.69	81.62	3.99	-4.26	9.55E-05	2.69E-03
cg22172494	H19	11	Body	N_Shore	48.94	3.80	53.20	4.42	-4.26	8.74E-06	3.33E-04
cg10435454	MYOF;MYOF	10	Body;Body	Open sea	82.24	3.84	86.51	4.95	-4.26	5.02E-06	2.01E-04
cg10706230	MAN1C1	1	1stExon	Island	43.63	4.73	47.89	5.42	-4.26	8.33E-06	3.19E-04
cg02097120	TDRD10;TDRD10	1	Body;Body	Open sea	39.13	7.27	43.39	6.73	-4.26	1.46E-03	2.26E-02
cg20891655	CCDC83	11	3'UTR	Open sea	76.38	6.44	80.64	5.30	-4.26	9.97E-04	1.71E-02
cg15988345	GALE;GALE;GALE	1	3'UTR;3'UTR;3'UTR	Open sea	54.39	4.03	58.65	4.12	-4.26	5.05E-05	1.57E-03
cg09370463	ARHGAP18	6	Body	Open sea	83.17	7.21	87.43	6.38	-4.26	1.04E-03	1.76E-02
cg27518892	CCDC102A	16	5'UTR	N_Shelf	54.03	4.15	58.29	3.77	-4.26	4.35E-06	1.76E-04
cg15708219		10	Intergenic	N_Shore	83.24	5.08	87.50	4.30	-4.26	8.43E-06	3.23E-04
cg12913832	SLC7A7;SLC7A7;SLC7A7	14	Body;Body;Body	Open sea	76.86	3.97	81.12	3.85	-4.26	3.31E-06	1.37E-04
cg22306579	ZBTB12	6	Body	Island	32.69	2.24	36.95	4.18	-4.26	2.03E-07	9.89E-06
cg24635109	IPCEF1;IPCEF1;IPCEF1	6	5'UTR;5'UTR;5'UTR	Open sea	56.84	6.09	61.10	5.55	-4.26	1.41E-03	2.21E-02
cg22586996	EBF1	5	Body	Island	19.86	4.77	24.12	4.24	-4.26	8.28E-05	2.39E-03
cg10790685	VIM	10	TSS1500	N_Shore	49.77	5.34	54.03	4.87	-4.26	7.91E-04	1.44E-02
cg19435526	LRRC8E	19	3'UTR	S_Shore	60.04	4.65	64.30	3.60	-4.26	8.70E-06	3.32E-04
cg18597188	XRCC3;XRCC3;XRCC3	14	5'UTR;5'UTR;5'UTR	N_Shelf	46.25	4.68	50.50	5.04	-4.26	1.04E-03	1.76E-02
cg01203153		5	Intergenic	N_Shore	34.95	5.53	39.21	6.13	-4.26	1.88E-04	4.70E-03
cg10788939	EFNA5	5	Body	Open sea	74.38	4.88	78.64	4.87	-4.26	1.03E-04	2.88E-03
cg21097354		7	Intergenic	Island	46.27	5.53	50.53	6.25	-4.26	2.94E-03	3.69E-02
cg16016431	PHRF1	11	Body	N_Shore	61.69	4.76	65.95	5.08	-4.26	8.02E-04	1.45E-02
cg10163299	OTOR	20	TSS200	Open sea	68.80	4.66	73.06	4.70	-4.26	1.63E-05	5.84E-04
cg04388657	RPL35	9	Body	N_Shore	31.91	4.82	36.16	5.47	-4.26	9.27E-05	2.63E-03
cg12222588	BRUNOL4;BRUNOL4;BRUNOL4;BRUN(	18	3'UTR;3'UTR;3'UTR;3'UTR	Island	30.12	5.28	34.38	6.15	-4.26	4.55E-04	9.43E-03
cg06328100	ITPR3	6	Body	Open sea	81.95	4.61	86.20	3.79	-4.26	3.72E-05	1.20E-03
cg19805033	FRMD4A	10	Body	Open sea	68.07	5.73	72.32	4.45	-4.26	7.51E-05	2.20E-03
cg18118198	GZMB	14	Body	Open sea	70.40	6.79	74.66	3.92	-4.26	7.60E-05	2.22E-03
cg08494390	AFF1;AFF1	4	Body;Body	Open sea	59.97	4.47	64.23	3.77	-4.26	1.13E-04	3.09E-03
cg16409977		12	Intergenic	Open sea	69.52	4.25	73.78	4.10	-4.25	2.67E-05	9.03E-04
cg26217813	ASPA;ASPA	17	TSS1500;5'UTR	S_Shelf	36.79	4.51	41.04	4.87	-4.25	6.32E-04	1.21E-02
cg05797594	MIR1910;C16orf74	16	TSS1500;5'UTR	Open sea	59.70	4.78	63.95	4.62	-4.25	2.28E-04	5.49E-03
cg02052726	DCPS	11	Body	Open sea	22.92	3.48	27.18	4.20	-4.25	1.65E-06	7.18E-05
cg24621371		12	Intergenic	N_Shelf	58.73	4.06	62.98	4.21	-4.25	4.67E-05	1.47E-03
cg02162720		11	Intergenic	S_Shelf	69.27	4.71	73.53	4.60	-4.25	1.90E-05	6.68E-04
cg08142096	C11orf53	11	TSS1500	Open sea	36.72	5.67	40.98	6.64	-4.25	2.08E-04	5.09E-03
cg20710258		3	Intergenic	Open sea	48.34	5.49	52.59	5.39	-4.25	3.26E-03	3.97E-02
cg02977954	ECHDC2	1	Body	Open sea	57.40	4.56	61.66	4.74	-4.25	1.24E-04	3.34E-03
cg22707446		2	Intergenic	Open sea	39.04	2.85	43.30	3.88	-4.25	1.82E-06	7.86E-05
cg25233024	SPTBN4;SPTBN4	19	3'UTR;Body	Open sea	66.30	5.68	70.55	4.21	-4.25	1.28E-04	3.42E-03
cg03151319	LY6G5B;CSNK2B	6	TSS1500;Body	S_Shelf	73.50	4.39	77.75	3.67	-4.25	3.16E-05	1.04E-03
cg00929322		7	Intergenic	Open sea	71.91	5.45	76.16	4.69	-4.25	7.63E-04	1.40E-02
cg03190219		7	Intergenic	Island	30.82	5.45	35.07	6.25	-4.25	2.53E-03	3.33E-02
cg25866143	HOXA5;HOXA5	7	5'UTR;1stExon	Island	28.11	4.79	32.36	5.57	-4.25	1.11E-03	1.85E-02

cg19316897		1	Intergenic	Open sea	68.41	4.32	72.66	4.41	-4.25	9.07E-05	2.58E-03
cg08580254	RARA;RARA;RARA	17	5'UTR;5'UTR;5'UTR	S_Shelf	6.60	1.14	10.85	2.82	-4.25	7.64E-18	5.27E-16
cg01797043	RPL3L	16	TSS200	Open sea	37.80	2.77	42.05	3.36	-4.25	1.87E-08	9.81E-07
cg07355688	BARX2	11	Body	S_Shore	25.80	4.35	30.05	5.76	-4.25	7.29E-05	2.15E-03
cg13388830	NOLC1	10	TSS1500	N_Shore	67.59	4.79	71.84	4.46	-4.25	1.98E-05	6.92E-04
cg06550704	ITGB4;ITGB4;ITGB4	17	Body;Body;Body	Island	62.00	5.66	66.25	6.35	-4.25	4.04E-03	4.59E-02
cg25665606	KLHDC7B	22	TSS1500	Island	69.47	7.28	73.72	4.09	-4.25	1.44E-03	2.23E-02
cg00873517	KHDRBS3	8	Body	Open sea	47.81	7.32	52.06	6.71	-4.25	1.64E-03	2.45E-02
cg12486533	BMP1;SFTPC;BMP1;BMP1	8	TSS1500;Body;TSS1500;TSS1500	Open sea	39.12	3.82	43.37	3.41	-4.25	3.32E-07	1.59E-05
cg11084334	LHFPL4	3	Body	Island	58.01	4.85	62.26	5.85	-4.25	4.02E-04	8.55E-03
cg17291767	TRERF1	6	Body	S_Shelf	83.52	5.86	87.77	4.62	-4.25	2.25E-03	3.07E-02
cg13955931		2	Intergenic	Island	33.69	5.17	37.94	6.18	-4.25	3.54E-03	4.20E-02
cg27311341		12	Intergenic	Open sea	42.02	5.93	46.27	4.08	-4.25	3.92E-04	8.38E-03
cg01679108	SALL1;SALL1	16	Body;Body	Open sea	27.33	3.68	31.57	4.40	-4.25	1.93E-05	6.76E-04
cg08333283		12	Intergenic	S_Shore	46.35	5.18	50.60	5.35	-4.25	6.35E-04	1.22E-02
cg01883195	DCBLD1	6	TSS1500	N_Shore	62.25	4.90	66.49	5.73	-4.25	3.68E-04	7.99E-03
cg21140898		1	Intergenic	N_Shore	21.54	4.70	25.78	5.71	-4.24	8.23E-04	1.48E-02
cg10586317	CD276;CD276	15	5'UTR;5'UTR	S_Shore	26.39	6.13	30.64	8.15	-4.24	1.64E-03	2.45E-02
cg07052568	NFIX	19	Body	S_Shelf	45.15	4.65	49.39	6.49	-4.24	1.53E-03	2.34E-02
cg10505024	KEAP1;KEAP1	19	Body;Body	Island	71.17	5.55	75.41	5.59	-4.24	3.81E-03	4.41E-02
cg00639475		12	Intergenic	Open sea	59.84	3.84	64.08	3.39	-4.24	6.84E-07	3.14E-05
cg00546153	PPIE;PPIE;PPIE	1	5'UTR;Body;Body	S_Shore	64.57	4.70	68.81	4.22	-4.24	1.59E-04	4.11E-03
cg17999642	LY6G5C	6	Body	N_Shelf	77.36	4.74	81.60	3.89	-4.24	1.81E-05	6.41E-04
cg12173150		6	Intergenic	Island	37.08	4.75	41.32	5.36	-4.24	1.07E-04	2.96E-03
cg02815014	PLEKHM3	2	Body	N_Shelf	75.07	4.34	79.31	3.48	-4.24	4.18E-06	1.70E-04
cg10620305	TRIM4;TRIM4	7	3'UTR;3'UTR	Open sea	68.18	2.88	72.42	3.11	-4.24	3.15E-08	1.63E-06
cg09030212	NTNG1;NTNG1;NTNG1	1	TSS1500;TSS1500;TSS1500	N_Shore	34.91	4.84	39.15	7.09	-4.24	2.54E-04	5.98E-03
cg18451035	STRN3;STRN3	14	Body;Body	Open sea	78.59	7.92	82.83	4.69	-4.24	3.38E-03	4.07E-02
cg02991753	SYNPO2	4	Body	Open sea	77.06	4.53	81.30	4.01	-4.24	3.03E-06	1.26E-04
cg06421138	REV3L	6	Body	Open sea	72.18	4.30	76.42	3.53	-4.24	1.36E-05	4.96E-04
cg20486986		1	Intergenic	Open sea	67.87	5.75	72.10	4.00	-4.24	9.17E-04	1.61E-02
cg13427748		15	Intergenic	Open sea	27.35	3.41	31.59	4.37	-4.24	5.97E-06	2.35E-04
cg02009648	MRPL14	6	5'UTR	N_Shelf	34.33	5.54	38.57	5.64	-4.24	3.02E-03	3.76E-02
cg00337703	TDRD1	10	TSS1500	N_Shore	45.39	6.21	49.63	6.56	-4.24	4.21E-03	4.72E-02
cg03630056	CYP11A1;CYP11A1	15	Body;5'UTR	Open sea	83.16	3.44	87.40	2.53	-4.24	1.17E-09	6.52E-08
cg01192745		3	Intergenic	Open sea	74.95	5.03	79.19	4.71	-4.24	2.55E-05	8.66E-04
cg05136471	PLXNC1	12	Body	N_Shelf	48.09	4.77	52.33	5.33	-4.24	1.04E-03	1.76E-02
cg03933290	C17orf85;C17orf85	17	Body;Body	Open sea	69.43	6.97	73.67	6.24	-4.24	4.06E-03	4.61E-02
cg24091982	SCOC	4	5'UTR	Open sea	80.94	5.61	85.18	3.56	-4.24	1.15E-04	3.14E-03
cg14559409		10	Intergenic	Island	47.22	4.85	51.46	5.80	-4.24	5.99E-04	1.16E-02
cg27492103	ASXL2	2	Body	Open sea	49.56	4.62	53.80	4.75	-4.24	1.68E-04	4.29E-03
cg21467717	KCNG2	18	TSS1500	Island	83.66	7.19	87.90	4.81	-4.24	3.10E-03	3.83E-02
cg18986048	MYPN	10	Body	Open sea	35.19	5.50	39.43	5.87	-4.24	1.55E-03	2.35E-02
cg24433207	STXBP2;STXBP2	19	Body;Body	Island	84.62	5.75	88.86	2.48	-4.24	2.53E-04	5.96E-03
cg16678446	GLI4	8	Body	S_Shelf	61.71	4.52	65.94	3.72	-4.24	1.37E-04	3.64E-03
cg04334496		6	Intergenic	Open sea	63.03	5.13	67.27	4.95	-4.24	8.01E-06	3.08E-04
cg10506715	C14orf68	14	Body	N_Shore	49.69	4.33	53.93	6.46	-4.24	2.78E-03	3.56E-02
cg12178900	ATP7B;ATP7B	13	Body;Body	Open sea	46.63	5.84	50.86	6.25	-4.24	1.31E-03	2.09E-02
cg11620672		6	Intergenic	Open sea	31.77	4.92	36.00	5.82	-4.24	3.49E-03	4.16E-02
cg10972873	NR5A2;NR5A2	1	Body;Body	N_Shore	25.95	4.28	30.19	6.06	-4.24	1.00E-04	2.81E-03
cg05996213	TNR;TNR	1	5'UTR;1stExon	Open sea	41.16	5.36	45.40	6.30	-4.24	8.63E-04	1.53E-02
cg21107103	SPTY2D1	11	3'UTR	Open sea	59.78	6.32	64.02	6.08	-4.23	3.90E-03	4.49E-02
cg12412902	PACSIN1	6	Body	Island	77.86	6.20	82.09	4.57	-4.23	8.57E-04	1.53E-02

cg07093389		17	Intergenic	Island	24.67	4.28	28.91	5.48	-4.23	3.28E-04	7.31E-03
cg00801512		17	Intergenic	Open sea	11.99	3.13	16.23	5.54	-4.23	7.33E-06	2.84E-04
cg19351398	SSPO	7	TSS1500	S_Shore	43.62	4.64	47.85	4.83	-4.23	6.69E-04	1.27E-02
cg09665406		1	Intergenic	Open sea	69.86	4.72	74.09	4.41	-4.23	1.47E-04	3.84E-03
cg00325918	ACOXL;ACOXL;ACOXL	2	5'UTR;5'UTR;5'UTR	Open sea	70.49	5.07	74.73	4.41	-4.23	7.04E-05	2.09E-03
cg14160968		4	Intergenic	N_Shore	51.42	6.35	55.65	4.27	-4.23	1.81E-03	2.64E-02
cg07169618	SLC35F3	1	3'UTR	Open sea	79.85	7.31	84.08	5.61	-4.23	1.52E-03	2.32E-02
cg13624631	DHRS7	14	Body	Open sea	25.41	6.32	29.64	6.97	-4.23	3.06E-04	6.90E-03
cg02714994		19	Intergenic	N_Shore	49.75	3.74	53.98	4.12	-4.23	9.52E-06	3.60E-04
cg09825146	TRMT12	8	TSS200	Open sea	10.19	4.15	14.42	8.35	-4.23	9.98E-04	1.71E-02
cg22088248	ZADH2	18	Body	S_Shore	71.60	5.55	75.83	5.04	-4.23	1.60E-04	4.14E-03
cg14718995		3	Intergenic	Open sea	70.07	4.87	74.30	4.96	-4.23	5.05E-05	1.57E-03
cg19084059	C15orf39	15	Body	Island	86.22	4.54	90.45	3.42	-4.23	1.37E-05	4.99E-04
cg10062065	SPEG	2	Body	N_Shore	80.73	6.05	84.96	5.68	-4.23	2.54E-03	3.33E-02
cg03035495		6	Intergenic	Open sea	74.87	5.07	79.10	4.01	-4.23	3.51E-04	7.69E-03
cg11959316	PPAP2B;PPAP2B	1	Body;Body	Open sea	47.14	4.44	51.37	4.40	-4.23	7.57E-05	2.22E-03
cg11689700	ABCD3	1	3'UTR	Open sea	80.33	4.64	84.56	3.85	-4.23	1.06E-04	2.93E-03
cg21418854	C1orf113;C1orf113	1	TSS1500;TSS1500	N_Shore	49.93	3.89	54.16	4.02	-4.23	6.55E-05	1.96E-03
cg02277868	MYOT;MYOT	5	Body;Body	N_Shelf	77.15	5.15	81.38	5.29	-4.23	8.32E-05	2.40E-03
cg14250881	KIAA1161	9	3'UTR	Island	71.65	4.90	75.88	5.71	-4.23	2.47E-03	3.28E-02
cg04995717	TEK;TEK	9	5'UTR;1stExon	Open sea	41.06	6.60	45.29	5.44	-4.23	1.89E-03	2.72E-02
cg13368923		6	Intergenic	Island	19.57	4.34	23.80	4.26	-4.23	1.07E-05	4.01E-04
cg03018562		19	Intergenic	N_Shelf	83.31	5.62	87.54	4.37	-4.23	5.73E-04	1.12E-02
cg16155207		10	Intergenic	S_Shelf	63.84	4.92	68.07	5.91	-4.23	6.88E-04	1.29E-02
cg20249839	HRC;HRC	19	5'UTR;1stExon	N_Shelf	64.73	4.34	68.96	4.19	-4.23	2.66E-05	9.00E-04
cg01588224	JTB	1	TSS1500	S_Shore	29.30	4.84	33.53	5.43	-4.23	1.61E-03	2.43E-02
cg10614773	EEF1D;EEF1D;EEF1D;EEF1D;EEF1D;EE	8	Body;Body;Body;Body;Body;Body;Body	Open sea	65.28	4.64	69.51	5.30	-4.23	4.81E-04	9.83E-03
cg21432062		3	Intergenic	N_Shore	26.51	4.55	30.73	5.32	-4.23	3.22E-04	7.19E-03
cg04066265	CSGALNACT1;CSGALNACT1;CSGALNA	8	TSS200;Body;5'UTR	S_Shore	30.51	5.27	34.74	6.03	-4.23	1.95E-03	2.77E-02
cg00424354		11	Intergenic	N_Shelf	39.13	4.72	43.36	4.75	-4.23	6.96E-04	1.31E-02
cg18649438	NTRK1;INSRR	1	Body;TSS1500	Island	18.35	3.10	22.58	3.97	-4.23	5.08E-08	2.59E-06
cg08491661	DPPA2	3	3'UTR	Open sea	65.45	4.59	69.68	4.36	-4.23	1.12E-04	3.06E-03
cg23854008	LOC650368	11	Body	S_Shelf	57.04	5.28	61.27	4.67	-4.23	1.16E-03	1.92E-02
cg03623097	ARMS2	10	TSS1500	Open sea	56.67	6.49	60.90	5.14	-4.23	1.21E-03	1.97E-02
cg19186089	ATP5G1;ATP5G1	17	3'UTR;3'UTR	S_Shelf	55.53	3.66	59.76	3.64	-4.23	2.49E-06	1.05E-04
cg10900455	TOX2;TOX2;TOX2	20	5'UTR;Body;5'UTR	Island	16.11	4.85	20.34	6.50	-4.23	7.10E-04	1.32E-02
cg14991764		6	Intergenic	Open sea	61.85	6.28	66.07	6.24	-4.23	4.44E-04	9.23E-03
cg02347212		17	Intergenic	Open sea	68.12	5.66	72.35	6.11	-4.23	6.76E-04	1.28E-02
cg18786123		12	Intergenic	Open sea	28.88	5.02	33.10	3.79	-4.23	2.57E-06	1.08E-04
cg09949789	TBC1D14;TBC1D14;TBC1D14	4	Body;Body;Body	S_Shore	60.12	4.76	64.34	4.54	-4.23	2.22E-04	5.38E-03
cg12600174	HOXA9	7	TSS200	Island	12.76	2.97	16.98	4.72	-4.22	2.80E-07	1.35E-05
cg01369479		2	Intergenic	Open sea	84.92	6.31	89.14	4.39	-4.22	1.28E-03	2.05E-02
cg13425174	DEF6	6	TSS200	Open sea	35.06	4.94	39.28	4.81	-4.22	6.68E-06	2.61E-04
cg01010211	CECR2	22	TSS200	Open sea	45.12	5.25	49.35	5.65	-4.22	2.34E-04	5.59E-03
cg00232483	IQCK	16	Body	Open sea	59.18	4.40	63.40	5.38	-4.22	6.68E-05	1.99E-03
cg23947326	CCDC61	19	Body	Island	21.17	4.44	25.39	6.09	-4.22	1.02E-04	2.84E-03
cg20060598		4	Intergenic	N_Shore	43.38	5.42	47.60	6.13	-4.22	3.17E-03	3.89E-02
cg26174329		7	Intergenic	N_Shelf	53.60	3.67	57.82	3.31	-4.22	3.58E-07	1.70E-05
cg02661464	LATS1	6	5'UTR	Open sea	74.03	5.91	78.25	5.40	-4.22	1.30E-03	2.08E-02
cg04994708		10	Intergenic	Open sea	76.29	4.83	80.51	4.11	-4.22	1.21E-04	3.27E-03
cg24057218	RAD51L1;RAD51L1;RAD51L1	14	Body;Body;Body	Open sea	61.01	4.80	65.24	4.85	-4.22	2.45E-04	5.80E-03
cg00511334	NRN1L	16	1stExon	Island	46.91	4.51	51.13	5.41	-4.22	1.37E-03	2.16E-02
cg05606082		2	Intergenic	S_Shelf	57.25	5.43	61.47	4.84	-4.22	3.04E-04	6.88E-03

cg24869834	SLC7A11	4	Body	Open sea	66.50	6.34	70.72	6.53	-4.22	5.07E-04	1.02E-02
cg18022012	HPCAL1;HPCAL1	2	5'UTR;5'UTR	S_Shore	13.38	3.23	17.60	5.09	-4.22	5.29E-05	1.63E-03
cg18887458		7	Intergenic	Open sea	63.89	5.90	68.11	5.58	-4.22	4.54E-05	1.43E-03
cg07166654		12	Intergenic	Island	18.24	4.03	22.46	4.97	-4.22	3.31E-06	1.37E-04
cg12020179	MMP20	11	TSS200	Open sea	75.07	4.88	79.29	4.01	-4.22	1.58E-04	4.09E-03
cg25610515	LUZP1;LUZP1	1	TSS1500;TSS1500	Open sea	36.04	6.29	40.27	6.66	-4.22	2.00E-03	2.83E-02
cg24321297	ST6GALNAC2	17	Body	N_Shelf	70.37	4.95	74.59	5.35	-4.22	5.84E-04	1.14E-02
cg20831413	ARAP1	11	5'UTR	N_Shelf	34.38	5.38	38.60	6.24	-4.22	3.02E-05	1.00E-03
cg03597347	ZSCAN12L1	6	TSS200	Open sea	19.84	2.86	24.06	4.69	-4.22	1.36E-06	6.00E-05
cg16970232	APC;APC;APC	5	TSS200;TSS200;5'UTR	Open sea	34.30	9.22	38.52	10.65	-4.22	2.21E-03	3.03E-02
cg27303733	EIF3F	11	TSS1500	N_Shore	57.51	4.51	61.73	5.70	-4.22	7.39E-04	1.37E-02
cg04320316		17	Intergenic	Open sea	43.75	5.19	47.97	5.70	-4.22	1.54E-03	2.35E-02
cg08404225	IL5RA;IL5RA;IL5RA;IL5RA;IL5RA;IL5RA	3	5'UTR;5'UTR;1stExon;5'UTR;1stExon;1stEx	Open sea	20.50	4.53	24.72	5.46	-4.22	5.11E-04	1.03E-02
cg17566560	DLGAP4;DLGAP4;DLGAP4	20	Body;5'UTR;Body	Open sea	71.10	5.45	75.32	5.13	-4.22	1.84E-05	6.47E-04
cg02509204	PIGT	20	TSS1500	N_Shore	75.74	5.54	79.96	5.23	-4.22	7.68E-04	1.41E-02
cg22910862		8	Intergenic	Open sea	55.10	5.99	59.32	6.15	-4.22	8.20E-05	2.37E-03
cg20668952	CSK;CSK	15	5'UTR;5'UTR	Open sea	34.49	6.16	38.71	5.55	-4.22	8.38E-05	2.41E-03
cg04419883		16	Intergenic	Open sea	47.61	5.30	51.83	4.33	-4.22	2.29E-04	5.51E-03
cg27639268	CUX2	12	Body	N_Shore	17.95	6.06	22.16	7.80	-4.22	1.51E-03	2.31E-02
cg11827082	ARL11	13	TSS200	Open sea	46.83	4.52	51.04	5.34	-4.22	5.04E-06	2.01E-04
cg00282953	ARL10	5	Body	S_Shore	77.98	4.60	82.19	3.46	-4.22	1.59E-05	5.70E-04
cg07729453	COL23A1	5	Body	Open sea	78.21	5.20	82.43	5.04	-4.22	3.66E-04	7.96E-03
cg13907255		5	Intergenic	Open sea	42.70	5.17	46.92	6.93	-4.22	4.32E-03	4.81E-02
cg02534744	CFD	19	Body	Island	27.92	5.64	32.14	7.09	-4.22	4.28E-03	4.78E-02
cg14465207	PDIA6	2	Body	N_Shore	52.75	5.86	56.97	4.68	-4.22	2.84E-04	6.51E-03
cg18633340		19	Intergenic	S_Shelf	59.55	4.48	63.76	3.96	-4.22	1.04E-06	4.65E-05
cg08202720	PER2	2	5'UTR	N_Shore	39.59	4.08	43.81	5.76	-4.22	9.32E-04	1.63E-02
cg05467716	LOC146880	17	Body	N_Shore	29.51	4.62	33.73	6.54	-4.22	2.57E-03	3.37E-02
cg24025508	EXOC2	6	Body	Open sea	76.22	5.55	80.43	5.13	-4.22	1.59E-03	2.40E-02
cg24939838	PRDM16;PRDM16	1	Body;Body	Island	7.16	3.13	11.38	5.94	-4.22	2.21E-05	7.64E-04
cg01833196	SH3PXD2A	10	Body	Island	52.41	6.45	56.62	7.02	-4.22	2.24E-04	5.42E-03
cg12595306		15	Intergenic	Open sea	65.96	5.58	70.17	7.20	-4.22	1.42E-03	2.22E-02
cg07209624	PRPF18	10	TSS1500	N_Shore	45.71	5.52	49.93	6.26	-4.22	3.95E-03	4.53E-02
cg11543592		12	Intergenic	N_Shore	47.92	4.84	52.14	5.74	-4.22	1.50E-04	3.91E-03
cg12264934	OPCML;OPCML	11	Body;Body	Open sea	70.72	4.03	74.93	3.51	-4.21	9.52E-06	3.60E-04
cg10081811		12	Intergenic	Open sea	68.58	5.30	72.79	5.14	-4.21	4.94E-04	1.00E-02
cg08164326	CFD	19	3'UTR	S_Shore	38.45	5.19	42.66	6.02	-4.21	2.53E-03	3.33E-02
cg19821713		15	Intergenic	Island	52.16	4.58	56.38	5.49	-4.21	2.32E-04	5.57E-03
cg24804144		15	Intergenic	Open sea	45.90	5.50	50.12	5.39	-4.21	2.91E-04	6.64E-03
cg12592387		12	Intergenic	Open sea	68.75	4.76	72.97	4.93	-4.21	1.27E-04	3.41E-03
cg15988887	PPP1CC	12	TSS1500	S_Shore	43.40	6.24	47.61	6.16	-4.21	3.85E-04	8.27E-03
cg00300822	ALDOA;ALDOA;ALDOA	16	TSS200;TSS1500;5'UTR	N_Shore	11.24	3.69	15.46	6.04	-4.21	7.13E-04	1.33E-02
cg04863197	MYADML2	17	3'UTR	N_Shore	82.48	5.10	86.69	3.16	-4.21	1.49E-05	5.40E-04
cg24654674		7	Intergenic	Open sea	61.98	6.01	66.19	5.35	-4.21	7.19E-04	1.34E-02
cg12120205		3	Intergenic	Open sea	77.67	4.43	81.88	4.67	-4.21	8.64E-05	2.48E-03
cg13877631	HTT	4	Body	Open sea	51.12	5.13	55.33	4.54	-4.21	3.61E-05	1.17E-03
cg00264799	IGF1;IGF1;IGF1;IGF1	12	Body;Body;Body;Body	Open sea	34.40	5.22	38.61	6.06	-4.21	3.42E-03	4.10E-02
cg04629133	PLAGL2	20	5'UTR	N_Shelf	77.02	4.37	81.24	3.81	-4.21	2.39E-05	8.19E-04
cg12371147	PCSK6;PCSK6;PCSK6;PCSK6;PCSK6;PCS	15	Body;Body;Body;Body;Body;Body;Body;Bo	Open sea	37.67	5.30	41.88	5.83	-4.21	8.95E-04	1.58E-02
cg00901574	POFUT1;POFUT1	20	Body;3'UTR	Open sea	40.88	5.51	45.10	5.51	-4.21	2.79E-03	3.56E-02
cg05872255		14	Intergenic	Open sea	75.74	3.83	79.95	3.81	-4.21	7.64E-06	2.95E-04
cg08081323	RTN4RL2	11	Body	Island	63.02	4.61	67.23	4.65	-4.21	1.74E-04	4.43E-03
cg15397593	CACNA1C;CACNA1C;CACNA1C;CACNA	12	Body;Body;Body;Body;Body;Body;Body;Bo	Open sea	61.47	7.15	65.68	5.99	-4.21	3.10E-03	3.83E-02

cg27213925	PREPL;PREPL;PREPL;PREPL;PREPL;PRE	2	Body;Body;Body;Body;Body;Body;Body	Open sea	83.67	5.66	87.88	4.53	-4.21	1.18E-04	3.20E-03
cg05687091	NFATC1;NFATC1;NFATC1;NFATC1;NF/	18	Body;Body;Body;Body;Body	Island	46.39	6.32	50.60	5.30	-4.21	1.76E-03	2.59E-02
cg26026416	NFIC;NFIC	19	Body;Body	Island	40.23	4.01	44.45	5.41	-4.21	3.93E-05	1.26E-03
cg13632983		11	Intergenic	S_Shelf	46.15	5.01	50.36	5.72	-4.21	1.18E-04	3.20E-03
cg13283952		2	Intergenic	Island	37.74	5.92	41.95	5.50	-4.21	2.97E-03	3.71E-02
cg04085768	ALPK1;ALPK1	4	Body;Body	Island	67.07	4.79	71.28	4.66	-4.21	1.10E-04	3.03E-03
cg09868780	LVRN	5	Body	Island	16.71	3.89	20.92	6.61	-4.21	1.01E-03	1.73E-02
cg02018277	PIF1	15	Body	Island	28.58	6.16	32.79	4.91	-4.21	7.08E-04	1.32E-02
cg10248878	GSX2	4	TSS1500	N_Shore	46.52	5.47	50.73	5.11	-4.21	1.97E-05	6.88E-04
cg06747330	NAPSA	19	Body	Island	35.65	6.21	39.86	7.71	-4.21	1.51E-03	2.32E-02
cg03311353		7	Intergenic	Open sea	69.74	5.77	73.95	4.53	-4.21	7.96E-04	1.44E-02
cg07284801	CYFIP2;CYFIP2;CYFIP2	5	Body;Body;Body	Open sea	73.28	4.96	77.49	4.83	-4.21	9.10E-05	2.59E-03
cg23511118		3	Intergenic	Open sea	27.81	4.89	32.02	6.20	-4.21	1.81E-03	2.64E-02
cg11009362	KSR2	12	Body	Open sea	43.15	4.07	47.35	5.68	-4.21	4.59E-05	1.44E-03
cg26222247	ANPEP	15	5'UTR	Island	14.65	4.25	18.86	6.11	-4.21	6.16E-04	1.19E-02
cg06985721	BSND;BSND	1	1stExon;5'UTR	S_Shore	73.71	6.68	77.92	5.25	-4.21	2.24E-03	3.06E-02
cg22607266		1	Intergenic	Open sea	72.20	4.99	76.41	4.69	-4.21	2.96E-04	6.73E-03
cg20864214	ARHGEF17	11	Body	Island	26.10	3.94	30.31	4.42	-4.21	1.10E-04	3.03E-03
cg13530528		9	Intergenic	N_Shelf	74.53	4.75	78.73	3.91	-4.21	4.16E-05	1.33E-03
cg01281231		13	Intergenic	Open sea	29.43	5.42	33.64	6.22	-4.21	1.86E-03	2.68E-02
cg19471433	ADAP1	7	Body	S_Shore	49.38	5.39	53.59	6.07	-4.21	2.68E-03	3.47E-02
cg09178384	SH3RF1	4	TSS1500	S_Shore	67.06	5.65	71.27	4.89	-4.21	1.27E-04	3.41E-03
cg08458912	MAP7D1	1	Body	Island	45.62	3.38	49.82	5.35	-4.21	9.47E-05	2.68E-03
cg19894253	ZHX1;ZHX1	8	3'UTR;3'UTR	Open sea	81.25	5.21	85.45	4.13	-4.21	5.41E-05	1.66E-03
cg04277056	S100B	21	Body	S_Shelf	68.38	4.55	72.58	3.91	-4.21	3.66E-05	1.19E-03
cg25407876	DNAJC9	10	Body	N_Shore	65.55	5.62	69.76	5.41	-4.21	8.02E-04	1.45E-02
cg07595602		1	Intergenic	Open sea	37.93	6.53	42.13	6.92	-4.21	2.06E-03	2.89E-02
cg13388615		2	Intergenic	Island	71.93	5.36	76.13	5.83	-4.20	1.08E-03	1.82E-02
cg12589566		5	Intergenic	N_Shelf	68.16	5.67	72.37	5.50	-4.20	6.62E-04	1.26E-02
cg04254389		19	Intergenic	N_Shelf	49.68	4.89	53.88	4.79	-4.20	1.27E-04	3.40E-03
cg01941586	PPIE;PPIE;PPIE	1	TSS1500;TSS1500;TSS1500	N_Shore	75.31	4.10	79.51	3.19	-4.20	8.80E-06	3.36E-04
cg14483431	ZDHHC19	3	Body	Island	35.44	3.62	39.64	4.17	-4.20	4.68E-06	1.88E-04
cg22240717		21	Intergenic	Island	78.62	5.75	82.83	4.61	-4.20	2.48E-04	5.86E-03
cg03678274	LYST;MIR1537	1	5'UTR;TSS1500	Open sea	69.12	5.50	73.33	4.36	-4.20	8.80E-04	1.56E-02
cg15923936		6	Intergenic	N_Shelf	38.28	4.89	42.48	4.76	-4.20	2.68E-04	6.22E-03
cg26678920	PRKCDBP	11	TSS200	Island	22.96	4.16	27.16	5.88	-4.20	9.12E-05	2.59E-03
cg20038070	STX18	4	Body	Open sea	46.65	4.78	50.85	4.62	-4.20	3.74E-05	1.21E-03
cg26510723	PTPRN2;PTPRN2;PTPRN2	7	Body;Body;Body	Open sea	52.24	5.73	56.44	6.34	-4.20	3.20E-03	3.91E-02
cg19961013	KIAA1324L;KIAA1324L;KIAA1324L	7	Body;Body;Body	Open sea	56.50	5.44	60.70	6.46	-4.20	3.60E-03	4.25E-02
cg03027800	DNAH2	17	Body	Open sea	63.08	5.82	67.28	5.70	-4.20	1.01E-03	1.73E-02
cg19497082		2	Intergenic	Open sea	28.97	5.17	33.17	6.63	-4.20	4.33E-03	4.82E-02
cg25336874		11	Intergenic	N_Shore	30.04	4.12	34.24	6.54	-4.20	9.55E-04	1.66E-02
cg07871971	CNTNAP1	17	Body	Island	55.31	5.33	59.51	6.33	-4.20	4.18E-03	4.70E-02
cg11887532	PKHD1;PKHD1	6	Body;Body	Open sea	62.03	5.56	66.23	5.03	-4.20	2.71E-04	6.29E-03
cg20654074	DLL4	15	Body	Island	34.92	5.09	39.12	4.50	-4.20	9.08E-05	2.58E-03
cg02051239	BOLA3;BOLA3	2	TSS1500;TSS1500	S_Shore	35.91	4.57	40.11	5.22	-4.20	2.23E-06	9.49E-05
cg23051248		8	Intergenic	S_Shore	63.58	3.76	67.78	5.62	-4.20	3.53E-04	7.73E-03
cg15618758	ARID1B;ARID1B;ARID1B	6	Body;Body;Body	Open sea	69.74	5.95	73.94	5.62	-4.20	2.69E-03	3.48E-02
cg11812143	CEP350	1	5'UTR	S_Shelf	45.35	3.93	49.55	4.16	-4.20	3.08E-05	1.02E-03
cg08968184	ZDHHC1	16	Body	S_Shore	48.70	3.95	52.90	4.97	-4.20	7.86E-05	2.29E-03
cg05910850	FLYWCH2;FLYWCH2;FLYWCH2	16	5'UTR;5'UTR;5'UTR	Island	51.94	6.04	56.14	5.47	-4.20	1.97E-03	2.79E-02
cg18550003	FGF21;FUT1	19	TSS200;TSS1500	S_Shelf	42.60	4.95	46.80	4.81	-4.20	4.99E-04	1.01E-02
cg20246907		13	Intergenic	Open sea	82.58	4.70	86.78	4.09	-4.20	3.98E-05	1.27E-03

cg26256263	RUNX3;RUNX3	1	TSS1500;Body	Island	20.63	3.33	24.83	3.73	-4.20	9.92E-07	4.45E-05
cg01994750		11	Intergenic	Open sea	73.30	5.79	77.50	5.14	-4.20	9.57E-05	2.70E-03
cg11199046		12	Intergenic	Island	20.18	4.48	24.38	4.71	-4.20	3.14E-06	1.30E-04
cg10123421	ZNF833	19	Body	Island	56.08	5.92	60.27	6.00	-4.20	3.65E-03	4.29E-02
cg24469464	MSX1	4	TSS1500	Island	34.86	5.45	39.06	5.60	-4.20	5.80E-04	1.14E-02
cg06085204	XAF1;XAF1;XAF1;XAF1	17	5'UTR;5'UTR;1stExon;1stExon	Open sea	10.15	4.52	14.35	6.51	-4.20	1.60E-03	2.41E-02
cg14597930		13	Intergenic	Open sea	62.95	4.78	67.15	5.83	-4.20	5.26E-04	1.05E-02
cg20929551	C15orf33	15	Body	Open sea	80.71	4.79	84.91	4.45	-4.20	1.21E-05	4.48E-04
cg01050010	MYO1D	17	Body	Island	15.46	5.08	19.65	7.01	-4.20	1.61E-03	2.42E-02
cg02560744	MALL	2	Body	N_Shore	76.84	6.67	81.04	7.14	-4.20	3.03E-03	3.77E-02
cg26947583	KIAA0247	14	5'UTR	Open sea	75.00	3.91	79.19	3.05	-4.20	1.00E-06	4.49E-05
cg12163845	HAPLN4	19	TSS200	S_Shore	29.53	3.68	33.73	4.90	-4.20	5.35E-06	2.13E-04
cg06438517		1	Intergenic	Island	22.53	5.40	26.73	5.18	-4.20	1.66E-03	2.47E-02
cg27228976		2	Intergenic	Island	63.56	5.85	67.76	5.53	-4.20	6.77E-04	1.28E-02
cg12814335	PITPNA	17	TSS1500	S_Shore	45.68	4.86	49.88	5.92	-4.20	2.12E-03	2.94E-02
cg13160058	BNIP3L	8	Body	S_Shelf	35.23	4.73	39.43	4.64	-4.20	7.80E-05	2.27E-03
cg23412630	RGL2;RGL2	6	Body;Body	N_Shore	68.30	4.33	72.49	3.86	-4.19	1.92E-05	6.76E-04
cg03034070	COL5A2	2	TSS1500	Open sea	53.27	6.02	57.47	5.16	-4.19	1.39E-03	2.18E-02
cg00469771		17	Intergenic	Island	53.63	6.39	57.82	6.45	-4.19	3.21E-03	3.93E-02
cg06871074		1	Intergenic	Island	23.52	5.50	27.71	5.85	-4.19	3.34E-05	1.10E-03
cg24208415		4	Intergenic	S_Shelf	39.18	5.87	43.38	6.58	-4.19	2.67E-03	3.45E-02
cg03214385	PTGFRN	1	Body	Open sea	83.76	5.41	87.95	4.67	-4.19	4.32E-04	9.05E-03
cg03140889	SULF1;SULF1;SULF1;SULF1	8	Body;Body;Body;Body	Open sea	72.83	4.91	77.02	5.10	-4.19	1.12E-03	1.86E-02
cg12657970	ZNF346	5	Body	S_Shelf	82.21	6.08	86.40	5.20	-4.19	2.44E-04	5.78E-03
cg18677148	CLTC	17	Body	Open sea	45.30	6.09	49.49	6.88	-4.19	2.73E-05	9.18E-04
cg04125370	LARP1	5	Body	Open sea	40.02	4.64	44.22	5.69	-4.19	8.06E-05	2.34E-03
cg07197290		10	Intergenic	Island	51.62	6.43	55.81	4.56	-4.19	1.42E-03	2.21E-02
cg02143404		16	Intergenic	Open sea	50.44	5.18	54.63	5.04	-4.19	6.34E-04	1.22E-02
cg01215762	HOXA10;HOXA10	7	TSS200;Body	Island	23.00	4.66	27.19	5.53	-4.19	2.03E-04	5.00E-03
cg15358549	FBN2	5	Body	Island	27.03	4.69	31.23	4.76	-4.19	8.97E-05	2.56E-03
cg04814352	MBNL2;MBNL2	13	5'UTR;5'UTR	Open sea	37.06	3.66	41.25	4.48	-4.19	2.45E-05	8.37E-04
cg14746387		5	Intergenic	Open sea	26.81	4.81	31.00	4.77	-4.19	3.34E-04	7.41E-03
cg15491950		3	Intergenic	Open sea	38.45	4.88	42.65	4.19	-4.19	7.31E-05	2.15E-03
cg00985960		18	Intergenic	N_Shelf	73.31	5.79	77.50	5.30	-4.19	8.53E-04	1.52E-02
cg11311053	NCOR2;NCOR2	12	Body;Body	S_Shore	51.81	4.54	56.00	3.76	-4.19	3.43E-05	1.12E-03
cg00515437		15	Intergenic	S_Shelf	81.20	5.70	85.39	3.67	-4.19	2.00E-04	4.94E-03
cg14383815	MCPH1	8	Body	Open sea	40.50	4.83	44.69	5.35	-4.19	1.32E-03	2.10E-02
cg26931208	IZUMO1;RASIP1	19	Body;TSS1500	S_Shore	70.22	4.78	74.41	3.41	-4.19	2.02E-05	7.06E-04
cg18175808		1	Intergenic	Island	16.36	2.49	20.55	3.24	-4.19	5.55E-11	3.23E-09
cg09968461	SLC8A1;SLC8A1;SLC8A1;SLC8A1	2	Body;Body;Body;Body	Open sea	55.73	6.27	59.92	4.77	-4.19	2.48E-03	3.29E-02
cg02147797	PRICKLE2	3	TSS200	Open sea	39.76	6.69	43.95	7.92	-4.19	2.29E-04	5.51E-03
cg09721612		8	Intergenic	Open sea	67.13	5.14	71.32	5.20	-4.19	3.65E-04	7.94E-03
cg13179087		17	Intergenic	Open sea	85.91	4.61	90.10	3.32	-4.19	1.13E-05	4.20E-04
cg01277890	LOC389333	5	TSS1500	S_Shore	64.26	4.16	68.45	4.22	-4.19	2.02E-05	7.05E-04
cg17663406	GRID1	10	Body	Island	29.02	6.43	33.21	6.48	-4.19	4.54E-03	4.97E-02
cg13095737	B4GALT7	5	Body	N_Shore	61.84	4.11	66.03	3.95	-4.19	3.24E-05	1.07E-03
cg27087243	SLC17A4	6	Body	Open sea	62.66	4.60	66.84	4.76	-4.19	1.03E-04	2.88E-03
cg18762806		8	Intergenic	N_Shore	50.62	6.06	54.80	5.61	-4.19	1.68E-03	2.50E-02
cg08835915	ZSCAN5B	19	3'UTR	Open sea	65.19	5.49	69.38	5.26	-4.19	3.80E-03	4.40E-02
cg21266798	IRS2	13	Body	N_Shore	53.26	4.71	57.44	5.79	-4.19	4.43E-04	9.23E-03
cg14251005	KLC4;KLC4;KLC4;KLC4	6	Body;Body;Body;Body	S_Shore	40.94	3.95	45.12	5.20	-4.19	1.69E-04	4.32E-03
cg16267920	ABLIM2;ABLIM2;ABLIM2;ABLIM2;ABL	4	Body;Body;Body;Body;Body;Body;Body	N_Shelf	65.77	6.64	69.96	5.99	-4.19	9.05E-04	1.59E-02
cg22562363	TACC2;TACC2	10	TSS200;TSS200	Open sea	69.96	3.88	74.15	2.87	-4.19	1.82E-06	7.86E-05

cg26262482	CACHD1	1	Body	S_Shore	36.13	6.23	40.32	6.88	-4.19	2.76E-04	6.38E-03
cg17176558		8	Intergenic	Island	75.58	5.13	79.76	4.60	-4.19	1.07E-04	2.95E-03
cg16004501		18	Intergenic	Open sea	79.71	5.51	83.90	5.75	-4.19	5.82E-04	1.14E-02
cg24445165	RASAL1	12	Body	N_Shore	70.38	5.05	74.57	5.41	-4.19	1.02E-03	1.74E-02
cg08896939	RNF135;RNF135	17	TSS1500;TSS1500	N_Shore	69.52	6.06	73.71	5.12	-4.19	2.34E-03	3.16E-02
cg13252730		14	Intergenic	Open sea	64.78	5.19	68.97	4.51	-4.19	1.37E-04	3.63E-03
cg06226703	SPATS2L;SPATS2L;SPATS2L;SPATS2L	2	5'UTR;5'UTR;5'UTR;5'UTR	Open sea	65.81	5.19	69.99	5.03	-4.19	9.72E-04	1.68E-02
cg14052235	ELANE	19	Body	Island	10.88	3.41	15.07	4.77	-4.19	2.46E-05	8.41E-04
cg00577488		8	Intergenic	Open sea	59.92	4.74	64.10	4.91	-4.19	4.81E-04	9.83E-03
cg23691894	ADD3;ADD3	10	1stExon;5'UTR	N_Shore	28.08	4.03	32.26	5.59	-4.18	5.60E-04	1.10E-02
cg12264626	CA3	8	TSS1500	N_Shore	32.24	6.08	36.43	6.45	-4.18	1.27E-03	2.05E-02
cg08169778		11	Intergenic	Open sea	26.55	4.66	30.73	5.27	-4.18	3.59E-04	7.84E-03
cg04838832	ADAMTS14;ADAMTS14	10	Body;Body	Open sea	17.35	4.67	21.53	5.73	-4.18	3.00E-06	1.25E-04
cg11869862		15	Intergenic	N_Shelf	69.80	5.87	73.98	5.17	-4.18	3.61E-04	7.86E-03
cg20889774	TOX2;TOX2;TOX2	20	5'UTR;Body;5'UTR	S_Shelf	35.87	5.19	40.06	4.16	-4.18	1.94E-04	4.83E-03
cg18499339	KLHL26	19	Body	Island	44.36	3.45	48.55	4.26	-4.18	4.68E-06	1.88E-04
cg19691410		14	Intergenic	N_Shelf	72.69	4.92	76.87	3.87	-4.18	3.04E-05	1.01E-03
cg09343421	SRRT;SRRT;SRRT;SRRT	7	Body;Body;Body;Body	N_Shore	72.96	4.17	77.15	3.14	-4.18	1.02E-06	4.56E-05
cg26807107	AQP8	16	Body	Open sea	88.22	5.21	92.40	1.46	-4.18	1.56E-06	6.80E-05
cg17859772	PRPH2;PRPH2	6	1stExon;5'UTR	Open sea	66.61	5.65	70.79	5.55	-4.18	5.56E-05	1.70E-03
cg12647970		6	Intergenic	S_Shelf	75.15	5.11	79.33	4.77	-4.18	3.90E-04	8.35E-03
cg13601472		9	Intergenic	Open sea	85.80	6.32	89.98	3.81	-4.18	3.58E-03	4.23E-02
cg03650429	FBXO17;FBXO17	19	Body;Body	Island	25.40	6.14	29.58	7.39	-4.18	6.07E-04	1.18E-02
cg06959021	TCHP;TCHP	12	Body;Body	Open sea	33.89	3.20	38.07	4.92	-4.18	4.10E-05	1.31E-03
cg14189782	EHBP1L1	11	TSS1500	Island	27.37	4.71	31.55	5.19	-4.18	2.66E-04	6.20E-03
cg24096855		19	Intergenic	Island	61.56	4.07	65.74	3.91	-4.18	2.09E-05	7.25E-04
cg02481956	SLC4A9	5	TSS1500	Open sea	67.57	4.14	71.75	4.75	-4.18	4.21E-05	1.34E-03
cg27276613	FCHSD2	11	Body	Open sea	61.54	3.70	65.72	3.50	-4.18	9.51E-08	4.75E-06
cg14363954	NOTCH1	9	Body	N_Shore	63.33	4.67	67.51	4.46	-4.18	3.79E-04	8.17E-03
cg07392601		9	Intergenic	Island	38.49	5.02	42.67	7.06	-4.18	8.62E-05	2.47E-03
cg13070650	STK3	8	Body	Open sea	78.66	5.95	82.84	4.43	-4.18	1.08E-04	2.99E-03
cg24645602	FAM49B	8	5'UTR	Open sea	73.56	4.52	77.74	4.36	-4.18	7.34E-05	2.16E-03
cg17662683	TNXB	6	Body	Island	33.47	4.72	37.65	5.16	-4.18	2.00E-04	4.94E-03
cg27506210	DEPDC5;DEPDC5;DEPDC5	22	5'UTR;TSS200;5'UTR	S_Shore	23.55	4.83	27.73	6.03	-4.18	2.11E-03	2.94E-02
cg04799329	RASA3	13	Body	S_Shore	72.49	5.60	76.67	5.78	-4.18	3.17E-04	7.11E-03
cg04944682		2	Intergenic	Island	61.86	6.50	66.04	6.14	-4.18	2.26E-03	3.08E-02
cg20592734	COL11A2;COL11A2;COL11A2	6	Body;Body;Body	Open sea	57.36	4.24	61.54	5.46	-4.18	2.79E-05	9.37E-04
cg14319773	PPAP2C;PPAP2C;PPAP2C	19	TSS1500;TSS1500;TSS1500	S_Shore	76.70	5.13	80.88	4.54	-4.18	4.16E-04	8.79E-03
cg17044356	TMEM88	17	TSS200	N_Shore	45.55	6.38	49.73	5.46	-4.18	2.25E-03	3.07E-02
cg01461862		10	Intergenic	S_Shore	45.47	5.61	49.65	4.64	-4.18	2.41E-04	5.74E-03
cg12140144	FLJ42875;PRDM16;FLJ42875;PRDM16	1	Body;TSS1500;Body;TSS1500	Island	12.70	3.66	16.88	5.84	-4.18	1.70E-04	4.34E-03
cg21208943	RABIF	1	TSS1500	S_Shore	70.30	5.54	74.48	5.03	-4.18	1.36E-03	2.15E-02
cg06270206	ANKRD11	16	5'UTR	N_Shore	82.95	4.44	87.12	3.95	-4.18	3.70E-05	1.20E-03
cg19163067	TFAP2E	1	Body	Island	43.94	4.84	48.12	5.72	-4.18	1.48E-03	2.28E-02
cg12561125	DTX3;GEFT	12	3'UTR;TSS1500	N_Shore	70.54	4.21	74.71	4.68	-4.18	1.52E-04	3.95E-03
cg14592468	NTF3;NTF3	12	TSS200;Body	N_Shore	68.59	5.14	72.76	4.62	-4.18	5.94E-07	2.75E-05
cg13568348	EML6	2	1stExon	Island	24.99	3.98	29.17	4.47	-4.18	6.41E-06	2.51E-04
cg27539233		14	Intergenic	Open sea	48.94	5.46	53.12	4.97	-4.18	4.29E-04	9.00E-03
cg12886580	ZNF513	2	Body	Island	77.91	4.85	82.08	4.25	-4.18	2.10E-04	5.12E-03
cg12001120	WNT10A	2	Body	S_Shelf	61.75	4.28	65.93	5.31	-4.18	1.97E-04	4.88E-03
cg05593162	SDCCAG8	1	Body	Open sea	77.89	5.33	82.06	2.52	-4.18	2.83E-05	9.48E-04
cg01603862		8	Intergenic	Open sea	60.72	5.48	64.89	5.47	-4.18	3.20E-04	7.15E-03
cg02364408	C19orf44	19	Body	N_Shelf	39.73	5.28	43.91	4.35	-4.17	4.30E-04	9.03E-03

cg08747557	MMP8	11	3'UTR	Open sea	50.77	6.27	54.94	5.76	-4.17	2.86E-03	3.62E-02
cg00367424	HCCA2	11	Body	Island	58.85	4.79	63.02	4.00	-4.17	1.15E-04	3.14E-03
cg05587756	CSDA;CSDA	12	Body;Body	N_Shore	28.29	5.35	32.46	6.05	-4.17	8.55E-05	2.46E-03
cg04317047	MIR143;LOC728264	5	TSS200;Body	Open sea	32.84	5.40	37.02	5.40	-4.17	4.02E-05	1.29E-03
cg19895047	CSGALNACT1;CSGALNACT1;CSGALNA(	8	TSS200;Body;5'UTR	S_Shore	35.75	5.49	39.92	5.16	-4.17	2.95E-03	3.70E-02
cg10056520	LOC100130872-SPON2	4	Body	Island	52.92	5.07	57.09	4.97	-4.17	4.67E-04	9.61E-03
cg10381113	SPATA13;SPATA13	13	Body;5'UTR	Open sea	54.38	3.72	58.55	4.20	-4.17	6.34E-05	1.91E-03
cg21887591	EDEM1	3	3'UTR	Open sea	24.02	3.69	28.19	4.71	-4.17	8.21E-05	2.37E-03
cg21808258		1	Intergenic	S_Shore	53.34	6.25	57.51	4.89	-4.17	2.54E-03	3.34E-02
cg02500691		17	Intergenic	N_Shelf	47.43	4.84	51.61	4.76	-4.17	1.01E-04	2.83E-03
cg02962630	DLL4	15	Body	Island	38.65	3.94	42.83	4.15	-4.17	7.54E-06	2.91E-04
cg21055528	LTB;LTB	6	Body;Body	S_Shore	40.51	4.40	44.68	4.71	-4.17	1.63E-06	7.08E-05
cg03443455	GAD1;GAD1	2	TSS1500;TSS1500	Island	25.45	4.89	29.62	4.55	-4.17	1.60E-04	4.14E-03
cg07118491	HCCA2	11	Body	Open sea	70.97	5.69	75.14	5.72	-4.17	1.65E-03	2.47E-02
cg12022630		10	Intergenic	Open sea	40.72	4.67	44.89	5.77	-4.17	6.79E-04	1.28E-02
cg13039454		7	Intergenic	Open sea	62.22	5.50	66.39	4.28	-4.17	3.21E-06	1.33E-04
cg20403557	LVRN	5	Body	Island	17.39	4.62	21.56	7.38	-4.17	3.08E-03	3.81E-02
cg04006266	BBS1	11	3'UTR	Open sea	44.74	4.94	48.91	4.71	-4.17	3.71E-05	1.20E-03
cg03500965	SCML4	6	TSS1500	Open sea	50.85	9.36	55.02	7.90	-4.17	7.11E-04	1.33E-02
cg04298811		1	Intergenic	S_Shelf	38.98	5.54	43.15	5.73	-4.17	1.96E-03	2.79E-02
cg24736217	C8orf41;C8orf41	8	1stExon;Body	N_Shore	12.01	4.65	16.18	6.20	-4.17	3.19E-04	7.15E-03
cg02334987	FBXL16	16	5'UTR	Island	17.05	4.65	21.22	4.60	-4.17	1.79E-04	4.53E-03
cg04264638	CLOCK	4	5'UTR	N_Shore	37.79	5.43	41.96	5.36	-4.17	9.68E-04	1.67E-02
cg06828015	NGEF;NGEF	2	Body;TSS1500	Island	47.89	6.53	52.06	6.51	-4.17	4.81E-04	9.83E-03
cg08652499	MITF;MITF;MITF	3	Body;Body;Body	Open sea	73.74	4.71	77.91	5.83	-4.17	2.98E-04	6.76E-03
cg06530563		12	Intergenic	N_Shelf	60.06	4.73	64.23	4.53	-4.17	1.97E-05	6.89E-04
cg25347526	LOC285419;LOC285419	4	Body;Body	Open sea	28.74	4.99	32.91	6.52	-4.17	2.29E-03	3.11E-02
cg09137301	C7orf29;C7orf29;LRRC61;LRRC61	7	5'UTR;1stExon;5'UTR;5'UTR	Open sea	32.96	4.55	37.13	5.38	-4.17	1.15E-03	1.90E-02
cg23392804	DNAH7	2	Body	N_Shelf	68.82	5.07	72.99	6.02	-4.17	1.87E-03	2.70E-02
cg07394719	C10orf90	10	Body	N_Shore	29.64	6.00	33.80	6.49	-4.17	4.49E-03	4.94E-02
cg18669388	SLIT3	5	TSS1500	S_Shore	38.10	5.17	42.27	4.78	-4.17	3.12E-04	7.02E-03
cg20560115		12	Intergenic	Open sea	81.41	4.77	85.58	4.44	-4.17	2.20E-04	5.33E-03
cg25371919		2	Intergenic	N_Shore	32.01	4.42	36.18	5.09	-4.17	5.01E-05	1.56E-03
cg08339189	GGTA1	9	Body	N_Shore	21.55	3.96	25.72	4.00	-4.17	5.58E-07	2.59E-05
cg06164683		3	Intergenic	Open sea	77.19	6.71	81.35	6.15	-4.17	8.09E-04	1.46E-02
cg09802893		7	Intergenic	S_Shore	64.39	4.66	68.56	5.21	-4.17	4.11E-04	8.71E-03
cg12315284	SH2B2	7	Body	Island	31.01	4.46	35.17	6.11	-4.17	3.08E-03	3.81E-02
cg16187919	ORC4L;ORC4L;ORC4L	2	Body;Body;Body	Open sea	59.95	4.83	64.12	4.39	-4.17	1.13E-04	3.10E-03
cg19155007	MAP6D1	3	TSS200	Island	42.60	6.19	46.76	7.75	-4.17	4.88E-04	9.94E-03
cg22059469		5	Intergenic	Open sea	34.16	4.39	38.33	5.25	-4.17	1.01E-04	2.82E-03
cg08679238	CCRL2;CCRL2;CCRL2	3	1stExon;5'UTR;5'UTR	Open sea	30.58	6.69	34.74	7.32	-4.16	1.43E-03	2.23E-02
cg04240553	TRADD	16	Body	Island	39.86	4.24	44.03	6.54	-4.16	9.54E-04	1.65E-02
cg19939969	LAMB3;LAMB3;LAMB3	1	Body;Body;Body	S_Shore	21.43	5.21	25.59	5.59	-4.16	1.94E-03	2.76E-02
cg26389955	PRKCA	17	Body	Open sea	77.72	5.38	81.89	4.65	-4.16	4.49E-05	1.42E-03
cg14754713		3	Intergenic	Open sea	54.98	3.62	59.14	5.51	-4.16	1.08E-04	2.98E-03
cg15741433	HIST1H1A	6	TSS200	N_Shelf	24.17	4.91	28.34	6.74	-4.16	4.28E-03	4.78E-02
cg14015502	C10orf26;C10orf26	10	TSS1500;Body	Open sea	31.84	5.07	36.00	5.57	-4.16	2.02E-03	2.85E-02
cg00498360	PRKCA	17	Body	Open sea	76.00	4.58	80.16	4.29	-4.16	3.32E-05	1.09E-03
cg27426442	SCUBE3	6	TSS1500	Island	52.56	3.72	56.72	4.95	-4.16	8.55E-05	2.46E-03
cg00165360	CNPY3	6	3'UTR	Open sea	78.34	5.72	82.50	5.05	-4.16	1.87E-03	2.70E-02
cg12838902	SLC29A4;SLC29A4;SLC29A4;SLC29A4	7	5'UTR;1stExon;1stExon;5'UTR	N_Shore	19.54	3.57	23.70	4.85	-4.16	3.90E-05	1.25E-03
cg15833534	FABP3	1	TSS200	Open sea	25.88	4.27	30.04	5.17	-4.16	1.10E-04	3.02E-03
cg27049654		1	Intergenic	Open sea	72.34	5.22	76.50	5.35	-4.16	4.44E-04	9.24E-03

cg22091053	P2RX3	11	TSS200	S_Shelf	51.90	5.39	56.06	4.45	-4.16	4.38E-04	9.15E-03
cg11123303	TAAR2	6	TSS1500	Open sea	63.27	7.24	67.43	7.52	-4.16	3.92E-03	4.50E-02
cg17651890	RIMS2	8	Body	Open sea	56.06	6.15	60.22	5.33	-4.16	1.12E-03	1.86E-02
cg15641657		12	Intergenic	Island	14.20	2.88	18.36	3.22	-4.16	6.00E-09	3.23E-07
cg19721764	PLXNA2	1	Body	Open sea	54.70	5.30	58.86	7.11	-4.16	3.98E-03	4.55E-02
cg11023328		15	Intergenic	Island	11.42	4.87	15.58	6.10	-4.16	1.35E-04	3.59E-03
cg03111404		6	Intergenic	S_Shore	22.09	5.16	26.25	4.86	-4.16	4.15E-04	8.77E-03
cg03499234		1	Intergenic	Island	27.71	4.75	31.87	5.44	-4.16	4.30E-05	1.36E-03
cg07016288	CYP2D6;CYP2D6	22	Body;Body	Island	46.98	4.34	51.14	4.23	-4.16	1.38E-04	3.66E-03
cg00975680	NUP133	1	Body	Open sea	45.26	5.24	49.42	5.52	-4.16	4.12E-03	4.65E-02
cg07461370		5	Intergenic	N_Shore	69.38	3.22	73.54	3.61	-4.16	1.74E-06	7.53E-05
cg22463553	PCDHGA4;PCDHGA9;PCDHGA1;PCDH(	5	Body;Body;Body;Body;1stExon;1stExon;Bo	N_Shore	54.83	6.41	58.99	5.39	-4.16	2.22E-03	3.04E-02
cg24906157	MED21	12	Body	S_Shelf	78.02	5.72	82.18	4.05	-4.16	5.79E-04	1.13E-02
cg25596287	SNHG3-RCC1;RCC1;SNHG3-RCC1;RCC:	1	Body;TSS1500;Body;TSS1500;5'UTR;TSS15	N_Shore	52.90	5.73	57.06	5.86	-4.16	1.60E-04	4.13E-03
cg00400448		15	Intergenic	N_Shore	28.69	4.99	32.85	4.89	-4.16	1.43E-03	2.23E-02
cg03051392	HYAL2;HYAL2;HYAL2	3	5'UTR;1stExon;TSS1500	S_Shore	35.92	3.32	40.08	3.67	-4.16	3.29E-06	1.36E-04
cg26802291	HAPLN4	19	Body	Island	13.08	4.43	17.23	5.82	-4.16	4.14E-05	1.32E-03
cg14116153		13	Intergenic	S_Shelf	36.28	7.73	40.43	7.20	-4.16	3.84E-03	4.44E-02
cg03312716	TRPV4;TRPV4	12	TSS1500;TSS1500	Island	45.21	5.63	49.37	5.59	-4.16	1.24E-03	2.01E-02
cg02307288	TRPC7;TRPC7;TRPC7	5	3'UTR;3'UTR;3'UTR	Open sea	74.72	5.19	78.88	4.26	-4.16	6.54E-05	1.96E-03
cg13172549	HOXA3;HOXA3;HOXA3	7	5'UTR;5'UTR;TSS200	Island	33.90	5.27	38.06	5.03	-4.16	2.55E-03	3.34E-02
cg09255851	RBBP6;RBBP6;RBBP6	16	Body;Body;Body	S_Shelf	38.80	6.46	42.96	6.19	-4.16	1.04E-03	1.76E-02
cg15166079		12	Intergenic	S_Shelf	64.04	3.24	68.19	4.21	-4.15	2.50E-06	1.05E-04
cg17220749	GALNT2	1	Body	Open sea	59.78	4.62	63.93	4.82	-4.15	4.48E-05	1.41E-03
cg07429623	KLHDC8A	1	TSS200	Open sea	38.50	5.00	42.65	6.30	-4.15	2.61E-03	3.40E-02
cg11637721	KAT5;KAT5;KAT5;RNASEH2C	11	3'UTR;3'UTR;3'UTR;3'UTR	N_Shore	27.56	4.12	31.71	5.05	-4.15	4.16E-04	8.78E-03
cg20449074		7	Intergenic	Open sea	42.06	4.80	46.21	5.67	-4.15	8.34E-05	2.40E-03
cg08885708		15	Intergenic	N_Shelf	51.25	3.99	55.41	4.21	-4.15	7.09E-06	2.75E-04
cg24366657	INS-IGF2;IGF2AS;IGF2;IGF2;IGF2AS;IG	11	Body;TSS1500;Body;5'UTR;TSS1500;TSS15	Island	48.76	5.06	52.91	6.23	-4.15	1.15E-04	3.14E-03
cg26346167	NEK11;NEK11	3	Body;Body	Open sea	60.58	5.88	64.73	5.98	-4.15	1.20E-03	1.96E-02
cg07317366	SCGB3A1	5	Body	Island	12.98	3.26	17.13	6.31	-4.15	1.42E-04	3.74E-03
cg11244402	FOXO1	13	Body	N_Shore	64.17	5.02	68.33	4.04	-4.15	7.26E-06	2.81E-04
cg00265277	MIR602	9	TSS1500	S_Shore	73.94	4.31	78.09	4.41	-4.15	4.24E-05	1.35E-03
cg22657059	PCDHB8	5	TSS200	N_Shore	34.67	6.50	38.83	6.50	-4.15	2.58E-03	3.38E-02
cg11410436	TOX3;TOX3	16	5'UTR;TSS1500	Island	20.93	6.08	25.08	7.01	-4.15	9.69E-04	1.67E-02
cg05060607	C3AR1	12	Body	Open sea	77.06	4.45	81.22	4.50	-4.15	3.22E-05	1.06E-03
cg09248429	LOC100133545	11	TSS200	N_Shelf	82.78	6.14	86.93	4.28	-4.15	2.40E-04	5.71E-03
cg13996426	AMIGO3;AMIGO3;RNF123	3	5'UTR;1stExon;Body	Island	21.01	5.35	25.16	5.68	-4.15	6.31E-04	1.21E-02
cg11643457	PTPRQ	12	Body	Open sea	65.70	5.73	69.85	5.88	-4.15	2.83E-04	6.50E-03
cg02540736	SLC25A29;MIR345	14	TSS1500;TSS200	S_Shore	27.85	4.25	32.00	5.03	-4.15	3.46E-04	7.60E-03
cg24382521	C7	5	TSS1500	Open sea	58.00	5.97	62.15	6.10	-4.15	1.45E-03	2.25E-02
cg10201141	ENO2;ATN1	12	3'UTR;TSS1500	Open sea	49.53	5.50	53.69	5.08	-4.15	4.09E-03	4.63E-02
cg00377332	SACM1L	3	Body	Open sea	77.50	4.40	81.65	4.23	-4.15	2.65E-05	8.95E-04
cg18832247	JARID2	6	Body	Open sea	51.72	6.67	55.87	5.70	-4.15	5.92E-04	1.15E-02
cg06898168	TNRC18	7	Body	Open sea	51.26	3.83	55.41	3.66	-4.15	3.61E-05	1.17E-03
cg15473411	OGFR	20	Body	S_Shore	73.51	4.67	77.66	4.92	-4.15	1.79E-04	4.53E-03
cg18124162	MYO16	13	Body	Open sea	57.71	3.02	61.86	3.78	-4.15	2.72E-07	1.31E-05
cg03461967	TP53I13	17	Body	S_Shore	37.78	6.09	41.93	5.55	-4.15	1.91E-03	2.74E-02
cg25265360		6	Intergenic	Open sea	32.40	3.97	36.55	4.30	-4.15	8.53E-05	2.45E-03
cg08783639	ANXA5	4	Body	Open sea	82.82	4.49	86.97	3.68	-4.15	4.92E-05	1.53E-03
cg26545245		12	Intergenic	Open sea	45.15	6.27	49.29	6.87	-4.15	3.09E-03	3.82E-02
cg08787251	EVX1	7	TSS200	N_Shore	26.12	4.09	30.26	4.83	-4.15	2.81E-05	9.41E-04
cg11763066		6	Intergenic	Open sea	41.19	6.62	45.34	6.97	-4.15	1.54E-03	2.34E-02

cg23182200	PTDSS1	8	3'UTR	Open sea	70.99	5.05	75.14	4.96	-4.15	4.37E-04	9.13E-03
cg15417365	SEPT4;SEPT4	17	Body;Body	Island	40.09	5.86	44.24	4.85	-4.15	4.98E-04	1.01E-02
cg02829845		4	Intergenic	Open sea	72.59	5.95	76.74	4.89	-4.15	1.32E-03	2.11E-02
cg05316864		4	Intergenic	Open sea	49.28	4.02	53.43	4.13	-4.15	1.50E-05	5.41E-04
cg22118944		13	Intergenic	Open sea	71.28	4.57	75.42	3.79	-4.15	7.72E-05	2.25E-03
cg26152983	MAGEF1;MAGEF1	3	1stExon;3'UTR	N_Shore	53.16	5.64	57.31	5.30	-4.15	1.60E-04	4.12E-03
cg16931076	FBXL14	12	Body	N_Shore	40.52	6.91	44.67	6.69	-4.15	5.88E-05	1.79E-03
cg04924185	MT1IP	16	Body	S_Shore	37.59	5.46	41.74	5.31	-4.15	2.21E-05	7.62E-04
cg14160480	FHAD1	1	Body	Open sea	31.96	4.51	36.11	4.76	-4.15	3.20E-04	7.16E-03
cg08927754	ANKRD11	16	Body	N_Shore	71.43	4.57	75.57	4.02	-4.15	1.65E-05	5.89E-04
cg05329578		9	Intergenic	N_Shore	30.99	4.44	35.13	6.22	-4.15	7.50E-04	1.38E-02
cg25954194	IL5	5	TSS200	Open sea	66.36	6.89	70.51	6.41	-4.15	1.93E-03	2.76E-02
cg25626012	PIPSL	10	Body	Open sea	32.07	3.28	36.21	3.76	-4.15	1.18E-06	5.26E-05
cg17857241		22	Intergenic	Island	14.30	3.31	18.45	4.83	-4.14	4.75E-05	1.49E-03
cg05397812	CALCA;CALCA;CALCA	11	TSS1500;TSS1500;TSS1500	N_Shore	25.42	4.03	29.57	4.70	-4.14	1.55E-07	7.62E-06
cg05004142	CCDC102A	16	Body	Island	23.59	3.13	27.74	4.96	-4.14	1.98E-05	6.92E-04
cg09583939	PCDHA1;PCDHA1;PCDHA1	5	1stExon;1stExon;1stExon	Island	42.14	4.85	46.29	5.01	-4.14	2.22E-04	5.38E-03
cg14897563	CHD3;CHD3;CHD3	17	Body;Body;Body	Open sea	77.41	4.45	81.55	4.11	-4.14	4.64E-05	1.46E-03
cg17344040	EHD1	11	Body	S_Shelf	61.13	6.89	65.27	6.17	-4.14	3.62E-03	4.26E-02
cg04572706		11	Intergenic	Open sea	82.68	4.09	86.82	3.29	-4.14	1.32E-06	5.84E-05
cg10076954		8	Intergenic	Open sea	75.65	5.10	79.79	3.46	-4.14	5.96E-05	1.81E-03
cg04155086	YTHDC1;YTHDC1	4	Body;Body	Open sea	56.67	5.51	60.81	5.17	-4.14	8.50E-04	1.52E-02
cg16273979		4	Intergenic	Open sea	24.49	4.84	28.64	5.86	-4.14	1.17E-03	1.93E-02
cg22385669	AQR	15	TSS1500	S_Shore	42.86	5.52	47.00	5.25	-4.14	1.05E-03	1.78E-02
cg21220462	SEMA4D;SEMA4D	9	Body;Body	Open sea	72.33	5.76	76.47	6.08	-4.14	1.48E-03	2.29E-02
cg07263570	C17orf104	17	Body	S_Shelf	63.78	5.96	67.92	5.89	-4.14	9.54E-05	2.69E-03
cg16893861		7	Intergenic	Open sea	65.03	3.64	69.17	3.15	-4.14	1.08E-07	5.39E-06
cg07839208	N4BP1	16	Body	Open sea	59.00	5.54	63.14	6.58	-4.14	9.78E-04	1.68E-02
cg04202892	ACOXL	2	3'UTR	Island	35.30	6.37	39.44	5.76	-4.14	7.82E-04	1.42E-02
cg24120800	TFG;TFG	3	Body;Body	Open sea	65.76	4.30	69.90	6.04	-4.14	1.57E-04	4.06E-03
cg26113809		12	Intergenic	Island	20.35	4.60	24.49	5.56	-4.14	2.95E-04	6.71E-03
cg04448487	GDAP1L1	20	TSS200	Island	28.63	5.09	32.77	5.55	-4.14	1.02E-04	2.85E-03
cg10757679		19	Intergenic	N_Shelf	63.54	4.06	67.68	5.35	-4.14	1.20E-04	3.25E-03
cg04193015	ACACA;ACACA;ACACA;ACACA;ACACA	17	Body;Body;Body;Body;Body	Open sea	48.19	6.76	52.33	6.24	-4.14	3.40E-03	4.09E-02
cg05033401	CAPN1	11	Body	Island	15.86	3.63	20.00	4.37	-4.14	1.20E-05	4.44E-04
cg11790673		1	Intergenic	Open sea	60.16	5.06	64.30	3.80	-4.14	4.34E-05	1.37E-03
cg00934987	SEPT4;SEPT4;SEPT4	17	Body;Body;Body	N_Shelf	54.03	6.78	58.17	5.30	-4.14	8.05E-04	1.45E-02
cg04540383	RASSF1;RASSF1;ZMYND10	3	TSS1500;TSS1500;3'UTR	S_Shore	53.05	5.40	57.19	4.71	-4.14	1.00E-04	2.80E-03
cg21995800	WDR77	1	Body	Open sea	71.22	4.08	75.36	3.64	-4.14	6.93E-06	2.69E-04
cg15147693		6	Intergenic	S_Shelf	82.82	4.65	86.96	4.51	-4.14	1.47E-04	3.85E-03
cg22209445	TCTEX1D2	3	Body	N_Shelf	65.30	4.65	69.44	4.78	-4.14	2.36E-05	8.09E-04
cg24502339	ME2;ME2	18	5'UTR;5'UTR	S_Shelf	74.00	6.24	78.14	5.20	-4.14	5.89E-04	1.15E-02
cg03718509		6	Intergenic	Open sea	50.98	5.16	55.12	4.81	-4.14	1.18E-04	3.21E-03
cg17845732		19	Intergenic	S_Shore	72.36	5.00	76.50	4.20	-4.14	5.26E-04	1.05E-02
cg03946762	ZNF385A;ZNF385A;ZNF385A;ZNF385A	12	5'UTR;1stExon;1stExon;5'UTR	Island	54.05	5.13	58.19	5.58	-4.14	4.25E-05	1.35E-03
cg23371436	NAV2;NAV2;NAV2;NAV2	11	Body;Body;Body;Body	Open sea	74.49	5.81	78.63	4.68	-4.14	1.16E-04	3.15E-03
cg18561676		1	Intergenic	Island	28.97	4.47	33.11	5.78	-4.14	1.48E-03	2.29E-02
cg15197657	PDLIM7;PDLIM7	5	Body;Body	N_Shelf	52.73	3.65	56.87	4.12	-4.14	1.14E-05	4.23E-04
cg14128708	INS-IGF2;INS-IGF2	11	Body;Body	N_Shore	79.74	5.17	83.87	4.01	-4.14	1.24E-03	2.01E-02
cg25526519	RCCD1;RCCD1	15	5'UTR;5'UTR	N_Shore	48.79	5.30	52.92	4.51	-4.14	9.60E-04	1.66E-02
cg02543993	RNF216;RNF216	7	Body;Body	Open sea	78.09	4.73	82.23	2.70	-4.14	4.19E-05	1.33E-03
cg01234420	LOC150381	22	Body	N_Shelf	31.25	4.94	35.38	5.92	-4.14	6.59E-04	1.25E-02
cg27227156	FAM55A	11	5'UTR	Open sea	68.96	5.94	73.09	6.65	-4.13	1.25E-03	2.02E-02

cg14299235	ABCA1	9	Body	Open sea	76.00	6.11	80.13	6.44	-4.13	1.74E-03	2.56E-02
cg03132166	FAM83F	22	TSS200	Island	16.94	4.60	21.07	6.18	-4.13	2.97E-05	9.89E-04
cg01650618	MPHOSPH8	13	Body	S_Shelf	65.83	4.42	69.96	4.15	-4.13	1.38E-04	3.66E-03
cg14561934	FAM171A1	10	Body	N_Shore	41.88	10.04	46.01	10.55	-4.13	2.90E-03	3.66E-02
cg07224067	RSAD1	17	TSS1500	N_Shore	56.69	4.58	60.82	4.16	-4.13	2.67E-04	6.21E-03
cg26280349		22	Intergenic	N_Shore	41.50	3.59	45.64	4.37	-4.13	9.56E-06	3.62E-04
cg15616511	CRY2;CRY2	11	Body;Body	Open sea	59.58	3.42	63.71	3.24	-4.13	7.90E-07	3.60E-05
cg13115683	HCG9	6	Body	N_Shore	17.74	6.08	21.87	6.44	-4.13	1.91E-03	2.74E-02
cg22356117	LAMP3	3	TSS1500	S_Shore	82.63	5.73	86.76	3.80	-4.13	7.56E-04	1.39E-02
cg24496475		4	Intergenic	Island	20.14	5.72	24.27	7.26	-4.13	4.19E-03	4.71E-02
cg27544046	VCAM1;VCAM1	1	Body;Body	Open sea	24.45	4.80	28.59	5.64	-4.13	1.27E-03	2.04E-02
cg12188410	LAMA4;LAMA4;LAMA4	6	Body;Body;Body	Open sea	69.83	5.32	73.96	4.60	-4.13	1.58E-04	4.09E-03
cg10982460	RASA3	13	Body	N_Shore	61.36	5.39	65.49	6.15	-4.13	6.64E-04	1.26E-02
cg01014615	HOXD3	2	TSS1500	Island	23.06	4.10	27.20	4.50	-4.13	7.77E-05	2.26E-03
cg14727777	C4orf49	4	TSS200	S_Shore	38.31	7.09	42.44	7.47	-4.13	2.37E-03	3.18E-02
cg04229487	APCDD1	18	Body	Island	62.23	4.72	66.36	5.14	-4.13	1.79E-04	4.53E-03
cg06798483	ST3GAL6	3	Body	Open sea	42.66	3.94	46.79	5.63	-4.13	7.76E-04	1.42E-02
cg02523679	SLC6A13	12	TSS1500	Open sea	59.07	4.70	63.20	4.20	-4.13	1.97E-05	6.89E-04
cg23327851	RNASE2;RNASE2	14	1stExon;5'UTR	Open sea	55.82	4.03	59.95	3.61	-4.13	5.08E-05	1.57E-03
cg11348249	AKAP12	6	TSS1500	Island	46.64	5.86	50.77	6.59	-4.13	4.06E-03	4.61E-02
cg14391855	RIN1	11	TSS200	S_Shore	33.50	6.42	37.63	5.58	-4.13	8.68E-04	1.54E-02
cg21204904	RASSF5;RASSF5;RASSF5	1	Body;TSS200;Body	Island	8.00	1.89	12.13	3.42	-4.13	8.15E-12	4.86E-10
cg16891075		6	Intergenic	Open sea	72.72	4.98	76.85	5.77	-4.13	1.79E-03	2.62E-02
cg13683301		10	Intergenic	Open sea	39.92	4.71	44.05	5.39	-4.13	8.79E-04	1.56E-02
cg10574494	SPATA18	4	Body	S_Shore	19.65	6.58	23.78	4.63	-4.13	7.93E-05	2.30E-03
cg21390363		17	Intergenic	S_Shelf	37.51	4.30	41.64	4.45	-4.13	5.39E-05	1.66E-03
cg18190861		12	Intergenic	S_Shelf	50.05	6.94	54.18	5.80	-4.13	2.05E-03	2.87E-02
cg23785612		6	Intergenic	Open sea	48.97	5.46	53.10	5.76	-4.13	4.04E-03	4.59E-02
cg22528172	PUM2	2	TSS1500	Open sea	80.86	4.76	84.99	3.47	-4.13	1.35E-05	4.92E-04
cg20849935		8	Intergenic	Open sea	71.04	8.26	75.17	7.55	-4.13	1.81E-03	2.64E-02
cg14150727	LGR6;LGR6;LGR6	1	TSS1500;Body;Body	N_Shore	52.62	6.13	56.75	5.87	-4.13	2.18E-03	3.00E-02
cg02019333	UPK1B	3	TSS1500	Open sea	63.24	6.52	67.37	7.32	-4.13	2.51E-03	3.32E-02
cg26391902	FAM174B	15	Body	Open sea	51.73	4.91	55.86	4.21	-4.13	9.56E-05	2.70E-03
cg14494620		15	Intergenic	Island	66.59	6.45	70.72	6.11	-4.13	9.81E-04	1.69E-02
cg01260810	HCG9	6	Body	N_Shore	72.92	6.34	77.04	4.98	-4.13	8.04E-04	1.45E-02
cg23055921		7	Intergenic	Open sea	66.32	4.89	70.45	5.50	-4.13	2.86E-04	6.56E-03
cg16751781	CHRNA5	15	Body	S_Shore	23.62	4.44	27.74	4.39	-4.13	9.38E-06	3.55E-04
cg01227537	ZIC1	3	TSS200	Island	20.65	4.62	24.78	5.91	-4.13	3.94E-04	8.41E-03
cg01844274	SYNE1;SYNE1	6	Body;Body	Open sea	83.55	4.61	87.68	3.30	-4.13	3.01E-05	1.00E-03
cg10725316	KCNIP2;KCNIP2;KCNIP2;KCNIP2;KCNIP	10	TSS200;TSS200;TSS200;TSS200;TSS200;TSS	S_Shore	5.65	2.05	9.77	5.53	-4.13	1.30E-06	5.73E-05
cg02886033		7	Intergenic	N_Shore	43.02	4.78	47.14	4.77	-4.13	4.06E-04	8.61E-03
cg22238209	MAG;MAG	19	Body;Body	Island	66.18	6.59	70.31	6.37	-4.13	4.53E-03	4.96E-02
cg23793213	RNF39;RNF39	6	Body;Body	Island	67.22	5.33	71.35	6.69	-4.13	4.51E-03	4.96E-02
cg10018862		14	Intergenic	Open sea	31.64	9.67	35.77	10.75	-4.13	3.68E-03	4.31E-02
cg18066206		22	Intergenic	S_Shore	39.46	4.95	43.59	5.81	-4.13	2.23E-03	3.05E-02
cg07540652		8	Intergenic	Island	40.57	4.71	44.70	4.88	-4.13	3.74E-05	1.21E-03
cg19897330	SCGB3A1	5	Body	Island	11.67	2.93	15.80	6.60	-4.13	1.84E-04	4.63E-03
cg14322157	GABBR2	9	Body	Open sea	33.19	4.71	37.31	5.78	-4.13	1.35E-03	2.14E-02
cg08972081	RBP1;RBP1;RBP1	3	TSS1500;TSS1500;TSS1500	S_Shore	47.86	8.34	51.99	7.06	-4.12	2.04E-03	2.87E-02
cg06122230	ZNFX1	20	Body	Open sea	60.21	6.75	64.34	5.28	-4.12	2.67E-03	3.45E-02
cg01881062	KLHL21	1	Body	N_Shore	32.14	5.79	36.26	6.49	-4.12	2.03E-03	2.86E-02
cg12111714	ATP8A2	13	Body	Island	31.57	5.61	35.70	4.25	-4.12	4.97E-04	1.01E-02
cg20647118	GPC5	13	Body	S_Shore	19.57	4.19	23.70	6.10	-4.12	7.41E-04	1.37E-02

cg25746489	TRIM26	6	5'UTR	Open sea	48.14	4.20	52.26	4.31	-4.12	6.78E-05	2.02E-03
cg22020299	MRPL14	6	5'UTR	Open sea	72.22	4.84	76.34	3.51	-4.12	9.01E-05	2.57E-03
cg24946253	PXDNL	8	TSS200	Open sea	68.96	4.72	73.08	4.96	-4.12	1.08E-04	2.99E-03
cg16559361	ZFPM1	16	Body	S_Shelf	52.42	4.49	56.55	5.63	-4.12	1.83E-03	2.66E-02
cg03437479	ZNF397OS;ZNF397OS	18	5'UTR;5'UTR	N_Shore	36.62	6.76	40.74	7.66	-4.12	3.40E-04	7.51E-03
cg06660522		1	Intergenic	Open sea	49.23	7.38	53.36	6.30	-4.12	3.37E-03	4.06E-02
cg24113496	MGC29506	5	TSS1500	N_Shore	49.10	4.58	53.23	5.36	-4.12	2.29E-05	7.89E-04
cg04058153	DLGAP2	8	5'UTR	N_Shore	68.22	6.32	72.35	5.39	-4.12	3.38E-03	4.07E-02
cg07953307	LAT;LAT;LAT;LAT	16	Body;Body;Body;Body	Open sea	78.07	2.82	82.20	2.42	-4.12	3.96E-10	2.24E-08
cg06004017	MN1	22	Body	N_Shore	77.98	5.08	82.10	3.52	-4.12	6.20E-05	1.87E-03
cg27508551	HOXA7	7	1stExon	Island	22.43	3.95	26.55	4.72	-4.12	6.63E-06	2.59E-04
cg21634218	FLJ26850	19	TSS1500	N_Shore	31.89	4.68	36.01	4.35	-4.12	4.43E-05	1.40E-03
cg14194983	NPPA	1	TSS1500	Open sea	56.08	5.93	60.20	6.61	-4.12	7.82E-04	1.42E-02
cg03786933	NQO1;NQO1;NQO1;NQO1;NQO1;NQ(	16	1stExon;5'UTR;1stExon;5'UTR;5'UTR;1stEx	Island	23.90	5.94	28.02	7.34	-4.12	3.13E-04	7.02E-03
cg06590662	TMCO7	16	Body	Open sea	46.61	4.96	50.73	5.80	-4.12	4.10E-03	4.64E-02
cg22232952	ANGPTL6	19	Body	Island	17.53	3.57	21.65	3.88	-4.12	1.85E-05	6.52E-04
cg07382752	TLX3	5	Body	Island	31.06	3.33	35.18	4.06	-4.12	1.12E-06	5.00E-05
cg08421051	LOC338799	12	Body	S_Shore	22.25	4.13	26.37	6.30	-4.12	2.80E-03	3.57E-02
cg12026095	FTL	19	TSS200	Island	40.43	4.33	44.55	5.18	-4.12	2.32E-04	5.56E-03
cg08448725	TOX3;TOX3	16	TSS1500;TSS1500	S_Shore	23.65	4.66	27.77	5.64	-4.12	7.81E-04	1.42E-02
cg11445634		15	Intergenic	Open sea	39.64	5.70	43.75	5.84	-4.12	4.43E-04	9.22E-03
cg22404498	RFPL2;RFPL2	22	TSS1500;TSS200	Open sea	28.63	6.92	32.75	7.19	-4.12	3.24E-03	3.95E-02
cg26669159	CAMTA1	1	Body	Open sea	32.89	5.15	37.00	4.72	-4.12	1.28E-03	2.05E-02
cg01933079	MAST4;MAST4	5	Body;Body	Open sea	74.65	6.22	78.76	4.92	-4.12	1.09E-03	1.83E-02
cg10979209	CNGA1	4	TSS1500	N_Shelf	77.74	3.54	81.85	4.25	-4.12	1.08E-06	4.83E-05
cg17644557	NT5E	6	TSS200	N_Shore	32.48	5.17	36.60	5.56	-4.12	8.07E-05	2.34E-03
cg19045437	PLXNA4	7	Body	Open sea	75.13	4.53	79.24	5.47	-4.12	6.61E-04	1.26E-02
cg04718145		1	Intergenic	Island	34.88	4.94	39.00	5.50	-4.12	1.66E-04	4.26E-03
cg10283371		19	Intergenic	Island	43.69	3.63	47.80	4.70	-4.12	1.61E-06	7.02E-05
cg26168651	NOS3	7	Body	Island	16.70	4.99	20.81	5.70	-4.12	6.51E-04	1.24E-02
cg06761203	09-Mar	12	TSS1500	N_Shore	33.38	4.16	37.49	4.08	-4.12	2.90E-05	9.67E-04
cg11263011	MIR195;MIR497	17	TSS1500;TSS1500	N_Shelf	71.67	4.36	75.79	4.38	-4.12	1.17E-05	4.34E-04
cg07260806		6	Intergenic	Open sea	72.53	5.44	76.64	5.15	-4.12	8.21E-05	2.37E-03
cg14796563	EFNA2	19	Body	Island	56.20	5.55	60.31	6.06	-4.12	2.08E-03	2.90E-02
cg26414564	FGF2	4	Body	Open sea	55.10	3.82	59.22	3.25	-4.12	5.38E-06	2.14E-04
cg02646423	HOXA5	7	TSS1500	Island	24.21	5.09	28.32	5.88	-4.12	4.44E-03	4.90E-02
cg22971501	LDLR	19	TSS1500	N_Shore	25.46	4.86	29.58	5.06	-4.12	6.93E-04	1.30E-02
cg15205428	TNFRSF25;TNFRSF25;TNFRSF25;TNFR!	1	Body;Body;Body;Body;Body;Body	Island	30.21	4.44	34.33	5.88	-4.11	9.31E-04	1.63E-02
cg26476173	LONP2	16	Body	Open sea	38.61	5.59	42.72	4.55	-4.11	1.30E-03	2.08E-02
cg19273101		2	Intergenic	Open sea	80.68	5.36	84.80	6.51	-4.11	1.97E-03	2.80E-02
cg11196848	FGF20	8	TSS200	S_Shore	46.46	6.09	50.58	5.58	-4.11	2.01E-03	2.84E-02
cg03387585	KIRREL3;KIRREL3	11	Body;Body	Open sea	56.93	5.07	61.04	3.95	-4.11	2.06E-04	5.06E-03
cg06985993	FAM57B	16	Body	N_Shelf	39.23	6.10	43.35	7.59	-4.11	5.37E-04	1.07E-02
cg01000657	ZNF254	19	Body	Open sea	35.26	8.10	39.38	9.14	-4.11	2.82E-03	3.59E-02
cg01311805	LDLRAD3	11	Body	Open sea	68.57	4.81	72.69	4.63	-4.11	2.64E-04	6.15E-03
cg08125539	IGF2BP1;IGF2BP1	17	Body;Body	S_Shelf	62.98	7.04	67.09	5.50	-4.11	3.51E-03	4.17E-02
cg00421173	MGST2	4	Body	Open sea	34.13	4.09	38.24	4.86	-4.11	2.51E-04	5.93E-03
cg12510724	SCNN1A;SCNN1A;SCNN1A	12	Body;TSS1500;5'UTR	Open sea	38.30	4.83	42.41	7.39	-4.11	1.44E-03	2.24E-02
cg04905829	CYP27B1;CYP27B1	12	5'UTR;1stExon	S_Shore	34.76	4.35	38.87	4.44	-4.11	5.55E-04	1.10E-02
cg08550353	SGK1;SGK1;SGK1	6	TSS1500;Body;Body	Island	20.45	3.96	24.56	5.11	-4.11	2.83E-04	6.50E-03
cg26562691	PRKCB;PRKCB	16	Body;Body	S_Shelf	46.70	5.67	50.81	6.12	-4.11	3.73E-05	1.20E-03
cg13398368		7	Intergenic	N_Shelf	67.08	4.47	71.19	4.18	-4.11	3.88E-05	1.25E-03
cg12122057	MICAL2	11	5'UTR	Island	42.63	4.56	46.75	6.18	-4.11	1.83E-03	2.66E-02

cg15341575	CPEB3	10	Body	Open sea	29.13	4.68	33.24	5.22	-4.11	1.22E-03	1.99E-02
cg13208704	SRCIN1	17	Body	N_Shore	37.87	4.22	41.98	4.63	-4.11	1.03E-05	3.89E-04
cg01247454	COL5A2	2	Body	Open sea	44.85	6.78	48.97	6.71	-4.11	4.37E-03	4.85E-02
cg13525276	TSHR;TSHR;TSHR	14	Body;Body;Body	Open sea	27.18	6.10	31.29	6.56	-4.11	2.56E-03	3.36E-02
cg16707262	TRIM26	6	Body	Open sea	75.37	4.59	79.48	4.35	-4.11	1.38E-05	5.03E-04
cg18385371	NFIC;NFIC	19	Body;Body	N_Shelf	47.32	4.61	51.43	4.78	-4.11	9.33E-04	1.63E-02
cg08305808		15	Intergenic	N_Shelf	67.79	4.75	71.90	3.68	-4.11	1.11E-04	3.06E-03
cg16503279	FHOD1	16	Body	N_Shelf	40.58	4.44	44.69	5.14	-4.11	3.94E-04	8.42E-03
cg03054277	OBSCN;OBSCN	1	Body;Body	Island	38.75	3.98	42.86	5.81	-4.11	1.57E-03	2.37E-02
cg08457620	RTN4RL1	17	Body	N_Shore	31.51	4.63	35.62	5.95	-4.11	1.63E-03	2.44E-02
cg20553982		14	Intergenic	Open sea	72.11	5.10	76.22	3.78	-4.11	1.03E-04	2.88E-03
cg04804726		10	Intergenic	Open sea	48.75	4.58	52.86	4.65	-4.11	5.87E-05	1.79E-03
cg12427163		1	Intergenic	Open sea	69.97	5.71	74.08	4.35	-4.11	5.43E-04	1.08E-02
cg09860601	VWA1;VWA1	1	Body;3'UTR	Island	70.36	5.04	74.47	2.96	-4.11	9.51E-05	2.69E-03
cg01801191	OSGIN1;OSGIN1;OSGIN1	16	Body;Body;Body	Open sea	63.82	5.22	67.93	5.21	-4.11	5.15E-04	1.04E-02
cg13494428	AKR7A2;PQLC2;PQLC2;PQLC2	1	TSS1500;5'UTR;5'UTR;5'UTR	S_Shore	30.65	3.83	34.76	4.67	-4.11	2.30E-04	5.53E-03
cg14149172	PI4KB	1	TSS200	N_Shore	24.64	4.55	28.75	4.33	-4.11	1.22E-05	4.49E-04
cg22816091		1	Intergenic	Island	49.82	6.23	53.93	5.87	-4.11	3.41E-03	4.09E-02
cg16911220	KRT86	12	1stExon	Island	74.83	5.80	78.94	4.59	-4.11	1.20E-03	1.96E-02
cg07074974	RBMS1;RBMS1	2	Body;Body	Open sea	71.38	6.69	75.49	4.72	-4.11	1.51E-03	2.32E-02
cg05270535		20	Intergenic	N_Shore	32.64	4.57	36.75	5.08	-4.11	6.10E-04	1.18E-02
cg08245317		11	Intergenic	N_Shore	44.80	5.47	48.91	5.83	-4.11	2.14E-03	2.97E-02
cg14248031		19	Intergenic	N_Shore	41.14	4.18	45.24	5.36	-4.11	4.56E-04	9.44E-03
cg13756879	INS-IGF2;IGF2AS;IGF2;IGF2;IGF2AS;IG	11	Body;TSS1500;Body;5'UTR;TSS1500;TSS15	Island	53.89	5.20	58.00	5.31	-4.11	5.33E-04	1.06E-02
cg07509364	FRG1B	20	TSS1500	N_Shore	78.75	4.47	82.86	4.43	-4.11	4.72E-05	1.48E-03
cg01559356	C19orf35	19	TSS1500	S_Shelf	31.66	6.97	35.76	7.51	-4.11	3.39E-03	4.08E-02
cg16371229	PCNXL2	1	Body	Open sea	32.16	6.40	36.27	5.62	-4.11	2.35E-03	3.16E-02
cg05214088	C10orf93	10	Body	S_Shelf	52.52	3.28	56.63	3.63	-4.11	3.50E-07	1.66E-05
cg15520962	TMEM190	19	TSS200	N_Shore	57.55	4.44	61.66	4.29	-4.11	4.30E-05	1.36E-03
cg04248332	SNORD103A;PUM1;PUM1	1	TSS1500;Body;Body	Open sea	71.46	4.64	75.57	4.09	-4.11	2.70E-04	6.27E-03
cg16133681	IFLTD1	12	TSS200	Open sea	45.38	6.59	49.49	5.49	-4.11	4.35E-03	4.83E-02
cg21651000	NCOA2	8	5'UTR	Open sea	46.71	5.48	50.82	5.69	-4.11	7.13E-04	1.33E-02
cg13581362	MGC23270	14	TSS1500	Open sea	25.39	3.73	29.49	5.09	-4.11	2.56E-04	6.01E-03
cg26195583		4	Intergenic	Island	31.66	4.73	35.77	5.58	-4.11	2.17E-05	7.53E-04
cg26747317	NEU1	6	Body	N_Shore	54.72	5.75	58.82	4.76	-4.11	1.74E-03	2.56E-02
cg18848446	RNASE9;RNASE9;RNASE9;RNASE9;RN/	14	TSS1500;TSS1500;TSS1500;TSS1500;TSS15	Open sea	52.16	5.91	56.27	6.49	-4.11	4.58E-04	9.46E-03
cg04093645	FBN2	5	Body	Island	32.89	4.93	36.99	6.18	-4.11	1.14E-03	1.89E-02
cg08432583		2	Intergenic	S_Shore	23.74	5.53	27.84	5.94	-4.10	5.45E-04	1.08E-02
cg24926924		2	Intergenic	Open sea	70.02	4.72	74.12	3.42	-4.10	3.88E-05	1.25E-03
cg20328588	FXYD6;FXYD6;FXYD6;FXYD6;FXYD6	11	5'UTR;TSS200;TSS200;TSS200;TSS200	S_Shore	22.78	4.29	26.88	6.19	-4.10	2.17E-03	2.99E-02
cg16869622	FBRSL1	12	Body	Island	77.26	5.46	81.37	3.50	-4.10	1.57E-04	4.06E-03
cg13624964	FLRT2	14	Body	Open sea	78.28	5.76	82.38	4.32	-4.10	1.74E-03	2.56E-02
cg20104126		12	Intergenic	S_Shelf	65.25	5.46	69.35	5.53	-4.10	1.14E-03	1.89E-02
cg10261735		8	Intergenic	Open sea	43.37	4.38	47.47	5.07	-4.10	4.34E-04	9.08E-03
cg22621310	MAD1L1;MAD1L1;MAD1L1	7	Body;Body;Body	Island	87.04	5.90	91.14	2.12	-4.10	2.80E-04	6.44E-03
cg19668951	SLC39A13;SLC39A13	11	5'UTR;5'UTR	S_Shore	35.28	6.73	39.38	6.94	-4.10	6.09E-05	1.84E-03
cg04483989	C4orf22	4	Body	Open sea	68.74	6.82	72.84	6.10	-4.10	1.23E-03	2.00E-02
cg23419756		13	Intergenic	S_Shore	26.83	6.24	30.94	5.81	-4.10	5.51E-04	1.09E-02
cg23004466	HBP1	7	5'UTR	Open sea	54.91	6.32	59.01	5.25	-4.10	1.30E-03	2.08E-02
cg00220677	PRLR	5	TSS200	Open sea	38.92	5.28	43.02	4.98	-4.10	2.24E-04	5.41E-03
cg08611402	ANTXR2;ANTXR2	4	Body;Body	Open sea	65.37	5.45	69.47	5.38	-4.10	6.78E-04	1.28E-02
cg11075745	CLDN19;CLDN19	1	TSS200;TSS200	S_Shore	50.07	4.58	54.17	5.18	-4.10	3.09E-04	6.96E-03
cg17285883	ARHGEF10L	1	5'UTR	Open sea	12.47	3.30	16.57	4.36	-4.10	5.61E-07	2.60E-05

cg19529583	SRRM3	7	Body	Open sea	65.37	4.48	69.47	4.57	-4.10	1.55E-05	5.57E-04
cg17158564	UCN3	10	TSS200	Open sea	61.10	6.03	65.20	5.79	-4.10	2.38E-03	3.19E-02
cg15269503	ANO1	11	TSS200	Island	6.84	3.43	10.94	5.41	-4.10	6.06E-06	2.38E-04
cg22235407	MEGF11	15	Body	Open sea	29.77	4.09	33.87	4.71	-4.10	1.66E-05	5.92E-04
cg18156136		7	Intergenic	Open sea	83.15	5.70	87.25	2.63	-4.10	1.14E-04	3.12E-03
cg20970369	DENND2D	1	TSS1500	N_Shelf	34.17	6.71	38.27	7.77	-4.10	2.96E-03	3.71E-02
cg03931865		16	Intergenic	Island	54.74	5.69	58.85	5.30	-4.10	2.62E-03	3.41E-02
cg25976440		4	Intergenic	Open sea	21.03	4.91	25.13	6.70	-4.10	3.82E-03	4.42E-02
cg05335893	THADA;THADA	2	Body;Body	Open sea	30.24	4.83	34.34	6.03	-4.10	2.49E-03	3.30E-02
cg13477806		1	Intergenic	Island	22.72	4.28	26.82	5.03	-4.10	1.21E-04	3.28E-03
cg15743907	PDE4DIP;PDE4DIP;PDE4DIP;PDE4DIP	1	Body;Body;Body;Body	Open sea	43.29	4.06	47.39	4.03	-4.10	8.02E-06	3.08E-04
cg10782923	DGKA;DGKA;DGKA;DGKA	12	5'UTR;5'UTR;5'UTR;5'UTR	Island	63.07	5.35	67.17	4.45	-4.10	3.56E-04	7.79E-03
cg08968034	GRK4;GRK4;GRK4	4	Body;Body;Body	Open sea	53.15	5.13	57.25	6.05	-4.10	2.31E-05	7.94E-04
cg13204977		7	Intergenic	N_Shelf	55.85	5.62	59.95	6.24	-4.10	4.12E-03	4.65E-02
cg08526209		3	Intergenic	S_Shelf	63.67	5.05	67.77	4.48	-4.10	2.88E-05	9.62E-04
cg03773647	YPEL4	11	TSS1500	S_Shelf	51.43	5.93	55.53	4.84	-4.10	2.45E-04	5.81E-03
cg23633210	NPAS2	2	Body	Open sea	67.77	5.32	71.87	4.84	-4.10	9.73E-04	1.68E-02
cg02742969		5	Intergenic	Island	41.43	3.08	45.53	4.19	-4.10	2.68E-06	1.12E-04
cg00330953	C6orf27	6	Body	Open sea	69.18	4.42	73.28	3.80	-4.10	2.07E-05	7.20E-04
cg02043679	ROBO4	11	TSS200	Open sea	44.66	4.25	48.76	5.00	-4.10	2.23E-05	7.70E-04
cg23102632	ZFP64	20	Body	S_Shore	74.33	5.57	78.43	5.60	-4.10	2.60E-03	3.40E-02
cg01998345	DDX56	7	Body	N_Shore	80.18	3.60	84.28	2.95	-4.10	3.65E-07	1.73E-05
cg12125772		5	Intergenic	N_Shore	39.95	4.64	44.05	5.37	-4.10	8.75E-04	1.55E-02
cg11498582	MDC1	6	5'UTR	N_Shore	65.78	4.91	69.87	5.06	-4.10	1.91E-03	2.73E-02
cg12208770	TMEM88B	1	1stExon	Island	62.55	4.77	66.65	4.59	-4.10	2.67E-04	6.22E-03
cg10043090	HCCA2	11	Body	Island	35.34	5.06	39.43	5.18	-4.10	2.28E-03	3.09E-02
cg01361263	FLNB;FLNB;FLNB;FLNB	3	Body;Body;Body;Body	Open sea	34.43	3.76	38.52	4.74	-4.10	5.72E-05	1.75E-03
cg26292150	ZDHHC19	3	Body	Island	30.26	6.08	34.35	7.06	-4.10	4.57E-03	5.00E-02
cg13512948		14	Intergenic	Open sea	37.19	4.60	41.28	5.25	-4.10	1.55E-03	2.36E-02
cg10799327		3	Intergenic	Open sea	41.31	2.98	45.40	3.07	-4.10	8.40E-09	4.49E-07
cg25495534		10	Intergenic	Open sea	71.07	5.45	75.16	3.90	-4.10	6.76E-04	1.28E-02
cg06206801	KCTD1	18	5'UTR	S_Shore	17.89	5.15	21.99	7.49	-4.09	2.83E-03	3.60E-02
cg27641532		6	Intergenic	S_Shore	33.45	5.38	37.54	6.04	-4.09	4.18E-03	4.70E-02
cg11336860		13	Intergenic	Island	23.07	6.31	27.16	5.36	-4.09	1.18E-03	1.94E-02
cg24645447	SDCCAG8	1	Body	Open sea	79.60	5.13	83.69	4.72	-4.09	4.13E-04	8.74E-03
cg18695800	FLOT1	6	Body	N_Shelf	88.14	3.91	92.23	1.74	-4.09	4.07E-08	2.09E-06
cg01766699	DNAH7	2	Body	Open sea	83.97	5.79	88.06	4.74	-4.09	5.68E-04	1.12E-02
cg00927494	ANO1	11	TSS200	N_Shore	14.25	4.05	18.34	5.46	-4.09	1.61E-04	4.15E-03
cg16902863	SSPO	7	TSS1500	S_Shore	64.36	4.96	68.45	3.95	-4.09	2.83E-04	6.50E-03
cg22531668	VAX1	10	3'UTR	N_Shore	26.60	4.08	30.69	5.32	-4.09	9.53E-05	2.69E-03
cg19720441	FAM155A	13	Body	Open sea	67.33	5.90	71.42	5.10	-4.09	4.15E-03	4.68E-02
cg01175550	HOXA3;HOXA3;HOXA3	7	3'UTR;3'UTR;3'UTR	N_Shore	27.18	4.46	31.27	5.90	-4.09	2.04E-03	2.87E-02
cg02902102	GRIN2D	19	Body	S_Shore	39.82	5.28	43.91	4.27	-4.09	5.82E-04	1.14E-02
cg07593582	SERTAD4	1	Body	Open sea	61.74	5.63	65.82	5.35	-4.09	6.21E-04	1.20E-02
cg09122197	LOC388692	1	Body	N_Shelf	38.49	3.79	42.58	2.97	-4.09	6.12E-06	2.41E-04
cg16328023	KIAA0895;KIAA0895	7	Body;Body	Open sea	51.25	4.50	55.34	4.09	-4.09	2.17E-06	9.24E-05
cg15862165	CRADD	12	Body	Open sea	35.01	5.48	39.10	5.36	-4.09	4.63E-04	9.54E-03
cg24690071	ZIC2	13	1stExon	Island	19.39	3.36	23.48	4.07	-4.09	3.70E-07	1.76E-05
cg10060065	ACCN1	17	Body	Island	61.02	5.91	65.11	5.19	-4.09	2.06E-03	2.89E-02
cg26872780	EPC2	2	Body	Open sea	66.83	4.24	70.92	4.32	-4.09	4.42E-05	1.40E-03
cg16639860	KCNIP4;KCNIP4;KCNIP4;KCNIP4;KCNIP	4	Body;Body;5'UTR;5'UTR;TSS1500	Open sea	39.37	5.57	43.46	5.27	-4.09	1.23E-03	2.00E-02
cg11857452	HIVEP3;HIVEP3	1	5'UTR;5'UTR	Open sea	58.10	7.04	62.19	5.88	-4.09	1.11E-03	1.85E-02
cg11966803	STK24;STK24	13	Body;Body	Open sea	44.59	5.29	48.68	6.11	-4.09	4.15E-03	4.68E-02

cg26608693	PRDM2	1	5'UTR	S_Shore	49.06	7.40	53.15	6.51	-4.09	1.51E-03	2.31E-02
cg17676428	TOLLIP	11	Body	N_Shore	63.46	4.92	67.55	5.32	-4.09	1.54E-03	2.35E-02
cg00901598	GHSR	3	Body	N_Shore	59.27	7.39	63.36	6.71	-4.09	4.50E-03	4.95E-02
cg02168291	CDH13	16	Body	Open sea	69.39	5.68	73.48	6.47	-4.09	6.36E-04	1.22E-02
cg11857033	RNF135;RNF135	17	TSS1500;TSS1500	N_Shore	32.18	4.61	36.27	5.35	-4.09	1.31E-04	3.49E-03
cg11389756	TRIM27	6	Body	Open sea	60.36	5.57	64.45	4.72	-4.09	1.01E-03	1.72E-02
cg26965236		8	Intergenic	Open sea	77.23	5.15	81.32	3.65	-4.09	7.31E-05	2.15E-03
cg22234962	RUSC1;RUSC1;C1orf104	1	5'UTR;5'UTR;5'UTR	N_Shore	21.25	4.76	25.34	4.74	-4.09	1.99E-04	4.93E-03
cg01915822	ADCK5	8	TSS1500	N_Shore	43.79	4.66	47.88	5.70	-4.09	1.86E-03	2.69E-02
cg14043104	KLF12	13	5'UTR	Open sea	58.52	5.08	62.61	4.75	-4.09	1.48E-03	2.28E-02
cg23196756	EIF4EBP2	10	Body	S_Shore	26.78	4.06	30.87	5.13	-4.09	8.48E-04	1.51E-02
cg10062945	EYS	6	Body	Open sea	72.69	6.38	76.78	4.15	-4.09	2.14E-03	2.96E-02
cg02416781		5	Intergenic	Open sea	39.36	5.92	43.45	5.38	-4.09	2.99E-03	3.73E-02
cg15936990	WASF2	1	5'UTR	N_Shore	35.69	6.91	39.77	6.49	-4.09	1.08E-04	2.98E-03
cg21330207	ZADH2	18	Body	Island	28.81	4.45	32.90	5.32	-4.09	1.16E-04	3.16E-03
cg20423064	FER	5	Body	Open sea	72.91	7.64	76.99	5.97	-4.09	3.76E-03	4.37E-02
cg06058209		5	Intergenic	Open sea	68.58	5.55	72.66	6.19	-4.09	1.87E-04	4.68E-03
cg04549076	PRG2	11	5'UTR	Open sea	67.26	3.85	71.34	3.65	-4.09	8.41E-07	3.81E-05
cg14176930		6	Intergenic	S_Shore	32.07	4.86	36.16	6.48	-4.09	2.65E-03	3.44E-02
cg21177257	ZNF323;ZKSCAN3;ZNF323	6	5'UTR;5'UTR;TSS200	Open sea	45.09	4.07	49.18	4.16	-4.08	1.48E-04	3.86E-03
cg24986868	PKP4;CCDC148;PKP4;CCDC148	2	TSS1500;5'UTR;TSS1500;Body	N_Shore	40.76	6.43	44.84	6.70	-4.08	7.61E-04	1.40E-02
cg14412111		7	Intergenic	Open sea	46.02	4.44	50.11	5.87	-4.08	7.98E-04	1.45E-02
cg27036456	NARS	18	Body	N_Shelf	79.76	4.81	83.84	3.76	-4.08	8.66E-06	3.31E-04
cg19804249	BEST4	1	TSS200	S_Shore	76.33	4.24	80.41	4.44	-4.08	2.48E-05	8.45E-04
cg18840931	SAPS3;SAPS3;SAPS3;SAPS3;SAPS3;SAI	11	Body;Body;Body;Body;Body;Body	Open sea	63.60	6.17	67.68	8.20	-4.08	9.47E-04	1.64E-02
cg13727089	KIAA1529	9	Body	S_Shore	65.77	4.93	69.85	3.38	-4.08	1.52E-04	3.96E-03
cg12441126	PRKAR1B;PRKAR1B;PRKAR1B;PRKAR1	7	5'UTR;5'UTR;5'UTR;5'UTR;5'UTR;5'UTR	Island	27.94	6.20	32.02	6.42	-4.08	1.98E-03	2.81E-02
cg25398112	ACOX3;ACOX3	4	5'UTR;5'UTR	S_Shelf	79.23	5.17	83.31	4.69	-4.08	2.67E-04	6.21E-03
cg13917099	SND1	7	Body	Open sea	80.13	5.06	84.21	5.45	-4.08	2.38E-03	3.19E-02
cg26417713	GPR179	17	Body	Open sea	67.71	4.95	71.79	5.32	-4.08	2.35E-04	5.61E-03
cg04362853	ZNF580	19	TSS1500	Island	27.79	5.23	31.87	6.01	-4.08	1.79E-03	2.62E-02
cg20439889	CHST8;CHST8;CHST8;CHST8	19	5'UTR;5'UTR;1stExon;5'UTR	Island	28.09	3.59	32.17	4.44	-4.08	1.64E-05	5.86E-04
cg15708405		20	Intergenic	Island	71.25	4.09	75.33	4.77	-4.08	6.62E-05	1.98E-03
cg03998636	RAB20	13	Body	N_Shelf	48.22	4.26	52.30	4.48	-4.08	1.39E-04	3.68E-03
cg00168191	GNA11	19	Body	Island	70.67	5.46	74.75	4.61	-4.08	5.81E-04	1.14E-02
cg03899098		15	Intergenic	N_Shelf	53.16	6.22	57.24	5.68	-4.08	1.90E-03	2.72E-02
cg03820048		17	Intergenic	Open sea	72.73	6.38	76.82	6.15	-4.08	3.26E-03	3.97E-02
cg17797591	AXIN2	17	5'UTR	N_Shore	55.11	8.53	59.19	7.10	-4.08	3.81E-03	4.42E-02
cg07485417	NPM2	8	Body	Island	47.63	5.01	51.71	6.05	-4.08	2.80E-03	3.57E-02
cg17826893	CLYBL	13	Body	Open sea	67.54	5.77	71.62	5.85	-4.08	2.44E-03	3.25E-02
cg18418457	TGFBI	5	TSS1500	N_Shore	28.44	3.82	32.52	4.45	-4.08	9.99E-06	3.76E-04
cg09888297		1	Intergenic	S_Shelf	25.17	5.63	29.25	6.20	-4.08	2.14E-06	9.14E-05
cg24241928	SFRP2	4	TSS1500	N_Shore	29.65	4.70	33.73	3.96	-4.08	7.36E-06	2.85E-04
cg04829143		10	Intergenic	Open sea	81.45	4.25	85.53	3.59	-4.08	1.93E-05	6.76E-04
cg23424125	DLGAP2	8	5'UTR	N_Shore	56.19	4.92	60.27	5.15	-4.08	8.11E-04	1.46E-02
cg23890469	MMRN2	10	Body	Open sea	50.64	3.68	54.72	3.93	-4.08	1.45E-05	5.26E-04
cg14958018	NKX6-3	8	TSS200	S_Shore	80.48	5.11	84.56	5.19	-4.08	2.88E-04	6.60E-03
cg12136950	ITPKB	1	Body	Open sea	60.53	4.63	64.60	4.31	-4.08	8.37E-05	2.41E-03
cg07558209		12	Intergenic	S_Shelf	66.57	6.95	70.65	5.97	-4.08	3.87E-03	4.46E-02
cg05074709	TGFBR2;TGFBR2	3	3'UTR;3'UTR	Open sea	80.20	3.71	84.28	3.49	-4.08	2.01E-06	8.63E-05
cg02598872		15	Intergenic	N_Shore	21.26	3.49	25.34	5.39	-4.08	1.64E-04	4.22E-03
cg08471738	PELI1	2	TSS1500	S_Shore	49.69	5.20	53.76	4.73	-4.08	7.25E-04	1.35E-02
cg24168538		4	Intergenic	Open sea	46.41	5.16	50.49	4.60	-4.08	8.38E-04	1.50E-02

cg26075408		2	Intergenic	Open sea	46.70	4.98	50.78	4.82	-4.08	4.82E-05	1.51E-03
cg19428722	HPSE2;HPSE2;HPSE2;HPSE2	10	Body;Body;Body;Body	S_Shore	19.30	4.06	23.37	4.95	-4.08	1.82E-06	7.85E-05
cg27579805	TNRC6C;TNRC6C	17	3'UTR;3'UTR	S_Shore	61.14	5.74	65.22	4.38	-4.08	8.66E-04	1.54E-02
cg21279601	DRD5	4	TSS1500	N_Shore	50.59	4.91	54.66	4.81	-4.08	3.70E-04	8.02E-03
cg16553083		1	Intergenic	Open sea	89.86	4.97	93.93	1.82	-4.08	3.14E-07	1.50E-05
cg10096215	DPEP2	16	TSS1500	Open sea	57.92	5.69	62.00	5.29	-4.08	5.70E-04	1.12E-02
cg26475369	TRIM31	6	Body	Open sea	57.82	3.90	61.90	4.26	-4.08	5.07E-05	1.57E-03
cg18397730	TRAM2	6	Body	N_Shelf	74.20	4.74	78.28	4.10	-4.08	9.96E-05	2.79E-03
cg12167548		2	Intergenic	Open sea	55.57	6.17	59.64	5.11	-4.07	9.58E-04	1.66E-02
cg19511229		12	Intergenic	N_Shelf	79.14	6.62	83.22	4.47	-4.07	2.40E-03	3.21E-02
cg11765288	HDAC9;HDAC9;HDAC9;HDAC9;HDAC9	7	TSS200;5'UTR;5'UTR;TSS200;TSS200	Open sea	17.21	3.33	21.28	4.34	-4.07	1.12E-06	4.99E-05
cg25732381	NPY2R	4	5'UTR	S_Shelf	63.43	5.71	67.50	5.43	-4.07	1.48E-04	3.88E-03
cg19024352	USP32	17	Body	Open sea	61.15	6.42	65.23	4.81	-4.07	4.49E-03	4.94E-02
cg14372606	EEF1DP3	13	Body	S_Shelf	80.38	4.84	84.46	3.35	-4.07	2.72E-05	9.17E-04
cg08827307	ADAM32	8	TSS200	Island	26.56	5.03	30.64	7.07	-4.07	4.81E-04	9.82E-03
cg07536600	NPC1L1;NPC1L1	7	Body;Body	Open sea	35.91	3.95	39.98	5.50	-4.07	8.67E-04	1.54E-02
cg03053374	RARA	17	TSS200	Open sea	13.39	3.37	17.47	4.29	-4.07	6.89E-07	3.16E-05
cg16435910		2	Intergenic	Open sea	73.19	4.16	77.26	3.94	-4.07	1.51E-05	5.45E-04
cg06795425		2	Intergenic	Open sea	82.13	5.33	86.20	3.90	-4.07	3.31E-05	1.09E-03
cg22622328		21	Intergenic	N_Shelf	59.40	6.87	63.48	6.43	-4.07	8.39E-04	1.50E-02
cg14511498		3	Intergenic	N_Shelf	79.90	5.81	83.97	4.64	-4.07	2.17E-03	2.99E-02
cg06902099	ANKS1A	6	Body	Open sea	54.13	4.83	58.20	4.02	-4.07	1.23E-04	3.33E-03
cg07042489	SLC38A9	5	Body	Open sea	77.00	5.91	81.07	4.15	-4.07	1.57E-03	2.38E-02
cg15424882		12	Intergenic	Open sea	71.79	4.29	75.86	5.09	-4.07	1.37E-04	3.64E-03
cg11752894	TMCC2	1	TSS1500	N_Shore	79.62	4.30	83.69	4.18	-4.07	1.13E-04	3.09E-03
cg11796455		6	Intergenic	S_Shore	22.60	4.27	26.67	5.27	-4.07	2.99E-05	9.94E-04
cg04656051	SLC11A1	2	Body	Island	36.07	4.65	40.15	6.10	-4.07	7.75E-05	2.26E-03
cg06982190		7	Intergenic	Island	36.71	4.33	40.78	4.90	-4.07	4.09E-05	1.31E-03
cg11409510	PRICKLE1	12	5'UTR	N_Shore	38.99	4.67	43.06	5.35	-4.07	1.08E-03	1.81E-02
cg09662930		15	Intergenic	Open sea	28.71	5.39	32.78	6.47	-4.07	4.13E-03	4.67E-02
cg05257610	CEBPZ;C2orf56;C2orf56	2	Body;TSS1500;TSS1500	N_Shore	17.07	3.83	21.15	3.95	-4.07	3.61E-07	1.71E-05
cg10616694	SLC12A1	15	Body	Open sea	78.49	4.85	82.56	4.40	-4.07	1.39E-04	3.68E-03
cg20003638	SLC12A5;SLC12A5	20	3'UTR;3'UTR	S_Shore	58.67	4.74	62.74	6.43	-4.07	2.17E-03	2.99E-02
cg06638433	IGF2BP1;IGF2BP1	17	1stExon;1stExon	Island	28.32	3.67	32.39	4.05	-4.07	8.69E-07	3.93E-05
cg01005486		3	Intergenic	Island	40.77	5.15	44.84	5.75	-4.07	1.30E-04	3.49E-03
cg20741474	MYRIP	3	5'UTR	S_Shelf	80.83	5.29	84.90	3.55	-4.07	2.35E-04	5.62E-03
cg21242417	EXD3	9	Body	N_Shore	52.01	4.40	56.08	4.85	-4.07	4.94E-05	1.54E-03
cg11376227	ALPK1;ALPK1	4	Body;Body	Island	80.54	4.99	84.61	4.20	-4.07	2.13E-04	5.19E-03
cg01205011	ZNF76	6	Body	Open sea	34.39	3.69	38.45	4.17	-4.07	6.04E-06	2.38E-04
cg15123428	KLF11	2	Body	S_Shore	23.74	4.48	27.81	4.92	-4.07	4.91E-04	9.98E-03
cg06910580		12	Intergenic	Open sea	59.93	5.88	64.00	4.02	-4.07	3.51E-04	7.70E-03
cg03541057	ANP32E;ANP32E;ANP32E	1	Body;5'UTR;Body	N_Shore	26.41	4.05	30.48	4.58	-4.07	9.84E-07	4.42E-05
cg23615488		1	Intergenic	Open sea	86.96	2.80	91.03	1.79	-4.07	3.18E-11	1.86E-09
cg15081844	BFSP2	3	TSS1500	Open sea	79.81	3.44	83.87	2.82	-4.07	6.98E-09	3.74E-07
cg26672202	GAB2;GAB2	11	Body;Body	Open sea	51.45	4.80	55.52	5.21	-4.07	3.29E-04	7.32E-03
cg12078229	TMEM161A	19	Body	N_Shelf	28.00	4.56	32.06	5.67	-4.07	3.43E-03	4.11E-02
cg12961080	BCL11B;BCL11B	14	Body;Body	Island	31.32	5.12	35.38	5.47	-4.07	5.55E-04	1.10E-02
cg16032841	C13orf29	13	Body	Open sea	50.57	5.99	54.64	7.14	-4.07	6.22E-04	1.20E-02
cg02947125		14	Intergenic	Open sea	45.02	5.08	49.09	5.93	-4.07	3.48E-03	4.15E-02
cg19494811	NUDT3	6	Body	N_Shelf	71.02	7.09	75.08	7.22	-4.07	2.96E-03	3.71E-02
cg19844653	TCF7;TCF7;TCF7;TCF7;TCF7;TCF7;TCF7	5	TSS200;TSS1500;TSS1500;TSS200;Body;TS	S_Shore	16.36	3.77	20.43	4.51	-4.07	3.79E-08	1.95E-06
cg13978352	DPCR1	6	TSS200	Open sea	36.40	4.92	40.47	5.81	-4.07	1.28E-03	2.06E-02
cg23173688		1	Intergenic	Island	45.45	4.66	49.51	6.15	-4.07	1.91E-04	4.77E-03

cg12741994	CLDN11	3	Body	Island	13.84	3.58	17.91	5.12	-4.06	8.83E-06	3.37E-04
cg07551200		10	Intergenic	Open sea	65.54	3.99	69.61	3.96	-4.06	2.90E-05	9.68E-04
cg20235117	ATP2B2;ATP2B2	3	3'UTR;3'UTR	N_Shelf	55.81	6.43	59.88	5.04	-4.06	1.56E-03	2.37E-02
cg01185754	F3	1	Body	N_Shore	32.74	6.15	36.80	5.94	-4.06	2.47E-04	5.85E-03
cg09501047	PBX1	1	Body	Open sea	84.90	4.10	88.97	2.82	-4.06	2.26E-08	1.18E-06
cg04467618	TCF21;TCF21	6	1stExon;1stExon	Island	24.43	4.21	28.49	4.67	-4.06	3.95E-05	1.27E-03
cg24528602	SPTBN1;SPTBN1	2	Body;Body	Open sea	36.61	4.72	40.67	5.70	-4.06	3.96E-03	4.54E-02
cg19920029	MDGA2;MDGA2	14	Body;Body	Open sea	81.50	4.90	85.57	3.81	-4.06	1.77E-05	6.27E-04
cg11659942		2	Intergenic	Open sea	23.75	4.41	27.81	5.66	-4.06	3.11E-04	6.99E-03
cg26952618	FAM18A;FAM18A	16	1stExon;5'UTR	Island	49.17	5.77	53.23	6.00	-4.06	3.25E-03	3.96E-02
cg20235510	ZNF311	6	Body	Open sea	63.92	5.13	67.99	5.12	-4.06	1.52E-03	2.33E-02
cg22851557	PRDM16;PRDM16	1	Body;Body	Island	47.61	3.77	51.67	3.96	-4.06	3.57E-05	1.16E-03
cg10266121	PDLIM1	10	Body	Open sea	41.58	6.31	45.64	5.91	-4.06	3.16E-05	1.05E-03
cg22773661	ZAR1	4	TSS200	Island	15.44	5.91	19.50	8.32	-4.06	1.39E-03	2.18E-02
cg06711175		5	Intergenic	Open sea	39.96	7.14	44.03	6.88	-4.06	3.65E-03	4.28E-02
cg12761626	NFATC1;NFATC1;NFATC1;NFATC1;NFA	18	Body;5'UTR;Body;Body;Body	N_Shelf	85.88	4.70	89.94	2.96	-4.06	8.09E-06	3.11E-04
cg07471943	SSTR1	14	Body	Island	46.53	5.81	50.59	4.97	-4.06	4.35E-04	9.09E-03
cg15127443	EIF2C4	1	3'UTR	Open sea	78.87	3.93	82.93	4.02	-4.06	4.26E-06	1.73E-04
cg08665243	PCDHB16	5	1stExon	N_Shore	25.57	5.64	29.63	7.06	-4.06	4.17E-03	4.69E-02
cg05657618	NGEF;NGEF;NGEF	2	1stExon;Body;5'UTR	Island	48.64	7.22	52.70	7.05	-4.06	6.56E-04	1.25E-02
cg22147598	MAF1;KIAA1875	8	3'UTR;TSS200	N_Shore	45.90	4.53	49.96	4.99	-4.06	9.53E-04	1.65E-02
cg20026664	AFF1;AFF1	4	Body;Body	Open sea	64.23	4.92	68.29	4.48	-4.06	8.43E-05	2.43E-03
cg12612465		6	Intergenic	Open sea	68.04	6.17	72.10	4.72	-4.06	2.39E-03	3.21E-02
cg06808089	LIMD1	3	Body	Open sea	61.91	4.87	65.97	4.22	-4.06	3.61E-04	7.87E-03
cg22165175	KCNA2	1	TSS1500	N_Shore	43.48	5.18	47.54	5.41	-4.06	9.72E-04	1.68E-02
cg14423651		8	Intergenic	Open sea	60.18	5.75	64.24	6.07	-4.06	2.79E-03	3.57E-02
cg08082862	RANBP10	16	Body	Open sea	35.48	4.99	39.54	4.36	-4.06	5.54E-04	1.10E-02
cg25144558	SDK1	7	Body	Open sea	53.59	5.14	57.65	6.20	-4.06	5.73E-04	1.13E-02
cg00999904	ALLC	2	TSS1500	Open sea	80.07	6.53	84.13	4.74	-4.06	1.29E-03	2.06E-02
cg10467557		13	Intergenic	N_Shore	67.71	6.19	71.77	6.07	-4.06	4.42E-03	4.89E-02
cg05151395	SLC25A35;RANGRF	17	3'UTR;Body	Island	17.92	4.66	21.98	5.21	-4.06	1.98E-04	4.90E-03
cg20454073	NFIX	19	Body	Island	57.51	4.82	61.57	5.46	-4.06	2.18E-04	5.30E-03
cg26005973	PCBD1	10	3'UTR	Open sea	67.06	4.11	71.12	3.59	-4.06	6.52E-05	1.95E-03
cg09694722	INS-IGF2;IGF2AS;IGF2;IGF2;IGF2AS;IG	11	Body;TSS1500;Body;5'UTR;TSS1500;TSS15	Island	44.18	5.17	48.24	4.28	-4.06	4.44E-04	9.25E-03
cg27178045	STK31;STK31;STK31	7	Body;Body;Body	Open sea	57.59	5.22	61.65	3.84	-4.06	1.26E-06	5.57E-05
cg09780180		16	Intergenic	N_Shore	25.82	5.12	29.88	6.79	-4.06	1.12E-03	1.87E-02
cg04076044		6	Intergenic	Open sea	65.16	5.30	69.21	6.50	-4.06	1.47E-03	2.27E-02
cg06685949	C16orf88	16	Body	Open sea	58.27	4.06	62.33	4.55	-4.06	3.60E-05	1.17E-03
cg10146780	TXNDC16;TXNDC16	14	3'UTR;3'UTR	Open sea	82.69	4.70	86.74	4.69	-4.06	1.96E-04	4.86E-03
cg14003974	CDC42BPB	14	Body	Open sea	27.06	4.56	31.12	4.82	-4.06	4.32E-04	9.05E-03
cg18466965	FBXO21;FBXO21	12	TSS1500;TSS1500	S_Shore	51.52	6.30	55.58	7.02	-4.06	2.55E-03	3.34E-02
cg03173942	RYBP	3	Body	Open sea	80.96	4.90	85.02	4.03	-4.06	3.13E-04	7.03E-03
cg15981851	AGAP1;AGAP1	2	Body;Body	Island	44.28	3.92	48.34	4.16	-4.06	2.58E-06	1.09E-04
cg26424649		7	Intergenic	Open sea	63.74	5.12	67.80	5.15	-4.06	1.21E-03	1.97E-02
cg00363813	IFFO1;IFFO1	12	1stExon;1stExon	Island	4.81	2.14	8.86	5.86	-4.06	1.86E-05	6.54E-04
cg24574546	TMEM9B	11	3'UTR	Open sea	69.58	5.02	73.63	4.88	-4.06	2.33E-05	7.99E-04
cg06867571	LTBP3;LTBP3;LTBP3	11	Body;Body;Body	Island	72.15	6.35	76.20	6.62	-4.06	1.57E-03	2.38E-02
cg00019678	C12orf34	12	5'UTR	S_Shore	32.92	4.29	36.98	5.35	-4.06	8.89E-04	1.57E-02
cg26271591	NFE2L2;NFE2L2;NFE2L2	2	Body;5'UTR;5'UTR	N_Shelf	24.88	4.58	28.94	5.33	-4.06	1.15E-03	1.90E-02
cg07544102	COL6A2	21	Body	N_Shore	65.54	4.85	69.59	4.57	-4.05	1.46E-03	2.26E-02
cg25676335	RB1	13	Body	S_Shore	68.17	4.85	72.23	4.64	-4.05	6.75E-04	1.27E-02
cg05149097		11	Intergenic	N_Shelf	47.33	6.01	51.39	5.30	-4.05	9.87E-04	1.70E-02
cg11409350	NT5C	17	3'UTR	N_Shore	69.62	5.82	73.67	5.25	-4.05	3.32E-03	4.02E-02

cg12190613	SDC2	8	3'UTR	Open sea	60.86	4.56	64.91	4.67	-4.05	9.29E-04	1.62E-02
cg09725939		20	Intergenic	S_Shelf	71.41	5.10	75.46	4.73	-4.05	2.10E-03	2.93E-02
cg21229268	OLIG1	21	TSS200	Island	10.43	2.21	14.49	3.07	-4.05	2.73E-12	1.65E-10
cg14037728	ZNF618	9	Body	Open sea	38.52	5.74	42.58	4.38	-4.05	2.72E-04	6.31E-03
cg26942801	C5orf39;C5orf39	5	1stExon;3'UTR	N_Shore	55.94	7.11	59.99	6.50	-4.05	2.67E-03	3.46E-02
cg13033898	AKAP12	6	TSS200	N_Shore	20.87	4.02	24.92	5.72	-4.05	4.27E-04	8.98E-03
cg10655371	CYP51A1;CYP51A1	7	Body;Body	Open sea	67.79	4.21	71.85	3.99	-4.05	1.55E-05	5.58E-04
cg27631817		6	Intergenic	Open sea	30.26	4.96	34.31	6.29	-4.05	2.53E-04	5.97E-03
cg11857548		6	Intergenic	Open sea	71.55	4.89	75.61	5.68	-4.05	2.96E-03	3.71E-02
cg01208537	CDC42BPG	11	Body	Island	79.47	5.44	83.52	4.22	-4.05	1.30E-03	2.08E-02
cg01911386	ERICH1	8	Body	S_Shore	63.70	5.88	67.75	6.19	-4.05	2.67E-03	3.45E-02
cg14342532		2	Intergenic	Open sea	25.83	5.00	29.88	5.09	-4.05	2.00E-04	4.95E-03
cg03299042	OTX1	2	3'UTR	N_Shore	60.34	4.58	64.39	5.54	-4.05	6.04E-05	1.83E-03
cg25560518		4	Intergenic	Open sea	64.20	5.95	68.25	5.00	-4.05	4.90E-05	1.53E-03
cg05511924	DIRC3	2	Body	Open sea	54.81	4.72	58.86	5.10	-4.05	4.66E-06	1.88E-04
cg25742037	INS-IGF2;IGF2;INS-IGF2	11	Body;TSS1500;Body	Open sea	59.82	4.93	63.87	3.47	-4.05	9.21E-04	1.61E-02
cg27504782	SGCE;SGCE;PEG10;SGCE;PEG10	7	Body;Body;TSS1500;Body;TSS1500	N_Shore	22.42	5.16	26.47	6.12	-4.05	7.46E-04	1.38E-02
cg09001440	CA5A	16	Body	Open sea	29.91	3.81	33.96	4.22	-4.05	1.32E-04	3.52E-03
cg15659527	TTC7A	2	Body	Open sea	68.33	6.89	72.38	5.18	-4.05	3.79E-03	4.40E-02
cg15702661	NFATC1;NFATC1;NFATC1;NFATC1;NFA	18	Body;5'UTR;Body;Body;Body	Island	68.09	8.05	72.14	7.07	-4.05	3.15E-03	3.87E-02
cg16942327		3	Intergenic	Open sea	81.19	5.25	85.24	4.05	-4.05	5.36E-04	1.07E-02
cg06210447	SHANK2	11	Body	Open sea	27.12	3.49	31.16	4.40	-4.05	4.04E-05	1.29E-03
cg05913684	PALM;PALM	19	Body;Body	Island	53.63	4.65	57.68	5.02	-4.05	6.27E-04	1.21E-02
cg24720242		4	Intergenic	Open sea	58.74	5.64	62.79	6.21	-4.05	1.93E-03	2.76E-02
cg21190593		11	Intergenic	Open sea	67.38	3.91	71.43	4.16	-4.05	3.54E-06	1.45E-04
cg15223157	CHEK1;CHEK1;CHEK1	11	Body;Body;Body	S_Shelf	82.94	7.00	86.99	5.76	-4.05	1.44E-03	2.24E-02
cg11782091	SQSTM1;SQSTM1;SQSTM1	5	Body;Body;Body	S_Shelf	53.62	4.39	57.67	4.63	-4.05	4.68E-04	9.63E-03
cg08608800	ZC3H13	13	TSS200	Island	6.09	2.99	10.14	7.63	-4.05	1.09E-03	1.82E-02
cg13557773	RASA3	13	Body	Island	51.13	4.52	55.18	3.94	-4.05	6.81E-05	2.03E-03
cg14000856	SLC28A3	9	5'UTR	Open sea	76.05	4.90	80.09	4.03	-4.05	4.78E-05	1.49E-03
cg05095466	C5orf42	5	Body	N_Shore	78.71	6.53	82.75	5.33	-4.05	2.37E-03	3.19E-02
cg14479889	APC;APC;APC	5	TSS200;TSS200;5'UTR	Open sea	30.08	7.83	34.13	9.30	-4.05	1.05E-03	1.78E-02
cg12219715	FAM20C	7	Body	S_Shore	48.75	4.34	52.80	5.21	-4.05	1.15E-03	1.90E-02
cg24335984	HYAL2	3	TSS1500	S_Shore	59.76	4.00	63.80	3.77	-4.05	1.95E-05	6.85E-04
cg18573082	PDE7B	6	Body	Open sea	35.46	4.47	39.50	6.35	-4.05	3.35E-03	4.04E-02
cg14251622	INSM2	14	1stExon	Island	12.69	3.75	16.73	5.49	-4.05	7.98E-06	3.07E-04
cg11738543	SOCS2	12	Body	Island	10.17	2.36	14.21	3.85	-4.04	4.69E-08	2.40E-06
cg04265797	SSTR1	14	3'UTR	Island	23.97	5.66	28.01	5.76	-4.04	3.54E-04	7.75E-03
cg15555017	ETS1	11	5'UTR	Open sea	32.67	5.42	36.71	4.84	-4.04	1.64E-03	2.45E-02
cg11711057	BTBD11;BTBD11	12	TSS200;Body	N_Shore	35.73	4.71	39.77	5.43	-4.04	1.85E-03	2.68E-02
cg26128481		14	Intergenic	Open sea	51.68	4.57	55.72	4.61	-4.04	1.13E-04	3.11E-03
cg23063647	ZNF876P	4	TSS200	N_Shore	30.05	5.10	34.10	5.45	-4.04	1.14E-03	1.89E-02
cg16703135		7	Intergenic	Island	45.77	4.76	49.81	5.08	-4.04	1.19E-03	1.95E-02
cg11552829	FOXI1;FOXI1	5	TSS200;TSS200	N_Shore	80.34	6.41	84.38	5.53	-4.04	1.70E-03	2.51E-02
cg04823492	ENTPD1	10	TSS1500	Open sea	65.78	5.13	69.82	4.18	-4.04	5.00E-05	1.56E-03
cg27527630	WIPF3	7	Body	N_Shore	49.35	6.35	53.39	5.65	-4.04	3.51E-04	7.69E-03
cg18375707	PLCB3	11	Body	Island	73.97	4.17	78.01	3.18	-4.04	1.99E-05	6.96E-04
cg17499822		1	Intergenic	Open sea	78.89	4.87	82.93	3.56	-4.04	6.54E-05	1.96E-03
cg05995220	RBM38;RBM38	20	TSS1500;TSS1500	Island	29.22	4.44	33.26	5.31	-4.04	7.77E-05	2.26E-03
cg22581896	FOXK1	7	Body	Open sea	22.70	4.50	26.75	5.63	-4.04	1.30E-03	2.08E-02
cg22137572	CDH18;CDH18	5	5'UTR;5'UTR	Open sea	27.09	4.19	31.13	5.12	-4.04	3.90E-04	8.36E-03
cg11464297		10	Intergenic	Open sea	47.85	6.33	51.89	6.90	-4.04	1.19E-03	1.95E-02
cg00012362	S100A13;S100A13;S100A13;S100A13;	1	5'UTR;1stExon;5'UTR;5'UTR;TSS1500;5'UT	Open sea	23.84	4.60	27.88	4.62	-4.04	8.29E-04	1.49E-02

cg26309929		3	Intergenic	Open sea	59.99	5.65	64.03	3.48	-4.04	4.01E-04	8.53E-03
cg06752482	C17orf64	17	TSS200	S_Shore	70.30	4.38	74.34	3.34	-4.04	3.20E-05	1.06E-03
cg03623968	RAB7A	3	Body	Open sea	40.78	6.88	44.82	6.65	-4.04	2.20E-04	5.32E-03
cg25422895		8	Intergenic	Open sea	82.48	3.41	86.52	3.51	-4.04	1.26E-06	5.58E-05
cg13287523	HAPLN3	15	TSS1500	S_Shore	68.99	5.25	73.03	6.03	-4.04	8.02E-04	1.45E-02
cg12876594	NPR2	9	TSS1500	Island	14.58	4.67	18.62	5.18	-4.04	2.53E-03	3.33E-02
cg03065614	TMEM63B;MRPL14	6	5'UTR;TSS1500	S_Shore	56.12	4.46	60.16	4.48	-4.04	1.33E-04	3.53E-03
cg08414647	GPR156;GPR156	3	TSS1500;TSS1500	Open sea	39.75	3.76	43.79	4.14	-4.04	6.37E-06	2.49E-04
cg06613392		6	Intergenic	Island	38.56	5.01	42.59	4.14	-4.04	1.46E-04	3.82E-03
cg17022727	MIR25;MCM7;MCM7;MIR106B;MIR9	7	TSS1500;Body;Body;Body;TSS200	Open sea	64.37	4.68	68.41	3.80	-4.04	1.57E-04	4.06E-03
cg24064055		8	Intergenic	Island	37.37	4.97	41.41	5.87	-4.04	4.76E-04	9.75E-03
cg13049862	MYO1C;MYO1C;MYO1C	17	Body;Body;Body	S_Shelf	56.09	3.14	60.13	3.19	-4.04	1.64E-08	8.66E-07
cg05407909	STX2;STX2	12	Body;Body	N_Shore	50.69	6.93	54.73	6.84	-4.04	1.31E-03	2.09E-02
cg08792272		8	Intergenic	Open sea	57.58	7.12	61.62	5.76	-4.04	9.32E-04	1.63E-02
cg10277770	MAPK8IP3;MAPK8IP3	16	Body;Body	Island	76.85	5.15	80.89	3.83	-4.04	2.14E-04	5.21E-03
cg26153162	CCDC141	2	TSS1500	Open sea	43.39	4.62	47.43	5.64	-4.04	1.64E-03	2.45E-02
cg14199261	CDK6;CDK6	7	Body;Body	Open sea	67.64	5.78	71.68	5.71	-4.04	3.64E-04	7.91E-03
cg24989534	MREG	2	TSS200	S_Shore	23.11	8.08	27.15	9.30	-4.04	1.96E-03	2.79E-02
cg26727768	LOC145845	15	Body	N_Shore	66.77	6.50	70.81	7.56	-4.04	1.82E-03	2.65E-02
cg21253087	SNAPC4	9	Body	N_Shelf	44.14	4.20	48.17	4.89	-4.04	7.40E-04	1.37E-02
cg07344338		3	Intergenic	Open sea	71.77	6.13	75.80	6.35	-4.04	3.89E-03	4.47E-02
cg07577837	ARID1B;ARID1B;ARID1B	6	Body;Body;Body	Open sea	80.45	6.32	84.48	5.25	-4.04	7.36E-04	1.36E-02
cg14329680		11	Intergenic	Open sea	73.77	7.03	77.81	6.20	-4.04	1.54E-03	2.34E-02
cg09085216	PLEKHA6	1	TSS200	Open sea	28.24	4.57	32.28	3.94	-4.04	3.49E-04	7.66E-03
cg06354193	PRDM16;PRDM16	1	Body;Body	Island	64.80	4.32	68.84	3.95	-4.04	2.75E-05	9.27E-04
cg06704567		2	Intergenic	Open sea	43.67	5.63	47.70	5.52	-4.04	7.62E-04	1.40E-02
cg20150017	ANO1	11	TSS200	Island	8.99	3.40	13.02	5.26	-4.03	1.02E-05	3.85E-04
cg06042565	CPT1A;CPT1A	11	Body;Body	Open sea	40.99	4.12	45.02	4.11	-4.03	1.11E-04	3.04E-03
cg11342453		6	Intergenic	N_Shore	53.51	5.65	57.54	6.12	-4.03	4.25E-04	8.94E-03
cg03853587	LEPROT;LEPR;LEPR;LEPR	1	TSS1500;TSS1500;TSS1500;TSS1500	N_Shore	17.93	4.14	21.96	4.84	-4.03	4.74E-05	1.48E-03
cg26955287	ASPSCR1	17	Body	S_Shore	61.58	5.74	65.61	4.92	-4.03	3.53E-03	4.19E-02
cg15394558	CCDC42;CCDC42	17	TSS200;TSS200	N_Shore	33.94	4.90	37.97	6.09	-4.03	3.50E-03	4.17E-02
cg26864388		10	Intergenic	Open sea	76.97	7.12	81.01	5.09	-4.03	1.88E-03	2.71E-02
cg19948457	PPP3CA;PPP3CA;PPP3CA	4	Body;Body;Body	Open sea	76.47	6.33	80.50	6.38	-4.03	2.57E-03	3.37E-02
cg12973904		7	Intergenic	N_Shelf	43.59	5.30	47.63	5.14	-4.03	3.37E-04	7.46E-03
cg02166532	INS-IGF2;IGF2;IGF2;IGF2;IGF2	11	Body;Body;5'UTR;5'UTR;1stExon	Island	34.01	4.36	38.05	4.05	-4.03	2.81E-04	6.47E-03
cg05475091	FRYL	4	5'UTR	Open sea	80.14	4.85	84.18	3.67	-4.03	1.33E-05	4.86E-04
cg09695996	C1QTNF8	16	3'UTR	N_Shore	27.45	4.50	31.49	4.22	-4.03	9.52E-05	2.69E-03
cg01604051	GRB10	7	5'UTR	S_Shore	73.51	4.33	77.54	4.54	-4.03	1.27E-05	4.67E-04
cg19908768	SULT1C4	2	TSS200	Open sea	43.60	5.13	47.63	5.38	-4.03	4.43E-04	9.23E-03
cg06464468	SIPA1;SIPA1	11	Body;Body	Island	72.91	6.09	76.94	6.09	-4.03	8.16E-04	1.47E-02
cg03188976		7	Intergenic	Open sea	42.82	4.72	46.85	4.64	-4.03	9.58E-05	2.70E-03
cg02367655	RASAL2;LOC100302401	1	Body;TSS1500	S_Shore	31.44	6.59	35.47	8.04	-4.03	5.24E-04	1.05E-02
cg22475353	ZNF331;ZNF331	19	TSS200;5'UTR	Island	20.12	4.11	24.15	4.43	-4.03	6.51E-05	1.95E-03
cg11224984	SKI	1	Body	Open sea	68.74	4.48	72.77	4.34	-4.03	2.67E-04	6.21E-03
cg03238687	ZNF487	10	Body	Open sea	68.84	3.38	72.87	3.94	-4.03	1.55E-06	6.78E-05
cg23770142	APOM	6	Body	N_Shelf	83.15	3.88	87.18	3.66	-4.03	1.07E-05	4.02E-04
cg02869163	AGPAT3;AGPAT3	21	5'UTR;5'UTR	S_Shore	55.96	4.63	59.99	5.11	-4.03	3.10E-04	6.97E-03
cg10089963	NR4A2	2	Body	N_Shore	80.94	4.59	84.97	3.40	-4.03	5.46E-06	2.17E-04
cg00081714		5	Intergenic	Open sea	37.31	4.56	41.34	5.48	-4.03	1.01E-03	1.73E-02
cg15697036	FGFR3;FGFR3;FGFR3	4	Body;Body;Body	S_Shore	64.05	4.61	68.08	5.52	-4.03	1.30E-03	2.07E-02
cg17748520		1	Intergenic	S_Shore	15.98	5.90	20.01	5.72	-4.03	1.97E-03	2.80E-02
cg09643054	C3AR1	12	3'UTR	Open sea	71.48	4.93	75.51	5.79	-4.03	8.78E-04	1.55E-02

cg16948335	PSMD9	12	Body	Open sea	56.13	4.44	60.16	4.92	-4.03	4.05E-04	8.59E-03
cg15782852	KCNQ1;KCNQ1	11	Body;Body	Open sea	67.57	5.94	71.60	6.13	-4.03	4.34E-03	4.83E-02
cg02479022	SLC39A14;SLC39A14;SLC39A14;SLC39.	8	5'UTR;5'UTR;5'UTR;5'UTR	Open sea	33.63	4.93	37.65	5.01	-4.03	2.77E-03	3.54E-02
cg03357077	SNAPC5	15	TSS1500	S_Shore	32.23	3.46	36.26	4.08	-4.03	2.04E-05	7.10E-04
cg16531622		3	Intergenic	Open sea	71.69	5.40	75.72	5.51	-4.03	4.10E-04	8.68E-03
cg25034941	LRCH1	13	3'UTR	Open sea	58.03	5.30	62.05	6.53	-4.03	3.62E-04	7.89E-03
cg21810108	LOC255411;LOC255411	12	Body;Body	Open sea	45.11	5.76	49.13	5.85	-4.03	2.73E-03	3.51E-02
cg03355101		19	Intergenic	Open sea	14.92	4.71	18.95	6.24	-4.03	6.68E-04	1.27E-02
cg00142378		18	Intergenic	N_Shelf	71.56	6.36	75.58	4.82	-4.03	5.95E-04	1.16E-02
cg07783843		2	Intergenic	S_Shore	35.76	5.08	39.78	4.51	-4.03	7.14E-05	2.11E-03
cg07915884	KIF26B	1	Body	Open sea	25.76	4.45	29.78	6.17	-4.03	6.65E-04	1.26E-02
cg13847322	RXRA	9	Body	Open sea	68.34	3.79	72.37	3.06	-4.03	1.95E-06	8.38E-05
cg00210127	SNX29	16	Body	N_Shelf	45.40	4.52	49.42	5.68	-4.03	1.84E-03	2.67E-02
cg09980176	CCDC151	19	Body	N_Shore	25.63	4.77	29.66	6.12	-4.03	1.27E-03	2.04E-02
cg21197905	PPP2R1B	11	Body	Open sea	42.82	4.34	46.84	6.18	-4.03	3.45E-03	4.13E-02
cg12357534		13	Intergenic	Open sea	49.74	6.11	53.76	5.93	-4.02	3.62E-03	4.26E-02
cg19222480	UBE2C;UBE2C;UBE2C;UBE2C;UBE2C;U	20	TSS1500;1stExon;5'UTR;Body;Body;Body;E	Island	11.40	3.19	15.42	5.32	-4.02	5.26E-06	2.10E-04
cg14927519	CREB5;CREB5	7	TSS1500;5'UTR	S_Shore	28.79	3.78	32.82	3.94	-4.02	1.84E-05	6.50E-04
cg07513323	PLCG2	16	Body	Open sea	81.61	6.55	85.64	4.93	-4.02	1.54E-04	4.00E-03
cg13252152	SH2B2	7	Body	Open sea	35.70	4.36	39.72	5.39	-4.02	1.83E-04	4.60E-03
cg18645081	HLX	1	TSS1500	N_Shore	43.38	5.30	47.40	5.71	-4.02	2.09E-04	5.12E-03
cg10962762		1	Intergenic	Island	29.91	5.91	33.93	6.48	-4.02	1.90E-03	2.73E-02
cg03668078	C6orf103;LOC729176	6	Body;Body	Open sea	45.01	5.89	49.04	6.51	-4.02	4.24E-03	4.74E-02
cg23363484	SOX5	12	5'UTR	Open sea	68.36	4.90	72.39	4.17	-4.02	1.45E-05	5.25E-04
cg25421530	SORBS1;SORBS1;SORBS1;SORBS1;SOF	10	5'UTR;TSS200;TSS200;TSS200;5'UTR;TSS2(	Open sea	50.31	4.44	54.33	5.10	-4.02	3.23E-04	7.21E-03
cg07637516	MXI1;MXI1	10	Body;5'UTR	S_Shelf	65.31	5.82	69.34	5.24	-4.02	3.29E-04	7.32E-03
cg25026755		5	Intergenic	Open sea	49.96	5.26	53.98	6.55	-4.02	4.36E-03	4.84E-02
cg17824939	C12orf50	12	5'UTR	Open sea	62.76	7.00	66.78	6.26	-4.02	9.66E-04	1.67E-02
cg04162118	TRAM1	8	Body	N_Shore	66.34	5.77	70.36	4.72	-4.02	3.75E-04	8.10E-03
cg10003657	FRMD8	11	3'UTR	Open sea	60.61	3.80	64.63	2.94	-4.02	5.58E-07	2.59E-05
cg19943238	H19;MIR675	11	Body;TSS200	Island	64.76	3.14	68.78	4.66	-4.02	6.42E-06	2.51E-04
cg17052041		3	Intergenic	Open sea	81.04	4.77	85.06	4.01	-4.02	1.54E-04	4.00E-03
cg11983038		13	Intergenic	Open sea	23.93	4.84	27.95	6.12	-4.02	2.46E-03	3.27E-02
cg13019844		6	Intergenic	Island	47.07	3.80	51.09	4.85	-4.02	6.38E-05	1.92E-03
cg20782724	TNS3	7	5'UTR	Open sea	71.32	5.07	75.34	4.49	-4.02	3.38E-04	7.47E-03
cg00588621	EID3;TXNRD1;TXNRD1;TXNRD1;EID3;T	12	1stExon;5'UTR;5'UTR;Body;5'UTR;Body;5'U	Island	27.87	8.17	31.89	7.78	-4.02	1.40E-03	2.19E-02
cg02625623		13	Intergenic	S_Shelf	83.55	3.40	87.57	2.56	-4.02	1.73E-08	9.10E-07
cg00827989	SP9	2	Body	Island	19.66	3.99	23.68	4.40	-4.02	6.45E-07	2.97E-05
cg19645534		11	Intergenic	Open sea	44.57	4.41	48.59	5.79	-4.02	8.45E-04	1.51E-02
cg03671597	SEZ6;SEZ6	17	TSS1500;TSS1500	S_Shore	32.23	4.37	36.25	5.55	-4.02	4.80E-05	1.50E-03
cg02906874	CHST15	10	3'UTR	Open sea	76.25	5.56	80.26	5.56	-4.02	2.85E-04	6.54E-03
cg13194425	FGF11	17	TSS1500	N_Shore	67.52	4.87	71.54	4.46	-4.02	1.43E-04	3.75E-03
cg14354292		7	Intergenic	Open sea	54.87	6.16	58.89	5.75	-4.02	1.47E-03	2.28E-02
cg07961479	VSIG8;C1orf204	1	Body;TSS200	S_Shore	33.08	3.60	37.10	4.38	-4.02	2.27E-04	5.48E-03
cg04565067	GPR177;GPR177	1	TSS1500;TSS1500	S_Shore	15.13	4.28	19.14	4.90	-4.02	3.63E-05	1.18E-03
cg11070419	C4BPA	1	TSS1500	Open sea	29.79	5.00	33.81	5.11	-4.02	7.02E-04	1.31E-02
cg11469051		4	Intergenic	S_Shelf	67.53	6.34	71.55	5.50	-4.02	3.70E-03	4.33E-02
cg22443762	LHX9;LHX9	1	Body;Body	Island	46.12	5.04	50.14	6.24	-4.02	9.24E-04	1.61E-02
cg15443166		7	Intergenic	N_Shelf	67.76	5.16	71.78	4.65	-4.02	6.74E-05	2.01E-03
cg06850005		10	Intergenic	N_Shore	61.85	5.36	65.87	4.55	-4.02	6.93E-04	1.30E-02
cg08717402		2	Intergenic	Open sea	58.68	5.09	62.69	4.50	-4.02	1.94E-04	4.83E-03
cg10118513		14	Intergenic	Open sea	22.85	8.77	26.87	9.15	-4.02	4.22E-03	4.73E-02
cg18038361	TTR	18	TSS1500	Open sea	64.54	5.69	68.56	5.44	-4.02	1.68E-03	2.50E-02

cg00022900		8	Intergenic	Open sea	58.88	5.32	62.89	5.40	-4.02	2.11E-04	5.15E-03
cg19438860	CTNND2	5	Body	S_Shore	13.49	2.94	17.51	5.86	-4.02	4.82E-05	1.50E-03
cg01697865	IL10RA;IL10RA	11	TSS1500;TSS1500	N_Shore	51.82	4.62	55.84	5.93	-4.02	2.02E-03	2.85E-02
cg22719992		11	Intergenic	Open sea	68.81	6.10	72.83	4.51	-4.02	4.94E-04	1.00E-02
cg02508229	FAM46B	1	Body	N_Shore	69.73	4.82	73.74	4.58	-4.02	7.05E-04	1.32E-02
cg22713192	GAB2	11	Body	Open sea	79.79	4.37	83.81	3.45	-4.02	3.19E-05	1.05E-03
cg15920791		8	Intergenic	Island	21.79	4.43	25.81	5.51	-4.01	1.29E-03	2.06E-02
cg18716679	IDO2	8	Body	Open sea	55.96	5.26	59.98	3.66	-4.01	8.82E-05	2.52E-03
cg07204711	CDK3	17	Body	N_Shelf	79.52	4.46	83.53	3.91	-4.01	8.69E-06	3.32E-04
cg00087244		7	Intergenic	Open sea	36.71	5.48	40.72	5.22	-4.01	3.52E-03	4.18E-02
cg25199575	TMEM232	5	Body	Open sea	81.51	6.26	85.52	4.54	-4.01	6.32E-04	1.21E-02
cg04321753	GABBR1;GABBR1;GABBR1	6	Body;Body;Body	Open sea	71.49	6.39	75.50	4.63	-4.01	1.46E-03	2.26E-02
cg17078427	CLDN11	3	Body	Island	8.69	2.43	12.70	4.95	-4.01	1.01E-07	5.05E-06
cg11808677	USP5;USP5;CDCA3	12	TSS1500;TSS1500;Body	N_Shore	46.40	5.03	50.42	5.64	-4.01	1.49E-03	2.30E-02
cg19592898		6	Intergenic	Island	51.25	4.79	55.27	6.18	-4.01	2.21E-03	3.03E-02
cg12971867	MOV10L1;MOV10L1;MOV10L1	22	1stExon;TSS200;1stExon	Island	75.70	5.66	79.71	5.17	-4.01	4.54E-03	4.97E-02
cg22309696		2	Intergenic	Island	51.18	4.28	55.19	4.51	-4.01	2.55E-05	8.65E-04
cg00445997	TBXAS1;HIPK2;HIPK2;TBXAS1	7	5'UTR;TSS1500;TSS1500;5'UTR	Island	16.64	5.02	20.66	5.04	-4.01	1.55E-05	5.57E-04
cg17432513		5	Intergenic	N_Shore	34.55	6.12	38.57	5.75	-4.01	6.93E-04	1.30E-02
cg14400292	WNT7B	22	Body	Island	6.58	1.93	10.60	5.05	-4.01	4.70E-07	2.20E-05
cg26076428		3	Intergenic	S_Shore	21.94	3.54	25.95	3.39	-4.01	3.54E-07	1.68E-05
cg14180696	LRRC15;LRRC15	3	5'UTR;5'UTR	Open sea	53.27	4.05	57.28	4.94	-4.01	1.48E-05	5.37E-04
cg13565138	S1PR3	9	5'UTR	N_Shore	29.70	3.82	33.72	4.12	-4.01	1.53E-05	5.51E-04
cg08667275		11	Intergenic	N_Shore	34.34	6.00	38.35	5.79	-4.01	6.17E-04	1.19E-02
cg15148078	C19orf77	19	TSS200	N_Shore	32.46	4.33	36.48	4.94	-4.01	8.93E-04	1.57E-02
cg00060800		5	Intergenic	Open sea	41.78	6.30	45.79	5.90	-4.01	5.46E-04	1.08E-02
cg06623057		17	Intergenic	N_Shelf	64.85	3.37	68.86	3.19	-4.01	4.32E-07	2.04E-05
cg24002541		10	Intergenic	Open sea	63.26	4.59	67.27	4.47	-4.01	4.81E-04	9.83E-03
cg18431640	ZIC2	13	Body	Island	20.38	3.56	24.39	3.51	-4.01	8.23E-08	4.13E-06
cg18916060		14	Intergenic	S_Shore	58.31	5.19	62.32	6.04	-4.01	2.48E-03	3.29E-02
cg00698444	FAM155A	13	Body	Open sea	56.66	5.54	60.67	4.69	-4.01	4.72E-04	9.69E-03
cg26229201	LOC150568	2	TSS1500	Open sea	85.20	5.11	89.21	2.75	-4.01	3.72E-05	1.20E-03
cg00266647	WNK1;HSN2	12	Body;TSS1500	Open sea	59.99	4.59	64.00	5.46	-4.01	2.28E-04	5.49E-03
cg04532906	CNIH	14	Body	N_Shelf	72.15	4.64	76.16	5.38	-4.01	2.47E-03	3.28E-02
cg20090859		8	Intergenic	Open sea	55.24	5.11	59.25	4.90	-4.01	1.82E-03	2.65E-02
cg25921194	RNF169	11	Body	Open sea	83.08	4.71	87.09	3.45	-4.01	9.83E-06	3.71E-04
cg10069394		11	Intergenic	N_Shelf	57.82	6.19	61.83	4.91	-4.01	9.53E-04	1.65E-02
cg27061889	LASP1	17	Body	Open sea	42.52	5.53	46.53	5.97	-4.01	4.23E-03	4.74E-02
cg24659696		1	Intergenic	N_Shelf	70.06	4.48	74.07	3.27	-4.01	2.08E-05	7.23E-04
cg01907106	NEFH	22	TSS1500	N_Shore	38.53	5.11	42.54	4.18	-4.01	6.35E-04	1.22E-02
cg01610165	ZNF608	5	Body	Open sea	36.88	5.89	40.89	6.92	-4.01	1.50E-03	2.30E-02
cg23463608	GNG7	19	5'UTR	Island	38.18	4.58	42.19	3.87	-4.01	3.51E-05	1.14E-03
cg20929922	C5orf28	5	TSS1500	S_Shore	20.32	4.39	24.33	5.47	-4.01	3.20E-04	7.17E-03
cg17969789	SLC12A7	5	Body	Island	73.74	5.68	77.75	5.51	-4.01	1.44E-03	2.24E-02
cg07459121	SLC12A7	5	Body	N_Shore	53.51	4.59	57.52	3.81	-4.01	1.63E-04	4.19E-03
cg09590897	MIR637;DAPK3	19	TSS200;Body	S_Shore	66.63	4.56	70.64	4.21	-4.01	1.10E-04	3.03E-03
cg09071834		2	Intergenic	Open sea	58.30	6.71	62.31	5.61	-4.01	1.54E-03	2.35E-02
cg12214366	ANTXR2;ANTXR2	4	Body;Body	Open sea	71.22	5.03	75.22	5.16	-4.01	1.05E-04	2.93E-03
cg05081263	CARKD	13	Body	S_Shelf	79.73	6.26	83.74	8.17	-4.01	3.91E-03	4.50E-02
cg06369349	RUVBL2	19	Body	Open sea	32.43	5.28	36.44	5.34	-4.01	4.50E-04	9.34E-03
cg06874016	NKIRAS2;NKIRAS2;NKIRAS2;NKIRAS2;I	17	3'UTR;3'UTR;3'UTR;3'UTR;3'UTR	Open sea	50.89	5.34	54.90	5.82	-4.01	2.47E-04	5.85E-03
cg23781467	VARS	6	Body	N_Shelf	59.02	5.47	63.02	4.89	-4.01	1.43E-04	3.77E-03
cg02402209		4	Intergenic	S_Shelf	60.29	4.87	64.29	3.65	-4.01	8.18E-05	2.37E-03

cg13130398	RABGAP1L;RABGAP1L	1	TSS1500;Body	Open sea	41.18	5.83	45.18	4.89	-4.01	2.37E-03	3.18E-02
cg21283066		6	Intergenic	Open sea	39.45	6.83	43.46	7.07	-4.01	3.50E-03	4.17E-02
cg06604249	ABAT;ABAT;ABAT;ABAT	16	5'UTR;5'UTR;5'UTR;1stExon	Open sea	75.21	6.45	79.22	5.54	-4.01	8.28E-04	1.49E-02
cg19690486		19	Intergenic	S_Shelf	53.10	5.49	57.11	4.67	-4.01	2.05E-03	2.87E-02
cg27532621	PBX1	1	Body	Open sea	84.00	4.27	88.01	3.25	-4.01	2.64E-07	1.27E-05
cg08602008		19	Intergenic	Island	45.65	5.52	49.65	5.27	-4.01	1.10E-03	1.84E-02
cg20676972		7	Intergenic	Open sea	78.00	6.04	82.01	5.99	-4.00	2.10E-03	2.93E-02
cg18668813	MARCKS	6	TSS1500	N_Shelf	12.72	3.74	16.72	4.95	-4.00	1.74E-06	7.52E-05
cg02009395	ACSM1	16	TSS200	Open sea	71.76	5.47	75.76	4.14	-4.00	6.84E-05	2.03E-03
cg20691595		15	Intergenic	Open sea	40.12	6.34	44.13	5.46	-4.00	4.57E-03	5.00E-02
cg08773226		6	Intergenic	Open sea	66.07	5.28	70.07	5.79	-4.00	1.18E-03	1.94E-02
cg02607544	IFFO1;IFFO1;IFFO1	12	Body;Body;Body	S_Shore	47.46	6.49	51.47	6.72	-4.00	8.79E-04	1.56E-02
cg13572369	DLG2;DLG2;DLG2;DLG2	11	5'UTR;Body;Body;Body	Open sea	74.75	4.42	78.76	3.66	-4.00	5.37E-06	2.14E-04
cg20786074	EFEMP1;EFEMP1;EFEMP1;EFEMP1	2	1stExon;5'UTR;5'UTR;5'UTR	N_Shore	26.97	4.51	30.97	5.44	-4.00	1.02E-04	2.85E-03
cg11725101	NBPF16;NBPF16	1	1stExon;5'UTR	Open sea	54.34	3.24	58.34	3.01	-4.00	2.71E-06	1.13E-04
cg01393327	OSTBETA	15	5'UTR	Open sea	53.44	6.22	57.45	7.04	-4.00	1.09E-03	1.82E-02
cg03819089		2	Intergenic	Open sea	63.80	5.07	67.80	5.78	-4.00	4.48E-03	4.93E-02
cg00349404		10	Intergenic	S_Shore	27.85	4.93	31.85	5.63	-4.00	1.02E-03	1.73E-02
cg18912541	DCAF5	14	Body	Open sea	32.90	3.80	36.90	4.20	-4.00	4.28E-05	1.36E-03
cg09263647		14	Intergenic	Open sea	47.79	5.77	51.79	6.24	-4.00	3.54E-03	4.20E-02
cg19463471	C19orf66	19	Body	Island	17.76	3.34	21.76	6.00	-4.00	3.99E-04	8.50E-03
cg05620095		3	Intergenic	Open sea	83.74	3.99	87.74	3.47	-4.00	2.74E-06	1.15E-04
cg05548945	LPP;LPP;LPP	3	Body;Body;Body	Open sea	70.67	5.49	74.67	4.15	-4.00	3.56E-05	1.16E-03
cg02280226	ASPSCR1	17	Body	N_Shore	71.04	4.24	75.04	4.06	-4.00	6.23E-05	1.88E-03
cg01075271		14	Intergenic	Island	33.61	5.34	37.61	5.36	-4.00	1.89E-04	4.73E-03
cg22822803	WDR8	1	TSS1500	Island	51.60	4.67	55.60	4.91	-4.00	1.33E-05	4.87E-04
cg13016916	CREB3L2	7	Body	Open sea	78.66	7.94	82.66	3.71	-4.00	1.24E-03	2.01E-02
cg17912835		2	Intergenic	Island	30.53	4.01	34.52	4.78	-4.00	2.33E-05	8.01E-04
cg25216696	GATA4	8	5'UTR	Island	44.03	3.74	48.03	3.93	-4.00	3.21E-06	1.33E-04
cg17692429	WDR86	7	Body	Island	32.91	5.32	36.91	6.26	-4.00	4.98E-05	1.55E-03
cg27031484	UBE2H;UBE2H	7	Body;Body	Open sea	83.50	3.60	87.50	3.08	-4.00	1.56E-07	7.69E-06
cg23698269	SBF2	11	TSS1500	S_Shore	49.16	6.19	53.16	4.64	-4.00	5.72E-04	1.12E-02
cg04084026	KIFC3;KIFC3;KIFC3	16	TSS1500;5'UTR;5'UTR	N_Shelf	47.67	4.39	51.67	4.72	-4.00	2.56E-04	6.02E-03
cg06968926	MAPK8IP3;MAPK8IP3	16	Body;Body	S_Shore	76.00	4.07	80.00	3.88	-4.00	2.13E-06	9.08E-05
cg01721410	FAM96B;FAM96B	16	Body;3'UTR	N_Shore	50.38	4.54	54.38	5.48	-4.00	1.90E-03	2.73E-02
cg07537699	LY6G5C	6	Body	N_Shelf	60.90	4.16	64.90	3.98	-4.00	6.58E-05	1.97E-03
cg00115102	LOC96610	22	TSS200	Open sea	50.15	1.74	54.15	2.16	-4.00	1.21E-11	7.18E-10
cg19682367	TPRG1	3	TSS1500	Open sea	25.37	4.48	29.37	6.01	-4.00	2.17E-03	2.99E-02
cg19714308		18	Intergenic	N_Shelf	78.10	4.54	82.10	3.10	-4.00	7.62E-06	2.94E-04
cg04083076	SLC19A2	1	Body	N_Shelf	67.24	6.25	71.24	4.78	-4.00	3.32E-03	4.01E-02
cg15415673		14	Intergenic	Open sea	68.91	5.10	72.91	5.16	-4.00	4.00E-03	4.57E-02
cg02363677	SVIL;SVIL	10	Body;Body	Open sea	65.52	5.16	69.52	5.21	-4.00	1.76E-03	2.58E-02
cg07596401	LHPP;LHPP	10	TSS1500;TSS1500	N_Shore	36.54	5.29	40.54	5.14	-4.00	2.81E-03	3.58E-02
cg02067994		5	Intergenic	Open sea	75.57	3.58	79.57	3.56	-4.00	1.64E-06	7.15E-05
cg26594205	OGFRL1	6	Body	S_Shelf	79.48	5.38	83.48	4.84	-3.99	1.96E-03	2.79E-02
cg15355146	MYCL1;MYCL1;MYCL1	1	TSS1500;TSS1500;TSS1500	S_Shore	40.31	5.13	44.30	5.56	-3.99	5.39E-05	1.66E-03
cg06455149		8	Intergenic	Island	44.10	4.68	48.10	5.37	-3.99	9.75E-04	1.68E-02
cg17161718	COL11A2;COL11A2;COL11A2;COL11A2	6	5'UTR;5'UTR;1stExon;1stExon;5'UTR;1stEx	Island	34.00	5.02	38.00	5.96	-3.99	1.57E-03	2.37E-02
cg00535994		2	Intergenic	Open sea	54.83	3.66	58.82	3.61	-3.99	1.20E-06	5.34E-05
cg15637765	TRIO	5	Body	S_Shore	21.52	4.76	25.51	4.20	-3.99	7.85E-05	2.28E-03
cg22045288	C10orf91	10	TSS1500	Open sea	49.62	4.29	53.61	3.89	-3.99	1.50E-04	3.91E-03
cg11410718	HOXA4	7	TSS200	Island	12.71	3.81	16.71	4.47	-3.99	4.02E-05	1.29E-03
cg00282877	LOC619207	10	TSS1500	Open sea	61.55	4.98	65.54	3.91	-3.99	2.79E-04	6.43E-03

cg22000727		6	Intergenic	S_Shore	46.81	4.87	50.81	4.83	-3.99	2.75E-04	6.36E-03
cg23414876	HSF4;HSF4;HSF4;HSF4;FBXL8	16	5'UTR;1stExon;1stExon;5'UTR;Body	Island	25.80	5.33	29.80	5.71	-3.99	6.04E-04	1.17E-02
cg04334016	CNKSR3	6	Body	Open sea	31.56	4.56	35.55	4.11	-3.99	5.83E-05	1.77E-03
cg22463915	EEF1A2	20	TSS1500	S_Shelf	45.73	5.39	49.72	5.94	-3.99	1.20E-03	1.97E-02
cg18097224	TCF7;TCF7;TCF7;TCF7;TCF7;TCF7;TCF7	5	TSS200;TSS1500;TSS1500;TSS200;Body;TS	Island	12.08	5.18	16.07	5.92	-3.99	2.26E-05	7.79E-04
cg20799176		11	Intergenic	Open sea	22.13	5.29	26.13	5.76	-3.99	4.40E-04	9.18E-03
cg07730329	PCDHGA4;PCDHGA11;PCDHGA11;PCD	5	Body;Body;Body;Body;Body;Body;Body;TS	N_Shore	24.09	6.33	28.08	6.97	-3.99	2.13E-03	2.96E-02
cg25426203	BMPR1A	10	5'UTR	Open sea	77.43	5.14	81.42	4.25	-3.99	1.64E-04	4.21E-03
cg03850198		1	Intergenic	Open sea	58.30	4.93	62.29	5.53	-3.99	1.21E-03	1.97E-02
cg06289138	C6orf227	6	TSS200	Island	38.45	4.69	42.44	5.66	-3.99	1.07E-03	1.80E-02
cg13431464	PTPN2;PTPN2;PTPN2	18	Body;Body;Body	N_Shelf	37.07	3.59	41.07	3.81	-3.99	1.76E-06	7.61E-05
cg19619036		19	Intergenic	N_Shelf	79.42	3.21	83.41	3.13	-3.99	1.93E-07	9.42E-06
cg02330371		15	Intergenic	Island	55.17	4.74	59.16	4.83	-3.99	1.53E-03	2.33E-02
cg03740978	KCTD1;KCTD1;KCTD1	18	5'UTR;5'UTR;1stExon	Island	11.94	3.49	15.93	4.50	-3.99	4.88E-07	2.28E-05
cg04223044	C22orf9;C22orf9	22	Body;TSS200	Open sea	61.82	5.19	65.81	4.83	-3.99	5.57E-05	1.71E-03
cg24596116		12	Intergenic	Open sea	37.87	4.09	41.86	4.28	-3.99	1.56E-04	4.04E-03
cg08128650	RELA;RELA	11	Body;Body	N_Shelf	61.61	3.83	65.61	2.95	-3.99	1.15E-05	4.27E-04
cg25652610	TNFAIP8L1;TNFAIP8L1	19	5'UTR;5'UTR	N_Shelf	58.25	3.73	62.24	3.26	-3.99	2.25E-05	7.77E-04
cg10598711	TMEM174	5	3'UTR	Open sea	48.93	5.31	52.93	4.80	-3.99	2.37E-05	8.14E-04
cg23146197	HMGA2;HMGA2	12	Body;Body	Open sea	55.96	4.26	59.95	4.57	-3.99	1.53E-04	3.97E-03
cg05620046		6	Intergenic	Open sea	55.65	4.96	59.64	6.56	-3.99	4.47E-03	4.92E-02
cg12973803		2	Intergenic	Open sea	41.33	5.23	45.32	5.46	-3.99	1.42E-03	2.21E-02
cg26740494	MMP23A;MMP23B	1	TSS1500;TSS1500	Island	49.77	4.54	53.77	4.66	-3.99	7.06E-04	1.32E-02
cg01642579	ANKS3	16	3'UTR	S_Shelf	83.78	4.04	87.77	3.60	-3.99	1.82E-06	7.87E-05
cg11015497		2	Intergenic	Open sea	71.61	6.74	75.60	5.07	-3.99	1.06E-03	1.78E-02
cg26992603		12	Intergenic	S_Shore	17.46	6.63	21.45	7.17	-3.99	9.86E-04	1.69E-02
cg24700494	MDC1	6	5'UTR	N_Shore	56.59	5.19	60.58	5.34	-3.99	3.05E-03	3.79E-02
cg15539420	HOXB8;HOXB8	17	5'UTR;1stExon	S_Shore	34.05	4.47	38.04	5.16	-3.99	2.59E-04	6.07E-03
cg10029177		4	Intergenic	Open sea	39.36	5.01	43.35	4.95	-3.99	1.31E-03	2.09E-02
cg27205904	TXNRD1;TXNRD1;TXNRD1;EID3;TXNRI	12	5'UTR;5'UTR;Body;TSS200;Body;5'UTR	Island	28.81	6.15	32.80	5.73	-3.99	4.17E-04	8.79E-03
cg20386303		2	Intergenic	Open sea	53.52	4.37	57.50	5.21	-3.99	2.78E-04	6.41E-03
cg20546296	LVRN	5	3'UTR	Open sea	79.18	3.86	83.17	3.56	-3.99	2.30E-07	1.11E-05
cg12217400		6	Intergenic	Island	27.57	6.13	31.55	5.22	-3.99	2.12E-03	2.94E-02
cg13855454		12	Intergenic	Open sea	62.34	4.85	66.33	3.79	-3.99	2.18E-04	5.29E-03
cg13777287	CNKSR3	6	Body	N_Shore	25.92	4.23	29.91	4.90	-3.99	7.82E-04	1.42E-02
cg27129725	CELSR1	22	Body	Island	74.79	3.73	78.78	4.00	-3.99	3.44E-06	1.42E-04
cg20931867		4	Intergenic	S_Shelf	76.72	3.90	80.71	2.92	-3.99	3.65E-06	1.50E-04
cg13916255	CALCR;CALCR;CALCR	7	5'UTR;5'UTR;TSS1500	Open sea	74.10	4.14	78.09	3.68	-3.99	1.54E-06	6.74E-05
cg01477361		2	Intergenic	Open sea	54.71	5.81	58.70	5.96	-3.99	1.66E-03	2.48E-02
cg08225986	PATZ1;PATZ1;PATZ1;PATZ1	22	Body;Body;Body;Body	N_Shelf	45.22	3.99	49.21	3.62	-3.99	2.35E-06	9.95E-05
cg22649349	FAM50B	6	TSS1500	N_Shore	50.40	4.83	54.39	4.90	-3.99	1.87E-03	2.70E-02
cg23080769	FMO1	1	TSS1500	Open sea	62.97	5.94	66.95	5.29	-3.99	4.98E-04	1.01E-02
cg26190835		2	Intergenic	Open sea	45.14	4.60	49.13	4.39	-3.99	5.29E-05	1.63E-03
cg20635409		12	Intergenic	Island	12.15	2.63	16.14	2.10	-3.99	5.53E-11	3.21E-09
cg09121543	LIMD2	17	3'UTR	N_Shelf	22.61	3.59	26.60	4.25	-3.99	5.07E-06	2.03E-04
cg01572694	MIR10A	17	TSS1500	N_Shore	41.68	4.52	45.67	4.61	-3.99	3.91E-04	8.37E-03
cg08938155	TBCA	5	Body	Open sea	61.92	4.91	65.91	3.99	-3.99	4.69E-04	9.65E-03
cg09426825	WDFY2	13	Body	Open sea	27.67	4.33	31.66	5.66	-3.99	1.45E-03	2.25E-02
cg27445347	FAM50B	6	5'UTR	Island	46.76	5.12	50.74	4.67	-3.99	1.38E-03	2.17E-02
cg05543520	LRIG1	3	Body	Open sea	59.97	4.58	63.96	4.03	-3.99	1.81E-04	4.56E-03
cg14824495	SOX15	17	1stExon	Island	29.40	5.45	33.39	4.99	-3.99	1.03E-03	1.75E-02
cg12423278		5	Intergenic	Open sea	75.22	5.20	79.21	6.74	-3.99	3.85E-03	4.45E-02
cg01522553	KCNC4;KCNC4	1	Body;3'UTR	Open sea	60.69	4.43	64.67	5.69	-3.99	5.39E-04	1.07E-02

cg04699519	ATXN1;ATXN1	6	3'UTR;3'UTR	Open sea	78.61	4.84	82.59	3.75	-3.99	1.38E-04	3.64E-03
cg17083209	LPHN2	1	5'UTR	Open sea	19.08	4.89	23.07	5.25	-3.99	1.07E-03	1.80E-02
cg19988566	ZRANB1	10	3'UTR	Open sea	74.65	5.18	78.63	6.32	-3.99	3.52E-03	4.18E-02
cg07751331	KIF3C	2	TSS1500	S_Shore	55.83	4.08	59.81	3.88	-3.99	6.47E-05	1.94E-03
cg10796749	RBP7	1	TSS1500	N_Shore	47.79	4.58	51.77	4.67	-3.98	1.62E-04	4.18E-03
cg26511321	HOXA7	7	TSS1500	N_Shore	34.34	4.56	38.32	5.84	-3.98	8.75E-05	2.50E-03
cg17559156	SLC43A1	11	Body	S_Shelf	40.81	5.10	44.79	6.34	-3.98	4.13E-03	4.67E-02
cg20448053	SLC25A35;RANGRF	17	3'UTR;Body	Island	9.69	3.96	13.68	5.57	-3.98	1.97E-04	4.89E-03
cg16837557	KIRREL3;KIRREL3	11	Body;Body	Open sea	43.09	5.44	47.07	5.44	-3.98	3.68E-03	4.31E-02
cg06379340	UNC80;UNC80	2	3'UTR;3'UTR	N_Shelf	65.73	5.30	69.72	5.30	-3.98	3.58E-04	7.81E-03
cg10117884		1	Intergenic	N_Shelf	79.37	5.78	83.35	4.24	-3.98	1.00E-03	1.71E-02
cg00032701	EVI5L;EVI5L	19	5'UTR;TSS200	N_Shore	80.62	5.18	84.60	3.59	-3.98	3.93E-04	8.40E-03
cg04484415	NDRG4;NDRG4;NDRG4	16	Body;Body;Body	N_Shore	61.41	6.79	65.39	6.76	-3.98	3.83E-03	4.43E-02
cg02823132	FAM38A	16	Body	S_Shore	67.55	4.24	71.53	4.31	-3.98	1.18E-04	3.20E-03
cg09379229	APOC1	19	TSS1500	Open sea	65.48	5.33	69.46	4.87	-3.98	2.74E-03	3.52E-02
cg21604803	CPT1C;CPT1C	19	5'UTR;5'UTR	Island	23.93	4.56	27.92	5.93	-3.98	3.67E-04	7.96E-03
cg01827999	TOM1L2;TOM1L2	17	Body;Body	Open sea	51.85	3.65	55.83	3.58	-3.98	2.20E-05	7.60E-04
cg26191586	LRP8;LRP8;LRP8;LRP8	1	TSS1500;TSS1500;TSS1500;TSS1500	S_Shore	65.36	4.80	69.34	3.89	-3.98	1.13E-04	3.09E-03
cg12368542	TRIM13;TRIM13;TRIM13;TRIM13;DLE	13	TSS1500;TSS1500;TSS1500;TSS1500;Body	N_Shore	49.17	6.99	53.15	6.18	-3.98	1.49E-03	2.29E-02
cg01744217	C20orf108	20	TSS1500	N_Shore	19.81	4.26	23.79	4.16	-3.98	4.43E-05	1.40E-03
cg16426714		17	Intergenic	Island	23.83	3.95	27.81	4.52	-3.98	1.75E-06	7.57E-05
cg01591591	CREBBP;CREBBP	16	Body;Body	Open sea	55.00	6.91	58.98	6.83	-3.98	1.05E-03	1.78E-02
cg01970407	AHRR	5	Body	S_Shore	60.91	5.46	64.89	6.67	-3.98	4.37E-03	4.85E-02
cg04304450	BIK	22	3'UTR	Open sea	52.89	6.65	56.87	6.10	-3.98	1.39E-03	2.18E-02
cg23283614	DHRS7	14	TSS1500	S_Shore	27.37	7.39	31.35	7.60	-3.98	4.37E-03	4.85E-02
cg06824423	ARID1B;ARID1B;ARID1B	6	Body;Body;Body	Open sea	23.70	4.22	27.68	4.69	-3.98	6.83E-04	1.29E-02
cg17913386	COL7A1	3	1stExon	Island	19.74	4.80	23.72	5.61	-3.98	3.09E-04	6.96E-03
cg03663788	PYGM;PYGM	11	Body;Body	S_Shore	82.79	4.72	86.77	2.77	-3.98	5.27E-06	2.10E-04
cg15442583	CDGAP	3	Body	Open sea	54.74	4.04	58.72	5.62	-3.98	6.47E-04	1.23E-02
cg22746681		10	Intergenic	Island	12.67	3.13	16.65	4.97	-3.98	4.73E-05	1.48E-03
cg16742481		4	Intergenic	Open sea	45.64	5.10	49.62	4.90	-3.98	2.03E-04	5.00E-03
cg14650464	FANCC	9	Body	Open sea	4.43	1.38	8.40	3.23	-3.98	2.69E-13	1.67E-11
cg10180052	RNF151	16	Body	Island	66.06	6.42	70.04	7.18	-3.98	2.60E-03	3.39E-02
cg02300379	LGI1	10	Body	Open sea	62.16	4.31	66.14	4.76	-3.98	2.94E-05	9.80E-04
cg02376827	HIC1;HIC1	17	Body;Body	Island	41.54	4.03	45.52	4.96	-3.98	2.48E-04	5.86E-03
cg14178533	VAV2;VAV2	9	Body;Body	Island	70.33	3.08	74.31	3.30	-3.98	5.11E-07	2.38E-05
cg00014998	HOXD4;MIR10B	2	TSS1500;TSS200	Island	23.79	5.19	27.77	5.41	-3.98	2.90E-03	3.65E-02
cg25065716	ADARB2	10	Body	Open sea	46.78	5.55	50.76	7.39	-3.98	4.25E-03	4.76E-02
cg01426921	RMRP;CCDC107	9	TSS1500;Body	Island	22.61	4.64	26.59	5.13	-3.98	1.32E-03	2.10E-02
cg21818470		2	Intergenic	N_Shore	64.51	6.63	68.48	5.79	-3.98	3.73E-03	4.35E-02
cg23478547		11	Intergenic	S_Shore	70.20	5.95	74.18	5.45	-3.98	3.99E-03	4.56E-02
cg15067606	NAA50	3	Body	Open sea	75.40	4.30	79.38	4.36	-3.98	1.31E-04	3.50E-03
cg13784283	RXRB	6	Body	S_Shelf	28.67	4.58	32.65	5.37	-3.98	1.98E-03	2.81E-02
cg05860956	SLC6A12;SLC6A12;SLC6A12	12	Body;Body;Body	Island	52.01	5.95	55.98	6.37	-3.98	6.29E-04	1.21E-02
cg20934881	ATP8B2;ATP8B2	1	Body;Body	Island	22.56	4.05	26.54	4.46	-3.97	2.06E-05	7.16E-04
cg08571859	APC;APC;APC	5	TSS1500;5'UTR;TSS1500	Open sea	18.95	6.90	22.92	8.19	-3.97	3.09E-04	6.96E-03
cg25470148	RUNX3;RUNX3	1	TSS1500;Body	Island	23.68	4.76	27.65	4.26	-3.97	7.64E-04	1.40E-02
cg14375496	VPS53;VPS53	17	Body;Body	Open sea	76.79	6.30	80.77	4.74	-3.97	3.12E-03	3.85E-02
cg06568323	PCCA;PCCA	13	Body;Body	S_Shelf	45.29	4.71	49.26	6.07	-3.97	3.62E-03	4.26E-02
cg16817891	IGF2AS;INS-IGF2;IGF2;IGF2AS	11	Body;Body;5'UTR;Body	S_Shore	49.57	4.03	53.55	4.83	-3.97	2.96E-04	6.73E-03
cg20489317	AMY2B;LOC648740	1	5'UTR;TSS200	Open sea	74.20	4.91	78.18	3.87	-3.97	1.17E-04	3.18E-03
cg02485697	TSSC4	11	Body	S_Shore	75.52	4.36	79.50	4.14	-3.97	1.77E-05	6.26E-04
cg03367275		10	Intergenic	S_Shelf	80.01	4.91	83.98	4.10	-3.97	3.36E-04	7.44E-03

cg08754268	RAPGEF4;RAPGEF4	2	Body;Body	Open sea	71.00	5.78	74.97	4.67	-3.97	1.72E-03	2.54E-02
cg23948240		12	Intergenic	N_Shore	8.89	2.46	12.87	2.58	-3.97	4.83E-10	2.73E-08
cg14424407	SRCIN1	17	Body	Island	44.42	2.94	48.39	3.54	-3.97	9.47E-07	4.27E-05
cg03437912	POMP	13	TSS1500	N_Shore	20.35	3.99	24.33	5.46	-3.97	2.58E-04	6.04E-03
cg18793806	ZNF514	2	TSS1500	S_Shore	47.51	5.19	51.48	4.88	-3.97	1.35E-03	2.14E-02
cg19026977		5	Intergenic	Open sea	37.25	3.82	41.23	3.62	-3.97	5.23E-05	1.61E-03
cg27178401	EMP2	16	5'UTR	Open sea	34.59	4.57	38.56	4.58	-3.97	1.55E-03	2.36E-02
cg18862597	CROCC	1	Body	Open sea	65.39	6.83	69.36	5.62	-3.97	3.16E-03	3.88E-02
cg14018233		19	Intergenic	Island	58.92	5.96	62.89	6.52	-3.97	2.33E-03	3.14E-02
cg07348922	B3GALT4;B3GALT4	6	1stExon;5'UTR	Island	41.11	6.12	45.08	4.22	-3.97	5.28E-04	1.06E-02
cg15635633		2	Intergenic	Open sea	71.91	4.86	75.88	3.93	-3.97	4.42E-04	9.21E-03
cg15460093	ADAM8;ADAM8;ADAM8;ADAM8;ADA	10	1stExon;1stExon;5'UTR;1stExon;5'UTR;5'U	Island	33.21	6.98	37.18	6.62	-3.97	2.37E-03	3.19E-02
cg09744051	A4GALT	22	5'UTR	N_Shore	24.96	4.28	28.93	5.93	-3.97	7.86E-04	1.43E-02
cg02807271		19	Intergenic	S_Shelf	79.58	3.60	83.55	3.25	-3.97	1.48E-06	6.49E-05
cg10579941		1	Intergenic	Open sea	46.37	5.07	50.34	5.04	-3.97	2.52E-03	3.32E-02
cg16595607	OTX1	2	5'UTR	N_Shore	33.88	4.01	37.85	5.84	-3.97	2.76E-04	6.37E-03
cg13459493		9	Intergenic	S_Shore	76.60	5.56	80.57	4.13	-3.97	4.14E-04	8.76E-03
cg06861736	MED15;MED15	22	Body;Body	Open sea	35.86	3.31	39.83	4.34	-3.97	1.11E-05	4.15E-04
cg20366633	TAP2;TAP2	6	Body;Body	Open sea	65.02	3.91	68.99	5.05	-3.97	1.31E-04	3.49E-03
cg11788367	ACVRL1;ACVRL1	12	Body;Body	Island	76.72	4.36	80.69	3.49	-3.97	2.85E-05	9.53E-04
cg15133208	SNCA;SNCA;SNCA;SNCA	4	5'UTR;5'UTR;5'UTR;5'UTR	N_Shore	27.58	5.43	31.54	5.38	-3.97	1.09E-03	1.82E-02
cg17965684		7	Intergenic	Open sea	80.79	6.28	84.76	5.62	-3.97	1.85E-04	4.64E-03
cg09522706	MCM7;MCM7;MIR25;MIR93	7	Body;Body;TSS200;Body	Open sea	28.10	4.04	32.07	4.90	-3.97	5.33E-04	1.06E-02
cg23736754	EXT1	8	Body	Open sea	70.13	4.48	74.10	4.63	-3.97	3.66E-06	1.50E-04
cg05462761	PLEKHG5;PLEKHG5;PLEKHG5;PLEKHG5	1	5'UTR;5'UTR;Body;5'UTR;Body	Island	33.71	4.24	37.68	4.73	-3.97	1.05E-04	2.92E-03
cg08234178		7	Intergenic	N_Shelf	62.06	3.29	66.02	4.01	-3.97	1.02E-05	3.84E-04
cg01385312	TNRC18	7	Body	N_Shore	65.76	6.98	69.73	6.22	-3.97	4.54E-03	4.97E-02
cg09581226	PCDHGA4;PCDHGA12;PCDHGA11;PCD	5	Body;Body;Body;Body;Body;Body;Body;Bo	N_Shore	61.54	5.94	65.51	5.87	-3.97	3.03E-03	3.77E-02
cg17093877	MGC16275	17	Body	N_Shelf	53.09	3.79	57.06	4.10	-3.97	1.84E-05	6.49E-04
cg16554099		6	Intergenic	S_Shore	52.23	5.25	56.20	6.23	-3.97	3.43E-03	4.11E-02
cg15938050		17	Intergenic	Open sea	38.88	5.93	42.85	5.81	-3.97	2.17E-03	2.99E-02
cg22748470	SLC25A2	5	TSS1500	S_Shore	68.30	4.96	72.26	4.70	-3.97	1.16E-04	3.16E-03
cg20938346	SIM1	6	Body	Open sea	46.50	6.94	50.47	6.19	-3.97	6.52E-04	1.24E-02
cg12743248	EFCAB1;EFCAB1;EFCAB1	8	3'UTR;3'UTR;Body	Open sea	58.65	4.92	62.61	3.97	-3.97	7.66E-05	2.24E-03
cg07622096	C2orf48	2	TSS1500	Open sea	78.92	5.47	82.89	4.52	-3.97	2.85E-03	3.61E-02
cg15035382	CNTD2	19	3'UTR	Island	78.89	4.30	82.86	3.37	-3.97	1.26E-05	4.62E-04
cg16017420		12	Intergenic	S_Shelf	33.68	4.71	37.65	6.19	-3.97	2.60E-03	3.40E-02
cg26434048	IGFBP3;IGFBP3	7	TSS1500;TSS1500	S_Shore	37.31	4.88	41.27	6.03	-3.97	2.54E-04	5.97E-03
cg22719871	BRMS1;BRMS1;RIN1	11	3'UTR;Body;TSS1500	S_Shelf	73.35	4.66	77.31	3.41	-3.97	1.21E-05	4.48E-04
cg26884581	PYGM;PYGM;PYGM;PYGM	11	1stExon;1stExon;5'UTR;5'UTR	Open sea	61.34	4.64	65.31	3.56	-3.97	1.02E-04	2.86E-03
cg16848873	LOC404266;LOC404266;HOXB6;HOXB6	17	Body;Body;1stExon;5'UTR	N_Shelf	24.51	4.65	28.48	5.46	-3.97	1.60E-03	2.41E-02
cg19083133		12	Intergenic	Open sea	72.38	5.74	76.34	4.18	-3.96	6.57E-04	1.25E-02
cg08550517		16	Intergenic	Open sea	69.75	6.40	73.71	4.36	-3.96	7.34E-04	1.36E-02
cg08123207	PRRT1	6	Body	Island	49.36	5.18	53.33	5.42	-3.96	4.26E-03	4.76E-02
cg04270085		2	Intergenic	Open sea	63.28	5.34	67.25	6.16	-3.96	2.54E-03	3.33E-02
cg26264697	HMG20B	19	Body	Island	87.54	5.99	91.50	3.30	-3.96	9.97E-05	2.79E-03
cg01054354	LYSMD4	15	5'UTR	N_Shore	19.62	5.27	23.58	4.60	-3.96	1.01E-03	1.72E-02
cg02268674	ESYT1	12	Body	S_Shore	50.19	4.45	54.16	4.70	-3.96	2.38E-04	5.68E-03
cg13270350	HDAC4	2	Body	Open sea	84.85	4.79	88.81	3.49	-3.96	4.92E-05	1.53E-03
cg19568544	PCDHB5;PCDHB5	5	1stExon;5'UTR	N_Shore	22.62	4.46	26.58	5.00	-3.96	2.50E-04	5.90E-03
cg18786171	ADK;ADK	10	Body;TSS1500	N_Shore	52.88	5.31	56.84	5.95	-3.96	6.59E-04	1.25E-02
cg14171516	MORN1	1	Body	N_Shore	67.83	3.89	71.79	4.33	-3.96	1.46E-05	5.29E-04
cg00986598	TMCC1;TMCC1;TMCC1	3	3'UTR;3'UTR;3'UTR	Open sea	68.52	4.68	72.48	5.28	-3.96	3.16E-04	7.09E-03

cg24759064	BBS10	12	3'UTR	N_Shelf	80.11	5.14	84.07	5.24	-3.96	4.37E-04	9.13E-03
cg17329287	RNF220	1	TSS1500	N_Shore	30.93	4.10	34.89	5.05	-3.96	1.01E-04	2.82E-03
cg06684503	MLLT6	17	Body	Open sea	65.92	5.00	69.88	5.48	-3.96	6.34E-04	1.22E-02
cg05961962		16	Intergenic	Open sea	75.26	3.84	79.22	3.38	-3.96	1.60E-06	6.97E-05
cg06941429		12	Intergenic	Open sea	66.64	4.69	70.60	4.22	-3.96	1.29E-04	3.46E-03
cg18864561		5	Intergenic	Open sea	63.17	4.68	67.13	4.55	-3.96	8.99E-05	2.56E-03
cg10135717	ADAMTS17	15	TSS1500	S_Shore	36.97	3.87	40.93	5.56	-3.96	5.95E-04	1.16E-02
cg12990614	SOX10	22	Body	Island	55.66	4.99	59.62	5.49	-3.96	3.32E-04	7.37E-03
cg27109284	NPEPPS	17	Body	Open sea	50.60	5.09	54.56	5.87	-3.96	1.64E-03	2.45E-02
cg23990435	CXXC5	5	5'UTR	S_Shelf	61.27	4.41	65.23	5.81	-3.96	3.21E-03	3.92E-02
cg11485451	TCF7;TCF7;TCF7;TCF7;TCF7;TCF7;TCF7	5	TSS200;TSS1500;TSS1500;TSS200;Body;TS	Island	13.01	5.45	16.96	6.24	-3.96	8.08E-05	2.34E-03
cg24940230		11	Intergenic	Open sea	63.15	5.17	67.11	4.54	-3.96	9.27E-05	2.63E-03
cg02172819	KCNMA1;KCNMA1;KCNMA1;KCNMA1	10	Body;Body;Body;Body	Open sea	47.50	5.09	51.46	4.39	-3.96	5.59E-04	1.10E-02
cg19142026	HOXA4;HOXA4	7	5'UTR;1stExon	Island	10.47	2.62	14.43	3.89	-3.96	1.27E-08	6.74E-07
cg02843417	IRF8	16	Body	Open sea	88.22	5.41	92.17	2.88	-3.96	2.84E-05	9.49E-04
cg03837313	SLC43A3;SLC43A3;SLC43A3	11	TSS1500;TSS1500;TSS1500	S_Shore	29.73	4.59	33.69	5.37	-3.96	1.51E-03	2.32E-02
cg10824810	IRX5	16	Body	Island	47.43	5.30	51.39	4.52	-3.96	9.71E-04	1.67E-02
cg14249348	ZAR1	4	TSS200	Island	34.25	5.61	38.21	6.58	-3.96	1.17E-03	1.93E-02
cg06928695	PITPNM3;PITPNM3	17	Body;Body	Open sea	65.81	5.42	69.76	5.26	-3.96	4.34E-04	9.09E-03
cg23767994	DLX6AS	7	Body	Open sea	28.95	4.10	32.91	5.04	-3.96	2.83E-04	6.50E-03
cg23405351		7	Intergenic	Open sea	85.53	4.14	89.49	3.62	-3.96	2.52E-05	8.58E-04
cg16669339	SH3PXD2A	10	Body	Island	46.53	4.13	50.49	4.67	-3.96	1.37E-05	5.01E-04
cg14003931	GPR123	10	Body	Island	32.46	5.24	36.42	5.76	-3.96	1.39E-03	2.18E-02
cg18590502	CCDC71	3	5'UTR	N_Shore	34.91	4.13	38.87	4.39	-3.96	7.33E-05	2.16E-03
cg12407070		3	Intergenic	Open sea	70.59	5.10	74.55	5.21	-3.96	1.02E-05	3.83E-04
cg11408952	RB1	13	Body	N_Shore	64.67	4.74	68.63	5.21	-3.96	4.96E-04	1.01E-02
cg18625610	ZFR2	19	Body	N_Shore	48.67	5.70	52.63	2.92	-3.96	6.76E-05	2.01E-03
cg09290655		6	Intergenic	Open sea	70.10	4.63	74.06	4.93	-3.96	3.79E-04	8.16E-03
cg00396667	PITX1	5	3'UTR	Island	17.44	4.61	21.39	5.10	-3.96	1.74E-04	4.43E-03
cg05217213	AKIRIN2	6	TSS1500	S_Shore	72.38	4.96	76.34	5.50	-3.96	3.71E-04	8.04E-03
cg16014725		17	Intergenic	S_Shelf	79.99	5.10	83.95	4.39	-3.96	1.29E-03	2.07E-02
cg09245872	INO80E;DOC2A	16	3'UTR;3'UTR	Open sea	58.27	5.26	62.23	4.23	-3.96	7.57E-04	1.39E-02
cg21491405	C14orf133	14	Body	N_Shelf	76.17	5.22	80.13	4.29	-3.95	6.31E-04	1.21E-02
cg22388347		8	Intergenic	Open sea	60.08	5.27	64.04	5.10	-3.95	8.51E-04	1.52E-02
cg19761110	AGAP1;AGAP1	2	Body;Body	Island	50.06	5.19	54.01	5.64	-3.95	5.87E-04	1.15E-02
cg02928957	VEPH1;VEPH1;VEPH1;VEPH1;VEPH1;V	3	5'UTR;5'UTR;TSS1500;5'UTR;5'UTR;5'UTR	Open sea	72.67	5.77	76.63	5.18	-3.95	9.80E-04	1.69E-02
cg00491548	C19orf28;C19orf28;C19orf71;C19orf7	19	Body;3'UTR;1stExon;5'UTR	Open sea	59.81	4.97	63.76	4.29	-3.95	2.93E-04	6.67E-03
cg05459233		3	Intergenic	Open sea	26.17	5.39	30.13	5.64	-3.95	7.72E-04	1.41E-02
cg19377326	PUM1;PUM1	1	Body;Body	Open sea	54.29	5.95	58.24	4.98	-3.95	2.48E-03	3.28E-02
cg02985871		8	Intergenic	Open sea	18.38	5.80	22.34	3.46	-3.95	8.37E-04	1.50E-02
cg09171111	HIAT1	1	Body	S_Shelf	76.62	4.37	80.58	5.03	-3.95	1.66E-04	4.26E-03
cg07219844	ZNF511;TUBGCP2	10	Body;TSS1500	S_Shore	64.06	5.26	68.01	6.48	-3.95	4.35E-03	4.83E-02
cg04988729	PHRF1	11	Body	Open sea	49.29	3.16	53.25	4.17	-3.95	2.54E-05	8.64E-04
cg21889703	BCLAF1;BCLAF1;BCLAF1	6	5'UTR;5'UTR;5'UTR	N_Shelf	31.88	5.48	35.83	5.32	-3.95	2.83E-03	3.60E-02
cg08636638		5	Intergenic	N_Shelf	46.21	5.50	50.16	5.98	-3.95	2.69E-03	3.48E-02
cg07728250	MSI2;MSI2	17	Body;Body	Open sea	46.52	4.63	50.47	4.07	-3.95	1.72E-04	4.37E-03
cg20511832	CHRNB4	15	TSS1500	S_Shore	34.02	4.96	37.97	5.13	-3.95	1.16E-04	3.15E-03
cg21608519		7	Intergenic	Island	32.88	4.51	36.83	5.59	-3.95	4.81E-04	9.82E-03
cg00259886	MPZ	1	TSS200	Open sea	40.29	5.81	44.25	4.94	-3.95	2.51E-03	3.31E-02
cg02830265	FUBP1	1	Body	N_Shelf	75.60	4.62	79.55	4.77	-3.95	1.32E-04	3.52E-03
cg06784036	SPIC	12	Body	Open sea	58.95	3.41	62.90	2.92	-3.95	1.36E-06	5.97E-05
cg18299835	NFIX	19	3'UTR	Island	33.64	4.60	37.59	6.46	-3.95	3.18E-03	3.90E-02
cg26553741	MIR548H4	15	Body	Open sea	79.50	5.05	83.45	3.58	-3.95	4.78E-05	1.49E-03

cg07287444	HERPUD2	7	Body	Open sea	70.08	5.60	74.03	5.25	-3.95	3.69E-03	4.31E-02
cg17813225		3	Intergenic	Open sea	47.86	5.83	51.81	5.32	-3.95	1.79E-03	2.61E-02
cg14018429	FAM125B;FAM125B	9	Body;Body	Open sea	62.86	5.46	66.81	5.10	-3.95	8.13E-05	2.35E-03
cg05202616	PPTC7	12	Body	Open sea	38.04	4.83	41.99	5.18	-3.95	1.57E-03	2.38E-02
cg22456251	MIR7-2	15	Body	Open sea	70.81	5.59	74.76	5.42	-3.95	4.32E-03	4.81E-02
cg11256802	WDR8	1	TSS1500	Island	32.47	4.95	36.42	5.60	-3.95	1.85E-04	4.64E-03
cg12573849	POU3F3	2	TSS1500	Island	16.98	3.05	20.93	3.33	-3.95	1.06E-08	5.66E-07
cg25994725	LOC285847;C6orf81	6	Body;TSS1500	S_Shelf	64.22	4.61	68.17	3.91	-3.95	2.67E-04	6.21E-03
cg07676517		19	Intergenic	Island	21.55	4.07	25.49	5.89	-3.95	1.26E-03	2.03E-02
cg25912037		2	Intergenic	Open sea	51.39	5.37	55.34	5.66	-3.95	2.73E-03	3.51E-02
cg26760212	ZNF512B	20	TSS1500	S_Shore	25.00	4.11	28.95	4.63	-3.95	3.34E-04	7.41E-03
cg15822656		7	Intergenic	N_Shelf	55.88	5.32	59.83	5.87	-3.95	1.74E-03	2.56E-02
cg19547547	PCDHA1;PCDHA1;PCDHA1	5	1stExon;1stExon;1stExon	Island	31.72	3.77	35.67	5.54	-3.95	3.11E-04	7.00E-03
cg15076824	CRP	1	TSS1500	Open sea	60.52	5.46	64.47	4.60	-3.95	4.63E-04	9.54E-03
cg11288831	RASA3	13	Body	S_Shore	50.52	6.04	54.47	5.85	-3.95	1.99E-04	4.92E-03
cg03457195		2	Intergenic	Island	25.58	4.49	29.52	4.87	-3.95	2.70E-04	6.28E-03
cg18881440		2	Intergenic	Open sea	66.13	5.58	70.08	5.73	-3.95	3.76E-03	4.38E-02
cg15377283	SOX17	8	Body	Island	27.03	4.04	30.98	5.04	-3.95	4.82E-05	1.51E-03
cg05245094	KIAA0182;KIAA0182	16	5'UTR;Body	N_Shore	47.34	4.59	51.29	4.35	-3.95	1.66E-03	2.48E-02
cg09329930	PAX3;PAX3;PAX3;PAX3;PAX3;PAX3	2	Body;Body;Body;Body;Body;Body	Open sea	65.91	5.79	69.85	5.28	-3.95	2.47E-03	3.28E-02
cg03027244		15	Intergenic	Island	18.74	3.94	22.69	4.81	-3.95	5.42E-05	1.67E-03
cg04764812		10	Intergenic	N_Shelf	61.01	5.46	64.95	3.98	-3.95	4.24E-04	8.91E-03
cg03225444		2	Intergenic	Island	45.72	3.10	49.66	3.86	-3.95	1.08E-06	4.84E-05
cg04367427		2	Intergenic	N_Shore	63.46	5.33	67.41	4.64	-3.95	6.30E-04	1.21E-02
cg11952236		7	Intergenic	S_Shore	42.24	4.33	46.18	5.83	-3.95	1.91E-03	2.73E-02
cg26940518	IFT140;TMEM204	16	Body;Body	S_Shore	40.56	5.11	44.51	5.82	-3.95	4.99E-05	1.55E-03
cg00328376	SKAP2	7	Body	N_Shore	71.93	5.91	75.87	5.46	-3.95	6.30E-05	1.90E-03
cg21704109	ALX4	11	TSS1500	Island	38.04	4.13	41.98	4.49	-3.94	3.08E-05	1.02E-03
cg08410996	ZBED3	5	Body	Island	46.94	5.46	50.88	4.10	-3.94	8.97E-04	1.58E-02
cg09889477	FLJ45079	17	TSS1500	N_Shelf	82.85	4.27	86.79	2.94	-3.94	8.51E-06	3.25E-04
cg04751276		15	Intergenic	Island	50.97	3.76	54.92	4.54	-3.94	4.13E-05	1.31E-03
cg25666547		8	Intergenic	Open sea	74.44	6.10	78.39	5.72	-3.94	1.70E-04	4.34E-03
cg25529875	GFOD1	6	Body	Open sea	33.13	4.86	37.07	4.78	-3.94	1.09E-03	1.82E-02
cg18275733	NCKAP5;NCKAP5	2	Body;Body	Open sea	65.19	4.24	69.13	4.06	-3.94	2.42E-06	1.02E-04
cg25047056	KIAA1462	10	5'UTR	Island	20.72	4.03	24.66	4.67	-3.94	2.88E-06	1.20E-04
cg25264265	MAP3K13;MAP3K13	3	1stExon;5'UTR	Open sea	43.62	5.40	47.57	7.63	-3.94	1.83E-04	4.61E-03
cg21970626		13	Intergenic	Island	48.69	4.86	52.64	4.30	-3.94	2.65E-04	6.18E-03
cg15487646		1	Intergenic	S_Shelf	46.46	3.59	50.40	3.35	-3.94	2.40E-06	1.01E-04
cg14511739	APC;APC;APC	5	TSS200;TSS200;5'UTR	Open sea	18.82	6.97	22.76	8.68	-3.94	1.03E-03	1.75E-02
cg12145783	GIGYF1	7	TSS200	S_Shore	75.95	4.76	79.89	3.30	-3.94	9.78E-05	2.75E-03
cg24315421		1	Intergenic	N_Shore	56.59	5.87	60.53	5.21	-3.94	1.61E-03	2.42E-02
cg00827176	NPM2	8	Body	S_Shelf	53.77	3.93	57.71	3.51	-3.94	3.00E-05	9.97E-04
cg25223716	ADAM12;ADAM12	10	Body;Body	Open sea	75.13	5.79	79.08	4.86	-3.94	1.12E-03	1.87E-02
cg15087178	ANK3	10	Body	Open sea	44.20	4.59	48.14	4.78	-3.94	2.12E-03	2.95E-02
cg17711527	LAX1;LAX1;LAX1;LAX1	1	1stExon;1stExon;5'UTR;5'UTR	Open sea	41.63	5.39	45.57	4.74	-3.94	2.32E-04	5.56E-03
cg19218509	ASIP	20	Body	Island	51.00	5.14	54.94	6.26	-3.94	5.26E-04	1.05E-02
cg11237948	FOSB;FOSB	19	3'UTR;3'UTR	S_Shore	28.12	3.74	32.06	5.89	-3.94	1.61E-03	2.42E-02
cg14788049	PLCB3	11	TSS1500	Island	41.68	6.03	45.62	7.12	-3.94	5.12E-04	1.03E-02
cg09639151	PCDHGA4;PCDHGA11;PCDHGA11;PCD	5	Body;Body;Body;Body;Body;Body;Body;1s	Island	14.44	3.96	18.38	6.16	-3.94	1.01E-03	1.73E-02
cg11253737	NFASC;NFASC;NFASC;NFASC;NFASC	1	TSS1500;TSS1500;TSS1500;TSS1500;TSS15	N_Shore	46.12	5.06	50.06	5.34	-3.94	2.83E-03	3.60E-02
cg24211214		2	Intergenic	Open sea	55.02	5.77	58.96	3.91	-3.94	6.68E-04	1.27E-02
cg06575692	DUS2L	16	3'UTR	Open sea	72.42	4.86	76.36	4.16	-3.94	4.08E-04	8.66E-03
cg04899563	KNDC1	10	Body	S_Shore	49.54	5.71	53.48	6.69	-3.94	1.86E-03	2.69E-02

cg00162806	VENTX	10	Body	Island	40.84	4.72	44.78	5.67	-3.94	4.41E-03	4.88E-02
cg16046375	RASA3	13	Body	S_Shore	44.08	6.93	48.02	6.22	-3.94	1.01E-03	1.72E-02
cg08939418	IGF2BP3	7	TSS1500	S_Shore	24.43	4.39	28.37	6.02	-3.94	3.63E-03	4.27E-02
cg19165640	ECE1	1	Body	Open sea	60.18	5.61	64.12	4.13	-3.94	1.14E-03	1.89E-02
cg13741406	PRKD2;PRKD2;PRKD2;PRKD2	19	Body;Body;Body;Body	S_Shore	30.77	3.56	34.71	4.11	-3.94	3.50E-05	1.14E-03
cg14389924	PELP1	17	Body	N_Shore	41.52	4.25	45.46	5.02	-3.94	7.46E-05	2.19E-03
cg20062978	NUP153	6	3'UTR	Open sea	79.69	6.54	83.63	6.50	-3.94	1.56E-03	2.36E-02
cg18699025	FGFRL1;FGFRL1;FGFRL1	4	Body;Body;Body	S_Shore	81.47	4.83	85.41	4.21	-3.94	3.96E-04	8.45E-03
cg12577850	C6orf115	6	5'UTR	S_Shore	12.71	3.52	16.64	5.15	-3.94	1.58E-05	5.68E-04
cg25796963	ITIH3	3	TSS1500	Open sea	75.76	4.20	79.70	4.13	-3.94	1.73E-04	4.41E-03
cg15979152	PRSSL1	19	Body	N_Shore	74.97	6.94	78.91	5.02	-3.94	2.52E-03	3.32E-02
cg27370104	TRERF1	6	5'UTR	N_Shore	49.95	3.78	53.89	4.69	-3.94	9.92E-05	2.78E-03
cg16008979		7	Intergenic	Open sea	54.45	6.20	58.39	5.22	-3.94	4.66E-04	9.59E-03
cg20272155	HCCA2;LOC402778	11	Body;TSS200	Island	44.04	5.10	47.98	5.16	-3.94	1.17E-03	1.92E-02
cg23652093	MAP6;MAP6	11	Body;Body	Open sea	78.29	4.72	82.23	4.26	-3.94	4.78E-07	2.23E-05
cg08026735	VWA3B	2	5'UTR	Island	53.52	5.56	57.46	4.46	-3.94	5.72E-05	1.75E-03
cg02598335	GRM3	7	5'UTR	Open sea	45.13	4.95	49.06	6.57	-3.94	2.70E-03	3.48E-02
cg06620896		15	Intergenic	S_Shore	12.87	3.50	16.80	4.66	-3.94	1.18E-04	3.20E-03
cg02746640		7	Intergenic	Open sea	76.69	6.99	80.63	4.79	-3.94	2.75E-03	3.52E-02
cg09347160		4	Intergenic	Open sea	56.64	5.13	60.57	5.30	-3.94	6.91E-04	1.30E-02
cg14566819	HEMGN;HEMGN	9	3'UTR;3'UTR	Open sea	36.98	6.42	40.91	4.74	-3.94	3.09E-03	3.83E-02
cg25818583	GP6;GP6	19	TSS200;TSS200	Open sea	57.27	4.77	61.21	4.77	-3.94	1.04E-03	1.76E-02
cg19107264	DLGAP2	8	Body	S_Shelf	55.20	5.04	59.14	7.53	-3.94	2.22E-03	3.04E-02
cg06212412	TRIM26	6	3'UTR	Open sea	76.39	4.72	80.33	5.25	-3.93	1.57E-03	2.38E-02
cg01975714	DGCR5	22	Body	N_Shelf	57.31	4.74	61.25	4.18	-3.93	4.05E-04	8.60E-03
cg03453449	USP44;USP44	12	1stExon;5'UTR	S_Shelf	68.90	6.09	72.83	5.83	-3.93	4.09E-03	4.63E-02
cg03118633	AFAP1L2;AFAP1L2	10	Body;Body	Open sea	78.12	5.12	82.05	4.41	-3.93	5.23E-04	1.05E-02
cg21093043	ZMAT4;ZMAT4	8	Body;Body	Open sea	52.97	4.98	56.91	4.31	-3.93	3.44E-04	7.57E-03
cg15723028	RIPK2	8	Body	Open sea	72.47	6.94	76.41	4.59	-3.93	8.15E-04	1.47E-02
cg20873416		6	Intergenic	Open sea	40.95	10.31	44.88	11.62	-3.93	1.88E-03	2.71E-02
cg01977582	CBFA2T3;CBFA2T3	16	5'UTR;Body	S_Shore	51.59	5.38	55.53	6.01	-3.93	4.14E-03	4.67E-02
cg14333304	ZNF467	7	3'UTR	N_Shore	87.44	5.96	91.37	3.78	-3.93	4.06E-04	8.62E-03
cg11691844	SYTL2;SYTL2;SYTL2	11	Body;Body;Body	Open sea	74.43	5.13	78.36	4.77	-3.93	1.07E-04	2.97E-03
cg24228952		2	Intergenic	Open sea	56.40	5.43	60.34	5.45	-3.93	4.28E-03	4.78E-02
cg11341215	MCCC1	3	Body	S_Shelf	79.80	3.03	83.73	2.58	-3.93	6.82E-09	3.66E-07
cg11358945	NR4A2	2	Body	Island	50.73	3.89	54.66	3.51	-3.93	5.32E-06	2.12E-04
cg12989128	GDAP1L1;GDAP1L1	20	5'UTR;1stExon	Island	15.35	3.98	19.28	4.70	-3.93	8.54E-06	3.27E-04
cg00144335	ZNF718	4	Body	Island	42.17	4.68	46.10	6.29	-3.93	1.34E-03	2.12E-02
cg26069230	ADAP2	17	Body	S_Shore	15.88	3.50	19.81	4.24	-3.93	3.50E-05	1.14E-03
cg10789314		19	Intergenic	N_Shore	16.10	3.67	20.04	3.60	-3.93	8.01E-06	3.08E-04
cg11854806	DYDC2;DYDC1	10	TSS200;5'UTR	Island	38.02	7.11	41.96	6.38	-3.93	4.46E-03	4.92E-02
cg21433558	CNTNAP1	17	Body	Island	50.80	3.57	54.73	3.24	-3.93	9.32E-06	3.53E-04
cg01145124	USP34	2	Body	Open sea	85.72	4.10	89.65	2.74	-3.93	1.53E-06	6.67E-05
cg05675514	FLJ22536	6	Body	Open sea	34.37	5.64	38.30	5.94	-3.93	8.17E-04	1.47E-02
cg21330960	RTDR1;GNAZ	22	Body;5'UTR	S_Shelf	53.31	3.87	57.24	4.87	-3.93	2.98E-04	6.77E-03
cg07819160	MPST;MPST;MPST;MPST	22	Body;Body;Body;Body	Island	37.06	5.61	40.99	4.35	-3.93	3.65E-03	4.28E-02
cg27630540	KIAA1530	4	Body	Open sea	88.96	5.86	92.89	4.38	-3.93	2.33E-04	5.59E-03
cg15990972		2	Intergenic	Island	20.87	3.94	24.80	4.65	-3.93	3.54E-04	7.76E-03
cg21481950	GABBR1;GABBR1	6	TSS1500;TSS1500	S_Shore	35.67	5.44	39.59	4.52	-3.93	2.79E-04	6.43E-03
cg17037101	INS-IGF2;IGF2AS;IGF2;IGF2;IGF2AS;IG	11	Body;TSS1500;Body;5'UTR;TSS1500;TSS15	Island	54.53	4.63	58.45	4.07	-3.93	2.32E-04	5.57E-03
cg08602500	PTPRN2;PTPRN2;PTPRN2	7	Body;Body;Body	Island	26.72	5.16	30.65	5.33	-3.93	6.67E-04	1.26E-02
cg05600496		13	Intergenic	Open sea	41.77	4.98	45.70	5.30	-3.93	6.63E-04	1.26E-02
cg13114497		22	Intergenic	S_Shore	42.52	5.13	46.45	5.20	-3.93	3.42E-04	7.55E-03

cg08491681	STARD13;STARD13	13	TSS1500;Body	Open sea	63.18	5.76	67.11	4.74	-3.93	4.14E-03	4.67E-02
cg14239655		12	Intergenic	Open sea	31.12	5.69	35.05	5.94	-3.93	2.43E-03	3.24E-02
cg20124223	DIXDC1;DIXDC1	11	TSS1500;Body	N_Shore	46.25	5.18	50.18	4.33	-3.93	1.12E-04	3.09E-03
cg01553004	C14orf4	14	TSS1500	S_Shore	27.67	4.21	31.60	4.84	-3.93	2.89E-04	6.61E-03
cg03739409	TPH2	12	Body	Open sea	72.64	6.39	76.56	4.66	-3.93	2.26E-03	3.08E-02
cg20071427	ZNF331;ZNF331;ZNF331	19	5'UTR;5'UTR;TSS1500	Island	52.57	6.43	56.49	6.44	-3.93	1.33E-03	2.12E-02
cg12633817	C7orf50;C7orf50;C7orf50	7	Body;Body;Body	N_Shelf	73.26	4.87	77.19	5.05	-3.93	1.08E-03	1.81E-02
cg09626833	TUBGCP3	13	Body	Open sea	80.05	3.68	83.97	3.18	-3.93	3.18E-06	1.32E-04
cg26950568		13	Intergenic	Open sea	28.34	5.21	32.26	5.22	-3.93	7.72E-04	1.41E-02
cg21518248		2	Intergenic	Island	29.35	5.12	33.28	6.05	-3.93	2.16E-03	2.98E-02
cg19141448	SEMA5B	3	Body	Island	30.79	3.45	34.71	4.43	-3.93	3.54E-05	1.15E-03
cg05473387	SCAP	3	5'UTR	Open sea	31.70	4.80	35.63	6.07	-3.93	4.10E-03	4.64E-02
cg10618704	GAS7;GAS7	17	TSS1500;Body	Open sea	67.83	5.15	71.76	3.96	-3.93	8.51E-04	1.52E-02
cg05652445		16	Intergenic	N_Shore	62.04	5.60	65.97	5.58	-3.93	3.03E-03	3.77E-02
cg07737802	FGD6	12	Body	Open sea	69.37	6.55	73.29	4.22	-3.93	8.13E-04	1.47E-02
cg04724873	KIAA1751	1	Body	Open sea	21.37	3.72	25.30	3.51	-3.93	3.53E-05	1.15E-03
cg08201451	VAC14	16	Body	Open sea	61.09	4.97	65.02	5.23	-3.93	3.55E-03	4.21E-02
cg04752871		2	Intergenic	S_Shore	33.48	3.91	37.40	4.14	-3.92	3.99E-05	1.28E-03
cg11232245		10	Intergenic	S_Shore	40.89	5.50	44.81	6.21	-3.92	3.56E-03	4.21E-02
cg05938624		8	Intergenic	S_Shelf	47.96	4.16	51.89	4.97	-3.92	1.76E-04	4.46E-03
cg17842562	CLASP1;CLASP1;CLASP1	2	Body;Body;Body	Open sea	51.49	4.69	55.41	3.64	-3.92	1.63E-04	4.20E-03
cg23146172		1	Intergenic	Island	42.29	4.97	46.22	5.79	-3.92	3.22E-04	7.20E-03
cg25747190	SND1	7	Body	S_Shelf	65.88	5.07	69.80	5.15	-3.92	8.04E-04	1.45E-02
cg11580676	BGLAP	1	TSS1500	N_Shelf	82.69	3.44	86.62	2.15	-3.92	1.63E-08	8.61E-07
cg06829266	NME4	16	TSS1500	N_Shore	17.51	5.95	21.43	5.36	-3.92	9.62E-04	1.66E-02
cg11247378		22	Intergenic	Island	54.55	6.42	58.47	6.98	-3.92	1.79E-03	2.62E-02
cg01351337	C10orf76	10	Body	Open sea	36.71	5.85	40.63	5.14	-3.92	1.85E-03	2.67E-02
cg17198308	IFFO1;IFFO1	12	1stExon;1stExon	Island	3.40	1.52	7.33	5.71	-3.92	7.20E-06	2.79E-04
cg13232118	MIR149;PP14571;GPC1	2	TSS1500;Body;Body	N_Shore	46.95	7.08	50.87	7.36	-3.92	3.52E-03	4.18E-02
cg16044251		6	Intergenic	Island	37.33	6.25	41.25	5.10	-3.92	2.36E-03	3.17E-02
cg15525489	CAMK2D;CAMK2D;CAMK2D	4	3'UTR;3'UTR;3'UTR	Open sea	14.54	3.18	18.46	4.58	-3.92	3.95E-05	1.27E-03
cg11368946	GCAT	22	Body	S_Shore	9.05	2.52	12.97	4.06	-3.92	7.15E-07	3.28E-05
cg16417409		11	Intergenic	S_Shelf	29.07	6.06	32.99	5.87	-3.92	1.49E-03	2.30E-02
cg02286549	TFEB;TFEB	6	5'UTR;5'UTR	N_Shore	18.76	3.79	22.68	4.06	-3.92	4.82E-06	1.94E-04
cg12616923		8	Intergenic	Open sea	34.09	5.40	38.02	5.24	-3.92	2.09E-03	2.92E-02
cg24660199	LRDD;LRDD;LRDD	11	Body;Body;Body	N_Shore	55.71	4.84	59.63	4.47	-3.92	7.79E-05	2.27E-03
cg11397301	SCG5;SCG5	15	TSS1500;TSS1500	Open sea	53.85	4.10	57.77	5.52	-3.92	3.24E-04	7.23E-03
cg01058342	TMEM108;TMEM108	3	Body;Body	Open sea	53.08	4.68	57.00	5.11	-3.92	1.81E-04	4.57E-03
cg26915889		1	Intergenic	Open sea	27.71	4.25	31.63	5.08	-3.92	3.02E-04	6.84E-03
cg27257822	EPHB3	3	Body	S_Shore	56.58	3.93	60.50	3.25	-3.92	4.06E-05	1.30E-03
cg13817046		7	Intergenic	S_Shelf	54.84	6.23	58.76	5.52	-3.92	3.19E-03	3.91E-02
cg07529428	PTPRO;PTPRO;PTPRO;PTPRO;PTPRO;F	12	Body;Body;Body;Body;Body;Body	Open sea	67.68	5.86	71.60	4.41	-3.92	3.43E-03	4.11E-02
cg24053748	FAM196A;DOCK1	10	TSS1500;Body	S_Shore	16.47	3.36	20.39	3.89	-3.92	2.45E-07	1.19E-05
cg12847502		2	Intergenic	Open sea	49.95	3.49	53.87	5.06	-3.92	4.84E-04	9.87E-03
cg26441372	LTBP4;LTBP4;LTBP4	19	Body;Body;Body	S_Shore	28.91	4.39	32.83	5.24	-3.92	7.22E-04	1.34E-02
cg07035320	CLSTN1;CLSTN1	1	TSS1500;TSS1500	S_Shore	23.37	4.39	27.29	2.90	-3.92	1.05E-04	2.93E-03
cg16788286		8	Intergenic	Island	26.17	4.61	30.08	5.78	-3.92	4.82E-04	9.84E-03
cg15443535	RIN3	14	Body	Island	35.32	3.31	39.24	3.52	-3.92	5.25E-06	2.09E-04
cg26215406	MCF2L2;B3GNT5	3	Body;5'UTR	Island	64.76	9.52	68.68	7.48	-3.92	1.55E-03	2.36E-02
cg23677778		12	Intergenic	Open sea	75.12	6.55	79.04	5.55	-3.92	8.26E-04	1.48E-02
cg23010048	ZNF577;ZNF577;ZNF577	19	TSS200;TSS200;TSS200	Island	18.21	4.45	22.12	4.33	-3.92	2.56E-04	6.02E-03
cg06109284	TBC1D14;TBC1D14	4	Body;Body	Open sea	60.84	4.44	64.76	3.70	-3.92	7.48E-05	2.19E-03
cg09177194	C11orf48	11	Body	N_Shelf	71.98	4.24	75.90	3.70	-3.92	1.92E-05	6.75E-04

cg09989847	NT5E	6	3'UTR	Open sea	65.35	4.96	69.27	4.33	-3.92	5.85E-04	1.14E-02
cg07875069	CPA5;CPA5;CPA5;CPA5;CPA5;CPA5	7	1stExon;5'UTR;5'UTR;1stExon;5'UTR;1stEx	Open sea	67.09	5.35	71.00	6.96	-3.92	1.93E-03	2.76E-02
cg15607292		3	Intergenic	Open sea	43.31	4.11	47.23	4.76	-3.92	1.69E-04	4.31E-03
cg20594303		10	Intergenic	Open sea	52.49	6.13	56.41	5.73	-3.92	2.67E-04	6.21E-03
cg09618778	RHOD	11	Body	Island	47.64	3.80	51.55	4.52	-3.92	2.30E-04	5.53E-03
cg27438889	TACC2;TACC2;TACC2;TACC2	10	TSS1500;Body;TSS1500;Body	N_Shore	23.95	3.41	27.86	4.66	-3.92	1.86E-04	4.66E-03
cg21513138	MUC20;MUC20	3	3'UTR;3'UTR	Open sea	51.61	5.44	55.53	5.91	-3.92	1.76E-03	2.58E-02
cg09862509		1	Intergenic	Open sea	51.21	3.17	55.12	3.87	-3.92	2.65E-06	1.11E-04
cg17945789	AGAP1;AGAP1	2	Body;Body	S_Shore	71.14	4.81	75.06	4.19	-3.92	3.03E-04	6.87E-03
cg17901584	DHCR24	1	TSS1500	S_Shore	25.03	4.55	28.95	4.82	-3.92	6.96E-04	1.30E-02
cg21467371	ARL16	17	3'UTR	N_Shore	34.14	3.94	38.05	3.95	-3.92	1.22E-04	3.30E-03
cg04959196	VARS	6	Body	N_Shelf	62.10	5.26	66.01	5.26	-3.91	4.29E-04	9.00E-03
cg21175246		14	Intergenic	Open sea	24.37	5.14	28.28	5.14	-3.91	3.78E-03	4.39E-02
cg03951394	DEAF1	11	Body	Island	86.99	12.70	90.90	8.28	-3.91	3.20E-03	3.92E-02
cg15635392	HLA-DRA	6	TSS1500	Open sea	61.46	5.26	65.37	4.76	-3.91	2.25E-03	3.07E-02
cg07080244		11	Intergenic	Open sea	53.48	3.70	57.39	3.77	-3.91	7.44E-06	2.88E-04
cg07703690	BBOX1	11	5'UTR	Open sea	81.74	5.82	85.65	3.76	-3.91	3.17E-04	7.12E-03
cg09998189	SMOC1;SMOC1	14	Body;Body	S_Shore	26.95	3.88	30.86	4.06	-3.91	3.16E-05	1.04E-03
cg09830866	FAM173A	16	Body	Island	48.92	4.40	52.83	4.47	-3.91	1.24E-04	3.33E-03
cg26562921	USP10	16	Body	Open sea	55.98	5.34	59.89	5.26	-3.91	4.03E-03	4.59E-02
cg13348944	SCN2B	11	1stExon	Open sea	67.96	4.77	71.87	4.07	-3.91	3.70E-04	8.01E-03
cg16478737		16	Intergenic	S_Shore	80.46	3.85	84.37	3.45	-3.91	2.08E-06	8.90E-05
cg12001078	DAPP1	4	3'UTR	Open sea	73.72	4.34	77.63	4.46	-3.91	4.55E-05	1.43E-03
cg23713742	SPAG4	20	TSS1500	N_Shore	53.12	5.22	57.03	5.34	-3.91	7.63E-05	2.23E-03
cg22400068		11	Intergenic	Open sea	77.91	5.58	81.82	5.27	-3.91	2.44E-03	3.25E-02
cg25999604	XRCC3;XRCC3;XRCC3	14	5'UTR;5'UTR;5'UTR	N_Shelf	60.76	5.35	64.67	5.08	-3.91	1.78E-03	2.60E-02
cg10937807	DIP2C	10	Body	N_Shelf	70.49	5.46	74.40	5.70	-3.91	2.60E-03	3.39E-02
cg00523379	SASH1	6	Body	Open sea	62.27	4.38	66.18	4.21	-3.91	4.78E-04	9.78E-03
cg25724712		19	Intergenic	Open sea	86.80	7.53	90.71	4.46	-3.91	4.46E-03	4.92E-02
cg09153713	SLC44A4	6	Body	S_Shelf	69.55	4.66	73.46	5.19	-3.91	5.18E-05	1.60E-03
cg11116758		19	Intergenic	Island	25.56	4.34	29.47	4.23	-3.91	4.76E-05	1.49E-03
cg08060501	GLIS2	16	3'UTR	Open sea	71.89	3.70	75.80	2.88	-3.91	1.91E-06	8.21E-05
cg16947394	TMEM110	3	Body	N_Shore	54.66	4.18	58.57	4.42	-3.91	5.36E-05	1.65E-03
cg06803257	SLC12A4;SLC12A4;SLC12A4;SLC12A4;S	16	Body;Body;TSS1500;Body;Body	N_Shelf	88.03	3.44	91.94	2.29	-3.91	2.00E-09	1.10E-07
cg15037583	MACROD1	11	Body	Island	46.14	4.81	50.05	5.46	-3.91	2.91E-04	6.64E-03
cg20522398		7	Intergenic	Open sea	70.86	5.96	74.77	4.89	-3.91	1.14E-04	3.12E-03
cg14619716		11	Intergenic	Open sea	73.77	4.50	77.68	3.40	-3.91	1.41E-05	5.13E-04
cg15911493	AP4S1;AP4S1	14	5'UTR;5'UTR	Open sea	55.85	3.82	59.76	4.40	-3.91	2.71E-04	6.29E-03
cg22799499	COL9A2	1	Body	S_Shelf	60.58	5.04	64.49	4.07	-3.91	3.52E-04	7.71E-03
cg20097712		11	Intergenic	Open sea	71.66	7.47	75.57	6.07	-3.91	2.95E-03	3.70E-02
cg06480138	HDDC3;UNC45A;UNC45A	15	TSS1500;5'UTR;TSS1500	N_Shore	79.44	4.32	83.35	3.75	-3.91	2.04E-04	5.02E-03
cg15811568	RIMBP2	12	Body	N_Shelf	66.14	4.66	70.05	5.24	-3.91	6.32E-05	1.90E-03
cg17690002	SMARCD3;SMARCD3	7	5'UTR;5'UTR	N_Shore	58.82	4.39	62.72	4.52	-3.91	5.13E-04	1.03E-02
cg24003508	KLF6;KLF6;KLF6;KLF6	10	Body;Body;Body;Body	S_Shore	21.02	8.98	24.92	10.96	-3.91	4.54E-03	4.97E-02
cg23089723	NOTCH4	6	Body	Open sea	65.58	4.26	69.48	5.01	-3.91	7.93E-06	3.05E-04
cg07379741	NLGN2	17	TSS1500	N_Shore	62.81	4.07	66.72	3.30	-3.91	5.34E-06	2.13E-04
cg19328475	PRDM6	5	Body	N_Shore	51.76	5.62	55.67	5.15	-3.91	1.18E-04	3.21E-03
cg09597806		15	Intergenic	Island	42.05	4.62	45.96	3.82	-3.91	4.17E-05	1.33E-03
cg17297354	MTR	1	Body	Open sea	71.08	5.09	74.99	6.21	-3.91	7.90E-04	1.43E-02
cg22507723	AGAP2;AGAP2	12	1stExon;Body	Island	27.79	3.59	31.69	4.18	-3.91	4.04E-05	1.29E-03
cg18547849	C19orf28;C19orf28;C19orf28	19	3'UTR;Body;Body	N_Shore	72.69	5.24	76.59	4.03	-3.91	8.45E-04	1.51E-02
cg09258981	CDC42EP2	11	Body	Open sea	69.25	3.57	73.16	3.04	-3.91	3.31E-06	1.37E-04
cg23630380	SRCIN1	17	Body	S_Shore	52.05	5.55	55.95	5.46	-3.91	2.41E-04	5.74E-03

cg13385203		17	Intergenic	S_Shelf	77.02	3.75	80.93	4.05	-3.91	2.42E-05	8.29E-04
cg20701333	KCNJ14;KCNJ14	19	1stExon;Body	Island	68.80	5.07	72.71	5.03	-3.90	3.21E-03	3.92E-02
cg04416734	ALDOA;ALDOA;ALDOA	16	TSS1500;TSS1500;5'UTR	N_Shore	35.43	4.50	39.34	6.44	-3.90	3.73E-03	4.35E-02
cg11728143	TBC1D7;TBC1D7;TBC1D7;TBC1D7	6	Body;Body;Body;Body	N_Shelf	75.15	4.98	79.06	4.63	-3.90	4.94E-04	1.00E-02
cg23156059	KIAA1274	10	5'UTR	Open sea	48.94	5.43	52.84	4.82	-3.90	1.08E-03	1.82E-02
cg03489382	CCDC136;CCDC136	7	1stExon;5'UTR	S_Shore	55.18	5.00	59.08	6.03	-3.90	2.43E-03	3.24E-02
cg24084836	POMT2	14	Body	Open sea	38.37	4.99	42.27	5.20	-3.90	2.03E-03	2.86E-02
cg04270799	KIF5A	12	1stExon	S_Shore	33.30	4.00	37.21	3.83	-3.90	1.39E-06	6.09E-05
cg05603896	SCML4	6	TSS200	Open sea	32.44	3.68	36.34	3.92	-3.90	6.91E-06	2.69E-04
cg24002887		7	Intergenic	Island	24.54	5.21	28.44	5.10	-3.90	1.64E-03	2.46E-02
cg20515423		14	Intergenic	Open sea	82.83	4.31	86.73	3.72	-3.90	4.84E-05	1.51E-03
cg06148480	TBR1	2	Body	N_Shore	31.70	4.86	35.61	6.16	-3.90	3.67E-03	4.30E-02
cg03096954		16	Intergenic	Open sea	66.13	4.53	70.03	3.69	-3.90	1.88E-04	4.71E-03
cg16625109		2	Intergenic	N_Shore	20.95	3.77	24.85	4.37	-3.90	2.16E-05	7.48E-04
cg07921371	GJB2	13	TSS1500	S_Shore	35.01	6.35	38.92	7.39	-3.90	1.69E-03	2.50E-02
cg23229261	OTX1	2	3'UTR	Island	14.82	3.56	18.73	3.88	-3.90	1.22E-05	4.49E-04
cg01469864	CPLX2;CPLX2	5	5'UTR;5'UTR	Island	34.00	6.56	37.90	6.39	-3.90	2.83E-03	3.60E-02
cg16214034	SLC25A16	10	Body	Open sea	72.31	4.86	76.21	4.51	-3.90	1.67E-04	4.29E-03
cg13341720	TRIP10	19	Body	Island	28.62	5.65	32.52	7.33	-3.90	1.34E-04	3.57E-03
cg17033622	FLI1;FLI1	11	Body;5'UTR	S_Shelf	69.01	7.09	72.92	7.00	-3.90	6.08E-04	1.18E-02
cg16725721	FSCN3;FSCN3	7	1stExon;5'UTR	Open sea	37.71	4.53	41.61	4.97	-3.90	2.05E-03	2.88E-02
cg08513622	MAD1L1;MAD1L1;MAD1L1	7	Body;Body;Body	N_Shore	86.96	5.65	90.87	4.43	-3.90	2.59E-04	6.07E-03
cg14360405		11	Intergenic	S_Shelf	67.05	5.42	70.95	5.00	-3.90	2.35E-03	3.16E-02
cg10167512		13	Intergenic	Open sea	60.61	4.23	64.51	3.85	-3.90	1.63E-05	5.83E-04
cg24524485	BUB3;BUB3	10	Body;Body	S_Shore	77.99	4.34	81.89	3.91	-3.90	1.03E-05	3.87E-04
cg06015781		11	Intergenic	S_Shore	34.55	4.81	38.45	4.46	-3.90	2.72E-04	6.30E-03
cg14180029	LDLRAD3	11	Body	Open sea	72.45	5.09	76.36	5.58	-3.90	4.76E-04	9.75E-03
cg19943578	ARHGAP27;ARHGAP27	17	TSS200;5'UTR	S_Shore	84.26	4.69	88.16	5.17	-3.90	1.00E-04	2.80E-03
cg14206576		15	Intergenic	S_Shelf	78.36	4.58	82.26	4.77	-3.90	9.32E-05	2.64E-03
cg05864010	CREBZF;CREBZF;CREBZF;CREBZF;CREB	11	TSS1500;TSS1500;TSS1500;TSS1500;TSS15	S_Shore	55.19	4.82	59.09	3.60	-3.90	1.18E-04	3.20E-03
cg27627854	ATM;ATM	11	Body;1stExon	Open sea	75.41	5.73	79.31	5.28	-3.90	1.07E-03	1.80E-02
cg10037894	DNM3;DNM3	1	Body;Body	Open sea	72.36	4.92	76.26	3.56	-3.90	1.36E-04	3.60E-03
cg03657573	MCF2L2;B3GNT5	3	Body;5'UTR	Island	6.56	3.35	10.46	3.61	-3.90	1.91E-08	1.00E-06
cg00988577	HLA-DOA	6	TSS1500	S_Shelf	76.39	7.01	80.29	6.00	-3.90	4.10E-03	4.64E-02
cg08700546	SLC39A13;SLC39A13	11	Body;Body	Open sea	70.78	4.16	74.68	3.36	-3.90	1.12E-04	3.08E-03
cg14950344	NSF	17	Body	Open sea	82.14	5.02	86.04	4.29	-3.90	8.35E-04	1.50E-02
cg00199059	TP53RK	20	Body	N_Shore	27.20	4.67	31.10	3.33	-3.90	3.21E-05	1.06E-03
cg19856705		1	Intergenic	Open sea	43.91	4.61	47.81	5.46	-3.90	2.27E-03	3.09E-02
cg04647529	TNS4	17	3'UTR	Open sea	33.09	4.45	36.99	4.64	-3.90	6.81E-04	1.28E-02
cg04850017	RCOR2	11	Body	N_Shore	46.95	4.26	50.85	4.33	-3.90	2.00E-04	4.94E-03
cg11619797		7	Intergenic	S_Shelf	79.93	6.20	83.83	5.94	-3.90	1.24E-03	2.01E-02
cg22873725	ANKRD28	3	Body	N_Shelf	82.37	4.75	86.27	4.63	-3.90	2.03E-04	5.00E-03
cg10984680		4	Intergenic	S_Shelf	46.83	4.65	50.73	6.11	-3.90	3.37E-03	4.06E-02
cg20733761	PCBP3;PCBP3	21	Body;Body	Island	76.93	5.16	80.82	5.30	-3.90	1.15E-03	1.90E-02
cg00589541	FNDC3B;FNDC3B	3	Body;Body	Open sea	64.06	7.00	67.96	5.01	-3.90	4.16E-03	4.69E-02
cg17487170	TCF7;TCF7;TCF7;TCF7;TCF7;TCF7;TCF7	5	TSS200;TSS1500;TSS1500;TSS200;Body;TS	Island	30.77	5.54	34.66	5.78	-3.90	9.15E-05	2.60E-03
cg26162564		20	Intergenic	S_Shelf	30.78	3.89	34.68	4.06	-3.90	1.71E-06	7.40E-05
cg14353201	ALDH3B2	11	5'UTR	Open sea	55.03	5.80	58.93	5.18	-3.90	6.42E-04	1.23E-02
cg14030586	VAV2;VAV2	9	Body;Body	N_Shore	16.24	3.84	20.13	4.13	-3.90	1.42E-04	3.73E-03
cg18759260	DNM3;DNM3	1	Body;Body	Open sea	69.11	3.44	73.00	2.91	-3.90	4.66E-07	2.18E-05
cg23433008	RASGEF1C	5	5'UTR	Island	59.07	5.15	62.97	5.11	-3.90	1.16E-03	1.92E-02
cg19039672	ZNHIT3	17	Body	S_Shelf	58.12	6.33	62.02	5.65	-3.90	2.04E-03	2.86E-02
cg10806236		5	Intergenic	Open sea	41.57	4.39	45.47	5.57	-3.90	9.39E-04	1.63E-02

cg06478085		19	Intergenic	N_Shelf	42.22	6.28	46.12	5.97	-3.90	3.26E-03	3.96E-02
cg07815836	ZNF274;ZNF274;ZNF274	19	Body;Body;5'UTR	Island	41.68	4.02	45.57	4.91	-3.90	2.03E-04	5.00E-03
cg13346820	BRD9;BRD9;BRD9	5	Body;Body;Body	Island	68.88	5.64	72.77	4.14	-3.90	2.06E-03	2.89E-02
cg07311419		11	Intergenic	Open sea	62.36	6.07	66.26	5.66	-3.90	6.23E-04	1.20E-02
cg15665203		13	Intergenic	Open sea	66.98	5.35	70.87	4.18	-3.90	2.62E-04	6.13E-03
cg27257452		3	Intergenic	N_Shelf	67.76	4.24	71.66	4.39	-3.90	9.09E-05	2.59E-03
cg18127922	LOC404266;LOC404266;LOC404266;H	17	Body;Body;Body;TSS1500;TSS1500;Body	N_Shore	54.01	4.05	57.91	4.39	-3.90	1.87E-04	4.68E-03
cg06155620		1	Intergenic	Open sea	49.61	5.11	53.51	5.66	-3.90	1.22E-03	1.99E-02
cg02713669	LOC100287216;SH3RF3	2	TSS200;Body	Island	17.37	4.52	21.26	5.31	-3.90	1.49E-03	2.29E-02
cg19925112	GHSR	3	Body	N_Shelf	62.53	5.49	66.43	5.99	-3.90	3.44E-03	4.11E-02
cg06651450	TRIM71	3	Body	N_Shore	49.96	3.40	53.86	3.77	-3.90	3.38E-05	1.11E-03
cg20139800	GABBR1;GABBR1	6	Body;Body	N_Shore	32.54	4.24	36.43	6.05	-3.90	3.53E-03	4.19E-02
cg13718539	PCDHGA4;PCDHGA9;PCDHGA1;PCDH(	5	Body;Body;Body;Body;Body;Body;1stExon	N_Shore	24.43	4.53	28.33	5.47	-3.90	7.23E-04	1.34E-02
cg05684891	DAB2IP	9	Body	Island	13.10	4.31	16.99	4.81	-3.90	1.68E-04	4.29E-03
cg25904344		7	Intergenic	Island	46.12	6.14	50.02	6.62	-3.90	8.56E-04	1.52E-02
cg04132452	PPM1L	3	Body	S_Shore	37.74	5.01	41.64	5.55	-3.89	1.31E-03	2.09E-02
cg08056069		1	Intergenic	Open sea	62.60	5.90	66.49	5.46	-3.89	3.80E-04	8.18E-03
cg05058354	INTS1	7	Body	N_Shore	63.53	5.10	67.43	4.01	-3.89	1.04E-04	2.90E-03
cg14028671	NPDC1	9	Body	N_Shelf	61.41	4.45	65.31	5.10	-3.89	9.87E-04	1.69E-02
cg09889479	SLC9A3R2;SLC9A3R2	16	Body;Body	N_Shelf	13.76	4.05	17.65	4.54	-3.89	3.41E-04	7.52E-03
cg24085930		3	Intergenic	Open sea	83.63	3.75	87.52	2.80	-3.89	1.23E-06	5.43E-05
cg03278564		13	Intergenic	Open sea	20.99	4.69	24.88	6.13	-3.89	3.55E-03	4.20E-02
cg08457514	NFIA;NFIA;NFIA;NFIA	1	Body;Body;Body;Body	Open sea	17.71	4.20	21.61	5.36	-3.89	1.32E-03	2.10E-02
cg04625905	ATP10B	5	TSS1500	Open sea	74.39	5.52	78.28	5.54	-3.89	1.45E-03	2.24E-02
cg17583158	MYOM2	8	Body	Island	70.17	7.59	74.07	5.80	-3.89	2.73E-03	3.51E-02
cg19403323	C1orf107	1	Body	Open sea	77.52	4.58	81.41	3.76	-3.89	7.15E-04	1.33E-02
cg06282247		2	Intergenic	Open sea	36.45	3.28	40.34	4.57	-3.89	1.64E-05	5.87E-04
cg01811187	CACNA1G;CACNA1G;CACNA1G;CACN/	17	TSS1500;TSS1500;TSS1500;TSS1500;TSS15	Island	34.64	4.22	38.53	5.66	-3.89	7.88E-05	2.29E-03
cg12880300		10	Intergenic	Island	78.47	5.58	82.36	3.97	-3.89	1.58E-03	2.38E-02
cg11827998	TMEM121	14	Body	Island	22.43	5.26	26.32	6.34	-3.89	3.87E-04	8.31E-03
cg00421335	ANKRD53;ANKRD53	2	Body;Body	Island	71.24	5.63	75.13	5.34	-3.89	3.23E-03	3.94E-02
cg19684151		5	Intergenic	Open sea	71.86	5.05	75.75	5.70	-3.89	5.69E-04	1.12E-02
cg26475541	BAIAP2;BAIAP2;BAIAP2;BAIAP2	17	Body;Body;Body;Body	Island	67.30	4.50	71.19	4.10	-3.89	4.91E-04	9.98E-03
cg20675971	SBNO2	19	5'UTR	Island	55.29	3.87	59.18	3.29	-3.89	1.48E-05	5.34E-04
cg08630923		11	Intergenic	Open sea	73.91	4.41	77.80	3.22	-3.89	1.54E-05	5.55E-04
cg04292941	DNHD1;DNHD1	11	TSS200;TSS200	Open sea	62.54	4.74	66.43	4.63	-3.89	3.88E-04	8.32E-03
cg16262392	SLC39A1;CRTC2	1	3'UTR;TSS1500	S_Shore	62.55	4.44	66.44	4.99	-3.89	4.72E-04	9.69E-03
cg26562772	DUSP10;DUSP10;DUSP10	1	5'UTR;Body;5'UTR	Open sea	44.48	4.76	48.37	4.81	-3.89	1.10E-03	1.83E-02
cg07353266		11	Intergenic	Open sea	40.07	3.88	43.96	5.40	-3.89	1.23E-04	3.32E-03
cg07464859	C16orf45	16	TSS1500	N_Shore	63.60	5.78	67.50	3.91	-3.89	4.70E-04	9.66E-03
cg10911004		3	Intergenic	S_Shelf	80.87	5.30	84.76	4.31	-3.89	4.34E-04	9.09E-03
cg25255113	AP2A2	11	Body	Open sea	72.95	5.49	76.84	4.46	-3.89	2.86E-03	3.62E-02
cg20225999		2	Intergenic	N_Shore	20.46	3.75	24.35	4.76	-3.89	4.68E-04	9.63E-03
cg19786988	TLN2	15	Body	Open sea	43.11	4.30	47.00	5.16	-3.89	8.56E-04	1.52E-02
cg19507032	OXER1	2	TSS200	Open sea	54.33	3.95	58.22	4.15	-3.89	1.67E-05	5.94E-04
cg23731742	NOC2L;SAMD11	1	3'UTR;3'UTR	S_Shore	56.19	3.76	60.08	3.62	-3.89	2.36E-05	8.08E-04
cg04289880	C21orf90;C21orf29;C21orf90	21	Body;Body;Body	Open sea	61.60	5.34	65.49	5.58	-3.89	1.07E-03	1.80E-02
cg27365324		15	Intergenic	N_Shore	46.31	5.41	50.20	4.30	-3.89	9.51E-05	2.68E-03
cg25486143	RASSF1;RASSF1	3	TSS200;TSS200	Island	28.77	3.78	32.66	4.36	-3.89	3.78E-05	1.22E-03
cg10259872	PDIA5;PDIA5	3	Body;Body	Open sea	46.33	3.84	50.22	3.01	-3.89	4.58E-05	1.44E-03
cg15399122		6	Intergenic	N_Shelf	64.97	4.59	68.86	3.92	-3.89	1.94E-04	4.83E-03
cg15467112	MLH3;MLH3	14	Body;Body	Open sea	58.39	6.18	62.27	4.60	-3.89	1.47E-03	2.27E-02
cg27133310	PLSCR1	3	TSS1500	S_Shore	68.84	3.39	72.73	2.47	-3.89	6.68E-08	3.38E-06

cg22672187	PLEKHH2	2	Body	Open sea	65.56	4.77	69.45	4.43	-3.89	1.89E-05	6.65E-04
cg12883767	SLC26A10	12	TSS1500	N_Shore	52.42	5.41	56.31	5.53	-3.89	1.80E-03	2.62E-02
cg00986350	ZFPM1	16	Body	Island	38.45	3.97	42.34	5.05	-3.89	1.90E-03	2.73E-02
cg11896550	SYT5	19	TSS200	S_Shore	22.32	5.02	26.20	5.21	-3.89	3.47E-04	7.62E-03
cg09868496	RERE;RERE;RERE	1	5'UTR;Body;Body	Open sea	68.96	5.98	72.85	5.79	-3.89	1.28E-03	2.06E-02
cg10056728	PA2G4;ERBB3	12	TSS1500;3'UTR	N_Shore	62.01	4.39	65.90	4.57	-3.89	2.63E-04	6.15E-03
cg22389215	PSMC2	7	TSS1500	S_Shore	74.81	4.64	78.69	3.88	-3.89	1.67E-05	5.96E-04
cg19062489	CYP4F22	19	Body	Island	36.62	5.67	40.50	5.33	-3.89	1.86E-03	2.69E-02
cg09900893	RPS6KA1	1	Body	Open sea	33.54	3.72	37.43	4.03	-3.89	1.04E-05	3.89E-04
cg07453773	ST5;ST5;ST5	11	5'UTR;5'UTR;5'UTR	Open sea	71.29	4.97	75.18	4.55	-3.89	1.04E-04	2.90E-03
cg14089425	KCNQ1;KCNQ1	11	Body;Body	Open sea	65.99	5.84	69.88	4.37	-3.88	1.44E-03	2.24E-02
cg27119612	TCF12;TCF12;TCF12;TCF12	15	Body;Body;Body;Body	Open sea	64.60	5.16	68.48	5.23	-3.88	2.72E-04	6.30E-03
cg13695288	PCBP3;PCBP3	21	Body;Body	Island	60.79	4.90	64.67	6.22	-3.88	2.27E-03	3.09E-02
cg17862558	TET2;TET2	4	5'UTR;5'UTR	Open sea	57.10	6.02	60.99	5.29	-3.88	2.75E-04	6.35E-03
cg03715182		16	Intergenic	Open sea	74.37	6.46	78.25	7.13	-3.88	4.16E-03	4.69E-02
cg03806501		7	Intergenic	Open sea	47.97	7.79	51.86	6.21	-3.88	1.51E-03	2.32E-02
cg18618206	NXN	17	Body	Open sea	65.14	6.81	69.02	7.02	-3.88	8.03E-04	1.45E-02
cg09984096	MTMR7	8	Body	Open sea	32.54	5.40	36.43	6.60	-3.88	3.14E-03	3.86E-02
cg19868495		13	Intergenic	Open sea	61.68	4.70	65.56	5.07	-3.88	1.03E-04	2.87E-03
cg05521619	MAPK8IP2;MAPK8IP2	22	Body;Body	Island	59.02	5.12	62.90	5.79	-3.88	3.41E-03	4.10E-02
cg08638929	TSPAN1	1	TSS1500	Open sea	71.61	5.26	75.49	4.81	-3.88	1.45E-03	2.25E-02
cg20459495		2	Intergenic	Open sea	47.53	6.40	51.41	6.02	-3.88	2.03E-03	2.86E-02
cg04865290	TMEM110	3	Body	N_Shelf	35.81	6.24	39.69	4.26	-3.88	3.70E-03	4.33E-02
cg09411922		10	Intergenic	S_Shelf	58.93	4.70	62.81	4.64	-3.88	6.50E-04	1.24E-02
cg01966105	C16orf79	16	TSS1500	N_Shelf	68.64	4.80	72.52	4.95	-3.88	7.05E-05	2.09E-03
cg04156219		8	Intergenic	Open sea	48.75	4.03	52.63	4.93	-3.88	3.03E-06	1.26E-04
cg08329473	AFAP1L2;AFAP1L2	10	Body;Body	Open sea	68.66	4.31	72.54	3.60	-3.88	2.83E-05	9.48E-04
cg27583037	SPTBN1	2	TSS1500	N_Shore	12.24	4.61	16.12	5.87	-3.88	2.48E-04	5.86E-03
cg03174043		2	Intergenic	N_Shore	34.37	4.78	38.25	4.40	-3.88	7.12E-04	1.33E-02
cg03041920	CLYBL	13	Body	Island	31.50	5.57	35.38	4.44	-3.88	1.02E-03	1.73E-02
cg05955332	CHCHD6	3	Body	Open sea	62.42	4.79	66.30	3.33	-3.88	6.45E-04	1.23E-02
cg19961545	GPR75;LOC100302652;LOC100302652	2	5'UTR;1stExon;5'UTR;1stExon	Island	30.69	4.03	34.58	4.16	-3.88	2.02E-04	4.99E-03
cg06868026		21	Intergenic	S_Shelf	50.31	4.55	54.19	5.38	-3.88	1.67E-03	2.48E-02
cg24210426		18	Intergenic	S_Shelf	61.78	5.00	65.66	4.39	-3.88	7.32E-05	2.15E-03
cg02023167		11	Intergenic	Island	47.98	4.34	51.86	4.70	-3.88	1.57E-04	4.07E-03
cg20645601	AMZ1	7	Body	Island	84.85	5.07	88.73	3.75	-3.88	2.73E-04	6.31E-03
cg03907995		4	Intergenic	N_Shelf	78.69	3.59	82.57	4.06	-3.88	1.29E-06	5.70E-05
cg26802256		7	Intergenic	Open sea	21.45	6.77	25.33	6.60	-3.88	2.62E-04	6.13E-03
cg14298200		8	Intergenic	Island	28.60	4.70	32.48	5.17	-3.88	1.09E-03	1.82E-02
cg26413708	TRERF1	6	5'UTR	Open sea	51.17	4.08	55.05	5.39	-3.88	4.49E-04	9.33E-03
cg24582851		2	Intergenic	Open sea	68.60	4.60	72.48	5.63	-3.88	1.10E-03	1.84E-02
cg02253535	PDXK	21	Body	N_Shore	29.82	4.04	33.70	5.27	-3.88	5.64E-04	1.11E-02
cg26655294	RGS7BP	5	3'UTR	Open sea	54.56	4.84	58.44	4.17	-3.88	4.64E-05	1.46E-03
cg15367592	FOLR3	11	TSS1500	Open sea	80.75	8.15	84.63	3.22	-3.88	4.16E-03	4.69E-02
cg13022461	NCAPH2;NCAPH2	22	Body;Body	S_Shelf	82.19	4.24	86.07	3.24	-3.88	1.50E-06	6.54E-05
cg14063008	DAB2IP	9	Body	N_Shore	52.37	5.87	56.25	5.48	-3.88	4.41E-03	4.88E-02
cg09343092	HOXA6	7	Body	Island	36.41	3.97	40.28	4.66	-3.88	3.62E-04	7.89E-03
cg19696870		17	Intergenic	Open sea	61.64	5.53	65.51	5.59	-3.88	5.49E-04	1.09E-02
cg24359392		1	Intergenic	S_Shelf	72.92	4.77	76.79	3.87	-3.88	1.60E-04	4.13E-03
cg04141796		5	Intergenic	N_Shore	30.67	4.66	34.55	4.68	-3.88	1.15E-04	3.14E-03
cg04233521	WASF2	1	Body	Open sea	52.23	5.26	56.11	5.85	-3.88	1.05E-05	3.94E-04
cg10505580		1	Intergenic	Island	23.02	3.99	26.89	4.46	-3.88	1.33E-05	4.87E-04
cg13046271	TCF7;TCF7;TCF7;TCF7;TCF7;TCF7;TCF7	5	TSS200;TSS1500;TSS1500;TSS200;Body;TS	Island	13.18	5.03	17.05	5.42	-3.88	1.10E-05	4.10E-04

cg27642784			3	Intergenic	N_Shore	31.71	4.45	35.59	4.96	-3.88	6.53E-04	1.24E-02
cg21131022	HAPLN4		19	Body	Island	49.26	6.30	53.14	5.60	-3.88	3.17E-04	7.10E-03
cg14539086	RDH16		12	TSS1500	Open sea	43.29	4.97	47.17	4.77	-3.88	3.66E-03	4.29E-02
cg01118868			20	Intergenic	N_Shore	57.07	4.62	60.94	3.82	-3.88	3.91E-04	8.37E-03
cg26102434			3	Intergenic	S_Shelf	74.51	7.43	78.39	6.67	-3.88	3.77E-03	4.38E-02
cg17689899			4	Intergenic	Open sea	68.44	4.85	72.32	5.02	-3.88	8.55E-04	1.52E-02
cg16008138		10-Mar	17	TSS200	S_Shore	52.35	4.17	56.23	5.56	-3.87	1.01E-03	1.72E-02
cg22111014	LIMD1		3	Body	Open sea	82.38	4.96	86.25	5.24	-3.87	5.01E-04	1.01E-02
cg14453012	C10orf110;C10orf110;C10orf110;C10c		10	Body;Body;Body;Body	Open sea	86.18	6.32	90.05	2.77	-3.87	7.04E-04	1.32E-02
cg10034990	FAM75A6		9	TSS1500	Open sea	51.97	5.73	55.85	4.69	-3.87	3.76E-04	8.12E-03
cg03460534	MACROD1		11	Body	Open sea	65.15	4.29	69.02	4.91	-3.87	2.92E-04	6.67E-03
cg19399100			4	Intergenic	Open sea	78.25	5.18	82.13	5.66	-3.87	1.70E-04	4.35E-03
cg08041279	POFUT2;POFUT2;POFUT2		21	3'UTR;Body;3'UTR	Island	84.30	3.91	88.17	3.35	-3.87	1.63E-05	5.84E-04
cg08046335	TUG1		22	Body	S_Shore	44.86	5.52	48.74	3.77	-3.87	8.41E-04	1.50E-02
cg20126546	ANO4		12	TSS200	Open sea	32.35	6.04	36.22	6.35	-3.87	1.20E-03	1.96E-02
cg20605818	EIF4EBP2		10	Body	S_Shore	46.32	5.33	50.19	4.95	-3.87	7.04E-04	1.32E-02
cg14515996			7	Intergenic	Open sea	30.10	7.20	33.97	7.43	-3.87	2.03E-03	2.86E-02
cg16525435			3	Intergenic	Open sea	42.77	3.05	46.65	4.15	-3.87	2.14E-05	7.44E-04
cg23549972	PXK		3	Body	Open sea	78.29	4.65	82.16	4.72	-3.87	2.18E-05	7.55E-04
cg25698842			8	Intergenic	N_Shelf	56.71	4.09	60.58	4.33	-3.87	1.96E-05	6.87E-04
cg04212092	AHDC1		1	5'UTR	Open sea	56.50	5.02	60.38	4.12	-3.87	8.20E-04	1.47E-02
cg24006721	RUNX3;RUNX3		1	TSS1500;Body	Island	21.69	3.31	25.56	4.08	-3.87	1.71E-05	6.07E-04
cg19182597	HOXC4;HOXC5;HOXC5		12	5'UTR;TSS200;Body	N_Shore	14.31	2.47	18.19	3.80	-3.87	8.83E-08	4.42E-06
cg03896542	GNAO1;GNAO1		16	Body;3'UTR	Open sea	72.16	5.69	76.03	5.20	-3.87	2.32E-03	3.13E-02
cg08881785			3	Intergenic	S_Shelf	62.74	5.22	66.61	4.08	-3.87	3.68E-04	7.99E-03
cg01308409	KL		13	Body	S_Shore	15.76	3.93	19.63	5.63	-3.87	1.67E-04	4.28E-03
cg14825033			4	Intergenic	N_Shelf	75.23	5.10	79.10	4.46	-3.87	1.34E-04	3.57E-03
cg01788444	ADPRH		3	3'UTR	Open sea	55.45	5.43	59.33	6.44	-3.87	9.57E-04	1.66E-02
cg25672060			10	Intergenic	Open sea	52.77	4.56	56.64	4.81	-3.87	8.07E-05	2.34E-03
cg25509184	CFTR		7	TSS1500	Open sea	35.66	5.81	39.53	7.70	-3.87	4.79E-04	9.79E-03
cg00366103			7	Intergenic	N_Shore	34.12	4.89	37.99	5.81	-3.87	2.83E-03	3.60E-02
cg12506930	ZNF154		19	TSS200	S_Shore	16.23	4.40	20.10	5.11	-3.87	4.16E-05	1.32E-03
cg13838578			6	Intergenic	Open sea	65.48	4.33	69.35	4.93	-3.87	1.98E-04	4.90E-03
cg14064694	HIST1H2BI		6	1stExon	Island	34.87	4.55	38.74	5.54	-3.87	1.28E-04	3.44E-03
cg14088142			5	Intergenic	Open sea	31.76	3.94	35.63	4.18	-3.87	8.55E-05	2.46E-03
cg02717454	CREBBP;CREBBP		16	Body;Body	N_Shore	18.33	4.04	22.20	5.49	-3.87	5.69E-04	1.12E-02
cg16012773	SLC26A7;SLC26A7		8	Body;Body	Open sea	67.25	5.78	71.12	5.54	-3.87	2.31E-03	3.13E-02
cg27644095	HCG22		6	Body	Open sea	65.78	5.47	69.65	5.31	-3.87	1.04E-03	1.76E-02
cg23390762	SERPINA11		14	Body	Open sea	52.27	5.34	56.14	4.86	-3.87	1.20E-03	1.96E-02
cg11133732			20	Intergenic	Island	54.86	5.35	58.73	6.25	-3.87	3.68E-04	7.98E-03
cg23943309			5	Intergenic	Open sea	79.17	4.74	83.04	3.49	-3.87	6.73E-04	1.27E-02
cg04474049	CAV1		7	TSS1500	N_Shore	49.80	5.97	53.67	5.59	-3.87	6.52E-04	1.24E-02
cg14059339	TRIM27		6	Body	Open sea	79.37	5.94	83.24	3.93	-3.87	4.76E-04	9.74E-03
cg21188652	RQCD1		2	Body	S_Shore	37.97	6.06	41.84	6.24	-3.87	3.04E-04	6.87E-03
cg04420862	IGF2BP2;IGF2BP2		3	Body;Body	Open sea	78.10	5.14	81.96	4.85	-3.87	4.17E-05	1.33E-03
cg09073701	NOTCH1		9	Body	Island	65.03	4.99	68.90	4.14	-3.87	1.87E-03	2.70E-02
cg11744075	RNF220		1	Body	Open sea	44.72	4.11	48.59	4.21	-3.87	7.85E-04	1.43E-02
cg24033859	MME;MME;MME;MME		3	TSS1500;TSS1500;TSS1500;TSS1500	N_Shore	53.72	5.05	57.59	5.06	-3.87	5.13E-05	1.59E-03
cg16461139	TBC1D16		17	Body	N_Shore	41.44	5.31	45.31	5.44	-3.87	4.21E-03	4.72E-02
cg18485596	SLC16A10		6	Body	Open sea	34.08	5.39	37.94	6.63	-3.87	4.51E-04	9.37E-03
cg14129790	DYNC1H1		14	Body	Open sea	74.99	4.25	78.86	3.84	-3.87	4.78E-05	1.50E-03
cg19408140			5	Intergenic	Open sea	49.30	4.73	53.17	4.18	-3.87	3.49E-04	7.66E-03
cg26458072	SEC31B		10	TSS200	Island	15.22	4.40	19.08	5.16	-3.87	1.26E-03	2.03E-02

cg00178427		1	Intergenic	N_Shore	65.96	6.00	69.82	3.92	-3.87	4.89E-04	9.95E-03
cg12513880		12	Intergenic	Island	12.32	3.93	16.19	4.98	-3.87	5.58E-07	2.59E-05
cg05898524	LMNA;LMNA;LMNA	1	TSS1500;TSS1500;TSS1500	N_Shore	21.06	4.42	24.93	5.36	-3.87	8.50E-04	1.52E-02
cg03701575		16	Intergenic	Open sea	18.94	3.66	22.81	3.57	-3.87	9.94E-06	3.75E-04
cg06493963	TFEC;TFEC	7	Body;Body	Open sea	33.48	4.17	37.34	5.83	-3.87	4.54E-04	9.41E-03
cg10547005	PLEKHB1;PLEKHB1;PLEKHB1;PLEKHB1	11	3'UTR;3'UTR;3'UTR;3'UTR;3'UTR	S_Shore	28.64	4.09	32.51	6.29	-3.87	2.82E-03	3.59E-02
cg26392708		10	Intergenic	Open sea	30.33	3.90	34.19	4.14	-3.87	5.43E-05	1.67E-03
cg14130459	SLC15A3;SLC15A3	11	1stExon;Body	Island	51.51	4.17	55.38	5.45	-3.87	3.60E-04	7.86E-03
cg17904988	NDUFS2;ADAMTS4;ADAMTS4	1	TSS1500;1stExon;5'UTR	N_Shelf	29.17	4.49	33.03	4.41	-3.87	3.08E-05	1.02E-03
cg11421097	RGL2;RGL2	6	Body;Body	N_Shelf	82.89	4.15	86.76	3.62	-3.87	1.57E-05	5.63E-04
cg19857227		14	Intergenic	Island	51.09	4.97	54.95	5.32	-3.87	2.07E-03	2.89E-02
cg01022012		2	Intergenic	N_Shore	70.28	4.67	74.15	4.60	-3.86	1.04E-03	1.77E-02
cg13743091	RALGPS1	9	Body	Open sea	73.38	5.35	77.24	5.42	-3.86	1.10E-03	1.84E-02
cg04332818	FGF2	4	TSS200	Island	19.37	4.02	23.24	3.65	-3.86	8.66E-07	3.92E-05
cg13892257	MIR1276;KLHL25	15	TSS200;5'UTR	S_Shore	33.19	4.30	37.06	4.60	-3.86	1.08E-03	1.82E-02
cg08439122	CAPN2;CAPN2	1	TSS1500;Body	N_Shore	76.86	5.71	80.72	5.10	-3.86	3.59E-05	1.17E-03
cg04149405	UBE2L6;UBE2L6	11	Body;5'UTR	N_Shelf	65.17	4.99	69.03	4.98	-3.86	2.43E-04	5.77E-03
cg00485050		16	Intergenic	S_Shelf	72.13	4.67	76.00	4.54	-3.86	3.84E-04	8.24E-03
cg14795560	PDIA4	7	3'UTR	Open sea	73.09	4.67	76.95	4.81	-3.86	7.09E-04	1.32E-02
cg24211908	GPRC5C;GPRC5C	17	3'UTR;3'UTR	S_Shore	23.92	4.22	27.79	5.01	-3.86	2.32E-03	3.14E-02
cg08495239	LOC619207	10	Body	Island	58.68	4.99	62.55	6.27	-3.86	6.02E-04	1.17E-02
cg10131972	TXNRD2	22	Body	Open sea	51.32	4.02	55.18	5.55	-3.86	2.45E-05	8.35E-04
cg14255417	TRIM8	10	3'UTR	S_Shore	17.52	4.56	21.38	5.41	-3.86	5.07E-04	1.02E-02
cg13924715		11	Intergenic	Open sea	38.58	4.54	42.45	4.75	-3.86	2.26E-03	3.08E-02
cg00723338	MYL5	4	Body	N_Shore	60.62	5.68	64.48	4.94	-3.86	2.36E-03	3.18E-02
cg22811212		3	Intergenic	Open sea	25.75	4.85	29.62	5.07	-3.86	2.47E-03	3.28E-02
cg26581503		22	Intergenic	N_Shore	38.53	4.98	42.39	5.29	-3.86	9.79E-04	1.68E-02
cg23863619	PRPF40A	2	Body	Open sea	69.04	4.88	72.90	4.19	-3.86	1.65E-05	5.88E-04
cg14918119		14	Intergenic	S_Shelf	77.31	3.84	81.17	3.32	-3.86	1.27E-05	4.67E-04
cg26188290	CINP	14	Body	N_Shelf	61.37	5.46	65.23	5.20	-3.86	1.42E-03	2.22E-02
cg22053945	HOXB3;HOXB3	17	5'UTR;1stExon	N_Shelf	41.84	4.99	45.70	5.49	-3.86	2.23E-03	3.05E-02
cg25498969	STAG1	3	Body	Open sea	73.95	6.69	77.81	5.16	-3.86	9.02E-04	1.59E-02
cg08971592		5	Intergenic	N_Shelf	55.73	6.07	59.59	5.94	-3.86	4.20E-03	4.71E-02
cg09084256	C22orf42	22	TSS200	Open sea	44.76	4.82	48.62	4.98	-3.86	1.58E-03	2.39E-02
cg17555373		13	Intergenic	Island	19.66	4.03	23.52	5.51	-3.86	8.51E-04	1.52E-02
cg15972148	RASA3	13	Body	S_Shore	61.42	5.75	65.28	4.93	-3.86	6.54E-05	1.96E-03
cg21375869	LOC145845	15	Body	N_Shore	23.09	4.62	26.94	5.14	-3.86	1.10E-04	3.04E-03
cg06943835	ATG2A	11	Body	Island	73.05	3.33	76.91	2.87	-3.86	6.64E-07	3.05E-05
cg27652925	C6orf27	6	Body	Open sea	40.55	4.73	44.40	5.08	-3.86	2.08E-03	2.91E-02
cg18190433		17	Intergenic	Open sea	64.61	5.27	68.47	4.65	-3.86	5.57E-04	1.10E-02
cg13001289	INPP5K;INPP5K;INPP5K	17	Body;Body;Body	N_Shelf	54.26	4.35	58.12	5.21	-3.86	1.30E-03	2.07E-02
cg18447740	OSCAR;OSCAR;OSCAR;OSCAR	19	TSS200;TSS200;TSS200;TSS200	N_Shore	48.76	5.12	52.62	4.53	-3.86	2.70E-03	3.48E-02
cg18335223	TNS3	7	Body	Open sea	32.59	4.26	36.45	4.81	-3.86	6.64E-04	1.26E-02
cg18129046	ARSJ	4	Body	N_Shelf	56.49	6.04	60.35	5.99	-3.86	4.35E-04	9.10E-03
cg06010032	FAM135B	8	5'UTR	Island	16.05	3.38	19.90	4.21	-3.86	3.78E-05	1.22E-03
cg24337235	EPHA6	3	Body	Open sea	69.72	4.52	73.57	4.01	-3.86	1.45E-04	3.81E-03
cg10085057	TRIM72	16	Body	Island	43.22	5.11	47.08	6.47	-3.86	3.30E-03	4.00E-02
cg26552163	IFT140;TMEM204	16	Body;Body	N_Shore	77.07	4.94	80.93	3.75	-3.86	3.09E-05	1.02E-03
cg08851727	ICMT	1	TSS1500	S_Shore	72.14	6.11	76.00	5.54	-3.86	3.02E-03	3.76E-02
cg02681400	BRD4;BRD4	19	Body;Body	Open sea	44.19	4.06	48.05	4.45	-3.86	8.09E-04	1.46E-02
cg09487449	KPTN	19	Body	N_Shelf	41.38	3.35	45.23	2.79	-3.86	1.58E-07	7.75E-06
cg03477679		11	Intergenic	S_Shore	25.78	5.04	29.64	5.93	-3.86	2.18E-03	3.00E-02
cg05033529	ZNF709	19	TSS200	S_Shore	76.52	5.46	80.38	4.86	-3.86	1.50E-03	2.31E-02

cg21121496	EPS8L1;EPS8L1	19	Body;Body	Island	7.56	2.60	11.42	6.63	-3.86	6.62E-04	1.26E-02
cg26477792		6	Intergenic	Island	40.99	5.19	44.85	5.26	-3.86	1.43E-03	2.22E-02
cg26863172	TMEM88B	1	1stExon	Island	64.43	5.66	68.29	5.97	-3.86	1.95E-03	2.77E-02
cg05985988	JARID2	6	Body	Open sea	46.14	5.15	50.00	5.29	-3.86	1.07E-03	1.80E-02
cg26249135	TNXB	6	Body	Open sea	60.85	6.30	64.70	5.03	-3.86	1.69E-04	4.32E-03
cg10177518		15	Intergenic	Open sea	53.67	6.78	57.53	7.27	-3.86	2.19E-03	3.01E-02
cg23660356	PPT2;PPT2	6	Body;Body	S_Shore	66.64	4.94	70.49	3.83	-3.86	4.48E-04	9.30E-03
cg15078681	AMIGO3;RNF123	3	1stExon;Body	Island	36.85	4.24	40.71	3.91	-3.86	7.29E-05	2.15E-03
cg15320854	SLC26A4;LOC286002	7	TSS200;Body	N_Shore	23.69	3.55	27.54	5.46	-3.86	6.37E-05	1.91E-03
cg07815131		6	Intergenic	N_Shore	38.58	4.51	42.43	5.58	-3.86	1.92E-04	4.78E-03
cg13710553		7	Intergenic	Open sea	19.73	4.54	23.58	4.06	-3.86	2.06E-05	7.17E-04
cg18563005		12	Intergenic	Open sea	78.83	4.88	82.68	3.03	-3.85	5.48E-04	1.09E-02
cg07018026	RNF220	1	Body	Open sea	84.67	4.77	88.52	3.01	-3.85	1.54E-04	4.01E-03
cg21090075	SLC16A8	22	Body	Island	53.56	4.07	57.41	3.73	-3.85	3.21E-05	1.06E-03
cg02039404		2	Intergenic	N_Shore	44.82	5.22	48.67	5.75	-3.85	1.40E-03	2.19E-02
cg02306639	ZNF860	3	5'UTR	S_Shelf	47.78	5.13	51.64	4.75	-3.85	2.91E-04	6.64E-03
cg00298065	ZNF274;ZNF274;ZNF274	19	Body;Body;5'UTR	Island	48.85	5.22	52.71	5.31	-3.85	2.69E-03	3.47E-02
cg09337852	CALD1;CALD1;CALD1;CALD1;CALD1;C/	7	5'UTR;1stExon;1stExon;1stExon;5'UTR;5'U	Open sea	37.56	5.59	41.42	5.48	-3.85	2.22E-03	3.04E-02
cg17802364	FARP1	13	3'UTR	Open sea	63.76	4.59	67.62	4.22	-3.85	7.92E-04	1.44E-02
cg21581873	PLEKHA6	1	TSS1500	Open sea	37.11	4.89	40.97	4.33	-3.85	6.03E-04	1.17E-02
cg00897799	ESD	13	5'UTR	N_Shelf	74.70	4.27	78.55	4.62	-3.85	7.66E-04	1.40E-02
cg21123417	SLC12A7	5	Body	S_Shelf	18.75	4.48	22.60	5.84	-3.85	2.80E-03	3.57E-02
cg20510012	PHLDB1;PHLDB1;PHLDB1	11	TSS200;TSS1500;TSS1500	N_Shore	61.36	6.57	65.21	7.53	-3.85	8.37E-04	1.50E-02
cg15340178	SEMA4G	10	TSS1500	S_Shelf	64.56	3.90	68.41	4.73	-3.85	2.42E-04	5.76E-03
cg15124820		3	Intergenic	S_Shore	49.62	4.53	53.47	4.72	-3.85	4.02E-04	8.55E-03
cg16954236	RAB12	18	3'UTR	Open sea	76.04	6.54	79.89	7.60	-3.85	1.40E-03	2.20E-02
cg08509427	CSNK2B	6	Body	S_Shelf	78.03	3.79	81.88	3.91	-3.85	6.58E-06	2.57E-04
cg26574408	FAM124A	13	Body	Open sea	66.07	4.66	69.93	4.69	-3.85	5.42E-04	1.08E-02
cg19871173		1	Intergenic	Open sea	57.68	6.05	61.53	6.59	-3.85	4.49E-03	4.94E-02
cg26917367	PEG3;PEG3;ZIM2;ZIM2;PEG3;ZIM2;MI	19	TSS1500;TSS1500;TSS1500;TSS1500;TSS15	S_Shore	72.15	4.23	76.00	3.49	-3.85	1.14E-05	4.24E-04
cg05592114	C1R	12	Body	Open sea	31.22	3.66	35.07	4.86	-3.85	2.03E-04	4.99E-03
cg02686793		6	Intergenic	Open sea	25.47	4.78	29.32	5.38	-3.85	2.68E-03	3.47E-02
cg17771652	VCAN;VCAN;VCAN;VCAN	5	5'UTR;5'UTR;5'UTR;5'UTR	N_Shore	21.79	4.53	25.64	4.98	-3.85	8.35E-04	1.49E-02
cg08846467	IFT140	16	Body	Open sea	75.03	4.54	78.88	4.54	-3.85	5.98E-05	1.82E-03
cg25261694	BMPR1A	10	5'UTR	Open sea	65.32	6.76	69.17	5.40	-3.85	2.33E-03	3.14E-02
cg11722581	DIP2C;C10orf108;C10orf108	10	Body;TSS200;TSS200	Open sea	25.73	5.17	29.57	4.89	-3.85	1.65E-03	2.47E-02
cg11952239	TSG1	6	Body	Open sea	81.40	5.88	85.24	3.73	-3.85	2.92E-04	6.66E-03
cg08104568	SLC7A8;SLC7A8	14	Body;TSS200	Open sea	25.80	3.65	29.65	4.80	-3.85	2.19E-04	5.32E-03
cg18447645	CNPY1	7	5'UTR	S_Shore	43.88	4.62	47.73	4.32	-3.85	2.56E-05	8.68E-04
cg08272151	SYNPO	5	Body	Island	5.64	2.48	9.48	5.89	-3.85	2.85E-05	9.54E-04
cg06559389	CACNA2D1	7	Body	Open sea	46.75	4.47	50.59	4.43	-3.85	8.86E-05	2.53E-03
cg03593189	STX1A;STX1A	7	Body;Body	Island	59.59	4.41	63.44	3.71	-3.85	1.15E-05	4.28E-04
cg25117523	CYTH1;CYTH1	17	Body;Body	Open sea	63.55	3.96	67.40	3.92	-3.85	8.20E-05	2.37E-03
cg03982787	RARA	17	5'UTR	Open sea	46.81	4.91	50.65	4.68	-3.85	4.23E-04	8.90E-03
cg16392797	LOC100128788;LOC100128788	16	TSS200;Body	Open sea	89.27	5.77	93.11	1.65	-3.85	8.64E-05	2.48E-03
cg02481000	PRKCZ;PRKCZ;PRKCZ	1	Body;Body;Body	Island	78.85	3.78	82.70	2.69	-3.85	2.07E-06	8.85E-05
cg11650460	RAB35;RAB35	12	Body;Body	S_Shore	51.61	5.25	55.45	4.15	-3.85	2.35E-03	3.17E-02
cg07191152		1	Intergenic	Open sea	43.84	4.59	47.69	4.76	-3.85	7.18E-04	1.34E-02
cg27064708	RAD51L1	14	Body	Open sea	46.10	4.06	49.94	5.28	-3.85	3.41E-03	4.10E-02
cg18346402	TIGD1	2	1stExon	N_Shelf	57.75	5.32	61.59	3.92	-3.85	3.89E-04	8.34E-03
cg12707135		6	Intergenic	Open sea	28.08	5.26	31.92	6.58	-3.85	6.75E-04	1.28E-02
cg24850121	SRGAP3;SRGAP3	3	Body;Body	Island	45.67	4.20	49.52	4.74	-3.84	2.95E-05	9.82E-04
cg04274288		6	Intergenic	Island	14.26	3.41	18.11	4.91	-3.84	9.43E-05	2.67E-03

cg11024722	UCN3	10	TSS200	Open sea	46.49	4.99	50.34	5.62	-3.84	7.57E-04	1.39E-02
cg17966227		1	Intergenic	N_Shelf	39.24	5.10	43.09	6.49	-3.84	3.96E-03	4.54E-02
cg01596289	PACRG;PACRG;PACRG	6	Body;Body;Body	N_Shore	74.71	5.39	78.56	5.52	-3.84	4.62E-04	9.54E-03
cg00929332	TEX14;TEX14	17	Body;Body	Open sea	75.90	3.97	79.74	4.46	-3.84	1.35E-04	3.58E-03
cg11496593	TBC1D16	17	Body	Open sea	72.78	4.40	76.63	4.98	-3.84	4.96E-05	1.54E-03
cg03656632		7	Intergenic	S_Shore	40.91	4.08	44.76	4.53	-3.84	7.66E-04	1.40E-02
cg08672695		6	Intergenic	N_Shelf	65.12	4.23	68.96	4.17	-3.84	2.17E-05	7.51E-04
cg26332630	C14orf2;C14orf2	14	3'UTR;3'UTR	Open sea	69.68	4.77	73.52	4.09	-3.84	6.00E-04	1.17E-02
cg04478080		8	Intergenic	Open sea	29.75	4.61	33.60	5.20	-3.84	6.91E-04	1.30E-02
cg16616957	EYS	6	Body	Open sea	83.43	6.35	87.27	4.35	-3.84	8.09E-04	1.46E-02
cg23974739	SRD5A1	5	Body	S_Shelf	51.13	4.63	54.97	5.57	-3.84	3.46E-03	4.14E-02
cg23413954	SERINC5	5	Body	Open sea	76.83	4.87	80.68	4.90	-3.84	5.55E-04	1.10E-02
cg26154888	RUNX2;RUNX2	6	Body;Body	Open sea	81.34	5.49	85.18	5.04	-3.84	1.40E-04	3.69E-03
cg23686352	RSPO2	8	Body	Open sea	59.93	5.20	63.78	5.41	-3.84	2.72E-03	3.50E-02
cg17045844	C4orf6	4	TSS200	Open sea	35.90	4.94	39.74	5.22	-3.84	7.82E-04	1.42E-02
cg13474734	C15orf52	15	Body	Open sea	57.84	3.30	61.68	3.85	-3.84	7.49E-06	2.89E-04
cg10970751	DGKH;DGKH	13	Body;Body	Open sea	83.16	3.22	87.01	2.27	-3.84	3.80E-08	1.95E-06
cg14539856	PNLIPRP3	10	TSS1500	Open sea	65.40	6.39	69.24	4.48	-3.84	6.24E-05	1.88E-03
cg05019016		14	Intergenic	N_Shelf	69.32	5.71	73.16	5.07	-3.84	9.38E-04	1.63E-02
cg01876288	LOC154822	7	TSS200	S_Shore	63.58	5.69	67.42	4.79	-3.84	1.63E-03	2.44E-02
cg23072973	C11orf49;C11orf49;C11orf49;C11orf4	11	3'UTR;3'UTR;Body;3'UTR	Open sea	53.47	3.84	57.31	4.17	-3.84	1.14E-04	3.13E-03
cg03352717	WFIKKN1	16	TSS1500	S_Shore	28.00	3.95	31.84	4.90	-3.84	6.86E-04	1.29E-02
cg13707189	FAM154A	9	Body	S_Shore	77.11	5.45	80.95	5.04	-3.84	1.97E-04	4.88E-03
cg04351807	LDLRAD3	11	Body	Open sea	76.99	6.85	80.83	5.95	-3.84	2.13E-03	2.95E-02
cg19497998		6	Intergenic	N_Shore	56.86	5.18	60.70	4.57	-3.84	1.09E-03	1.83E-02
cg04488373		7	Intergenic	N_Shore	8.15	4.51	11.99	6.24	-3.84	4.61E-04	9.51E-03
cg22788657		6	Intergenic	Open sea	28.58	4.60	32.42	4.99	-3.84	1.78E-04	4.50E-03
cg13085980	PLXNC1	12	Body	S_Shore	9.48	4.05	13.32	5.56	-3.84	1.60E-06	6.97E-05
cg10040530		14	Intergenic	Open sea	41.84	5.17	45.68	5.15	-3.84	2.61E-03	3.40E-02
cg00272903		16	Intergenic	N_Shelf	63.85	6.07	67.69	5.92	-3.84	4.15E-03	4.68E-02
cg24003216	VPS13B;VPS13B	8	Body;Body	Open sea	81.91	6.22	85.75	4.87	-3.84	1.68E-03	2.49E-02
cg09040752	AOC3	17	TSS200	Open sea	49.83	4.50	53.67	4.29	-3.84	8.19E-05	2.37E-03
cg17732690	RFPL3	22	TSS200	Open sea	83.69	4.27	87.52	2.70	-3.84	1.76E-05	6.23E-04
cg01912119		5	Intergenic	Open sea	46.56	4.62	50.40	4.63	-3.84	1.07E-03	1.80E-02
cg26952697		6	Intergenic	S_Shore	39.49	4.44	43.32	4.54	-3.84	4.40E-04	9.18E-03
cg08644058		17	Intergenic	Open sea	69.37	6.17	73.21	5.46	-3.84	2.33E-03	3.15E-02
cg17001958		7	Intergenic	Open sea	62.55	4.13	66.39	4.78	-3.84	1.10E-03	1.84E-02
cg12864235	CDH9	5	TSS200	Open sea	25.44	4.31	29.28	5.71	-3.84	3.48E-03	4.15E-02
cg13138089	ECEL1P2	2	TSS200	Island	48.18	5.15	52.02	4.34	-3.84	1.23E-03	2.00E-02
cg19645788	AKT1;AKT1;AKT1	14	Body;Body;Body	Island	64.53	3.94	68.37	4.30	-3.84	8.21E-05	2.37E-03
cg01101677		2	Intergenic	N_Shore	74.59	4.77	78.42	4.92	-3.84	4.79E-04	9.80E-03
cg26795312	C8orf77	8	Body	S_Shelf	53.84	4.89	57.68	5.27	-3.84	4.22E-05	1.34E-03
cg02193425	FAM50B	6	TSS1500	N_Shore	72.59	3.75	76.42	3.62	-3.84	2.56E-06	1.07E-04
cg24047736	GEMIN6	2	Body	Open sea	60.09	4.98	63.92	4.36	-3.84	3.84E-05	1.24E-03
cg06422471	SHANK3	22	Body	Island	46.96	5.56	50.79	5.21	-3.83	4.49E-03	4.94E-02
cg19409133	SNHG3-RCC1;RCC1;SNHG3-RCC1;RCC	1	Body;TSS1500;Body;TSS1500;5'UTR;TSS15	N_Shore	42.88	5.59	46.71	5.06	-3.83	2.35E-04	5.63E-03
cg24664957	VPS37B	12	TSS1500	S_Shore	77.00	3.51	80.83	3.47	-3.83	3.61E-06	1.48E-04
cg19598584	HOXA2	7	TSS1500	N_Shore	18.13	4.33	21.97	3.75	-3.83	3.03E-05	1.01E-03
cg27441354		7	Intergenic	Open sea	37.55	5.47	41.39	4.51	-3.83	9.96E-04	1.71E-02
cg22764186		4	Intergenic	Open sea	53.59	5.04	57.42	4.28	-3.83	1.04E-03	1.77E-02
cg26693601	WDR1;WDR1	4	TSS1500;TSS1500	S_Shore	56.59	6.98	60.43	6.24	-3.83	3.01E-03	3.75E-02
cg05860723	MYF6	12	Body	Island	21.33	4.15	25.16	4.58	-3.83	5.42E-06	2.15E-04
cg13320181	HSPB7	1	TSS200	Open sea	68.84	5.58	72.67	4.65	-3.83	4.39E-04	9.17E-03

cg21806100	IQGAP2	5	Body	Open sea	42.58	4.76	46.42	5.23	-3.83	3.00E-03	3.75E-02
cg11963006		1	Intergenic	N_Shore	34.64	4.11	38.47	4.23	-3.83	7.32E-05	2.15E-03
cg06287140	BCL7A;BCL7A	12	Body;Body	Open sea	50.54	5.39	54.37	4.27	-3.83	2.14E-04	5.21E-03
cg23126342	PCDH9;PCDH9	13	Body;Body	N_Shelf	63.33	6.22	67.16	6.92	-3.83	2.55E-03	3.34E-02
cg14851544	DYNC1H1	14	Body	Open sea	21.08	5.42	24.91	5.51	-3.83	1.06E-03	1.79E-02
cg02703695		11	Intergenic	Open sea	51.56	3.64	55.40	4.42	-3.83	2.38E-04	5.67E-03
cg27220999	ABLIM2;ABLIM2;ABLIM2;ABLIM2;ABL	4	Body;Body;Body;Body;Body;Body;Body	Open sea	60.42	7.31	64.26	5.53	-3.83	1.22E-03	1.99E-02
cg04875128	OTUD7A	15	Body	Island	36.49	4.56	40.32	4.17	-3.83	7.94E-06	3.05E-04
cg21577639	GPR110;GPR110	6	Body;Body	Open sea	77.65	3.53	81.48	3.93	-3.83	1.04E-05	3.90E-04
cg12790874	CLYBL	13	Body	Island	32.15	5.82	35.98	4.66	-3.83	1.08E-03	1.81E-02
cg18689929	AMACR;AMACR;AMACR;AMACR;AMA	5	Body;Body;Body;Body;Body;Body	N_Shelf	59.51	5.22	63.35	5.73	-3.83	1.87E-03	2.70E-02
cg07474266	ZCCHC14	16	Body	Open sea	70.67	5.42	74.51	4.71	-3.83	1.62E-03	2.43E-02
cg07107824		10	Intergenic	Open sea	57.00	6.70	60.83	5.53	-3.83	1.72E-03	2.54E-02
cg06444195		6	Intergenic	S_Shore	35.79	3.98	39.62	4.70	-3.83	3.96E-04	8.46E-03
cg24509398	EYA3	1	TSS1500	S_Shore	60.86	5.68	64.69	5.40	-3.83	3.52E-03	4.18E-02
cg23956760	SLC25A42	19	5'UTR	S_Shelf	46.05	5.70	49.88	6.06	-3.83	2.91E-03	3.67E-02
cg05573378	STARD9	15	Body	S_Shelf	73.70	4.58	77.53	4.65	-3.83	4.20E-04	8.86E-03
cg17143303		17	Intergenic	Island	44.95	4.60	48.78	4.26	-3.83	4.25E-04	8.94E-03
cg13622546	ASB6;ASB6	9	Body;Body	N_Shore	26.76	4.66	30.59	5.04	-3.83	9.36E-04	1.63E-02
cg24945462	LOC642852	21	Body	Island	34.59	5.95	38.42	5.58	-3.83	2.45E-03	3.26E-02
cg13529623	ANO8	19	Body	N_Shore	74.68	3.94	78.51	3.40	-3.83	4.16E-06	1.69E-04
cg19908188	MUC6	11	Body	S_Shore	70.90	5.02	74.73	4.02	-3.83	2.19E-03	3.01E-02
cg22076972	TRIP10	19	Body	Island	36.16	5.67	39.99	6.12	-3.83	4.50E-05	1.42E-03
cg08617916	RASSF5;RASSF5;RASSF5	1	Body;TSS200;Body	Island	5.44	2.01	9.27	2.95	-3.83	7.30E-12	4.36E-10
cg14270302		6	Intergenic	N_Shore	30.60	4.09	34.43	3.29	-3.83	3.88E-05	1.25E-03
cg09553651		15	Intergenic	Open sea	64.91	5.19	68.74	4.87	-3.83	1.37E-04	3.62E-03
cg03504078	PCDHB3	5	TSS200	N_Shore	36.33	4.69	40.16	5.48	-3.83	1.04E-03	1.76E-02
cg04875514	SOAT2	12	TSS200	Open sea	45.32	4.96	49.15	5.54	-3.83	2.80E-03	3.57E-02
cg03261929		13	Intergenic	N_Shore	21.26	3.75	25.09	4.23	-3.83	1.08E-04	2.98E-03
cg16875104	GARS	7	Body	S_Shore	14.58	3.26	18.40	4.59	-3.83	6.86E-05	2.04E-03
cg24366168	GNA11	19	Body	Island	65.84	4.64	69.67	3.49	-3.83	1.49E-04	3.89E-03
cg19850728	MINA;MINA;MINA	3	5'UTR;5'UTR;5'UTR	N_Shelf	64.56	5.24	68.39	3.58	-3.83	4.12E-05	1.31E-03
cg26429042	TRAPPC9;TRAPPC9	8	Body;Body	Open sea	60.27	4.67	64.10	4.48	-3.83	3.11E-04	6.99E-03
cg04908548	CDH15	16	Body	Open sea	47.90	4.23	51.73	5.65	-3.83	3.98E-03	4.55E-02
cg11752829	TAP2;TAP2	6	Body;Body	Open sea	62.93	4.19	66.76	4.48	-3.83	2.07E-04	5.08E-03
cg07297832	SEMA5B	3	Body	N_Shore	34.05	5.30	37.88	5.35	-3.83	3.45E-03	4.13E-02
cg03602014		5	Intergenic	N_Shore	48.77	4.66	52.60	6.52	-3.83	1.91E-03	2.74E-02
cg02102889	SLC7A11	4	TSS1500	Open sea	82.99	5.54	86.82	4.79	-3.83	6.53E-04	1.24E-02
cg25267258	OR14J1	6	1stExon	Open sea	77.20	4.36	81.03	2.58	-3.83	6.04E-06	2.37E-04
cg21433125	HDAC9;HDAC9;HDAC9;HDAC9;HDAC9	7	TSS200;5'UTR;5'UTR;TSS200;TSS200	Open sea	16.59	3.87	20.42	6.26	-3.83	1.43E-03	2.23E-02
cg04004787		7	Intergenic	Island	37.31	5.48	41.14	4.88	-3.83	2.72E-04	6.31E-03
cg02608124	MRGPRX4	11	TSS1500	Open sea	49.22	5.95	53.04	5.85	-3.83	3.21E-03	3.92E-02
cg18708402		4	Intergenic	Open sea	76.30	5.81	80.13	6.46	-3.83	4.40E-03	4.87E-02
cg22796353		7	Intergenic	Island	15.22	4.85	19.04	5.55	-3.83	2.59E-03	3.38E-02
cg22070406	ST8SIA3	18	TSS200	Island	24.53	4.00	28.35	5.11	-3.83	2.49E-05	8.48E-04
cg06694040	GALNTL4	11	Body	Open sea	56.46	4.77	60.29	5.45	-3.83	5.27E-05	1.62E-03
cg17878501		3	Intergenic	Open sea	73.36	4.75	77.18	6.04	-3.83	1.32E-04	3.52E-03
cg00650809	ANO8	19	Body	Island	48.38	6.05	52.20	6.75	-3.83	1.98E-03	2.81E-02
cg07040220	LOC285419	4	Body	Open sea	78.66	5.96	82.48	4.64	-3.83	1.13E-03	1.88E-02
cg01681525	BAHCC1	17	Body	Island	21.75	4.24	25.57	4.32	-3.83	9.40E-04	1.64E-02
cg24663638	HSD17B4	5	Body	Open sea	80.64	5.36	84.46	3.40	-3.83	1.78E-04	4.51E-03
cg20594304		12	Intergenic	N_Shore	46.16	6.44	49.98	6.53	-3.83	3.54E-03	4.20E-02
cg08286551	SBNO2	19	5'UTR	Island	43.57	4.47	47.40	3.83	-3.83	4.35E-04	9.10E-03

cg02393591	OCA2	15	Body	N_Shelf	73.37	4.81	77.20	4.31	-3.83	3.85E-05	1.24E-03
cg05492974		15	Intergenic	Open sea	65.13	3.47	68.95	3.79	-3.82	9.75E-06	3.68E-04
cg03138955	SH2D4A	8	TSS1500	N_Shore	43.34	5.52	47.16	5.33	-3.82	2.05E-03	2.88E-02
cg02477677	RARA;RARA;RARA;RARA	17	Body;Body;Body;Body	Open sea	56.91	4.29	60.74	3.69	-3.82	2.25E-04	5.43E-03
cg13426224	P2RX2;P2RX2;P2RX2;P2RX2;P2RX2;P2	12	Body;Body;Body;Body;Body;Body	Island	31.53	4.33	35.35	5.05	-3.82	3.74E-04	8.09E-03
cg00521239	PLEC1;PLEC1;PLEC1;PLEC1;PLEC1;PLE(	8	Body;Body;Body;Body;Body;Body;Body	N_Shore	29.25	4.64	33.07	3.79	-3.82	3.75E-05	1.21E-03
cg26919186	LOC390858	18	TSS1500	Open sea	46.86	5.30	50.69	6.34	-3.82	4.35E-03	4.83E-02
cg02712587	EIF2AK4	15	Body	N_Shore	69.77	6.46	73.59	5.06	-3.82	1.80E-03	2.63E-02
cg22577252	TIGIT	3	TSS1500	Open sea	66.26	4.63	70.08	4.86	-3.82	2.90E-04	6.62E-03
cg14686207	ACCSL	11	TSS1500	Open sea	61.41	3.97	65.23	4.23	-3.82	9.37E-06	3.55E-04
cg13643585	HOXB9	17	TSS200	Island	18.90	3.65	22.72	4.31	-3.82	4.17E-06	1.70E-04
cg21916479	LOC100131551	3	Body	Open sea	46.54	3.26	50.36	3.69	-3.82	4.78E-06	1.92E-04
cg13466901	OMP;CAPN5	11	1stExon;Body	Island	83.14	3.85	86.96	3.46	-3.82	7.11E-05	2.10E-03
cg02470587		12	Intergenic	S_Shelf	67.70	6.06	71.52	4.99	-3.82	1.33E-03	2.11E-02
cg14858786	PLEKHG6;PLEKHG6;PLEKHG6	12	Body;Body;Body	N_Shore	75.36	5.01	79.18	4.61	-3.82	1.11E-03	1.85E-02
cg07451641	SRGAP3;SRGAP3	3	Body;Body	S_Shore	22.85	6.53	26.67	9.02	-3.82	5.00E-04	1.01E-02
cg19791637		2	Intergenic	Open sea	65.08	5.76	68.90	5.77	-3.82	7.71E-05	2.25E-03
cg20268054	PKP3	11	TSS1500	N_Shore	58.24	4.10	62.06	5.73	-3.82	2.00E-03	2.83E-02
cg14384283		9	Intergenic	Open sea	76.43	5.79	80.25	3.88	-3.82	6.80E-05	2.02E-03
cg12297642	LACTB;LACTB	15	TSS1500;TSS1500	N_Shore	67.06	4.69	70.88	4.27	-3.82	8.18E-04	1.47E-02
cg22225849	NEDD9;NEDD9	6	Body;Body	Open sea	43.80	6.23	47.62	6.49	-3.82	5.85E-04	1.14E-02
cg12462771		2	Intergenic	Open sea	71.51	4.59	75.33	4.55	-3.82	4.77E-04	9.77E-03
cg13035743	PRRT1;PRRT1	6	1stExon;5'UTR	S_Shore	63.86	5.67	67.68	5.12	-3.82	8.49E-04	1.52E-02
cg26116639		4	Intergenic	N_Shelf	63.66	6.81	67.48	5.66	-3.82	2.75E-03	3.53E-02
cg22805491		14	Intergenic	Open sea	53.63	5.33	57.45	5.23	-3.82	1.82E-04	4.58E-03
cg03309176	GEFT;GEFT	12	TSS1500;TSS200	N_Shore	17.39	4.06	21.21	6.25	-3.82	4.51E-03	4.95E-02
cg23673344	KRT76	12	Body	Open sea	68.07	4.47	71.89	4.76	-3.82	8.90E-05	2.54E-03
cg11059934	LOC340357	8	TSS1500	Open sea	53.58	3.93	57.39	4.10	-3.82	1.21E-04	3.27E-03
cg15650209		5	Intergenic	Open sea	59.52	6.04	63.34	3.93	-3.82	9.69E-04	1.67E-02
cg15769472	MACROD1	11	Body	Island	53.52	5.13	57.34	5.16	-3.82	4.18E-03	4.70E-02
cg13942283	RBM20	10	Body	Open sea	25.32	5.17	29.14	6.14	-3.82	7.53E-04	1.39E-02
cg21590021	HEATR7A;HEATR7A;HEATR7A	8	Body;Body;Body	Open sea	37.92	5.09	41.74	5.87	-3.82	1.47E-03	2.27E-02
cg02889647	IVL	1	TSS1500	Open sea	73.89	5.08	77.71	4.67	-3.82	5.84E-05	1.78E-03
cg27278227	WIZ	19	Body	Open sea	64.74	3.07	68.56	3.68	-3.82	5.93E-06	2.34E-04
cg02470874	PPM1L	3	Body	S_Shore	32.14	5.04	35.95	6.07	-3.82	3.02E-03	3.76E-02
cg13240962	USH2A;USH2A	1	Body;Body	Open sea	44.78	6.18	48.60	4.73	-3.82	2.11E-03	2.94E-02
cg07017022	B3GNTL1	17	Body	N_Shelf	81.08	5.83	84.90	4.68	-3.82	1.03E-03	1.76E-02
cg10162691	RND2	17	1stExon	Island	43.18	5.46	47.00	4.63	-3.82	1.39E-03	2.18E-02
cg00250391		11	Intergenic	Open sea	48.75	5.28	52.56	5.32	-3.82	2.33E-03	3.14E-02
cg24527636		1	Intergenic	Open sea	71.27	6.66	75.08	6.37	-3.82	1.91E-03	2.74E-02
cg16616587	NAV3	12	Body	Open sea	80.16	4.47	83.98	3.61	-3.82	7.91E-05	2.30E-03
cg02245810	CPT1B;CHKB;CPT1B;CHKB-CPT1B;CPT:	22	TSS1500;3'UTR;TSS1500;Body;TSS1500;TS	S_Shore	81.12	4.49	84.94	3.59	-3.82	1.66E-04	4.25E-03
cg00559439	VGLL4;VGLL4	3	Body;Body	Open sea	82.83	5.90	86.65	3.57	-3.82	7.49E-04	1.38E-02
cg10456591	NCOA2	8	5'UTR	Open sea	59.77	5.63	63.58	5.73	-3.82	4.31E-03	4.80E-02
cg06691250		10	Intergenic	Open sea	57.81	5.39	61.63	4.41	-3.82	3.51E-03	4.18E-02
cg24253367	FLJ25006;KIAA0100	17	TSS1500;3'UTR	Open sea	51.15	3.17	54.97	3.11	-3.82	3.94E-06	1.61E-04
cg19316148	FABP3	1	TSS200	Open sea	22.60	3.99	26.42	5.29	-3.82	1.66E-04	4.25E-03
cg04834204	ANTXR1	2	Body	Open sea	40.68	4.66	44.50	4.75	-3.82	1.04E-03	1.76E-02
cg27181253	WNT2B;WNT2B;WNT2B	1	Body;1stExon;5'UTR	Island	42.63	4.67	46.44	5.21	-3.82	1.15E-03	1.90E-02
cg23016129	SPAG6;SPAG6	10	TSS200;TSS200	Island	18.34	3.55	22.16	4.34	-3.82	2.19E-06	9.35E-05
cg24232214	SGK269	15	TSS1500	Open sea	77.98	5.74	81.79	4.73	-3.82	5.47E-04	1.08E-02
cg12367274	C2orf51	2	Body	Open sea	60.29	3.71	64.11	3.69	-3.82	4.85E-06	1.95E-04
cg18378955		1	Intergenic	Open sea	76.10	4.83	79.92	2.81	-3.82	6.94E-05	2.06E-03

cg25669699		3	Intergenic	Open sea	80.41	4.29	84.22	3.70	-3.81	1.24E-05	4.57E-04
cg22675593	CCDC27	1	TSS1500	S_Shelf	60.71	5.00	64.53	5.05	-3.81	1.43E-03	2.23E-02
cg21771714		6	Intergenic	Open sea	28.87	4.93	32.68	6.27	-3.81	2.12E-03	2.95E-02
cg26661640	NRXN2;NRXN2	11	Body;Body	Open sea	59.37	4.37	63.19	3.95	-3.81	2.26E-06	9.59E-05
cg06251978	OR10J5	1	TSS1500	Open sea	60.19	5.94	64.00	5.49	-3.81	4.31E-04	9.03E-03
cg02988984		1	Intergenic	Open sea	48.23	4.57	52.05	4.38	-3.81	4.40E-04	9.18E-03
cg14386193	ALPK2;ALPK2	18	1stExon;5'UTR	Open sea	63.07	5.06	66.88	5.71	-3.81	2.34E-03	3.16E-02
cg02399275	SYCP3	12	Body	N_Shelf	78.26	5.64	82.08	5.79	-3.81	1.26E-03	2.03E-02
cg01976810	NXN	17	Body	Open sea	78.59	7.58	82.40	7.42	-3.81	2.45E-03	3.26E-02
cg15216812		3	Intergenic	N_Shelf	50.03	5.69	53.84	4.95	-3.81	2.87E-03	3.63E-02
cg21674927	IL1R2;IL1R2	2	Body;Body	Open sea	68.31	5.06	72.12	3.68	-3.81	1.24E-04	3.34E-03
cg26176141	TBCD	17	Body	Open sea	52.24	5.04	56.05	4.25	-3.81	2.20E-04	5.33E-03
cg13792147		16	Intergenic	Island	53.05	5.35	56.87	4.87	-3.81	8.18E-04	1.47E-02
cg21473741	ATP6V0A4;ATP6V0A4;ATP6V0A4	7	Body;Body;Body	Open sea	46.76	5.46	50.57	5.28	-3.81	3.86E-03	4.46E-02
cg09324018	FAM49A	2	5'UTR	Open sea	70.09	6.03	73.90	5.25	-3.81	5.43E-04	1.08E-02
cg18456689		15	Intergenic	Open sea	65.87	4.51	69.69	4.29	-3.81	1.30E-05	4.75E-04
cg22880122	HCG22	6	Body	Open sea	70.80	6.74	74.61	5.95	-3.81	2.33E-03	3.15E-02
cg26792948		16	Intergenic	N_Shelf	64.91	6.14	68.72	4.55	-3.81	3.96E-03	4.54E-02
cg24518098		1	Intergenic	S_Shore	38.37	3.93	42.18	4.60	-3.81	1.50E-04	3.92E-03
cg05530484	LOC162632	17	TSS1500	Open sea	72.46	5.31	76.27	5.29	-3.81	4.97E-05	1.55E-03
cg19200592	TRIM71	3	Body	Open sea	44.28	5.69	48.09	4.41	-3.81	1.66E-03	2.47E-02
cg18381051	PRDM16;PRDM16	1	Body;Body	Island	21.73	4.69	25.54	5.03	-3.81	1.94E-04	4.83E-03
cg08584759	C10orf47	10	Body	Island	51.65	4.94	55.46	5.86	-3.81	2.14E-03	2.97E-02
cg02058870	SIPA1L1	14	5'UTR	Open sea	23.95	5.38	27.76	6.10	-3.81	3.64E-03	4.27E-02
cg04204526	FLJ42875;FLJ42875	1	Body;Body	S_Shore	39.77	3.95	43.58	6.15	-3.81	1.15E-03	1.91E-02
cg10046893	SNORD114-30	14	TSS1500	Open sea	63.14	5.77	66.95	5.86	-3.81	1.65E-03	2.46E-02
cg25313090	NAT14	19	Body	Island	63.59	4.80	67.40	3.76	-3.81	6.21E-05	1.88E-03
cg00788177		6	Intergenic	Island	19.17	3.86	22.98	5.21	-3.81	1.14E-04	3.13E-03
cg09729511	EHMT2;EHMT2	6	Body;Body	N_Shore	80.75	4.39	84.56	3.88	-3.81	1.13E-04	3.09E-03
cg10959198	RGMA;RGMA	15	TSS1500;TSS1500	Island	21.98	4.41	25.79	5.14	-3.81	2.38E-03	3.19E-02
cg26666580	SNX29	16	Body	Open sea	40.16	4.42	43.97	5.06	-3.81	1.11E-04	3.06E-03
cg07360028	EMP1	12	5'UTR	Open sea	55.11	4.99	58.92	5.52	-3.81	6.93E-04	1.30E-02
cg11955845	MYH9	22	Body	Open sea	33.39	3.74	37.19	4.79	-3.81	5.41E-04	1.08E-02
cg26498574	OPN4;OPN4	10	3'UTR;3'UTR	Open sea	67.81	5.01	71.62	4.74	-3.81	9.64E-04	1.67E-02
cg18174000		10	Intergenic	Open sea	64.30	4.35	68.11	5.17	-3.81	1.25E-04	3.36E-03
cg14285533		7	Intergenic	N_Shore	7.34	4.69	11.15	4.80	-3.81	4.39E-05	1.39E-03
cg02924425	LOC100130522;LOC100130522;PARD6	18	Body;Body;3'UTR	N_Shelf	67.41	4.57	71.22	4.54	-3.81	5.28E-04	1.05E-02
cg06851971	PIGZ	3	Body	Open sea	50.77	4.83	54.57	4.69	-3.81	1.50E-03	2.30E-02
cg14592365		11	Intergenic	Open sea	54.37	4.00	58.18	4.59	-3.81	4.87E-06	1.95E-04
cg02071600	HOMER1;HOMER1	5	1stExon;5'UTR	N_Shore	27.63	3.89	31.44	4.95	-3.81	2.68E-04	6.23E-03
cg19059495		6	Intergenic	Island	28.70	5.55	32.51	4.96	-3.81	1.79E-03	2.62E-02
cg26503078		7	Intergenic	S_Shore	67.13	4.95	70.94	3.49	-3.81	5.03E-04	1.02E-02
cg23128495		12	Intergenic	Open sea	30.63	4.31	34.44	4.88	-3.81	3.80E-04	8.19E-03
cg08905629	LAMC2;LAMC2	1	Body;Body	Open sea	46.48	4.62	50.28	6.17	-3.81	4.46E-04	9.28E-03
cg04665604	DDAH2	6	Body	Island	34.99	6.46	38.79	5.68	-3.81	4.16E-03	4.69E-02
cg21946667	CNTNAP1	17	Body	Island	49.39	4.90	53.20	5.29	-3.81	1.51E-03	2.32E-02
cg02803486		16	Intergenic	S_Shore	75.86	4.77	79.67	3.95	-3.81	2.98E-04	6.76E-03
cg00356361	NFATC1;NFATC1;NFATC1;NFATC1	18	Body;Body;Body;Body	Island	59.18	5.53	62.99	5.62	-3.81	3.96E-03	4.54E-02
cg23847314	SEPT9;SEPT9;SEPT9;SEPT9;SEPT9	17	5'UTR;5'UTR;Body;Body;Body	N_Shelf	79.19	5.20	83.00	5.12	-3.81	3.31E-04	7.35E-03
cg16500916		16	Intergenic	N_Shore	66.05	5.24	69.86	3.86	-3.80	2.71E-04	6.28E-03
cg06633162	SYNJ2	6	Body	Open sea	60.47	7.34	64.27	6.59	-3.80	1.63E-03	2.44E-02
cg09471587	LOC407835	7	Body	Island	63.67	5.59	67.47	4.81	-3.80	3.35E-03	4.04E-02
cg02477557		21	Intergenic	Open sea	14.83	3.00	18.63	3.61	-3.80	7.26E-07	3.32E-05

cg11394361		17	Intergenic	Open sea	39.05	4.64	42.86	3.85	-3.80	7.04E-05	2.09E-03
cg13165758	GAB2	11	Body	Open sea	37.72	6.15	41.52	6.62	-3.80	1.45E-03	2.25E-02
cg02741882	SEZ6L2;SEZ6L2;SEZ6L2;SEZ6L2	16	Body;Body;Body;Body	Island	15.01	3.36	18.81	3.98	-3.80	1.09E-07	5.44E-06
cg15784814	NFATC1;NFATC1;NFATC1;NFATC1;NF/	18	Body;5'UTR;Body;Body;Body	Island	83.84	5.60	87.64	3.45	-3.80	7.77E-04	1.42E-02
cg27556150	LIPE	19	Body	S_Shore	55.46	3.50	59.26	4.26	-3.80	1.56E-05	5.61E-04
cg13174253	C8orf33	8	Body	S_Shore	64.29	5.02	68.09	5.23	-3.80	1.00E-03	1.72E-02
cg08796706	HLA-DPB1	6	Body	S_Shore	62.73	4.38	66.53	4.90	-3.80	1.07E-03	1.81E-02
cg04636269		17	Intergenic	Island	22.03	3.32	25.83	3.84	-3.80	1.45E-06	6.34E-05
cg13232249	FLRT2	14	5'UTR	Open sea	80.55	4.48	84.35	4.21	-3.80	5.59E-05	1.71E-03
cg24211976		15	Intergenic	N_Shore	27.64	3.81	31.44	5.49	-3.80	1.07E-03	1.80E-02
cg05462339	SIL1;SIL1	5	Body;Body	Open sea	51.22	4.57	55.02	4.39	-3.80	5.35E-05	1.65E-03
cg01552919	GAK	4	Body	S_Shore	65.98	4.60	69.78	3.90	-3.80	3.45E-04	7.59E-03
cg21391046		4	Intergenic	Open sea	84.17	5.84	87.97	4.87	-3.80	1.79E-03	2.62E-02
cg17552029	MPP2	17	TSS200	S_Shore	35.60	5.07	39.40	6.04	-3.80	1.12E-03	1.86E-02
cg17177528		7	Intergenic	N_Shelf	68.10	5.30	71.90	5.53	-3.80	1.02E-03	1.74E-02
cg20058744	C11orf66;C11orf66	11	Body;Body	Open sea	27.88	4.56	31.68	4.65	-3.80	8.67E-04	1.54E-02
cg04279449	TMEM80;TMEM80	11	3'UTR;3'UTR	N_Shore	63.42	3.91	67.22	4.17	-3.80	3.05E-04	6.89E-03
cg14189381		9	Intergenic	N_Shelf	77.08	4.97	80.88	4.28	-3.80	2.58E-04	6.06E-03
cg11363972	KCNQ1	11	TSS1500	Island	38.32	3.99	42.12	4.98	-3.80	2.14E-04	5.22E-03
cg19430694	HEATR2	7	Body	N_Shelf	39.78	4.31	43.58	4.15	-3.80	8.51E-04	1.52E-02
cg22870280	BCL2L10	15	Body	N_Shore	88.07	5.04	91.87	2.35	-3.80	4.20E-04	8.86E-03
cg05254095	ZDHHC7;ZDHHC7	16	3'UTR;3'UTR	Open sea	46.58	4.21	50.38	5.13	-3.80	8.98E-04	1.58E-02
cg13556079		10	Intergenic	N_Shore	46.29	3.34	50.09	2.59	-3.80	7.00E-07	3.21E-05
cg04370634	OSBPL10	3	Body	Open sea	72.54	4.56	76.34	5.38	-3.80	1.54E-03	2.35E-02
cg12233463		15	Intergenic	Open sea	30.81	5.92	34.61	4.18	-3.80	1.04E-03	1.76E-02
cg12596692	DAB1	1	5'UTR	Open sea	77.37	4.72	81.17	3.71	-3.80	4.05E-05	1.29E-03
cg15798890	NFATC1;NFATC1;NFATC1;NFATC1;NF/	18	Body;5'UTR;Body;Body;Body	Island	83.30	7.11	87.10	6.05	-3.80	2.37E-03	3.19E-02
cg25061755	STON1-GTF2A1L;STON1	2	Body;1stExon	Open sea	73.30	6.52	77.10	5.51	-3.80	1.38E-03	2.17E-02
cg16875182	LRRC2;TDGF1;TDGF1	3	5'UTR;1stExon;5'UTR	S_Shore	67.20	4.29	71.00	4.05	-3.80	3.65E-04	7.93E-03
cg13319757	COL12A1;COL12A1	6	Body;Body	Open sea	79.04	4.59	82.84	4.08	-3.80	4.08E-06	1.66E-04
cg09404516	SPTBN1;SPTBN1	2	Body;Body	Open sea	50.77	4.13	54.57	3.68	-3.80	5.82E-05	1.77E-03
cg16313343	BRF1	14	TSS1500	S_Shore	23.71	4.43	27.51	5.12	-3.80	3.64E-04	7.92E-03
cg13168698	SGK269	15	5'UTR	Open sea	73.74	5.05	77.54	3.98	-3.80	2.52E-04	5.94E-03
cg06958636	EFCAB2;EFCAB2;EFCAB2	1	Body;Body;Body	Open sea	59.18	4.50	62.97	4.41	-3.80	8.39E-05	2.42E-03
cg16062320		5	Intergenic	Open sea	37.38	4.88	41.18	4.74	-3.80	3.50E-04	7.67E-03
cg27583026	CLEC18B	16	TSS1500	Open sea	68.50	5.01	72.30	3.96	-3.80	1.38E-03	2.17E-02
cg07010088	LBXCOR1	15	TSS1500	Island	18.55	4.26	22.35	4.02	-3.80	4.24E-05	1.35E-03
cg04480280	LOC100130872-SPON2	4	Body	Island	83.53	5.42	87.33	3.86	-3.80	4.58E-05	1.44E-03
cg19227710		3	Intergenic	Open sea	45.30	6.00	49.10	5.49	-3.80	4.15E-03	4.68E-02
cg26914299	CADPS2;CADPS2;CADPS2	7	Body;Body;Body	Open sea	63.46	5.52	67.26	4.39	-3.80	5.42E-04	1.08E-02
cg01362358		17	Intergenic	N_Shelf	42.16	4.53	45.96	6.00	-3.80	2.89E-03	3.65E-02
cg16558485		7	Intergenic	Open sea	85.16	4.91	88.95	2.64	-3.80	1.11E-05	4.15E-04
cg15242223	GABBR1;GABBR1;GABBR1	6	Body;Body;Body	Open sea	67.95	3.86	71.74	4.29	-3.80	2.36E-04	5.63E-03
cg01968002	SNX33;SNX33	15	5'UTR;1stExon	S_Shore	58.60	4.46	62.40	3.85	-3.80	6.79E-05	2.02E-03
cg14192935	FLJ35024	9	Body	Open sea	61.16	4.78	64.95	4.30	-3.80	6.90E-05	2.05E-03
cg17692971	ZNF101	19	Body	S_Shore	64.06	4.44	67.86	3.92	-3.80	1.53E-04	3.98E-03
cg10990993	EPM2AIP1;MLH1	3	1stExon;TSS1500	N_Shore	39.44	5.24	43.24	6.87	-3.80	2.95E-03	3.70E-02
cg07841866	NOS3	7	Body	S_Shore	69.25	6.33	73.04	5.34	-3.80	2.73E-03	3.51E-02
cg24094775	TFEB;PGC	6	TSS1500;Body	S_Shore	77.42	3.89	81.22	3.24	-3.79	4.66E-07	2.18E-05
cg05369942		4	Intergenic	Open sea	13.73	3.26	17.53	3.43	-3.79	2.76E-06	1.16E-04
cg12063490	MAN2B1	19	Body	Island	31.90	4.09	35.70	4.41	-3.79	4.46E-04	9.27E-03
cg21636683	SLC25A2	5	TSS200	Island	62.14	6.40	65.93	6.79	-3.79	3.23E-03	3.94E-02
cg27417469	ADAM32	8	TSS200	N_Shore	37.44	5.13	41.24	6.55	-3.79	1.70E-03	2.52E-02

cg15805998		15	Intergenic	Open sea	71.50	4.57	75.30	3.54	-3.79	7.89E-05	2.29E-03
cg19892195	AGAP1;AGAP1	2	Body;Body	Open sea	65.00	4.23	68.80	3.97	-3.79	7.40E-07	3.38E-05
cg08865099	EVX1	7	TSS1500	N_Shore	34.18	5.84	37.97	5.01	-3.79	1.78E-03	2.60E-02
cg15653828	SPDEF	6	5'UTR	S_Shore	79.86	5.53	83.65	3.94	-3.79	7.29E-05	2.15E-03
cg11622330	REXO1	19	Body	N_Shelf	78.89	4.30	82.69	3.97	-3.79	1.17E-04	3.20E-03
cg03476105	ACN9	7	Body	Open sea	59.73	6.36	63.52	6.64	-3.79	3.22E-03	3.93E-02
cg24029050	SPRY4;SPRY4	5	3'UTR;3'UTR	Open sea	76.39	4.28	80.19	4.11	-3.79	4.32E-05	1.37E-03
cg04564481	CYP26C1	10	TSS200	Island	58.36	4.68	62.15	4.53	-3.79	1.96E-03	2.79E-02
cg21919857	SCGB3A1	5	Body	Island	8.19	1.77	11.98	4.46	-3.79	3.81E-08	1.96E-06
cg20919942	SSH2	17	Body	Island	17.90	6.10	21.69	7.57	-3.79	1.85E-03	2.67E-02
cg06576318	NDRG1;NDRG1	8	3'UTR;3'UTR	Open sea	32.34	4.82	36.13	5.13	-3.79	3.40E-03	4.09E-02
cg00133476		17	Intergenic	Open sea	60.87	3.94	64.67	3.14	-3.79	5.43E-05	1.67E-03
cg17445097	PHF12;PHF12	17	Body;Body	Open sea	67.54	6.58	71.33	5.71	-3.79	5.09E-04	1.03E-02
cg15337646	CHFR;CHFR;CHFR;CHFR;CHFR	12	TSS1500;TSS1500;TSS1500;TSS1500;TSS15	S_Shore	48.15	4.55	51.94	5.19	-3.79	3.57E-04	7.80E-03
cg03074210	PHRF1	11	Body	Open sea	32.66	3.99	36.46	5.37	-3.79	1.23E-03	2.00E-02
cg10363005	KIRREL3;KIRREL3	11	Body;Body	N_Shelf	56.43	5.38	60.22	5.38	-3.79	1.72E-03	2.54E-02
cg03157700	MAP1D	2	Body	Open sea	74.07	5.45	77.86	4.95	-3.79	1.00E-03	1.71E-02
cg08785871	INTS1	7	Body	N_Shelf	80.48	4.97	84.27	3.49	-3.79	7.37E-05	2.16E-03
cg25373579	VEGFA;VEGFA;VEGFA;VEGFA;VEGFA;\	6	3'UTR;3'UTR;3'UTR;3'UTR;3'UTR;3'UTR;Bo	Open sea	63.74	3.71	67.53	3.13	-3.79	6.96E-05	2.07E-03
cg07938743	OTX1	2	3'UTR	Island	48.16	4.40	51.95	5.52	-3.79	8.02E-04	1.45E-02
cg10453756	C6orf10	6	Body	Open sea	78.58	5.76	82.37	4.33	-3.79	5.60E-04	1.10E-02
cg23494863	STAR;STAR	8	TSS1500;TSS1500	Open sea	48.22	4.52	52.01	4.78	-3.79	1.95E-03	2.77E-02
cg23279335		11	Intergenic	Open sea	31.06	3.07	34.85	4.02	-3.79	4.63E-07	2.17E-05
cg21657059	SFRP2	4	TSS1500	N_Shore	47.92	3.36	51.71	4.28	-3.79	7.13E-06	2.77E-04
cg12636971	CD55;CD55	1	3'UTR;3'UTR	Open sea	79.93	6.35	83.72	5.15	-3.79	1.26E-03	2.03E-02
cg18261491	FAAH	1	TSS200	Island	27.83	4.92	31.62	5.59	-3.79	5.34E-05	1.64E-03
cg16484473		10	Intergenic	Open sea	72.10	5.76	75.88	4.55	-3.79	2.92E-03	3.67E-02
cg14724265	PPEF2;PPEF2	4	1stExon;5'UTR	Open sea	79.46	4.39	83.25	3.61	-3.79	1.19E-04	3.23E-03
cg26335633		7	Intergenic	Island	17.94	3.27	21.73	3.98	-3.79	8.47E-06	3.24E-04
cg00043004	NOXO1;NOXO1;NOXO1	16	Body;Body;Body	S_Shore	14.79	5.37	18.58	6.84	-3.79	2.48E-03	3.29E-02
cg23288099		11	Intergenic	Open sea	28.39	5.29	32.18	5.49	-3.79	2.00E-03	2.83E-02
cg00386961	CLCN6	1	Body	Open sea	79.34	5.13	83.13	3.39	-3.79	1.03E-03	1.75E-02
cg27195956	TRIM71	3	Body	N_Shore	73.22	4.40	77.00	4.56	-3.79	3.68E-04	7.99E-03
cg14598387	OR1N1	9	1stExon	Open sea	34.49	5.56	38.28	5.88	-3.79	5.51E-04	1.09E-02
cg10625575	C12orf4	12	Body	N_Shelf	66.78	4.18	70.57	4.07	-3.79	1.43E-04	3.75E-03
cg20503956	CA13	8	TSS1500	N_Shore	57.40	5.43	61.19	4.88	-3.79	6.09E-04	1.18E-02
cg23243608	PIGZ	3	Body	Open sea	65.84	5.72	69.63	4.34	-3.79	4.54E-03	4.97E-02
cg25994622	PGLYRP2	19	Body	Island	84.79	5.88	88.58	4.90	-3.79	1.39E-03	2.18E-02
cg08456334	PI4KB;PI4KB	1	1stExon;5'UTR	N_Shore	65.93	4.72	69.72	4.60	-3.79	8.15E-04	1.47E-02
cg08474478		4	Intergenic	Open sea	16.17	3.65	19.96	5.46	-3.79	4.44E-04	9.24E-03
cg04731038		17	Intergenic	Island	63.97	5.82	67.76	4.43	-3.79	1.38E-03	2.17E-02
cg00948524	RNF135;RNF135	17	TSS200;TSS200	N_Shore	38.23	4.40	42.01	5.18	-3.79	1.63E-04	4.19E-03
cg04376719	CNN2;CNN2	19	Body;Body	Island	23.79	6.32	27.57	6.83	-3.79	4.54E-03	4.98E-02
cg18049638		6	Intergenic	Open sea	17.33	4.06	21.11	4.88	-3.79	1.48E-04	3.86E-03
cg14659511	DOCK9;DOCK9	13	Body;Body	Open sea	55.46	4.65	59.25	3.51	-3.79	2.02E-04	4.98E-03
cg19938167		1	Intergenic	Open sea	65.82	6.51	69.61	5.69	-3.79	3.36E-03	4.05E-02
cg14074251	SPEG	2	TSS1500	N_Shore	61.18	5.29	64.97	6.04	-3.78	1.87E-03	2.69E-02
cg26174583	FOXF2	6	Body	N_Shore	55.51	5.53	59.30	5.35	-3.78	3.88E-03	4.47E-02
cg18603581		3	Intergenic	Open sea	83.25	5.01	87.04	4.72	-3.78	9.80E-04	1.69E-02
cg14237025	CCL16	17	TSS1500	Open sea	34.44	5.01	38.23	5.15	-3.78	3.28E-03	3.98E-02
cg24734184	PPP1R12A;PPP1R12A	12	Body;Body	Open sea	63.92	5.75	67.70	4.75	-3.78	1.51E-04	3.92E-03
cg07096305		6	Intergenic	Island	15.56	5.29	19.34	6.45	-3.78	1.02E-03	1.74E-02
cg25651608		3	Intergenic	Open sea	39.48	4.56	43.26	5.49	-3.78	2.31E-03	3.12E-02

cg24812167	SNAPC4	9	TSS1500	N_Shore	36.47	4.71	40.25	5.29	-3.78	5.25E-04	1.05E-02
cg06432342	CAPN11	6	Body	Open sea	70.81	4.78	74.59	4.51	-3.78	3.57E-04	7.79E-03
cg04600699		16	Intergenic	N_Shelf	48.56	5.15	52.35	5.26	-3.78	2.81E-03	3.58E-02
cg06954855	SLAMF8	1	Body	Open sea	42.50	4.67	46.29	4.61	-3.78	2.19E-04	5.32E-03
cg10861541	MAP2;MAP2;MAP2;MAP2	2	TSS1500;TSS1500;TSS1500;5'UTR	Open sea	68.75	5.69	72.54	6.25	-3.78	2.67E-03	3.46E-02
cg25966016	INPP5A	10	Body	S_Shelf	53.14	5.44	56.92	4.00	-3.78	7.59E-04	1.39E-02
cg18670076	LEPREL1;LEPREL1	3	3'UTR;3'UTR	Open sea	72.25	5.80	76.03	5.07	-3.78	7.31E-04	1.35E-02
cg03552317		10	Intergenic	S_Shelf	80.55	5.95	84.33	6.04	-3.78	1.05E-03	1.78E-02
cg13893782	PDE7B	6	Body	Open sea	56.01	5.89	59.79	5.20	-3.78	1.01E-03	1.72E-02
cg08043998	KDM2B;KDM2B	12	Body;Body	Island	71.66	4.23	75.44	3.79	-3.78	1.05E-04	2.92E-03
cg24601716	PDX1	13	Body	Island	23.88	4.48	27.66	5.61	-3.78	3.09E-03	3.82E-02
cg09658645	ENPP7	17	TSS200	Open sea	46.92	4.43	50.70	3.97	-3.78	4.87E-04	9.92E-03
cg02761778	NFAT5;NFAT5;NFAT5;NFAT5;NFAT5	16	5'UTR;Body;Body;5'UTR;Body	Open sea	72.08	4.04	75.86	4.07	-3.78	1.44E-04	3.79E-03
cg14526021	CUX1;CUX1;CUX1	7	Body;Body;Body	Open sea	84.87	5.17	88.65	3.73	-3.78	8.48E-04	1.51E-02
cg27474357		19	Intergenic	N_Shelf	66.81	6.28	70.59	4.91	-3.78	4.22E-03	4.73E-02
cg08246800	PLEKHB1;PLEKHB1	11	TSS1500;TSS1500	Open sea	53.66	5.03	57.43	5.77	-3.78	2.20E-03	3.02E-02
cg09951338	ARHGAP23	17	Body	S_Shore	62.50	4.65	66.28	4.93	-3.78	9.39E-04	1.64E-02
cg08063911	TCF7;TCF7;TCF7;TCF7;TCF7;TCF7;TCF7	5	5'UTR;TSS200;5'UTR;5'UTR;5'UTR;Body;TS	S_Shore	45.99	5.06	49.76	5.79	-3.78	8.46E-05	2.43E-03
cg13916885	EBF3	10	Body	Island	48.35	3.29	52.13	4.39	-3.78	1.52E-05	5.49E-04
cg08435131	PLAC9	10	TSS200	N_Shore	31.20	4.00	34.98	4.86	-3.78	2.56E-04	6.01E-03
cg10978401		7	Intergenic	S_Shelf	78.91	6.99	82.69	4.88	-3.78	5.79E-04	1.13E-02
cg20819482	TNKS	8	Body	Open sea	72.48	5.56	76.25	5.43	-3.78	3.74E-04	8.08E-03
cg16811455	KCNQ1;KCNQ1	11	Body;Body	Open sea	49.97	4.78	53.74	4.75	-3.78	1.27E-03	2.05E-02
cg03435979	GRHL1	2	3'UTR	Open sea	70.92	5.62	74.70	4.61	-3.78	2.32E-03	3.14E-02
cg26116326		5	Intergenic	Open sea	29.41	5.11	33.18	5.55	-3.78	4.27E-03	4.77E-02
cg12172441		6	Intergenic	S_Shore	19.25	4.12	23.03	4.19	-3.78	5.11E-04	1.03E-02
cg16406611	IFITM5	11	TSS1500	S_Shore	27.05	7.11	30.83	8.06	-3.78	4.43E-03	4.90E-02
cg19967668	PCDHB17	5	Body	N_Shore	19.16	4.64	22.94	6.30	-3.77	1.00E-03	1.71E-02
cg13320585	MIR1252;SYT1;SYT1;SYT1	12	TSS200;Body;Body;Body	Open sea	64.67	5.83	68.45	4.83	-3.77	3.17E-03	3.89E-02
cg11705747	UGGT1;UGGT1	2	Body;Body	Open sea	87.34	6.85	91.11	4.00	-3.77	9.06E-04	1.59E-02
cg18236734	HTR3E	3	TSS200	Open sea	40.39	6.42	44.16	5.52	-3.77	4.37E-03	4.85E-02
cg24686358	COL9A2	1	Body	Island	18.11	3.04	21.89	3.80	-3.77	1.46E-06	6.39E-05
cg02304233	BCL9	1	5'UTR	N_Shore	25.08	3.93	28.85	5.07	-3.77	7.31E-04	1.35E-02
cg08087868		16	Intergenic	Island	38.04	4.83	41.81	5.40	-3.77	3.95E-03	4.53E-02
cg16289618	LOC285830;LOC285830	6	Body;Body	Open sea	37.52	4.13	41.30	4.75	-3.77	6.45E-04	1.23E-02
cg04220416	C14orf180	14	TSS200	Open sea	25.40	3.37	29.17	4.01	-3.77	4.02E-05	1.29E-03
cg03737367	MIR150	19	TSS1500	Open sea	67.55	5.23	71.32	5.05	-3.77	2.52E-03	3.32E-02
cg06361845		9	Intergenic	N_Shore	62.27	6.15	66.05	5.36	-3.77	1.64E-03	2.45E-02
cg27168858	DHCR24	1	Body	N_Shore	34.66	4.81	38.43	5.16	-3.77	3.00E-03	3.75E-02
cg11820929	PPP3CA;PPP3CA;PPP3CA	4	Body;Body;Body	Open sea	73.49	4.34	77.26	4.95	-3.77	5.57E-05	1.70E-03
cg14676825	PROCA1	17	TSS200	Island	20.66	3.33	24.44	4.64	-3.77	5.40E-05	1.66E-03
cg17916916	ARSG;SLC16A6	17	5'UTR;5'UTR	N_Shore	44.50	4.28	48.27	5.91	-3.77	2.92E-04	6.66E-03
cg01881828	MFSD7	4	Body	N_Shore	69.30	4.16	73.08	3.82	-3.77	1.50E-05	5.41E-04
cg19230867		3	Intergenic	Open sea	28.34	4.75	32.11	5.50	-3.77	2.69E-03	3.47E-02
cg27052537		7	Intergenic	Open sea	67.92	5.44	71.69	4.26	-3.77	3.73E-03	4.35E-02
cg01416712	PHOX2B;PHOX2B	4	1stExon;5'UTR	N_Shore	19.27	3.59	23.04	4.56	-3.77	1.85E-05	6.51E-04
cg09233658		1	Intergenic	Open sea	52.43	3.78	56.20	4.47	-3.77	6.15E-05	1.86E-03
cg09985455		10	Intergenic	Open sea	37.71	4.02	41.49	5.94	-3.77	1.05E-03	1.78E-02
cg13716026	TMC1	9	5'UTR	Open sea	30.46	4.65	34.24	5.30	-3.77	2.40E-03	3.21E-02
cg26578156		16	Intergenic	S_Shelf	71.22	6.46	74.99	5.82	-3.77	7.17E-04	1.33E-02
cg18046394	WIPF3	7	Body	Island	53.94	4.79	57.71	4.37	-3.77	4.18E-04	8.81E-03
cg11870261	TRANK1	3	TSS200	Island	8.69	3.93	12.46	5.87	-3.77	1.18E-03	1.94E-02
cg08439276		2	Intergenic	Open sea	59.06	5.93	62.83	4.53	-3.77	2.85E-04	6.53E-03

cg19820609	NAT14;SSC5D	19	3'UTR;TSS1500	N_Shore	66.16	4.31	69.93	3.27	-3.77	3.23E-05	1.06E-03
cg21431929		4	Intergenic	Open sea	17.05	3.43	20.82	3.72	-3.77	2.64E-06	1.11E-04
cg13657659	FZD5	2	Body	Island	50.39	4.32	54.16	5.38	-3.77	1.92E-03	2.75E-02
cg00490004		3	Intergenic	S_Shelf	68.59	5.23	72.36	4.67	-3.77	1.01E-03	1.72E-02
cg01156077	LOC100132215	2	TSS1500	Island	24.63	3.55	28.40	5.31	-3.77	4.79E-04	9.79E-03
cg16201598	DNAJC12;DNAJC12	10	Body;Body	Open sea	68.53	5.33	72.30	4.20	-3.77	1.49E-03	2.29E-02
cg23777946	KCTD1;KCTD1;KCTD1	18	5'UTR;Body;5'UTR	N_Shore	46.22	4.89	49.99	5.24	-3.77	1.50E-03	2.31E-02
cg23729443	BGLAP	1	TSS200	N_Shelf	58.34	4.02	62.11	4.53	-3.77	2.77E-05	9.30E-04
cg25937451	C21orf29;KRTAP10-5	21	Body;TSS1500	Open sea	57.91	5.91	61.68	5.71	-3.77	1.30E-03	2.08E-02
cg22561727	DPP9	19	Body	N_Shelf	75.43	3.82	79.20	4.22	-3.77	6.80E-05	2.02E-03
cg03958979	NR2E1	6	TSS1500	Island	42.28	3.79	46.05	4.61	-3.77	1.09E-04	3.02E-03
cg22696984		2	Intergenic	Open sea	55.93	4.03	59.70	4.37	-3.77	2.15E-04	5.24E-03
cg11897387		5	Intergenic	Open sea	64.34	4.98	68.11	4.25	-3.77	7.63E-06	2.94E-04
cg08317252	STK11	19	Body	N_Shelf	63.03	3.77	66.80	4.20	-3.77	7.59E-05	2.22E-03
cg06749480	PBX1	1	Body	Open sea	87.91	2.99	91.67	2.06	-3.77	1.92E-11	1.13E-09
cg08535779		1	Intergenic	N_Shore	49.63	4.29	53.40	3.45	-3.77	1.95E-04	4.83E-03
cg23012310		14	Intergenic	N_Shore	69.33	5.52	73.10	6.50	-3.77	1.21E-03	1.98E-02
cg04674315		7	Intergenic	N_Shore	38.68	5.32	42.45	5.07	-3.77	1.86E-03	2.69E-02
cg25882256	RUNX3;RUNX3	1	TSS1500;Body	Island	21.76	3.75	25.52	3.75	-3.77	1.61E-04	4.15E-03
cg03875195	SLC30A3	2	TSS1500	Island	37.54	4.47	41.30	4.74	-3.77	2.93E-05	9.77E-04
cg04896534		4	Intergenic	Open sea	38.98	3.99	42.75	4.43	-3.77	1.35E-04	3.59E-03
cg09186818	C1QTNF5;C1QTNF5;MFRP;MFRP	11	1stExon;5'UTR;1stExon;5'UTR	Open sea	54.33	3.60	58.09	3.92	-3.77	1.06E-05	3.98E-04
cg11924260		12	Intergenic	N_Shelf	51.49	5.14	55.26	4.66	-3.77	6.99E-04	1.31E-02
cg09602452	AP2M1;AP2M1	3	Body;Body	S_Shelf	70.56	4.09	74.33	3.40	-3.77	3.35E-05	1.10E-03
cg04476626		5	Intergenic	Island	42.46	5.53	46.22	5.56	-3.77	2.02E-03	2.84E-02
cg12798970		17	Intergenic	Open sea	75.56	3.99	79.33	3.57	-3.77	2.96E-06	1.23E-04
cg19384697	UPK3B;UPK3B;UPK3B;UPK3B	7	5'UTR;5'UTR;1stExon;1stExon	Open sea	29.34	3.96	33.10	4.75	-3.77	2.63E-04	6.13E-03
cg14003039	VARS	6	Body	N_Shelf	73.68	4.25	77.45	3.94	-3.76	3.17E-05	1.05E-03
cg27496116	DNAJC15	13	Body	Open sea	79.52	4.98	83.29	3.19	-3.76	1.08E-04	2.98E-03
cg17469934	EPS8L2	11	Body	N_Shore	47.46	4.53	51.22	4.65	-3.76	3.83E-04	8.23E-03
cg25278343	ITGB7	12	Body	N_Shore	82.25	4.84	86.01	3.16	-3.76	1.24E-04	3.33E-03
cg15550100	ATG4B;ATG4B	2	Body;Body	Island	82.03	4.85	85.80	4.52	-3.76	4.54E-04	9.41E-03
cg05789704	ADAM32	8	TSS200	Island	12.83	2.83	16.59	4.97	-3.76	6.01E-06	2.37E-04
cg01259220	C7orf20	7	TSS1500	N_Shore	76.52	4.96	80.28	4.85	-3.76	5.65E-04	1.11E-02
cg07823496		5	Intergenic	Island	76.55	6.33	80.32	6.29	-3.76	3.69E-03	4.32E-02
cg10182697	NKX6-2	10	TSS1500	Island	26.92	5.37	30.68	6.45	-3.76	4.43E-03	4.89E-02
cg13924015		9	Intergenic	Open sea	68.97	4.46	72.73	3.55	-3.76	1.31E-04	3.50E-03
cg09105334		17	Intergenic	N_Shelf	55.19	3.77	58.95	4.09	-3.76	1.04E-04	2.90E-03
cg20689224	PZP	12	Body	Open sea	82.86	2.48	86.62	2.31	-3.76	1.35E-10	7.78E-09
cg22946774	MUC15;ANO3;MUC15;MUC15	11	3'UTR;Body;3'UTR;3'UTR	Open sea	64.94	4.78	68.71	3.52	-3.76	6.12E-04	1.18E-02
cg05037556	C2orf34	2	Body	Open sea	65.00	5.97	68.76	4.49	-3.76	6.50E-04	1.24E-02
cg11992351	TRPV3	17	Body	Island	7.77	5.85	11.53	7.14	-3.76	3.05E-03	3.79E-02
cg01271726		6	Intergenic	Open sea	86.06	4.47	89.82	3.18	-3.76	1.61E-04	4.15E-03
cg12608507	EEF1B2;EEF1B2;SNORD51;NDUFS1;SN	2	Body;Body;TSS1500;TSS1500;TSS1500;Boc	S_Shore	31.36	5.00	35.13	5.87	-3.76	1.99E-04	4.92E-03
cg22952787	CLN5	13	Body	S_Shore	29.67	5.91	33.43	4.71	-3.76	8.81E-04	1.56E-02
cg03536664	UQCRC1	3	3'UTR	S_Shelf	74.20	4.48	77.96	3.98	-3.76	7.50E-05	2.20E-03
cg27115990	OLA1;OLA1	2	Body;Body	Open sea	78.03	6.62	81.80	6.08	-3.76	1.52E-03	2.33E-02
cg19624681	MBNL2;MBNL2	13	5'UTR;5'UTR	Open sea	60.84	5.65	64.60	5.01	-3.76	1.48E-03	2.28E-02
cg02984184	LDLRAD3	11	Body	Island	40.62	5.07	44.38	4.64	-3.76	5.72E-04	1.12E-02
cg00781658	HNRNPUL1;HNRNPUL1	19	Body;Body	Open sea	45.53	5.02	49.29	4.51	-3.76	9.11E-04	1.60E-02
cg14350800	VAV2;VAV2	9	Body;Body	Open sea	53.49	3.87	57.25	3.69	-3.76	9.46E-05	2.67E-03
cg07200939	PLXNC1	12	3'UTR	Open sea	64.35	4.81	68.11	5.53	-3.76	7.24E-04	1.34E-02
cg22095128	STARD13;STARD13;STARD13	13	5'UTR;Body;Body	Open sea	57.22	4.66	60.98	5.66	-3.76	1.34E-03	2.13E-02

cg10679688	ZIC5	13	TSS1500	S_Shore	16.81	3.21	20.57	3.81	-3.76	9.79E-07	4.40E-05
cg11201401	SLC6A7	5	Body	Island	52.87	3.29	56.62	3.24	-3.76	1.29E-06	5.69E-05
cg25617012		4	Intergenic	S_Shore	9.65	2.99	13.41	4.53	-3.76	1.17E-06	5.21E-05
cg21121119	GNAT1;GNAT1	3	TSS200;TSS200	N_Shore	30.70	3.95	34.46	4.63	-3.76	1.34E-03	2.13E-02
cg25669249	FAM189A1	15	Body	Open sea	86.28	4.08	90.04	2.45	-3.76	6.79E-07	3.12E-05
cg03205252		16	Intergenic	Island	11.74	3.38	15.50	4.89	-3.76	2.89E-04	6.60E-03
cg13871633	ZNF662;ZNF662	3	TSS1500;TSS200	N_Shore	24.47	4.97	28.23	4.36	-3.76	4.90E-04	9.96E-03
cg02197228	ACCN4;ACCN4	2	3'UTR;3'UTR	N_Shore	60.84	4.02	64.60	4.18	-3.76	7.51E-05	2.20E-03
cg15231205		21	Intergenic	Island	85.79	5.05	89.55	3.71	-3.76	1.76E-04	4.46E-03
cg21620540	DKK3;DKK3;DKK3	11	TSS1500;TSS1500;TSS1500	S_Shore	56.65	5.50	60.41	5.96	-3.76	3.77E-04	8.14E-03
cg23987491	MAP1A	15	Body	Open sea	73.04	5.16	76.80	4.51	-3.76	1.31E-03	2.09E-02
cg12213930	CPNE4	3	5'UTR	N_Shelf	52.90	5.37	56.66	5.46	-3.76	6.19E-04	1.19E-02
cg01373248	SATB1	3	TSS200	Open sea	55.41	4.72	59.16	5.45	-3.76	3.22E-05	1.06E-03
cg18479961	MIR671;CHPF2	7	TSS200;Body	S_Shore	82.40	3.24	86.16	2.60	-3.76	2.68E-08	1.39E-06
cg02647520	HSD17B8	6	Body	S_Shore	62.54	4.33	66.30	3.68	-3.76	8.76E-06	3.34E-04
cg05141384		18	Intergenic	N_Shore	45.48	3.99	49.23	3.55	-3.76	8.46E-06	3.24E-04
cg17421890	KLC2;KLC2;KLC2;KLC2	11	Body;Body;Body;Body	S_Shore	46.08	4.42	49.84	5.01	-3.76	3.09E-04	6.95E-03
cg00487155	LOC100129637	16	TSS1500	N_Shore	74.99	4.29	78.74	3.89	-3.76	1.00E-04	2.81E-03
cg20335293		2	Intergenic	Open sea	65.77	4.70	69.52	4.89	-3.76	2.96E-04	6.72E-03
cg18252037	RAI14;RAI14;RAI14;RAI14;RAI14;RAI1	5	Body;Body;Body;Body;Body;Body	Open sea	54.00	5.40	57.76	6.91	-3.76	3.91E-03	4.50E-02
cg16570669		11	Intergenic	Open sea	65.54	4.62	69.30	4.18	-3.76	8.80E-05	2.52E-03
cg18141295	CDC73	1	Body	Open sea	69.83	5.25	73.59	3.65	-3.76	1.42E-03	2.21E-02
cg12243007	RPH3AL	17	5'UTR	N_Shore	43.14	3.43	46.90	4.58	-3.76	4.12E-06	1.67E-04
cg20391833	RPS6KA2	6	Body	Open sea	37.19	4.10	40.94	4.88	-3.75	2.61E-04	6.10E-03
cg06686789	SLC18A1;SLC18A1;SLC18A1;SLC18A1	8	TSS1500;TSS1500;TSS1500;TSS1500	Open sea	52.72	4.87	56.48	4.66	-3.75	8.39E-04	1.50E-02
cg02977810	RNF13;RNF13	3	Body;Body	Open sea	79.16	3.44	82.92	3.46	-3.75	6.34E-07	2.92E-05
cg12799029	ABR;ABR;ABR	17	Body;Body;Body	S_Shelf	50.36	5.18	54.11	5.92	-3.75	1.30E-03	2.08E-02
cg14562327		1	Intergenic	N_Shelf	46.58	3.93	50.34	5.18	-3.75	3.49E-04	7.66E-03
cg07813065	MTSS1L	16	Body	S_Shore	75.62	4.31	79.37	3.48	-3.75	3.11E-04	6.99E-03
cg20442078	UCN	2	TSS1500	Island	33.38	4.84	37.13	4.93	-3.75	1.63E-03	2.44E-02
cg20459712	DST	6	Body	N_Shelf	44.33	6.03	48.08	5.55	-3.75	9.20E-04	1.61E-02
cg01201215	ATOX1	5	Body	N_Shore	24.19	4.42	27.94	5.48	-3.75	3.28E-03	3.98E-02
cg23205629	RNF122	8	Body	N_Shelf	61.60	3.87	65.35	3.78	-3.75	5.62E-05	1.72E-03
cg13954292	ZFR	5	TSS1500	S_Shore	84.34	4.28	88.09	3.03	-3.75	1.81E-05	6.40E-04
cg04548261		19	Intergenic	N_Shelf	81.62	5.31	85.37	4.46	-3.75	3.47E-04	7.63E-03
cg26164767	SLC10A5	8	TSS1500	Open sea	66.03	4.33	69.79	4.27	-3.75	1.59E-04	4.11E-03
cg00786390	LOC100240726	20	Body	Open sea	70.02	3.13	73.77	3.02	-3.75	2.25E-07	1.09E-05
cg20383370		4	Intergenic	S_Shelf	82.37	4.67	86.12	3.37	-3.75	1.30E-05	4.76E-04
cg14848685	KIAA1244	6	Body	Island	82.13	4.58	85.88	4.40	-3.75	2.36E-04	5.64E-03
cg12394289	EHMT2;EHMT2	6	Body;Body	N_Shore	49.73	5.23	53.48	4.51	-3.75	1.56E-04	4.05E-03
cg07226470	GMPR	6	Body	Open sea	59.39	4.39	63.14	3.86	-3.75	2.99E-04	6.78E-03
cg05384123	FOXP1;FOXP1	3	5'UTR;5'UTR	Open sea	30.09	4.88	33.84	4.59	-3.75	3.84E-03	4.43E-02
cg03400139	NAV1	1	Body	S_Shore	44.28	5.57	48.03	4.24	-3.75	6.50E-04	1.24E-02
cg19434410	KIAA1688	8	Body	S_Shelf	64.27	4.36	68.02	4.57	-3.75	7.63E-05	2.23E-03
cg24010952	BDKRB2	14	TSS1500	Open sea	33.22	5.25	36.97	5.98	-3.75	2.08E-04	5.09E-03
cg03495053	THBS2	6	Body	S_Shore	53.95	4.32	57.70	5.33	-3.75	3.15E-04	7.07E-03
cg01878980	C2orf81	2	TSS200	N_Shore	76.24	4.59	79.99	4.88	-3.75	2.93E-03	3.68E-02
cg07825495	KRT85	12	1stExon	Island	65.57	5.22	69.32	4.73	-3.75	1.89E-04	4.72E-03
cg08692903		16	Intergenic	Open sea	27.51	4.01	31.26	5.35	-3.75	2.05E-03	2.88E-02
cg17783827	ADAMTS10	19	Body	Island	79.88	3.91	83.63	3.20	-3.75	1.10E-05	4.10E-04
cg01482860		12	Intergenic	Island	59.34	4.91	63.09	5.40	-3.75	4.15E-04	8.77E-03
cg17548932	RNF113B;FARP1;FARP1	13	3'UTR;5'UTR;5'UTR	N_Shore	65.73	6.05	69.47	4.13	-3.75	2.59E-04	6.07E-03
cg11468819		5	Intergenic	Open sea	25.70	4.25	29.45	5.27	-3.75	6.88E-04	1.29E-02

cg07818063	NFIC;NFIC	19	Body;Body	N_Shelf	62.99	5.27	66.74	4.01	-3.75	1.76E-03	2.58E-02
cg05227215	CXXC5	5	5'UTR	S_Shore	27.77	4.31	31.51	4.91	-3.75	3.18E-03	3.90E-02
cg21039221		2	Intergenic	Open sea	77.17	6.35	80.92	5.14	-3.75	2.67E-03	3.45E-02
cg06502149		2	Intergenic	Open sea	72.00	4.20	75.75	4.51	-3.75	5.34E-05	1.64E-03
cg24631784		8	Intergenic	Open sea	81.95	6.19	85.70	3.66	-3.75	1.05E-03	1.77E-02
cg07191657	MIR1265	10	TSS200	Open sea	53.88	4.78	57.62	4.99	-3.75	1.09E-03	1.82E-02
cg23009508	KLHL29	2	Body	Island	69.38	3.96	73.13	3.93	-3.75	6.48E-05	1.94E-03
cg03633268	RBPMS;RBPMS;RBPMS;RBPMS	8	Body;Body;Body;Body	Open sea	63.59	3.97	67.34	4.53	-3.75	2.04E-05	7.11E-04
cg02185666	NRP2;NRP2;NRP2;NRP2;NRP2;NRP2	2	Body;Body;Body;Body;Body;Body	Island	35.39	4.99	39.14	4.46	-3.75	7.10E-04	1.33E-02
cg03776080	IGFBP3;IGFBP3	7	TSS1500;TSS1500	S_Shore	20.99	3.78	24.74	5.26	-3.75	1.99E-04	4.92E-03
cg08839164		2	Intergenic	Island	72.72	4.35	76.47	3.86	-3.75	7.24E-05	2.13E-03
cg25273520		15	Intergenic	Open sea	68.54	5.07	72.29	5.01	-3.75	1.09E-03	1.82E-02
cg03316934		2	Intergenic	Open sea	77.22	4.69	80.97	3.09	-3.75	7.74E-05	2.26E-03
cg12036817	SIGIRR;SIGIRR;SIGIRR	11	3'UTR;3'UTR;3'UTR	N_Shore	16.11	3.26	19.86	3.88	-3.75	2.30E-05	7.89E-04
cg18944099	C6orf47	6	TSS1500	S_Shore	68.76	4.66	72.51	3.75	-3.75	4.54E-04	9.41E-03
cg27487796		7	Intergenic	Open sea	43.00	4.65	46.75	4.92	-3.75	4.72E-04	9.69E-03
cg03641740	PPP3CA;PPP3CA;PPP3CA	4	Body;Body;Body	Open sea	82.11	4.29	85.86	3.71	-3.75	1.15E-04	3.13E-03
cg27370248	RAB27A	15	5'UTR	N_Shelf	64.08	3.59	67.82	3.02	-3.75	8.22E-07	3.73E-05
cg12686055	ANO6;ANO6;ANO6	12	Body;Body;Body	Open sea	58.77	7.16	62.52	5.61	-3.75	9.10E-04	1.60E-02
cg03634775	PHKG2	16	TSS1500	N_Shore	30.87	4.46	34.61	4.69	-3.75	4.53E-04	9.39E-03
cg20478120	PFDN6;WDR46;WDR46	6	Body;TSS1500;TSS1500	S_Shore	32.99	4.67	36.73	5.32	-3.75	1.14E-03	1.89E-02
cg11522612	LY6G6C	6	Body	N_Shelf	51.63	4.61	55.38	5.04	-3.75	3.80E-04	8.19E-03
cg10261191	FEZ1;FEZ1	11	5'UTR;5'UTR	N_Shore	34.96	5.00	38.71	4.02	-3.75	2.30E-04	5.52E-03
cg12465412	PCNXL2	1	Body	N_Shelf	69.99	5.63	73.73	6.06	-3.75	4.42E-03	4.88E-02
cg15534212		5	Intergenic	Open sea	24.03	6.41	27.78	7.19	-3.75	1.16E-03	1.92E-02
cg06323896		16	Intergenic	S_Shelf	84.81	4.50	88.56	3.04	-3.75	1.07E-05	4.00E-04
cg02105458		15	Intergenic	Island	29.29	4.44	33.04	5.68	-3.74	1.10E-03	1.84E-02
cg01056272		6	Intergenic	Open sea	65.17	5.03	68.92	5.23	-3.74	3.52E-03	4.18E-02
cg01114033		11	Intergenic	Open sea	31.58	3.61	35.33	4.39	-3.74	9.95E-06	3.75E-04
cg11036831	DYDC2;DYDC1	10	TSS200;5'UTR	Island	30.46	5.19	34.21	5.71	-3.74	4.74E-04	9.73E-03
cg24450631	IGF2BP2;IGF2BP2	3	1stExon;1stExon	Island	31.69	3.77	35.44	4.15	-3.74	7.49E-05	2.19E-03
cg07008962		6	Intergenic	N_Shelf	81.02	5.72	84.76	4.43	-3.74	9.64E-04	1.67E-02
cg09246103	RERE;RERE;RERE	1	5'UTR;Body;Body	Open sea	59.16	5.29	62.90	4.86	-3.74	3.57E-03	4.22E-02
cg19042141	RXRB	6	Body	N_Shore	77.27	4.67	81.02	3.95	-3.74	7.05E-05	2.09E-03
cg04005843	LPCAT1	5	3'UTR	N_Shore	49.20	4.89	52.94	6.13	-3.74	2.59E-03	3.39E-02
cg11622324		13	Intergenic	N_Shore	20.66	3.13	24.40	3.71	-3.74	3.53E-07	1.68E-05
cg05406635	CCDC85C	14	1stExon	Island	18.40	3.21	22.14	3.57	-3.74	8.76E-07	3.96E-05
cg03457400	MEGF6	1	Body	N_Shore	71.84	5.15	75.58	5.62	-3.74	5.21E-04	1.04E-02
cg14168269		9	Intergenic	Open sea	70.76	4.59	74.50	3.61	-3.74	3.52E-04	7.71E-03
cg20630887	ACOT7;ACOT7;ACOT7;ACOT7	1	Body;Body;Body;Body	Open sea	38.15	4.63	41.89	3.82	-3.74	1.38E-04	3.66E-03
cg12631085	ROS1	6	TSS1500	Open sea	75.77	3.40	79.51	2.61	-3.74	2.47E-06	1.04E-04
cg15439110		3	Intergenic	Open sea	64.52	5.69	68.26	5.86	-3.74	6.33E-04	1.22E-02
cg26745143	CHRNA10	11	TSS1500	Open sea	72.68	3.94	76.42	3.61	-3.74	4.32E-05	1.37E-03
cg20993386	PITPNC1;PITPNC1	17	Body;Body	Open sea	39.36	4.15	43.10	5.92	-3.74	3.44E-03	4.12E-02
cg09181792	CFTR	7	TSS1500	Open sea	57.34	7.86	61.08	9.87	-3.74	9.04E-04	1.59E-02
cg11718780	CAMSAP1L1	1	Body	Open sea	62.34	4.05	66.08	3.73	-3.74	4.53E-05	1.43E-03
cg11246007	OR2H2	6	1stExon	Open sea	56.68	6.77	60.42	5.82	-3.74	3.93E-03	4.51E-02
cg13120424	FLOT1	6	Body	Open sea	77.38	4.57	81.12	4.61	-3.74	4.35E-04	9.10E-03
cg13826452		6	Intergenic	Open sea	30.64	4.27	34.38	5.08	-3.74	7.48E-04	1.38E-02
cg08996678	TRIM40	6	Body	Open sea	76.53	5.59	80.27	5.16	-3.74	3.56E-03	4.21E-02
cg19959677		14	Intergenic	N_Shelf	38.08	4.39	41.82	4.60	-3.74	3.70E-05	1.20E-03
cg24714100	HCG18;HCG18	6	Body;Body	N_Shelf	35.18	5.53	38.92	4.63	-3.74	2.98E-03	3.73E-02
cg05145428	SDCCAG3;SDCCAG3;SDCCAG3	9	Body;Body;Body	N_Shore	78.53	3.55	82.27	4.32	-3.74	7.48E-05	2.19E-03

cg20373401		6	Intergenic	N_Shore	79.27	4.97	83.01	3.63	-3.74	1.83E-04	4.60E-03
cg04297922	MEX3A	1	3'UTR	N_Shelf	68.22	3.91	71.95	3.94	-3.74	1.20E-04	3.24E-03
cg02991469	ZFP64	20	Body	N_Shore	18.68	5.24	22.41	6.84	-3.74	2.55E-03	3.34E-02
cg07931652	RXRB	6	Body	N_Shore	54.75	3.92	58.48	4.29	-3.74	2.99E-04	6.79E-03
cg24774396	NXN	17	Body	N_Shelf	75.66	6.02	79.40	6.03	-3.74	3.48E-04	7.64E-03
cg03423149	MACROD1	11	Body	Island	5.51	2.03	9.25	4.82	-3.74	6.00E-06	2.36E-04
cg08198401		14	Intergenic	N_Shelf	72.49	3.67	76.23	3.33	-3.74	1.35E-05	4.93E-04
cg11697978		1	Intergenic	N_Shelf	38.00	5.09	41.74	6.33	-3.74	3.42E-03	4.10E-02
cg26761572	PRSS27	16	Body	S_Shore	65.04	5.62	68.78	4.88	-3.74	9.70E-04	1.67E-02
cg25779645	R3HDML	20	TSS1500	Open sea	63.33	5.14	67.06	6.13	-3.74	3.15E-03	3.87E-02
cg14991110	PLAC9	10	TSS200	N_Shore	39.45	3.90	43.19	5.93	-3.74	2.09E-03	2.92E-02
cg23357257	SEZ6L2;SEZ6L2;SEZ6L2;SEZ6L2	16	Body;Body;Body;Body	Island	17.17	4.61	20.91	5.82	-3.74	2.92E-03	3.67E-02
cg27113015	SASH1	6	Body	Open sea	54.39	5.00	58.13	4.97	-3.74	3.74E-04	8.09E-03
cg22566906	GRASP	12	TSS1500	N_Shore	51.24	5.48	54.97	4.12	-3.74	7.91E-04	1.44E-02
cg02087565		6	Intergenic	S_Shore	52.56	4.06	56.29	3.97	-3.73	1.64E-04	4.22E-03
cg08165083	PEX14	1	Body	Open sea	72.63	4.48	76.37	2.91	-3.73	9.60E-05	2.71E-03
cg20803916	TRIM22	11	TSS1500	Open sea	83.58	4.03	87.32	3.65	-3.73	2.00E-05	6.99E-04
cg10181419	ACP1;SH3YL1;SH3YL1;ACP1;ACP1;ACP	2	TSS1500;TSS200;TSS200;TSS1500;TSS1500	Island	29.72	8.53	33.45	8.50	-3.73	3.45E-03	4.13E-02
cg00846166	OLFM2	19	Body	N_Shore	71.07	4.32	74.80	3.72	-3.73	2.51E-05	8.55E-04
cg16196968		8	Intergenic	Open sea	75.51	4.48	79.24	3.65	-3.73	4.42E-05	1.39E-03
cg02893348	RAD51L1;RAD51L1;RAD51L1	14	Body;Body;Body	Open sea	69.08	4.66	72.82	5.06	-3.73	2.21E-03	3.03E-02
cg11492403	RUNDC1;AARSD1;AARSD1;AARSD1;AA	17	TSS1500;Body;Body;Body;Body	N_Shore	65.89	3.65	69.63	4.09	-3.73	8.19E-05	2.37E-03
cg26539455		6	Intergenic	S_Shore	25.47	4.78	29.20	5.49	-3.73	2.42E-03	3.23E-02
cg01858157		20	Intergenic	N_Shelf	61.54	5.41	65.28	4.81	-3.73	1.94E-03	2.77E-02
cg14671982	SLC12A7	5	Body	S_Shore	51.91	4.21	55.64	5.18	-3.73	2.03E-03	2.86E-02
cg00362401		20	Intergenic	S_Shelf	63.79	5.65	67.52	6.28	-3.73	5.97E-04	1.16E-02
cg14881601		19	Intergenic	N_Shore	35.16	3.77	38.90	3.89	-3.73	2.33E-04	5.58E-03
cg05981034		17	Intergenic	Open sea	60.22	4.55	63.96	5.54	-3.73	3.25E-04	7.24E-03
cg01134139	KIF25;KIF25	6	Body;Body	N_Shore	78.93	5.86	82.67	5.45	-3.73	2.76E-03	3.54E-02
cg18004856	FYCO1	3	5'UTR	Open sea	39.13	4.57	42.86	5.54	-3.73	1.67E-03	2.49E-02
cg20777257		5	Intergenic	Open sea	43.88	4.39	47.61	4.54	-3.73	1.63E-04	4.20E-03
cg05508084	ZNF667;ZNF667	19	5'UTR;Body	Island	20.61	4.28	24.34	4.38	-3.73	5.17E-06	2.06E-04
cg17102205		2	Intergenic	Open sea	43.24	4.11	46.97	3.70	-3.73	2.31E-04	5.54E-03
cg15191795		4	Intergenic	Open sea	22.02	3.70	25.75	4.58	-3.73	1.39E-04	3.67E-03
cg20019356	ESR2	14	5'UTR	S_Shelf	64.67	5.67	68.40	5.92	-3.73	3.88E-03	4.47E-02
cg00277685		16	Intergenic	Open sea	79.84	5.53	83.57	4.73	-3.73	1.05E-03	1.77E-02
cg08964756	EFR3A	8	Body	Open sea	79.48	5.00	83.21	4.14	-3.73	8.38E-05	2.41E-03
cg01919941		12	Intergenic	Open sea	26.50	4.89	30.23	5.65	-3.73	3.77E-03	4.38E-02
cg06642012	LOC100129637	16	Body	Island	58.59	5.21	62.32	7.08	-3.73	1.22E-03	1.99E-02
cg01586332	SPG7	16	Body	Open sea	71.10	3.55	74.83	3.05	-3.73	5.16E-06	2.06E-04
cg02993069	C1orf113;C1orf113	1	3'UTR;3'UTR	Island	27.91	3.77	31.64	5.88	-3.73	1.04E-03	1.76E-02
cg15020894	SLC22A3	6	Body	N_Shelf	37.51	4.17	41.24	5.87	-3.73	1.57E-03	2.38E-02
cg10406414		8	Intergenic	Open sea	40.67	5.21	44.40	5.26	-3.73	4.02E-03	4.58E-02
cg05377754	LOXL2	8	5'UTR	N_Shore	50.84	3.61	54.57	3.12	-3.73	1.40E-05	5.09E-04
cg01763051	ULBP1	6	Body	Island	30.09	4.37	33.82	4.61	-3.73	6.93E-06	2.69E-04
cg05213661	SLC6A18	5	Body	S_Shore	47.07	5.83	50.80	6.62	-3.73	2.83E-03	3.60E-02
cg00581334		3	Intergenic	N_Shore	62.01	4.59	65.74	5.11	-3.73	9.39E-04	1.63E-02
cg19021197	TBX2	17	TSS1500	Island	22.19	3.14	25.92	3.57	-3.73	9.60E-07	4.32E-05
cg15829535	HLA-DPB1	6	Body	Island	24.32	4.17	28.05	5.21	-3.73	2.36E-03	3.17E-02
cg01176103	SSH1;SSH1;SSH1	12	Body;Body;Body	Open sea	63.81	4.61	67.54	4.18	-3.73	6.58E-04	1.25E-02
cg23029851	ATHL1	11	Body	S_Shelf	80.92	4.94	84.65	4.17	-3.73	1.70E-03	2.52E-02
cg17980508	IFI44L	1	TSS1500	Open sea	18.44	4.83	22.17	6.07	-3.73	4.55E-03	4.98E-02
cg26709285	KCNB1;KCNB1	20	1stExon;5'UTR	Island	27.63	4.21	31.36	4.94	-3.73	1.16E-04	3.17E-03

cg15601807	C10orf25;ZNF22	10	3'UTR;TSS1500	N_Shore	51.90	5.04	55.63	4.69	-3.73	1.61E-03	2.42E-02
cg21415060	FCER1A	1	Body	Open sea	51.47	4.18	55.20	4.68	-3.73	4.85E-04	9.89E-03
cg06013632		15	Intergenic	Open sea	33.99	5.24	37.72	4.09	-3.73	9.37E-04	1.63E-02
cg17751414		1	Intergenic	Open sea	85.08	4.46	88.81	3.51	-3.73	3.17E-06	1.31E-04
cg22786748		6	Intergenic	Open sea	39.87	5.84	43.60	5.46	-3.73	3.97E-03	4.55E-02
cg01432620	ZIC2	13	Body	N_Shore	17.25	3.67	20.98	3.53	-3.73	3.79E-07	1.80E-05
cg14825688	LEAP2	5	TSS1500	Open sea	44.08	4.57	47.80	4.94	-3.73	4.10E-04	8.69E-03
cg01120391	ANO1	11	TSS1500	N_Shore	7.94	2.66	11.66	4.13	-3.73	2.67E-07	1.29E-05
cg22453113	UBTF;UBTF;UBTF	17	Body;Body;Body	Island	8.22	3.15	11.95	5.21	-3.73	8.61E-05	2.47E-03
cg22691587	TRIT1	1	TSS1500	S_Shore	58.90	5.13	62.62	4.85	-3.73	1.20E-03	1.97E-02
cg05211480		14	Intergenic	N_Shelf	64.18	5.22	67.90	5.59	-3.73	3.77E-04	8.14E-03
cg20632143	SLC11A2	12	5'UTR	N_Shelf	36.57	6.56	40.30	6.00	-3.73	7.75E-04	1.41E-02
cg02836046	SIM2	21	3'UTR	S_Shore	82.96	5.24	86.68	2.84	-3.73	1.46E-03	2.26E-02
cg16960758	NOSTRIN;NOSTRIN;NOSTRIN;NOSTRIN	2	5'UTR;TSS200;TSS200;TSS200	Open sea	35.60	5.58	39.32	4.55	-3.73	6.94E-04	1.30E-02
cg21505886	TMEM129;TACC3;TMEM129	4	TSS1500;5'UTR;TSS1500	S_Shore	61.18	3.90	64.91	4.44	-3.73	7.20E-05	2.12E-03
cg09152949	TBCD	17	Body	Open sea	53.87	6.14	57.60	5.34	-3.73	2.27E-03	3.09E-02
cg06297194		7	Intergenic	Open sea	84.16	3.72	87.89	2.91	-3.73	5.99E-06	2.36E-04
cg08610862	LHX9;LHX9	1	Body;Body	S_Shore	25.14	4.63	28.87	5.06	-3.73	2.51E-04	5.92E-03
cg01204210		10	Intergenic	Island	20.64	4.59	24.37	5.40	-3.73	6.66E-04	1.26E-02
cg27171201	NFIC;NFIC	19	TSS1500;Body	N_Shore	72.10	3.81	75.82	3.78	-3.73	1.28E-04	3.44E-03
cg20824200		2	Intergenic	Open sea	39.71	5.49	43.44	4.84	-3.73	2.24E-03	3.06E-02
cg11172682	ANXA6;ANXA6	5	Body;Body	Open sea	44.09	4.53	47.82	5.32	-3.72	1.79E-03	2.62E-02
cg01775492		1	Intergenic	N_Shelf	76.93	4.93	80.66	3.78	-3.72	2.12E-04	5.18E-03
cg19712979	HSD17B8	6	Body	S_Shore	46.35	3.71	50.08	4.27	-3.72	1.29E-04	3.44E-03
cg14317046	MAST3	19	Body	N_Shore	75.60	3.67	79.32	4.33	-3.72	1.75E-04	4.44E-03
cg24516399	SCARNA6;ATG16L1;ATG16L1	2	TSS1500;Body;Body	Open sea	74.82	4.85	78.54	3.78	-3.72	6.35E-05	1.91E-03
cg03035359	LRP5	11	Body	S_Shelf	84.07	4.20	87.80	2.49	-3.72	7.35E-06	2.85E-04
cg13646480		9	Intergenic	S_Shore	64.42	4.86	68.14	4.68	-3.72	1.37E-03	2.16E-02
cg16253870		12	Intergenic	Open sea	65.29	5.81	69.01	6.47	-3.72	1.67E-03	2.48E-02
cg20187049		3	Intergenic	Open sea	27.09	4.53	30.81	6.02	-3.72	3.50E-03	4.16E-02
cg13511576	CSNK2B	6	Body	S_Shore	64.05	4.08	67.78	3.96	-3.72	1.43E-04	3.76E-03
cg13349035	HLA-DPB1	6	Body	N_Shore	30.42	4.72	34.14	4.15	-3.72	2.57E-03	3.37E-02
cg25483749	BZW2;BZW2;BZW2	7	Body;5'UTR;5'UTR	Open sea	81.02	5.91	84.74	5.67	-3.72	7.83E-04	1.43E-02
cg01347048	SUFU	10	Body	Open sea	21.49	4.11	25.22	4.60	-3.72	7.32E-04	1.36E-02
cg21204492	XKR4	8	Body	Open sea	27.10	4.64	30.83	6.18	-3.72	1.92E-03	2.75E-02
cg18440123		7	Intergenic	N_Shelf	75.45	4.90	79.17	4.49	-3.72	6.74E-05	2.01E-03
cg25252197	RBM20	10	Body	Open sea	31.67	3.68	35.39	5.16	-3.72	1.20E-03	1.96E-02
cg19885037	ITPR1;ITPR1;ITPR1	3	Body;Body;Body	Open sea	23.56	3.89	27.29	5.35	-3.72	1.38E-03	2.17E-02
cg03260566	SLIT2	4	Body	Island	14.36	3.30	18.09	4.75	-3.72	5.34E-06	2.12E-04
cg03678035		7	Intergenic	Open sea	55.28	5.23	59.01	5.35	-3.72	1.80E-03	2.62E-02
cg18250453	TERF1;TERF1	8	Body;Body	S_Shelf	84.26	4.94	87.98	3.15	-3.72	3.73E-04	8.07E-03
cg15963714	H19	11	Body	Island	60.80	2.43	64.52	3.89	-3.72	1.77E-06	7.64E-05
cg23901212		2	Intergenic	Open sea	73.66	4.65	77.38	4.81	-3.72	1.05E-04	2.92E-03
cg20538707		17	Intergenic	Open sea	68.17	4.76	71.89	3.47	-3.72	1.17E-04	3.17E-03
cg19854744	FOXR1	11	Body	Open sea	48.58	4.52	52.30	5.48	-3.72	1.09E-04	3.02E-03
cg05163056	CSF3R;CSF3R;CSF3R;CSF3R	1	Body;Body;Body;Body	S_Shelf	85.28	5.06	89.00	3.41	-3.72	1.39E-04	3.67E-03
cg27058882	ERAL1	17	3'UTR	Open sea	70.81	3.61	74.53	3.69	-3.72	2.44E-05	8.34E-04
cg25191573	P4HTM;P4HTM	3	Body;Body	Open sea	73.13	4.05	76.85	2.87	-3.72	9.75E-06	3.68E-04
cg11823382		12	Intergenic	S_Shelf	70.07	6.47	73.79	4.36	-3.72	1.44E-03	2.24E-02
cg17502434	FOSB;FOSB	19	Body;Body	Island	14.53	3.83	18.26	5.76	-3.72	4.74E-04	9.72E-03
cg06296067	PPA1	10	3'UTR	Open sea	73.89	4.87	77.61	5.25	-3.72	8.99E-05	2.56E-03
cg00232827	NEURL1B	5	Body	Island	64.87	6.33	68.59	4.49	-3.72	1.41E-03	2.21E-02
cg11342234	KCNQ1;KCNQ1OT1;KCNQ1	11	Body;Body;Body	Open sea	58.91	4.40	62.63	4.50	-3.72	1.85E-04	4.65E-03

cg12798853	ERCC6	10	Body	Open sea	88.32	7.57	92.04	4.35	-3.72	7.94E-04	1.44E-02
cg01069468	SYNGAP1	6	Body	S_Shelf	67.67	3.78	71.39	3.65	-3.72	3.65E-05	1.18E-03
cg00040411		6	Intergenic	Island	62.36	5.87	66.08	5.25	-3.72	4.28E-03	4.77E-02
cg14841011	PRDM16;PRDM16	1	Body;Body	Island	30.03	3.73	33.75	5.58	-3.72	3.48E-03	4.14E-02
cg15015070	GIPC2	1	Body	Open sea	79.62	5.73	83.34	3.98	-3.72	3.98E-04	8.48E-03
cg26489413	AMPD3;AMPD3;AMPD3;AMPD3	11	1stExon;TSS1500;5'UTR;Body	Open sea	71.29	4.51	75.01	4.78	-3.72	2.92E-04	6.66E-03
cg14862445	BAT4;CSNK2B	6	TSS200;TSS1500	Island	4.80	3.20	8.52	8.19	-3.72	2.03E-03	2.85E-02
cg02098905		8	Intergenic	Open sea	78.20	4.70	81.92	5.38	-3.72	2.95E-04	6.71E-03
cg26757562	MIR519A1	19	TSS200	Open sea	72.37	5.69	76.09	6.02	-3.72	1.79E-03	2.61E-02
cg18841298	SLC5A8	12	TSS1500	S_Shore	63.52	6.01	67.24	4.92	-3.72	1.96E-03	2.79E-02
cg16231241		6	Intergenic	N_Shore	50.79	6.39	54.51	6.52	-3.72	6.17E-04	1.19E-02
cg02764478	SIM1	6	Body	N_Shore	24.49	5.83	28.21	4.96	-3.72	2.39E-03	3.21E-02
cg22601123	L3MBTL;L3MBTL	20	TSS200;TSS200	N_Shore	39.53	5.66	43.25	4.51	-3.72	1.88E-04	4.71E-03
cg22670503	FRMPD2;FRMPD2;FRMPD2;FRMPD2	10	5'UTR;1stExon;1stExon;5'UTR	Open sea	70.40	4.55	74.11	5.41	-3.72	2.34E-03	3.15E-02
cg12947833	HSPB2;CRYAB;HSPB2	11	5'UTR;TSS1500;1stExon	Open sea	28.13	3.40	31.85	5.14	-3.72	4.76E-04	9.75E-03
cg19865727		14	Intergenic	Open sea	70.87	4.89	74.59	5.78	-3.72	9.69E-04	1.67E-02
cg18850264	MBNL1;MBNL1;MBNL1;MBNL1;MBNL	3	Body;Body;Body;Body;Body;Body;Body	Open sea	79.28	4.72	82.99	4.76	-3.72	1.65E-04	4.22E-03
cg19907964	TCFL5	20	3'UTR	S_Shore	73.44	5.67	77.16	5.12	-3.72	2.59E-03	3.39E-02
cg19611886		8	Intergenic	N_Shore	19.92	3.73	23.64	5.10	-3.72	5.62E-05	1.72E-03
cg26429925	BNC1	15	TSS1500	Island	16.96	3.65	20.68	3.80	-3.72	8.96E-06	3.41E-04
cg23544107	SSBP4;SSBP4	19	Body;Body	S_Shelf	74.52	4.38	78.23	3.55	-3.72	5.10E-05	1.58E-03
cg23259813	ELL	19	Body	Island	69.97	4.37	73.69	4.60	-3.72	7.04E-05	2.08E-03
cg14624207	LRP5	11	Body	Open sea	18.31	3.98	22.03	4.21	-3.72	2.66E-04	6.20E-03
cg07928502	MBOAT7;MBOAT7;MBOAT7;MBOAT7	19	Body;Body;Body;Body	N_Shore	61.71	4.16	65.42	4.00	-3.72	1.11E-04	3.05E-03
cg27036638	RARG;RARG	12	Body;Body	Open sea	53.51	4.24	57.23	4.05	-3.72	8.19E-05	2.37E-03
cg13527233	GNA11	19	Body	Island	69.66	5.38	73.37	4.53	-3.72	2.10E-03	2.93E-02
cg08348100	SETD1B	12	Body	S_Shore	61.41	4.63	65.12	3.81	-3.72	3.37E-04	7.46E-03
cg16347123		7	Intergenic	Open sea	52.36	3.66	56.08	5.00	-3.72	6.15E-06	2.41E-04
cg22969914		15	Intergenic	N_Shore	52.28	4.72	55.99	4.86	-3.72	3.71E-05	1.20E-03
cg27440751	ANKRD29	18	Body	N_Shelf	56.31	5.21	60.03	4.70	-3.72	3.29E-03	4.00E-02
cg15578535		2	Intergenic	Open sea	69.38	4.98	73.09	4.26	-3.72	3.83E-04	8.24E-03
cg04284535	ETV4;ETV4	17	TSS1500;TSS1500	Island	26.84	3.54	30.55	3.91	-3.71	2.44E-06	1.03E-04
cg01275006	GGN	19	Body	Island	89.11	5.42	92.83	3.23	-3.71	9.15E-05	2.60E-03
cg21854560	ABLIM2;ABLIM2;ABLIM2;ABLIM2;ABL	4	Body;Body;Body;Body;Body;Body;Body	Open sea	68.50	4.69	72.22	4.83	-3.71	4.99E-04	1.01E-02
cg00070870	TUBB	6	Body	S_Shelf	58.44	4.20	62.15	4.77	-3.71	1.57E-05	5.63E-04
cg01942318		2	Intergenic	Open sea	40.25	4.89	43.96	4.10	-3.71	6.26E-04	1.21E-02
cg27124512	TRIM72;TRIM72	16	5'UTR;1stExon	N_Shore	76.73	4.87	80.44	5.15	-3.71	2.59E-03	3.39E-02
cg03184441	BAI1	8	Body	Island	74.43	4.67	78.15	4.60	-3.71	6.10E-04	1.18E-02
cg17867776	AMZ1	7	5'UTR	Island	15.82	7.19	19.53	9.40	-3.71	3.05E-03	3.78E-02
cg22457287	TOX2;TOX2;TOX2;TOX2	20	Body;Body;Body;Body	Open sea	72.63	5.07	76.34	4.16	-3.71	2.59E-04	6.06E-03
cg21213332	AGPAT3;AGPAT3	21	5'UTR;5'UTR	Open sea	58.99	5.72	62.70	4.98	-3.71	2.73E-03	3.51E-02
cg17669581		4	Intergenic	S_Shore	21.76	3.70	25.48	4.29	-3.71	3.27E-05	1.08E-03
cg21157904	TRIM14;NANS	9	3'UTR;Body	Open sea	17.30	3.42	21.01	4.64	-3.71	3.50E-04	7.67E-03
cg21635858		15	Intergenic	Open sea	74.02	5.94	77.73	4.91	-3.71	3.01E-03	3.75E-02
cg08493463	ENPP6;ENPP6	4	1stExon;5'UTR	Open sea	45.89	3.55	49.60	4.76	-3.71	4.28E-04	8.99E-03
cg14741811		3	Intergenic	Open sea	79.40	4.91	83.11	3.99	-3.71	1.58E-04	4.08E-03
cg15460899	CNTN2	1	Body	Island	83.88	5.40	87.59	4.72	-3.71	4.87E-04	9.92E-03
cg23540704	C1QL1	17	TSS1500	Island	28.36	3.89	32.07	4.32	-3.71	6.40E-05	1.92E-03
cg03793270	NOX4;NOX4;NOX4;NOX4	11	5'UTR;TSS200;TSS200;TSS200	Island	28.97	4.13	32.69	4.51	-3.71	1.38E-04	3.66E-03
cg26400885	ZIC1	3	TSS1500	N_Shore	38.73	6.53	42.44	6.80	-3.71	2.29E-03	3.11E-02
cg23779626		6	Intergenic	Open sea	49.55	4.10	53.26	3.55	-3.71	3.16E-06	1.31E-04
cg12269002	PAK1;PAK1	11	5'UTR;5'UTR	Island	25.91	3.86	29.62	4.94	-3.71	2.11E-05	7.34E-04
cg09231673	DEPDC7;DEPDC7	11	Body;Body	Open sea	82.29	4.00	86.01	3.27	-3.71	2.00E-05	7.00E-04

cg15107132	FERMT3;FERMT3	11	3'UTR;3'UTR	N_Shelf	55.96	5.00	59.67	4.83	-3.71	1.59E-03	2.40E-02
cg20986996	TCF21;TCF21	6	TSS200;TSS200	N_Shore	16.46	3.35	20.17	3.57	-3.71	4.84E-06	1.94E-04
cg08744727	ZNF876P	4	TSS1500	N_Shore	52.65	4.37	56.36	4.66	-3.71	3.60E-04	7.86E-03
cg14848450		10	Intergenic	N_Shore	70.20	5.37	73.91	4.22	-3.71	1.10E-03	1.84E-02
cg20040691	ASB4;ASB4	7	TSS200;TSS200	Open sea	81.22	5.16	84.93	4.09	-3.71	2.25E-03	3.07E-02
cg03173502	JARID2	6	Body	Open sea	28.17	4.74	31.88	5.42	-3.71	4.14E-03	4.68E-02
cg04365123	GDF6	8	3'UTR	N_Shore	36.71	3.93	40.42	6.12	-3.71	1.41E-03	2.20E-02
cg07576525	SHANK3	22	Body	S_Shelf	73.32	5.16	77.03	4.31	-3.71	2.24E-04	5.41E-03
cg16228380		8	Intergenic	Open sea	79.96	4.01	83.67	3.05	-3.71	1.37E-06	6.03E-05
cg12756893		4	Intergenic	Open sea	81.78	6.10	85.49	4.60	-3.71	6.33E-04	1.21E-02
cg24435924	MAST1	19	Body	N_Shore	14.72	3.72	18.43	4.51	-3.71	4.63E-07	2.17E-05
cg23756322	RIMS1;RIMS1;RIMS1;RIMS1	6	TSS1500;Body;Body;Body	Open sea	84.67	5.51	88.38	3.39	-3.71	1.90E-04	4.74E-03
cg09662411	GFI1;GFI1;GFI1	1	Body;Body;Body	Island	48.91	4.60	52.62	5.20	-3.71	2.47E-04	5.85E-03
cg01816014	PRDM2	1	5'UTR	S_Shore	33.64	4.56	37.35	4.56	-3.71	2.12E-04	5.18E-03
cg19137676	INS-IGF2;IGF2;INS-IGF2	11	Body;TSS1500;Body	Open sea	58.27	3.65	61.97	3.75	-3.71	1.66E-06	7.19E-05
cg10025830		12	Intergenic	N_Shelf	18.56	3.12	22.27	4.45	-3.71	4.23E-05	1.34E-03
cg05000772	SAMD14	17	Body	N_Shore	64.33	4.80	68.04	4.89	-3.71	7.86E-04	1.43E-02
cg04192740	STK10	5	Body	Open sea	62.37	5.22	66.08	5.83	-3.71	3.29E-05	1.08E-03
cg02107842	MAST4;MAST4;MAST4	5	Body;Body;Body	Open sea	77.91	4.36	81.61	3.40	-3.71	1.91E-05	6.72E-04
cg05546863	CMTM2	16	TSS200	Island	24.85	3.41	28.56	3.44	-3.71	1.12E-06	5.01E-05
cg18913872		14	Intergenic	Open sea	78.99	4.66	82.70	3.40	-3.71	8.97E-05	2.56E-03
cg03410231	CLCN1	7	Body	Island	17.90	3.46	21.60	5.32	-3.71	4.95E-05	1.54E-03
cg21006425	PDAP1	7	Body	N_Shelf	71.63	4.44	75.34	3.71	-3.71	3.02E-05	1.00E-03
cg14649874		3	Intergenic	Open sea	62.88	5.43	66.59	4.06	-3.71	1.74E-04	4.43E-03
cg18007641		4	Intergenic	Open sea	46.90	6.37	50.61	7.56	-3.71	1.58E-03	2.39E-02
cg01892620		7	Intergenic	Open sea	79.45	4.84	83.15	4.32	-3.71	5.22E-05	1.61E-03
cg03146065	POLR2A	17	3'UTR	Open sea	29.37	3.63	33.07	4.06	-3.71	1.86E-04	4.67E-03
cg03845941		17	Intergenic	Open sea	56.83	4.72	60.53	4.51	-3.71	6.58E-05	1.97E-03
cg00724839	NPTXR	22	TSS1500	S_Shore	51.60	4.91	55.31	6.43	-3.71	1.91E-03	2.74E-02
cg09299086	RCC2;RCC2	1	Body;Body	Island	20.14	4.51	23.84	5.31	-3.71	1.57E-03	2.38E-02
cg06690689	CNGB3	8	Body	Open sea	77.64	4.97	81.35	3.80	-3.71	6.37E-04	1.22E-02
cg04543273		5	Intergenic	N_Shore	50.82	3.13	54.52	3.72	-3.70	1.05E-05	3.93E-04
cg20498637		6	Intergenic	S_Shore	49.70	4.65	53.41	5.22	-3.70	7.84E-04	1.43E-02
cg02892367	PDIA4	7	Body	N_Shore	45.98	4.36	49.69	5.12	-3.70	2.25E-03	3.07E-02
cg12149609	JPH2;JPH2	20	TSS1500;TSS1500	Open sea	35.16	4.61	38.87	4.62	-3.70	1.52E-05	5.48E-04
cg09607276	DRD4	11	1stExon	Island	29.92	5.36	33.63	6.54	-3.70	3.35E-03	4.04E-02
cg07679322	INPP5F;INPP5F	10	Body;Body	Open sea	72.66	6.58	76.36	5.90	-3.70	1.29E-03	2.07E-02
cg21127068	ZIC4;ZIC4	3	TSS1500;TSS200	N_Shelf	17.23	5.68	20.94	6.35	-3.70	9.94E-04	1.70E-02
cg24502192	TSPAN31	12	TSS1500	Open sea	19.98	4.41	23.68	4.75	-3.70	1.70E-03	2.51E-02
cg13490352		7	Intergenic	N_Shore	53.84	5.52	57.54	3.81	-3.70	2.64E-04	6.16E-03
cg26891150	ANKRD34C	15	5'UTR	N_Shore	25.14	4.50	28.85	5.01	-3.70	4.14E-04	8.75E-03
cg24842760	EYA4;EYA4;EYA4	6	TSS1500;TSS1500;TSS1500	N_Shore	29.99	4.26	33.69	5.32	-3.70	1.73E-04	4.40E-03
cg04956244	RARA;RARA;RARA;RARA	17	Body;Body;Body;Body	Open sea	47.55	3.54	51.25	3.24	-3.70	8.69E-05	2.49E-03
cg00040588	MXRA8	1	Body	Island	28.13	5.26	31.83	5.74	-3.70	1.12E-03	1.87E-02
cg06009042	ANXA6;ANXA6	5	3'UTR;3'UTR	Open sea	28.74	3.83	32.44	5.56	-3.70	2.26E-03	3.08E-02
cg05180182		10	Intergenic	Open sea	72.26	4.80	75.96	5.41	-3.70	2.67E-04	6.21E-03
cg26718213	SNED1	2	Body	Island	58.55	6.17	62.25	5.79	-3.70	2.27E-03	3.09E-02
cg02577078	CABP1;CABP1;CABP1	12	Body;Body;Body	Open sea	69.32	4.20	73.03	3.83	-3.70	5.01E-05	1.56E-03
cg09541256	RUNX2;RUNX2;RUNX2	6	TSS1500;Body;Body	N_Shore	22.87	4.60	26.57	4.28	-3.70	2.03E-04	5.01E-03
cg08660883		12	Intergenic	Open sea	68.92	5.29	72.62	4.77	-3.70	9.32E-04	1.63E-02
cg22999343	NEU1	6	Body	N_Shelf	47.46	3.59	51.17	4.11	-3.70	1.22E-04	3.31E-03
cg11824509	C10orf57;LOC219347;LOC219347;LOC	10	TSS1500;Body;Body;Body;Body;Body	N_Shore	67.53	4.64	71.24	6.25	-3.70	3.11E-03	3.84E-02
cg00866054	COL11A2;COL11A2;COL11A2	6	Body;Body;Body	Open sea	63.58	4.20	67.28	5.27	-3.70	2.41E-04	5.74E-03

cg01817286	CBS	21	Body	N_Shore	54.25	3.78	57.95	3.20	-3.70	1.03E-04	2.86E-03
cg16786703	ADAM8;ADAM8;ADAM8	10	Body;Body;Body	Island	33.10	5.52	36.80	6.47	-3.70	1.29E-03	2.06E-02
cg19101417	TRIM54;TRIM54	2	TSS1500;TSS1500	Open sea	64.47	3.66	68.17	3.83	-3.70	1.29E-05	4.73E-04
cg03361789	EVPL	17	Body	S_Shore	74.32	4.61	78.02	3.44	-3.70	1.15E-04	3.15E-03
cg13109410	GPR44	11	TSS1500	S_Shelf	75.20	4.28	78.90	4.09	-3.70	6.95E-05	2.06E-03
cg19828220	LOC404266;LOC404266;HOXB6	17	Body;Body;TSS200	N_Shelf	22.82	4.21	26.52	4.86	-3.70	1.33E-03	2.11E-02
cg20941855	TAGLN3;TAGLN3;TAGLN3	3	Body;Body;Body	Open sea	48.03	5.00	51.73	5.44	-3.70	3.06E-03	3.80E-02
cg25131922	GPR177;GPR177	1	TSS1500;TSS1500	S_Shore	11.72	3.38	15.42	4.11	-3.70	1.15E-07	5.73E-06
cg11606215	PLAC8;PLAC8	4	TSS200;TSS200	Open sea	59.37	5.06	63.07	6.69	-3.70	8.44E-04	1.51E-02
cg24528866	MSTN	2	1stExon	Open sea	81.29	5.02	84.99	3.54	-3.70	4.70E-05	1.47E-03
cg02782426	ENTPD3	3	5'UTR	Island	30.80	5.08	34.50	5.43	-3.70	7.76E-04	1.42E-02
cg22562494	ETV4;ETV4	17	Body;Body	Open sea	30.61	6.70	34.31	6.74	-3.70	1.67E-03	2.48E-02
cg27372482	CAPN3;CAPN3;CAPN3;CAPN3;CAPN3;	15	Body;Body;Body;Body;TSS1500;Body;TSS1	Open sea	87.07	4.61	90.77	3.19	-3.70	1.05E-04	2.91E-03
cg18146737	GFI1;GFI1;GFI1	1	Body;Body;Body	Island	29.40	3.67	33.10	3.39	-3.70	7.16E-05	2.11E-03
cg05162750	TBX2	17	TSS1500	Island	9.65	3.06	13.35	3.32	-3.70	5.90E-08	3.00E-06
cg09463204	FMN1	15	Body	Open sea	66.54	5.49	70.24	5.16	-3.70	1.41E-03	2.21E-02
cg02251243		2	Intergenic	N_Shore	25.32	4.16	29.01	5.55	-3.70	2.47E-03	3.28E-02
cg23723793	BATF	14	TSS1500	Open sea	77.30	5.10	81.00	5.37	-3.70	1.72E-03	2.54E-02
cg22678754		1	Intergenic	S_Shore	62.06	5.43	65.76	6.73	-3.70	4.55E-03	4.98E-02
cg06915570		2	Intergenic	Open sea	46.21	4.53	49.90	4.89	-3.70	1.45E-03	2.25E-02
cg03422757		1	Intergenic	S_Shelf	77.55	4.88	81.25	3.91	-3.70	3.36E-04	7.44E-03
cg20746552	ARID5B	10	Body	Open sea	16.91	3.14	20.60	3.48	-3.70	2.44E-06	1.03E-04
cg21562750		12	Intergenic	Open sea	78.70	4.69	82.40	5.23	-3.70	5.61E-04	1.11E-02
cg05907398		15	Intergenic	Open sea	73.37	5.25	77.07	5.46	-3.70	1.36E-03	2.15E-02
cg12584785	CHRNA7	15	Body	S_Shelf	23.68	3.63	27.38	5.42	-3.70	3.41E-04	7.51E-03
cg07146104		1	Intergenic	Open sea	40.47	4.74	44.16	6.05	-3.70	2.15E-03	2.97E-02
cg18698884		11	Intergenic	Island	32.63	4.13	36.32	5.33	-3.69	1.39E-03	2.18E-02
cg03165195	PDE4C;PDE4C;PDE4C	19	Body;Body;Body	Island	54.81	5.16	58.50	5.71	-3.69	3.45E-04	7.58E-03
cg08904369	UNC93A;UNC93A	6	TSS1500;TSS1500	Open sea	71.26	3.99	74.96	3.37	-3.69	6.19E-06	2.43E-04
cg04486013	AKR1A1;AKR1A1	1	Body;Body	Open sea	64.68	3.61	68.38	3.16	-3.69	2.21E-05	7.65E-04
cg26964875	C3orf63	3	Body	N_Shore	58.24	6.30	61.93	6.18	-3.69	3.29E-04	7.32E-03
cg02486161	NOD2	16	3'UTR	Open sea	65.25	4.51	68.94	5.42	-3.69	1.48E-03	2.28E-02
cg05979020	HOXD10	2	TSS200	Island	28.39	4.27	32.08	5.39	-3.69	1.10E-03	1.84E-02
cg15084405		3	Intergenic	Open sea	74.03	5.74	77.73	5.42	-3.69	1.66E-03	2.48E-02
cg22365167	AHNAK;AHNAK	11	5'UTR;5'UTR	Open sea	24.04	6.58	27.73	7.22	-3.69	9.23E-04	1.61E-02
cg25381563	ZNF831	20	TSS1500	N_Shore	81.25	4.41	84.94	2.47	-3.69	3.00E-05	9.98E-04
cg05102670	CD81	11	TSS1500	N_Shore	43.95	5.79	47.64	5.35	-3.69	4.40E-03	4.87E-02
cg09624910	NIPA2;NIPA2;NIPA2;NIPA2	15	TSS1500;TSS1500;TSS1500;TSS1500	S_Shore	85.77	5.98	89.46	5.18	-3.69	1.04E-03	1.76E-02
cg08337420	ZSCAN22	19	TSS1500	N_Shore	65.80	5.18	69.49	5.12	-3.69	1.82E-04	4.58E-03
cg22292345	LOC100129354	3	TSS1500	Island	49.71	4.33	53.40	5.86	-3.69	1.49E-03	2.29E-02
cg02352240		16	Intergenic	Island	39.92	4.07	43.61	4.14	-3.69	9.48E-04	1.65E-02
cg10933003	HAND2	4	3'UTR	Island	26.90	3.89	30.59	5.02	-3.69	1.30E-03	2.08E-02
cg14849422		15	Intergenic	Open sea	75.46	4.30	79.15	4.43	-3.69	1.73E-04	4.40E-03
cg27551706	CAMTA1	1	Body	Open sea	50.31	3.82	54.00	3.65	-3.69	6.39E-05	1.92E-03
cg04104329	RALA	7	5'UTR	Open sea	63.38	4.20	67.07	4.32	-3.69	6.55E-04	1.25E-02
cg25864024	FAM174B	15	TSS200	Island	16.85	4.79	20.54	4.64	-3.69	1.41E-05	5.12E-04
cg26509250		2	Intergenic	Open sea	57.30	5.49	61.00	4.81	-3.69	1.14E-03	1.89E-02
cg18973101	TMEM79;SMG5	1	TSS1500;Body	Open sea	15.04	5.59	18.73	6.22	-3.69	2.08E-03	2.90E-02
cg16294045	NPR1	1	Body	S_Shelf	66.37	5.49	70.06	5.14	-3.69	2.62E-03	3.41E-02
cg02756176		4	Intergenic	Open sea	39.95	5.36	43.64	5.60	-3.69	1.26E-03	2.04E-02
cg18813159		11	Intergenic	N_Shelf	18.19	3.94	21.88	5.40	-3.69	1.57E-03	2.38E-02
cg22995255		2	Intergenic	Open sea	80.18	4.92	83.87	4.12	-3.69	7.96E-04	1.44E-02
cg14286140		9	Intergenic	Open sea	67.94	6.28	71.63	5.33	-3.69	7.30E-05	2.15E-03

cg15294150		18	Intergenic	N_Shelf	55.75	4.62	59.44	4.88	-3.69	4.04E-04	8.58E-03
cg09160020		5	Intergenic	Island	39.47	4.57	43.16	4.73	-3.69	6.16E-04	1.19E-02
cg15188010	ZIC2	13	Body	N_Shore	27.17	3.06	30.86	4.54	-3.69	9.78E-06	3.69E-04
cg26401541		6	Intergenic	Open sea	47.13	6.95	50.82	9.26	-3.69	7.90E-04	1.43E-02
cg16536450	LCN15	9	1stExon	S_Shelf	68.69	4.03	72.38	4.33	-3.69	8.38E-05	2.41E-03
cg02970696	ZNF148	3	5'UTR	Island	23.54	5.78	27.23	6.66	-3.69	5.44E-04	1.08E-02
cg19145055		3	Intergenic	Open sea	69.59	5.36	73.28	5.27	-3.69	1.47E-03	2.26E-02
cg19714347	TRIM26	6	5'UTR	Open sea	24.65	3.11	28.33	4.28	-3.69	2.91E-05	9.72E-04
cg16029939	DRD4	11	Body	Island	53.73	6.93	57.42	6.42	-3.69	4.15E-03	4.68E-02
cg20980653	COL11A2;COL11A2;COL11A2	6	Body;Body;Body	Open sea	38.74	4.43	42.43	4.36	-3.69	3.89E-05	1.25E-03
cg07098722	PARK2;PARK2;PARK2	6	Body;Body;Body	Open sea	53.32	3.97	57.01	4.42	-3.69	1.05E-04	2.91E-03
cg21945260		2	Intergenic	Open sea	60.73	6.45	64.42	5.18	-3.69	5.96E-04	1.16E-02
cg03692651		19	Intergenic	Island	12.31	4.61	15.99	5.71	-3.69	1.99E-03	2.82E-02
cg21240283	FAM3C;FAM3C	7	5'UTR;5'UTR	N_Shelf	42.09	4.72	45.78	4.25	-3.69	1.25E-03	2.02E-02
cg12220663	MATN3	2	Body	S_Shelf	77.48	4.27	81.17	4.14	-3.69	2.66E-05	9.00E-04
cg07238439	TPCN1;TPCN1	12	Body;Body	Open sea	33.49	4.10	37.17	5.18	-3.69	9.77E-04	1.68E-02
cg26089753	ESR1;ESR1;ESR1;ESR1	6	TSS1500;5'UTR;TSS1500;5'UTR	N_Shore	78.56	5.58	82.24	4.78	-3.69	7.82E-04	1.42E-02
cg17626372	NOD1	7	Body	Open sea	61.09	4.27	64.78	3.98	-3.69	8.62E-05	2.47E-03
cg08449558		3	Intergenic	S_Shelf	87.29	7.38	90.98	2.53	-3.69	3.61E-03	4.26E-02
cg07948480		2	Intergenic	Open sea	79.26	4.38	82.95	3.88	-3.69	5.36E-05	1.65E-03
cg03817727		2	Intergenic	S_Shore	57.83	6.73	61.51	4.66	-3.69	2.84E-03	3.60E-02
cg26239297	ABHD12B;ABHD12B	14	Body;Body	Open sea	57.17	6.31	60.85	4.30	-3.69	2.63E-03	3.42E-02
cg13424029		10	Intergenic	Island	34.36	3.92	38.05	3.85	-3.69	1.12E-04	3.07E-03
cg14378924	SLC44A4	6	Body	S_Shore	68.20	4.67	71.89	4.67	-3.68	3.50E-04	7.68E-03
cg07533100		16	Intergenic	Open sea	67.72	6.46	71.40	5.57	-3.68	1.69E-03	2.51E-02
cg08670281	CYP4F11;CYP4F11	19	3'UTR;3'UTR	S_Shore	79.90	4.79	83.59	3.25	-3.68	1.23E-04	3.32E-03
cg22404450	LY6G5C	6	TSS1500	Island	9.97	4.20	13.66	4.91	-3.68	3.32E-04	7.37E-03
cg22804362		13	Intergenic	Open sea	37.79	3.90	41.47	4.81	-3.68	1.83E-03	2.65E-02
cg03467813	FAM50B	6	3'UTR	Island	48.10	4.92	51.79	3.71	-3.68	2.39E-04	5.69E-03
cg15537269		8	Intergenic	Open sea	53.98	5.74	57.67	5.91	-3.68	1.03E-03	1.76E-02
cg25745379	GPR12	13	Body	N_Shore	79.73	4.78	83.41	4.35	-3.68	1.19E-03	1.95E-02
cg26468696	PCDH12	5	Body	Open sea	31.68	4.18	35.37	4.09	-3.68	9.01E-05	2.57E-03
cg06971096	PTPRN	2	Body	N_Shore	18.73	4.04	22.41	4.21	-3.68	2.06E-04	5.05E-03
cg27075917		17	Intergenic	N_Shelf	70.50	4.75	74.18	3.67	-3.68	1.20E-03	1.96E-02
cg24731952		19	Intergenic	Island	69.37	5.31	73.05	4.70	-3.68	1.78E-03	2.60E-02
cg17478282	TTC24	1	TSS200	Open sea	57.84	3.52	61.52	3.87	-3.68	2.95E-05	9.85E-04
cg26516200		15	Intergenic	N_Shelf	58.79	6.16	62.47	4.75	-3.68	3.39E-04	7.49E-03
cg12724894	DIP2C	10	Body	S_Shelf	51.57	5.10	55.25	5.13	-3.68	3.48E-03	4.15E-02
cg13863078	SPCS3	4	Body	S_Shelf	72.05	5.67	75.73	5.97	-3.68	6.26E-04	1.20E-02
cg13827458	ERO1L	14	3'UTR	Open sea	80.42	6.12	84.09	4.96	-3.68	2.05E-03	2.87E-02
cg02399048	REEP4	8	TSS1500	S_Shore	26.58	4.95	30.26	4.11	-3.68	9.40E-04	1.64E-02
cg14479139	SLC10A2	13	TSS1500	Open sea	70.04	4.85	73.72	4.25	-3.68	1.29E-03	2.06E-02
cg17851046	RFX4	12	Body	N_Shore	11.93	4.04	15.61	5.49	-3.68	1.14E-03	1.89E-02
cg02778334	EHMT2;EHMT2	6	Body;Body	N_Shore	23.50	4.18	27.18	4.97	-3.68	1.18E-05	4.37E-04
cg07489607		5	Intergenic	S_Shelf	77.53	4.31	81.21	3.29	-3.68	9.16E-05	2.60E-03
cg03078586	FTL	19	Body	S_Shore	47.50	4.52	51.18	5.86	-3.68	2.27E-03	3.09E-02
cg26772788		18	Intergenic	N_Shelf	60.37	3.80	64.05	4.86	-3.68	8.36E-04	1.50E-02
cg18824330	UPF1	19	Body	Open sea	66.49	4.57	70.16	3.98	-3.68	1.15E-04	3.14E-03
cg23716690	KIF1B;KIF1B	1	Body;Body	Open sea	42.58	5.74	46.26	5.75	-3.68	6.41E-04	1.23E-02
cg11832395		5	Intergenic	S_Shelf	34.97	5.13	38.65	5.72	-3.68	2.42E-03	3.23E-02
cg10635122	ZNF577;ZNF577;ZNF577;ZNF577	19	Body;5'UTR;5'UTR;1stExon	Island	13.44	4.67	17.12	6.75	-3.68	1.90E-03	2.73E-02
cg22438810	LCN2;LCN2	9	1stExon;5'UTR	Open sea	44.36	4.02	48.04	4.80	-3.68	7.49E-04	1.38E-02
cg19798735	IMMP2L;LRRN3;LRRN3;LRRN3	7	Body;TSS1500;TSS1500;TSS1500	Open sea	65.97	4.95	69.65	4.57	-3.68	1.49E-03	2.29E-02

cg22168987	SLC4A2	7	Body	S_Shore	60.96	4.73	64.64	5.08	-3.68	6.11E-04	1.18E-02
cg02249657	PCDHGA4;PCDHGC3;PCDHGA12;PCDH	5	Body;Body;Body;Body;Body;Body;Body;Bo	S_Shore	65.78	4.53	69.46	4.46	-3.68	6.20E-04	1.20E-02
cg14333565	NRTN;NRTN	19	5'UTR;1stExon	N_Shore	44.53	4.79	48.21	5.47	-3.68	4.41E-03	4.88E-02
cg24257776	LOC100129354	3	TSS1500	Island	49.37	3.78	53.05	4.93	-3.68	2.54E-04	5.97E-03
cg19002131	C1QTNF5;MFRP	11	3'UTR;3'UTR	N_Shore	64.64	4.97	68.32	4.78	-3.68	6.29E-04	1.21E-02
cg16282160	NCOA2	8	5'UTR	Open sea	54.02	4.96	57.70	5.19	-3.68	3.87E-03	4.47E-02
cg09622715		11	Intergenic	N_Shore	60.96	5.28	64.64	5.41	-3.68	2.08E-03	2.90E-02
cg05988266	SUCLG2	3	Body	Open sea	81.31	4.55	84.98	3.82	-3.68	8.25E-04	1.48E-02
cg21414905		6	Intergenic	Open sea	56.60	6.41	60.28	7.16	-3.68	2.90E-03	3.65E-02
cg26790091	MAPK11	22	3'UTR	N_Shelf	53.42	4.94	57.10	4.86	-3.68	5.88E-04	1.15E-02
cg03982998	LOC100130331	1	TSS1500	Open sea	40.02	4.39	43.70	4.07	-3.68	1.26E-04	3.38E-03
cg20966187		5	Intergenic	Open sea	75.40	2.57	79.07	3.23	-3.68	1.62E-07	7.96E-06
cg19074170	CNTNAP5	2	Body	Open sea	49.12	5.39	52.80	6.68	-3.68	4.17E-03	4.69E-02
cg00278984		6	Intergenic	Open sea	75.45	4.17	79.13	3.97	-3.68	6.12E-05	1.85E-03
cg05041984	SGMS1	10	5'UTR	Open sea	86.39	3.66	90.07	2.64	-3.67	5.85E-07	2.71E-05
cg23370360	CRISP1;CRISP1	6	TSS200;TSS200	Open sea	84.09	4.33	87.77	3.30	-3.67	6.07E-05	1.84E-03
cg13060236	DAB1	1	Body	Open sea	79.18	5.65	82.86	4.28	-3.67	4.75E-05	1.49E-03
cg11446193		11	Intergenic	Open sea	8.59	2.94	12.26	4.43	-3.67	8.40E-07	3.81E-05
cg05600023	WNT4	1	Body	S_Shore	64.64	3.93	68.32	4.76	-3.67	3.30E-04	7.34E-03
cg09187936	SETD1B	12	3'UTR	S_Shelf	71.85	4.04	75.52	4.51	-3.67	3.04E-04	6.88E-03
cg07317062	HOXA4;HOXA4	7	5'UTR;1stExon	Island	14.33	3.24	18.01	4.02	-3.67	7.24E-06	2.81E-04
cg18030133	PHACTR2;PHACTR2;PHACTR2;PHACTR	6	5'UTR;1stExon;5'UTR;1stExon	Open sea	70.92	4.46	74.60	4.13	-3.67	9.53E-05	2.69E-03
cg15226170	PMM1	22	TSS1500	S_Shore	18.72	4.42	22.39	4.25	-3.67	1.87E-04	4.69E-03
cg03513464		1	Intergenic	Open sea	63.99	6.01	67.66	4.34	-3.67	2.47E-03	3.27E-02
cg26822572	MAPK12	22	3'UTR	S_Shore	62.95	4.07	66.62	3.75	-3.67	2.49E-05	8.48E-04
cg24678767	CLK3	15	5'UTR	N_Shelf	59.61	4.24	63.28	4.79	-3.67	2.83E-05	9.48E-04
cg04616793	C16orf68	16	Body	Open sea	39.40	4.59	43.07	4.80	-3.67	6.38E-04	1.22E-02
cg14315198	UNC119;UNC119	17	TSS1500;TSS1500	S_Shore	47.35	3.59	51.02	3.28	-3.67	1.56E-05	5.60E-04
cg03894103	PREPL;PREPL;PREPL;PREPL;PREPL;PRE	2	5'UTR;1stExon;5'UTR;5'UTR;TSS200;TSS20	N_Shelf	81.03	4.83	84.70	3.39	-3.67	1.49E-04	3.88E-03
cg25021359		7	Intergenic	Open sea	43.87	4.94	47.54	5.38	-3.67	1.70E-03	2.52E-02
cg09117114	SLC12A4;SLC12A4;SLC12A4;SLC12A4;S	16	Body;Body;TSS200;Body;Body	N_Shelf	55.73	4.29	59.40	3.70	-3.67	3.34E-04	7.40E-03
cg07102210		14	Intergenic	S_Shore	21.19	3.96	24.86	5.43	-3.67	2.23E-03	3.05E-02
cg09795195	DHX40;DHX40	17	TSS1500;TSS1500	N_Shore	74.07	4.62	77.74	4.52	-3.67	3.74E-03	4.36E-02
cg21961487	RFX4	12	Body	Island	14.24	3.26	17.91	5.03	-3.67	1.11E-04	3.05E-03
cg12064373	BRSK2	11	Body	Open sea	24.79	4.61	28.46	4.98	-3.67	6.29E-04	1.21E-02
cg00055679		13	Intergenic	Island	15.50	2.87	19.17	5.78	-3.67	1.37E-04	3.63E-03
cg12699433	L3MBTL;L3MBTL	20	TSS200;TSS200	N_Shore	37.08	4.42	40.75	4.35	-3.67	5.85E-06	2.31E-04
cg01720033	LOC146880;LOC146880	17	Body;TSS200	Island	29.38	5.13	33.05	5.72	-3.67	3.09E-04	6.96E-03
cg15995592	07-Mar	2	Body	Open sea	37.96	4.51	41.63	4.70	-3.67	2.08E-03	2.90E-02
cg04599026	DLX6AS;DLX6	7	Body;TSS1500	Island	46.28	5.00	49.95	5.66	-3.67	1.58E-04	4.09E-03
cg20625523		2	Intergenic	Open sea	66.08	4.48	69.75	4.99	-3.67	1.16E-04	3.16E-03
cg14290616	FAM83F	22	Body	S_Shelf	9.33	3.86	13.00	6.17	-3.67	2.37E-03	3.19E-02
cg01043134	MSH5;MSH5;MSH5;MSH5	6	Body;Body;Body;Body	S_Shore	40.64	4.65	44.31	3.70	-3.67	7.86E-04	1.43E-02
cg09701582	HOXD4	2	3'UTR	S_Shore	38.93	5.52	42.60	5.32	-3.67	4.03E-03	4.59E-02
cg26530557		11	Intergenic	N_Shelf	59.61	6.73	63.28	6.96	-3.67	2.64E-03	3.43E-02
cg24601011	NRSN2	20	Body	S_Shelf	55.14	5.04	58.81	4.44	-3.67	1.90E-03	2.73E-02
cg10528989	BDKRB1	14	TSS1500	Open sea	67.43	4.40	71.10	4.28	-3.67	3.86E-04	8.28E-03
cg10400390		1	Intergenic	N_Shore	48.21	3.98	51.88	3.95	-3.67	5.85E-05	1.78E-03
cg02722214	INPP5F;INPP5F	10	Body;TSS1500	Island	56.29	4.09	59.96	4.05	-3.67	5.18E-04	1.04E-02
cg09098025	CHMP7	8	TSS1500	N_Shelf	74.19	4.97	77.86	3.79	-3.67	9.02E-06	3.43E-04
cg17884383	KLC3	19	5'UTR	N_Shore	53.93	4.38	57.60	5.17	-3.67	2.67E-04	6.22E-03
cg15855170		11	Intergenic	N_Shelf	21.00	3.79	24.67	4.81	-3.67	1.89E-04	4.72E-03
cg11128216	PAX6;PAX6;PAX6	11	Body;Body;Body	Island	20.59	3.61	24.26	3.47	-3.67	5.55E-07	2.58E-05

cg08246318		6	Intergenic	S_Shore	71.87	4.26	75.53	3.66	-3.67	1.75E-04	4.45E-03
cg25149218	PARD3B;PARD3B;PARD3B	2	Body;Body;Body	S_Shore	22.90	5.05	26.57	5.09	-3.67	4.57E-04	9.45E-03
cg07249149		7	Intergenic	Open sea	64.16	4.06	67.82	2.41	-3.67	5.93E-06	2.34E-04
cg01025836	C20orf95	20	TSS1500	N_Shore	23.91	5.27	27.58	4.79	-3.67	4.90E-04	9.97E-03
cg02017200	FUT1	19	5'UTR	Island	19.77	5.33	23.44	5.69	-3.67	1.97E-04	4.88E-03
cg07004950	PWWP2A	5	Body	Open sea	70.25	3.82	73.92	4.25	-3.67	1.16E-05	4.29E-04
cg19682666	CHAF1A	19	3'UTR	N_Shelf	73.12	3.85	76.78	4.37	-3.67	7.07E-05	2.09E-03
cg07551677	EXTL3	8	5'UTR	N_Shelf	87.22	3.63	90.89	1.95	-3.67	1.23E-06	5.44E-05
cg24519146	COL11A2;COL11A2;COL11A2;COL11A2	6	TSS200;TSS200;TSS200;TSS200	N_Shore	23.19	4.50	26.86	4.58	-3.67	2.78E-06	1.16E-04
cg03453309	PGC;PGC	6	Body;3'UTR	Open sea	50.01	3.64	53.67	4.01	-3.67	2.18E-05	7.54E-04
cg21915962		1	Intergenic	Open sea	45.79	5.08	49.45	4.97	-3.67	7.14E-04	1.33E-02
cg02293410	COMP	19	Body	Island	18.48	3.56	22.14	4.13	-3.67	7.60E-06	2.93E-04
cg09722277	PDLIM3;PDLIM3	4	Body;Body	Open sea	78.76	5.00	82.42	3.66	-3.67	7.31E-05	2.15E-03
cg18550212	ATL3	11	Body	N_Shelf	60.26	6.08	63.93	4.81	-3.67	4.15E-03	4.68E-02
cg26870725	MAST1	19	Body	S_Shore	40.62	5.38	44.29	6.24	-3.67	2.41E-04	5.73E-03
cg23199907	PDS5B	13	Body	Open sea	54.24	4.99	57.91	4.81	-3.66	1.13E-03	1.88E-02
cg14667799		17	Intergenic	Open sea	78.08	4.39	81.74	3.41	-3.66	3.41E-04	7.52E-03
cg20174247	FRMD4A	10	Body	Open sea	66.22	3.81	69.88	4.09	-3.66	5.57E-05	1.71E-03
cg09463706	CACNA1H;CACNA1H	16	Body;Body	N_Shore	42.39	3.83	46.05	5.46	-3.66	3.81E-03	4.42E-02
cg13239348		10	Intergenic	Island	17.69	3.24	21.36	4.16	-3.66	4.74E-06	1.91E-04
cg16992870		7	Intergenic	Open sea	57.20	6.89	60.87	6.62	-3.66	2.05E-03	2.88E-02
cg25140133		5	Intergenic	Open sea	68.41	4.66	72.07	4.55	-3.66	2.26E-04	5.45E-03
cg01350330		16	Intergenic	S_Shore	27.29	3.79	30.96	4.52	-3.66	3.58E-04	7.81E-03
cg27120246		14	Intergenic	S_Shelf	69.05	4.05	72.71	4.26	-3.66	1.11E-04	3.04E-03
cg12197557	LILRB5;LILRB5;LILRB5	19	Body;Body;Body	Open sea	50.95	3.41	54.62	2.63	-3.66	1.38E-06	6.08E-05
cg14559422	FAM54B;FAM54B;FAM54B;LOC64647	1	5'UTR;5'UTR;5'UTR;Body;5'UTR	S_Shore	51.81	4.08	55.48	4.47	-3.66	3.30E-04	7.34E-03
cg17092985		8	Intergenic	Open sea	62.82	5.58	66.48	5.58	-3.66	1.71E-03	2.53E-02
cg19239040		7	Intergenic	S_Shore	39.26	3.86	42.92	4.34	-3.66	1.81E-04	4.57E-03
cg27144670	TMOD3	15	Body	Open sea	29.28	4.76	32.94	4.91	-3.66	3.67E-03	4.30E-02
cg07107420	GLCCI1	7	Body	S_Shore	55.47	6.61	59.13	4.98	-3.66	7.41E-04	1.37E-02
cg08836861	NQO1;NQO1;NQO1	16	Body;Body;Body	N_Shelf	72.30	5.72	75.97	3.94	-3.66	9.76E-04	1.68E-02
cg04025761	CUX2	12	Body	Open sea	26.07	4.27	29.73	4.93	-3.66	2.34E-03	3.15E-02
cg21710408	ZNF532	18	5'UTR	S_Shelf	50.82	3.49	54.48	3.55	-3.66	3.19E-05	1.05E-03
cg17745803	MOG;MOG;MOG;MOG;MOG;MOG;M	6	Body;Body;Body;Body;Body;Body;Bo	Open sea	42.43	4.98	46.10	4.79	-3.66	2.97E-03	3.71E-02
cg23311195	DAZAP1;DAZAP1	19	3'UTR;3'UTR	Island	76.34	4.86	80.00	4.40	-3.66	1.48E-04	3.87E-03
cg08710841		6	Intergenic	S_Shore	49.26	5.50	52.92	5.41	-3.66	3.63E-03	4.27E-02
cg25889475		2	Intergenic	Open sea	85.83	5.81	89.49	4.62	-3.66	7.17E-04	1.33E-02
cg20904286		6	Intergenic	S_Shelf	77.95	4.36	81.61	4.17	-3.66	1.40E-04	3.69E-03
cg14375632	C9orf3	9	Body	N_Shelf	58.74	4.96	62.40	5.20	-3.66	3.64E-03	4.28E-02
cg17962384	KCNIP2;KCNIP2;KCNIP2;KCNIP2;KCNIP	10	Body;Body;Body;Body;Body;Body	N_Shore	19.17	4.11	22.84	5.06	-3.66	3.62E-04	7.88E-03
cg23712370		8	Intergenic	Open sea	74.16	5.74	77.83	4.75	-3.66	3.26E-03	3.96E-02
cg07747871		19	Intergenic	N_Shelf	40.25	4.29	43.91	3.90	-3.66	5.79E-06	2.29E-04
cg02462195	ADAMTS5	21	TSS1500	S_Shore	15.28	4.58	18.94	5.79	-3.66	7.29E-05	2.15E-03
cg22820316	TBX15	1	TSS1500	S_Shore	32.68	4.65	36.34	5.40	-3.66	1.50E-04	3.90E-03
cg09347176	DPEP1;DPEP1	16	5'UTR;TSS1500	Open sea	46.88	6.07	50.54	5.61	-3.66	2.70E-03	3.48E-02
cg26395193	HCCA2;DUSP8	11	Body;5'UTR	N_Shelf	55.49	4.59	59.15	4.43	-3.66	2.66E-04	6.19E-03
cg01608304		16	Intergenic	Open sea	42.94	5.31	46.60	4.89	-3.66	1.62E-03	2.43E-02
cg18090145		6	Intergenic	Open sea	71.69	5.41	75.35	4.89	-3.66	1.32E-04	3.51E-03
cg16196274		8	Intergenic	S_Shelf	30.72	5.48	34.38	5.03	-3.66	6.34E-04	1.22E-02
cg11232868		3	Intergenic	Open sea	61.24	3.67	64.90	3.90	-3.66	7.98E-05	2.32E-03
cg00009750	CDK15	2	Body	Open sea	79.89	5.40	83.55	3.89	-3.66	6.19E-05	1.87E-03
cg23454003		11	Intergenic	Open sea	53.78	7.27	57.44	8.25	-3.66	5.04E-04	1.02E-02
cg16046937	JAKMIP3	10	3'UTR	N_Shore	82.36	4.02	86.02	3.51	-3.66	4.61E-06	1.86E-04

cg09549073	HOXA5;HOXA5	7	5'UTR;1stExon	Island	18.89	3.81	22.55	3.55	-3.66	1.49E-04	3.90E-03
cg00028211		2	Intergenic	N_Shore	54.99	5.44	58.65	5.04	-3.66	9.09E-04	1.60E-02
cg24244695		11	Intergenic	Open sea	60.46	4.38	64.12	4.90	-3.66	3.61E-03	4.25E-02
cg17099072	GABBR1;GABBR1	6	TSS1500;TSS1500	S_Shore	18.56	4.53	22.22	4.77	-3.66	4.61E-04	9.52E-03
cg16103604	C8orf12;FAM167A	8	Body;Body	Open sea	42.10	4.62	45.76	5.41	-3.66	4.11E-03	4.65E-02
cg10298205	EEF1D;EEF1D;EEF1D;EEF1D;NAPRT1;E	8	3'UTR;3'UTR;3'UTR;3'UTR;TSS1500;3'UTR;	S_Shore	75.58	4.14	79.24	4.43	-3.66	3.09E-04	6.97E-03
cg20127102		11	Intergenic	S_Shelf	45.83	4.69	49.49	4.60	-3.66	2.01E-03	2.83E-02
cg05992400		10	Intergenic	Open sea	69.04	4.99	72.70	4.03	-3.66	5.23E-04	1.05E-02
cg06314001		6	Intergenic	Open sea	74.61	5.42	78.27	3.50	-3.66	3.30E-03	4.00E-02
cg07325001		22	Intergenic	S_Shore	49.92	3.83	53.58	4.92	-3.66	8.72E-04	1.55E-02
cg10099808		7	Intergenic	Open sea	74.78	4.46	78.43	5.17	-3.66	1.61E-03	2.42E-02
cg21915449	HAVCR1;HAVCR1	5	3'UTR;3'UTR	Open sea	40.07	6.28	43.72	5.50	-3.66	3.56E-03	4.22E-02
cg06779421		16	Intergenic	S_Shelf	75.52	5.21	79.17	4.19	-3.66	8.02E-04	1.45E-02
cg13423054		3	Intergenic	Open sea	69.02	4.03	72.68	4.17	-3.66	2.65E-04	6.18E-03
cg13998850	CPNE6;CPNE6	14	1stExon;5'UTR	Open sea	65.47	4.24	69.12	2.91	-3.66	8.96E-05	2.56E-03
cg26344867		4	Intergenic	Open sea	51.72	5.79	55.37	5.61	-3.66	1.28E-03	2.06E-02
cg27092995	FAM38A	16	Body	Open sea	80.90	6.45	84.56	4.87	-3.66	6.83E-04	1.29E-02
cg18810681		12	Intergenic	Open sea	21.04	4.13	24.70	4.83	-3.66	7.74E-04	1.41E-02
cg17588293	ZBTB7B	1	5'UTR	N_Shelf	18.92	2.44	22.57	2.27	-3.66	1.54E-08	8.14E-07
cg04363536	NICN1	3	TSS200	S_Shore	50.20	6.37	53.86	6.97	-3.66	2.25E-03	3.07E-02
cg00067274	DCHS2;DCHS2	4	TSS1500;Body	Open sea	46.61	5.77	50.26	5.49	-3.66	2.26E-03	3.08E-02
cg08642771		1	Intergenic	Open sea	77.97	4.77	81.63	5.04	-3.66	4.17E-06	1.69E-04
cg20100445	C10orf93	10	TSS1500	Island	26.92	5.07	30.58	5.13	-3.66	1.06E-03	1.79E-02
cg05510560	KRTAP4-8	17	TSS1500	Open sea	67.48	5.64	71.14	4.58	-3.66	1.45E-03	2.25E-02
cg18025133	RPS15AP10;GPBP1L1	1	Body;Body	Open sea	73.92	4.34	77.58	4.36	-3.66	1.55E-04	4.03E-03
cg26661718	THRAP3	1	5'UTR	Open sea	26.83	3.93	30.49	5.75	-3.66	3.33E-03	4.02E-02
cg09034580		2	Intergenic	N_Shelf	77.75	4.64	81.41	4.48	-3.66	1.02E-04	2.84E-03
cg19476788	NFATC1;NFATC1;NFATC1;NFATC1;NF/	18	Body;5'UTR;Body;Body;Body	Island	48.19	6.01	51.85	4.90	-3.66	3.44E-03	4.12E-02
cg22729543	ARPC1B	7	Body	S_Shore	84.47	3.97	88.12	2.53	-3.66	3.13E-05	1.03E-03
cg26640547	MT1B	16	TSS200	Open sea	69.16	4.57	72.82	3.83	-3.66	2.55E-04	5.99E-03
cg02010711	CCHCR1;CCHCR1;TCF19;TCF19;CCHCR	6	TSS1500;TSS1500;5'UTR;5'UTR;TSS1500	S_Shore	42.18	5.22	45.84	5.98	-3.66	3.08E-04	6.95E-03
cg23101469	PROCR	20	TSS1500	N_Shelf	57.80	3.65	61.45	3.09	-3.65	2.71E-05	9.14E-04
cg25649641		15	Intergenic	Island	28.18	4.70	31.83	5.58	-3.65	3.40E-03	4.09E-02
cg22709783		5	Intergenic	Open sea	69.59	4.38	73.24	4.72	-3.65	7.73E-04	1.41E-02
cg16974014	MIR130B	22	TSS200	S_Shore	73.44	5.14	77.09	5.66	-3.65	4.02E-03	4.58E-02
cg08621447		2	Intergenic	Island	17.76	3.91	21.42	4.76	-3.65	9.75E-05	2.74E-03
cg01517776	CD81	11	Body	Island	56.56	3.93	60.21	4.64	-3.65	1.17E-03	1.93E-02
cg26074349		12	Intergenic	Island	18.54	4.05	22.20	3.67	-3.65	7.96E-05	2.31E-03
cg04305912	BCL11A;BCL11A;BCL11A	2	3'UTR;Body;Body	N_Shelf	77.43	5.26	81.08	4.98	-3.65	1.02E-03	1.74E-02
cg16601494	C1orf70;C1orf70	1	5'UTR;1stExon	N_Shore	19.70	4.03	23.35	5.40	-3.65	3.78E-04	8.16E-03
cg12837552	RAI1	17	5'UTR	S_Shore	10.78	3.71	14.43	3.50	-3.65	4.42E-06	1.79E-04
cg06257058		7	Intergenic	N_Shelf	70.69	4.88	74.34	3.84	-3.65	1.26E-04	3.38E-03
cg22201588	CCDC151	19	Body	Island	18.22	3.36	21.87	3.39	-3.65	8.69E-07	3.93E-05
cg21355828		7	Intergenic	Island	20.26	3.91	23.91	4.78	-3.65	4.79E-04	9.80E-03
cg10586423		1	Intergenic	Open sea	80.60	5.18	84.25	3.30	-3.65	3.92E-05	1.26E-03
cg23400222		5	Intergenic	Open sea	72.33	4.65	75.99	3.86	-3.65	3.55E-05	1.16E-03
cg11117131	SLN	11	TSS200	Open sea	39.07	4.68	42.72	4.81	-3.65	2.32E-03	3.14E-02
cg07176692	SLC16A1	1	TSS1500	S_Shore	52.26	8.00	55.91	7.74	-3.65	4.03E-03	4.59E-02
cg10211454	USPL1	13	Body	S_Shelf	79.50	6.36	83.15	6.59	-3.65	1.86E-03	2.69E-02
cg06754606	PITPNM1;PITPNM1	11	3'UTR;3'UTR	N_Shore	58.60	4.08	62.25	3.39	-3.65	1.89E-04	4.71E-03
cg01750261	EMP2	16	5'UTR	N_Shelf	42.23	4.31	45.89	4.59	-3.65	2.98E-04	6.77E-03
cg22225886		8	Intergenic	Open sea	58.74	4.77	62.39	4.79	-3.65	1.48E-03	2.28E-02
cg08061463		15	Intergenic	N_Shelf	60.77	5.74	64.42	5.27	-3.65	3.10E-03	3.83E-02

cg08048699	TIMM10	11	Body	Open sea	74.20	3.36	77.85	3.08	-3.65	1.94E-05	6.81E-04
cg09030187		1	Intergenic	Open sea	25.95	4.48	29.60	5.05	-3.65	2.40E-03	3.21E-02
cg25953310	ACAD10;ACAD10	12	Body;Body	Open sea	79.87	3.67	83.52	3.20	-3.65	2.63E-05	8.92E-04
cg07068735	RAB8B	15	Body	Open sea	44.90	7.84	48.55	9.49	-3.65	7.31E-04	1.35E-02
cg23815646	ADCY8	8	Body	Open sea	14.78	4.54	18.43	6.13	-3.65	3.10E-03	3.83E-02
cg06224893	DNMT3A;DNMT3A;DNMT3A	2	Body;Body;Body	Open sea	79.74	6.26	83.39	5.41	-3.65	8.07E-04	1.46E-02
cg13460073	SFXN3	10	3'UTR	N_Shore	34.00	3.96	37.65	4.58	-3.65	5.65E-04	1.11E-02
cg06759781	SHE	1	3'UTR	Open sea	61.97	5.50	65.62	5.65	-3.65	1.76E-03	2.59E-02
cg10472320	ZFHX3;ZFHX3	16	Body;Body	Open sea	56.74	5.78	60.39	4.30	-3.65	2.42E-03	3.23E-02
cg05045027		11	Intergenic	Open sea	59.69	7.13	63.34	6.70	-3.65	2.71E-03	3.49E-02
cg18449062	CNNM2;CNNM2;CNNM2	10	1stExon;1stExon;1stExon	S_Shore	28.31	3.49	31.96	4.38	-3.65	4.25E-05	1.35E-03
cg26502852	XAF1;XAF1;XAF1;XAF1	17	5'UTR;5'UTR;1stExon;1stExon	Open sea	11.13	3.80	14.78	5.95	-3.65	2.06E-03	2.89E-02
cg03720605		6	Intergenic	Open sea	24.29	4.25	27.94	4.29	-3.65	1.24E-04	3.34E-03
cg19271658	WDR46;WDR46	6	Body;Body	S_Shore	46.79	3.34	50.43	3.68	-3.65	1.69E-04	4.32E-03
cg03854238	FLJ22536	6	Body	Open sea	64.97	5.17	68.61	4.26	-3.65	6.67E-04	1.26E-02
cg05567435	METTL7B	12	TSS1500	Open sea	55.65	4.58	59.30	4.66	-3.65	9.75E-04	1.68E-02
cg09590377		2	Intergenic	Island	23.80	5.17	27.45	5.40	-3.65	1.65E-03	2.46E-02
cg09310065	WDR20;WDR20;WDR20;WDR20	14	Body;Body;Body;Body	Open sea	82.17	5.66	85.81	4.01	-3.65	3.59E-03	4.24E-02
cg16700364		2	Intergenic	S_Shore	35.14	4.92	38.79	5.36	-3.65	1.05E-04	2.92E-03
cg12012319	GRM8;GRM8;GRM8	7	Body;Body;Body	Open sea	42.98	4.64	46.62	5.11	-3.65	3.12E-03	3.84E-02
cg23262073		20	Intergenic	Island	67.86	5.25	71.51	5.87	-3.65	2.40E-03	3.21E-02
cg06479607	BAIAP2;BAIAP2;BAIAP2;BAIAP2	17	Body;Body;Body;Body	S_Shelf	64.60	4.37	68.24	4.40	-3.65	1.11E-04	3.06E-03
cg14496799	PALM2;PALM2-AKAP2;PALM2;PALM2	9	Body;Body;Body;Body	Open sea	53.96	4.08	57.61	5.87	-3.65	2.37E-03	3.18E-02
cg10855773		12	Intergenic	Open sea	53.47	5.48	57.11	5.37	-3.65	2.61E-03	3.40E-02
cg04201347	PRRG2	19	Body	S_Shelf	66.65	3.94	70.30	3.26	-3.65	2.10E-05	7.30E-04
cg03645522		4	Intergenic	Open sea	41.02	3.86	44.66	5.79	-3.65	3.35E-03	4.04E-02
cg25523704	DIRC3	2	TSS200	Island	18.97	2.93	22.62	4.09	-3.65	6.83E-06	2.66E-04
cg19618601		2	Intergenic	Open sea	52.24	5.29	55.89	5.91	-3.65	1.14E-03	1.89E-02
cg24418182	BHLHA15	7	TSS200	N_Shore	82.25	5.41	85.90	3.90	-3.65	5.86E-04	1.15E-02
cg09896211	VAV3;VAV3	1	Body;TSS1500	Open sea	77.00	3.35	80.65	2.72	-3.65	2.57E-07	1.24E-05
cg27444437	FARP2	2	5'UTR	S_Shore	16.98	4.85	20.62	5.26	-3.65	3.23E-03	3.94E-02
cg20277416	TM7SF2;C11orf2	11	TSS1500;3'UTR	Island	62.27	4.48	65.92	4.90	-3.65	2.21E-03	3.03E-02
cg22094306	FAM168B	2	Body	Open sea	42.90	4.04	46.54	5.34	-3.64	2.14E-03	2.96E-02
cg15833353	ADAMTS12	5	Body	Open sea	20.85	3.68	24.50	3.84	-3.64	3.56E-05	1.16E-03
cg01717201	PDK2	17	Body	S_Shore	52.87	4.27	56.52	4.25	-3.64	5.69E-04	1.12E-02
cg21908972	PPAP2C;PPAP2C;PPAP2C	19	3'UTR;3'UTR;3'UTR	Open sea	53.76	3.86	57.40	6.11	-3.64	1.85E-03	2.67E-02
cg13482375		17	Intergenic	Open sea	71.20	7.20	74.85	5.95	-3.64	1.50E-03	2.30E-02
cg11547613	FGFR1;FGFR1;FGFR1;FGFR1;FGFR1;FG	8	Body;Body;Body;Body;Body;Body;Body	Open sea	64.54	4.61	68.18	4.70	-3.64	2.47E-04	5.85E-03
cg11039361	MARK2;MARK2;MARK2;MARK2;MARI	11	TSS1500;Body;Body;Body;Body	Open sea	20.74	5.45	24.38	5.34	-3.64	1.56E-05	5.60E-04
cg21587469	DGKA	12	TSS1500	N_Shore	56.73	5.16	60.38	4.79	-3.64	2.15E-03	2.97E-02
cg02909790	HIST1H3G	6	1stExon	Island	19.00	3.08	22.64	4.14	-3.64	3.40E-06	1.40E-04
cg14156650	ZNF598	16	Body	Island	48.47	4.40	52.11	4.15	-3.64	3.14E-04	7.06E-03
cg18605987	ZCCHC24	10	Body	N_Shore	17.90	3.66	21.54	4.65	-3.64	3.77E-04	8.14E-03
cg00336700		2	Intergenic	N_Shore	26.64	4.24	30.28	3.50	-3.64	7.08E-05	2.09E-03
cg22092521	CFD	19	TSS1500	N_Shore	45.12	7.19	48.76	5.94	-3.64	4.18E-03	4.70E-02
cg08106319	ZFAND3	6	Body	Open sea	74.35	5.69	77.99	4.22	-3.64	3.82E-04	8.22E-03
cg15374751	LPIN1	2	Body	Island	38.74	3.28	42.38	3.73	-3.64	5.03E-06	2.01E-04
cg25520679	HIC1;HIC1	17	TSS1500;5'UTR	Island	13.98	3.34	17.62	3.89	-3.64	1.19E-06	5.29E-05
cg06086563	ZNF764	16	Body	Island	81.29	4.26	84.93	3.71	-3.64	8.06E-05	2.34E-03
cg01051318	CAD	2	Body	Island	35.76	3.84	39.40	4.76	-3.64	1.22E-04	3.31E-03
cg22016447		4	Intergenic	Open sea	62.60	5.24	66.24	4.61	-3.64	8.52E-04	1.52E-02
cg21344200		12	Intergenic	Open sea	64.98	3.55	68.62	3.80	-3.64	8.68E-06	3.31E-04
cg17525418	ACTR2;ACTR2	2	Body;Body	Open sea	68.10	5.01	71.74	4.91	-3.64	1.78E-03	2.61E-02

cg06704884		4	Intergenic	Open sea	28.14	4.13	31.78	4.95	-3.64	2.38E-03	3.19E-02
cg20679513	RGL2;RGL2	6	Body;Body	N_Shore	77.19	4.00	80.84	3.91	-3.64	1.06E-04	2.95E-03
cg13811168	DUSP8;HCCA2	11	Body;Body	S_Shelf	74.72	5.05	78.37	4.76	-3.64	3.62E-05	1.18E-03
cg24676120	IGF1;IGF1;IGF1;IGF1	12	Body;Body;Body;Body	Open sea	80.15	5.62	83.79	3.68	-3.64	3.98E-03	4.55E-02
cg07933682	TLE2;TLE2;TLE2	19	Body;Body;Body	N_Shelf	72.83	4.69	76.47	3.75	-3.64	4.59E-04	9.48E-03
cg20208394	TADA2B	4	Body	Island	38.01	3.87	41.65	4.52	-3.64	2.42E-03	3.23E-02
cg02955548	FAM155A	13	Body	Open sea	63.79	5.20	67.43	3.80	-3.64	3.31E-04	7.36E-03
cg17900356		3	Intergenic	Island	33.48	5.95	37.12	6.89	-3.64	2.71E-04	6.29E-03
cg02691325	TMEM151B	6	Body	Island	29.13	5.14	32.77	6.69	-3.64	2.46E-03	3.27E-02
cg03673688	SEC14L2	22	3'UTR	N_Shore	44.73	4.37	48.37	4.68	-3.64	2.00E-03	2.83E-02
cg00745835	LOC283050;LOC283050;LOC283050	10	Body;Body;Body	Open sea	65.92	6.89	69.56	5.64	-3.64	4.36E-03	4.84E-02
cg07638500	MYLK;MYLK;MYLK;MYLK	3	Body;Body;Body;Body	Open sea	32.82	4.37	36.46	3.69	-3.64	4.38E-04	9.15E-03
cg04481096	HOXA2	7	1stExon	N_Shore	17.72	3.67	21.36	3.76	-3.64	8.28E-06	3.17E-04
cg05754179		11	Intergenic	Open sea	58.76	4.98	62.40	5.82	-3.64	1.03E-03	1.75E-02
cg16664394	CCNO	5	Body	Island	20.68	3.21	24.32	3.49	-3.64	6.13E-08	3.11E-06
cg01837218		14	Intergenic	Open sea	60.88	4.20	64.52	4.23	-3.64	5.04E-05	1.57E-03
cg15720017	CUL3	2	Body	Open sea	84.58	6.25	88.22	5.00	-3.64	5.71E-04	1.12E-02
cg23642392	SLC2A9	4	TSS1500	Open sea	56.96	5.79	60.60	6.21	-3.64	2.54E-03	3.34E-02
cg08005992	PAX6;PAX6;PAX6	11	TSS200;5'UTR;TSS200	Island	25.59	3.75	29.23	4.21	-3.64	1.20E-05	4.43E-04
cg11821439		7	Intergenic	Open sea	36.37	4.60	40.01	4.97	-3.64	7.47E-04	1.38E-02
cg17736443	HIST1H2AJ;HIST1H2BM	6	TSS1500;1stExon	S_Shore	15.78	3.88	19.42	5.56	-3.64	4.07E-06	1.66E-04
cg24862548	PTDSS1	8	3'UTR	Open sea	83.42	4.44	87.06	3.53	-3.64	3.18E-04	7.12E-03
cg04038844		9	Intergenic	Island	60.35	6.43	63.99	4.77	-3.64	2.80E-03	3.57E-02
cg15006681	H1F0	22	TSS1500	N_Shore	64.07	4.23	67.70	3.63	-3.64	2.89E-04	6.61E-03
cg00349607	MACF1	1	TSS1500	N_Shore	17.75	3.28	21.39	3.57	-3.64	3.69E-06	1.51E-04
cg14409958	ENPP2;ENPP2;ENPP2	8	TSS1500;TSS1500;TSS1500	Open sea	30.65	5.83	34.28	6.97	-3.64	1.56E-03	2.36E-02
cg11719049	NFIC;NFIC	19	Body;Body	N_Shore	55.36	3.72	58.99	4.42	-3.64	7.70E-05	2.25E-03
cg09989727	TCF12;TCF12;TCF12;TCF12;TCF12	15	Body;Body;Body;Body;Body	Open sea	83.18	4.71	86.82	3.69	-3.64	2.82E-05	9.43E-04
cg01655158		8	Intergenic	Open sea	78.80	4.77	82.44	4.24	-3.64	5.55E-04	1.10E-02
cg11580790	DHDPSL;DHDPSL	10	Body;Body	Open sea	30.95	4.31	34.58	3.83	-3.64	1.13E-04	3.09E-03
cg12952924		18	Intergenic	N_Shore	68.79	4.94	72.43	3.94	-3.64	3.37E-04	7.45E-03
cg02501166	TMEM132C	12	Body	Open sea	61.28	6.69	64.91	5.64	-3.63	2.03E-03	2.86E-02
cg05355346	UTP23	8	TSS1500	N_Shore	80.04	5.93	83.67	5.03	-3.63	2.51E-03	3.31E-02
cg00475509	ZIC1	3	TSS1500	N_Shore	43.50	6.28	47.14	5.99	-3.63	3.86E-03	4.46E-02
cg07745707	ZNF469	16	1stExon	Island	75.14	6.07	78.77	5.62	-3.63	2.58E-03	3.37E-02
cg14819891	SLIT3	5	Body	Open sea	39.71	4.20	43.34	5.99	-3.63	2.00E-03	2.83E-02
cg09221450		13	Intergenic	Open sea	80.67	3.65	84.30	3.61	-3.63	8.02E-06	3.08E-04
cg13406085	ABCD2	12	Body	Open sea	73.91	4.97	77.54	4.35	-3.63	6.57E-05	1.96E-03
cg13744039		3	Intergenic	Open sea	61.57	5.10	65.21	4.45	-3.63	7.23E-04	1.34E-02
cg00432328	MYST4	10	5'UTR	S_Shelf	74.19	6.22	77.82	6.06	-3.63	2.62E-03	3.41E-02
cg12439199	GABRG3	15	Body	Open sea	52.09	5.83	55.72	4.44	-3.63	3.10E-03	3.83E-02
cg01559597		6	Intergenic	Open sea	81.14	4.57	84.77	3.54	-3.63	1.43E-04	3.75E-03
cg14153126	ODF2;ODF2;ODF2	9	1stExon;5'UTR;5'UTR	Island	35.11	7.57	38.74	8.25	-3.63	9.15E-04	1.60E-02
cg21806580		5	Intergenic	Open sea	28.66	4.52	32.29	5.55	-3.63	3.32E-03	4.02E-02
cg16967722		7	Intergenic	Open sea	61.10	4.37	64.73	4.20	-3.63	3.64E-05	1.18E-03
cg19107504	LDB3;LDB3;LDB3	10	Body;Body;Body	N_Shelf	61.21	4.37	64.84	3.98	-3.63	6.44E-04	1.23E-02
cg03131366		12	Intergenic	Open sea	20.73	3.47	24.36	5.36	-3.63	1.84E-03	2.66E-02
cg00716277	PTPRN2;PTPRN2;PTPRN2	7	Body;Body;Body	Island	48.08	5.47	51.71	5.12	-3.63	1.75E-03	2.57E-02
cg22997768	TAS2R14;PRR4;PRH1	12	TSS1500;Body;5'UTR	Open sea	83.54	5.41	87.17	4.27	-3.63	9.76E-04	1.68E-02
cg23090283	CSNK2B	6	Body	S_Shelf	72.65	3.93	76.28	3.26	-3.63	2.42E-06	1.02E-04
cg23698023	PTK2B;PTK2B;PTK2B;PTK2B	8	TSS1500;TSS1500;5'UTR;5'UTR	N_Shore	49.69	4.07	53.32	3.73	-3.63	2.20E-04	5.33E-03
cg07848409	GHSR;GHSR	3	TSS1500;TSS1500	N_Shore	25.08	5.92	28.71	6.50	-3.63	1.54E-03	2.35E-02
cg24759599	OSBPL5;OSBPL5;OSBPL5	11	Body;Body;Body	Island	62.29	3.58	65.92	4.28	-3.63	5.01E-05	1.56E-03

cg21656251	HDAC4	2	Body	N_Shore	76.59	3.72	80.22	3.15	-3.63	3.91E-05	1.26E-03
cg27480727	PAX3;PAX3;PAX3;PAX3;PAX3;CCDC14(	2	TSS1500;TSS1500;TSS1500;TSS1500;TSS15	S_Shore	18.67	3.58	22.31	4.36	-3.63	2.75E-04	6.37E-03
cg05063632	COL4A2	13	Body	N_Shelf	35.76	5.40	39.39	5.31	-3.63	1.92E-03	2.74E-02
cg24803517	MCTP1	5	TSS1500	S_Shore	32.19	4.66	35.82	4.97	-3.63	1.06E-03	1.78E-02
cg20207829		8	Intergenic	Open sea	60.57	5.96	64.20	6.10	-3.63	3.01E-03	3.75E-02
cg10279148	CARHSP1;CARHSP1	16	5'UTR;5'UTR	N_Shelf	28.67	4.22	32.30	5.29	-3.63	6.56E-04	1.25E-02
cg26372400	ACVRL1	12	TSS1500	Island	15.44	3.04	19.07	4.25	-3.63	2.35E-06	9.95E-05
cg01660999	ANGPT1	8	Body	Open sea	58.39	5.63	62.02	5.44	-3.63	2.25E-03	3.07E-02
cg08486507		16	Intergenic	N_Shelf	32.19	5.15	35.82	4.14	-3.63	7.11E-05	2.10E-03
cg04930596	NCOR2;NCOR2	12	Body;Body	Open sea	57.55	5.07	61.18	4.79	-3.63	1.97E-03	2.80E-02
cg11385616	ACHE;ACHE	7	Body;Body	Island	73.34	5.27	76.97	5.25	-3.63	8.13E-04	1.47E-02
cg05798501	NOD1	7	Body	Open sea	74.45	4.16	78.08	3.74	-3.63	2.87E-05	9.60E-04
cg20546782	TRPV1	17	5'UTR	Open sea	68.45	4.04	72.08	3.80	-3.63	1.43E-04	3.76E-03
cg24796272		8	Intergenic	Island	29.45	3.24	33.08	4.31	-3.63	7.26E-08	3.66E-06
cg21609584	NPAS2	2	5'UTR	Open sea	46.45	4.30	50.08	4.58	-3.63	7.14E-05	2.11E-03
cg03512692	SAMM50	22	TSS1500	N_Shore	53.41	5.55	57.04	5.48	-3.63	1.59E-03	2.40E-02
cg09757430		13	Intergenic	Open sea	20.29	3.96	23.92	4.95	-3.63	1.22E-03	1.99E-02
cg19333963	APC2	19	Body	Island	14.85	4.88	18.48	6.79	-3.63	2.52E-03	3.32E-02
cg17711915	SLC6A12;SLC6A12;SLC6A12	12	Body;Body;Body	S_Shore	79.51	4.65	83.14	4.26	-3.63	6.01E-04	1.17E-02
cg27662539		7	Intergenic	N_Shelf	55.06	3.82	58.69	4.13	-3.63	1.90E-04	4.75E-03
cg02347652	PSMD9	12	Body	Open sea	52.16	4.32	55.79	3.54	-3.63	4.99E-04	1.01E-02
cg10736208	TNXB	6	TSS1500	Open sea	29.30	3.83	32.92	5.18	-3.63	1.08E-03	1.82E-02
cg03731268	L1TD1;L1TD1	1	5'UTR;5'UTR	Island	21.40	3.69	25.03	4.39	-3.63	3.48E-04	7.64E-03
cg20644425	MIR372;MIR371;MIR373	19	TSS1500;TSS200;TSS1500	Open sea	86.24	5.41	89.87	4.07	-3.63	2.66E-04	6.20E-03
cg15089567	MAD1L1;MAD1L1;MAD1L1	7	Body;Body;Body	Open sea	78.33	4.33	81.96	3.75	-3.63	1.63E-05	5.84E-04
cg17162682	ZRANB3	2	Body	Open sea	84.11	5.57	87.74	3.13	-3.63	2.26E-03	3.08E-02
cg03817107	FOXI1;FOXI1	5	TSS200;TSS200	N_Shore	39.35	4.75	42.97	3.76	-3.63	5.86E-04	1.15E-02
cg04035828	SNX29	16	Body	Open sea	63.96	4.04	67.59	3.92	-3.63	2.10E-04	5.14E-03
cg10397819		14	Intergenic	Island	62.88	3.98	66.51	4.62	-3.63	4.66E-04	9.60E-03
cg10546977	ZSWIM4	19	Body	Island	83.27	6.45	86.90	4.30	-3.63	4.14E-03	4.67E-02
cg16247183		1	Intergenic	Island	62.52	5.66	66.15	5.53	-3.63	2.16E-03	2.98E-02
cg27385590		7	Intergenic	Open sea	20.91	4.87	24.54	4.64	-3.63	1.39E-03	2.18E-02
cg04482331	TMEM63B	6	Body	Island	32.68	4.09	36.30	5.45	-3.63	1.13E-03	1.88E-02
cg06059360	NKTR	3	Body	Open sea	80.62	4.78	84.24	4.93	-3.63	1.12E-03	1.86E-02
cg08161163	VWA1;VWA1	1	Body;Body	N_Shore	41.46	3.34	45.09	3.41	-3.63	6.88E-05	2.04E-03
cg00031973		7	Intergenic	Open sea	50.96	4.91	54.59	4.11	-3.63	2.16E-04	5.25E-03
cg13157980	EIF2C2;EIF2C2	8	Body;Body	Open sea	47.69	8.27	51.32	7.95	-3.63	2.25E-03	3.07E-02
cg19366381	PPP1R15A	19	TSS1500	N_Shore	74.93	3.55	78.55	3.45	-3.63	2.31E-05	7.93E-04
cg20360454		2	Intergenic	S_Shelf	44.81	5.11	48.44	5.03	-3.63	5.26E-04	1.05E-02
cg11413152	ERI3	1	Body	Open sea	66.07	4.15	69.69	4.14	-3.63	7.47E-04	1.38E-02
cg02278959	ZNF286B;FOXO3B	17	Body;Body	Island	84.54	5.02	88.16	3.00	-3.63	2.49E-04	5.89E-03
cg04429589	TMEM121	14	TSS1500	Island	21.39	4.10	25.02	5.12	-3.63	8.96E-05	2.56E-03
cg16408970	BAMBI	10	TSS1500	N_Shore	18.74	2.81	22.37	3.43	-3.63	5.70E-08	2.90E-06
cg21530915	TMEM181	6	Body	S_Shore	32.85	4.72	36.47	4.90	-3.62	1.19E-03	1.95E-02
cg00322262	ABLIM2;ABLIM2;ABLIM2;ABLIM2;ABL	4	Body;Body;Body;Body;Body;Body;Body	N_Shore	75.78	5.14	79.40	4.04	-3.62	6.43E-04	1.23E-02
cg03723270	MYO3B;MYO3B;MYO3B	2	Body;Body;Body	Open sea	72.13	4.61	75.76	4.28	-3.62	1.25E-03	2.03E-02
cg24137543		5	Intergenic	S_Shore	31.49	6.44	35.12	6.33	-3.62	3.75E-03	4.37E-02
cg24531009	PILRA;PILRA;PILRA	7	Body;Body;Body	Open sea	62.52	4.74	66.14	4.12	-3.62	1.08E-04	2.98E-03
cg11399546		1	Intergenic	Open sea	32.43	4.13	36.06	5.64	-3.62	3.58E-03	4.23E-02
cg21759837		15	Intergenic	Open sea	18.98	3.64	22.60	5.46	-3.62	1.26E-04	3.39E-03
cg18219226	GUSB	7	TSS1500	S_Shore	46.65	4.72	50.28	5.46	-3.62	1.52E-03	2.32E-02
cg19586576	GJC1;GJC1	17	5'UTR;5'UTR	Island	31.88	4.41	35.50	3.84	-3.62	9.03E-06	3.43E-04
cg23194912		2	Intergenic	Open sea	39.36	4.32	42.98	4.75	-3.62	9.66E-05	2.72E-03

cg20893714	LOC100240734;FLJ12825	12	TSS200;Body	Open sea	46.29	5.32	49.91	6.24	-3.62	2.83E-03	3.60E-02
cg01717609	TRIM17;TRIM17;TRIM17;TRIM17	1	TSS1500;TSS1500;TSS1500;TSS1500	S_Shore	79.77	3.81	83.39	2.62	-3.62	3.82E-07	1.81E-05
cg09337391	ZMYND8;ZMYND8;ZMYND8	20	Body;Body;Body	S_Shelf	30.36	4.28	33.98	4.92	-3.62	2.09E-03	2.91E-02
cg19828499		2	Intergenic	N_Shelf	76.30	3.93	79.92	4.44	-3.62	1.14E-05	4.24E-04
cg13577076	PRKAR1B;PRKAR1B;PRKAR1B;PRKAR1	7	5'UTR;5'UTR;TSS1500;5'UTR;5'UTR;5'UTR	S_Shore	18.77	3.68	22.39	4.29	-3.62	1.25E-06	5.54E-05
cg11590420	MYOZ3;MYOZ3	5	Body;Body	Island	32.29	4.06	35.91	4.29	-3.62	1.03E-04	2.87E-03
cg26919149		6	Intergenic	Island	27.66	5.01	31.28	5.50	-3.62	3.96E-03	4.53E-02
cg12022621	LAX1;LAX1;LAX1	1	1stExon;1stExon;5'UTR	Open sea	61.88	4.55	65.50	5.10	-3.62	8.61E-05	2.47E-03
cg20865048	SLC39A10;SLC39A10	2	Body;Body	Open sea	76.17	5.97	79.79	6.01	-3.62	2.43E-03	3.24E-02
cg15357118	UGGT1;UGGT1	2	Body;Body	Open sea	69.25	4.56	72.87	3.87	-3.62	3.04E-06	1.26E-04
cg10092867	COL9A3	20	TSS1500	N_Shore	32.86	4.20	36.48	5.04	-3.62	2.92E-03	3.67E-02
cg08198377	TMX1	14	Body	S_Shore	24.73	4.76	28.35	6.11	-3.62	2.95E-03	3.70E-02
cg06486409	MKLN1	7	Body	Open sea	64.76	5.72	68.38	5.10	-3.62	6.66E-04	1.26E-02
cg03248292		12	Intergenic	Open sea	79.86	4.63	83.48	4.18	-3.62	6.40E-05	1.92E-03
cg02491234	PLEKHA6	1	Body	Island	54.63	3.94	58.25	5.06	-3.62	1.58E-03	2.39E-02
cg25133887	C1orf85	1	TSS1500	Open sea	79.74	6.01	83.36	5.55	-3.62	1.18E-03	1.93E-02
cg11028075	SORBS1;SORBS1;SORBS1;SORBS1;SOF	10	1stExon;5'UTR;5'UTR;5'UTR;1stExon;5'UTI	Open sea	28.47	3.74	32.09	4.50	-3.62	2.80E-04	6.45E-03
cg22940848	SKI	1	Body	Island	36.64	5.19	40.26	4.60	-3.62	6.16E-04	1.19E-02
cg17392018	CALD1;CALD1;CALD1;CALD1;CALD1	7	Body;Body;Body;Body;Body	Open sea	60.51	4.60	64.13	5.12	-3.62	1.55E-03	2.36E-02
cg24408057	SHD	19	TSS1500	N_Shore	49.92	5.39	53.54	4.52	-3.62	2.15E-03	2.97E-02
cg08344524		15	Intergenic	Open sea	43.00	4.55	46.62	3.80	-3.62	5.32E-05	1.64E-03
cg07611790	RASIP1	19	Body	N_Shore	62.74	5.53	66.36	4.91	-3.62	2.77E-03	3.54E-02
cg03511628	NFATC1;NFATC1;NFATC1;NFATC1	18	Body;Body;Body;Body	S_Shore	65.92	5.90	69.54	5.69	-3.62	1.85E-03	2.67E-02
cg16780771	CMIP;CMIP	16	Body;Body	Open sea	64.95	5.19	68.57	4.14	-3.62	9.90E-04	1.70E-02
cg18902641		6	Intergenic	S_Shore	34.42	4.60	38.04	4.62	-3.62	9.29E-04	1.62E-02
cg15114013	NFYC;NFYC;LOC100130557;NFYC;NFY(	1	5'UTR;5'UTR;TSS1500;5'UTR;5'UTR;5'UTR	S_Shore	43.15	4.10	46.77	5.18	-3.62	1.74E-03	2.56E-02
cg20579480	APOBEC4;RGL1	1	5'UTR;5'UTR	Open sea	65.00	4.90	68.62	5.44	-3.62	3.95E-03	4.53E-02
cg14545590	ZMAT4;ZMAT4	8	3'UTR;3'UTR	Open sea	65.44	5.31	69.06	4.77	-3.62	3.87E-03	4.46E-02
cg20640281	HLF	17	TSS1500	N_Shore	21.36	3.98	24.98	5.65	-3.62	3.79E-03	4.40E-02
cg21172322	BCAT1	12	Body	N_Shore	47.97	6.86	51.59	6.02	-3.62	3.26E-03	3.96E-02
cg07126235	CUL9	6	3'UTR	Open sea	44.72	8.37	48.34	8.07	-3.62	1.80E-03	2.62E-02
cg04368664		19	Intergenic	S_Shelf	71.36	4.30	74.97	3.67	-3.62	2.95E-04	6.71E-03
cg19604496		9	Intergenic	Island	14.14	2.99	17.76	3.09	-3.62	6.85E-08	3.46E-06
cg21526172	GDF1;LASS1	19	Body;3'UTR	Island	48.22	3.12	51.84	4.37	-3.62	8.12E-05	2.35E-03
cg04264633	BFSP1	20	TSS1500	Open sea	42.20	4.49	45.81	4.90	-3.62	3.31E-03	4.01E-02
cg22585954	MCC	5	Body	Open sea	58.15	5.29	61.76	4.64	-3.62	2.73E-04	6.32E-03
cg27628536		5	Intergenic	N_Shore	26.35	5.64	29.96	5.48	-3.62	3.17E-03	3.89E-02
cg01294808	IRX1	5	Body	Island	28.23	5.90	31.84	5.37	-3.62	4.17E-03	4.69E-02
cg17430268	CASKIN2;CASKIN2	17	Body;Body	Island	78.82	4.60	82.44	2.60	-3.62	8.99E-05	2.56E-03
cg11735311		1	Intergenic	S_Shore	27.20	4.03	30.82	4.68	-3.62	2.63E-04	6.14E-03
cg15626105	ABI3BP	3	Body	Open sea	50.80	4.79	54.42	4.28	-3.62	2.71E-04	6.29E-03
cg13989852		15	Intergenic	N_Shore	43.63	4.52	47.24	3.79	-3.62	4.18E-04	8.82E-03
cg25716814	NFIX	19	Body	Island	51.50	5.34	55.12	5.61	-3.62	7.31E-04	1.35E-02
cg05622422	FAM171A2	17	Body	Island	63.01	4.70	66.63	5.19	-3.61	7.53E-04	1.39E-02
cg25183263	KSR1	17	3'UTR	Open sea	81.72	3.80	85.33	3.12	-3.61	2.23E-05	7.69E-04
cg22888787		17	Intergenic	Island	60.48	4.83	64.09	4.52	-3.61	5.26E-04	1.05E-02
cg19997502		2	Intergenic	S_Shore	35.52	5.54	39.14	6.72	-3.61	2.55E-03	3.35E-02
cg05645677	SLC6A5;SLC6A5	11	5'UTR;1stExon	N_Shore	22.57	5.08	26.18	5.45	-3.61	1.17E-03	1.93E-02
cg02856842	SH3BP5L	1	3'UTR	N_Shore	43.82	4.40	47.43	4.24	-3.61	3.83E-04	8.24E-03
cg14837557	SEPSECS;SEPSECS;SEPSECS	4	Body;Body;Body	N_Shelf	83.72	5.04	87.33	3.15	-3.61	7.18E-04	1.34E-02
cg19048331	BTBD3;BTBD3	20	5'UTR;TSS1500	Open sea	69.93	6.46	73.55	7.41	-3.61	3.56E-03	4.21E-02
cg11576349	IL22RA1	1	TSS1500	Open sea	68.45	4.16	72.06	3.67	-3.61	2.42E-05	8.28E-04
cg20234910		13	Intergenic	Open sea	48.57	5.54	52.19	5.29	-3.61	2.97E-03	3.72E-02

cg04500377	DDHD2;DDHD2;DDHD2	8	5'UTR;5'UTR;5'UTR	S_Shore	40.19	4.99	43.81	5.08	-3.61	3.47E-03	4.14E-02
cg19679250	CALD1;CALD1;CALD1	7	TSS1500;TSS1500;TSS1500	Open sea	58.62	4.85	62.23	5.28	-3.61	1.63E-03	2.44E-02
cg13781678	PRDM16;PRDM16	1	Body;Body	Island	63.24	5.38	66.85	4.26	-3.61	4.56E-03	4.99E-02
cg15120525	LPP;LPP;LPP	3	5'UTR;5'UTR;5'UTR	Open sea	72.82	4.93	76.44	4.63	-3.61	6.25E-04	1.20E-02
cg08258582		1	Intergenic	Island	30.12	3.15	33.74	4.32	-3.61	9.57E-08	4.78E-06
cg14612733	EFNA1;EFNA1	1	Body;Body	S_Shelf	33.96	4.12	37.58	4.85	-3.61	2.00E-03	2.83E-02
cg01695643	GSC	14	TSS1500	N_Shore	62.69	4.23	66.30	5.55	-3.61	3.08E-04	6.94E-03
cg14211735	PSMA4;PSMA4;PSMA4	15	Body;Body;Body	Open sea	72.77	4.36	76.38	3.51	-3.61	3.63E-05	1.18E-03
cg15773072		3	Intergenic	N_Shelf	67.18	5.14	70.79	4.98	-3.61	3.44E-03	4.12E-02
cg01979888	IFFO1;IFFO1	12	TSS200;TSS200	S_Shore	7.12	2.03	10.73	4.85	-3.61	1.14E-05	4.22E-04
cg09711214	TEAD1	11	Body	Open sea	75.59	5.07	79.21	3.14	-3.61	1.14E-04	3.13E-03
cg20608259		11	Intergenic	S_Shelf	72.58	4.04	76.19	3.82	-3.61	4.72E-04	9.69E-03
cg07553761	TRIM59	3	TSS1500	Island	23.16	3.26	26.77	4.48	-3.61	5.97E-06	2.35E-04
cg18049167	PPT2;PPT2	6	TSS200;TSS1500	N_Shore	26.32	5.34	29.93	5.47	-3.61	7.90E-04	1.43E-02
cg26580480	TTBK1	6	Body	N_Shelf	78.17	4.77	81.78	3.82	-3.61	1.34E-04	3.55E-03
cg07749943	DOK7;DOK7	4	Body;Body	S_Shelf	44.15	2.92	47.76	3.48	-3.61	5.16E-06	2.06E-04
cg09184853	HRK	12	3'UTR	N_Shore	15.86	3.46	19.47	4.45	-3.61	6.12E-05	1.85E-03
cg09741404	KIAA1875	8	Body	Island	78.04	5.01	81.65	4.96	-3.61	3.65E-03	4.28E-02
cg26815414	C13orf35	13	TSS1500	N_Shelf	84.71	3.86	88.32	3.07	-3.61	3.65E-06	1.50E-04
cg10279922		2	Intergenic	Open sea	65.00	5.23	68.61	5.01	-3.61	5.69E-04	1.12E-02
cg26767614	NFIX	19	Body	Island	80.01	4.80	83.62	3.99	-3.61	2.66E-03	3.44E-02
cg03902718	PRR5L	11	TSS200	Open sea	77.59	4.48	81.20	4.30	-3.61	3.43E-05	1.12E-03
cg24383305	PTK2;PTK2	8	5'UTR;Body	Open sea	70.61	5.85	74.21	4.54	-3.61	1.20E-04	3.26E-03
cg05005217	CDCA2	8	5'UTR	S_Shore	50.77	5.39	54.38	7.18	-3.61	3.61E-03	4.25E-02
cg09803262	DLX6AS	7	Body	Open sea	25.97	4.38	29.58	4.10	-3.61	2.43E-04	5.77E-03
cg24075990		1	Intergenic	Open sea	57.77	4.29	61.38	4.08	-3.61	3.74E-05	1.21E-03
cg18406708	FAM82A1;FAM82A1;FAM82A1;FAM8	2	Body;Body;Body;Body	Open sea	38.80	6.12	42.41	5.52	-3.61	2.85E-03	3.62E-02
cg24328884	PRKACB;PRKACB;PRKACB	1	Body;Body;TSS1500	Open sea	74.49	4.21	78.10	3.97	-3.61	4.07E-05	1.30E-03
cg00464757		4	Intergenic	Open sea	69.15	5.07	72.76	4.40	-3.61	2.63E-03	3.42E-02
cg24163668	NFATC1;NFATC1;NFATC1;NFATC1;NF/	18	Body;Body;Body;Body;Body	Island	77.83	5.77	81.43	4.25	-3.61	2.51E-03	3.31E-02
cg14243778	CNTN1;CNTN1	12	5'UTR;5'UTR	Island	28.35	4.05	31.95	4.72	-3.61	1.80E-04	4.54E-03
cg03448169		16	Intergenic	Open sea	20.56	3.79	24.17	5.27	-3.61	1.49E-03	2.30E-02
cg15082014		3	Intergenic	Open sea	30.03	5.48	33.64	4.86	-3.61	1.23E-03	2.00E-02
cg10027639		11	Intergenic	Open sea	56.80	6.29	60.41	4.83	-3.61	2.52E-03	3.32E-02
cg18829068		11	Intergenic	Open sea	51.25	5.83	54.86	5.07	-3.61	2.90E-04	6.63E-03
cg09509673	CCR10;CNTNAP1	17	Body;TSS1500	N_Shore	73.91	4.98	77.52	5.34	-3.61	2.67E-03	3.46E-02
cg24264042		3	Intergenic	Open sea	72.32	4.99	75.92	3.44	-3.61	1.25E-04	3.36E-03
cg26047127	FXYD6;FXYD6;FXYD6;FXYD6;FXYD6	11	5'UTR;5'UTR;5'UTR;5'UTR;5'UTR	N_Shelf	51.40	3.49	55.00	4.68	-3.61	2.50E-04	5.90E-03
cg20224218	FAM125B	9	Body	N_Shore	38.73	3.20	42.34	3.64	-3.61	3.85E-05	1.24E-03
cg13053396	C1S;C1S	12	5'UTR;5'UTR	Open sea	26.71	3.55	30.31	5.64	-3.60	2.66E-03	3.45E-02
cg03855291	DRD4	11	Body	Island	63.17	4.97	66.77	4.32	-3.60	7.60E-04	1.39E-02
cg04932082		6	Intergenic	Open sea	31.42	4.79	35.02	5.33	-3.60	3.47E-03	4.14E-02
cg13101887	SVIL;SVIL	10	5'UTR;5'UTR	Open sea	82.25	4.52	85.86	4.05	-3.60	2.41E-04	5.74E-03
cg16912957	TRIM10;TRIM10	6	Body;Body	Open sea	28.27	7.00	31.87	8.47	-3.60	3.85E-03	4.44E-02
cg04813677		13	Intergenic	Open sea	71.77	6.31	75.38	6.76	-3.60	2.72E-03	3.50E-02
cg15624109		1	Intergenic	S_Shelf	56.07	5.63	59.68	6.12	-3.60	4.49E-03	4.94E-02
cg25563256	FGF11	17	TSS1500	N_Shore	55.19	4.31	58.80	3.77	-3.60	2.82E-04	6.47E-03
cg13039082	PAK1;PAK1	11	5'UTR;5'UTR	S_Shore	35.87	4.09	39.48	5.39	-3.60	1.63E-04	4.18E-03
cg17544364	TAF3	10	Body	Open sea	78.85	5.82	82.46	4.47	-3.60	2.16E-03	2.98E-02
cg00034154	S1PR2	19	5'UTR	S_Shore	57.53	5.57	61.13	5.16	-3.60	8.62E-04	1.53E-02
cg08813062	SP9	2	TSS1500	Island	16.88	2.83	20.48	3.25	-3.60	7.63E-08	3.84E-06
cg00662446	COL18A1;COL18A1;COL18A1	21	Body;Body;Body	Island	74.25	5.42	77.85	5.02	-3.60	2.91E-03	3.67E-02
cg03283929	NETO2	16	3'UTR	Open sea	83.70	4.08	87.31	3.49	-3.60	4.61E-05	1.45E-03

cg07660750	PAX6;PAX6;PAX6	11	5'UTR;5'UTR;5'UTR	S_Shore	25.67	5.03	29.27	4.43	-3.60	5.59E-04	1.10E-02
cg09196534	RPL13AP17;MAGI2	7	TSS1500;Body	Open sea	81.33	3.77	84.93	3.57	-3.60	7.03E-08	3.55E-06
cg10140309	ADNP;ADNP	20	TSS1500;TSS1500	S_Shore	74.72	4.99	78.33	5.20	-3.60	2.07E-03	2.90E-02
cg19702274	ARHGAP23	17	3'UTR	S_Shore	71.53	4.59	75.13	4.77	-3.60	1.01E-03	1.72E-02
cg19312305	MIR122	18	TSS1500	Open sea	33.93	4.09	37.53	4.05	-3.60	2.94E-04	6.69E-03
cg11038843	B3GAT1;B3GAT1	11	5'UTR;5'UTR	Island	11.95	4.16	15.55	4.17	-3.60	2.52E-04	5.94E-03
cg21871261	SDCBP2;SDCBP2	20	TSS200;Body	S_Shore	9.04	2.52	12.64	4.91	-3.60	4.65E-05	1.46E-03
cg18011188		2	Intergenic	Open sea	59.49	5.07	63.09	5.29	-3.60	4.57E-03	5.00E-02
cg02086801	OTP	5	Body	S_Shore	44.72	4.12	48.32	4.64	-3.60	2.16E-04	5.25E-03
cg27310024	IQGAP1	15	Body	Open sea	65.85	4.75	69.45	4.77	-3.60	1.22E-04	3.30E-03
cg14455421	ASB16	17	TSS1500	Open sea	19.83	3.83	23.43	3.32	-3.60	2.28E-05	7.85E-04
cg25770176	PANK1	10	TSS1500	S_Shore	14.82	3.05	18.42	4.68	-3.60	5.30E-04	1.06E-02
cg09607482		10	Intergenic	Open sea	66.36	5.55	69.96	3.47	-3.60	4.42E-03	4.88E-02
cg21449463		8	Intergenic	Open sea	61.80	5.07	65.40	5.36	-3.60	1.99E-03	2.82E-02
cg23926670	PTHLH;PTHLH;PTHLH;PTHLH	12	Body;Body;Body;Body	N_Shore	32.69	4.07	36.29	4.20	-3.60	8.74E-05	2.50E-03
cg07327335	PP14571;GPC1	2	Body;Body	N_Shelf	51.90	3.45	55.50	4.53	-3.60	3.21E-05	1.06E-03
cg07030287	HDLBP;HDLBP	2	Body;Body	Open sea	47.39	2.87	50.99	4.34	-3.60	8.87E-05	2.53E-03
cg05653887	CELSR1	22	Body	N_Shelf	53.33	5.79	56.93	6.03	-3.60	1.12E-03	1.87E-02
cg16433012	LOC100129637	16	TSS1500	N_Shore	46.25	3.91	49.85	3.86	-3.60	8.38E-05	2.41E-03
cg25998928		6	Intergenic	N_Shore	58.92	3.77	62.52	4.30	-3.60	8.67E-05	2.48E-03
cg06399419	C21orf90;C21orf29;C21orf90	21	Body;Body;Body	Open sea	86.68	5.53	90.28	4.03	-3.60	1.97E-03	2.80E-02
cg07437360		11	Intergenic	N_Shelf	72.82	3.38	76.42	3.16	-3.60	6.05E-06	2.38E-04
cg18511798	H19;MIR675	11	Body;TSS200	Island	67.72	3.57	71.31	4.55	-3.60	3.69E-04	7.99E-03
cg20123298		9	Intergenic	Island	72.20	3.57	75.80	3.56	-3.60	6.96E-06	2.70E-04
cg06344211	MYH9	22	Body	Island	88.75	3.96	92.35	2.26	-3.60	3.64E-06	1.49E-04
cg01533397	MDC1	6	5'UTR	N_Shore	77.81	3.04	81.41	3.18	-3.60	1.00E-05	3.77E-04
cg17083982	KIF5A	12	Body	S_Shelf	70.49	5.25	74.09	4.40	-3.60	2.81E-03	3.58E-02
cg14779990	HSD17B7	1	Body	S_Shelf	53.48	4.74	57.08	4.58	-3.60	2.45E-03	3.26E-02
cg04227961	RASAL1	12	Body	Island	12.92	2.77	16.52	4.16	-3.60	5.74E-06	2.27E-04
cg17308758	TTC7B	14	Body	Open sea	41.95	4.97	45.54	4.55	-3.60	6.00E-04	1.17E-02
cg27586596		7	Intergenic	Open sea	55.70	5.07	59.30	4.73	-3.60	4.08E-04	8.65E-03
cg06323052	EXOC1;EXOC1;EXOC1	4	5'UTR;5'UTR;5'UTR	S_Shore	61.78	4.55	65.37	6.19	-3.60	3.96E-03	4.53E-02
cg27615366	PDLIM4;PDLIM4	5	TSS1500;TSS1500	N_Shore	52.88	3.93	56.48	3.96	-3.60	8.86E-05	2.53E-03
cg01019082	ISCA1L;ISCA1L	5	1stExon;5'UTR	Open sea	74.11	3.63	77.71	2.79	-3.60	1.40E-06	6.13E-05
cg18651026	COL11A2;COL11A2;COL11A2	6	Body;Body;Body	Open sea	53.27	4.16	56.86	3.96	-3.60	2.27E-04	5.47E-03
cg09386376	DRD4	11	Body	Island	73.14	6.67	76.74	6.84	-3.60	3.11E-03	3.84E-02
cg01731500	SLC16A3;SLC16A3;SLC16A3	17	5'UTR;5'UTR;5'UTR	N_Shelf	82.03	3.94	85.63	2.87	-3.60	1.54E-05	5.54E-04
cg24620592	MGC72080	7	TSS1500	S_Shore	33.49	4.42	37.08	4.02	-3.60	4.55E-04	9.43E-03
cg02051771	PCDHA7;PCDHA12;PCDHA6;PCDHA10	5	Body;Body;Body;Body;Body;Body;Body;Bo	N_Shore	17.89	4.17	21.48	5.70	-3.59	3.79E-03	4.40E-02
cg25940946	DERL3;DERL3;DERL3	22	TSS200;TSS200;TSS200	Island	18.08	6.12	21.68	5.83	-3.59	2.95E-03	3.70E-02
cg27616541	CNTNAP1	17	Body	Island	43.10	2.97	46.69	3.83	-3.59	9.82E-05	2.76E-03
cg14190975	OLFM1	9	Body	S_Shore	78.13	4.64	81.73	4.88	-3.59	2.91E-04	6.63E-03
cg13828579	GSN;GSN;GSN;GSN;GSN;GSN;GSN;GS	9	Body;Body;Body;Body;Body;Body;Body;Bo	Open sea	58.88	5.10	62.47	4.02	-3.59	1.18E-03	1.93E-02
cg21632158		6	Intergenic	Island	41.03	5.78	44.63	4.51	-3.59	2.68E-03	3.46E-02
cg16061177	PPP2R5A;SNORA16B	1	Body;TSS1500	Open sea	51.81	4.82	55.41	4.37	-3.59	1.38E-03	2.18E-02
cg05332854		16	Intergenic	S_Shore	51.66	4.34	55.25	4.19	-3.59	8.09E-04	1.46E-02
cg19022717	C1orf92	1	Body	S_Shore	62.27	5.49	65.87	5.27	-3.59	4.35E-03	4.83E-02
cg07159518		13	Intergenic	Island	27.65	3.12	31.24	3.92	-3.59	3.63E-05	1.18E-03
cg24332743	LOC143188	10	TSS200	Open sea	60.84	5.12	64.43	4.46	-3.59	1.26E-03	2.03E-02
cg12895760	SYTL3	6	Body	Open sea	61.01	4.96	64.60	6.58	-3.59	2.82E-03	3.59E-02
cg17895149	HYMAI;PLAGL1;PLAGL1;PLAGL1;PLAG	6	Body;TSS1500;TSS1500;5'UTR;TSS1500;TS	Island	43.85	3.93	47.45	3.67	-3.59	3.73E-05	1.20E-03
cg13426340		1	Intergenic	Open sea	56.95	4.21	60.54	4.85	-3.59	6.88E-04	1.29E-02
cg07571734	PRKAG2;PRKAG2	7	Body;Body	N_Shore	51.69	4.44	55.28	4.53	-3.59	4.87E-04	9.92E-03

cg21173239	ELK3	12	TSS1500	N_Shore	69.96	4.85	73.55	4.10	-3.59	8.84E-04	1.56E-02
cg17332573	HIST1H2BH;HIST1H3F	6	TSS1500;TSS200	N_Shore	25.83	4.15	29.43	5.13	-3.59	3.20E-04	7.15E-03
cg10596768	G6PC	17	1stExon	Open sea	33.81	4.97	37.41	5.00	-3.59	2.37E-04	5.65E-03
cg26155492	GPR61	1	TSS200	Open sea	48.97	4.33	52.56	3.97	-3.59	1.73E-04	4.41E-03
cg05856387	TLX2	2	Body	Island	29.40	2.82	33.00	2.99	-3.59	8.80E-07	3.98E-05
cg14194263		9	Intergenic	Island	47.24	4.31	50.83	4.79	-3.59	2.67E-03	3.45E-02
cg03408433	CMKLR1;CMKLR1;CMKLR1	12	TSS1500;TSS1500;TSS1500	Open sea	36.09	3.05	39.68	4.07	-3.59	1.05E-05	3.93E-04
cg07440775	GAD2;GAD2	10	Body;Body	Island	20.46	3.72	24.05	5.21	-3.59	8.09E-04	1.46E-02
cg12614968		10	Intergenic	N_Shore	58.45	3.84	62.04	4.54	-3.59	5.87E-04	1.15E-02
cg05366160	HLA-DPA1	6	Body	Open sea	67.30	6.53	70.89	6.19	-3.59	1.79E-03	2.62E-02
cg04552811		3	Intergenic	Open sea	53.64	5.75	57.23	4.95	-3.59	3.58E-03	4.23E-02
cg25558617	PVT1	8	Body	Open sea	76.46	4.15	80.05	3.95	-3.59	4.76E-05	1.49E-03
cg07188513		7	Intergenic	N_Shelf	31.52	4.26	35.11	5.18	-3.59	5.11E-04	1.03E-02
cg08053686		4	Intergenic	Island	15.49	2.74	19.08	3.60	-3.59	2.53E-07	1.22E-05
cg18105139	ZSCAN12L1	6	TSS200	Open sea	32.86	4.58	36.45	4.38	-3.59	4.18E-04	8.81E-03
cg21411834	KCTD3	1	TSS1500	N_Shore	43.94	5.56	47.53	6.20	-3.59	3.28E-03	3.98E-02
cg24901447		12	Intergenic	Open sea	55.07	5.36	58.66	4.26	-3.59	1.06E-03	1.79E-02
cg14542367	TYRP1	9	Body	Open sea	74.05	5.00	77.64	5.12	-3.59	1.96E-04	4.87E-03
cg06333307	ENOX1;ENOX1	13	Body;Body	Open sea	77.62	4.34	81.21	5.97	-3.59	2.23E-04	5.40E-03
cg23502475	SFRP2	4	TSS1500	N_Shore	31.82	3.74	35.41	4.12	-3.59	6.38E-05	1.92E-03
cg14662585		7	Intergenic	Island	51.30	4.55	54.89	4.61	-3.59	4.78E-04	9.78E-03
cg06090053	KPNA2	17	5'UTR	S_Shore	34.21	6.29	37.79	6.45	-3.59	1.66E-03	2.48E-02
cg05641048	GCNT3	15	5'UTR	Open sea	53.61	4.70	57.20	4.91	-3.59	2.49E-03	3.29E-02
cg08893575	PAK4;PAK4;PAK4;PAK4;PAK4	19	5'UTR;5'UTR;5'UTR;5'UTR;5'UTR	N_Shore	68.93	4.03	72.52	3.41	-3.59	1.12E-05	4.18E-04
cg12700283	TNRC6C;TNRC6C	17	5'UTR;5'UTR	N_Shore	44.38	4.66	47.96	5.51	-3.59	1.90E-03	2.73E-02
cg01312412		13	Intergenic	Open sea	83.78	4.88	87.37	4.45	-3.59	4.36E-04	9.12E-03
cg18151262	MIDN	19	Body	Island	54.41	4.21	58.00	3.86	-3.59	5.12E-04	1.03E-02
cg14095959		4	Intergenic	Island	29.26	4.39	32.85	4.72	-3.59	3.38E-04	7.47E-03
cg00281100	RNLS	10	Body	Open sea	81.41	4.96	85.00	3.88	-3.59	1.68E-03	2.50E-02
cg15584194		2	Intergenic	Open sea	45.35	4.92	48.94	5.37	-3.59	2.43E-03	3.24E-02
cg08273750	LOC100133545	11	TSS200	N_Shelf	67.55	7.03	71.14	6.30	-3.59	3.50E-03	4.17E-02
cg00767000	ZCCHC14	16	1stExon	Open sea	17.62	3.66	21.21	4.28	-3.59	3.87E-04	8.30E-03
cg09697245	XYLT1	16	Body	Open sea	31.93	4.33	35.52	4.78	-3.59	5.48E-04	1.09E-02
cg07119001	RNPEPL1	2	5'UTR	N_Shore	55.56	4.01	59.15	3.33	-3.59	5.66E-05	1.73E-03
cg15388974	PRKD1	14	Body	Open sea	58.35	4.17	61.93	6.35	-3.59	1.26E-03	2.04E-02
cg02748089	WWOX	16	Body	Open sea	17.82	3.33	21.41	4.40	-3.59	3.75E-04	8.11E-03
cg22216952		12	Intergenic	N_Shore	76.82	5.32	80.41	4.04	-3.59	1.17E-03	1.93E-02
cg15661352	DEAF1	11	Body	Open sea	84.82	3.02	88.41	2.39	-3.59	3.62E-07	1.72E-05
cg13816428	OR5AU1	14	TSS1500	Open sea	32.87	5.67	36.45	4.17	-3.59	1.92E-03	2.74E-02
cg01422416	HNRPLL;HNRPLL	2	Body;Body	Open sea	85.02	4.26	88.61	1.72	-3.59	1.97E-05	6.91E-04
cg26413307	LRP1B	2	Body	Open sea	67.16	5.59	70.75	4.72	-3.58	2.68E-03	3.46E-02
cg02859516	ELOVL6;ELOVL6	4	Body;Body	Open sea	22.22	3.72	25.81	4.94	-3.58	6.57E-04	1.25E-02
cg24815001		7	Intergenic	Open sea	70.72	5.22	74.31	4.73	-3.58	5.07E-04	1.02E-02
cg01759226		17	Intergenic	Island	39.75	5.76	43.34	5.42	-3.58	1.77E-03	2.60E-02
cg00491021	PLAT;PLAT	8	5'UTR;5'UTR	Open sea	40.60	4.21	44.18	5.65	-3.58	3.55E-03	4.20E-02
cg09870542	CDC34	19	Body	Island	60.60	3.85	64.19	4.04	-3.58	4.95E-04	1.00E-02
cg13848707	TET1	10	5'UTR	S_Shore	19.07	5.39	22.65	6.15	-3.58	1.93E-03	2.76E-02
cg24433287	MANEAL;MANEAL;MANEAL	1	Body;Body;TSS200	S_Shore	28.03	6.25	31.61	6.51	-3.58	1.93E-03	2.76E-02
cg10556064	SMPD3	16	5'UTR	Island	42.33	4.95	45.91	5.18	-3.58	6.71E-04	1.27E-02
cg14215309		7	Intergenic	N_Shore	56.02	4.58	59.60	5.15	-3.58	2.90E-04	6.62E-03
cg23181433	PDGFRL	8	Body	Open sea	79.07	4.40	82.65	3.54	-3.58	9.35E-06	3.54E-04
cg27586487	LOC100128292;DLG5	10	Body;TSS1500	S_Shore	23.66	3.78	27.24	4.50	-3.58	3.59E-05	1.17E-03
cg17594003	ZEB2;ZEB2;ZEB2	2	TSS1500;TSS1500;TSS1500	N_Shelf	39.64	5.01	43.22	5.79	-3.58	3.60E-03	4.25E-02

cg00341732	SMTN;SMTN;SMTN	22	Body;Body;Body	N_Shore	59.92	5.54	63.50	3.90	-3.58	8.10E-04	1.46E-02
cg14882807		11	Intergenic	Open sea	74.62	4.15	78.20	4.46	-3.58	1.06E-03	1.79E-02
cg25275372	ABR;ABR;ABR	17	Body;Body;Body	Open sea	79.03	5.30	82.61	4.65	-3.58	3.46E-03	4.14E-02
cg23663332	MAK16	8	TSS1500	N_Shore	74.04	4.66	77.62	4.30	-3.58	6.31E-04	1.21E-02
cg10195726	ZFPM1	16	Body	N_Shore	67.78	4.22	71.36	4.66	-3.58	1.32E-03	2.11E-02
cg25889581	LDB2;LDB2	4	Body;Body	Open sea	60.42	4.66	64.00	3.94	-3.58	6.60E-04	1.25E-02
cg07946633	FLJ42875;PRDM16;FLJ42875;PRDM16	1	Body;TSS1500;Body;TSS1500	Island	31.71	3.68	35.29	5.01	-3.58	7.85E-04	1.43E-02
cg16444001		17	Intergenic	S_Shelf	75.12	5.54	78.70	4.98	-3.58	4.46E-03	4.91E-02
cg19026233	UNC80;UNC80	2	Body;Body	Open sea	60.29	5.59	63.87	5.78	-3.58	4.19E-03	4.70E-02
cg03732506	SLC16A5	17	5'UTR	S_Shelf	72.86	4.33	76.44	3.99	-3.58	9.03E-04	1.59E-02
cg19005390	COL4A1	13	Body	Open sea	62.33	4.91	65.91	4.51	-3.58	2.26E-04	5.45E-03
cg07099186	PTPRG	3	Body	Open sea	73.65	6.09	77.23	5.98	-3.58	1.76E-03	2.59E-02
cg14132103	TMEM109	11	TSS1500	N_Shore	45.55	4.19	49.12	4.10	-3.58	2.22E-04	5.37E-03
cg10315420	ATF1	12	3'UTR	Open sea	85.95	5.50	89.53	4.26	-3.58	1.96E-04	4.86E-03
cg08753560		7	Intergenic	Open sea	59.66	4.75	63.24	5.43	-3.58	2.49E-03	3.29E-02
cg16097191		1	Intergenic	S_Shelf	43.69	4.72	47.27	4.84	-3.58	7.31E-04	1.35E-02
cg03557916		13	Intergenic	Open sea	89.13	7.21	92.71	4.26	-3.58	3.45E-03	4.13E-02
cg12153593		19	Intergenic	Island	12.24	3.12	15.82	7.28	-3.58	1.92E-03	2.74E-02
cg24035164		10	Intergenic	N_Shore	22.22	4.61	25.80	5.33	-3.58	1.41E-04	3.70E-03
cg18959554	SPATS1	6	TSS200	Open sea	17.01	5.43	20.59	6.57	-3.58	4.41E-05	1.39E-03
cg09143195	CNTN1;CNTN1	12	TSS1500;TSS1500	N_Shore	26.56	5.45	30.14	6.16	-3.58	4.08E-03	4.63E-02
cg05247821		13	Intergenic	S_Shore	53.41	8.43	56.99	7.49	-3.58	4.27E-03	4.77E-02
cg02827572	C6orf106;C6orf106	6	Body;Body	Open sea	63.06	4.46	66.64	4.73	-3.58	7.64E-04	1.40E-02
cg07294785	HLA-DOA	6	Body	S_Shore	78.12	4.66	81.70	5.31	-3.58	5.88E-04	1.15E-02
cg19753641	CD37;CD37	19	Body;5'UTR	N_Shore	78.87	5.16	82.45	3.13	-3.58	1.66E-03	2.48E-02
cg18890215	MCAM	11	Body	S_Shore	74.37	4.39	77.94	3.05	-3.58	2.96E-04	6.72E-03
cg22909085	ACSL3;ACSL3	2	5'UTR;5'UTR	Open sea	73.77	4.68	77.35	4.15	-3.58	1.05E-03	1.78E-02
cg01208566	PELI2	14	Body	S_Shore	56.20	4.66	59.78	4.20	-3.58	4.91E-04	9.98E-03
cg05433642	MBNL2;MBNL2	13	TSS1500;TSS1500	Open sea	62.70	5.35	66.28	4.78	-3.58	1.74E-04	4.42E-03
cg26353844		1	Intergenic	Open sea	64.33	3.77	67.91	3.47	-3.58	3.18E-05	1.05E-03
cg20153240	PRKCZ;PRKCZ	1	Body;5'UTR	Open sea	84.48	3.79	88.06	2.81	-3.58	5.08E-05	1.58E-03
cg26958124	OPA3	19	Body	Island	22.06	5.63	25.63	5.23	-3.58	6.84E-04	1.29E-02
cg00642042		15	Intergenic	Open sea	77.26	3.85	80.84	3.39	-3.57	1.83E-05	6.45E-04
cg24466377	SIX5	19	3'UTR	N_Shore	35.13	4.02	38.70	5.57	-3.57	2.51E-03	3.31E-02
cg21441256	MRPL42;MRPL42;MRPL42	12	3'UTR;3'UTR;3'UTR	Open sea	67.42	4.22	71.00	3.84	-3.57	1.84E-04	4.63E-03
cg05765254	RPTN	1	TSS1500	Open sea	62.13	6.15	65.70	5.68	-3.57	4.50E-03	4.94E-02
cg14602697	RAD9A	11	Body	N_Shelf	75.06	3.95	78.63	3.82	-3.57	3.41E-05	1.11E-03
cg20684180	DLEC1;DLEC1	3	TSS200;TSS200	Island	26.35	5.73	29.93	5.99	-3.57	5.05E-04	1.02E-02
cg11548411		10	Intergenic	S_Shore	19.64	3.51	23.21	3.67	-3.57	1.32E-05	4.85E-04
cg23207305		11	Intergenic	Open sea	81.05	3.89	84.62	3.45	-3.57	1.19E-05	4.39E-04
cg02965157	TRIT1	1	TSS1500	S_Shore	53.22	4.83	56.79	4.69	-3.57	1.35E-03	2.14E-02
cg04241863	NAT1;NAT1;NAT1;NAT1;NAT1;NAT1;N	8	TSS1500;TSS1500;TSS1500;TSS1500;TSS15	Open sea	26.10	3.84	29.67	4.00	-3.57	3.88E-04	8.32E-03
cg03530006		13	Intergenic	N_Shelf	70.29	5.32	73.86	4.09	-3.57	2.25E-04	5.43E-03
cg12100463	AKT1;AKT1;AKT1;AKT1	14	5'UTR;1stExon;5'UTR;5'UTR	N_Shore	59.45	3.15	63.02	4.11	-3.57	2.53E-04	5.96E-03
cg14797004	CYTH3	7	Body	Open sea	75.48	3.79	79.05	3.64	-3.57	7.53E-07	3.44E-05
cg19811863	HLA-DOB	6	TSS1500	Open sea	73.58	4.27	77.15	3.85	-3.57	1.17E-05	4.33E-04
cg15802555	MPP2;MPP2	17	5'UTR;1stExon	Island	25.13	4.50	28.70	4.04	-3.57	7.56E-05	2.21E-03
cg03895221		1	Intergenic	Open sea	50.34	4.94	53.91	4.24	-3.57	7.29E-04	1.35E-02
cg04742334	LOC339568	20	Body	Open sea	47.86	5.33	51.43	5.75	-3.57	7.28E-04	1.35E-02
cg14094347	QSOX2	9	Body	S_Shelf	70.02	4.13	73.59	3.26	-3.57	1.80E-04	4.55E-03
cg05486260	FAM135B	8	Body	Open sea	55.75	5.54	59.32	5.64	-3.57	2.69E-03	3.48E-02
cg18760502	NXN	17	Body	Open sea	77.01	4.47	80.58	4.10	-3.57	7.61E-06	2.94E-04
cg00146676		4	Intergenic	N_Shore	20.30	4.01	23.87	4.02	-3.57	1.03E-05	3.85E-04

cg13951190	DENND1A	9	Body	S_Shore	59.74	4.89	63.31	3.66	-3.57	1.84E-03	2.67E-02
cg16212145	GZMB	14	TSS1500	Open sea	63.15	6.00	66.72	6.02	-3.57	4.17E-03	4.69E-02
cg03029752	NLRX1;NLRX1	11	TSS1500;TSS1500	N_Shore	68.05	4.24	71.62	4.39	-3.57	1.54E-04	3.99E-03
cg13551227	KCNIP2;KCNIP2;KCNIP2;KCNIP2;KCNIP	10	TSS1500;TSS1500;TSS1500;TSS1500;TSS15	S_Shore	18.10	2.94	21.66	4.92	-3.57	1.21E-04	3.27E-03
cg15996342		3	Intergenic	Island	31.28	4.69	34.85	4.83	-3.57	3.67E-06	1.50E-04
cg20890145	IFNG	12	TSS1500	Open sea	79.86	4.84	83.43	5.18	-3.57	6.75E-04	1.27E-02
cg10724867	HOXA10	7	Body	N_Shore	44.06	3.77	47.63	4.40	-3.57	7.04E-04	1.32E-02
cg16317295		14	Intergenic	Open sea	67.10	4.25	70.67	4.21	-3.57	8.32E-04	1.49E-02
cg11342645	RBM28;RBM28	7	Body;Body	N_Shelf	74.89	4.37	78.46	3.29	-3.57	7.44E-05	2.18E-03
cg19509778	FOXI2	10	3'UTR	Island	17.12	3.65	20.69	3.86	-3.57	2.76E-04	6.38E-03
cg25099065	ANXA4	2	5'UTR	Open sea	72.41	5.62	75.98	4.12	-3.57	1.01E-03	1.72E-02
cg05584597		3	Intergenic	S_Shore	41.07	4.50	44.64	5.39	-3.57	2.79E-04	6.43E-03
cg11996741	CCDC157	22	Body	Open sea	69.41	4.46	72.98	4.52	-3.57	1.11E-04	3.05E-03
cg06230247	MPST;MPST;MPST;MPST	22	Body;Body;Body;Body	Island	45.21	4.59	48.77	4.76	-3.57	4.00E-03	4.56E-02
cg20763327	MYO10	5	Body	Open sea	43.50	4.43	47.07	5.00	-3.57	3.22E-03	3.93E-02
cg05058758	KYNU	2	Body	Open sea	49.61	3.88	53.17	4.22	-3.57	1.43E-05	5.20E-04
cg02542103		6	Intergenic	Open sea	70.08	5.08	73.65	5.00	-3.57	7.03E-04	1.32E-02
cg26858540	ZNF787	19	Body	S_Shelf	76.86	5.50	80.43	5.22	-3.57	1.14E-03	1.89E-02
cg24501831	ZCCHC24	10	Body	Open sea	53.45	3.63	57.02	4.15	-3.57	3.32E-05	1.09E-03
cg12086936	PAX6;PAX6;PAX6	11	TSS200;5'UTR;TSS200	Island	18.43	3.62	22.00	4.68	-3.57	1.83E-06	7.88E-05
cg04962005	CAPN1	11	3'UTR	Open sea	82.32	4.98	85.88	3.14	-3.57	1.28E-03	2.06E-02
cg03058038		18	Intergenic	Island	61.05	3.98	64.62	5.35	-3.57	5.57E-04	1.10E-02
cg19100996	FBRSL1	12	Body	N_Shore	68.95	3.49	72.52	4.35	-3.57	1.15E-05	4.28E-04
cg20282158	C8orf48	8	1stExon	Open sea	74.29	5.39	77.86	3.91	-3.56	1.10E-03	1.83E-02
cg20777829	MED13L	12	Body	Open sea	62.55	5.42	66.12	4.85	-3.56	9.48E-04	1.65E-02
cg10503359	TRPC1	3	Body	Open sea	76.77	4.14	80.33	4.36	-3.56	1.35E-04	3.57E-03
cg02726018	SEMA5B	3	5'UTR	Open sea	54.12	4.72	57.69	5.87	-3.56	4.08E-03	4.62E-02
cg23258881	PLEKHO1	1	Body	Island	19.33	3.96	22.89	5.01	-3.56	2.10E-04	5.14E-03
cg15852787	FRMD6	14	5'UTR	Open sea	76.77	6.75	80.34	5.42	-3.56	3.43E-03	4.11E-02
cg19100779	ASB4;ASB4	7	TSS200;TSS200	Open sea	84.30	5.42	87.87	4.28	-3.56	2.34E-03	3.16E-02
cg16399136		1	Intergenic	Island	31.68	7.38	35.24	7.31	-3.56	3.36E-03	4.05E-02
cg22239963		4	Intergenic	N_Shore	58.44	5.17	62.00	4.95	-3.56	4.42E-04	9.20E-03
cg08069287		11	Intergenic	Open sea	62.37	5.15	65.94	5.15	-3.56	4.09E-04	8.67E-03
cg04311403	EDARADD;EDARADD	1	Body;Body	S_Shore	60.11	5.05	63.67	5.47	-3.56	5.38E-04	1.07E-02
cg10038907	POLE2	14	Body	N_Shore	20.06	6.02	23.62	5.34	-3.56	4.22E-03	4.73E-02
cg07643267	LYPD3;LYPD3	19	1stExon;5'UTR	S_Shore	38.22	4.75	41.78	4.59	-3.56	1.45E-04	3.81E-03
cg25330232	FABP5	8	Body	S_Shore	16.21	2.98	19.77	4.91	-3.56	2.18E-06	9.28E-05
cg04075024	PPP1CA;PPP1CA;PPP1CA	11	Body;Body;Body	N_Shore	35.12	4.11	38.69	5.26	-3.56	3.23E-03	3.94E-02
cg07251857	ALPK3	15	1stExon	Island	48.02	4.14	51.58	5.15	-3.56	3.85E-03	4.44E-02
cg00204262	ECH1	19	TSS1500	S_Shore	25.47	4.55	29.03	5.31	-3.56	1.20E-03	1.96E-02
cg15039036	LY6G5C	6	Body	N_Shelf	71.54	3.85	75.10	4.08	-3.56	1.65E-04	4.23E-03
cg02909866	CNNM1	10	3'UTR	Open sea	13.35	2.44	16.91	3.91	-3.56	1.24E-06	5.49E-05
cg05133323	MED13L	12	Body	Open sea	75.93	3.98	79.50	4.56	-3.56	2.00E-04	4.95E-03
cg24608717	CELSR3	3	Body	S_Shore	66.40	3.96	69.96	3.87	-3.56	1.92E-04	4.79E-03
cg04013650		8	Intergenic	Open sea	53.15	4.64	56.71	5.37	-3.56	2.99E-03	3.73E-02
cg02166473	C14orf1	14	3'UTR	Open sea	78.69	4.24	82.25	3.65	-3.56	1.32E-05	4.83E-04
cg02408252		11	Intergenic	Island	41.23	3.68	44.79	4.98	-3.56	9.82E-04	1.69E-02
cg24677302	JDP2;JDP2;JDP2;JDP2	14	Body;Body;Body;Body	Open sea	41.17	4.04	44.73	4.42	-3.56	1.91E-04	4.77E-03
cg20070618	KIF13A;KIF13A;KIF13A;KIF13A	6	Body;Body;Body;Body	Open sea	71.96	4.17	75.52	4.60	-3.56	2.09E-05	7.26E-04
cg03906073	TERF1;TERF1	8	3'UTR;3'UTR	Open sea	48.81	3.44	52.37	3.06	-3.56	1.15E-06	5.14E-05
cg12288982	PHRF1	11	Body	S_Shore	85.03	3.75	88.59	2.12	-3.56	2.01E-06	8.62E-05
cg15841063	FOXD3	1	1stExon	Island	18.07	3.94	21.62	4.51	-3.56	1.10E-05	4.11E-04
cg09902641	PLXNA4;PLXNA4;PLXNA4	7	5'UTR;5'UTR;5'UTR	Open sea	81.36	5.11	84.91	4.94	-3.56	7.82E-04	1.42E-02

cg09026600	SETD4	21	TSS200	S_Shelf	79.67	4.69	83.23	3.68	-3.56	1.16E-04	3.16E-03
cg23356451		13	Intergenic	Open sea	74.55	4.74	78.11	5.89	-3.56	3.19E-03	3.91E-02
cg12713583	ARID3A	19	Body	Island	20.97	4.06	24.52	4.09	-3.56	9.86E-05	2.77E-03
cg07636338	FBXO46	19	3'UTR	N_Shore	75.75	4.67	79.31	3.92	-3.56	1.15E-03	1.90E-02
cg09381003	SHKBP1;SPTBN4	19	TSS1500;3'UTR	N_Shore	31.97	4.04	35.52	4.22	-3.56	8.29E-05	2.39E-03
cg15907278	STT3B	3	Body	Open sea	74.41	5.27	77.97	5.40	-3.56	9.77E-04	1.68E-02
cg09490007	TFEB;TFEB	6	TSS200;TSS1500	S_Shore	65.77	3.94	69.33	4.70	-3.56	1.52E-04	3.96E-03
cg00122944	PFKFB3	10	Body	Open sea	83.82	5.58	87.38	3.38	-3.56	2.12E-03	2.94E-02
cg02149880	MREG	2	TSS200	S_Shore	21.91	5.50	25.47	7.99	-3.56	2.79E-03	3.56E-02
cg19917412	NAALADL1	11	Body	Island	49.86	5.62	53.42	5.39	-3.56	2.02E-03	2.84E-02
cg08792812	ANXA4	2	Body	Open sea	37.30	4.25	40.86	4.97	-3.56	4.57E-03	5.00E-02
cg19871722		10	Intergenic	Open sea	74.76	3.85	78.32	4.15	-3.56	2.57E-04	6.04E-03
cg18412185		1	Intergenic	Open sea	17.89	3.66	21.45	4.52	-3.56	9.58E-04	1.66E-02
cg08178072		2	Intergenic	N_Shore	35.94	4.54	39.49	5.11	-3.56	3.31E-03	4.01E-02
cg13138954		15	Intergenic	Island	41.56	5.11	45.12	4.92	-3.56	4.18E-03	4.70E-02
cg01592387	AFAP1;AFAP1;LOC84740	4	Body;Body;Body	N_Shore	78.90	4.44	82.46	3.66	-3.56	8.16E-05	2.36E-03
cg06741896	CCND1	11	3'UTR	N_Shore	75.31	3.37	78.86	3.10	-3.56	9.05E-07	4.09E-05
cg16003857	ITGAE;GSG2;GSG2	17	Body;3'UTR;1stExon	S_Shelf	62.45	5.63	66.00	3.71	-3.56	3.21E-03	3.92E-02
cg13764778	CHAD;ACSF2	17	TSS1500;Body	Island	42.68	4.58	46.24	4.99	-3.56	3.20E-04	7.17E-03
cg04583529	TMEM161A	19	3'UTR	N_Shore	80.19	3.89	83.75	3.89	-3.56	6.49E-05	1.95E-03
cg16464735	EXOC5	14	Body	Open sea	86.20	5.57	89.75	4.00	-3.56	6.83E-04	1.29E-02
cg10996039		7	Intergenic	N_Shore	47.75	5.03	51.30	4.36	-3.56	2.16E-03	2.98E-02
cg26354191	WDR8	1	3'UTR	Open sea	61.79	4.70	65.34	3.69	-3.56	4.98E-04	1.01E-02
cg08516181	ZNF451;ZNF451	6	Body;Body	Open sea	76.02	5.40	79.57	5.90	-3.56	2.00E-03	2.82E-02
cg14851471	SYT7	11	TSS1500	S_Shore	62.05	2.88	65.60	4.20	-3.56	6.01E-06	2.36E-04
cg08455781	KIAA0182;KIAA0182	16	5'UTR;Body	N_Shore	76.55	4.56	80.11	4.80	-3.56	2.81E-03	3.58E-02
cg13431205	RB1	13	Body	Island	44.72	4.03	48.28	6.34	-3.56	4.27E-03	4.77E-02
cg01982788	SMARCC2;SMARCC2;SMARCC2	12	3'UTR;3'UTR;3'UTR	Open sea	67.53	4.34	71.09	4.35	-3.56	5.40E-04	1.08E-02
cg24558378		1	Intergenic	Open sea	81.71	4.08	85.26	2.96	-3.55	2.42E-05	8.28E-04
cg14800014	GRAMD3;GRAMD3;GRAMD3;GRAMD	5	Body;5'UTR;TSS200;Body;Body	Open sea	90.75	4.64	94.31	1.54	-3.55	6.42E-07	2.96E-05
cg14099718	PAX5	9	Body	N_Shelf	45.14	4.40	48.69	4.97	-3.55	8.61E-05	2.47E-03
cg14609289	ENGASE	17	3'UTR	Open sea	47.05	2.99	50.60	3.87	-3.55	2.21E-05	7.64E-04
cg14151440	ZNF326	1	Body	Open sea	85.13	4.03	88.68	3.29	-3.55	1.12E-05	4.17E-04
cg03436478	SGCE;PEG10;SGCE;PEG10;SGCE	7	TSS1500;5'UTR;TSS1500;5'UTR;TSS1500	S_Shore	43.54	3.75	47.09	4.92	-3.55	2.04E-04	5.02E-03
cg24191816	RGL2;RGL2	6	Body;Body	N_Shore	81.23	3.68	84.78	3.36	-3.55	6.40E-06	2.50E-04
cg12960696	SH3TC2	5	Body	Open sea	28.60	4.24	32.16	4.45	-3.55	3.35E-04	7.43E-03
cg07760794		17	Intergenic	S_Shelf	27.06	3.87	30.62	4.89	-3.55	1.19E-03	1.95E-02
cg14646548	ZNF484;ZNF484	9	TSS1500;TSS1500	Open sea	60.18	3.73	63.74	4.34	-3.55	7.14E-04	1.33E-02
cg26877905		6	Intergenic	Open sea	73.55	3.75	77.11	3.04	-3.55	6.95E-06	2.70E-04
cg18378811	ARSJ;ARSJ	4	1stExon;5'UTR	N_Shore	52.55	7.87	56.10	9.08	-3.55	4.40E-03	4.87E-02
cg17240157	PBX1	1	3'UTR	Open sea	85.68	3.44	89.23	2.58	-3.55	4.74E-09	2.57E-07
cg01541178		13	Intergenic	N_Shore	28.60	4.08	32.15	4.90	-3.55	1.64E-04	4.21E-03
cg15255390	HOXB3	17	5'UTR	Island	31.58	3.06	35.13	3.71	-3.55	5.20E-05	1.61E-03
cg27071797		15	Intergenic	Island	80.64	4.22	84.19	3.38	-3.55	1.68E-04	4.29E-03
cg06661450	LHX9;LHX9	1	Body;Body	N_Shore	27.00	4.17	30.55	3.94	-3.55	2.98E-05	9.91E-04
cg00774375	CTXN1	19	3'UTR	Island	52.06	4.59	55.61	3.54	-3.55	2.51E-04	5.93E-03
cg16148270	DIRAS3	1	TSS200	Island	56.45	3.08	60.00	3.85	-3.55	8.92E-06	3.39E-04
cg18958591		12	Intergenic	N_Shelf	65.64	3.63	69.19	4.02	-3.55	9.91E-06	3.74E-04
cg15604046	TRHDE	12	Body	Open sea	64.82	5.49	68.37	4.71	-3.55	1.48E-03	2.28E-02
cg03760951	INS-IGF2;IGF2AS;IGF2;IGF2;IGF2AS;IG	11	Body;TSS1500;Body;5'UTR;TSS1500;TSS15	Island	58.60	4.55	62.15	3.67	-3.55	5.45E-04	1.08E-02
cg18315312	DCAF6;DCAF6	1	Body;Body	S_Shore	83.68	6.31	87.23	5.50	-3.55	9.97E-04	1.71E-02
cg09408023		1	Intergenic	S_Shelf	54.67	3.73	58.22	2.85	-3.55	4.75E-05	1.49E-03
cg16365445	ITGB1;ITGB1	10	5'UTR;5'UTR	Open sea	66.54	5.08	70.09	3.52	-3.55	6.70E-04	1.27E-02

cg06368429	KPNA7	7	Body	Open sea	68.53	3.67	72.08	3.65	-3.55	6.00E-06	2.36E-04
cg13840308	FAM107B	10	Body	Open sea	84.35	3.34	87.90	3.04	-3.55	4.80E-08	2.45E-06
cg00606396	CDH18;CDH18	5	Body;Body	Open sea	79.85	5.55	83.40	4.34	-3.55	8.07E-04	1.46E-02
cg23326689	STMN2	8	Body	N_Shore	21.52	5.03	25.07	4.72	-3.55	1.32E-03	2.11E-02
cg06470727	OTOF	2	Body	N_Shelf	45.50	5.68	49.05	5.64	-3.55	3.26E-04	7.27E-03
cg23255283	LOC284276	18	TSS200	Open sea	22.64	3.87	26.19	3.72	-3.55	1.38E-04	3.64E-03
cg03915648		12	Intergenic	Island	20.87	4.48	24.42	5.00	-3.55	2.09E-03	2.92E-02
cg05264881	KIAA1026;KIAA1026;KIAA1026;KIAA1(	1	Body;Body;Body;Body;Body	Island	52.87	5.80	56.42	4.74	-3.55	1.04E-03	1.76E-02
cg14375543		6	Intergenic	Open sea	14.35	6.02	17.90	7.70	-3.55	1.90E-03	2.73E-02
cg08455916	C2orf73	2	Body	S_Shelf	75.75	5.03	79.30	5.23	-3.55	2.19E-03	3.01E-02
cg11137160		12	Intergenic	S_Shelf	41.05	4.88	44.60	4.86	-3.55	1.73E-04	4.40E-03
cg16352830	PLXNA2	1	Body	Open sea	52.18	5.45	55.73	4.85	-3.55	2.83E-03	3.60E-02
cg09141965		7	Intergenic	Island	22.85	4.23	26.40	3.64	-3.55	2.07E-05	7.20E-04
cg07986413	STK38	6	Body	Open sea	64.25	5.31	67.79	4.21	-3.55	8.14E-04	1.47E-02
cg24708097		7	Intergenic	Open sea	84.86	3.81	88.41	2.62	-3.55	1.10E-05	4.12E-04
cg20432384		1	Intergenic	Open sea	74.12	4.63	77.66	5.56	-3.55	2.87E-03	3.63E-02
cg07991621	SH3BP2;SH3BP2;SH3BP2;SH3BP2	4	Body;Body;5'UTR;TSS200	S_Shore	50.06	6.07	53.61	6.41	-3.55	9.01E-04	1.59E-02
cg19535004		11	Intergenic	S_Shelf	74.87	4.22	78.42	3.56	-3.55	1.56E-05	5.60E-04
cg12474881	MYH9	22	Body	Open sea	31.34	3.81	34.89	4.74	-3.55	9.91E-04	1.70E-02
cg04730685		13	Intergenic	S_Shore	22.33	4.34	25.88	4.36	-3.55	5.42E-04	1.08E-02
cg00864530		14	Intergenic	Open sea	76.93	4.63	80.47	4.40	-3.55	1.24E-04	3.33E-03
cg15159104	MAP1A;MAP1A	15	5'UTR;1stExon	Open sea	42.17	4.02	45.72	4.93	-3.55	1.42E-03	2.22E-02
cg05773108		16	Intergenic	S_Shore	75.89	3.99	79.44	4.00	-3.55	1.45E-05	5.25E-04
cg18795433	TSC2;TSC2;TSC2	16	Body;Body;Body	Island	80.30	4.28	83.84	3.00	-3.55	4.38E-05	1.39E-03
cg25001102		1	Intergenic	Island	66.36	5.89	69.91	5.02	-3.55	2.88E-03	3.64E-02
cg13035910		6	Intergenic	N_Shore	20.53	3.09	24.07	3.98	-3.55	5.66E-06	2.24E-04
cg12855166	MYO1D	17	Body	Island	8.86	4.75	12.40	6.66	-3.55	2.71E-03	3.50E-02
cg27017357	KDM4B	19	5'UTR	N_Shelf	68.30	4.49	71.85	4.84	-3.55	1.49E-04	3.90E-03
cg18049414		11	Intergenic	Open sea	73.11	4.63	76.66	4.12	-3.55	1.34E-04	3.57E-03
cg05500240	SPIRE2	16	Body	Open sea	68.16	5.41	71.70	4.73	-3.55	3.17E-03	3.89E-02
cg08114852	BTBD10	11	Body	Open sea	28.78	6.05	32.33	4.76	-3.55	1.73E-03	2.55E-02
cg04943586	RFX4;RFX4	12	TSS1500;Body	Open sea	50.75	5.85	54.29	5.79	-3.55	2.04E-03	2.86E-02
cg23014747	TM2D1	1	TSS1500	S_Shore	68.46	4.35	72.01	5.41	-3.55	1.61E-03	2.42E-02
cg18960629		17	Intergenic	Island	56.50	6.80	60.04	7.58	-3.54	2.48E-03	3.28E-02
cg19731541	NRP2;NRP2;NRP2;NRP2;NRP2;NRP2	2	Body;Body;Body;Body;Body;Body	S_Shelf	31.62	3.99	35.16	4.50	-3.54	2.52E-03	3.32E-02
cg08584377	FSCN1	7	TSS200	N_Shore	10.89	2.87	14.44	4.56	-3.54	4.45E-08	2.28E-06
cg26331247	LOC284805	20	Body	Open sea	68.32	5.51	71.87	4.74	-3.54	1.72E-04	4.38E-03
cg01189604	INPP5A	10	Body	Open sea	85.53	3.88	89.07	1.78	-3.54	1.76E-06	7.60E-05
cg10755058	ENTPD3;ENTPD3	3	1stExon;5'UTR	Island	12.39	5.75	15.93	6.20	-3.54	4.03E-03	4.59E-02
cg22378678	SERPINI2	3	Body	Open sea	64.49	5.70	68.04	4.89	-3.54	4.72E-04	9.69E-03
cg18115040	HOXD10	2	TSS200	Island	14.88	4.29	18.42	4.99	-3.54	7.00E-04	1.31E-02
cg00492741	CBFA2T3	16	Body	Open sea	64.13	4.82	67.67	5.64	-3.54	2.36E-03	3.17E-02
cg07893474	C3orf43	3	TSS200	Open sea	70.65	5.89	74.19	4.37	-3.54	5.21E-04	1.05E-02
cg17107540	NODAL	10	Body	N_Shelf	46.24	6.02	49.78	5.90	-3.54	2.57E-03	3.36E-02
cg08527448	RBM22	5	3'UTR	Open sea	72.87	5.57	76.41	3.91	-3.54	6.12E-04	1.18E-02
cg03723247		12	Intergenic	N_Shore	27.36	7.10	30.90	8.36	-3.54	2.33E-03	3.14E-02
cg22336162		7	Intergenic	Island	54.29	4.98	57.83	5.48	-3.54	2.19E-03	3.01E-02
cg20941894	LOC285696	5	Body	Open sea	65.68	4.36	69.22	5.02	-3.54	9.54E-05	2.69E-03
cg12684999		2	Intergenic	Open sea	74.21	4.56	77.75	4.88	-3.54	9.44E-04	1.64E-02
cg17140299	TXNL1;TXNL1	18	Body;3'UTR	Open sea	74.05	6.10	77.60	5.95	-3.54	4.03E-03	4.59E-02
cg19790640	BAALC;BAALC;C8orf56	8	Body;Body;TSS1500	S_Shore	29.47	4.78	33.01	5.68	-3.54	1.08E-03	1.81E-02
cg26845138	STK10	5	Body	Open sea	58.25	3.30	61.79	3.96	-3.54	1.12E-04	3.08E-03
cg04114121	NOC4L	12	Body	N_Shore	87.53	4.67	91.07	3.12	-3.54	1.53E-04	3.97E-03

cg16137285	CDC40	6	3'UTR	Open sea	84.73	4.96	88.27	4.64	-3.54	3.36E-04	7.44E-03
cg04353586	CAMTA1	1	Body	Open sea	73.17	4.33	76.72	4.35	-3.54	1.40E-03	2.19E-02
cg13242090	PDLIM1	10	Body	Open sea	75.03	6.28	78.57	6.25	-3.54	1.02E-03	1.74E-02
cg09231862	FOXG1	14	TSS1500	Island	19.75	4.08	23.30	4.66	-3.54	6.80E-04	1.28E-02
cg20339650	INS-IGF2;IGF2AS;IGF2;IGF2;IGF2AS;IG	11	Body;TSS1500;Body;5'UTR;TSS1500;TSS15	Island	51.70	4.29	55.24	4.52	-3.54	4.56E-04	9.44E-03
cg12349422	TNNC1	3	3'UTR	Open sea	61.45	5.39	64.99	4.86	-3.54	4.67E-04	9.61E-03
cg08832624		7	Intergenic	Island	29.04	4.86	32.58	5.00	-3.54	8.31E-04	1.49E-02
cg14525247	MIR219-2	9	Body	Island	88.63	5.07	92.18	2.94	-3.54	3.53E-04	7.73E-03
cg08463788	LHX9;LHX9	1	Body;Body	S_Shore	34.36	4.17	37.90	3.84	-3.54	1.32E-04	3.52E-03
cg09685601	SYCP1	1	TSS1500	N_Shore	76.95	3.80	80.49	3.46	-3.54	1.30E-05	4.76E-04
cg00163199	ALKBH5	17	Body	S_Shore	30.09	5.69	33.63	5.72	-3.54	6.75E-04	1.27E-02
cg10358623		11	Intergenic	N_Shore	41.67	4.34	45.21	4.37	-3.54	2.41E-03	3.22E-02
cg21703606	HOXC4;HOXC5;HOXC5	12	5'UTR;Body;Body	Island	24.58	3.81	28.12	4.81	-3.54	2.73E-05	9.21E-04
cg24837277		3	Intergenic	Open sea	63.79	4.91	67.33	4.65	-3.54	1.12E-03	1.86E-02
cg01134637	CUEDC1	17	Body	N_Shore	80.93	5.61	84.47	6.75	-3.54	2.81E-03	3.58E-02
cg20981919	RARB;RARB	3	Body;Body	Open sea	75.86	4.69	79.40	5.58	-3.54	2.64E-05	8.93E-04
cg01743846	PSMB7	9	Body	N_Shore	27.15	3.79	30.69	5.12	-3.54	2.42E-03	3.23E-02
cg10531355	SERINC5	5	Body	Open sea	25.44	3.18	28.98	4.65	-3.54	2.13E-04	5.20E-03
cg10069644		14	Intergenic	Open sea	63.27	5.39	66.81	5.80	-3.54	1.66E-03	2.47E-02
cg20670946		12	Intergenic	Open sea	57.94	5.13	61.48	4.45	-3.54	5.53E-04	1.09E-02
cg24197059		22	Intergenic	S_Shore	62.67	5.33	66.21	6.53	-3.54	1.70E-03	2.51E-02
cg21062760	ZBTB32	19	Body	N_Shore	52.84	4.21	56.37	4.86	-3.54	3.63E-04	7.90E-03
cg21577193		7	Intergenic	Open sea	52.85	4.24	56.39	3.71	-3.54	1.17E-04	3.18E-03
cg09413450	CLCNKB	1	TSS200	Open sea	65.21	4.15	68.75	3.60	-3.54	1.09E-04	3.02E-03
cg17076485		11	Intergenic	S_Shelf	63.30	5.65	66.84	5.87	-3.54	1.24E-03	2.01E-02
cg13391313		17	Intergenic	N_Shore	51.64	4.68	55.18	4.10	-3.54	2.75E-04	6.37E-03
cg14040131	GPSM1;GPSM1	9	Body;TSS200	Open sea	78.96	5.44	82.50	4.20	-3.54	6.56E-04	1.25E-02
cg11852212	GNA11	19	Body	N_Shelf	86.75	3.69	90.29	2.34	-3.54	1.68E-06	7.28E-05
cg07915206	AVEN;CHRM5	15	Body;TSS1500	Open sea	45.85	4.48	49.39	4.68	-3.54	1.71E-04	4.35E-03
cg19841369	SYNE2;SYNE2	14	Body;Body	Open sea	56.61	5.65	60.15	5.74	-3.54	3.10E-03	3.83E-02
cg16075139	CD81	11	Body	Island	46.68	3.71	50.22	4.62	-3.54	1.58E-03	2.39E-02
cg15147833	OR1C1	1	1stExon	Open sea	44.64	4.65	48.18	5.45	-3.54	2.90E-03	3.66E-02
cg23500444	UBAC2;UBAC2;UBAC2	13	Body;Body;Body	Open sea	82.87	3.71	86.41	3.09	-3.54	5.34E-06	2.12E-04
cg07162320	RUSC1;RUSC1;RUSC1;C1orf104;RUSC1	1	Body;Body;Body;TSS1500;Body	Island	45.94	5.05	49.48	5.15	-3.54	9.18E-04	1.61E-02
cg11749264	AKD1	6	Body	Open sea	79.60	6.04	83.14	5.04	-3.54	2.61E-03	3.40E-02
cg24619378	PCDHGA4;PCDHGC3;PCDHGA12;PCDH	5	Body;Body;Body;Body;Body;Body;Body;B	Island	51.24	3.92	54.78	3.61	-3.54	1.52E-04	3.95E-03
cg02916332	HOXA5	7	TSS1500	Island	26.89	3.77	30.43	4.80	-3.54	1.19E-03	1.94E-02
cg27282564	RDH13	19	5'UTR	S_Shelf	26.85	3.51	30.39	4.23	-3.54	4.40E-04	9.18E-03
cg07791011		3	Intergenic	N_Shore	25.76	4.04	29.30	4.55	-3.54	4.05E-04	8.60E-03
cg04767484	ISL2	15	TSS200	Island	24.75	4.18	28.28	4.30	-3.54	3.11E-05	1.03E-03
cg24485951	PLEKHG2	19	3'UTR	Open sea	74.87	4.66	78.40	3.63	-3.54	1.02E-04	2.85E-03
cg24287110	KLF6;KLF6;KLF6;KLF6	10	Body;Body;Body;Body	S_Shore	17.97	8.34	21.50	10.34	-3.54	2.51E-03	3.31E-02
cg12371991		15	Intergenic	Open sea	57.49	4.33	61.03	4.13	-3.53	3.28E-04	7.31E-03
cg07613994		16	Intergenic	S_Shore	69.56	3.78	73.09	4.17	-3.53	7.88E-05	2.29E-03
cg05203776	IGF2AS;INS-IGF2;IGF2;IGF2;IGF2AS	11	Body;Body;5'UTR;TSS1500;Body	S_Shore	64.23	3.99	67.77	4.58	-3.53	6.34E-05	1.91E-03
cg00773810	FAM133B;FAM133B	7	Body;5'UTR	N_Shore	72.82	4.95	76.36	4.77	-3.53	1.59E-03	2.40E-02
cg05774978	PDIA3P	1	Body	Island	36.10	6.61	39.63	5.87	-3.53	4.36E-03	4.84E-02
cg04737350	PPP1R12A;PPP1R12A;PPP1R12A	12	Body;Body;Body	Open sea	82.57	4.50	86.10	5.12	-3.53	2.01E-04	4.96E-03
cg02177951		11	Intergenic	Island	22.11	3.52	25.64	5.54	-3.53	3.81E-03	4.42E-02
cg18479068	MREG	2	TSS200	Island	10.89	4.65	14.43	7.28	-3.53	9.12E-04	1.60E-02
cg08834459	C3orf26;MIR548G	3	Body;Body	S_Shelf	48.58	3.14	52.11	3.69	-3.53	4.71E-05	1.48E-03
cg24158728	TMCO3	13	Body	N_Shelf	78.48	5.42	82.01	6.00	-3.53	6.01E-04	1.17E-02
cg20202413	PTH1R	3	5'UTR	N_Shore	22.75	4.61	26.28	5.38	-3.53	2.69E-03	3.47E-02

cg16092390	MYH9	22	Body	S_Shore	73.23	6.14	76.77	4.30	-3.53	4.37E-03	4.84E-02
cg07330145	STARD10	11	3'UTR	N_Shore	47.82	4.53	51.36	4.75	-3.53	3.24E-03	3.94E-02
cg10069809		2	Intergenic	N_Shore	39.40	3.89	42.93	4.70	-3.53	4.84E-04	9.87E-03
cg11575738	DLX1;DLX1	2	TSS200;TSS200	N_Shore	22.79	4.40	26.32	4.66	-3.53	1.75E-05	6.21E-04
cg16587174	HSPA1L	6	Body	S_Shelf	79.44	5.17	82.97	3.31	-3.53	6.10E-04	1.18E-02
cg09941208	PTCH2;PTCH2	1	Body;Body	N_Shore	75.05	4.91	78.58	4.36	-3.53	1.01E-03	1.72E-02
cg21185686	PCDHGA4;PCDHGA11;PCDHGA11;PCD	5	Body;Body;Body;Body;Body;Body;Body;1s	N_Shore	14.49	3.78	18.02	4.95	-3.53	5.70E-05	1.74E-03
cg03041696		14	Intergenic	S_Shore	16.96	3.47	20.49	3.23	-3.53	6.11E-06	2.40E-04
cg05977276	EHBP1;EHBP1;EHBP1;EHBP1	2	Body;Body;Body;Body	Open sea	80.78	5.63	84.31	4.69	-3.53	4.84E-04	9.88E-03
cg01834979	AGAP2;LOC100130776;AGAP2	12	3'UTR;TSS200;3'UTR	Island	34.66	4.61	38.19	4.02	-3.53	2.67E-03	3.46E-02
cg11134777	INTS1	7	Body	N_Shore	83.15	3.51	86.68	2.68	-3.53	2.39E-06	1.01E-04
cg03024537		8	Intergenic	N_Shore	31.10	4.03	34.63	4.10	-3.53	4.88E-04	9.93E-03
cg23640701	ACVRL1	12	5'UTR	S_Shore	77.78	4.24	81.31	3.35	-3.53	3.41E-04	7.52E-03
cg03843031	UBE2C;UBE2C;UBE2C;UBE2C;UBE2C;U	20	TSS1500;TSS1500;TSS1500;TSS1500;TSS15	Island	9.27	2.48	12.80	4.01	-3.53	5.82E-08	2.96E-06
cg02406092	PAF1	19	3'UTR	Open sea	13.16	1.84	16.69	3.19	-3.53	1.38E-08	7.32E-07
cg24505713		11	Intergenic	N_Shelf	61.86	5.11	65.39	3.58	-3.53	1.85E-04	4.65E-03
cg07969619		12	Intergenic	Island	11.71	4.42	15.24	5.38	-3.53	5.95E-06	2.35E-04
cg06720578	THSD4	15	Body	Open sea	75.26	6.00	78.79	4.56	-3.53	2.56E-03	3.36E-02
cg05222995		12	Intergenic	Island	39.23	5.41	42.76	5.21	-3.53	6.14E-04	1.19E-02
cg19222525	C20orf135	20	TSS1500	N_Shore	43.88	3.05	47.42	4.02	-3.53	7.55E-05	2.21E-03
cg20587945	SEMA3E	7	Body	Open sea	60.88	5.84	64.41	5.86	-3.53	2.37E-03	3.19E-02
cg06174704		1	Intergenic	S_Shelf	61.62	3.17	65.15	3.27	-3.53	3.65E-06	1.50E-04
cg15262317	FZD5	2	Body	Island	18.48	3.98	22.01	3.73	-3.53	3.72E-04	8.06E-03
cg06458527		9	Intergenic	Island	55.98	5.21	59.51	5.38	-3.53	3.72E-03	4.34E-02
cg07671011		8	Intergenic	Open sea	40.36	3.83	43.89	5.25	-3.53	2.47E-04	5.85E-03
cg25002069		17	Intergenic	Open sea	54.26	4.49	57.79	4.62	-3.53	1.70E-03	2.51E-02
cg01494994	SNTG2	2	Body	Open sea	72.24	4.82	75.77	4.72	-3.53	2.13E-04	5.20E-03
cg25499397	GPR62	3	1stExon	Island	27.83	6.72	31.35	7.31	-3.53	3.01E-03	3.75E-02
cg10785153		13	Intergenic	Open sea	70.68	5.68	74.21	4.84	-3.53	2.54E-04	5.98E-03
cg08766099	PCGF3	4	Body	N_Shelf	78.82	3.07	82.35	2.74	-3.53	1.86E-08	9.77E-07
cg20728156	IGFBP2	2	Body	S_Shore	29.02	4.15	32.55	5.14	-3.53	2.18E-03	3.01E-02
cg11182257	KIAA1598;KIAA1598	10	Body;Body	Open sea	51.73	5.58	55.26	6.36	-3.53	1.10E-03	1.84E-02
cg19080053	LYRM4;LYRM4	6	3'UTR;Body	N_Shelf	57.39	5.61	60.92	4.26	-3.53	7.00E-04	1.31E-02
cg03987752	PDZD8	10	Body	Open sea	71.54	5.07	75.07	4.17	-3.53	2.46E-03	3.27E-02
cg12178904	SLC45A3	1	Body	Island	80.07	4.49	83.60	4.28	-3.53	2.78E-04	6.42E-03
cg15014684	DLG4;DLG4	17	3'UTR;3'UTR	Open sea	75.18	3.94	78.70	4.56	-3.53	1.73E-04	4.41E-03
cg19935845	TNXB	6	5'UTR	Open sea	72.21	4.23	75.74	3.96	-3.53	1.51E-04	3.92E-03
cg22378065	HYMAI;PLAGL1;PLAGL1;PLAGL1;PLAG	6	Body;5'UTR;5'UTR;5'UTR;5'UTR;5'UTR;5'U	Island	47.86	3.68	51.39	3.59	-3.53	1.83E-04	4.60E-03
cg02592062	HOMER3;HOMER3;HOMER3;HOMER3	19	Body;Body;Body;Body;Body	S_Shore	44.56	3.67	48.09	4.64	-3.53	9.71E-04	1.67E-02
cg25265930	HLA-DMB	6	Body	Open sea	31.16	4.65	34.69	5.25	-3.53	1.80E-03	2.62E-02
cg20284982	IER3	6	TSS1500	S_Shore	53.03	4.20	56.56	4.14	-3.53	8.06E-04	1.46E-02
cg21924571	IQSEC1	3	Body	N_Shore	42.91	4.14	46.44	4.53	-3.53	3.58E-04	7.82E-03
cg13561879	UNC5D	8	TSS1500	Island	27.66	4.02	31.18	3.97	-3.53	1.45E-04	3.80E-03
cg20611334	KIAA1522	1	Body	S_Shore	42.40	4.39	45.93	5.32	-3.53	1.33E-03	2.11E-02
cg04915618	KALRN;KALRN	3	Body;Body	S_Shore	73.16	5.53	76.69	4.51	-3.53	7.04E-04	1.32E-02
cg13725899	NOTCH1	9	Body	S_Shore	75.28	4.31	78.81	3.51	-3.53	7.24E-06	2.81E-04
cg04834436	IRX6	16	Body	Island	13.77	3.71	17.30	5.40	-3.52	7.11E-04	1.33E-02
cg06970190		3	Intergenic	Island	26.77	2.90	30.29	4.78	-3.52	2.54E-05	8.65E-04
cg09296730	VARS2;VARS2;VARS2	6	Body;Body;Body	S_Shelf	70.80	4.11	74.33	3.55	-3.52	3.10E-05	1.03E-03
cg01963035	GPR98;GPR98	5	Body;Body	Open sea	80.77	7.65	84.30	4.94	-3.52	2.01E-03	2.84E-02
cg20577019	PLEKHO1	1	Body	Island	28.08	4.62	31.61	6.23	-3.52	6.97E-04	1.31E-02
cg07787240	SLC2A5;SLC2A5;SLC2A5	1	TSS1500;Body;TSS1500	N_Shore	51.39	4.23	54.92	4.81	-3.52	1.26E-03	2.03E-02
cg13934301		9	Intergenic	N_Shelf	71.67	3.50	75.19	3.67	-3.52	8.40E-05	2.42E-03

cg13656738	SMARCA4;SMARCA4;SMARCA4;SMAF	19	Body;Body;Body;Body;Body;Body;Body	Island	85.64	3.80	89.16	3.23	-3.52	1.59E-05	5.70E-04
cg00527440	THAP4;THAP4	2	Body;Body	Island	7.78	3.17	11.31	5.57	-3.52	1.65E-04	4.24E-03
cg12978232		12	Intergenic	Open sea	79.39	4.17	82.91	4.30	-3.52	2.79E-05	9.36E-04
cg18286285	LDB3;LDB3;LDB3;LDB3;LDB3;LDB3;LDI	10	TSS200;TSS200;1stExon;TSS200;5'UTR;TSS	Open sea	70.78	5.18	74.30	4.47	-3.52	2.67E-03	3.46E-02
cg05957730	MPHOSPH10	2	Body	S_Shelf	40.95	4.35	44.47	4.79	-3.52	1.70E-03	2.52E-02
cg24993400	SELO	22	Body	S_Shore	15.47	3.40	18.99	4.09	-3.52	1.66E-04	4.26E-03
cg08974492	C6orf127	6	TSS1500	S_Shelf	63.30	4.72	66.82	5.36	-3.52	3.05E-03	3.79E-02
cg10331884	MEGF6	1	Body	N_Shelf	79.40	4.46	82.92	3.61	-3.52	1.78E-04	4.50E-03
cg05122453	SH2D3C;SH2D3C	9	Body;Body	N_Shore	55.98	4.39	59.50	4.60	-3.52	5.10E-04	1.03E-02
cg25098478	SLC9A1	1	Body	Open sea	62.08	4.32	65.60	3.81	-3.52	5.04E-04	1.02E-02
cg04779161	DMPK;DMPK;DMPK	19	TSS1500;TSS1500;TSS1500	N_Shelf	63.39	4.40	66.92	3.67	-3.52	1.21E-04	3.27E-03
cg01409873	TOLLIP	11	Body	Island	83.36	4.62	86.88	3.77	-3.52	1.84E-03	2.66E-02
cg05992079	SIT1	9	TSS200	S_Shelf	50.41	5.01	53.93	5.10	-3.52	7.89E-04	1.43E-02
cg18837947		4	Intergenic	Open sea	64.41	6.15	67.94	6.21	-3.52	2.47E-03	3.28E-02
cg23212916	LOC100133545	11	Body	Open sea	31.37	2.97	34.90	3.08	-3.52	3.02E-06	1.25E-04
cg13878360		3	Intergenic	Open sea	78.05	4.19	81.58	3.74	-3.52	1.34E-05	4.91E-04
cg22861561	COL2A1;COL2A1	12	Body;Body	N_Shore	20.10	3.57	23.63	3.96	-3.52	1.24E-04	3.35E-03
cg10604168	RASL12;RASL12	15	5'UTR;1stExon	Island	46.92	4.99	50.44	6.06	-3.52	3.88E-04	8.32E-03
cg01099448	SPAG16;SPAG16	2	Body;3'UTR	Open sea	78.46	5.31	81.98	3.88	-3.52	2.30E-03	3.11E-02
cg03742286	LBX2	2	Body	Island	30.30	4.72	33.82	5.25	-3.52	1.75E-03	2.58E-02
cg13038025	JMJD7-PLA2G4B;JMJD7-PLA2G4B;PLA	15	Body;Body;TSS1500	Open sea	56.71	5.52	60.23	5.14	-3.52	2.17E-03	3.00E-02
cg00422474		8	Intergenic	Open sea	47.11	5.88	50.63	5.85	-3.52	5.98E-04	1.16E-02
cg09884451	ZFYVE28	4	Body	Island	59.07	5.62	62.59	5.16	-3.52	4.01E-04	8.53E-03
cg02605461	RALY;RALY	20	5'UTR;5'UTR	S_Shore	54.52	7.82	58.04	7.52	-3.52	1.59E-03	2.40E-02
cg17116500	RNF41;RNF41;RNF41	12	3'UTR;3'UTR;3'UTR	Open sea	84.03	3.98	87.55	2.77	-3.52	6.77E-05	2.02E-03
cg21450529	PREB	2	3'UTR	N_Shelf	77.31	3.19	80.83	2.55	-3.52	7.66E-07	3.50E-05
cg26065836	INTS7	1	Body	Open sea	77.11	5.26	80.63	4.53	-3.52	3.97E-03	4.54E-02
cg06986537	GPR180	13	Body	Open sea	67.68	4.72	71.20	4.79	-3.52	2.77E-04	6.39E-03
cg04656424	FAM38A	16	Body	S_Shore	54.00	4.09	57.52	3.63	-3.52	7.32E-05	2.15E-03
cg11654024		1	Intergenic	Open sea	40.39	4.23	43.91	5.40	-3.52	2.10E-03	2.93E-02
cg10091408		17	Intergenic	Open sea	63.95	4.63	67.47	5.03	-3.52	1.43E-04	3.75E-03
cg21792737	NOX3;NOX3	6	1stExon;5'UTR	Open sea	34.17	5.65	37.69	5.30	-3.52	2.36E-03	3.17E-02
cg16117273	PRKD1	14	TSS1500	S_Shore	26.49	3.91	30.01	5.82	-3.52	2.12E-03	2.95E-02
cg13195955	NEU1	6	Body	N_Shore	68.40	4.23	71.92	3.79	-3.52	2.49E-04	5.88E-03
cg18055067	LOC728264	5	Body	Open sea	70.70	5.58	74.22	5.01	-3.52	1.69E-03	2.51E-02
cg22707397		19	Intergenic	S_Shelf	82.25	6.44	85.76	4.18	-3.52	3.45E-03	4.13E-02
cg02775414	SLC44A4	6	Body	S_Shelf	68.82	4.25	72.33	3.74	-3.52	1.05E-04	2.92E-03
cg06494105	MIR637;DAPK3	19	TSS200;Body	S_Shore	78.25	3.61	81.77	3.49	-3.52	2.25E-05	7.74E-04
cg25594106		14	Intergenic	N_Shore	86.43	3.54	89.95	2.43	-3.52	1.30E-06	5.72E-05
cg12697237	KATNAL1;KATNAL1	13	5'UTR;5'UTR	N_Shelf	54.82	4.41	58.34	4.55	-3.52	1.14E-03	1.88E-02
cg26413942	ZNF608	5	TSS1500	Open sea	17.07	4.26	20.59	5.61	-3.52	4.37E-03	4.85E-02
cg01757255	RIMS1;RIMS1;RIMS1;RIMS1;RIMS1	6	Body;Body;Body;Body;Body	Open sea	63.78	4.94	67.29	3.42	-3.52	5.99E-04	1.17E-02
cg24481882	PDE7B	6	Body	Open sea	58.58	5.18	62.09	5.85	-3.52	1.64E-03	2.45E-02
cg24108888		6	Intergenic	S_Shelf	61.59	4.39	65.11	4.22	-3.52	2.84E-04	6.52E-03
cg04110886	PEX14	1	Body	Open sea	77.72	3.27	81.24	3.25	-3.52	9.65E-06	3.65E-04
cg02981853		19	Intergenic	Open sea	74.20	4.20	77.72	3.41	-3.52	1.49E-04	3.88E-03
cg14786726		5	Intergenic	Open sea	85.99	5.41	89.51	3.29	-3.52	2.09E-04	5.11E-03
cg06748966	HCST;HCST	19	TSS200;TSS200	S_Shore	63.79	5.55	67.30	6.00	-3.52	2.19E-04	5.32E-03
cg16095464	SOX5	12	5'UTR	Open sea	75.33	5.61	78.85	3.77	-3.52	1.48E-03	2.28E-02
cg11308046	LARP1;LARP1	5	Body;Body	Open sea	82.05	3.71	85.57	2.30	-3.52	9.33E-06	3.54E-04
cg00094269	CROCC	1	Body	Open sea	68.84	4.81	72.36	3.12	-3.52	1.58E-04	4.08E-03
cg23017594		14	Intergenic	Open sea	68.31	5.95	71.82	5.82	-3.52	1.94E-03	2.77E-02
cg21583565	CDCA7;CDCA7	2	TSS1500;TSS1500	N_Shore	25.70	4.70	29.22	5.80	-3.52	2.01E-04	4.95E-03

cg20591728	LOC404266;HOXB6;LOC404266	17	Body;TSS1500;Body	N_Shore	52.44	4.45	55.95	4.64	-3.52	1.37E-03	2.16E-02
cg20265043	IGF2BP3	7	TSS200	S_Shore	20.23	4.65	23.75	4.85	-3.51	1.10E-04	3.03E-03
cg12193731	ACVRL1	12	TSS1500	Island	28.25	4.91	31.77	5.42	-3.51	2.17E-03	2.99E-02
cg14425869		9	Intergenic	Open sea	58.53	4.29	62.04	6.31	-3.51	1.91E-03	2.74E-02
cg23633028		6	Intergenic	Open sea	79.52	5.55	83.04	4.90	-3.51	1.80E-03	2.62E-02
cg01803759		1	Intergenic	Open sea	46.28	3.07	49.80	3.12	-3.51	6.08E-06	2.39E-04
cg15649702	ABTB2	11	Body	Open sea	40.04	4.12	43.56	4.86	-3.51	3.60E-03	4.25E-02
cg00169792	SALL4	20	Body	Island	31.17	5.35	34.68	5.01	-3.51	2.08E-03	2.90E-02
cg06616221		15	Intergenic	Open sea	76.48	5.24	79.99	4.42	-3.51	1.55E-03	2.36E-02
cg26547947	TRMT12;TRMT12	8	1stExon;5'UTR	Open sea	6.56	2.90	10.07	5.25	-3.51	3.22E-05	1.06E-03
cg17126622	MICALL2	7	Body	S_Shore	50.82	4.95	54.33	4.48	-3.51	3.29E-04	7.31E-03
cg24765079	CDH1	16	Body	S_Shore	20.54	3.69	24.05	4.95	-3.51	2.82E-03	3.59E-02
cg00420742	NLRP12;NLRP12	19	Body;TSS1500	Open sea	66.31	4.70	69.82	4.41	-3.51	4.10E-04	8.69E-03
cg13611432	SPP2	2	TSS1500	Open sea	78.61	4.55	82.12	3.84	-3.51	3.60E-04	7.85E-03
cg08415998		10	Intergenic	N_Shore	22.04	4.81	25.55	5.61	-3.51	2.34E-03	3.15E-02
cg14312133	NCRNA00092	9	TSS1500	S_Shore	15.87	2.66	19.38	4.11	-3.51	4.91E-06	1.97E-04
cg12292060	GABBR1;GABBR1	6	Body;Body	N_Shelf	64.55	4.52	68.06	4.61	-3.51	4.34E-03	4.82E-02
cg17181046	RORA;RORA;RORA	15	TSS1500;Body;TSS1500	Open sea	60.30	5.79	63.81	5.09	-3.51	3.29E-03	3.99E-02
cg00069002		1	Intergenic	Island	49.15	3.42	52.66	3.70	-3.51	1.24E-06	5.48E-05
cg08392484	ELL2	5	Body	Open sea	86.66	4.61	90.17	3.25	-3.51	2.84E-04	6.51E-03
cg23266398		1	Intergenic	Open sea	69.68	4.17	73.19	3.38	-3.51	2.86E-04	6.56E-03
cg17166812	NDUFS2;ADAMTS4	1	5'UTR;TSS1500	N_Shelf	20.86	4.18	24.37	4.41	-3.51	2.32E-03	3.14E-02
cg10491628		6	Intergenic	Island	40.84	5.66	44.35	4.57	-3.51	4.11E-03	4.65E-02
cg22698744	RUNX1	21	Body	S_Shore	20.28	4.20	23.79	5.10	-3.51	3.84E-03	4.44E-02
cg26329618	RANBP3L;RANBP3L	5	TSS1500;TSS1500	Open sea	18.12	3.42	21.63	5.24	-3.51	1.25E-03	2.02E-02
cg08729059	ZNF593	1	TSS1500	N_Shore	39.93	4.53	43.44	4.60	-3.51	1.24E-03	2.01E-02
cg12629264		5	Intergenic	S_Shelf	80.52	5.96	84.03	5.26	-3.51	1.58E-03	2.39E-02
cg05118587	ETV3L	1	3'UTR	Open sea	68.81	4.91	72.32	4.99	-3.51	3.28E-05	1.08E-03
cg26304237	DNAJC6	1	TSS200	N_Shore	13.01	3.69	16.52	5.49	-3.51	3.74E-04	8.08E-03
cg26004810	FADD	11	TSS1500	N_Shore	63.62	5.06	67.13	5.06	-3.51	3.60E-03	4.25E-02
cg06569861		17	Intergenic	Open sea	55.46	4.84	58.97	4.89	-3.51	3.87E-03	4.47E-02
cg01200893	HLA-DOB	6	TSS1500	Open sea	74.87	4.58	78.38	3.16	-3.51	5.59E-04	1.10E-02
cg24447707	STK19;STK19;STK19	6	Body;Body;Body	S_Shelf	60.33	4.37	63.84	4.56	-3.51	2.12E-03	2.95E-02
cg23455651	BCAS4;BCAS4;BCAS4	20	Body;Body;Body	Island	81.59	3.24	85.10	2.19	-3.51	3.38E-07	1.61E-05
cg04526104		2	Intergenic	Open sea	53.08	4.58	56.59	4.66	-3.51	1.51E-04	3.94E-03
cg18120446		4	Intergenic	Island	25.26	4.29	28.77	4.18	-3.51	7.61E-06	2.94E-04
cg07638462	ZNF19	16	TSS1500	Open sea	29.25	4.89	32.76	5.07	-3.51	6.70E-04	1.27E-02
cg27016307	HRC	19	TSS1500	N_Shore	39.60	5.29	43.11	6.60	-3.51	2.29E-03	3.11E-02
cg08160091		4	Intergenic	Island	25.97	3.24	29.48	3.94	-3.51	1.21E-04	3.28E-03
cg17429000		5	Intergenic	Island	11.94	2.40	15.45	3.58	-3.51	3.19E-07	1.52E-05
cg26144819	HSPA4	5	Body	Open sea	66.40	4.76	69.91	3.46	-3.51	8.91E-04	1.57E-02
cg03573168		7	Intergenic	N_Shore	61.90	4.01	65.41	4.07	-3.51	2.42E-05	8.28E-04
cg21813187	LY6G6F	6	TSS200	S_Shelf	75.79	4.21	79.30	4.72	-3.51	1.10E-03	1.84E-02
cg04835284	SEC31B	10	TSS200	S_Shore	16.99	3.99	20.50	4.73	-3.51	2.15E-03	2.98E-02
cg00106345		7	Intergenic	S_Shore	26.14	4.09	29.65	5.58	-3.51	3.26E-03	3.96E-02
cg18752774	NLRP14	11	Body	Open sea	79.55	5.03	83.06	3.77	-3.51	3.47E-04	7.62E-03
cg11138547		1	Intergenic	Open sea	82.87	3.95	86.38	3.55	-3.51	1.39E-05	5.08E-04
cg16461692		13	Intergenic	Open sea	75.28	5.97	78.79	5.37	-3.51	1.66E-03	2.48E-02
cg01621080	SULT1A2;SULT1A2	16	5'UTR;TSS1500	N_Shelf	27.58	4.23	31.09	4.97	-3.51	4.50E-03	4.94E-02
cg26779406	NOTCH1	9	Body	Island	69.29	5.04	72.80	5.68	-3.51	4.08E-03	4.62E-02
cg09038599	SDR16C5	8	TSS200	S_Shore	75.19	5.55	78.70	4.95	-3.51	1.60E-03	2.42E-02
cg16407998	MAGI2	7	Body	Open sea	69.82	4.46	73.33	4.98	-3.51	1.70E-06	7.37E-05
cg27604702	NGEF;NGEF	2	Body;TSS1500	Island	39.25	5.08	42.76	4.81	-3.51	1.10E-04	3.03E-03

cg00039441	HOXD13	2	TSS200	Island	14.57	3.21	18.07	3.17	-3.51	2.19E-08	1.14E-06
cg02154269	OTOP1	4	TSS200	Island	46.86	3.79	50.36	4.85	-3.51	1.12E-03	1.86E-02
cg06607764	CYTH1;CYTH1	17	Body;Body	Open sea	65.40	4.79	68.91	3.98	-3.51	3.17E-03	3.89E-02
cg21466107	GLI3	7	Body	Open sea	69.19	4.65	72.70	4.48	-3.51	6.95E-04	1.30E-02
cg25051052	SPIRE1;SPIRE1;SPIRE1	18	3'UTR;3'UTR;3'UTR	Open sea	81.32	5.11	84.83	3.89	-3.50	1.46E-03	2.25E-02
cg24963147	LOC389332	5	TSS1500	S_Shore	45.03	4.07	48.54	4.94	-3.50	1.87E-03	2.70E-02
cg18521743	ZBTB45	19	Body	Island	22.46	4.56	25.97	4.61	-3.50	2.18E-03	3.00E-02
cg25045785	INPP5F;INPP5F	10	Body;TSS200	S_Shore	62.52	4.63	66.02	4.76	-3.50	3.23E-03	3.94E-02
cg26804848	CDAN1	15	TSS1500	S_Shore	17.58	3.71	21.08	4.64	-3.50	7.81E-04	1.42E-02
cg07691152		12	Intergenic	S_Shelf	24.03	4.62	27.53	4.22	-3.50	3.31E-04	7.35E-03
cg22262753	CDKAL1	6	Body	Open sea	85.35	3.99	88.86	2.62	-3.50	3.37E-05	1.10E-03
cg03126177	MAST1	19	Body	N_Shelf	81.65	4.02	85.15	3.55	-3.50	2.79E-05	9.36E-04
cg05194864	ARHGDIB	12	Body	Open sea	51.02	6.42	54.53	5.84	-3.50	3.71E-03	4.34E-02
cg02099390	OSBPL10	3	Body	Open sea	30.94	5.31	34.44	6.16	-3.50	2.90E-04	6.62E-03
cg03557857	KCNIP2;KCNIP2;KCNIP2;KCNIP2;KCNIP	10	Body;Body;Body;Body;Body;Body;Body	N_Shore	45.51	6.59	49.01	7.04	-3.50	6.34E-04	1.22E-02
cg15182222	PPFIBP2	11	Body	Open sea	85.13	4.63	88.63	2.92	-3.50	1.19E-04	3.24E-03
cg09994418		14	Intergenic	Island	24.08	4.49	27.58	5.10	-3.50	1.53E-03	2.34E-02
cg09315367	SLC44A2;SLC44A2	19	TSS200;Body	Island	28.80	4.18	32.30	4.45	-3.50	9.13E-04	1.60E-02
cg02728101	PDE2A	11	TSS1500	Open sea	42.45	5.00	45.96	5.78	-3.50	2.37E-04	5.66E-03
cg04431629	DMRTA2	1	TSS1500	Island	17.66	3.83	21.16	3.47	-3.50	2.95E-06	1.23E-04
cg18105361		5	Intergenic	Open sea	34.92	5.10	38.42	6.02	-3.50	3.80E-03	4.40E-02
cg22183049		5	Intergenic	Island	31.12	5.62	34.62	5.19	-3.50	5.94E-04	1.16E-02
cg03027547	PLEKHG1	6	TSS1500	N_Shore	59.38	5.35	62.89	5.09	-3.50	1.80E-03	2.63E-02
cg04998498	USP28	11	Body	Open sea	82.86	5.05	86.36	5.57	-3.50	1.93E-04	4.80E-03
cg15724945		1	Intergenic	N_Shore	36.83	5.41	40.33	5.83	-3.50	4.39E-04	9.17E-03
cg03948870		9	Intergenic	N_Shore	18.38	3.89	21.89	4.48	-3.50	1.06E-04	2.95E-03
cg25762974	FAIM2	12	Body	N_Shelf	66.39	3.85	69.89	4.67	-3.50	1.21E-04	3.27E-03
cg18728076	TNRC18	7	Body	S_Shore	56.41	5.88	59.91	5.73	-3.50	4.03E-03	4.59E-02
cg07362800		5	Intergenic	Open sea	63.02	3.71	66.53	4.31	-3.50	1.78E-05	6.29E-04
cg08981980	ANKLE2	12	3'UTR	N_Shore	61.73	4.16	65.24	4.01	-3.50	3.67E-04	7.97E-03
cg15936366	ALPK1;ALPK1	4	Body;Body	N_Shore	84.46	4.36	87.96	3.38	-3.50	1.22E-04	3.30E-03
cg27384070	HEATR2	7	Body	N_Shore	58.46	5.77	61.96	5.99	-3.50	2.84E-03	3.61E-02
cg26561943	PALM;PALM	19	Body;Body	Island	34.79	4.47	38.29	5.68	-3.50	9.28E-04	1.62E-02
cg09973102	C16orf7	16	Body	N_Shelf	83.09	3.02	86.60	3.04	-3.50	5.07E-06	2.03E-04
cg05266474	BCL11B;BCL11B	14	Body;Body	N_Shelf	40.04	4.89	43.54	6.25	-3.50	7.38E-04	1.36E-02
cg03208024		12	Intergenic	N_Shelf	58.71	2.97	62.21	3.17	-3.50	1.32E-05	4.82E-04
cg04615460		12	Intergenic	Island	38.14	5.76	41.64	5.59	-3.50	9.56E-04	1.66E-02
cg22351924	IFI6;IFI6;IFI6	1	Body;Body;Body	Island	80.99	3.26	84.49	2.59	-3.50	2.24E-06	9.54E-05
cg01289544	SUPT7L;SLC4A1AP	2	TSS1500;1stExon	Island	24.70	4.93	28.20	5.76	-3.50	2.83E-03	3.59E-02
cg01193217		8	Intergenic	Island	34.22	4.80	37.72	4.90	-3.50	1.24E-03	2.01E-02
cg20780953	RAB3D	19	TSS200	S_Shore	31.48	5.93	34.98	6.81	-3.50	7.42E-05	2.18E-03
cg08439109		17	Intergenic	N_Shore	30.52	3.74	34.02	4.16	-3.50	1.63E-05	5.84E-04
cg00422699	MAN2B1	19	Body	Island	23.56	3.38	27.06	3.90	-3.50	3.24E-05	1.07E-03
cg00500770		20	Intergenic	N_Shore	84.31	4.30	87.81	3.31	-3.50	2.17E-05	7.52E-04
cg04665204	BAHCC1	17	Body	Island	58.43	4.79	61.93	4.52	-3.50	8.48E-04	1.51E-02
cg04907738		10	Intergenic	Open sea	23.94	4.68	27.44	4.75	-3.50	4.56E-03	4.99E-02
cg07241532	CCDC102A	16	3'UTR	Open sea	67.38	4.16	70.88	3.63	-3.50	1.18E-04	3.20E-03
cg12392998	NPLOC4	17	Body	Open sea	89.15	3.30	92.65	1.80	-3.50	1.17E-07	5.82E-06
cg21807694	CROCC	1	TSS1500	Open sea	39.53	6.83	43.03	7.72	-3.50	1.09E-03	1.83E-02
cg19462976	GMCL1L	5	Body	Island	55.95	5.50	59.45	5.32	-3.50	3.95E-03	4.53E-02
cg01142693	TSTA3	8	Body	N_Shore	73.60	3.53	77.10	3.45	-3.50	6.99E-06	2.71E-04
cg23391090		10	Intergenic	Open sea	77.05	4.30	80.55	4.50	-3.50	2.89E-04	6.60E-03
cg14159747		11	Intergenic	N_Shelf	51.46	4.28	54.96	4.70	-3.50	1.55E-04	4.02E-03

cg06230736	FLJ45983;GATA3;GATA3;FLJ45983	10	TSS1500;TSS200;TSS200;TSS1500	Island	29.43	5.50	32.93	5.80	-3.50	1.80E-04	4.55E-03
cg27234747	PTPN4	2	5'UTR	Open sea	85.84	4.15	89.34	3.07	-3.50	1.25E-05	4.58E-04
cg06880420	NR5A2;NR5A2	1	Body;Body	S_Shelf	36.79	4.49	40.29	5.57	-3.50	1.23E-03	2.00E-02
cg15012054	GSG1;GSG1	12	Body;Body	N_Shore	26.28	4.13	29.78	4.37	-3.50	1.70E-04	4.34E-03
cg02579377		16	Intergenic	S_Shelf	48.01	4.98	51.50	4.84	-3.50	1.21E-03	1.98E-02
cg25118606	ACTG2	2	5'UTR	Open sea	71.18	4.80	74.67	4.41	-3.50	2.29E-03	3.11E-02
cg14222434	PDE10A;PDE10A	6	Body;5'UTR	Open sea	68.42	4.59	71.92	4.51	-3.50	7.19E-04	1.34E-02
cg02406098	TRIM71	3	Body	Open sea	77.69	4.55	81.18	4.19	-3.50	2.17E-03	2.99E-02
cg00083359	EEF1D;EEF1D;EEF1D;EEF1D;EEF1D;EEI	8	5'UTR;5'UTR;5'UTR;5'UTR;5'UTR;5'UTR;5'U	Island	78.58	3.58	82.07	3.80	-3.50	4.76E-05	1.49E-03
cg25714774	TTC15	2	Body	Open sea	84.27	4.79	87.77	3.52	-3.50	4.32E-04	9.05E-03
cg19380722	PKD2L1	10	TSS200	Open sea	57.39	4.02	60.89	4.22	-3.50	1.55E-04	4.01E-03
cg25123670		14	Intergenic	Open sea	45.97	4.08	49.46	4.83	-3.50	3.30E-04	7.33E-03
cg00501876	CSRNP1	3	5'UTR	N_Shore	29.91	4.31	33.40	5.56	-3.50	1.57E-03	2.37E-02
cg18553589		11	Intergenic	Open sea	85.30	4.75	88.79	4.58	-3.50	1.26E-03	2.03E-02
cg25960834	C20orf96	20	TSS1500	S_Shore	19.10	3.49	22.59	4.24	-3.50	3.31E-05	1.09E-03
cg24683680	EVX2	2	TSS200	N_Shore	21.47	4.60	24.97	4.89	-3.50	7.75E-04	1.41E-02
cg02787120	IMMP2L	7	5'UTR	N_Shore	55.29	6.11	58.78	5.16	-3.50	4.55E-03	4.98E-02
cg23963984	DLG5	10	Body	Open sea	33.90	8.13	37.39	9.95	-3.49	1.38E-03	2.17E-02
cg18646521		2	Intergenic	Island	21.81	3.41	25.30	4.20	-3.49	3.03E-05	1.01E-03
cg06872138	ETF1	5	Body	N_Shelf	53.09	4.66	56.58	4.58	-3.49	3.70E-03	4.33E-02
cg09221960		11	Intergenic	N_Shore	78.22	4.88	81.71	4.38	-3.49	2.71E-03	3.49E-02
cg11679124	FRMD4A	10	Body	Open sea	74.82	5.23	78.32	5.29	-3.49	2.00E-03	2.83E-02
cg10438089	FAM117A	17	Body	Open sea	60.23	7.26	63.72	6.54	-3.49	5.44E-04	1.08E-02
cg01148127	SLC43A2	17	3'UTR	Island	71.21	3.83	74.70	3.30	-3.49	6.39E-05	1.92E-03
cg06598091		12	Intergenic	Island	6.81	2.51	10.30	5.58	-3.49	7.09E-05	2.10E-03
cg08251815	C2orf89	2	Body	N_Shore	39.86	5.29	43.35	5.35	-3.49	6.86E-04	1.29E-02
cg05572584	MIR1910;C16orf74	16	TSS1500;5'UTR	Open sea	60.44	5.25	63.93	5.17	-3.49	1.24E-03	2.01E-02
cg02416986	B4GALT2;B4GALT2;B4GALT2	1	1stExon;5'UTR;5'UTR	S_Shore	79.39	4.20	82.88	3.18	-3.49	4.09E-05	1.30E-03
cg18496318		3	Intergenic	S_Shelf	54.09	4.21	57.58	4.50	-3.49	3.26E-05	1.07E-03
cg14154859	MARK1	1	Body	S_Shore	22.57	4.41	26.06	4.38	-3.49	3.75E-04	8.09E-03
cg07433663	MEIS2;MEIS2;MEIS2;MEIS2;MEIS2;ME	15	1stExon;Body;Body;5'UTR;Body;Body;Bod	Island	16.56	4.18	20.05	5.02	-3.49	5.13E-05	1.59E-03
cg00391668	ATAD3C	1	3'UTR	N_Shelf	36.32	4.54	39.81	5.06	-3.49	1.37E-03	2.16E-02
cg04424621	HIST1H2BJ	6	TSS1500	S_Shore	31.80	5.17	35.30	6.57	-3.49	8.08E-04	1.46E-02
cg26526897	VPS52	6	Body	Open sea	68.43	4.23	71.92	4.04	-3.49	2.90E-04	6.63E-03
cg12671247	RAD1;RAD1	5	Body;3'UTR	Open sea	78.48	5.10	81.97	4.57	-3.49	3.24E-03	3.95E-02
cg11785625	MYBL1;MYBL1	8	Body;Body	N_Shelf	75.58	4.68	79.07	4.81	-3.49	3.39E-04	7.49E-03
cg19871940		14	Intergenic	N_Shore	8.56	2.17	12.06	2.99	-3.49	3.06E-08	1.58E-06
cg16902385	ST8SIA3	18	TSS200	N_Shore	19.58	5.37	23.07	6.21	-3.49	2.04E-03	2.86E-02
cg11061343	C17orf106	17	Body	Open sea	44.70	3.39	48.19	3.51	-3.49	5.11E-06	2.04E-04
cg03705396	MAP6D1	3	TSS1500	Island	29.61	4.54	33.10	5.17	-3.49	1.71E-04	4.36E-03
cg01525376	LCK	1	TSS1500	S_Shelf	55.85	3.89	59.34	4.84	-3.49	1.08E-04	2.98E-03
cg10096040		14	Intergenic	Open sea	43.34	5.05	46.83	3.94	-3.49	9.27E-04	1.62E-02
cg15345743	RASSF3	12	Body	Open sea	68.21	6.70	71.70	6.32	-3.49	3.77E-04	8.14E-03
cg08528970		4	Intergenic	Open sea	32.84	5.14	36.33	4.79	-3.49	1.10E-03	1.84E-02
cg23089840	LRRC3	21	TSS1500	N_Shore	34.25	4.25	37.74	4.46	-3.49	1.10E-04	3.02E-03
cg25345520		7	Intergenic	Open sea	77.67	3.72	81.16	3.75	-3.49	1.33E-05	4.86E-04
cg16504661	CAPN14	2	TSS1500	Open sea	54.89	5.33	58.38	5.05	-3.49	3.31E-03	4.01E-02
cg05871425	LRBA	4	3'UTR	Open sea	86.07	3.45	89.56	3.05	-3.49	5.20E-06	2.07E-04
cg04878159		17	Intergenic	N_Shelf	50.44	4.24	53.93	4.33	-3.49	4.67E-04	9.61E-03
cg05744332	MNX1;MNX1	7	Body;Body	N_Shore	44.45	4.30	47.94	5.26	-3.49	2.80E-03	3.57E-02
cg09566995	TOLLIP	11	Body	S_Shelf	20.28	3.93	23.77	4.61	-3.49	2.48E-04	5.86E-03
cg17262755	ARHGAP26;ARHGAP26	5	Body;Body	Open sea	71.14	5.04	74.63	4.73	-3.49	1.27E-03	2.05E-02
cg01409736		3	Intergenic	N_Shelf	75.48	5.88	78.97	5.91	-3.49	1.04E-03	1.76E-02

cg18732064	REM2	14	Body	Island	15.26	4.33	18.75	5.10	-3.49	9.05E-05	2.58E-03
cg26002103	DLX2	2	TSS200	Island	14.66	3.31	18.15	4.31	-3.49	2.92E-05	9.74E-04
cg16145209	KCNQ1;KCNQ1	11	TSS1500;Body	Open sea	61.37	4.59	64.86	4.97	-3.49	2.76E-03	3.54E-02
cg05756446	RAVER2	1	TSS1500	N_Shore	52.84	5.33	56.33	5.38	-3.49	2.67E-03	3.46E-02
cg19922825		2	Intergenic	N_Shore	22.27	4.45	25.76	4.57	-3.49	1.99E-05	6.96E-04
cg04657684	ELMOD1;ELMOD1	11	Body;Body	Open sea	53.25	6.60	56.74	5.90	-3.49	4.50E-03	4.94E-02
cg09035882	ZFP64	20	Body	N_Shore	28.86	4.69	32.35	5.45	-3.49	2.83E-03	3.60E-02
cg08197571		5	Intergenic	Open sea	76.29	3.63	79.78	3.15	-3.49	2.99E-05	9.94E-04
cg11684897		17	Intergenic	N_Shore	36.73	7.19	40.22	4.56	-3.49	2.83E-03	3.60E-02
cg02405329	ARSA;ARSA;ARSA;ARSA;ARSA	22	Body;Body;Body;Body;Body	N_Shelf	83.44	4.07	86.93	3.89	-3.49	2.29E-04	5.51E-03
cg00969087		1	Intergenic	Island	44.71	4.01	48.20	4.80	-3.49	2.64E-04	6.16E-03
cg10145196	KIAA1949;KIAA1949	6	Body;Body	Open sea	27.85	4.29	31.34	3.97	-3.49	1.62E-04	4.17E-03
cg02761568	RNF182;RNF182;RNF182;RNF182	6	5'UTR;TSS200;TSS1500;TSS200	N_Shore	26.04	4.11	29.52	4.38	-3.49	3.37E-05	1.10E-03
cg10553890		11	Intergenic	Open sea	48.79	5.48	52.28	4.24	-3.49	1.59E-03	2.40E-02
cg27429663		2	Intergenic	N_Shelf	66.53	3.68	70.02	2.98	-3.49	9.81E-06	3.70E-04
cg11636127	GCNT2	6	5'UTR	Open sea	35.96	5.14	39.45	4.90	-3.49	1.01E-03	1.72E-02
cg15510325	KCNH2;KCNH2	7	Body;Body	N_Shelf	64.27	6.61	67.75	7.10	-3.49	3.77E-03	4.38E-02
cg16523078	C1orf52;C1orf52	1	Body;3'UTR	Open sea	64.79	5.41	68.27	6.19	-3.49	5.22E-04	1.05E-02
cg01721544	MEIS1	2	Body	Open sea	49.63	4.58	53.11	4.75	-3.49	1.70E-03	2.51E-02
cg18942451	ACCN2;ACCN2	12	Body;Body	N_Shore	53.49	3.18	56.98	4.18	-3.48	6.89E-05	2.05E-03
cg21391741	MMEL1	1	5'UTR	Island	20.68	3.97	24.17	4.04	-3.48	3.81E-04	8.21E-03
cg25023298		10	Intergenic	Island	36.75	4.06	40.23	3.77	-3.48	6.42E-04	1.23E-02
cg04858155	ALX4	11	TSS1500	Island	23.74	3.55	27.22	4.77	-3.48	6.60E-05	1.97E-03
cg25491247		2	Intergenic	Open sea	82.84	5.17	86.33	5.41	-3.48	9.00E-04	1.58E-02
cg26020069	TRAM2	6	Body	Open sea	25.42	3.48	28.90	3.62	-3.48	8.27E-06	3.17E-04
cg13357249	PIP5K1A;PIP5K1A;PIP5K1A;PIP5K1A	1	TSS1500;TSS1500;TSS1500;TSS1500	N_Shore	51.01	5.87	54.50	3.96	-3.48	4.22E-04	8.88E-03
cg26996818	COL23A1	5	TSS1500	S_Shore	24.75	3.89	28.23	5.01	-3.48	3.11E-04	7.00E-03
cg14838970	PPFIBP2	11	TSS1500	N_Shore	33.58	3.59	37.06	3.48	-3.48	9.25E-05	2.62E-03
cg21829420	MYH13	17	TSS1500	Open sea	41.00	3.32	44.49	3.07	-3.48	2.96E-05	9.87E-04
cg20784098	COL16A1	1	Body	Open sea	68.27	4.14	71.75	4.92	-3.48	2.04E-04	5.02E-03
cg09271279		14	Intergenic	Open sea	57.61	4.47	61.10	4.34	-3.48	3.11E-04	7.00E-03
cg01640945		7	Intergenic	Open sea	62.63	4.21	66.12	3.96	-3.48	2.54E-04	5.98E-03
cg11304234	IL18;IL18	11	5'UTR;1stExon	Open sea	25.32	4.60	28.80	4.72	-3.48	1.02E-03	1.74E-02
cg23005227	CISH;CISH	3	Body;Body	N_Shelf	85.92	4.60	89.40	4.23	-3.48	6.59E-06	2.57E-04
cg13426079	KCNQ4;KCNQ4	1	Body;Body	Island	58.82	4.66	62.30	4.24	-3.48	1.14E-03	1.89E-02
cg13623749	JARID2	6	Body	Open sea	59.49	5.23	62.97	4.72	-3.48	2.76E-03	3.54E-02
cg10342304	NXN	17	Body	Open sea	77.98	4.28	81.46	3.59	-3.48	2.14E-05	7.42E-04
cg08193132	FAM38A	16	Body	S_Shore	60.67	4.25	64.15	4.50	-3.48	8.43E-04	1.51E-02
cg19522940	HPGDS	4	TSS1500	Open sea	51.65	5.00	55.13	5.46	-3.48	2.15E-03	2.97E-02
cg16619378	HS3ST3B1;MGC12916	17	Body;Body	Island	19.73	4.88	23.21	4.40	-3.48	2.59E-03	3.38E-02
cg05343808	GRB10	7	5'UTR	N_Shelf	58.82	4.01	62.30	4.77	-3.48	1.14E-03	1.89E-02
cg19254163	GPR44	11	TSS1500	S_Shelf	40.78	4.52	44.26	4.76	-3.48	3.39E-03	4.08E-02
cg16929041	TMEM97	17	Body	S_Shelf	79.40	8.45	82.88	7.07	-3.48	1.98E-03	2.81E-02
cg19662219	AVEN;CHRM5	15	Body;TSS1500	Open sea	72.19	4.76	75.67	4.24	-3.48	4.31E-04	9.04E-03
cg00881207	AMZ1	7	5'UTR	S_Shore	29.92	6.04	33.40	5.76	-3.48	1.88E-03	2.70E-02
cg24906410	C19orf61	19	Body	Open sea	46.75	4.06	50.23	4.37	-3.48	3.40E-05	1.11E-03
cg16013309		5	Intergenic	N_Shelf	41.32	3.81	44.80	5.18	-3.48	2.60E-03	3.39E-02
cg16874669	GULP1	2	5'UTR	Open sea	69.77	5.16	73.25	5.35	-3.48	2.04E-03	2.87E-02
cg10424681	C6orf201;C6orf146;C6orf146	6	TSS200;5'UTR;1stExon	Island	54.13	4.60	57.61	4.77	-3.48	3.58E-04	7.82E-03
cg00316222		6	Intergenic	N_Shore	23.51	3.62	26.99	4.65	-3.48	1.05E-04	2.92E-03
cg20653375	FARP1	13	3'UTR	Open sea	77.49	4.69	80.97	4.03	-3.48	6.88E-04	1.29E-02
cg14665413	TLR6	4	TSS1500	Open sea	30.37	6.35	33.85	6.06	-3.48	2.92E-03	3.68E-02
cg05302531	SH2B2	7	Body	Island	32.07	3.63	35.55	3.50	-3.48	2.40E-04	5.72E-03

cg19907380		2	Intergenic	Open sea	72.97	5.22	76.45	4.30	-3.48	2.05E-04	5.05E-03
cg06834661	HK1;HK1;HK1;HK1;HK1	10	Body;Body;Body;Body;Body	Open sea	12.31	3.83	15.79	4.88	-3.48	7.06E-04	1.32E-02
cg02082342	VSIG2	11	TSS1500	Open sea	52.70	4.32	56.18	4.56	-3.48	3.84E-04	8.24E-03
cg11018723	RUNX3;RUNX3	1	Body;TSS200	Island	13.63	2.87	17.11	3.02	-3.48	1.55E-08	8.17E-07
cg14931062	IRS1	2	3'UTR	N_Shore	59.41	4.72	62.89	4.22	-3.48	2.59E-03	3.38E-02
cg01998806	CCDC88B	11	Body	S_Shelf	61.09	4.90	64.57	4.37	-3.48	1.67E-03	2.48E-02
cg03749154		1	Intergenic	N_Shelf	76.35	5.87	79.83	5.16	-3.48	2.07E-06	8.87E-05
cg04526480		1	Intergenic	S_Shelf	44.35	4.92	47.83	4.59	-3.48	1.08E-03	1.81E-02
cg16554981		11	Intergenic	N_Shelf	59.99	4.02	63.47	4.71	-3.48	2.07E-04	5.08E-03
cg25400358	GPR137;GPR137;GPR137;GPR137	11	Body;Body;1stExon;1stExon	N_Shore	65.00	5.11	68.48	4.25	-3.48	1.42E-03	2.22E-02
cg06932616	GRIN2D	19	Body	Island	68.97	4.61	72.45	5.50	-3.48	3.53E-03	4.19E-02
cg16728651		6	Intergenic	Open sea	38.01	5.01	41.49	6.39	-3.48	1.76E-03	2.58E-02
cg11021810	FOXN3;FOXN3	14	Body;Body	Open sea	71.61	4.07	75.08	4.03	-3.48	2.27E-04	5.47E-03
cg08103144	SYNPO;SYNPO;SYNPO;SYNPO	5	Body;Body;Body;Body	Island	75.48	4.65	78.96	4.05	-3.48	4.92E-04	1.00E-02
cg06363129	SOSTDC1	7	TSS200	Open sea	46.12	7.26	49.59	6.60	-3.48	1.60E-03	2.42E-02
cg26408224	MSI2;MSI2	17	Body;Body	Open sea	64.23	4.47	67.71	4.43	-3.48	3.59E-04	7.84E-03
cg00903041	HHAT;HHAT;HHAT;HHAT;HHAT;HHAT	1	Body;Body;Body;Body;Body;Body	Open sea	85.63	4.19	89.11	2.24	-3.48	1.61E-05	5.78E-04
cg06257936		7	Intergenic	Open sea	41.67	4.26	45.15	5.57	-3.48	3.62E-03	4.26E-02
cg26880661	KPNA5	6	Body	S_Shore	67.28	4.56	70.75	4.65	-3.48	1.47E-03	2.27E-02
cg04902929	C1orf203;C1orf203;C1orf203;C1orf20	1	Body;Body;Body;Body;Body	N_Shelf	30.77	4.22	34.24	4.77	-3.48	3.79E-03	4.40E-02
cg17244218	EHD4	15	Body	Open sea	35.54	5.77	39.02	4.93	-3.48	3.69E-05	1.20E-03
cg19488206	STK25	2	Body	Open sea	64.28	3.77	67.75	3.43	-3.48	3.49E-05	1.14E-03
cg00445443	HOXA2	7	TSS1500	Island	23.43	6.05	26.91	4.52	-3.48	4.31E-03	4.80E-02
cg21929875	VSIG2;VSIG2	11	5'UTR;1stExon	Open sea	39.41	3.68	42.89	3.68	-3.47	3.97E-04	8.46E-03
cg19722748	DLX6AS	7	Body	N_Shelf	23.49	3.15	26.97	4.01	-3.47	8.65E-06	3.30E-04
cg08830300		11	Intergenic	Island	47.57	3.88	51.05	4.81	-3.47	1.25E-03	2.02E-02
cg12564285	PDLIM4;PDLIM4	5	TSS1500;TSS1500	Island	39.84	4.50	43.32	5.56	-3.47	8.20E-05	2.37E-03
cg22855968		6	Intergenic	Open sea	71.60	4.79	75.07	4.70	-3.47	3.23E-04	7.21E-03
cg21291134	APCS	1	3'UTR	Open sea	36.03	4.72	39.51	5.82	-3.47	4.05E-03	4.61E-02
cg27413290	ZC3H3	8	Body	Island	89.04	5.63	92.51	2.18	-3.47	3.59E-03	4.24E-02
cg00915092	HHLA2	3	5'UTR	Open sea	59.88	4.29	63.36	5.36	-3.47	4.10E-03	4.64E-02
cg13607323		5	Intergenic	Open sea	45.45	4.46	48.92	4.10	-3.47	7.58E-05	2.22E-03
cg05497216	ANKRD11	16	5'UTR	N_Shore	56.27	4.09	59.75	3.24	-3.47	9.69E-05	2.73E-03
cg01898964		2	Intergenic	N_Shore	35.37	4.34	38.84	5.22	-3.47	1.40E-03	2.19E-02
cg05673287	SGK269	15	Body	Open sea	70.37	4.64	73.84	4.21	-3.47	2.54E-04	5.97E-03
cg12057368	SNHG3-RCC1;SNHG3-RCC1;SNHG3-RC	1	Body;Body;5'UTR	N_Shelf	63.55	4.00	67.02	3.92	-3.47	6.38E-04	1.22E-02
cg20847292	ARHGEF2;ARHGEF2;ARHGEF2	1	Body;Body;Body	Open sea	72.26	4.28	75.73	3.28	-3.47	1.11E-04	3.04E-03
cg26183265	MYO1C;MYO1C;MYO1C	17	Body;Body;Body	N_Shore	72.81	3.66	76.28	3.03	-3.47	2.27E-06	9.64E-05
cg06524039	BRI3;BRI3	7	Body;Body	S_Shore	15.39	3.74	18.86	5.97	-3.47	2.75E-03	3.52E-02
cg05793375		4	Intergenic	Open sea	67.40	4.61	70.87	4.69	-3.47	1.23E-03	1.99E-02
cg23829395		12	Intergenic	Open sea	51.23	6.37	54.70	4.51	-3.47	1.84E-03	2.66E-02
cg24464158		11	Intergenic	Open sea	74.97	4.62	78.44	3.76	-3.47	3.55E-04	7.76E-03
cg19776793	SLC38A10;SLC38A10	17	Body;Body	Open sea	76.34	5.41	79.81	3.22	-3.47	2.45E-03	3.26E-02
cg13077262	SPATS1	6	TSS200	Open sea	22.37	5.48	25.84	5.37	-3.47	3.17E-05	1.05E-03
cg10500570	MIR599;VPS13B;VPS13B;MIR875	8	TSS1500;Body;Body;TSS1500	Open sea	85.17	4.74	88.65	3.99	-3.47	1.67E-04	4.28E-03
cg05327192	KLHL35	11	3'UTR	N_Shelf	42.09	5.33	45.56	7.21	-3.47	9.84E-04	1.69E-02
cg16525330	SRL	16	1stExon	Open sea	75.20	4.18	78.68	3.45	-3.47	9.51E-05	2.69E-03
cg03673965	NR2E1	6	Body	Island	10.68	3.08	14.15	4.01	-3.47	2.27E-06	9.66E-05
cg02964555	NR4A2	2	Body	Island	78.77	4.40	82.24	4.00	-3.47	3.72E-04	8.05E-03
cg21772953		3	Intergenic	Open sea	75.04	4.15	78.51	4.49	-3.47	5.03E-04	1.02E-02
cg05283804		16	Intergenic	Open sea	44.65	3.96	48.12	3.53	-3.47	1.47E-04	3.86E-03
cg08299299	EP400	12	Body	Open sea	84.80	2.92	88.27	2.17	-3.47	5.99E-08	3.04E-06
cg20219381	RGS22	8	Body	Island	29.08	4.55	32.56	5.01	-3.47	4.74E-04	9.73E-03

cg08370082	SENP5	3	Body	Open sea	68.04	5.55	71.51	5.90	-3.47	2.86E-03	3.62E-02
cg05736186	PPFIBP2	11	Body	Open sea	77.60	4.52	81.07	3.71	-3.47	5.97E-04	1.16E-02
cg20995573	MAPK8IP2	22	TSS1500	N_Shore	15.90	4.17	19.37	3.74	-3.47	1.99E-04	4.92E-03
cg03774463	DLX6AS;DLX6	7	Body;Body	S_Shore	19.26	3.36	22.73	3.72	-3.47	3.59E-05	1.17E-03
cg27136643	NAT15;ZNF597	16	TSS200;TSS200	Island	42.48	5.33	45.95	4.11	-3.47	3.13E-03	3.86E-02
cg16200799	MRC2	17	Body	Open sea	64.44	4.90	67.90	4.65	-3.47	2.16E-03	2.98E-02
cg06942814	HOXA4	7	TSS1500	S_Shore	34.07	3.21	37.54	4.47	-3.47	6.91E-05	2.05E-03
cg24903144	PAX2;PAX2;PAX2;PAX2;PAX2	10	Body;Body;Body;Body;Body	Island	41.06	4.43	44.53	3.78	-3.47	6.70E-04	1.27E-02
cg11631547	USP28	11	3'UTR	Open sea	75.60	3.81	79.07	4.28	-3.47	8.74E-04	1.55E-02
cg23192604	ARID2	12	Body	Open sea	81.94	4.01	85.41	3.51	-3.47	6.48E-05	1.94E-03
cg17391741		5	Intergenic	N_Shore	69.82	4.13	73.29	3.43	-3.47	2.10E-04	5.13E-03
cg13560204		7	Intergenic	S_Shelf	76.42	5.18	79.88	4.19	-3.47	1.38E-03	2.17E-02
cg07508304		16	Intergenic	S_Shelf	64.31	4.35	67.78	4.28	-3.47	6.09E-04	1.18E-02
cg20727217	SFRP2	4	TSS1500	N_Shore	38.74	4.53	42.21	4.86	-3.47	5.41E-04	1.08E-02
cg25513108	NCRNA00171	6	Body	Open sea	60.66	3.87	64.13	2.45	-3.47	2.61E-05	8.84E-04
cg26413192		10	Intergenic	Open sea	70.39	5.95	73.86	4.45	-3.47	9.56E-04	1.66E-02
cg21054521		13	Intergenic	Island	13.41	3.04	16.88	3.76	-3.47	1.49E-05	5.40E-04
cg15974053	HSD17B14;HSD17B14	19	5'UTR;1stExon	N_Shore	10.30	4.52	13.76	5.38	-3.47	2.61E-03	3.40E-02
cg20747380	HOXA2;HOXA2	7	1stExon;5'UTR	N_Shore	35.14	4.06	38.61	3.66	-3.47	5.46E-05	1.68E-03
cg05958127	PHGDH	1	TSS1500	N_Shore	47.91	5.48	51.38	5.55	-3.47	2.54E-03	3.34E-02
cg22307058	SYNJ2	6	Body	Open sea	72.24	4.55	75.71	3.54	-3.47	2.01E-04	4.96E-03
cg04167239	ADRA2C	4	1stExon	Island	22.19	2.39	25.66	3.31	-3.47	2.56E-08	1.33E-06
cg22065604		4	Intergenic	Open sea	73.99	5.04	77.46	4.38	-3.47	1.46E-05	5.29E-04
cg05784856		13	Intergenic	Open sea	65.32	4.52	68.78	3.84	-3.47	2.45E-04	5.81E-03
cg26403608	LOC284009;METT10D	17	TSS1500;3'UTR	Open sea	47.87	4.55	51.34	4.80	-3.47	2.40E-03	3.21E-02
cg02466798	ARID3A	19	Body	Island	35.73	3.60	39.20	4.62	-3.47	4.43E-04	9.22E-03
cg03067403	C11orf16	11	TSS200	Open sea	75.19	4.07	78.65	3.67	-3.47	5.96E-04	1.16E-02
cg03498309		6	Intergenic	Open sea	80.73	5.20	84.20	4.40	-3.47	7.24E-04	1.34E-02
cg07274618	GALR2	17	TSS200	Island	16.68	3.35	20.14	3.67	-3.47	6.78E-05	2.02E-03
cg06495615	ARID3A	19	Body	Island	65.50	4.83	68.96	4.53	-3.47	1.64E-03	2.46E-02
cg20247853	CARKD	13	Body	N_Shore	83.55	4.64	87.02	4.88	-3.47	4.43E-04	9.22E-03
cg03854487	NKD1	16	Body	Open sea	75.05	4.69	78.51	4.87	-3.47	1.42E-03	2.21E-02
cg10496511	C14orf145	14	5'UTR	Open sea	59.99	3.03	63.45	2.99	-3.46	6.54E-06	2.55E-04
cg07150862	GGA2	16	Body	N_Shore	14.83	4.94	18.30	5.17	-3.46	2.47E-03	3.28E-02
cg16615154		6	Intergenic	Island	19.35	4.18	22.81	4.50	-3.46	7.34E-04	1.36E-02
cg03456938	HSF4;FBXL8;HSF4	16	TSS1500;Body;TSS1500	Island	18.49	4.27	21.96	4.62	-3.46	6.16E-05	1.86E-03
cg19368103	C6orf123	6	Body	Open sea	71.75	5.12	75.22	5.34	-3.46	4.49E-03	4.94E-02
cg03621470	FBRSL1	12	Body	Island	31.08	5.24	34.54	6.99	-3.46	6.04E-04	1.17E-02
cg01894846		14	Intergenic	S_Shore	29.61	3.68	33.07	3.88	-3.46	2.94E-04	6.69E-03
cg24333473	ZNF597;ZNF597;NAT15	16	1stExon;5'UTR;TSS1500	Island	16.92	3.87	20.38	4.29	-3.46	1.18E-03	1.94E-02
cg17029237	LLGL2;LLGL2;LLGL2	17	Body;Body;Body	N_Shelf	41.32	3.92	44.78	5.02	-3.46	3.39E-03	4.07E-02
cg00635560	CDK2;CDK2;SILV	12	TSS1500;TSS1500;Body	N_Shore	67.74	4.11	71.20	3.67	-3.46	6.37E-04	1.22E-02
cg04508984	MCAT;MCAT	22	Body;Body	N_Shelf	75.70	4.96	79.16	4.38	-3.46	2.40E-03	3.22E-02
cg12414557	RNF212;RNF212;RNF212;RNF212	4	1stExon;5'UTR;1stExon;5'UTR	Island	19.09	3.74	22.55	5.17	-3.46	4.00E-04	8.52E-03
cg10846328	BIN1;BIN1;BIN1;BIN1;BIN1;BIN1;BIN1	2	Body;Body;Body;Body;Body;Body;Bo	Island	25.00	3.65	28.46	4.93	-3.46	2.17E-03	2.99E-02
cg17001194		11	Intergenic	Open sea	76.80	4.50	80.26	5.09	-3.46	3.28E-04	7.31E-03
cg15147215	STAB1	3	Body	Island	60.30	4.40	63.76	5.34	-3.46	2.49E-03	3.29E-02
cg04939026		7	Intergenic	Island	41.29	4.59	44.75	4.20	-3.46	2.27E-04	5.48E-03
cg16864658	CCK	3	5'UTR	Island	21.19	4.74	24.65	4.84	-3.46	2.21E-03	3.03E-02
cg19026231	GALK1	17	TSS1500	S_Shore	24.48	4.11	27.94	4.74	-3.46	1.71E-03	2.53E-02
cg09385650	BANP;BANP	16	Body;Body	N_Shore	41.89	3.84	45.35	5.29	-3.46	1.43E-03	2.22E-02
cg24204017	FLNB;FLNB;FLNB;FLNB	3	Body;Body;Body;Body	Open sea	58.61	5.98	62.07	5.68	-3.46	3.74E-03	4.36E-02
cg22740547	RASSF10	11	1stExon	Island	14.48	4.81	17.95	5.45	-3.46	7.30E-04	1.35E-02

cg04774505	NRL	14	3'UTR	N_Shore	53.49	5.02	56.95	3.85	-3.46	6.63E-04	1.26E-02
cg01032650	ADAM33;ADAM33	20	Body;Body	Island	72.03	6.32	75.50	5.03	-3.46	3.09E-03	3.82E-02
cg00294534	PSMG3;PSMG3	7	3'UTR;3'UTR	N_Shelf	81.69	3.59	85.16	3.30	-3.46	1.55E-06	6.75E-05
cg16771783	DYDC2;DYDC1	10	TSS200;5'UTR	Island	40.74	5.06	44.20	4.70	-3.46	7.35E-04	1.36E-02
cg00199753	DTX1	12	Body	S_Shelf	81.17	5.48	84.63	3.13	-3.46	1.15E-03	1.90E-02
cg04543865	GAN	16	TSS1500	N_Shore	20.42	3.31	23.88	3.26	-3.46	2.18E-05	7.56E-04
cg10741418		14	Intergenic	S_Shelf	71.80	5.90	75.26	4.92	-3.46	6.34E-04	1.22E-02
cg01612158	FCRL4	1	1stExon	Open sea	47.77	5.04	51.23	5.28	-3.46	2.44E-03	3.25E-02
cg15185570		11	Intergenic	Island	43.70	4.07	47.16	4.02	-3.46	5.32E-04	1.06E-02
cg06990194	TBL3	16	Body	N_Shore	65.87	3.52	69.33	3.08	-3.46	2.36E-06	9.98E-05
cg04770764		8	Intergenic	S_Shelf	80.60	6.36	84.06	4.14	-3.46	2.73E-03	3.51E-02
cg26141173	TRMT12	8	TSS200	Open sea	5.93	1.75	9.39	4.56	-3.46	1.54E-06	6.72E-05
cg00779476	TNXB	6	Body	Island	56.83	5.11	60.29	4.47	-3.46	2.80E-03	3.57E-02
cg07197831	DNAJC5G	2	3'UTR	Open sea	32.10	5.45	35.56	5.04	-3.46	3.54E-04	7.75E-03
cg00651005	GPR179	17	1stExon	Open sea	74.57	4.04	78.03	3.81	-3.46	4.79E-05	1.50E-03
cg13370754	SSBP2	5	Body	Open sea	86.66	4.92	90.12	4.59	-3.46	2.20E-04	5.33E-03
cg14037088	SMAD4	18	Body	Open sea	72.80	4.95	76.26	4.02	-3.46	3.58E-04	7.82E-03
cg06910100	USHBP1	19	Body	Open sea	76.07	4.41	79.53	3.79	-3.46	1.44E-04	3.78E-03
cg00254524		2	Intergenic	Island	37.18	5.13	40.64	5.60	-3.46	4.03E-04	8.56E-03
cg16423186		15	Intergenic	Open sea	57.34	4.29	60.80	5.39	-3.46	3.20E-04	7.16E-03
cg20672131		1	Intergenic	N_Shelf	68.46	5.42	71.92	5.41	-3.46	2.29E-03	3.11E-02
cg17831440	MCF2L;MCF2L	13	Body;Body	N_Shelf	79.74	4.72	83.20	4.28	-3.46	3.04E-04	6.87E-03
cg03323953	PBX1	1	Body	N_Shore	87.02	4.09	90.47	2.04	-3.46	3.65E-06	1.50E-04
cg18191605	SPATA2;SPATA2;SPATA2	20	5'UTR;1stExon;5'UTR	N_Shore	77.04	5.07	80.49	4.97	-3.46	3.91E-04	8.37E-03
cg00003578		1	Intergenic	Island	42.01	3.53	45.47	4.62	-3.46	2.45E-03	3.26E-02
cg15440868		2	Intergenic	S_Shelf	72.22	4.45	75.68	4.16	-3.46	4.96E-04	1.01E-02
cg01723876	RAP1GAP;RAP1GAP;RAP1GAP	1	5'UTR;TSS1500;5'UTR	S_Shore	30.70	5.65	34.15	5.80	-3.46	1.82E-03	2.64E-02
cg12771852		4	Intergenic	Open sea	57.96	3.64	61.42	3.65	-3.46	3.98E-05	1.28E-03
cg25307665	HOXA5	7	TSS1500	Island	13.10	2.91	16.56	4.91	-3.46	5.29E-04	1.06E-02
cg17004906	FBXO6	1	5'UTR	S_Shelf	41.52	5.31	44.97	5.72	-3.46	3.34E-03	4.04E-02
cg22236681		4	Intergenic	Open sea	82.55	4.53	86.01	3.07	-3.46	5.51E-05	1.69E-03
cg03221702	ILF3;ILF3;ILF3;ILF3;ILF3	19	Body;Body;Body;Body;Body	Open sea	74.28	4.28	77.73	3.25	-3.46	9.35E-05	2.65E-03
cg00211496	POU6F2;POU6F2	7	Body;Body	Open sea	57.32	5.83	60.77	5.79	-3.46	1.02E-03	1.73E-02
cg22686240	C1QTNF7;C1QTNF7	4	TSS1500;Body	Open sea	66.71	5.99	70.17	4.83	-3.46	1.73E-03	2.55E-02
cg17300736	IGF2AS;INS-IGF2;IGF2;IGF2AS;IGF2	11	Body;Body;5'UTR;Body;TSS200	Island	53.31	5.81	56.77	5.09	-3.46	9.69E-04	1.67E-02
cg00659204		4	Intergenic	Open sea	56.34	4.81	59.80	5.47	-3.46	2.48E-03	3.28E-02
cg16260424		1	Intergenic	N_Shelf	23.88	3.39	27.34	5.03	-3.46	8.89E-04	1.57E-02
cg26538140		12	Intergenic	N_Shelf	37.59	3.97	41.05	5.34	-3.46	3.18E-03	3.90E-02
cg22878441		3	Intergenic	Island	20.14	3.94	23.59	3.54	-3.46	1.66E-04	4.26E-03
cg16176095	PRDM16;PRDM16	1	Body;Body	Open sea	78.13	5.07	81.59	5.31	-3.46	2.75E-03	3.52E-02
cg00983899	CABP2;CABP2;CABP2;CABP2	11	1stExon;5'UTR;1stExon;5'UTR	S_Shelf	79.55	3.92	83.01	3.48	-3.45	5.97E-05	1.81E-03
cg00803004	KLK3;KLK3;KLK3;KLK3;KLK3	19	Body;Body;Body;Body;Body	Open sea	61.63	4.03	65.09	3.54	-3.45	6.85E-05	2.04E-03
cg04019164		22	Intergenic	N_Shore	74.92	5.14	78.37	4.51	-3.45	1.67E-03	2.49E-02
cg08907282	LOC441089	5	TSS1500	Open sea	43.09	4.52	46.54	3.97	-3.45	2.62E-05	8.87E-04
cg13341941	YTHDC1;YTHDC1	4	Body;Body	Open sea	80.29	5.73	83.75	4.86	-3.45	3.26E-03	3.96E-02
cg00764514	ZNF138;ZNF138;ZNF138;ZNF138	7	TSS1500;TSS1500;TSS1500;TSS1500	N_Shore	44.28	4.99	47.74	4.88	-3.45	1.83E-03	2.66E-02
cg23657388	EMID2	7	Body	Open sea	41.20	4.16	44.65	4.72	-3.45	3.57E-03	4.23E-02
cg02418535	NIT2	3	TSS1500	N_Shore	57.28	5.02	60.74	5.40	-3.45	2.45E-03	3.26E-02
cg04237436	TRIM44	11	Body	Open sea	62.80	4.46	66.25	3.67	-3.45	1.02E-03	1.74E-02
cg10370305		11	Intergenic	Open sea	31.52	4.39	34.97	5.11	-3.45	3.16E-03	3.88E-02
cg02286249		13	Intergenic	N_Shelf	77.35	5.98	80.80	5.70	-3.45	4.51E-03	4.95E-02
cg13174660	VAX2	2	Body	S_Shelf	63.80	5.91	67.25	6.94	-3.45	4.47E-03	4.93E-02
cg09080522	PARVG;PARVG	22	TSS1500;TSS1500	Open sea	56.66	6.29	60.11	6.61	-3.45	1.26E-03	2.03E-02

cg03258749	MLLT11	1	3'UTR	N_Shelf	69.53	5.31	72.99	5.57	-3.45	3.17E-03	3.89E-02
cg14675871	CSF2	5	TSS1500	Open sea	70.41	4.26	73.87	3.47	-3.45	1.50E-04	3.91E-03
cg04766834	TPT1;SNORA31	13	Body;TSS1500	N_Shelf	63.38	4.95	66.83	5.09	-3.45	4.32E-03	4.81E-02
cg21733560	NUDT1;NUDT1;NUDT1;NUDT1;NUDT1	7	Body;Body;Body;Body;Body;Body;Body	S_Shelf	64.56	4.97	68.01	3.71	-3.45	4.17E-03	4.69E-02
cg19668034	GLIPR1	12	Body	Open sea	68.45	5.63	71.90	4.49	-3.45	1.46E-03	2.26E-02
cg11453079	HNF4A;HNF4A	20	3'UTR;3'UTR	Open sea	34.38	4.07	37.84	5.42	-3.45	4.11E-03	4.65E-02
cg26421169		11	Intergenic	Open sea	77.17	5.35	80.62	5.48	-3.45	1.04E-03	1.76E-02
cg10477878		15	Intergenic	Open sea	77.98	6.55	81.43	5.12	-3.45	3.65E-03	4.28E-02
cg15114672	VCAN;VCAN;VCAN;VCAN	5	TSS1500;TSS1500;TSS1500;TSS1500	N_Shore	12.96	3.27	16.41	5.55	-3.45	1.10E-03	1.84E-02
cg00840516	HYAL2;HYAL2;HYAL2	3	5'UTR;1stExon;TSS1500	S_Shore	50.45	3.73	53.90	3.43	-3.45	2.62E-04	6.12E-03
cg06912665	LARP1	5	TSS200	Open sea	51.29	4.64	54.74	5.17	-3.45	2.43E-03	3.24E-02
cg00602416		10	Intergenic	Island	70.96	4.66	74.41	5.93	-3.45	3.15E-03	3.88E-02
cg09247319	DNMT3L;DNMT3L	21	TSS1500;TSS1500	Open sea	71.75	4.74	75.20	4.39	-3.45	8.53E-04	1.52E-02
cg25799879	STMN4	8	Body	Open sea	48.95	4.68	52.40	5.20	-3.45	2.58E-03	3.38E-02
cg13929988	KLHL8	4	5'UTR	N_Shore	43.95	3.48	47.40	4.00	-3.45	5.81E-04	1.14E-02
cg26779147	FMN1	15	Body	Open sea	66.89	5.11	70.34	4.58	-3.45	1.12E-04	3.08E-03
cg11284973	RXRB	6	Body	N_Shelf	43.84	4.19	47.29	5.40	-3.45	3.56E-03	4.21E-02
cg25714956	AUTS2;AUTS2	7	Body;Body	Island	71.40	5.28	74.85	3.94	-3.45	3.18E-03	3.90E-02
cg04904388	LOXL3	2	Body	N_Shelf	77.82	3.79	81.27	4.33	-3.45	3.96E-05	1.27E-03
cg14428048		7	Intergenic	Island	28.71	3.77	32.16	4.09	-3.45	1.30E-05	4.78E-04
cg06843180		4	Intergenic	Open sea	78.64	4.68	82.09	5.21	-3.45	2.05E-03	2.88E-02
cg04993276	DNAJC6	1	TSS200	N_Shore	28.21	4.07	31.66	4.39	-3.45	3.52E-04	7.71E-03
cg26150981		7	Intergenic	Open sea	84.07	3.62	87.52	3.14	-3.45	5.11E-06	2.04E-04
cg07156336	RAB7A	3	5'UTR	Open sea	42.61	4.58	46.06	4.63	-3.45	3.12E-04	7.02E-03
cg19592732		3	Intergenic	S_Shelf	54.45	5.03	57.90	5.19	-3.45	6.23E-04	1.20E-02
cg13493071	GNA12	7	Body	Open sea	62.32	4.66	65.77	4.41	-3.45	6.75E-05	2.01E-03
cg01899911	C7orf50;C7orf50;C7orf50	7	Body;Body;Body	N_Shore	71.22	4.02	74.67	3.51	-3.45	1.24E-04	3.35E-03
cg27145740	PLXNB2	22	Body	Island	79.94	4.21	83.39	3.23	-3.45	1.44E-04	3.78E-03
cg07147475		4	Intergenic	S_Shore	29.46	3.56	32.90	4.49	-3.45	2.71E-05	9.13E-04
cg26369614	RASGRP3;RASGRP3;RASGRP3	2	Body;Body;Body	Open sea	79.19	4.89	82.64	3.99	-3.45	2.14E-03	2.96E-02
cg13197450	STEAP2;STEAP2;STEAP2	7	5'UTR;5'UTR;5'UTR	Open sea	60.24	5.01	63.69	3.85	-3.45	1.22E-04	3.30E-03
cg09791673	KIAA0754;MACF1;MACF1	1	1stExon;Body;Body	S_Shore	38.02	4.23	41.46	4.76	-3.45	2.68E-03	3.47E-02
cg10000195		4	Intergenic	Open sea	75.18	5.89	78.63	4.36	-3.45	1.93E-03	2.76E-02
cg15287353	BMPR1B	4	5'UTR	Open sea	33.55	4.43	36.99	4.82	-3.45	3.25E-03	3.95E-02
cg26433494	PLXNA4;PLXNA4	7	Body;3'UTR	Open sea	76.12	5.68	79.57	4.17	-3.45	3.63E-04	7.90E-03
cg00820922		21	Intergenic	N_Shore	72.06	4.92	75.51	5.23	-3.45	2.79E-04	6.43E-03
cg00161770	DOCK7	1	Body	Open sea	81.88	5.88	85.32	6.57	-3.45	2.30E-03	3.12E-02
cg19927373		8	Intergenic	Open sea	72.86	4.14	76.30	4.05	-3.45	3.75E-04	8.11E-03
cg06244016	MTHFD1L	6	TSS200	N_Shore	25.87	3.26	29.31	5.03	-3.45	2.36E-05	8.09E-04
cg27509510	KIF3C	2	Body	Open sea	84.90	4.90	88.35	3.07	-3.45	2.74E-04	6.35E-03
cg03047400		14	Intergenic	S_Shelf	73.03	3.88	76.48	3.51	-3.45	1.83E-04	4.60E-03
cg12798259	PAX6;PAX6;PAX6	11	Body;Body;Body	S_Shore	48.72	4.22	52.17	4.79	-3.45	1.54E-03	2.34E-02
cg10484965		2	Intergenic	Open sea	79.40	6.95	82.85	6.59	-3.45	3.26E-03	3.96E-02
cg13873269	SUSD1	9	TSS1500	S_Shore	45.07	4.02	48.51	3.93	-3.44	3.94E-05	1.26E-03
cg24251439	SBNO2;SBNO2	19	Body;Body	Island	38.63	3.75	42.07	3.74	-3.44	5.30E-04	1.06E-02
cg21013745	SORCS2	4	Body	Island	63.62	3.72	67.06	3.66	-3.44	1.11E-04	3.04E-03
cg06580725	ZIC2	13	Body	S_Shore	19.25	3.15	22.69	3.76	-3.44	1.29E-05	4.73E-04
cg19463228	THTPA;THTPA;THTPA	14	3'UTR;Body;3'UTR	S_Shelf	77.64	3.77	81.09	3.97	-3.44	4.70E-05	1.47E-03
cg04999435	HRASLS5;HRASLS5;HRASLS5	11	3'UTR;3'UTR;3'UTR	Open sea	64.70	4.65	68.14	4.82	-3.44	2.98E-05	9.91E-04
cg15696816	RAI1	17	3'UTR	Island	50.20	3.31	53.64	4.72	-3.44	2.60E-03	3.39E-02
cg09567439	FOXB1	15	Body	Island	16.22	3.36	19.66	3.41	-3.44	2.79E-07	1.34E-05
cg05937737	SLC7A14	3	5'UTR	Island	24.45	3.87	27.90	4.50	-3.44	2.36E-04	5.64E-03
cg18036143	SCARB1;SCARB1	12	Body;Body	Open sea	81.97	3.36	85.42	2.83	-3.44	5.40E-07	2.51E-05

cg12683813		2	Intergenic	Open sea	64.29	6.02	67.73	5.02	-3.44	4.23E-03	4.74E-02
cg09979356	ERN2	16	TSS1500	S_Shore	56.97	4.88	60.42	3.47	-3.44	1.21E-04	3.28E-03
cg01877937	L3MBTL;L3MBTL	20	TSS1500;TSS1500	N_Shore	40.52	3.67	43.97	3.50	-3.44	1.86E-05	6.54E-04
cg02853954	SH2B3	12	3'UTR	Open sea	80.41	5.13	83.85	4.14	-3.44	1.77E-03	2.60E-02
cg09238746	EIF4A3	17	TSS200	Island	18.54	4.68	21.98	5.03	-3.44	1.16E-03	1.91E-02
cg06964030	MIR1306;DGCR8	22	Body;Body	Island	72.11	4.77	75.56	3.87	-3.44	5.01E-04	1.01E-02
cg03363161		3	Intergenic	Island	22.03	3.78	25.47	4.06	-3.44	7.65E-04	1.40E-02
cg02755956	FYN	6	5'UTR	Open sea	62.79	5.21	66.23	5.43	-3.44	3.96E-04	8.44E-03
cg11213983	CCDC46;CCDC46	17	Body;Body	Open sea	46.38	5.10	49.82	5.10	-3.44	2.93E-03	3.68E-02
cg14631333	KIAA0415	7	Body	N_Shore	70.91	5.12	74.35	4.70	-3.44	3.74E-03	4.36E-02
cg02311801	ARHGEF17	11	Body	N_Shelf	77.79	4.43	81.23	3.06	-3.44	1.69E-04	4.32E-03
cg08794157	PMP22;PMP22;PMP22	17	5'UTR;TSS1500;5'UTR	Island	43.51	4.16	46.95	4.62	-3.44	6.87E-05	2.04E-03
cg00402516	MBD3	19	Body	Island	68.07	4.62	71.52	3.61	-3.44	1.71E-03	2.52E-02
cg11613015	APC;APC;APC	5	TSS200;TSS200;5'UTR	Open sea	20.75	7.41	24.19	8.15	-3.44	1.11E-03	1.85E-02
cg09024124	GATA2;GATA2;GATA2;GATA2	3	1stExon;5'UTR;5'UTR;TSS1500	Island	13.96	2.14	17.40	3.79	-3.44	3.93E-09	2.14E-07
cg13741781		14	Intergenic	Open sea	44.71	3.50	48.15	3.43	-3.44	2.25E-05	7.76E-04
cg02872373		7	Intergenic	N_Shore	31.66	4.28	35.10	5.02	-3.44	1.87E-03	2.69E-02
cg13760105	ZNF654	3	TSS1500	Open sea	81.97	4.90	85.41	2.91	-3.44	3.25E-04	7.25E-03
cg16204357		14	Intergenic	N_Shore	31.49	5.15	34.93	5.21	-3.44	1.21E-03	1.97E-02
cg22960952	C4orf26	4	TSS200	Open sea	48.19	6.18	51.63	6.13	-3.44	3.56E-03	4.21E-02
cg06621080	NAV1	1	Body	Open sea	55.06	4.67	58.50	4.06	-3.44	3.73E-05	1.20E-03
cg23197297		8	Intergenic	Open sea	47.00	4.03	50.44	4.69	-3.44	9.65E-04	1.67E-02
cg13996453		7	Intergenic	Open sea	49.45	4.23	52.89	4.06	-3.44	2.23E-05	7.71E-04
cg19389468	HMG20A	15	Body	Open sea	82.77	5.15	86.21	4.69	-3.44	7.86E-04	1.43E-02
cg06912403		7	Intergenic	S_Shelf	67.72	5.25	71.16	5.22	-3.44	8.53E-04	1.52E-02
cg14408978	SDC2	8	Body	Open sea	78.92	4.51	82.36	4.20	-3.44	2.64E-03	3.43E-02
cg20558612		7	Intergenic	Open sea	64.11	4.82	67.55	5.00	-3.44	4.21E-04	8.87E-03
cg08071130	LOC646498	3	TSS1500	Open sea	51.95	4.73	55.39	4.82	-3.44	4.32E-03	4.81E-02
cg25246281	MIR451;MIR144	17	TSS1500;TSS200	Open sea	74.95	3.69	78.39	2.59	-3.44	3.41E-05	1.11E-03
cg05361096	AQP2	12	Body	Island	49.73	3.95	53.17	3.87	-3.44	3.68E-04	7.99E-03
cg17632096		4	Intergenic	Open sea	77.28	4.14	80.72	4.39	-3.44	1.48E-05	5.37E-04
cg11967431	LOC440461	17	TSS1500	Island	38.58	3.55	42.02	3.79	-3.44	1.81E-04	4.57E-03
cg13414212	C2orf39;C2orf39	2	5'UTR;1stExon	Island	17.93	4.40	21.37	5.01	-3.44	1.42E-04	3.75E-03
cg23395749		5	Intergenic	S_Shelf	59.15	4.46	62.58	5.05	-3.44	1.30E-03	2.08E-02
cg25689499	METRNL	17	Body	N_Shore	78.24	5.34	81.68	4.51	-3.44	3.15E-03	3.87E-02
cg05314799		3	Intergenic	Open sea	58.05	4.78	61.49	4.97	-3.44	2.68E-03	3.46E-02
cg14967804	C1orf116;C1orf116	1	5'UTR;Body	Open sea	36.11	4.65	39.55	4.36	-3.44	1.01E-03	1.72E-02
cg22801949	IER5	1	TSS1500	N_Shore	44.78	4.29	48.22	4.88	-3.44	2.02E-03	2.85E-02
cg06427779		5	Intergenic	Island	32.10	3.72	35.54	4.86	-3.44	2.19E-04	5.31E-03
cg09992184	FAM46C	1	5'UTR	S_Shelf	71.31	4.11	74.74	3.44	-3.44	4.73E-05	1.48E-03
cg06763054	MTMR7;MTMR7	8	5'UTR;1stExon	Island	17.89	3.54	21.33	3.66	-3.44	5.34E-06	2.12E-04
cg01564135	EVX1	7	TSS1500	N_Shore	35.43	5.20	38.87	5.06	-3.44	2.97E-04	6.75E-03
cg01038940	EXOC6;EXOC6	10	Body;Body	Open sea	30.30	3.84	33.73	3.63	-3.43	2.40E-05	8.21E-04
cg23463099	CLSTN2	3	Body	Open sea	17.11	3.85	20.54	4.87	-3.43	1.72E-03	2.54E-02
cg00577935	APC;APC;APC	5	TSS1500;5'UTR;TSS1500	Open sea	15.51	6.31	18.94	7.46	-3.43	5.90E-04	1.15E-02
cg02406148	C1QTNF9B	13	TSS1500	Open sea	71.29	4.70	74.72	6.05	-3.43	3.25E-04	7.25E-03
cg08493294	DNMT3A;DNMT3A	2	Body;Body	Island	38.55	4.50	41.99	4.81	-3.43	1.18E-03	1.93E-02
cg21746050	PRPS1L1	7	TSS200	Open sea	86.19	4.88	89.63	3.00	-3.43	1.86E-04	4.67E-03
cg22703162		15	Intergenic	N_Shore	40.79	5.32	44.22	4.97	-3.43	2.68E-03	3.47E-02
cg12088314		6	Intergenic	Island	36.10	4.23	39.53	5.11	-3.43	1.45E-03	2.25E-02
cg13944644	GULP1	2	Body	Open sea	73.00	5.69	76.43	5.45	-3.43	2.41E-03	3.22E-02
cg05042708	CALHM2;CALHM2	10	TSS1500;TSS1500	S_Shore	45.33	4.56	48.76	5.32	-3.43	2.15E-03	2.97E-02
cg10512292		12	Intergenic	N_Shore	34.14	4.40	37.57	4.78	-3.43	6.79E-04	1.28E-02

cg04559293	KIF21A	12	Body	Open sea	88.24	3.14	91.68	2.39	-3.43	4.87E-08	2.49E-06
cg09121047	LOC339524	1	Body	Island	42.60	5.47	46.03	5.78	-3.43	3.69E-03	4.32E-02
cg22907295	DLG2	11	Body	Open sea	36.61	3.83	40.04	4.10	-3.43	3.26E-05	1.07E-03
cg24467825	TRIM39;TRIM39	6	Body;Body	Open sea	65.04	4.27	68.48	4.51	-3.43	6.74E-04	1.27E-02
cg21840875	SMCR7L	22	3'UTR	Open sea	42.57	3.39	46.00	2.93	-3.43	3.68E-05	1.19E-03
cg09577115		20	Intergenic	S_Shore	26.09	4.59	29.52	4.91	-3.43	1.68E-04	4.29E-03
cg03756929	KIAA1383;KIAA1383	1	3'UTR;1stExon	S_Shelf	72.28	6.10	75.71	5.01	-3.43	2.03E-03	2.86E-02
cg14703829	SCARF2;SCARF2	22	Body;Body	Island	30.97	5.00	34.40	4.83	-3.43	1.72E-03	2.54E-02
cg21708130	LRRFIP1	2	Body	N_Shelf	25.13	4.29	28.57	5.44	-3.43	1.33E-04	3.54E-03
cg12595013	ZIC1	3	1stExon	Island	30.81	6.07	34.24	6.43	-3.43	2.70E-03	3.48E-02
cg02404759		6	Intergenic	Open sea	76.44	6.28	79.87	5.10	-3.43	3.20E-03	3.91E-02
cg00454864	AGAP3;AGAP3	7	Body;Body	N_Shore	80.40	3.93	83.84	2.64	-3.43	8.42E-05	2.42E-03
cg14646244	SLC26A4;LOC286002	7	TSS1500;Body	N_Shore	70.32	5.18	73.75	6.02	-3.43	4.02E-03	4.58E-02
cg00021123	CROCCL1	1	Body	Open sea	82.89	4.33	86.32	3.97	-3.43	3.60E-04	7.85E-03
cg26806016	EIF4G3	1	Body	Open sea	77.50	5.12	80.93	4.60	-3.43	1.84E-03	2.67E-02
cg15952990	FERMT2;FERMT2;FERMT2	14	Body;Body;Body	Open sea	62.40	4.85	65.84	5.32	-3.43	4.52E-03	4.96E-02
cg24394097	KCNAB1;KCNAB1;KCNAB1	3	1stExon;5'UTR;Body	Open sea	76.67	4.01	80.11	3.41	-3.43	3.81E-05	1.23E-03
cg00038736	GGN	19	Body	Island	62.42	5.26	65.85	5.56	-3.43	2.44E-03	3.25E-02
cg10992686	ARL3	10	Body	Open sea	50.20	4.05	53.63	4.15	-3.43	2.85E-04	6.53E-03
cg20393695		6	Intergenic	Open sea	48.49	3.64	51.92	4.34	-3.43	1.39E-04	3.66E-03
cg03644034		6	Intergenic	Open sea	54.22	4.25	57.65	4.70	-3.43	7.21E-04	1.34E-02
cg11101032		15	Intergenic	Open sea	71.99	3.64	75.42	3.44	-3.43	2.77E-05	9.31E-04
cg06275853		11	Intergenic	N_Shore	30.02	3.68	33.45	4.11	-3.43	5.41E-05	1.66E-03
cg20435474		14	Intergenic	Island	24.80	4.02	28.23	3.72	-3.43	1.14E-04	3.12E-03
cg07441518		11	Intergenic	N_Shore	18.10	2.92	21.53	3.77	-3.43	6.28E-06	2.46E-04
cg22697572	LOC654342	2	TSS200	Open sea	28.52	4.54	31.95	6.27	-3.43	2.25E-03	3.07E-02
cg12528408	FSHR;FSHR	2	Body;Body	Open sea	18.45	3.11	21.88	4.50	-3.43	1.77E-04	4.48E-03
cg04643437	ATF7IP;ATF7IP	12	1stExon;5'UTR	Island	19.19	3.19	22.61	3.73	-3.43	1.71E-05	6.09E-04
cg20587874	MIR548N	2	Body	Open sea	56.88	4.59	60.31	4.46	-3.43	1.08E-04	2.99E-03
cg18260397	C5orf4	5	TSS1500	Open sea	32.81	5.00	36.24	5.53	-3.43	8.44E-04	1.51E-02
cg04951822	OAS1;OAS1;OAS1	12	Body;Body;Body	Open sea	25.98	4.62	29.40	4.97	-3.43	2.37E-03	3.19E-02
cg05641535	PRDM16;PRDM16	1	Body;Body	N_Shelf	67.98	4.66	71.41	4.42	-3.43	1.86E-03	2.69E-02
cg16815829		8	Intergenic	Open sea	76.66	5.49	80.09	4.04	-3.43	1.96E-03	2.79E-02
cg10490888	BRSK2	11	Body	S_Shore	79.08	4.35	82.51	3.80	-3.43	2.77E-04	6.39E-03
cg18084609	COCH;COCH	14	Body;Body	S_Shore	61.33	5.94	64.76	5.95	-3.43	3.76E-03	4.37E-02
cg25929589	MFSD2B	2	Body	Open sea	58.18	4.06	61.61	4.31	-3.43	2.00E-04	4.95E-03
cg14992656		2	Intergenic	Open sea	78.09	4.14	81.52	3.66	-3.43	2.05E-04	5.03E-03
cg12127863		13	Intergenic	Open sea	70.79	4.97	74.21	5.31	-3.43	1.98E-03	2.81E-02
cg07441953	ODZ4	11	Body	Open sea	76.70	5.51	80.13	3.85	-3.43	4.40E-04	9.17E-03
cg25827022	TNK1	17	Body	Island	58.29	4.98	61.72	5.14	-3.43	1.06E-03	1.78E-02
cg12193153	GPR142	17	TSS1500	Open sea	61.83	4.20	65.26	4.37	-3.43	1.49E-04	3.89E-03
cg17496990	SPINK5L2	5	Body	Open sea	63.13	6.39	66.56	4.17	-3.43	1.45E-03	2.25E-02
cg02624770	TRIL	7	1stExon	Island	27.95	4.77	31.38	5.45	-3.43	2.48E-03	3.28E-02
cg24197567	MET;MET	7	Body;Body	Open sea	41.44	5.73	44.87	6.76	-3.43	8.84E-04	1.56E-02
cg11189107	CTSG	14	3'UTR	Open sea	33.80	5.14	37.22	5.76	-3.43	4.10E-03	4.64E-02
cg26671120	TMEM195	7	Body	Open sea	82.52	3.82	85.95	2.54	-3.43	1.34E-05	4.90E-04
cg00101436	CDK14	7	3'UTR	Open sea	85.19	4.78	88.62	3.90	-3.43	6.62E-05	1.98E-03
cg12116462	LOC100130872-SPON2;SPON2;SPON2	4	Body;TSS1500;TSS1500	S_Shore	45.80	5.19	49.23	4.41	-3.43	2.79E-03	3.56E-02
cg13719771	NDUFA9	12	Body	S_Shelf	85.89	4.40	89.32	1.76	-3.43	2.98E-05	9.91E-04
cg06670742	EPS15L1	19	Body	Island	49.36	3.60	52.79	3.99	-3.43	2.24E-04	5.42E-03
cg26967619	DIRC3	2	TSS200	Island	36.65	4.64	40.07	5.47	-3.43	3.69E-03	4.32E-02
cg19471031		6	Intergenic	Open sea	87.19	4.72	90.62	3.36	-3.43	2.03E-04	5.00E-03
cg23006519	LOC84931	2	Body	Open sea	49.47	4.20	52.90	4.76	-3.43	7.20E-05	2.12E-03

cg00052385	ST3GAL1;ST3GAL1	8	5'UTR;5'UTR	N_Shore	39.29	3.76	42.72	5.03	-3.43	1.50E-03	2.31E-02
cg14975015	ATP6V0A4;ATP6V0A4;ATP6V0A4	7	5'UTR;5'UTR;TSS1500	Open sea	66.87	5.55	70.30	5.33	-3.43	1.65E-03	2.47E-02
cg09838568		21	Intergenic	Open sea	15.92	3.40	19.35	3.92	-3.43	1.50E-05	5.40E-04
cg07967918		6	Intergenic	N_Shore	47.63	5.00	51.05	4.90	-3.43	1.64E-03	2.46E-02
cg12961236	C14orf28	14	5'UTR	S_Shelf	80.58	5.85	84.00	4.88	-3.43	4.36E-03	4.84E-02
cg02237629		14	Intergenic	N_Shore	18.54	4.60	21.97	4.08	-3.42	2.32E-04	5.57E-03
cg05021842		19	Intergenic	Island	21.43	3.53	24.85	4.76	-3.42	1.02E-03	1.74E-02
cg15590912		1	Intergenic	S_Shelf	64.87	4.12	68.29	4.14	-3.42	6.41E-04	1.23E-02
cg21972567	EBF1	5	Body	Open sea	67.17	5.99	70.59	4.20	-3.42	9.73E-04	1.68E-02
cg08539965	EIF4G3	1	5'UTR	Open sea	73.48	7.22	76.90	5.04	-3.42	4.22E-03	4.73E-02
cg05482306	LOC100286948;TIMD4;TIMD4	5	TSS200;Body;Body	Open sea	54.39	5.45	57.81	5.50	-3.42	4.53E-03	4.97E-02
cg25985185		19	Intergenic	N_Shelf	76.26	4.71	79.69	3.42	-3.42	7.08E-05	2.10E-03
cg24829483	C1QTNF7;C1QTNF7	4	5'UTR;1stExon	Open sea	75.99	4.40	79.41	3.33	-3.42	2.18E-05	7.55E-04
cg01707976		16	Intergenic	S_Shelf	65.71	4.09	69.14	3.80	-3.42	2.94E-04	6.70E-03
cg25047379		2	Intergenic	S_Shore	50.32	5.34	53.74	5.53	-3.42	1.11E-03	1.85E-02
cg13209481	SFTA3	14	Body	Island	22.65	3.52	26.07	2.86	-3.42	1.45E-06	6.35E-05
cg23677243	MEIS2;MEIS2;MEIS2;MEIS2;MEIS2;ME	15	Body;Body;Body;Body;Body;Body	Island	55.44	3.96	58.87	3.66	-3.42	2.76E-05	9.27E-04
cg11453712	PHPT1;PHPT1	9	TSS1500;TSS1500	Island	16.18	3.41	19.61	4.85	-3.42	3.97E-04	8.46E-03
cg10580397	UBE2E2	3	Body	Open sea	84.49	4.80	87.92	2.97	-3.42	2.04E-04	5.02E-03
cg15464481	ADCY5	3	Body	Open sea	27.61	4.20	31.03	5.14	-3.42	1.86E-03	2.68E-02
cg10708189		1	Intergenic	Open sea	56.56	6.48	59.99	5.03	-3.42	3.83E-03	4.43E-02
cg07365127		7	Intergenic	Island	10.50	2.99	13.92	4.95	-3.42	5.81E-05	1.77E-03
cg06836725	IGFBP2	2	Body	Open sea	77.64	4.06	81.06	3.33	-3.42	7.01E-05	2.08E-03
cg24783785	VPS53;VPS53	17	TSS1500;TSS1500	S_Shore	13.40	3.55	16.82	4.39	-3.42	3.18E-04	7.12E-03
cg00061792		10	Intergenic	Open sea	70.06	4.96	73.48	3.51	-3.42	3.47E-04	7.62E-03
cg14163119	SARDH;SARDH	9	Body;Body	Open sea	60.25	4.61	63.67	4.26	-3.42	1.80E-03	2.62E-02
cg24319836	ANGPTL6	19	TSS200	N_Shelf	56.97	4.70	60.40	4.35	-3.42	6.83E-04	1.29E-02
cg13501279	CREG1	1	TSS1500	S_Shore	68.26	5.91	71.68	5.64	-3.42	7.35E-04	1.36E-02
cg03223100		2	Intergenic	Open sea	78.29	4.57	81.72	4.44	-3.42	8.63E-04	1.53E-02
cg09578877		2	Intergenic	Open sea	57.22	5.80	60.64	6.04	-3.42	1.87E-03	2.69E-02
cg04958055	ACTN1;ACTN1;ACTN1	14	Body;Body;Body	Open sea	38.56	7.19	41.98	6.96	-3.42	2.39E-03	3.20E-02
cg09158821	SLC43A2	17	Body	N_Shore	62.44	4.80	65.86	4.80	-3.42	8.30E-04	1.49E-02
cg03940662	UBE2I;UBE2I;UBE2I;UBE2I	16	Body;Body;Body;Body	Open sea	45.30	3.52	48.72	3.50	-3.42	2.57E-04	6.04E-03
cg17721910	KLF1	19	Body	Island	40.26	4.45	43.68	3.79	-3.42	1.08E-04	2.98E-03
cg08476984		10	Intergenic	Open sea	71.44	5.21	74.86	3.92	-3.42	2.62E-04	6.12E-03
cg12727398		2	Intergenic	Island	25.91	4.24	29.33	4.33	-3.42	3.05E-04	6.89E-03
cg14824266	RICH2	17	Body	S_Shore	30.82	5.16	34.24	5.41	-3.42	2.92E-03	3.68E-02
cg15586953		4	Intergenic	Open sea	67.07	5.18	70.49	5.77	-3.42	2.56E-04	6.02E-03
cg09861436	SLC35D1	1	Body	N_Shelf	82.74	4.36	86.15	4.79	-3.42	2.45E-03	3.26E-02
cg04272355		8	Intergenic	Open sea	36.92	3.57	40.34	3.74	-3.42	1.04E-04	2.90E-03
cg07495470	POLDIP3;POLDIP3	22	Body;Body	N_Shore	17.08	3.78	20.50	4.61	-3.42	2.29E-03	3.11E-02
cg05651820		3	Intergenic	Open sea	25.00	4.73	28.42	4.94	-3.42	4.08E-03	4.63E-02
cg04334108	TMEM184C	4	3'UTR	Open sea	84.82	6.28	88.24	4.02	-3.42	1.33E-03	2.11E-02
cg08599475	IL20RA	6	Body	N_Shelf	81.04	5.21	84.46	4.47	-3.42	1.59E-03	2.40E-02
cg17214107	FGF2	4	TSS1500	N_Shore	48.48	5.30	51.90	4.71	-3.42	2.59E-03	3.38E-02
cg04574480	NTF3	12	Body	S_Shelf	49.16	3.81	52.58	4.22	-3.42	1.48E-03	2.28E-02
cg02578184	RBMS3;RBMS3;RBMS3	3	Body;Body;Body	Open sea	67.65	5.60	71.07	5.82	-3.42	2.07E-04	5.07E-03
cg09618893	EHMT2;EHMT2	6	Body;Body	N_Shore	45.93	5.17	49.35	4.84	-3.42	9.68E-04	1.67E-02
cg22427313	EGFR;EGFR;EGFR;EGFR	7	Body;Body;Body;Body	Open sea	37.19	5.06	40.61	3.99	-3.42	2.83E-03	3.60E-02
cg04153551	FBLN5	14	Body	Open sea	45.36	4.39	48.78	4.35	-3.42	1.66E-03	2.48E-02
cg23817981	CCR4	3	TSS200	Open sea	57.26	6.03	60.68	5.72	-3.42	9.10E-04	1.60E-02
cg04472009	CREB3L2	7	Body	N_Shelf	76.53	3.30	79.95	3.53	-3.42	1.72E-05	6.11E-04
cg25137314		2	Intergenic	Open sea	67.01	5.02	70.43	4.23	-3.42	1.37E-04	3.64E-03

cg08242070	ADAP2	17	TSS1500	N_Shore	72.50	5.97	75.92	6.28	-3.42	1.36E-03	2.15E-02
cg27592453	PARVB;PARVB	22	3'UTR;3'UTR	Open sea	79.21	4.80	82.63	4.95	-3.42	2.84E-03	3.61E-02
cg03747002	GUCY2D	17	Body	Island	50.28	5.06	53.69	4.07	-3.42	2.37E-03	3.19E-02
cg23136742	KCNK3	2	Body	Island	77.15	5.46	80.57	4.67	-3.42	4.14E-03	4.67E-02
cg17128371	RGL2;RGL2	6	Body;Body	N_Shelf	68.48	4.11	71.90	3.73	-3.42	1.40E-03	2.20E-02
cg04396711	FGD5	3	Body	Open sea	53.88	4.96	57.29	5.20	-3.42	6.68E-04	1.27E-02
cg19579183		11	Intergenic	N_Shelf	30.34	4.21	33.75	5.93	-3.42	1.41E-03	2.20E-02
cg05544413	ECHDC1;ECHDC1;ECHDC1;ECHDC1;EC	6	5'UTR;5'UTR;5'UTR;TSS200;5'UTR	N_Shore	23.86	3.38	27.27	4.20	-3.42	3.59E-04	7.83E-03
cg08373622	VGLL3	3	Body	N_Shelf	75.11	5.41	78.53	5.56	-3.42	9.92E-04	1.70E-02
cg26159990	ERI3	1	Body	Open sea	69.26	4.32	72.67	3.54	-3.42	2.06E-03	2.89E-02
cg27346982	RNF157	17	Body	Open sea	58.58	4.01	62.00	3.90	-3.42	4.29E-04	9.01E-03
cg17995050		7	Intergenic	Island	68.34	4.76	71.75	3.44	-3.42	4.01E-04	8.54E-03
cg00976328	PLEKHM3	2	3'UTR	Open sea	87.29	3.54	90.71	2.33	-3.42	5.81E-07	2.69E-05
cg12315353	GABBR1;GABBR1;GABBR1	6	Body;Body;Body	Open sea	34.89	4.62	38.31	4.89	-3.41	1.56E-03	2.37E-02
cg08425760	AGTRAP;AGTRAP;AGTRAP;AGTRAP;A	1	TSS1500;TSS1500;TSS1500;TSS1500;TSS15	N_Shore	44.49	3.64	47.91	3.92	-3.41	4.46E-04	9.28E-03
cg04686264	SHPK	17	Body	N_Shelf	81.51	4.29	84.93	3.53	-3.41	5.18E-04	1.04E-02
cg03977084	HSPG2	1	Body	N_Shore	67.57	4.73	70.99	5.65	-3.41	1.68E-03	2.49E-02
cg22846067		15	Intergenic	S_Shore	59.73	4.93	63.14	4.24	-3.41	4.56E-04	9.44E-03
cg25090510	NIPAL4	5	TSS200	Island	23.69	4.98	27.10	4.91	-3.41	7.68E-05	2.24E-03
cg19400113	TP63;TP63;TP63;TP63;MIR944;TP63;T	3	Body;Body;Body;Body;TSS1500;Body;Bod	Open sea	76.45	4.84	79.86	3.63	-3.41	3.72E-04	8.06E-03
cg27423177	C3AR1	12	TSS1500	Open sea	62.27	6.55	65.68	6.39	-3.41	2.41E-03	3.22E-02
cg10228283	LOR	1	3'UTR	Open sea	26.94	4.18	30.35	5.40	-3.41	1.06E-03	1.79E-02
cg14771240	ZBTB22;ZBTB22	6	Body;Body	N_Shore	68.46	4.29	71.87	3.61	-3.41	2.20E-04	5.33E-03
cg05005609		11	Intergenic	Island	38.73	6.46	42.14	6.42	-3.41	1.14E-03	1.88E-02
cg21674153	B4GALT6	18	Body	N_Shore	24.73	4.85	28.15	5.18	-3.41	1.69E-03	2.51E-02
cg13662634	ANGPTL2;ANGPTL2;RALGPS1	9	1stExon;5'UTR;Body	Open sea	13.68	2.89	17.09	4.96	-3.41	4.61E-04	9.51E-03
cg15368264	C6orf191;C6orf191	6	5'UTR;1stExon	Open sea	77.15	5.26	80.56	4.49	-3.41	7.70E-04	1.41E-02
cg01243658	MRPL23	11	Body	N_Shore	85.53	4.61	88.94	3.64	-3.41	4.93E-04	1.00E-02
cg11951169	ASH1L	1	Body	Open sea	74.36	5.74	77.78	5.56	-3.41	3.67E-03	4.30E-02
cg14263139	RIN1	11	TSS200	S_Shore	35.17	5.15	38.58	4.77	-3.41	2.90E-04	6.62E-03
cg13711885		3	Intergenic	N_Shore	62.66	5.24	66.07	5.18	-3.41	2.29E-03	3.10E-02
cg17818471		2	Intergenic	Island	22.36	2.77	25.77	2.98	-3.41	3.08E-07	1.47E-05
cg02421824		12	Intergenic	Open sea	84.36	4.77	87.77	4.46	-3.41	1.98E-04	4.90E-03
cg00028014		2	Intergenic	Open sea	54.22	4.73	57.63	4.30	-3.41	3.29E-03	3.99E-02
cg13545297	HOXC8	12	Body	S_Shore	42.86	4.01	46.27	3.78	-3.41	5.25E-04	1.05E-02
cg22331032	NLRP6	11	TSS200	N_Shore	43.60	3.82	47.02	5.46	-3.41	3.88E-03	4.47E-02
cg15902297	VOPP1	7	Body	Open sea	51.71	4.92	55.12	4.26	-3.41	1.78E-03	2.60E-02
cg10321302	CCNY;CCNY	10	Body;5'UTR	Open sea	63.64	4.16	67.05	3.91	-3.41	1.54E-05	5.53E-04
cg16793019		6	Intergenic	N_Shelf	82.49	5.06	85.91	4.89	-3.41	2.92E-04	6.66E-03
cg00876112	MED12L	3	TSS1500	Island	24.54	4.61	27.96	3.34	-3.41	4.81E-05	1.50E-03
cg25720969	DLG2	11	Body	Open sea	70.22	4.50	73.63	4.67	-3.41	2.68E-04	6.23E-03
cg23081357		2	Intergenic	N_Shelf	68.17	5.00	71.58	4.15	-3.41	2.93E-04	6.67E-03
cg26078665	ENPP2;ENPP2;ENPP2	8	Body;Body;Body	Open sea	73.41	4.12	76.82	3.61	-3.41	1.67E-04	4.27E-03
cg08738722	MUC5B	11	Body	N_Shore	64.14	4.86	67.55	4.37	-3.41	4.21E-04	8.87E-03
cg17909996		19	Intergenic	Island	21.15	4.39	24.56	4.21	-3.41	9.93E-04	1.70E-02
cg26640866	TMTC1	12	Body	Open sea	75.95	4.98	79.36	4.15	-3.41	1.45E-04	3.80E-03
cg19850931	FHL2;FHL2;FHL2;FHL2	2	Body;Body;Body;Body	Open sea	80.50	4.31	83.90	3.39	-3.41	1.76E-05	6.23E-04
cg12839080		6	Intergenic	Open sea	83.39	3.60	86.79	2.93	-3.41	1.21E-05	4.47E-04
cg27055156		1	Intergenic	Open sea	57.86	4.53	61.27	5.16	-3.41	4.05E-03	4.61E-02
cg20318096	FABP3	1	TSS200	Open sea	27.10	3.74	30.51	4.53	-3.41	2.41E-04	5.74E-03
cg10403171	RCCD1;RCCD1	15	Body;Body	Island	74.53	4.66	77.94	4.84	-3.41	2.68E-03	3.46E-02
cg21368063	CGREF1;CGREF1;CGREF1	2	5'UTR;5'UTR;5'UTR	N_Shelf	58.71	3.97	62.11	4.31	-3.41	7.41E-05	2.17E-03
cg23666299	GRIK2;GRIK2;GRIK2	6	Body;Body;Body	Open sea	42.01	5.98	45.41	5.89	-3.41	3.09E-03	3.83E-02

cg07437024	RAB40C	16	Body	Island	47.23	4.41	50.63	4.36	-3.41	4.13E-03	4.67E-02
cg10337079	INS-IGF2;IGF2;IGF2;IGF2	11	Body;Body;5'UTR;5'UTR	N_Shore	10.01	2.52	13.41	5.28	-3.41	6.94E-04	1.30E-02
cg07673740		4	Intergenic	Island	24.39	2.91	27.80	4.31	-3.41	8.06E-06	3.10E-04
cg21900073		4	Intergenic	Open sea	37.76	4.26	41.17	5.68	-3.41	1.78E-03	2.60E-02
cg00218415	TAF4	20	Body	N_Shore	76.04	4.57	79.44	3.33	-3.41	9.71E-04	1.68E-02
cg02152650		1	Intergenic	S_Shelf	84.54	4.08	87.94	2.37	-3.41	3.48E-05	1.13E-03
cg21306588		1	Intergenic	Open sea	56.70	5.36	60.11	5.42	-3.41	3.52E-03	4.18E-02
cg24985672	IQCH	15	Body	Open sea	71.64	4.64	75.05	4.01	-3.41	3.69E-04	8.01E-03
cg01163071		16	Intergenic	S_Shelf	81.45	4.23	84.86	3.49	-3.41	8.25E-05	2.38E-03
cg04122161	SCYL3;SCYL3	1	Body;Body	Open sea	79.94	3.90	83.35	3.33	-3.41	1.76E-05	6.25E-04
cg16415875	TIMD4;TIMD4	5	TSS1500;TSS1500	Open sea	59.46	5.12	62.87	4.75	-3.41	2.51E-03	3.31E-02
cg10444683	PRDM2;PRDM2	1	Body;Body	Open sea	49.91	4.60	53.32	4.65	-3.41	1.44E-03	2.24E-02
cg16548961		5	Intergenic	N_Shelf	41.26	4.86	44.67	4.09	-3.41	4.43E-04	9.23E-03
cg13799081	RBM20	10	Body	Open sea	67.06	5.73	70.47	5.71	-3.41	2.38E-03	3.20E-02
cg13062888		1	Intergenic	Open sea	73.75	4.12	77.15	4.69	-3.41	3.12E-05	1.03E-03
cg11804721	SLC27A3	1	3'UTR	N_Shelf	23.37	3.64	26.78	4.15	-3.41	3.39E-04	7.49E-03
cg09685096	ZNHIT6;ZNHIT6	1	Body;Body	Open sea	76.42	4.91	79.83	4.12	-3.41	3.97E-03	4.54E-02
cg20530204	B3GALT4	6	TSS1500	Island	25.45	5.56	28.85	4.86	-3.41	1.33E-03	2.12E-02
cg08231697		1	Intergenic	S_Shore	67.25	4.20	70.66	4.21	-3.40	7.66E-04	1.40E-02
cg17031773	KLK9	19	TSS1500	Open sea	60.93	5.22	64.33	4.24	-3.40	5.40E-04	1.08E-02
cg02566952		2	Intergenic	S_Shelf	60.82	4.17	64.22	4.45	-3.40	2.53E-04	5.96E-03
cg23416952		3	Intergenic	Open sea	81.24	4.33	84.65	4.28	-3.40	1.09E-04	3.00E-03
cg16544463	INPP5A	10	Body	N_Shelf	57.46	4.48	60.87	4.36	-3.40	3.77E-05	1.22E-03
cg04180046	MYO1G	7	Body	Island	28.20	4.58	31.60	4.49	-3.40	3.00E-03	3.75E-02
cg14303017	NOTCH1	9	Body	S_Shore	78.77	3.82	82.17	3.04	-3.40	2.78E-05	9.34E-04
cg25839877		17	Intergenic	Open sea	66.79	4.98	70.20	4.48	-3.40	5.42E-04	1.08E-02
cg09178385	GJB3;GJB3	1	TSS200;TSS1500	N_Shore	49.97	5.31	53.37	4.20	-3.40	1.54E-03	2.35E-02
cg16153042	SLC22A23;SLC22A23	6	Body;Body	Open sea	61.52	4.83	64.92	4.39	-3.40	8.50E-04	1.52E-02
cg16596604	CRYM	16	5'UTR	N_Shore	41.29	5.90	44.70	5.71	-3.40	1.13E-03	1.88E-02
cg17302455	ABCA5	17	5'UTR	N_Shelf	82.93	4.73	86.34	3.83	-3.40	2.15E-03	2.97E-02
cg10204325		5	Intergenic	N_Shelf	69.93	4.40	73.33	3.84	-3.40	5.12E-04	1.03E-02
cg13799242	HEY2	6	Body	S_Shore	32.17	4.12	35.57	4.57	-3.40	3.84E-03	4.44E-02
cg01200585	C1orf69	1	Body	Island	89.59	5.91	92.99	3.33	-3.40	2.86E-03	3.62E-02
cg03231860	RICS;RICS;RICS	11	1stExon;Body;5'UTR	Open sea	74.41	5.75	77.81	4.10	-3.40	1.62E-03	2.43E-02
cg13354872	PARP16	15	3'UTR	Open sea	64.50	4.26	67.90	3.67	-3.40	6.66E-05	1.99E-03
cg10651168	ICAM1	19	Body	S_Shore	7.69	2.95	11.10	4.81	-3.40	1.83E-04	4.60E-03
cg19851647		4	Intergenic	Open sea	86.57	2.17	89.97	1.53	-3.40	3.28E-12	1.98E-10
cg16008327	GDF15	19	TSS1500	N_Shore	65.76	3.83	69.16	4.00	-3.40	6.79E-05	2.02E-03
cg13460389	MRPS31	13	Body	N_Shelf	60.09	4.61	63.49	4.31	-3.40	1.50E-03	2.31E-02
cg27134767	CHMP1A;CHMP1A	16	Body;Body	N_Shore	62.22	4.12	65.63	4.89	-3.40	1.11E-03	1.86E-02
cg06632207	ATP5G2;ATP5G2	12	TSS1500;TSS1500	N_Shore	27.41	4.40	30.81	5.37	-3.40	9.31E-04	1.62E-02
cg12687069	OR51I2	11	TSS1500	Open sea	74.55	5.52	77.95	5.37	-3.40	2.54E-03	3.34E-02
cg04204246	LAMA4;LAMA4;LAMA4	6	Body;Body;Body	Open sea	81.66	4.69	85.06	4.12	-3.40	5.36E-04	1.07E-02
cg04118849	ANKRD11	16	Body	N_Shore	64.69	4.18	68.09	4.34	-3.40	1.18E-03	1.94E-02
cg15911153	LOC389333;LOC389333	5	1stExon;5'UTR	Island	47.04	4.63	50.44	4.33	-3.40	4.47E-03	4.92E-02
cg16421726	ZRANB1	10	TSS1500	Open sea	72.40	4.07	75.80	3.13	-3.40	1.63E-04	4.20E-03
cg21267232		1	Intergenic	Open sea	75.27	3.59	78.67	2.87	-3.40	6.86E-06	2.67E-04
cg01996643	KLC1;KLC1;KLC1	14	5'UTR;5'UTR;5'UTR	Open sea	50.49	4.12	53.89	4.16	-3.40	1.11E-03	1.85E-02
cg26050975	MGAT4B;MGAT4B	5	Body;TSS1500	N_Shore	39.34	3.67	42.74	2.88	-3.40	7.45E-06	2.88E-04
cg09438522	NR2C2	3	5'UTR	Open sea	70.71	5.71	74.11	4.49	-3.40	2.35E-03	3.16E-02
cg01543654	C17orf93;PRAC	17	Body;TSS1500	Island	25.73	3.99	29.13	4.23	-3.40	2.22E-04	5.37E-03
cg08472797	SLC22A18;SLC22A18;SLC22A18AS	11	Body;Body;TSS1500	S_Shore	48.59	4.82	51.99	5.78	-3.40	3.77E-03	4.38E-02
cg05205647		2	Intergenic	N_Shore	10.78	2.70	14.18	2.96	-3.40	1.74E-06	7.52E-05

cg13989519	TSPAN18	11	5'UTR	Open sea	62.30	4.84	65.70	4.48	-3.40	3.18E-04	7.13E-03
cg12051956	C12orf53	12	Body	N_Shelf	36.90	5.73	40.30	5.33	-3.40	3.75E-03	4.36E-02
cg18753664		12	Intergenic	Open sea	70.91	5.65	74.31	5.44	-3.40	2.88E-03	3.64E-02
cg14462645	PAQR8	6	TSS1500	N_Shore	47.25	5.43	50.65	5.43	-3.40	2.35E-04	5.62E-03
cg08646570	STX8	17	Body	Open sea	81.59	4.58	84.99	5.02	-3.40	2.29E-03	3.11E-02
cg07465028	ALDH1A3	15	Body	N_Shelf	59.44	4.52	62.84	4.69	-3.40	4.28E-03	4.78E-02
cg02080857	LPAR3	1	3'UTR	Open sea	74.39	4.44	77.79	4.39	-3.40	3.33E-04	7.39E-03
cg23482259		6	Intergenic	N_Shelf	32.01	4.31	35.41	3.79	-3.40	1.74E-04	4.43E-03
cg13313598	MAP7	6	Body	Open sea	50.06	6.53	53.46	5.42	-3.40	4.07E-03	4.61E-02
cg09057763		16	Intergenic	Open sea	76.96	5.35	80.36	3.25	-3.40	8.90E-04	1.57E-02
cg06768944		8	Intergenic	Island	18.06	4.40	21.45	5.40	-3.40	2.20E-03	3.02E-02
cg03354772	C10orf93	10	TSS1500	Island	15.81	5.91	19.21	6.62	-3.40	2.45E-03	3.26E-02
cg06012959	TMEM232	5	Body	Open sea	68.75	3.81	72.14	3.13	-3.40	5.96E-07	2.76E-05
cg25408707	IGF2R	6	Body	S_Shelf	82.87	4.37	86.27	4.05	-3.40	1.85E-03	2.68E-02
cg06870728	FLJ45983;GATA3;GATA3;FLJ45983	10	Body;TSS1500;TSS1500;Body	Island	19.66	3.05	23.06	4.77	-3.40	5.44E-05	1.67E-03
cg01624348	DLC1	8	Body	Open sea	84.36	6.53	87.76	6.31	-3.40	1.70E-03	2.51E-02
cg17987458		1	Intergenic	Open sea	72.00	4.70	75.40	4.92	-3.40	4.72E-04	9.69E-03
cg26330063	FAM105A	5	3'UTR	Open sea	56.19	4.17	59.59	4.51	-3.40	8.53E-04	1.52E-02
cg24134261	RASGRP3	2	TSS200	Open sea	66.39	5.01	69.78	4.86	-3.40	7.95E-04	1.44E-02
cg15434778	NRXN3;NRXN3;NRXN3	14	Body;Body;Body	Open sea	55.72	5.37	59.12	5.88	-3.40	1.13E-03	1.87E-02
cg24607535	CDH26;CDH26	20	1stExon;5'UTR	Open sea	85.83	4.23	89.22	2.86	-3.40	6.41E-05	1.93E-03
cg26206284		7	Intergenic	S_Shelf	63.91	5.54	67.31	4.54	-3.40	1.72E-03	2.54E-02
cg21523574	SLC7A8;SLC7A8	14	3'UTR;3'UTR	Open sea	78.53	5.07	81.92	5.45	-3.40	1.45E-03	2.25E-02
cg17715419		6	Intergenic	Open sea	61.61	4.47	65.01	3.49	-3.40	4.74E-04	9.72E-03
cg17688460		6	Intergenic	S_Shelf	78.10	6.25	81.50	5.31	-3.40	2.48E-03	3.28E-02
cg13107144	TPM1;TPM1;TPM1;TPM1;TPM1;TPM1	15	Body;Body;Body;Body;Body;Body;Body	Open sea	66.31	4.58	69.71	3.51	-3.40	6.93E-04	1.30E-02
cg24676666		5	Intergenic	Open sea	87.42	3.59	90.81	2.21	-3.40	4.44E-08	2.27E-06
cg01013317		19	Intergenic	N_Shelf	84.88	4.46	88.27	2.89	-3.40	1.98E-04	4.90E-03
cg15791180	NUAK1	12	Body	Open sea	57.74	4.18	61.14	4.50	-3.40	2.95E-03	3.70E-02
cg01917259	INPP5B	1	TSS1500	Open sea	64.16	5.22	67.55	4.14	-3.40	1.87E-03	2.70E-02
cg02218200	SHANK3	22	Body	N_Shore	45.01	5.32	48.40	5.29	-3.40	3.53E-03	4.19E-02
cg21964481	SLC34A3	9	5'UTR	N_Shelf	66.06	4.53	69.45	5.61	-3.39	2.19E-03	3.01E-02
cg04107939	PLD1;PLD1	3	5'UTR;5'UTR	Open sea	42.23	4.24	45.63	5.11	-3.39	3.84E-03	4.44E-02
cg06688763	CDK6;CDK6	7	3'UTR;3'UTR	Open sea	15.95	3.33	19.34	5.13	-3.39	2.80E-03	3.57E-02
cg23210971	AFF1;AFF1	4	Body;Body	Open sea	67.90	5.27	71.29	4.30	-3.39	5.69E-04	1.12E-02
cg01057882		14	Intergenic	Open sea	85.66	3.66	89.06	3.20	-3.39	2.70E-05	9.11E-04
cg08493581	TCF7L1	2	Body	Open sea	56.25	4.52	59.64	4.21	-3.39	1.05E-04	2.92E-03
cg26314966		17	Intergenic	Island	71.62	4.68	75.01	7.25	-3.39	3.71E-03	4.34E-02
cg15089742	KSR1	17	Body	N_Shelf	57.99	4.34	61.38	3.18	-3.39	1.05E-04	2.91E-03
cg07620838	FILIP1	6	Body	Open sea	82.47	4.18	85.86	3.79	-3.39	6.34E-05	1.91E-03
cg04805512	RAB40B	17	Body	N_Shelf	77.93	4.03	81.32	3.91	-3.39	2.16E-04	5.25E-03
cg23429457		14	Intergenic	Open sea	41.20	6.23	44.60	6.58	-3.39	2.66E-04	6.19E-03
cg26607258		1	Intergenic	Open sea	64.52	4.80	67.91	4.65	-3.39	9.28E-04	1.62E-02
cg17427702		10	Intergenic	Open sea	29.15	3.95	32.55	5.34	-3.39	4.75E-04	9.73E-03
cg07479337	KIFC3;KIFC3;KIFC3	16	Body;Body;5'UTR	Open sea	61.27	3.51	64.66	3.83	-3.39	2.02E-05	7.05E-04
cg09153282		10	Intergenic	Open sea	66.48	7.32	69.87	5.74	-3.39	3.76E-03	4.37E-02
cg01742263	SLC23A1;SLC23A1	5	Body;Body	Island	74.19	5.09	77.58	5.09	-3.39	2.74E-03	3.51E-02
cg03873322	NMBR	6	Body	N_Shore	18.43	3.85	21.82	5.41	-3.39	1.65E-03	2.46E-02
cg01836906		2	Intergenic	Open sea	37.70	6.78	41.09	6.30	-3.39	2.29E-03	3.11E-02
cg23171179		12	Intergenic	Open sea	63.98	4.55	67.37	3.98	-3.39	5.75E-04	1.13E-02
cg06593118	FXYD6;FXYD6;FXYD6;FXYD6;FXYD6	11	Body;Body;Body;Body;Body	Open sea	54.57	4.75	57.96	4.80	-3.39	3.80E-03	4.41E-02
cg00727630	ZFP64	20	Body	Island	26.18	3.92	29.57	4.45	-3.39	2.99E-05	9.95E-04
cg14379108		9	Intergenic	Open sea	65.02	4.84	68.41	4.84	-3.39	3.57E-03	4.22E-02

cg13728386	RDX	11	3'UTR	Open sea	50.59	4.79	53.98	5.02	-3.39	2.84E-03	3.61E-02
cg18892244	HEATR2	7	Body	Island	79.30	4.10	82.69	2.50	-3.39	3.95E-05	1.27E-03
cg22522920	FAIM2	12	Body	Open sea	50.89	3.75	54.27	4.06	-3.39	2.18E-04	5.30E-03
cg10719746	MIMT1;PEG3;PEG3;ZIM2;PEG3;PEG3;	19	TSS1500;5'UTR;5'UTR;5'UTR;5'UTR;5'UTR;	N_Shore	59.60	6.32	62.98	4.59	-3.39	2.00E-03	2.82E-02
cg23068772		7	Intergenic	Open sea	65.39	4.48	68.78	5.52	-3.39	2.11E-03	2.93E-02
cg00268185	MYLK;MYLK;MYLK;MYLK	3	5'UTR;5'UTR;5'UTR;5'UTR	Island	29.14	6.05	32.53	6.13	-3.39	6.75E-04	1.28E-02
cg23009042	CSPG5	3	3'UTR	Open sea	54.80	4.94	58.19	5.76	-3.39	6.25E-04	1.20E-02
cg14879645		22	Intergenic	Open sea	76.02	4.57	79.41	4.45	-3.39	7.47E-04	1.38E-02
cg14070755	MLPH;MLPH	2	TSS1500;TSS1500	N_Shore	23.40	4.38	26.79	4.38	-3.39	1.91E-03	2.74E-02
cg07921535	SGCE;PEG10;SGCE;PEG10;SGCE	7	TSS1500;5'UTR;TSS1500;5'UTR;TSS1500	Island	21.92	3.71	25.31	3.87	-3.39	9.57E-05	2.70E-03
cg18187680	FLJ45983;GATA3;GATA3;FLJ45983	10	TSS1500;TSS1500;TSS1500;TSS1500	Island	16.25	3.55	19.64	4.85	-3.39	2.91E-04	6.65E-03
cg09222577	ANKRD11	16	5'UTR	Open sea	25.94	3.10	29.33	4.55	-3.39	6.42E-04	1.23E-02
cg07776163	ANO1	11	TSS200	N_Shore	4.27	3.00	7.66	5.79	-3.39	8.96E-05	2.56E-03
cg14487111		9	Intergenic	S_Shelf	33.82	4.20	37.21	5.37	-3.39	3.61E-03	4.26E-02
cg22460466	MAD1L1;MAD1L1;MAD1L1	7	Body;Body;Body	Island	79.43	4.54	82.81	3.46	-3.39	1.09E-03	1.82E-02
cg01827761	KLHDC7A	1	TSS1500	N_Shelf	28.58	4.59	31.96	4.44	-3.39	4.31E-03	4.80E-02
cg09945801	PURG;WRN;PURG	8	1stExon;TSS1500;1stExon	Open sea	27.91	3.88	31.30	4.96	-3.39	4.01E-03	4.57E-02
cg02258139	BHLHA15	7	TSS200	N_Shore	86.13	5.16	89.52	2.77	-3.39	8.55E-04	1.52E-02
cg08411094	MIR613;APOLD1	12	TSS1500;Body	Open sea	82.43	5.18	85.81	4.33	-3.39	2.97E-03	3.72E-02
cg16652347	PLXNA4;PLXNA4;PLXNA4	7	Body;Body;Body	Open sea	72.41	5.69	75.80	4.35	-3.39	2.17E-03	2.99E-02
cg00806239	TJAP1;TJAP1;TJAP1;TJAP1;TJAP1;TJAP	6	5'UTR;5'UTR;5'UTR;5'UTR;Body;TSS1500;5	Open sea	70.40	4.69	73.79	4.59	-3.39	7.54E-04	1.39E-02
cg16789707	RNF166	16	Body	S_Shelf	38.13	3.57	41.52	4.10	-3.39	1.81E-05	6.38E-04
cg15441473		10	Intergenic	S_Shelf	70.71	4.85	74.09	3.75	-3.39	2.69E-04	6.25E-03
cg12183124	XPO5	6	Body	Open sea	57.71	4.74	61.10	4.42	-3.39	1.08E-03	1.81E-02
cg13797673	PASK	2	Body	Open sea	74.89	3.98	78.27	4.12	-3.39	4.96E-05	1.54E-03
cg02475283	AP2A2	11	Body	N_Shore	76.01	4.45	79.40	3.97	-3.39	2.48E-03	3.28E-02
cg27476279		6	Intergenic	Open sea	72.47	4.73	75.86	4.09	-3.39	6.78E-04	1.28E-02
cg21225430		22	Intergenic	N_Shelf	80.28	3.71	83.67	3.67	-3.39	4.56E-05	1.44E-03
cg06813862		12	Intergenic	Open sea	35.73	3.92	39.12	5.32	-3.39	1.84E-04	4.63E-03
cg23534210	TBCD	17	Body	S_Shelf	66.27	5.18	69.66	3.85	-3.39	6.39E-04	1.22E-02
cg23527366	ABHD14A	3	Body	S_Shore	78.97	4.54	82.36	3.69	-3.39	3.85E-03	4.44E-02
cg13433278		2	Intergenic	N_Shore	43.86	5.17	47.25	3.86	-3.38	1.25E-03	2.02E-02
cg05365685	ATP2B2;MIR885;ATP2B2	3	Body;TSS200;Body	Open sea	72.72	4.87	76.10	4.58	-3.38	2.15E-03	2.97E-02
cg14972271	CAPN2;CAPN2	1	TSS1500;Body	N_Shore	44.69	5.32	48.08	4.94	-3.38	3.25E-05	1.07E-03
cg27541541	PRL;PRL	6	TSS1500;5'UTR	Open sea	73.63	5.01	77.02	4.27	-3.38	2.15E-03	2.98E-02
cg02574502	CACNG8	19	Body	Island	15.91	3.94	19.29	4.38	-3.38	6.04E-05	1.83E-03
cg01694554	SYTL2	11	5'UTR	Open sea	54.15	3.60	57.53	3.65	-3.38	6.42E-05	1.93E-03
cg15091747	RUNX1	21	Body	Island	71.75	4.57	75.13	4.09	-3.38	1.63E-04	4.19E-03
cg20982735	PSKH2	8	TSS200	Island	34.79	4.32	38.17	5.26	-3.38	4.37E-03	4.84E-02
cg22673543	FRMD4A	10	Body	N_Shore	45.99	5.51	49.38	5.08	-3.38	1.08E-03	1.81E-02
cg17488628	MARK1	1	Body	Open sea	62.63	5.12	66.01	5.10	-3.38	2.06E-03	2.89E-02
cg08088222	ORAI1	12	Body	Open sea	70.50	5.47	73.88	4.75	-3.38	4.24E-03	4.75E-02
cg02863489	PRSS27	16	Body	Island	16.04	3.32	19.43	3.54	-3.38	2.82E-05	9.44E-04
cg10466351		13	Intergenic	Open sea	74.76	4.67	78.15	3.83	-3.38	1.32E-04	3.52E-03
cg11607882	EFR3A	8	Body	Open sea	79.32	4.96	82.70	4.76	-3.38	2.45E-03	3.26E-02
cg20067334		7	Intergenic	S_Shore	8.00	4.45	11.38	5.38	-3.38	4.60E-04	9.50E-03
cg10247711	WDR25;WDR25	14	Body;Body	Open sea	68.02	5.40	71.40	5.86	-3.38	9.04E-04	1.59E-02
cg10622551	ZFYVE28	4	Body	Island	46.66	4.42	50.04	5.03	-3.38	1.65E-04	4.24E-03
cg02659920	EPS8L2	11	Body	Island	29.52	4.90	32.90	3.95	-3.38	2.40E-03	3.21E-02
cg10359230	ZNF331;ZNF331	19	1stExon;5'UTR	Island	20.40	4.71	23.79	5.45	-3.38	1.02E-03	1.74E-02
cg24569492		2	Intergenic	Island	27.72	4.20	31.10	4.33	-3.38	1.85E-04	4.64E-03
cg22116492	MET;MET	7	Body;Body	Open sea	78.98	4.01	82.37	3.73	-3.38	9.01E-05	2.57E-03
cg20260721		15	Intergenic	Island	60.96	4.80	64.34	5.19	-3.38	1.42E-03	2.22E-02

cg18853935		7	Intergenic	Island	14.76	2.83	18.14	3.86	-3.38	3.42E-06	1.41E-04
cg12475507	TP73;WDR8	1	TSS1500;TSS1500	Island	32.81	3.16	36.19	3.59	-3.38	7.63E-06	2.94E-04
cg11953334	GRIN2D	19	TSS1500	S_Shore	15.69	2.68	19.07	4.30	-3.38	1.14E-05	4.22E-04
cg17481278		4	Intergenic	Open sea	84.10	4.34	87.48	3.26	-3.38	2.53E-04	5.96E-03
cg13899814	ACVRL1;ACVRL1	12	Body;Body	Island	86.67	4.60	90.05	3.57	-3.38	4.56E-04	9.43E-03
cg19086570		3	Intergenic	N_Shore	57.93	4.83	61.31	6.42	-3.38	2.14E-03	2.96E-02
cg00911794	HIC1;HIC1	17	3'UTR;3'UTR	Island	27.44	5.61	30.81	4.91	-3.38	4.12E-03	4.66E-02
cg10799440		8	Intergenic	Open sea	18.99	3.64	22.37	4.22	-3.38	4.32E-04	9.06E-03
cg00720375	LOC100271715	2	TSS1500	Open sea	78.78	5.46	82.16	4.49	-3.38	3.19E-03	3.91E-02
cg07088882		11	Intergenic	N_Shelf	26.58	4.67	29.96	5.06	-3.38	5.42E-04	1.08E-02
cg14494379		13	Intergenic	Open sea	43.92	4.04	47.30	4.59	-3.38	1.15E-03	1.90E-02
cg11862551		6	Intergenic	N_Shore	19.21	5.06	22.59	5.36	-3.38	3.94E-03	4.52E-02
cg17840178	FLOT1	6	Body	N_Shore	17.44	4.31	20.82	4.69	-3.38	9.27E-05	2.63E-03
cg17416280	HIC1	17	TSS1500	Island	7.45	3.05	10.83	4.14	-3.38	5.19E-06	2.07E-04
cg13575161	CCND2	12	TSS1500	Island	24.94	5.24	28.32	5.74	-3.38	1.44E-04	3.77E-03
cg19096460		4	Intergenic	Open sea	78.06	4.54	81.44	3.68	-3.38	4.33E-04	9.08E-03
cg08373610	RFTN2	2	TSS200	Open sea	17.71	3.97	21.09	4.11	-3.38	5.74E-04	1.13E-02
cg07811160	MRO;MRO;MRO;MRO	18	TSS1500;TSS1500;5'UTR;TSS1500	Open sea	74.03	5.17	77.41	4.53	-3.38	1.54E-03	2.35E-02
cg26966828	MSRA;MSRA;MSRA	8	Body;Body;Body	Open sea	57.34	4.73	60.72	4.86	-3.38	3.38E-03	4.07E-02
cg00318877	METT5D1;METT5D1	11	Body;Body	Open sea	87.28	4.27	90.65	2.82	-3.38	1.67E-05	5.94E-04
cg24021343		3	Intergenic	Open sea	84.91	5.48	88.29	4.03	-3.38	1.26E-03	2.03E-02
cg18636739	CSDA;CSDA	12	Body;Body	Open sea	71.08	6.25	74.46	5.04	-3.38	1.37E-03	2.16E-02
cg23116540	THBS1	15	TSS1500	N_Shore	24.13	4.44	27.51	5.38	-3.38	4.29E-03	4.79E-02
cg11992621		2	Intergenic	Island	17.25	2.67	20.63	4.12	-3.38	6.22E-06	2.44E-04
cg21838013	CRTAM	11	TSS1500	Open sea	62.53	4.24	65.90	4.78	-3.38	7.23E-04	1.34E-02
cg07603382	TFPI2	7	TSS1500	S_Shore	74.90	4.97	78.28	4.45	-3.38	3.90E-04	8.35E-03
cg00523161	LPIN1	2	5'UTR	S_Shore	55.09	6.12	58.47	5.90	-3.38	3.65E-03	4.29E-02
cg15604993	MYO5C	15	Body	Open sea	64.14	4.31	67.52	3.60	-3.38	7.79E-04	1.42E-02
cg24950222	LYPD3	19	TSS200	S_Shore	38.00	4.13	41.38	3.99	-3.38	2.42E-04	5.75E-03
cg13615963	CCR6;CCR6;CCR6	6	5'UTR;5'UTR;1stExon	Open sea	21.40	3.90	24.77	4.01	-3.38	2.12E-04	5.18E-03
cg17611932	TRIM54;TRIM54	2	TSS200;TSS200	Open sea	55.26	4.32	58.63	5.26	-3.38	2.73E-03	3.51E-02
cg26077099		17	Intergenic	Open sea	68.59	5.02	71.96	3.63	-3.38	1.24E-03	2.01E-02
cg12877935	IGF2AS;INS-IGF2;IGF2;IGF2AS	11	Body;Body;5'UTR;Body	N_Shore	34.73	4.51	38.10	5.01	-3.38	3.22E-04	7.19E-03
cg12151942		3	Intergenic	N_Shore	43.35	5.56	46.73	4.23	-3.38	2.05E-03	2.88E-02
cg07760369	FAM195A	16	Body	Island	45.23	3.50	48.61	4.04	-3.37	7.42E-04	1.37E-02
cg15474894	TRIM26	6	Body	Open sea	82.13	5.43	85.51	4.54	-3.37	2.28E-03	3.10E-02
cg08204050	MIR451;MIR144	17	TSS1500;TSS1500	Open sea	68.47	5.22	71.84	3.36	-3.37	1.17E-03	1.93E-02
cg15881404	ARHGAP22	10	Body	Island	34.58	5.96	37.96	5.67	-3.37	9.48E-04	1.65E-02
cg17187163	MLPH;MLPH	2	TSS1500;TSS1500	N_Shore	16.82	3.41	20.20	3.88	-3.37	1.93E-05	6.78E-04
cg01106909	HCCA2;CTSD	11	Body;Body	S_Shelf	47.12	3.97	50.49	5.05	-3.37	1.24E-03	2.01E-02
cg11780096	RBM45	2	TSS1500	N_Shore	53.21	3.58	56.59	3.17	-3.37	2.03E-05	7.06E-04
cg05106770		4	Intergenic	S_Shore	52.14	6.35	55.51	6.43	-3.37	2.61E-03	3.40E-02
cg20098471	RASSF10	11	Body	S_Shore	27.05	5.86	30.42	5.32	-3.37	3.74E-03	4.36E-02
cg03960589	CNTNAP1	17	Body	S_Shore	48.42	4.11	51.79	5.56	-3.37	1.59E-03	2.40E-02
cg12301619		1	Intergenic	S_Shelf	86.18	4.93	89.55	2.89	-3.37	1.05E-03	1.77E-02
cg03629948	FGFR3;FGFR3;FGFR3	4	Body;Body;Body	Island	78.93	5.09	82.30	3.72	-3.37	2.41E-03	3.22E-02
cg02555579	SLC25A18	22	TSS200	Open sea	77.28	5.41	80.65	3.26	-3.37	1.94E-03	2.77E-02
cg13389502	HIC1;HIC1	17	Body;Body	Island	49.79	3.57	53.17	4.67	-3.37	2.61E-04	6.11E-03
cg03562414	ZNF577;ZNF577;ZNF577	19	Body;5'UTR;5'UTR	Island	15.60	4.22	18.97	5.42	-3.37	7.85E-04	1.43E-02
cg24368031	MRC2	17	Body	N_Shelf	28.00	4.78	31.38	4.77	-3.37	2.16E-03	2.98E-02
cg00193963	WIPF3	7	Body	N_Shore	10.58	2.93	13.95	4.57	-3.37	1.40E-05	5.11E-04
cg21274724	PITPNM3;PITPNM3	17	Body;Body	Open sea	61.36	3.82	64.73	4.05	-3.37	2.66E-04	6.20E-03
cg26341003		3	Intergenic	S_Shelf	76.41	4.23	79.79	3.21	-3.37	2.30E-04	5.53E-03

cg04006327	ATG16L2	11	Body	Island	29.29	4.00	32.66	4.46	-3.37	7.34E-04	1.36E-02
cg26292232		10	Intergenic	Open sea	74.43	6.47	77.80	5.43	-3.37	3.39E-03	4.07E-02
cg08144588	CNTN4;CNTN4;CNTN4	3	Body;TSS1500;Body	Open sea	69.80	5.24	73.18	4.41	-3.37	1.28E-03	2.05E-02
cg08679013	KLF1;KLF1	19	5'UTR;1stExon	S_Shore	56.06	3.59	59.43	3.20	-3.37	2.03E-05	7.08E-04
cg14123232		2	Intergenic	Island	32.63	3.92	36.00	4.29	-3.37	4.84E-05	1.51E-03
cg24024511	C7orf71	7	TSS1500	Open sea	70.91	5.99	74.28	5.52	-3.37	2.08E-03	2.90E-02
cg00230120	LOC100292680	12	TSS1500	Open sea	59.44	5.83	62.81	5.95	-3.37	2.11E-03	2.94E-02
cg15513620	ZNF697	1	5'UTR	Open sea	15.34	2.89	18.71	4.29	-3.37	1.10E-04	3.03E-03
cg00445126	LOC100132215	2	TSS1500	Island	23.75	2.96	27.12	3.85	-3.37	6.12E-05	1.85E-03
cg07093748	ARHGEF10L;ARHGEF10L	1	Body;Body	Open sea	82.46	3.53	85.83	2.80	-3.37	2.85E-06	1.19E-04
cg24936095	LIMS1	2	TSS1500	Open sea	52.84	4.68	56.21	3.80	-3.37	1.99E-04	4.93E-03
cg14713153	DIP2C	10	Body	Open sea	70.39	3.97	73.76	4.64	-3.37	3.88E-05	1.25E-03
cg00754947	GRB10	7	TSS1500	S_Shore	68.41	5.23	71.78	5.36	-3.37	3.61E-03	4.26E-02
cg16514473	ECE1	1	Body	Open sea	66.26	5.40	69.63	4.96	-3.37	3.71E-03	4.34E-02
cg23920642		7	Intergenic	S_Shore	42.47	3.99	45.84	4.34	-3.37	2.58E-04	6.06E-03
cg06627334	PCDH12	5	TSS1500	Open sea	65.95	4.12	69.32	4.09	-3.37	1.33E-03	2.11E-02
cg07701837	MAP2;MAP2;MAP2;MAP2	2	5'UTR;5'UTR;5'UTR;5'UTR	Open sea	73.69	4.57	77.06	4.03	-3.37	1.12E-03	1.86E-02
cg08289177	CXCL17;LIPE	19	3'UTR;TSS1500	Open sea	78.82	2.91	82.19	2.63	-3.37	2.48E-07	1.20E-05
cg02705163	NHSL1	6	TSS200	Open sea	17.49	3.26	20.86	4.58	-3.37	1.77E-04	4.48E-03
cg15686202	FAM124A	13	Body	N_Shelf	74.42	5.14	77.78	4.49	-3.37	9.09E-04	1.60E-02
cg12283724		6	Intergenic	N_Shelf	68.65	5.58	72.02	5.23	-3.37	1.53E-03	2.33E-02
cg09316772		7	Intergenic	Open sea	80.81	3.93	84.18	4.30	-3.37	7.81E-05	2.27E-03
cg19816379		7	Intergenic	N_Shelf	69.79	5.09	73.16	5.02	-3.37	2.56E-03	3.36E-02
cg12593515	RAPGEF5	7	Body	Open sea	64.79	3.66	68.16	4.46	-3.37	7.62E-04	1.40E-02
cg24467535		4	Intergenic	Open sea	24.33	3.92	27.70	3.74	-3.37	6.77E-04	1.28E-02
cg23999932		4	Intergenic	Island	11.21	2.82	14.57	3.79	-3.37	5.17E-07	2.41E-05
cg02924836	MIR25;MCM7;MCM7;MIR106B;MIR9	7	TSS1500;Body;Body;TSS200;TSS1500	Open sea	63.41	5.55	66.77	4.32	-3.37	3.09E-03	3.82E-02
cg12539415	HOXD4;HOXD4	2	5'UTR;1stExon	N_Shore	22.38	4.27	25.75	4.50	-3.37	4.73E-04	9.70E-03
cg06345767	KCNH3	12	Body	S_Shelf	72.20	4.20	75.56	4.48	-3.37	1.11E-03	1.85E-02
cg20307598		12	Intergenic	Open sea	62.27	4.69	65.63	4.05	-3.37	7.30E-04	1.35E-02
cg20160996	FERMT3;FERMT3	11	Body;Body	N_Shelf	69.75	5.02	73.12	5.10	-3.37	8.58E-04	1.53E-02
cg13327407	CLIP2;CLIP2	7	3'UTR;3'UTR	Open sea	61.15	4.65	64.52	4.07	-3.37	1.34E-03	2.12E-02
cg12520276	DIP2C	10	Body	N_Shore	53.65	4.43	57.01	4.38	-3.37	7.40E-05	2.17E-03
cg07142009		17	Intergenic	Open sea	64.58	6.13	67.94	6.78	-3.36	8.83E-04	1.56E-02
cg00089966		5	Intergenic	N_Shelf	27.17	4.11	30.53	5.00	-3.36	3.28E-03	3.98E-02
cg18347602	MAN1C1	1	Body	Open sea	61.17	4.95	64.54	4.89	-3.36	3.83E-06	1.57E-04
cg22150763	TCF19;TCF19	6	Body;Body	S_Shelf	76.97	4.09	80.33	3.93	-3.36	1.11E-03	1.85E-02
cg07037750	TMEM121	14	TSS1500	N_Shore	45.36	4.62	48.73	3.94	-3.36	5.62E-04	1.11E-02
cg23827284	MYLK2	20	TSS200	N_Shelf	77.59	4.56	80.95	4.21	-3.36	9.76E-04	1.68E-02
cg12497581	LFNG;LFNG	7	Body;TSS1500	N_Shore	66.92	4.59	70.29	4.97	-3.36	1.60E-05	5.72E-04
cg13857354		2	Intergenic	Open sea	33.61	4.88	36.97	4.64	-3.36	7.77E-04	1.42E-02
cg23517954	PSMD14	2	5'UTR	S_Shelf	85.21	5.54	88.57	3.49	-3.36	1.60E-03	2.41E-02
cg00161225	P2RX1	17	Body	Open sea	64.67	4.43	68.03	4.10	-3.36	3.82E-04	8.21E-03
cg17120578		8	Intergenic	S_Shelf	81.90	4.27	85.27	2.94	-3.36	2.04E-04	5.01E-03
cg02676476	H6PD	1	Body	N_Shore	75.31	4.80	78.67	3.52	-3.36	2.19E-03	3.01E-02
cg20556951	SP6	17	TSS1500	Open sea	26.92	4.01	30.29	3.37	-3.36	2.13E-04	5.19E-03
cg02771202	C1QTNF5;MFRP	11	Body;3'UTR	Island	30.83	3.34	34.19	4.29	-3.36	3.45E-05	1.13E-03
cg09397087		11	Intergenic	Open sea	63.72	4.86	67.09	5.09	-3.36	2.74E-03	3.52E-02
cg11324953	HTT	4	Body	S_Shore	76.96	4.35	80.32	3.59	-3.36	3.66E-04	7.95E-03
cg02690117		1	Intergenic	N_Shore	61.60	4.94	64.97	4.44	-3.36	2.64E-03	3.43E-02
cg00864012	TEX2	17	5'UTR	Open sea	42.73	4.98	46.10	4.23	-3.36	1.24E-03	2.01E-02
cg13575217		5	Intergenic	Island	51.46	5.57	54.82	6.87	-3.36	1.81E-03	2.64E-02
cg20326786	ASTN1;ASTN1	1	Body;Body	Open sea	48.32	4.32	51.68	4.40	-3.36	8.99E-04	1.58E-02

cg09116520		13	Intergenic	Open sea	65.39	5.29	68.75	4.40	-3.36	4.21E-03	4.73E-02
cg11871421	FBXO39	17	TSS200	Island	15.52	3.11	18.88	4.33	-3.36	1.89E-05	6.64E-04
cg06934774		7	Intergenic	N_Shore	17.22	3.46	20.58	4.13	-3.36	7.43E-05	2.18E-03
cg15421520	C14orf180;C14orf180	14	1stExon;5'UTR	Open sea	34.63	2.97	37.99	3.59	-3.36	1.47E-04	3.84E-03
cg18402166	LOC146880;LOC146880	17	TSS1500;TSS200	S_Shore	78.69	4.07	82.05	3.51	-3.36	9.36E-05	2.65E-03
cg08462879	PDE4B;PDE4B;PDE4B	1	Body;TSS1500;Body	Open sea	27.30	4.00	30.66	4.54	-3.36	8.58E-04	1.53E-02
cg04219099	MANEAL;MANEAL;MANEAL	1	Body;Body;TSS200	S_Shore	39.97	5.52	43.33	5.40	-3.36	2.54E-03	3.34E-02
cg26598540		8	Intergenic	Open sea	60.16	4.32	63.52	6.19	-3.36	4.07E-03	4.62E-02
cg07121900		13	Intergenic	N_Shore	56.12	3.55	59.48	4.54	-3.36	1.36E-04	3.60E-03
cg10256839	ST5	11	5'UTR	Open sea	66.54	4.49	69.90	3.72	-3.36	6.61E-05	1.97E-03
cg07015424		7	Intergenic	Open sea	73.21	5.55	76.57	4.51	-3.36	2.69E-04	6.25E-03
cg11549972		12	Intergenic	N_Shore	16.93	4.52	20.29	3.80	-3.36	9.06E-06	3.44E-04
cg02990289	MIR27A;MIR24-2	19	Body;TSS200	S_Shelf	29.37	2.89	32.72	3.26	-3.36	3.04E-06	1.26E-04
cg12013258		12	Intergenic	Open sea	74.87	5.53	78.23	4.95	-3.36	9.89E-04	1.70E-02
cg00873953	TMCC3	12	Body	Open sea	53.17	5.18	56.52	5.27	-3.36	2.75E-04	6.37E-03
cg25884399	GPSM1	9	Body	Island	14.44	5.77	17.80	6.13	-3.36	4.09E-03	4.63E-02
cg22790777	SLC25A39;SLC25A39	17	Body;Body	N_Shelf	84.06	3.20	87.42	2.54	-3.36	4.26E-06	1.73E-04
cg24568579		12	Intergenic	N_Shore	48.08	4.96	51.44	5.36	-3.36	1.08E-03	1.81E-02
cg00243854		18	Intergenic	N_Shelf	63.42	4.06	66.78	3.32	-3.36	1.52E-04	3.95E-03
cg09714184	NDE1	16	5'UTR	N_Shelf	75.89	4.54	79.24	3.53	-3.36	1.79E-03	2.62E-02
cg00318899	GNAI2;GNAI2	3	TSS200;Body	Open sea	38.46	4.13	41.82	4.39	-3.36	1.85E-05	6.52E-04
cg02685896	PROCA1;PROCA1	17	5'UTR;1stExon	Island	25.83	3.57	29.19	4.09	-3.36	9.26E-05	2.63E-03
cg12062133		8	Intergenic	Open sea	41.13	3.95	44.49	5.52	-3.36	3.27E-03	3.97E-02
cg22791643	PRR4;TAS2R20;PRH1	12	Body;TSS1500;5'UTR	Open sea	89.38	4.72	92.73	1.95	-3.36	1.50E-05	5.42E-04
cg20354882	AMDHD1	12	Body	S_Shore	69.50	5.06	72.85	3.77	-3.36	3.45E-04	7.58E-03
cg01566170	CAPN2;CAPN2	1	TSS1500;Body	N_Shore	59.43	5.37	62.78	5.05	-3.36	1.47E-04	3.84E-03
cg09262230	TMEM181	6	3'UTR	Open sea	26.08	3.82	29.43	4.49	-3.36	1.99E-03	2.82E-02
cg10311173	DPEP1	16	5'UTR	Open sea	56.44	4.03	59.79	4.35	-3.36	9.63E-04	1.67E-02
cg11708778		1	Intergenic	Open sea	80.95	4.18	84.31	4.26	-3.36	1.36E-03	2.15E-02
cg20288565	GNG4;GNG4;GNG4	1	5'UTR;5'UTR;5'UTR	Island	63.62	5.42	66.97	6.17	-3.36	2.57E-04	6.04E-03
cg03110165	GPC6	13	Body	S_Shelf	40.22	3.60	43.57	5.79	-3.36	3.13E-03	3.86E-02
cg01211097	USP10	16	TSS1500	N_Shore	32.61	4.18	35.97	5.47	-3.36	7.48E-04	1.38E-02
cg08999895	SLC22A18;SLC22A18;SLC22A18AS	11	Body;Body;TSS1500	S_Shelf	75.74	5.77	79.10	4.19	-3.36	1.35E-03	2.13E-02
cg06786153	TBC1D2B;TBC1D2B	15	Body;Body	Open sea	27.95	5.72	31.31	5.07	-3.35	4.43E-03	4.90E-02
cg17768957	SYTL3;SYTL3	6	1stExon;5'UTR	Open sea	39.93	5.05	43.28	5.16	-3.35	8.59E-04	1.53E-02
cg18459489	VAX1	10	3'UTR	N_Shore	25.25	4.27	28.61	5.00	-3.35	3.22E-04	7.19E-03
cg17307230		8	Intergenic	Open sea	60.15	4.32	63.51	4.16	-3.35	1.73E-04	4.41E-03
cg01451808		8	Intergenic	Open sea	73.55	5.46	76.90	5.41	-3.35	7.24E-04	1.34E-02
cg20659418		6	Intergenic	N_Shelf	18.43	4.71	21.79	4.90	-3.35	1.60E-04	4.13E-03
cg26852286	OPA3	19	Body	Island	34.36	4.91	37.71	5.33	-3.35	1.27E-03	2.04E-02
cg08271852		5	Intergenic	Open sea	62.89	4.53	66.24	4.52	-3.35	2.05E-03	2.87E-02
cg24003649		14	Intergenic	Open sea	60.84	4.97	64.20	4.20	-3.35	2.95E-03	3.70E-02
cg05131940	PRKG1;PRKG1	10	Body;Body	Open sea	39.36	4.77	42.71	5.02	-3.35	4.17E-03	4.69E-02
cg15081566	C11orf92;C11orf93	11	5'UTR;TSS200	Island	7.91	3.18	11.26	3.46	-3.35	1.05E-06	4.68E-05
cg10983208	SPOCK2;SPOCK2	10	5'UTR;5'UTR	S_Shore	23.75	3.69	27.11	4.38	-3.35	1.50E-05	5.41E-04
cg15914335	PXDN	2	Body	N_Shore	62.87	5.11	66.23	4.55	-3.35	3.34E-03	4.04E-02
cg03938060	C16orf71	16	Body	N_Shelf	49.75	3.97	53.10	4.59	-3.35	1.12E-03	1.87E-02
cg12879713	C11orf2;TM7SF2	11	Body;TSS1500	N_Shore	75.11	5.01	78.46	3.56	-3.35	2.83E-03	3.60E-02
cg27105465	HCRT	17	TSS200	S_Shore	74.97	4.62	78.32	3.98	-3.35	7.80E-04	1.42E-02
cg22702960	TRPM4	19	Body	Island	86.24	5.00	89.59	3.96	-3.35	1.69E-03	2.51E-02
cg12880095		17	Intergenic	Open sea	77.53	4.65	80.88	4.66	-3.35	8.75E-04	1.55E-02
cg21432842	CSF3;CSF3;CSF3;CSF3;CSF3;CSF3	17	1stExon;5'UTR;5'UTR;1stExon;5'UTR;1stEx	Open sea	46.88	3.96	50.24	3.82	-3.35	1.51E-04	3.94E-03
cg07973591	CHRNA3;CHRNA3	15	Body;Body	N_Shore	54.59	4.78	57.94	4.26	-3.35	1.07E-03	1.80E-02

cg09347495	CLIC5	6	TSS1500	Open sea	83.11	4.23	86.46	3.26	-3.35	5.21E-04	1.04E-02
cg21934015	GP1BA	17	TSS1500	Open sea	83.48	3.36	86.83	2.65	-3.35	9.57E-07	4.31E-05
cg05045981		7	Intergenic	Island	22.41	3.66	25.76	4.38	-3.35	5.46E-04	1.08E-02
cg14734916	ZBTB12	6	Body	Island	73.89	3.53	77.24	2.80	-3.35	3.25E-05	1.07E-03
cg04409872	FGFR1OP;FGFR1OP	6	Body;Body	Open sea	75.39	4.21	78.74	4.39	-3.35	5.22E-04	1.05E-02
cg22394530		1	Intergenic	S_Shelf	82.53	4.66	85.88	3.11	-3.35	2.55E-04	6.00E-03
cg13714344	LEPREL2	12	Body	Island	42.17	6.03	45.52	6.35	-3.35	4.61E-04	9.52E-03
cg05044809		5	Intergenic	Open sea	69.29	5.02	72.64	4.58	-3.35	1.93E-03	2.76E-02
cg06913330	NAT8L	4	TSS1500	Island	15.01	3.76	18.36	4.69	-3.35	3.19E-05	1.05E-03
cg11350504		13	Intergenic	N_Shore	77.82	3.96	81.17	3.51	-3.35	2.03E-05	7.09E-04
cg18528640	MSTO1	1	TSS200	Island	20.53	2.66	23.88	4.84	-3.35	2.08E-04	5.10E-03
cg03013070	PLCE1;PLCE1	10	Body;Body	Open sea	26.22	3.65	29.57	4.05	-3.35	5.14E-04	1.03E-02
cg13307384	PRPF40B;PRPF40B	12	5'UTR;1stExon	S_Shore	49.96	3.21	53.31	3.21	-3.35	3.61E-05	1.17E-03
cg05986505	OSR2;OSR2	8	TSS1500;TSS1500	N_Shore	32.71	3.72	36.06	4.28	-3.35	5.11E-04	1.03E-02
cg02273477	NXN	17	Body	Open sea	72.87	5.69	76.22	4.99	-3.35	3.19E-03	3.91E-02
cg15612945	VPS54;VPS54	2	5'UTR;5'UTR	N_Shelf	83.80	5.14	87.15	4.39	-3.35	3.83E-03	4.43E-02
cg22697821		12	Intergenic	Open sea	73.94	5.46	77.29	5.80	-3.35	1.19E-03	1.95E-02
cg14254080	C2orf89	2	Body	N_Shore	41.25	4.83	44.59	4.18	-3.35	2.70E-04	6.27E-03
cg19878076	IGFBP4	17	Body	Open sea	79.95	5.29	83.29	5.68	-3.35	2.22E-03	3.04E-02
cg09317658	KTN1;KTN1;KTN1;KTN1	14	5'UTR;5'UTR;5'UTR;5'UTR	Open sea	82.91	4.77	86.25	3.33	-3.35	1.92E-03	2.75E-02
cg13424878		11	Intergenic	Open sea	51.37	4.19	54.72	5.62	-3.35	1.01E-03	1.72E-02
cg23815702	MUSTN1	3	TSS1500	Open sea	66.04	3.99	69.39	4.21	-3.35	5.49E-04	1.09E-02
cg06010514	SPSB3	16	Body	Island	54.57	3.83	57.92	4.15	-3.35	8.40E-04	1.50E-02
cg09821936	FAM53A	4	3'UTR	N_Shelf	82.98	5.95	86.33	4.75	-3.35	4.56E-03	4.99E-02
cg14167688	GTF2H4	6	TSS1500	N_Shore	55.69	3.49	59.04	4.40	-3.35	1.75E-03	2.58E-02
cg24405291	TNXB	6	Body	S_Shelf	71.32	5.07	74.67	4.61	-3.35	1.99E-04	4.92E-03
cg21504505	C4orf6	4	3'UTR	Open sea	75.83	4.71	79.18	4.39	-3.35	1.06E-03	1.79E-02
cg27204432		7	Intergenic	Open sea	77.68	5.05	81.03	4.24	-3.35	1.01E-03	1.72E-02
cg13583602	GFOD1	6	Body	Open sea	89.13	4.51	92.47	2.16	-3.35	1.60E-04	4.12E-03
cg26875384	FAM107B	10	TSS1500	Open sea	80.27	4.12	83.62	3.59	-3.35	1.15E-04	3.15E-03
cg07395436	LYPLAL1	1	Body	Open sea	80.09	6.18	83.44	6.73	-3.35	2.66E-03	3.44E-02
cg04540061	TEF;TEF	22	Body;Body	N_Shore	81.99	4.35	85.34	3.66	-3.35	2.65E-03	3.44E-02
cg25930962	FRMD5	15	Body	Open sea	70.88	5.08	74.23	6.14	-3.35	2.28E-03	3.09E-02
cg17044306	PARP11	12	TSS1500	S_Shore	67.72	4.34	71.06	4.20	-3.35	1.20E-03	1.96E-02
cg02125104	FER1L6	8	TSS1500	Open sea	71.94	5.26	75.29	4.89	-3.35	1.27E-03	2.05E-02
cg08215400	CD163L1	12	Body	Island	37.02	4.49	40.36	4.23	-3.35	1.33E-03	2.11E-02
cg03390207	CORO6	17	TSS1500	N_Shore	62.97	4.14	66.31	4.61	-3.34	2.33E-04	5.58E-03
cg17996603	NPHP1;NPHP1;NPHP1;NPHP1	2	Body;Body;Body;Body	N_Shelf	84.14	5.99	87.49	5.20	-3.34	3.35E-03	4.04E-02
cg12593746		1	Intergenic	Open sea	27.47	4.54	30.81	4.77	-3.34	1.06E-03	1.78E-02
cg06971092		17	Intergenic	N_Shore	35.68	5.29	39.02	4.63	-3.34	1.20E-04	3.25E-03
cg21350413		6	Intergenic	Open sea	41.34	4.49	44.69	4.22	-3.34	1.67E-03	2.48E-02
cg14515812	RAP1GAP;RAP1GAP;RAP1GAP	1	5'UTR;TSS1500;5'UTR	S_Shore	58.71	5.72	62.06	5.16	-3.34	4.48E-03	4.93E-02
cg13460858		9	Intergenic	Island	37.15	3.81	40.49	4.53	-3.34	3.68E-05	1.19E-03
cg17018096	ADAMTS10	19	Body	Island	24.72	3.77	28.06	3.74	-3.34	6.17E-06	2.42E-04
cg11571942	PPP1R12C	19	Body	S_Shore	61.36	4.03	64.71	3.06	-3.34	2.29E-04	5.51E-03
cg03294424		8	Intergenic	Open sea	36.32	4.76	39.66	3.70	-3.34	5.26E-04	1.05E-02
cg05524038	CSF1R	5	TSS1500	Open sea	75.70	4.50	79.05	5.06	-3.34	4.57E-04	9.45E-03
cg20005415	BICD1;BICD1	12	Body;Body	Open sea	67.26	4.03	70.60	2.98	-3.34	3.38E-04	7.47E-03
cg24692386	CROCC	1	3'UTR	Open sea	77.19	2.57	80.53	2.07	-3.34	4.79E-09	2.59E-07
cg12341202		11	Intergenic	S_Shore	26.80	5.35	30.15	4.78	-3.34	1.85E-03	2.68E-02
cg02056653	PCDHGA4;PCDHGC3;PCDHGA12;PCDH	5	Body;Body;Body;Body;Body;Body;Body;Bo	Open sea	34.92	4.56	38.26	4.27	-3.34	1.56E-03	2.37E-02
cg18082490		1	Intergenic	N_Shelf	78.34	4.12	81.68	3.40	-3.34	3.16E-04	7.08E-03
cg22886575	HMX2	10	3'UTR	Island	27.51	4.06	30.85	4.84	-3.34	1.44E-03	2.24E-02

cg12359158	TCF21;TCF21	6	TSS200;TSS200	N_Shore	40.44	4.28	43.78	4.26	-3.34	6.93E-04	1.30E-02
cg25500444	CCDC81;CCDC81;CCDC81;CCDC81	11	1stExon;5'UTR;1stExon;5'UTR	S_Shore	18.07	3.81	21.41	3.88	-3.34	1.63E-05	5.83E-04
cg07589300		4	Intergenic	Island	64.08	4.06	67.43	4.51	-3.34	1.13E-03	1.88E-02
cg13502215	GABBR2	9	Body	N_Shore	17.52	3.67	20.86	4.29	-3.34	2.53E-04	5.96E-03
cg18606098	NHEDC2	4	TSS1500	S_Shore	84.68	4.29	88.02	3.82	-3.34	9.79E-05	2.75E-03
cg24684348	TCF7L1	2	Body	Open sea	81.55	5.27	84.89	3.53	-3.34	3.72E-03	4.34E-02
cg06993099		2	Intergenic	N_Shelf	55.75	5.50	59.09	5.23	-3.34	1.95E-03	2.77E-02
cg04601957		2	Intergenic	N_Shore	41.49	4.21	44.83	6.63	-3.34	1.79E-04	4.52E-03
cg18147543	ZBTB17	1	5'UTR	Open sea	29.16	4.75	32.51	4.79	-3.34	1.23E-04	3.33E-03
cg01835620	CHRNE	17	TSS1500	S_Shelf	38.51	3.89	41.85	4.21	-3.34	1.83E-03	2.66E-02
cg05710071		1	Intergenic	S_Shelf	75.84	3.42	79.18	3.62	-3.34	2.01E-06	8.63E-05
cg14791639	ATXN1;ATXN1	6	5'UTR;5'UTR	Open sea	69.96	5.10	73.30	4.58	-3.34	1.58E-03	2.39E-02
cg02700328	EPHA4	2	Body	N_Shelf	85.62	5.31	88.96	4.21	-3.34	8.85E-04	1.56E-02
cg12892638		8	Intergenic	Open sea	72.08	3.91	75.42	3.93	-3.34	4.66E-05	1.46E-03
cg03190938	DIRC3	2	TSS1500	S_Shore	14.47	3.33	17.81	4.51	-3.34	1.98E-05	6.93E-04
cg23238719	VWDE	7	Body	Open sea	83.04	4.86	86.38	3.65	-3.34	3.51E-04	7.69E-03
cg19440035	ACER1	19	TSS200	Open sea	69.55	5.12	72.89	4.28	-3.34	8.32E-04	1.49E-02
cg01658815	ABLIM1;ABLIM1	10	Body;Body	Open sea	42.69	5.30	46.03	4.68	-3.34	1.04E-03	1.76E-02
cg16514214	SSH2	17	Body	Open sea	75.74	4.33	79.08	3.28	-3.34	5.76E-05	1.76E-03
cg12024906	HKR1;HKR1	19	1stExon;5'UTR	Island	9.64	3.76	12.98	4.31	-3.34	3.28E-04	7.31E-03
cg02593507	PLIN5	19	TSS1500	S_Shore	41.61	4.55	44.95	4.70	-3.34	1.77E-03	2.59E-02
cg10172584	YJEFN3	19	TSS200	Island	71.94	4.69	75.28	3.94	-3.34	2.75E-03	3.52E-02
cg02453726	PCDH12	5	TSS1500	Open sea	66.33	4.07	69.67	3.47	-3.34	1.60E-04	4.13E-03
cg15693937	LOC728723	5	Body	Open sea	34.02	4.68	37.36	4.73	-3.34	2.80E-03	3.57E-02
cg25592206	CDKN2C;CDKN2C	1	Body;Body	N_Shelf	80.60	5.99	83.94	5.81	-3.34	2.20E-03	3.02E-02
cg25119690		3	Intergenic	S_Shore	41.96	6.05	45.29	6.34	-3.34	3.57E-04	7.79E-03
cg08440690	ETFA;ETFA	15	Body;Body	N_Shelf	84.79	4.45	88.13	5.26	-3.34	1.81E-04	4.56E-03
cg09152866	C6orf105;C6orf105	6	3'UTR;3'UTR	Open sea	70.34	5.33	73.67	4.76	-3.34	3.20E-03	3.91E-02
cg23198262		14	Intergenic	Open sea	63.05	3.77	66.39	3.99	-3.34	1.19E-04	3.22E-03
cg02507501	RASA3	13	Body	S_Shelf	82.80	4.76	86.14	5.38	-3.34	4.28E-04	8.99E-03
cg08071142		15	Intergenic	S_Shelf	58.71	5.31	62.05	6.57	-3.34	4.40E-03	4.87E-02
cg19706472	SLC6A9;SLC6A9;SLC6A9	1	3'UTR;3'UTR;3'UTR	Open sea	79.79	4.28	83.13	3.47	-3.34	1.27E-03	2.04E-02
cg26512635	MAGI2	7	Body	Open sea	77.39	4.15	80.73	3.76	-3.34	9.90E-05	2.78E-03
cg07305514		2	Intergenic	Open sea	49.17	5.37	52.51	4.71	-3.34	2.09E-03	2.91E-02
cg27214960	CYP2E1	10	Body	S_Shore	18.39	6.55	21.72	5.97	-3.34	3.30E-03	4.00E-02
cg09798023	HOXA3	7	5'UTR	N_Shore	57.45	5.69	60.79	5.17	-3.34	2.37E-03	3.19E-02
cg00419117	C1orf69	1	Body	N_Shore	87.56	4.25	90.90	2.83	-3.34	1.16E-04	3.17E-03
cg18195130	EGF	4	TSS200	Open sea	40.01	5.87	43.35	5.51	-3.34	9.46E-04	1.64E-02
cg15844835	FCHSD1	5	TSS200	Island	17.10	5.37	20.44	6.71	-3.34	2.79E-03	3.56E-02
cg26301489	LRRC46	17	Body	S_Shelf	46.64	3.56	49.98	3.02	-3.34	2.54E-05	8.63E-04
cg26667287		22	Intergenic	N_Shelf	67.92	3.71	71.26	3.31	-3.34	9.77E-05	2.75E-03
cg22361413	PBX1	1	Body	Open sea	76.68	4.45	80.02	4.41	-3.34	6.56E-06	2.56E-04
cg22674613	SPATS1	6	TSS200	Open sea	14.97	5.08	18.31	5.71	-3.34	5.37E-05	1.65E-03
cg21755709	PDXK	21	Body	S_Shore	61.80	5.10	65.13	5.56	-3.33	1.29E-03	2.07E-02
cg08701816		8	Intergenic	S_Shelf	65.60	5.28	68.94	4.40	-3.33	1.58E-03	2.39E-02
cg19406367	SGIP1;SGIP1	1	1stExon;5'UTR	S_Shore	51.68	5.40	55.02	3.98	-3.33	3.05E-04	6.89E-03
cg02222761		4	Intergenic	Open sea	65.18	4.49	68.52	3.97	-3.33	2.85E-04	6.54E-03
cg20150443		1	Intergenic	Open sea	66.35	2.84	69.69	3.08	-3.33	4.89E-06	1.96E-04
cg08529825	HIPK1;HIPK1	1	Body;Body	Open sea	60.94	5.76	64.28	6.79	-3.33	1.35E-03	2.14E-02
cg09932730		7	Intergenic	S_Shelf	55.14	6.12	58.47	5.13	-3.33	8.75E-04	1.55E-02
cg16624994	FAM107B	10	1stExon	Open sea	73.45	4.58	76.78	4.34	-3.33	5.53E-04	1.09E-02
cg07716847	ELMO3	16	Body	Island	23.20	3.76	26.53	4.16	-3.33	1.12E-03	1.86E-02
cg25205121	STARD10	11	Body	Open sea	70.58	4.79	73.92	4.38	-3.33	6.09E-04	1.18E-02

cg22341513		18	Intergenic	N_Shelf	61.37	4.33	64.70	4.36	-3.33	1.98E-04	4.90E-03
cg13604166		3	Intergenic	Open sea	51.30	3.95	54.63	4.06	-3.33	9.84E-04	1.69E-02
cg07019315		14	Intergenic	Open sea	21.68	3.40	25.02	4.65	-3.33	1.85E-03	2.68E-02
cg23981150		1	Intergenic	Island	19.71	2.88	23.04	3.30	-3.33	1.03E-05	3.86E-04
cg26896918	C10orf93	10	Body	Open sea	67.00	5.01	70.33	4.66	-3.33	2.36E-03	3.17E-02
cg18778727	PLSCR1	3	Body	Open sea	75.94	4.34	79.27	3.76	-3.33	1.28E-04	3.42E-03
cg11856711	ZNF385A;ZNF385A	12	TSS1500;TSS1500	S_Shore	43.37	6.18	46.71	6.28	-3.33	8.83E-04	1.56E-02
cg21115691	FCAMR;FCAMR;FCAMR;FCAMR	1	Body;Body;Body;Body	Open sea	28.46	5.66	31.80	5.76	-3.33	1.11E-03	1.86E-02
cg02633371	LHX1	17	Body	Island	54.52	4.75	57.85	4.60	-3.33	6.39E-04	1.22E-02
cg07119973	DCAKD;DCAKD	17	Body;Body	N_Shelf	70.39	4.02	73.72	2.94	-3.33	3.29E-05	1.08E-03
cg03331924		1	Intergenic	N_Shelf	73.85	5.49	77.18	4.16	-3.33	2.35E-03	3.16E-02
cg02004632		11	Intergenic	Open sea	46.09	5.09	49.42	6.18	-3.33	6.08E-04	1.18E-02
cg10784341	PHACTR2;PHACTR2	6	TSS200;TSS200	Open sea	64.53	4.39	67.86	4.01	-3.33	1.02E-03	1.74E-02
cg09356020	KLHL18	3	Body	Open sea	73.70	3.97	77.03	3.62	-3.33	3.91E-04	8.36E-03
cg20743720	HIVEP2	6	5'UTR	Open sea	78.09	5.34	81.42	5.61	-3.33	3.77E-04	8.13E-03
cg25528806	OR8D2	11	1stExon	Open sea	44.00	4.93	47.33	5.76	-3.33	7.97E-04	1.44E-02
cg07660473	BEAN	16	5'UTR	Open sea	56.17	4.78	59.50	3.90	-3.33	9.87E-04	1.70E-02
cg12770741	NXN	17	TSS1500	S_Shore	54.51	4.76	57.84	5.02	-3.33	2.05E-03	2.88E-02
cg14308375	PITX2;PITX2;PITX2	4	TSS1500;Body;Body	S_Shore	22.80	3.50	26.13	4.12	-3.33	2.86E-04	6.55E-03
cg03805929	ABCA5;ABCA5	17	5'UTR;TSS1500	Open sea	86.29	3.47	89.62	2.82	-3.33	1.59E-07	7.79E-06
cg03275375		8	Intergenic	Open sea	43.36	4.32	46.69	4.22	-3.33	2.77E-04	6.40E-03
cg19808620	S100A5	1	TSS1500	Open sea	40.47	5.30	43.80	3.41	-3.33	2.35E-03	3.16E-02
cg11444332		10	Intergenic	N_Shelf	25.24	3.97	28.57	4.34	-3.33	3.02E-03	3.76E-02
cg26933107	NR4A1;NR4A1	12	Body;Body	Open sea	83.38	3.54	86.71	2.25	-3.33	5.36E-06	2.13E-04
cg14514716	TANC1;TANC1	2	5'UTR;5'UTR	Open sea	64.66	6.01	67.99	6.07	-3.33	1.09E-03	1.83E-02
cg02524754	HSF2BP	21	Body	N_Shore	74.81	3.32	78.14	3.64	-3.33	2.64E-05	8.93E-04
cg23707091	MCM3APAS	21	Body	S_Shelf	62.19	2.82	65.52	3.23	-3.33	3.13E-06	1.30E-04
cg07842062	MEFV	16	1stExon	S_Shore	66.39	4.30	69.72	4.74	-3.33	2.45E-03	3.26E-02
cg27181013	USP5;USP5;CDCA3	12	TSS1500;TSS1500;Body	N_Shore	54.93	4.10	58.25	3.86	-3.33	8.96E-05	2.56E-03
cg21937554	ALX4	11	TSS1500	N_Shore	44.60	6.53	47.93	7.17	-3.33	4.54E-03	4.97E-02
cg13668339	SUCLG2	3	Body	N_Shore	21.04	3.58	24.37	4.81	-3.33	1.52E-03	2.33E-02
cg26012608		1	Intergenic	Open sea	68.12	4.69	71.45	5.00	-3.33	1.54E-03	2.35E-02
cg06012269	SLC27A1	19	Body	Island	88.97	5.54	92.30	3.59	-3.33	1.69E-03	2.51E-02
cg25037610	UBE2O	17	Body	Open sea	82.56	3.58	85.88	2.71	-3.33	2.05E-05	7.15E-04
cg03987786	LOC100128288	17	TSS1500	Open sea	47.25	5.60	50.58	4.28	-3.33	1.79E-03	2.62E-02
cg16395911	EXOC5	14	Body	Open sea	83.66	5.97	86.99	5.47	-3.33	2.71E-03	3.49E-02
cg07131540	SOLH	16	5'UTR	Island	86.16	4.36	89.49	2.44	-3.33	6.46E-04	1.23E-02
cg16320788	KCNB2	8	Body	Open sea	74.76	5.53	78.09	6.56	-3.33	2.19E-04	5.32E-03
cg00923880		8	Intergenic	N_Shore	66.14	4.76	69.47	4.07	-3.33	1.33E-04	3.53E-03
cg13133560		1	Intergenic	Open sea	47.52	4.63	50.85	4.17	-3.33	1.06E-03	1.79E-02
cg16996144	TCTEX1D1	1	TSS200	N_Shore	39.79	4.86	43.12	5.67	-3.33	8.15E-04	1.47E-02
cg20969061	PI4KAP1	22	TSS1500	Open sea	63.18	4.04	66.51	4.24	-3.33	1.42E-03	2.22E-02
cg16401850		14	Intergenic	Open sea	36.36	2.86	39.69	2.74	-3.33	2.02E-06	8.67E-05
cg24534872	ARMC7	17	Body	Open sea	20.66	5.56	23.99	5.06	-3.33	2.46E-03	3.27E-02
cg26389381	SCEL;SCEL;SCEL	13	TSS1500;TSS1500;TSS1500	Open sea	70.79	4.92	74.11	4.65	-3.33	3.78E-03	4.39E-02
cg04606779	C4orf49	4	TSS200	S_Shore	64.97	5.74	68.30	5.29	-3.33	1.91E-03	2.74E-02
cg16520038	MIR130A	11	TSS200	Open sea	14.94	2.89	18.27	3.69	-3.33	5.31E-05	1.64E-03
cg20412356	FOXK2	17	Body	Open sea	74.99	4.05	78.32	3.30	-3.33	1.02E-04	2.84E-03
cg26876647	RUSC1;RUSC1;C1orf104	1	5'UTR;5'UTR;5'UTR	N_Shore	57.40	4.61	60.73	4.28	-3.33	2.38E-03	3.19E-02
cg03370077	GRB10;GRB10;GRB10	7	5'UTR;TSS1500;TSS1500	Open sea	80.89	4.57	84.22	3.51	-3.33	3.58E-04	7.82E-03
cg19896351	IKBIP;IKBIP;IKBIP	12	Body;Body;Body	N_Shelf	72.15	5.51	75.48	6.04	-3.33	4.04E-03	4.59E-02
cg09545880		2	Intergenic	Open sea	83.77	4.09	87.10	3.49	-3.33	1.33E-05	4.85E-04
cg14093553		14	Intergenic	N_Shore	36.17	4.38	39.50	3.29	-3.33	4.50E-04	9.35E-03

cg15005526	NOTO	2	3'UTR	N_Shelf	79.18	4.12	82.50	4.45	-3.33	1.37E-05	4.99E-04
cg27403071		3	Intergenic	Open sea	76.75	6.38	80.07	4.46	-3.33	1.65E-03	2.47E-02
cg24575485	FASTKD3	5	Body	N_Shelf	81.14	5.13	84.47	4.09	-3.33	7.38E-04	1.36E-02
cg00719439	MYLK2	20	Body	N_Shore	35.85	3.79	39.17	4.33	-3.32	3.83E-04	8.24E-03
cg08890976	UHRF1BP1L;UHRF1BP1L	12	TSS1500;TSS1500	S_Shore	53.61	4.13	56.93	3.23	-3.32	8.01E-04	1.45E-02
cg07200348	NBEA	13	Body	Island	9.66	2.75	12.98	4.82	-3.32	4.45E-05	1.40E-03
cg25605771	SLC6A9;SLC6A9;SLC6A9	1	Body;Body;Body	Open sea	69.73	5.00	73.05	6.26	-3.32	2.91E-03	3.67E-02
cg10952190	PTPN14	1	TSS1500	S_Shore	25.01	5.09	28.34	6.75	-3.32	1.51E-03	2.31E-02
cg09127007		17	Intergenic	Open sea	79.15	4.13	82.47	4.10	-3.32	9.83E-05	2.76E-03
cg14425054		9	Intergenic	S_Shore	37.09	3.79	40.42	4.78	-3.32	1.84E-03	2.66E-02
cg01693598		1	Intergenic	N_Shore	59.76	4.91	63.09	4.55	-3.32	3.95E-03	4.53E-02
cg08048577	TPP2	13	Body	S_Shore	20.95	3.46	24.27	4.47	-3.32	4.30E-04	9.02E-03
cg16601590		13	Intergenic	Open sea	77.69	5.04	81.01	3.84	-3.32	3.48E-03	4.15E-02
cg20143111	CLNK	4	5'UTR	Open sea	47.60	5.19	50.93	4.87	-3.32	3.05E-03	3.79E-02
cg03307717	U2AF1;U2AF1;U2AF1	21	5'UTR;Body;Body	N_Shelf	11.19	4.34	14.51	4.13	-3.32	5.15E-04	1.04E-02
cg13687915	MDGA2;MDGA2	14	Body;Body	Open sea	79.48	5.22	82.80	4.67	-3.32	6.98E-05	2.07E-03
cg17529041		6	Intergenic	N_Shelf	75.45	4.41	78.77	3.55	-3.32	2.19E-04	5.32E-03
cg16232444	GABBR1;GABBR1;GABBR1	6	Body;Body;Body	Open sea	57.70	4.48	61.02	4.35	-3.32	9.82E-04	1.69E-02
cg18756536		11	Intergenic	Open sea	16.76	4.04	20.08	4.39	-3.32	4.92E-05	1.53E-03
cg08040395	ENGASE	17	Body	Open sea	55.54	3.85	58.86	3.90	-3.32	5.88E-04	1.15E-02
cg13134650	CHI3L1	1	TSS1500	Open sea	75.34	3.91	78.66	3.72	-3.32	2.01E-04	4.96E-03
cg23842796		1	Intergenic	S_Shore	12.58	3.43	15.90	4.71	-3.32	1.15E-04	3.13E-03
cg01819707	CROCC	1	TSS200	Open sea	21.15	4.29	24.47	4.64	-3.32	3.57E-04	7.79E-03
cg08276645	ANKMY1;ANKMY1	2	Body;Body	N_Shore	69.92	5.26	73.24	4.37	-3.32	3.98E-04	8.49E-03
cg07247419	NKX2-4	20	3'UTR	Island	28.91	3.93	32.23	4.37	-3.32	1.00E-05	3.77E-04
cg02933639	SH3TC1	4	Body	Open sea	61.33	3.77	64.65	3.47	-3.32	3.23E-04	7.21E-03
cg05921889	BMPER	7	Body	Open sea	26.87	3.96	30.19	4.77	-3.32	3.99E-03	4.56E-02
cg00475322	C7orf20	7	Body	Island	26.50	3.79	29.82	4.74	-3.32	1.12E-03	1.86E-02
cg05289698	GCNT3	15	5'UTR	Open sea	62.60	4.70	65.92	5.77	-3.32	1.49E-03	2.29E-02
cg26311932	LTB;LTB	6	Body;Body	S_Shore	50.17	4.12	53.49	5.27	-3.32	1.43E-04	3.76E-03
cg18975791	ICMT	1	3'UTR	Open sea	81.36	3.75	84.68	3.11	-3.32	4.75E-06	1.91E-04
cg20521277	C8orf75	8	TSS1500	Open sea	70.04	4.41	73.36	3.00	-3.32	4.23E-04	8.90E-03
cg24169822	HOXA4	7	TSS1500	S_Shore	19.85	3.79	23.17	3.34	-3.32	5.94E-05	1.80E-03
cg04031361	EDARADD;EDARADD	1	TSS1500;1stExon	N_Shore	70.15	4.92	73.47	5.28	-3.32	3.19E-04	7.14E-03
cg19639427		1	Intergenic	S_Shelf	65.81	4.56	69.13	4.38	-3.32	1.18E-03	1.94E-02
cg01001791	POLR3H;ACO2;POLR3H;POLR3H	22	3'UTR;3'UTR;3'UTR;3'UTR	Open sea	60.57	4.05	63.89	3.70	-3.32	7.98E-04	1.45E-02
cg13456470	PTCH1;PTCH1;PTCH1;PTCH1;PTCH1;P	9	5'UTR;5'UTR;Body;Body;5'UTR;5'UTR;Bod	N_Shore	44.14	5.60	47.46	5.01	-3.32	2.07E-03	2.90E-02
cg11800844		5	Intergenic	Open sea	48.61	5.52	51.93	5.11	-3.32	3.99E-03	4.56E-02
cg18068561	NNT;NNT	5	TSS1500;TSS1500	N_Shore	46.78	2.97	50.10	3.17	-3.32	6.90E-06	2.68E-04
cg14997582	OSGEPL1	2	3'UTR	Open sea	68.53	5.10	71.85	4.83	-3.32	2.01E-03	2.84E-02
cg09988671		4	Intergenic	S_Shelf	39.58	4.11	42.90	5.07	-3.32	4.02E-04	8.55E-03
cg26378179		14	Intergenic	Open sea	64.24	5.76	67.56	5.34	-3.32	1.51E-03	2.32E-02
cg09573761	GALR3	22	TSS200	N_Shore	69.01	5.33	72.33	4.47	-3.32	3.96E-03	4.54E-02
cg00448191	NKX6-3	8	TSS200	S_Shore	51.11	5.10	54.43	5.72	-3.32	2.59E-03	3.39E-02
cg02051616	NKX2-5;NKX2-5;NKX2-5	5	Body;Body;Body	Island	23.73	3.72	27.05	4.83	-3.32	6.38E-04	1.22E-02
cg19645200		5	Intergenic	N_Shore	78.23	4.15	81.55	3.78	-3.32	5.58E-04	1.10E-02
cg12547166	ZNF876P	4	Body	Island	23.20	3.39	26.52	3.34	-3.32	7.07E-05	2.09E-03
cg06469542	ISM2;ISM2	14	TSS1500;TSS1500	S_Shore	31.15	4.20	34.47	4.61	-3.32	1.76E-03	2.58E-02
cg11152012	SNRPN;SNRPN;SNRPN;SNRPN	15	5'UTR;5'UTR;5'UTR;5'UTR	N_Shelf	47.54	4.03	50.85	4.53	-3.32	3.36E-03	4.05E-02
cg02666820		17	Intergenic	Open sea	78.59	4.77	81.91	5.27	-3.32	3.79E-03	4.40E-02
cg24426405	AGR2	7	TSS200	Open sea	38.35	5.06	41.66	4.71	-3.32	2.40E-03	3.21E-02
cg11170041	GPR113;GPR113;GPR113	2	TSS200;TSS1500;Body	Open sea	70.18	4.14	73.49	4.86	-3.32	7.38E-04	1.36E-02
cg21238376	RPTOR;RPTOR	17	Body;Body	N_Shelf	76.71	3.82	80.02	3.31	-3.32	1.14E-04	3.13E-03

cg11912239	BIRC5;BIRC5;BIRC5;EPR1	17	3'UTR;3'UTR;3'UTR;Body	Open sea	75.95	4.29	79.26	4.01	-3.32	1.66E-04	4.25E-03
cg12639933	SHMT2;SHMT2;SHMT2;SHMT2;SHMT	12	Body;Body;Body;Body;Body;Body;Body;Bo	N_Shelf	48.93	3.35	52.24	3.62	-3.32	3.69E-04	8.01E-03
cg12587213	SRGAP2;SRGAP2;SRGAP2	1	Body;Body;Body	Open sea	78.52	4.32	81.84	3.37	-3.32	3.91E-04	8.36E-03
cg01481879	SNX8	7	Body	N_Shore	82.12	3.01	85.43	2.69	-3.32	9.44E-07	4.25E-05
cg13339930		19	Intergenic	S_Shelf	63.34	3.99	66.66	4.57	-3.32	3.17E-05	1.05E-03
cg14127910		2	Intergenic	N_Shore	19.42	4.42	22.74	5.86	-3.32	3.38E-03	4.07E-02
cg23985941	ZBTB42	14	TSS1500	Island	24.36	4.88	27.67	4.29	-3.32	2.44E-04	5.78E-03
cg06489830		6	Intergenic	N_Shore	28.54	3.83	31.85	5.84	-3.32	2.83E-03	3.60E-02
cg06624368	ZNF629	16	5'UTR	N_Shore	21.71	4.20	25.03	4.67	-3.31	2.86E-03	3.62E-02
cg17522207	NKPD1	19	Body	Island	14.75	4.38	18.07	5.81	-3.31	2.09E-03	2.91E-02
cg00024471		3	Intergenic	Open sea	40.08	3.77	43.40	4.66	-3.31	2.12E-03	2.95E-02
cg25834415	KIF1A	2	5'UTR	Island	16.09	3.38	19.41	3.74	-3.31	2.44E-06	1.03E-04
cg04384638	GDF1;LASS1;LASS1	19	TSS1500;TSS1500;TSS1500	S_Shore	63.37	5.27	66.69	5.42	-3.31	2.80E-03	3.57E-02
cg22035501	APC;APC;APC	5	TSS200;TSS200;5'UTR	Open sea	18.67	6.21	21.99	6.55	-3.31	7.20E-04	1.34E-02
cg19216731	CSRP3;CSRP3	11	TSS200;TSS200	Open sea	60.37	5.12	63.68	3.77	-3.31	6.95E-04	1.30E-02
cg14853406	SLFNL1;SLFNL1	1	TSS1500;TSS1500	Open sea	82.67	4.84	85.98	3.69	-3.31	2.25E-03	3.07E-02
cg03932459	RGL2;RGL2	6	Body;Body	N_Shelf	74.43	3.51	77.74	3.07	-3.31	7.76E-05	2.26E-03
cg19485748		11	Intergenic	Open sea	50.42	6.04	53.73	4.68	-3.31	1.07E-03	1.80E-02
cg16477417	GRB2;GRB2	17	Body;Body	Open sea	29.51	3.84	32.83	4.74	-3.31	1.64E-03	2.45E-02
cg06005892	OSBPL5;OSBPL5;OSBPL5	11	5'UTR;5'UTR;5'UTR	N_Shelf	75.53	5.27	78.84	4.71	-3.31	1.07E-03	1.80E-02
cg03362882		11	Intergenic	N_Shelf	56.95	5.38	60.27	5.36	-3.31	2.88E-03	3.64E-02
cg15852446	PLCB2	15	Body	Island	34.71	2.99	38.02	3.70	-3.31	1.77E-04	4.49E-03
cg05217050	DLGAP4	20	Body	S_Shore	61.41	3.78	64.72	3.49	-3.31	4.37E-04	9.13E-03
cg08721908	ANKRD20B	2	TSS200	Island	44.03	4.49	47.35	4.46	-3.31	1.12E-04	3.07E-03
cg03598159	RYR1;RYR1	19	Body;Body	Island	16.88	4.77	20.19	5.41	-3.31	4.31E-03	4.80E-02
cg22400703	CNRIP1;CNRIP1	2	Body;Body	N_Shore	18.42	4.10	21.73	3.44	-3.31	9.41E-04	1.64E-02
cg06047881	GNAS;GNAS;GNAS;GNAS;GNAS;GNAS	20	3'UTR;TSS1500;Body;TSS1500;3'UTR;TSS1!	Island	30.44	4.21	33.75	4.66	-3.31	1.42E-03	2.21E-02
cg15955907	CCDC85C	14	Body	Open sea	70.25	6.51	73.56	4.30	-3.31	2.20E-03	3.02E-02
cg21171978		22	Intergenic	S_Shelf	21.08	3.40	24.39	4.16	-3.31	9.38E-05	2.65E-03
cg15487867	C1orf70	1	TSS200	N_Shore	6.99	2.66	10.30	3.99	-3.31	2.20E-06	9.39E-05
cg22186694		12	Intergenic	Open sea	33.30	6.06	36.61	5.31	-3.31	2.19E-03	3.01E-02
cg11489638	LOC285205	3	Body	Open sea	76.06	5.66	79.37	4.94	-3.31	2.33E-03	3.15E-02
cg03356494	CLDN10;CLDN10	13	Body;Body	Open sea	62.15	4.14	65.46	4.26	-3.31	1.10E-03	1.84E-02
cg06015439		12	Intergenic	Open sea	51.84	5.31	55.15	5.81	-3.31	1.40E-03	2.20E-02
cg18094414		3	Intergenic	S_Shore	77.39	4.55	80.70	3.91	-3.31	2.82E-04	6.49E-03
cg05105529	SAV1	14	Body	N_Shelf	83.55	4.76	86.86	3.52	-3.31	3.33E-04	7.38E-03
cg16097357	MYO15B	17	Body	Island	26.44	4.74	29.75	5.86	-3.31	2.50E-04	5.90E-03
cg14427590		17	Intergenic	Open sea	60.05	3.43	63.36	3.94	-3.31	2.75E-04	6.36E-03
cg10999122		6	Intergenic	Open sea	62.62	3.30	65.93	3.54	-3.31	3.07E-05	1.02E-03
cg10099957	SLC7A5	16	Body	Open sea	82.99	5.25	86.30	5.84	-3.31	6.85E-04	1.29E-02
cg09265955		1	Intergenic	Island	34.47	3.37	37.78	4.21	-3.31	1.00E-03	1.71E-02
cg02288341	GFRA3;GFRA3	5	1stExon;5'UTR	Island	39.76	6.20	43.07	7.50	-3.31	1.80E-03	2.62E-02
cg10011623	GNAS;GNAS;GNAS;GNAS	20	3'UTR;3'UTR;TSS1500;Body	N_Shore	30.59	5.41	33.90	4.89	-3.31	2.07E-03	2.89E-02
cg25977504	OR14A16	1	TSS200	Open sea	57.80	5.76	61.11	5.25	-3.31	4.29E-03	4.78E-02
cg00448868	DYNC1LI2	16	Body	Open sea	13.97	3.81	17.28	5.33	-3.31	4.05E-03	4.60E-02
cg26117025		1	Intergenic	Open sea	14.99	3.15	18.30	4.18	-3.31	3.30E-06	1.36E-04
cg14347819		8	Intergenic	Open sea	79.17	5.87	82.48	6.14	-3.31	2.18E-03	3.01E-02
cg17098147	SPAG6;SPAG6	10	TSS1500;TSS1500	Island	15.32	3.20	18.63	4.44	-3.31	2.01E-04	4.95E-03
cg01314810		5	Intergenic	N_Shelf	68.48	4.92	71.79	4.74	-3.31	2.28E-03	3.10E-02
cg00714377	SLA2;SLA2	20	TSS200;TSS200	Open sea	44.74	5.34	48.04	4.34	-3.31	2.47E-04	5.85E-03
cg16866941	MAFF;MAFF;MAFF;MAFF	22	5'UTR;5'UTR;5'UTR;5'UTR	S_Shelf	63.98	6.17	67.29	4.54	-3.31	1.96E-03	2.79E-02
cg01511234		12	Intergenic	Open sea	43.25	5.17	46.56	5.04	-3.31	5.01E-04	1.01E-02
cg02304370	PHRF1	11	Body	N_Shore	31.66	5.23	34.97	4.07	-3.31	2.86E-03	3.62E-02

cg26332016	FBXW8;FBXW8	12	TSS1500;TSS1500	N_Shore	69.82	4.41	73.13	3.43	-3.31	1.05E-03	1.77E-02
cg14530834		9	Intergenic	S_Shore	19.98	4.68	23.29	4.41	-3.31	7.81E-04	1.42E-02
cg27302255		12	Intergenic	Open sea	65.96	6.03	69.27	4.58	-3.31	2.35E-03	3.17E-02
cg21663580	PTK7;PTK7;PTK7;PTK7	6	Body;Body;Body;Body	Island	50.43	3.74	53.73	3.77	-3.31	1.04E-05	3.89E-04
cg08293528	CDH23	10	Body	Island	31.21	4.39	34.51	4.22	-3.31	2.61E-04	6.11E-03
cg05857060	HIP1	7	Body	N_Shore	76.92	4.13	80.22	3.24	-3.31	1.07E-04	2.97E-03
cg25832084	AQP6	12	TSS1500	S_Shelf	69.11	3.54	72.41	3.42	-3.31	5.90E-05	1.79E-03
cg08035151	LSM2	6	Body	N_Shelf	69.23	4.92	72.54	3.66	-3.31	8.41E-04	1.50E-02
cg24332570	ANK3	10	Body	Open sea	65.69	5.43	69.00	4.82	-3.31	2.52E-03	3.32E-02
cg13184019	NOTCH4	6	Body	Open sea	71.96	4.02	75.27	3.69	-3.31	1.64E-04	4.21E-03
cg07879070		3	Intergenic	Open sea	69.35	5.56	72.65	5.03	-3.31	1.81E-03	2.63E-02
cg13805654		2	Intergenic	Island	29.79	4.65	33.10	4.68	-3.30	3.55E-03	4.20E-02
cg22299694	KANK2;KANK2	19	Body;Body	Island	76.08	4.94	79.38	3.78	-3.30	5.41E-04	1.08E-02
cg23550704		12	Intergenic	Open sea	77.77	4.39	81.07	4.67	-3.30	1.43E-03	2.22E-02
cg23695990		12	Intergenic	Open sea	50.80	4.77	54.10	5.46	-3.30	3.37E-03	4.06E-02
cg07798774	SCAND3	6	Body	Open sea	73.83	4.63	77.13	4.38	-3.30	1.38E-04	3.65E-03
cg16177922		2	Intergenic	Open sea	38.52	4.48	41.83	5.03	-3.30	2.64E-03	3.43E-02
cg09464461	ZDHHC7;ZDHHC7	16	3'UTR;3'UTR	Open sea	84.26	4.35	87.57	2.89	-3.30	1.16E-03	1.92E-02
cg17220116	SOX13	1	5'UTR	Open sea	80.00	4.86	83.30	3.74	-3.30	4.53E-03	4.97E-02
cg12620499	MREG	2	1stExon	Island	24.61	5.74	27.91	8.34	-3.30	5.84E-04	1.14E-02
cg26492368	SPAG6;SPAG6	10	Body;Body	Island	21.60	4.42	24.90	5.01	-3.30	2.15E-03	2.97E-02
cg22312050		2	Intergenic	S_Shore	89.54	2.40	92.84	1.60	-3.30	4.36E-11	2.54E-09
cg12506373	CAD	2	Body	Island	18.74	4.79	22.05	5.82	-3.30	4.35E-03	4.83E-02
cg25420482	NFKBIL1;NFKBIL1;NFKBIL1;NFKBIL1	6	Body;Body;Body;Body	Open sea	72.24	4.49	75.54	5.41	-3.30	4.67E-04	9.61E-03
cg00920572	HOXC12	12	Body	Island	43.16	3.73	46.47	4.51	-3.30	1.51E-03	2.31E-02
cg20415697	ERMAP	1	5'UTR	S_Shelf	80.69	3.94	83.99	3.20	-3.30	1.98E-05	6.93E-04
cg13447033	AUTS2;AUTS2;AUTS2	7	Body;Body;Body	Open sea	65.63	5.70	68.93	4.75	-3.30	4.47E-03	4.92E-02
cg05650559	SCHIP1	3	Body	Open sea	75.39	4.73	78.70	4.91	-3.30	2.30E-04	5.53E-03
cg01485439	CABP4	11	Body	Open sea	66.56	3.21	69.86	3.47	-3.30	8.33E-06	3.19E-04
cg01852611	DSCR8;DSCR8;DSCR8;DSCR4;DSCR8;D	21	TSS200;TSS200;TSS200;TSS200;TSS200;TS	Open sea	71.70	3.37	75.00	4.29	-3.30	1.14E-04	3.13E-03
cg15128801	GNG12	1	5'UTR	Open sea	39.83	6.86	43.14	6.52	-3.30	2.75E-03	3.52E-02
cg06797617		2	Intergenic	N_Shore	50.67	4.85	53.98	4.64	-3.30	1.86E-03	2.69E-02
cg01560119	SETDB2;SETDB2;SETDB2	13	5'UTR;1stExon;5'UTR	Open sea	84.58	5.04	87.88	4.56	-3.30	1.22E-03	1.98E-02
cg08516507	TNXB	6	Body	Island	20.20	3.68	23.50	4.50	-3.30	5.41E-04	1.08E-02
cg07696485	TRANK1	3	TSS200	Island	11.99	3.28	15.29	4.69	-3.30	3.05E-04	6.89E-03
cg19939178	BTBD11;BTBD11	12	Body;Body	S_Shore	80.38	4.05	83.68	3.19	-3.30	1.96E-05	6.87E-04
cg14012059	LRSAM1;LRSAM1;LRSAM1	9	Body;Body;Body	Open sea	65.95	4.45	69.25	4.30	-3.30	1.94E-03	2.77E-02
cg07971716		1	Intergenic	Open sea	72.32	5.63	75.62	5.56	-3.30	1.45E-03	2.25E-02
cg11977488		1	Intergenic	N_Shore	67.50	4.46	70.80	5.44	-3.30	1.96E-03	2.79E-02
cg18126097	NR5A2;NR5A2	1	Body;Body	S_Shelf	69.66	4.84	72.96	5.96	-3.30	8.57E-04	1.53E-02
cg12844117		7	Intergenic	Open sea	73.88	5.21	77.18	4.18	-3.30	8.42E-04	1.50E-02
cg12598837	HTR3A;HTR3A	11	TSS200;TSS200	Open sea	30.86	4.30	34.16	3.88	-3.30	1.53E-03	2.34E-02
cg12259522		13	Intergenic	Open sea	43.99	4.08	47.29	4.29	-3.30	8.34E-05	2.40E-03
cg00067414	MTHFD1L	6	Body	Island	49.58	4.86	52.88	5.09	-3.30	1.08E-04	2.98E-03
cg18708217		10	Intergenic	N_Shore	66.33	4.50	69.63	5.19	-3.30	7.14E-04	1.33E-02
cg23173307	CPLX1	4	Body	Island	14.57	4.38	17.87	5.81	-3.30	3.93E-04	8.39E-03
cg00547365	NOL9	1	Body	Open sea	74.15	4.00	77.45	3.50	-3.30	4.70E-05	1.47E-03
cg27228191		1	Intergenic	S_Shore	61.80	3.65	65.11	3.47	-3.30	2.22E-04	5.38E-03
cg19305488	APC2	19	Body	Island	21.12	3.01	24.42	3.77	-3.30	1.38E-07	6.82E-06
cg12176856	PDE8A;PDE8A	15	Body;Body	Open sea	83.58	5.78	86.87	4.63	-3.30	3.10E-03	3.83E-02
cg01797727	CYP27A1	2	TSS1500	N_Shore	48.73	3.89	52.03	3.56	-3.30	1.92E-04	4.78E-03
cg24417499	HPCA	1	TSS1500	N_Shore	29.69	5.04	32.99	4.49	-3.30	1.89E-03	2.72E-02
cg04987071		16	Intergenic	Open sea	76.36	6.06	79.66	4.72	-3.30	2.79E-03	3.56E-02

cg22516105	BTBD7;BTBD7;BTBD7;KIAA1409;BTBD	14	5'UTR;1stExon;5'UTR;TSS1500;1stExon	Island	26.58	3.90	29.88	3.92	-3.30	1.31E-04	3.50E-03
cg07770049		4	Intergenic	N_Shore	57.85	3.43	61.15	3.43	-3.30	8.87E-05	2.54E-03
cg08446111	LONP2	16	Body	S_Shore	18.52	3.96	21.82	4.64	-3.30	4.08E-03	4.62E-02
cg05760722	SCN8A	12	Body	N_Shore	50.81	5.73	54.11	5.55	-3.30	4.02E-03	4.58E-02
cg18663886	TBC1D23	3	Body	Open sea	79.95	5.70	83.25	4.90	-3.30	2.07E-03	2.90E-02
cg02379560	APBB2;APBB2;APBB2;APBB2;APBB2;A	4	Body;TSS200;TSS200;Body;TSS200;Body;T	S_Shore	51.35	7.08	54.65	6.72	-3.30	1.32E-03	2.11E-02
cg12505390	RNF167	17	3'UTR	N_Shelf	24.57	3.16	27.87	3.52	-3.30	3.15E-06	1.30E-04
cg03460777		10	Intergenic	S_Shelf	59.02	4.38	62.32	4.16	-3.30	1.14E-03	1.89E-02
cg00425918	NKX2-6	8	Body	Island	17.65	2.69	20.94	3.63	-3.30	2.61E-05	8.83E-04
cg07390023		13	Intergenic	Island	58.35	3.82	61.64	4.09	-3.30	2.63E-04	6.15E-03
cg06200634	H2AFX	11	TSS1500	S_Shore	30.59	4.73	33.89	4.86	-3.30	7.03E-04	1.32E-02
cg00852534	TTBK1	6	5'UTR	N_Shore	49.10	3.74	52.40	4.54	-3.30	1.75E-04	4.45E-03
cg15873301	SYN2;SYN2	3	TSS1500;TSS1500	N_Shore	20.09	5.52	23.39	4.84	-3.30	7.61E-04	1.40E-02
cg07745344		8	Intergenic	Open sea	66.26	4.71	69.56	4.67	-3.30	3.43E-03	4.11E-02
cg10585948	HOXB4;HOXB3	17	3'UTR;TSS1500	N_Shore	37.20	3.75	40.49	4.68	-3.30	2.15E-03	2.97E-02
cg01071811	L3MBTL;L3MBTL;L3MBTL;L3MBTL	20	5'UTR;1stExon;1stExon;5'UTR	N_Shore	52.60	4.98	55.90	4.77	-3.30	7.85E-04	1.43E-02
cg07762961	NLRC3	16	5'UTR	S_Shelf	47.49	4.17	50.79	3.99	-3.30	4.91E-04	9.98E-03
cg23914149		10	Intergenic	Open sea	72.54	3.89	75.83	4.10	-3.30	4.21E-04	8.86E-03
cg16038120	FOXG1	14	TSS1500	Island	21.06	3.70	24.36	4.04	-3.29	1.77E-05	6.26E-04
cg17320403		2	Intergenic	Open sea	59.69	5.09	62.98	4.84	-3.29	2.16E-03	2.98E-02
cg26402405	AFAP1L1;AFAP1L1	5	Body;Body	Open sea	71.26	4.59	74.55	5.30	-3.29	3.10E-03	3.83E-02
cg18649737	WDR20	14	Body	Island	81.45	4.91	84.75	3.23	-3.29	3.49E-03	4.16E-02
cg09494876		5	Intergenic	Open sea	68.00	4.95	71.29	3.72	-3.29	1.32E-03	2.11E-02
cg15224741	TMEM41A	3	3'UTR	Open sea	78.04	3.74	81.33	4.31	-3.29	4.16E-04	8.78E-03
cg11823736	IDO2	8	TSS200	Open sea	77.64	4.30	80.93	4.07	-3.29	1.49E-04	3.89E-03
cg04034458	C15orf23;C15orf23;C15orf23	15	TSS1500;TSS1500;TSS1500	N_Shore	60.77	4.17	64.06	4.21	-3.29	4.86E-04	9.90E-03
cg08324906	PVRL1;PVRL1;PVRL1	11	Body;Body;3'UTR	Open sea	84.34	3.64	87.63	2.86	-3.29	1.57E-05	5.64E-04
cg24085344	PHB	17	Body	Open sea	47.16	6.12	50.45	5.41	-3.29	1.83E-03	2.66E-02
cg03905757	KCNQ1;KCNQ1	11	Body;Body	Open sea	78.60	4.19	81.89	4.83	-3.29	1.32E-04	3.52E-03
cg05849319	FRMD8	11	Body	Island	79.58	3.26	82.87	2.80	-3.29	2.00E-05	6.99E-04
cg25830692	ASRGL1;ASRGL1	11	Body;Body	Open sea	79.77	5.97	83.06	6.27	-3.29	3.41E-03	4.09E-02
cg03957687	CENPT	16	Body	N_Shelf	66.54	4.88	69.84	4.25	-3.29	1.74E-03	2.57E-02
cg20921405		12	Intergenic	Open sea	83.65	3.21	86.94	2.71	-3.29	2.45E-06	1.03E-04
cg15105060	TBC1D14;TBC1D14;TBC1D14	4	Body;Body;Body	N_Shelf	82.02	5.91	85.31	5.07	-3.29	4.46E-03	4.91E-02
cg21633669	SPTBN1;SPTBN1	2	Body;Body	Open sea	63.25	6.01	66.54	6.09	-3.29	2.16E-04	5.24E-03
cg11947985	TMEM63B	6	Body	Island	35.57	4.04	38.86	5.13	-3.29	3.74E-03	4.36E-02
cg03193299	LOC100130872-SPON2;SPON2;SPON2	4	Body;TSS1500;TSS1500	S_Shore	39.06	3.58	42.36	4.26	-3.29	2.50E-03	3.31E-02
cg15254559		7	Intergenic	Island	18.62	2.74	21.91	3.36	-3.29	9.78E-07	4.40E-05
cg24465935		6	Intergenic	Open sea	61.17	5.78	64.46	5.92	-3.29	6.36E-04	1.22E-02
cg01987925	PPP2R2B;PPP2R2B	5	TSS200;TSS200	Open sea	37.37	4.68	40.66	4.30	-3.29	4.56E-04	9.44E-03
cg02593766	MYCBPAP;EPN3	17	Body;TSS1500	Open sea	81.50	4.00	84.79	3.12	-3.29	1.33E-05	4.88E-04
cg15028548	ABI3BP;ABI3BP	3	5'UTR;1stExon	Open sea	18.86	3.91	22.15	3.97	-3.29	1.42E-04	3.74E-03
cg24767968	HOXB3	17	TSS200	N_Shelf	28.35	3.61	31.64	4.90	-3.29	2.00E-03	2.83E-02
cg02943305	CCDC129	7	Body	Open sea	30.43	4.78	33.72	4.18	-3.29	6.04E-04	1.17E-02
cg22654656		4	Intergenic	N_Shelf	78.42	5.64	81.71	6.14	-3.29	2.27E-03	3.08E-02
cg23712462	FAM135B	8	TSS1500	S_Shore	55.19	4.21	58.48	4.45	-3.29	2.20E-04	5.33E-03
cg01427957	TNRC4	1	TSS1500	Open sea	27.31	4.92	30.60	4.46	-3.29	7.04E-04	1.32E-02
cg23677657	DLX6AS	7	Body	Open sea	27.70	3.43	30.98	4.11	-3.29	1.93E-05	6.77E-04
cg00965203		16	Intergenic	Open sea	77.40	4.06	80.69	3.19	-3.29	2.19E-04	5.30E-03
cg14384748	GALNT9	12	Body	Island	37.26	5.32	40.55	5.31	-3.29	2.30E-03	3.12E-02
cg16133244	C16orf42;GNPTG	16	Body;TSS1500	N_Shore	70.42	4.89	73.71	4.01	-3.29	1.44E-03	2.24E-02
cg05862356		2	Intergenic	Island	15.23	2.73	18.51	3.16	-3.29	7.78E-08	3.91E-06
cg10607455	PCSK1	5	Body	N_Shore	38.76	5.91	42.05	6.31	-3.29	2.09E-03	2.92E-02

cg26312071	SEMA4B	15	TSS1500	N_Shore	20.37	4.16	23.66	4.68	-3.29	2.75E-03	3.53E-02
cg19306847	BRMS1;BRMS1	11	5'UTR;5'UTR	N_Shore	24.85	4.89	28.14	5.10	-3.29	2.50E-03	3.30E-02
cg16705395		2	Intergenic	Open sea	68.02	6.08	71.31	4.35	-3.29	3.06E-03	3.79E-02
cg01378812	DDX52;DDX52	17	TSS1500;TSS1500	S_Shore	79.48	4.24	82.77	3.49	-3.29	5.28E-04	1.06E-02
cg08933409		10	Intergenic	Island	46.59	4.86	49.88	3.80	-3.29	3.84E-04	8.24E-03
cg10039160	NFASC;NFASC;NFASC;NFASC	1	Body;Body;Body;Body	Open sea	79.40	3.83	82.69	3.20	-3.29	1.89E-04	4.73E-03
cg00236576	METRNL	17	Body	Island	41.02	3.69	44.31	5.65	-3.29	1.71E-04	4.37E-03
cg17145370		2	Intergenic	N_Shore	25.89	4.59	29.18	4.27	-3.29	6.11E-04	1.18E-02
cg07471614		8	Intergenic	Open sea	65.61	3.67	68.90	3.98	-3.29	1.85E-04	4.65E-03
cg17915961	LDB1	10	TSS1500	S_Shore	62.63	5.68	65.92	3.97	-3.29	3.37E-03	4.06E-02
cg14231297	ZSCAN18;ZSCAN18	19	TSS200;TSS200	Island	17.23	3.82	20.52	4.05	-3.29	7.42E-04	1.37E-02
cg08753468		7	Intergenic	Open sea	51.16	4.08	54.45	4.21	-3.29	1.57E-03	2.38E-02
cg12782180	LEP	7	TSS1500	Island	35.61	4.57	38.90	5.26	-3.29	3.93E-03	4.51E-02
cg16947612	EFEMP1;EFEMP1;EFEMP1	2	Body;Body;Body	Open sea	62.66	4.66	65.94	4.17	-3.29	1.95E-03	2.77E-02
cg02375065	PLEKHG4;PLEKHG4;PLEKHG4;PLEKHG4	16	Body;Body;Body;Body;Body	S_Shelf	70.77	5.04	74.06	4.76	-3.29	2.95E-03	3.70E-02
cg02264229		14	Intergenic	S_Shore	55.60	4.56	58.89	4.89	-3.29	2.11E-03	2.93E-02
cg12140755		1	Intergenic	Open sea	60.35	4.65	63.63	3.55	-3.29	1.33E-04	3.55E-03
cg26538224		22	Intergenic	N_Shelf	77.28	4.25	80.56	3.95	-3.29	3.52E-04	7.71E-03
cg12793610	TRIM2;TRIM2;TRIM2	4	5'UTR;1stExon;5'UTR	Open sea	41.88	6.41	45.16	5.13	-3.29	3.08E-03	3.81E-02
cg11726150	SLC44A4	6	Body	Open sea	64.65	3.79	67.93	3.31	-3.29	1.09E-04	3.00E-03
cg07400877		20	Intergenic	Island	23.59	6.03	26.87	6.06	-3.29	2.89E-03	3.64E-02
cg14510333		16	Intergenic	Island	15.36	4.09	18.64	5.23	-3.29	2.33E-03	3.15E-02
cg13817395	TNXB;TNXB	6	Body;TSS1500	Open sea	65.10	4.79	68.38	4.61	-3.28	1.43E-03	2.23E-02
cg21032395		3	Intergenic	S_Shelf	77.21	4.85	80.49	3.48	-3.28	1.14E-03	1.89E-02
cg03822198	MAP1LC3B2	12	5'UTR	Open sea	39.33	5.53	42.61	5.28	-3.28	4.07E-04	8.64E-03
cg22820921	PEG10;PEG10	7	5'UTR;5'UTR	S_Shore	29.55	3.33	32.83	3.57	-3.28	9.67E-05	2.72E-03
cg17004353	CARS;CARS;CARS;CARS	11	Body;Body;5'UTR;Body	Open sea	63.27	4.73	66.56	4.61	-3.28	1.33E-03	2.11E-02
cg02377915	PPM1F	22	TSS1500	S_Shore	54.11	4.76	57.40	5.03	-3.28	3.70E-03	4.33E-02
cg00373148	GNAT1;GNAT1	3	TSS1500;TSS1500	N_Shelf	83.08	3.87	86.36	3.38	-3.28	2.32E-04	5.57E-03
cg03743720	ANKMY1;ANKMY1	2	Body;Body	Island	44.78	4.10	48.06	4.55	-3.28	2.33E-04	5.59E-03
cg03506216		8	Intergenic	Island	17.57	4.71	20.86	6.08	-3.28	1.85E-04	4.64E-03
cg04804772	CALHM3;CALHM3	10	5'UTR;1stExon	Open sea	76.55	5.66	79.84	5.15	-3.28	1.93E-03	2.75E-02
cg10557578	RAB11FIP1;RAB11FIP1	8	TSS1500;TSS1500	S_Shore	73.46	4.38	76.74	4.14	-3.28	2.55E-04	6.00E-03
cg06107487	NCKAP5;NCKAP5	2	Body;Body	Open sea	71.75	3.59	75.03	3.19	-3.28	7.06E-05	2.09E-03
cg07379055		19	Intergenic	Island	27.45	4.85	30.74	4.25	-3.28	2.15E-03	2.97E-02
cg00766220		5	Intergenic	N_Shore	21.01	3.99	24.29	4.16	-3.28	6.48E-04	1.24E-02
cg21820610		1	Intergenic	S_Shore	76.46	4.50	79.74	4.60	-3.28	4.73E-04	9.70E-03
cg16423811	UTS2R	17	TSS1500	N_Shore	46.63	4.95	49.92	5.22	-3.28	2.38E-03	3.19E-02
cg13916117	CNTN4;CNTN4;CNTN4	3	Body;TSS1500;Body	Open sea	74.40	4.76	77.68	3.52	-3.28	2.97E-04	6.75E-03
cg11375547	C19orf51;TNNI3	19	Body;TSS1500	N_Shore	73.21	4.33	76.49	4.13	-3.28	1.17E-04	3.19E-03
cg05492839	ELOVL4	6	TSS200	Island	12.29	3.06	15.57	3.07	-3.28	1.61E-08	8.47E-07
cg05348366		1	Intergenic	Island	21.99	3.62	25.27	4.56	-3.28	2.28E-03	3.09E-02
cg04025498		6	Intergenic	N_Shelf	73.72	2.83	77.00	2.61	-3.28	1.67E-06	7.24E-05
cg24655222	ZNF513	2	Body	Island	83.56	4.78	86.84	4.08	-3.28	1.82E-03	2.65E-02
cg02507604	CHFR;CHFR;CHFR;CHFR;CHFR	12	3'UTR;3'UTR;3'UTR;3'UTR;3'UTR	Island	70.82	4.93	74.10	4.55	-3.28	3.19E-03	3.91E-02
cg10658240	UBE2E3;UBE2E3	2	Body;Body	Open sea	82.63	5.64	85.91	5.05	-3.28	2.62E-03	3.41E-02
cg20532370	RBP1;RBP1;RBP1	3	TSS200;TSS200;TSS200	Island	8.38	2.55	11.66	3.14	-3.28	3.65E-09	1.99E-07
cg03604930	NEDD4L;NEDD4L;NEDD4L;NEDD4L;NE	18	5'UTR;Body;Body;Body;5'UTR;5'UTR;Body	Open sea	80.32	4.20	83.60	3.84	-3.28	2.77E-04	6.39E-03
cg05013912		16	Intergenic	Open sea	72.75	5.50	76.03	4.44	-3.28	3.06E-03	3.80E-02
cg09666532	B4GALT2;B4GALT2	1	5'UTR;5'UTR	S_Shore	64.27	3.96	67.55	4.16	-3.28	9.27E-05	2.63E-03
cg14530679	NUDT9;NUDT9	4	Body;Body	Open sea	40.41	4.46	43.69	4.67	-3.28	2.13E-04	5.20E-03
cg05481929	POLR2C	16	3'UTR	Open sea	44.19	4.72	47.47	5.14	-3.28	1.38E-03	2.17E-02
cg26634121	GALNS	16	Body	N_Shore	64.71	4.57	67.99	4.17	-3.28	3.28E-03	3.98E-02

cg16065922		7	Intergenic	Open sea	30.22	4.83	33.50	5.79	-3.28	1.30E-03	2.08E-02
cg03575122	UBR5	8	Body	Open sea	65.02	4.71	68.30	4.01	-3.28	7.32E-04	1.36E-02
cg03576863	TERF2	16	Body	N_Shelf	50.26	4.47	53.54	3.80	-3.28	1.22E-03	1.99E-02
cg25959474	FOLR2;FOLR2;FOLR2;FOLR2	11	TSS1500;TSS1500;TSS1500;TSS1500	Open sea	76.82	5.21	80.10	4.61	-3.28	3.90E-03	4.49E-02
cg00088989	SNRNP200	2	Body	N_Shelf	79.97	5.34	83.25	5.54	-3.28	1.71E-03	2.52E-02
cg01422797	PRDM13	6	TSS1500	N_Shore	21.07	3.22	24.35	4.07	-3.28	5.36E-05	1.65E-03
cg23032421	IL5RA;IL5RA;IL5RA;IL5RA;IL5RA;IL5RA	3	5'UTR;5'UTR;1stExon;5'UTR;1stExon;1stEx	Open sea	23.44	3.40	26.72	3.30	-3.28	3.53E-05	1.15E-03
cg11666857	SLC6A19	5	Body	Island	75.75	5.54	79.02	5.11	-3.28	1.60E-03	2.41E-02
cg24415154	LRP1	12	Body	Open sea	78.09	3.61	81.36	3.60	-3.28	1.24E-04	3.35E-03
cg04230060	SUSD1	9	TSS1500	S_Shore	51.62	4.56	54.89	4.79	-3.28	3.13E-03	3.85E-02
cg20064422		14	Intergenic	S_Shore	61.32	3.38	64.60	3.72	-3.28	2.59E-05	8.78E-04
cg24770230	AP3S1	5	Body	Open sea	73.99	5.66	77.27	5.26	-3.28	4.43E-03	4.89E-02
cg07326149		15	Intergenic	Open sea	63.41	5.62	66.68	4.80	-3.28	4.47E-03	4.93E-02
cg26885268		7	Intergenic	Island	23.74	4.09	27.02	4.40	-3.28	6.72E-04	1.27E-02
cg15057995		12	Intergenic	Open sea	73.36	4.70	76.64	6.05	-3.28	2.06E-03	2.89E-02
cg05201970	HCN1;HCN1	5	5'UTR;1stExon	Island	17.66	3.36	20.94	3.76	-3.28	2.00E-05	7.00E-04
cg15538767		5	Intergenic	Open sea	52.60	4.08	55.87	3.56	-3.28	1.20E-03	1.96E-02
cg21340821		2	Intergenic	Open sea	50.00	4.27	53.27	3.71	-3.28	2.44E-04	5.78E-03
cg13127421	SAMD14	17	Body	Open sea	83.53	5.37	86.81	5.22	-3.28	1.70E-03	2.52E-02
cg06688770	TMEM90A	14	5'UTR	N_Shore	19.33	3.96	22.61	4.15	-3.28	6.27E-04	1.21E-02
cg08726417	MYLK2	20	TSS200	N_Shelf	51.12	4.39	54.40	4.37	-3.28	2.01E-03	2.84E-02
cg15960928	TROAP;TROAP	12	Body;Body	S_Shore	11.33	2.40	14.61	2.62	-3.28	1.53E-08	8.08E-07
cg26524899	AKAP5	14	5'UTR	S_Shore	14.28	3.62	17.55	3.67	-3.28	3.96E-05	1.27E-03
cg08269316	HMGB2;HMGB2;HMGB2	4	Body;Body;Body	N_Shore	17.46	4.05	20.73	5.83	-3.27	3.25E-04	7.24E-03
cg18759102	PRDM16;PRDM16	1	Body;Body	N_Shelf	39.01	5.05	42.28	5.05	-3.27	4.45E-03	4.91E-02
cg09553242		5	Intergenic	N_Shore	20.81	3.67	24.09	3.53	-3.27	2.51E-04	5.93E-03
cg04897900		3	Intergenic	Open sea	77.99	3.99	81.27	3.94	-3.27	9.24E-05	2.62E-03
cg11229497	TRIM31	6	Body	Open sea	56.57	3.74	59.85	3.33	-3.27	7.71E-05	2.25E-03
cg16083711	GRIN2B	12	5'UTR	Open sea	76.31	4.48	79.58	3.50	-3.27	3.42E-04	7.54E-03
cg15393903	GRID1	10	Body	Open sea	82.29	5.84	85.57	5.77	-3.27	2.72E-03	3.50E-02
cg07083902		6	Intergenic	N_Shelf	41.78	4.63	45.06	4.34	-3.27	3.27E-03	3.97E-02
cg15649236	MIR10A	17	TSS200	N_Shore	27.95	3.67	31.22	3.91	-3.27	1.24E-04	3.34E-03
cg10274022	PLD3;PLD3	19	5'UTR;5'UTR	Island	41.21	4.93	44.49	5.67	-3.27	1.94E-03	2.76E-02
cg06356696	SLITRK3	3	3'UTR	Open sea	79.91	5.06	83.18	4.04	-3.27	1.67E-03	2.48E-02
cg24385322	RELL2;RELL2;HDAC3;RELL2	5	5'UTR;1stExon;TSS1500;5'UTR	S_Shore	29.90	5.24	33.17	4.62	-3.27	1.31E-04	3.51E-03
cg10206052	CPSF3L	1	Body	N_Shelf	84.07	5.62	87.34	2.58	-3.27	4.49E-04	9.32E-03
cg12319602		5	Intergenic	Island	18.95	4.06	22.22	4.60	-3.27	7.20E-05	2.12E-03
cg18070504	HRH1	3	5'UTR	Open sea	64.67	4.83	67.94	3.70	-3.27	3.00E-03	3.74E-02
cg00236832	RARA;RARA	17	5'UTR;1stExon	Open sea	16.59	5.30	19.86	5.44	-3.27	4.82E-04	9.85E-03
cg01300684	SCOC	4	5'UTR	Open sea	55.44	3.97	58.72	3.67	-3.27	1.40E-05	5.10E-04
cg07496078		9	Intergenic	N_Shore	67.84	4.47	71.12	4.31	-3.27	6.42E-04	1.23E-02
cg01441945	CHERP	19	Body	Island	82.56	4.89	85.84	3.80	-3.27	7.76E-04	1.42E-02
cg10998570		5	Intergenic	Open sea	81.09	4.60	84.37	3.99	-3.27	4.45E-03	4.91E-02
cg18508425		19	Intergenic	S_Shelf	68.92	4.85	72.19	4.30	-3.27	2.76E-03	3.53E-02
cg20602244	FGF14	13	Body	S_Shore	83.04	4.81	86.31	3.86	-3.27	2.72E-03	3.50E-02
cg26896255	DNM3;DNM3	1	Body;Body	Open sea	82.67	5.02	85.94	4.05	-3.27	8.24E-04	1.48E-02
cg10808367	INPP5A	10	Body	Open sea	73.08	4.06	76.35	4.06	-3.27	2.12E-04	5.17E-03
cg14684088	ZCCHC24	10	Body	Open sea	72.41	4.32	75.68	2.92	-3.27	1.05E-03	1.78E-02
cg19377674	DIS3L;DIS3L	15	Body;Body	S_Shelf	74.53	4.21	77.80	3.89	-3.27	5.62E-04	1.11E-02
cg06062945	SUPT7L	2	Body	N_Shelf	79.03	5.61	82.30	5.19	-3.27	3.52E-03	4.18E-02
cg20313873	ENTPD7	10	Body	Open sea	69.91	5.85	73.18	4.47	-3.27	9.59E-04	1.66E-02
cg02092276	COL6A6	3	3'UTR	Open sea	87.94	5.99	91.21	3.98	-3.27	1.49E-03	2.29E-02
cg23473276		7	Intergenic	Open sea	82.40	5.70	85.67	5.22	-3.27	1.91E-03	2.74E-02

cg10376838		4	Intergenic	Open sea	81.86	4.34	85.13	4.80	-3.27	7.56E-04	1.39E-02
cg24026146	SEMA6A	5	Body	S_Shelf	82.02	4.74	85.29	3.29	-3.27	5.77E-04	1.13E-02
cg08889687		3	Intergenic	N_Shore	33.30	5.78	36.57	6.03	-3.27	3.60E-03	4.24E-02
cg22721579	FAM176B	1	Body	Island	57.96	5.29	61.23	5.15	-3.27	3.48E-03	4.15E-02
cg23398487		13	Intergenic	Open sea	37.48	3.40	40.75	4.72	-3.27	1.19E-03	1.95E-02
cg18943317		11	Intergenic	Open sea	74.77	4.12	78.04	3.20	-3.27	1.01E-04	2.83E-03
cg00123090		1	Intergenic	Open sea	62.69	3.95	65.96	3.85	-3.27	2.77E-04	6.39E-03
cg00314003		11	Intergenic	S_Shore	51.73	5.84	55.00	6.67	-3.27	1.83E-03	2.66E-02
cg15808924	P2RX3	11	Body	Island	45.80	4.65	49.07	5.29	-3.27	2.08E-03	2.90E-02
cg26026748	PBX1	1	Body	Open sea	59.47	6.00	62.74	6.96	-3.27	2.27E-04	5.47E-03
cg04923020		12	Intergenic	Open sea	25.94	3.94	29.21	4.24	-3.27	1.22E-03	1.99E-02
cg06073695		6	Intergenic	Open sea	79.57	4.38	82.83	3.76	-3.27	3.16E-04	7.09E-03
cg27517681	TBX15	1	5'UTR	N_Shore	29.35	3.41	32.62	3.41	-3.27	8.33E-05	2.40E-03
cg12545687	PCDH8;PCDH8	13	TSS1500;TSS1500	N_Shore	13.91	3.73	17.18	4.62	-3.27	4.47E-04	9.29E-03
cg17676618		10	Intergenic	Open sea	62.57	3.93	65.84	3.99	-3.27	1.52E-03	2.33E-02
cg00552753		17	Intergenic	S_Shelf	82.81	4.09	86.08	3.17	-3.27	2.32E-04	5.56E-03
cg07794409		1	Intergenic	N_Shore	45.12	4.49	48.39	4.72	-3.27	4.15E-05	1.32E-03
cg23960736		1	Intergenic	Open sea	54.95	5.33	58.22	4.58	-3.27	1.55E-03	2.36E-02
cg17813310	AFF1;AFF1	4	Body;Body	Open sea	87.47	3.57	90.73	2.27	-3.27	1.71E-05	6.07E-04
cg19035788		2	Intergenic	S_Shelf	71.95	5.06	75.22	3.99	-3.27	3.88E-03	4.47E-02
cg15378445	SIPA1L1	14	Body	Open sea	66.25	3.65	69.52	3.94	-3.27	2.52E-04	5.93E-03
cg13811315		2	Intergenic	Open sea	80.38	3.19	83.65	1.93	-3.27	1.76E-06	7.60E-05
cg02909446	SPOCK2;SPOCK2	10	TSS1500;TSS1500	S_Shore	39.28	4.44	42.55	4.40	-3.27	3.66E-03	4.29E-02
cg00952050	MYH7B	20	Body	Island	71.17	5.13	74.44	4.22	-3.27	1.06E-03	1.78E-02
cg12848808	LBXCOR1	15	Body	N_Shore	30.62	3.90	33.89	4.81	-3.27	2.62E-04	6.12E-03
cg14473568	ROBO2	3	TSS1500	Island	22.28	3.37	25.54	4.48	-3.27	5.78E-04	1.13E-02
cg10573521	HNF1A	12	3'UTR	Open sea	80.92	4.46	84.18	3.85	-3.27	1.23E-03	2.00E-02
cg19092735	KRT80;KRT80	12	Body;Body	Open sea	70.18	4.81	73.45	4.58	-3.27	8.11E-04	1.46E-02
cg19847283		19	Intergenic	Open sea	12.97	3.25	16.23	4.44	-3.27	6.15E-05	1.86E-03
cg05376937	CNIH3	1	Body	Open sea	40.08	5.48	43.35	6.59	-3.27	3.70E-03	4.33E-02
cg21124175	ZNF835	19	Body	Island	83.68	4.77	86.95	3.06	-3.27	1.07E-03	1.80E-02
cg10983484	LYSMD2;LYSMD2	15	Body;5'UTR	Open sea	53.91	8.06	57.18	6.90	-3.27	1.54E-03	2.34E-02
cg18067514		5	Intergenic	S_Shelf	80.98	5.71	84.25	3.86	-3.27	3.73E-03	4.35E-02
cg21477176	CACNG7	19	3'UTR	S_Shore	22.54	4.19	25.80	5.21	-3.27	2.35E-03	3.17E-02
cg26790897	SUMF2;SUMF2;SUMF2;SUMF2;SUMF	7	3'UTR;3'UTR;3'UTR;3'UTR;3'UTR	Open sea	25.12	3.81	28.39	3.87	-3.27	1.37E-03	2.16E-02
cg00015261		16	Intergenic	Open sea	53.24	4.41	56.51	4.40	-3.27	1.47E-03	2.27E-02
cg20944379	IL24;IL24	1	TSS1500;TSS1500	Open sea	53.11	5.61	56.37	5.25	-3.27	3.20E-03	3.91E-02
cg05960039	GNAS;GNAS;GNAS;GNAS;GNAS;GNAS	20	3'UTR;TSS1500;Body;TSS1500;3'UTR;TSS1	Island	23.23	3.47	26.49	3.96	-3.27	2.41E-04	5.73E-03
cg04091816	PDK2	17	Body	N_Shelf	49.78	3.93	53.05	4.42	-3.27	2.62E-03	3.41E-02
cg00160886	VEPH1;VEPH1;VEPH1;VEPH1;VEPH1	3	5'UTR;5'UTR;5'UTR;5'UTR;5'UTR	Open sea	60.94	5.29	64.21	5.08	-3.27	6.37E-04	1.22E-02
cg16806077	ATP6V1G2;NFKBIL1;NFKBIL1;ATP6V1C	6	Body;TSS1500;TSS1500;Body	S_Shelf	72.46	3.93	75.73	4.44	-3.27	2.96E-03	3.71E-02
cg03483632	FJX1	11	TSS1500	N_Shore	38.17	5.67	41.43	6.32	-3.27	3.66E-03	4.29E-02
cg18265162	C1orf133;SERTAD4	1	TSS200;5'UTR	Island	23.87	3.16	27.14	3.67	-3.27	9.46E-06	3.58E-04
cg07710883	CAPN1	11	Body	Island	59.64	4.77	62.90	4.63	-3.27	4.51E-03	4.95E-02
cg11936089		11	Intergenic	Open sea	62.18	3.65	65.45	3.98	-3.27	1.75E-04	4.45E-03
cg08853487	CASQ1	1	Body	N_Shelf	78.45	3.60	81.72	2.66	-3.27	1.44E-05	5.23E-04
cg00458878		7	Intergenic	Island	17.59	4.32	20.86	4.24	-3.27	1.32E-04	3.52E-03
cg16987900	PCDHA2;PCDHA1;PCDHA1;PCDHA6;P	5	Body;Body;Body;TSS200;Body;TSS200;Boc	N_Shore	19.15	4.77	22.41	5.08	-3.27	3.99E-03	4.56E-02
cg25758167	AGL;AGL;AGL;AGL;AGL	1	TSS1500;TSS1500;TSS1500;TSS1500;TSS15	N_Shore	73.97	4.91	77.23	3.58	-3.27	3.57E-03	4.22E-02
cg20116804	C6orf138	6	1stExon	Island	15.99	3.45	19.25	4.42	-3.26	9.97E-04	1.71E-02
cg02933228	CDC42BPG	11	Body	N_Shore	74.62	3.61	77.89	3.84	-3.26	7.48E-06	2.89E-04
cg11826452	SLC25A33	1	TSS1500	N_Shore	54.71	4.13	57.97	3.93	-3.26	1.96E-03	2.79E-02
cg18542495	THADA;THADA	2	Body;Body	Open sea	64.80	5.53	68.06	5.00	-3.26	5.64E-04	1.11E-02

cg08884928	YPEL2	17	Body	Open sea	35.35	3.85	38.62	5.12	-3.26	4.17E-03	4.69E-02
cg24253904	NAV1	1	Body	Open sea	53.48	5.03	56.75	6.11	-3.26	3.18E-04	7.13E-03
cg19059861	PLUNC;PLUNC	20	TSS200;TSS200	Open sea	30.96	4.63	34.23	5.13	-3.26	4.54E-03	4.98E-02
cg05270007	C6orf204;C6orf204;PLN	6	Body;Body;3'UTR	Open sea	83.08	5.49	86.35	5.77	-3.26	2.18E-03	3.00E-02
cg03572760	UBTF;UBTF;UBTF	17	Body;Body;Body	N_Shore	71.96	3.78	75.22	3.53	-3.26	1.07E-04	2.96E-03
cg15321396	KIAA0895L;EXOC3L	16	TSS1500;Body	Island	64.41	3.79	67.67	3.15	-3.26	2.46E-04	5.83E-03
cg17495715		2	Intergenic	Island	26.38	3.19	29.64	3.13	-3.26	3.07E-05	1.02E-03
cg06166490	HOXA2	7	TSS1500	Island	26.33	4.11	29.59	3.92	-3.26	1.76E-03	2.59E-02
cg09512548	FYN;FYN;FYN	6	Body;Body;Body	Open sea	44.27	4.39	47.54	4.69	-3.26	3.07E-03	3.81E-02
cg15247245		3	Intergenic	Open sea	16.16	3.09	19.42	5.46	-3.26	1.91E-03	2.74E-02
cg00370608	VAC14	16	Body	Open sea	63.54	4.39	66.80	4.25	-3.26	1.44E-03	2.24E-02
cg17010657	PHAX	5	TSS1500	N_Shore	81.88	4.89	85.14	4.11	-3.26	3.98E-03	4.55E-02
cg02713180		10	Intergenic	S_Shore	71.45	4.51	74.71	4.40	-3.26	3.78E-04	8.15E-03
cg15965383		1	Intergenic	Open sea	76.44	4.91	79.70	5.02	-3.26	3.78E-03	4.39E-02
cg14014731	RPS6	9	Body	N_Shore	53.28	7.59	56.54	7.47	-3.26	1.59E-03	2.40E-02
cg16747109	PITX2;PITX2;PITX2	4	TSS1500;Body;Body	Island	15.16	2.63	18.42	3.63	-3.26	1.17E-06	5.20E-05
cg09175137	FAM92B	16	Body	Open sea	83.41	3.90	86.67	3.01	-3.26	8.11E-05	2.35E-03
cg18036068	C18orf62	18	3'UTR	Open sea	81.82	5.00	85.08	4.22	-3.26	2.95E-03	3.70E-02
cg14412490		11	Intergenic	Open sea	19.64	3.67	22.90	4.89	-3.26	7.64E-04	1.40E-02
cg06056880	GABRB2;GABRB2	5	3'UTR;3'UTR	Open sea	54.55	4.97	57.81	5.23	-3.26	8.55E-04	1.52E-02
cg21544633	PARVA	11	Body	Open sea	49.74	4.43	53.00	4.16	-3.26	3.98E-05	1.28E-03
cg13592721	HIST1H2BK;HIST1H4I	6	3'UTR;1stExon	Island	33.65	5.90	36.91	6.80	-3.26	1.87E-03	2.70E-02
cg23933731	GFPT2	5	Body	Island	41.12	3.57	44.38	5.21	-3.26	1.08E-03	1.81E-02
cg04436554		5	Intergenic	N_Shore	46.01	5.68	49.27	5.98	-3.26	1.18E-03	1.94E-02
cg15052217	BRD9;BRD9;BRD9	5	Body;3'UTR;3'UTR	N_Shore	74.10	3.89	77.36	3.42	-3.26	1.19E-04	3.24E-03
cg25535652	EPHA7	6	Body	Open sea	57.12	3.94	60.38	4.66	-3.26	1.87E-03	2.69E-02
cg07132223	DTX3;GEFT	12	3'UTR;TSS1500	N_Shore	78.10	3.62	81.36	3.02	-3.26	4.37E-04	9.13E-03
cg21453209		8	Intergenic	S_Shelf	64.21	5.30	67.46	4.21	-3.26	2.17E-03	3.00E-02
cg02361903	CSF1;CSF1;CSF1;CSF1	1	TSS1500;TSS1500;TSS1500;TSS1500	N_Shore	32.07	4.63	35.33	4.92	-3.26	4.04E-03	4.59E-02
cg04323622	NAA15	4	Body	S_Shelf	76.30	5.08	79.55	4.33	-3.26	3.12E-03	3.85E-02
cg10283844	HPCAL1;HPCAL1	2	5'UTR;5'UTR	Open sea	79.15	4.64	82.41	4.02	-3.26	2.08E-04	5.10E-03
cg05231308	UCN	2	TSS200	Island	36.84	4.40	40.10	3.58	-3.26	2.04E-03	2.87E-02
cg04726446	C2orf39	2	1stExon	Island	24.78	5.20	28.04	5.85	-3.26	1.57E-03	2.37E-02
cg11755933	PITPNM1;PITPNM1	11	Body;Body	S_Shelf	78.41	4.02	81.67	3.60	-3.26	6.04E-04	1.17E-02
cg14477581	SMC5	9	Body	Open sea	73.23	5.80	76.49	4.41	-3.26	3.00E-03	3.74E-02
cg26124318	ADAM32;ADAM32	8	1stExon;5'UTR	Island	8.17	2.67	11.43	5.23	-3.26	2.83E-04	6.51E-03
cg03037761	FZD4	11	Body	N_Shelf	73.09	3.79	76.35	3.78	-3.26	1.14E-04	3.11E-03
cg08650290	TRIO	5	Body	Open sea	72.15	3.98	75.41	3.24	-3.26	1.20E-04	3.25E-03
cg13590966		3	Intergenic	N_Shelf	81.82	3.70	85.08	3.30	-3.26	3.61E-05	1.17E-03
cg27565368	UQCRFS1	19	TSS1500	S_Shore	73.14	3.84	76.40	4.41	-3.26	3.54E-04	7.75E-03
cg00070529	ENOX1;ENOX1	13	Body;Body	Open sea	78.77	4.93	82.02	4.63	-3.26	8.27E-04	1.48E-02
cg25470758		1	Intergenic	Island	65.12	4.73	68.38	4.77	-3.26	3.79E-03	4.40E-02
cg27296330	ZNF331;ZNF331	19	TSS200;5'UTR	Island	24.49	4.44	27.74	4.97	-3.26	2.31E-03	3.12E-02
cg17431301	GAK	4	Body	S_Shore	76.29	5.05	79.55	4.13	-3.26	2.75E-03	3.52E-02
cg14868747	ALX4	11	TSS1500	N_Shore	18.62	3.39	21.88	3.88	-3.26	1.28E-05	4.71E-04
cg01055824	CCDC88C	14	Body	Open sea	55.63	4.30	58.89	4.71	-3.26	4.47E-03	4.93E-02
cg19193931	SIM2;SIM2	21	Body;Body	S_Shore	25.77	2.68	29.02	2.80	-3.26	4.17E-06	1.69E-04
cg06633243	CAMK2D;CAMK2D;CAMK2D;CAMK2D	4	Body;Body;Body;Body;Body;Body	Open sea	81.72	3.10	84.98	2.68	-3.26	3.95E-06	1.61E-04
cg00437124		6	Intergenic	Open sea	44.04	4.44	47.30	4.82	-3.26	3.30E-05	1.08E-03
cg04098052		7	Intergenic	Open sea	59.10	5.26	62.36	4.75	-3.26	1.88E-03	2.71E-02
cg13521678	LOC100133920	9	Body	Open sea	60.64	4.27	63.90	3.71	-3.26	1.10E-04	3.03E-03
cg24209126	IMMP2L	7	Body	Open sea	63.17	3.40	66.42	2.67	-3.26	1.47E-06	6.45E-05
cg15007813	LOC100302401;RASAL2	1	Body;TSS1500	N_Shore	77.92	3.86	81.18	3.86	-3.26	1.16E-04	3.16E-03

cg00600521	AASS	7	TSS1500	S_Shore	81.94	3.89	85.20	3.62	-3.26	1.63E-04	4.20E-03
cg20098446	FLCN	17	Body	Open sea	76.52	3.46	79.77	2.25	-3.26	1.76E-05	6.23E-04
cg14775296	MEIS1	2	Body	Island	23.96	3.54	27.22	3.50	-3.26	7.06E-06	2.74E-04
cg15977933	NMNAT2	1	TSS1500	S_Shore	69.76	5.28	73.01	3.92	-3.26	2.10E-03	2.93E-02
cg00813264		3	Intergenic	Open sea	77.12	4.45	80.37	3.69	-3.26	4.58E-04	9.46E-03
cg24591300	HPSE2;HPSE2;HPSE2;HPSE2	10	Body;Body;Body;Body	N_Shore	39.48	3.40	42.73	4.22	-3.26	2.64E-04	6.16E-03
cg00791406	GRIN2B	12	5'UTR	Open sea	59.19	5.23	62.45	4.09	-3.25	2.35E-03	3.17E-02
cg24878454	EPB41;EPB41;EPB41;EPB41;EPB41;EPI	1	5'UTR;5'UTR;5'UTR;5'UTR;5'UTR;5'UTR	Island	35.99	6.14	39.24	5.53	-3.25	9.75E-04	1.68E-02
cg21789085	MYOM2	8	Body	Open sea	84.47	5.37	87.73	3.72	-3.25	7.54E-05	2.21E-03
cg27198632		4	Intergenic	N_Shore	25.64	3.73	28.89	5.43	-3.25	3.05E-03	3.79E-02
cg11818127		12	Intergenic	S_Shelf	77.68	3.54	80.94	3.47	-3.25	5.15E-05	1.59E-03
cg09981361		8	Intergenic	Open sea	21.79	3.74	25.04	4.46	-3.25	1.14E-03	1.89E-02
cg05037622	JPH2;JPH2	20	TSS1500;TSS1500	Open sea	29.46	4.17	32.72	4.79	-3.25	1.41E-03	2.20E-02
cg13252401	NGEF;NGEF	2	Body;TSS1500	Island	60.53	6.38	63.79	5.43	-3.25	2.10E-03	2.93E-02
cg06815715	SHC4	15	Body	Open sea	37.42	4.16	40.67	4.85	-3.25	3.65E-03	4.29E-02
cg21534774		17	Intergenic	N_Shelf	67.97	5.62	71.22	6.08	-3.25	1.33E-03	2.11E-02
cg23906191	C7orf50;C7orf50;C7orf50	7	Body;Body;Body	S_Shore	79.92	3.67	83.18	2.47	-3.25	1.16E-05	4.30E-04
cg18968920	PRPF40A	2	Body	N_Shore	46.52	5.18	49.77	5.20	-3.25	2.44E-03	3.25E-02
cg08265548	PHOX2A	11	Body	N_Shore	80.48	4.70	83.73	3.49	-3.25	1.31E-03	2.09E-02
cg05787265		16	Intergenic	N_Shore	63.06	4.74	66.32	4.69	-3.25	1.24E-03	2.01E-02
cg08461903		21	Intergenic	S_Shore	80.48	4.65	83.73	3.16	-3.25	1.25E-04	3.37E-03
cg18616091	MPP2	17	TSS1500	S_Shore	44.65	5.02	47.90	5.68	-3.25	1.86E-03	2.69E-02
cg05624853	HSD3B7;HSD3B7;HSD3B7	16	3'UTR;3'UTR;3'UTR	Open sea	59.26	4.18	62.51	3.98	-3.25	2.26E-03	3.08E-02
cg17858100	YOD1	1	TSS200	N_Shore	22.30	4.21	25.56	4.99	-3.25	4.45E-03	4.91E-02
cg23033845	TMEM88	17	Body	Island	75.60	3.94	78.86	2.68	-3.25	2.50E-04	5.90E-03
cg13457462		8	Intergenic	Open sea	68.18	4.45	71.43	5.09	-3.25	1.30E-03	2.08E-02
cg19762075	UTP18	17	3'UTR	Open sea	78.95	4.47	82.20	4.72	-3.25	2.98E-03	3.72E-02
cg19711782	TMEM115	3	1stExon	N_Shore	60.62	4.25	63.87	4.23	-3.25	3.21E-04	7.17E-03
cg15017496	ARHGEF17	11	Body	S_Shelf	68.23	4.21	71.49	3.34	-3.25	4.98E-04	1.01E-02
cg12589936	C15orf24	15	Body	N_Shelf	81.90	5.88	85.15	5.05	-3.25	4.12E-03	4.66E-02
cg03882437	GRIN1;GRIN1;GRIN1	9	Body;Body;Body	Island	20.81	4.48	24.06	5.24	-3.25	4.12E-03	4.65E-02
cg03994184		7	Intergenic	Open sea	77.50	4.99	80.76	3.74	-3.25	3.03E-03	3.76E-02
cg16660677	SELE	1	TSS200	Open sea	55.30	5.30	58.55	4.07	-3.25	3.38E-03	4.07E-02
cg14697268		13	Intergenic	Open sea	70.47	4.77	73.72	4.18	-3.25	2.79E-03	3.57E-02
cg00167076	IQSEC1;IQSEC1	3	3'UTR;3'UTR	N_Shore	78.70	3.31	81.95	4.04	-3.25	5.33E-05	1.64E-03
cg00070460	CTBP1;CTBP1	4	Body;Body	N_Shore	61.71	3.95	64.96	3.88	-3.25	2.03E-04	5.00E-03
cg23361659		2	Intergenic	Open sea	51.23	3.89	54.48	4.54	-3.25	1.91E-04	4.76E-03
cg24784036	SEMA3B;SEMA3B	3	Body;Body	N_Shelf	55.05	5.07	58.30	4.37	-3.25	5.88E-04	1.15E-02
cg14128641	HAPLN4	19	Body	Island	40.21	5.52	43.46	6.52	-3.25	1.34E-03	2.12E-02
cg13738144	PRDM13;PRDM13	6	1stExon;5'UTR	Island	23.06	2.83	26.31	3.80	-3.25	4.76E-05	1.49E-03
cg20889818		15	Intergenic	S_Shore	36.18	4.42	39.43	5.33	-3.25	1.91E-03	2.74E-02
cg26939539	LOC146336	16	Body	N_Shore	43.83	4.52	47.08	4.41	-3.25	1.16E-03	1.91E-02
cg10024587	CALHM2;CALHM2	10	5'UTR;Body	N_Shore	23.45	3.73	26.70	4.66	-3.25	3.62E-04	7.89E-03
cg14601621	C9orf3	9	3'UTR	Island	87.75	4.44	91.00	3.37	-3.25	1.57E-03	2.38E-02
cg06732228	LTBP4;LTBP4;LTBP4	19	Body;Body;Body	Island	24.93	4.12	28.18	4.26	-3.25	9.48E-04	1.65E-02
cg12285988		1	Intergenic	Island	20.73	4.17	23.98	3.85	-3.25	5.41E-04	1.08E-02
cg09911755	OSBPL7	17	TSS1500	Open sea	47.30	4.27	50.55	3.58	-3.25	1.79E-03	2.62E-02
cg27621576		6	Intergenic	Island	12.69	2.84	15.94	3.58	-3.25	1.83E-08	9.59E-07
cg11448557	FAM13C;FAM13C;FAM13C;FAM13C	10	Body;Body;Body;Body	Open sea	81.29	3.91	84.53	3.28	-3.25	7.64E-05	2.23E-03
cg07516958		5	Intergenic	Open sea	82.45	4.40	85.70	4.00	-3.25	5.81E-04	1.14E-02
cg11613559	INPP5F;INPP5F	10	Body;TSS1500	Island	54.45	3.80	57.70	3.82	-3.25	1.16E-03	1.91E-02
cg06211323	MYO16	13	TSS200	Open sea	45.98	4.30	49.22	4.55	-3.25	3.42E-04	7.54E-03
cg20434300	ETS2	21	Body	Island	75.48	4.39	78.73	3.43	-3.25	2.35E-04	5.62E-03

cg23568643		13	Intergenic	Open sea	40.89	4.71	44.14	5.20	-3.25	3.42E-03	4.10E-02
cg12112151	KLC1;KLC1	14	Body;Body	N_Shelf	83.07	4.19	86.32	3.80	-3.25	4.80E-04	9.81E-03
cg16933922	OAT	10	5'UTR	N_Shore	10.33	3.02	13.58	4.50	-3.25	4.71E-04	9.67E-03
cg15403961	GALNTL6	4	Body	Open sea	66.59	3.37	69.84	3.86	-3.25	1.52E-04	3.95E-03
cg00511577		7	Intergenic	Open sea	79.29	4.73	82.54	4.12	-3.25	4.42E-04	9.21E-03
cg18048897	GNL1	6	Body	N_Shore	79.92	3.01	83.16	2.35	-3.25	3.93E-07	1.86E-05
cg24953321	TRIM71	3	Body	N_Shore	20.36	2.63	23.61	3.66	-3.25	1.11E-05	4.14E-04
cg03628033	ZBTB42	14	TSS1500	N_Shore	64.91	4.17	68.16	4.83	-3.25	9.78E-04	1.68E-02
cg04380873		3	Intergenic	Open sea	84.57	3.77	87.82	3.14	-3.25	4.65E-05	1.46E-03
cg03575765		14	Intergenic	S_Shore	31.18	4.33	34.43	5.01	-3.25	2.39E-03	3.20E-02
cg17480461	SCARNA11;CHD4	12	TSS1500;Body	Open sea	69.14	4.93	72.38	5.03	-3.25	4.45E-03	4.91E-02
cg00299893	LRRK1	15	3'UTR	Open sea	70.05	4.11	73.29	3.47	-3.25	3.03E-04	6.86E-03
cg14883961		8	Intergenic	Open sea	65.13	4.50	68.37	3.80	-3.25	9.18E-04	1.61E-02
cg03732056	DOK2	8	1stExon	S_Shore	45.28	5.48	48.53	5.97	-3.25	1.59E-03	2.40E-02
cg05226707	FABP3	1	TSS1500	Open sea	19.82	4.88	23.07	6.19	-3.25	4.48E-05	1.41E-03
cg19143639		8	Intergenic	Open sea	22.41	6.18	25.65	6.80	-3.25	7.84E-04	1.43E-02
cg26726370	LY6G5B;CSNK2B	6	TSS1500;Body	S_Shelf	74.82	4.01	78.07	3.08	-3.25	3.39E-04	7.49E-03
cg12205903	ZIM2;PEG3;ZIM2;ZIM2;PEG3;PEG3;PE	19	1stExon;5'UTR;1stExon;5'UTR;1stExon;1st	N_Shore	38.35	3.39	41.60	3.39	-3.25	5.74E-05	1.75E-03
cg24116317	STK10	5	Body	Open sea	79.23	4.18	82.47	3.58	-3.25	2.34E-04	5.61E-03
cg08607625	NOS3;NOS3;NOS3;NOS3	7	5'UTR;TSS200;TSS200;TSS200	Open sea	66.73	3.96	69.97	3.59	-3.25	6.22E-04	1.20E-02
cg05265257	GUCY1B2	13	Body	Open sea	63.87	5.11	67.11	5.42	-3.24	2.90E-03	3.65E-02
cg03740682		7	Intergenic	Open sea	47.35	4.23	50.59	3.64	-3.24	5.83E-04	1.14E-02
cg23256401		4	Intergenic	Open sea	56.82	4.05	60.06	4.20	-3.24	2.38E-04	5.68E-03
cg15323638	PODXL;PODXL	7	Body;Body	Open sea	63.57	5.29	66.81	5.89	-3.24	2.16E-04	5.25E-03
cg16601385	CFD	19	TSS1500	N_Shore	74.93	3.59	78.18	3.70	-3.24	1.16E-04	3.16E-03
cg21609640	ILDR2	1	Body	Island	18.60	2.89	21.84	4.24	-3.24	2.79E-05	9.36E-04
cg19035908		19	Intergenic	S_Shore	64.93	4.21	68.17	3.44	-3.24	2.28E-05	7.83E-04
cg22189125	ZNF302;ZNF302	19	Body;Body	S_Shelf	81.82	6.55	85.06	5.43	-3.24	1.84E-03	2.67E-02
cg06452513	UNC119;UNC119	17	TSS1500;TSS1500	S_Shore	42.79	4.08	46.04	4.07	-3.24	2.71E-04	6.29E-03
cg15856832	QRICH1;QRICH1	3	5'UTR;5'UTR	N_Shelf	73.77	4.27	77.01	4.69	-3.24	8.81E-04	1.56E-02
cg13868604	GDAP1L1;GDAP1L1	20	5'UTR;1stExon	Island	14.16	3.88	17.40	4.94	-3.24	1.63E-04	4.19E-03
cg04492369	PTDSS2	11	3'UTR	S_Shore	74.27	4.19	77.51	3.93	-3.24	5.95E-04	1.16E-02
cg18674446		14	Intergenic	S_Shore	79.99	4.51	83.23	3.63	-3.24	2.78E-04	6.41E-03
cg21287356		4	Intergenic	Open sea	45.67	5.41	48.91	4.10	-3.24	6.45E-04	1.23E-02
cg25137711		1	Intergenic	Island	18.46	3.38	21.71	4.54	-3.24	2.26E-04	5.45E-03
cg07257505	MYH10	17	Body	S_Shelf	74.70	4.84	77.94	3.51	-3.24	9.83E-04	1.69E-02
cg18355311	ME3;ME3;ME3	11	Body;Body;Body	Open sea	78.82	4.47	82.06	3.46	-3.24	1.93E-04	4.81E-03
cg13210467	STAG3;GPC2	7	TSS200;TSS1500	Island	47.64	4.12	50.88	4.64	-3.24	3.92E-04	8.39E-03
cg27634020	AGAP1;AGAP1	2	Body;Body	Open sea	74.18	4.55	77.42	3.80	-3.24	2.01E-03	2.84E-02
cg16896205	CD53	1	TSS200	Open sea	48.27	4.65	51.51	4.48	-3.24	1.92E-03	2.75E-02
cg01811418		4	Intergenic	N_Shore	68.62	4.08	71.86	2.82	-3.24	3.44E-04	7.58E-03
cg04935965	SH3BGR;SH3BGR	21	5'UTR;TSS1500	Open sea	79.40	4.60	82.64	3.95	-3.24	2.09E-03	2.92E-02
cg10857905		10	Intergenic	Open sea	40.14	3.52	43.38	3.82	-3.24	3.33E-04	7.39E-03
cg27350104		11	Intergenic	N_Shelf	49.78	4.03	53.02	4.71	-3.24	1.92E-03	2.75E-02
cg24361808		22	Intergenic	N_Shelf	50.51	5.13	53.75	4.84	-3.24	2.60E-03	3.39E-02
cg01833057	AFAP1;AFAP1	4	5'UTR;5'UTR	Open sea	64.45	4.85	67.69	4.36	-3.24	2.50E-03	3.30E-02
cg12265810	FOSB;FOSB	19	Body;Body	N_Shore	13.37	3.67	16.61	5.02	-3.24	2.69E-03	3.47E-02
cg15082394	RGL2;RGL2	6	Body;Body	N_Shore	79.46	4.46	82.70	2.94	-3.24	1.76E-04	4.46E-03
cg22041590		2	Intergenic	Open sea	73.89	4.47	77.12	4.08	-3.24	2.11E-04	5.15E-03
cg16561893		6	Intergenic	Island	9.30	1.78	12.54	2.90	-3.24	1.01E-08	5.36E-07
cg16022575	FOXD2	1	1stExon	Island	11.93	5.31	15.17	6.41	-3.24	4.61E-04	9.52E-03
cg24670151	GGA3;MRPS7;GGA3	17	Body;TSS1500;Body	N_Shore	45.14	5.59	48.38	5.34	-3.24	2.58E-03	3.37E-02
cg06828335	KCNMA1;KCNMA1;KCNMA1;KCNMA1	10	Body;Body;Body;Body	Open sea	80.08	5.94	83.31	3.78	-3.24	1.21E-03	1.97E-02

cg17624802	FAM171A2	17	Body	Island	19.07	2.90	22.31	3.06	-3.24	5.10E-06	2.03E-04
cg09806671	LMX1A	1	Body	Island	24.19	3.30	27.43	3.75	-3.24	4.18E-05	1.33E-03
cg08151809	PRKAG2;PRKAG2	7	Body;Body	Island	33.15	3.93	36.39	5.02	-3.24	9.10E-04	1.60E-02
cg10119082	ARPC1B	7	Body	Island	8.96	2.95	12.20	4.06	-3.24	2.10E-06	8.96E-05
cg12447744		1	Intergenic	Open sea	76.80	3.99	80.04	4.08	-3.24	7.45E-04	1.37E-02
cg22354175	C13orf35	13	5'UTR	S_Shelf	73.30	4.46	76.54	3.95	-3.24	4.56E-04	9.44E-03
cg03233426	TMEM100;TMEM100	17	TSS1500;5'UTR	Open sea	50.37	5.94	53.60	6.25	-3.24	1.92E-03	2.75E-02
cg19496364		8	Intergenic	Open sea	47.25	4.80	50.49	4.79	-3.24	1.67E-03	2.48E-02
cg00955967		4	Intergenic	Open sea	17.12	3.81	20.35	4.63	-3.24	1.64E-04	4.22E-03
cg05095158	RASSF10	11	1stExon	Island	15.62	4.22	18.86	5.17	-3.24	2.26E-04	5.45E-03
cg06069100	SGK269	15	5'UTR	Open sea	74.35	4.24	77.58	4.85	-3.24	6.18E-04	1.19E-02
cg21984532	FLJ43663;FLJ43663	7	Body;Body	N_Shore	31.44	5.53	34.67	4.67	-3.24	1.78E-03	2.60E-02
cg05933736	MYO16	13	TSS200	Open sea	48.32	5.92	51.56	6.24	-3.24	2.31E-03	3.13E-02
cg20899266	SETD5	3	Body	Open sea	80.47	4.58	83.71	4.78	-3.24	5.32E-04	1.06E-02
cg10484958	PCDH8;PCDH8	13	TSS1500;TSS1500	N_Shore	17.19	4.17	20.43	5.85	-3.24	2.60E-03	3.39E-02
cg00470044	BRE;BRE;BRE;BRE;BRE	2	Body;Body;Body;Body;Body	Open sea	56.70	6.77	59.94	6.78	-3.24	3.31E-03	4.01E-02
cg05511285		3	Intergenic	Island	49.04	3.53	52.28	3.00	-3.24	1.31E-06	5.80E-05
cg10291238	XPO4	13	Body	Open sea	80.23	5.37	83.46	3.71	-3.24	3.02E-03	3.76E-02
cg24037976		6	Intergenic	Island	82.58	5.44	85.82	4.10	-3.24	5.61E-04	1.11E-02
cg26719542		16	Intergenic	N_Shore	86.07	4.30	89.30	3.73	-3.24	1.00E-03	1.72E-02
cg20005056	FAM59B	2	Body	S_Shore	60.02	4.57	63.26	5.10	-3.24	3.16E-03	3.88E-02
cg02657611	TESK2	1	Body	Open sea	25.56	3.77	28.79	5.15	-3.24	4.50E-03	4.95E-02
cg22972858	GPR81	12	TSS200	Open sea	50.28	4.49	53.52	3.47	-3.24	1.67E-03	2.49E-02
cg14277543	FAM129B;FAM129B	9	Body;Body	S_Shelf	44.83	3.55	48.06	3.96	-3.24	4.79E-04	9.79E-03
cg00627045	SHISA6	17	Body	Island	78.23	4.47	81.47	4.16	-3.24	1.15E-03	1.90E-02
cg11644596	LRRC3	21	TSS1500	N_Shore	51.20	5.12	54.43	4.32	-3.23	1.97E-03	2.80E-02
cg07252575	DHX40P	17	TSS200	Open sea	71.61	3.99	74.84	4.11	-3.23	8.05E-04	1.45E-02
cg15383276	RASL10A;RASL10A	22	3'UTR;3'UTR	Island	45.07	3.86	48.30	4.94	-3.23	8.28E-04	1.49E-02
cg07015195	RSPO2	8	Body	N_Shore	31.77	4.73	35.00	4.27	-3.23	4.15E-03	4.68E-02
cg04460984	CHRNB4	15	TSS200	Island	23.05	5.60	26.28	5.22	-3.23	9.97E-04	1.71E-02
cg18309286	PAK1;PAK1	11	5'UTR;5'UTR	Island	37.65	5.05	40.88	5.63	-3.23	2.07E-03	2.89E-02
cg02178846		11	Intergenic	Open sea	54.43	3.36	57.66	4.69	-3.23	3.56E-04	7.79E-03
cg20452609	LSM2	6	TSS1500	S_Shore	56.05	3.72	59.28	4.36	-3.23	2.32E-05	7.97E-04
cg05382012	AGRP;AGRP	16	TSS200;TSS1500	S_Shelf	78.55	4.58	81.79	3.49	-3.23	1.34E-03	2.12E-02
cg13607230	MFSD9	2	Body	N_Shore	23.13	3.89	26.36	3.83	-3.23	1.13E-03	1.88E-02
cg14206523		15	Intergenic	Open sea	71.95	6.46	75.18	6.11	-3.23	7.83E-04	1.43E-02
cg09368716	TMEM88B	1	1stExon	Island	60.08	3.97	63.32	5.02	-3.23	1.51E-03	2.32E-02
cg27384569	GABRB3;GABRB3	15	Body;Body	Open sea	81.75	3.34	84.99	3.22	-3.23	1.27E-05	4.67E-04
cg12076479	DNAJC8	1	Body	Open sea	19.94	3.15	23.18	4.00	-3.23	4.28E-05	1.36E-03
cg09889228		16	Intergenic	N_Shore	67.45	4.26	70.69	4.23	-3.23	1.76E-03	2.59E-02
cg13329242	DSCAML1	11	Body	Open sea	56.44	4.78	59.67	4.28	-3.23	4.08E-03	4.63E-02
cg02392882		15	Intergenic	N_Shore	58.21	4.76	61.45	5.97	-3.23	1.48E-03	2.28E-02
cg01653727	FBXL18	7	Body	N_Shore	68.17	4.78	71.41	4.93	-3.23	2.25E-04	5.43E-03
cg09612786	ATG9B	7	Body	N_Shore	24.23	3.59	27.46	4.06	-3.23	5.71E-05	1.74E-03
cg15655097		2	Intergenic	N_Shelf	81.76	5.00	84.99	3.76	-3.23	1.50E-03	2.30E-02
cg23716866	CLIC5	6	TSS1500	Open sea	72.10	4.98	75.34	4.29	-3.23	3.68E-03	4.31E-02
cg01566965	HAND2	4	3'UTR	N_Shore	19.89	3.47	23.13	4.05	-3.23	1.41E-03	2.21E-02
cg00137840		4	Intergenic	Open sea	85.13	4.47	88.36	3.80	-3.23	1.72E-05	6.12E-04
cg24715245	UCHL1	4	TSS200	Island	25.26	3.36	28.49	3.97	-3.23	2.68E-05	9.05E-04
cg20682639	PDZD2;PDZD2	5	5'UTR;1stExon	Open sea	36.38	4.40	39.61	3.57	-3.23	1.40E-03	2.19E-02
cg22677637		2	Intergenic	N_Shelf	80.11	4.98	83.34	4.36	-3.23	1.28E-03	2.06E-02
cg00122406		8	Intergenic	N_Shelf	68.90	4.29	72.13	4.11	-3.23	7.96E-04	1.44E-02
cg01420688		6	Intergenic	N_Shore	21.32	4.05	24.55	3.92	-3.23	4.24E-04	8.91E-03

cg17308606	FAM102A;FAM102A	9	Body;Body	N_Shore	43.32	6.28	46.55	7.04	-3.23	2.63E-03	3.42E-02
cg11534519	STK10	5	Body	Open sea	54.76	5.18	57.99	4.65	-3.23	2.30E-03	3.12E-02
cg15080361		2	Intergenic	Open sea	64.65	4.78	67.88	5.49	-3.23	2.98E-03	3.72E-02
cg07409292	GPRIN3	4	5'UTR	N_Shore	19.69	4.63	22.92	4.90	-3.23	7.64E-04	1.40E-02
cg03074126	BCAM;BCAM	19	Body;Body	S_Shore	59.60	3.31	62.83	2.95	-3.23	4.10E-05	1.31E-03
cg04919982	RERGL	12	TSS200	Open sea	77.27	5.53	80.50	3.93	-3.23	6.41E-04	1.23E-02
cg23542449	KIAA0528	12	3'UTR	Open sea	77.54	4.44	80.76	3.97	-3.23	1.99E-03	2.82E-02
cg08265387		14	Intergenic	Open sea	80.02	2.74	83.25	3.10	-3.23	1.93E-06	8.29E-05
cg14064503	TUB	11	Body	Open sea	35.45	3.66	38.68	4.09	-3.23	5.95E-04	1.16E-02
cg09554509	HOXA13	7	1stExon	Island	21.06	3.48	24.29	3.69	-3.23	5.44E-05	1.67E-03
cg08533710	SLC1A5	19	TSS1500	S_Shore	55.84	6.00	59.07	6.09	-3.23	4.05E-03	4.61E-02
cg10023469	DYNC1H1	14	Body	S_Shelf	73.17	2.98	76.40	3.46	-3.23	1.45E-05	5.25E-04
cg23206461		1	Intergenic	Island	24.27	4.69	27.50	4.29	-3.23	4.46E-03	4.92E-02
cg14294321		19	Intergenic	S_Shore	59.45	6.16	62.67	3.61	-3.23	3.74E-03	4.36E-02
cg16165748	POLA2	11	Body	S_Shore	38.15	5.10	41.37	5.01	-3.23	2.28E-03	3.10E-02
cg02703787		15	Intergenic	N_Shore	47.69	4.23	50.92	2.86	-3.23	1.85E-04	4.65E-03
cg18447772	HOXA9	7	TSS200	Island	11.52	2.41	14.74	3.93	-3.23	6.21E-06	2.43E-04
cg16000022	TPCN2	11	3'UTR	Open sea	22.18	2.82	25.41	3.24	-3.23	2.78E-05	9.33E-04
cg08040148		17	Intergenic	S_Shelf	57.75	5.06	60.97	4.75	-3.23	3.19E-03	3.91E-02
cg01876812		1	Intergenic	Open sea	51.02	4.68	54.25	4.93	-3.23	1.40E-03	2.20E-02
cg11634496		11	Intergenic	N_Shore	15.89	3.78	19.12	5.16	-3.23	4.60E-05	1.45E-03
cg00574740	LMO7	13	Body	Island	35.95	4.49	39.18	4.98	-3.23	8.93E-04	1.57E-02
cg25715816	FAM136A	2	TSS1500	S_Shore	55.31	4.56	58.54	4.24	-3.23	4.81E-04	9.83E-03
cg21203643	DEGS2	14	TSS1500	S_Shore	82.29	4.31	85.51	3.77	-3.23	1.55E-04	4.03E-03
cg00701946	LOC100302652;GPR75	2	TSS200;TSS200	Island	31.04	3.59	34.26	4.39	-3.23	7.42E-04	1.37E-02
cg17120366	MPP7	10	TSS1500	Open sea	79.37	2.94	82.59	2.64	-3.23	9.61E-07	4.33E-05
cg06417962	RNH1;RNH1;RNH1;RNH1;RNH1;RNH1	11	TSS1500;TSS1500;TSS1500;TSS1500;TSS15	Island	78.34	4.10	81.56	2.95	-3.23	2.49E-04	5.89E-03
cg10298741	ZFHX3;ZFHX3	16	5'UTR;5'UTR	Open sea	77.60	4.38	80.82	3.07	-3.23	1.08E-04	2.98E-03
cg26743024	CACNG4	17	TSS1500	N_Shore	30.48	3.86	33.70	5.25	-3.23	1.89E-03	2.72E-02
cg15744128	CCDC121;GPN1;GPN1;CCDC121;GPN1	2	Body;TSS1500;TSS1500;Body;TSS1500;TSS	Open sea	55.10	5.22	58.32	5.05	-3.23	1.21E-03	1.97E-02
cg14749678	CCRL2;CCRL2	3	Body;Body	Open sea	68.47	4.52	71.70	4.52	-3.23	2.87E-03	3.63E-02
cg02393428		12	Intergenic	Open sea	50.05	3.61	53.27	4.77	-3.23	2.01E-05	7.01E-04
cg08752080	GC	4	Body	Open sea	77.95	3.80	81.18	3.47	-3.22	2.90E-04	6.63E-03
cg21118780	MAP4K4;MAP4K4;MAP4K4	2	Body;Body;Body	Open sea	71.70	4.05	74.92	3.98	-3.22	5.50E-04	1.09E-02
cg06744117		16	Intergenic	Open sea	23.36	5.27	26.59	5.39	-3.22	6.44E-04	1.23E-02
cg15580304	MIR941-3;MIR941-3;MIR941-2;MIR94	20	TSS1500;TSS200;TSS1500;TSS1500;TSS200	Island	74.20	4.72	77.43	4.61	-3.22	3.71E-03	4.33E-02
cg04519870	ALPI	2	TSS200	N_Shelf	69.93	4.11	73.16	3.51	-3.22	1.68E-05	5.98E-04
cg12045443		12	Intergenic	Open sea	19.65	3.52	22.87	4.14	-3.22	3.88E-04	8.32E-03
cg02898094	DLX6AS;DLX6	7	Body;TSS1500	Island	25.38	3.43	28.60	3.82	-3.22	1.95E-05	6.82E-04
cg24907762	ZNF22	10	5'UTR	S_Shore	81.77	4.87	85.00	5.06	-3.22	1.55E-03	2.36E-02
cg11831419	LMO7;LMO7	13	Body;Body	Open sea	80.38	4.31	83.60	3.72	-3.22	7.02E-04	1.31E-02
cg05392513	SRRT;SRRT;SRRT;SRRT	7	Body;Body;Body;Body	N_Shore	59.23	4.91	62.45	4.04	-3.22	4.82E-04	9.84E-03
cg16255816	HAP1;HAP1;HAP1	17	Body;Body;Body	N_Shore	28.19	3.77	31.41	3.47	-3.22	3.41E-04	7.53E-03
cg22886275		7	Intergenic	S_Shore	19.07	3.57	22.30	3.95	-3.22	6.89E-04	1.30E-02
cg18459342	TPD52;TPD52	8	TSS1500;TSS1500	Island	25.50	4.10	28.72	4.36	-3.22	1.15E-03	1.90E-02
cg25915982	GRB10	7	5'UTR	N_Shore	53.94	3.83	57.17	3.71	-3.22	1.23E-03	2.00E-02
cg05129858		8	Intergenic	Open sea	69.34	4.89	72.56	3.55	-3.22	5.26E-04	1.05E-02
cg20348196	HLA-DRA	6	Body	Open sea	65.23	7.20	68.45	6.16	-3.22	4.15E-03	4.68E-02
cg08938584	PCDHGA4;PCDHGA9;PCDHGA1;PCDH(	5	Body;Body;Body;Body;1stExon;Body;Body	Island	20.67	4.34	23.89	3.97	-3.22	4.00E-04	8.52E-03
cg20074340	MCF2L;MCF2L	13	Body;Body	S_Shore	74.00	4.00	77.23	4.22	-3.22	1.04E-04	2.88E-03
cg25578728	CHD7	8	Body	Open sea	85.35	5.98	88.57	2.55	-3.22	3.60E-03	4.25E-02
cg01566552	UPF1	19	3'UTR	Island	69.44	4.66	72.66	4.92	-3.22	1.21E-03	1.97E-02
cg09074856	FLJ42875	1	Body	Island	31.40	3.58	34.62	3.09	-3.22	1.03E-04	2.88E-03

cg22454313	NAV1;NAV1	1	Body;Body	Open sea	57.61	5.59	60.83	4.46	-3.22	7.44E-04	1.37E-02
cg07871532		21	Intergenic	N_Shelf	70.04	4.34	73.26	4.38	-3.22	3.97E-04	8.46E-03
cg10532489	SEZ6L2;SEZ6L2;SEZ6L2;SEZ6L2	16	Body;Body;Body;Body	Island	15.43	3.73	18.65	4.66	-3.22	2.15E-05	7.46E-04
cg04597449	GPR150	5	TSS1500	N_Shore	47.77	6.28	50.99	5.33	-3.22	1.70E-03	2.52E-02
cg18989499		13	Intergenic	S_Shelf	68.58	4.48	71.80	3.52	-3.22	2.08E-04	5.10E-03
cg12028936		4	Intergenic	Open sea	76.54	4.54	79.76	4.56	-3.22	6.96E-05	2.06E-03
cg10932018		5	Intergenic	Island	12.14	4.33	15.37	4.72	-3.22	8.37E-04	1.50E-02
cg10722426	CNTD2	19	Body	Island	48.90	4.71	52.13	4.47	-3.22	2.36E-03	3.17E-02
cg17868687	HDLBP;HDLBP	2	Body;Body	Open sea	74.07	4.58	77.30	3.98	-3.22	1.99E-03	2.82E-02
cg08264572		7	Intergenic	S_Shore	75.04	4.03	78.26	3.77	-3.22	2.78E-04	6.41E-03
cg24066601	HIST1H3G	6	1stExon	Island	26.14	3.73	29.36	3.35	-3.22	5.71E-06	2.26E-04
cg05207107	ERBB3	12	Body	N_Shelf	73.00	4.17	76.23	3.68	-3.22	1.06E-04	2.93E-03
cg25360861	ZNF257	19	TSS200	Open sea	12.01	3.55	15.23	3.78	-3.22	9.10E-05	2.59E-03
cg15617609	VPS37B	12	Body	S_Shore	27.95	3.53	31.17	4.78	-3.22	2.41E-03	3.23E-02
cg18983417		15	Intergenic	Open sea	77.38	4.29	80.60	5.40	-3.22	2.86E-03	3.62E-02
cg15034962	PLEKHA8	7	3'UTR	Open sea	70.82	5.62	74.04	5.09	-3.22	3.14E-03	3.86E-02
cg09746992	SLFN14	17	Body	Open sea	82.99	4.61	86.21	4.17	-3.22	8.43E-04	1.51E-02
cg18204145	C6orf154	6	Body	N_Shore	55.97	3.94	59.19	3.63	-3.22	8.01E-04	1.45E-02
cg07362003		2	Intergenic	Open sea	74.92	4.77	78.14	3.65	-3.22	4.23E-05	1.34E-03
cg00142642		10	Intergenic	N_Shelf	42.12	4.20	45.34	3.47	-3.22	1.11E-03	1.86E-02
cg05573844		12	Intergenic	Island	24.48	4.23	27.70	3.72	-3.22	2.57E-04	6.04E-03
cg02045981		21	Intergenic	N_Shore	71.04	4.34	74.26	3.26	-3.22	5.95E-04	1.16E-02
cg23118549	NPC1L1;NPC1L1	7	TSS200;TSS200	Open sea	39.52	3.23	42.74	5.16	-3.22	3.57E-03	4.22E-02
cg22685807		6	Intergenic	Open sea	34.51	5.25	37.73	7.03	-3.22	4.11E-03	4.65E-02
cg23154849		2	Intergenic	Open sea	63.91	4.84	67.13	4.77	-3.22	4.90E-04	9.96E-03
cg18333041	CLSTN2	3	Body	Open sea	88.27	4.72	91.49	2.96	-3.22	2.24E-05	7.71E-04
cg04811592	MITF;MITF	3	Body;Body	Open sea	64.06	4.27	67.28	4.16	-3.22	1.46E-03	2.26E-02
cg00802478	RBM47	4	5'UTR	Open sea	39.86	5.56	43.08	5.39	-3.22	1.08E-03	1.81E-02
cg08600862		5	Intergenic	N_Shore	25.89	3.95	29.10	4.12	-3.22	6.98E-04	1.31E-02
cg11413862		2	Intergenic	Open sea	51.10	3.43	54.32	2.98	-3.22	3.33E-05	1.09E-03
cg16160637	DEPDC5;DEPDC5;DEPDC5	22	1stExon;Body;Body	S_Shore	22.33	4.69	25.55	4.61	-3.22	2.93E-03	3.69E-02
cg16144331		8	Intergenic	Open sea	68.46	4.22	71.67	4.69	-3.22	1.32E-03	2.10E-02
cg23506042		5	Intergenic	N_Shelf	31.25	4.62	34.46	6.53	-3.22	2.16E-03	2.98E-02
cg27443224	CCL21;CCL21	9	1stExon;5'UTR	Open sea	51.83	5.92	55.05	5.26	-3.22	3.29E-03	3.99E-02
cg01528492	TMEM64;TMEM64	8	TSS1500;TSS1500	S_Shore	36.88	4.15	40.10	5.17	-3.22	4.14E-03	4.67E-02
cg14957943	COL7A1	3	TSS200	Island	14.45	3.21	17.67	4.43	-3.22	4.02E-05	1.29E-03
cg01414464		5	Intergenic	N_Shore	42.66	7.35	45.87	9.49	-3.22	2.77E-03	3.54E-02
cg10374813	MED24;MED24;SNORD124	17	Body;Body;TSS200	Open sea	53.20	4.40	56.42	4.41	-3.22	2.38E-04	5.68E-03
cg21180793	RAB3C	5	Body	Open sea	81.62	4.27	84.84	4.06	-3.22	1.36E-03	2.14E-02
cg14586500	DPM2	9	3'UTR	N_Shelf	53.25	5.10	56.47	5.33	-3.22	3.72E-04	8.05E-03
cg13639341	C20orf96	20	TSS1500	S_Shore	16.75	3.56	19.97	4.15	-3.22	4.66E-04	9.60E-03
cg13948456	GPSM1	9	Body	Island	49.19	3.86	52.41	3.77	-3.22	5.23E-04	1.05E-02
cg10979602	IL23R	1	Body	Open sea	77.42	4.06	80.64	3.83	-3.22	4.12E-05	1.31E-03
cg18695750	FAM135A;FAM135A;FAM135A	6	TSS1500;5'UTR;5'UTR	Open sea	78.55	4.30	81.77	4.49	-3.22	5.84E-04	1.14E-02
cg26325497	MIR525	19	TSS1500	Open sea	25.81	3.44	29.03	3.96	-3.22	1.45E-04	3.80E-03
cg03611618	TNXB	6	Body	S_Shelf	45.48	2.91	48.69	4.02	-3.22	1.31E-04	3.50E-03
cg24346464		1	Intergenic	Open sea	67.38	4.95	70.60	4.53	-3.22	1.85E-03	2.68E-02
cg17239103	CCDC19	1	TSS200	Island	41.65	3.62	44.86	3.84	-3.21	3.02E-05	1.00E-03
cg05278307	DSCAML1	11	Body	S_Shore	42.69	2.58	45.91	4.12	-3.21	9.85E-05	2.76E-03
cg16122796	COL16A1	1	Body	Open sea	75.05	4.07	78.26	4.61	-3.21	5.22E-04	1.05E-02
cg23317818	MYO1B;MYO1B;MYO1B	2	Body;Body;Body	Open sea	83.30	4.32	86.52	4.19	-3.21	3.75E-04	8.11E-03
cg16043357	VAX1;VAX1	10	TSS200;TSS200	Island	17.32	2.98	20.53	3.77	-3.21	2.73E-05	9.19E-04
cg22301854	ABLIM2;ABLIM2;ABLIM2;ABLIM2;ABL	4	Body;Body;Body;Body;Body;Body;Body	Open sea	49.86	6.23	53.07	6.98	-3.21	4.12E-03	4.65E-02

cg10149054		15	Intergenic	N_Shore	27.05	4.02	30.26	4.68	-3.21	5.05E-04	1.02E-02
cg01119072	SLC38A8	16	TSS1500	Open sea	54.34	4.35	57.56	4.55	-3.21	6.88E-04	1.29E-02
cg12109221		3	Intergenic	Open sea	77.70	3.91	80.91	3.93	-3.21	3.50E-04	7.68E-03
cg03565274		3	Intergenic	Open sea	25.87	3.29	29.08	4.67	-3.21	1.69E-03	2.51E-02
cg18581663	WIPF3	7	TSS1500	Open sea	75.33	4.12	78.54	3.94	-3.21	1.44E-04	3.78E-03
cg19643053	HOXA5	7	TSS1500	Island	30.28	4.15	33.49	5.15	-3.21	4.38E-03	4.85E-02
cg01800884	ARHGEF17	11	Body	S_Shore	63.63	4.46	66.84	4.14	-3.21	8.66E-04	1.54E-02
cg22660542	HOXC8	12	TSS1500	N_Shore	37.64	5.07	40.85	3.81	-3.21	4.01E-03	4.58E-02
cg00474798	SUCLG1	2	Body	N_Shore	47.75	5.65	50.97	5.72	-3.21	3.41E-03	4.10E-02
cg00988346		1	Intergenic	Open sea	53.72	5.34	56.93	4.78	-3.21	3.43E-03	4.11E-02
cg17105041	DLGAP2	8	Body	Open sea	47.52	3.82	50.73	3.84	-3.21	1.39E-03	2.18E-02
cg08064068		14	Intergenic	Open sea	43.96	5.46	47.17	5.09	-3.21	1.48E-03	2.28E-02
cg11463271	DOK3;DOK3;DOK3	5	TSS200;5'UTR;5'UTR	Open sea	53.61	5.30	56.82	4.73	-3.21	3.47E-03	4.14E-02
cg26915704	NFATC1;NFATC1;NFATC1;NFATC1;NF/	18	Body;Body;Body;Body;Body	S_Shore	76.03	4.50	79.24	3.46	-3.21	1.29E-03	2.06E-02
cg16132186		10	Intergenic	Island	76.88	3.59	80.09	3.04	-3.21	4.18E-05	1.33E-03
cg19760241	LHX1	17	Body	Island	22.49	3.37	25.70	3.79	-3.21	3.70E-05	1.20E-03
cg00216061	ZFPM1	16	Body	Open sea	87.15	4.43	90.36	3.71	-3.21	3.74E-04	8.08E-03
cg26207372		7	Intergenic	Open sea	76.18	5.21	79.39	4.20	-3.21	3.72E-04	8.05E-03
cg23851027	ETFB;ETFB	19	1stExon;Body	Open sea	73.77	4.45	76.98	3.36	-3.21	1.81E-03	2.64E-02
cg04822456		4	Intergenic	Open sea	80.56	4.63	83.77	3.41	-3.21	3.41E-04	7.52E-03
cg02364826	FEM1C	5	Body	N_Shore	24.27	3.92	27.48	5.09	-3.21	1.59E-03	2.40E-02
cg12399860	BAT1;BAT1	6	TSS1500;TSS1500	S_Shore	65.33	4.22	68.53	3.49	-3.21	1.83E-04	4.61E-03
cg08148697	ZNF598	16	3'UTR	N_Shore	75.36	3.94	78.57	3.61	-3.21	2.44E-04	5.79E-03
cg03391776	RREB1;RREB1;RREB1;RREB1	6	5'UTR;5'UTR;5'UTR;5'UTR	Open sea	75.47	4.45	78.68	4.01	-3.21	1.44E-03	2.24E-02
cg01589587	BATF	14	Body	Open sea	13.57	3.29	16.78	3.92	-3.21	4.91E-04	9.99E-03
cg23225637	CANT1;CANT1;CANT1	17	Body;Body;Body	Island	88.85	5.73	92.06	3.69	-3.21	8.34E-04	1.49E-02
cg02836864		2	Intergenic	Open sea	55.92	5.20	59.13	5.59	-3.21	2.37E-04	5.67E-03
cg09200263	ATP6V0A4;ATP6V0A4;ATP6V0A4	7	Body;Body;Body	Open sea	62.11	4.29	65.32	3.82	-3.21	7.27E-04	1.35E-02
cg01418536	USP32	17	Body	N_Shelf	76.08	4.91	79.29	5.60	-3.21	7.27E-04	1.35E-02
cg15567297	RHCG	15	Body	N_Shore	64.44	5.16	67.64	4.48	-3.21	7.55E-04	1.39E-02
cg25671837	RSPO3	6	Body	Open sea	67.80	5.32	71.01	5.00	-3.21	6.37E-04	1.22E-02
cg07611334	NRSN2	20	5'UTR	Island	13.05	3.05	16.25	3.72	-3.21	1.24E-05	4.56E-04
cg17678847		4	Intergenic	Open sea	71.59	4.91	74.80	5.08	-3.21	1.46E-03	2.26E-02
cg10378521	FBRSL1	12	Body	N_Shore	88.02	5.61	91.23	2.70	-3.21	1.26E-03	2.03E-02
cg19389293	C18orf1;C18orf1;C18orf1;C18orf1;C18	18	Body;Body;Body;Body;Body;Body	Island	47.35	5.24	50.56	4.31	-3.21	3.69E-03	4.32E-02
cg13894444	LEPREL1;LEPREL1	3	5'UTR;Body	Open sea	78.40	4.76	81.61	3.41	-3.21	4.18E-04	8.82E-03
cg15660418	HOXC4;HOXC6;HOXC5	12	TSS1500;TSS1500;TSS1500	S_Shore	21.02	3.86	24.23	3.28	-3.21	6.05E-04	1.17E-02
cg21643649	XAB2	19	Body	N_Shore	64.31	3.09	67.51	2.93	-3.21	2.54E-05	8.65E-04
cg01714452	FYN;FYN;FYN	6	Body;Body;Body	Open sea	82.45	3.39	85.65	2.53	-3.21	1.46E-06	6.39E-05
cg10583893	ZNF808	19	5'UTR	Island	30.19	3.47	33.39	4.05	-3.21	1.49E-05	5.39E-04
cg00303183		2	Intergenic	N_Shore	27.19	2.60	30.40	3.22	-3.21	1.11E-05	4.14E-04
cg15846886	FAM195B;FAM195B	17	5'UTR;5'UTR	N_Shore	42.61	7.46	45.82	7.12	-3.21	2.16E-03	2.98E-02
cg06465175		7	Intergenic	N_Shore	47.40	3.83	50.60	4.07	-3.21	6.30E-04	1.21E-02
cg15367892	EMX2OS	10	Body	S_Shore	46.64	4.12	49.85	4.27	-3.21	1.35E-04	3.59E-03
cg19247124		1	Intergenic	Island	28.46	2.95	31.67	3.82	-3.21	2.99E-06	1.24E-04
cg16193278	UBAC2;MIR623;UBAC2;UBAC2	13	Body;Body;Body;Body	Open sea	49.08	7.14	52.28	5.97	-3.21	4.38E-03	4.85E-02
cg11649376	ACSS3	12	Body	S_Shore	26.24	4.25	29.45	4.06	-3.21	1.04E-04	2.89E-03
cg23970645	FOXN3;FOXN3	14	Body;Body	Open sea	76.69	5.84	79.90	4.56	-3.21	3.07E-03	3.81E-02
cg21422672	COL18A1	21	Body	N_Shelf	74.08	2.85	77.29	3.23	-3.21	3.09E-06	1.28E-04
cg02946697		8	Intergenic	Open sea	85.45	4.43	88.66	3.22	-3.21	1.61E-04	4.15E-03
cg11102098		10	Intergenic	N_Shelf	77.30	4.68	80.50	3.51	-3.21	1.05E-03	1.78E-02
cg03846960	JARID2	6	Body	Open sea	84.27	4.64	87.48	3.85	-3.21	7.68E-04	1.41E-02
cg18887483	ISLR2;LOC283731;ISLR2;ISLR2;ISLR2	15	5'UTR;TSS1500;TSS200;TSS200;5'UTR	Island	12.84	2.46	16.04	2.85	-3.20	5.21E-07	2.43E-05

cg24080291	NARS2;NARS2	11	Body;Body	N_Shelf	85.67	4.20	88.88	3.79	-3.20	2.68E-05	9.05E-04
cg17120792	OR5I1	11	TSS1500	Open sea	51.84	5.39	55.04	5.34	-3.20	2.51E-03	3.31E-02
cg25814795	C3orf25	3	Body	Open sea	81.59	5.02	84.79	3.73	-3.20	3.17E-03	3.89E-02
cg17105755	C2orf65	2	TSS200	Island	42.26	3.83	45.46	3.79	-3.20	4.55E-04	9.42E-03
cg21362478	PCSK5	9	Body	Island	13.48	3.66	16.69	5.76	-3.20	1.22E-04	3.31E-03
cg19636308	FLCN;FLCN	17	Body;Body	Island	84.13	4.01	87.33	2.66	-3.20	6.08E-05	1.84E-03
cg27398720	PITX2;PITX2;PITX2	4	Body;Body;Body	N_Shore	36.00	3.75	39.21	3.58	-3.20	5.91E-05	1.80E-03
cg13768953	DLG4;DLG4	17	Body;Body	N_Shelf	51.05	2.80	54.26	3.41	-3.20	6.58E-05	1.97E-03
cg07296841		12	Intergenic	Open sea	57.16	6.39	60.37	5.09	-3.20	4.55E-03	4.98E-02
cg07524398	CBX6	22	3'UTR	N_Shore	66.72	4.16	69.93	2.99	-3.20	3.02E-04	6.85E-03
cg01748892	HOXA5	7	TSS1500	Island	24.64	3.53	27.85	3.74	-3.20	1.11E-03	1.85E-02
cg14499532	FOXJ1	17	5'UTR	Island	12.97	2.20	16.17	3.40	-3.20	2.77E-06	1.16E-04
cg17978548		8	Intergenic	Open sea	57.92	4.65	61.12	4.36	-3.20	2.64E-03	3.43E-02
cg22846776	METT10D	17	Body	Open sea	71.09	4.07	74.29	2.93	-3.20	1.09E-04	3.02E-03
cg11156132		3	Intergenic	S_Shelf	46.28	3.31	49.48	3.26	-3.20	1.95E-04	4.84E-03
cg19488874		2	Intergenic	Island	17.44	2.37	20.64	3.34	-3.20	2.91E-07	1.39E-05
cg14251267	ZBTB46	20	Body	N_Shore	70.62	4.38	73.82	4.42	-3.20	8.55E-04	1.52E-02
cg04422003	CREB5	7	5'UTR	N_Shore	20.50	3.18	23.70	5.49	-3.20	7.60E-04	1.39E-02
cg08792995	SCML4	6	TSS200	Open sea	21.16	5.11	24.36	4.60	-3.20	7.66E-05	2.24E-03
cg27254661	SPR	2	Body	S_Shelf	61.69	4.05	64.89	3.82	-3.20	2.58E-03	3.38E-02
cg21374324	FLJ45340	7	Body	Open sea	78.88	4.44	82.08	3.94	-3.20	1.26E-03	2.03E-02
cg16596536	PTRH2	17	5'UTR	N_Shelf	81.55	4.32	84.75	4.19	-3.20	5.73E-05	1.75E-03
cg09233429	PLCB2	15	Body	Island	48.16	3.25	51.36	3.66	-3.20	1.04E-04	2.90E-03
cg19061112	NUAK1	12	Body	Open sea	37.55	4.12	40.75	3.69	-3.20	9.19E-04	1.61E-02
cg25720885		12	Intergenic	Open sea	60.24	3.61	63.44	3.77	-3.20	2.76E-04	6.38E-03
cg21750589	ZNF323;ZKSCAN3;ZNF323	6	5'UTR;5'UTR;5'UTR	Open sea	80.50	4.24	83.70	3.43	-3.20	2.14E-04	5.21E-03
cg17806162		1	Intergenic	Island	40.40	4.53	43.60	4.93	-3.20	3.80E-03	4.41E-02
cg16735495		7	Intergenic	Open sea	64.20	4.71	67.40	5.57	-3.20	5.92E-04	1.15E-02
cg20497968		11	Intergenic	Open sea	73.57	4.55	76.77	4.60	-3.20	9.60E-05	2.71E-03
cg06760518		2	Intergenic	Island	22.44	4.34	25.64	4.38	-3.20	3.07E-04	6.92E-03
cg19055323	KRTAP4-1	17	TSS1500	Open sea	54.56	6.05	57.76	4.62	-3.20	1.64E-03	2.45E-02
cg08134803		11	Intergenic	N_Shelf	79.62	4.94	82.82	3.52	-3.20	2.25E-03	3.07E-02
cg09240515	EPAS1	2	Body	Open sea	67.22	3.51	70.41	3.31	-3.20	3.07E-05	1.02E-03
cg17939889	HDAC4	2	Body	Open sea	45.12	5.03	48.31	4.35	-3.20	1.91E-03	2.74E-02
cg18527751	C18orf25;C18orf25	18	5'UTR;5'UTR	S_Shore	74.07	4.91	77.26	5.29	-3.20	7.41E-04	1.37E-02
cg00389446	CLRN2	4	TSS200	S_Shelf	82.92	4.16	86.12	3.41	-3.20	3.23E-04	7.21E-03
cg17108388	PLEKHA6	1	Body	Island	70.89	3.58	74.09	4.36	-3.20	1.52E-04	3.96E-03
cg09755872		7	Intergenic	Island	59.12	5.10	62.31	3.97	-3.20	1.13E-03	1.87E-02
cg24851490	RNASE2	14	5'UTR	Open sea	37.40	4.59	40.59	4.43	-3.20	1.65E-03	2.46E-02
cg05243389	MYEF2	15	TSS1500	S_Shore	31.40	4.23	34.59	5.54	-3.20	3.66E-04	7.95E-03
cg05394663	WNT3A	1	3'UTR	S_Shore	13.98	3.02	17.17	4.30	-3.20	1.14E-04	3.13E-03
cg26665785	SPDYE4	17	TSS1500	Open sea	61.31	4.87	64.50	4.39	-3.20	6.06E-04	1.18E-02
cg05904016	ADAP1	7	Body	S_Shelf	66.63	4.78	69.83	4.07	-3.20	1.12E-03	1.87E-02
cg00182758		17	Intergenic	S_Shore	17.76	4.47	20.96	5.66	-3.20	3.44E-04	7.57E-03
cg09782026	LOC154822	7	Body	Open sea	43.13	3.73	46.32	4.30	-3.20	6.35E-05	1.91E-03
cg04612349	ZNF207;ZNF207;ZNF207	17	Body;Body;Body	S_Shelf	43.28	4.74	46.47	4.52	-3.20	2.20E-03	3.02E-02
cg18196811		1	Intergenic	Open sea	81.43	4.40	84.63	3.46	-3.20	3.32E-04	7.38E-03
cg09245901		6	Intergenic	Open sea	71.64	4.53	74.84	5.13	-3.20	7.50E-04	1.38E-02
cg16072304		5	Intergenic	S_Shore	20.53	3.29	23.72	3.16	-3.20	2.23E-05	7.69E-04
cg07771160	ADAMTS5	21	Body	N_Shore	60.75	6.07	63.95	5.21	-3.20	2.22E-03	3.04E-02
cg07236912	PDLIM5;PDLIM5;PDLIM5;PDLIM5;PDL	4	Body;Body;Body;Body;Body	Open sea	41.99	4.78	45.19	4.30	-3.20	3.47E-03	4.14E-02
cg10658666	OTX1	2	TSS1500	Island	20.49	3.22	23.69	3.87	-3.20	5.36E-06	2.13E-04
cg20461196		19	Intergenic	Island	37.11	5.78	40.31	7.49	-3.20	6.95E-04	1.30E-02

cg17546623		10	Intergenic	Open sea	61.73	4.09	64.93	4.36	-3.20	1.78E-03	2.61E-02
cg10155725	ME3;ME3;ME3	11	Body;Body;Body	N_Shore	64.74	5.96	67.94	5.72	-3.20	2.12E-03	2.95E-02
cg22158992	NKD2	5	Body	N_Shelf	68.66	4.95	71.86	5.69	-3.20	4.50E-03	4.94E-02
cg20920549		13	Intergenic	Open sea	58.08	4.09	61.28	4.95	-3.20	3.80E-05	1.23E-03
cg01797257	PAX7;PAX7;PAX7	1	Body;Body;Body	Island	18.22	3.10	21.41	3.51	-3.20	3.90E-06	1.59E-04
cg13549760	RRAGA	9	TSS1500	N_Shore	74.15	3.00	77.35	2.85	-3.20	3.78E-06	1.54E-04
cg04052235		11	Intergenic	Open sea	34.18	5.01	37.37	5.32	-3.20	4.06E-03	4.61E-02
cg14891209	LOC145845	15	Body	N_Shelf	40.82	4.69	44.01	4.30	-3.19	1.59E-03	2.40E-02
cg01763890		5	Intergenic	Island	31.39	3.34	34.59	3.81	-3.19	6.07E-05	1.84E-03
cg08748308	LCP2	5	Body	Open sea	52.85	5.58	56.04	5.08	-3.19	4.45E-03	4.91E-02
cg03191384	MYO9B;MYO9B	19	Body;Body	S_Shore	53.70	2.73	56.89	2.45	-3.19	6.26E-07	2.89E-05
cg25472720	SEMA5A	5	Body	Open sea	85.13	4.44	88.32	3.15	-3.19	8.43E-04	1.51E-02
cg22517740	RHOU	1	TSS1500	N_Shore	18.93	3.99	22.12	3.82	-3.19	1.14E-03	1.89E-02
cg15787544	PRKAG2;PRKAG2	7	5'UTR;Body	Open sea	82.08	3.86	85.27	3.29	-3.19	8.74E-04	1.55E-02
cg25221496	MEIS2;MEIS2;MEIS2;MEIS2;MEIS2;ME	15	Body;Body;Body;Body;Body;Body;Body	N_Shore	83.57	3.67	86.76	2.81	-3.19	1.51E-04	3.94E-03
cg05938007	MRPL33;MRPL33	2	TSS1500;TSS1500	N_Shore	23.10	3.59	26.29	5.19	-3.19	1.11E-03	1.85E-02
cg04006397	NEU1	6	Body	N_Shelf	77.91	4.28	81.11	3.89	-3.19	6.14E-04	1.19E-02
cg03653137	RGL2;RGL2	6	Body;Body	N_Shelf	75.67	3.23	78.86	3.04	-3.19	9.74E-06	3.68E-04
cg17219656	ACTN2	1	Body	Open sea	76.63	4.54	79.82	3.87	-3.19	2.67E-03	3.45E-02
cg07071389	TSPYL3	20	Body	Island	41.53	5.84	44.72	6.23	-3.19	2.20E-03	3.02E-02
cg11471805	SHISA6	17	Body	Island	87.50	5.41	90.69	4.12	-3.19	1.98E-03	2.81E-02
cg02230495	HIBCH;HIBCH	2	Body;Body	Open sea	78.93	5.09	82.12	3.97	-3.19	3.22E-03	3.93E-02
cg14087083	C10orf26;C10orf26	10	3'UTR;3'UTR	Open sea	74.20	4.17	77.39	3.68	-3.19	4.98E-04	1.01E-02
cg15105326	ZIC4;ZIC4;ZIC4;ZIC4;ZIC4	3	Body;Body;Body;Body;Body	Island	21.31	4.03	24.50	3.57	-3.19	6.96E-04	1.30E-02
cg09176199	SMYD3;SMYD3	1	Body;Body	Open sea	72.18	5.51	75.37	3.86	-3.19	1.72E-03	2.54E-02
cg02726819	LOC154822	7	TSS200	S_Shore	56.65	4.26	59.85	3.38	-3.19	5.99E-04	1.16E-02
cg19711783	NTN1	17	Body	Island	42.67	5.66	45.86	6.53	-3.19	4.44E-03	4.90E-02
cg19498600	CCDC7;C10orf68;CCDC7	10	Body;TSS1500;Body	Open sea	76.43	4.84	79.62	5.33	-3.19	2.27E-03	3.08E-02
cg09495207	SLC12A4;SLC12A4;SLC12A4;SLC12A4;S	16	3'UTR;3'UTR;3'UTR;3'UTR;3'UTR;TSS1500	Open sea	43.79	3.88	46.98	4.74	-3.19	4.14E-03	4.67E-02
cg08310837	FAM65A	16	Body	S_Shore	42.36	5.18	45.55	5.19	-3.19	2.53E-04	5.96E-03
cg20794828	LY6G6C	6	Body	N_Shelf	60.47	5.26	63.66	5.02	-3.19	3.76E-03	4.37E-02
cg11172423	CLDN19;CLDN19	1	1stExon;1stExon	S_Shore	81.88	5.13	85.07	5.35	-3.19	2.72E-03	3.50E-02
cg18073688		3	Intergenic	Open sea	42.18	3.88	45.37	4.55	-3.19	1.53E-03	2.34E-02
cg03053750	APCDD1L	20	Body	S_Shelf	74.30	4.07	77.49	4.04	-3.19	4.06E-04	8.62E-03
cg05346256		12	Intergenic	Open sea	54.18	4.68	57.37	4.74	-3.19	3.99E-04	8.51E-03
cg18637901		4	Intergenic	N_Shelf	75.10	3.80	78.29	2.73	-3.19	1.73E-04	4.41E-03
cg15916004	GAL3ST3;GAL3ST3	11	5'UTR;1stExon	Island	19.38	3.30	22.57	3.06	-3.19	1.75E-05	6.22E-04
cg06700146	BCL6B;BCL6B	17	1stExon;5'UTR	Island	23.26	3.18	26.45	3.75	-3.19	2.01E-06	8.61E-05
cg01405107	LOC404266;LOC404266;LOC404266;H	17	Body;Body;Body;TSS1500;Body	N_Shore	16.08	3.77	19.27	3.80	-3.19	1.14E-03	1.89E-02
cg02193513		4	Intergenic	N_Shore	49.23	4.97	52.42	4.12	-3.19	5.55E-04	1.10E-02
cg13502474		2	Intergenic	Island	51.45	3.16	54.64	4.52	-3.19	1.13E-03	1.88E-02
cg01925594		8	Intergenic	N_Shore	82.30	5.75	85.49	5.41	-3.19	4.35E-03	4.83E-02
cg14301212	HCG9	6	Body	N_Shore	35.91	5.62	39.10	4.55	-3.19	2.28E-03	3.10E-02
cg23120061		2	Intergenic	N_Shore	31.33	3.91	34.52	4.78	-3.19	5.57E-04	1.10E-02
cg25149238		2	Intergenic	S_Shore	27.20	5.10	30.39	5.74	-3.19	3.32E-03	4.02E-02
cg05933189	CSNK1G1	15	TSS1500	S_Shore	59.90	4.83	63.09	4.41	-3.19	1.11E-03	1.85E-02
cg02556345	PLEKHA1;PLEKHA1	10	Body;Body	Open sea	71.64	4.15	74.83	3.21	-3.19	1.45E-04	3.81E-03
cg23153707	DLG4;DLG4	17	Body;TSS200	N_Shore	23.47	4.40	26.66	4.49	-3.19	4.58E-04	9.46E-03
cg07572052	HIST2H2AA4;HIST2H2AA3	1	TSS1500;TSS1500	N_Shore	36.21	5.39	39.40	6.08	-3.19	1.39E-03	2.18E-02
cg02106155		7	Intergenic	Open sea	84.12	4.93	87.31	2.78	-3.19	1.35E-04	3.57E-03
cg10626728		3	Intergenic	S_Shelf	63.75	4.17	66.94	3.97	-3.19	1.22E-03	1.98E-02
cg03185622	DLGAP2	8	Body	N_Shore	68.91	4.02	72.10	4.64	-3.19	2.21E-03	3.03E-02
cg13518270	SALL4	20	Body	N_Shore	65.45	5.44	68.64	6.03	-3.19	2.62E-03	3.41E-02

cg09278187	FOXJ3	1	3'UTR	Open sea	68.39	5.34	71.57	3.79	-3.19	2.57E-03	3.36E-02
cg10955995	PPARGC1A	4	Body	Open sea	60.08	7.21	63.27	6.65	-3.19	2.87E-03	3.63E-02
cg00143308	RRM2B	8	3'UTR	Open sea	77.57	5.69	80.76	5.85	-3.19	3.63E-03	4.27E-02
cg11735846	AJAP1;AJAP1	1	Body;Body	N_Shore	9.89	2.36	13.08	3.40	-3.19	7.46E-07	3.41E-05
cg25574603	GSG1;GSG1	12	TSS200;TSS200	S_Shelf	63.22	4.33	66.41	4.86	-3.19	2.84E-04	6.52E-03
cg08019736		11	Intergenic	N_Shore	19.50	3.25	22.68	3.91	-3.19	6.39E-05	1.92E-03
cg21746120	LRP5	11	Body	Open sea	21.19	3.74	24.38	4.50	-3.19	1.43E-03	2.23E-02
cg14173662	TNXB	6	Body	N_Shelf	71.10	4.06	74.28	3.68	-3.19	2.60E-04	6.08E-03
cg02480320		5	Intergenic	N_Shore	49.31	5.21	52.50	4.53	-3.19	1.18E-03	1.93E-02
cg27057027		7	Intergenic	N_Shore	43.94	5.45	47.13	5.88	-3.19	1.60E-03	2.41E-02
cg13487037	SRI;SRI	7	Body;Body	Open sea	74.85	2.69	78.04	2.56	-3.19	1.53E-07	7.53E-06
cg06800942	C2orf81;C2orf81	2	1stExon;5'UTR	N_Shore	61.53	3.86	64.72	4.80	-3.19	1.71E-03	2.53E-02
cg12636435		5	Intergenic	S_Shore	30.58	3.40	33.77	3.74	-3.19	5.07E-04	1.02E-02
cg14015656	KIAA1529	9	Body	Island	19.01	3.80	22.20	4.35	-3.19	4.28E-03	4.78E-02
cg26400835	RFX4;RFX4;RFX4	12	Body;Body;Body	Open sea	78.91	4.61	82.10	4.59	-3.19	2.82E-03	3.59E-02
cg24806812	GC	4	Body	Open sea	83.93	3.65	87.12	2.82	-3.19	1.04E-05	3.91E-04
cg00830283	PDZK1	1	5'UTR	Open sea	65.11	5.12	68.29	4.42	-3.19	1.07E-03	1.80E-02
cg20331595	NRSN2;NRSN2	20	1stExon;5'UTR	Island	11.38	2.07	14.56	3.43	-3.19	9.58E-07	4.31E-05
cg06806214	FBXO31;FBXO31	16	Body;Body	Open sea	87.74	3.58	90.92	2.39	-3.19	2.84E-05	9.49E-04
cg13771313	ATG16L2	11	Body	Island	14.59	3.19	17.77	4.00	-3.19	7.70E-04	1.41E-02
cg21584759	NFIC;NFIC	19	Body;Body	Island	67.79	3.41	70.98	3.54	-3.19	1.66E-04	4.25E-03
cg08432712	UBA2	19	Body	S_Shelf	59.60	4.91	62.79	4.21	-3.19	2.75E-03	3.53E-02
cg12975597		6	Intergenic	Open sea	61.66	5.09	64.84	5.23	-3.19	4.06E-03	4.61E-02
cg05377165	FOXN3;FOXN3	14	3'UTR;3'UTR	N_Shore	92.14	1.70	95.32	0.86	-3.19	2.49E-18	1.74E-16
cg14121845	NINL	20	TSS1500	Island	25.63	5.27	28.81	7.03	-3.18	1.58E-03	2.39E-02
cg13627062		15	Intergenic	N_Shore	28.00	4.28	31.18	4.57	-3.18	1.44E-03	2.24E-02
cg06659791	ZNF354C	5	5'UTR	S_Shore	67.87	4.67	71.05	4.10	-3.18	1.43E-03	2.22E-02
cg21638625	FSD1;TMIGD2;TMIGD2	19	TSS1500;TSS1500;TSS1500	N_Shore	53.72	5.35	56.90	5.13	-3.18	4.00E-03	4.56E-02
cg22300615		20	Intergenic	S_Shelf	82.63	4.45	85.81	2.96	-3.18	1.03E-03	1.75E-02
cg11502921		1	Intergenic	Open sea	69.10	3.02	72.28	2.81	-3.18	1.07E-05	4.00E-04
cg25784280	DTNA;DTNA;DTNA;DTNA;DTNA	18	5'UTR;TSS1500;5'UTR;5'UTR;5'UTR	Open sea	66.28	5.68	69.46	7.09	-3.18	5.37E-04	1.07E-02
cg12469001		7	Intergenic	Open sea	51.76	4.27	54.94	4.56	-3.18	1.10E-04	3.02E-03
cg03700230		7	Intergenic	Open sea	40.62	6.38	43.81	4.84	-3.18	3.70E-03	4.33E-02
cg27424692	MAP4K4;MAP4K4;MAP4K4	2	Body;Body;Body	Open sea	75.15	4.71	78.33	4.53	-3.18	2.92E-03	3.68E-02
cg01283300	LIMK2;LIMK2;LIMK2	22	TSS200;TSS200;Body	Open sea	47.46	3.65	50.64	3.94	-3.18	1.25E-05	4.59E-04
cg12460254	ECE1	1	Body	Open sea	57.09	3.55	60.27	3.62	-3.18	3.52E-04	7.72E-03
cg02955020	PBX1	1	Body	Open sea	88.80	2.95	91.99	1.91	-3.18	4.40E-08	2.25E-06
cg02329358		7	Intergenic	Open sea	46.24	4.59	49.42	4.84	-3.18	1.80E-03	2.63E-02
cg18570800	ZMYND11;ZMYND11;ZMYND11	10	Body;Body;Body	Open sea	86.21	4.04	89.39	2.92	-3.18	2.69E-05	9.08E-04
cg04571130	COPG	3	TSS1500	N_Shore	24.11	4.29	27.29	4.06	-3.18	3.41E-03	4.10E-02
cg00044995	C2CD4C	19	5'UTR	Island	26.46	3.85	29.65	4.77	-3.18	1.94E-04	4.83E-03
cg10644916	KLHL17	1	Body	Island	69.65	4.69	72.83	3.87	-3.18	3.64E-03	4.28E-02
cg19978242	INPP5F;INPP5F	10	Body;Body	S_Shore	76.81	4.07	80.00	3.56	-3.18	4.85E-04	9.89E-03
cg15623339		6	Intergenic	N_Shelf	62.95	3.93	66.13	4.12	-3.18	4.99E-04	1.01E-02
cg25590826	CCDC33	15	Body	Open sea	25.06	3.95	28.24	5.26	-3.18	1.61E-03	2.43E-02
cg05767143	PITPNM3;PITPNM3	17	Body;Body	Open sea	84.23	4.06	87.41	3.61	-3.18	1.40E-04	3.69E-03
cg08350797	DUSP7	3	Body	Open sea	89.24	5.52	92.42	3.03	-3.18	1.94E-03	2.77E-02
cg08110610	SAG	2	TSS200	Open sea	81.06	3.77	84.24	3.07	-3.18	2.05E-04	5.03E-03
cg10855081	EML6	2	Body	Island	17.71	2.67	20.89	3.44	-3.18	2.62E-05	8.87E-04
cg00695760		1	Intergenic	N_Shore	46.77	4.09	49.95	3.99	-3.18	1.42E-03	2.22E-02
cg19352038	PAX3;PAX3;PAX3;PAX3;PAX3;CCDC14(	2	TSS1500;TSS1500;TSS1500;TSS1500;TSS15	S_Shore	21.25	3.24	24.43	4.57	-3.18	1.25E-04	3.37E-03
cg14962628	C4orf40	4	Body	Open sea	83.09	4.62	86.27	3.35	-3.18	1.33E-03	2.12E-02
cg20808693	SMARCD3;SMARCD3	7	Body;Body	S_Shelf	65.22	4.28	68.40	4.63	-3.18	1.28E-03	2.06E-02

cg09346118	C1orf61;MIR9-1	1	5'UTR;TSS1500	Island	38.30	4.62	41.48	4.81	-3.18	6.40E-04	1.23E-02
cg17265790	ADRA1B	5	Body	N_Shelf	55.31	3.78	58.49	4.45	-3.18	6.40E-04	1.23E-02
cg03204399		7	Intergenic	S_Shelf	84.78	5.26	87.96	5.35	-3.18	3.91E-04	8.36E-03
cg22049732	SRF	6	Body	S_Shore	5.38	1.77	8.56	3.39	-3.18	7.29E-08	3.68E-06
cg26303165	OBSCN;OBSCN	1	Body;Body	Island	40.28	4.89	43.46	5.48	-3.18	2.36E-03	3.17E-02
cg02746658	CMIP;CMIP;CMIP	16	1stExon;5'UTR;Body	Open sea	18.99	3.56	22.17	3.47	-3.18	1.89E-04	4.71E-03
cg19653967	SLC1A2	11	Body	Open sea	66.23	4.09	69.41	4.05	-3.18	1.04E-04	2.90E-03
cg03190876	NXNL1	19	Body	Island	27.29	3.45	30.47	4.08	-3.18	4.66E-05	1.46E-03
cg00319774	DUSP26	8	5'UTR	N_Shore	51.93	5.73	55.11	5.33	-3.18	2.44E-03	3.25E-02
cg14111332		19	Intergenic	Island	49.58	5.38	52.76	4.55	-3.18	3.24E-03	3.95E-02
cg04767756	EMP2	16	5'UTR	Open sea	28.09	3.68	31.27	4.01	-3.18	2.05E-03	2.88E-02
cg20802214	FBXL13;FBXL13	7	Body;Body	Open sea	82.69	5.04	85.86	3.78	-3.18	2.98E-03	3.73E-02
cg22504175	CLCN1	7	Body	Island	38.19	6.06	41.37	7.60	-3.18	2.67E-03	3.45E-02
cg17980570	SEC63	6	Body	N_Shelf	63.62	2.86	66.80	3.37	-3.18	9.11E-06	3.46E-04
cg10502231		1	Intergenic	Open sea	36.56	4.52	39.74	5.26	-3.18	2.65E-03	3.44E-02
cg01142969	RHOV	15	Body	N_Shore	21.16	4.52	24.33	4.86	-3.18	1.92E-03	2.75E-02
cg03928874	LY86	6	3'UTR	Open sea	44.96	4.81	48.14	4.10	-3.18	9.64E-05	2.72E-03
cg17029120	XPOT	12	3'UTR	N_Shelf	83.92	5.36	87.10	5.74	-3.18	2.51E-03	3.31E-02
cg14644846	EEFSEC	3	Body	Open sea	57.47	4.60	60.65	4.73	-3.18	9.46E-04	1.64E-02
cg04348222	MARCKSL1	1	TSS1500	S_Shore	54.27	3.94	57.44	4.45	-3.18	3.73E-04	8.06E-03
cg02849695	CCDC19	1	TSS200	Island	18.26	3.95	21.44	4.73	-3.18	3.27E-03	3.97E-02
cg13023014	STOX2	4	Body	Open sea	70.56	5.43	73.74	4.40	-3.18	3.75E-03	4.36E-02
cg19761304	GDF1;LASS1	19	Body;3'UTR	Island	46.54	3.56	49.72	5.02	-3.18	3.73E-03	4.35E-02
cg09326409	ITGAM;ITGAM	16	Body;Body	Island	21.38	4.34	24.56	5.04	-3.18	2.13E-03	2.96E-02
cg20136009	PPFIBP1;PPFIBP1	12	5'UTR;5'UTR	Open sea	81.64	4.94	84.81	4.53	-3.18	7.58E-04	1.39E-02
cg10234998	GABBR1;GABBR1	6	TSS1500;TSS1500	S_Shore	16.55	4.18	19.72	4.07	-3.18	1.37E-03	2.16E-02
cg05778440	LOC440461	17	Body	N_Shore	24.75	3.94	27.93	5.04	-3.18	3.18E-03	3.90E-02
cg25521400	B3GNT2	2	5'UTR	Open sea	56.03	4.13	59.21	4.55	-3.18	2.57E-03	3.36E-02
cg11723850		14	Intergenic	Island	25.81	4.85	28.99	5.68	-3.18	8.92E-05	2.55E-03
cg09183671	LOC282997;PDCD4;PDCD4	10	TSS200;TSS1500;TSS1500	N_Shore	59.63	4.71	62.80	3.86	-3.18	6.09E-04	1.18E-02
cg22187354	NRXN2;NRXN2	11	Body;Body	Island	16.47	4.57	19.64	5.81	-3.18	1.34E-03	2.13E-02
cg05025332		2	Intergenic	Open sea	34.78	3.95	37.96	5.13	-3.18	3.85E-03	4.44E-02
cg24584367	GPATCH4;GPATCH4	1	Body;Body	S_Shelf	76.47	4.25	79.64	2.53	-3.18	7.79E-06	3.00E-04
cg14975881	PRKCG	19	Body	N_Shelf	45.41	5.10	48.58	4.47	-3.17	6.22E-04	1.20E-02
cg21105620	HGFAC	4	Body	Island	69.60	4.10	72.77	3.56	-3.17	5.91E-04	1.15E-02
cg25898111		10	Intergenic	Open sea	79.41	4.70	82.58	5.19	-3.17	2.90E-03	3.66E-02
cg02796568	SYNE1;SYNE1	6	Body;Body	Open sea	68.81	4.42	71.98	5.31	-3.17	9.27E-04	1.62E-02
cg17451176	PCDHGA4;PCDHGA2;PCDHGB2;PCDH	5	Body;Body;Body;TSS200;Body;Body;TSS2	N_Shore	16.35	3.77	19.52	3.77	-3.17	1.42E-04	3.73E-03
cg03886085	MUPCDH;MUPCDH	11	Body;Body	N_Shelf	83.04	3.62	86.21	2.55	-3.17	5.55E-06	2.20E-04
cg06544989	UNC84B	22	3'UTR	Open sea	75.90	3.56	79.08	4.01	-3.17	4.42E-04	9.21E-03
cg23618477		6	Intergenic	S_Shelf	48.22	5.25	51.40	5.07	-3.17	1.87E-03	2.70E-02
cg10779492		1	Intergenic	Island	16.71	4.08	19.89	4.76	-3.17	2.39E-03	3.20E-02
cg05238522	SLC4A4;SLC4A4;SLC4A4	4	Body;Body;Body	Open sea	86.83	3.96	90.00	2.68	-3.17	1.51E-05	5.44E-04
cg09871315	HOXA2	7	TSS1500	N_Shore	23.44	4.10	26.62	3.61	-3.17	4.17E-04	8.81E-03
cg26182317		10	Intergenic	Open sea	84.17	3.27	87.34	2.43	-3.17	1.96E-06	8.41E-05
cg05619622	MTA2	11	Body	N_Shelf	77.45	4.11	80.62	3.43	-3.17	7.96E-04	1.44E-02
cg05925811		5	Intergenic	N_Shore	91.77	5.52	94.94	1.73	-3.17	5.70E-05	1.74E-03
cg07643096	DCBLD1	6	TSS1500	N_Shore	60.27	6.40	63.45	5.78	-3.17	1.65E-03	2.47E-02
cg22053720	PTK7;PTK7;PTK7;PTK7	6	Body;Body;Body;Body	Open sea	68.01	4.39	71.18	3.90	-3.17	8.22E-04	1.48E-02
cg06069685	C18orf20	18	Body	Open sea	75.03	3.53	78.20	3.18	-3.17	3.93E-06	1.60E-04
cg17385549	PTPRN2;PTPRN2;PTPRN2	7	Body;Body;Body	N_Shore	82.14	2.73	85.31	2.53	-3.17	4.34E-07	2.05E-05
cg00155526	NPR3	5	Body	Island	16.26	5.63	19.43	7.08	-3.17	2.53E-03	3.33E-02
cg11710211	ZNF787	19	Body	Island	75.23	4.12	78.40	4.77	-3.17	2.52E-03	3.32E-02

cg04565740		10	Intergenic	N_Shore	35.43	3.90	38.60	5.07	-3.17	3.74E-04	8.08E-03
cg13941830		1	Intergenic	Island	18.20	3.83	21.37	3.97	-3.17	8.63E-05	2.48E-03
cg02707152	SLC22A20	11	TSS1500	Open sea	52.44	4.90	55.61	4.51	-3.17	1.46E-03	2.26E-02
cg15256305	SRCIN1	17	Body	Island	51.53	3.64	54.70	3.61	-3.17	5.52E-04	1.09E-02
cg06301550	C20orf95	20	TSS200	N_Shore	18.30	3.20	21.47	3.44	-3.17	2.17E-05	7.52E-04
cg01258522	GFI1;GFI1;GFI1	1	5'UTR;5'UTR;TSS1500	Island	24.81	3.92	27.98	4.15	-3.17	5.58E-05	1.71E-03
cg24586758	PCDHB3	5	TSS200	N_Shore	19.31	4.71	22.48	4.64	-3.17	4.28E-03	4.78E-02
cg03093897	CRAMP1L	16	3'UTR	N_Shelf	74.72	5.00	77.89	3.15	-3.17	4.14E-03	4.67E-02
cg02598807	AP2A2	11	Body	S_Shore	67.68	4.97	70.85	4.37	-3.17	3.42E-03	4.10E-02
cg08010865	SPATA13;SPATA13	13	Body;5'UTR	Open sea	72.97	6.49	76.14	6.26	-3.17	1.34E-03	2.13E-02
cg04788390	ARHGEF10L;ARHGEF10L	1	Body;Body	Open sea	57.23	4.34	60.39	3.91	-3.17	1.25E-04	3.36E-03
cg17381305		6	Intergenic	Open sea	81.99	5.59	85.16	4.32	-3.17	2.42E-03	3.23E-02
cg01061480		6	Intergenic	Open sea	81.85	4.42	85.02	3.55	-3.17	6.27E-04	1.21E-02
cg12411858	MED15;MED15	22	Body;Body	S_Shore	75.09	4.33	78.26	3.64	-3.17	4.99E-04	1.01E-02
cg06889975		8	Intergenic	N_Shelf	83.92	5.13	87.09	4.52	-3.17	2.71E-03	3.49E-02
cg20935363	PDE2A;PDE2A;PDE2A;PDE2A	11	Body;Body;Body;Body	Island	32.11	4.89	35.27	5.32	-3.17	1.75E-03	2.57E-02
cg24642523	HSPA2	14	1stExon	S_Shore	21.70	3.55	24.87	4.96	-3.17	7.33E-04	1.36E-02
cg03938043	INTS7;DTL	1	Body;TSS1500	N_Shore	28.22	3.63	31.39	4.28	-3.17	2.65E-03	3.44E-02
cg20623645	NAGLU	17	TSS1500	N_Shore	27.83	3.71	30.99	4.14	-3.17	2.44E-03	3.25E-02
cg14021478	CUTA;CUTA;CUTA;CUTA;CUTA	6	Body;Body;Body;Body;Body	N_Shore	56.12	3.74	59.29	4.30	-3.17	2.21E-05	7.63E-04
cg15653254		2	Intergenic	Open sea	43.66	6.13	46.82	5.13	-3.17	1.04E-03	1.76E-02
cg06777902	FANK1	10	Body	Open sea	20.41	4.55	23.58	4.55	-3.17	2.11E-03	2.93E-02
cg11193419		8	Intergenic	Island	81.53	4.70	84.70	3.10	-3.17	2.00E-04	4.95E-03
cg07907745	FLJ45983;GATA3;GATA3;FLJ45983	10	TSS200;TSS1500;TSS1500;TSS200	Island	14.80	2.51	17.96	4.46	-3.17	8.88E-05	2.54E-03
cg06023791	ADAM33;ADAM33	20	Body;Body	Island	12.60	3.50	15.76	4.74	-3.17	5.45E-04	1.08E-02
cg23497529		14	Intergenic	N_Shelf	67.41	3.47	70.58	3.37	-3.17	2.52E-04	5.94E-03
cg07342322		1	Intergenic	Open sea	44.11	5.79	47.28	6.43	-3.17	2.43E-03	3.24E-02
cg19696388	HOMER3;HOMER3;HOMER3;HOMER3	19	Body;Body;Body;Body;Body	Island	80.07	4.98	83.23	3.95	-3.17	4.07E-03	4.62E-02
cg01961025		12	Intergenic	S_Shelf	75.96	3.99	79.13	3.72	-3.17	3.05E-04	6.88E-03
cg03859028		15	Intergenic	Open sea	51.66	4.87	54.83	4.73	-3.17	3.83E-04	8.24E-03
cg24362026		7	Intergenic	Open sea	73.53	5.63	76.70	5.74	-3.17	3.35E-03	4.04E-02
cg09916783	POMC;POMC	2	TSS1500;TSS1500	S_Shore	38.63	4.94	41.79	4.56	-3.17	1.08E-03	1.81E-02
cg15842431	HIST1H4K	6	TSS1500	S_Shore	61.34	4.22	64.50	5.69	-3.17	2.28E-03	3.10E-02
cg22571664	NPR3	5	Body	Island	31.83	6.52	34.99	6.74	-3.17	3.08E-03	3.81E-02
cg03665229	CNOT1	16	Body	Open sea	71.65	3.31	74.82	4.15	-3.17	3.11E-04	6.99E-03
cg25939793	NRG2;NRG2;NRG2;NRG2	5	Body;Body;Body;Body	N_Shore	16.83	4.53	19.99	5.09	-3.16	1.28E-03	2.05E-02
cg01624068		13	Intergenic	N_Shelf	53.25	3.11	56.41	3.41	-3.16	1.28E-04	3.42E-03
cg02617560		8	Intergenic	Open sea	54.68	4.61	57.84	4.01	-3.16	3.47E-04	7.62E-03
cg10501629	ATP2B1;ATP2B1	12	1stExon;1stExon	Open sea	86.26	3.74	89.42	3.04	-3.16	1.45E-04	3.80E-03
cg18117347	SHANK1	19	Body	N_Shore	15.61	3.69	18.77	3.51	-3.16	3.06E-05	1.02E-03
cg11579630	CCNG2	4	Body	S_Shelf	74.95	5.14	78.11	5.85	-3.16	1.43E-03	2.23E-02
cg05937532		16	Intergenic	Island	55.53	4.14	58.70	4.90	-3.16	2.13E-03	2.95E-02
cg23819016		13	Intergenic	Open sea	23.28	4.08	26.45	5.09	-3.16	3.53E-03	4.19E-02
cg03517913		3	Intergenic	Open sea	80.61	3.40	83.77	3.72	-3.16	1.42E-04	3.73E-03
cg04444949	AGTRAP;AGTRAP;AGTRAP;AGTRAP;AG	1	TSS1500;TSS1500;TSS1500;TSS1500;TSS15	N_Shore	27.29	3.95	30.46	3.83	-3.16	7.69E-04	1.41E-02
cg19279310		11	Intergenic	N_Shelf	54.44	4.56	57.60	3.82	-3.16	4.02E-04	8.55E-03
cg06135765	GOLGA4	3	TSS1500	N_Shore	84.93	4.30	88.09	3.16	-3.16	2.20E-03	3.02E-02
cg07257747	ADAM19	5	Body	Open sea	79.86	4.78	83.03	3.93	-3.16	1.80E-03	2.62E-02
cg04757901	TNXB	6	Body	N_Shelf	66.18	5.74	69.34	6.02	-3.16	4.48E-03	4.93E-02
cg22301418	ATP11A;ATP11A	13	Body;Body	Open sea	69.71	4.80	72.88	4.05	-3.16	1.20E-03	1.96E-02
cg25482091		2	Intergenic	Open sea	80.34	5.07	83.50	3.94	-3.16	4.15E-03	4.68E-02
cg11052958	DNAH11	7	Body	Island	15.82	3.79	18.98	4.10	-3.16	3.43E-04	7.56E-03
cg13798746	TNXB	6	Body	Open sea	78.31	3.82	81.47	4.07	-3.16	3.06E-04	6.90E-03

cg10332003	ENPP6	4	Body	Open sea	76.19	5.35	79.36	4.61	-3.16	2.02E-03	2.85E-02
cg09568483	PHF17;PHF17	4	Body;Body	Open sea	30.18	3.09	33.34	4.32	-3.16	8.87E-04	1.57E-02
cg07637658	PPP1R13L;PPP1R13L	19	Body;Body	N_Shore	60.27	3.55	63.43	3.61	-3.16	7.72E-05	2.25E-03
cg01460382	IQSEC1;IQSEC1	3	Body;Body	N_Shore	83.17	3.70	86.33	2.67	-3.16	5.92E-05	1.80E-03
cg07534892		12	Intergenic	Open sea	80.41	4.93	83.58	4.44	-3.16	1.50E-03	2.30E-02
cg03893395	C6orf123	6	Body	Open sea	80.64	4.28	83.80	4.63	-3.16	1.09E-03	1.82E-02
cg00522276		8	Intergenic	N_Shore	19.85	3.52	23.01	4.10	-3.16	2.05E-05	7.14E-04
cg24393700		12	Intergenic	N_Shore	79.44	4.55	82.60	4.76	-3.16	3.68E-03	4.31E-02
cg26628847	PIP	7	Body	Open sea	47.57	4.30	50.73	4.82	-3.16	1.79E-04	4.53E-03
cg00145118	GNPDA1	5	TSS1500	S_Shore	14.48	3.13	17.64	4.77	-3.16	2.80E-03	3.57E-02
cg02487061	C1orf52;C1orf52	1	Body;Body	N_Shelf	52.39	4.73	55.55	4.99	-3.16	3.14E-03	3.86E-02
cg25836271	RASGRP2;RASGRP2;RASGRP2;RASGRI	11	1stExon;TSS1500;TSS1500;5'UTR	Island	18.79	3.70	21.95	4.88	-3.16	1.18E-05	4.37E-04
cg27067781	PRRT1	6	3'UTR	Island	25.83	4.60	28.99	4.85	-3.16	3.09E-03	3.82E-02
cg11544106		11	Intergenic	N_Shelf	66.90	4.65	70.06	3.65	-3.16	1.10E-03	1.84E-02
cg00133403	FAM192A	16	5'UTR	Open sea	85.86	3.91	89.02	2.12	-3.16	3.61E-05	1.17E-03
cg22340747		15	Intergenic	Open sea	82.14	4.37	85.30	3.37	-3.16	6.25E-04	1.20E-02
cg26104591	SLC38A10;SLC38A10	17	Body;Body	N_Shore	73.40	4.22	76.56	4.24	-3.16	4.68E-04	9.62E-03
cg13780065	UBTF;UBTF;UBTF	17	Body;Body;Body	Island	55.14	3.57	58.30	3.57	-3.16	2.04E-04	5.02E-03
cg15036874	MAPK11	22	Body	Island	43.72	4.00	46.87	4.06	-3.16	7.64E-04	1.40E-02
cg26271435		1	Intergenic	Open sea	35.61	5.41	38.77	5.74	-3.16	1.25E-03	2.02E-02
cg19047158		2	Intergenic	Open sea	83.23	3.97	86.39	3.28	-3.16	2.51E-04	5.93E-03
cg12843130	CAMK2B;CAMK2B;CAMK2B;CAMK2B;	7	Body;Body;Body;Body;Body;Body;Body;Bo	Island	56.65	4.14	59.81	4.87	-3.16	1.37E-03	2.16E-02
cg11265874		3	Intergenic	N_Shelf	60.73	5.28	63.89	4.64	-3.16	3.83E-03	4.43E-02
cg06002516	R3HDM2	12	Body	Open sea	78.42	4.48	81.58	3.50	-3.16	1.29E-03	2.07E-02
cg01101522	NKD2	5	Body	N_Shore	59.56	4.74	62.72	4.72	-3.16	4.00E-03	4.56E-02
cg15789106	RASSF7;RASSF7;RASSF7	11	3'UTR;3'UTR;3'UTR	N_Shelf	55.43	4.01	58.59	4.27	-3.16	5.03E-04	1.02E-02
cg16247184	CCDC46;CCDC46;CCDC46	17	Body;1stExon;5'UTR	Open sea	72.20	4.00	75.36	4.21	-3.16	1.05E-04	2.92E-03
cg16116663	PAIP2B	2	Body	Open sea	47.69	3.22	50.84	3.27	-3.16	7.61E-05	2.22E-03
cg21794419	MCF2L2;B3GNT5	3	Body;5'UTR	S_Shore	86.61	7.25	89.77	4.54	-3.16	9.77E-04	1.68E-02
cg07795016	NOS3	7	Body	S_Shore	60.54	3.54	63.69	3.93	-3.16	9.63E-04	1.67E-02
cg17008454	TNFAIP8;TNFAIP8	5	Body;Body	Open sea	59.74	4.47	62.90	3.83	-3.16	3.05E-04	6.90E-03
cg01794555	LCP1	13	5'UTR	Open sea	32.28	4.59	35.44	6.14	-3.16	1.11E-03	1.85E-02
cg07313162		5	Intergenic	Open sea	53.10	4.20	56.25	4.06	-3.16	4.35E-04	9.10E-03
cg14787093	DGKZ;DGKZ;DGKZ;DGKZ	11	Body;Body;Body;Body	N_Shore	74.25	3.93	77.41	2.99	-3.16	4.53E-04	9.38E-03
cg09135065	IQCE;IQCE	7	Body;Body	Island	76.70	4.64	79.86	4.48	-3.16	7.64E-04	1.40E-02
cg17508816		8	Intergenic	Open sea	66.12	4.48	69.28	4.93	-3.16	2.37E-04	5.65E-03
cg13097800		14	Intergenic	Open sea	41.39	4.41	44.54	4.34	-3.15	3.64E-04	7.92E-03
cg06675531	SYNPO;SYNPO;SYNPO;SYNPO	5	TSS1500;Body;Body;TSS1500	Open sea	75.54	3.60	78.69	4.50	-3.15	1.31E-03	2.09E-02
cg05300029		14	Intergenic	N_Shore	84.93	4.39	88.08	3.75	-3.15	1.37E-03	2.16E-02
cg10049789	SH3TC1	4	5'UTR	Open sea	56.28	5.97	59.44	5.24	-3.15	1.67E-03	2.48E-02
cg00691906		6	Intergenic	Open sea	67.05	5.10	70.20	4.50	-3.15	1.82E-03	2.64E-02
cg00221658		7	Intergenic	S_Shelf	46.29	5.52	49.45	6.18	-3.15	4.11E-03	4.65E-02
cg10848272	SLC8A3;SLC8A3;SLC8A3;SLC8A3	14	5'UTR;5'UTR;5'UTR;5'UTR	N_Shore	36.47	4.47	39.62	5.10	-3.15	2.31E-04	5.55E-03
cg15666214		6	Intergenic	Island	29.99	4.14	33.14	3.74	-3.15	1.50E-04	3.92E-03
cg21122225		2	Intergenic	Open sea	22.94	3.81	26.09	4.92	-3.15	4.79E-04	9.80E-03
cg15552249	SPARCL1;SPARCL1	4	TSS200;TSS200	Open sea	75.40	4.72	78.55	4.13	-3.15	6.57E-04	1.25E-02
cg08965655	NXN	17	Body	N_Shore	72.61	7.56	75.77	8.22	-3.15	4.37E-03	4.85E-02
cg22152492	SCAND3	6	1stExon	N_Shore	75.38	4.13	78.53	3.32	-3.15	2.62E-03	3.41E-02
cg13721134	CA3	8	TSS1500	N_Shore	14.59	3.50	17.74	4.70	-3.15	7.59E-05	2.22E-03
cg27589003	MEST;MEST;MESTIT1;MEST	7	5'UTR;5'UTR;TSS1500;Body	Island	34.38	2.93	37.53	3.46	-3.15	6.79E-05	2.02E-03
cg26137121	C8ORFK29	8	TSS200	S_Shore	85.53	3.78	88.68	2.35	-3.15	6.82E-05	2.03E-03
cg27553955	KCNG3;KCNG3	2	1stExon;1stExon	Island	34.10	3.20	37.26	3.40	-3.15	3.95E-05	1.27E-03
cg22250390	EDEM1	3	Body	Open sea	82.87	3.98	86.02	2.96	-3.15	1.79E-04	4.52E-03

cg23200506	APOA4	11	TSS200	S_Shore	46.16	4.02	49.32	5.27	-3.15	1.46E-03	2.26E-02
cg16163140	ADAMTS16	5	Body	Open sea	24.12	4.57	27.27	5.49	-3.15	1.21E-03	1.97E-02
cg22213386	NIPAL4	5	TSS200	Island	15.02	3.48	18.17	3.53	-3.15	3.83E-07	1.81E-05
cg18948488	FLJ41350	10	Body	S_Shore	25.52	3.51	28.68	4.26	-3.15	2.47E-04	5.85E-03
cg05957543	ZBTB39	12	Body	N_Shelf	84.39	3.75	87.54	3.53	-3.15	6.32E-05	1.90E-03
cg02519208	CLDN16	3	TSS200	Open sea	55.37	3.71	58.53	3.47	-3.15	5.30E-05	1.63E-03
cg19371526	INS-IGF2;IGF2AS;IGF2;IGF2AS;IG	11	Body;TSS1500;Body;5'UTR;TSS1500;TSS15	Island	54.92	4.50	58.07	4.68	-3.15	1.63E-03	2.44E-02
cg02927074	ARNT2	15	Body	Open sea	19.04	3.63	22.19	4.91	-3.15	3.17E-03	3.88E-02
cg01597882	TRIM2	4	5'UTR	S_Shelf	63.65	5.01	66.80	5.46	-3.15	1.76E-03	2.59E-02
cg09547119	ZNF577;ZNF577;ZNF577	19	TSS200;TSS200;TSS200	Island	20.06	4.24	23.21	4.48	-3.15	5.27E-04	1.05E-02
cg18682277		7	Intergenic	S_Shelf	68.51	3.40	71.66	4.04	-3.15	2.33E-04	5.58E-03
cg23566793	LLGL1	17	Body	Open sea	51.25	2.96	54.40	3.76	-3.15	1.16E-04	3.17E-03
cg00103785		1	Intergenic	N_Shore	13.47	3.71	16.62	4.43	-3.15	1.13E-04	3.10E-03
cg11145826	HDDC3;UNC45A;UNC45A	15	TSS1500;5'UTR;TSS1500	N_Shore	29.71	3.53	32.86	3.31	-3.15	3.80E-04	8.19E-03
cg21451869	MMP9	20	TSS1500	N_Shelf	20.99	4.30	24.14	4.88	-3.15	7.91E-04	1.44E-02
cg02686091	PSG5;PSG5	19	3'UTR;3'UTR	Open sea	77.73	4.70	80.88	4.34	-3.15	6.86E-05	2.04E-03
cg01772700	ZNF582	19	5'UTR	N_Shore	36.98	4.87	40.13	4.51	-3.15	4.27E-03	4.77E-02
cg13585324		4	Intergenic	Open sea	76.52	4.84	79.67	4.12	-3.15	4.26E-04	8.95E-03
cg02667467	OTOP2;USH1G	17	TSS200;TSS1500	Island	33.33	3.21	36.48	3.64	-3.15	2.75E-05	9.26E-04
cg10957151	HOXD9	2	TSS1500	Island	30.58	4.22	33.73	4.29	-3.15	7.76E-04	1.42E-02
cg02938926	GPRC5B	16	TSS1500	S_Shore	9.42	2.92	12.58	4.21	-3.15	1.02E-04	2.85E-03
cg19939732	GPR160	3	Body	Open sea	83.77	5.37	86.92	4.89	-3.15	2.56E-04	6.01E-03
cg21140028	PLEKHA5;PLEKHA5	12	5'UTR;Body	Open sea	83.37	4.36	86.52	3.60	-3.15	9.18E-04	1.61E-02
cg20655350	GPBAR1;GPBAR1;GPBAR1	2	TSS1500;TSS1500;TSS1500	Open sea	70.22	3.70	73.37	3.01	-3.15	1.02E-04	2.84E-03
cg14626309		4	Intergenic	Island	18.44	3.56	21.59	4.13	-3.15	5.76E-04	1.13E-02
cg19140429		17	Intergenic	Island	60.00	4.61	63.15	4.50	-3.15	1.49E-03	2.29E-02
cg18789620	EEF2	19	Body	S_Shore	74.07	4.60	77.22	4.16	-3.15	1.54E-03	2.35E-02
cg26559689	CDYL;CDYL;CDYL;CDYL	6	Body;Body;Body;Body	Open sea	80.16	4.20	83.30	2.91	-3.15	6.81E-04	1.28E-02
cg01836683		7	Intergenic	Open sea	59.46	5.16	62.61	4.28	-3.15	2.70E-04	6.27E-03
cg07535191		11	Intergenic	Open sea	64.36	5.10	67.51	6.43	-3.15	3.73E-04	8.07E-03
cg05656688	RUNX3;RUNX3	1	Body;Body	N_Shore	41.59	4.02	44.74	5.28	-3.15	2.06E-03	2.89E-02
cg13560452	OLIG3;OLIG3	6	1stExon;3'UTR	N_Shore	29.85	4.83	33.00	4.10	-3.15	3.33E-03	4.03E-02
cg13446874	SCEL;SCEL;SCEL	13	5'UTR;5'UTR;5'UTR	Open sea	82.90	4.95	86.05	5.27	-3.15	4.46E-03	4.92E-02
cg06158985	C3orf74	3	TSS200	Open sea	72.87	3.39	76.02	3.46	-3.15	2.30E-05	7.91E-04
cg11513700		7	Intergenic	Island	13.16	3.29	16.31	3.68	-3.15	5.21E-05	1.61E-03
cg06972641		10	Intergenic	Open sea	63.24	5.15	66.39	6.08	-3.15	4.08E-03	4.62E-02
cg06155697	TUB;TUB	11	Body;Body	Open sea	77.38	4.74	80.53	4.39	-3.15	3.44E-03	4.12E-02
cg16158681	MT3	16	TSS200	Island	19.48	3.49	22.62	4.39	-3.15	7.37E-05	2.16E-03
cg15460921	LARP4;LARP4;LARP4;LARP4;LARP4;LA	12	Body;Body;Body;Body;Body;Body;Body	Open sea	53.08	4.17	56.23	4.30	-3.15	1.86E-03	2.69E-02
cg01150982	MRAP;MRAP	21	5'UTR;5'UTR	Open sea	79.53	3.43	82.68	3.16	-3.15	2.27E-05	7.83E-04
cg10403849	TET1	10	5'UTR	S_Shore	63.05	5.54	66.20	6.39	-3.15	3.53E-03	4.19E-02
cg01212010		10	Intergenic	Open sea	69.23	5.19	72.38	4.20	-3.15	1.23E-03	1.99E-02
cg18202521	AKR7L;AKR7L	1	TSS200;TSS200	Island	8.38	2.90	11.53	4.67	-3.15	1.29E-03	2.07E-02
cg23904441	TNFSF11;TNFSF11	13	TSS1500;5'UTR	N_Shore	76.29	5.98	79.43	4.26	-3.15	1.42E-03	2.21E-02
cg14169333		9	Intergenic	Island	17.43	4.14	20.58	3.98	-3.15	9.82E-05	2.76E-03
cg10368507	GHDC;GHDC;GHDC;GHDC	17	TSS1500;TSS1500;TSS1500;TSS1500	Open sea	53.32	3.29	56.47	3.18	-3.15	1.75E-04	4.45E-03
cg13535212	FOXI3	2	TSS1500	Island	27.42	4.12	30.57	4.62	-3.15	1.25E-03	2.02E-02
cg10679266	DEFA4	8	TSS200	Open sea	80.82	4.46	83.97	2.51	-3.15	1.65E-03	2.47E-02
cg13023239	GNAL;GNAL;GNAL	18	Body;Body;Body	S_Shore	79.06	4.98	82.20	4.77	-3.15	1.73E-03	2.55E-02
cg23455440	IGFBP3;IGFBP3	7	TSS1500;TSS1500	S_Shore	10.55	2.76	13.70	5.19	-3.15	7.94E-04	1.44E-02
cg26027669	GFI1;GFI1	1	TSS1500;TSS200	Island	18.18	4.93	21.32	5.45	-3.15	3.70E-03	4.33E-02
cg19550914	DUSP7	3	3'UTR	Open sea	68.70	4.42	71.84	3.56	-3.15	4.14E-03	4.67E-02
cg11521563	MCF2L;MCF2L	13	Body;Body	N_Shore	67.15	4.77	70.30	4.26	-3.15	1.61E-03	2.42E-02

cg21278764	UTP11L	1	Body	S_Shelf	76.23	4.88	79.37	3.66	-3.15	6.23E-04	1.20E-02
cg01305421	IGF1;IGF1;IGF1;IGF1;IGF1;IGF1	12	5'UTR;5'UTR;5'UTR;1stExon;1stExon;1stEx	Open sea	14.98	3.30	18.12	4.26	-3.15	1.87E-03	2.69E-02
cg03891318	NMNAT3	3	5'UTR	Open sea	74.47	3.61	77.62	3.96	-3.15	3.20E-05	1.05E-03
cg00925722	EDC4;NRN1L	16	Body;TSS1500	N_Shore	77.84	3.39	80.98	3.37	-3.15	1.01E-04	2.84E-03
cg06852461		1	Intergenic	Open sea	41.88	4.57	45.02	4.38	-3.15	1.14E-03	1.89E-02
cg27400070	DISP2	15	Body	Island	9.75	2.65	12.90	3.25	-3.15	1.01E-06	4.53E-05
cg00048759	STAG3;GPC2	7	TSS200;TSS1500	Island	49.82	3.76	52.97	3.78	-3.15	1.85E-04	4.65E-03
cg03656020	VGF	7	3'UTR	N_Shore	34.02	4.21	37.16	4.08	-3.15	3.31E-04	7.35E-03
cg13314694	CDYL	6	Body	Open sea	57.57	4.39	60.71	5.32	-3.15	3.48E-04	7.64E-03
cg21467850	ICA1L	2	Body	Open sea	63.40	4.46	66.55	3.94	-3.14	4.77E-04	9.76E-03
cg26752613	NOD1	7	Body	Open sea	71.35	4.42	74.50	4.19	-3.14	5.16E-04	1.04E-02
cg11304838	FYN	6	5'UTR	Open sea	77.13	5.81	80.27	6.47	-3.14	3.60E-03	4.24E-02
cg05838358	AMIGO2	12	3'UTR	N_Shelf	70.07	5.30	73.21	4.86	-3.14	4.15E-03	4.68E-02
cg04252145		5	Intergenic	S_Shelf	88.16	3.74	91.30	2.29	-3.14	6.49E-05	1.94E-03
cg05768047	AP2A2	11	Body	N_Shore	77.32	3.82	80.46	3.41	-3.14	7.52E-04	1.38E-02
cg01713348	C16orf91	16	TSS1500	N_Shore	59.79	4.05	62.94	3.31	-3.14	3.87E-04	8.29E-03
cg18227471	NKX6-3	8	TSS1500	S_Shore	74.29	5.08	77.43	4.46	-3.14	2.91E-03	3.66E-02
cg12065138	AJAP1;AJAP1	1	Body;Body	N_Shore	15.35	3.90	18.49	5.24	-3.14	1.29E-03	2.07E-02
cg04587617		17	Intergenic	N_Shelf	71.70	4.23	74.84	3.60	-3.14	3.74E-04	8.08E-03
cg06057079		4	Intergenic	N_Shore	28.78	5.18	31.92	4.79	-3.14	6.33E-04	1.22E-02
cg22883353		19	Intergenic	N_Shelf	59.05	4.17	62.20	5.06	-3.14	1.70E-04	4.34E-03
cg26040708		8	Intergenic	N_Shore	59.18	4.18	62.32	4.98	-3.14	3.67E-03	4.30E-02
cg16458153	COL11A2;COL11A2;COL11A2;COL11A2	6	5'UTR;5'UTR;1stExon;1stExon;5'UTR;1stEx	Island	29.42	3.45	32.56	4.66	-3.14	1.81E-04	4.56E-03
cg15158310	IGFL2;IGFL2	19	TSS1500;TSS200	Open sea	49.62	3.16	52.76	3.32	-3.14	1.30E-04	3.48E-03
cg13033722	PTK7;PTK7;PTK7;PTK7	6	Body;Body;Body;Body	Open sea	60.27	3.63	63.41	3.59	-3.14	9.05E-05	2.58E-03
cg03719978	KDM6B;TMEM88	17	3'UTR;TSS1500	N_Shore	59.65	4.48	62.79	4.27	-3.14	3.39E-03	4.08E-02
cg22747380	EXT1	8	Body	Open sea	15.71	3.81	18.85	5.03	-3.14	1.84E-03	2.67E-02
cg14039925	CNGA4	11	Body	S_Shelf	69.46	3.91	72.60	3.69	-3.14	3.71E-05	1.20E-03
cg12850075	CAGE1;CAGE1;CAGE1	6	Body;Body;Body	Open sea	81.67	4.36	84.81	3.73	-3.14	5.80E-04	1.13E-02
cg14341418	APOM	6	Body	S_Shelf	82.35	4.00	85.49	5.02	-3.14	7.20E-04	1.34E-02
cg25139410	FAM179A	2	TSS200	Open sea	50.79	4.51	53.93	4.79	-3.14	6.01E-04	1.17E-02
cg16698913		5	Intergenic	Open sea	49.66	4.27	52.80	4.05	-3.14	2.49E-03	3.30E-02
cg06889535	KIRREL3;KIRREL3	11	Body;Body	Open sea	20.35	4.54	23.49	4.55	-3.14	3.58E-03	4.23E-02
cg19906672	TBC1D14;TBC1D14	4	5'UTR;5'UTR	Open sea	64.14	4.87	67.28	4.61	-3.14	2.04E-04	5.02E-03
cg06508062		21	Intergenic	N_Shore	38.41	4.26	41.55	4.70	-3.14	4.77E-04	9.77E-03
cg06492796		12	Intergenic	N_Shore	26.24	5.50	29.38	5.79	-3.14	2.74E-03	3.52E-02
cg24688946		1	Intergenic	Open sea	56.27	4.87	59.41	4.96	-3.14	4.33E-03	4.82E-02
cg24616539		11	Intergenic	S_Shore	30.17	3.72	33.31	4.25	-3.14	1.18E-04	3.20E-03
cg09301052		6	Intergenic	S_Shelf	87.16	5.35	90.29	3.47	-3.14	3.83E-03	4.43E-02
cg14724590		17	Intergenic	Open sea	65.72	5.42	68.86	5.81	-3.14	4.31E-03	4.80E-02
cg13055199	ULK3	15	TSS1500	S_Shore	62.51	5.06	65.64	3.88	-3.14	9.61E-04	1.66E-02
cg05629015	FZD8	10	1stExon	Island	35.48	6.54	38.62	6.72	-3.14	1.36E-03	2.15E-02
cg25141264	TBC1D22A	22	Body	S_Shelf	70.28	4.03	73.42	3.58	-3.14	1.13E-03	1.87E-02
cg04306817	ALDOA;ALDOA;ALDOA;ALDOA	16	TSS200;TSS1500;TSS1500;5'UTR	N_Shore	6.93	2.68	10.06	4.65	-3.14	1.28E-04	3.43E-03
cg23299469	IRAK4;IRAK4;IRAK4;IRAK4;IRAK4	12	5'UTR;5'UTR;5'UTR;5'UTR;5'UTR	S_Shelf	78.07	3.90	81.21	3.60	-3.14	9.24E-05	2.62E-03
cg26048101	ATP9A	20	Body	Open sea	14.70	3.37	17.83	4.64	-3.14	1.47E-03	2.27E-02
cg26028853	ZHX2	8	5'UTR	Open sea	71.11	5.70	74.25	5.57	-3.14	3.80E-03	4.40E-02
cg09738156	MAP6D1	3	TSS200	Island	53.33	4.91	56.47	6.04	-3.14	2.77E-04	6.39E-03
cg14648424	POLE3;POLE3	9	Body;3'UTR	N_Shelf	79.57	3.06	82.71	3.01	-3.14	1.25E-07	6.17E-06
cg10761978	RTP2	3	TSS1500	S_Shelf	66.78	3.71	69.92	3.89	-3.14	5.98E-05	1.82E-03
cg06996423	CALHM3	10	Body	S_Shelf	61.20	3.48	64.34	3.88	-3.14	1.23E-04	3.32E-03
cg22246344		2	Intergenic	S_Shore	43.40	3.05	46.54	3.54	-3.14	2.13E-04	5.19E-03
cg23195510	RLTPR	16	TSS1500	Island	45.64	3.97	48.77	5.75	-3.14	3.04E-05	1.01E-03

cg12099472		1	Intergenic	Open sea	67.23	4.67	70.37	3.76	-3.14	2.31E-03	3.12E-02
cg10808783	APC2	19	Body	Island	9.99	3.31	13.12	4.63	-3.14	1.44E-04	3.78E-03
cg17962527	LOC116437	12	TSS1500	Island	74.24	4.89	77.38	3.57	-3.14	4.71E-04	9.68E-03
cg09532898	EPS8L2	11	Body	Island	28.23	3.48	31.37	3.41	-3.14	2.89E-04	6.60E-03
cg08405119	RASL11B	4	Body	Island	44.38	4.20	47.51	4.24	-3.14	2.51E-03	3.31E-02
cg03566513	LIN7A	12	TSS1500	S_Shore	79.22	5.31	82.36	4.22	-3.14	3.08E-03	3.81E-02
cg08935125	CLDN6	16	5'UTR	Island	62.05	4.34	65.18	4.03	-3.14	1.02E-03	1.74E-02
cg00833562	CCNF	16	Body	Open sea	85.97	3.70	89.10	2.77	-3.14	2.96E-05	9.85E-04
cg11698647	BAIAP2;BAIAP2;BAIAP2;BAIAP2	17	Body;Body;Body;Body	N_Shelf	77.28	4.48	80.41	4.37	-3.14	1.27E-03	2.04E-02
cg25024442	SLC4A1AP	2	Body	Open sea	62.02	3.99	65.15	4.11	-3.14	6.36E-04	1.22E-02
cg21382890	NFE2L2;NFE2L2;NFE2L2	2	Body;5'UTR;5'UTR	Open sea	35.33	6.38	38.46	6.81	-3.14	4.45E-03	4.91E-02
cg19267253		7	Intergenic	S_Shelf	56.42	4.33	59.55	4.44	-3.14	1.04E-03	1.76E-02
cg07144297	CDH15	16	Body	Open sea	55.09	3.74	58.22	4.26	-3.13	2.36E-03	3.17E-02
cg10597997	STK10	5	Body	N_Shelf	84.84	3.90	87.98	3.44	-3.13	6.87E-04	1.29E-02
cg13856126	RALGPS1;ANGPTL2	9	Body;Body	Open sea	78.49	4.29	81.62	4.17	-3.13	1.97E-04	4.89E-03
cg18849256	BICD1;BICD1	12	Body;Body	Open sea	80.29	4.97	83.42	4.25	-3.13	3.18E-03	3.90E-02
cg00213142	RAD54L2	3	Body	Open sea	80.21	3.71	83.34	2.93	-3.13	3.42E-04	7.54E-03
cg03864479	LIFR;LIFR	5	5'UTR;5'UTR	Open sea	36.63	4.15	39.76	4.93	-3.13	5.24E-05	1.62E-03
cg12619940	OSR1	2	3'UTR	Island	20.33	3.40	23.46	2.63	-3.13	8.72E-05	2.50E-03
cg00990385	CCDC42B	12	1stExon	N_Shelf	44.50	6.03	47.63	4.88	-3.13	2.01E-03	2.84E-02
cg08496521	ARHGDIG	16	Body	N_Shore	54.73	4.16	57.87	4.31	-3.13	2.05E-03	2.88E-02
cg01455766		16	Intergenic	S_Shore	65.08	4.97	68.22	5.23	-3.13	1.54E-03	2.35E-02
cg24862483	CD300LG;CD300LG;CD300LG;CD300L(	17	TSS200;TSS200;TSS200;TSS200	Open sea	22.04	2.90	25.17	4.19	-3.13	2.42E-04	5.75E-03
cg10263401	UBE2C;UBE2C;UBE2C;UBE2C;UBE2C;U	20	5'UTR;TSS1500;5'UTR;1stExon;5'UTR;5'UT	Island	4.14	1.56	7.27	3.95	-3.13	7.78E-08	3.91E-06
cg24861859	TNR	1	5'UTR	Open sea	48.05	4.57	51.18	4.48	-3.13	4.01E-03	4.57E-02
cg25353721		7	Intergenic	S_Shelf	84.95	4.11	88.09	3.18	-3.13	1.15E-05	4.27E-04
cg08125824	HECTD1	14	Body	Open sea	80.91	5.33	84.04	4.64	-3.13	3.95E-03	4.53E-02
cg05007997	SLC11A1;SLC11A1	2	1stExon;5'UTR	Open sea	67.77	4.76	70.90	4.64	-3.13	3.94E-04	8.41E-03
cg06517813		1	Intergenic	Open sea	70.77	4.90	73.90	3.84	-3.13	7.64E-04	1.40E-02
cg16686185	GAA;GAA;GAA	17	Body;Body;Body	N_Shore	80.33	3.94	83.46	3.44	-3.13	8.00E-05	2.32E-03
cg11716064		10	Intergenic	Open sea	55.57	5.11	58.70	4.81	-3.13	3.70E-03	4.33E-02
cg12114735	TNNI3	19	Body	Island	13.58	2.99	16.71	4.34	-3.13	1.02E-04	2.84E-03
cg00894757	CLDN11	3	Body	Island	9.34	2.53	12.48	4.04	-3.13	1.05E-05	3.93E-04
cg13057898	LRRC47	1	Body	Island	76.07	3.91	79.20	4.33	-3.13	1.30E-04	3.47E-03
cg00393837		2	Intergenic	Island	15.35	2.85	18.48	3.71	-3.13	1.29E-05	4.72E-04
cg05772917	MAPT;MAPT;MAPT;MAPT;MAPT;MAF	17	5'UTR;5'UTR;5'UTR;5'UTR;5'UTR;5'UTR	S_Shore	50.59	5.04	53.72	4.85	-3.13	2.27E-03	3.09E-02
cg18711369	ORMDL3	17	5'UTR	N_Shelf	37.52	4.41	40.66	4.76	-3.13	1.95E-03	2.78E-02
cg04973669	TAPBP;TAPBP;TAPBP	6	Body;Body;Body	Open sea	61.19	3.82	64.32	3.13	-3.13	5.10E-05	1.58E-03
cg02399831		3	Intergenic	Open sea	64.09	4.21	67.22	4.44	-3.13	5.44E-04	1.08E-02
cg12035325	LRBA	4	Body	Open sea	82.17	3.78	85.30	3.38	-3.13	5.50E-04	1.09E-02
cg05937445	C1orf86	1	5'UTR	N_Shore	61.09	4.47	64.23	4.10	-3.13	1.46E-03	2.26E-02
cg22917372	TMEM120B	12	Body	Open sea	58.13	4.94	61.26	3.75	-3.13	4.19E-03	4.71E-02
cg01367899	MTVR2	17	Body	Open sea	73.04	3.62	76.17	3.03	-3.13	6.99E-05	2.07E-03
cg04771433	PRKCZ;PRKCZ;PRKCZ	1	5'UTR;Body;5'UTR	N_Shore	36.71	4.86	39.84	5.55	-3.13	7.60E-04	1.39E-02
cg04452713	DST;DST;DST;DST	6	Body;Body;Body;Body	N_Shore	12.04	3.24	15.17	4.81	-3.13	1.02E-03	1.74E-02
cg00628277	TBCD	17	Body	Open sea	54.38	5.13	57.51	4.87	-3.13	4.55E-03	4.98E-02
cg14663042	LASS6	2	Body	Open sea	81.52	5.48	84.65	4.40	-3.13	3.69E-03	4.32E-02
cg02072796		1	Intergenic	S_Shore	78.78	3.10	81.91	3.01	-3.13	1.53E-06	6.68E-05
cg20405384	TCF23	2	TSS200	N_Shore	44.93	3.15	48.06	3.53	-3.13	4.00E-04	8.53E-03
cg27176129	MATN4;RBPJL;MATN4;MATN4	20	TSS200;Body;TSS200;TSS200	S_Shore	55.77	4.66	58.90	4.36	-3.13	2.03E-03	2.86E-02
cg12769519		17	Intergenic	N_Shore	81.58	2.97	84.71	2.70	-3.13	2.37E-06	1.00E-04
cg14266770	VAV2;VAV2	9	Body;Body	Open sea	25.12	4.78	28.25	4.00	-3.13	2.48E-03	3.29E-02
cg14878302		18	Intergenic	S_Shelf	82.44	3.83	85.57	3.31	-3.13	1.12E-05	4.16E-04

cg12411312	HEMK1	3	Body	S_Shore	79.77	3.85	82.90	2.32	-3.13	2.26E-04	5.45E-03
cg22408503	CNGA3;CNGA3	2	TSS200;TSS200	N_Shore	57.38	5.39	60.51	4.79	-3.13	1.76E-03	2.58E-02
cg12669543	SRGAP3;SRGAP3	3	Body;Body	Open sea	75.53	4.11	78.66	3.52	-3.13	7.60E-04	1.39E-02
cg24817154	HIST1H3J	6	1stExon	Island	19.14	3.95	22.27	5.09	-3.13	5.14E-05	1.59E-03
cg08918057	SLC30A5	5	Body	Open sea	79.94	4.46	83.07	4.43	-3.13	1.14E-03	1.89E-02
cg24981400	REV1;REV1	2	Body;Body	Open sea	57.12	5.24	60.24	5.70	-3.13	1.22E-03	1.98E-02
cg05212465	SNX9	6	Body	Open sea	43.38	2.95	46.50	2.95	-3.13	3.85E-05	1.24E-03
cg02350211	SMTN;SMTN;SMTN	22	Body;Body;Body	Island	77.11	4.57	80.24	3.54	-3.13	6.17E-04	1.19E-02
cg17408935		17	Intergenic	Open sea	79.31	4.37	82.44	3.88	-3.13	2.01E-04	4.95E-03
cg13144588	DAPK3	19	TSS200	N_Shore	23.14	6.43	26.27	5.80	-3.13	2.01E-03	2.83E-02
cg04499011	FGD2	6	TSS1500	Open sea	73.29	3.96	76.42	3.87	-3.13	1.75E-04	4.44E-03
cg08057038	MUPCDH;MUPCDH	11	TSS1500;TSS1500	N_Shore	28.24	4.10	31.36	4.02	-3.13	2.80E-03	3.57E-02
cg20885782		4	Intergenic	Island	21.34	4.50	24.47	5.18	-3.13	1.29E-03	2.07E-02
cg00625939		4	Intergenic	Island	63.63	3.63	66.76	3.36	-3.13	5.55E-04	1.10E-02
cg08703808		1	Intergenic	N_Shelf	81.81	3.83	84.94	3.25	-3.13	2.82E-06	1.18E-04
cg21333589		4	Intergenic	Open sea	74.29	3.59	77.41	2.92	-3.13	2.01E-04	4.97E-03
cg26396415	MGC23270	14	Body	Open sea	83.72	4.57	86.85	3.13	-3.13	1.16E-03	1.91E-02
cg13461890	RCL1	9	Body	S_Shore	45.13	4.18	48.26	4.54	-3.13	5.97E-04	1.16E-02
cg04584275		2	Intergenic	Open sea	70.72	4.56	73.85	3.63	-3.13	2.47E-04	5.85E-03
cg27574071	CTBP1;CTBP1	4	Body;Body	Island	75.65	4.00	78.78	3.57	-3.13	3.01E-04	6.81E-03
cg13682140	MPP2	17	Body	S_Shore	43.22	6.05	46.34	6.01	-3.13	2.97E-03	3.72E-02
cg18935743		17	Intergenic	Open sea	73.01	4.55	76.13	4.41	-3.13	2.13E-03	2.95E-02
cg23146472	TTBK1	6	Body	Island	31.54	3.50	34.66	3.54	-3.13	1.69E-04	4.32E-03
cg07117311	CTRL	16	TSS1500	N_Shelf	79.53	4.42	82.66	3.41	-3.13	1.35E-04	3.58E-03
cg09807875	BMPER	7	Body	Open sea	20.20	3.03	23.33	3.56	-3.13	1.31E-04	3.50E-03
cg07569264	CBFA2T3	16	Body	Open sea	73.78	4.01	76.90	3.86	-3.13	1.63E-03	2.45E-02
cg06998282	ENPP2;ENPP2;ENPP2	8	TSS1500;TSS1500;TSS1500	Open sea	10.78	3.86	13.90	5.58	-3.13	6.69E-05	1.99E-03
cg13986840	MEST;MEST;MESTIT1;MEST	7	5'UTR;5'UTR;TSS1500;Body	Island	39.89	5.38	43.02	3.63	-3.13	3.38E-04	7.47E-03
cg13634864	ADAP1	7	TSS1500	S_Shore	79.72	5.01	82.85	3.90	-3.13	1.50E-03	2.31E-02
cg26517849	INS-IGF2;IGF2AS;IGF2;IGF2;IGF2AS;IG	11	Body;TSS1500;Body;5'UTR;TSS1500;TSS15	Island	41.93	4.08	45.06	4.58	-3.13	7.42E-04	1.37E-02
cg03870497	SKAP2	7	Body	Island	15.17	3.57	18.29	5.09	-3.13	3.29E-03	3.99E-02
cg17616453	C21orf62;C21orf62;C21orf62	21	5'UTR;5'UTR;5'UTR	Open sea	55.50	4.68	58.63	4.94	-3.13	1.17E-03	1.93E-02
cg17485681	CDH23	10	Body	Island	55.10	5.65	58.23	3.93	-3.13	4.32E-03	4.81E-02
cg24244500		13	Intergenic	N_Shore	34.78	4.73	37.91	4.53	-3.13	6.29E-04	1.21E-02
cg10602543	H19	11	Body	N_Shore	52.26	3.34	55.39	3.37	-3.13	7.62E-05	2.23E-03
cg09386653		7	Intergenic	S_Shore	73.71	3.93	76.83	3.36	-3.13	2.72E-04	6.30E-03
cg18378551	DLG2	11	Body	Open sea	83.14	4.15	86.27	3.32	-3.12	2.65E-06	1.11E-04
cg12778007	SIPA1L1	14	5'UTR	Open sea	78.30	5.11	81.43	5.48	-3.12	2.43E-03	3.24E-02
cg18416096	SYNPO;SYNPO;SYNPO;SYNPO	5	Body;Body;Body;Body	Island	55.88	2.95	59.00	2.83	-3.12	4.54E-05	1.43E-03
cg17140034	TCF12;TCF12;TCF12;TCF12	15	Body;Body;Body;Body	Open sea	85.49	5.11	88.61	3.85	-3.12	1.51E-03	2.31E-02
cg01524860		6	Intergenic	Island	30.54	3.64	33.66	4.15	-3.12	4.57E-04	9.45E-03
cg04008905	GPR126;GPR126;GPR126;GPR126	6	Body;Body;Body;Body	Open sea	90.50	3.08	93.62	2.02	-3.12	1.77E-08	9.32E-07
cg18383585	NOS1AP;NOS1AP	1	Body;Body	Open sea	16.27	2.71	19.39	3.43	-3.12	1.99E-05	6.96E-04
cg15503722	BSX	11	Body	N_Shore	20.13	3.32	23.25	4.34	-3.12	5.62E-04	1.11E-02
cg07487112		6	Intergenic	S_Shelf	74.32	4.56	77.44	4.01	-3.12	4.26E-04	8.95E-03
cg08778287	IGF1R	15	Body	N_Shore	75.58	4.57	78.71	3.51	-3.12	1.82E-03	2.64E-02
cg08480413		2	Intergenic	Open sea	79.52	4.52	82.64	3.64	-3.12	2.96E-04	6.73E-03
cg24740868	C1orf87;C1orf87	1	1stExon;5'UTR	Island	20.65	3.95	23.77	4.38	-3.12	6.86E-04	1.29E-02
cg13139203	C11orf84	11	Body	Island	32.21	4.86	35.33	5.11	-3.12	4.18E-04	8.81E-03
cg10219223	ACOT7;ACOT7;ACOT7;ACOT7	1	Body;5'UTR;1stExon;Body	Open sea	37.97	3.29	41.09	4.20	-3.12	1.26E-03	2.04E-02
cg18050139		12	Intergenic	Open sea	67.23	4.60	70.35	4.24	-3.12	3.32E-03	4.02E-02
cg09823543	SRF	6	Body	N_Shelf	71.65	4.29	74.78	2.94	-3.12	8.70E-05	2.49E-03
cg21199072		4	Intergenic	Open sea	61.32	2.48	64.44	2.18	-3.12	2.93E-07	1.41E-05

cg03719083	TFAP2A;TFAP2A;TFAP2A	6	Body;5'UTR;1stExon	Island	25.95	2.94	29.07	3.08	-3.12	7.02E-06	2.73E-04
cg20981848	BTBD3;BTBD3	20	Body;Body	Open sea	59.72	6.02	62.85	3.86	-3.12	2.91E-03	3.66E-02
cg20795500	OLA1;OLA1	2	Body;Body	Open sea	84.38	5.20	87.50	5.55	-3.12	9.83E-04	1.69E-02
cg04278105	INF2;INF2;INF2	14	Body;Body;Body	S_Shore	66.70	4.63	69.82	4.10	-3.12	1.31E-03	2.09E-02
cg21697794		6	Intergenic	Island	42.22	3.72	45.35	3.75	-3.12	3.66E-04	7.96E-03
cg18907363	CCDC85C	14	Body	N_Shelf	46.03	4.92	49.15	4.99	-3.12	3.64E-03	4.28E-02
cg25370567		13	Intergenic	Open sea	79.13	4.08	82.25	3.44	-3.12	2.16E-04	5.24E-03
cg01756461		15	Intergenic	Open sea	53.93	5.56	57.06	3.90	-3.12	4.22E-03	4.73E-02
cg03155271	PPFIA3	19	Body	S_Shore	69.16	4.14	72.28	2.94	-3.12	2.96E-04	6.74E-03
cg06750167	TESC;TESC;TESC	12	TSS1500;TSS1500;TSS1500	S_Shore	26.77	4.05	29.89	5.09	-3.12	3.12E-03	3.84E-02
cg07331204		6	Intergenic	Open sea	78.50	4.16	81.62	3.23	-3.12	4.84E-05	1.51E-03
cg09774007	RFX4	12	Body	N_Shore	8.97	2.96	12.09	5.06	-3.12	3.00E-03	3.74E-02
cg17025642	TRIM22	11	TSS1500	Open sea	82.01	4.66	85.13	4.87	-3.12	3.22E-04	7.19E-03
cg00200667		17	Intergenic	N_Shelf	39.44	4.75	42.56	4.78	-3.12	1.29E-03	2.07E-02
cg18706888	PEG3;PEG3;ZIM2;ZIM2;PEG3;ZIM2;MI	19	TSS1500;TSS1500;TSS1500;TSS1500;TSS15	S_Shore	53.68	4.04	56.80	3.49	-3.12	4.78E-04	9.78E-03
cg03257179	LTK;LTK;LTK;LTK;LTK;LTK	15	1stExon;1stExon;5'UTR;5'UTR;1stExon;5'U	S_Shore	21.91	3.86	25.03	4.34	-3.12	5.02E-04	1.02E-02
cg10504365	TRIM67	1	TSS1500	N_Shore	18.75	4.06	21.87	4.08	-3.12	8.24E-05	2.38E-03
cg20299254	WFDC2	20	TSS1500	N_Shore	79.14	4.55	82.26	5.13	-3.12	3.91E-03	4.50E-02
cg01468656	HTR6	1	TSS200	Island	29.41	3.87	32.53	5.01	-3.12	2.41E-04	5.74E-03
cg11065015	NRSN2	20	TSS200	N_Shore	70.62	4.50	73.74	3.48	-3.12	1.01E-03	1.72E-02
cg05907976	HCG4	6	Body	Island	15.74	4.37	18.86	5.16	-3.12	1.85E-03	2.68E-02
cg26939227	MYO1C;MYO1C;MYO1C	17	Body;Body;Body	N_Shelf	53.76	4.49	56.88	4.61	-3.12	3.60E-03	4.25E-02
cg02489371	RNF43	17	TSS1500	Open sea	76.06	4.56	79.18	4.05	-3.12	4.82E-04	9.84E-03
cg21754746	WDR46;WDR46;PFDN6	6	1stExon;1stExon;TSS1500	Island	39.08	5.20	42.20	4.42	-3.12	4.51E-04	9.36E-03
cg21123160	SLC6A11	3	TSS200	Island	29.87	4.07	32.99	4.34	-3.12	5.03E-04	1.02E-02
cg10635061	FHL2;FHL2	2	1stExon;5'UTR	Open sea	42.30	4.35	45.42	4.63	-3.12	1.62E-04	4.18E-03
cg13847070	KLHL3;KLHL3	5	5'UTR;1stExon	Open sea	8.89	3.64	12.01	5.24	-3.12	2.01E-03	2.84E-02
cg06390536	MYO15B	17	TSS200	Island	47.81	5.30	50.93	6.86	-3.12	2.16E-03	2.98E-02
cg00236766	CTDSP1;CTDSP1	2	Body;Body	N_Shelf	75.91	4.34	79.03	2.88	-3.12	2.33E-03	3.14E-02
cg17716617	KIAA1522	1	Body	Island	5.57	2.13	8.69	4.20	-3.12	3.95E-06	1.61E-04
cg12879425	STAP1;STAP1	4	5'UTR;1stExon	Open sea	61.23	5.01	64.35	6.13	-3.12	2.06E-04	5.05E-03
cg23339693		6	Intergenic	Open sea	48.42	5.24	51.54	5.24	-3.12	3.92E-03	4.50E-02
cg18609078		4	Intergenic	S_Shore	27.51	4.28	30.63	4.76	-3.12	2.26E-03	3.08E-02
cg25725418		10	Intergenic	Open sea	22.40	4.52	25.52	4.91	-3.12	9.53E-04	1.65E-02
cg03512637		6	Intergenic	N_Shore	46.57	5.95	49.69	5.59	-3.12	3.83E-03	4.43E-02
cg24828811	LRRC58	3	3'UTR	Open sea	74.65	4.32	77.77	3.64	-3.12	1.22E-03	1.99E-02
cg18407752	RHOD	11	3'UTR	S_Shore	5.94	2.20	9.06	4.43	-3.12	1.19E-05	4.38E-04
cg17871910	TMEM90A	14	5'UTR	Island	19.71	3.70	22.83	3.95	-3.12	3.01E-04	6.82E-03
cg27384918		16	Intergenic	Open sea	59.56	4.30	62.68	4.15	-3.12	3.60E-04	7.86E-03
cg11165752		7	Intergenic	Island	30.64	3.88	33.76	4.32	-3.12	2.38E-03	3.19E-02
cg04338156		16	Intergenic	N_Shore	63.55	5.71	66.67	4.52	-3.12	7.39E-04	1.37E-02
cg05291773		21	Intergenic	N_Shore	82.22	3.99	85.34	3.16	-3.12	8.21E-04	1.47E-02
cg21836370		12	Intergenic	Open sea	19.58	3.55	22.70	3.55	-3.12	1.72E-05	6.10E-04
cg09760574	C20orf152	20	Body	Open sea	62.35	5.53	65.47	5.02	-3.12	1.54E-03	2.35E-02
cg08284233	PDILT	16	3'UTR	Open sea	47.54	6.01	50.66	6.36	-3.12	5.89E-04	1.15E-02
cg14456490		3	Intergenic	Open sea	61.18	4.69	64.29	4.37	-3.12	1.43E-04	3.76E-03
cg15531386		11	Intergenic	Open sea	78.90	5.53	82.02	5.20	-3.12	2.19E-03	3.01E-02
cg07724243	C9orf50	9	TSS1500	S_Shore	85.60	3.70	88.72	2.49	-3.12	3.79E-05	1.22E-03
cg13891220	PITX2;PITX2	4	Body;Body	Island	25.93	3.81	29.05	3.83	-3.12	3.77E-04	8.13E-03
cg00326902	ROS1	6	TSS1500	Open sea	72.84	4.98	75.95	5.27	-3.12	1.38E-03	2.17E-02
cg24825722	ACADVL;DLG4;DLG4;ACADVL	17	TSS1500;TSS1500;Body;TSS1500	N_Shore	68.54	3.22	71.66	4.21	-3.12	1.66E-05	5.92E-04
cg02479744	ATXN1;ATXN1	6	3'UTR;3'UTR	Open sea	82.39	4.05	85.51	3.23	-3.12	4.05E-04	8.60E-03
cg07927072	BRE;BRE;BRE;BRE;BRE	2	5'UTR;5'UTR;5'UTR;5'UTR;5'UTR	S_Shelf	71.37	3.67	74.49	3.37	-3.12	5.44E-04	1.08E-02

cg24167422		1	Intergenic	Island	58.96	5.74	62.07	4.65	-3.12	3.67E-03	4.30E-02
cg12950829		5	Intergenic	Open sea	70.82	4.78	73.93	4.17	-3.12	3.89E-03	4.48E-02
cg27104695	SH3BP5;SH3BP5	3	Body;5'UTR	Open sea	50.47	3.96	53.59	3.45	-3.12	1.01E-04	2.82E-03
cg06157570	LAG3	12	Body	S_Shelf	50.43	3.70	53.55	3.79	-3.12	1.15E-03	1.90E-02
cg05069835	UPK3B;UPK3B	7	TSS1500;TSS1500	Open sea	56.85	4.37	59.96	3.96	-3.12	2.99E-03	3.73E-02
cg04264129		7	Intergenic	Open sea	69.34	4.81	72.46	3.90	-3.11	1.61E-03	2.43E-02
cg21984481	NPLOC4	17	Body	Open sea	78.74	4.82	81.85	4.40	-3.11	4.13E-04	8.74E-03
cg18016128	C17orf76;C17orf76	17	TSS1500;TSS1500	S_Shore	83.64	4.13	86.76	2.40	-3.11	2.55E-04	6.00E-03
cg15732451		1	Intergenic	Open sea	44.04	4.95	47.15	4.45	-3.11	2.69E-03	3.48E-02
cg27634344	ZFP42	4	5'UTR	S_Shore	15.73	3.88	18.84	4.76	-3.11	2.21E-04	5.34E-03
cg00937359	SYNCRIP;SYNCRIP;SYNCRIP;SYNCRIP;S	6	Body;Body;Body;Body;Body;5'UTR	N_Shore	53.59	6.02	56.71	4.96	-3.11	1.93E-03	2.76E-02
cg08541497	C8orf76	8	TSS1500	S_Shore	37.05	4.33	40.16	6.00	-3.11	2.94E-03	3.69E-02
cg19861858		8	Intergenic	Open sea	46.60	3.43	49.71	4.70	-3.11	2.50E-03	3.30E-02
cg26853855	CSRNP3	2	TSS200	Open sea	66.46	4.85	69.57	4.73	-3.11	2.24E-03	3.06E-02
cg00688311	FBRSL1	12	Body	N_Shore	55.14	3.21	58.26	2.92	-3.11	1.36E-05	4.98E-04
cg09156097	LOC389333	5	1stExon	Island	36.92	4.44	40.03	5.14	-3.11	3.33E-03	4.03E-02
cg04380519	LIMD2	17	TSS1500	Island	13.79	2.78	16.91	4.01	-3.11	5.48E-05	1.68E-03
cg19535358		13	Intergenic	Island	76.96	3.12	80.07	3.67	-3.11	1.39E-05	5.06E-04
cg07537819		6	Intergenic	Open sea	75.32	4.17	78.43	3.53	-3.11	2.64E-04	6.17E-03
cg03075534		1	Intergenic	Island	11.93	3.89	15.04	3.53	-3.11	1.21E-04	3.28E-03
cg26699292		16	Intergenic	Island	15.82	3.30	18.93	3.54	-3.11	9.75E-06	3.68E-04
cg21691166		18	Intergenic	Island	18.61	3.26	21.73	3.97	-3.11	7.46E-05	2.19E-03
cg01816748	PDGFRB	5	Body	N_Shore	65.81	4.25	68.92	4.41	-3.11	2.02E-03	2.84E-02
cg02619464		8	Intergenic	Open sea	65.64	3.50	68.75	4.02	-3.11	7.24E-04	1.34E-02
cg02622209	EPN2;EPN2;EPN2	17	3'UTR;3'UTR;3'UTR	Open sea	85.41	4.33	88.52	4.20	-3.11	1.83E-03	2.66E-02
cg16001865	NFIA;NFIA;NFIA;NFIA	1	Body;Body;Body;Body	Open sea	47.59	5.70	50.71	3.70	-3.11	2.96E-03	3.71E-02
cg24683414	GFI1;GFI1	1	TSS1500;TSS200	Island	22.43	4.58	25.54	5.03	-3.11	6.88E-04	1.29E-02
cg11991058		2	Intergenic	Island	14.51	3.02	17.62	3.78	-3.11	1.31E-04	3.51E-03
cg03277560	NR4A3;NR4A3;NR4A3	9	5'UTR;5'UTR;5'UTR	Island	41.99	4.02	45.10	5.38	-3.11	1.74E-03	2.56E-02
cg17214902	ADAM8;ADAM8;ADAM8	10	Body;Body;Body	S_Shore	78.70	5.06	81.81	4.30	-3.11	2.44E-03	3.25E-02
cg01773144		8	Intergenic	N_Shore	66.73	5.16	69.84	4.02	-3.11	2.87E-03	3.63E-02
cg03321020		1	Intergenic	Open sea	38.38	3.49	41.49	3.55	-3.11	1.29E-04	3.45E-03
cg05091585		13	Intergenic	Open sea	34.86	4.42	37.97	4.79	-3.11	2.57E-03	3.37E-02
cg08194650	TTBK2	15	5'UTR	Open sea	57.81	4.35	60.92	3.57	-3.11	1.46E-04	3.82E-03
cg24921858	BCL2L14;BCL2L14;BCL2L14;BCL2L14	12	5'UTR;TSS1500;1stExon;TSS1500	Open sea	22.63	3.54	25.74	4.93	-3.11	2.27E-03	3.09E-02
cg13935577	BTBD11;BTBD11	12	Body;Body	Island	37.37	3.78	40.48	3.51	-3.11	3.88E-03	4.47E-02
cg02925601		6	Intergenic	S_Shore	18.45	4.65	21.56	4.78	-3.11	2.64E-03	3.43E-02
cg24279243	KLF3	4	5'UTR	Open sea	14.50	3.61	17.61	4.83	-3.11	4.16E-03	4.69E-02
cg03100209	CCDC155	19	3'UTR	Open sea	59.57	5.12	62.68	4.23	-3.11	6.40E-04	1.23E-02
cg07204724	TBC1D16	17	Body	Open sea	84.08	4.18	87.19	3.56	-3.11	2.56E-04	6.02E-03
cg11256152	TRAPPC9;TRAPPC9	8	Body;Body	S_Shore	70.31	5.25	73.42	5.33	-3.11	2.94E-03	3.69E-02
cg05821464	GRAMD4	22	Body	N_Shore	62.43	6.21	65.54	5.50	-3.11	3.62E-03	4.26E-02
cg07368507	REEP2	5	TSS1500	Open sea	18.91	3.76	22.02	4.34	-3.11	5.34E-04	1.07E-02
cg22078560	DDX52;DDX52	17	TSS1500;TSS1500	S_Shore	38.80	3.13	41.91	4.46	-3.11	1.47E-03	2.27E-02
cg20194364	HSP90AB1	6	Body	S_Shelf	61.22	5.37	64.33	4.66	-3.11	6.78E-04	1.28E-02
cg26821681		8	Intergenic	Open sea	62.70	6.30	65.81	5.98	-3.11	1.74E-03	2.57E-02
cg11500788	FZD3	8	5'UTR	S_Shelf	83.81	4.29	86.91	4.16	-3.11	3.54E-03	4.20E-02
cg17296447	MRGPRF;MRGPRF	11	Body;Body	S_Shore	32.21	4.02	35.32	4.28	-3.11	3.46E-03	4.13E-02
cg04542977	EPS15	1	Body	N_Shore	42.41	5.64	45.52	6.01	-3.11	5.13E-04	1.03E-02
cg15015980		1	Intergenic	N_Shelf	80.21	4.56	83.32	5.07	-3.11	3.70E-03	4.33E-02
cg14599091		13	Intergenic	S_Shore	39.35	4.41	42.46	4.61	-3.11	1.34E-03	2.13E-02
cg19276110	PLA2G5	1	TSS200	Open sea	29.80	5.45	32.91	5.03	-3.11	2.76E-03	3.54E-02
cg11653134		2	Intergenic	S_Shore	20.94	3.36	24.05	3.82	-3.11	1.22E-03	1.99E-02

cg23916205	GATA4	8	Body	Open sea	73.99	5.08	77.10	3.98	-3.11	3.96E-03	4.54E-02
cg16620526	LVRN	5	TSS200	Island	20.56	3.46	23.67	4.27	-3.11	7.91E-04	1.44E-02
cg01294327	LINGO3	19	Body	Island	23.25	3.43	26.36	3.60	-3.11	2.14E-03	2.96E-02
cg09365259	TUBA8	22	Body	Island	85.11	4.28	88.22	2.58	-3.11	3.34E-04	7.41E-03
cg11870561		7	Intergenic	Open sea	79.79	3.92	82.90	4.05	-3.11	1.57E-04	4.06E-03
cg25181684	SH3PXD2A	10	Body	S_Shelf	49.68	5.22	52.78	5.80	-3.11	9.68E-04	1.67E-02
cg23250019	SLC29A2	11	Body	N_Shore	74.99	4.28	78.09	4.04	-3.11	1.64E-03	2.46E-02
cg26816421	PISD	22	5'UTR	N_Shelf	61.86	4.12	64.96	4.57	-3.11	1.39E-03	2.18E-02
cg04606076		13	Intergenic	Open sea	57.28	5.33	60.39	5.03	-3.11	1.90E-03	2.73E-02
cg16141784	ACVR1;ACVR1	2	5'UTR;5'UTR	Open sea	83.65	4.26	86.76	3.63	-3.11	1.91E-05	6.70E-04
cg05798641	HLA-DPB1	6	Body	S_Shore	61.51	3.89	64.61	3.60	-3.11	4.37E-04	9.13E-03
cg23346408	ZMIZ1	10	Body	N_Shore	44.67	3.04	47.78	3.49	-3.11	3.37E-06	1.39E-04
cg21024969	QKI	6	3'UTR	Open sea	78.77	3.14	81.88	3.07	-3.11	5.42E-05	1.67E-03
cg06975914		1	Intergenic	Open sea	69.72	5.11	72.82	4.57	-3.11	9.63E-04	1.67E-02
cg12045999		12	Intergenic	Open sea	78.79	4.62	81.90	3.69	-3.11	1.06E-03	1.78E-02
cg07095230	TBX2	17	Body	Island	31.70	4.59	34.81	6.41	-3.11	8.84E-04	1.56E-02
cg14258504		12	Intergenic	Open sea	61.11	5.28	64.22	4.36	-3.11	1.68E-03	2.49E-02
cg23021168	PAM;PAM;PAM;PAM	5	TSS1500;TSS1500;TSS1500;TSS1500	N_Shore	50.75	5.48	53.86	4.81	-3.11	2.81E-03	3.58E-02
cg13729116	LETM1	4	TSS1500	S_Shore	66.49	4.22	69.60	3.29	-3.11	1.02E-03	1.74E-02
cg19710662	KIAA1026;KIAA1026;KIAA1026;KIAA1(	1	Body;TSS200;Body;Body	Open sea	49.24	4.63	52.35	3.87	-3.11	3.10E-03	3.83E-02
cg10708898		1	Intergenic	Open sea	48.63	3.44	51.74	4.03	-3.11	1.47E-04	3.85E-03
cg23290100	PRDM6	5	Body	S_Shore	32.53	8.34	35.64	7.29	-3.11	4.44E-03	4.90E-02
cg10887069	FAM20B	1	TSS1500	N_Shore	76.00	3.26	79.10	3.53	-3.11	6.87E-05	2.04E-03
cg24537993	PLEKHA6	1	5'UTR	Open sea	50.93	3.34	54.04	5.04	-3.11	1.00E-03	1.71E-02
cg09194742		10	Intergenic	Open sea	76.18	3.52	79.29	2.59	-3.11	8.20E-05	2.37E-03
cg23450481		19	Intergenic	S_Shelf	71.35	4.20	74.45	4.54	-3.11	7.63E-04	1.40E-02
cg03576425	CYC1	8	Body	S_Shore	44.67	3.59	47.78	4.12	-3.10	1.90E-03	2.73E-02
cg23313963	TNXB	6	Body	Open sea	68.23	4.17	71.33	3.72	-3.10	3.95E-04	8.43E-03
cg03646234	TMIE	3	Body	Island	30.12	5.23	33.22	6.65	-3.10	1.47E-03	2.27E-02
cg00289362		6	Intergenic	Open sea	82.64	4.34	85.74	3.39	-3.10	1.04E-03	1.76E-02
cg11721273	RFWD2;RFWD2	1	Body;Body	Open sea	83.78	5.01	86.89	3.68	-3.10	6.81E-04	1.28E-02
cg11801524	SVIL;SVIL	10	Body;Body	Open sea	75.39	4.44	78.50	4.14	-3.10	2.21E-03	3.03E-02
cg26687579		1	Intergenic	N_Shelf	36.07	4.80	39.18	3.95	-3.10	3.32E-03	4.02E-02
cg07618409	MSI2	17	Body	Open sea	67.98	3.67	71.09	3.55	-3.10	2.46E-04	5.83E-03
cg06649257	IPO11;IPO11	5	3'UTR;3'UTR	Open sea	73.64	4.26	76.75	3.95	-3.10	4.80E-04	9.82E-03
cg03219838	H2AFY;H2AFY;H2AFY;H2AFY	5	Body;Body;Body;Body	Open sea	76.10	4.52	79.21	3.57	-3.10	3.33E-03	4.03E-02
cg27367721	LYRM1;LYRM1;LYRM1	16	5'UTR;5'UTR;5'UTR	Open sea	71.71	4.44	74.81	4.12	-3.10	3.77E-03	4.38E-02
cg04673737		1	Intergenic	N_Shore	68.17	4.14	71.27	4.61	-3.10	6.72E-04	1.27E-02
cg17950410	TUBB;MDC1	6	TSS1500;TSS1500	N_Shore	53.01	4.06	56.11	5.68	-3.10	4.49E-04	9.32E-03
cg14618996	ADAM32	8	TSS200	N_Shore	13.37	2.97	16.47	4.92	-3.10	4.82E-04	9.85E-03
cg07371530	RPUSD1;CHTF18	16	TSS1500;Body	N_Shore	66.50	3.48	69.60	3.82	-3.10	1.41E-04	3.72E-03
cg14799927	TRIM26	6	5'UTR	Open sea	24.82	3.98	27.92	4.37	-3.10	2.84E-03	3.61E-02
cg10262052	IDO1	8	TSS1500	Open sea	63.79	6.52	66.89	5.46	-3.10	4.57E-04	9.45E-03
cg06130072	MUC6	11	TSS200	Island	42.99	3.22	46.09	4.12	-3.10	1.21E-03	1.98E-02
cg08985669	ZNF34	8	TSS1500	S_Shore	65.07	3.90	68.17	4.09	-3.10	8.25E-04	1.48E-02
cg02650667	APBB2;APBB2;APBB2	4	5'UTR;5'UTR;5'UTR	Open sea	83.35	3.72	86.45	3.11	-3.10	1.25E-04	3.37E-03
cg16775198	C12orf42;C12orf42	12	5'UTR;5'UTR	N_Shelf	56.85	3.22	59.95	3.24	-3.10	3.25E-04	7.24E-03
cg07576409	ZNF703	8	Body	Island	31.63	3.30	34.73	3.22	-3.10	4.20E-05	1.34E-03
cg26641935	CD109;CD109;CD109	6	Body;Body;Body	Open sea	66.90	5.42	70.00	4.58	-3.10	1.67E-04	4.27E-03
cg11321035		19	Intergenic	S_Shore	39.36	3.51	42.46	3.57	-3.10	1.70E-04	4.33E-03
cg02323296	C2orf53	2	TSS1500	Open sea	59.64	4.07	62.74	3.71	-3.10	7.80E-04	1.42E-02
cg05999049	HOXB8	17	TSS1500	S_Shore	25.95	3.52	29.05	3.69	-3.10	2.64E-04	6.16E-03
cg09333471	DFNA5;DFNA5;DFNA5	7	5'UTR;5'UTR;5'UTR	N_Shore	28.54	5.00	31.64	4.58	-3.10	4.04E-03	4.59E-02

cg11389618	GTDC1;GTDC1;GTDC1	2	Body;Body;Body	Open sea	82.05	5.66	85.15	5.75	-3.10	2.69E-03	3.47E-02
cg26152485	LOC729991-MEF2B;LOC729991-MEF2	19	Body;5'UTR;TSS1500;Body	Island	42.80	6.25	45.90	5.09	-3.10	1.89E-03	2.71E-02
cg12127159		7	Intergenic	Open sea	63.62	4.92	66.72	3.94	-3.10	1.62E-04	4.17E-03
cg20416574	FBXO11;FBXO11;FBXO11	2	5'UTR;5'UTR;5'UTR	Open sea	62.31	4.09	65.41	3.96	-3.10	3.11E-04	7.00E-03
cg26590064	LOC390595	15	Body	S_Shore	61.82	4.47	64.92	4.15	-3.10	9.51E-04	1.65E-02
cg10752753	SLC45A2;SLC45A2	5	TSS200;TSS200	Open sea	17.96	4.33	21.06	3.67	-3.10	3.03E-03	3.77E-02
cg00906476	CXXC5	5	5'UTR	N_Shore	66.06	3.95	69.16	3.26	-3.10	8.06E-04	1.46E-02
cg13259205	DKK3;DKK3;DKK3	11	TSS200;5'UTR;5'UTR	Island	17.83	3.54	20.93	3.97	-3.10	2.30E-05	7.90E-04
cg26915329		13	Intergenic	Open sea	39.43	4.82	42.53	4.70	-3.10	3.17E-03	3.89E-02
cg02986791	ABCA7	19	Body	N_Shore	87.72	5.63	90.82	4.21	-3.10	3.94E-03	4.52E-02
cg00243574	HOXC4;HOXC5;HOXC4;HOXC6;HOXC6	12	5'UTR;Body;1stExon;1stExon;5'UTR	N_Shore	12.13	2.34	15.23	2.54	-3.10	1.18E-07	5.87E-06
cg25219643	CCDC81;CCDC81	11	Body;Body	Open sea	42.96	4.76	46.06	3.95	-3.10	3.65E-04	7.93E-03
cg16103959	NR5A2;NR5A2	1	Body;Body	Island	50.58	4.98	53.68	4.63	-3.10	2.14E-03	2.96E-02
cg19871903		4	Intergenic	Open sea	65.21	4.68	68.31	4.75	-3.10	1.37E-03	2.16E-02
cg01862688	PCDHB3	5	1stExon	N_Shore	48.42	4.47	51.52	5.43	-3.10	1.63E-03	2.44E-02
cg14085952		9	Intergenic	Open sea	79.68	5.11	82.78	4.29	-3.10	1.59E-03	2.40E-02
cg00183886	USP46	4	Body	N_Shore	69.65	4.49	72.75	4.18	-3.10	2.22E-03	3.04E-02
cg13236481	C1orf105	1	Body	Open sea	87.55	3.44	90.65	2.59	-3.10	2.22E-05	7.66E-04
cg05496203	MEIS1	2	Body	S_Shelf	56.11	5.12	59.21	6.33	-3.10	5.10E-04	1.03E-02
cg14294077	C13orf16	13	Body	S_Shelf	64.71	5.06	67.80	5.06	-3.10	3.59E-03	4.24E-02
cg16606195		7	Intergenic	Island	46.59	4.05	49.69	4.90	-3.10	3.28E-03	3.99E-02
cg20536432	XRCC4;XRCC4;XRCC4	5	Body;Body;Body	Open sea	83.54	5.42	86.64	4.68	-3.10	1.59E-03	2.40E-02
cg01904145	PROK2;PROK2	3	Body;Body	N_Shelf	46.89	5.63	49.99	5.50	-3.10	3.67E-03	4.30E-02
cg04330881	CCNYL1;CCNYL1	2	Body;Body	Open sea	80.55	4.46	83.64	3.57	-3.10	2.14E-03	2.97E-02
cg05528623		1	Intergenic	Island	52.78	5.59	55.88	4.87	-3.10	1.41E-03	2.21E-02
cg13669080	AGPAT2;AGPAT2	9	Body;Body	S_Shelf	69.08	3.33	72.18	3.80	-3.10	4.65E-05	1.46E-03
cg10430077		5	Intergenic	N_Shore	40.19	4.82	43.28	5.50	-3.10	3.41E-03	4.09E-02
cg12484845	ZNF826	19	5'UTR	Island	50.98	3.00	54.08	2.92	-3.10	1.41E-04	3.72E-03
cg23906641	SOAT2;IGFBP6	12	TSS1500;3'UTR	Open sea	59.79	3.73	62.88	3.57	-3.10	4.57E-04	9.44E-03
cg21932416		2	Intergenic	N_Shelf	18.68	3.81	21.78	3.32	-3.10	1.97E-04	4.88E-03
cg18081338		6	Intergenic	Open sea	77.26	4.71	80.36	3.04	-3.10	1.54E-03	2.34E-02
cg20108357	BDNF;BDNF;BDNF;BDNF;BDNF;BDNF;	11	Body;5'UTR;Body;5'UTR;5'UTR;5'UTR;Body	N_Shelf	66.78	5.39	69.88	5.44	-3.10	2.25E-03	3.07E-02
cg10570754	NTF3	12	Body	Open sea	52.65	3.92	55.74	3.96	-3.10	2.66E-04	6.19E-03
cg18817158	PLXNA4	7	Body	Open sea	82.80	5.16	85.90	3.51	-3.10	1.72E-03	2.53E-02
cg26256901		1	Intergenic	Open sea	62.02	4.36	65.11	4.65	-3.10	8.42E-05	2.42E-03
cg19153828		2	Intergenic	Island	31.38	4.21	34.48	4.07	-3.10	4.20E-03	4.72E-02
cg02928797	PLEKHG5	1	Body	Open sea	79.98	3.60	83.07	4.13	-3.10	7.27E-05	2.14E-03
cg13681752		1	Intergenic	Open sea	80.20	4.84	83.30	3.52	-3.10	9.81E-04	1.69E-02
cg20936107	CDK12;CDK12	17	3'UTR;3'UTR	Open sea	66.89	5.07	69.99	4.19	-3.10	1.09E-03	1.83E-02
cg02427576	GRAMD3;GRAMD3;GRAMD3;GRAMD	5	Body;Body;Body;Body;Body	Open sea	75.08	5.67	78.18	4.76	-3.10	3.38E-03	4.06E-02
cg01944288	NTNG2	9	TSS1500	N_Shore	38.10	4.78	41.19	5.26	-3.10	2.87E-03	3.63E-02
cg20170809	TNXB	6	Body	N_Shelf	72.14	4.43	75.24	4.00	-3.10	7.14E-05	2.11E-03
cg05456713	GNG7	19	3'UTR	Island	75.56	5.25	78.65	6.59	-3.10	3.89E-03	4.48E-02
cg23015917	SLC35D1	1	Body	Open sea	65.64	5.04	68.74	4.75	-3.09	2.21E-03	3.03E-02
cg13201187	SDHB	1	Body	N_Shelf	67.90	3.28	71.00	3.83	-3.09	1.79E-05	6.34E-04
cg04852275	HSPG2	1	Body	S_Shore	79.26	3.79	82.36	3.96	-3.09	5.28E-05	1.63E-03
cg26772854		3	Intergenic	Island	12.03	2.81	15.13	3.64	-3.09	2.36E-05	8.09E-04
cg01885839		5	Intergenic	Open sea	78.32	3.88	81.42	2.71	-3.09	2.14E-04	5.21E-03
cg25590444		1	Intergenic	Open sea	59.09	3.41	62.19	2.88	-3.09	3.45E-04	7.58E-03
cg19747271	SCAND3	6	Body	N_Shore	67.87	6.28	70.96	5.28	-3.09	1.40E-04	3.70E-03
cg24789335	TOP1MT	8	Body	Island	83.29	5.59	86.39	4.09	-3.09	4.01E-03	4.58E-02
cg04215287	WWTR1;WWTR1;WWTR1	3	Body;Body;Body	Open sea	81.78	4.40	84.87	5.71	-3.09	2.34E-03	3.15E-02
cg17122437	FAM107B	10	1stExon	Open sea	81.68	4.11	84.77	3.53	-3.09	4.80E-07	2.25E-05

cg03614193	ST8SIA3	18	Body	Island	47.08	5.85	50.17	4.48	-3.09	3.62E-03	4.26E-02
cg16377117	LRP1	12	Body	Open sea	71.95	4.28	75.05	3.13	-3.09	5.58E-04	1.10E-02
cg22899037	CPNE3	8	Body	Open sea	77.44	3.72	80.53	4.30	-3.09	5.18E-05	1.60E-03
cg12627844	VPS54;VPS54	2	5'UTR;5'UTR	N_Shore	17.08	2.92	20.17	3.76	-3.09	2.83E-04	6.51E-03
cg21819782		2	Intergenic	Open sea	48.23	5.29	51.32	4.72	-3.09	7.72E-04	1.41E-02
cg19215764		6	Intergenic	Open sea	68.20	4.59	71.29	4.74	-3.09	3.19E-03	3.91E-02
cg04275695	ZC3H12D	6	TSS200	Open sea	54.87	5.36	57.96	5.54	-3.09	1.21E-04	3.28E-03
cg22369009		19	Intergenic	N_Shelf	59.32	2.78	62.41	3.64	-3.09	1.70E-05	6.05E-04
cg12055316	ZNF513	2	Body	Island	68.99	4.57	72.08	2.91	-3.09	2.06E-03	2.89E-02
cg16667251	SPTBN1	2	5'UTR	Open sea	49.08	5.94	52.17	5.30	-3.09	6.01E-04	1.17E-02
cg22146245		12	Intergenic	N_Shelf	81.04	4.97	84.14	3.94	-3.09	2.31E-03	3.12E-02
cg25959798	PAPOLG	2	3'UTR	Open sea	77.93	5.03	81.02	4.79	-3.09	2.56E-03	3.35E-02
cg13570660	ZNF483;ZNF483	9	TSS200;TSS200	N_Shore	41.89	4.42	44.98	4.28	-3.09	4.36E-04	9.12E-03
cg01008361	GEMIN7;GEMIN7;GEMIN7	19	Body;Body;Body	N_Shore	87.51	2.89	90.60	1.90	-3.09	1.84E-07	8.96E-06
cg02060465	SPRR4	1	TSS1500	Open sea	77.81	4.36	80.90	3.54	-3.09	1.31E-04	3.50E-03
cg12150784		11	Intergenic	Island	19.91	4.18	23.01	4.71	-3.09	1.71E-03	2.52E-02
cg11657155	GSTCD	4	3'UTR	Open sea	81.36	4.83	84.45	3.93	-3.09	7.25E-04	1.35E-02
cg26233373	ADORA3;ADORA3;ADORA3	1	Body;TSS1500;TSS1500	Open sea	77.21	4.08	80.30	4.28	-3.09	5.55E-04	1.10E-02
cg20345948		4	Intergenic	Island	45.40	4.22	48.49	4.01	-3.09	6.17E-04	1.19E-02
cg23454038	FOXD1;FOXD1	5	3'UTR;1stExon	S_Shore	12.96	2.59	16.05	3.12	-3.09	1.97E-06	8.44E-05
cg18504014	ZBTB38	3	3'UTR	Open sea	81.31	4.18	84.40	4.07	-3.09	3.11E-04	7.00E-03
cg16703420		14	Intergenic	Open sea	69.30	5.44	72.39	5.04	-3.09	4.39E-03	4.86E-02
cg04542030	BOLA3;BOLA3	2	TSS1500;TSS1500	S_Shore	42.95	4.70	46.04	5.91	-3.09	1.00E-03	1.71E-02
cg09278723	SLC25A21;SLC25A21	14	Body;Body	Open sea	83.77	3.86	86.86	3.95	-3.09	7.79E-05	2.27E-03
cg17821664	OSTBETA;OSTBETA	15	1stExon;5'UTR	Open sea	34.19	3.51	37.28	4.22	-3.09	8.29E-04	1.49E-02
cg08327690	EPS8L2	11	Body	Island	27.68	4.86	30.77	3.98	-3.09	7.84E-04	1.43E-02
cg21406402	LOC84931	2	Body	Open sea	57.29	3.99	60.38	4.17	-3.09	8.60E-04	1.53E-02
cg10409860	PLCG2	16	Body	Open sea	87.29	4.44	90.38	2.53	-3.09	8.03E-04	1.45E-02
cg23381232	DYNC1I1;DYNC1I1;DYNC1I1	7	Body;Body;Body	Open sea	70.20	6.32	73.29	5.37	-3.09	3.10E-04	6.98E-03
cg08034070		17	Intergenic	Island	33.60	5.17	36.69	4.78	-3.09	7.22E-04	1.34E-02
cg26025224	MRPS11;MRPS11	15	Body;Body	Open sea	25.27	3.46	28.36	3.68	-3.09	9.14E-04	1.60E-02
cg24938077		1	Intergenic	N_Shore	40.42	2.68	43.51	2.68	-3.09	4.61E-05	1.45E-03
cg25680628	MAP3K8	10	5'UTR	S_Shore	13.95	4.48	17.04	4.94	-3.09	2.77E-03	3.54E-02
cg19992906	TMEM132B	12	Body	Open sea	75.59	5.34	78.68	5.31	-3.09	2.48E-03	3.28E-02
cg04959634	HOXA10	7	Body	N_Shore	19.00	3.33	22.09	4.66	-3.09	2.67E-04	6.21E-03
cg01861537		11	Intergenic	Open sea	69.18	4.74	72.27	5.05	-3.09	4.51E-03	4.96E-02
cg04527918	UCN	2	TSS200	Island	35.48	4.28	38.57	4.13	-3.09	3.11E-03	3.83E-02
cg27116863	FAM190A	4	Body	Open sea	72.42	5.01	75.51	3.56	-3.09	1.15E-03	1.90E-02
cg25123427		3	Intergenic	Open sea	67.82	5.13	70.91	4.65	-3.09	7.44E-04	1.37E-02
cg25874953	ANP32A	15	Body	Open sea	55.72	4.34	58.81	4.43	-3.09	4.18E-04	8.81E-03
cg15205547		7	Intergenic	Island	17.24	2.75	20.33	3.02	-3.09	2.67E-07	1.29E-05
cg03666500		6	Intergenic	Open sea	16.71	3.59	19.79	4.23	-3.09	1.80E-04	4.55E-03
cg07134608	DHDH	19	TSS1500	N_Shore	32.09	4.57	35.17	4.48	-3.09	2.83E-04	6.50E-03
cg09851465	C1orf87	1	TSS1500	S_Shore	14.54	3.68	17.62	5.95	-3.09	4.18E-03	4.70E-02
cg05977992	MTHFD1L	6	TSS1500	N_Shore	35.24	4.49	38.33	4.51	-3.09	5.11E-04	1.03E-02
cg14923188		6	Intergenic	Open sea	82.33	5.18	85.41	4.20	-3.09	8.85E-04	1.56E-02
cg08204843	BDH1;BDH1;BDH1	3	3'UTR;3'UTR;3'UTR	Open sea	6.76	2.41	9.85	4.85	-3.09	6.24E-05	1.88E-03
cg07948968	SEPT12;SEPT12;LOC440335;LOC44033	16	5'UTR;5'UTR;TSS1500;TSS1500	Open sea	62.82	4.23	65.90	4.63	-3.09	1.01E-04	2.83E-03
cg15707163		7	Intergenic	Open sea	74.82	4.61	77.90	4.41	-3.09	1.12E-04	3.07E-03
cg17736116	ZNF280D;ZNF280D	15	3'UTR;3'UTR	Open sea	89.33	5.14	92.42	3.67	-3.09	3.69E-03	4.32E-02
cg11870956	NRM;KIAA1949	6	Body;TSS1500	S_Shore	76.90	4.04	79.99	3.47	-3.09	8.09E-04	1.46E-02
cg20624698	LOC339788	2	Body	Open sea	61.53	3.87	64.62	3.60	-3.09	1.25E-03	2.02E-02
cg22094599	CCRN4L	4	Body	Open sea	69.12	4.39	72.21	4.21	-3.09	1.17E-03	1.93E-02

cg07349217	TG	8	TSS1500	Open sea	76.56	5.08	79.65	4.16	-3.09	2.16E-03	2.98E-02
cg18588794	CDC42BPB	14	Body	Open sea	73.57	3.87	76.66	3.06	-3.09	7.86E-04	1.43E-02
cg12036829	ANKMY1;ANKMY1	2	Body;Body	Open sea	74.45	5.81	77.53	4.95	-3.09	3.78E-03	4.39E-02
cg23062198	FAM84A	2	5'UTR	Island	26.35	4.70	29.43	5.03	-3.09	4.52E-03	4.96E-02
cg17806224	WIPF3	7	Body	S_Shelf	80.66	3.99	83.75	3.94	-3.08	5.99E-04	1.16E-02
cg02479691	ZNF707;ZNF707;ZNF707	8	Body;Body;Body	N_Shelf	71.82	3.85	74.90	3.99	-3.08	1.93E-03	2.76E-02
cg17500055	GNG4;GNG4;GNG4	1	5'UTR;5'UTR;5'UTR	Island	53.33	5.29	56.41	6.50	-3.08	8.58E-04	1.53E-02
cg17608082	SEMA5A	5	Body	Open sea	65.20	5.49	68.28	4.32	-3.08	4.19E-03	4.71E-02
cg26780915		7	Intergenic	S_Shore	84.68	4.07	87.77	3.68	-3.08	8.75E-04	1.55E-02
cg18966495		6	Intergenic	Open sea	84.48	4.64	87.57	3.17	-3.08	2.68E-03	3.46E-02
cg25542244	FNTA;FNTA;FNTA	8	TSS1500;TSS1500;TSS1500	N_Shore	69.85	3.30	72.93	2.84	-3.08	1.50E-05	5.41E-04
cg04394267		3	Intergenic	S_Shore	43.96	5.25	47.05	6.69	-3.08	3.27E-03	3.98E-02
cg00669964	SEC63	6	3'UTR	Open sea	81.34	4.02	84.43	4.00	-3.08	1.07E-04	2.96E-03
cg19856701	NF1;NF1;NF1	17	3'UTR;Body;Body	Open sea	84.63	4.62	87.71	3.97	-3.08	5.24E-04	1.05E-02
cg03755177	CAMK2B;CAMK2B;CAMK2B;CAMK2B;	7	Body;Body;Body;Body;Body;Body;Body;Bo	Island	24.89	5.64	27.97	8.24	-3.08	3.87E-03	4.46E-02
cg11540707	IDE;IDE	10	Body;5'UTR	Open sea	82.02	4.32	85.10	3.49	-3.08	4.52E-04	9.37E-03
cg14360917	SP2	17	Body	Open sea	70.20	4.65	73.28	3.56	-3.08	1.56E-03	2.37E-02
cg13092082		1	Intergenic	Open sea	21.66	3.74	24.75	4.20	-3.08	2.98E-03	3.73E-02
cg01480708	MYO1D	17	Body	Island	45.28	4.90	48.36	5.52	-3.08	8.91E-04	1.57E-02
cg21172377	HOXA10;HOXA10	7	Body;1stExon	Island	28.40	3.41	31.48	3.76	-3.08	6.26E-05	1.89E-03
cg04851702	CMIP;CMIP;CMIP	16	1stExon;5'UTR;Body	Open sea	62.78	4.46	65.86	3.88	-3.08	1.32E-03	2.11E-02
cg13742532	CCBP2	3	TSS200	Open sea	72.64	2.96	75.73	3.21	-3.08	2.26E-05	7.79E-04
cg04421553	VILL	3	Body	S_Shore	6.47	2.24	9.55	3.48	-3.08	2.22E-06	9.43E-05
cg11777506		2	Intergenic	Open sea	71.59	5.68	74.68	5.47	-3.08	4.73E-04	9.70E-03
cg00271311	CNTF;ZFP91-CNTF	11	TSS1500;Body	Open sea	83.78	7.38	86.86	3.03	-3.08	2.03E-03	2.86E-02
cg17452615	KDM2B;KDM2B	12	TSS200;TSS1500	S_Shore	44.99	5.13	48.07	4.99	-3.08	8.28E-04	1.49E-02
cg19267457	ROR1;ROR1	1	Body;Body	Open sea	54.08	4.30	57.16	4.14	-3.08	2.64E-03	3.43E-02
cg11817804	KIAA0895	7	Body	N_Shelf	75.87	3.28	78.95	4.17	-3.08	2.67E-04	6.21E-03
cg02300809	PRSS33	16	3'UTR	N_Shore	86.52	4.35	89.60	4.20	-3.08	4.68E-04	9.63E-03
cg11522905	LRRC47	1	Body	S_Shelf	69.66	4.37	72.74	3.06	-3.08	5.28E-04	1.06E-02
cg01886988	IFITM1	11	TSS1500	S_Shore	26.12	4.59	29.20	4.03	-3.08	2.08E-03	2.90E-02
cg04837832		10	Intergenic	Island	25.18	3.52	28.26	3.48	-3.08	9.20E-05	2.61E-03
cg18377133	WTIP	19	Body	S_Shelf	65.75	4.98	68.84	3.58	-3.08	2.94E-03	3.70E-02
cg20305595	H6PD	1	TSS1500	N_Shore	39.97	3.81	43.05	4.85	-3.08	1.73E-03	2.55E-02
cg19432993	HOXA2	7	TSS1500	Island	30.52	3.53	33.60	3.76	-3.08	1.01E-03	1.72E-02
cg11131508		11	Intergenic	Open sea	71.06	3.60	74.14	3.16	-3.08	2.62E-05	8.87E-04
cg25851671		21	Intergenic	Island	15.98	5.31	19.06	5.82	-3.08	1.81E-03	2.64E-02
cg05921324	APOA4	11	TSS200	S_Shore	65.47	5.78	68.55	6.12	-3.08	3.65E-03	4.29E-02
cg06401197	PCGF3	4	Body	N_Shelf	78.84	4.19	81.92	3.67	-3.08	1.18E-03	1.94E-02
cg21937152	PARP8	5	Body	Open sea	70.64	3.13	73.72	3.34	-3.08	5.58E-05	1.71E-03
cg10300293	ERICH1	8	Body	Open sea	59.32	3.31	62.40	2.84	-3.08	1.48E-04	3.87E-03
cg04167075	C14orf138;C14orf138	14	3'UTR;3'UTR	Open sea	76.64	3.67	79.73	3.56	-3.08	2.20E-04	5.34E-03
cg07087850	RGL2;RGL2	6	Body;Body	N_Shore	81.89	4.19	84.98	2.83	-3.08	2.94E-04	6.69E-03
cg25475538	SORBS3	8	Body	Open sea	71.18	4.12	74.27	3.48	-3.08	2.95E-04	6.71E-03
cg01371420		2	Intergenic	S_Shelf	80.38	4.41	83.46	3.65	-3.08	3.32E-04	7.37E-03
cg11175192	WNT6	2	Body	Island	21.72	3.68	24.80	4.76	-3.08	2.27E-04	5.47E-03
cg16040614	PRRT1	6	Body	S_Shore	73.50	4.18	76.58	4.66	-3.08	2.03E-03	2.86E-02
cg24163448	RPSAP9	9	TSS200	S_Shelf	72.29	4.30	75.37	3.74	-3.08	4.91E-04	9.99E-03
cg08799394	PDLIM1	10	Body	N_Shore	25.10	3.45	28.18	4.38	-3.08	6.50E-04	1.24E-02
cg14643586		9	Intergenic	Island	23.57	3.64	26.65	4.63	-3.08	3.31E-03	4.01E-02
cg22156632	WNT6	2	Body	S_Shore	38.82	4.35	41.90	3.93	-3.08	6.08E-04	1.18E-02
cg12723425		11	Intergenic	S_Shelf	40.95	4.19	44.03	4.28	-3.08	3.94E-03	4.52E-02
cg11521969		7	Intergenic	Island	35.32	3.52	38.40	3.06	-3.08	1.01E-04	2.83E-03

cg06800962	TM4SF1	3	TSS1500	Open sea	57.57	7.58	60.65	6.29	-3.08	2.35E-03	3.16E-02
cg05939041	ITPK1;ITPK1;ITPK1	14	Body;Body;Body	Open sea	67.36	4.24	70.43	4.12	-3.08	1.11E-03	1.86E-02
cg10850725	ZNF233	19	5'UTR	S_Shore	15.84	4.06	18.92	4.82	-3.08	6.64E-04	1.26E-02
cg26757793	MMP20	11	TSS200	Open sea	76.70	4.76	79.78	3.92	-3.08	3.68E-04	7.99E-03
cg27507254	CLDN19;CLDN19	1	TSS200;TSS200	S_Shore	52.83	3.68	55.91	4.59	-3.08	2.26E-03	3.08E-02
cg05146436	C2orf81	2	TSS1500	N_Shore	55.94	3.28	59.02	3.70	-3.08	2.12E-05	7.36E-04
cg02366262		11	Intergenic	N_Shore	37.80	4.37	40.88	4.20	-3.08	2.03E-03	2.85E-02
cg01327685		13	Intergenic	N_Shore	76.73	4.23	79.80	3.34	-3.08	4.43E-04	9.22E-03
cg04178207	GTF2H4	6	Body	N_Shelf	71.01	4.03	74.09	3.80	-3.08	5.27E-04	1.05E-02
cg07560510	CA3	8	TSS1500	N_Shore	14.07	3.73	17.15	5.35	-3.08	1.44E-03	2.24E-02
cg22970357	EN1	2	3'UTR	Island	25.07	3.40	28.15	3.34	-3.08	1.45E-05	5.26E-04
cg03811829	ISG15	1	Body	Island	75.82	4.51	78.90	4.52	-3.08	1.19E-03	1.95E-02
cg22916586	DUSP16	12	5'UTR	Open sea	75.43	4.01	78.51	3.85	-3.08	1.47E-05	5.31E-04
cg08099115	ERC2	3	3'UTR	Open sea	59.97	4.52	63.05	3.96	-3.08	2.68E-04	6.24E-03
cg03040513	STAB2	12	Body	Open sea	80.66	5.16	83.74	4.61	-3.08	1.91E-03	2.74E-02
cg04922763	LMOD1	1	Body	Open sea	75.09	4.49	78.17	5.19	-3.08	8.96E-04	1.58E-02
cg08260959	HIST1H4F	6	1stExon	Island	23.24	3.48	26.32	3.77	-3.08	2.45E-04	5.81E-03
cg19664267	B3GALT4;B3GALT4	6	1stExon;5'UTR	Island	54.97	5.73	58.05	4.96	-3.08	4.47E-03	4.93E-02
cg13609544		17	Intergenic	Open sea	58.60	4.80	61.67	4.14	-3.08	2.84E-03	3.61E-02
cg00583733	ALDOA;ALDOA;ALDOA	16	TSS1500;TSS1500;5'UTR	N_Shore	61.70	4.67	64.78	4.54	-3.08	3.87E-03	4.46E-02
cg09332655	PTK7;PTK7;PTK7;PTK7	6	Body;Body;Body;Body	Island	41.95	4.75	45.03	5.34	-3.08	8.11E-04	1.46E-02
cg24987622	PDE2A;PDE2A;PDE2A;PDE2A	11	3'UTR;3'UTR;Body;3'UTR	Open sea	59.89	3.73	62.97	4.36	-3.08	1.40E-04	3.70E-03
cg24630195	IRX2;IRX2	5	Body;Body	Island	23.35	3.76	26.43	3.73	-3.08	6.81E-04	1.28E-02
cg13468680	NOTCH1	9	Body	N_Shore	81.34	3.76	84.42	3.31	-3.08	1.02E-04	2.84E-03
cg02749843		22	Intergenic	Island	65.38	4.45	68.45	4.46	-3.08	4.45E-03	4.91E-02
cg01632474	PDCD1	2	Body	N_Shore	77.83	4.19	80.91	3.47	-3.08	4.96E-05	1.54E-03
cg19382697	GNG7	19	5'UTR	S_Shore	79.01	4.23	82.08	3.82	-3.08	1.44E-03	2.23E-02
cg26286961	CSGALNACT1;CSGALNACT1;CSGALNA(	8	TSS200;Body;5'UTR	S_Shore	21.89	3.88	24.97	4.30	-3.08	4.42E-03	4.88E-02
cg05599723	TNFRSF1B	1	Body	Open sea	73.68	4.36	76.75	4.43	-3.08	1.15E-03	1.90E-02
cg20309975		8	Intergenic	Island	63.02	4.26	66.09	4.69	-3.08	4.52E-04	9.37E-03
cg05384139	PITPNM2	12	5'UTR	Open sea	65.69	4.51	68.77	4.75	-3.08	2.15E-04	5.23E-03
cg23031493	LHX5	12	Body	Island	18.89	3.79	21.97	3.94	-3.07	1.58E-04	4.09E-03
cg05516100		17	Intergenic	N_Shelf	78.81	3.61	81.89	3.80	-3.07	5.50E-06	2.18E-04
cg14601416	COL8A2	1	Body	Open sea	65.20	4.56	68.28	4.37	-3.07	5.69E-04	1.12E-02
cg04223671		1	Intergenic	Island	19.27	5.90	22.34	7.24	-3.07	2.05E-03	2.88E-02
cg06433350	OSBPL5;OSBPL5;OSBPL5	11	Body;Body;Body	Island	55.13	4.28	58.21	4.00	-3.07	2.23E-03	3.05E-02
cg25469870	PINK1	1	Body	S_Shelf	78.50	3.95	81.58	3.28	-3.07	3.15E-04	7.06E-03
cg14703589	LIMK2;LIMK2;LIMK2;LIMK2;LIMK2	22	1stExon;1stExon;5'UTR;Body;5'UTR	Open sea	72.56	4.91	75.63	4.20	-3.07	1.58E-03	2.39E-02
cg16948053		3	Intergenic	Island	14.72	3.12	17.79	3.27	-3.07	4.07E-06	1.66E-04
cg26566407	SKI	1	Body	Open sea	63.46	3.70	66.53	4.02	-3.07	3.00E-03	3.74E-02
cg08139234	EPB41;EPB41;EPB41;EPB41;EPB41;EPI	1	5'UTR;5'UTR;5'UTR;TSS1500;5'UTR;5'UTR	N_Shore	21.18	3.19	24.25	4.40	-3.07	4.51E-04	9.36E-03
cg27297176	TRIM43;TRIM43	2	5'UTR;1stExon	Open sea	85.81	5.34	88.88	3.56	-3.07	3.85E-03	4.45E-02
cg05105016	PIAS1	15	Body	Open sea	61.23	3.56	64.31	3.67	-3.07	2.66E-04	6.21E-03
cg15492820	SYT7	11	Body	Island	18.47	4.12	21.54	5.10	-3.07	1.66E-03	2.47E-02
cg09307412		8	Intergenic	Open sea	72.57	5.21	75.64	4.26	-3.07	2.68E-03	3.47E-02
cg11146821	L1TD1;L1TD1	1	5'UTR;5'UTR	S_Shelf	39.61	5.14	42.68	5.62	-3.07	6.04E-04	1.17E-02
cg08198713	KLRC1;KLRC1;KLRC1;KLRC1	12	Body;Body;Body;Body	Open sea	73.87	4.91	76.95	3.67	-3.07	1.21E-03	1.97E-02
cg01751992	DCUN1D4;DCUN1D4	4	Body;Body	S_Shore	41.26	3.72	44.33	4.64	-3.07	2.93E-03	3.68E-02
cg06952752	MARVELD3;MARVELD3	16	Body;Body	S_Shore	7.45	2.38	10.53	3.60	-3.07	1.19E-06	5.29E-05
cg26668276	DLX6AS	7	Body	N_Shore	18.37	2.99	21.45	3.24	-3.07	3.34E-06	1.38E-04
cg03284866		8	Intergenic	Island	10.88	3.82	13.96	5.71	-3.07	4.55E-03	4.98E-02
cg09858655		2	Intergenic	Island	21.89	2.38	24.97	3.37	-3.07	1.13E-05	4.19E-04
cg11555257		8	Intergenic	N_Shelf	57.26	4.00	60.34	3.75	-3.07	3.06E-04	6.90E-03

cg04560098	TRIM10;TRIM10	6	Body;Body	Open sea	45.05	4.00	48.12	4.17	-3.07	2.52E-03	3.32E-02
cg17232217	LOC100130872;LOC100130872-SPON2	4	TSS1500;TSS1500	Island	77.71	5.18	80.79	4.30	-3.07	2.26E-03	3.08E-02
cg27307729		5	Intergenic	N_Shore	21.38	3.66	24.45	4.06	-3.07	3.56E-04	7.77E-03
cg23801168	NAV1	1	TSS1500	N_Shore	79.75	4.28	82.83	3.77	-3.07	1.40E-03	2.19E-02
cg00157043	LDLRAD3	11	TSS1500	N_Shore	22.57	4.26	25.65	4.37	-3.07	1.47E-04	3.84E-03
cg17663463	PEG3;PEG3;ZIM2;PEG3;PEG3;ZIM2;ZII	19	5'UTR;5'UTR;5'UTR;5'UTR;5'UTR;5'UTR;5'U	N_Shore	54.42	4.37	57.49	4.25	-3.07	7.29E-04	1.35E-02
cg04704198		11	Intergenic	Open sea	80.39	4.08	83.46	3.73	-3.07	5.88E-04	1.15E-02
cg21029045	PITX2;PITX2	4	Body;Body	N_Shore	20.34	3.13	23.41	3.18	-3.07	2.60E-05	8.82E-04
cg26103805	UPK2	11	3'UTR	Open sea	72.59	4.52	75.66	3.36	-3.07	9.56E-04	1.66E-02
cg21836358	FOXD3;FOXD3	1	3'UTR;1stExon	Island	22.13	3.41	25.20	3.82	-3.07	6.10E-05	1.84E-03
cg03489020	MITF;MITF	3	Body;Body	Open sea	79.54	3.65	82.61	3.98	-3.07	7.11E-06	2.76E-04
cg06402401		4	Intergenic	Open sea	75.83	4.05	78.91	3.87	-3.07	1.32E-04	3.52E-03
cg26753088	EFNA2	19	Body	N_Shore	82.63	3.68	85.70	2.63	-3.07	8.87E-06	3.38E-04
cg12253469	MPST;MPST;MPST;MPST	22	Body;Body;Body;Body	Island	38.50	3.89	41.57	4.57	-3.07	2.09E-03	2.92E-02
cg25461513	CADM1;CADM1	11	Body;Body	Open sea	84.67	4.90	87.74	3.62	-3.07	6.31E-04	1.21E-02
cg04582295	SGCA;SGCA	17	TSS1500;TSS1500	S_Shelf	83.96	4.30	87.03	3.39	-3.07	8.94E-04	1.58E-02
cg03852656	RFC4;RFC4	3	TSS1500;TSS1500	S_Shore	64.22	4.06	67.29	4.67	-3.07	1.38E-04	3.65E-03
cg05684266		3	Intergenic	Open sea	32.20	3.58	35.27	4.13	-3.07	2.41E-04	5.73E-03
cg02804275		12	Intergenic	Open sea	69.79	4.68	72.86	3.55	-3.07	3.17E-03	3.89E-02
cg04998634	KLF16	19	Body	Island	29.41	4.65	32.48	4.49	-3.07	8.15E-04	1.47E-02
cg13448906	IGFBP5	2	Body	N_Shore	11.61	3.22	14.68	4.39	-3.07	9.02E-06	3.43E-04
cg02003117	SLC9A8	20	TSS1500	N_Shore	64.86	4.22	67.93	3.28	-3.07	6.13E-04	1.19E-02
cg14523095	GCLC	6	Body	Open sea	86.00	3.56	89.07	2.37	-3.07	3.25E-05	1.07E-03
cg00321007	CDRT4	17	3'UTR	Open sea	31.83	3.19	34.90	4.31	-3.07	1.50E-03	2.30E-02
cg24544003		6	Intergenic	Open sea	83.19	4.59	86.26	3.67	-3.07	4.70E-04	9.66E-03
cg06890747	LOC646999	7	Body	Island	15.89	2.98	18.96	3.80	-3.07	1.27E-04	3.41E-03
cg23873338	OGDHL;OGDHL;OGDHL	10	Body;5'UTR;Body	N_Shelf	84.41	3.75	87.48	3.22	-3.07	1.20E-04	3.25E-03
cg09567511	JAKMIP1;JAKMIP1	4	5'UTR;5'UTR	Open sea	45.90	4.87	48.97	3.32	-3.07	4.31E-03	4.80E-02
cg01720920	ZFYVE21	14	Body	S_Shelf	63.96	4.09	67.03	4.44	-3.07	4.26E-03	4.76E-02
cg00078759	IFT140;TMEM204	16	Body;Body	S_Shore	79.66	4.90	82.72	4.22	-3.07	2.33E-04	5.58E-03
cg21234561	TXNRD1;TXNRD1;TXNRD1;EID3;TXNRI	12	5'UTR;5'UTR;Body;TSS200;Body;5'UTR	Island	18.91	5.39	21.97	5.17	-3.07	3.55E-03	4.21E-02
cg19210858	ELF2	4	5'UTR	Open sea	74.29	4.98	77.36	5.79	-3.07	3.25E-03	3.95E-02
cg03775330	DPP9	19	Body	Island	76.80	4.58	79.87	4.95	-3.07	4.45E-03	4.91E-02
cg18810947	TNPO2;TNPO2;TNPO2	19	Body;Body;Body	N_Shore	47.38	3.93	50.44	4.44	-3.07	2.42E-04	5.76E-03
cg11158430	UTS2D	3	5'UTR	Open sea	85.93	5.06	89.00	4.48	-3.07	8.75E-04	1.55E-02
cg15066416	LOC100272146;C17orf57	17	Body;Body	Island	27.09	5.59	30.15	4.71	-3.07	4.20E-03	4.71E-02
cg15976910	MAP3K11	11	Body	S_Shore	79.37	4.60	82.44	3.31	-3.07	1.39E-03	2.18E-02
cg16838723	PDGFRA	4	Body	Open sea	74.64	3.81	77.71	2.97	-3.07	1.35E-05	4.94E-04
cg22361106		1	Intergenic	Open sea	59.39	4.75	62.46	4.05	-3.07	2.69E-04	6.25E-03
cg08194212	YTHDC1;YTHDC1	4	Body;Body	Open sea	67.06	3.50	70.13	2.31	-3.07	1.66E-04	4.25E-03
cg00344445	FLI1;FLI1	11	Body;Body	Open sea	25.24	2.79	28.31	3.67	-3.07	2.08E-04	5.10E-03
cg14340889	LTBP2	14	Body	Open sea	72.52	3.41	75.58	3.97	-3.07	2.17E-05	7.52E-04
cg05873359	HLA-F;HLA-F;HLA-F	6	TSS1500;TSS1500;TSS1500	N_Shore	42.52	3.95	45.58	3.28	-3.07	2.09E-03	2.92E-02
cg24487489	CUTA;CUTA;CUTA;CUTA;CUTA	6	3'UTR;3'UTR;3'UTR;3'UTR;3'UTR	N_Shore	69.01	4.21	72.08	4.12	-3.07	1.02E-04	2.84E-03
cg08362210	MIR196B	7	TSS1500	S_Shore	21.25	3.19	24.31	3.12	-3.07	3.11E-05	1.03E-03
cg19005438	FOXB1	15	TSS1500	N_Shore	28.14	3.40	31.21	4.55	-3.07	4.06E-04	8.62E-03
cg00584840		3	Intergenic	Open sea	25.52	3.17	28.59	3.77	-3.07	9.90E-05	2.78E-03
cg05468429	RREB1;RREB1;RREB1;RREB1	6	Body;Body;Body;Body	S_Shore	79.58	2.92	82.65	2.41	-3.07	1.17E-05	4.35E-04
cg04660410	VILL	3	Body	S_Shore	7.61	2.44	10.67	3.99	-3.07	5.47E-05	1.68E-03
cg07035454	OXR1	8	Body	Open sea	78.15	5.57	81.21	4.00	-3.07	2.34E-03	3.15E-02
cg06974930		1	Intergenic	S_Shore	41.30	4.93	44.36	5.47	-3.06	1.36E-03	2.14E-02
cg07479786	ANXA9	1	3'UTR	Open sea	70.00	3.64	73.06	3.15	-3.06	1.31E-03	2.09E-02
cg06545166	ETNK2	1	1stExon	Island	10.17	3.22	13.23	4.07	-3.06	1.97E-04	4.88E-03

cg27104448		11	Intergenic	Open sea	67.34	5.53	70.41	4.79	-3.06	3.42E-03	4.10E-02
cg12779301	BAIAP2;BAIAP2;BAIAP2;BAIAP2	17	Body;Body;Body;Body	Open sea	58.90	5.31	61.97	5.57	-3.06	7.94E-04	1.44E-02
cg22637867	ZNF709	19	5'UTR	Island	24.93	4.95	27.99	5.29	-3.06	2.68E-03	3.46E-02
cg00063535	TPCN1;TPCN1	12	Body;Body	Open sea	84.18	4.07	87.25	3.37	-3.06	2.33E-04	5.59E-03
cg25964019	DNAH11	7	Body	Open sea	84.73	2.81	87.79	2.13	-3.06	2.30E-07	1.11E-05
cg06842913	TRIM50;FKBP6	7	5'UTR;TSS1500	N_Shore	41.74	3.92	44.80	4.77	-3.06	3.93E-03	4.51E-02
cg08881610		6	Intergenic	N_Shelf	57.09	6.07	60.16	6.27	-3.06	3.83E-03	4.43E-02
cg26372517	TFAP2E;TFAP2E	1	5'UTR;1stExon	Island	25.78	3.98	28.84	5.07	-3.06	6.41E-04	1.23E-02
cg13101705	NMNAT2;NMNAT2	1	Body;Body	Open sea	55.35	3.41	58.42	3.15	-3.06	2.42E-04	5.75E-03
cg04968806	SLC6A5	11	Body	S_Shelf	32.05	3.41	35.11	3.03	-3.06	4.30E-05	1.36E-03
cg17265115	PCGF3	4	Body	N_Shelf	78.35	4.31	81.41	4.82	-3.06	2.55E-03	3.35E-02
cg22412649	SPATA13;MIR2276;SPATA13	13	5'UTR;Body;5'UTR	S_Shore	57.07	4.61	60.14	4.37	-3.06	2.96E-03	3.71E-02
cg07485775	NEURL1B	5	Body	Island	83.73	4.67	86.80	3.33	-3.06	1.08E-03	1.81E-02
cg24541835	DHRS3	1	Body	Open sea	25.15	3.45	28.22	4.56	-3.06	1.25E-03	2.02E-02
cg13716167		5	Intergenic	Open sea	75.80	4.55	78.86	3.71	-3.06	1.97E-03	2.80E-02
cg00686477	TNXB	6	Body	Open sea	74.56	4.95	77.62	4.37	-3.06	1.74E-04	4.42E-03
cg18134731	RSBN1L	7	Body	Open sea	82.24	5.39	85.30	4.48	-3.06	2.70E-03	3.48E-02
cg24924958	NMBR	6	TSS200	Island	26.80	4.11	29.86	4.28	-3.06	2.89E-04	6.60E-03
cg25974380		14	Intergenic	Open sea	38.81	5.05	41.87	5.07	-3.06	1.21E-04	3.28E-03
cg09686497	VPS13D;SNORA59A;VPS13D;SNORA5	1	Body;TSS200;Body;TSS200	Open sea	80.50	4.04	83.57	3.12	-3.06	8.71E-04	1.54E-02
cg05212464		5	Intergenic	S_Shore	41.78	4.33	44.85	5.36	-3.06	6.22E-05	1.88E-03
cg18033335	YIPF2	19	Body	S_Shelf	72.18	4.41	75.24	3.29	-3.06	1.89E-03	2.72E-02
cg26925980		10	Intergenic	S_Shelf	39.23	5.52	42.29	6.14	-3.06	1.76E-03	2.58E-02
cg22029396		12	Intergenic	Open sea	39.39	4.80	42.45	4.24	-3.06	3.34E-03	4.03E-02
cg20745684	WIPF3	7	Body	N_Shore	40.05	5.43	43.12	4.95	-3.06	2.18E-03	3.00E-02
cg00703885	LIMS2	2	TSS1500	Open sea	90.11	4.97	93.18	2.99	-3.06	6.23E-04	1.20E-02
cg04250451	RUNX3;RUNX3	1	TSS1500;Body	Island	35.69	3.48	38.75	3.64	-3.06	1.59E-03	2.40E-02
cg10179949	SLAMF8	1	TSS1500	Open sea	32.47	3.86	35.53	4.22	-3.06	7.60E-05	2.22E-03
cg06875179	SKI	1	Body	Island	70.03	3.42	73.09	3.85	-3.06	1.05E-03	1.77E-02
cg20706495		1	Intergenic	Open sea	14.34	5.95	17.40	7.85	-3.06	9.56E-04	1.66E-02
cg21949830	SLC43A2	17	Body	Island	37.60	3.25	40.66	4.41	-3.06	2.17E-03	2.99E-02
cg00030604		1	Intergenic	Open sea	69.37	4.54	72.43	4.61	-3.06	1.92E-04	4.79E-03
cg24123518	ISLR2;LOC283731;ISLR2;ISLR2;ISLR2	15	5'UTR;TSS1500;TSS200;TSS200;5'UTR	Island	12.42	2.13	15.48	2.93	-3.06	3.84E-07	1.82E-05
cg05297956		14	Intergenic	Open sea	84.65	5.72	87.71	2.20	-3.06	3.51E-03	4.18E-02
cg18530324	KIAA0427;KIAA0427	18	TSS1500;TSS1500	N_Shore	34.64	3.99	37.70	4.02	-3.06	1.97E-03	2.80E-02
cg24092907	ARL4A;ARL4A;ARL4A	7	3'UTR;3'UTR;3'UTR	S_Shelf	80.68	5.75	83.74	4.60	-3.06	1.03E-03	1.75E-02
cg04638265	FAM107B	10	Body	Open sea	61.20	4.64	64.26	3.67	-3.06	2.25E-04	5.43E-03
cg19459600	C21orf84	21	TSS1500	Open sea	74.24	6.50	77.30	5.24	-3.06	1.75E-03	2.58E-02
cg00778084		1	Intergenic	N_Shelf	72.19	5.46	75.25	4.10	-3.06	3.70E-03	4.33E-02
cg09643136		2	Intergenic	Island	28.98	3.94	32.04	4.37	-3.06	3.58E-03	4.23E-02
cg19056772	FGF11	17	TSS1500	N_Shore	56.26	4.58	59.32	4.26	-3.06	2.91E-03	3.67E-02
cg12384802	MAPK8IP3;MAPK8IP3	16	Body;Body	Island	74.20	4.98	77.26	3.70	-3.06	4.12E-03	4.66E-02
cg01449144		8	Intergenic	N_Shore	31.97	3.94	35.03	3.86	-3.06	3.36E-04	7.45E-03
cg08549806		16	Intergenic	N_Shelf	50.34	4.35	53.40	3.78	-3.06	1.73E-03	2.55E-02
cg08923271		1	Intergenic	Island	39.20	4.14	42.26	4.02	-3.06	6.63E-04	1.26E-02
cg03207121	MAFK	7	5'UTR	S_Shore	72.51	4.61	75.57	4.14	-3.06	1.60E-03	2.42E-02
cg18048309	KIAA1688	8	Body	Open sea	76.92	4.25	79.98	4.75	-3.06	9.06E-04	1.59E-02
cg20521963		3	Intergenic	Open sea	79.31	4.52	82.37	3.26	-3.06	1.93E-03	2.76E-02
cg03683207	NR2F6	19	3'UTR	N_Shelf	64.89	4.69	67.95	4.14	-3.06	7.97E-04	1.44E-02
cg12136274	PDIA3P	1	Body	Island	55.15	5.06	58.21	5.25	-3.06	2.94E-03	3.69E-02
cg13982220	NCOR2;NCOR2	12	Body;Body	Open sea	71.33	3.95	74.39	4.58	-3.06	3.99E-04	8.50E-03
cg23677352		4	Intergenic	Open sea	75.87	3.43	78.93	3.35	-3.06	8.21E-06	3.15E-04
cg15955277	MOGAT2	11	1stExon	Open sea	84.86	3.64	87.92	3.70	-3.06	2.27E-04	5.47E-03

cg27585641	RGNEF	5	Body	Open sea	29.05	3.65	32.11	3.96	-3.06	6.62E-04	1.26E-02
cg04929173	GAS2L1;GAS2L1;GAS2L1	22	Body;Body;Body	Island	86.82	4.86	89.88	3.86	-3.06	2.11E-03	2.93E-02
cg06674062	SGCE;PEG10;PEG10;PEG10;SGCE;PEG1	7	TSS1500;5'UTR;1stExon;1stExon;TSS1500;	Island	40.97	4.72	44.02	4.13	-3.06	9.69E-04	1.67E-02
cg19689322	KCNQ4;KCNQ4	1	Body;Body	N_Shore	37.50	4.05	40.56	4.77	-3.06	9.01E-04	1.59E-02
cg02337836	GSTM2;GSTM2	1	TSS200;TSS200	Island	21.93	6.41	24.99	5.32	-3.06	7.32E-04	1.36E-02
cg24500630		11	Intergenic	Open sea	50.21	4.94	53.26	5.12	-3.06	2.22E-03	3.04E-02
cg17165695	LOC100271715;LOC375196	2	Body;TSS1500	Island	53.49	4.33	56.55	4.42	-3.06	1.46E-03	2.26E-02
cg05225996	ZNF205;MGC3771;ZNF205;MGC3771	16	5'UTR;Body;5'UTR;Body	Open sea	12.50	3.23	15.55	3.97	-3.06	9.65E-04	1.67E-02
cg25606773	ENPP4	6	Body	Open sea	82.97	5.49	86.02	4.25	-3.06	2.03E-03	2.85E-02
cg19704558	LHX1	17	Body	Island	38.13	2.75	41.19	3.37	-3.06	3.83E-05	1.23E-03
cg09971754	ANKRD11	16	TSS1500	Island	3.80	0.62	6.85	1.08	-3.06	4.69E-30	4.04E-28
cg20038346	YJEFN3	19	Body	N_Shore	66.47	4.04	69.52	3.37	-3.06	3.29E-04	7.32E-03
cg14977875		5	Intergenic	Island	47.06	3.63	50.11	3.79	-3.06	5.09E-04	1.03E-02
cg23254650		2	Intergenic	S_Shelf	56.67	4.78	59.73	4.76	-3.06	1.64E-03	2.46E-02
cg13259290	CSF2	5	TSS1500	Open sea	71.80	3.69	74.86	4.14	-3.06	1.18E-04	3.21E-03
cg06064254	VAPA;VAPA	18	3'UTR;3'UTR	Open sea	86.33	4.28	89.38	3.04	-3.06	1.96E-03	2.78E-02
cg11732492	NXPH3	17	TSS1500	N_Shore	52.67	4.55	55.73	4.79	-3.05	4.23E-03	4.74E-02
cg07558275	VGLL3	3	TSS1500	S_Shore	30.77	5.99	33.83	6.71	-3.05	4.49E-03	4.94E-02
cg08171351	CECR6;CECR6	22	Body;1stExon	Island	21.92	2.97	24.98	3.98	-3.05	2.09E-05	7.26E-04
cg00469602		2	Intergenic	N_Shelf	21.07	3.22	24.12	3.42	-3.05	8.27E-05	2.39E-03
cg10508780		2	Intergenic	Open sea	66.31	4.62	69.37	3.83	-3.05	3.02E-04	6.84E-03
cg02677388	HIST1H3C	6	1stExon	Island	22.44	2.75	25.50	3.30	-3.05	6.40E-06	2.50E-04
cg15270235		3	Intergenic	S_Shore	71.22	4.52	74.27	4.90	-3.05	6.87E-04	1.29E-02
cg18515046	SLC6A19	5	3'UTR	N_Shelf	49.91	4.86	52.96	5.10	-3.05	2.92E-03	3.68E-02
cg19371652	OAS2;OAS2;OAS2	12	TSS1500;TSS1500;TSS1500	Open sea	52.64	5.32	55.69	5.40	-3.05	1.74E-03	2.57E-02
cg06817772	LIMA1;LIMA1	12	TSS1500;TSS1500	Open sea	78.63	4.52	81.68	4.44	-3.05	6.47E-04	1.24E-02
cg26266708	ISL2	15	Body	Island	58.18	4.78	61.23	4.64	-3.05	2.25E-03	3.07E-02
cg06719900	C11orf87;C11orf87	11	5'UTR;1stExon	N_Shore	13.84	3.29	16.89	4.14	-3.05	6.32E-04	1.21E-02
cg23064609		2	Intergenic	Island	15.99	2.86	19.05	3.42	-3.05	3.57E-05	1.16E-03
cg06902558	PFKP	10	Body	Open sea	78.98	3.50	82.04	3.29	-3.05	2.41E-04	5.73E-03
cg16720578		14	Intergenic	N_Shelf	58.65	5.36	61.70	6.07	-3.05	1.78E-03	2.61E-02
cg19028099	TRAPPC9;TRAPPC9	8	Body;Body	Open sea	69.44	4.51	72.49	3.97	-3.05	2.92E-03	3.68E-02
cg14583124	SNX25	4	Body	Open sea	74.93	3.72	77.99	3.08	-3.05	2.31E-04	5.54E-03
cg24139874	KIF14	1	Body	Open sea	80.17	4.32	83.23	3.66	-3.05	1.54E-03	2.35E-02
cg15419073	FGD5	3	Body	Open sea	35.86	3.71	38.91	3.52	-3.05	9.97E-05	2.79E-03
cg00116838	RTN2;FLJ40125;RTN2	19	TSS1500;1stExon;TSS1500	N_Shore	16.74	3.75	19.80	4.25	-3.05	2.04E-04	5.02E-03
cg10465839	IFT140;TMEM204	16	Body;TSS200	Island	54.98	4.81	58.04	4.85	-3.05	3.49E-03	4.16E-02
cg02474076	GCC1	7	Body	N_Shore	77.28	3.94	80.34	3.68	-3.05	5.55E-04	1.10E-02
cg03100814	BTNL2	6	Body	Open sea	75.07	4.32	78.12	3.89	-3.05	2.07E-03	2.89E-02
cg02182272	ATP4B	13	Body	Open sea	66.83	3.62	69.88	3.97	-3.05	4.20E-04	8.85E-03
cg19409163		6	Intergenic	Open sea	63.94	5.46	66.99	5.25	-3.05	3.39E-03	4.07E-02
cg24210742		15	Intergenic	Open sea	68.68	5.14	71.73	4.39	-3.05	3.02E-03	3.76E-02
cg14809932	UBD	6	Body	S_Shelf	65.23	5.75	68.29	5.04	-3.05	3.49E-03	4.16E-02
cg13538006	NINJ1	9	TSS1500	S_Shore	29.35	3.41	32.40	3.58	-3.05	6.02E-05	1.82E-03
cg13997668	SPOCK2;SPOCK2	10	Body;Body	Island	23.56	3.86	26.61	3.93	-3.05	1.60E-05	5.73E-04
cg13371839	ZNF98	19	TSS200	Open sea	20.70	4.34	23.75	4.24	-3.05	3.09E-03	3.82E-02
cg19286672	SGCA;SGCA	17	TSS1500;TSS1500	S_Shelf	49.49	3.95	52.54	4.19	-3.05	3.05E-03	3.79E-02
cg07266046		2	Intergenic	Open sea	68.53	5.39	71.58	5.69	-3.05	2.27E-04	5.47E-03
cg25616514		3	Intergenic	Open sea	50.94	4.38	53.99	4.63	-3.05	2.71E-05	9.14E-04
cg27542224	WDR27	6	Body	Open sea	84.32	4.44	87.37	2.63	-3.05	6.06E-04	1.17E-02
cg21176127	C14orf68	14	Body	Island	66.57	4.67	69.62	4.19	-3.05	3.10E-03	3.83E-02
cg19350360		15	Intergenic	Island	21.08	4.85	24.13	4.90	-3.05	1.57E-03	2.38E-02
cg00887511	C1orf183;C1orf183	1	Body;Body	Island	9.54	3.24	12.59	3.51	-3.05	7.44E-06	2.88E-04

cg12865381		10	Intergenic	Island	10.66	3.34	13.71	4.46	-3.05	6.18E-04	1.19E-02
cg00005861		10	Intergenic	S_Shelf	81.59	4.44	84.64	3.52	-3.05	3.35E-03	4.04E-02
cg09318731		19	Intergenic	N_Shelf	81.85	4.33	84.90	3.61	-3.05	5.78E-04	1.13E-02
cg20187309		6	Intergenic	N_Shelf	77.32	4.18	80.37	4.35	-3.05	7.54E-04	1.39E-02
cg12383621	RPA3	7	5'UTR	Open sea	80.58	4.82	83.63	4.03	-3.05	8.34E-04	1.49E-02
cg21385748	LIMS1	2	TSS1500	Open sea	75.31	4.36	78.36	2.70	-3.05	1.77E-04	4.48E-03
cg05703230		4	Intergenic	Open sea	63.74	4.26	66.79	3.76	-3.05	2.73E-04	6.32E-03
cg06966613		16	Intergenic	Open sea	80.84	5.70	83.89	3.63	-3.05	4.11E-03	4.65E-02
cg02181036	ZNRF2;MIR550-1	7	Body;TSS1500	S_Shelf	71.57	4.03	74.62	3.09	-3.05	1.34E-03	2.13E-02
cg16830930	SOX17	8	Body	Island	40.44	3.15	43.49	3.95	-3.05	1.21E-04	3.28E-03
cg21845457		8	Intergenic	N_Shore	36.36	4.37	39.41	4.66	-3.05	2.08E-04	5.09E-03
cg06933908	WDR60	7	Body	S_Shelf	79.56	3.69	82.61	3.36	-3.05	2.80E-04	6.44E-03
cg16258657	RBMS3;RBMS3;RBMS3	3	Body;Body;Body	Open sea	53.75	5.87	56.80	5.50	-3.05	3.94E-03	4.52E-02
cg13748755	RFPL2;RFPL2;RFPL2;RFPL2	22	Body;Body;Body;Body	Open sea	69.29	4.08	72.34	4.04	-3.05	3.54E-03	4.20E-02
cg22164009		6	Intergenic	Open sea	53.68	4.54	56.72	4.26	-3.05	2.22E-04	5.38E-03
cg04298857	TRIO	5	Body	Open sea	68.29	3.80	71.33	3.96	-3.05	3.06E-04	6.90E-03
cg14424538		20	Intergenic	Island	28.81	4.85	31.85	5.42	-3.05	1.55E-03	2.36E-02
cg19279342	CFD	19	Body	Island	15.94	3.72	18.98	4.61	-3.05	1.96E-03	2.79E-02
cg13801510		6	Intergenic	Island	67.03	3.12	70.08	3.20	-3.05	9.08E-05	2.58E-03
cg22626866		19	Intergenic	N_Shelf	76.69	5.37	79.74	4.97	-3.05	2.58E-03	3.38E-02
cg21002264	C17orf59;C17orf59	17	3'UTR;1stExon	N_Shore	71.43	3.78	74.47	2.80	-3.05	3.10E-04	6.98E-03
cg26967842	PTPRN2;PTPRN2;PTPRN2	7	Body;Body;Body	Island	43.47	3.71	46.52	4.49	-3.05	3.09E-03	3.82E-02
cg21410283	PDLIM7;PDLIM7;PDLIM7	5	Body;Body;Body	Island	31.00	3.55	34.04	4.84	-3.05	1.47E-03	2.27E-02
cg05885668		19	Intergenic	N_Shore	34.14	3.57	37.19	3.88	-3.05	3.42E-03	4.11E-02
cg01504030	BTBD9;BTBD9;BTBD9	6	3'UTR;3'UTR;3'UTR	Open sea	86.06	4.12	89.10	3.09	-3.05	1.04E-04	2.90E-03
cg23840338	MYBPC1;MYBPC1;MYBPC1;MYBPC1	12	3'UTR;3'UTR;3'UTR;3'UTR	Open sea	83.34	5.52	86.38	5.97	-3.05	1.27E-03	2.05E-02
cg27186851	LFNG;LFNG;LFNG;LFNG	7	Body;Body;Body;Body	S_Shore	81.28	3.53	84.32	3.68	-3.05	1.07E-04	2.97E-03
cg14724492	FGFRL1;FGFRL1;FGFRL1	4	Body;Body;Body	N_Shelf	65.27	3.99	68.31	3.58	-3.05	1.31E-03	2.09E-02
cg20709370	AMBRA1	11	Body	Open sea	81.60	4.29	84.64	3.55	-3.05	1.40E-03	2.20E-02
cg13551841	EHMT1	9	Body	S_Shelf	67.75	4.70	70.80	4.43	-3.05	1.78E-03	2.61E-02
cg22001807	TUBB	6	Body	S_Shore	38.05	5.06	41.09	4.55	-3.05	1.79E-03	2.62E-02
cg00419702	BAIAP2L2	22	Body	Island	9.72	3.58	12.77	4.28	-3.05	1.76E-03	2.58E-02
cg02197005		4	Intergenic	Open sea	78.49	6.16	81.54	4.69	-3.05	4.21E-03	4.72E-02
cg05832070		7	Intergenic	S_Shore	77.63	4.00	80.67	3.53	-3.05	9.73E-04	1.68E-02
cg23471067	BNIP3	10	Body	Island	12.58	2.95	15.62	4.22	-3.05	1.25E-03	2.02E-02
cg14839501	TUBGCP5;TUBGCP5	15	Body;Body	S_Shelf	67.93	4.97	70.97	4.01	-3.05	4.07E-03	4.61E-02
cg04026169		4	Intergenic	N_Shore	21.99	2.89	25.04	3.45	-3.04	7.49E-05	2.19E-03
cg04608829	CYP4F12	19	TSS200	Open sea	23.40	3.56	26.45	4.75	-3.04	2.73E-03	3.51E-02
cg11835978	SEMA5A	5	3'UTR	Open sea	83.99	5.08	87.03	4.52	-3.04	2.45E-03	3.26E-02
cg04714939	ZMIZ1	10	5'UTR	Open sea	32.50	4.16	35.54	4.50	-3.04	3.63E-03	4.27E-02
cg04057455	INS-IGF2;IGF2;INS-IGF2	11	Body;TSS1500;Body	Open sea	56.72	4.11	59.76	4.39	-3.04	1.94E-03	2.77E-02
cg08862717	TBC1D8	2	Body	N_Shelf	74.45	3.70	77.50	2.92	-3.04	1.70E-04	4.34E-03
cg23629393	MDGA2	14	Body	Open sea	48.71	4.15	51.75	3.61	-3.04	9.06E-05	2.58E-03
cg18455249	CEP135	4	TSS1500	N_Shore	34.58	6.00	37.62	4.29	-3.04	2.40E-03	3.22E-02
cg20719439	FHL5;FHL5	6	TSS1500;TSS1500	Open sea	62.11	5.08	65.16	5.29	-3.04	7.11E-04	1.33E-02
cg18254183		2	Intergenic	Open sea	46.37	4.65	49.42	5.26	-3.04	2.69E-03	3.47E-02
cg11145632	AP1B1;AP1B1;AP1B1	22	5'UTR;5'UTR;5'UTR	N_Shelf	17.99	3.74	21.03	3.97	-3.04	9.49E-04	1.65E-02
cg19737037	TNFRSF13C	22	1stExon	Island	23.84	5.64	26.88	5.28	-3.04	2.94E-04	6.69E-03
cg15922174	CRB2	9	Body	Island	10.26	4.25	13.30	4.70	-3.04	2.52E-03	3.32E-02
cg09942210	NCOR2;NCOR2	12	Body;Body	Open sea	10.44	2.63	13.48	2.67	-3.04	4.67E-07	2.19E-05
cg05194316	FARP1;FARP1	13	Body;Body	Open sea	70.33	4.19	73.37	3.55	-3.04	1.81E-03	2.63E-02
cg21653149	ANKFY1;ANKFY1	17	Body;Body	Open sea	81.04	4.68	84.09	3.63	-3.04	2.32E-03	3.14E-02
cg15398976	TRPC4;TRPC4;TRPC4;TRPC4;TRPC4;TR	13	TSS1500;TSS1500;TSS1500;TSS1500;TSS15	S_Shore	25.15	3.89	28.19	4.47	-3.04	1.55E-03	2.36E-02

cg15188252		5	Intergenic	Island	30.86	3.84	33.90	4.08	-3.04	2.81E-03	3.58E-02
cg24120960	STAG1	3	3'UTR	Open sea	81.20	4.57	84.25	3.36	-3.04	4.31E-04	9.04E-03
cg11445155		5	Intergenic	Open sea	78.36	4.36	81.41	3.23	-3.04	9.64E-04	1.67E-02
cg26779330	TOLLIP	11	3'UTR	N_Shore	81.62	2.82	84.66	2.42	-3.04	7.32E-07	3.35E-05
cg01465177		3	Intergenic	Open sea	16.07	4.05	19.11	4.14	-3.04	3.27E-03	3.97E-02
cg00491404	EPS8L3;EPS8L3;EPS8L3	1	TSS200;TSS200;TSS200	Open sea	43.72	4.35	46.77	4.63	-3.04	3.24E-04	7.23E-03
cg18766755	SPRR2A	1	5'UTR	Open sea	60.62	5.74	63.66	3.69	-3.04	3.61E-03	4.26E-02
cg04265492	BAI2	1	Body	N_Shore	63.21	7.61	66.25	6.40	-3.04	2.94E-03	3.69E-02
cg18813545	SRGAP3;SRGAP3	3	Body;Body	Open sea	54.63	5.19	57.67	5.63	-3.04	2.51E-03	3.32E-02
cg00772091	BUB3;BUB3	10	Body;Body	S_Shore	28.10	4.11	31.14	3.92	-3.04	1.01E-03	1.72E-02
cg14176966	NRIP2	12	TSS200	Open sea	18.81	4.62	21.85	5.74	-3.04	1.57E-03	2.38E-02
cg15543523	ESR1;ESR1;ESR1;ESR1	6	TSS1500;5'UTR;TSS1500;5'UTR	N_Shore	85.48	3.94	88.52	3.60	-3.04	8.94E-05	2.55E-03
cg14158968	GALNTL4	11	Body	Open sea	70.62	3.90	73.66	4.74	-3.04	4.23E-04	8.90E-03
cg00985983	SF3A1;SF3A1	22	3'UTR;3'UTR	Open sea	68.38	3.94	71.42	3.20	-3.04	2.53E-04	5.96E-03
cg21959757		5	Intergenic	Open sea	78.23	4.18	81.27	3.63	-3.04	3.71E-04	8.04E-03
cg07468062	KIAA1257	3	Body	Open sea	83.37	4.07	86.41	4.47	-3.04	9.30E-04	1.62E-02
cg10057065	RUSC1;C1orf104;RUSC1;RUSC1	1	Body;5'UTR;Body;TSS1500	N_Shore	14.57	2.79	17.61	4.52	-3.04	2.33E-03	3.14E-02
cg04808506	OTX1	2	Body	N_Shore	62.57	4.39	65.61	5.21	-3.04	2.51E-03	3.31E-02
cg13961400	BAT5	6	Body	Open sea	54.56	3.92	57.60	4.29	-3.04	3.64E-04	7.92E-03
cg10982500	FBXL17	5	Body	Open sea	87.88	2.98	90.92	2.74	-3.04	7.96E-07	3.62E-05
cg22392038	PAX6;PAX6;PAX6	11	Body;Body;Body	S_Shore	15.99	2.24	19.03	2.10	-3.04	1.75E-08	9.17E-07
cg18131626		6	Intergenic	Island	49.73	3.60	52.77	3.70	-3.04	3.61E-04	7.87E-03
cg13720362	NR2E1	6	Body	S_Shore	36.61	4.31	39.65	5.00	-3.04	2.21E-03	3.03E-02
cg06824181		7	Intergenic	S_Shore	24.00	3.87	27.04	4.62	-3.04	3.22E-04	7.20E-03
cg03943115	SPRR2D	1	TSS1500	Open sea	24.63	4.16	27.67	4.65	-3.04	2.95E-04	6.72E-03
cg14504697	MTX2;MTX2	2	Body;Body	Open sea	87.02	4.42	90.05	3.37	-3.04	9.56E-05	2.70E-03
cg13570658	CENPP;OGN;OGN	9	Body;3'UTR;3'UTR	Open sea	84.67	4.79	87.70	4.37	-3.04	1.41E-03	2.20E-02
cg22837714		2	Intergenic	Open sea	44.48	4.01	47.52	4.05	-3.04	4.85E-04	9.88E-03
cg03395546	ADCK4;ADCK4;ITPKC	19	1stExon;5'UTR;TSS1500	Island	35.67	5.33	38.70	4.26	-3.04	3.75E-03	4.37E-02
cg26944725	LYPD6B	2	5'UTR	Open sea	67.70	4.46	70.74	4.02	-3.04	1.36E-03	2.15E-02
cg18428134	C9orf4	9	TSS200	S_Shore	55.78	3.95	58.81	3.32	-3.04	1.63E-03	2.44E-02
cg26362491		1	Intergenic	Open sea	83.20	5.47	86.24	5.81	-3.04	2.92E-03	3.67E-02
cg17353412	HOXA2	7	1stExon	N_Shore	31.42	4.01	34.46	4.73	-3.04	2.82E-03	3.59E-02
cg25044651	LVRN	5	1stExon	Island	17.85	2.97	20.88	4.56	-3.04	3.72E-04	8.06E-03
cg22273168	MARCH10;MARCH10	17	Body;Body	Open sea	78.52	4.71	81.56	4.01	-3.04	2.56E-03	3.36E-02
cg11561909		7	Intergenic	S_Shore	61.69	3.98	64.72	3.58	-3.04	1.22E-03	1.99E-02
cg08978092		5	Intergenic	Open sea	75.56	5.59	78.60	4.68	-3.04	3.63E-03	4.27E-02
cg10720699	GLTSCR1	19	Body	Island	65.89	4.06	68.93	2.91	-3.04	6.59E-04	1.25E-02
cg13728039	ZNF585A;ZNF585A	19	5'UTR;5'UTR	Open sea	83.24	4.92	86.28	4.93	-3.04	1.22E-03	1.98E-02
cg08368875		10	Intergenic	Open sea	49.41	4.10	52.45	4.66	-3.04	2.42E-03	3.23E-02
cg04018023		7	Intergenic	Open sea	54.89	3.22	57.92	4.34	-3.04	7.77E-04	1.42E-02
cg16697004		7	Intergenic	Open sea	65.25	3.02	68.29	3.44	-3.04	3.51E-05	1.14E-03
cg14628343		8	Intergenic	Open sea	38.81	3.91	41.84	3.17	-3.04	1.34E-04	3.56E-03
cg05922437		15	Intergenic	N_Shelf	66.04	4.46	69.08	3.36	-3.04	9.67E-04	1.67E-02
cg02045294	MAMSTR	19	TSS1500	N_Shore	52.65	4.30	55.69	4.04	-3.04	9.14E-04	1.60E-02
cg05887421	ATPBD4;ATPBD4	15	TSS1500;TSS1500	S_Shore	25.54	4.34	28.57	4.34	-3.04	4.51E-03	4.95E-02
cg09351082	SORBS1;SORBS1	10	5'UTR;5'UTR	N_Shore	65.98	5.24	69.02	4.42	-3.04	3.90E-03	4.49E-02
cg25987102	APOA1	11	TSS1500	S_Shelf	57.05	4.14	60.08	3.81	-3.04	1.88E-04	4.70E-03
cg23855989	AQP5	12	1stExon	Island	43.11	3.77	46.14	2.92	-3.04	9.08E-05	2.58E-03
cg12673103	PITX1	5	Body	Island	21.89	3.93	24.92	3.59	-3.04	5.80E-04	1.14E-02
cg16155588	CUL4A;PCID2;PCID2;PCID2	13	TSS1500;Body;Body;Body	N_Shore	66.70	5.06	69.74	2.90	-3.04	2.01E-04	4.97E-03
cg20000718		4	Intergenic	Island	22.50	4.07	25.54	4.63	-3.04	5.22E-05	1.61E-03
cg14129855		12	Intergenic	Open sea	75.36	4.48	78.40	3.68	-3.04	1.15E-04	3.14E-03

cg22249529	KLHDC7B	22	TSS1500	Island	57.67	5.95	60.71	4.84	-3.04	3.65E-03	4.28E-02
cg15102655		6	Intergenic	Open sea	50.82	3.17	53.86	3.73	-3.04	1.96E-04	4.86E-03
cg05036656		4	Intergenic	Island	22.50	4.32	25.53	4.04	-3.04	3.01E-04	6.82E-03
cg03597491	ZBTB16;ZBTB16	11	Body;Body	Open sea	55.36	3.10	58.39	3.14	-3.03	1.13E-05	4.19E-04
cg01556466	ADCYAP1R1	7	Body	Open sea	65.42	4.77	68.46	4.56	-3.03	5.60E-04	1.10E-02
cg26191447		2	Intergenic	Open sea	48.90	5.37	51.94	5.38	-3.03	4.12E-03	4.65E-02
cg01770355	VSX2	14	Body	Island	19.26	3.12	22.30	3.83	-3.03	3.96E-06	1.61E-04
cg02667022	JAKMIP2;JAKMIP2	5	5'UTR;1stExon	Open sea	42.47	6.25	45.50	7.19	-3.03	3.54E-04	7.75E-03
cg04509100	IL34	16	TSS200	Open sea	73.89	4.31	76.93	3.44	-3.03	2.34E-04	5.59E-03
cg18016254	PARP15;PARP15	3	5'UTR;1stExon	N_Shore	31.17	5.89	34.20	6.05	-3.03	1.60E-03	2.41E-02
cg20776317	SNAPC4	9	1stExon	N_Shore	80.47	4.34	83.51	3.14	-3.03	7.23E-04	1.34E-02
cg05438378	SMAD3	15	Body	Open sea	45.21	5.47	48.25	6.32	-3.03	7.47E-04	1.38E-02
cg15093715		3	Intergenic	Open sea	81.45	3.83	84.48	4.02	-3.03	6.29E-05	1.89E-03
cg24518873		1	Intergenic	S_Shelf	76.62	4.39	79.66	3.17	-3.03	3.41E-04	7.52E-03
cg24201173		15	Intergenic	Open sea	76.75	4.33	79.79	4.06	-3.03	8.42E-04	1.51E-02
cg25458175	ZBTB45	19	Body	Island	10.32	3.77	13.35	4.23	-3.03	1.98E-03	2.81E-02
cg27250180		21	Intergenic	Island	79.90	4.78	82.93	5.05	-3.03	2.51E-03	3.32E-02
cg11398452	VAX1;VAX1	10	Body;Body	Island	12.81	3.71	15.84	4.66	-3.03	5.18E-04	1.04E-02
cg13787758	ZNF347	19	5'UTR	N_Shore	82.73	4.54	85.76	3.75	-3.03	3.45E-03	4.12E-02
cg00434535		7	Intergenic	Open sea	65.87	5.27	68.90	5.70	-3.03	4.50E-03	4.95E-02
cg05570237	CYYR1	21	Body	Open sea	53.89	4.72	56.92	5.12	-3.03	2.94E-03	3.69E-02
cg22402224	ORAI2;ORAI2	7	Body;Body	Open sea	72.62	5.58	75.65	5.29	-3.03	1.85E-03	2.68E-02
cg16132151	MINPP1	10	TSS1500	N_Shore	62.08	5.52	65.12	5.23	-3.03	3.16E-03	3.88E-02
cg17655320	ZNF346	5	Body	Open sea	72.20	4.38	75.23	4.48	-3.03	3.33E-04	7.40E-03
cg14066993	LOC100130872;LOC100130872-SPON2	4	TSS1500;TSS1500	Island	73.85	5.41	76.89	5.37	-3.03	1.73E-03	2.55E-02
cg14909424	EIF3B;EIF3B	7	Body;Body	N_Shelf	71.18	4.49	74.21	4.61	-3.03	2.32E-03	3.14E-02
cg16452224	CPNE9	3	TSS200	N_Shore	13.99	3.20	17.02	3.98	-3.03	7.62E-05	2.23E-03
cg13799941	DLX6AS	7	Body	S_Shore	34.40	3.42	37.43	3.28	-3.03	2.39E-04	5.70E-03
cg25022709		1	Intergenic	S_Shore	84.02	5.11	87.05	4.98	-3.03	1.86E-03	2.69E-02
cg14987431		10	Intergenic	Island	27.12	3.84	30.15	4.06	-3.03	3.79E-03	4.40E-02
cg16669607		2	Intergenic	Open sea	62.46	4.36	65.49	4.86	-3.03	3.92E-03	4.50E-02
cg23058239	GTSF1	12	TSS1500	Open sea	30.08	4.64	33.11	4.38	-3.03	7.78E-04	1.42E-02
cg18345539		19	Intergenic	N_Shelf	52.25	3.35	55.28	4.74	-3.03	1.19E-03	1.95E-02
cg04746199	PAX8;PAX8;PAX8;PAX8;PAX8;LOC4408	2	Body;Body;Body;Body;Body;Body	N_Shore	8.05	3.52	11.08	5.22	-3.03	3.53E-03	4.19E-02
cg09887220		13	Intergenic	N_Shore	30.19	4.46	33.23	4.60	-3.03	3.73E-03	4.35E-02
cg03541338	RGS10	10	Body	N_Shore	21.25	3.65	24.28	4.04	-3.03	2.71E-04	6.29E-03
cg18483404	CUZD1	10	3'UTR	Open sea	77.12	4.72	80.16	4.35	-3.03	1.25E-03	2.02E-02
cg01983248	C5orf28	5	5'UTR	N_Shore	80.83	5.27	83.87	4.62	-3.03	2.88E-04	6.59E-03
cg22415594	ZC3H13;ZC3H13	13	5'UTR;1stExon	Island	8.48	1.83	11.51	4.44	-3.03	8.12E-06	3.12E-04
cg17403397		2	Intergenic	Island	31.25	2.85	34.28	4.55	-3.03	4.31E-04	9.04E-03
cg11159417		5	Intergenic	Open sea	76.83	3.92	79.86	3.05	-3.03	3.88E-04	8.32E-03
cg27119412	VCL;VCL	10	Body;Body	Open sea	77.87	4.20	80.90	3.29	-3.03	5.33E-04	1.06E-02
cg16125371	ALX4	11	TSS1500	Island	26.41	2.85	29.44	3.45	-3.03	2.13E-04	5.19E-03
cg13832135	FAM189A1	15	Body	Open sea	81.92	4.47	84.95	4.03	-3.03	1.02E-03	1.74E-02
cg20845305	SLC7A1	13	Body	Open sea	42.73	3.35	45.76	3.12	-3.03	7.60E-05	2.22E-03
cg04963553		16	Intergenic	N_Shelf	47.92	4.08	50.94	5.07	-3.03	1.00E-03	1.72E-02
cg11720686	SLC8A1;SLC8A1;SLC8A1;SLC8A1	2	Body;Body;Body;Body	Open sea	73.97	5.17	77.00	4.97	-3.03	2.48E-03	3.28E-02
cg16317318	CDC42EP2	11	3'UTR	Open sea	52.82	3.39	55.85	3.20	-3.03	3.04E-05	1.01E-03
cg23984312	NUP210L;NUP210L	1	Body;Body	Open sea	84.95	4.59	87.98	3.67	-3.03	1.91E-03	2.74E-02
cg09175338	MYH4	17	5'UTR	Open sea	52.05	5.20	55.08	5.60	-3.03	9.27E-04	1.62E-02
cg20535723	KIF20B	10	TSS1500	N_Shore	81.92	3.65	84.95	2.93	-3.03	6.04E-04	1.17E-02
cg03761477		6	Intergenic	N_Shore	30.83	3.48	33.86	5.35	-3.03	4.50E-03	4.94E-02
cg13192180	NOL11	17	Body	S_Shelf	78.94	4.20	81.97	4.96	-3.03	8.29E-05	2.39E-03

cg14659606		2	Intergenic	Open sea	53.26	5.04	56.29	4.84	-3.03	2.80E-03	3.57E-02
cg02954562	IFFO1;IFFO1;IFFO1	12	Body;Body;Body	Island	50.76	5.62	53.79	5.30	-3.03	3.34E-03	4.04E-02
cg18645906	BST1;BST1	4	1stExon;5'UTR	N_Shore	26.71	4.72	29.74	6.66	-3.03	2.46E-03	3.27E-02
cg21704793		6	Intergenic	N_Shelf	43.91	3.69	46.94	3.21	-3.03	3.58E-04	7.81E-03
cg13321077	RESP18	2	Body	Island	16.25	3.10	19.28	3.72	-3.03	1.09E-04	3.01E-03
cg22345692	NPR3	5	Body	Island	14.62	4.61	17.64	6.01	-3.03	1.50E-03	2.30E-02
cg23483765	NIPAL4	5	TSS200	Island	27.07	5.27	30.10	4.80	-3.03	5.63E-04	1.11E-02
cg14628274	ACER2	9	Body	Open sea	36.93	3.88	39.96	3.69	-3.03	7.89E-04	1.43E-02
cg14781778	C22orf9;C22orf9	22	Body;TSS1500	Open sea	68.13	4.55	71.16	3.84	-3.03	3.39E-03	4.07E-02
cg08699646	TEPP;TEPP	16	Body;Body	Island	22.59	4.24	25.62	4.97	-3.03	1.03E-03	1.76E-02
cg06086785	DHX30;DHX30	3	Body;Body	S_Shore	76.52	4.80	79.55	3.77	-3.03	2.62E-03	3.41E-02
cg22775642	SCARB1;SCARB1	12	Body;Body	Open sea	57.30	4.39	60.33	3.88	-3.03	1.66E-03	2.47E-02
cg05355055	SPATA1	1	Body	Open sea	67.67	4.65	70.70	5.15	-3.03	3.08E-03	3.81E-02
cg02623400	ELAVL4;ELAVL4	1	1stExon;5'UTR	Island	22.09	3.64	25.12	3.24	-3.03	4.47E-05	1.41E-03
cg10319663	SLC22A25	11	1stExon	Open sea	84.86	3.40	87.88	3.10	-3.03	2.78E-05	9.33E-04
cg22705929	YPEL4	11	TSS1500	S_Shelf	60.17	5.99	63.19	4.82	-3.03	3.25E-03	3.96E-02
cg14784922		11	Intergenic	Open sea	68.06	4.53	71.09	4.94	-3.03	2.66E-03	3.45E-02
cg23194229	MAGI1;MAGI1;MAGI1	3	Body;Body;Body	Open sea	85.72	4.78	88.75	3.61	-3.03	2.09E-03	2.92E-02
cg03494017		5	Intergenic	N_Shore	52.97	4.16	56.00	5.47	-3.03	3.84E-03	4.44E-02
cg09476228	FAM133B;FAM133B	7	Body;5'UTR	N_Shelf	69.60	3.95	72.63	3.83	-3.03	2.65E-04	6.18E-03
cg21241560	RORA	15	Body	Open sea	44.08	4.24	47.11	4.38	-3.02	1.83E-03	2.66E-02
cg04645039	ARHGAP23	17	3'UTR	Island	44.04	4.26	47.07	3.92	-3.02	3.69E-03	4.31E-02
cg05218678		6	Intergenic	Open sea	50.07	5.73	53.10	4.17	-3.02	3.45E-03	4.13E-02
cg13149245		15	Intergenic	Open sea	23.81	3.48	26.84	4.05	-3.02	9.63E-04	1.67E-02
cg15559737	CRYBA4	22	TSS1500	Open sea	68.13	3.76	71.15	3.13	-3.02	7.14E-05	2.11E-03
cg22741626	GNAS;GNAS;GNAS;GNAS	20	3'UTR;3'UTR;TSS1500;Body	N_Shore	53.69	3.58	56.71	4.66	-3.02	2.12E-04	5.17E-03
cg10247061	LOC220115	13	Body	S_Shore	61.95	4.33	64.97	4.27	-3.02	9.15E-04	1.60E-02
cg03239178		12	Intergenic	N_Shore	7.91	1.62	10.93	2.18	-3.02	3.29E-12	1.98E-10
cg12566756		12	Intergenic	N_Shore	13.36	3.23	16.38	3.66	-3.02	2.02E-04	4.99E-03
cg02440744	SLC26A5;SLC26A5;SLC26A5;SLC26A5;S	7	TSS200;TSS200;TSS200;TSS200;TSS200	Island	28.47	3.65	31.49	5.47	-3.02	1.21E-03	1.97E-02
cg21634602	APC;APC;APC;APC;APC	5	1stExon;5'UTR;1stExon;5'UTR;5'UTR	Open sea	44.00	9.48	47.02	9.35	-3.02	3.54E-03	4.19E-02
cg04749507	PPT2;PRRT1;PPT2	6	TSS1500;TSS1500;TSS1500	N_Shore	54.89	3.63	57.91	4.04	-3.02	4.90E-04	9.97E-03
cg04159302	C5orf62	5	Body	S_Shelf	68.98	4.93	72.00	4.23	-3.02	8.54E-04	1.52E-02
cg08777421		7	Intergenic	N_Shore	21.98	4.69	25.01	4.68	-3.02	1.59E-03	2.40E-02
cg08237565		8	Intergenic	N_Shore	82.46	3.28	85.48	2.92	-3.02	6.69E-05	2.00E-03
cg22215392	IQSEC1;IQSEC1	3	Body;Body	N_Shelf	75.70	4.31	78.72	4.83	-3.02	9.54E-04	1.65E-02
cg04207549	RTN4RL1	17	Body	Island	73.45	4.55	76.47	4.32	-3.02	1.82E-03	2.65E-02
cg26925644	PHLDB1	11	TSS1500	N_Shore	54.40	4.75	57.42	5.20	-3.02	2.82E-03	3.59E-02
cg03742238	PHOX2B	4	Body	N_Shore	16.61	2.60	19.63	4.26	-3.02	2.05E-04	5.03E-03
cg15787389	ERC2	3	Body	Open sea	65.81	6.09	68.84	5.43	-3.02	1.19E-03	1.95E-02
cg21580416		2	Intergenic	Open sea	63.97	3.68	66.99	3.52	-3.02	7.42E-04	1.37E-02
cg22838050	MED16	19	Body	S_Shore	73.09	4.98	76.11	4.50	-3.02	1.30E-03	2.08E-02
cg03125427	CYBA	16	TSS200	Island	52.64	6.95	55.66	8.19	-3.02	4.03E-03	4.59E-02
cg08885657	FGR	1	5'UTR	N_Shelf	47.10	4.24	50.12	5.01	-3.02	2.12E-04	5.17E-03
cg16678099		17	Intergenic	N_Shore	49.42	5.89	52.44	6.04	-3.02	3.38E-04	7.47E-03
cg09854734	CMTM2	16	TSS200	Island	38.95	4.88	41.97	4.04	-3.02	2.08E-03	2.91E-02
cg26226988	MUC6	11	TSS1500	S_Shore	63.48	3.57	66.50	3.61	-3.02	2.51E-04	5.93E-03
cg21510189		5	Intergenic	Open sea	52.06	4.11	55.08	3.88	-3.02	1.82E-03	2.64E-02
cg13689006	FRMPD1	9	Body	Open sea	87.19	4.35	90.21	3.69	-3.02	1.32E-03	2.11E-02
cg25127315	ARMC2	6	TSS1500	N_Shore	15.02	4.22	18.05	4.41	-3.02	1.70E-03	2.52E-02
cg27545985		3	Intergenic	S_Shelf	75.84	4.01	78.86	3.95	-3.02	5.01E-04	1.01E-02
cg22544881	FLJ43663;FLJ43663	7	Body;Body	Open sea	59.84	4.99	62.86	4.64	-3.02	3.25E-03	3.96E-02
cg09499849	ACVR1;ACVR1	2	5'UTR;5'UTR	Open sea	11.57	2.72	14.59	3.96	-3.02	1.64E-04	4.22E-03

cg12557278		6	Intergenic	Open sea	84.25	4.34	87.27	4.47	-3.02	8.58E-05	2.46E-03
cg23690808	CASZ1;CASZ1	1	5'UTR;5'UTR	Open sea	75.34	4.31	78.36	5.15	-3.02	2.57E-04	6.03E-03
cg01782798	IL34	16	TSS200	Open sea	57.66	4.41	60.68	4.68	-3.02	3.77E-03	4.38E-02
cg07802625	SPOCD1	1	TSS200	Open sea	43.07	4.07	46.09	3.63	-3.02	3.42E-04	7.54E-03
cg04518808	PRKCG	19	TSS1500	N_Shore	15.89	2.78	18.91	5.14	-3.02	7.87E-04	1.43E-02
cg23299109	SLC26A4;LOC286002	7	TSS200;Body	N_Shore	37.10	3.62	40.12	4.22	-3.02	7.34E-04	1.36E-02
cg09052244	DLGAP2	8	5'UTR	Island	84.85	5.21	87.87	3.79	-3.02	1.69E-03	2.51E-02
cg15941449	DLX1;DLX1	2	TSS1500;TSS1500	Island	14.71	2.71	17.73	3.73	-3.02	2.64E-06	1.11E-04
cg14522034	POFUT2;POFUT2;LOC642852;POFUT2	21	Body;Body;TSS1500;Body	Island	52.69	3.42	55.71	3.12	-3.02	1.91E-04	4.77E-03
cg04286194		5	Intergenic	N_Shore	24.78	3.49	27.80	3.98	-3.02	5.91E-04	1.15E-02
cg23637354		6	Intergenic	Open sea	51.30	3.96	54.32	4.35	-3.02	4.11E-04	8.70E-03
cg00350642	VTCN1	1	Body	Open sea	47.72	3.01	50.74	2.80	-3.02	1.01E-05	3.79E-04
cg11235787	MIR195	17	Body	S_Shelf	66.42	4.84	69.43	3.50	-3.02	2.33E-03	3.15E-02
cg01911239	PCDHGA4;PCDHGA2;PCDHGA5;PCDH(	5	Body;Body;1stExon;Body;Body;Body;Body	Island	30.08	4.05	33.10	4.08	-3.02	2.79E-03	3.56E-02
cg09561125	NRCAM;NRCAM	7	TSS1500;TSS1500	S_Shore	33.07	4.65	36.09	4.11	-3.02	3.40E-03	4.09E-02
cg08662017	DKK3;DKK3;DKK3	11	TSS1500;TSS200;TSS1500	S_Shore	18.81	4.11	21.83	5.64	-3.02	3.12E-04	7.02E-03
cg09095892		10	Intergenic	Island	14.49	4.61	17.51	5.94	-3.02	2.26E-03	3.08E-02
cg00667048	MORN1	1	Body	N_Shore	46.09	3.64	49.11	4.17	-3.02	3.54E-03	4.20E-02
cg11476878	NMT2	10	Body	N_Shelf	65.05	3.44	68.06	4.25	-3.02	5.49E-04	1.09E-02
cg14102634		6	Intergenic	Open sea	12.47	4.44	15.49	5.59	-3.02	1.45E-03	2.25E-02
cg04831352		3	Intergenic	Open sea	77.54	3.96	80.56	3.36	-3.02	3.44E-04	7.57E-03
cg00787282	PLB1;PLB1	2	Body;Body	N_Shore	35.25	4.10	38.26	4.36	-3.02	9.17E-04	1.61E-02
cg10319331	TMEM132B	12	Body	Open sea	42.29	3.90	45.31	4.94	-3.02	3.83E-04	8.23E-03
cg07987843	TSPAN9;TSPAN9	12	5'UTR;5'UTR	Island	24.97	4.20	27.99	5.90	-3.02	1.82E-04	4.59E-03
cg18552939	NDN	15	TSS200	S_Shore	24.94	5.32	27.96	4.67	-3.02	6.36E-04	1.22E-02
cg01013685		1	Intergenic	S_Shelf	70.02	3.94	73.04	3.70	-3.02	6.20E-04	1.20E-02
cg00883019	ARHGEF3;ARHGEF3;ARHGEF3	3	Body;Body;Body	Open sea	87.60	4.81	90.62	3.29	-3.02	9.59E-04	1.66E-02
cg25348336	GEMIN6	2	TSS1500	Open sea	64.98	4.05	68.00	4.00	-3.02	3.99E-04	8.50E-03
cg10511988	VAX1	10	3'UTR	N_Shore	22.25	4.10	25.26	4.57	-3.02	1.91E-04	4.76E-03
cg27321913	DEF6	6	TSS1500	Open sea	46.96	4.41	49.97	5.09	-3.02	4.98E-04	1.01E-02
cg08013227	FAM166A	9	TSS200	Open sea	74.72	4.55	77.74	4.62	-3.02	4.15E-03	4.68E-02
cg24363848		1	Intergenic	Open sea	66.91	3.94	69.92	4.87	-3.02	7.75E-05	2.26E-03
cg01052276		2	Intergenic	N_Shore	71.66	5.75	74.68	4.01	-3.02	2.52E-03	3.32E-02
cg06925855		1	Intergenic	S_Shore	29.26	3.55	32.28	4.06	-3.02	2.14E-03	2.97E-02
cg01793445	RCSD1	1	Body	Open sea	54.68	3.61	57.69	3.77	-3.02	1.32E-04	3.51E-03
cg12964970		2	Intergenic	Open sea	78.88	3.91	81.89	4.01	-3.02	4.33E-03	4.82E-02
cg05356496	SLC26A4;LOC286002	7	TSS200;Body	N_Shore	28.15	3.71	31.17	5.03	-3.02	7.62E-04	1.40E-02
cg08996445	MTSS1L	16	Body	S_Shelf	80.23	4.32	83.25	3.79	-3.01	2.83E-03	3.60E-02
cg11972677	MPPED2	11	5'UTR	Island	25.38	3.26	28.39	4.38	-3.01	2.01E-04	4.95E-03
cg12640482	SNHG12;SNORD99	1	Body;TSS1500	N_Shelf	73.77	5.54	76.79	5.16	-3.01	1.84E-04	4.63E-03
cg23445461	PCDHGA4;PCDHGC3;PCDHGA12;PCDH	5	Body;Body;Body;Body;Body;Body;Body;Bo	Island	26.95	5.62	29.96	5.65	-3.01	2.92E-03	3.67E-02
cg06839854	HPCAL1;HPCAL1	2	5'UTR;5'UTR	Open sea	83.81	3.39	86.82	1.94	-3.01	3.29E-05	1.08E-03
cg13151361	PRKRA;MIR548N;PRKRA;PRKRA	2	Body;Body;Body;Body	Open sea	68.17	3.50	71.18	4.08	-3.01	6.03E-04	1.17E-02
cg08125726	FSCN1	7	Body	N_Shelf	67.39	4.53	70.40	5.48	-3.01	4.27E-03	4.77E-02
cg03668982	UBE2E1;UBE2E1	3	Body;Body	S_Shore	84.21	4.69	87.22	3.89	-3.01	1.64E-04	4.21E-03
cg06636541	BAHCC1	17	Body	Island	14.99	3.59	18.00	3.57	-3.01	3.01E-03	3.75E-02
cg11884030		7	Intergenic	Open sea	46.80	6.92	49.81	5.77	-3.01	2.61E-03	3.40E-02
cg13557402	NKD2	5	Body	S_Shelf	73.51	4.43	76.53	5.01	-3.01	2.49E-03	3.30E-02
cg12906748	C11orf16	11	TSS200	Open sea	75.91	3.51	78.92	3.72	-3.01	1.82E-04	4.59E-03
cg19425603	PCDH7	4	Body	Open sea	18.49	4.13	21.50	4.18	-3.01	1.82E-03	2.65E-02
cg15006175	KIFC2	8	Body	Island	43.70	4.92	46.71	6.37	-3.01	2.83E-03	3.60E-02
cg17542333	MRVI1;MRVI1;MRVI1;MRVI1;MRVI1	11	5'UTR;1stExon;5'UTR;5'UTR;5'UTR	Open sea	66.19	3.58	69.20	3.45	-3.01	1.32E-04	3.52E-03
cg13950362	DEFB123	20	TSS1500	Open sea	55.56	4.26	58.58	4.66	-3.01	3.97E-04	8.46E-03

cg20381985	HOXC4;HOXC5;HOXC4;HOXC6;HOXC6	12	5'UTR;Body;1stExon;1stExon;5'UTR	N_Shore	25.78	4.07	28.79	3.89	-3.01	6.04E-04	1.17E-02
cg22242614	SYNJ2	6	Body	S_Shore	58.95	6.81	61.97	7.43	-3.01	3.80E-03	4.41E-02
cg17253709	B3GNT2	2	5'UTR	Open sea	32.78	4.06	35.80	4.06	-3.01	3.09E-03	3.83E-02
cg11744021	MTHFD1L	6	Body	Open sea	77.69	4.66	80.71	4.05	-3.01	2.36E-03	3.17E-02
cg06007761	SLIT3	5	Body	Open sea	75.61	4.47	78.62	3.76	-3.01	1.94E-03	2.77E-02
cg02832697	TRIM2;TRIM2	4	Body;5'UTR	Open sea	36.71	4.11	39.72	4.23	-3.01	5.20E-05	1.61E-03
cg16326743	FGFR4;FGFR4;FGFR4	5	Body;Body;Body	S_Shore	81.23	3.73	84.24	3.35	-3.01	2.60E-04	6.10E-03
cg14405813	HIPK2;HIPK2	7	Body;Body	N_Shore	62.59	4.57	65.61	3.36	-3.01	3.30E-03	4.00E-02
cg11244758	GNAS;GNAS;GNAS;GNAS	20	3'UTR;3'UTR;TSS1500;Body	Island	42.65	3.00	45.67	3.25	-3.01	7.03E-05	2.08E-03
cg19160629	SIK3	11	Body	Open sea	66.24	4.72	69.25	3.83	-3.01	2.31E-03	3.13E-02
cg21962204	GPATCH2	1	3'UTR	Open sea	85.04	3.11	88.05	2.70	-3.01	2.26E-07	1.09E-05
cg09970590	MEIS2;MEIS2;MEIS2;MEIS2;MEIS2;ME	15	Body;Body;Body;Body;Body;Body;Body	Open sea	77.51	4.90	80.52	4.38	-3.01	2.78E-03	3.55E-02
cg09743437		5	Intergenic	Island	17.56	3.74	20.57	4.67	-3.01	4.92E-04	1.00E-02
cg27337176	C20orf166;MIR1-1	20	Body;TSS200	N_Shore	61.43	5.03	64.44	4.05	-3.01	2.35E-03	3.17E-02
cg04138361	COL11A2;COL11A2;COL11A2;COL11A2	6	TSS200;TSS200;TSS200;TSS200	N_Shore	14.66	3.18	17.67	3.99	-3.01	1.27E-05	4.67E-04
cg24260710	MIR33A;SREBF2	22	TSS1500;Body	Open sea	77.67	5.94	80.68	4.64	-3.01	1.46E-03	2.26E-02
cg02056325		11	Intergenic	Open sea	67.27	4.44	70.28	4.13	-3.01	3.55E-03	4.20E-02
cg19007731	RUNX1T1;RUNX1T1	8	5'UTR;1stExon	Open sea	27.73	3.03	30.74	4.37	-3.01	6.33E-04	1.21E-02
cg19924104	PEG10;PEG10;PEG10;SGCE;SGCE;SGCI	7	5'UTR;1stExon;1stExon;TSS200;TSS200;TS:	Island	33.49	2.50	36.50	2.62	-3.01	3.86E-06	1.58E-04
cg10276301	LOC100133545	11	TSS1500	N_Shore	88.00	3.75	91.01	2.00	-3.01	4.85E-05	1.51E-03
cg04339462	CCDC85C	14	Body	Open sea	79.00	5.92	82.01	5.17	-3.01	1.57E-04	4.06E-03
cg02670903	MIR548H4;NOX5	15	Body;Body	Island	82.12	5.22	85.13	4.31	-3.01	2.58E-03	3.37E-02
cg22937649	NTN1	17	Body	Island	36.28	3.84	39.29	4.09	-3.01	2.72E-04	6.30E-03
cg13521941	ENTPD8;ENTPD8	9	Body;Body	Island	61.69	7.43	64.70	8.17	-3.01	3.99E-04	8.51E-03
cg04114665	BCAR1;BCAR1;BCAR1;BCAR1;BCAR1	16	Body;5'UTR;Body;5'UTR;Body	Island	20.73	3.47	23.74	3.70	-3.01	1.93E-04	4.80E-03
cg00581783	ABCC5;ABCC5	3	Body;Body	N_Shelf	84.36	4.11	87.37	3.35	-3.01	1.34E-04	3.56E-03
cg00448889	NARG2;NARG2	15	3'UTR;3'UTR	Open sea	66.64	4.02	69.65	3.64	-3.01	6.52E-04	1.24E-02
cg05214676	MAML3	4	Body	Open sea	67.04	4.47	70.05	4.25	-3.01	3.40E-03	4.08E-02
cg23898497	PVT1	8	Body	Open sea	68.32	5.17	71.33	4.62	-3.01	7.64E-04	1.40E-02
cg07811421	GYLTL1B	11	Body	S_Shelf	76.70	3.65	79.71	3.73	-3.01	1.91E-04	4.76E-03
cg23317859	EPB41L1;EPB41L1	20	TSS1500;5'UTR	Open sea	14.84	3.83	17.85	4.27	-3.01	5.41E-04	1.08E-02
cg14938629	NUP205	7	Body	Open sea	79.20	4.72	82.21	3.27	-3.01	1.34E-03	2.13E-02
cg24252287		17	Intergenic	Island	5.84	1.88	8.85	3.50	-3.01	7.36E-08	3.71E-06
cg02482603	RABGAP1L;RABGAP1L	1	TSS1500;Body	Open sea	17.90	3.90	20.91	4.98	-3.01	2.68E-03	3.46E-02
cg00324725		16	Intergenic	Open sea	40.85	4.41	43.86	4.16	-3.01	2.53E-03	3.33E-02
cg12883629		6	Intergenic	Open sea	42.48	5.57	45.49	5.40	-3.01	3.84E-03	4.44E-02
cg12874219	TM4SF4	3	Body	Open sea	80.97	3.38	83.98	4.08	-3.01	3.12E-06	1.29E-04
cg01022871		4	Intergenic	Island	11.78	4.96	14.79	5.69	-3.01	9.41E-04	1.64E-02
cg26283008		15	Intergenic	Open sea	75.61	3.21	78.62	3.04	-3.01	1.81E-04	4.56E-03
cg21177626	FAM50B	6	TSS1500	Island	31.69	4.04	34.70	2.76	-3.01	7.10E-04	1.33E-02
cg03206537	CTSA;CTSA;CTSA	20	Body;Body;Body	S_Shore	61.33	4.26	64.34	5.01	-3.01	5.93E-05	1.80E-03
cg11894951	PALM3	19	Body	Island	43.01	5.13	46.02	4.43	-3.01	2.33E-03	3.15E-02
cg05372597	MST1P2	1	Body	N_Shore	67.29	3.11	70.30	3.22	-3.01	7.61E-04	1.40E-02
cg16984132	SIDT2	11	Body	S_Shore	23.15	2.86	26.16	2.93	-3.01	1.03E-06	4.63E-05
cg25905674		17	Intergenic	Island	9.48	3.01	12.48	4.69	-3.01	3.93E-05	1.26E-03
cg21888373		6	Intergenic	Open sea	64.79	4.16	67.80	4.51	-3.01	3.89E-04	8.33E-03
cg13102028	RPTOR;RPTOR	17	Body;Body	Open sea	76.33	3.58	79.34	3.39	-3.01	4.67E-04	9.61E-03
cg01355739	GNASAS;GNAS	20	Body;3'UTR	Island	51.77	3.42	54.78	3.51	-3.01	1.22E-03	1.98E-02
cg07263393	GATA2;GATA2;GATA2	3	Body;Body;Body	N_Shore	33.66	4.25	36.66	4.89	-3.01	4.27E-03	4.77E-02
cg06405640	QSER1	11	3'UTR	Open sea	75.84	3.23	78.84	3.37	-3.01	4.48E-04	9.31E-03
cg08827756	NRN1	6	Body	Island	16.23	3.11	19.23	3.46	-3.01	3.76E-04	8.12E-03
cg10831642	SH3PXD2A	10	Body	Open sea	61.78	4.40	64.78	4.26	-3.01	2.60E-03	3.39E-02
cg02573234	CDC42EP2	11	5'UTR	Open sea	36.99	3.79	40.00	4.01	-3.01	3.85E-03	4.45E-02

cg18314690	RPA3	7	5'UTR	Open sea	79.56	4.85	82.57	3.88	-3.01	2.82E-03	3.59E-02
cg17736252	FAM73A	1	Body	N_Shelf	80.58	3.88	83.58	2.61	-3.01	7.57E-04	1.39E-02
cg17526483	EVX1	7	TSS1500	N_Shore	32.27	3.81	35.28	3.77	-3.01	2.45E-04	5.81E-03
cg24719489	PBX1	1	Body	Open sea	72.80	5.01	75.81	5.01	-3.01	5.47E-05	1.68E-03
cg18439358	MPP3;MPP3	17	TSS1500;TSS1500	S_Shore	16.93	4.29	19.93	5.61	-3.00	8.48E-05	2.44E-03
cg14844559		18	Intergenic	N_Shore	64.31	4.34	67.31	4.51	-3.00	1.55E-03	2.36E-02
cg12738764	MYO1B;MYO1B;MYO1B	2	5'UTR;5'UTR;5'UTR	Open sea	34.22	3.03	37.23	3.66	-3.00	1.59E-03	2.40E-02
cg21325154	UCP1	4	TSS1500	Island	17.63	3.57	20.63	3.95	-3.00	3.16E-04	7.09E-03
cg21782315		6	Intergenic	S_Shore	80.71	4.02	83.72	4.05	-3.00	2.01E-04	4.97E-03
cg22679120	SNX8	7	Body	N_Shore	51.97	3.84	54.98	4.87	-3.00	9.84E-04	1.69E-02
cg01628245	CNNM4	2	Body	Open sea	75.14	3.96	78.15	3.59	-3.00	1.37E-03	2.16E-02
cg04270358	LOC645323;LOC645323;LOC645323	5	Body;Body;TSS1500	N_Shore	22.39	4.63	25.39	4.49	-3.00	2.58E-03	3.37E-02
cg00555695	PVT1	8	Body	Open sea	40.51	3.92	43.51	4.20	-3.00	1.22E-04	3.30E-03
cg17259183	NKX6-2	10	TSS1500	Island	21.75	4.66	24.75	5.55	-3.00	2.12E-03	2.95E-02
cg22771548	RGNEF	5	Body	Open sea	55.45	4.58	58.45	3.77	-3.00	3.37E-04	7.46E-03
cg08042710	SNORD115-11;SNORD115-36;SNORD1	15	TSS200;TSS200;TSS200;TSS200	Open sea	79.83	5.06	82.83	4.34	-3.00	2.01E-03	2.83E-02
cg06616512	BDKRB2	14	5'UTR	Open sea	30.79	3.08	33.79	3.45	-3.00	1.15E-04	3.13E-03
cg12308110	STEAP4	7	3'UTR	Open sea	84.52	4.32	87.53	3.55	-3.00	5.38E-04	1.07E-02
cg24596898		1	Intergenic	Island	19.19	3.57	22.20	4.91	-3.00	2.04E-03	2.87E-02
cg22431438	UBE2C;UBE2C;UBE2C;UBE2C;UBE2C;U	20	1stExon;5'UTR;TSS200;Body;Body;Body;Bo	Island	9.83	2.23	12.84	4.11	-3.00	1.87E-06	8.07E-05
cg01616356	SBF1	22	Body	N_Shelf	60.20	3.08	63.20	3.02	-3.00	8.93E-05	2.55E-03
cg02258703	MOGAT3	7	TSS1500	Island	28.59	4.33	31.59	4.05	-3.00	8.11E-04	1.46E-02
cg14660024	C17orf81;DULLARD;C17orf81;C17orf8	17	TSS1500;Body;TSS1500;TSS1500;TSS1500;	Island	41.04	4.09	44.05	4.08	-3.00	1.79E-03	2.61E-02
cg26517171	TLX3	5	TSS1500	Island	17.78	2.93	20.78	3.11	-3.00	1.28E-05	4.71E-04
cg11186011		7	Intergenic	S_Shore	85.10	4.46	88.10	3.27	-3.00	1.53E-04	3.98E-03
cg01707361	ENGASE	17	Body	Open sea	52.09	3.00	55.09	5.37	-3.00	9.43E-04	1.64E-02
cg15321363		17	Intergenic	Open sea	70.88	4.64	73.88	4.51	-3.00	2.58E-03	3.37E-02
cg23026906	CR1L	1	Body	S_Shore	68.91	4.27	71.92	3.77	-3.00	6.74E-04	1.27E-02
cg16908257	HOXC8	12	Body	S_Shore	28.92	4.85	31.93	3.59	-3.00	6.34E-04	1.22E-02
cg16099282	PGS1	17	Body	Open sea	36.62	2.78	39.62	3.50	-3.00	2.01E-04	4.97E-03
cg26328626	FGF12;FGF12	3	Body;Body	Open sea	57.44	3.81	60.44	4.68	-3.00	3.00E-03	3.74E-02
cg24747537	SYNGAP1	6	Body	S_Shelf	61.39	3.88	64.39	4.17	-3.00	2.67E-03	3.46E-02
cg16875863	GRIN2D;GRWD1	19	3'UTR;TSS1500	Island	18.79	2.65	21.79	3.17	-3.00	3.94E-05	1.27E-03
cg19025113		2	Intergenic	Island	24.76	3.89	27.76	3.93	-3.00	2.60E-03	3.39E-02
cg08033031	EPS15L1	19	Body	Island	40.46	3.93	43.47	3.42	-3.00	2.77E-03	3.55E-02
cg03555836		8	Intergenic	N_Shore	30.05	5.19	33.05	5.60	-3.00	2.05E-03	2.88E-02
cg03464224	KIAA0922;KIAA0922	4	Body;Body	Open sea	16.92	4.16	19.92	4.33	-3.00	3.14E-03	3.87E-02
cg05975710	ARHGEF17	11	3'UTR	Open sea	48.33	3.97	51.33	4.29	-3.00	1.12E-04	3.08E-03
cg00766322	AUTS2;AUTS2	7	Body;Body	N_Shelf	61.18	4.27	64.18	3.69	-3.00	1.92E-03	2.75E-02
cg18858144	SRC	20	TSS200	N_Shore	53.12	4.67	56.11	4.73	-3.00	2.05E-03	2.87E-02
cg11649016	ITGA7;ITGA7;ITGA7	12	Body;Body;Body	Open sea	75.14	3.17	78.14	2.93	-3.00	5.99E-05	1.82E-03
cg16550656	LRRC70;IPO11;IPO11	5	TSS1500;Body;Body	Open sea	71.12	4.55	74.12	4.92	-3.00	3.03E-03	3.77E-02
cg27364741	OTX1	2	Body	Island	13.54	3.04	16.53	3.52	-3.00	4.22E-05	1.34E-03
cg10883621	C2orf39	2	TSS200	Island	31.35	4.44	34.35	4.38	-3.00	1.25E-03	2.02E-02
cg01892547	NBEAL2	3	Body	Open sea	69.88	3.56	72.88	3.96	-3.00	1.12E-03	1.87E-02
cg01516005		10	Intergenic	S_Shore	11.75	2.74	14.75	7.47	-3.00	3.62E-03	4.26E-02
cg16215362		2	Intergenic	S_Shelf	81.11	3.29	84.11	2.38	-3.00	3.71E-05	1.20E-03
cg19483007	WWTR1;WWTR1;WWTR1	3	Body;Body;Body	Open sea	17.89	4.10	20.89	3.55	-3.00	2.38E-05	8.17E-04
cg00467160	CAB39L	13	5'UTR	N_Shelf	55.10	3.30	58.10	2.42	-3.00	2.05E-04	5.03E-03
cg20698421	SLC1A4;SLC1A4	2	Body;5'UTR	S_Shore	68.58	3.52	71.58	4.03	-3.00	5.76E-05	1.76E-03
cg04005677	FBXL16	16	Body	Island	25.54	4.10	28.53	4.58	-3.00	7.35E-04	1.36E-02
cg01734062	TMEM92;TMEM92;TMEM92	17	1stExon;5'UTR;5'UTR	S_Shore	23.92	3.84	26.91	2.64	-3.00	4.46E-05	1.41E-03
cg12133272	PCM1	8	Body	Open sea	69.40	3.35	72.40	3.18	-3.00	2.87E-04	6.58E-03

cg18504989	DMPK;DMPK;DMPK	19	TSS1500;TSS1500;TSS1500	N_Shelf	72.84	3.89	75.84	4.02	-3.00	4.82E-04	9.84E-03
cg23021329	TLR9	3	Body	S_Shore	42.70	2.93	45.70	3.80	-3.00	8.87E-04	1.57E-02
cg22810403	FAM176B	1	TSS1500	S_Shore	81.38	4.49	84.37	3.24	-3.00	5.07E-04	1.02E-02
cg04370174	SH3TC1	4	Body	Open sea	62.14	3.55	65.14	4.05	-3.00	2.31E-04	5.55E-03
cg14401592	ACCN5	4	TSS1500	Open sea	85.91	5.56	88.90	4.14	-3.00	1.49E-03	2.29E-02
cg08832695	LOC404266;LOC404266;LOC404266;L(	17	Body;Body;Body;Body;5'UTR;Body	S_Shore	35.36	3.36	38.36	4.69	-3.00	2.06E-03	2.88E-02
cg00456086	BDH1;BDH1;BDH1	3	TSS1500;TSS1500;5'UTR	Island	51.36	5.39	54.36	5.29	-3.00	3.73E-03	4.35E-02
cg25722983	STK40	1	5'UTR	Open sea	19.33	3.38	22.32	3.91	-3.00	3.08E-04	6.94E-03
cg00166770	C20orf43	20	TSS1500	N_Shore	22.07	3.24	25.07	4.05	-3.00	7.30E-05	2.15E-03
cg08532862		5	Intergenic	Open sea	38.82	3.74	41.81	4.89	-3.00	3.83E-03	4.43E-02
cg15420906	FAM71D	14	Body	Open sea	82.15	4.20	85.15	4.39	-3.00	1.63E-03	2.45E-02
cg09119665	PNMA1	14	TSS200	S_Shore	32.75	5.15	35.75	5.46	-3.00	1.43E-03	2.22E-02
cg26240970		7	Intergenic	Open sea	82.63	4.92	85.63	4.11	-3.00	2.34E-03	3.16E-02
cg11964823	MICB	6	Body	S_Shore	14.80	5.11	17.79	4.64	-3.00	2.23E-03	3.05E-02
cg05221738		6	Intergenic	N_Shore	84.29	4.82	87.29	4.81	-3.00	3.10E-03	3.83E-02
cg01336912	PALM3	19	TSS200	S_Shore	29.50	4.04	32.49	4.34	-3.00	1.43E-03	2.23E-02
cg26856257		1	Intergenic	S_Shelf	62.12	6.32	65.11	5.15	-3.00	5.22E-04	1.05E-02
cg07986058	ATP10A	15	Body	Open sea	44.70	5.11	47.69	3.98	-3.00	5.06E-04	1.02E-02
cg20433521	C1orf130	1	5'UTR	Open sea	24.13	4.98	27.12	4.62	-3.00	2.78E-04	6.41E-03
cg14286256		9	Intergenic	S_Shelf	71.78	4.60	74.78	3.23	-2.99	2.25E-03	3.07E-02
cg06213233		4	Intergenic	N_Shelf	76.82	2.44	79.82	3.17	-2.99	7.82E-07	3.56E-05
cg01561092	HEATR3	16	Body	S_Shore	23.27	4.31	26.26	4.62	-2.99	2.69E-04	6.24E-03
cg11961813	PSME3;PSME3;AOC2;AOC2	17	3'UTR;3'UTR;TSS1500;TSS1500	Open sea	11.12	3.27	14.12	3.94	-2.99	1.55E-03	2.35E-02
cg08092199	DLX4;DLX4	17	TSS200;Body	Island	33.53	3.70	36.53	3.82	-2.99	7.46E-04	1.37E-02
cg15110403	ANKLE1	19	Body	Island	16.90	3.82	19.89	4.38	-2.99	3.14E-04	7.06E-03
cg07571282	BCMO1	16	Body	Open sea	81.26	4.71	84.25	3.29	-2.99	4.32E-03	4.81E-02
cg12776287	KCTD1;KCTD1;KCTD1	18	5'UTR;Body;5'UTR	N_Shore	65.04	5.38	68.03	4.92	-2.99	3.46E-03	4.13E-02
cg23686556	ADCY8	8	Body	Open sea	9.61	2.70	12.60	5.34	-2.99	3.04E-03	3.77E-02
cg05349542	GRID1	10	TSS1500	S_Shore	58.61	4.27	61.61	4.88	-2.99	1.05E-03	1.77E-02
cg20039983	CDH24;CDH24	14	3'UTR;3'UTR	N_Shore	68.88	3.56	71.88	3.60	-2.99	1.02E-03	1.74E-02
cg04836530		11	Intergenic	N_Shore	73.12	4.93	76.11	4.64	-2.99	1.63E-03	2.44E-02
cg16862729	DSCR9	21	Body	N_Shelf	57.98	3.49	60.97	2.98	-2.99	2.44E-04	5.80E-03
cg01550716	SDCCAG8	1	Body	N_Shore	11.67	2.72	14.67	4.06	-2.99	2.28E-04	5.49E-03
cg19586698	OTUD6B	8	3'UTR	Open sea	85.07	5.63	88.06	5.11	-2.99	4.02E-03	4.58E-02
cg13840419		13	Intergenic	Open sea	80.47	3.94	83.46	3.34	-2.99	2.31E-04	5.55E-03
cg22400015	RESP18	2	Body	Island	29.47	3.91	32.46	3.63	-2.99	1.82E-03	2.65E-02
cg01271485		2	Intergenic	S_Shelf	74.75	4.45	77.74	4.38	-2.99	2.32E-03	3.14E-02
cg13103473		11	Intergenic	N_Shelf	63.60	3.44	66.60	3.11	-2.99	9.73E-05	2.74E-03
cg10976009	RNASE9;RNASE9;RNASE9;RNASE9;RN/	14	5'UTR;5'UTR;5'UTR;5'UTR;5'UTR;5'UTR;5'U	Open sea	82.07	4.04	85.06	5.19	-2.99	6.19E-04	1.19E-02
cg00432454	C20orf54	20	Body	Island	81.55	4.47	84.54	3.33	-2.99	2.84E-03	3.61E-02
cg10634273	DNASE2B;UOX	1	TSS200;TSS1500	Open sea	83.75	5.17	86.75	5.10	-2.99	3.17E-03	3.89E-02
cg02129245	C7orf52	7	Body	Island	15.76	3.69	18.76	4.60	-2.99	5.86E-04	1.14E-02
cg06401138	SEC23B;SEC23B;SEC23B	20	TSS1500;TSS1500;TSS1500	N_Shore	65.31	3.57	68.30	3.61	-2.99	5.53E-04	1.09E-02
cg24091474	TYROBP;TYROBP;TYROBP;TYROBP	19	1stExon;1stExon;5'UTR;5'UTR	Open sea	63.23	4.42	66.22	4.13	-2.99	7.04E-04	1.32E-02
cg04655699	GNL1	6	3'UTR	Open sea	12.29	2.60	15.28	3.03	-2.99	2.50E-06	1.05E-04
cg06432200	NFATC1;NFATC1;NFATC1;NFATC1	18	Body;Body;Body;Body	S_Shore	70.62	3.70	73.61	3.42	-2.99	6.99E-05	2.07E-03
cg01603079		10	Intergenic	Island	15.44	3.16	18.43	3.56	-2.99	3.64E-05	1.18E-03
cg05939956	TIA1;TIA1	2	3'UTR;3'UTR	Open sea	82.53	4.67	85.52	5.10	-2.99	1.35E-04	3.59E-03
cg06652523	PEG3;PEG3;ZIM2;ZIM2;PEG3;ZIM2;MI	19	TSS1500;TSS1500;TSS1500;TSS1500;TSS15	S_Shore	47.37	3.30	50.36	3.20	-2.99	2.36E-04	5.63E-03
cg08049061	LY6G5C	6	TSS1500	Island	12.46	4.25	15.45	5.65	-2.99	3.50E-04	7.68E-03
cg01839452		14	Intergenic	Open sea	66.48	6.02	69.47	5.06	-2.99	1.68E-03	2.50E-02
cg05259881	ZNF423	16	Body	N_Shelf	81.80	4.17	84.79	4.22	-2.99	6.64E-05	1.98E-03
cg23208791		1	Intergenic	N_Shelf	83.59	3.90	86.58	3.25	-2.99	6.92E-04	1.30E-02

cg09270346		19	Intergenic	Island	35.04	3.90	38.03	4.02	-2.99	4.10E-04	8.68E-03
cg18464402	PLIN4	19	3'UTR	Open sea	66.80	4.29	69.79	3.41	-2.99	3.86E-04	8.28E-03
cg04521867	SPTBN4;SPTBN4	19	Body;Body	N_Shore	68.38	5.50	71.37	5.09	-2.99	2.62E-03	3.41E-02
cg19671553	MIR150	19	TSS1500	Open sea	38.74	4.51	41.73	4.62	-2.99	5.48E-04	1.09E-02
cg17746395		10	Intergenic	Open sea	56.34	4.88	59.33	4.63	-2.99	1.17E-03	1.92E-02
cg25221637	C17orf79	17	TSS1500	S_Shore	66.78	4.68	69.77	3.98	-2.99	3.05E-03	3.79E-02
cg05347914	GJD3	17	TSS200	S_Shore	46.14	3.74	49.13	3.77	-2.99	9.85E-04	1.69E-02
cg22586221		15	Intergenic	Open sea	61.13	3.56	64.12	3.91	-2.99	1.38E-04	3.64E-03
cg16965605	HMGA2	12	Body	Open sea	49.31	5.49	52.30	6.06	-2.99	2.51E-03	3.31E-02
cg05835241	PSD3;PSD3	8	Body;Body	Open sea	60.78	4.72	63.76	5.32	-2.99	8.93E-04	1.57E-02
cg20650035		12	Intergenic	Island	63.36	3.52	66.35	2.77	-2.99	1.15E-04	3.14E-03
cg06005098		16	Intergenic	Open sea	47.41	4.01	50.40	3.66	-2.99	1.57E-03	2.38E-02
cg23477460		3	Intergenic	Open sea	21.49	5.13	24.48	7.05	-2.99	1.82E-03	2.65E-02
cg09409930	ZBTB17	1	Body	N_Shore	82.59	4.05	85.58	3.57	-2.99	1.33E-03	2.11E-02
cg03019505	TFIP11;TFIP11	22	Body;Body	S_Shelf	52.38	4.19	55.37	3.97	-2.99	4.40E-03	4.87E-02
cg03055693	LAMA5	20	Body	N_Shore	75.56	3.43	78.54	2.94	-2.99	7.60E-05	2.22E-03
cg11704631	RUNX1	21	Body	N_Shelf	77.29	3.42	80.27	3.89	-2.99	7.75E-05	2.26E-03
cg04051152	LRP6	12	Body	N_Shore	21.00	3.39	23.99	4.28	-2.99	4.02E-03	4.58E-02
cg03451959	PROS1	3	Body	Open sea	84.85	5.09	87.84	4.34	-2.99	3.79E-03	4.40E-02
cg22810790	CA4	17	3'UTR	Open sea	42.03	3.12	45.02	4.22	-2.99	1.29E-03	2.07E-02
cg08583411	ZBTB12	6	Body	Island	42.79	2.98	45.78	4.22	-2.99	2.35E-03	3.17E-02
cg27201679	WDR37	10	Body	Open sea	84.33	3.76	87.32	4.66	-2.99	1.03E-03	1.75E-02
cg03073691		13	Intergenic	S_Shore	43.36	3.92	46.34	4.40	-2.99	8.20E-04	1.47E-02
cg25789721	ATP2B2;ATP2B2	3	3'UTR;3'UTR	N_Shore	58.45	4.81	61.44	4.42	-2.99	1.64E-03	2.45E-02
cg22775000	TMEFF1	9	TSS1500	Island	40.88	5.23	43.87	6.01	-2.99	4.48E-03	4.93E-02
cg01054402	TGOLN2	2	Body	N_Shelf	23.13	3.58	26.11	3.50	-2.99	6.57E-05	1.97E-03
cg18533237		5	Intergenic	S_Shelf	80.91	4.56	83.89	4.54	-2.99	1.53E-03	2.34E-02
cg12964033		18	Intergenic	S_Shore	35.65	5.85	38.64	5.92	-2.99	3.77E-03	4.38E-02
cg09866659	COPA;SUMO1P3;COPA	1	Body;TSS1500;Body	Open sea	52.85	4.92	55.84	4.13	-2.99	1.94E-03	2.77E-02
cg06285575	ZNF267	16	Body	S_Shelf	21.03	3.75	24.02	3.51	-2.99	5.35E-06	2.13E-04
cg01238073	GRB10;GRB10;GRB10;GRB10	7	5'UTR;Body;Body;5'UTR	Open sea	77.50	4.00	80.49	4.09	-2.99	1.58E-03	2.39E-02
cg11329058	RPTOR;RPTOR	17	Body;Body	Open sea	48.42	5.51	51.41	5.08	-2.99	1.34E-03	2.12E-02
cg11312674	GRK5	10	Body	Open sea	57.39	4.55	60.37	4.52	-2.98	1.52E-03	2.33E-02
cg24594459	SLC30A3	2	3'UTR	Open sea	40.46	4.09	43.45	5.13	-2.98	2.34E-04	5.61E-03
cg14649140	HOXA10;HOXA10	7	TSS1500;Body	Island	12.78	2.61	15.76	3.04	-2.98	9.42E-06	3.57E-04
cg25664715	LST1;LST1;LST1;LST1;LST1;LST1;LST1;L	6	Body;3'UTR;3'UTR;3'UTR;3'UTR;3'UTR;Boc	Open sea	75.70	4.69	78.68	4.46	-2.98	1.99E-03	2.82E-02
cg07918509	ICAM2;ICAM2;ICAM2;ICAM2;ICAM2	17	Body;Body;Body;Body;Body	Open sea	65.03	4.74	68.02	3.92	-2.98	1.06E-04	2.94E-03
cg14417687	RNF39;RNF39	6	Body;Body	N_Shore	23.55	3.46	26.54	3.98	-2.98	5.58E-04	1.10E-02
cg25588826	KDM6B	17	Body	Island	59.65	3.89	62.63	4.40	-2.98	2.84E-03	3.61E-02
cg12598340	TMEM54	1	TSS1500	S_Shore	38.99	4.08	41.97	3.77	-2.98	3.60E-04	7.85E-03
cg00393193	FAM193B;FAM193B	5	Body;Body	S_Shore	77.63	3.63	80.61	2.91	-2.98	4.93E-04	1.00E-02
cg04259907	LYPD3	19	TSS200	S_Shore	39.60	3.62	42.58	4.04	-2.98	8.25E-04	1.48E-02
cg08209549		10	Intergenic	Island	78.81	5.21	81.79	4.23	-2.98	1.58E-03	2.40E-02
cg03330678	SEPT9;SEPT9;SEPT9;SEPT9	17	5'UTR;5'UTR;Body;1stExon	Open sea	53.64	4.90	56.62	4.72	-2.98	2.12E-03	2.94E-02
cg22672639	FAT2	5	3'UTR	Open sea	79.53	4.31	82.51	3.80	-2.98	7.54E-04	1.39E-02
cg00964997		4	Intergenic	Island	29.55	3.40	32.53	4.38	-2.98	3.21E-04	7.18E-03
cg08820507		7	Intergenic	Open sea	72.88	4.50	75.87	4.06	-2.98	1.55E-03	2.36E-02
cg14214182	NCRNA00092	9	TSS1500	S_Shore	24.65	3.29	27.63	4.77	-2.98	1.08E-04	2.98E-03
cg08129420	SNTG1	8	Body	Open sea	72.89	5.72	75.88	6.16	-2.98	1.84E-03	2.67E-02
cg21852757	USP13	3	Body	Open sea	81.82	3.45	84.81	2.80	-2.98	3.11E-04	6.99E-03
cg02754084	TPH2	12	Body	Open sea	57.81	4.87	60.80	5.93	-2.98	9.94E-04	1.70E-02
cg25620901	HEATR2	7	Body	Island	88.83	4.18	91.81	2.01	-2.98	1.12E-03	1.87E-02
cg09155997		15	Intergenic	Island	17.04	3.43	20.02	3.57	-2.98	1.35E-05	4.94E-04

cg05721376	SH3BGR;SH3BGR	21	5'UTR;TSS1500	Open sea	76.30	4.77	79.28	4.78	-2.98	4.11E-03	4.65E-02
cg12946080	PLEKHG5;PLEKHG5;PLEKHG5;PLEKHG	1	5'UTR;5'UTR;Body;5'UTR;Body	N_Shore	60.64	3.88	63.62	4.44	-2.98	9.26E-04	1.62E-02
cg22830895	CRYGN	7	TSS1500	S_Shore	22.15	3.61	25.14	4.00	-2.98	3.36E-04	7.45E-03
cg04561571	UBE2N	12	Body	N_Shore	33.01	5.41	35.99	4.79	-2.98	4.22E-03	4.73E-02
cg18941614	UBTF;UBTF;UBTF	17	Body;Body;Body	Island	10.73	2.00	13.71	3.63	-2.98	1.13E-05	4.20E-04
cg25377206	TBX20;TBX20	7	TSS1500;TSS1500	Island	39.41	4.28	42.39	4.63	-2.98	1.88E-03	2.70E-02
cg01250603		10	Intergenic	S_Shore	27.92	3.55	30.91	2.78	-2.98	3.42E-04	7.54E-03
cg06750292	SHISA4;SHISA4	1	TSS1500;TSS1500	Open sea	67.29	4.35	70.27	4.52	-2.98	8.89E-04	1.57E-02
cg22754206	C20orf166;MIR1-1	20	Body;TSS200	N_Shore	53.64	3.89	56.62	4.28	-2.98	3.89E-03	4.48E-02
cg22858169	SMO	7	Body	S_Shelf	55.21	3.54	58.19	3.24	-2.98	9.43E-05	2.67E-03
cg08812235	IGF2BP3	7	Body	Open sea	24.39	5.12	27.37	4.83	-2.98	2.37E-03	3.19E-02
cg06087623	SKI	1	Body	Open sea	83.33	3.57	86.31	3.40	-2.98	3.50E-04	7.68E-03
cg10911365	COL17A1	10	Body	Open sea	79.23	4.27	82.21	3.66	-2.98	1.05E-03	1.78E-02
cg25620321		2	Intergenic	Open sea	85.50	4.82	88.48	3.57	-2.98	1.08E-03	1.81E-02
cg11964099		17	Intergenic	N_Shore	47.80	3.33	50.78	3.78	-2.98	2.05E-03	2.88E-02
cg05614161		5	Intergenic	N_Shelf	61.80	4.57	64.78	4.28	-2.98	1.96E-04	4.87E-03
cg06399148	BAALC;BAALC;C8orf56	8	1stExon;1stExon;Body	Island	14.37	3.39	17.35	3.11	-2.98	5.67E-06	2.24E-04
cg15148933		4	Intergenic	Island	13.38	2.22	16.36	4.47	-2.98	2.04E-04	5.01E-03
cg12569007		10	Intergenic	Open sea	30.65	3.68	33.63	4.80	-2.98	2.52E-03	3.32E-02
cg20124336	RNLS;RNLS	10	Body;Body	Open sea	86.14	4.94	89.12	3.29	-2.98	1.81E-03	2.64E-02
cg23460578	MIR10B	2	TSS1500	N_Shore	27.62	4.41	30.60	4.22	-2.98	1.36E-03	2.14E-02
cg10516080		12	Intergenic	Open sea	40.53	5.06	43.51	5.15	-2.98	2.29E-03	3.10E-02
cg09328518		1	Intergenic	Open sea	87.67	3.84	90.65	1.97	-2.98	1.39E-05	5.07E-04
cg19360104	TDRD10;TDRD10;TDRD10;TDRD10;SH	1	5'UTR;1stExon;1stExon;5'UTR;TSS1500	Island	42.07	5.02	45.05	4.95	-2.98	3.07E-04	6.92E-03
cg08861493	MCAM	11	TSS1500	S_Shore	71.24	3.56	74.22	3.10	-2.98	4.99E-04	1.01E-02
cg23504411	HIST1H3J	6	TSS1500	N_Shore	35.79	4.90	38.77	6.63	-2.98	3.90E-03	4.48E-02
cg04770127	EPSTI1;EPSTI1	13	Body;Body	Open sea	81.86	5.02	84.84	4.36	-2.98	3.05E-03	3.79E-02
cg14382215	NDUFS2;ADAMTS4	1	TSS200;TSS200	N_Shelf	55.33	3.13	58.31	3.00	-2.98	5.03E-05	1.56E-03
cg04405466		10	Intergenic	Open sea	73.88	4.06	76.86	3.26	-2.98	1.69E-03	2.51E-02
cg21451309		20	Intergenic	Open sea	39.60	3.53	42.58	4.12	-2.98	4.23E-03	4.74E-02
cg19356324	PRMT7;SLC7A6OS	16	5'UTR;TSS1500	S_Shore	76.75	5.17	79.73	4.84	-2.98	2.58E-03	3.37E-02
cg07286682	MIR548H4	15	Body	Open sea	63.75	6.20	66.73	5.57	-2.98	2.73E-03	3.51E-02
cg13382117		4	Intergenic	N_Shelf	71.90	3.24	74.88	3.22	-2.98	1.06E-04	2.93E-03
cg10145926	C14orf139	14	Body	Open sea	83.36	3.76	86.34	2.74	-2.98	1.48E-04	3.87E-03
cg07613459		17	Intergenic	N_Shore	12.38	2.63	15.36	4.20	-2.98	1.81E-04	4.57E-03
cg10849393	IDE	10	Body	N_Shore	43.50	2.77	46.48	3.26	-2.98	9.10E-05	2.59E-03
cg04843085	C11orf45;KCNJ5	11	TSS1500;5'UTR	Open sea	62.04	3.28	65.02	2.65	-2.98	4.34E-05	1.37E-03
cg13361558	PLEKHB1;PLEKHB1;PLEKHB1;PLEKHB1	11	5'UTR;5'UTR;5'UTR;Body;Body;1stExon	Open sea	32.15	4.70	35.13	4.44	-2.98	2.01E-03	2.84E-02
cg01526854	BYSL	6	3'UTR	Open sea	66.74	3.98	69.72	3.97	-2.98	3.24E-04	7.23E-03
cg22980877	EXOC6B	2	Body	Open sea	81.07	4.31	84.05	3.27	-2.98	4.76E-04	9.75E-03
cg20804613		7	Intergenic	Open sea	69.94	4.18	72.91	3.90	-2.98	1.58E-03	2.39E-02
cg14417676	TRAK2;STRADB;STRADB	2	TSS200;5'UTR;1stExon	Island	15.21	2.93	18.18	3.57	-2.98	3.46E-05	1.13E-03
cg13323489		3	Intergenic	Island	52.46	3.26	55.44	3.63	-2.98	1.80E-04	4.54E-03
cg22542120		4	Intergenic	Open sea	63.14	3.81	66.11	3.14	-2.98	2.45E-04	5.81E-03
cg02600085	COMP	19	Body	Island	11.60	2.72	14.58	3.35	-2.98	1.42E-05	5.16E-04
cg12782294	PLD1;PLD1	3	5'UTR;5'UTR	Open sea	63.55	6.42	66.53	5.95	-2.98	3.67E-03	4.30E-02
cg04636765		6	Intergenic	S_Shore	82.80	3.70	85.78	2.77	-2.98	8.84E-05	2.53E-03
cg27237814	DDR1;DDR1;DDR1	6	Body;Body;Body	Open sea	71.66	4.78	74.63	4.31	-2.98	6.15E-04	1.19E-02
cg12881854		4	Intergenic	S_Shelf	51.62	4.78	54.60	5.14	-2.98	1.32E-03	2.11E-02
cg01402948		13	Intergenic	S_Shelf	57.47	2.36	60.45	3.44	-2.98	6.30E-06	2.47E-04
cg20373081	SIM1	6	Body	S_Shore	32.76	4.41	35.73	3.35	-2.98	3.04E-04	6.87E-03
cg16556921		6	Intergenic	S_Shelf	28.03	3.27	31.01	3.52	-2.98	6.13E-04	1.19E-02
cg09379340	TIAM2	6	Body	Open sea	19.66	3.82	22.64	3.50	-2.98	1.07E-04	2.97E-03

cg14316431		9	Intergenic	Island	14.40	2.50	17.37	3.18	-2.98	2.41E-06	1.02E-04
cg05025007		7	Intergenic	Open sea	80.84	4.40	83.81	3.74	-2.98	1.25E-03	2.02E-02
cg20317123	TCF21	6	3'UTR	Island	22.65	3.64	25.63	4.09	-2.98	3.40E-04	7.51E-03
cg22800332	DNAJC18	5	TSS200	Open sea	11.22	2.54	14.20	3.76	-2.98	1.18E-05	4.37E-04
cg16289029	FAM60A;FAM60A;FAM60A	12	5'UTR;5'UTR;5'UTR	N_Shelf	77.01	5.65	79.98	5.18	-2.98	3.97E-03	4.54E-02
cg25273707		11	Intergenic	S_Shelf	50.90	4.53	53.88	5.74	-2.98	5.75E-04	1.13E-02
cg26404725	CYP26C1	10	TSS1500	N_Shore	85.53	4.46	88.51	3.92	-2.98	2.89E-03	3.65E-02
cg09496973	ZDHHC6	10	3'UTR	Open sea	85.10	4.45	88.07	3.05	-2.97	9.64E-04	1.67E-02
cg09223851	SH2B2	7	Body	S_Shore	64.70	4.06	67.67	3.13	-2.97	2.89E-04	6.61E-03
cg26558420	TPX2	20	5'UTR	S_Shelf	49.50	4.15	52.47	4.42	-2.97	2.93E-03	3.69E-02
cg13348475		5	Intergenic	Open sea	83.28	3.60	86.25	3.03	-2.97	1.07E-05	4.01E-04
cg16266809		20	Intergenic	N_Shelf	74.92	4.25	77.89	4.79	-2.97	1.08E-04	2.98E-03
cg02851558	MANEAL;MANEAL;MANEAL	1	Body;Body;TSS200	S_Shore	55.22	4.63	58.19	4.33	-2.97	6.84E-04	1.29E-02
cg03284554	HGSNAT	8	TSS1500	N_Shore	13.56	3.17	16.54	4.41	-2.97	2.57E-03	3.37E-02
cg26015087		3	Intergenic	Island	47.63	3.14	50.61	2.57	-2.97	1.44E-06	6.32E-05
cg22381248	HIVEP3;HIVEP3	1	Body;Body	Open sea	70.54	5.68	73.51	3.84	-2.97	1.92E-03	2.75E-02
cg25645064		3	Intergenic	Open sea	14.76	2.79	17.73	3.62	-2.97	2.90E-05	9.68E-04
cg00564426	FGF1;FGF1;FGF1;FGF1;FGF1;FGF1	5	5'UTR;5'UTR;TSS1500;TSS1500;Body;Body	Open sea	57.09	3.35	60.06	4.02	-2.97	9.11E-04	1.60E-02
cg09263904	CTDSP2;MIR26A2	12	Body;TSS1500	Open sea	75.66	4.16	78.63	3.60	-2.97	7.07E-04	1.32E-02
cg25750408	PLAUR;PLAUR;PLAUR	19	Body;Body;Body	Open sea	71.33	3.77	74.31	3.68	-2.97	1.52E-03	2.33E-02
cg25857798		17	Intergenic	Open sea	12.22	2.82	15.20	3.54	-2.97	4.08E-05	1.30E-03
cg16316755		12	Intergenic	N_Shelf	47.22	4.12	50.19	5.04	-2.97	1.93E-04	4.81E-03
cg03806784		8	Intergenic	Open sea	49.49	4.59	52.46	4.91	-2.97	2.24E-03	3.06E-02
cg15401913	CTBP2;CTBP2;CTBP2	10	Body;Body;Body	Open sea	79.31	3.79	82.29	3.28	-2.97	8.88E-04	1.57E-02
cg20967139	ZNF385A;ZNF385A;ZNF385A;ZNF385A	12	5'UTR;1stExon;1stExon;5'UTR	Island	42.09	4.39	45.06	4.98	-2.97	3.57E-04	7.80E-03
cg05662500	GPD1L	3	TSS1500	Island	14.38	3.42	17.36	4.23	-2.97	6.19E-05	1.87E-03
cg11409101	ZNF703	8	Body	Island	51.11	4.28	54.08	4.99	-2.97	2.77E-03	3.54E-02
cg11613164		6	Intergenic	S_Shore	68.98	4.54	71.95	3.69	-2.97	2.96E-03	3.71E-02
cg27234490		3	Intergenic	Open sea	81.44	3.51	84.41	3.45	-2.97	3.60E-04	7.85E-03
cg08177882		14	Intergenic	Open sea	80.47	5.56	83.44	3.96	-2.97	1.45E-03	2.25E-02
cg01757168		3	Intergenic	Island	22.89	3.81	25.86	4.22	-2.97	3.24E-03	3.95E-02
cg23977502		7	Intergenic	S_Shelf	75.43	5.11	78.40	4.79	-2.97	2.12E-03	2.95E-02
cg26724248	C22orf40	22	5'UTR	Island	10.71	1.76	13.68	3.21	-2.97	7.57E-08	3.81E-06
cg09245989	PANK4	1	Body	S_Shore	28.16	4.10	31.13	3.64	-2.97	8.86E-04	1.57E-02
cg03171924	RUNX3	1	Body	Island	24.09	3.49	27.06	3.67	-2.97	1.94E-03	2.77E-02
cg03764818	GALNT9	12	Body	N_Shore	81.90	4.19	84.87	4.46	-2.97	8.19E-04	1.47E-02
cg13702222	MBNL1;MBNL1;MBNL1;MBNL1;MBNL	3	1stExon;5'UTR;5'UTR;1stExon;5'UTR;5'UTF	Open sea	16.69	3.31	19.66	3.24	-2.97	5.86E-05	1.78E-03
cg24717695		7	Intergenic	N_Shore	12.32	2.82	15.29	4.18	-2.97	1.92E-04	4.79E-03
cg02464921		10	Intergenic	Island	76.94	4.56	79.91	4.23	-2.97	1.98E-03	2.81E-02
cg11888738	LOC100130872;LOC100130872-SPON2	4	TSS200;TSS1500	N_Shore	77.62	4.72	80.59	4.05	-2.97	4.68E-04	9.63E-03
cg00174851	AKR1B1	7	Body	N_Shore	61.77	4.18	64.74	4.48	-2.97	7.37E-04	1.36E-02
cg16673904	SHBG	17	Body	S_Shelf	82.29	3.34	85.26	3.30	-2.97	2.84E-05	9.51E-04
cg20385356		7	Intergenic	S_Shelf	77.66	4.19	80.63	4.34	-2.97	1.36E-03	2.15E-02
cg13588346	RAB14	9	Body	Open sea	74.18	3.58	77.15	3.97	-2.97	2.82E-04	6.48E-03
cg23379208	GNL1	6	Body	N_Shore	67.63	4.92	70.60	3.25	-2.97	2.77E-03	3.54E-02
cg16148891		15	Intergenic	Open sea	54.63	4.69	57.60	4.87	-2.97	3.25E-03	3.95E-02
cg09315172		1	Intergenic	S_Shore	78.64	3.26	81.61	2.87	-2.97	2.19E-04	5.31E-03
cg14744050	C19orf35	19	Body	S_Shore	73.87	4.49	76.83	3.51	-2.97	2.18E-03	3.00E-02
cg08835688	GRB10	7	5'UTR	Island	40.38	4.27	43.35	3.89	-2.97	2.28E-03	3.09E-02
cg24249775	KRT10;TMEM99	17	1stExon;5'UTR	Open sea	84.84	5.03	87.81	4.42	-2.97	1.37E-03	2.16E-02
cg24671202	RSPH4A;RSPH4A	6	1stExon;1stExon	Open sea	29.06	8.17	32.03	7.75	-2.97	3.01E-03	3.75E-02
cg05828992		7	Intergenic	Open sea	81.15	3.97	84.12	3.35	-2.97	1.19E-03	1.95E-02
cg21935393		8	Intergenic	Open sea	36.09	4.75	39.06	4.55	-2.97	3.56E-03	4.21E-02

cg06529761	DNASE1	16	TSS1500	Open sea	76.85	3.94	79.82	3.58	-2.97	1.31E-03	2.09E-02
cg17238766	C1orf87	1	TSS1500	S_Shore	21.20	3.99	24.16	5.18	-2.97	2.50E-03	3.30E-02
cg00503866	MACF1	1	TSS1500	N_Shore	47.70	5.09	50.67	4.49	-2.97	2.46E-03	3.27E-02
cg03964554		17	Intergenic	Open sea	74.05	5.82	77.01	4.81	-2.97	2.40E-03	3.21E-02
cg27634130	TMPRSS11D	4	3'UTR	Open sea	86.36	4.36	89.32	3.75	-2.97	4.25E-04	8.94E-03
cg00889599	C10orf140	10	5'UTR	S_Shelf	79.33	3.54	82.30	3.68	-2.97	1.37E-03	2.16E-02
cg24639902	PEBP4	8	Body	Open sea	49.53	4.20	52.49	4.42	-2.97	2.89E-03	3.65E-02
cg04006984		7	Intergenic	Open sea	73.41	5.10	76.38	5.17	-2.97	3.24E-03	3.94E-02
cg01408476	KLKBL4	16	TSS1500	Open sea	65.26	4.89	68.23	4.32	-2.97	1.26E-05	4.63E-04
cg01759562		12	Intergenic	Open sea	18.62	2.58	21.59	3.33	-2.97	6.79E-07	3.12E-05
cg16441347	FIG4	6	Body	Open sea	88.26	3.69	91.23	2.00	-2.97	8.03E-06	3.09E-04
cg00699392	CCDC140	2	3'UTR	N_Shore	22.38	3.54	25.35	4.33	-2.97	4.31E-04	9.04E-03
cg12969533	GPC6	13	Body	Open sea	86.82	4.30	89.79	2.67	-2.97	9.04E-05	2.57E-03
cg12353688		7	Intergenic	N_Shore	23.51	2.95	26.47	4.07	-2.97	5.68E-05	1.73E-03
cg20124551		11	Intergenic	S_Shelf	65.04	4.80	68.00	5.38	-2.97	3.26E-03	3.96E-02
cg07083513	TFAP4	16	Body	N_Shore	66.74	4.19	69.71	3.86	-2.97	2.81E-03	3.58E-02
cg01442889	PLCH2	1	Body	Island	71.83	4.53	74.79	4.19	-2.96	8.50E-04	1.52E-02
cg09906647	FLJ25006;LOC645851	17	Body;TSS1500	Open sea	79.15	3.90	82.11	2.80	-2.96	3.74E-04	8.09E-03
cg26599006	GSC2	22	Body	Island	10.60	2.15	13.56	3.30	-2.96	3.41E-07	1.62E-05
cg02483593		1	Intergenic	S_Shore	20.60	3.82	23.57	4.13	-2.96	1.94E-03	2.76E-02
cg10828900	EGFLAM;EGFLAM;EGFLAM;EGFLAM	5	Body;Body;Body;Body	Open sea	67.73	4.10	70.70	4.22	-2.96	2.04E-04	5.02E-03
cg13130319	ISM2;ISM2	14	Body;Body	N_Shore	27.88	4.06	30.85	4.40	-2.96	3.72E-03	4.34E-02
cg02232421		20	Intergenic	N_Shore	73.42	4.01	76.38	3.17	-2.96	3.46E-04	7.61E-03
cg13247117	RIMS3	1	5'UTR	N_Shore	78.89	3.30	81.85	2.83	-2.96	8.12E-05	2.35E-03
cg02530321	TTPAL;TTPAL	20	TSS1500;TSS1500	N_Shore	29.44	4.65	32.41	4.73	-2.96	5.94E-04	1.16E-02
cg01694400	RSPO3	6	Body	Open sea	48.39	5.16	51.36	4.56	-2.96	2.94E-03	3.69E-02
cg27246282	IQSEC1	3	TSS200	Island	34.84	4.22	37.81	4.66	-2.96	4.86E-04	9.90E-03
cg12927498	FCHSD1	5	TSS200	Island	9.88	3.20	12.85	4.73	-2.96	3.64E-04	7.91E-03
cg15736553	VGLL4;VGLL4;VGLL4;VGLL4;VGLL4	3	Body;Body;1stExon;Body;5'UTR	Island	50.59	3.75	53.56	3.36	-2.96	1.05E-03	1.77E-02
cg17052466	TSSC4	11	TSS200	S_Shore	81.99	3.50	84.95	3.09	-2.96	3.47E-05	1.13E-03
cg15621178	AFAP1L1;AFAP1L1	5	Body;Body	Open sea	62.09	4.33	65.05	4.50	-2.96	6.71E-04	1.27E-02
cg05241461	ZNF492	19	TSS200	N_Shore	11.89	3.39	14.86	3.80	-2.96	5.19E-04	1.04E-02
cg12904004	CALCA;CALCA;CALCA	11	TSS1500;TSS1500;TSS1500	N_Shore	44.72	4.01	47.68	3.54	-2.96	2.78E-04	6.41E-03
cg00309232		7	Intergenic	N_Shore	50.93	4.68	53.89	4.56	-2.96	5.75E-04	1.13E-02
cg06478504	HDAC4	2	Body	Open sea	47.80	4.70	50.76	4.26	-2.96	1.73E-03	2.55E-02
cg18982286	SHANK3	22	Body	S_Shore	16.37	3.83	19.33	4.43	-2.96	2.74E-03	3.52E-02
cg04797496	PCDH12	5	TSS1500	Open sea	82.27	4.51	85.23	3.59	-2.96	6.95E-04	1.30E-02
cg18434354	LOC84931	2	TSS200	Open sea	65.03	3.82	67.99	3.60	-2.96	1.79E-04	4.53E-03
cg24714905	ZIC2	13	Body	Island	24.10	3.40	27.07	3.42	-2.96	1.27E-05	4.68E-04
cg00806959	ST3GAL3;ST3GAL3;ST3GAL3;ST3GAL3;	1	Body;Body;Body;Body;Body;Body;Body;Bo	Open sea	69.45	3.23	72.41	3.25	-2.96	8.07E-05	2.34E-03
cg01135780	EBF1	5	TSS1500	Island	13.45	3.00	16.41	3.04	-2.96	2.78E-06	1.16E-04
cg03713640		15	Intergenic	S_Shelf	77.84	5.18	80.80	3.61	-2.96	3.98E-03	4.55E-02
cg20267775		1	Intergenic	Open sea	16.96	3.17	19.92	4.48	-2.96	3.94E-03	4.52E-02
cg06381000	LRRCC1;LRRCC1	8	TSS1500;TSS1500	N_Shore	81.76	3.62	84.72	3.31	-2.96	1.40E-04	3.70E-03
cg07160932	SEZ6L2;SEZ6L2;SEZ6L2;SEZ6L2	16	Body;Body;Body;Body	S_Shore	9.39	2.34	12.35	3.69	-2.96	5.81E-06	2.29E-04
cg04945457		16	Intergenic	Open sea	42.94	4.48	45.90	5.18	-2.96	2.82E-03	3.59E-02
cg16947583	GULP1	2	Body	Open sea	63.03	4.78	66.00	5.59	-2.96	3.68E-03	4.31E-02
cg14826425		7	Intergenic	Island	11.60	2.69	14.56	3.67	-2.96	1.98E-05	6.93E-04
cg00682002	KCP;KCP	7	Body;Body	S_Shore	32.46	4.00	35.42	2.75	-2.96	3.89E-04	8.34E-03
cg27518993	DUSP7	3	Body	N_Shore	84.08	3.61	87.04	2.98	-2.96	2.70E-04	6.27E-03
cg08159033		4	Intergenic	Open sea	31.92	4.66	34.88	3.84	-2.96	2.61E-03	3.40E-02
cg05740045		8	Intergenic	Island	37.21	4.21	40.17	4.17	-2.96	1.29E-03	2.07E-02
cg16445007	DNAH7	2	Body	Open sea	88.21	4.82	91.17	2.65	-2.96	2.19E-04	5.32E-03

cg14795163	LRRIQ3	1	Body	Open sea	69.55	6.06	72.51	5.46	-2.96	1.59E-03	2.40E-02
cg22878582		2	Intergenic	Open sea	69.18	5.39	72.14	4.03	-2.96	1.65E-03	2.46E-02
cg06263495	ASCL2;ASCL2	11	1stExon;5'UTR	Island	24.67	3.91	27.63	3.68	-2.96	2.10E-05	7.31E-04
cg15744963	HERC2	15	Body	Open sea	82.15	4.03	85.11	3.48	-2.96	1.10E-03	1.84E-02
cg15715049	FBXO6	1	Body	Open sea	87.74	3.56	90.70	2.19	-2.96	6.98E-05	2.07E-03
cg27573975		1	Intergenic	S_Shore	79.55	4.72	82.51	4.05	-2.96	4.02E-03	4.58E-02
cg04723680	SEZ6;SEZ6	17	Body;Body	N_Shore	64.94	4.34	67.90	4.15	-2.96	1.70E-03	2.52E-02
cg17877920	HLA-DPB2	6	Body	S_Shore	37.33	4.39	40.29	4.92	-2.96	6.90E-04	1.30E-02
cg21299458	SCARF2;SCARF2	22	Body;Body	Island	42.08	2.97	45.04	3.52	-2.96	2.93E-04	6.67E-03
cg26841114	CDT1	16	Body	Island	62.97	4.13	65.93	3.71	-2.96	2.68E-03	3.47E-02
cg01838443	HSPA13	21	Body	N_Shelf	74.26	4.37	77.22	4.49	-2.96	3.53E-03	4.19E-02
cg27589594	SLC5A10;SLC5A10	17	Body;Body	N_Shelf	59.57	5.22	62.53	4.75	-2.96	3.36E-03	4.05E-02
cg06549275	ASAP2;ASAP2	2	Body;Body	Open sea	74.58	3.63	77.54	3.78	-2.96	1.85E-03	2.67E-02
cg08083988		19	Intergenic	Island	85.66	2.67	88.62	2.05	-2.96	9.43E-08	4.71E-06
cg25872852	RPLP2;SNORA52	11	Body;TSS200	S_Shore	82.10	4.60	85.06	2.78	-2.96	1.24E-03	2.01E-02
cg27080986	ACOX3;ACOX3	4	Body;Body	Open sea	78.90	4.78	81.86	4.10	-2.96	3.14E-03	3.87E-02
cg12378831	ZC3H13	13	5'UTR	Island	3.80	2.03	6.76	5.31	-2.96	7.72E-04	1.41E-02
cg12292213	ST3GAL1;ST3GAL1	8	Body;Body	Open sea	78.58	3.72	81.54	3.07	-2.96	2.91E-04	6.65E-03
cg23369107		2	Intergenic	N_Shelf	55.62	5.32	58.58	5.16	-2.96	4.29E-03	4.79E-02
cg06421526		17	Intergenic	Open sea	61.87	5.56	64.82	6.38	-2.96	3.47E-03	4.14E-02
cg14144513		11	Intergenic	Island	29.36	5.34	32.32	5.60	-2.96	1.56E-03	2.37E-02
cg26722342	ITGA2	5	Body	Open sea	75.10	4.23	78.06	3.79	-2.96	1.24E-03	2.01E-02
cg00336800	APC2	19	5'UTR	Island	14.77	3.58	17.72	4.72	-2.96	2.67E-03	3.45E-02
cg20057103		11	Intergenic	Open sea	75.82	5.77	78.77	3.57	-2.96	3.81E-04	8.20E-03
cg17606675	NBPF9	1	Body	Open sea	66.72	4.71	69.68	4.22	-2.96	2.15E-03	2.97E-02
cg26328757	ELF3;ELF3;ELF3;ELF3	1	5'UTR;5'UTR;1stExon;1stExon	Open sea	51.61	3.59	54.57	3.54	-2.96	1.04E-03	1.77E-02
cg09042577	MCAM	11	Body	N_Shore	87.96	3.25	90.92	2.27	-2.96	1.43E-05	5.18E-04
cg01100208	CCL17	16	Body	Open sea	63.31	3.97	66.27	4.18	-2.96	2.79E-03	3.56E-02
cg24831725		20	Intergenic	Open sea	34.10	4.81	37.06	5.76	-2.96	3.46E-03	4.13E-02
cg01312546	ZC3H3	8	Body	Island	88.14	4.06	91.10	2.26	-2.96	6.60E-04	1.25E-02
cg13817545		13	Intergenic	Island	26.91	3.29	29.87	3.86	-2.96	2.29E-04	5.51E-03
cg13675051	RNF19A;RNF19A	8	5'UTR;TSS200	Open sea	34.39	4.21	37.35	3.45	-2.96	1.30E-03	2.07E-02
cg17715896		7	Intergenic	N_Shore	28.03	4.57	30.99	4.48	-2.96	1.25E-03	2.02E-02
cg24377560	AFAP1;AFAP1	4	Body;Body	Open sea	44.91	4.48	47.86	3.41	-2.96	1.74E-03	2.57E-02
cg00719767		7	Intergenic	N_Shelf	82.08	4.29	85.04	4.28	-2.96	4.75E-04	9.74E-03
cg20176098		3	Intergenic	Open sea	65.23	4.40	68.19	4.32	-2.96	2.12E-04	5.18E-03
cg21550107		5	Intergenic	Open sea	16.27	3.66	19.23	4.43	-2.96	2.59E-03	3.38E-02
cg26385126	NCOR2;NCOR2	12	Body;Body	S_Shore	44.15	3.98	47.10	3.52	-2.96	3.15E-03	3.87E-02
cg01845013		14	Intergenic	Open sea	77.38	4.38	80.34	3.82	-2.96	3.60E-03	4.24E-02
cg11377730	MTMR7	8	Body	Island	17.99	3.74	20.94	4.48	-2.96	1.19E-04	3.23E-03
cg13863103		9	Intergenic	Open sea	60.11	3.65	63.06	4.48	-2.96	4.06E-03	4.61E-02
cg14433983	DRD4	11	TSS1500	Island	70.10	4.93	73.06	4.33	-2.96	2.15E-03	2.97E-02
cg25387540	C19orf10	19	3'UTR	Open sea	77.17	3.87	80.13	3.51	-2.96	1.32E-03	2.10E-02
cg16946687		12	Intergenic	S_Shelf	73.10	4.93	76.06	4.81	-2.96	3.48E-03	4.15E-02
cg14394939	BTG3;BTG3	21	5'UTR;5'UTR	N_Shore	27.48	3.33	30.44	3.62	-2.95	1.72E-03	2.54E-02
cg04101060	PITX1	5	Body	Island	55.20	3.73	58.16	3.12	-2.95	8.83E-04	1.56E-02
cg03169180	NLGN2	17	TSS1500	N_Shore	63.42	4.81	66.38	4.63	-2.95	1.94E-03	2.77E-02
cg25772143	AMPD2;AMPD2;AMPD2	1	TSS200;Body;Body	Open sea	76.02	4.00	78.97	3.73	-2.95	8.03E-04	1.45E-02
cg19830128	DEFB121	20	TSS1500	Open sea	39.67	5.41	42.62	4.76	-2.95	3.83E-03	4.43E-02
cg09596336	ZEB2;ZEB2;ZEB2	2	Body;Body;Body	N_Shore	12.81	2.33	15.77	3.61	-2.95	9.53E-06	3.61E-04
cg15924738	AKAP6	14	3'UTR	Open sea	71.31	5.30	74.26	4.09	-2.95	8.43E-04	1.51E-02
cg09489128	HSPBP1;HSPBP1	19	TSS1500;TSS1500	S_Shore	64.06	3.95	67.01	4.03	-2.95	1.00E-03	1.71E-02
cg03547840		5	Intergenic	Island	17.75	3.63	20.70	3.54	-2.95	9.66E-05	2.72E-03

cg13089568	LRRC16A	6	Body	Open sea	76.35	5.05	79.31	4.22	-2.95	4.08E-03	4.62E-02
cg14550985	RIN1	11	TSS200	S_Shore	54.69	5.82	57.65	4.17	-2.95	4.28E-03	4.78E-02
cg12569736	KCNK9	8	Body	Open sea	22.64	3.24	25.59	3.12	-2.95	1.53E-05	5.51E-04
cg03843493	SEC24D	4	Body	Open sea	78.08	4.89	81.03	3.47	-2.95	3.76E-03	4.37E-02
cg09808028	RERE;RERE	1	Body;Body	Open sea	55.33	3.33	58.29	3.86	-2.95	5.77E-04	1.13E-02
cg16452067	COL11A2;COL11A2;COL11A2;COL11A2	6	Body;Body;Body;Body	N_Shelf	69.77	5.22	72.73	4.65	-2.95	3.42E-03	4.10E-02
cg18049099	NCOR2;NCOR2	12	Body;Body	S_Shore	84.37	4.48	87.33	3.45	-2.95	1.57E-03	2.37E-02
cg20731878		11	Intergenic	S_Shelf	79.42	5.03	82.38	4.54	-2.95	6.88E-04	1.29E-02
cg11423701	GJA5	1	TSS1500	Open sea	74.56	5.90	77.51	4.83	-2.95	3.89E-03	4.47E-02
cg05182416	TTC25;CNP	17	3'UTR;TSS1500	N_Shore	63.76	4.18	66.71	4.33	-2.95	9.44E-04	1.64E-02
cg26671784		2	Intergenic	S_Shelf	84.72	4.02	87.67	2.83	-2.95	1.69E-04	4.32E-03
cg05079049		16	Intergenic	Island	17.58	3.22	20.54	4.34	-2.95	1.21E-03	1.98E-02
cg11356950	CTBP1;CTBP1	4	Body;Body	Island	74.90	4.46	77.85	3.26	-2.95	2.37E-03	3.18E-02
cg06811183	SHISA5	3	3'UTR	Open sea	40.65	3.38	43.61	3.99	-2.95	1.54E-03	2.35E-02
cg09811317	RNF135;RNF135	17	TSS200;TSS200	N_Shore	10.19	2.63	13.15	4.12	-2.95	1.10E-04	3.02E-03
cg05446860	LOC284798;LOC284798;LOC284798	20	Body;TSS1500;Body	Island	21.60	3.89	24.55	4.90	-2.95	8.53E-04	1.52E-02
cg13823964		9	Intergenic	S_Shelf	60.07	5.33	63.02	4.14	-2.95	2.24E-03	3.06E-02
cg19027507	TRNP1	1	TSS200	Island	11.82	3.11	14.78	4.79	-2.95	2.58E-05	8.75E-04
cg02616565		3	Intergenic	Open sea	89.54	3.31	92.49	1.70	-2.95	1.02E-07	5.06E-06
cg01730666	CD300LG;CD300LG;CD300LG;CD300LG	17	TSS1500;TSS1500;TSS1500;TSS1500	Open sea	25.97	4.02	28.93	4.73	-2.95	3.48E-03	4.15E-02
cg23384017		4	Intergenic	Open sea	76.57	3.55	79.52	3.30	-2.95	1.07E-04	2.96E-03
cg22923050	DDX49	19	Body	N_Shore	84.19	3.91	87.15	2.81	-2.95	7.33E-04	1.36E-02
cg09070799	AKAP12	6	5'UTR	N_Shore	17.54	3.31	20.49	3.26	-2.95	3.79E-05	1.22E-03
cg00171421	KCNMA1;KCNMA1;KCNMA1;KCNMA1	10	Body;Body;Body;Body	Island	22.14	3.97	25.09	3.98	-2.95	6.39E-04	1.22E-02
cg16029760	OSTBETA	15	5'UTR	Open sea	26.72	3.92	29.67	4.97	-2.95	3.74E-04	8.08E-03
cg24173182	HIC1;HIC1	17	Body;Body	Island	55.01	3.28	57.96	4.41	-2.95	2.35E-04	5.62E-03
cg25598840	HOXA2	7	TSS1500	N_Shore	10.92	2.67	13.87	3.23	-2.95	2.93E-05	9.76E-04
cg14476397	C12orf34	12	TSS1500	N_Shore	21.51	3.81	24.46	3.86	-2.95	9.30E-04	1.62E-02
cg24879882	GBX1	7	Body	Island	23.33	4.59	26.28	3.47	-2.95	1.15E-03	1.90E-02
cg22976732	MN1	22	Body	N_Shore	71.90	5.78	74.85	5.08	-2.95	1.58E-03	2.39E-02
cg16102739		1	Intergenic	Open sea	40.41	7.00	43.36	7.28	-2.95	1.39E-04	3.68E-03
cg06284285	FAM188A	10	TSS1500	S_Shore	86.73	3.63	89.68	3.15	-2.95	1.83E-04	4.61E-03
cg07178968	JMJD7-PLA2G4B;JMJD7-PLA2G4B;PLA	15	Body;Body;TSS1500	Open sea	47.45	4.13	50.40	4.20	-2.95	4.34E-03	4.83E-02
cg17218041	ATP11A;ATP11A	13	Body;Body	Open sea	59.97	4.32	62.92	4.47	-2.95	2.85E-03	3.61E-02
cg19729649	NOTUM	17	TSS1500	Island	33.76	4.13	36.71	4.41	-2.95	5.71E-05	1.74E-03
cg09726123	LOC90784	2	TSS1500	Open sea	70.57	4.89	73.52	4.31	-2.95	4.18E-03	4.70E-02
cg13505608	SLC34A3	9	Body	N_Shore	71.66	5.10	74.61	4.52	-2.95	1.39E-03	2.18E-02
cg06952798	MGMT	10	Body	Open sea	82.13	3.90	85.08	3.34	-2.95	2.50E-05	8.51E-04
cg14375121	EXOC6;EXOC6	10	Body;Body	Open sea	87.17	6.57	90.12	4.67	-2.95	2.52E-03	3.32E-02
cg14131273	BARHL1	9	Body	N_Shore	29.52	3.58	32.47	4.02	-2.95	1.62E-04	4.17E-03
cg02083237	BTBD10	11	5'UTR	Open sea	87.52	3.33	90.46	2.99	-2.95	7.98E-06	3.07E-04
cg03149567	INPP5A	10	Body	Open sea	51.92	3.26	54.86	3.64	-2.95	5.81E-04	1.14E-02
cg17739643	GUCA2A	1	TSS200	Open sea	51.43	5.59	54.37	4.81	-2.95	1.96E-03	2.79E-02
cg15687869		8	Intergenic	Open sea	72.70	3.88	75.65	3.60	-2.95	4.73E-04	9.70E-03
cg13080716		7	Intergenic	S_Shore	17.41	3.10	20.36	3.76	-2.95	6.48E-05	1.94E-03
cg19772161	C6orf145	6	Body	Open sea	56.98	3.76	59.92	3.51	-2.95	1.81E-04	4.57E-03
cg22853986	TRMT12	8	1stExon	Open sea	3.61	1.61	6.56	4.30	-2.95	1.68E-05	5.97E-04
cg11669284	WDR70	5	Body	Open sea	60.24	4.86	63.19	5.95	-2.95	2.47E-03	3.28E-02
cg27289227		13	Intergenic	Open sea	76.59	4.47	79.53	4.00	-2.95	8.95E-04	1.58E-02
cg13047336		7	Intergenic	S_Shelf	68.54	3.98	71.49	4.27	-2.95	3.20E-03	3.92E-02
cg21126020	TRIM10;TRIM10	6	TSS200;TSS200	Open sea	71.09	4.08	74.04	3.37	-2.95	4.00E-04	8.52E-03
cg12891342	LPXN;LPXN	11	Body;TSS1500	N_Shore	68.22	4.62	71.17	4.18	-2.95	4.02E-03	4.58E-02
cg22563785	RUFY1;RUFY1;RUFY1	5	Body;Body;Body	S_Shore	73.85	4.07	76.80	3.84	-2.95	1.56E-03	2.37E-02

cg26550337	NFATC1;NFATC1;NFATC1;NFATC1;NFA	18	Body;5'UTR;Body;Body;Body	Island	85.72	3.78	88.67	2.73	-2.95	9.96E-05	2.79E-03
cg16969368	DHX40;DHX40	17	TSS200;TSS200	Island	31.35	4.63	34.30	4.88	-2.95	3.45E-03	4.12E-02
cg26226254	CNTN4	3	5'UTR	Open sea	78.20	4.33	81.15	4.26	-2.95	1.63E-03	2.45E-02
cg17760043	FAT4	4	Body	Open sea	64.62	3.53	67.57	2.95	-2.95	3.89E-04	8.34E-03
cg01522592	GDPD5	11	5'UTR	N_Shore	69.88	3.76	72.82	4.29	-2.95	1.44E-03	2.24E-02
cg23115907		6	Intergenic	N_Shore	26.97	3.50	29.92	4.05	-2.95	7.95E-04	1.44E-02
cg01468579		10	Intergenic	Open sea	43.39	5.07	46.34	3.89	-2.95	5.40E-04	1.07E-02
cg04688596	FBN3	19	Body	N_Shore	44.68	3.94	47.62	4.09	-2.95	8.30E-04	1.49E-02
cg02349096	RAB3D;RAB3D	19	5'UTR;1stExon	Island	28.44	4.54	31.38	4.71	-2.95	1.08E-03	1.81E-02
cg27046528	CYFIP1;CYFIP1	15	Body;Body	S_Shore	56.94	3.79	59.88	3.09	-2.95	6.36E-04	1.22E-02
cg00506540	HAUS3	4	TSS1500	S_Shore	84.32	3.99	87.27	3.09	-2.95	4.83E-04	9.86E-03
cg12457238		10	Intergenic	Island	29.52	3.83	32.46	3.74	-2.95	2.14E-04	5.21E-03
cg20971407	BHLHE40	3	Body	S_Shore	14.73	3.12	17.68	4.63	-2.95	1.35E-03	2.14E-02
cg16832407	TRPM3	9	TSS200	Open sea	18.51	3.89	21.45	3.31	-2.95	8.16E-04	1.47E-02
cg24740320		14	Intergenic	Open sea	42.54	5.26	45.48	5.22	-2.95	7.78E-04	1.42E-02
cg16162611	PRSS35;PRSS35	6	5'UTR;5'UTR	Open sea	72.90	4.87	75.84	4.19	-2.95	1.49E-03	2.30E-02
cg18374948	PRIC285;PRIC285	20	Body;Body	Island	84.03	4.58	86.97	3.49	-2.95	1.10E-03	1.84E-02
cg18415686	INSM2;INSM2	14	3'UTR;1stExon	S_Shore	41.37	4.97	44.32	4.98	-2.94	2.33E-03	3.15E-02
cg15176213	COX7A1;COX7A1	19	5'UTR;1stExon	Island	57.84	4.47	60.78	5.09	-2.94	3.47E-03	4.14E-02
cg14375205		6	Intergenic	Open sea	76.18	4.12	79.13	3.40	-2.94	4.35E-05	1.38E-03
cg23202545	NRSN2	20	Body	Open sea	79.24	3.30	82.18	3.17	-2.94	8.59E-05	2.47E-03
cg24136205	ZIC5	13	TSS200	Island	15.29	2.79	18.23	3.88	-2.94	4.06E-04	8.62E-03
cg09104001		7	Intergenic	N_Shelf	76.92	5.31	79.87	4.93	-2.94	4.06E-03	4.61E-02
cg09898070	MSH6	2	TSS1500	Island	19.18	3.29	22.12	3.48	-2.94	1.70E-04	4.34E-03
cg01301560		19	Intergenic	S_Shelf	65.18	3.68	68.12	3.69	-2.94	6.52E-04	1.24E-02
cg16150979	TFB1M	6	Body	Open sea	73.56	4.95	76.50	3.99	-2.94	1.90E-03	2.73E-02
cg10317638	TMEM125	1	Body	Island	81.35	3.14	84.29	3.26	-2.94	2.91E-05	9.72E-04
cg03375056	YTHDF2	1	TSS1500	N_Shore	72.20	5.07	75.14	4.60	-2.94	4.13E-03	4.66E-02
cg27414487	ROBO4;ROBO4	11	5'UTR;1stExon	Open sea	52.59	3.60	55.53	3.50	-2.94	1.09E-04	3.01E-03
cg11646192	EREG	4	TSS1500	Open sea	23.06	3.54	26.00	5.04	-2.94	1.91E-03	2.74E-02
cg26730763	XPO6	16	Body	Open sea	43.39	5.12	46.33	3.93	-2.94	5.22E-04	1.05E-02
cg22434763	FAM159A	1	TSS1500	Island	65.99	4.54	68.93	4.97	-2.94	1.00E-03	1.71E-02
cg13598317	GPSM1	9	Body	N_Shore	72.89	2.97	75.84	3.11	-2.94	4.82E-05	1.51E-03
cg23654206	PIP5K1C	19	Body	N_Shelf	48.49	4.94	51.44	4.93	-2.94	2.33E-03	3.15E-02
cg21454362	IQCE;IQCE	7	Body;Body	Open sea	76.00	5.02	78.95	4.40	-2.94	4.04E-03	4.60E-02
cg03999452		16	Intergenic	Open sea	82.15	2.74	85.09	2.84	-2.94	2.17E-06	9.25E-05
cg00664416	MANEAL;MANEAL;MANEAL;MANEAL	1	Body;Body;5'UTR;1stExon	S_Shore	16.27	3.28	19.21	4.05	-2.94	3.70E-04	8.02E-03
cg14424530	SLC7A1	13	TSS1500	Island	9.41	2.57	12.35	2.87	-2.94	1.44E-06	6.33E-05
cg05381423	PTPRS;PTPRS;PTPRS;PTPRS;PTPRS;PTF	19	1stExon;1stExon;1stExon;5'UTR;5'UTR;5'U	Island	23.15	4.61	26.10	4.74	-2.94	1.61E-03	2.42E-02
cg24079727	TCF12;TCF12;TCF12;TCF12	15	Body;Body;Body;Body	Open sea	69.47	5.62	72.42	4.77	-2.94	3.76E-03	4.37E-02
cg16097041	FLAD1;LENEP;FLAD1	1	3'UTR;TSS1500;3'UTR	Open sea	77.11	3.71	80.05	3.90	-2.94	1.31E-03	2.09E-02
cg24498554	CSH1;CSH1;CSH1	17	TSS1500;TSS1500;TSS1500	Open sea	61.74	4.34	64.68	4.37	-2.94	2.65E-03	3.44E-02
cg03602071		6	Intergenic	Open sea	62.98	4.53	65.92	4.92	-2.94	1.94E-03	2.76E-02
cg23976431		11	Intergenic	Open sea	33.13	4.41	36.07	4.42	-2.94	2.98E-03	3.73E-02
cg01435515	ZNF687	1	5'UTR	Island	14.19	2.93	17.14	3.85	-2.94	9.27E-05	2.63E-03
cg26140911	SYNGAP1	6	Body	S_Shelf	46.10	3.32	49.04	3.97	-2.94	1.26E-04	3.38E-03
cg04061411	SH2D7	15	Body	Open sea	17.68	3.41	20.62	3.41	-2.94	2.02E-04	4.98E-03
cg25831439		2	Intergenic	Open sea	42.31	4.86	45.25	4.68	-2.94	1.26E-03	2.03E-02
cg12472603	MXRA7;MXRA7;MXRA7	17	Body;Body;Body	N_Shore	71.66	4.23	74.60	3.73	-2.94	2.05E-03	2.88E-02
cg26669806	COMP	19	Body	Island	12.18	3.01	15.12	3.13	-2.94	8.18E-06	3.14E-04
cg06933965	CMKLR1;CMKLR1;CMKLR1;CMKLR1;CI	12	1stExon;1stExon;1stExon;5'UTR;5'UTR;5'U	Open sea	36.29	3.93	39.23	3.97	-2.94	1.22E-03	1.98E-02
cg13900737	PRSS3	9	Body	S_Shelf	87.20	4.22	90.14	3.56	-2.94	3.52E-04	7.72E-03
cg10375267	ZCCHC14	16	Body	Open sea	22.08	3.74	25.02	4.27	-2.94	4.43E-03	4.90E-02

cg16416045	DLX2;DLX2	2	5'UTR;1stExon	Island	10.82	2.90	13.76	3.55	-2.94	1.20E-06	5.32E-05
cg08927739	ASCL1	12	TSS1500	N_Shore	13.59	2.86	16.53	2.40	-2.94	1.36E-06	5.98E-05
cg08702612	MRPL34;ABHD8	19	TSS1500;TSS1500	N_Shore	31.71	2.80	34.64	3.83	-2.94	1.25E-03	2.02E-02
cg21073459	OSCAR;OSCAR;OSCAR;OSCAR;OSCAR;	19	5'UTR;5'UTR;5'UTR;1stExon;1stExon;5'UTF	N_Shore	65.68	4.35	68.62	4.37	-2.94	4.23E-03	4.74E-02
cg12047860	CD83;CD83	6	Body;Body	S_Shelf	78.40	5.11	81.34	3.65	-2.94	3.57E-03	4.23E-02
cg19778822		18	Intergenic	Open sea	46.80	5.47	49.74	4.90	-2.94	1.95E-03	2.77E-02
cg24185864	MIR124-1;LOC157627	8	TSS1500;TSS1500	N_Shore	18.77	3.16	21.71	3.48	-2.94	1.25E-04	3.36E-03
cg18807466	AKAP5	14	5'UTR	S_Shore	10.63	2.95	13.57	3.49	-2.94	9.13E-05	2.59E-03
cg15891991		1	Intergenic	Open sea	48.63	5.08	51.56	5.46	-2.94	1.41E-05	5.13E-04
cg01103582	SLC9A3	5	Body	N_Shore	75.45	4.35	78.39	4.07	-2.94	8.47E-04	1.51E-02
cg16286597	MAST3	19	Body	Island	38.74	5.51	41.68	6.18	-2.94	1.36E-03	2.15E-02
cg01090482	NRG2;NRG2;NRG2;NRG2	5	Body;Body;Body;Body	Open sea	52.32	4.32	55.26	4.22	-2.94	5.84E-04	1.14E-02
cg08858201	DNM1;DNM1	9	Body;Body	Island	21.98	5.54	24.91	5.03	-2.94	3.40E-03	4.09E-02
cg13090238		14	Intergenic	Open sea	77.62	4.30	80.56	4.77	-2.94	3.67E-05	1.19E-03
cg22560214		4	Intergenic	Island	36.76	3.82	39.69	3.96	-2.94	1.94E-04	4.83E-03
cg14252492	ALX4	11	TSS1500	N_Shore	17.84	2.68	20.78	3.80	-2.94	3.35E-05	1.10E-03
cg25246246	ARHGAP27	17	TSS200	N_Shelf	46.23	4.97	49.17	5.22	-2.94	1.84E-03	2.66E-02
cg01919034		15	Intergenic	N_Shelf	62.38	4.34	65.31	4.55	-2.94	5.57E-04	1.10E-02
cg06215107	LRBA;MAB21L2	4	Body;1stExon	Island	18.70	3.63	21.64	4.19	-2.94	3.17E-03	3.89E-02
cg05349062	TLX2;TLX2	2	5'UTR;1stExon	Island	12.13	3.09	15.06	3.62	-2.94	1.62E-05	5.81E-04
cg16263025	TRPC3;TRPC3	4	Body;Body	N_Shore	75.06	4.70	78.00	4.17	-2.94	3.67E-03	4.30E-02
cg22012693	COL22A1	8	5'UTR	Open sea	62.60	4.00	65.54	3.42	-2.94	1.02E-03	1.74E-02
cg00398500	SLC2A9;SLC2A9	4	1stExon;5'UTR	Open sea	48.28	4.26	51.21	3.77	-2.94	1.73E-03	2.55E-02
cg26066724		14	Intergenic	Open sea	60.66	5.72	63.59	4.03	-2.94	1.28E-03	2.05E-02
cg08957484	CCNI2	5	1stExon	Island	35.37	4.43	38.30	4.61	-2.94	1.48E-03	2.28E-02
cg19354039		17	Intergenic	N_Shore	56.96	4.28	59.90	5.45	-2.94	2.76E-03	3.53E-02
cg02451508	RRBP1;RRBP1	20	Body;Body	Island	60.50	3.60	63.43	3.79	-2.94	1.84E-03	2.66E-02
cg21343189	LDB3;LDB3;LDB3;LDB3;LDB3;LDB3;LDI	10	TSS1500;TSS200;1stExon;TSS1500;5'UTR;T	Open sea	73.29	3.15	76.23	2.90	-2.93	1.78E-04	4.51E-03
cg19743103	HES3	1	TSS200	N_Shore	14.27	2.46	17.21	2.77	-2.93	5.24E-06	2.09E-04
cg13151102	KCNJ4	22	TSS1500	S_Shore	13.82	2.73	16.75	3.60	-2.93	7.09E-05	2.10E-03
cg04273431	GNL1;PRR3;PRR3	6	Body;TSS1500;TSS1500	N_Shore	74.82	3.91	77.76	3.16	-2.93	1.60E-04	4.14E-03
cg00718039	MFHAS1	8	Body	Open sea	83.68	4.67	86.61	4.30	-2.93	2.03E-03	2.85E-02
cg24373596		15	Intergenic	S_Shelf	84.65	3.03	87.59	2.28	-2.93	2.92E-05	9.76E-04
cg06915826	DGKA;DGKA;DGKA;DGKA	12	5'UTR;5'UTR;5'UTR;5'UTR	S_Shore	71.15	5.13	74.09	5.31	-2.93	4.53E-03	4.96E-02
cg17970341	LRP5	11	Body	Open sea	60.44	3.59	63.38	4.45	-2.93	6.66E-04	1.26E-02
cg24260359	DNASE1L3	3	TSS200	Open sea	32.50	4.94	35.43	3.97	-2.93	2.40E-03	3.21E-02
cg20897479		11	Intergenic	Open sea	71.80	3.81	74.73	3.35	-2.93	1.59E-05	5.71E-04
cg01456368	DNAH1	3	5'UTR	Open sea	38.54	3.89	41.48	3.95	-2.93	3.95E-04	8.44E-03
cg05256304	SIK3	11	TSS1500	S_Shore	14.56	3.06	17.49	4.81	-2.93	4.43E-03	4.89E-02
cg25786333	OR12D2	6	1stExon	Open sea	88.88	4.93	91.82	2.82	-2.93	1.29E-04	3.46E-03
cg22047469	TTLL10	1	5'UTR	Island	88.45	4.03	91.39	3.16	-2.93	3.59E-04	7.84E-03
cg02296145	IGFALS;IGFALS;IGFALS	16	Body;Body;Body	Island	84.37	3.88	87.31	4.47	-2.93	1.50E-03	2.30E-02
cg03520265	NHSL1;NHSL1	6	Body;Body	Open sea	59.29	3.03	62.22	3.27	-2.93	4.75E-04	9.73E-03
cg26060535		6	Intergenic	S_Shelf	85.72	3.29	88.65	2.88	-2.93	4.14E-05	1.32E-03
cg06230805	AGPAT3;AGPAT3	21	Body;Body	N_Shelf	59.02	3.50	61.95	3.71	-2.93	1.99E-03	2.82E-02
cg09644952	CD86;CD86	3	TSS1500;Body	Open sea	76.45	4.38	79.38	3.55	-2.93	1.90E-05	6.68E-04
cg02959637	RASGRP2;RASGRP2	11	TSS1500;TSS1500	Island	19.40	3.46	22.33	5.02	-2.93	3.13E-05	1.03E-03
cg13876481	C6orf103;LOC729176	6	Body;Body	Open sea	23.35	3.73	26.28	3.70	-2.93	2.86E-04	6.55E-03
cg26437123	SEMA7A;SEMA7A;SEMA7A	15	TSS200;TSS1500;TSS200	Island	22.46	3.87	25.39	5.53	-2.93	5.13E-04	1.03E-02
cg18621538	PLXNA4	7	Body	Open sea	57.81	3.56	60.74	3.38	-2.93	8.42E-04	1.51E-02
cg11122195	FAM134C;FAM134C	17	3'UTR;Body	S_Shelf	80.38	3.98	83.32	2.67	-2.93	1.12E-03	1.86E-02
cg21564128		7	Intergenic	N_Shelf	24.33	3.51	27.27	3.00	-2.93	6.98E-04	1.31E-02
cg09741789	FBN1	15	Body	Open sea	78.56	5.21	81.50	3.68	-2.93	2.03E-03	2.86E-02

cg05716671	ZIC1	3	Body	Island	25.96	5.36	28.89	6.28	-2.93	1.43E-03	2.22E-02
cg08639379		1	Intergenic	Open sea	70.95	3.92	73.88	4.08	-2.93	1.15E-03	1.90E-02
cg02598079		2	Intergenic	N_Shelf	48.01	5.49	50.94	5.49	-2.93	1.40E-03	2.19E-02
cg10519465	ROBO4	11	Body	Open sea	72.44	3.83	75.37	3.68	-2.93	7.71E-04	1.41E-02
cg16869647		8	Intergenic	Open sea	80.75	4.28	83.68	3.54	-2.93	4.68E-04	9.63E-03
cg09393448	WRNIP1;WRNIP1	6	Body;Body	S_Shore	64.02	6.21	66.95	4.67	-2.93	2.34E-03	3.15E-02
cg04904531		1	Intergenic	Open sea	80.08	4.84	83.01	3.82	-2.93	2.11E-03	2.93E-02
cg09510416		1	Intergenic	N_Shelf	38.66	3.95	41.60	4.38	-2.93	7.11E-04	1.33E-02
cg13555290	ANK2	4	5'UTR	Open sea	83.06	5.49	85.99	4.48	-2.93	2.20E-03	3.02E-02
cg07910065		10	Intergenic	Open sea	83.84	3.45	86.77	3.52	-2.93	2.84E-05	9.49E-04
cg25593948	TFAP2B	6	1stExon	N_Shore	29.79	3.68	32.72	3.58	-2.93	1.87E-05	6.57E-04
cg02944084		17	Intergenic	S_Shelf	57.52	5.54	60.45	4.57	-2.93	2.97E-03	3.72E-02
cg14215515	FAM125B;FAM125B	9	Body;Body	S_Shore	75.09	4.83	78.02	3.51	-2.93	3.94E-03	4.52E-02
cg12518410	LOC100287216;SH3RF3	2	Body;TSS200	Island	16.94	6.29	19.88	6.21	-2.93	1.76E-03	2.58E-02
cg08074477	TNS4	17	TSS200	Open sea	63.79	3.66	66.72	3.33	-2.93	1.55E-04	4.02E-03
cg00574563	LHX1	17	Body	Island	17.74	3.71	20.68	4.15	-2.93	2.86E-04	6.55E-03
cg03136486		8	Intergenic	Open sea	82.95	4.65	85.88	4.10	-2.93	8.54E-04	1.52E-02
cg14590826		8	Intergenic	Open sea	65.11	4.73	68.04	4.08	-2.93	7.62E-04	1.40E-02
cg27173469	PCLO;PCLO	7	Body;Body	Open sea	77.17	4.84	80.10	3.89	-2.93	1.61E-03	2.42E-02
cg14405625	VAV2;VAV2	9	Body;Body	N_Shore	54.20	4.54	57.13	3.49	-2.93	3.93E-03	4.51E-02
cg02563906	EFCAB4A	11	Body	Island	87.35	4.89	90.28	3.11	-2.93	4.47E-03	4.92E-02
cg03405834	RAB40C	16	3'UTR	Island	81.23	3.69	84.15	3.19	-2.93	2.91E-04	6.64E-03
cg04063936	FAM38A	16	Body	Open sea	67.90	3.97	70.83	3.75	-2.93	3.73E-03	4.35E-02
cg05222982		13	Intergenic	S_Shore	54.76	5.27	57.69	5.49	-2.93	4.42E-03	4.89E-02
cg17440987		7	Intergenic	Open sea	51.50	4.46	54.42	4.16	-2.93	2.02E-03	2.85E-02
cg05659262	MUC4;MUC4;MUC4	3	TSS200;TSS200;TSS200	Open sea	59.22	3.12	62.15	3.83	-2.93	6.87E-04	1.29E-02
cg04461678		12	Intergenic	Open sea	72.14	4.89	75.06	4.89	-2.93	8.99E-04	1.58E-02
cg14364472	NOTCH1	9	Body	N_Shore	88.92	4.28	91.84	2.63	-2.93	6.77E-04	1.28E-02
cg01539474	H19	11	TSS1500	Island	56.44	3.51	59.37	3.49	-2.93	7.72E-04	1.41E-02
cg25982066		15	Intergenic	Open sea	82.24	3.76	85.17	2.93	-2.93	6.49E-06	2.53E-04
cg25516822	ZNF815	7	Body	N_Shore	75.85	3.48	78.77	3.54	-2.93	3.99E-04	8.50E-03
cg23413494	CNOT7;CNOT7;VPS37A;CNOT7;VPS37.	8	5'UTR;1stExon;TSS200;1stExon;TSS200;5'U	Island	22.71	2.88	25.63	3.22	-2.93	1.59E-04	4.12E-03
cg14304702	YAP1;YAP1	11	Body;Body	Open sea	63.19	4.43	66.12	3.33	-2.93	1.40E-03	2.19E-02
cg01874672		4	Intergenic	N_Shore	46.58	3.24	49.50	3.56	-2.93	1.71E-03	2.52E-02
cg04709144		5	Intergenic	Open sea	69.00	3.65	71.93	3.79	-2.93	8.33E-04	1.49E-02
cg03535862	C6orf115	6	TSS1500	N_Shore	85.11	5.43	88.04	3.33	-2.93	2.30E-03	3.12E-02
cg21273746	TSSC1	2	Body	Island	74.12	5.71	77.05	5.73	-2.93	4.24E-03	4.74E-02
cg22912834		2	Intergenic	Island	29.71	4.31	32.63	4.08	-2.93	1.46E-03	2.26E-02
cg05150608		14	Intergenic	Open sea	60.11	4.10	63.04	3.57	-2.93	2.25E-03	3.07E-02
cg09707814		2	Intergenic	N_Shelf	53.70	4.84	56.63	3.91	-2.93	6.99E-04	1.31E-02
cg10462597		3	Intergenic	Open sea	77.31	4.34	80.24	3.63	-2.93	1.87E-03	2.70E-02
cg27135915	SYCE1;SYCE1;SYCE1	10	Body;5'UTR;Body	Open sea	60.90	6.72	63.83	6.03	-2.93	8.45E-04	1.51E-02
cg14415160		1	Intergenic	Island	27.53	4.05	30.45	3.43	-2.93	1.06E-03	1.79E-02
cg19474571	CLIC1	6	Body	S_Shelf	57.12	3.18	60.05	3.71	-2.93	2.06E-05	7.17E-04
cg04321618	HOXA4	7	TSS1500	S_Shore	21.79	3.66	24.71	3.71	-2.93	6.92E-04	1.30E-02
cg04505205	CNGA3;CNGA3	2	Body;Body	Open sea	73.57	4.24	76.50	4.83	-2.93	3.34E-03	4.04E-02
cg18776793	BCR;BCR	22	Body;Body	Island	83.97	4.11	86.90	3.20	-2.93	4.54E-04	9.40E-03
cg13644197	METRNL	17	Body	S_Shore	56.58	3.27	59.51	3.56	-2.93	1.93E-04	4.81E-03
cg25386676		5	Intergenic	Island	72.86	4.06	75.78	4.22	-2.93	3.00E-03	3.75E-02
cg08046411		11	Intergenic	S_Shore	86.38	3.04	89.31	2.51	-2.93	5.64E-06	2.23E-04
cg10455412		12	Intergenic	Open sea	85.25	4.49	88.18	4.26	-2.93	2.84E-03	3.61E-02
cg26493188	FLYWCH1;FLYWCH1	16	5'UTR;5'UTR	Open sea	81.41	4.51	84.34	3.12	-2.93	3.28E-03	3.98E-02
cg22063206		15	Intergenic	Open sea	78.34	4.84	81.26	4.28	-2.93	2.59E-03	3.38E-02

cg06023994	CLDN11	3	Body	Island	61.19	4.71	64.12	5.76	-2.93	3.97E-03	4.54E-02
cg27324117		4	Intergenic	S_Shore	48.80	5.43	51.73	6.51	-2.93	2.89E-03	3.64E-02
cg09940675	SSBP3;SSBP3;SSBP3	1	Body;Body;Body	Open sea	40.35	3.39	43.28	4.21	-2.92	1.94E-03	2.77E-02
cg22988651	ANKRD28	3	Body	Open sea	83.76	4.25	86.69	4.80	-2.92	3.52E-03	4.18E-02
cg25570913		13	Intergenic	Island	10.62	3.33	13.54	3.99	-2.92	7.37E-05	2.16E-03
cg08891110		15	Intergenic	N_Shore	23.86	3.70	26.79	3.54	-2.92	4.05E-04	8.60E-03
cg13644646	CDYL;CDYL;CDYL	6	TSS1500;Body;Body	Open sea	69.52	3.65	72.45	4.27	-2.92	1.83E-03	2.66E-02
cg09675299		2	Intergenic	Open sea	80.88	4.39	83.80	2.88	-2.92	6.08E-04	1.18E-02
cg22331862	SLC6A11	3	TSS200	Island	34.00	3.38	36.93	3.17	-2.92	1.02E-04	2.86E-03
cg16874077	FSCN3	7	Body	Open sea	48.18	3.48	51.10	3.76	-2.92	1.00E-03	1.72E-02
cg25358903		10	Intergenic	Open sea	82.92	3.77	85.85	3.89	-2.92	1.92E-05	6.73E-04
cg21624228	MAP3K11	11	Body	N_Shelf	75.92	3.85	78.84	3.27	-2.92	6.23E-05	1.88E-03
cg00970396		16	Intergenic	Open sea	12.71	2.98	15.63	4.40	-2.92	1.32E-03	2.10E-02
cg14339068		4	Intergenic	Open sea	9.57	3.52	12.50	5.07	-2.92	4.05E-03	4.60E-02
cg06111486	TRIM10;TRIM10	6	Body;Body	Open sea	79.90	4.86	82.82	3.91	-2.92	3.11E-03	3.84E-02
cg08460590	FXR2	17	Body	S_Shelf	78.38	3.26	81.30	3.14	-2.92	8.56E-05	2.46E-03
cg07150038	FAM12A	14	TSS200	Open sea	40.79	3.94	43.72	5.50	-2.92	1.31E-03	2.09E-02
cg19714985		10	Intergenic	Open sea	41.96	5.03	44.88	5.60	-2.92	4.28E-03	4.78E-02
cg19322479	SNED1	2	Body	N_Shelf	62.57	5.66	65.49	4.85	-2.92	1.97E-03	2.80E-02
cg21475270		1	Intergenic	N_Shore	31.11	2.59	34.03	2.85	-2.92	2.94E-06	1.23E-04
cg03919453	AGAP3;AGAP3	7	3'UTR;Body	Island	89.04	4.63	91.96	2.49	-2.92	1.87E-03	2.69E-02
cg08832723	POLS	5	3'UTR	S_Shore	83.68	2.16	86.60	1.28	-2.92	6.92E-10	3.88E-08
cg08677617		10	Intergenic	N_Shore	27.65	4.55	30.57	4.87	-2.92	2.89E-03	3.65E-02
cg15260552	SRGAP1	12	Body	Open sea	75.59	4.42	78.51	3.67	-2.92	8.25E-04	1.48E-02
cg01285501	HOXA9	7	TSS1500	Island	24.41	3.03	27.33	3.22	-2.92	7.91E-05	2.30E-03
cg16158843	LHFPL4	3	TSS200	S_Shore	21.60	4.26	24.52	4.09	-2.92	1.47E-03	2.27E-02
cg01619796	DYRK1A;DYRK1A	21	5'UTR;TSS1500	Open sea	78.71	4.42	81.63	4.01	-2.92	1.13E-03	1.88E-02
cg20149840	HDAC4	2	Body	Open sea	77.75	3.16	80.67	3.32	-2.92	6.63E-05	1.98E-03
cg14134019	DAPK1	9	Body	Open sea	55.37	3.44	58.29	4.24	-2.92	9.22E-04	1.61E-02
cg19454081		7	Intergenic	S_Shore	69.53	4.89	72.45	4.75	-2.92	6.28E-04	1.21E-02
cg26815396	PACSIN1	6	Body	S_Shore	68.37	3.60	71.29	3.48	-2.92	1.02E-03	1.74E-02
cg10939579	GJB2	13	TSS1500	S_Shore	52.94	6.07	55.86	9.23	-2.92	1.47E-03	2.27E-02
cg16341159	DLG4;DLG4	17	TSS1500;Body	N_Shore	47.15	4.73	50.07	4.13	-2.92	4.53E-04	9.39E-03
cg03720950	C12orf51	12	Body	Open sea	70.78	4.72	73.70	3.69	-2.92	3.20E-03	3.91E-02
cg06216468	MLXIP	12	3'UTR	Open sea	70.08	4.52	73.00	3.30	-2.92	5.11E-04	1.03E-02
cg11152412	EDC3;EDC3;EDC3	15	Body;Body;Body	Open sea	22.19	3.71	25.11	3.79	-2.92	9.82E-05	2.76E-03
cg12139617	ALPK1;ALPK1	4	Body;Body	S_Shelf	87.67	3.73	90.58	3.15	-2.92	3.64E-05	1.18E-03
cg21398469	CCNG2	4	TSS1500	N_Shore	45.30	5.56	48.22	4.07	-2.92	2.09E-03	2.92E-02
cg04784499	ASPSCR1	17	Body	N_Shore	71.30	3.28	74.22	3.56	-2.92	3.40E-04	7.50E-03
cg01288724	ARHGAP26;ARHGAP26	5	Body;Body	Open sea	24.59	3.79	27.51	4.32	-2.92	2.27E-03	3.08E-02
cg12110980	EPS8L2	11	Body	N_Shore	70.67	4.58	73.59	3.94	-2.92	2.41E-03	3.22E-02
cg20171396		4	Intergenic	N_Shore	22.34	4.44	25.25	3.91	-2.92	7.34E-04	1.36E-02
cg10239319	JAKMIP1;JAKMIP1	4	Body;Body	Open sea	54.50	3.59	57.42	4.11	-2.92	1.12E-03	1.87E-02
cg03542754		16	Intergenic	Island	44.02	3.12	46.94	3.39	-2.92	3.06E-04	6.91E-03
cg14930864	YWHAQ	2	Body	Open sea	71.68	4.26	74.60	4.55	-2.92	1.75E-03	2.57E-02
cg10584766		19	Intergenic	N_Shore	78.92	3.53	81.83	3.17	-2.92	5.99E-04	1.17E-02
cg22006699		5	Intergenic	Open sea	43.08	3.07	46.00	4.15	-2.92	1.06E-03	1.79E-02
cg16952974		6	Intergenic	Island	30.42	3.99	33.34	4.16	-2.92	6.05E-04	1.17E-02
cg07167132	PABPC1P2	2	TSS1500	N_Shore	82.60	4.74	85.52	3.56	-2.92	5.09E-04	1.03E-02
cg20103093	ATP2B2;ATP2B2	3	Body;Body	Open sea	58.06	4.71	60.98	4.58	-2.92	7.50E-04	1.38E-02
cg13204699		13	Intergenic	Island	20.23	3.39	23.15	4.59	-2.92	1.17E-03	1.93E-02
cg05490023	LOC404266;LOC404266;LOC404266;L	17	Body;Body;Body;Body;5'UTR;Body	S_Shelf	49.21	4.16	52.13	4.65	-2.92	2.04E-03	2.86E-02
cg01807303		11	Intergenic	N_Shelf	48.00	3.59	50.92	3.65	-2.92	8.96E-04	1.58E-02

cg03877829	C5orf13;C5orf13	5	Body;Body	Open sea	70.21	6.90	73.13	4.91	-2.92	2.42E-04	5.76E-03
cg04964031	PCDHGA4;PCDHGC3;PCDHGA12;PCDH	5	Body;Body;Body;Body;Body;Body;Body;Bo	Island	41.71	4.70	44.63	4.38	-2.92	3.26E-03	3.96E-02
cg19529333	IQSEC3;IQSEC3	12	Body;5'UTR	S_Shore	56.52	5.15	59.44	5.27	-2.92	3.06E-03	3.80E-02
cg16516556		1	Intergenic	Island	11.39	3.04	14.30	3.98	-2.92	2.40E-05	8.22E-04
cg05340042	IRGM	5	TSS1500	Open sea	62.00	3.14	64.92	3.67	-2.92	1.99E-04	4.93E-03
cg01145928	MYO5A;MYO5A	15	Body;Body	Open sea	80.53	3.33	83.45	3.03	-2.92	7.08E-05	2.10E-03
cg07538425		2	Intergenic	N_Shelf	73.96	4.08	76.88	2.67	-2.92	8.99E-04	1.58E-02
cg01356044	MTSS1L	16	TSS1500	S_Shore	84.34	4.37	87.26	3.36	-2.92	3.91E-03	4.49E-02
cg16953031	NAALADL2	3	Body	Open sea	78.06	5.28	80.98	4.32	-2.91	1.00E-03	1.72E-02
cg03063277	ADAP1	7	3'UTR	S_Shore	71.17	4.02	74.09	3.31	-2.91	1.24E-03	2.01E-02
cg26388509	CNPY1	7	Body	N_Shelf	19.93	3.37	22.84	4.33	-2.91	1.32E-03	2.11E-02
cg17361154	ENTPD8;ENTPD8	9	TSS1500;TSS1500	S_Shore	19.79	3.95	22.71	4.09	-2.91	2.69E-03	3.47E-02
cg26689710		5	Intergenic	Open sea	42.72	3.68	45.64	4.61	-2.91	1.42E-03	2.21E-02
cg11263235	CORO1C	12	Body	Open sea	9.09	2.47	12.00	4.17	-2.91	1.76E-06	7.62E-05
cg22964775	LPHN3	4	TSS1500	Open sea	37.81	3.78	40.73	3.61	-2.91	4.96E-04	1.01E-02
cg14370752	DNHD1;DNHD1	11	TSS200;TSS200	Open sea	68.97	5.26	71.89	3.85	-2.91	1.92E-03	2.74E-02
cg06981948		7	Intergenic	Island	37.60	3.75	40.51	3.61	-2.91	1.42E-03	2.22E-02
cg05879505	RTN4RL1	17	Body	N_Shore	59.68	3.86	62.59	4.08	-2.91	8.62E-04	1.53E-02
cg10208303		1	Intergenic	Open sea	85.90	4.25	88.81	3.66	-2.91	4.76E-04	9.76E-03
cg08136772	PCDH8;PCDH8	13	1stExon;1stExon	Island	19.24	3.47	22.15	3.50	-2.91	1.31E-03	2.09E-02
cg15095913	MAFK	7	5'UTR	S_Shore	41.86	3.27	44.77	3.41	-2.91	1.67E-03	2.49E-02
cg17993985	CHD3;CHD3;CHD3;SCARNA21	17	Body;Body;Body;Body	Open sea	87.16	2.85	90.08	2.03	-2.91	7.81E-07	3.56E-05
cg02538199	PAX5;PAX5	9	1stExon;5'UTR	Island	34.80	4.59	37.71	5.40	-2.91	1.19E-03	1.95E-02
cg02273041	PRKCDBP	11	TSS200	Island	15.33	4.15	18.25	4.93	-2.91	3.02E-03	3.76E-02
cg02022808	PCDHGA4;PCDHGA2;PCDHGA5;PCDH	5	Body;Body;1stExon;Body;Body;Body;Body	N_Shore	15.80	3.86	18.71	3.98	-2.91	1.47E-03	2.27E-02
cg03058232		1	Intergenic	Island	11.18	4.11	14.09	4.84	-2.91	1.39E-03	2.19E-02
cg00584174	HRASLS5;HRASLS5;HRASLS5	11	Body;Body;Body	N_Shore	71.61	5.59	74.52	5.22	-2.91	2.92E-03	3.68E-02
cg09651522		6	Intergenic	Island	18.56	2.76	21.47	3.52	-2.91	1.24E-05	4.55E-04
cg24334335	WNT2B;WNT2B	1	Body;TSS1500	Island	17.90	2.99	20.81	3.88	-2.91	2.00E-05	6.99E-04
cg21848788		8	Intergenic	N_Shore	67.30	4.03	70.21	4.06	-2.91	1.61E-03	2.43E-02
cg16582517	BHLHE40	3	3'UTR	S_Shore	14.47	3.29	17.38	4.61	-2.91	2.05E-03	2.88E-02
cg12046506	ZNF710	15	Body	Open sea	64.20	6.53	67.11	6.37	-2.91	2.47E-03	3.28E-02
cg04816394	RNU5E;RNU5D;CKMT2;CKMT2;CKMT2	5	Body;Body;TSS200;TSS200;TSS200	Open sea	34.33	4.73	37.24	5.18	-2.91	1.98E-03	2.81E-02
cg00010742	BARHL2	1	TSS200	N_Shore	17.85	2.75	20.76	3.39	-2.91	6.64E-05	1.98E-03
cg10185099	THBD	20	TSS1500	Island	16.33	3.83	19.24	3.28	-2.91	9.55E-04	1.66E-02
cg13658899	LHX3;LHX3	9	Body;Body	Island	15.19	3.09	18.10	3.65	-2.91	3.81E-04	8.20E-03
cg12759387		1	Intergenic	Open sea	51.57	3.88	54.48	3.27	-2.91	1.74E-04	4.43E-03
cg02567119	TLX1;TLX1NB	10	TSS200;TSS200	Island	14.52	3.78	17.43	3.84	-2.91	1.67E-04	4.28E-03
cg06073220	ARHGEF10	8	5'UTR	Open sea	78.76	4.61	81.67	3.30	-2.91	1.63E-03	2.44E-02
cg04021372		2	Intergenic	Island	21.05	2.86	23.96	3.43	-2.91	4.98E-06	1.99E-04
cg14473102	HOXD8	2	TSS200	Island	10.64	3.74	13.55	4.44	-2.91	2.98E-03	3.72E-02
cg02983436		13	Intergenic	Island	80.75	3.08	83.66	2.56	-2.91	8.85E-06	3.37E-04
cg16349160	RGS8;RGS8	1	TSS1500;TSS1500	Open sea	80.46	4.19	83.37	3.71	-2.91	4.20E-04	8.86E-03
cg11799283		1	Intergenic	Open sea	78.27	3.85	81.18	3.38	-2.91	1.39E-03	2.18E-02
cg14824301		11	Intergenic	N_Shelf	77.44	4.69	80.35	4.74	-2.91	1.76E-03	2.58E-02
cg23487312	CADPS;CADPS;CADPS	3	Body;Body;Body	Open sea	54.48	4.46	57.39	5.46	-2.91	3.36E-04	7.44E-03
cg02929431	BIN2	12	Body	Open sea	56.85	5.09	59.76	3.80	-2.91	1.17E-03	1.93E-02
cg17861230	PDE4C	19	Body	Island	21.51	3.95	24.42	4.89	-2.91	2.55E-03	3.35E-02
cg03340026		19	Intergenic	S_Shore	58.96	5.71	61.87	6.73	-2.91	1.59E-03	2.40E-02
cg06447341		1	Intergenic	Open sea	74.72	4.19	77.63	3.68	-2.91	6.88E-04	1.29E-02
cg24217894	LOC100130776;AGAP2;AGAP2	12	Body;Body;Body	Island	29.66	3.95	32.57	3.43	-2.91	2.59E-03	3.38E-02
cg02176678	TTLL4	2	5'UTR	S_Shore	23.47	6.71	26.38	5.80	-2.91	2.75E-03	3.52E-02
cg06758350	RUNX1;RUNX1;RUNX1;RUNX1;RUNX1	21	5'UTR;1stExon;5'UTR;1stExon;Body	Island	16.26	4.14	19.17	5.10	-2.91	3.99E-03	4.56E-02

cg26883963	SLAMF1	1	3'UTR	Open sea	71.83	4.88	74.73	3.35	-2.91	3.26E-03	3.97E-02
cg05469614	ZSCAN10;ZSCAN10	16	1stExon;5'UTR	S_Shelf	40.61	3.16	43.51	3.74	-2.91	4.57E-04	9.46E-03
cg17932911	ERLIN1;ERLIN1	10	Body;Body	N_Shelf	83.73	3.68	86.63	3.91	-2.91	8.46E-05	2.43E-03
cg04291404		8	Intergenic	S_Shore	62.04	5.19	64.95	5.45	-2.91	1.94E-03	2.77E-02
cg14727987		5	Intergenic	Open sea	24.45	3.79	27.36	4.72	-2.91	2.47E-03	3.27E-02
cg09424415	RPS2P32	7	Body	N_Shore	21.27	2.38	24.18	2.70	-2.91	2.12E-06	9.07E-05
cg25658463	PKN1	19	TSS1500	Island	15.69	3.95	18.60	4.73	-2.91	9.73E-04	1.68E-02
cg07613952	CENPH	5	Body	S_Shelf	83.63	3.95	86.53	3.24	-2.91	1.43E-03	2.22E-02
cg05134054	CUX2	12	Body	S_Shelf	64.73	4.91	67.63	4.86	-2.91	3.17E-03	3.89E-02
cg20593599		17	Intergenic	Island	29.25	3.83	32.16	4.63	-2.91	1.73E-03	2.55E-02
cg00566320		1	Intergenic	S_Shelf	45.37	3.73	48.27	4.39	-2.91	2.07E-03	2.90E-02
cg22728128	DDX20;C1orf183	1	Body;TSS1500	S_Shore	74.13	3.41	77.03	2.99	-2.91	7.77E-05	2.26E-03
cg03749081		1	Intergenic	S_Shelf	75.92	4.29	78.82	4.21	-2.91	2.45E-03	3.26E-02
cg25086874	CD200;CD200	3	Body;Body	Open sea	67.73	4.87	70.64	4.83	-2.91	9.72E-04	1.68E-02
cg20710697	CAMK2A;CAMK2A	5	3'UTR;3'UTR	Open sea	62.12	4.13	65.03	4.12	-2.91	1.61E-03	2.42E-02
cg18647237		5	Intergenic	N_Shore	11.38	2.52	14.28	4.26	-2.91	3.73E-05	1.20E-03
cg01067151		1	Intergenic	N_Shelf	20.89	3.10	23.80	3.96	-2.91	3.73E-04	8.07E-03
cg20286956	MACF1	1	5'UTR	Island	55.39	5.20	58.29	4.90	-2.91	1.58E-03	2.39E-02
cg16184750		5	Intergenic	Open sea	85.34	3.77	88.25	2.98	-2.91	8.96E-05	2.56E-03
cg20747789	LOC286094	8	Body	Open sea	65.47	2.51	68.37	2.19	-2.91	2.94E-06	1.23E-04
cg23234261		14	Intergenic	Open sea	46.63	3.79	49.54	3.35	-2.91	7.41E-04	1.37E-02
cg04465730		17	Intergenic	Open sea	80.67	4.19	83.58	4.01	-2.91	4.14E-03	4.67E-02
cg21505571	DDX5	17	Body	S_Shelf	84.30	3.92	87.21	3.03	-2.91	2.89E-04	6.62E-03
cg16620233	CACNA1D;CACNA1D;CACNA1D	3	Body;Body;Body	Open sea	74.24	4.61	77.15	4.50	-2.91	9.95E-04	1.71E-02
cg05283716		6	Intergenic	Open sea	42.74	5.28	45.64	4.53	-2.91	3.99E-03	4.56E-02
cg02382037	PIWIL1	12	5'UTR	Island	21.65	5.05	24.55	5.07	-2.91	3.35E-03	4.04E-02
cg14882700	OTOP1	4	1stExon	Island	14.69	3.01	17.60	3.96	-2.91	2.99E-04	6.78E-03
cg14474293		15	Intergenic	Open sea	71.52	5.56	74.43	4.98	-2.91	4.27E-03	4.77E-02
cg21161394	CD247;CD247	1	Body;Body	Island	14.70	3.59	17.61	3.94	-2.91	1.97E-03	2.80E-02
cg11117305		14	Intergenic	Open sea	70.20	4.63	73.10	3.22	-2.90	6.37E-04	1.22E-02
cg24658321	ZC3HAV1;ZC3HAV1	7	Body;Body	Open sea	63.52	3.98	66.42	4.50	-2.90	2.58E-03	3.38E-02
cg04078980	C1orf35	1	TSS1500	S_Shore	27.46	3.45	30.37	4.00	-2.90	1.41E-03	2.21E-02
cg05957546	OR5P2	11	TSS1500	Open sea	68.51	4.33	71.41	3.55	-2.90	4.51E-04	9.36E-03
cg21587460	CAMK2B;CAMK2B;CAMK2B;CAMK2B;	7	Body;Body;Body;Body;Body;Body;Body;Bo	Island	16.00	3.37	18.91	4.12	-2.90	1.08E-04	2.98E-03
cg07233343	ARHGEF10L;ARHGEF10L	1	Body;Body	Open sea	65.23	4.52	68.14	4.33	-2.90	1.22E-03	1.99E-02
cg00405789	ZFAT;ZFAT;ZFATAS	8	Body;Body;TSS1500	Open sea	81.25	4.84	84.15	4.86	-2.90	4.13E-03	4.66E-02
cg08615070		16	Intergenic	N_Shore	23.36	4.51	26.26	3.76	-2.90	4.15E-03	4.68E-02
cg17182184	CRYBB1	22	TSS200	Open sea	54.48	4.89	57.38	4.56	-2.90	2.74E-03	3.52E-02
cg07611000	EHBP1L1	11	TSS1500	Island	12.39	2.98	15.29	3.05	-2.90	1.07E-05	4.00E-04
cg00460049	SGIP1	1	TSS1500	Island	24.83	4.48	27.74	5.32	-2.90	2.68E-04	6.23E-03
cg04029645	CDH13	16	Body	S_Shelf	48.72	4.24	51.62	4.55	-2.90	2.94E-03	3.69E-02
cg16450590		2	Intergenic	Island	33.82	4.29	36.73	4.28	-2.90	1.52E-03	2.33E-02
cg22335155	ABL2;ABL2;ABL2;ABL2;ABL2;ABL2;ABL	1	Body;Body;Body;Body;Body;Body;Body;Bo	Open sea	81.56	4.07	84.46	3.80	-2.90	3.20E-03	3.91E-02
cg04730882	HOXD9	2	TSS1500	Island	41.23	3.24	44.14	3.87	-2.90	1.08E-04	2.98E-03
cg11989421	OBSL1	2	Body	Island	76.24	3.91	79.14	4.54	-2.90	4.56E-04	9.44E-03
cg13080606	PROK1	1	3'UTR	Open sea	71.25	3.71	74.15	3.27	-2.90	6.73E-06	2.62E-04
cg18245160		7	Intergenic	N_Shore	20.00	3.97	22.90	3.93	-2.90	4.39E-04	9.16E-03
cg27447219	STK24;STK24	13	Body;TSS200	Open sea	62.87	3.83	65.77	3.52	-2.90	4.32E-04	9.05E-03
cg26433444	PI16	6	3'UTR	S_Shelf	76.32	2.99	79.23	3.37	-2.90	1.22E-04	3.30E-03
cg24449777		6	Intergenic	Island	15.86	2.41	18.76	3.11	-2.90	1.26E-06	5.58E-05
cg16733705		12	Intergenic	Open sea	13.66	2.72	16.56	3.31	-2.90	4.90E-05	1.53E-03
cg02069181		18	Intergenic	Island	71.68	4.13	74.58	3.89	-2.90	2.71E-03	3.49E-02
cg24960639	NOTCH4	6	Body	Open sea	84.54	4.19	87.44	3.55	-2.90	1.70E-03	2.52E-02

cg10599693	SF3B14	2	TSS1500	Island	24.63	4.20	27.53	4.75	-2.90	8.15E-05	2.36E-03
cg23274381	WIZ	19	TSS1500	N_Shore	66.30	2.94	69.20	3.26	-2.90	4.49E-04	9.32E-03
cg25339073	MPPED2	11	5'UTR	Island	17.69	3.77	20.59	4.26	-2.90	2.59E-03	3.38E-02
cg05020625	TTBK1	6	Body	Island	23.89	3.72	26.79	3.55	-2.90	2.84E-04	6.51E-03
cg02814054	MAST3	19	Body	Island	84.93	4.12	87.83	2.94	-2.90	5.07E-04	1.02E-02
cg11102324	C15orf33	15	Body	Open sea	74.99	3.85	77.89	3.48	-2.90	4.90E-04	9.97E-03
cg09195098	GCNT2	6	Body	Open sea	28.61	3.50	31.51	3.56	-2.90	8.98E-04	1.58E-02
cg04253876	LBH	2	TSS1500	Island	27.75	2.99	30.65	3.77	-2.90	1.44E-04	3.77E-03
cg13999188	WIPF1	2	TSS1500	S_Shore	11.26	2.97	14.16	4.18	-2.90	1.69E-04	4.32E-03
cg21807313	DOK3;DOK3;DOK3	5	Body;Body;Body	Island	62.87	3.94	65.77	3.30	-2.90	7.39E-04	1.36E-02
cg14428706	BAT5	6	Body	Open sea	61.06	3.13	63.96	3.56	-2.90	1.64E-04	4.21E-03
cg22977246		12	Intergenic	Island	73.30	3.99	76.20	3.43	-2.90	2.38E-03	3.20E-02
cg03184588	FAM60A;FAM60A;FAM60A	12	TSS1500;TSS1500;TSS1500	S_Shore	31.51	4.76	34.41	3.61	-2.90	2.10E-03	2.93E-02
cg23462338	EP400NL	12	Body	Open sea	81.09	4.61	83.99	4.66	-2.90	3.45E-03	4.12E-02
cg14950165	NUP98;NUP98;NUP98;NUP98	11	Body;Body;Body;Body	Open sea	81.10	3.82	84.00	3.81	-2.90	1.19E-04	3.22E-03
cg17787084	LMX1A	1	Body	N_Shore	34.47	3.28	37.37	3.37	-2.90	2.26E-05	7.79E-04
cg14306362		9	Intergenic	Open sea	68.51	3.95	71.41	4.86	-2.90	2.16E-03	2.98E-02
cg03361297	HLA-J;NCRNA00171	6	Body;Body	S_Shore	69.31	4.14	72.21	4.00	-2.90	9.31E-04	1.62E-02
cg13079234		6	Intergenic	Open sea	66.36	4.03	69.26	3.21	-2.90	1.19E-03	1.94E-02
cg07982082	SIPA1;SIPA1	11	Body;Body	Island	92.49	3.80	95.39	1.86	-2.90	3.98E-07	1.88E-05
cg04647234	H19	11	Body	N_Shore	83.41	4.99	86.31	3.23	-2.90	2.02E-03	2.85E-02
cg11999939	TPCN2	11	Body	Open sea	80.18	3.93	83.08	3.91	-2.90	1.58E-03	2.39E-02
cg16287740	TNPO3	7	3'UTR	Open sea	76.59	3.49	79.49	3.25	-2.90	1.59E-04	4.11E-03
cg06430387	UBE2D1	10	TSS1500	N_Shore	49.98	3.86	52.88	3.58	-2.90	3.29E-03	3.99E-02
cg06338710	GFI1;GFI1;GFI1	1	Body;Body;Body	Island	29.75	3.77	32.65	3.65	-2.90	1.63E-03	2.44E-02
cg05526832		3	Intergenic	Open sea	69.33	3.91	72.23	4.73	-2.90	1.76E-04	4.45E-03
cg17736336	LOC728264	5	Body	Open sea	63.65	4.52	66.55	4.77	-2.90	1.60E-03	2.41E-02
cg14984257	ID2	2	1stExon	Island	25.15	4.04	28.04	3.85	-2.90	1.30E-03	2.08E-02
cg20707679	NFIC;NFIC	19	Body;Body	Island	26.65	3.95	29.54	5.25	-2.90	1.49E-03	2.29E-02
cg20955458	ALDH1L2;ALDH1L2	12	Body;Body	N_Shelf	77.33	3.85	80.23	3.71	-2.90	2.13E-03	2.95E-02
cg27105598	PARD3B;PARD3B;PARD3B	2	Body;Body;Body	Open sea	69.35	5.68	72.24	4.88	-2.90	6.05E-05	1.83E-03
cg08098951	CCDC108	2	Body	Open sea	87.06	4.47	89.96	2.79	-2.90	1.04E-03	1.76E-02
cg10858677		15	Intergenic	Open sea	62.01	4.64	64.91	4.93	-2.90	1.86E-03	2.68E-02
cg04108438		3	Intergenic	N_Shelf	68.46	3.64	71.36	4.02	-2.90	9.54E-04	1.65E-02
cg08698856	RAB2A	8	Body	Open sea	74.83	3.14	77.72	4.08	-2.90	5.49E-04	1.09E-02
cg05897122		4	Intergenic	Open sea	43.30	5.53	46.20	5.10	-2.90	1.14E-03	1.89E-02
cg03594078	PIWIL2;PIWIL2	8	TSS1500;TSS1500	N_Shore	25.06	4.19	27.96	3.92	-2.90	3.62E-03	4.26E-02
cg01160998		16	Intergenic	Open sea	83.66	3.36	86.55	2.67	-2.90	2.03E-04	4.99E-03
cg06783955	CYP2W1	7	TSS1500	N_Shore	81.60	2.75	84.49	2.77	-2.90	1.02E-05	3.83E-04
cg05917537	CFTR	7	Body	Open sea	79.34	3.82	82.24	3.18	-2.90	3.22E-04	7.19E-03
cg00939684	IFT140;TMEM204	16	Body;Body	Island	78.05	4.12	80.95	3.24	-2.90	5.71E-04	1.12E-02
cg14449575	HAPLN4	19	TSS200	S_Shore	47.42	4.08	50.32	4.36	-2.90	1.68E-03	2.49E-02
cg21845300	PALLD;PALLD;PALLD	4	Body;Body;Body	Open sea	74.27	4.99	77.17	4.86	-2.90	1.85E-03	2.68E-02
cg17448336		3	Intergenic	N_Shore	33.12	4.88	36.02	5.10	-2.90	4.29E-03	4.78E-02
cg22953865	CCDC85C	14	Body	Open sea	67.42	4.22	70.32	4.41	-2.90	1.07E-03	1.80E-02
cg13790702		3	Intergenic	Open sea	75.03	4.58	77.92	3.94	-2.90	1.61E-03	2.42E-02
cg10188797	TBC1D16	17	Body	N_Shelf	41.30	3.82	44.20	4.52	-2.89	3.49E-03	4.16E-02
cg03209684	ST3GAL2	16	TSS1500	Island	18.64	3.48	21.53	3.34	-2.89	5.34E-04	1.07E-02
cg07205901	TMEM212	3	3'UTR	Open sea	71.66	4.65	74.55	3.65	-2.89	5.79E-04	1.13E-02
cg26584966	HMGN4	6	5'UTR	S_Shelf	75.99	2.53	78.88	2.23	-2.89	1.30E-06	5.72E-05
cg20682146	PBX1	1	Body	Island	35.68	3.54	38.57	3.56	-2.89	2.31E-03	3.12E-02
cg20445035	PLAUR;PLAUR;PLAUR	19	TSS200;TSS200;TSS200	S_Shore	14.29	3.33	17.18	4.67	-2.89	3.43E-03	4.11E-02
cg19159404	BAIAP2;BAIAP2;BAIAP2;BAIAP2	17	Body;Body;Body;Body	N_Shelf	86.84	4.22	89.74	3.88	-2.89	1.83E-03	2.65E-02

cg11091012	NDRG1;NDRG1	8	3'UTR;3'UTR	Open sea	67.80	4.26	70.69	3.64	-2.89	4.21E-03	4.73E-02
cg18231162		1	Intergenic	Open sea	80.26	2.67	83.15	2.83	-2.89	8.89E-06	3.38E-04
cg17488052	USP33;USP33;USP33	1	5'UTR;5'UTR;5'UTR	N_Shelf	76.35	5.05	79.24	5.19	-2.89	2.25E-03	3.07E-02
cg22311200		17	Intergenic	Island	11.90	2.64	14.79	3.80	-2.89	1.54E-04	4.00E-03
cg26703507	SLC20A1	2	Body	S_Shore	67.49	5.36	70.39	3.86	-2.89	2.45E-03	3.26E-02
cg09853648		11	Intergenic	S_Shelf	77.55	5.00	80.44	4.40	-2.89	1.76E-03	2.58E-02
cg14689595		6	Intergenic	Island	72.75	3.83	75.64	3.70	-2.89	1.88E-03	2.71E-02
cg08211091	NAT8L	4	Body	Island	22.10	3.57	25.00	3.90	-2.89	6.75E-04	1.28E-02
cg03276422	RAB40B	17	Body	Island	18.10	4.99	20.99	4.41	-2.89	6.32E-04	1.21E-02
cg00146004		16	Intergenic	N_Shore	20.05	4.02	22.95	4.15	-2.89	3.91E-03	4.50E-02
cg20162696		8	Intergenic	Open sea	24.83	4.03	27.72	4.38	-2.89	2.44E-03	3.25E-02
cg14306650	RALGPS1	9	Body	Open sea	75.68	5.41	78.57	5.19	-2.89	1.76E-03	2.58E-02
cg03260240	HOXB3	17	5'UTR	S_Shore	22.05	3.67	24.94	3.73	-2.89	3.88E-03	4.47E-02
cg06040894		6	Intergenic	S_Shelf	71.30	4.48	74.19	4.06	-2.89	2.12E-03	2.95E-02
cg00843787		15	Intergenic	N_Shelf	62.60	3.92	65.49	4.25	-2.89	1.86E-03	2.69E-02
cg27509052		8	Intergenic	Open sea	54.03	4.18	56.92	3.95	-2.89	2.36E-03	3.17E-02
cg11986385	NAV2	11	Body	Open sea	77.32	4.02	80.21	4.35	-2.89	2.39E-05	8.20E-04
cg08554536		13	Intergenic	S_Shore	81.91	3.53	84.80	3.31	-2.89	5.52E-06	2.19E-04
cg19815376	FLJ34503	6	TSS1500	Open sea	55.58	5.85	58.47	5.32	-2.89	4.13E-03	4.66E-02
cg05545808	CMIP;CMIP	16	Body;Body	Island	82.09	4.66	84.98	3.56	-2.89	2.79E-03	3.56E-02
cg08070590	ZNF197;ZNF197	3	Body;3'UTR	N_Shelf	72.51	4.59	75.40	4.37	-2.89	4.26E-03	4.77E-02
cg26181334		7	Intergenic	S_Shore	70.77	4.22	73.67	4.54	-2.89	6.54E-04	1.25E-02
cg20588162		15	Intergenic	Open sea	47.63	3.25	50.52	3.78	-2.89	1.85E-03	2.68E-02
cg01881054	LAMA2;LAMA2	6	TSS1500;TSS1500	Open sea	77.10	5.48	80.00	4.27	-2.89	1.38E-03	2.17E-02
cg09679014		13	Intergenic	S_Shelf	66.08	3.35	68.97	4.60	-2.89	1.13E-04	3.09E-03
cg08875705	IFFO1;IFFO1	12	TSS200;TSS200	Island	5.85	1.39	8.74	3.94	-2.89	1.09E-05	4.07E-04
cg19890431	ART5;ART5	11	3'UTR;3'UTR	N_Shelf	76.59	3.73	79.48	2.92	-2.89	1.01E-04	2.82E-03
cg20748656	TRIM39;TRIM39	6	Body;Body	Open sea	85.20	3.72	88.09	3.20	-2.89	7.86E-05	2.29E-03
cg14013195	C2orf39	2	TSS200	Island	17.15	3.88	20.04	4.84	-2.89	1.69E-04	4.32E-03
cg20611882	GPR177;GPR177	1	Body;Body	Island	8.78	2.72	11.67	2.93	-2.89	3.92E-05	1.26E-03
cg26493353	SHC3	9	TSS200	S_Shore	16.73	4.30	19.62	3.55	-2.89	1.53E-03	2.33E-02
cg10552446		2	Intergenic	S_Shore	20.08	3.37	22.97	4.45	-2.89	3.82E-03	4.42E-02
cg17675311	TSC22D1	13	Body	Open sea	79.60	4.96	82.49	5.44	-2.89	5.12E-04	1.03E-02
cg05150077		12	Intergenic	Open sea	45.45	4.67	48.34	4.04	-2.89	6.87E-04	1.29E-02
cg18485215	DEGS2	14	Body	N_Shore	40.91	3.87	43.80	4.43	-2.89	3.25E-03	3.96E-02
cg19014941	GRID1	10	TSS1500	Island	37.02	4.66	39.90	5.31	-2.89	3.37E-03	4.06E-02
cg02051981	PAPPA2;PAPPA2	1	TSS200;TSS200	Open sea	60.85	3.58	63.74	3.16	-2.89	3.41E-04	7.52E-03
cg10071277		8	Intergenic	N_Shelf	55.38	3.92	58.26	3.31	-2.89	1.28E-03	2.06E-02
cg00975876	PRICKLE4	6	Body	N_Shore	74.89	3.86	77.78	2.95	-2.89	3.23E-04	7.22E-03
cg03037030	TNF	6	TSS200	Open sea	61.98	5.34	64.87	5.69	-2.89	6.78E-04	1.28E-02
cg16284507		10	Intergenic	Open sea	46.16	5.14	49.05	5.13	-2.89	2.86E-03	3.62E-02
cg12315391	SHOX2;SHOX2;SHOX2	3	3'UTR;3'UTR;3'UTR	N_Shore	35.11	4.44	38.00	4.92	-2.89	3.97E-03	4.55E-02
cg22510139	PRDM16;PRDM16	1	Body;Body	N_Shore	59.88	4.20	62.77	3.65	-2.89	9.98E-04	1.71E-02
cg21109485	C1orf187	1	5'UTR	Open sea	56.02	4.60	58.90	3.49	-2.89	1.45E-03	2.25E-02
cg24076830	OR5V1	6	TSS1500	Open sea	79.11	4.98	82.00	4.23	-2.89	7.14E-04	1.33E-02
cg20235099	CARKD	13	Body	N_Shelf	79.61	4.63	82.50	3.62	-2.89	1.99E-03	2.82E-02
cg02578368	HIST1H2BH;HIST1H3F	6	TSS1500;1stExon	Island	12.92	2.38	15.81	2.60	-2.89	3.80E-06	1.55E-04
cg02614129	H2AFY;H2AFY;H2AFY;H2AFY	5	5'UTR;5'UTR;5'UTR;5'UTR	N_Shore	36.18	5.44	39.07	6.05	-2.89	7.96E-04	1.44E-02
cg07170253	P11	12	TSS1500	Open sea	46.10	5.14	48.99	5.06	-2.89	1.88E-03	2.71E-02
cg15109449	VPS13D;VPS13D	1	Body;Body	Open sea	78.25	3.38	81.14	3.55	-2.89	4.03E-05	1.29E-03
cg08208899		2	Intergenic	Open sea	85.64	5.20	88.53	3.79	-2.89	9.90E-04	1.70E-02
cg24147024	ZNF619;ZNF619;ZNF619;ZNF619;ZNF6	3	Body;Body;Body;Body;Body;Body	Open sea	53.76	3.44	56.65	4.11	-2.89	3.07E-03	3.80E-02
cg05471509		13	Intergenic	Open sea	91.62	6.02	94.51	3.79	-2.89	2.75E-03	3.53E-02

cg12407867	LOC100130872-SPON2;SPON2;SPON2	4	Body;TSS1500;TSS1500	S_Shore	26.33	2.97	29.22	3.51	-2.89	6.56E-04	1.25E-02
cg26247309	PDGFRB	5	Body	Open sea	24.66	3.31	27.54	4.18	-2.89	2.01E-04	4.96E-03
cg05537653	C1QTNF4	11	Body	Island	46.11	4.25	48.99	4.04	-2.89	2.86E-03	3.62E-02
cg01343313		6	Intergenic	Open sea	20.29	4.70	23.18	6.10	-2.89	2.35E-03	3.17E-02
cg14009688	CALD1;CALD1;CALD1;CALD1;CALD1;C/	7	5'UTR;1stExon;1stExon;1stExon;5'UTR;5'U	Open sea	22.83	4.47	25.72	4.04	-2.89	2.47E-03	3.28E-02
cg10593425	RASGRP3	2	TSS1500	Open sea	80.06	4.32	82.94	4.12	-2.89	5.52E-04	1.09E-02
cg04595807		8	Intergenic	N_Shore	81.02	3.55	83.91	2.95	-2.89	6.46E-05	1.94E-03
cg19855777	C22orf45;ADORA2A	22	Body;Body	Open sea	79.63	3.56	82.52	3.53	-2.89	3.60E-04	7.85E-03
cg06380449	GGA3;GGA3	17	Body;Body	N_Shelf	78.21	3.69	81.09	3.83	-2.89	3.13E-04	7.04E-03
cg19363607		7	Intergenic	N_Shelf	58.08	4.59	60.97	4.54	-2.89	1.56E-03	2.36E-02
cg02102791		8	Intergenic	Open sea	72.73	5.44	75.61	4.49	-2.89	4.13E-03	4.66E-02
cg24902842	UBA7	3	Body	Open sea	63.22	4.35	66.10	3.95	-2.89	2.58E-03	3.38E-02
cg25457007		13	Intergenic	Open sea	53.86	3.66	56.74	3.34	-2.89	3.10E-04	6.98E-03
cg02691192		17	Intergenic	Open sea	29.02	4.65	31.91	3.81	-2.89	1.10E-03	1.83E-02
cg00617292	TNXB	6	Body	Open sea	73.75	4.38	76.64	4.23	-2.89	1.13E-03	1.88E-02
cg17283781	PAQR8	6	TSS1500	N_Shore	33.97	5.24	36.86	6.41	-2.88	9.95E-04	1.70E-02
cg07389205		17	Intergenic	Island	71.18	4.04	74.06	3.99	-2.88	2.17E-03	2.99E-02
cg08528417		7	Intergenic	Open sea	83.76	3.92	86.64	3.11	-2.88	1.05E-03	1.78E-02
cg12552835	CARM1	19	Body	Island	77.99	4.52	80.88	3.71	-2.88	8.68E-04	1.54E-02
cg07128102	TAF1C;TAF1C	16	TSS1500;TSS1500	S_Shore	65.61	4.29	68.50	3.82	-2.88	4.38E-03	4.85E-02
cg10207277	CSMD3;CSMD3	8	TSS200;TSS200	Open sea	16.48	3.04	19.36	4.09	-2.88	1.74E-03	2.56E-02
cg08922729		1	Intergenic	Open sea	28.27	6.40	31.15	7.13	-2.88	1.97E-03	2.80E-02
cg14690181		5	Intergenic	Open sea	36.35	4.47	39.24	3.72	-2.88	2.45E-03	3.26E-02
cg01681881	MIR130A	11	TSS1500	Open sea	8.07	2.23	10.95	2.99	-2.88	1.10E-05	4.09E-04
cg00589493	MIR192;MIR194-2	11	TSS1500;TSS200	N_Shelf	39.32	3.35	42.20	4.86	-2.88	4.46E-03	4.92E-02
cg18236877	CASZ1;CASZ1	1	Body;Body	Open sea	81.87	3.80	84.76	4.10	-2.88	6.82E-04	1.28E-02
cg14305401	ANGPTL6	19	TSS1500	N_Shelf	78.31	5.80	81.20	5.78	-2.88	3.18E-03	3.90E-02
cg12967093	DIRC3	2	TSS200	Island	53.85	3.66	56.73	4.17	-2.88	2.77E-03	3.54E-02
cg04120686		3	Intergenic	N_Shore	20.95	2.55	23.83	3.29	-2.88	9.19E-06	3.49E-04
cg23494757	ECE2;ECE2;ECE2;ECE2	3	3'UTR;3'UTR;3'UTR;3'UTR	Open sea	65.67	3.60	68.55	3.64	-2.88	1.59E-03	2.40E-02
cg22542731	LIMS2	2	TSS1500	Open sea	86.62	5.43	89.50	3.36	-2.88	4.20E-03	4.72E-02
cg23183449	GATAD1	7	3'UTR	Open sea	83.23	5.57	86.12	3.90	-2.88	4.10E-03	4.64E-02
cg10096645	KCTD1;KCTD1	18	5'UTR;TSS1500	Island	21.46	3.40	24.35	4.26	-2.88	6.60E-05	1.97E-03
cg03329949	CACNA2D2;CACNA2D2	3	Body;Body	Open sea	56.53	4.12	59.41	4.64	-2.88	3.81E-03	4.42E-02
cg02625725	GLTPD1	1	3'UTR	N_Shore	66.20	3.33	69.08	2.76	-2.88	9.11E-04	1.60E-02
cg18936052	FAM76A;FAM76A;FAM76A;FAM76A;I	1	TSS1500;TSS1500;TSS1500;TSS1500;TSS15	N_Shore	66.82	4.85	69.70	5.30	-2.88	4.50E-03	4.95E-02
cg04066634	EPB41L2;EPB41L2;EPB41L2	6	3'UTR;3'UTR;3'UTR	Open sea	78.13	5.43	81.01	4.48	-2.88	7.05E-04	1.32E-02
cg11144056	HIC1	17	TSS1500	Island	10.11	4.03	12.99	3.92	-2.88	1.52E-04	3.96E-03
cg14379939		9	Intergenic	Open sea	79.13	5.38	82.02	4.60	-2.88	3.15E-03	3.87E-02
cg07451524	TACC2;TACC2;TACC2;TACC2	10	Body;Body;Body;Body	Island	21.25	4.76	24.13	4.44	-2.88	2.56E-03	3.36E-02
cg21512370	LPAR6;LPAR6;RB1	13	5'UTR;5'UTR;Body	Open sea	55.36	3.04	58.24	2.59	-2.88	5.33E-05	1.64E-03
cg01386424		6	Intergenic	Island	19.83	3.37	22.71	4.10	-2.88	3.94E-04	8.42E-03
cg24276787	RNF39;RNF39	6	TSS1500;TSS1500	S_Shore	67.31	3.81	70.19	3.49	-2.88	1.82E-04	4.59E-03
cg03193847	PALLD;PALLD;PALLD	4	Body;Body;Body	Open sea	69.91	5.17	72.80	5.28	-2.88	1.99E-03	2.82E-02
cg21524356	TPPP	5	Body	Island	74.21	3.80	77.09	3.79	-2.88	1.67E-04	4.28E-03
cg00678286		7	Intergenic	Island	76.05	3.69	78.94	3.87	-2.88	2.31E-04	5.55E-03
cg15156059	SRBD1	2	Body	Open sea	68.92	4.82	71.80	4.49	-2.88	3.28E-03	3.98E-02
cg26248581		10	Intergenic	N_Shore	43.21	4.25	46.09	4.31	-2.88	2.47E-03	3.27E-02
cg18278729	SERPINF2;SERPINF2;SERPINF2	17	Body;Body;Body	Island	59.37	4.08	62.25	4.60	-2.88	2.58E-03	3.38E-02
cg23804620		4	Intergenic	Open sea	22.60	5.83	25.48	6.53	-2.88	1.24E-03	2.01E-02
cg12673726	ABCC2	10	Body	Open sea	28.85	4.60	31.73	5.51	-2.88	2.76E-03	3.53E-02
cg03167948		10	Intergenic	Island	66.53	3.98	69.41	4.02	-2.88	1.20E-03	1.96E-02
cg19567689		14	Intergenic	Island	19.92	3.20	22.80	3.77	-2.88	1.48E-04	3.86E-03

cg16454852		16	Intergenic	Open sea	85.34	3.65	88.22	3.16	-2.88	2.45E-04	5.81E-03
cg22257487	LRIT3;LRIT3	4	5'UTR;1stExon	Open sea	82.22	4.76	85.10	3.77	-2.88	2.57E-03	3.36E-02
cg24226371	SNX33	15	Body	Open sea	37.17	3.10	40.05	3.76	-2.88	2.14E-04	5.21E-03
cg09463522	PIBF1	13	Body	Open sea	87.48	4.08	90.36	2.92	-2.88	1.42E-04	3.75E-03
cg06452665	TNFSF11;TNFSF11;TNFSF11	13	1stExon;5'UTR;5'UTR	Island	8.94	3.23	11.82	4.78	-2.88	1.83E-03	2.66E-02
cg10302472	RXRB	6	Body	N_Shore	60.43	4.40	63.31	4.77	-2.88	2.67E-03	3.45E-02
cg09867598	PRDM6	5	Body	N_Shore	30.37	4.09	33.25	3.84	-2.88	2.75E-03	3.52E-02
cg07237769	TNXB	6	Body	Island	65.51	3.53	68.39	4.31	-2.88	2.51E-03	3.31E-02
cg09274587	LEPREL2	12	TSS1500	N_Shore	34.93	4.88	37.81	5.84	-2.88	4.44E-03	4.90E-02
cg12144728		4	Intergenic	Open sea	66.36	5.50	69.24	4.12	-2.88	5.76E-04	1.13E-02
cg15913211		14	Intergenic	Open sea	86.13	3.68	89.01	2.93	-2.88	8.98E-05	2.56E-03
cg23786883	RASSF2;RASSF2	20	3'UTR;3'UTR	Open sea	73.02	4.37	75.89	4.98	-2.88	3.47E-03	4.14E-02
cg01047631		16	Intergenic	Island	19.18	2.62	22.05	3.06	-2.88	1.05E-05	3.93E-04
cg09978259	LZTR1	22	3'UTR	N_Shore	81.29	3.14	84.17	3.18	-2.88	5.58E-05	1.71E-03
cg18486590	HSPA2;HSPA2	14	3'UTR;1stExon	S_Shore	57.47	3.98	60.35	5.13	-2.88	3.29E-03	3.99E-02
cg01835725	PCBP4	3	TSS1500	Open sea	26.35	3.96	29.23	3.81	-2.88	3.27E-03	3.98E-02
cg08832596		11	Intergenic	N_Shore	54.45	3.83	57.33	3.99	-2.88	7.76E-04	1.42E-02
cg15730030		17	Intergenic	Open sea	20.27	4.83	23.14	4.16	-2.88	2.93E-03	3.69E-02
cg11954332	PRRX1;PRRX1	1	Body;Body	Open sea	17.84	4.24	20.71	4.04	-2.88	1.14E-03	1.89E-02
cg24678438		17	Intergenic	N_Shore	76.73	3.76	79.60	3.57	-2.88	1.20E-04	3.25E-03
cg02180979	CDH23	10	3'UTR	Open sea	83.21	3.42	86.09	2.48	-2.88	2.95E-05	9.84E-04
cg09328979		16	Intergenic	Island	47.27	3.20	50.14	3.61	-2.88	1.77E-04	4.49E-03
cg20930489	ZBTB8B	1	5'UTR	S_Shelf	68.92	4.14	71.80	3.46	-2.88	1.92E-03	2.75E-02
cg14566624	GPR153	1	TSS1500	S_Shore	24.58	3.73	27.46	4.15	-2.88	7.55E-04	1.39E-02
cg04463380	HNRNPA1L2;HNRNPA1L2	13	5'UTR;5'UTR	S_Shelf	77.88	4.62	80.76	4.04	-2.88	4.57E-03	5.00E-02
cg07066550		7	Intergenic	Island	17.50	2.93	20.37	3.06	-2.88	1.25E-05	4.61E-04
cg04229820	RGS13;RGS13	1	TSS1500;TSS1500	Open sea	84.92	3.66	87.79	3.03	-2.88	9.48E-06	3.59E-04
cg26277979		2	Intergenic	Open sea	78.44	4.07	81.31	3.22	-2.88	6.51E-04	1.24E-02
cg27508071	C7orf26	7	TSS1500	Island	64.12	5.22	66.99	4.39	-2.88	3.10E-03	3.83E-02
cg01898867		2	Intergenic	Open sea	68.77	5.26	71.64	5.70	-2.87	4.48E-03	4.93E-02
cg17653411	FBLN7;FBLN7	2	Body;Body	Open sea	71.88	4.99	74.76	3.70	-2.87	4.14E-03	4.67E-02
cg05274650	SERGEF	11	Body	Open sea	88.83	3.66	91.70	2.06	-2.87	5.36E-05	1.65E-03
cg21253459		15	Intergenic	N_Shore	16.38	2.87	19.25	3.34	-2.87	4.19E-06	1.70E-04
cg24947764		4	Intergenic	Island	18.55	4.24	21.43	4.69	-2.87	1.33E-03	2.12E-02
cg06774893	DPEP3;DPEP3	16	Body;Body	N_Shelf	42.75	3.39	45.62	4.02	-2.87	2.55E-05	8.68E-04
cg03533590	LRP5	11	Body	N_Shelf	84.83	3.51	87.70	2.28	-2.87	3.52E-05	1.15E-03
cg16604658	TBK1	12	5'UTR	S_Shore	50.27	5.48	53.15	5.29	-2.87	2.88E-03	3.64E-02
cg01908020	SHF	15	TSS1500	S_Shelf	58.21	4.45	61.08	4.77	-2.87	2.83E-03	3.60E-02
cg10069323	IGSF22	11	TSS200	Open sea	47.23	3.20	50.10	4.21	-2.87	2.30E-03	3.12E-02
cg13263512	MAGI2	7	Body	S_Shelf	77.33	4.55	80.20	3.58	-2.87	1.91E-03	2.74E-02
cg08109537		2	Intergenic	Open sea	84.58	2.61	87.45	2.34	-2.87	1.04E-06	4.64E-05
cg09449324		10	Intergenic	S_Shelf	81.51	3.77	84.38	3.82	-2.87	4.33E-04	9.07E-03
cg22583305		3	Intergenic	Open sea	67.43	3.70	70.31	4.09	-2.87	8.41E-05	2.42E-03
cg04631202	MAN1C1	1	TSS1500	N_Shore	24.41	4.02	27.28	4.35	-2.87	6.88E-04	1.29E-02
cg23444418		13	Intergenic	N_Shelf	61.93	4.28	64.80	4.40	-2.87	3.89E-03	4.48E-02
cg13982454	PRTG	15	Body	N_Shore	30.09	4.58	32.97	4.32	-2.87	1.62E-03	2.44E-02
cg13233407	HOXC9	12	TSS200	Island	14.88	2.95	17.76	3.99	-2.87	4.90E-04	9.96E-03
cg27536559		22	Intergenic	S_Shelf	64.31	5.15	67.18	3.95	-2.87	1.90E-04	4.75E-03
cg00862443	EFHD1	2	Body	S_Shelf	76.63	4.24	79.51	3.58	-2.87	1.54E-04	3.99E-03
cg02395127	LMAN1L	15	TSS1500	Open sea	64.49	4.55	67.36	4.32	-2.87	3.10E-03	3.83E-02
cg01354473	HOXA9	7	1stExon	Island	20.90	2.59	23.77	2.36	-2.87	2.04E-06	8.74E-05
cg11750883	CRCT1	1	TSS200	N_Shore	63.85	4.21	66.72	4.64	-2.87	3.78E-03	4.39E-02
cg06511276	ZDHHC18	1	Body	S_Shore	57.41	4.85	60.28	4.61	-2.87	4.51E-04	9.35E-03

cg00808935		16	Intergenic	Open sea	75.52	5.24	78.39	3.52	-2.87	2.88E-03	3.64E-02
cg22477731		13	Intergenic	N_Shore	79.62	3.20	82.49	2.87	-2.87	4.20E-05	1.33E-03
cg11820833	PLEKHG3	14	5'UTR	Open sea	36.81	5.53	39.68	5.73	-2.87	1.17E-03	1.93E-02
cg14579957	ROR2	9	Body	Island	38.38	3.92	41.25	4.80	-2.87	3.42E-03	4.10E-02
cg02602489	GDPD5	11	Body	Open sea	77.45	5.16	80.32	5.15	-2.87	3.59E-03	4.24E-02
cg17355042	ACADVL;ACADVL	17	3'UTR;3'UTR	Open sea	80.27	4.01	83.14	2.67	-2.87	5.42E-04	1.08E-02
cg23999422		19	Intergenic	Island	11.97	3.55	14.84	3.51	-2.87	7.99E-04	1.45E-02
cg11324660		5	Intergenic	Open sea	48.45	5.34	51.32	6.45	-2.87	2.86E-03	3.62E-02
cg03048889	RALGAPA2	20	Body	Island	31.64	4.67	34.52	5.50	-2.87	3.93E-03	4.51E-02
cg08345189		5	Intergenic	N_Shelf	80.88	3.57	83.75	3.60	-2.87	7.87E-04	1.43E-02
cg02705909	CRTC1;CRTC1	19	Body;Body	Open sea	72.21	3.32	75.08	3.87	-2.87	4.27E-05	1.35E-03
cg16266925	TXNDC6	3	TSS1500	S_Shore	34.75	5.04	37.63	4.73	-2.87	6.40E-04	1.23E-02
cg05020409	DST;DST;DST;DST	6	Body;Body;Body;Body	Open sea	79.08	4.84	81.95	3.95	-2.87	1.93E-03	2.76E-02
cg20908789		8	Intergenic	S_Shore	76.15	4.74	79.02	4.96	-2.87	1.58E-03	2.38E-02
cg07363855	PRDM16;PRDM16;FLJ42875;FLJ42875	1	TSS1500;TSS1500;TSS200;TSS200	Island	21.07	3.15	23.94	4.20	-2.87	5.47E-04	1.09E-02
cg08161802	SDC3	1	Body	N_Shelf	39.12	4.61	41.99	3.58	-2.87	2.13E-03	2.95E-02
cg21178711	BAT2L1	9	Body	N_Shore	74.15	4.25	77.02	3.61	-2.87	2.98E-03	3.72E-02
cg06021552	FOXS1;FOXS1	20	3'UTR;1stExon	N_Shore	49.29	3.83	52.16	3.99	-2.87	6.96E-04	1.30E-02
cg12442899	DDAH2;DDAH2	6	1stExon;5'UTR	Island	28.24	4.57	31.11	2.68	-2.87	1.13E-03	1.87E-02
cg16928066	EMX1	2	Body	Island	27.89	3.76	30.76	3.03	-2.87	6.65E-06	2.59E-04
cg15611336	RPP25	15	1stExon	Island	17.05	3.72	19.92	3.81	-2.87	4.67E-04	9.61E-03
cg27193813	BCL7A;BCL7A	12	Body;Body	Open sea	81.95	4.11	84.82	3.63	-2.87	7.52E-04	1.38E-02
cg08637446		11	Intergenic	Open sea	46.68	3.23	49.55	4.03	-2.87	3.04E-03	3.78E-02
cg07661704	SLC7A11	4	Body	Open sea	69.41	4.68	72.28	4.41	-2.87	2.11E-03	2.93E-02
cg21809694		17	Intergenic	S_Shelf	49.38	3.36	52.25	2.68	-2.87	3.93E-05	1.26E-03
cg00586817		2	Intergenic	S_Shore	19.08	2.99	21.94	4.36	-2.87	9.26E-04	1.62E-02
cg04949153	CADPS;CADPS;CADPS	3	Body;Body;Body	Open sea	45.59	4.47	48.46	4.91	-2.87	4.12E-03	4.66E-02
cg03595199	IDO2	8	Body	Open sea	87.85	3.95	90.72	3.16	-2.87	2.33E-04	5.58E-03
cg02466016	SGCD;SGCD;SGCD	5	Body;Body;Body	Open sea	84.62	4.40	87.49	3.28	-2.87	7.69E-04	1.41E-02
cg23305129		7	Intergenic	S_Shore	55.07	3.57	57.93	3.77	-2.87	1.77E-03	2.59E-02
cg03415804		11	Intergenic	S_Shelf	84.73	3.71	87.60	3.09	-2.87	2.56E-05	8.68E-04
cg14199723	PLEC1;PARP10	8	TSS1500;Body	Island	65.73	4.86	68.60	5.44	-2.87	6.83E-04	1.29E-02
cg00155619	HSD17B8	6	Body	S_Shore	42.62	3.63	45.48	3.93	-2.87	2.86E-04	6.55E-03
cg16069079		15	Intergenic	Island	32.92	4.15	35.79	4.50	-2.87	3.04E-03	3.78E-02
cg18383890	ZIC2	13	Body	N_Shore	16.86	3.85	19.73	4.22	-2.87	2.21E-03	3.03E-02
cg25319570	BAG2	6	TSS1500	N_Shore	73.31	4.62	76.18	4.60	-2.87	1.64E-03	2.45E-02
cg01768686	SPNS3	17	TSS200	Open sea	51.25	5.19	54.12	4.08	-2.87	9.31E-04	1.63E-02
cg00027081	TSSC1	2	Body	S_Shelf	74.79	4.04	77.66	3.32	-2.87	1.76E-03	2.58E-02
cg00268831	LPAR6;LPAR6;RB1	13	TSS200;5'UTR;Body	Open sea	81.08	4.62	83.94	3.77	-2.87	1.43E-03	2.23E-02
cg25157472	IQSEC1;IQSEC1	3	Body;Body	Open sea	72.42	3.88	75.29	3.56	-2.87	1.70E-03	2.52E-02
cg01793368	PLCB3	11	Body	N_Shelf	82.46	3.86	85.33	2.98	-2.87	1.54E-03	2.35E-02
cg09794680	CPNE6	14	TSS1500	Open sea	58.94	3.99	61.81	4.27	-2.87	2.48E-04	5.86E-03
cg20055101	ZSCAN10	16	1stExon	S_Shelf	76.63	4.10	79.50	3.90	-2.87	2.02E-03	2.84E-02
cg15815117	LOC100240726	20	TSS1500	Open sea	41.08	3.87	43.95	3.12	-2.87	1.01E-03	1.73E-02
cg06544643	ELF2	4	Body	Island	48.90	3.75	51.77	4.83	-2.87	4.53E-03	4.96E-02
cg09456065		15	Intergenic	S_Shelf	62.98	4.04	65.84	4.22	-2.87	2.04E-03	2.86E-02
cg24233679	KLHL26	19	Body	S_Shore	66.16	4.21	69.03	4.49	-2.87	9.30E-04	1.62E-02
cg17101703	AAK1	2	Body	Open sea	65.99	3.41	68.86	4.05	-2.87	9.22E-05	2.62E-03
cg25487008	C21orf33;C21orf33	21	Body;Body	N_Shore	54.47	3.83	57.34	4.80	-2.87	6.03E-04	1.17E-02
cg02921923		11	Intergenic	S_Shelf	83.49	4.72	86.36	3.66	-2.87	9.29E-05	2.63E-03
cg12218389	CLCNKB	1	TSS200	Open sea	38.14	3.88	41.01	4.11	-2.87	2.31E-03	3.13E-02
cg04794183	HOXC4;HOXC6;HOXC5	12	TSS200;TSS200;TSS200	N_Shore	14.80	2.49	17.67	3.62	-2.87	5.13E-06	2.05E-04
cg13761843	ENG;ENG	9	Body;Body	Open sea	59.18	4.04	62.04	3.69	-2.87	2.41E-03	3.22E-02

cg22883125		8	Intergenic	S_Shore	19.47	3.41	22.33	3.03	-2.87	1.09E-04	3.01E-03
cg01310768	LYSMD3	5	5'UTR	N_Shore	62.40	4.32	65.27	3.83	-2.87	3.14E-03	3.86E-02
cg22361604		1	Intergenic	Open sea	67.07	4.49	69.93	4.56	-2.87	1.23E-03	1.99E-02
cg11785183	EHMT2;SLC44A4;EHMT2	6	Body;TSS1500;Body	Open sea	76.38	4.35	79.24	3.10	-2.86	3.90E-03	4.48E-02
cg12837744	SYNGAP1	6	1stExon	S_Shore	22.64	3.76	25.51	3.71	-2.86	3.52E-04	7.72E-03
cg23632752	LOC100133612	1	Body	Open sea	67.78	3.53	70.65	3.26	-2.86	2.86E-06	1.19E-04
cg09035079	RING1	6	3'UTR	N_Shore	69.82	3.55	72.68	2.77	-2.86	1.42E-04	3.74E-03
cg16515600	RSBN1	1	Body	Open sea	46.68	5.88	49.54	5.55	-2.86	3.10E-03	3.83E-02
cg17834632	METRNL	17	Body	S_Shore	50.29	2.67	53.16	3.19	-2.86	7.94E-05	2.30E-03
cg26102503	GNASAS;GNAS	20	TSS200;3'UTR	N_Shore	41.74	4.31	44.61	3.90	-2.86	2.17E-03	2.99E-02
cg17384897	KLHL29	2	5'UTR	S_Shore	25.89	5.86	28.75	4.99	-2.86	1.03E-03	1.76E-02
cg03841832	SLC2A9;SLC2A9	4	Body;Body	Open sea	66.65	3.92	69.51	4.53	-2.86	3.67E-04	7.97E-03
cg15499560	C14orf43;C14orf43	14	Body;Body	Open sea	79.01	4.11	81.87	3.16	-2.86	8.26E-04	1.48E-02
cg11385906	INTS9;INTS9;INTS9	8	Body;Body;Body	N_Shelf	64.85	4.38	67.72	4.03	-2.86	3.57E-03	4.22E-02
cg08872313	FRMD4B	3	Body	Open sea	14.49	3.59	17.35	4.60	-2.86	1.92E-03	2.75E-02
cg22479842	IGFBP2	2	TSS200	Island	11.25	1.91	14.11	2.33	-2.86	8.73E-08	4.37E-06
cg13436334	CDK17;CDK17	12	5'UTR;5'UTR	N_Shelf	72.99	5.54	75.85	4.50	-2.86	4.42E-03	4.89E-02
cg07273125		16	Intergenic	N_Shelf	12.96	3.69	15.82	5.27	-2.86	1.32E-03	2.10E-02
cg05611723		2	Intergenic	S_Shelf	27.55	4.05	30.42	4.11	-2.86	1.27E-03	2.04E-02
cg23320649	C3orf18	3	5'UTR	N_Shore	38.71	4.39	41.57	4.69	-2.86	1.24E-03	2.01E-02
cg05794310	VAC14	16	Body	Open sea	44.65	3.35	47.51	4.69	-2.86	3.13E-03	3.86E-02
cg02990598	C19orf34;CSNK1G2	19	TSS1500;5'UTR	Island	76.68	4.72	79.54	4.35	-2.86	2.98E-03	3.72E-02
cg26996458	EIF2C2;EIF2C2	8	Body;Body	Open sea	89.74	2.84	92.61	1.83	-2.86	1.26E-07	6.23E-06
cg03565777		12	Intergenic	Island	42.66	4.05	45.52	3.56	-2.86	1.56E-03	2.37E-02
cg20825416	PBX1	1	Body	S_Shore	86.90	3.18	89.76	1.93	-2.86	9.85E-07	4.43E-05
cg20902757		8	Intergenic	Island	35.04	2.75	37.91	3.42	-2.86	5.07E-04	1.02E-02
cg17758148	JMY	5	Body	Open sea	80.54	4.33	83.40	4.25	-2.86	2.30E-03	3.12E-02
cg10085305	ENPP7	17	1stExon	N_Shelf	49.09	3.77	51.95	3.66	-2.86	3.75E-03	4.37E-02
cg25096749		12	Intergenic	Open sea	74.70	4.50	77.56	4.13	-2.86	4.53E-03	4.97E-02
cg04821375	NEURL4;NEURL4	17	Body;Body	Island	45.97	3.03	48.83	3.57	-2.86	4.48E-04	9.31E-03
cg03738365		6	Intergenic	Open sea	59.25	3.18	62.11	4.30	-2.86	1.77E-03	2.60E-02
cg14891410	WIT1	11	Body	N_Shore	33.13	3.19	35.99	4.09	-2.86	1.67E-03	2.48E-02
cg04905173	GTF2E2	8	5'UTR	N_Shelf	52.98	3.17	55.84	2.77	-2.86	1.38E-04	3.66E-03
cg13997645	INSC;INSC	11	TSS200;Body	Island	13.51	2.51	16.37	2.42	-2.86	8.82E-08	4.42E-06
cg02137809	HEATR2	7	Body	Open sea	62.28	3.73	65.14	4.04	-2.86	2.47E-03	3.27E-02
cg24012811	GFRA1	10	TSS1500	S_Shore	10.49	2.38	13.35	3.23	-2.86	2.08E-05	7.23E-04
cg21527370	PPIL6;PPIL6	6	Body;Body	Open sea	47.91	5.25	50.77	4.16	-2.86	4.35E-03	4.83E-02
cg08381046		5	Intergenic	Open sea	60.51	8.33	63.37	7.80	-2.86	1.29E-03	2.07E-02
cg24706992		1	Intergenic	Open sea	73.95	3.10	76.81	3.35	-2.86	1.03E-04	2.87E-03
cg08780063	TRIM37;TRIM37	17	3'UTR;Body	Open sea	78.40	4.13	81.26	4.31	-2.86	2.68E-03	3.46E-02
cg00902104		10	Intergenic	Island	14.05	3.38	16.91	3.46	-2.86	5.46E-04	1.08E-02
cg02829706	TBX2	17	TSS200	Island	11.00	2.77	13.86	2.89	-2.86	8.69E-07	3.93E-05
cg14474520	FAM101A	12	5'UTR	S_Shore	53.83	4.04	56.68	4.26	-2.86	1.52E-03	2.32E-02
cg11496616	FAM190B	10	Body	Open sea	86.74	4.68	89.59	2.27	-2.86	1.08E-04	2.98E-03
cg00765144	MLLT10;MLLT10	10	Body;Body	Open sea	85.91	4.81	88.77	4.25	-2.86	1.92E-03	2.74E-02
cg00575896	HYAL2;HYAL2	3	Body;Body	N_Shore	66.67	4.12	69.53	3.61	-2.86	2.21E-03	3.03E-02
cg03046819	SHISA4;SHISA4	1	Body;Body	Open sea	35.18	4.34	38.04	4.28	-2.86	1.20E-03	1.96E-02
cg01803844		17	Intergenic	Open sea	68.08	4.90	70.93	4.53	-2.86	4.53E-03	4.97E-02
cg22759096	C6orf134	6	TSS1500	Open sea	55.94	3.45	58.80	3.36	-2.86	2.94E-04	6.69E-03
cg17383334	GABRR3	3	Body	Open sea	85.19	4.45	88.04	3.43	-2.86	9.50E-04	1.65E-02
cg18121989		8	Intergenic	Open sea	74.29	4.05	77.15	3.74	-2.86	2.99E-03	3.73E-02
cg09851981	GOLGB1	3	5'UTR	Open sea	82.78	4.26	85.64	3.56	-2.86	1.10E-04	3.04E-03
cg21850934		11	Intergenic	Open sea	84.87	5.09	87.73	3.69	-2.86	1.05E-03	1.77E-02

cg27054144	ENPP6	4	Body	S_Shelf	82.45	3.34	85.31	2.82	-2.86	9.81E-07	4.41E-05
cg19402413		1	Intergenic	Open sea	78.50	3.56	81.35	3.21	-2.86	3.26E-05	1.07E-03
cg09662034	TFAP2C	20	Body	Island	10.60	2.77	13.45	4.55	-2.86	7.89E-04	1.43E-02
cg07124642	NT5C3L	17	Body	N_Shore	53.56	5.16	56.41	4.26	-2.86	1.27E-03	2.04E-02
cg03697411	SNORD114-4;SNORD114-3	14	TSS1500;TSS1500	Open sea	45.36	3.68	48.21	4.09	-2.86	1.38E-03	2.17E-02
cg25469923		1	Intergenic	Open sea	61.20	4.74	64.06	4.44	-2.86	3.55E-03	4.21E-02
cg12100109	COG6;COG6;COG6	13	Body;3'UTR;Body	Open sea	85.51	3.79	88.37	3.01	-2.86	3.66E-03	4.29E-02
cg26423041	CDYL;CDYL	6	Body;Body	Open sea	67.19	3.19	70.05	3.66	-2.86	1.36E-04	3.60E-03
cg27598407	ATF7IP	12	5'UTR	Open sea	82.91	5.27	85.76	5.17	-2.86	3.85E-03	4.45E-02
cg04045009	TNNI3	19	Body	Island	9.47	2.29	12.33	3.41	-2.86	1.46E-05	5.29E-04
cg15012468	MFSD4	1	Body	S_Shore	73.55	4.23	76.40	3.96	-2.86	1.49E-03	2.30E-02
cg12945444		8	Intergenic	Island	12.65	3.39	15.50	4.60	-2.86	1.70E-03	2.52E-02
cg26572896		2	Intergenic	Open sea	63.53	5.63	66.39	5.55	-2.86	5.01E-04	1.01E-02
cg07775468		12	Intergenic	S_Shore	53.36	4.05	56.22	3.97	-2.86	3.17E-03	3.89E-02
cg15782845	CEP63;CEP63;CEP63;CEP63	3	3'UTR;Body;3'UTR;3'UTR	Open sea	69.77	5.06	72.62	5.21	-2.86	2.55E-03	3.35E-02
cg19744051		5	Intergenic	S_Shelf	63.92	4.02	66.78	3.54	-2.86	7.93E-04	1.44E-02
cg08614441	MCF2L;MCF2L;MCF2L	13	Body;5'UTR;1stExon	N_Shore	62.41	3.71	65.26	3.86	-2.85	1.59E-04	4.12E-03
cg12148129		2	Intergenic	Island	14.49	2.36	17.34	3.20	-2.85	7.65E-08	3.85E-06
cg13004587	SCGB3A1	5	Body	Island	6.06	2.56	8.92	4.38	-2.85	1.25E-04	3.35E-03
cg01477959	TNFSF11	13	TSS200	Open sea	74.84	3.94	77.70	3.99	-2.85	8.93E-04	1.57E-02
cg03643423	MAP2K4	17	Body	Open sea	83.72	4.58	86.57	3.29	-2.85	2.67E-04	6.22E-03
cg00146928	A2M	12	TSS1500	Open sea	79.96	4.35	82.81	3.72	-2.85	1.21E-03	1.98E-02
cg14665592	RBMS1;RBMS1	2	Body;Body	Open sea	85.34	3.52	88.19	3.44	-2.85	5.31E-05	1.64E-03
cg07756477	LMAN1L	15	TSS200	Open sea	60.63	3.87	63.49	3.83	-2.85	4.36E-04	9.11E-03
cg23881725	DLEC1;DLEC1	3	TSS200;TSS200	Island	28.98	4.87	31.84	5.40	-2.85	1.15E-03	1.90E-02
cg00389463	ICAM4;ICAM4;ICAM4	19	TSS200;TSS200;TSS200	Island	20.17	3.78	23.03	3.91	-2.85	7.76E-05	2.26E-03
cg26125625	SLC12A8	3	Body	Island	32.86	5.58	35.71	5.45	-2.85	1.04E-03	1.77E-02
cg27533454		16	Intergenic	Open sea	66.66	5.53	69.51	5.28	-2.85	4.02E-03	4.58E-02
cg03944089	SGK1;SGK1;SGK1;SGK1	6	TSS1500;Body;Body;TSS1500	N_Shore	11.73	2.62	14.58	3.54	-2.85	2.00E-04	4.95E-03
cg11839020	LRRC8D;LRRC8D	1	5'UTR;5'UTR	S_Shore	69.90	4.00	72.75	4.68	-2.85	4.33E-05	1.37E-03
cg00346208	VWA5B1	1	Body	Island	23.99	4.48	26.84	4.84	-2.85	2.09E-04	5.11E-03
cg27422141	PCDH9;PCDH9	13	5'UTR;5'UTR	N_Shore	15.74	4.70	18.59	4.29	-2.85	9.60E-04	1.66E-02
cg01168115	EHMT2;EHMT2	6	Body;Body	S_Shelf	79.67	3.73	82.52	3.23	-2.85	1.02E-03	1.74E-02
cg20218280		7	Intergenic	N_Shore	24.62	2.91	27.47	2.63	-2.85	5.66E-05	1.73E-03
cg01567672	MXD4	4	Body	N_Shore	67.72	3.96	70.57	3.99	-2.85	2.23E-04	5.40E-03
cg14030055	MIR130B;MIR301B	22	TSS1500;TSS1500	Island	14.74	4.66	17.59	5.73	-2.85	3.99E-04	8.50E-03
cg06867285		7	Intergenic	Open sea	82.22	3.34	85.07	4.18	-2.85	2.97E-04	6.74E-03
cg08771171	CTNNA1;LRRTM2	5	Body;TSS200	Open sea	75.80	4.01	78.65	3.25	-2.85	9.95E-04	1.70E-02
cg18936304	JAKMIP3	10	Body	S_Shore	70.15	3.17	73.01	3.41	-2.85	1.58E-04	4.08E-03
cg24362829		1	Intergenic	N_Shore	64.81	4.25	67.67	4.08	-2.85	2.83E-04	6.49E-03
cg09057080	RPUSD1;CHTF18	16	Body;TSS1500	Island	79.25	3.70	82.10	3.67	-2.85	1.88E-03	2.70E-02
cg03303515	TTC7A	2	Body	Open sea	32.59	3.05	35.45	2.81	-2.85	4.98E-05	1.55E-03
cg00293981	LYPD1;NCKAP5;NCKAP5	2	TSS1500;3'UTR;3'UTR	S_Shore	73.51	4.48	76.36	4.76	-2.85	2.44E-03	3.25E-02
cg27530136	SH2D2A;SH2D2A;SH2D2A;SH2D2A;SH	1	Body;Body;Body;Body;Body	N_Shore	52.98	4.04	55.83	3.68	-2.85	5.95E-04	1.16E-02
cg26330479	MIR429	1	TSS200	S_Shelf	64.84	4.41	67.69	5.22	-2.85	2.14E-03	2.97E-02
cg01269044		22	Intergenic	Island	80.59	4.00	83.45	3.96	-2.85	4.26E-03	4.76E-02
cg13813710		6	Intergenic	Open sea	80.02	4.45	82.87	3.45	-2.85	8.42E-04	1.51E-02
cg00093433		16	Intergenic	N_Shore	40.34	4.12	43.19	3.67	-2.85	1.98E-03	2.81E-02
cg23010765	C6orf103	6	Body	S_Shelf	47.63	4.89	50.48	4.27	-2.85	2.48E-03	3.29E-02
cg12573289	CCM2;CCM2;CCM2;CCM2;CCM2	7	Body;Body;Body;Body;Body	Open sea	58.47	3.90	61.32	3.83	-2.85	3.20E-04	7.17E-03
cg13013841	EVPLL	17	TSS200	Open sea	39.19	3.70	42.04	4.42	-2.85	2.07E-03	2.89E-02
cg13208159	DIRAS3	1	TSS200	N_Shore	38.73	4.13	41.58	3.39	-2.85	2.73E-03	3.51E-02
cg06931905	PLAT;PLAT	8	Body;Body	Open sea	26.79	2.91	29.64	3.85	-2.85	7.61E-04	1.40E-02

cg21822834	AKT1;AKT1;AKT1		14	3'UTR;3'UTR;3'UTR	N_Shelf	80.82	2.79	83.67	2.53	-2.85	2.68E-06	1.12E-04
cg04505076	APOB		2	Body	Open sea	83.34	3.90	86.19	3.05	-2.85	2.91E-03	3.67E-02
cg00524443	HOXB3		17	5'UTR	N_Shelf	20.23	3.29	23.08	4.75	-2.85	4.57E-03	4.99E-02
cg02867250	PSORS1C1;PSORS1C2		6	3'UTR;TSS1500	Open sea	81.48	3.54	84.33	3.28	-2.85	1.54E-04	3.99E-03
cg27417677	MESTIT1;MEST;MEST;MEST		7	Body;TSS1500;5'UTR;TSS1500	Island	48.02	3.69	50.87	3.82	-2.85	7.12E-04	1.33E-02
cg08119325	TNK2;TNK2		3	Body;Body	N_Shore	69.84	4.00	72.69	3.14	-2.85	1.47E-03	2.27E-02
cg25221254	ACER1		19	1stExon	Open sea	84.94	4.73	87.79	3.15	-2.85	3.25E-03	3.96E-02
cg06786064			7	Intergenic	Island	14.99	2.51	17.84	2.67	-2.85	5.18E-06	2.07E-04
cg17087314			10	Intergenic	N_Shore	58.28	4.02	61.13	4.79	-2.85	2.19E-03	3.01E-02
cg04935521	AATK		17	Body	N_Shelf	80.31	3.94	83.16	3.11	-2.85	1.87E-04	4.69E-03
cg07355069	HMGCS1;HMGCS1		5	3'UTR;3'UTR	Open sea	91.73	2.07	94.58	1.30	-2.85	1.25E-11	7.40E-10
cg05965288			16	Intergenic	Island	16.41	4.48	19.26	4.90	-2.85	3.89E-03	4.48E-02
cg16950726	NLGN2		17	Body	N_Shelf	71.44	4.06	74.29	3.99	-2.85	2.58E-04	6.05E-03
cg24630419	ESRRG		1	TSS1500	Island	18.01	3.36	20.86	4.31	-2.85	2.98E-03	3.73E-02
cg01751337			4	Intergenic	N_Shore	65.68	4.12	68.53	3.86	-2.85	3.66E-03	4.30E-02
cg20547455			14	Intergenic	Island	15.12	2.09	17.97	3.02	-2.85	5.91E-07	2.74E-05
cg27223827	FHL2;FHL2		2	1stExon;5'UTR	Open sea	51.87	5.60	54.72	5.44	-2.85	3.29E-03	4.00E-02
cg14207728	TCTEX1D1		1	TSS1500	N_Shore	30.59	5.90	33.44	7.36	-2.85	3.26E-03	3.96E-02
cg01486183			14	Intergenic	Open sea	81.51	4.33	84.36	4.44	-2.85	5.09E-04	1.03E-02
cg23991845	PRDM5		4	Body	Open sea	70.58	4.99	73.42	4.85	-2.85	2.95E-03	3.70E-02
cg16169950	PLEKHG4B		5	Body	Island	81.39	5.73	84.23	5.09	-2.85	4.45E-03	4.91E-02
cg14823740	SYNGAP1		6	Body	N_Shelf	71.63	4.24	74.47	4.08	-2.85	1.36E-03	2.14E-02
cg14225195	CACNG4		17	Body	S_Shelf	53.90	4.04	56.75	4.02	-2.85	3.05E-03	3.79E-02
cg02442496	CD37;CD37		19	TSS1500;TSS1500	N_Shelf	44.24	2.62	47.09	2.19	-2.85	1.03E-05	3.88E-04
cg15270813	MMP24		20	TSS1500	N_Shore	27.31	5.24	30.16	4.46	-2.85	3.94E-03	4.52E-02
cg16376447	ISCA1L		5	3'UTR	Open sea	76.21	4.88	79.06	3.99	-2.85	1.75E-03	2.57E-02
cg27001643	PIAS4		19	Body	N_Shore	58.32	3.37	61.17	3.36	-2.85	3.23E-04	7.21E-03
cg10010004	KCTD19;LRRC36		16	TSS1500;Body	Open sea	48.80	2.97	51.65	3.55	-2.85	1.86E-04	4.67E-03
cg18347426		03-Mar	5	5'UTR	Open sea	70.66	3.99	73.51	4.59	-2.85	7.81E-04	1.42E-02
cg09578794			14	Intergenic	Island	46.79	3.99	49.63	3.97	-2.84	5.60E-04	1.10E-02
cg26617929			16	Intergenic	Open sea	90.55	3.49	93.39	2.28	-2.84	4.84E-05	1.51E-03
cg00831726			3	Intergenic	Open sea	76.39	4.13	79.23	4.39	-2.84	1.88E-04	4.70E-03
cg18887005			22	Intergenic	Island	87.16	3.93	90.00	2.76	-2.84	5.15E-05	1.59E-03
cg24831862	COX7B2;COX7B2		4	1stExon;5'UTR	Open sea	80.09	3.58	82.94	3.35	-2.84	2.35E-04	5.62E-03
cg17906168	EFNB2		13	3'UTR	N_Shore	77.41	3.76	80.26	3.52	-2.84	3.90E-03	4.49E-02
cg02573566	S100A13;S100A13;S100A13;S100A13;		1	5'UTR;1stExon;5'UTR;5'UTR;TSS1500;5'UT	Open sea	21.98	3.75	24.83	3.81	-2.84	3.60E-03	4.25E-02
cg15520279	HOXD8;HOXD8		2	5'UTR;1stExon	Island	11.98	3.06	14.82	3.93	-2.84	1.50E-05	5.42E-04
cg21022002	ENGASE		17	Body	Open sea	37.35	4.05	40.19	4.82	-2.84	4.97E-04	1.01E-02
cg01511806			16	Intergenic	Island	70.97	4.20	73.81	4.24	-2.84	1.55E-03	2.36E-02
cg07201319			6	Intergenic	Open sea	53.33	5.63	56.18	6.13	-2.84	2.57E-03	3.37E-02
cg13142374	FLNB;FLNB;FLNB;FLNB		3	Body;Body;Body;Body	Open sea	39.10	3.11	41.95	4.15	-2.84	1.16E-03	1.92E-02
cg23261107			7	Intergenic	Open sea	23.01	5.09	25.85	5.01	-2.84	1.28E-03	2.06E-02
cg19779211	KCNQ1		11	TSS1500	Island	19.51	3.44	22.36	3.59	-2.84	3.81E-04	8.19E-03
cg13933293			3	Intergenic	Island	47.29	4.48	50.13	4.16	-2.84	1.22E-03	1.98E-02
cg13832201	MAPK7;MAPK7;MAPK7;MAPK7		17	Body;Body;5'UTR;Body	Island	45.69	3.50	48.53	3.45	-2.84	2.38E-03	3.20E-02
cg05380127	NOTCH4		6	Body	Open sea	73.13	3.73	75.97	3.45	-2.84	3.29E-04	7.32E-03
cg17206420	SLC30A3		2	Body	N_Shelf	52.88	4.91	55.72	5.03	-2.84	4.11E-03	4.65E-02
cg10541755	EIF5A2		3	5'UTR	Island	17.43	3.98	20.27	4.07	-2.84	2.03E-04	5.00E-03
cg25326952	PDIA4		7	Body	Open sea	67.62	3.42	70.47	2.68	-2.84	1.35E-04	3.58E-03
cg06834712	FBRSL1		12	Body	N_Shore	78.17	4.14	81.01	3.66	-2.84	3.66E-03	4.29E-02
cg19056004	LRRC23;ENO2;LRRC23;LRRC23		12	3'UTR;TSS1500;3'UTR;3'UTR	Island	29.08	5.19	31.92	4.28	-2.84	2.26E-03	3.08E-02
cg25390440	GYPC;GYPC		2	TSS1500;TSS1500	N_Shore	12.46	2.81	15.31	4.29	-2.84	2.59E-03	3.39E-02
cg08976874			11	Intergenic	Open sea	76.43	5.46	79.27	4.71	-2.84	1.13E-03	1.87E-02

cg08425070	SRRM3	7	5'UTR	Island	51.02	4.07	53.86	4.20	-2.84	2.47E-03	3.28E-02
cg06022942	FLJ45983;GATA3;GATA3;FLJ45983	10	TSS200;TSS1500;TSS1500;TSS200	Island	7.99	2.66	10.83	5.11	-2.84	3.32E-03	4.02E-02
cg18147104		14	Intergenic	Island	86.88	4.09	89.72	4.04	-2.84	7.30E-04	1.35E-02
cg05928448		18	Intergenic	N_Shore	18.99	3.77	21.83	3.24	-2.84	5.04E-04	1.02E-02
cg19764006		4	Intergenic	Open sea	13.78	3.97	16.62	5.49	-2.84	4.29E-03	4.79E-02
cg18343556	C3orf45	3	TSS200	S_Shore	59.23	4.73	62.07	4.66	-2.84	6.57E-04	1.25E-02
cg09142858	FAM92B;FAM92B	16	5'UTR;1stExon	Open sea	77.91	4.30	80.74	3.13	-2.84	4.94E-04	1.00E-02
cg16872595	PQLC1;PQLC1;PQLC1	18	Body;Body;Body	N_Shore	21.41	3.26	24.25	2.83	-2.84	3.98E-04	8.49E-03
cg22157398	MBOAT2	2	Body	Open sea	48.99	3.72	51.83	4.27	-2.84	5.87E-04	1.15E-02
cg24870729		1	Intergenic	Open sea	65.66	5.84	68.50	4.96	-2.84	3.56E-03	4.21E-02
cg08325842	ZFP91;ZFP91-CNTF	11	Body;Body	Open sea	82.54	3.63	85.38	2.94	-2.84	2.69E-04	6.25E-03
cg13315471	TMEM214;TMEM214	2	Body;Body	S_Shelf	75.15	2.90	77.99	3.32	-2.84	7.49E-05	2.20E-03
cg08293324	THBS1	15	TSS1500	N_Shore	15.93	4.07	18.77	4.57	-2.84	4.09E-03	4.63E-02
cg27185793	ACAP3	1	Body	N_Shelf	67.76	4.35	70.60	4.28	-2.84	2.85E-03	3.61E-02
cg15965241	P2RX7	12	TSS200	Open sea	29.12	2.74	31.96	2.80	-2.84	7.14E-05	2.11E-03
cg01068332		8	Intergenic	Open sea	70.09	6.20	72.93	5.67	-2.84	2.05E-03	2.87E-02
cg09621472	NAA50	3	3'UTR	Open sea	90.65	2.04	93.48	1.53	-2.84	3.88E-11	2.27E-09
cg03972852	SBF2	11	Body	N_Shore	66.45	3.58	69.29	3.15	-2.84	4.23E-04	8.90E-03
cg24850535	SLC8A2	19	Body	N_Shelf	18.47	4.20	21.30	4.74	-2.84	4.45E-03	4.91E-02
cg03228209	KIAA1688	8	Body	Island	71.89	4.25	74.73	3.14	-2.84	1.16E-03	1.91E-02
cg09871424	PBX1	1	Body	Open sea	87.07	3.00	89.90	2.24	-2.84	1.31E-08	6.94E-07
cg09646197	ZNF467	7	Body	Island	93.00	4.46	95.84	2.32	-2.84	8.93E-05	2.55E-03
cg09037726	ART3;ART3;ART3	4	TSS1500;TSS1500;5'UTR	Open sea	83.96	4.12	86.79	3.44	-2.84	1.61E-03	2.42E-02
cg25316749	C11orf93;C11orf92	11	TSS1500;5'UTR	N_Shore	52.27	5.20	55.11	4.19	-2.84	2.90E-03	3.65E-02
cg10035270		16	Intergenic	Island	61.54	4.41	64.38	3.28	-2.84	2.46E-04	5.83E-03
cg00604202		6	Intergenic	Island	16.99	2.86	19.82	3.01	-2.84	6.32E-06	2.48E-04
cg17054006	PDE1A;PDE1A	2	Body;Body	Open sea	76.39	4.80	79.22	5.11	-2.84	1.06E-03	1.78E-02
cg15339981	NRIP2	12	Body	Open sea	67.16	3.72	70.00	3.76	-2.84	2.26E-03	3.08E-02
cg06004033	NT5DC3	12	Body	Open sea	64.74	5.61	67.57	4.45	-2.84	7.54E-04	1.39E-02
cg08276125	SKIV2L	6	Body	N_Shelf	65.79	3.39	68.62	3.80	-2.84	8.66E-04	1.54E-02
cg23042230	STAU2;STAU2;STAU2;STAU2;STAU2;S	8	Body;Body;Body;Body;Body;Body;5'UTR	Open sea	85.75	4.65	88.58	3.49	-2.84	1.65E-03	2.46E-02
cg07622957	CLCN7;CLCN7	16	Body;Body	S_Shore	87.01	4.68	89.85	3.59	-2.84	2.18E-03	3.01E-02
cg12004787	FGF19	11	TSS1500	Island	33.67	5.31	36.51	5.52	-2.84	1.13E-03	1.88E-02
cg15405572	NAV3	12	TSS1500	Open sea	11.66	3.44	14.50	4.44	-2.84	3.12E-03	3.84E-02
cg11577355	AFF3;AFF3	2	TSS1500;5'UTR	S_Shore	62.18	3.31	65.01	3.72	-2.84	1.28E-03	2.06E-02
cg03608583		2	Intergenic	N_Shelf	77.82	5.59	80.65	5.23	-2.84	3.64E-03	4.28E-02
cg02776448	NEU1	6	Body	N_Shore	75.70	3.52	78.53	3.13	-2.84	9.16E-04	1.61E-02
cg05569742	PRDM6	5	Body	N_Shore	19.86	3.38	22.69	3.23	-2.84	1.84E-04	4.62E-03
cg20824189		7	Intergenic	Island	19.56	4.02	22.40	4.21	-2.84	2.55E-03	3.35E-02
cg07292311		12	Intergenic	Open sea	51.84	3.59	54.67	4.18	-2.84	1.23E-03	2.00E-02
cg15557036		17	Intergenic	Island	19.33	3.22	22.17	4.43	-2.83	2.14E-03	2.96E-02
cg25617328		5	Intergenic	N_Shelf	29.59	3.00	32.43	3.77	-2.83	4.45E-04	9.25E-03
cg22417733	FBXO5;FBXO5	6	5'UTR;Body	Island	45.76	4.81	48.60	4.84	-2.83	1.27E-03	2.04E-02
cg21310090	PLTP;PLTP	20	Body;Body	N_Shore	45.69	4.15	48.52	4.47	-2.83	3.00E-03	3.74E-02
cg22704780	LIPE	19	Body	S_Shore	64.52	3.87	67.36	4.24	-2.83	2.07E-04	5.07E-03
cg10766792	WBP2NL	22	3'UTR	Open sea	85.39	4.15	88.22	3.87	-2.83	2.63E-03	3.42E-02
cg10094078	APC2	19	Body	Island	21.09	3.30	23.92	4.52	-2.83	3.35E-04	7.42E-03
cg20303028	DCTN1;DCTN1	2	TSS200;TSS200	Open sea	60.77	4.68	63.60	4.71	-2.83	2.39E-03	3.20E-02
cg27268574	FCHO1;FCHO1	19	TSS1500;TSS1500	N_Shore	67.94	4.95	70.77	5.32	-2.83	4.46E-03	4.92E-02
cg20782286		6	Intergenic	Island	56.63	3.57	59.46	3.73	-2.83	1.18E-03	1.93E-02
cg09132790	ZNF423	16	Body	Open sea	69.29	3.39	72.13	3.39	-2.83	5.26E-04	1.05E-02
cg07720334	DNMT3A;DNMT3A;DNMT3A	2	Body;Body;Body	Open sea	78.49	4.80	81.33	3.75	-2.83	1.15E-03	1.90E-02
cg25422797	CASKIN2;CASKIN2	17	Body;TSS200	Open sea	78.70	3.27	81.53	3.50	-2.83	4.67E-04	9.61E-03

cg05340269	ODZ4	11	5'UTR	Open sea	56.61	6.74	59.45	5.47	-2.83	4.00E-03	4.57E-02
cg26880216		17	Intergenic	Open sea	53.52	3.57	56.36	4.03	-2.83	2.59E-03	3.39E-02
cg22890130	FNDC3B;FNDC3B	3	Body;Body	Open sea	42.44	5.98	45.27	8.12	-2.83	2.98E-03	3.73E-02
cg15828915	ITPR2	12	Body	Open sea	85.77	3.90	88.61	3.20	-2.83	7.29E-04	1.35E-02
cg04157587		7	Intergenic	N_Shore	47.36	4.18	50.19	4.21	-2.83	1.51E-03	2.31E-02
cg09696159		3	Intergenic	N_Shore	29.18	3.76	32.01	3.77	-2.83	9.90E-04	1.70E-02
cg03420846	PUM1;PUM1	1	Body;Body	Open sea	73.61	4.00	76.44	4.34	-2.83	1.30E-03	2.08E-02
cg19314992	ACER3	11	Body	Open sea	40.74	4.35	43.58	4.76	-2.83	4.29E-03	4.79E-02
cg24170248	SSC5D	19	Body	Island	45.93	4.20	48.76	4.96	-2.83	2.27E-03	3.09E-02
cg00912738		8	Intergenic	N_Shore	45.92	4.83	48.75	4.71	-2.83	3.28E-03	3.98E-02
cg02242681		2	Intergenic	Island	38.03	3.60	40.86	4.35	-2.83	1.21E-03	1.97E-02
cg18052511	CCR5;CCR5	3	TSS1500;TSS1500	Open sea	36.56	5.08	39.39	6.14	-2.83	1.62E-03	2.43E-02
cg02128625		12	Intergenic	Open sea	84.65	4.01	87.48	3.49	-2.83	2.85E-04	6.53E-03
cg01803704	SNORD114-26;SNORD114-27	14	TSS200;TSS1500	Open sea	81.63	5.42	84.46	4.17	-2.83	2.32E-03	3.14E-02
cg24576050	PCDHGA4;PCDHGA11;PCDHGA11;PCD	5	Body;Body;Body;Body;Body;Body;Body;1s	Island	20.65	3.04	23.49	3.37	-2.83	1.37E-04	3.64E-03
cg25193885	SHANK2;SHANK2	11	Body;Body	N_Shelf	83.67	4.23	86.51	2.39	-2.83	2.78E-03	3.55E-02
cg08513123	TRIM10;TRIM10	6	Body;Body	Open sea	78.13	3.91	80.96	3.96	-2.83	3.06E-03	3.80E-02
cg27496615	HOXB4	17	Body	Island	30.84	3.26	33.67	3.27	-2.83	1.34E-03	2.12E-02
cg09118558	NAV2;NAV2;NAV2;NAV2	11	Body;Body;Body;Body	Open sea	15.73	3.29	18.56	4.09	-2.83	3.37E-04	7.46E-03
cg03450046		11	Intergenic	S_Shelf	72.86	4.99	75.69	4.29	-2.83	5.31E-04	1.06E-02
cg08534830		18	Intergenic	Island	68.54	3.80	71.37	3.84	-2.83	2.46E-03	3.27E-02
cg01761729	LIMS2;LIMS2;LIMS2;LIMS2	2	Body;Body;Body;5'UTR	N_Shelf	65.23	3.87	68.06	3.81	-2.83	1.13E-03	1.88E-02
cg21179618	NOTCH1	9	Body	N_Shore	65.08	3.46	67.91	3.55	-2.83	4.18E-04	8.81E-03
cg05208196	SLC26A1;IDUA;SLC26A1;SLC26A1	4	Body;Body;Body;Body	Island	86.43	4.95	89.26	3.03	-2.83	2.32E-03	3.14E-02
cg03365354	MCAM	11	Body	Island	18.36	3.96	21.19	3.99	-2.83	3.40E-04	7.51E-03
cg04143348	TMEM20;TMEM20	10	Body;Body	S_Shelf	86.70	3.60	89.53	2.86	-2.83	3.41E-05	1.11E-03
cg20155035		1	Intergenic	Open sea	17.34	3.63	20.17	3.75	-2.83	9.41E-04	1.64E-02
cg19615731	DIDO1;DIDO1;DIDO1;DIDO1	20	3'UTR;3'UTR;Body;Body	Open sea	73.15	5.08	75.98	4.45	-2.83	4.26E-03	4.76E-02
cg09661865		17	Intergenic	Open sea	77.31	4.84	80.14	5.02	-2.83	3.11E-03	3.83E-02
cg24154326	XKR7	20	Body	S_Shore	32.36	3.74	35.19	4.24	-2.83	6.08E-04	1.18E-02
cg22622440	ALKBH5	17	Body	Island	20.74	3.87	23.57	3.66	-2.83	9.14E-04	1.60E-02
cg06044899	TMSL3;TMSL3;FAM190A	4	1stExon;5'UTR;Body	Island	52.44	2.51	55.27	2.67	-2.83	4.62E-05	1.45E-03
cg21889604	BCR;BCR	22	Body;Body	S_Shelf	88.40	3.20	91.23	2.11	-2.83	4.78E-05	1.49E-03
cg13607709	PTH1R	3	Body	Island	37.93	4.62	40.76	5.06	-2.83	9.31E-04	1.63E-02
cg24393470	UTRN	6	Body	Open sea	78.23	4.43	81.06	5.00	-2.83	2.15E-03	2.97E-02
cg01794501	PCP4L1	1	Body	Island	22.45	3.44	25.28	4.10	-2.83	3.91E-03	4.50E-02
cg21626848	SC65;FKBP10;FKBP10	17	TSS1500;5'UTR;1stExon	S_Shore	52.57	4.40	55.40	4.79	-2.83	2.56E-03	3.36E-02
cg25738688		12	Intergenic	Open sea	82.93	4.89	85.76	4.33	-2.83	3.18E-03	3.90E-02
cg16934235	EXPH5;EXPH5	11	TSS200;Body	Open sea	76.38	3.98	79.21	3.51	-2.83	3.46E-03	4.14E-02
cg11309456	RAD9B	12	Body	S_Shelf	60.15	4.39	62.98	3.18	-2.83	1.30E-03	2.08E-02
cg00259097	VAC14	16	Body	Open sea	82.66	3.44	85.49	2.74	-2.83	1.19E-03	1.95E-02
cg26185056	PAPSS2;PAPSS2	10	Body;Body	S_Shelf	84.56	3.83	87.39	3.04	-2.83	3.05E-04	6.88E-03
cg07918341	GFAP;GFAP	17	1stExon;1stExon	S_Shelf	62.49	4.31	65.32	4.45	-2.83	3.73E-03	4.35E-02
cg16127617	TNFAIP2	14	Body	S_Shore	75.38	5.21	78.21	4.24	-2.83	1.40E-03	2.19E-02
cg21899283	C12orf69;C12orf60	12	5'UTR;5'UTR	S_Shelf	68.35	5.57	71.18	6.01	-2.83	3.30E-03	4.00E-02
cg25684349	BTBD11	12	Body	Open sea	55.33	5.76	58.16	4.92	-2.83	4.71E-04	9.67E-03
cg13853186	DMRT3	9	TSS1500	N_Shore	15.00	3.20	17.83	3.11	-2.83	3.48E-04	7.65E-03
cg18100753		8	Intergenic	S_Shore	60.98	4.25	63.81	4.89	-2.83	3.28E-03	3.98E-02
cg16534163	PLSCR2;PLSCR2	3	1stExon;5'UTR	Open sea	87.83	3.53	90.66	2.69	-2.83	9.73E-06	3.67E-04
cg17520314	NPLOC4	17	TSS200	S_Shore	12.50	2.07	15.33	2.88	-2.83	1.28E-06	5.67E-05
cg09354381		3	Intergenic	Island	25.48	3.69	28.31	4.59	-2.83	3.47E-04	7.62E-03
cg15519237	PRX;PRX	19	Body;Body	S_Shore	73.77	3.40	76.60	3.84	-2.83	1.72E-04	4.39E-03
cg26521139	AGAP1;AGAP1	2	Body;Body	Open sea	71.06	4.60	73.89	3.75	-2.83	8.37E-04	1.50E-02

cg25594100	FOXK1	7	Body	Island	70.92	4.07	73.74	3.81	-2.83	1.90E-03	2.73E-02
cg10954938	NPLOC4	17	Body	Open sea	85.68	3.89	88.51	2.52	-2.83	2.40E-06	1.02E-04
cg12728097		1	Intergenic	N_Shelf	74.71	4.00	77.54	4.07	-2.83	1.75E-03	2.57E-02
cg11458974	HMOX1	22	TSS1500	N_Shore	79.57	3.84	82.39	4.34	-2.83	2.40E-03	3.22E-02
cg02313495	BMF;BMF;BMF;BMF	15	Body;Body;1stExon;1stExon	N_Shelf	65.97	5.18	68.80	5.48	-2.83	4.40E-04	9.18E-03
cg16194437	CASD1	7	3'UTR	Open sea	83.97	3.71	86.79	2.98	-2.83	2.39E-04	5.69E-03
cg07584563		1	Intergenic	N_Shore	51.69	4.28	54.51	4.97	-2.83	1.61E-03	2.42E-02
cg20046608	TBX2	17	TSS200	Island	14.59	2.58	17.41	2.74	-2.83	1.00E-05	3.78E-04
cg06516769	PCBP4;PCBP4;PCBP4	3	5'UTR;5'UTR;TSS1500	Open sea	74.59	3.97	77.41	3.35	-2.83	2.67E-04	6.21E-03
cg23549774	TBCD	17	Body	S_Shore	79.28	4.61	82.11	3.87	-2.83	1.39E-03	2.19E-02
cg19255722	SCN10A	3	Body	Open sea	72.39	4.41	75.22	4.43	-2.82	6.24E-04	1.20E-02
cg17862667	DACT3	19	Body	N_Shore	19.61	3.69	22.43	4.37	-2.82	6.07E-05	1.84E-03
cg22727641	FBXO43;FBXO43;FBXO43;FBXO43	8	1stExon;5'UTR;1stExon;5'UTR	Island	15.49	3.76	18.32	4.82	-2.82	1.46E-03	2.26E-02
cg23646820	GPR137B	1	TSS1500	N_Shore	28.33	3.74	31.16	4.78	-2.82	4.00E-03	4.56E-02
cg07866212		8	Intergenic	Open sea	23.30	4.41	26.13	4.22	-2.82	5.05E-04	1.02E-02
cg14727650	11-Sep	4	Body	Open sea	71.37	3.89	74.19	2.24	-2.82	7.88E-04	1.43E-02
cg00511167	CEP63;CEP63;CEP63;CEP63	3	5'UTR;5'UTR;5'UTR;5'UTR	S_Shore	87.27	3.38	90.10	2.27	-2.82	9.67E-06	3.65E-04
cg08707476		1	Intergenic	N_Shelf	60.75	4.05	63.57	4.20	-2.82	4.20E-03	4.71E-02
cg08244656	OR13A1	10	TSS1500	Open sea	87.27	4.08	90.10	2.40	-2.82	2.40E-04	5.71E-03
cg17484267		10	Intergenic	N_Shelf	57.34	3.53	60.17	3.02	-2.82	3.44E-04	7.57E-03
cg23996349		8	Intergenic	Open sea	31.51	4.00	34.34	3.98	-2.82	6.68E-04	1.27E-02
cg18943383	HIST1H3H	6	1stExon	Island	5.27	2.70	8.10	5.88	-2.82	2.24E-03	3.06E-02
cg04043052	FAM110B	8	5'UTR	N_Shore	72.79	4.61	75.62	3.86	-2.82	3.93E-03	4.51E-02
cg07027513	B4GALT6	18	TSS1500	S_Shore	42.37	4.86	45.20	4.87	-2.82	3.38E-04	7.46E-03
cg00756648	FBXO46	19	Body	Island	79.16	4.13	81.99	4.04	-2.82	3.47E-03	4.14E-02
cg01274028	PC;PC;PC	11	Body;Body;Body	S_Shelf	74.33	3.71	77.15	3.63	-2.82	7.08E-04	1.32E-02
cg21249214		8	Intergenic	Open sea	80.00	4.81	82.82	3.96	-2.82	1.77E-03	2.60E-02
cg26534460	ABCA12;ABCA12	2	TSS200;Body	Open sea	61.99	4.46	64.81	5.52	-2.82	2.27E-03	3.09E-02
cg23165541	DAPK2	15	5'UTR	Island	45.97	4.33	48.79	4.34	-2.82	6.44E-04	1.23E-02
cg08767182		5	Intergenic	Island	18.69	3.18	21.52	3.34	-2.82	1.66E-04	4.24E-03
cg04583074	LDLRAD3	11	Body	Open sea	77.97	3.85	80.79	5.05	-2.82	2.41E-03	3.22E-02
cg02183001	NRP2;NRP2;NRP2;NRP2;NRP2;NRP2	2	Body;Body;Body;Body;Body;Body	S_Shore	71.73	3.67	74.55	3.90	-2.82	2.00E-03	2.83E-02
cg12204423	PHF17;PHF17	4	5'UTR;5'UTR	Island	5.35	1.27	8.17	1.73	-2.82	8.96E-13	5.49E-11
cg11511777	WNT6	2	Body	S_Shore	38.74	4.85	41.56	4.37	-2.82	5.48E-04	1.09E-02
cg09144769	AP2B1;AP2B1	17	3'UTR;3'UTR	Open sea	81.12	4.01	83.94	2.88	-2.82	3.29E-04	7.31E-03
cg24761542	TSG101	11	TSS1500	S_Shore	16.48	2.76	19.30	4.56	-2.82	2.97E-03	3.72E-02
cg08425508		12	Intergenic	Open sea	58.19	3.18	61.01	4.32	-2.82	1.03E-03	1.75E-02
cg09030672	HSF4;HSF4;FBXL8	16	TSS200;TSS200;Body	Island	26.09	4.90	28.92	4.59	-2.82	2.28E-03	3.10E-02
cg20456243	SPEG	2	Body	S_Shelf	49.57	5.58	52.39	5.58	-2.82	2.64E-03	3.43E-02
cg16656493	PTCRA	6	Body	Island	72.83	4.10	75.65	3.64	-2.82	6.46E-04	1.23E-02
cg13491304	JAK2	9	Body	N_Shelf	77.78	4.11	80.60	3.34	-2.82	8.50E-04	1.52E-02
cg18235000	PNMA2	8	TSS1500	Island	15.34	3.58	18.16	4.16	-2.82	4.37E-04	9.14E-03
cg13770865	SGPL1	10	TSS1500	N_Shore	74.57	4.09	77.39	3.06	-2.82	3.77E-03	4.38E-02
cg17720390		4	Intergenic	S_Shore	73.13	3.82	75.95	4.07	-2.82	7.64E-04	1.40E-02
cg25340121		19	Intergenic	N_Shore	78.98	3.96	81.80	2.77	-2.82	9.29E-04	1.62E-02
cg13672342	CIAO1;TMEM127	2	TSS1500;Body	N_Shore	45.76	4.52	48.58	5.22	-2.82	2.43E-03	3.24E-02
cg17021798	FAM20C	7	Body	S_Shore	84.84	3.82	87.66	3.16	-2.82	2.29E-03	3.11E-02
cg25374494		3	Intergenic	Open sea	81.33	3.75	84.15	3.00	-2.82	1.24E-04	3.34E-03
cg21650737	ERICH1	8	Body	Open sea	80.87	4.51	83.69	4.11	-2.82	4.17E-03	4.69E-02
cg00178119	RIMS2;RIMS2	8	Body;Body	N_Shore	55.42	5.10	58.24	6.16	-2.82	2.33E-03	3.15E-02
cg04730365	RANBP1;TRMT2A;TRMT2A	22	TSS200;TSS1500;TSS1500	Island	30.32	2.71	33.14	2.77	-2.82	9.20E-05	2.61E-03
cg27627246	SATB2	2	Body	Open sea	64.44	5.36	67.26	5.18	-2.82	1.79E-03	2.61E-02
cg07652854		7	Intergenic	S_Shelf	70.65	5.50	73.47	4.84	-2.82	3.20E-03	3.92E-02

cg15372009		4	Intergenic	N_Shelf	48.10	4.02	50.92	3.05	-2.82	1.29E-03	2.07E-02
cg05509251	ERCC4	16	Body	S_Shore	40.99	4.66	43.81	4.29	-2.82	3.77E-04	8.14E-03
cg18824501		2	Intergenic	Open sea	71.38	2.57	74.20	2.66	-2.82	1.79E-05	6.34E-04
cg07790831	ENAH;ENAH	1	Body;Body	Open sea	62.15	2.71	64.97	2.96	-2.82	3.43E-05	1.12E-03
cg07620533	PCDHGA4;PCDHGA6;PCDHGA9;PCDH(	5	Body;Body;Body;Body;Body;Body;Body;1s	N_Shore	21.34	3.52	24.16	3.70	-2.82	5.56E-04	1.10E-02
cg01139493		5	Intergenic	Open sea	69.65	4.01	72.47	3.62	-2.82	4.09E-03	4.64E-02
cg19015708	PPP3R1	2	Body	Open sea	31.24	4.60	34.06	3.82	-2.82	2.28E-03	3.10E-02
cg14209037	DLL4	15	Body	Island	82.75	3.57	85.57	2.69	-2.82	2.26E-04	5.45E-03
cg22694818	LHX8	1	5'UTR	Island	18.54	2.92	21.35	3.64	-2.82	4.65E-05	1.46E-03
cg07053668	TCF25	16	Body	Open sea	85.43	3.63	88.24	3.32	-2.82	8.70E-05	2.49E-03
cg17432061	PIP4K2B	17	3'UTR	Open sea	68.78	3.50	71.60	3.07	-2.82	9.41E-05	2.66E-03
cg19628148	CCDC37	3	5'UTR	Island	21.57	3.90	24.39	4.15	-2.82	1.53E-03	2.34E-02
cg00516030	CTGF	6	TSS1500	S_Shore	54.41	5.55	57.23	5.41	-2.82	4.34E-03	4.83E-02
cg12083655		8	Intergenic	Open sea	48.77	3.64	51.59	4.30	-2.82	8.58E-04	1.53E-02
cg14659662	GLIS1	1	5'UTR	Open sea	15.35	2.93	18.17	3.57	-2.82	1.91E-04	4.76E-03
cg11536586	FOXK1	7	Body	Open sea	63.37	3.77	66.19	2.97	-2.82	6.64E-04	1.26E-02
cg03064793	FAT1	4	Body	Open sea	80.37	5.20	83.19	4.19	-2.82	2.75E-03	3.52E-02
cg21509457	UBE3C	7	Body	Open sea	79.12	4.18	81.93	3.78	-2.82	3.91E-03	4.50E-02
cg00599393	C8orf58	8	Body	Island	27.19	5.40	30.01	6.27	-2.82	2.27E-03	3.08E-02
cg07986887		22	Intergenic	S_Shelf	54.68	4.02	57.50	5.20	-2.82	1.34E-03	2.12E-02
cg09301932	ZNF764	16	TSS1500	N_Shore	72.98	3.93	75.79	4.29	-2.82	9.15E-04	1.60E-02
cg20233582	TRIM61	4	TSS1500	Open sea	84.14	4.39	86.96	3.20	-2.82	1.21E-03	1.98E-02
cg05901543	CDH15	16	Body	Open sea	57.14	5.16	59.95	4.32	-2.82	3.44E-03	4.11E-02
cg14854112	RAI14;RAI14;RAI14;RAI14;RAI14;RAI1	5	Body;Body;Body;Body;Body;Body	Open sea	79.23	4.24	82.04	3.57	-2.82	2.37E-03	3.19E-02
cg10133630	RNF141	11	TSS1500	S_Shore	66.22	4.56	69.04	4.06	-2.82	1.62E-03	2.43E-02
cg04686204	MGC23284;MGC23284	16	Body;Body	Open sea	78.72	4.54	81.53	2.80	-2.82	1.18E-03	1.94E-02
cg22256027	MSX1	4	Body	N_Shore	25.32	4.09	28.13	4.35	-2.82	5.75E-04	1.13E-02
cg13873733	ENOPH1;HNRPDL;HNRPDL	4	Body;TSS1500;TSS1500	S_Shore	31.96	5.36	34.78	5.05	-2.82	2.30E-04	5.52E-03
cg06105694		17	Intergenic	Island	53.95	5.61	56.77	6.49	-2.82	2.10E-03	2.93E-02
cg02272667	MAD1L1;MAD1L1;MAD1L1	7	Body;Body;Body	Open sea	56.64	3.14	59.45	4.18	-2.82	3.41E-04	7.52E-03
cg07130508	LDB1;LDB1	10	Body;TSS1500	Open sea	17.40	3.49	20.22	3.52	-2.82	1.40E-04	3.69E-03
cg25181144	HS3ST3B1	17	Body	N_Shelf	57.12	5.05	59.94	5.46	-2.82	1.87E-03	2.70E-02
cg03682823	SGCE;PEG10;SGCE;PEG10;SGCE	7	TSS1500;5'UTR;TSS1500;5'UTR;TSS1500	S_Shore	46.28	2.77	49.09	2.76	-2.82	1.79E-04	4.53E-03
cg18575532	PPP1R1B;PPP1R1B	17	5'UTR;Body	S_Shore	77.97	4.13	80.79	4.10	-2.82	2.96E-03	3.71E-02
cg03458836	LOC100130017;FLJ41562	4	Body;3'UTR	Open sea	60.89	4.37	63.71	4.39	-2.82	2.34E-03	3.15E-02
cg18166990	SHANK2	11	Body	Open sea	75.15	2.98	77.96	3.39	-2.81	5.16E-05	1.60E-03
cg10256790		3	Intergenic	S_Shelf	80.91	4.87	83.72	4.71	-2.81	2.98E-03	3.72E-02
cg10156905	R3HDML;R3HDML	20	5'UTR;1stExon	Open sea	70.97	4.96	73.79	3.98	-2.81	3.86E-03	4.45E-02
cg16311186	RAB19	7	Body	Open sea	71.68	5.03	74.49	4.17	-2.81	3.42E-03	4.10E-02
cg13014803		13	Intergenic	Island	38.35	4.36	41.17	3.90	-2.81	6.18E-04	1.19E-02
cg13198503	ANKMY1;ANKMY1	2	TSS200;TSS200	Island	37.06	4.72	39.88	4.99	-2.81	9.55E-04	1.66E-02
cg27167562		16	Intergenic	N_Shelf	36.09	3.35	38.90	3.48	-2.81	2.53E-03	3.33E-02
cg13703049	HOXA10;HOXA10	7	Body;1stExon	Island	11.21	2.63	14.02	4.71	-2.81	7.84E-04	1.43E-02
cg03279522	AGAP1;AGAP1	2	Body;Body	N_Shore	64.66	4.41	67.48	4.23	-2.81	1.54E-03	2.35E-02
cg01879723	ZBTB38	3	5'UTR	N_Shore	35.08	4.15	37.90	3.80	-2.81	4.12E-03	4.65E-02
cg03933062	ARHGEF15	17	3'UTR	Open sea	72.33	3.63	75.14	3.71	-2.81	3.58E-04	7.81E-03
cg06053850		11	Intergenic	S_Shore	62.42	3.41	65.24	3.39	-2.81	2.72E-04	6.31E-03
cg04194745	TMEM63B	6	Body	Island	57.12	3.00	59.94	2.59	-2.81	1.41E-04	3.71E-03
cg08621610	SAMD12;SAMD12	8	Body;Body	Open sea	68.75	3.68	71.56	2.92	-2.81	2.81E-04	6.47E-03
cg20038368	TTC14;TTC14	3	3'UTR;3'UTR	Open sea	87.29	5.16	90.10	3.33	-2.81	3.24E-03	3.95E-02
cg21542223		1	Intergenic	N_Shore	19.12	3.12	21.93	3.75	-2.81	1.91E-04	4.77E-03
cg16139912	GADL1	3	Body	Open sea	32.72	4.90	35.53	5.47	-2.81	1.85E-03	2.68E-02
cg08380311	NFIC;NFIC	19	Body;Body	Island	48.47	4.01	51.28	4.14	-2.81	1.59E-03	2.40E-02

cg17029019	HIC1;HIC1	17	TSS1500;5'UTR	Island	14.64	3.51	17.45	4.50	-2.81	1.73E-05	6.16E-04
cg02600978	TRIM26	6	Body	Open sea	80.35	4.19	83.17	4.00	-2.81	1.43E-03	2.23E-02
cg19852921	PHACTR2;PHACTR2;PHACTR2;PHACTF	6	Body;Body;Body;Body	Open sea	88.74	3.31	91.55	2.62	-2.81	5.79E-05	1.76E-03
cg03215422	FBXL21	5	5'UTR	Island	25.02	4.65	27.83	4.64	-2.81	1.93E-03	2.75E-02
cg22117107		15	Intergenic	N_Shelf	84.80	3.11	87.61	2.58	-2.81	2.75E-04	6.36E-03
cg03344806	PRPS1L1	7	1stExon	Open sea	76.62	3.78	79.43	2.90	-2.81	4.85E-05	1.51E-03
cg01035956	TRIM7;TRIM7;TRIM7;TRIM7;TRIM7;TI	5	5'UTR;Body;5'UTR;5'UTR;5'UTR;1stExon	N_Shelf	55.50	3.82	58.31	3.64	-2.81	1.40E-03	2.19E-02
cg07294541	DEF6	6	TSS200	Open sea	49.31	4.66	52.12	5.32	-2.81	6.26E-04	1.21E-02
cg01108112		9	Intergenic	N_Shore	57.09	3.83	59.91	4.55	-2.81	1.70E-03	2.51E-02
cg20396069	CALCOCO2	17	TSS1500	N_Shore	49.85	3.76	52.66	4.51	-2.81	3.42E-03	4.10E-02
cg14531862	ZNF233	19	5'UTR	Island	14.10	3.03	16.91	3.22	-2.81	1.46E-06	6.39E-05
cg24150986	JAG2;JAG2	14	Body;Body	N_Shore	78.91	4.20	81.72	3.71	-2.81	1.01E-03	1.73E-02
cg03881711		4	Intergenic	Open sea	83.29	3.42	86.10	2.73	-2.81	8.88E-05	2.54E-03
cg15572423	TRIM39;TRIM39	6	Body;Body	Open sea	74.81	3.72	77.62	3.55	-2.81	1.05E-04	2.92E-03
cg13895322	PTMS	12	Body	S_Shore	27.08	3.22	29.90	3.68	-2.81	4.04E-03	4.59E-02
cg27283345	TFAP2A;TFAP2A	6	Body;TSS1500	N_Shore	26.70	2.90	29.51	3.99	-2.81	1.03E-04	2.86E-03
cg22996083	RUNDC3B;RUNDC3B;RUNDC3B	7	Body;Body;Body	Open sea	72.38	4.80	75.19	2.90	-2.81	4.93E-04	1.00E-02
cg09538129	MIR25;MCM7;MCM7;MIR106B;MIR9:	7	TSS1500;Body;Body;TSS200;TSS1500	Open sea	62.07	3.86	64.88	3.70	-2.81	3.44E-03	4.12E-02
cg23859051	SLC43A2	17	3'UTR	Island	77.86	4.59	80.67	3.66	-2.81	3.90E-03	4.49E-02
cg21382649		12	Intergenic	S_Shelf	67.54	4.86	70.36	4.26	-2.81	3.89E-03	4.48E-02
cg10181555		5	Intergenic	Open sea	74.55	3.16	77.36	3.03	-2.81	4.33E-04	9.06E-03
cg26107028		14	Intergenic	Open sea	79.20	3.93	82.01	4.00	-2.81	3.18E-04	7.12E-03
cg08271622	LOC388796;SNORA71B;LOC388796	20	Body;Body;Body	Open sea	73.71	3.80	76.52	4.68	-2.81	2.53E-04	5.96E-03
cg08739244	AMPD2;AMPD2;AMPD2	1	Body;Body;Body	Open sea	86.44	3.77	89.25	2.20	-2.81	6.63E-04	1.26E-02
cg26549174	ICAM1	19	TSS200	Island	11.28	3.05	14.09	4.78	-2.81	1.35E-03	2.13E-02
cg21862529	EDN2	1	Body	Open sea	35.53	3.47	38.34	4.14	-2.81	2.21E-03	3.03E-02
cg22052010	GSS	20	5'UTR	N_Shore	82.88	4.28	85.69	3.18	-2.81	1.77E-03	2.60E-02
cg12684175	OGG1;CAMK1;OGG1;OGG1;OGG1;OG	3	Body;Body;3'UTR;Body;3'UTR;3'UTR	N_Shelf	74.64	3.78	77.45	2.98	-2.81	8.03E-04	1.45E-02
cg13732857		2	Intergenic	Island	20.02	3.80	22.83	4.28	-2.81	1.19E-03	1.95E-02
cg14535980	C2orf40	2	TSS200	N_Shore	29.19	3.02	32.00	3.54	-2.81	1.68E-05	5.97E-04
cg08191993	PHRF1	11	3'UTR	Island	86.87	3.21	89.68	2.90	-2.81	2.28E-04	5.49E-03
cg21765235	ODZ4	11	5'UTR	Open sea	58.17	4.08	60.98	4.42	-2.81	4.30E-03	4.80E-02
cg05260966	SYN3;TIMP3;TIMP3;SYN3;SYN3	22	Body;1stExon;5'UTR;Body;Body	Island	18.06	3.96	20.87	3.81	-2.81	3.55E-03	4.20E-02
cg24579705		1	Intergenic	Open sea	65.09	3.30	67.90	2.81	-2.81	3.94E-04	8.41E-03
cg02060919		4	Intergenic	Open sea	64.28	4.61	67.09	4.37	-2.81	2.06E-03	2.89E-02
cg06878709	PLCE1;PLCE1	10	Body;Body	Open sea	52.93	4.60	55.74	4.64	-2.81	1.07E-03	1.80E-02
cg13619408	SARS	1	TSS1500	N_Shore	38.32	4.00	41.12	4.21	-2.81	3.17E-03	3.89E-02
cg06957310	ADAP2	17	Body	Open sea	67.01	4.39	69.82	4.06	-2.81	1.38E-03	2.16E-02
cg05501742	MPG;MPG;MPG	16	TSS200;TSS1500;TSS1500	N_Shore	14.30	4.17	17.11	3.90	-2.81	5.09E-04	1.03E-02
cg05140736	SLC26A11;SLC26A11;SLC26A11;SLC26.	17	Body;Body;Body;Body;TSS1500	S_Shore	76.27	3.84	79.08	3.31	-2.81	7.87E-04	1.43E-02
cg17643864	LYN;LYN	8	5'UTR;5'UTR	Open sea	54.42	6.89	57.23	7.36	-2.81	4.12E-03	4.65E-02
cg09638944		3	Intergenic	Open sea	74.31	4.93	77.12	3.63	-2.81	2.91E-03	3.66E-02
cg20903160		6	Intergenic	Island	38.47	4.09	41.28	4.00	-2.81	7.02E-04	1.31E-02
cg21874213	NRG2;NRG2;NRG2;NRG2	5	Body;Body;Body;Body	S_Shore	63.83	2.98	66.64	3.38	-2.81	2.31E-04	5.55E-03
cg06521827	HSPB7	1	TSS1500	Open sea	50.95	5.68	53.76	6.08	-2.81	8.68E-04	1.54E-02
cg11521436	N4BP2	4	5'UTR	Open sea	76.93	4.21	79.74	3.92	-2.81	9.12E-04	1.60E-02
cg15865150	AMIGO3;AMIGO3;RNF123	3	5'UTR;1stExon;Body	Island	38.27	4.09	41.08	3.91	-2.81	2.98E-03	3.73E-02
cg06796713	MEIS1	2	Body	S_Shelf	63.08	5.48	65.89	4.61	-2.81	7.26E-04	1.35E-02
cg25909368	C11orf34	11	TSS1500	Open sea	78.66	4.19	81.46	4.29	-2.81	2.51E-03	3.31E-02
cg16537749	NACA2	17	TSS200	Open sea	83.11	2.61	85.91	1.79	-2.81	5.92E-07	2.74E-05
cg17191178	SHOX2;SHOX2;SHOX2	3	TSS1500;TSS1500;TSS1500	S_Shore	16.46	3.37	19.27	4.01	-2.81	4.14E-04	8.75E-03
cg19602446		5	Intergenic	Open sea	79.65	4.23	82.46	3.88	-2.81	3.24E-03	3.95E-02
cg19083007	RCAN2	6	TSS1500	Open sea	78.42	5.11	81.22	3.90	-2.81	3.52E-03	4.18E-02

cg20469847		18	Intergenic	S_Shelf	57.28	4.22	60.09	4.03	-2.81	2.35E-03	3.17E-02
cg03540175	CCDC36;CCDC36;CCDC36	3	1stExon;5'UTR;5'UTR	Island	16.04	3.22	18.84	3.78	-2.81	2.05E-04	5.05E-03
cg01191509	MAD1L1;MAD1L1;MAD1L1	7	Body;Body;Body	S_Shelf	76.03	2.55	78.84	3.02	-2.81	1.15E-05	4.25E-04
cg27306382	LAMB2;USP19	3	3'UTR;TSS1500	S_Shore	24.05	2.68	26.86	3.93	-2.81	1.48E-04	3.88E-03
cg25635916	SEMA3B;SEMA3B	3	Body;Body	N_Shelf	68.87	4.95	71.68	4.55	-2.81	5.45E-04	1.08E-02
cg05995496	TRIM10;TRIM10	6	Body;Body	Open sea	67.17	3.91	69.98	4.42	-2.81	5.66E-04	1.11E-02
cg22326985		6	Intergenic	Open sea	78.87	4.84	81.68	5.08	-2.81	3.77E-03	4.38E-02
cg16182267	NFATC1;NFATC1;NFATC1;NFATC1	18	Body;Body;Body;Body	Island	85.94	3.88	88.75	2.72	-2.81	2.18E-03	3.00E-02
cg18767278		5	Intergenic	Open sea	75.90	5.45	78.71	5.22	-2.81	1.84E-03	2.66E-02
cg03052606	SCUBE3	6	TSS1500	N_Shore	85.15	3.88	87.96	3.52	-2.81	3.33E-04	7.39E-03
cg11229862	CCDC37	3	TSS200	Island	31.25	4.03	34.05	4.48	-2.81	2.05E-04	5.03E-03
cg14847134	SEMA4A	1	TSS1500	Open sea	64.40	3.07	67.20	3.13	-2.81	8.26E-05	2.38E-03
cg10778379	WHSC1;WHSC1;WHSC1;WHSC1;WHS(	4	1stExon;Body;Body;Body;Body;Body	Open sea	76.14	3.82	78.95	3.66	-2.81	1.88E-03	2.70E-02
cg10575376	DNAH10	12	TSS1500	N_Shore	18.53	4.89	21.34	5.51	-2.81	1.26E-03	2.03E-02
cg22859054	PPP1R2P1	6	Body	Island	33.03	3.06	35.83	2.99	-2.81	4.64E-04	9.57E-03
cg12757890		6	Intergenic	Open sea	70.93	2.60	73.74	2.80	-2.81	2.92E-05	9.73E-04
cg20905984	NCRNA00171	6	Body	Open sea	77.55	4.48	80.36	3.28	-2.81	4.34E-03	4.82E-02
cg05287437	GALNTL2	3	TSS1500	Open sea	68.12	4.42	70.93	3.92	-2.81	3.34E-05	1.09E-03
cg00187380	HOXC4;HOXC5;HOXC5	12	5'UTR;Body;Body	Island	26.13	3.47	28.93	4.57	-2.81	5.43E-04	1.08E-02
cg16184131	IL5;IL5	5	5'UTR;1stExon	Open sea	83.36	3.18	86.16	2.56	-2.81	4.68E-05	1.47E-03
cg15324580		11	Intergenic	N_Shore	64.87	3.63	67.68	3.21	-2.81	6.66E-04	1.26E-02
cg21863946	CROCC;CROCC	1	5'UTR;1stExon	Open sea	39.62	5.59	42.43	4.91	-2.81	4.27E-03	4.77E-02
cg07439037		17	Intergenic	N_Shore	79.17	3.54	81.97	3.66	-2.80	2.72E-04	6.30E-03
cg26690592	E2F8	11	5'UTR	N_Shore	27.71	5.19	30.52	5.20	-2.80	4.54E-04	9.40E-03
cg09627247		12	Intergenic	S_Shelf	54.53	4.15	57.33	5.36	-2.80	2.48E-03	3.28E-02
cg23479988	ABCB1	7	Body	N_Shelf	86.33	5.12	89.13	3.07	-2.80	1.10E-03	1.84E-02
cg14797467		1	Intergenic	Open sea	79.17	3.47	81.97	3.33	-2.80	7.56E-04	1.39E-02
cg26555052	FLT4	5	3'UTR	Open sea	73.84	4.01	76.65	3.21	-2.80	2.52E-03	3.32E-02
cg19263469		7	Intergenic	S_Shelf	32.99	3.61	35.79	4.92	-2.80	2.37E-03	3.18E-02
cg02582940	FER1L5	2	TSS200	Open sea	66.24	4.70	69.05	4.58	-2.80	3.00E-03	3.74E-02
cg27197276	C2CD4A	15	TSS1500	N_Shelf	82.52	4.34	85.32	3.68	-2.80	2.08E-03	2.91E-02
cg10597337	PSMD12;PSMD12	17	Body;Body	N_Shelf	65.60	4.23	68.40	4.13	-2.80	2.34E-03	3.16E-02
cg22322158	MMP20	11	TSS200	Open sea	83.05	3.99	85.85	3.67	-2.80	2.19E-03	3.01E-02
cg19384379	TMEM40	3	TSS200	Open sea	68.86	3.70	71.66	3.27	-2.80	6.72E-04	1.27E-02
cg24026290		6	Intergenic	N_Shelf	85.63	3.29	88.43	2.61	-2.80	2.33E-05	8.00E-04
cg01321189	BICD1;BICD1	12	TSS1500;TSS1500	Island	23.77	4.58	26.57	4.43	-2.80	1.82E-04	4.58E-03
cg16165575		1	Intergenic	Open sea	22.08	3.88	24.88	4.31	-2.80	1.15E-03	1.90E-02
cg25034395	HPSE2;HPSE2;HPSE2;HPSE2	10	TSS1500;TSS1500;TSS1500;TSS1500	S_Shelf	17.65	3.72	20.45	4.67	-2.80	4.52E-03	4.96E-02
cg02730492	COL4A1	13	Body	Open sea	82.61	3.82	85.41	3.65	-2.80	9.80E-04	1.69E-02
cg02247838	CCHCR1;CCHCR1;CCHCR1	6	Body;Body;Body	Open sea	66.63	3.77	69.43	3.51	-2.80	2.97E-03	3.72E-02
cg16828491	SH3TC1	4	Body	Open sea	71.52	4.57	74.32	4.55	-2.80	3.84E-03	4.44E-02
cg08133228	SLC39A7;SLC39A7	6	Body;Body	N_Shore	76.28	4.59	79.09	3.33	-2.80	3.26E-04	7.26E-03
cg23918296	AMN	14	3'UTR	Island	62.27	3.85	65.07	4.49	-2.80	4.04E-03	4.60E-02
cg22972187	FGA;FGA	4	TSS1500;TSS1500	Open sea	72.49	4.61	75.29	4.94	-2.80	3.49E-03	4.15E-02
cg12492289	PCBP3;PCBP3	21	TSS1500;TSS1500	Open sea	63.12	3.71	65.93	3.20	-2.80	1.04E-03	1.77E-02
cg19061798		11	Intergenic	Open sea	80.29	4.77	83.09	3.81	-2.80	1.11E-03	1.85E-02
cg03997663	HELT	4	TSS200	Island	18.72	3.52	21.52	3.71	-2.80	5.97E-04	1.16E-02
cg02726278	TADA2B	4	Body	S_Shore	75.11	4.08	77.91	4.40	-2.80	1.26E-03	2.03E-02
cg02959108		9	Intergenic	N_Shore	15.23	4.63	18.03	4.72	-2.80	1.18E-03	1.94E-02
cg12598635	CPLX1	4	5'UTR	Island	10.00	2.70	12.81	3.09	-2.80	3.69E-06	1.51E-04
cg23406971	C19orf25	19	3'UTR	S_Shore	66.80	4.11	69.60	3.19	-2.80	2.54E-03	3.33E-02
cg24065270	FGF1;FGF1;FGF1;FGF1;FGF1;FGF1	5	5'UTR;5'UTR;5'UTR;5'UTR;Body;Body	Open sea	62.18	4.72	64.98	3.99	-2.80	2.53E-03	3.33E-02
cg03362840	CALD1;CALD1;CALD1;CALD1;CALD1;C/	7	5'UTR;1stExon;1stExon;1stExon;5'UTR;5'U	Open sea	20.87	3.58	23.67	4.13	-2.80	5.83E-04	1.14E-02

cg12367786	KCNJ6	21	TSS1500	S_Shore	54.91	7.49	57.71	7.13	-2.80	1.35E-03	2.13E-02
cg10492657	CDYL;CDYL;CDYL	6	5'UTR;Body;Body	Open sea	81.48	3.68	84.28	2.97	-2.80	2.00E-04	4.95E-03
cg16911296	KLHL29	2	Body	S_Shore	69.82	3.88	72.62	2.86	-2.80	1.50E-03	2.30E-02
cg27188056	NLRP12	19	TSS200	Open sea	69.16	5.69	71.96	5.60	-2.80	3.21E-03	3.92E-02
cg00260802		20	Intergenic	Island	30.07	3.42	32.87	4.16	-2.80	3.03E-04	6.85E-03
cg03760759	TTC24;TTC24	1	5'UTR;1stExon	Open sea	77.33	4.20	80.13	4.34	-2.80	2.77E-03	3.54E-02
cg13103280		1	Intergenic	S_Shore	15.37	2.60	18.17	3.87	-2.80	2.44E-05	8.33E-04
cg25027273	CHSY1	15	Body	Open sea	79.22	4.61	82.02	3.98	-2.80	7.95E-04	1.44E-02
cg02587606		10	Intergenic	Open sea	72.45	4.45	75.25	3.94	-2.80	2.19E-03	3.01E-02
cg20773127	ENPEP	4	TSS200	N_Shore	27.24	3.49	30.04	3.88	-2.80	2.99E-03	3.74E-02
cg06093506		8	Intergenic	S_Shelf	84.75	4.71	87.55	4.24	-2.80	2.76E-03	3.54E-02
cg00277769	BAIAP2L1	7	3'UTR	Open sea	79.40	3.84	82.20	3.53	-2.80	1.82E-03	2.65E-02
cg08620986	SOX13	1	5'UTR	Open sea	65.04	3.98	67.84	3.55	-2.80	2.40E-03	3.21E-02
cg24614671		8	Intergenic	N_Shelf	52.67	4.37	55.47	3.08	-2.80	1.51E-03	2.32E-02
cg25630588	HYAL2;HYAL2	3	Body;Body	N_Shore	77.96	3.81	80.76	3.75	-2.80	4.02E-03	4.58E-02
cg11785240	HDAC4	2	Body	S_Shelf	78.80	3.49	81.60	3.16	-2.80	8.57E-04	1.53E-02
cg15608397	PAX3;PAX3;PAX3;PAX3;PAX3;PAX3;PA	2	1stExon;5'UTR;1stExon;1stExon;5'UTR;1st	Island	21.55	3.12	24.35	3.16	-2.80	8.34E-05	2.40E-03
cg04656070	TRPS1	8	5'UTR	S_Shore	78.09	4.23	80.89	3.17	-2.80	9.42E-05	2.66E-03
cg06972908	ITGAL;ITGAL	16	Body;Body	S_Shelf	65.67	5.05	68.47	4.46	-2.80	1.30E-03	2.07E-02
cg12389397		1	Intergenic	Open sea	84.90	3.30	87.70	3.15	-2.80	1.35E-04	3.58E-03
cg21462440		19	Intergenic	S_Shelf	76.92	4.61	79.71	3.95	-2.80	4.11E-03	4.65E-02
cg03905795	SHANK2	11	Body	N_Shore	64.94	5.30	67.74	3.82	-2.80	2.36E-04	5.63E-03
cg07113802	C7orf52	7	TSS200	S_Shore	38.28	4.27	41.07	3.66	-2.80	9.69E-04	1.67E-02
cg04708391	FBXO8	4	Body	Open sea	82.37	5.04	85.17	3.16	-2.80	9.16E-04	1.61E-02
cg09411999	HOXA10;HOXA10	7	TSS1500;Body	Island	32.25	3.70	35.05	3.91	-2.80	2.30E-03	3.12E-02
cg10313167	FCRL5	1	3'UTR	Open sea	65.04	2.99	67.83	4.05	-2.80	1.31E-05	4.81E-04
cg18449120	SOX11	2	1stExon	Island	30.77	4.08	33.56	3.77	-2.80	1.07E-03	1.80E-02
cg10574376		16	Intergenic	Island	12.72	3.32	15.52	3.31	-2.80	2.89E-05	9.66E-04
cg07376547		1	Intergenic	Island	18.25	4.35	21.05	5.11	-2.80	1.72E-03	2.54E-02
cg02769624	SLCO1C1;SLCO1C1;SLCO1C1;SLCO1C1	12	Body;Body;Body;Body	Open sea	65.24	3.38	68.04	3.35	-2.80	2.17E-04	5.28E-03
cg01940102		12	Intergenic	N_Shelf	85.52	4.67	88.32	3.40	-2.80	4.25E-04	8.94E-03
cg23991636	IGF2BP3	7	Body	N_Shore	24.30	3.58	27.10	4.57	-2.80	3.86E-04	8.29E-03
cg07275218	BRD2;BRD2	6	Body;Body	S_Shore	11.39	2.30	14.19	2.83	-2.80	7.38E-06	2.85E-04
cg23941434	JAKMIP2;JAKMIP2	5	5'UTR;1stExon	Open sea	36.96	6.82	39.75	8.49	-2.80	2.25E-03	3.07E-02
cg18166959		1	Intergenic	Open sea	70.38	4.23	73.18	3.95	-2.80	1.27E-03	2.04E-02
cg07155553	NOTCH4	6	Body	Open sea	87.67	3.47	90.47	2.69	-2.80	4.34E-05	1.37E-03
cg02551646		2	Intergenic	Open sea	61.67	4.77	64.47	4.72	-2.80	1.74E-03	2.56E-02
cg10948220	FAM176A;FAM176A	2	5'UTR;5'UTR	Open sea	15.20	2.36	17.99	2.56	-2.80	1.79E-06	7.75E-05
cg06686180	ATP4A	19	TSS1500	Open sea	91.39	4.21	94.18	1.33	-2.80	1.55E-04	4.01E-03
cg08286957	LMF1	16	Body	N_Shore	79.83	4.69	82.62	4.32	-2.80	4.36E-03	4.84E-02
cg09624923	ITGB1;ITGB1	10	5'UTR;5'UTR	N_Shelf	23.95	3.54	26.74	3.75	-2.80	2.87E-03	3.63E-02
cg11678896		4	Intergenic	Island	13.52	3.00	16.31	4.30	-2.80	1.07E-03	1.80E-02
cg06178299	PAX7;PAX7;PAX7	1	Body;Body;Body	Island	23.17	3.32	25.97	3.82	-2.80	4.33E-04	9.08E-03
cg05539847	HSD3B7;HSD3B7;HSD3B7;SETD1A	16	TSS1500;TSS1500;TSS1500;Body	S_Shelf	81.96	4.36	84.76	3.93	-2.80	3.49E-03	4.16E-02
cg01354455		10	Intergenic	Open sea	62.12	3.21	64.92	3.59	-2.80	1.09E-03	1.82E-02
cg18592964	CAST;CAST;CAST;CAST;CAST;CAST;CA!	5	Body;Body;5'UTR;Body;5'UTR;5'UTR;5'UTI	Open sea	64.79	4.57	67.58	4.22	-2.80	4.31E-03	4.80E-02
cg00442458		12	Intergenic	Open sea	73.25	3.44	76.04	3.68	-2.80	1.58E-04	4.09E-03
cg01995086	PKHD1L1	8	Body	Open sea	82.66	5.21	85.45	4.18	-2.80	3.21E-03	3.92E-02
cg00476608	ATN1;ATN1	12	Body;Body	N_Shore	79.16	4.51	81.95	3.54	-2.80	2.99E-03	3.73E-02
cg11962566	HYOU1;HYOU1	11	3'UTR;3'UTR	Open sea	75.83	3.98	78.63	2.79	-2.80	6.77E-04	1.28E-02
cg23476065	WDR86	7	Body	N_Shore	35.56	4.52	38.36	5.93	-2.80	4.13E-03	4.67E-02
cg08749305		6	Intergenic	Island	17.50	3.39	20.30	3.68	-2.80	1.49E-04	3.88E-03
cg19497709	COPG	3	Body	N_Shelf	82.39	3.90	85.18	2.88	-2.80	9.76E-04	1.68E-02

cg11838668		7	Intergenic	N_Shore	21.01	3.52	23.81	3.59	-2.80	4.14E-04	8.75E-03
cg18081863	FCGRT;FCGRT	19	TSS200;TSS1500	N_Shore	63.27	4.03	66.07	3.91	-2.79	1.45E-03	2.25E-02
cg07171765		4	Intergenic	Open sea	85.82	4.51	88.62	2.84	-2.79	2.00E-04	4.94E-03
cg07850987	HOXB3	17	5'UTR	S_Shelf	16.03	3.43	18.83	3.44	-2.79	1.21E-03	1.97E-02
cg27559870	SDC3	1	Body	Island	21.69	3.47	24.48	4.49	-2.79	5.14E-04	1.03E-02
cg17362483		4	Intergenic	Open sea	40.84	4.58	43.63	5.01	-2.79	1.66E-03	2.48E-02
cg12449837	TUG1;MORC2	22	TSS1500;TSS200	N_Shore	80.08	3.95	82.87	4.74	-2.79	4.31E-03	4.80E-02
cg27244206	ZNF286B	17	TSS1500	S_Shore	85.67	4.18	88.46	3.37	-2.79	3.77E-04	8.13E-03
cg00245850	GML	8	Body	Open sea	29.04	4.89	31.84	4.72	-2.79	3.52E-03	4.19E-02
cg11072119	RAP1GAP;RAP1GAP;RAP1GAP	1	Body;Body;Body	S_Shelf	38.15	3.54	40.95	4.14	-2.79	7.50E-04	1.38E-02
cg08420572	NFIX	19	Body	Island	57.67	3.65	60.46	3.80	-2.79	2.93E-04	6.67E-03
cg06463142		8	Intergenic	Island	69.88	4.69	72.68	4.51	-2.79	3.17E-03	3.89E-02
cg10956096		10	Intergenic	Open sea	37.91	3.72	40.70	4.27	-2.79	9.41E-04	1.64E-02
cg20010135	HSD3B7;HSD3B7;HSD3B7	16	5'UTR;5'UTR;5'UTR	Open sea	31.58	2.87	34.38	4.34	-2.79	2.43E-03	3.24E-02
cg22237200	ATP1B2	17	Body	Island	23.92	6.24	26.71	5.74	-2.79	1.97E-03	2.80E-02
cg07811779	CAMK1D;CAMK1D	10	Body;Body	Open sea	75.41	4.15	78.20	3.77	-2.79	3.69E-03	4.32E-02
cg21244177		1	Intergenic	Island	33.90	3.10	36.69	2.94	-2.79	5.14E-05	1.59E-03
cg01307526	SETD1B	12	Body	N_Shelf	74.16	3.38	76.96	3.19	-2.79	1.36E-04	3.62E-03
cg03048432	NIN;NIN;NIN;NIN	14	5'UTR;5'UTR;TSS1500;5'UTR	Open sea	74.00	3.72	76.79	4.09	-2.79	7.39E-04	1.37E-02
cg26666835		5	Intergenic	Island	17.17	3.20	19.96	3.35	-2.79	6.19E-05	1.87E-03
cg26668744		7	Intergenic	Open sea	77.19	4.99	79.99	5.91	-2.79	2.55E-03	3.35E-02
cg26194477	FOXG1	14	TSS1500	Island	28.21	4.15	31.00	3.82	-2.79	1.98E-03	2.81E-02
cg13230172	TMEM136	11	TSS200	Island	15.48	3.67	18.27	3.49	-2.79	4.12E-05	1.31E-03
cg06747770		2	Intergenic	Open sea	83.61	3.18	86.40	2.97	-2.79	2.83E-04	6.50E-03
cg12829666	SGEF	3	Body	Island	28.40	4.18	31.19	4.85	-2.79	3.93E-03	4.51E-02
cg18824990	HOTAIR	12	Body	N_Shore	13.50	2.30	16.29	3.34	-2.79	1.44E-05	5.23E-04
cg02934082	SEMA5B	3	5'UTR	Open sea	74.63	4.21	77.42	4.38	-2.79	2.92E-03	3.68E-02
cg04984575	L3MBTL;L3MBTL	20	TSS1500;TSS1500	N_Shore	45.09	3.71	47.88	3.85	-2.79	6.81E-04	1.28E-02
cg14683017	TFAP2A	6	Body	N_Shore	43.95	3.92	46.74	4.16	-2.79	3.29E-03	3.99E-02
cg25813193	SNCA;SNCA;SNCA;SNCA	4	Body;Body;Body;Body	Open sea	69.14	5.43	71.93	4.61	-2.79	3.47E-03	4.14E-02
cg07709195	FADS1	11	TSS1500	S_Shore	76.87	4.93	79.66	5.42	-2.79	3.43E-04	7.55E-03
cg23900905		8	Intergenic	N_Shelf	76.26	5.05	79.05	5.15	-2.79	3.05E-03	3.79E-02
cg04319844	ANK1;ANK1;ANK1;ANK1;ANK1;ANK1;A	8	Body;Body;Body;Body;Body;Body;Body;TS	Open sea	64.05	3.14	66.84	3.58	-2.79	2.64E-03	3.43E-02
cg15980076	CYP26C1	10	Body	Island	21.93	3.14	24.72	5.06	-2.79	3.27E-03	3.97E-02
cg14054279		3	Intergenic	Open sea	74.18	4.13	76.97	2.99	-2.79	9.86E-04	1.69E-02
cg23629643	SHROOM3	4	Body	Open sea	78.05	2.85	80.84	2.15	-2.79	2.56E-06	1.08E-04
cg01337391		16	Intergenic	Island	13.80	2.15	16.59	2.79	-2.79	3.02E-06	1.25E-04
cg23632849	TTYH2;MGC16275	17	TSS1500;Body	N_Shore	57.18	3.63	59.97	3.55	-2.79	6.82E-04	1.29E-02
cg17288471		2	Intergenic	Open sea	49.95	3.70	52.73	2.91	-2.79	1.95E-04	4.85E-03
cg09234454	ABCA4	1	Body	Open sea	81.06	4.04	83.85	3.64	-2.79	2.81E-04	6.47E-03
cg07159871	C20orf95	20	TSS1500	N_Shore	67.09	3.88	69.88	4.05	-2.79	3.32E-03	4.02E-02
cg00677708	TDH	8	TSS200	Open sea	65.11	4.34	67.90	5.01	-2.79	1.46E-03	2.26E-02
cg15356529		7	Intergenic	Open sea	72.21	4.97	75.00	3.73	-2.79	2.29E-03	3.11E-02
cg11305121	RALB	2	5'UTR	S_Shore	53.33	3.90	56.12	3.69	-2.79	6.55E-04	1.25E-02
cg23688111	TRPM8	2	TSS200	Open sea	54.80	6.32	57.59	6.54	-2.79	2.35E-03	3.16E-02
cg20732478	HOXD8	2	TSS1500	Island	11.36	2.19	14.15	2.17	-2.79	4.23E-07	1.99E-05
cg07207670		2	Intergenic	Open sea	74.77	4.80	77.56	4.33	-2.79	3.91E-03	4.50E-02
cg00495658	GDF2	10	TSS1500	S_Shelf	37.54	5.26	40.33	5.59	-2.79	1.64E-03	2.45E-02
cg07502389	NEFM;NEFM	8	TSS200;TSS1500	Island	23.02	3.95	25.80	3.44	-2.79	2.21E-04	5.36E-03
cg15264525	SC5DL;SC5DL	11	5'UTR;5'UTR	Open sea	87.47	4.42	90.26	2.78	-2.79	9.23E-04	1.61E-02
cg15377585	TMEM196	7	TSS1500	S_Shore	13.85	2.29	16.64	2.92	-2.79	5.28E-06	2.10E-04
cg25035489	NPHP4	1	Body	Open sea	83.98	3.76	86.77	2.73	-2.79	3.50E-04	7.68E-03
cg14377922	KLHL29	2	Body	Open sea	71.82	4.17	74.61	3.57	-2.79	3.95E-03	4.53E-02

cg09420907		12	Intergenic	N_Shore	19.26	3.75	22.05	3.17	-2.79	2.10E-04	5.13E-03
cg15313320	DKFZp686O24166	11	Body	N_Shore	21.53	4.07	24.32	5.96	-2.79	2.41E-03	3.22E-02
cg20362689		8	Intergenic	Open sea	85.24	3.40	88.03	2.83	-2.79	5.26E-05	1.62E-03
cg26659805	AMH	19	Body	Island	17.54	4.29	20.33	4.52	-2.79	2.07E-03	2.89E-02
cg12622451	SKAP2	7	Body	Open sea	83.23	3.76	86.02	2.74	-2.79	2.47E-04	5.85E-03
cg13974534	FXN;FXN;FXN	9	Body;Body;Body	S_Shore	85.35	4.19	88.14	3.24	-2.79	1.27E-03	2.04E-02
cg22011526	C6orf89	6	Body	S_Shelf	84.90	4.83	87.69	4.92	-2.79	4.55E-03	4.98E-02
cg12653788		1	Intergenic	N_Shore	77.02	3.63	79.81	3.39	-2.79	2.70E-04	6.28E-03
cg14357055		18	Intergenic	N_Shelf	85.96	4.23	88.75	3.44	-2.79	3.41E-03	4.09E-02
cg08583888	DNAJB6;DNAJB6	7	Body;Body	Open sea	81.97	2.65	84.75	2.30	-2.79	4.47E-06	1.81E-04
cg07174453	GTF3C1	16	Body	Open sea	78.56	3.69	81.34	3.51	-2.79	2.18E-04	5.29E-03
cg04406620	CYB5B	16	3'UTR	Open sea	70.10	4.70	72.88	4.92	-2.79	2.52E-03	3.32E-02
cg16027727	RPTOR;RPTOR	17	Body;Body	Open sea	85.37	4.48	88.16	3.10	-2.79	1.40E-03	2.20E-02
cg08857545		3	Intergenic	Open sea	85.53	3.84	88.31	3.36	-2.79	7.45E-04	1.37E-02
cg12451099	SLC32A1	20	Body	Island	18.69	3.82	21.48	4.39	-2.79	2.27E-03	3.08E-02
cg11270895	JSRP1	19	TSS200	S_Shore	78.23	4.16	81.02	3.98	-2.79	3.55E-03	4.20E-02
cg22471112	TRIM2;TRIM2	4	TSS200;5'UTR	Open sea	16.38	3.57	19.16	4.15	-2.79	3.46E-03	4.13E-02
cg26330133	DLGAP4;DLGAP4;DLGAP4	20	TSS1500;TSS200;Body	Island	11.47	1.72	14.25	2.66	-2.79	8.61E-08	4.32E-06
cg23633905	RGL2;RGL2	6	Body;Body	N_Shelf	49.84	3.08	52.63	3.97	-2.79	1.17E-03	1.93E-02
cg23229770		2	Intergenic	N_Shelf	33.80	4.74	36.59	4.03	-2.78	1.74E-03	2.56E-02
cg19735514	ATF6	1	Body	Open sea	49.19	5.48	51.97	4.72	-2.78	1.76E-03	2.59E-02
cg15096377		7	Intergenic	S_Shore	21.15	3.02	23.94	3.72	-2.78	1.11E-04	3.06E-03
cg06769989		5	Intergenic	Open sea	84.54	4.04	87.32	2.62	-2.78	9.41E-05	2.66E-03
cg13087076	DYDC2;DYDC1	10	TSS200;5'UTR	Island	38.41	5.04	41.19	4.83	-2.78	4.12E-03	4.65E-02
cg21077999	FLJ23834	7	Body	Open sea	70.17	4.14	72.95	4.47	-2.78	2.97E-03	3.72E-02
cg02116437	GPBAR1;GPBAR1;GPBAR1	2	5'UTR;5'UTR;5'UTR	Open sea	68.95	3.78	71.73	3.51	-2.78	4.45E-05	1.40E-03
cg09650134	ADAP1	7	Body	Open sea	80.92	4.14	83.71	2.70	-2.78	1.16E-03	1.92E-02
cg08885197	CASZ1;CASZ1	1	Body;Body	Open sea	54.74	3.24	57.52	3.82	-2.78	2.10E-03	2.93E-02
cg01665970	C6orf10	6	Body	Open sea	59.51	4.23	62.30	4.07	-2.78	1.76E-03	2.58E-02
cg16901014	ZNF7	8	TSS1500	N_Shore	86.20	4.52	88.98	3.45	-2.78	7.32E-04	1.36E-02
cg14376289		11	Intergenic	Open sea	86.27	3.65	89.05	3.83	-2.78	4.12E-04	8.72E-03
cg16317273	EGFLAM	5	TSS1500	Island	14.67	2.53	17.45	2.62	-2.78	4.40E-06	1.78E-04
cg17054691	P4HB	17	Body	Open sea	45.90	3.21	48.69	3.80	-2.78	4.50E-03	4.94E-02
cg12518626		4	Intergenic	Open sea	88.72	3.37	91.50	2.38	-2.78	7.45E-05	2.18E-03
cg01597149		7	Intergenic	N_Shore	55.24	3.66	58.02	3.32	-2.78	2.22E-03	3.04E-02
cg04110478		5	Intergenic	Open sea	69.33	4.33	72.12	4.31	-2.78	2.96E-03	3.71E-02
cg15814717		6	Intergenic	Island	47.83	4.00	50.61	3.52	-2.78	9.17E-04	1.61E-02
cg15999796	MOG;MOG;MOG;MOG;MOG;MOG;M	6	TSS1500;TSS1500;TSS1500;TSS1500;TSS15	Open sea	52.30	3.67	55.08	3.51	-2.78	3.05E-04	6.89E-03
cg05025913	PPFIBP2	11	5'UTR	S_Shelf	74.91	3.43	77.69	3.58	-2.78	4.48E-04	9.32E-03
cg17479763	CHD5	1	Body	N_Shelf	59.33	4.43	62.11	4.28	-2.78	1.90E-03	2.73E-02
cg04695732	CECR6;CECR6;CECR6	22	1stExon;3'UTR;3'UTR	N_Shore	55.06	4.49	57.84	4.19	-2.78	2.83E-04	6.50E-03
cg22396119		8	Intergenic	Open sea	61.60	6.68	64.38	6.60	-2.78	2.64E-03	3.42E-02
cg22603569	NFIC;NFIC	19	Body;Body	Island	33.25	2.84	36.03	3.26	-2.78	9.81E-04	1.69E-02
cg25552017		1	Intergenic	N_Shelf	64.75	3.08	67.54	3.63	-2.78	8.85E-05	2.53E-03
cg26194089		12	Intergenic	Island	29.56	3.68	32.34	4.39	-2.78	3.91E-03	4.49E-02
cg08654915		3	Intergenic	S_Shore	20.75	2.48	23.53	3.57	-2.78	1.34E-04	3.56E-03
cg15454385	PDGFA;PDGFA	7	Body;Body	N_Shore	58.40	4.88	61.18	5.03	-2.78	1.97E-03	2.80E-02
cg23206851	HOXA2	7	TSS1500	N_Shore	12.72	2.53	15.50	3.19	-2.78	4.14E-05	1.32E-03
cg23109714		11	Intergenic	Open sea	35.68	3.19	38.46	4.40	-2.78	3.86E-03	4.46E-02
cg15077643	ABI2	2	Body	Open sea	73.05	3.94	75.83	3.87	-2.78	4.51E-03	4.95E-02
cg02168303		7	Intergenic	Island	15.48	2.47	18.26	2.69	-2.78	1.26E-06	5.59E-05
cg12548634	RNF220	1	Body	Island	16.89	4.16	19.67	5.15	-2.78	3.01E-04	6.82E-03
cg12507881		13	Intergenic	N_Shore	13.80	2.22	16.58	2.45	-2.78	2.97E-07	1.42E-05

cg05397646	FRMD4A	10	Body	Open sea	83.50	3.92	86.28	4.22	-2.78	3.03E-03	3.77E-02
cg14692920	RAPH1;RAPH1	2	Body;Body	Open sea	86.03	4.06	88.81	3.32	-2.78	9.96E-04	1.71E-02
cg07190752	CHIC2	4	Body	Open sea	80.33	3.84	83.11	4.36	-2.78	7.07E-04	1.32E-02
cg15299233	SCAMP2	15	Body	N_Shelf	74.56	3.99	77.34	4.03	-2.78	1.32E-04	3.52E-03
cg24636999	DNAH8	6	Body	Open sea	67.18	4.35	69.96	5.62	-2.78	1.75E-03	2.58E-02
cg03371669	HOXD12	2	Body	Island	27.54	3.33	30.32	4.40	-2.78	2.55E-03	3.35E-02
cg02047547	ITPRIPL2;ITPRIPL2	16	1stExon;Body	Island	48.72	3.85	51.49	3.78	-2.78	1.10E-03	1.84E-02
cg00834796	JAKMIP2	5	5'UTR	Open sea	38.76	6.66	41.54	6.60	-2.78	1.48E-03	2.28E-02
cg22858500	CACNB2;CACNB2;CACNB2;CACNB2;CA	10	Body;Body;Body;Body;Body;Body;TSS150(	Open sea	64.75	4.10	67.53	4.12	-2.78	3.64E-04	7.92E-03
cg19769182	PRRT2	16	5'UTR	Island	27.09	3.83	29.87	4.47	-2.78	7.36E-04	1.36E-02
cg11961401	FLJ41941	22	TSS1500	S_Shelf	53.94	5.95	56.72	6.76	-2.78	1.60E-03	2.41E-02
cg00082235	IFFO1;IFFO1	12	1stExon;1stExon	Island	4.30	1.41	7.08	3.20	-2.78	3.92E-07	1.85E-05
cg09408571	GPR88	1	TSS200	N_Shore	83.50	3.76	86.28	3.32	-2.78	9.07E-04	1.59E-02
cg16851584	SDK1;SDK1	7	Body;Body	Open sea	72.84	3.52	75.61	3.50	-2.78	4.55E-04	9.43E-03
cg23643813	DVL1	1	3'UTR	N_Shore	70.72	3.39	73.50	3.08	-2.78	1.13E-03	1.88E-02
cg12976883	SP140;SP140	2	TSS200;TSS200	Open sea	41.59	4.07	44.37	3.91	-2.78	2.97E-04	6.74E-03
cg27223728		2	Intergenic	S_Shelf	59.82	5.50	62.60	5.31	-2.78	3.41E-03	4.10E-02
cg05164758		17	Intergenic	Island	16.98	3.56	19.76	3.08	-2.78	6.67E-04	1.26E-02
cg13777700	USP20;USP20;USP20	9	3'UTR;3'UTR;3'UTR	Open sea	82.23	3.60	85.01	3.35	-2.78	2.83E-03	3.60E-02
cg21503646		7	Intergenic	S_Shore	57.69	5.51	60.46	6.21	-2.78	1.55E-03	2.35E-02
cg18625627	TSHR;TSHR;TSHR	14	Body;Body;Body	Open sea	16.78	4.04	19.56	4.58	-2.78	2.75E-03	3.52E-02
cg20728514	PARVG;PARVG	22	TSS1500;TSS1500	Open sea	39.95	3.41	42.72	3.02	-2.78	1.42E-03	2.21E-02
cg26411822	STX12	1	TSS1500	N_Shore	50.91	5.08	53.68	3.62	-2.78	5.83E-04	1.14E-02
cg00643876	TBC1D24	16	Body	Island	77.00	4.39	79.78	2.94	-2.78	3.35E-03	4.05E-02
cg24328095	CSNK1D;CSNK1D	17	Body;Body	N_Shore	53.91	3.69	56.68	3.36	-2.78	2.01E-04	4.97E-03
cg26196480	TBX5;TBX5	12	TSS200;TSS200	Island	21.42	4.06	24.19	5.23	-2.78	4.53E-03	4.97E-02
cg23942789	PDE8B;PDE8B;PDE8B;PDE8B;PDE8B	5	TSS1500;TSS1500;TSS1500;TSS1500;TSS15	N_Shore	60.50	4.21	63.28	4.25	-2.78	4.56E-03	4.99E-02
cg22179905		8	Intergenic	N_Shelf	76.98	3.75	79.76	3.99	-2.78	2.08E-03	2.90E-02
cg23599026	SEC31B	10	Body	Open sea	54.72	4.42	57.49	4.76	-2.78	1.15E-04	3.13E-03
cg26332310	BARHL2	1	1stExon	Island	14.43	3.16	17.21	3.95	-2.78	7.88E-04	1.43E-02
cg16932472	IPO5;IPO5	13	1stExon;5'UTR	Open sea	72.37	3.51	75.15	3.32	-2.78	8.66E-05	2.48E-03
cg01957599	FAM13AOS	4	Body	Open sea	84.74	4.40	87.52	3.80	-2.78	1.16E-03	1.92E-02
cg21984129		19	Intergenic	N_Shelf	67.78	2.90	70.55	3.10	-2.78	2.99E-04	6.79E-03
cg24115596		14	Intergenic	N_Shore	83.41	4.64	86.18	4.60	-2.78	1.83E-03	2.65E-02
cg25969318	SPAM1;SPAM1	7	Body;3'UTR	Open sea	85.13	4.79	87.91	3.15	-2.78	2.91E-03	3.67E-02
cg03742137	AGAP1;AGAP1	2	Body;Body	N_Shelf	17.15	3.41	19.92	2.92	-2.78	5.92E-04	1.15E-02
cg00583003	SPP1;SPP1;SPP1	4	TSS1500;TSS1500;TSS1500	Open sea	72.68	5.25	75.45	4.15	-2.77	1.64E-03	2.45E-02
cg01506298	FLJ40330	2	Body	Island	36.33	3.99	39.11	3.67	-2.77	1.42E-03	2.21E-02
cg04475157		6	Intergenic	Open sea	65.96	4.07	68.74	3.83	-2.77	6.75E-04	1.27E-02
cg00508334	IGF2BP3	7	TSS1500	S_Shore	9.38	2.08	12.15	2.29	-2.77	2.99E-08	1.55E-06
cg12847662	PDLIM3;PDLIM3	4	Body;Body	Open sea	91.18	4.69	93.95	1.66	-2.77	6.70E-04	1.27E-02
cg11830800	CNTN1;CNTN1	12	5'UTR;5'UTR	S_Shore	15.85	3.45	18.62	4.15	-2.77	3.46E-03	4.14E-02
cg22558389	GPRIN3	4	5'UTR	N_Shelf	85.44	4.66	88.21	3.77	-2.77	2.78E-05	9.34E-04
cg26840672	KCNQ5;KCNQ5;KCNQ5;KCNQ5;KCNQ5	6	Body;Body;Body;Body;Body	N_Shore	69.62	4.58	72.39	3.77	-2.77	1.35E-03	2.14E-02
cg17304276	CUX2	12	Body	Open sea	79.19	4.60	81.96	4.80	-2.77	7.92E-04	1.44E-02
cg21350007	ZNF828;ZNF828;ZNF828	13	5'UTR;5'UTR;5'UTR	S_Shelf	85.73	4.33	88.50	3.14	-2.77	6.22E-04	1.20E-02
cg18390495	DEFB132	20	Body	Open sea	53.45	5.10	56.23	5.20	-2.77	2.30E-03	3.12E-02
cg12085226	MYOF;MYOF	10	Body;Body	Open sea	89.07	3.26	91.84	2.74	-2.77	3.82E-05	1.23E-03
cg16113650	VARS2;VARS2;VARS2	6	Body;Body;Body	S_Shore	77.33	4.44	80.10	3.64	-2.77	4.33E-03	4.81E-02
cg15072832	AHDC1	1	5'UTR	S_Shore	12.69	2.27	15.46	2.97	-2.77	6.54E-06	2.55E-04
cg06446163	L3MBTL;L3MBTL	20	TSS1500;TSS1500	N_Shore	47.15	2.99	49.93	3.42	-2.77	1.82E-04	4.58E-03
cg08752135	SYT7	11	3'UTR	S_Shore	36.09	3.98	38.86	4.38	-2.77	1.30E-03	2.08E-02
cg10017641	ATP2B4;ATP2B4	1	5'UTR;5'UTR	Open sea	80.22	3.53	82.99	3.80	-2.77	3.15E-04	7.07E-03

cg05889186	TNXB	6	Body	Island	37.85	3.39	40.62	3.68	-2.77	5.19E-04	1.04E-02
cg19424122	IL17B	5	Body	Open sea	81.10	3.93	83.87	3.86	-2.77	1.58E-03	2.39E-02
cg17833197		22	Intergenic	S_Shore	73.74	4.03	76.51	3.22	-2.77	9.47E-04	1.65E-02
cg18345456	PROX1	1	Body	Open sea	68.44	4.50	71.21	3.88	-2.77	3.11E-03	3.84E-02
cg15924570		1	Intergenic	N_Shore	59.86	3.54	62.64	4.43	-2.77	1.47E-03	2.27E-02
cg20486246	FAM46B	1	Body	N_Shore	37.53	3.75	40.30	4.12	-2.77	8.19E-04	1.47E-02
cg00570000	SPAG17	1	TSS1500	S_Shore	29.78	3.80	32.55	3.87	-2.77	1.91E-03	2.74E-02
cg00669594	COL11A2;COL11A2;COL11A2;COL11A2	6	Body;Body;Body;Body	N_Shelf	73.72	3.54	76.49	4.56	-2.77	9.64E-04	1.67E-02
cg14760020		3	Intergenic	Open sea	68.11	3.77	70.88	3.70	-2.77	1.60E-04	4.13E-03
cg24629431		10	Intergenic	Open sea	83.90	4.37	86.67	3.64	-2.77	1.63E-04	4.19E-03
cg00153395	JAK1	1	Body	Open sea	63.81	3.44	66.58	3.12	-2.77	3.50E-04	7.67E-03
cg00868652	ATP8B4	15	TSS200	Open sea	65.16	5.18	67.93	4.41	-2.77	1.43E-03	2.23E-02
cg06433023	NR2E1	6	Body	Island	29.85	4.44	32.62	3.51	-2.77	6.10E-04	1.18E-02
cg18758796	PDLIM4;PDLIM4;PDLIM4;PDLIM4	5	5'UTR;1stExon;1stExon;5'UTR	Island	13.65	3.42	16.42	3.27	-2.77	1.24E-05	4.56E-04
cg03438943	NTRK1;INSRR;NTRK1;NTRK1	1	Body;TSS1500;TSS1500;TSS1500	Island	14.54	4.20	17.31	3.56	-2.77	5.21E-06	2.08E-04
cg10611444	ZC3H8	2	Body	N_Shelf	77.48	3.33	80.25	3.44	-2.77	3.07E-04	6.92E-03
cg12157156	NCOR2;NCOR2	12	Body;Body	Open sea	74.31	3.87	77.08	3.59	-2.77	6.22E-04	1.20E-02
cg07531268	OSR1	2	3'UTR	Island	11.16	2.98	13.93	3.73	-2.77	7.71E-05	2.25E-03
cg25513776	MYLK4	6	TSS200	Open sea	67.85	4.23	70.62	4.13	-2.77	1.60E-03	2.42E-02
cg16117070		2	Intergenic	N_Shelf	62.55	2.63	65.32	2.27	-2.77	1.29E-05	4.73E-04
cg10539808	KCTD1	18	TSS1500	Open sea	76.91	4.15	79.68	3.68	-2.77	4.45E-04	9.26E-03
cg22354583	HPCA	1	Body	Island	31.05	4.87	33.81	4.03	-2.77	3.42E-03	4.10E-02
cg12833465	SFTA3	14	Body	Island	11.22	2.01	13.98	2.83	-2.77	1.14E-07	5.65E-06
cg17743939	NOVA2	19	Body	Island	90.12	4.32	92.89	3.04	-2.77	4.07E-04	8.63E-03
cg04205041	SYTL3	6	TSS200	Open sea	59.83	4.75	62.59	5.80	-2.77	3.55E-03	4.21E-02
cg07009968		15	Intergenic	Open sea	58.78	3.65	61.55	3.09	-2.77	2.42E-05	8.29E-04
cg01522083	C16orf42;GNPTG	16	Body;TSS1500	N_Shore	71.61	4.28	74.38	3.88	-2.77	3.62E-03	4.26E-02
cg06885910	DOK7;DOK7	4	Body;Body	N_Shelf	75.08	5.25	77.84	4.51	-2.77	4.55E-03	4.98E-02
cg27336068	ADARB2	10	Body	Open sea	16.46	4.17	19.23	4.70	-2.77	4.13E-03	4.66E-02
cg01345136	GALNT9	12	Body	N_Shore	12.46	3.64	15.23	4.08	-2.77	3.19E-03	3.91E-02
cg16824584		20	Intergenic	Island	17.09	3.59	19.86	3.78	-2.77	1.88E-04	4.70E-03
cg08690859	FLJ32063	2	Body	Island	19.99	3.31	22.76	3.39	-2.77	1.46E-05	5.30E-04
cg15996397		2	Intergenic	Open sea	26.57	2.89	29.34	2.59	-2.77	2.06E-04	5.06E-03
cg09315586		13	Intergenic	Open sea	74.43	3.36	77.20	4.11	-2.77	1.88E-04	4.70E-03
cg03885037	ADAM8;ADAM8;ADAM8	10	Body;Body;Body	Island	20.88	4.93	23.65	5.41	-2.77	4.01E-04	8.54E-03
cg13458335	BMP8B	1	TSS1500	Island	11.34	3.21	14.10	3.99	-2.77	1.16E-04	3.17E-03
cg13979573		14	Intergenic	Open sea	80.58	3.58	83.35	3.11	-2.77	1.44E-04	3.78E-03
cg09729500		10	Intergenic	Island	73.41	4.39	76.17	3.36	-2.77	3.06E-03	3.79E-02
cg12562403	TSPAN9;TSPAN9	12	5'UTR;5'UTR	Open sea	18.82	3.66	21.58	3.42	-2.77	4.78E-04	9.78E-03
cg23315601	GRIK4	11	Body	Open sea	53.36	3.76	56.13	3.79	-2.77	5.51E-04	1.09E-02
cg08274821	PTPN14	1	Body	S_Shore	80.25	3.14	83.01	3.89	-2.77	1.42E-04	3.75E-03
cg05894754	UNC80;UNC80	2	Body;Body	S_Shore	79.07	4.84	81.84	2.99	-2.77	3.08E-03	3.81E-02
cg04895288		7	Intergenic	Island	13.42	2.93	16.19	3.45	-2.77	5.13E-05	1.59E-03
cg11369862		10	Intergenic	N_Shore	70.78	4.41	73.55	4.03	-2.77	3.21E-03	3.92E-02
cg21859603	WDR46;WDR46	6	Body;Body	S_Shore	70.92	3.59	73.69	3.62	-2.77	8.66E-04	1.54E-02
cg22164539		6	Intergenic	Open sea	73.71	4.60	76.48	4.82	-2.77	4.26E-03	4.76E-02
cg07140270	KLHDC1	14	Body	S_Shore	70.94	4.41	73.71	4.24	-2.77	3.28E-03	3.99E-02
cg20648501	CHD7	8	5'UTR	Island	60.13	3.87	62.90	3.77	-2.77	2.08E-03	2.90E-02
cg26568372		1	Intergenic	N_Shore	17.35	4.02	20.11	4.24	-2.77	2.61E-03	3.40E-02
cg10732215		10	Intergenic	Island	28.86	5.69	31.63	5.77	-2.77	2.47E-03	3.27E-02
cg05118364	NAT15;NAT15;NAT15	16	Body;Body;Body	N_Shore	59.91	3.42	62.67	3.29	-2.77	2.75E-03	3.53E-02
cg14955103	EHMT2;EHMT2	6	Body;Body	N_Shore	76.43	4.44	79.20	3.25	-2.77	7.19E-04	1.34E-02
cg21854952	HIC1;HIC1	17	Body;Body	Island	22.01	3.31	24.78	3.63	-2.77	4.89E-04	9.96E-03

cg14638662		17	Intergenic	S_Shelf	53.50	4.24	56.27	3.82	-2.77	6.93E-04	1.30E-02
cg16122628	C6orf145	6	Body	N_Shore	20.61	3.69	23.38	3.93	-2.77	2.43E-03	3.24E-02
cg06428196		2	Intergenic	Open sea	83.61	5.13	86.38	3.47	-2.77	3.39E-03	4.08E-02
cg16254375		4	Intergenic	S_Shore	83.02	3.37	85.78	2.62	-2.77	5.05E-05	1.57E-03
cg14193653	NFIL3	9	5'UTR	Open sea	44.24	4.79	47.01	3.73	-2.77	2.07E-03	2.90E-02
cg14440664	PDCD1LG2	9	TSS1500	Open sea	73.15	3.97	75.91	3.45	-2.77	6.12E-04	1.18E-02
cg18023041	NAV2;NAV2;NAV2	11	Body;Body;Body	Open sea	76.29	4.21	79.06	2.83	-2.77	3.57E-03	4.22E-02
cg16071219	LPAR6;LPAR6;RB1;LPAR6	13	Body;Body;Body;1stExon	Open sea	33.05	4.76	35.81	5.99	-2.77	6.51E-04	1.24E-02
cg04793272	ABCG4;ABCG4	11	Body;Body	S_Shore	58.76	4.27	61.52	4.01	-2.77	3.92E-03	4.50E-02
cg19008597	BCAT1	12	TSS200	S_Shore	58.22	4.24	60.99	3.94	-2.77	2.23E-03	3.05E-02
cg10507482		2	Intergenic	N_Shore	26.42	3.51	29.19	3.91	-2.77	9.47E-05	2.68E-03
cg17294725	UNCX	7	TSS200	Island	14.33	2.80	17.09	2.36	-2.77	5.35E-06	2.13E-04
cg04262471	B3GALT4	6	1stExon	S_Shore	49.63	5.01	52.39	6.18	-2.76	2.60E-03	3.39E-02
cg11944480	IGF2BP3	7	Body	N_Shelf	64.83	5.49	67.59	5.23	-2.76	3.12E-04	7.02E-03
cg23510131	GHRHR	7	3'UTR	Open sea	70.34	4.75	73.10	4.27	-2.76	3.91E-03	4.49E-02
cg20864568	MAP3K14	17	5'UTR	N_Shelf	43.28	5.57	46.05	5.68	-2.76	1.48E-03	2.28E-02
cg08284758	FAM189A1	15	Body	Open sea	54.60	3.75	57.37	3.65	-2.76	1.28E-03	2.06E-02
cg26320000		2	Intergenic	Open sea	87.20	2.79	89.96	2.45	-2.76	5.58E-06	2.21E-04
cg17981927	NEU4;NEU4;NEU4;NEU4;NEU4	2	Body;Body;Body;Body;Body	Island	82.20	4.47	84.97	3.72	-2.76	3.43E-03	4.11E-02
cg13487992	PSMB11	14	TSS1500	Open sea	71.19	4.95	73.95	4.24	-2.76	2.23E-03	3.05E-02
cg08215318		16	Intergenic	Open sea	76.17	4.59	78.94	3.70	-2.76	4.17E-03	4.69E-02
cg06698272		20	Intergenic	N_Shore	80.74	3.44	83.51	2.87	-2.76	6.88E-04	1.29E-02
cg07470341	GALM	2	TSS1500	Open sea	75.81	4.07	78.58	3.28	-2.76	7.44E-04	1.37E-02
cg11157208	KIAA1026;KIAA1026;KIAA1026;KIAA1(	1	Body;3'UTR;3'UTR;3'UTR;3'UTR	Open sea	80.62	3.49	83.38	2.69	-2.76	4.58E-05	1.44E-03
cg25989526		13	Intergenic	Open sea	70.29	5.05	73.05	3.64	-2.76	1.86E-03	2.69E-02
cg13597528	C17orf93;PRAC	17	Body;TSS1500	Island	12.66	2.46	15.43	3.56	-2.76	2.39E-05	8.20E-04
cg00014377		7	Intergenic	Open sea	83.15	6.59	85.92	3.83	-2.76	3.05E-03	3.79E-02
cg19643803	MEG3;MEG3;MEG3	14	TSS1500;TSS1500;TSS1500	N_Shore	46.67	3.17	49.43	3.19	-2.76	1.84E-04	4.63E-03
cg15215033	SLC38A10;SLC38A10	17	Body;Body	N_Shore	63.27	4.32	66.03	3.34	-2.76	4.51E-04	9.36E-03
cg21094737	B3GNTL1	17	Body	S_Shore	69.59	3.98	72.35	3.76	-2.76	3.00E-03	3.74E-02
cg10543501	HAND1	5	1stExon	Island	18.06	2.65	20.82	3.00	-2.76	1.56E-06	6.79E-05
cg17171786		15	Intergenic	Open sea	54.93	3.42	57.69	5.26	-2.76	1.67E-03	2.49E-02
cg18477188	HLA-DOA	6	Body	Island	88.54	3.81	91.30	2.66	-2.76	7.72E-04	1.41E-02
cg10716120	PLCH2	1	TSS1500	N_Shelf	40.03	3.17	42.79	3.60	-2.76	2.09E-03	2.91E-02
cg09115960		16	Intergenic	N_Shore	87.64	3.29	90.40	2.04	-2.76	2.80E-05	9.38E-04
cg04716021	ZMIZ1	10	Body	Open sea	40.82	3.24	43.58	3.33	-2.76	5.47E-04	1.09E-02
cg06303875	CDX2	13	TSS200	Island	18.27	3.81	21.03	4.36	-2.76	2.66E-03	3.45E-02
cg05239310	CLMN	14	3'UTR	Open sea	29.53	5.62	32.29	5.96	-2.76	4.15E-03	4.68E-02
cg05784029	PPP1R9B	17	Body	N_Shelf	74.06	4.88	76.82	4.54	-2.76	2.73E-03	3.51E-02
cg14023309		9	Intergenic	N_Shore	25.26	2.51	28.02	2.65	-2.76	1.65E-05	5.88E-04
cg07598341	ST3GAL3;ST3GAL3;ST3GAL3;ST3GAL3;	1	Body;Body;Body;Body;Body;Body;Body;Bo	Open sea	87.53	3.55	90.29	2.40	-2.76	1.18E-05	4.37E-04
cg17722656	FAM162B	6	TSS1500	S_Shore	64.11	4.30	66.87	3.71	-2.76	3.08E-03	3.82E-02
cg08117110	STARD5	15	3'UTR	Open sea	77.17	5.04	79.93	4.40	-2.76	3.87E-03	4.46E-02
cg26064870	NCALD;NCALD;NCALD;NCALD;NCALD;	8	5'UTR;5'UTR;5'UTR;5'UTR;5'UTR;5'UTR;5'U	Open sea	67.43	4.52	70.19	4.42	-2.76	3.83E-03	4.43E-02
cg22128918	ZBTB12	6	5'UTR	Island	68.87	3.05	71.63	3.15	-2.76	1.97E-04	4.88E-03
cg04526209		19	Intergenic	Island	47.81	3.08	50.57	3.39	-2.76	1.05E-03	1.78E-02
cg17094593	RNMTL1	17	Body	Open sea	74.80	3.43	77.56	3.39	-2.76	7.03E-05	2.08E-03
cg01637090	CACNA1H;CACNA1H	16	Body;Body	N_Shelf	58.45	5.04	61.21	4.70	-2.76	3.89E-03	4.48E-02
cg18221341		13	Intergenic	Open sea	80.02	4.43	82.78	4.28	-2.76	3.32E-03	4.01E-02
cg25062251		14	Intergenic	Island	81.92	3.63	84.67	3.00	-2.76	1.93E-04	4.81E-03
cg07373877		5	Intergenic	Open sea	43.69	4.56	46.45	5.28	-2.76	2.68E-03	3.46E-02
cg23683290	KRI1	19	Body	N_Shore	60.46	3.80	63.22	3.95	-2.76	4.26E-03	4.76E-02
cg10672726	AP2S1;AP2S1	19	Body;Body	N_Shore	48.01	3.69	50.77	2.91	-2.76	1.32E-03	2.10E-02

cg22584802	AHCYL2;AHCYL2;AHCYL2;AHCYL2	7	Body;TSS200;Body;TSS200	Open sea	20.38	3.96	23.13	4.71	-2.76	1.77E-03	2.59E-02
cg14644065	PTRH1	9	Body	N_Shore	67.09	4.16	69.85	4.08	-2.76	9.92E-04	1.70E-02
cg25393429	LRP5	11	Body	Open sea	50.48	4.44	53.24	4.17	-2.76	1.40E-03	2.19E-02
cg27300194		1	Intergenic	Open sea	30.60	4.29	33.36	4.46	-2.76	1.26E-03	2.04E-02
cg10593713		10	Intergenic	Open sea	73.62	4.99	76.38	3.70	-2.76	2.97E-03	3.72E-02
cg14197113		4	Intergenic	Open sea	22.96	3.72	25.72	3.26	-2.76	1.21E-03	1.98E-02
cg00449021		1	Intergenic	Open sea	27.79	3.68	30.55	4.19	-2.76	3.36E-03	4.05E-02
cg12141056	FAM129A	1	TSS1500	S_Shore	73.76	4.58	76.52	4.99	-2.76	5.55E-04	1.10E-02
cg19628469	ELANE	19	Body	Island	72.80	3.25	75.56	3.88	-2.76	7.77E-04	1.42E-02
cg27522060	RASL12;RASL12	15	5'UTR;1stExon	Island	20.42	3.52	23.18	4.13	-2.76	2.67E-04	6.22E-03
cg01907761	SPINK5L3	5	TSS1500	Open sea	67.18	5.73	69.94	5.78	-2.76	4.49E-03	4.94E-02
cg13575682	SMCP	1	TSS200	Open sea	19.72	3.31	22.47	3.65	-2.76	2.83E-05	9.48E-04
cg00690929	SLC2A5;SLC2A5;SLC2A5	1	Body;Body;Body	Open sea	56.68	4.85	59.44	4.29	-2.76	1.88E-03	2.71E-02
cg17421805	FCRL6	1	Body	Open sea	66.46	4.57	69.22	3.84	-2.76	1.04E-03	1.77E-02
cg18925094	C21orf82;C21orf82	21	TSS1500;TSS1500	Open sea	83.95	4.08	86.71	3.72	-2.76	2.42E-03	3.23E-02
cg06697595	TRIM38	6	TSS1500	Open sea	79.90	3.86	82.66	3.64	-2.76	1.23E-03	2.00E-02
cg22040728	IL27RA	19	Body	S_Shelf	62.46	5.53	65.22	4.86	-2.76	4.36E-03	4.84E-02
cg04704169	USP29	19	TSS1500	S_Shore	77.20	4.18	79.95	4.58	-2.76	2.40E-03	3.21E-02
cg17971555	CLK3;CLK3	15	3'UTR;3'UTR	Open sea	75.53	3.04	78.29	2.89	-2.76	2.84E-04	6.52E-03
cg02171804		14	Intergenic	S_Shore	84.79	3.77	87.55	2.36	-2.76	1.06E-03	1.78E-02
cg03009158		1	Intergenic	Island	9.53	3.44	12.29	3.93	-2.76	6.81E-04	1.28E-02
cg07632946	BRD8;BRD8;BRD8;KIF20A	5	TSS1500;TSS1500;TSS1500;Body	S_Shore	26.49	3.70	29.25	3.17	-2.76	1.92E-03	2.75E-02
cg07355926		19	Intergenic	Island	53.20	3.66	55.96	3.38	-2.76	9.99E-05	2.80E-03
cg06760507	NDUFS2;ADAMTS4	1	TSS1500;1stExon	N_Shelf	45.00	3.31	47.76	3.38	-2.76	5.58E-04	1.10E-02
cg21994818		8	Intergenic	Open sea	36.32	5.32	39.07	5.05	-2.76	4.46E-03	4.91E-02
cg04346128	KCTD16	5	5'UTR	N_Shore	22.88	3.01	25.64	4.13	-2.75	2.74E-03	3.52E-02
cg05803237	MTNR1B	11	TSS200	N_Shore	38.60	3.61	41.35	3.74	-2.75	5.10E-04	1.03E-02
cg08292519		4	Intergenic	Open sea	72.20	2.63	74.95	2.95	-2.75	2.37E-05	8.14E-04
cg10213658		7	Intergenic	Open sea	61.45	5.53	64.20	4.37	-2.75	7.57E-04	1.39E-02
cg01688688	PCDHB18	5	Body	N_Shore	21.94	4.02	24.69	4.13	-2.75	1.91E-03	2.74E-02
cg21636911		5	Intergenic	Open sea	45.03	3.32	47.78	3.40	-2.75	2.94E-04	6.69E-03
cg23623681	TNXB	6	Body	Open sea	84.20	3.43	86.96	2.36	-2.75	9.66E-05	2.72E-03
cg04710179	TBC1D1	4	TSS1500	N_Shore	69.31	4.17	72.07	3.37	-2.75	2.43E-03	3.24E-02
cg15442268	ZBTB12	6	Body	Island	55.67	2.27	58.42	3.19	-2.75	7.62E-05	2.23E-03
cg22373687		3	Intergenic	Open sea	53.43	5.70	56.18	5.62	-2.75	2.81E-03	3.58E-02
cg20532999	MIR548H4;NOX5	15	Body;Body	Island	8.62	2.07	11.37	3.58	-2.75	4.72E-06	1.90E-04
cg06474661		17	Intergenic	S_Shore	43.53	3.97	46.28	4.00	-2.75	2.85E-03	3.61E-02
cg24668061	FMNL1	17	Body	Island	40.07	2.93	42.83	3.72	-2.75	1.26E-03	2.03E-02
cg20630207	TECR;MIR639	19	TSS200;TSS200	Island	12.83	3.23	15.59	3.59	-2.75	4.87E-04	9.92E-03
cg22056218	FERMT3;FERMT3	11	TSS1500;TSS1500	N_Shore	62.94	4.30	65.69	3.86	-2.75	6.07E-04	1.18E-02
cg15480367	CHGA;CHGA	14	5'UTR;1stExon	Island	25.83	3.82	28.58	4.28	-2.75	8.11E-04	1.46E-02
cg03270395	TRAF3IP2;TRAF3IP2;TRAF3IP2;TRAF3I	6	Body;Body;Body;TSS200;Body	Open sea	60.40	4.83	63.15	4.64	-2.75	2.27E-03	3.09E-02
cg03479209	FLNB;FLNB;FLNB;FLNB	3	Body;Body;Body;Body	Open sea	35.72	5.04	38.48	5.07	-2.75	2.82E-03	3.59E-02
cg26858581		11	Intergenic	S_Shelf	84.03	4.33	86.78	2.94	-2.75	8.88E-04	1.57E-02
cg09287190		13	Intergenic	N_Shore	38.78	3.25	41.53	3.89	-2.75	4.65E-04	9.57E-03
cg01995548	RPL13;RPL13	16	Body;Body	S_Shore	66.66	4.12	69.41	4.44	-2.75	1.17E-03	1.93E-02
cg27125645	DNAH10	12	TSS1500	N_Shore	34.05	5.40	36.80	5.71	-2.75	2.59E-03	3.38E-02
cg14192174	FAM179A	2	TSS200	Open sea	46.13	3.73	48.89	4.76	-2.75	1.68E-03	2.50E-02
cg23498106		2	Intergenic	Open sea	80.23	4.17	82.99	4.61	-2.75	1.75E-03	2.57E-02
cg07028598		2	Intergenic	Open sea	74.97	4.27	77.72	3.33	-2.75	7.95E-04	1.44E-02
cg02482690	KCTD17	22	Body	S_Shore	72.29	3.62	75.04	3.85	-2.75	4.60E-04	9.50E-03
cg27230044	SGCE;PEG10;SGCE;PEG10;SGCE	7	TSS1500;5'UTR;TSS1500;5'UTR;TSS1500	Island	35.46	4.17	38.22	4.53	-2.75	4.05E-03	4.61E-02
cg26878734	KIAA1715	2	3'UTR	Open sea	85.16	4.60	87.91	4.01	-2.75	2.45E-03	3.26E-02

cg06651452	ZBTB7A	19	5'UTR	N_Shore	55.30	3.37	58.05	4.13	-2.75	3.29E-03	3.99E-02
cg14904034	HPCAL1;HPCAL1	2	5'UTR;5'UTR	Open sea	81.55	3.20	84.30	2.70	-2.75	2.67E-05	9.01E-04
cg14359292	HOXA4	7	TSS1500	S_Shore	13.92	2.71	16.67	2.92	-2.75	2.23E-05	7.70E-04
cg16804747		6	Intergenic	Open sea	80.01	4.35	82.76	3.68	-2.75	1.42E-03	2.21E-02
cg23300257		8	Intergenic	Open sea	69.03	4.17	71.78	4.35	-2.75	1.31E-03	2.09E-02
cg07143303	MAD1L1;MAD1L1;MAD1L1	7	Body;Body;Body	Open sea	75.63	3.98	78.38	3.42	-2.75	3.43E-03	4.11E-02
cg16900102		7	Intergenic	Open sea	62.28	4.80	65.03	4.02	-2.75	2.87E-03	3.63E-02
cg16123202		7	Intergenic	Island	19.13	2.63	21.88	2.98	-2.75	4.90E-05	1.53E-03
cg25492458	DHX8	17	Body	S_Shelf	55.62	2.94	58.37	3.26	-2.75	2.55E-04	6.00E-03
cg11007075	PEBP4	8	Body	N_Shore	47.00	3.38	49.75	4.52	-2.75	1.52E-03	2.32E-02
cg00705808	SFRP2	4	TSS1500	N_Shore	19.42	3.64	22.17	4.03	-2.75	2.22E-03	3.04E-02
cg03313075		13	Intergenic	Open sea	41.03	4.75	43.78	2.56	-2.75	7.33E-04	1.36E-02
cg11450546	LYPD3	19	3'UTR	N_Shelf	60.30	2.82	63.05	2.97	-2.75	3.23E-05	1.06E-03
cg27073113	DKFZP434H168;GNAO1;GNAO1	16	TSS1500;Body;Body	S_Shelf	63.84	4.35	66.59	4.36	-2.75	9.92E-04	1.70E-02
cg18638769	PACS2;PACS2	14	Body;Body	Open sea	81.82	3.66	84.57	3.44	-2.75	4.85E-04	9.89E-03
cg00705613		12	Intergenic	N_Shore	63.21	4.85	65.96	4.40	-2.75	3.74E-03	4.36E-02
cg03496114		17	Intergenic	Island	19.35	3.28	22.10	3.48	-2.75	6.22E-05	1.88E-03
cg02973377	EPHX3;EPHX3	19	Body;Body	N_Shore	45.15	3.87	47.90	4.44	-2.75	3.73E-03	4.35E-02
cg03593057	DNAJC7;DNAJC7;CNP;DNAJC7	17	3'UTR;Body;3'UTR;3'UTR	Open sea	15.39	2.80	18.14	4.31	-2.75	2.97E-03	3.72E-02
cg20895511	INS-IGF2;IGF2;IGF2;IGF2;IGF2	11	Body;Body;5'UTR;5'UTR;1stExon	Island	26.65	3.50	29.40	3.68	-2.75	1.82E-03	2.64E-02
cg11701604	LDLRAD3	11	Body	Open sea	66.15	3.76	68.89	3.93	-2.75	7.62E-04	1.40E-02
cg03249020	FAM131B;FAM131B	7	TSS1500;TSS1500	Island	26.61	3.52	29.35	4.79	-2.75	1.53E-04	3.97E-03
cg01057132	DOCK2	5	Body	Open sea	60.83	5.53	63.58	5.28	-2.75	4.48E-03	4.93E-02
cg11034626	RSPH9	6	TSS200	Island	41.01	2.75	43.76	2.47	-2.75	1.25E-05	4.59E-04
cg22725222	TARBP2;TARBP2;TARBP2	12	Body;Body;Body	S_Shelf	81.33	3.18	84.08	3.09	-2.75	5.25E-04	1.05E-02
cg06893560		11	Intergenic	Open sea	58.37	4.65	61.12	3.68	-2.75	9.92E-04	1.70E-02
cg16782675	ATXN7;ATXN7	3	Body;Body	Open sea	82.37	4.17	85.12	3.45	-2.75	1.33E-03	2.12E-02
cg01494399	CMIP;CMIP	16	Body;Body	N_Shore	83.16	4.00	85.91	3.34	-2.75	1.09E-03	1.83E-02
cg06991974	FLJ42875;FLJ42875	1	Body;Body	Island	17.45	3.10	20.19	4.83	-2.75	2.99E-03	3.73E-02
cg17632372		7	Intergenic	Island	11.95	2.54	14.69	4.12	-2.75	5.48E-04	1.09E-02
cg09542154		4	Intergenic	Open sea	77.05	3.78	79.80	3.73	-2.75	4.44E-04	9.24E-03
cg04188555	AGAP1;AGAP1	2	Body;Body	Island	30.74	3.48	33.49	4.99	-2.75	1.03E-03	1.75E-02
cg21740507	IQCA1	2	Body	N_Shelf	66.03	3.41	68.77	3.67	-2.75	1.13E-03	1.88E-02
cg15536116	HCCA2;LOC402778	11	Body;TSS1500	Island	21.52	3.44	24.26	2.84	-2.75	2.31E-04	5.54E-03
cg05869169	PGAP3	17	3'UTR	S_Shelf	85.85	4.02	88.60	2.46	-2.75	1.05E-03	1.78E-02
cg12491966	COL18A1;COL18A1;COL18A1	21	Body;Body;Body	N_Shelf	86.61	3.19	89.35	2.11	-2.75	1.28E-06	5.68E-05
cg00916179	PLEKHH2	2	3'UTR	Open sea	79.46	4.18	82.20	3.26	-2.75	4.47E-04	9.30E-03
cg08459783	MXD4	4	Body	N_Shelf	70.52	4.27	73.26	3.27	-2.75	2.96E-03	3.71E-02
cg16911759	SH3D19;SH3D19;SH3D19	4	Body;Body;Body	Open sea	72.52	3.60	75.26	3.60	-2.75	1.13E-04	3.09E-03
cg01147107	TAL1	1	TSS1500	Island	26.32	3.51	29.06	4.24	-2.75	8.78E-04	1.55E-02
cg22079098	WWC2	4	Body	Open sea	75.48	3.91	78.23	3.68	-2.75	1.51E-04	3.94E-03
cg27125107		3	Intergenic	Open sea	81.10	4.94	83.84	4.72	-2.75	3.00E-03	3.74E-02
cg26229043	KIAA1217	10	5'UTR	Open sea	11.32	3.28	14.06	4.05	-2.74	1.47E-03	2.27E-02
cg19614627	RBPMS;RBPMS;RBPMS;RBPMS	8	Body;Body;Body;Body	Open sea	70.55	4.40	73.29	4.59	-2.74	1.29E-03	2.07E-02
cg17746119	CYGB	17	TSS200	Island	12.56	2.87	15.30	4.26	-2.74	4.65E-04	9.58E-03
cg25206709		5	Intergenic	Open sea	88.60	3.98	91.34	2.51	-2.74	9.17E-05	2.61E-03
cg20998791	TAPBP;TAPBP;TAPBP	6	Body;Body;Body	N_Shore	19.37	3.64	22.11	3.78	-2.74	2.96E-03	3.71E-02
cg21116812	TMPRSS9	19	TSS200	Open sea	67.42	4.81	70.16	3.52	-2.74	1.23E-03	2.00E-02
cg00090197	TMEM125	1	Body	Island	78.72	3.33	81.47	3.43	-2.74	7.89E-04	1.43E-02
cg07709980		16	Intergenic	N_Shore	34.62	3.25	37.36	3.51	-2.74	1.13E-04	3.10E-03
cg12338417	TRIM71	3	1stExon	Island	12.73	3.08	15.48	3.88	-2.74	3.03E-04	6.86E-03
cg24707890	LRTOMT;LRTOMT;LRTOMT;LRTOMT;L	11	5'UTR;5'UTR;Body;Body;Body;5'UTR	Open sea	31.03	3.81	33.77	3.21	-2.74	2.08E-03	2.91E-02
cg15805451	COBL	7	Body	Open sea	85.49	4.10	88.24	2.20	-2.74	1.82E-03	2.65E-02

cg25315816	SNRPF	12	TSS1500	Island	23.56	3.85	26.30	4.86	-2.74	1.51E-04	3.92E-03
cg03467014	SDF4;SDF4	1	Body;Body	N_Shore	91.13	4.16	93.88	2.09	-2.74	3.81E-04	8.20E-03
cg02979487	AFF1;AFF1	4	Body;Body	Open sea	19.72	3.31	22.46	4.19	-2.74	2.91E-03	3.66E-02
cg14112555	AHI1;AHI1;AHI1;AHI1	6	Body;Body;Body;Body	Open sea	75.11	3.08	77.85	3.33	-2.74	2.12E-04	5.17E-03
cg11071202		14	Intergenic	S_Shelf	61.91	4.52	64.65	3.12	-2.74	1.64E-03	2.45E-02
cg18905856	EGF	4	TSS1500	Open sea	87.12	4.02	89.86	2.28	-2.74	1.29E-03	2.07E-02
cg27509867		8	Intergenic	Open sea	37.40	2.85	40.14	3.51	-2.74	5.52E-04	1.09E-02
cg23461870	DLX2	2	Body	Island	32.32	3.64	35.06	4.37	-2.74	6.59E-04	1.25E-02
cg20467348	BRD4;BRD4	19	Body;Body	Open sea	77.11	4.63	79.85	3.28	-2.74	4.50E-03	4.94E-02
cg09533868	HEATR2	7	Body	Island	83.73	3.38	86.47	2.49	-2.74	6.86E-06	2.67E-04
cg04163533		17	Intergenic	Open sea	68.65	4.51	71.39	4.14	-2.74	2.65E-03	3.44E-02
cg14241370	GRIN3A	9	1stExon	Island	32.75	4.15	35.49	4.74	-2.74	4.16E-04	8.78E-03
cg09995596	SYNGR1;SYNGR1;SYNGR1	22	Body;TSS200;Body	Open sea	77.97	3.86	80.71	3.44	-2.74	5.14E-04	1.03E-02
cg00281664	ATP11A;ATP11A	13	Body;Body	S_Shore	64.26	4.06	67.00	3.06	-2.74	4.13E-03	4.67E-02
cg11326166		17	Intergenic	Open sea	63.12	3.76	65.86	3.29	-2.74	1.99E-03	2.82E-02
cg19147499		6	Intergenic	N_Shelf	66.61	3.91	69.35	3.10	-2.74	1.18E-03	1.94E-02
cg09487078	CCR6;CCR6	6	5'UTR;1stExon	Open sea	58.17	4.28	60.91	4.59	-2.74	7.68E-04	1.41E-02
cg13209298	SEMA3B;SEMA3B	3	TSS1500;TSS1500	Open sea	78.03	3.87	80.77	4.05	-2.74	1.85E-04	4.64E-03
cg10777851	CD200;CD200	3	TSS200;TSS200	N_Shore	14.25	4.00	16.99	4.75	-2.74	4.65E-04	9.59E-03
cg18589543	EIF2S2	20	Body	N_Shelf	64.70	4.29	67.44	4.82	-2.74	4.40E-03	4.87E-02
cg26290543		11	Intergenic	S_Shore	35.14	3.98	37.88	3.63	-2.74	2.27E-04	5.47E-03
cg18793215	CRABP2	1	Body	N_Shore	40.21	4.29	42.95	4.38	-2.74	2.92E-03	3.67E-02
cg19085507		4	Intergenic	N_Shelf	14.14	2.71	16.88	3.39	-2.74	1.40E-04	3.69E-03
cg15789723	GNL1	6	3'UTR	Open sea	54.58	3.51	57.32	2.64	-2.74	2.87E-04	6.56E-03
cg00808086		16	Intergenic	N_Shore	36.59	5.02	39.33	4.23	-2.74	2.00E-03	2.83E-02
cg12438547		13	Intergenic	Island	26.87	2.80	29.61	3.15	-2.74	2.29E-05	7.88E-04
cg26440289	SNX31	8	TSS200	Island	17.29	4.60	20.03	4.43	-2.74	2.51E-03	3.31E-02
cg03792839		12	Intergenic	S_Shelf	86.77	3.77	89.51	2.81	-2.74	2.78E-04	6.41E-03
cg08877374	FABP3;FABP3	1	1stExon;5'UTR	Open sea	60.29	4.06	63.03	4.64	-2.74	4.12E-03	4.66E-02
cg08484506		17	Intergenic	N_Shelf	77.97	4.36	80.71	3.93	-2.74	2.43E-03	3.24E-02
cg06079966		6	Intergenic	N_Shelf	84.81	4.16	87.55	3.17	-2.74	8.33E-04	1.49E-02
cg23049458	L1TD1;L1TD1	1	5'UTR;5'UTR	Island	20.89	4.07	23.62	3.54	-2.74	1.29E-03	2.06E-02
cg04117356	SPNS2	17	Body	S_Shelf	75.40	4.26	78.14	4.78	-2.74	1.03E-03	1.76E-02
cg13692446		13	Intergenic	Island	12.01	2.96	14.74	3.26	-2.74	3.50E-05	1.14E-03
cg07510303	ADAT2	6	3'UTR	Open sea	16.60	2.83	19.34	3.64	-2.74	9.61E-05	2.71E-03
cg02066518		2	Intergenic	Open sea	71.50	3.85	74.24	3.47	-2.74	3.00E-03	3.74E-02
cg23058719		4	Intergenic	Open sea	63.78	4.45	66.52	4.10	-2.74	1.01E-04	2.82E-03
cg03783126	LOC150568	2	Body	Open sea	81.26	4.34	84.00	3.43	-2.74	1.69E-03	2.51E-02
cg03954927	KIF1B;KIF1B	1	Body;Body	Open sea	86.43	3.82	89.17	3.91	-2.74	3.60E-04	7.85E-03
cg05256179	BAT5	6	Body	Open sea	51.85	3.99	54.58	5.05	-2.73	1.48E-04	3.87E-03
cg10095968	TRPM2	21	Body	S_Shelf	86.50	3.16	89.23	2.69	-2.73	1.93E-05	6.77E-04
cg24504289	TNXB	6	Body	N_Shelf	72.21	3.89	74.94	4.65	-2.73	1.85E-04	4.65E-03
cg17333291	GPER;C7orf50;C7orf50;GPER;C7orf50;	7	TSS200;Body;Body;TSS200;Body;TSS1500	N_Shore	68.33	3.61	71.06	3.39	-2.73	2.61E-03	3.40E-02
cg22855237		1	Intergenic	Open sea	63.36	4.21	66.10	4.28	-2.73	3.22E-03	3.93E-02
cg16771862	FANCD2;FANCD2	3	Body;Body	S_Shelf	60.72	3.28	63.45	2.75	-2.73	5.78E-04	1.13E-02
cg11112931	FGFR4;FGFR4;FGFR4	5	Body;Body;Body	Island	89.00	2.97	91.74	2.39	-2.73	5.97E-06	2.35E-04
cg02579477	RGS5	1	TSS1500	Open sea	74.64	5.47	77.38	5.19	-2.73	3.55E-03	4.21E-02
cg17918270	MYT1L	2	Body	Open sea	86.57	2.49	89.31	2.37	-2.73	7.19E-07	3.29E-05
cg22600043	RSPO2	8	TSS200	S_Shore	16.17	2.14	18.91	3.15	-2.73	1.72E-05	6.10E-04
cg19222574	PLXNA1	3	TSS1500	N_Shore	40.73	4.06	43.46	3.98	-2.73	4.95E-04	1.01E-02
cg15065108	AP2A2	11	Body	S_Shore	70.49	4.04	73.23	3.62	-2.73	1.69E-03	2.50E-02
cg02088673	DAGLA	11	Body	S_Shelf	57.94	4.21	60.68	3.47	-2.73	2.72E-03	3.50E-02
cg15630827		13	Intergenic	Island	84.25	3.49	86.99	2.76	-2.73	2.07E-04	5.08E-03

cg12718832	XRCC6	22	3'UTR	N_Shelf	89.59	1.95	92.32	1.41	-2.73	9.24E-11	5.35E-09
cg19036773	MLL3	7	Body	Open sea	41.67	4.35	44.40	4.32	-2.73	3.89E-03	4.48E-02
cg04671541	TAGLN3;TAGLN3;TAGLN3;TAGLN3;TA(	3	1stExon;5'UTR;TSS1500;1stExon;5'UTR	Open sea	44.22	5.69	46.96	5.24	-2.73	2.02E-03	2.85E-02
cg16017800	ARID1B;ARID1B;ARID1B	6	Body;Body;Body	Open sea	69.30	4.07	72.04	3.74	-2.73	2.42E-03	3.23E-02
cg24756966	C6orf154	6	TSS1500	S_Shore	84.80	3.97	87.54	3.23	-2.73	2.19E-03	3.01E-02
cg02423267		7	Intergenic	Open sea	79.54	4.06	82.27	3.05	-2.73	1.12E-03	1.87E-02
cg10240587	PRAMEF20	1	5'UTR	Open sea	65.37	4.51	68.10	4.01	-2.73	6.48E-04	1.24E-02
cg21457856		3	Intergenic	Open sea	75.94	3.50	78.67	3.05	-2.73	8.42E-04	1.50E-02
cg04974062	PRDM13	6	Body	S_Shore	30.62	3.48	33.35	4.42	-2.73	4.55E-03	4.98E-02
cg00503302		3	Intergenic	Island	13.24	2.74	15.97	3.00	-2.73	7.12E-05	2.10E-03
cg18800785		3	Intergenic	S_Shelf	80.72	3.99	83.45	3.99	-2.73	1.47E-03	2.27E-02
cg23958464		1	Intergenic	Open sea	52.47	4.30	55.20	5.40	-2.73	9.04E-04	1.59E-02
cg22167267	CCDC57	17	Body	Open sea	83.31	4.37	86.04	3.37	-2.73	3.13E-03	3.85E-02
cg15587745	TEKT4	2	TSS200	Island	45.34	3.38	48.07	3.11	-2.73	2.21E-03	3.03E-02
cg24675094	UBE2C;UBE2C;UBE2C;UBE2C;UBE2C;U	20	TSS200;TSS1500;TSS200;TSS200;TSS200;T:	Island	6.86	1.94	9.59	3.99	-2.73	1.23E-05	4.52E-04
cg18017849	TOLLIP	11	Body	Open sea	82.98	2.10	85.71	2.31	-2.73	1.43E-07	7.02E-06
cg23832237	RAB37	17	TSS200	Island	27.25	3.46	29.98	4.60	-2.73	3.10E-03	3.83E-02
cg18714224	EYA4;EYA4;EYA4	6	5'UTR;5'UTR;5'UTR	S_Shore	35.03	4.90	37.76	5.64	-2.73	3.18E-03	3.89E-02
cg02519218	CHFR;CHFR;CHFR;CHFR;CHFR	12	Body;Body;Body;Body;Body	Open sea	67.72	4.11	70.45	4.50	-2.73	1.02E-03	1.74E-02
cg23977011	OBSL1;INHA	2	TSS1500;1stExon	S_Shore	74.83	3.99	77.56	2.68	-2.73	2.87E-03	3.63E-02
cg16235582	ODZ4	11	Body	Island	26.36	3.12	29.09	3.12	-2.73	2.26E-05	7.79E-04
cg01421539		17	Intergenic	Open sea	50.39	5.30	53.12	5.63	-2.73	1.80E-03	2.63E-02
cg02309841	VGLL4	3	5'UTR	N_Shelf	37.75	2.80	40.48	4.77	-2.73	3.75E-03	4.37E-02
cg11859087		14	Intergenic	N_Shore	64.58	4.16	67.31	3.76	-2.73	1.53E-03	2.34E-02
cg04690319	ZW10	11	3'UTR	Open sea	84.44	4.69	87.17	2.99	-2.73	1.38E-03	2.17E-02
cg09330551	KLK15;KLK15;KLK15	19	Body;Body;Body	S_Shelf	70.17	4.96	72.90	4.33	-2.73	2.45E-03	3.25E-02
cg20360285	PKD1L1	7	TSS1500	Open sea	73.15	4.64	75.88	3.78	-2.73	6.56E-04	1.25E-02
cg03610439	MYST4	10	Body	Open sea	87.36	3.47	90.09	1.60	-2.73	5.00E-05	1.55E-03
cg08294568		11	Intergenic	N_Shore	16.20	3.65	18.93	3.22	-2.73	1.46E-04	3.81E-03
cg10463626	TNXB	6	Body	N_Shore	72.42	5.28	75.15	4.76	-2.73	1.01E-03	1.72E-02
cg02762818		16	Intergenic	S_Shelf	84.12	4.20	86.85	2.71	-2.73	3.40E-03	4.09E-02
cg01513392		20	Intergenic	Island	41.27	5.11	44.00	4.99	-2.73	4.23E-03	4.74E-02
cg25155679	PCDH9;PCDH9	13	Body;Body	Open sea	85.55	5.26	88.28	4.44	-2.73	1.54E-03	2.34E-02
cg05258757	ROPN1B	3	Body	S_Shelf	11.72	2.73	14.45	2.59	-2.73	1.10E-05	4.10E-04
cg01989731	YTHDC2	5	Body	Open sea	84.55	4.48	87.28	3.93	-2.73	2.49E-03	3.30E-02
cg27332575	BMP8A	1	Body	Open sea	56.82	5.18	59.54	4.57	-2.73	3.22E-03	3.93E-02
cg20297423	MESTIT1;MEST;MEST;MEST	7	TSS200;TSS1500;TSS200;5'UTR	Island	35.85	3.16	38.58	3.13	-2.73	2.37E-04	5.67E-03
cg14219442		1	Intergenic	Open sea	69.68	5.00	72.41	4.69	-2.73	1.44E-03	2.24E-02
cg03229033		1	Intergenic	Island	44.78	3.29	47.50	2.55	-2.73	7.17E-05	2.12E-03
cg00215851		4	Intergenic	Open sea	79.22	4.29	81.94	4.21	-2.73	3.76E-05	1.22E-03
cg14082645		9	Intergenic	N_Shore	18.58	3.53	21.31	4.29	-2.73	3.57E-03	4.22E-02
cg16706749	LOXL1	15	Body	S_Shelf	68.32	4.56	71.04	3.76	-2.73	3.22E-03	3.93E-02
cg15816742	SNAR-B1;SNAR-B2;SNAR-D	19	TSS1500;TSS1500;Body	Open sea	72.78	3.76	75.51	3.66	-2.73	3.39E-04	7.50E-03
cg21534264	CADPS;CADPS;CADPS	3	TSS1500;TSS1500;TSS1500	S_Shore	12.98	2.90	15.71	3.36	-2.73	7.84E-05	2.28E-03
cg15582257		12	Intergenic	S_Shelf	83.48	4.58	86.21	2.81	-2.73	3.64E-03	4.28E-02
cg24498488		1	Intergenic	S_Shelf	67.23	3.21	69.96	2.64	-2.73	7.53E-05	2.21E-03
cg07584765	PKP4;PKP4	2	Body;Body	Open sea	84.89	3.41	87.62	2.42	-2.73	1.26E-04	3.38E-03
cg04768479		6	Intergenic	Island	43.51	4.39	46.24	3.83	-2.73	4.16E-03	4.69E-02
cg14763104	CD81	11	Body	N_Shore	66.91	4.49	69.64	4.28	-2.73	2.26E-03	3.08E-02
cg01035260		15	Intergenic	Open sea	78.47	4.44	81.20	4.08	-2.73	1.14E-03	1.89E-02
cg01726393	LOC729991-MEF2B;LOC729991-MEF2	19	Body;5'UTR;TSS1500;Body	Island	35.46	6.43	38.19	4.91	-2.73	3.62E-03	4.27E-02
cg15236484		21	Intergenic	S_Shelf	79.67	4.69	82.40	4.29	-2.73	1.65E-03	2.46E-02
cg15758702	BAHCC1	17	Body	Island	54.92	4.28	57.64	4.45	-2.73	3.23E-03	3.94E-02

cg09985845		16	Intergenic	Open sea	10.40	2.17	13.13	2.73	-2.73	8.46E-07	3.84E-05
cg03099832	IL15;IL15	4	5'UTR;5'UTR	Open sea	82.55	4.64	85.27	3.35	-2.73	6.12E-04	1.18E-02
cg26157972		5	Intergenic	Open sea	74.53	3.89	77.26	3.28	-2.73	5.81E-04	1.14E-02
cg17642796		7	Intergenic	Open sea	44.10	4.01	46.82	5.36	-2.73	6.98E-04	1.31E-02
cg11109845	CUL1	7	TSS1500	N_Shore	62.58	6.34	65.30	6.66	-2.73	1.94E-03	2.77E-02
cg03105663	SEMA4B;SEMA4B	15	TSS1500;5'UTR	N_Shore	78.40	4.71	81.12	4.68	-2.73	3.95E-03	4.53E-02
cg00574913	ESF1	20	Body	N_Shelf	73.04	4.08	75.77	2.80	-2.73	2.83E-03	3.60E-02
cg24162603	EEFSEC	3	Body	Open sea	69.87	3.56	72.59	2.97	-2.73	4.87E-04	9.92E-03
cg04978426	SNORD111;SF3B3	16	TSS1500;Body	Open sea	81.81	4.87	84.54	4.27	-2.73	4.04E-03	4.59E-02
cg09387152		14	Intergenic	Open sea	68.66	5.17	71.38	3.38	-2.73	2.83E-03	3.60E-02
cg16642695	ADHFE1	8	TSS1500	N_Shore	34.36	4.06	37.09	4.11	-2.73	4.34E-03	4.82E-02
cg19129747		3	Intergenic	Open sea	73.04	4.08	75.76	4.32	-2.73	2.41E-03	3.22E-02
cg27531553	TAS1R1;TAS1R1;TAS1R1;NOL9;TAS1R1	1	1stExon;1stExon;1stExon;TSS1500;1stExon	S_Shore	70.21	3.70	72.93	3.82	-2.73	8.70E-04	1.54E-02
cg08119607	RAET1L	6	TSS1500	Open sea	17.86	3.31	20.59	4.82	-2.73	9.73E-04	1.68E-02
cg20953187	SLC6A20;SLC6A20	3	1stExon;1stExon	Island	15.19	3.64	17.92	3.16	-2.73	9.45E-05	2.67E-03
cg01912578	AMFR	16	Body	S_Shore	80.68	3.21	83.40	2.18	-2.73	7.34E-05	2.16E-03
cg12615879	SLC26A10	12	TSS1500	Island	28.68	4.62	31.40	5.57	-2.72	3.19E-03	3.90E-02
cg13422347	RNF126	19	Body	Island	86.28	3.75	89.00	3.14	-2.72	1.79E-04	4.52E-03
cg13826459	SERPINA1;SERPINA1;SERPINA1;SERPII	14	TSS1500;5'UTR;5'UTR;5'UTR;5'UTR;5'UTR;	Open sea	19.75	3.01	22.48	3.60	-2.72	2.04E-03	2.87E-02
cg12562132		11	Intergenic	S_Shelf	72.71	4.87	75.44	4.74	-2.72	3.16E-03	3.88E-02
cg12966367	RASSF1;RASSF1	3	TSS200;TSS200	Island	28.12	4.02	30.84	4.16	-2.72	1.91E-03	2.74E-02
cg07102450		16	Intergenic	N_Shelf	84.58	3.96	87.30	2.90	-2.72	4.08E-04	8.65E-03
cg14300730	SULT1C4	2	TSS200	Open sea	25.60	4.49	28.32	4.18	-2.72	3.42E-03	4.10E-02
cg21582373	PAX6;PAX6;PAX6	11	Body;Body;Body	N_Shore	37.91	3.69	40.63	3.93	-2.72	5.44E-04	1.08E-02
cg15928016	05-Mar	10	Body	Open sea	86.29	4.64	89.02	3.68	-2.72	3.66E-03	4.29E-02
cg02203656	GPNMB;GPNMB	7	TSS1500;TSS1500	N_Shore	59.47	5.03	62.20	5.85	-2.72	4.07E-03	4.62E-02
cg19122460	SLC38A3	3	3'UTR	Open sea	74.98	3.45	77.70	3.00	-2.72	7.27E-04	1.35E-02
cg15727287		5	Intergenic	Island	18.71	3.65	21.43	4.33	-2.72	2.20E-03	3.02E-02
cg23154064	C3orf63;C3orf63	3	Body;TSS1500	Open sea	81.11	2.54	83.83	2.44	-2.72	2.43E-05	8.30E-04
cg23679141	MARCH1;ANP32C	4	5'UTR;TSS200	Open sea	15.98	3.14	18.70	3.47	-2.72	7.49E-04	1.38E-02
cg01202731	TMEM182	2	TSS1500	Open sea	80.84	4.44	83.56	4.36	-2.72	1.09E-03	1.83E-02
cg18295068	SCN3A;SCN3A;SCN3A;SCN3A;SCN3A;S	2	5'UTR;5'UTR;1stExon;1stExon;5'UTR;1stEx	Open sea	16.95	4.65	19.67	5.01	-2.72	3.15E-03	3.87E-02
cg26810767		16	Intergenic	Open sea	69.49	4.68	72.21	3.56	-2.72	7.51E-05	2.20E-03
cg00543443	LOC647979	20	Body	Island	26.66	2.34	29.38	2.41	-2.72	7.68E-06	2.96E-04
cg10419709		17	Intergenic	N_Shore	42.39	4.00	45.11	4.06	-2.72	5.24E-04	1.05E-02
cg07143451		17	Intergenic	Island	12.75	3.95	15.47	4.57	-2.72	2.03E-03	2.85E-02
cg03309938	HSPA8;HSPA8	11	Body;Body	N_Shore	36.42	3.35	39.14	3.97	-2.72	4.66E-04	9.59E-03
cg09276072	TSNAXIP1	16	Body	Open sea	77.51	3.33	80.23	2.84	-2.72	7.91E-05	2.30E-03
cg16240751		2	Intergenic	Island	81.75	3.43	84.47	3.37	-2.72	4.60E-04	9.50E-03
cg01250141		7	Intergenic	N_Shore	82.78	4.53	85.50	3.48	-2.72	1.34E-03	2.13E-02
cg25141995	VDAC1	5	TSS1500	Island	53.10	4.29	55.82	4.37	-2.72	4.17E-03	4.70E-02
cg23398720	B4GALT7	5	Body	N_Shore	62.69	4.03	65.41	3.57	-2.72	2.97E-03	3.72E-02
cg21020855	FABP7	6	TSS1500	Open sea	79.34	4.37	82.06	4.14	-2.72	2.21E-03	3.03E-02
cg12152352	TPCN2	11	3'UTR	Open sea	87.12	4.02	89.84	2.98	-2.72	1.71E-03	2.52E-02
cg11428886		11	Intergenic	Open sea	72.37	5.54	75.09	4.64	-2.72	1.89E-03	2.71E-02
cg05362850		7	Intergenic	N_Shore	62.79	4.36	65.51	4.69	-2.72	1.81E-03	2.64E-02
cg25713411	KANK2;KANK2	19	Body;Body	Island	78.15	3.95	80.87	2.96	-2.72	5.95E-04	1.16E-02
cg22512651	POU2F2	19	Body	S_Shelf	82.88	3.46	85.60	3.24	-2.72	3.72E-04	8.04E-03
cg08088171	CANT1;CANT1;CANT1	17	5'UTR;5'UTR;5'UTR	N_Shelf	82.73	4.60	85.45	3.23	-2.72	2.25E-03	3.07E-02
cg23103346	NAT8	2	TSS1500	Open sea	14.00	2.97	16.72	4.27	-2.72	3.15E-03	3.87E-02
cg19220282	SLC1A4;SLC1A4	2	Body;5'UTR	S_Shore	70.56	5.23	73.28	5.74	-2.72	3.17E-03	3.89E-02
cg04511104		17	Intergenic	S_Shore	64.72	4.98	67.44	5.15	-2.72	3.40E-03	4.09E-02
cg27600794	PNLIPRP1	10	TSS1500	Open sea	70.32	3.69	73.04	3.41	-2.72	1.52E-03	2.33E-02

cg14442890	BTN1A1	6	Body	S_Shore	28.04	4.01	30.76	4.20	-2.72	2.64E-03	3.43E-02
cg26040060	CYTH1;CYTH1	17	Body;Body	Open sea	82.42	3.22	85.14	2.60	-2.72	1.76E-04	4.46E-03
cg25431366	UBE2F	2	Body	Open sea	80.15	4.07	82.87	3.05	-2.72	1.22E-03	1.99E-02
cg12005811	GPR125	4	Body	Open sea	85.75	2.94	88.46	2.72	-2.72	1.29E-05	4.75E-04
cg24751155		17	Intergenic	Open sea	69.70	4.17	72.41	3.36	-2.72	1.81E-03	2.64E-02
cg06625730	POLR3F	20	Body	Open sea	83.05	4.19	85.77	3.65	-2.72	1.84E-03	2.67E-02
cg04109781	AFF3;AFF3;AFF3	2	5'UTR;1stExon;5'UTR	S_Shore	41.06	4.61	43.78	4.18	-2.72	1.73E-03	2.55E-02
cg01592801	KCNS2	8	TSS1500	Island	33.26	4.17	35.98	4.69	-2.72	1.35E-04	3.59E-03
cg06884254	LRRN4CL	11	3'UTR	N_Shore	82.69	3.56	85.40	3.57	-2.72	3.31E-04	7.35E-03
cg04592728	SLC6A5	11	TSS1500	S_Shore	26.72	4.69	29.44	5.18	-2.72	3.34E-03	4.03E-02
cg24145235	ELMOD1;ELMOD1	11	3'UTR;3'UTR	Open sea	81.53	4.15	84.24	3.30	-2.72	2.83E-03	3.59E-02
cg26140790	LHPP;LHPP	10	Body;Body	N_Shelf	51.61	3.34	54.33	3.55	-2.72	7.27E-05	2.14E-03
cg17480760	CA3	8	TSS200	Island	18.01	4.59	20.72	4.67	-2.72	1.03E-03	1.75E-02
cg12574576		10	Intergenic	Open sea	72.60	4.46	75.32	3.54	-2.72	1.12E-03	1.87E-02
cg05592292		2	Intergenic	Open sea	44.37	3.60	47.09	3.62	-2.72	1.01E-03	1.72E-02
cg00449930		16	Intergenic	Open sea	77.31	4.07	80.03	3.45	-2.72	2.01E-03	2.84E-02
cg26199241		17	Intergenic	Island	16.12	2.97	18.84	2.85	-2.72	7.62E-05	2.23E-03
cg23553576	SYTL2;SYTL2;SYTL2	11	Body;Body;Body	Open sea	77.83	4.97	80.54	3.59	-2.72	1.30E-03	2.08E-02
cg27051231		2	Intergenic	Open sea	71.66	4.58	74.37	4.27	-2.71	3.78E-03	4.39E-02
cg12003483	ETV4;ETV4	17	TSS1500;TSS1500	Island	10.48	1.70	13.20	2.43	-2.71	1.40E-08	7.41E-07
cg07734975		11	Intergenic	S_Shore	66.32	4.99	69.03	4.06	-2.71	4.51E-03	4.95E-02
cg20060759	THAP3;DNAJC11	1	3'UTR;3'UTR	Open sea	77.01	4.05	79.73	3.30	-2.71	4.33E-03	4.82E-02
cg19250799		1	Intergenic	Island	14.12	4.10	16.83	4.82	-2.71	3.78E-04	8.15E-03
cg03547355		1	Intergenic	Open sea	43.53	4.98	46.25	4.45	-2.71	3.63E-03	4.27E-02
cg21464289		4	Intergenic	N_Shelf	66.00	3.92	68.71	3.64	-2.71	7.72E-04	1.41E-02
cg26580413	FTO	16	Body	Open sea	60.44	7.16	63.15	6.27	-2.71	3.55E-03	4.21E-02
cg13267092	FLNC;FLNC	7	Body;Body	S_Shelf	73.01	4.31	75.72	4.93	-2.71	1.16E-03	1.92E-02
cg27275374	TNS3	7	5'UTR	Open sea	30.99	3.57	33.71	4.32	-2.71	1.71E-03	2.53E-02
cg16008440	OLFML2B	1	Body	Open sea	77.06	4.58	79.77	3.86	-2.71	2.74E-03	3.52E-02
cg00851303		16	Intergenic	N_Shelf	41.53	3.74	44.24	3.37	-2.71	9.98E-04	1.71E-02
cg12245876	FRMD8	11	Body	S_Shore	74.09	3.71	76.80	3.40	-2.71	3.38E-04	7.47E-03
cg08435945	LASS6	2	Body	Open sea	82.48	3.28	85.19	3.04	-2.71	7.46E-04	1.37E-02
cg02212389	NUB1	7	Body	Open sea	73.28	3.40	75.99	3.10	-2.71	1.19E-03	1.95E-02
cg05920909	LHX9;LHX9	1	Body;Body	N_Shore	26.15	4.39	28.86	3.42	-2.71	7.44E-04	1.37E-02
cg24428144		12	Intergenic	Open sea	46.68	5.42	49.39	4.48	-2.71	9.91E-04	1.70E-02
cg19218492	MGAT4C	12	5'UTR	Open sea	80.66	3.30	83.37	3.04	-2.71	1.14E-04	3.11E-03
cg01455568		7	Intergenic	Island	18.47	2.21	21.18	2.59	-2.71	1.80E-06	7.77E-05
cg04064327	LOC646982;LOC646982;LOC646982	13	TSS200;TSS200;TSS200	Open sea	81.47	3.22	84.18	3.01	-2.71	2.37E-04	5.66E-03
cg11095383	PCDHA1;PCDHA1;PCDHA2;PCDHA2	5	Body;Body;1stExon;1stExon	Island	17.33	3.59	20.04	4.15	-2.71	4.42E-03	4.88E-02
cg22840216		12	Intergenic	Open sea	55.47	3.91	58.18	4.87	-2.71	1.47E-03	2.27E-02
cg11032268	FSCN1	7	TSS200	N_Shore	7.29	2.08	10.00	3.52	-2.71	9.48E-08	4.74E-06
cg19956889	USP6NL	10	5'UTR	N_Shelf	79.46	5.52	82.17	4.01	-2.71	2.01E-03	2.84E-02
cg16897579	RAD54L2	3	5'UTR	Open sea	86.29	4.31	89.00	3.95	-2.71	3.28E-03	3.99E-02
cg15948836	LDLRAD3	11	Body	Open sea	36.60	4.23	39.31	3.95	-2.71	1.94E-03	2.77E-02
cg26363955		2	Intergenic	Open sea	87.49	3.71	90.20	2.40	-2.71	2.17E-04	5.27E-03
cg12676702	DLX1;DLX1	2	3'UTR;3'UTR	S_Shore	23.18	3.02	25.88	4.46	-2.71	3.81E-04	8.21E-03
cg18749629	ZC3H3	8	Body	S_Shore	90.58	2.87	93.29	2.51	-2.71	3.83E-06	1.56E-04
cg07695566	CYB561	17	TSS1500	S_Shore	69.03	3.67	71.74	3.67	-2.71	2.03E-03	2.85E-02
cg03941587	FGF2	4	TSS1500	N_Shore	54.41	4.23	57.12	3.52	-2.71	1.36E-03	2.15E-02
cg23072161		1	Intergenic	Open sea	55.84	4.96	58.55	5.21	-2.71	3.80E-03	4.40E-02
cg11948905		5	Intergenic	Open sea	76.08	4.04	78.78	3.69	-2.71	2.57E-04	6.03E-03
cg08364860	RAB18	10	3'UTR	Open sea	84.21	4.08	86.92	3.02	-2.71	8.62E-04	1.53E-02
cg19709928	CCDC111	4	Body	Open sea	60.64	3.69	63.34	2.70	-2.71	9.87E-04	1.69E-02

cg10427430	TEAD1	11	5'UTR	Open sea	76.38	3.93	79.09	3.53	-2.71	2.56E-04	6.02E-03
cg07636688	SMC1B;RIBC2	22	1stExon;TSS200	Island	17.85	3.52	20.56	3.38	-2.71	3.41E-04	7.52E-03
cg22023169		15	Intergenic	Open sea	81.71	3.73	84.42	3.49	-2.71	2.34E-04	5.60E-03
cg04374393	SOX14	3	TSS200	Island	32.35	2.47	35.06	4.02	-2.71	5.01E-04	1.01E-02
cg16739679	ZNF720	16	TSS1500	N_Shore	37.65	3.66	40.36	3.37	-2.71	3.15E-03	3.87E-02
cg22779845		5	Intergenic	Open sea	49.77	4.95	52.48	4.38	-2.71	3.80E-03	4.40E-02
cg20357807	PLEK	2	TSS1500	Open sea	62.60	4.17	65.31	3.59	-2.71	1.42E-03	2.21E-02
cg10441497	CHST11	12	Body	Open sea	87.09	4.25	89.80	4.17	-2.71	4.60E-04	9.51E-03
cg12249537	IMPG2	3	Body	Open sea	83.65	3.66	86.36	3.44	-2.71	4.68E-04	9.63E-03
cg26308012	TRAPPC9;TRAPPC9	8	Body;Body	Island	49.74	3.94	52.45	4.41	-2.71	1.34E-03	2.12E-02
cg19584038	C19orf28;C19orf28;C19orf28	19	Body;Body;Body	N_Shelf	59.14	4.10	61.84	4.23	-2.71	6.98E-04	1.31E-02
cg09370594	LINGO3	19	5'UTR	Island	42.53	3.61	45.24	4.10	-2.71	4.52E-03	4.96E-02
cg15933451	ZAP70	2	TSS200	Open sea	42.85	3.44	45.56	4.04	-2.71	6.74E-04	1.27E-02
cg00583535	IGFN1	1	TSS200	Open sea	41.34	4.05	44.05	3.80	-2.71	9.83E-04	1.69E-02
cg16597665	RNF2	1	TSS1500	N_Shore	78.61	5.21	81.31	3.94	-2.71	3.35E-03	4.05E-02
cg06808571	KCNH2;KCNH2	7	3'UTR;3'UTR	N_Shelf	53.23	3.93	55.94	3.92	-2.71	1.51E-03	2.31E-02
cg19344315	ATXN1;ATXN1	6	5'UTR;5'UTR	Open sea	73.81	4.77	76.52	6.58	-2.71	1.58E-03	2.40E-02
cg18555073	B2M	15	TSS1500	N_Shore	34.21	4.74	36.92	5.55	-2.71	1.49E-03	2.30E-02
cg05152503	MYLK2	20	TSS200	N_Shelf	79.42	3.61	82.13	3.11	-2.71	6.73E-04	1.27E-02
cg02392269	PARVA	11	Body	Open sea	88.95	3.51	91.66	2.84	-2.71	2.57E-04	6.03E-03
cg04956624		11	Intergenic	Open sea	27.67	3.45	30.38	3.97	-2.71	4.16E-03	4.69E-02
cg20637438	XKR6	8	Body	Open sea	68.76	4.15	71.47	4.22	-2.71	9.29E-04	1.62E-02
cg08926253	IRF7;IRF7;IRF7	11	Body;Body;Body	Island	29.37	3.31	32.07	3.97	-2.70	1.82E-03	2.64E-02
cg06236061	GLTP	12	Body	N_Shore	15.47	3.77	18.18	4.06	-2.70	2.68E-04	6.23E-03
cg10647445		4	Intergenic	Open sea	75.06	3.51	77.77	3.30	-2.70	4.52E-04	9.37E-03
cg04140161		13	Intergenic	Island	80.53	3.87	83.23	2.81	-2.70	1.24E-03	2.01E-02
cg19438261	IMPA1;IMPA1;IMPA1	8	TSS1500;TSS1500;TSS1500	S_Shore	56.74	3.62	59.44	3.36	-2.70	2.62E-03	3.41E-02
cg00480389	CBLN4	20	TSS200	Island	13.71	2.64	16.42	2.46	-2.70	9.01E-06	3.43E-04
cg08290471		16	Intergenic	Open sea	74.97	3.97	77.67	4.06	-2.70	4.52E-05	1.42E-03
cg18383660	HCRTR1	1	TSS1500	N_Shore	65.41	4.26	68.11	4.40	-2.70	1.37E-03	2.16E-02
cg16612998	LRP4	11	TSS1500	S_Shore	22.46	4.10	25.16	4.74	-2.70	3.30E-03	4.00E-02
cg23348764	PCDHGA4;PCDHGA2;PCDHGB2;PCDH(	5	Body;Body;Body;Body;TSS200;TSS200;Boc	N_Shore	15.48	4.01	18.18	3.96	-2.70	1.38E-03	2.17E-02
cg07534554	POU2AF1	11	TSS200	Open sea	40.37	4.26	43.08	4.80	-2.70	2.27E-03	3.09E-02
cg18802998		3	Intergenic	Open sea	64.97	4.31	67.68	4.01	-2.70	1.05E-03	1.77E-02
cg12303610	TRABD	22	Body	Island	69.02	4.43	71.72	4.41	-2.70	3.74E-03	4.36E-02
cg27143870		3	Intergenic	S_Shelf	71.99	4.93	74.70	4.88	-2.70	1.51E-03	2.31E-02
cg25078137	HOXB9	17	3'UTR	N_Shore	21.60	3.94	24.30	3.59	-2.70	3.37E-03	4.06E-02
cg23884149	NXN	17	Body	S_Shelf	76.58	4.82	79.28	5.25	-2.70	4.52E-03	4.96E-02
cg03260166	LOC388796;LOC388796;SNORA71C	20	Body;Body;TSS1500	N_Shelf	82.10	4.47	84.80	3.94	-2.70	1.21E-03	1.97E-02
cg24577933		1	Intergenic	Island	37.51	4.92	40.21	4.72	-2.70	2.86E-03	3.62E-02
cg06119016	C17orf101;C17orf101	17	Body;Body	Island	77.76	4.53	80.46	3.35	-2.70	4.50E-03	4.95E-02
cg15112910		1	Intergenic	Open sea	25.02	4.10	27.72	3.84	-2.70	3.54E-03	4.20E-02
cg26153631	HOXC10	12	1stExon	Island	35.82	2.20	38.52	3.13	-2.70	6.52E-05	1.95E-03
cg23146358	CDKN1C;CDKN1C;CDKN1C	11	Body;Body;Body	Island	13.09	5.01	15.79	4.33	-2.70	3.40E-04	7.51E-03
cg09725286		8	Intergenic	Open sea	22.83	3.76	25.53	3.65	-2.70	3.91E-04	8.36E-03
cg20610915		6	Intergenic	Open sea	17.29	2.73	19.99	4.03	-2.70	2.16E-03	2.98E-02
cg16057598	DLX2	2	TSS1500	Island	11.76	2.36	14.46	3.06	-2.70	7.84E-07	3.57E-05
cg05769106	LOC285501	4	TSS200	Open sea	59.89	5.23	62.59	5.08	-2.70	3.16E-03	3.88E-02
cg26608305	AP2A2	11	TSS1500	Island	68.06	4.05	70.76	3.87	-2.70	3.33E-03	4.03E-02
cg06830148	RPL3L	16	TSS200	Open sea	74.86	3.57	77.56	3.90	-2.70	1.33E-04	3.53E-03
cg03181117	SCARF1;SCARF1;SCARF1;SCARF1;SCAI	17	Body;Body;Body;Body;Body	N_Shelf	69.60	3.41	72.30	3.45	-2.70	2.96E-04	6.73E-03
cg21706229		17	Intergenic	Island	17.96	3.08	20.66	3.98	-2.70	8.18E-04	1.47E-02
cg15608483		2	Intergenic	N_Shore	22.08	3.09	24.78	3.66	-2.70	3.24E-04	7.23E-03

cg21520002	SUDS3	12	1stExon	Island	21.97	2.95	24.67	2.85	-2.70	3.32E-05	1.09E-03
cg20080702		10	Intergenic	Open sea	68.15	3.58	70.85	3.33	-2.70	9.31E-04	1.62E-02
cg10165167	SLIT3	5	TSS1500	S_Shore	20.37	4.14	23.07	3.08	-2.70	3.23E-03	3.94E-02
cg00446803	FAM83H	8	5'UTR	N_Shore	80.58	3.50	83.28	3.03	-2.70	2.67E-03	3.46E-02
cg19118904	DNAJB6	7	Body	Open sea	64.82	5.37	67.52	6.10	-2.70	3.23E-03	3.94E-02
cg21691893	TNXB	6	Body	Open sea	54.01	4.03	56.71	4.24	-2.70	1.13E-04	3.10E-03
cg22668740		12	Intergenic	S_Shelf	38.39	2.99	41.09	4.06	-2.70	4.27E-03	4.77E-02
cg17180237		1	Intergenic	S_Shore	27.17	4.81	29.87	5.71	-2.70	2.01E-03	2.84E-02
cg06837040		1	Intergenic	Open sea	71.37	4.28	74.07	4.85	-2.70	4.42E-04	9.22E-03
cg22743002		6	Intergenic	S_Shore	56.17	4.68	58.87	5.63	-2.70	3.06E-03	3.79E-02
cg25399333		13	Intergenic	Open sea	85.70	3.90	88.40	3.20	-2.70	9.32E-04	1.63E-02
cg08846870	RASAL3	19	Body	Island	18.47	3.33	21.17	3.55	-2.70	1.32E-03	2.11E-02
cg00289258		21	Intergenic	Island	19.86	3.41	22.56	3.88	-2.70	1.39E-05	5.06E-04
cg23132624	KL	13	TSS200	Island	16.06	3.79	18.76	4.56	-2.70	3.75E-04	8.10E-03
cg14380270	SLFN11;SLFN11;SLFN11;SLFN11;SLFN1	17	TSS200;TSS200;TSS200;TSS200;TSS200	Island	16.69	3.56	19.39	3.84	-2.70	1.19E-04	3.22E-03
cg24021113	C6orf145	6	TSS1500	S_Shore	8.49	2.80	11.18	2.87	-2.70	2.27E-05	7.82E-04
cg09666444	OSGIN1;OSGIN1;OSGIN1	16	Body;Body;Body	N_Shelf	82.87	3.89	85.57	3.24	-2.70	2.88E-03	3.63E-02
cg01101448		2	Intergenic	Open sea	82.62	5.36	85.32	3.98	-2.70	3.71E-03	4.33E-02
cg18202127		7	Intergenic	Open sea	65.94	3.60	68.64	3.80	-2.70	3.81E-04	8.21E-03
cg10934032	RNASE6;RNASE6	14	1stExon;5'UTR	Open sea	40.28	4.13	42.98	5.21	-2.70	3.31E-03	4.01E-02
cg23786205		10	Intergenic	S_Shelf	66.10	4.69	68.80	4.51	-2.70	2.33E-03	3.14E-02
cg08454125	TMIE	3	Body	Open sea	62.35	4.76	65.05	4.14	-2.70	1.84E-03	2.67E-02
cg21157725	VAV2;VAV2	9	Body;Body	Open sea	80.74	3.69	83.44	2.84	-2.70	8.36E-04	1.50E-02
cg10586837		10	Intergenic	Open sea	70.60	3.89	73.29	4.06	-2.70	1.46E-03	2.26E-02
cg06497674	PIP5K1C	19	Body	N_Shelf	87.59	4.03	90.29	2.19	-2.70	2.50E-03	3.31E-02
cg26009554		2	Intergenic	Open sea	82.01	3.00	84.70	2.82	-2.70	1.23E-04	3.31E-03
cg00567930		1	Intergenic	S_Shore	80.31	3.98	83.01	3.87	-2.70	4.22E-03	4.73E-02
cg26920864		16	Intergenic	Open sea	60.49	3.89	63.19	3.71	-2.70	1.48E-03	2.28E-02
cg16717713	CCDC85C	14	1stExon	Island	19.82	3.16	22.52	3.33	-2.70	9.86E-04	1.69E-02
cg03285428	PPP4R2	3	Body	S_Shelf	79.68	3.87	82.38	3.19	-2.70	2.31E-03	3.13E-02
cg14548962	HSDL2	9	Body	Open sea	85.97	4.89	88.67	4.34	-2.70	4.48E-03	4.94E-02
cg00036011	HIST1H3G;HIST1H2BI	6	TSS200;TSS1500	N_Shore	17.00	3.34	19.70	3.87	-2.70	2.81E-04	6.47E-03
cg17716699	UNC84A;UNC84A	7	Body;Body	S_Shore	80.69	3.63	83.39	2.72	-2.70	3.57E-04	7.79E-03
cg11456013		6	Intergenic	N_Shelf	84.45	3.99	87.15	2.51	-2.70	9.69E-04	1.67E-02
cg09408780		6	Intergenic	Open sea	70.40	3.81	73.10	3.37	-2.70	2.71E-03	3.49E-02
cg08299783	GTF2A1L;LHCGR	2	Body;Body	Open sea	17.25	3.18	19.94	2.85	-2.70	2.71E-04	6.29E-03
cg16771771		6	Intergenic	S_Shore	77.96	3.98	80.66	3.82	-2.70	8.30E-04	1.49E-02
cg06434738	FTO	16	Body	Open sea	77.47	4.73	80.17	4.76	-2.70	4.02E-03	4.58E-02
cg08243931		5	Intergenic	Open sea	75.23	3.35	77.92	2.98	-2.70	3.25E-05	1.07E-03
cg07997493	NEUROD2	17	Body	Island	13.87	2.57	16.57	3.53	-2.70	2.93E-06	1.22E-04
cg00253564	PLEKHG5;PLEKHG5;PLEKHG5	1	Body;Body;TSS1500	S_Shore	71.97	4.13	74.67	2.91	-2.70	1.24E-03	2.01E-02
cg04418585	DLG2	11	Body	Open sea	89.37	2.91	92.07	2.21	-2.69	9.54E-07	4.30E-05
cg05711235		8	Intergenic	Island	28.63	3.72	31.33	5.28	-2.69	3.21E-03	3.93E-02
cg24200426	PRMT1;PRMT1;PRMT1	19	Body;Body;Body	S_Shore	71.31	3.93	74.00	3.64	-2.69	3.28E-03	3.98E-02
cg23893898		4	Intergenic	Island	15.63	2.68	18.32	3.36	-2.69	3.39E-04	7.49E-03
cg21105787	SPOCK2;SPOCK2	10	Body;Body	Island	16.65	3.09	19.34	4.26	-2.69	2.95E-04	6.71E-03
cg23947251		14	Intergenic	Open sea	77.22	5.25	79.91	4.80	-2.69	3.04E-03	3.78E-02
cg08077337	FZD7	2	1stExon	Island	21.52	5.78	24.21	5.48	-2.69	3.63E-03	4.27E-02
cg01054891	PEG3;PEG3;ZIM2;ZIM2;PEG3;ZIM2;MI	19	TSS1500;TSS1500;TSS1500;TSS1500;TSS15	S_Shore	37.86	3.80	40.55	3.72	-2.69	1.80E-03	2.63E-02
cg26399903		1	Intergenic	N_Shelf	80.89	3.51	83.58	4.14	-2.69	2.90E-04	6.63E-03
cg02274336	OR2H1	6	5'UTR	Open sea	81.94	4.67	84.64	3.01	-2.69	2.97E-03	3.72E-02
cg18480781		15	Intergenic	N_Shore	26.94	3.06	29.63	2.43	-2.69	7.90E-05	2.30E-03
cg17263206		5	Intergenic	S_Shore	54.84	6.06	57.53	6.70	-2.69	3.35E-03	4.04E-02

cg10620866	HLA-DOA	6	Body	Island	84.71	3.97	87.40	3.82	-2.69	2.02E-04	4.97E-03
cg11844927	TNFRSF1B	1	Body	Open sea	69.22	4.01	71.91	2.83	-2.69	9.72E-04	1.68E-02
cg02830467	MYCT1	6	TSS1500	Open sea	67.41	5.15	70.10	5.35	-2.69	6.21E-04	1.20E-02
cg16265707	NCALD;NCALD;NCALD;NCALD;NCALD;	8	5'UTR;5'UTR;5'UTR;5'UTR;5'UTR;5'UTR;TS	Open sea	83.98	3.49	86.67	2.49	-2.69	7.88E-04	1.43E-02
cg11544138	GRIN3B	19	Body	Island	67.58	3.90	70.27	3.44	-2.69	1.84E-03	2.67E-02
cg15618978	TRIM59	3	TSS1500	Island	19.60	3.03	22.29	3.21	-2.69	8.86E-05	2.53E-03
cg25860795	FLI1;FLI1	11	TSS1500;5'UTR	Island	13.82	3.14	16.51	3.89	-2.69	8.87E-04	1.57E-02
cg07009563	PIK3C2B	1	3'UTR	Open sea	82.92	3.41	85.61	3.01	-2.69	4.82E-05	1.50E-03
cg26118435	RBMS1;RBMS1	2	TSS1500;TSS1500	S_Shore	18.32	4.07	21.02	3.85	-2.69	2.60E-03	3.39E-02
cg18993626	STAU2;STAU2;STAU2;STAU2;STAU2;S	8	Body;Body;Body;3'UTR;3'UTR;Body;3'UTR	Open sea	89.79	2.47	92.49	1.24	-2.69	7.20E-09	3.86E-07
cg21242918	VAX1;VAX1	10	Body;Body	Island	16.58	3.11	19.27	4.01	-2.69	5.11E-04	1.03E-02
cg15548101	DGKG;DGKG;DGKG	3	5'UTR;5'UTR;5'UTR	N_Shore	19.90	2.90	22.59	3.89	-2.69	1.14E-04	3.13E-03
cg00479240	KIAA1462	10	5'UTR	Island	60.39	3.85	63.08	4.29	-2.69	2.94E-04	6.69E-03
cg12549079	ALOX12P2	17	TSS200	Open sea	70.98	3.17	73.67	2.80	-2.69	5.82E-05	1.77E-03
cg22742060		2	Intergenic	Open sea	58.92	4.82	61.61	4.95	-2.69	3.68E-03	4.31E-02
cg19698403		15	Intergenic	Open sea	21.23	3.57	23.92	3.22	-2.69	3.49E-03	4.16E-02
cg01152302	ADAMTSL3	15	5'UTR	Island	7.72	3.07	10.41	3.97	-2.69	4.19E-03	4.71E-02
cg11692021	GNB3;GNB3	12	1stExon;5'UTR	Open sea	51.03	4.23	53.72	3.63	-2.69	2.88E-03	3.64E-02
cg13038241	FSCN1	7	TSS200	N_Shore	14.10	3.32	16.79	4.79	-2.69	1.24E-04	3.33E-03
cg11604426	RHBDL3	17	TSS1500	N_Shore	28.65	4.22	31.34	3.80	-2.69	4.03E-03	4.59E-02
cg18768414	DKK3;DKK3;DKK3	11	TSS200;TSS1500;5'UTR	Island	14.61	3.50	17.30	3.40	-2.69	5.86E-05	1.78E-03
cg18119977		8	Intergenic	Open sea	33.30	6.88	35.99	6.09	-2.69	3.05E-03	3.79E-02
cg18582992	SHF	15	Body	Island	8.22	2.23	10.91	3.97	-2.69	6.51E-04	1.24E-02
cg18327056	KCNK3	2	TSS1500	N_Shore	30.27	3.62	32.96	3.84	-2.69	5.57E-05	1.71E-03
cg09435929	CA3	8	TSS200	Island	18.39	4.91	21.08	5.32	-2.69	8.93E-04	1.57E-02
cg00202760	CDT1	16	Body	Island	77.65	3.94	80.34	3.96	-2.69	4.09E-03	4.63E-02
cg21324884	MIR548A2	3	Body	Open sea	60.61	5.01	63.30	5.04	-2.69	3.37E-03	4.06E-02
cg07737810		18	Intergenic	N_Shelf	62.55	3.82	65.24	3.26	-2.69	1.49E-04	3.89E-03
cg18665334	ITM2C;ITM2C;ITM2C	2	Body;Body;Body	Open sea	72.68	3.63	75.37	4.19	-2.69	8.53E-05	2.45E-03
cg26465666	PDE1A;PDE1A	2	Body;Body	Open sea	84.22	4.48	86.90	3.32	-2.69	6.79E-04	1.28E-02
cg22325145	KCNJ4;KCNJ4	22	5'UTR;5'UTR	S_Shore	51.46	4.72	54.15	4.51	-2.69	2.28E-03	3.09E-02
cg13493652	C7orf70	7	5'UTR	N_Shelf	78.46	3.96	81.15	3.22	-2.69	3.10E-03	3.83E-02
cg10530889		10	Intergenic	Island	49.99	3.61	52.68	4.19	-2.69	3.15E-03	3.88E-02
cg23136139	RASGEF1A	10	Body	Island	40.05	3.51	42.73	3.38	-2.69	1.59E-04	4.11E-03
cg06539434	ASCC3	6	Body	Open sea	85.30	4.50	87.99	5.06	-2.69	3.06E-03	3.80E-02
cg12926596	BLOC1S1	12	Body	S_Shore	75.16	5.12	77.85	3.70	-2.69	1.41E-03	2.20E-02
cg11243029	DDAH1;DDAH1	1	Body;5'UTR	N_Shore	27.33	3.31	30.01	3.21	-2.69	1.19E-03	1.95E-02
cg24298684	MC3R;MC3R	20	5'UTR;1stExon	N_Shore	29.45	3.71	32.14	4.50	-2.69	2.49E-03	3.29E-02
cg00431050	ELOVL3	10	TSS1500	N_Shore	63.15	3.85	65.83	3.67	-2.69	2.76E-03	3.53E-02
cg14610335	COL27A1	9	Body	Open sea	37.99	2.55	40.67	3.61	-2.69	2.85E-03	3.61E-02
cg02379107	KIAA1755	20	TSS1500	Island	31.34	4.44	34.02	6.21	-2.69	3.48E-03	4.15E-02
cg19763428	PDE1C	7	Body	N_Shore	12.23	3.53	14.92	3.95	-2.69	2.69E-03	3.48E-02
cg26599002		8	Intergenic	Open sea	57.48	3.61	60.17	3.65	-2.69	2.07E-03	2.90E-02
cg14640621	HMX2	10	TSS1500	N_Shore	48.36	3.98	51.05	3.97	-2.69	2.61E-03	3.40E-02
cg10580513	OR2L8;OR2L13	1	TSS1500;5'UTR	Open sea	49.96	4.17	52.64	4.82	-2.69	4.01E-03	4.57E-02
cg13824877		6	Intergenic	N_Shore	70.66	4.18	73.35	4.01	-2.69	1.24E-03	2.01E-02
cg00723041		22	Intergenic	N_Shore	69.89	4.04	72.58	4.74	-2.69	3.79E-04	8.17E-03
cg25175900		12	Intergenic	Open sea	59.96	3.43	62.65	4.20	-2.69	1.90E-03	2.72E-02
cg25657752	SAMD14	17	Body	Open sea	69.38	3.69	72.07	3.52	-2.69	1.98E-03	2.80E-02
cg00557402	FAM55D;FAM55D	11	TSS200;TSS200	Open sea	56.57	6.15	59.25	6.88	-2.69	1.13E-03	1.87E-02
cg03180134	MAML3	4	Body	Open sea	16.91	3.05	19.60	2.83	-2.69	1.77E-05	6.25E-04
cg23253460		1	Intergenic	Open sea	83.54	4.46	86.22	4.76	-2.69	2.68E-03	3.46E-02
cg05572930	RNF217	6	3'UTR	Open sea	87.77	4.09	90.46	2.59	-2.69	4.66E-04	9.59E-03

cg18474153	EEF2	19	Body	N_Shore	19.10	2.66	21.79	3.75	-2.68	6.24E-04	1.20E-02
cg05977376	BRE;BRE;BRE;BRE;BRE	2	3'UTR;3'UTR;3'UTR;3'UTR;3'UTR	Open sea	48.45	3.99	51.13	3.80	-2.68	6.03E-04	1.17E-02
cg12416929	FOXO3;FOXO3;FOXO3	6	5'UTR;1stExon;5'UTR	Island	20.36	3.61	23.04	3.50	-2.68	6.43E-04	1.23E-02
cg14812313	TAP2;TAP2	6	Body;Body	Open sea	86.15	3.99	88.83	4.06	-2.68	5.88E-04	1.15E-02
cg06944559	SEMA4C	2	Body	S_Shore	63.25	3.91	65.94	3.99	-2.68	1.15E-03	1.90E-02
cg15729062	PPP1R10	6	5'UTR	Open sea	85.58	5.10	88.27	4.42	-2.68	1.85E-03	2.68E-02
cg22781074	HAGHL;HAGHL;HAGHL;CCDC78	16	5'UTR;5'UTR;1stExon;TSS1500	Island	19.82	2.85	22.51	3.70	-2.68	2.20E-04	5.33E-03
cg03519441	DSCAM	21	Body	Island	75.23	4.53	77.92	3.97	-2.68	1.37E-03	2.16E-02
cg20750642	FARP1	13	3'UTR	Open sea	81.74	3.92	84.43	3.54	-2.68	7.14E-04	1.33E-02
cg02631189		1	Intergenic	Open sea	57.79	3.51	60.47	3.81	-2.68	1.86E-04	4.67E-03
cg17599958	AP2A2	11	Body	Island	74.94	4.36	77.62	4.74	-2.68	3.68E-03	4.31E-02
cg03396594	DNAJC13	3	5'UTR	S_Shelf	80.26	3.90	82.94	4.08	-2.68	5.52E-04	1.09E-02
cg08864042	DCTN1;DCTN1;DCTN1;DCTN1	2	5'UTR;1stExon;1stExon;5'UTR	Open sea	46.38	3.48	49.06	3.65	-2.68	8.91E-04	1.57E-02
cg20921751	FOXN2	2	3'UTR	Open sea	86.11	3.93	88.80	3.45	-2.68	5.50E-04	1.09E-02
cg20698781		2	Intergenic	Open sea	65.59	5.15	68.27	5.17	-2.68	4.42E-03	4.89E-02
cg01692279		7	Intergenic	N_Shore	37.94	4.53	40.63	5.13	-2.68	2.12E-03	2.95E-02
cg13526642	AKAP2;PALM2-AKAP2;PALM2-AKAP2	9	Body;Body;Body	Open sea	72.31	4.17	74.99	3.15	-2.68	3.52E-03	4.18E-02
cg11638760		5	Intergenic	Open sea	83.70	3.99	86.38	3.84	-2.68	9.20E-04	1.61E-02
cg13676665	RAB23;RAB23	6	5'UTR;5'UTR	N_Shelf	82.09	1.94	84.77	1.70	-2.68	6.18E-09	3.33E-07
cg13173369		1	Intergenic	Open sea	66.96	3.59	69.64	2.50	-2.68	9.07E-04	1.59E-02
cg00559234	CLSTN1;CLSTN1	1	TSS1500;TSS1500	S_Shore	35.53	5.86	38.21	6.62	-2.68	4.25E-03	4.76E-02
cg02068351	STX7	6	5'UTR	N_Shore	84.34	3.11	87.02	2.59	-2.68	1.56E-05	5.59E-04
cg24161793	ITPRIPL1;ITPRIPL1;ITPRIPL1;ITPRIPL1	2	TSS1500;TSS1500;TSS1500;TSS1500	N_Shore	43.18	5.44	45.86	5.79	-2.68	3.71E-03	4.33E-02
cg15371711	MYL9;MYL9	20	TSS200;TSS200	S_Shore	25.51	6.15	28.19	5.96	-2.68	3.33E-03	4.02E-02
cg23207280	MBNL1;MBNL1;MBNL1;MBNL1;MBNL	3	Body;Body;Body;Body;Body;Body;Body	Open sea	83.73	3.82	86.41	2.53	-2.68	2.76E-04	6.38E-03
cg16420354	NOS3;NOS3;NOS3;NOS3	7	5'UTR;TSS200;TSS200;TSS200	Open sea	74.09	4.03	76.77	3.41	-2.68	2.73E-03	3.51E-02
cg03542686	CAPZB	1	Body	Open sea	80.71	3.33	83.39	3.13	-2.68	1.91E-04	4.76E-03
cg06700871	SUCLG1	2	1stExon	Island	14.24	2.49	16.92	3.71	-2.68	2.46E-04	5.83E-03
cg20943251	COL1A2	7	3'UTR	Open sea	83.71	4.66	86.39	4.75	-2.68	1.02E-03	1.73E-02
cg13412283		7	Intergenic	Open sea	75.25	4.72	77.93	4.15	-2.68	1.33E-03	2.12E-02
cg23626798	L3MBTL;L3MBTL	20	TSS1500;TSS1500	N_Shore	47.34	2.14	50.02	3.00	-2.68	2.03E-05	7.07E-04
cg03203274	C6orf154	6	TSS200	Island	48.53	3.86	51.21	3.36	-2.68	1.50E-03	2.30E-02
cg18443392	LRP1	12	Body	Open sea	70.24	3.94	72.92	3.27	-2.68	3.58E-03	4.23E-02
cg12612420		7	Intergenic	N_Shore	15.18	2.75	17.86	3.76	-2.68	1.25E-04	3.36E-03
cg06736785	PLXNA4;PLXNA4;PLXNA4	7	5'UTR;5'UTR;5'UTR	N_Shore	30.03	3.95	32.71	3.29	-2.68	3.45E-03	4.12E-02
cg03044376	C20orf95	20	Body	N_Shore	44.08	3.26	46.76	4.32	-2.68	2.74E-03	3.52E-02
cg00979704	TNXB	6	5'UTR	Open sea	88.87	3.16	91.55	1.77	-2.68	8.57E-06	3.27E-04
cg02831604	ISL2	15	TSS1500	N_Shore	23.42	3.59	26.10	3.86	-2.68	2.44E-04	5.78E-03
cg06361405	ARL3	10	3'UTR	Open sea	47.85	4.73	50.53	6.20	-2.68	3.27E-03	3.97E-02
cg14708940		10	Intergenic	Open sea	73.26	5.49	75.94	5.78	-2.68	5.05E-04	1.02E-02
cg26217402	C14orf43	14	5'UTR	Open sea	52.49	4.16	55.17	4.35	-2.68	1.86E-03	2.69E-02
cg18738804		8	Intergenic	S_Shelf	77.80	4.76	80.48	4.50	-2.68	2.03E-03	2.86E-02
cg16560389		10	Intergenic	Island	80.05	5.06	82.73	4.37	-2.68	2.47E-03	3.28E-02
cg02341729		12	Intergenic	Open sea	76.12	4.49	78.80	4.03	-2.68	4.99E-04	1.01E-02
cg08095852	WDR17;WDR17;WDR17;WDR17	4	1stExon;5'UTR;1stExon;5'UTR	Island	14.71	4.31	17.38	4.52	-2.68	2.60E-04	6.08E-03
cg10502590		6	Intergenic	Open sea	86.26	2.64	88.94	2.14	-2.68	8.04E-07	3.66E-05
cg21231776	MCFD2;MCFD2;MCFD2;MCFD2;MCFD	2	5'UTR;5'UTR;5'UTR;5'UTR;5'UTR;Body;5'U	Open sea	85.26	3.94	87.93	3.59	-2.68	2.61E-03	3.40E-02
cg13916874		6	Intergenic	S_Shelf	67.18	4.07	69.85	3.27	-2.68	1.37E-03	2.16E-02
cg18416576	HOXA10;HOXA10	7	TSS200;Body	Island	13.57	2.25	16.25	2.66	-2.68	2.41E-07	1.16E-05
cg12952446	CYTH3	7	3'UTR	Island	73.57	3.95	76.25	3.11	-2.68	2.29E-03	3.10E-02
cg00809453		13	Intergenic	Open sea	67.94	3.38	70.62	3.71	-2.68	2.02E-03	2.85E-02
cg14327063		9	Intergenic	Open sea	85.38	3.38	88.06	2.81	-2.68	2.83E-04	6.50E-03
cg08482829	CHST3	10	5'UTR	Island	19.34	4.07	22.02	3.88	-2.68	4.59E-04	9.48E-03

cg02745847	IGF2BP1;IGF2BP1	17	Body;Body	Island	21.82	3.99	24.49	3.84	-2.68	2.16E-03	2.99E-02
cg04738673		3	Intergenic	Open sea	67.71	4.64	70.39	5.23	-2.68	2.02E-03	2.85E-02
cg08442334	RBM4	11	Body	Island	83.19	3.71	85.87	2.97	-2.68	3.05E-04	6.89E-03
cg26166935	PHLPP1	18	Body	Open sea	83.41	3.25	86.09	3.10	-2.68	1.54E-03	2.34E-02
cg23462632	TNXB	6	Body	Open sea	67.71	3.42	70.39	3.07	-2.68	3.14E-04	7.06E-03
cg24530510	RPS3;SNORD15B	11	Body;TSS200	Open sea	57.84	3.80	60.52	4.08	-2.68	1.38E-03	2.17E-02
cg08362880	TNXB	6	Body	N_Shore	42.92	3.20	45.60	3.67	-2.68	4.96E-04	1.01E-02
cg18467168	IQSEC1	3	TSS200	Island	36.30	4.79	38.97	4.59	-2.68	2.16E-03	2.99E-02
cg15330298	L3MBTL;L3MBTL;L3MBTL;L3MBTL	20	5'UTR;1stExon;1stExon;5'UTR	N_Shore	53.57	4.44	56.25	3.22	-2.68	3.67E-03	4.30E-02
cg12851570	SSH2	17	Body	N_Shore	31.92	3.52	34.60	5.40	-2.68	4.13E-04	8.74E-03
cg06173470	RNF212;RNF212	4	Body;Body	N_Shore	64.38	4.25	67.06	4.52	-2.68	3.89E-03	4.48E-02
cg08423575	ZBTB40;ZBTB40	1	Body;Body	Open sea	81.67	3.71	84.34	3.32	-2.68	2.09E-04	5.12E-03
cg11351809	HS3ST3B1	17	Body	S_Shore	58.56	3.93	61.24	4.22	-2.68	2.13E-03	2.96E-02
cg10502121		7	Intergenic	Island	27.10	3.23	29.77	3.80	-2.68	1.20E-03	1.97E-02
cg13740757		7	Intergenic	Open sea	85.18	3.71	87.85	2.79	-2.68	2.99E-04	6.79E-03
cg03730474		19	Intergenic	S_Shore	25.50	5.04	28.18	5.26	-2.68	3.37E-03	4.06E-02
cg04663842		7	Intergenic	N_Shelf	38.84	4.21	41.52	4.40	-2.67	1.58E-03	2.39E-02
cg08712082	GARS	7	3'UTR	Open sea	83.13	2.47	85.81	1.96	-2.67	2.85E-06	1.19E-04
cg01593673	HOXB3	17	5'UTR	Open sea	20.66	3.22	23.33	3.67	-2.67	4.31E-03	4.80E-02
cg03766373	KRT83	12	1stExon	Island	60.49	2.98	63.17	3.39	-2.67	4.22E-04	8.88E-03
cg27186280		6	Intergenic	Open sea	80.24	2.86	82.92	2.50	-2.67	2.37E-05	8.13E-04
cg07967308	ACP5;ACP5;ACP5;ACP5	19	5'UTR;5'UTR;5'UTR;5'UTR	N_Shore	80.57	3.88	83.24	3.71	-2.67	1.40E-03	2.19E-02
cg06423193	DERA	12	Body	Open sea	89.38	3.38	92.06	2.81	-2.67	8.22E-05	2.37E-03
cg06583577	SEMA6A	5	5'UTR	Open sea	70.09	4.76	72.76	4.71	-2.67	1.61E-03	2.42E-02
cg12632313	RND3	2	Body	Open sea	83.47	4.54	86.15	3.20	-2.67	3.99E-03	4.56E-02
cg00217306	TFDP2	3	Body	Open sea	22.06	3.43	24.73	3.90	-2.67	5.88E-04	1.15E-02
cg20603821	PTPRN2;PTPRN2;PTPRN2	7	Body;Body;Body	N_Shore	63.89	4.08	66.57	4.36	-2.67	5.98E-04	1.16E-02
cg24902920	TFAP2A;TFAP2A	6	Body;TSS1500	N_Shore	17.33	2.55	20.00	3.42	-2.67	8.62E-05	2.47E-03
cg10651637	SORD	15	Body	S_Shore	26.42	2.80	29.09	2.47	-2.67	1.09E-04	3.00E-03
cg06351965	PLK1	16	1stExon	Island	8.48	2.59	11.16	3.18	-2.67	4.92E-06	1.97E-04
cg03407622	UMPS	3	3'UTR	Open sea	70.90	3.58	73.58	2.78	-2.67	8.58E-04	1.53E-02
cg06722069		3	Intergenic	Island	41.89	3.35	44.57	3.75	-2.67	1.40E-03	2.20E-02
cg20700813	XYLT2	17	3'UTR	Open sea	80.11	2.49	82.78	2.33	-2.67	2.86E-07	1.37E-05
cg16443954	GALNT9;LOC100130238	12	Body;TSS200	N_Shelf	22.53	4.95	25.20	5.26	-2.67	3.53E-03	4.19E-02
cg27120543	TMEM195	7	Body	Open sea	83.91	3.60	86.58	2.91	-2.67	5.60E-04	1.10E-02
cg25019989		4	Intergenic	S_Shelf	27.69	3.93	30.36	3.97	-2.67	1.67E-03	2.48E-02
cg13564654		11	Intergenic	Open sea	74.40	3.69	77.08	4.14	-2.67	2.06E-03	2.89E-02
cg26783079	PLCH2	1	Body	Island	70.09	3.66	72.76	4.81	-2.67	2.73E-03	3.50E-02
cg10122434	IRF6	1	Body	Open sea	68.07	4.02	70.74	4.00	-2.67	2.66E-04	6.19E-03
cg07395930	CLSTN1;CLSTN1	1	Body;Body	Island	87.67	3.35	90.34	1.75	-2.67	1.45E-04	3.81E-03
cg08458197		8	Intergenic	Open sea	41.67	3.81	44.34	3.66	-2.67	3.24E-03	3.95E-02
cg05919975		12	Intergenic	S_Shelf	71.40	4.02	74.08	3.44	-2.67	3.60E-04	7.86E-03
cg02057391	TRPC4;TRPC4;TRPC4;TRPC4;TRPC4;TR	13	1stExon;1stExon;5'UTR;1stExon;5'UTR;5'U	Island	14.03	2.41	16.70	3.43	-2.67	2.84E-04	6.51E-03
cg14080888	FAM102A;FAM102A;FAM102A	9	5'UTR;Body;1stExon	Open sea	38.09	2.51	40.76	3.50	-2.67	6.94E-04	1.30E-02
cg03111498	VSX1;VSX1	20	TSS200;TSS200	Island	11.95	2.45	14.62	2.74	-2.67	7.92E-05	2.30E-03
cg20924735	ALS2	2	3'UTR	Open sea	81.10	3.99	83.77	3.32	-2.67	2.89E-03	3.65E-02
cg02297936	NFIX	19	3'UTR	Island	32.32	2.96	34.99	4.19	-2.67	1.98E-03	2.81E-02
cg14001893	SARDH;SARDH	9	Body;Body	N_Shelf	89.77	3.34	92.44	2.24	-2.67	4.38E-04	9.15E-03
cg15828044		13	Intergenic	Open sea	85.84	3.06	88.51	2.21	-2.67	2.00E-05	6.98E-04
cg00639656	SNORD42A;RPL23A;SNORD4B	17	TSS200;Body;TSS1500	N_Shelf	67.62	3.71	70.29	3.42	-2.67	8.01E-05	2.32E-03
cg08845025	TNXB	6	Body	Open sea	55.68	5.86	58.35	4.76	-2.67	2.61E-03	3.40E-02
cg01202155	EMX2;EMX2;EMX2OS;EMX2;EMX2	10	1stExon;5'UTR;Body;1stExon;5'UTR	Island	22.36	3.41	25.03	4.39	-2.67	2.99E-03	3.73E-02
cg24394856		6	Intergenic	Open sea	18.04	3.09	20.71	3.52	-2.67	9.99E-04	1.71E-02

cg05886115		15	Intergenic	Open sea	50.66	3.50	53.33	3.76	-2.67	1.52E-03	2.33E-02
cg07871590	LRRC4;SND1	7	TSS200;Body	Island	49.01	4.51	51.68	4.72	-2.67	6.26E-04	1.20E-02
cg07725206		1	Intergenic	Open sea	71.76	4.06	74.43	3.83	-2.67	1.59E-03	2.40E-02
cg09999510	ZFR2	19	Body	S_Shore	52.06	5.81	54.73	5.49	-2.67	1.65E-03	2.46E-02
cg05346476	SFRS2IP	12	Body	Open sea	87.77	3.89	90.44	2.45	-2.67	1.56E-04	4.03E-03
cg11823511	BARHL2	1	TSS1500	Island	14.53	3.96	17.20	5.01	-2.67	3.50E-03	4.16E-02
cg26573321	PIK3CD	1	TSS200	N_Shore	12.52	3.47	15.19	4.42	-2.67	2.68E-05	9.05E-04
cg12244205	ZNF311;ZNF311	6	5'UTR;1stExon	Open sea	54.73	5.02	57.39	4.09	-2.67	2.27E-03	3.08E-02
cg15261730		7	Intergenic	Open sea	56.67	4.35	59.34	3.86	-2.67	1.37E-03	2.15E-02
cg02736280	WDR81;WDR81;WDR81;WDR81	17	Body;Body;Body;Body	N_Shelf	71.14	3.59	73.81	3.44	-2.67	3.23E-03	3.94E-02
cg01754967	WDR72	15	5'UTR	N_Shelf	74.21	4.74	76.88	3.88	-2.67	2.04E-03	2.86E-02
cg14930320		6	Intergenic	S_Shelf	48.09	5.06	50.75	5.31	-2.67	7.19E-04	1.34E-02
cg01137401	ZIC4;ZIC4;ZIC4;ZIC4;ZIC4	3	Body;Body;Body;Body;Body	N_Shore	27.71	5.52	30.38	5.44	-2.67	3.55E-03	4.21E-02
cg05412784	CPA6;CPA6	8	Body;Body	Open sea	84.17	4.84	86.84	3.57	-2.67	4.04E-03	4.59E-02
cg20476058	LIMS2	2	5'UTR	Open sea	77.34	4.14	80.01	2.94	-2.67	2.79E-03	3.57E-02
cg09656690		5	Intergenic	S_Shelf	25.19	4.01	27.85	5.29	-2.67	1.78E-03	2.60E-02
cg14173815	RPL23A;SNORD42A;SNORD4B;SNORD	17	Body;TSS1500;TSS1500;TSS200	N_Shelf	66.07	4.38	68.74	4.35	-2.67	2.87E-04	6.57E-03
cg26037848	TUB	11	TSS1500	Open sea	81.34	3.42	84.00	2.98	-2.67	5.71E-04	1.12E-02
cg20022869	NPEPL1	20	TSS1500	N_Shore	71.63	4.15	74.30	3.56	-2.67	3.22E-03	3.93E-02
cg11879354	SYTL3	6	5'UTR	Open sea	40.67	3.45	43.33	3.16	-2.67	2.33E-03	3.15E-02
cg20026939		18	Intergenic	N_Shore	15.87	3.34	18.54	3.37	-2.67	2.88E-04	6.59E-03
cg01488306	F13B	1	TSS1500	Open sea	65.65	3.97	68.31	3.23	-2.67	1.04E-03	1.76E-02
cg00544337	DYDC2;DYDC1	10	TSS1500;5'UTR	Island	54.38	4.68	57.05	5.54	-2.67	4.44E-03	4.90E-02
cg04147940		8	Intergenic	Open sea	62.33	5.28	65.00	5.06	-2.67	3.46E-04	7.60E-03
cg26816907	LHX9;LHX9	1	Body;Body	Island	50.98	3.36	53.65	4.14	-2.67	8.23E-04	1.48E-02
cg23694028		8	Intergenic	Open sea	82.81	5.03	85.47	5.34	-2.67	3.08E-03	3.82E-02
cg17310258		11	Intergenic	Island	47.12	4.18	49.78	4.76	-2.67	4.18E-03	4.70E-02
cg00954545		8	Intergenic	Open sea	79.75	5.46	82.41	4.30	-2.67	2.65E-03	3.44E-02
cg00119314	RSAD1	17	TSS1500	N_Shore	72.42	4.16	75.08	3.92	-2.67	1.31E-03	2.09E-02
cg08872579	TTC22;TTC22	1	Body;Body	N_Shore	75.07	3.44	77.74	3.61	-2.67	1.55E-03	2.35E-02
cg00616000	KDELR2;KDELR2	7	Body;Body	N_Shore	20.94	4.09	23.61	3.65	-2.67	3.89E-03	4.48E-02
cg13868996		9	Intergenic	Open sea	75.01	2.80	77.67	2.67	-2.67	1.35E-05	4.93E-04
cg00262159	ABLIM2;ABLIM2;ABLIM2;ABLIM2;ABL	4	Body;Body;Body;Body;Body;Body	Open sea	78.00	5.00	80.66	4.66	-2.67	2.02E-03	2.85E-02
cg02482346	UBTF;UBTF;UBTF	17	3'UTR;3'UTR;3'UTR	N_Shelf	49.59	3.63	52.25	3.98	-2.67	2.62E-03	3.41E-02
cg04222582	DUOX2;DUOXA2	15	Body;TSS1500	Island	17.57	3.73	20.24	4.13	-2.67	3.90E-03	4.48E-02
cg16234890		13	Intergenic	N_Shore	85.44	4.50	88.11	4.52	-2.66	1.27E-03	2.04E-02
cg24691904	VPS37B	12	TSS200	Island	12.33	3.33	15.00	3.43	-2.66	1.92E-04	4.78E-03
cg24279744	ATP2B2;ATP2B2	3	Body;Body	Open sea	60.26	4.07	62.92	4.11	-2.66	1.30E-03	2.08E-02
cg06770076		1	Intergenic	Open sea	83.70	4.18	86.37	3.77	-2.66	1.43E-03	2.22E-02
cg26685033	C10orf79	10	Body	Open sea	67.44	3.53	70.10	4.23	-2.66	1.42E-03	2.21E-02
cg08179037	LOC285830;LOC285830	6	Body;Body	Open sea	23.78	3.86	26.44	4.00	-2.66	1.08E-03	1.81E-02
cg17127511	PODN	1	Body	S_Shore	75.63	3.43	78.29	3.36	-2.66	4.70E-05	1.47E-03
cg01259126	GAS2L1;GAS2L1;GAS2L1	22	Body;Body;Body	N_Shore	68.23	3.58	70.89	3.71	-2.66	7.56E-04	1.39E-02
cg19528963	LPP;LPP;LPP	3	TSS1500;5'UTR;5'UTR	Open sea	90.97	2.55	93.63	1.57	-2.66	1.35E-06	5.95E-05
cg17069437	TSC22D4	7	3'UTR	Open sea	74.69	2.95	77.36	2.78	-2.66	1.31E-04	3.51E-03
cg13434396	ADAMTSL5	19	Body	N_Shore	37.02	3.90	39.68	4.74	-2.66	1.30E-03	2.08E-02
cg20969151	BMP6	6	Body	Open sea	70.69	4.81	73.35	4.31	-2.66	3.93E-03	4.51E-02
cg06250127	CRISPLD2	16	3'UTR	Open sea	82.76	3.58	85.42	3.98	-2.66	1.47E-03	2.27E-02
cg01566127	MACROD1	11	Body	Open sea	19.21	3.96	21.88	4.38	-2.66	3.18E-05	1.05E-03
cg21933091	TMEM232	5	Body	Open sea	79.75	4.67	82.41	5.47	-2.66	1.18E-03	1.93E-02
cg01376402		1	Intergenic	Island	26.29	3.75	28.96	3.79	-2.66	2.44E-03	3.25E-02
cg26982323	YPEL3;YPEL3	16	Body;Body	S_Shore	71.62	3.41	74.28	3.98	-2.66	1.70E-03	2.51E-02
cg09757353	GRK5	10	Body	Open sea	83.93	3.09	86.59	2.78	-2.66	1.57E-04	4.08E-03

cg05578840	OTP	5	Body	N_Shore	18.78	3.85	21.44	4.06	-2.66	1.10E-03	1.84E-02
cg22974228	ALX4	11	TSS1500	Island	22.91	2.49	25.57	2.56	-2.66	1.84E-05	6.50E-04
cg10805039	NXN	17	Body	Open sea	63.84	5.40	66.50	5.62	-2.66	2.10E-03	2.93E-02
cg03854595		16	Intergenic	N_Shore	45.28	3.25	47.94	3.77	-2.66	1.70E-03	2.51E-02
cg10132208	ZSCAN1	19	5'UTR	Island	20.03	4.14	22.69	4.34	-2.66	1.55E-03	2.35E-02
cg04188238	MTNR1A	4	TSS1500	S_Shore	79.71	4.51	82.37	3.90	-2.66	8.73E-04	1.55E-02
cg26013733	ABR;ABR;ABR	17	Body;Body;Body	S_Shelf	85.03	4.09	87.70	4.31	-2.66	2.56E-03	3.36E-02
cg14409166	NGEF;NGEF	2	Body;Body	Open sea	81.52	4.05	84.19	3.44	-2.66	3.75E-03	4.37E-02
cg03365733	PRDM13	6	Body	Island	28.92	3.15	31.58	3.70	-2.66	4.49E-04	9.32E-03
cg14048487	BRCA1;BRCA1;BRCA1;BRCA1;BRCA1;E	17	Body;Body;Body;5'UTR;Body;Body	Open sea	69.23	4.27	71.89	4.14	-2.66	4.17E-03	4.69E-02
cg19265103	GALNT6	12	5'UTR	N_Shore	55.81	4.67	58.47	5.20	-2.66	2.21E-03	3.03E-02
cg02551604		5	Intergenic	N_Shore	22.51	3.51	25.17	4.04	-2.66	2.14E-03	2.97E-02
cg21515494	FLJ41856	19	TSS1500	Open sea	63.75	4.65	66.41	3.47	-2.66	3.29E-03	4.00E-02
cg06777815		8	Intergenic	Open sea	81.81	3.53	84.47	3.09	-2.66	9.72E-04	1.68E-02
cg01023504	PRKAR1B;PRKAR1B;PRKAR1B;PRKAR1	7	Body;Body;Body;Body;Body;Body	S_Shore	49.57	3.97	52.23	3.54	-2.66	3.35E-03	4.04E-02
cg17084361	CDS1	4	TSS1500	Island	12.76	3.22	15.42	3.53	-2.66	3.85E-05	1.24E-03
cg23325570	LOC285830;LOC285830	6	Body;Body	Open sea	71.53	4.06	74.19	3.95	-2.66	4.52E-03	4.96E-02
cg25237016	SPTBN4	19	Body	Island	24.56	3.48	27.22	3.17	-2.66	5.73E-04	1.12E-02
cg08934785	HOXA7	7	TSS1500	N_Shore	21.61	3.05	24.27	3.43	-2.66	8.89E-05	2.54E-03
cg14780416	MIR196B	7	TSS200	Island	12.92	2.57	15.58	3.04	-2.66	3.97E-06	1.62E-04
cg20366832	LLGL2;LLGL2;LLGL2	17	TSS1500;TSS1500;TSS1500	N_Shore	51.10	5.66	53.76	4.41	-2.66	3.79E-03	4.40E-02
cg26979339	RIC8B	12	TSS1500	Island	19.18	3.10	21.83	4.10	-2.66	1.80E-03	2.62E-02
cg21811021		4	Intergenic	Island	88.97	4.56	91.63	2.29	-2.66	1.22E-03	1.99E-02
cg22038124	TNFAIP8L3	15	Body	Open sea	55.99	3.70	58.65	3.75	-2.66	8.78E-04	1.55E-02
cg25801113	SHF	15	Body	N_Shelf	78.39	3.78	81.05	4.36	-2.66	2.47E-03	3.28E-02
cg24638828		7	Intergenic	S_Shelf	39.61	7.62	42.27	7.97	-2.66	2.28E-03	3.09E-02
cg02531437	PRLHR	10	TSS200	Island	13.90	2.94	16.56	3.22	-2.66	7.30E-04	1.35E-02
cg07360480		8	Intergenic	Open sea	81.13	4.49	83.79	4.40	-2.66	2.71E-03	3.49E-02
cg05452899	INS-IGF2;IGF2AS;IGF2;IGF2;IGF2AS;IG	11	Body;TSS1500;Body;5'UTR;TSS1500;TSS15	Island	65.68	4.39	68.34	4.41	-2.66	3.94E-03	4.52E-02
cg24008634	FRMD4A	10	Body	N_Shelf	77.06	3.88	79.71	3.44	-2.66	1.06E-03	1.78E-02
cg02002586	C6orf27	6	Body	Open sea	84.64	3.41	87.30	2.22	-2.66	4.12E-04	8.73E-03
cg14416523	TBR1	2	Body	N_Shore	17.52	2.88	20.18	3.26	-2.66	7.00E-05	2.07E-03
cg15648832		5	Intergenic	Open sea	75.79	4.87	78.45	4.58	-2.66	3.82E-03	4.42E-02
cg26053832		16	Intergenic	Island	31.02	3.98	33.68	3.37	-2.66	5.38E-04	1.07E-02
cg15900054	COL11A2;COL11A2;COL11A2	6	Body;Body;Body	Open sea	72.76	4.04	75.42	3.19	-2.66	2.32E-03	3.14E-02
cg02533423		7	Intergenic	Island	21.33	4.05	23.99	4.72	-2.66	5.94E-04	1.16E-02
cg00860372	ZSWIM4	19	Body	N_Shelf	70.03	4.07	72.68	4.15	-2.66	1.04E-03	1.76E-02
cg00250483	RGS5	1	TSS200	Open sea	66.32	4.48	68.98	3.93	-2.66	1.97E-03	2.80E-02
cg08562099	MSI2;MSI2	17	Body;Body	Open sea	75.57	3.76	78.23	3.55	-2.66	1.47E-03	2.27E-02
cg03445220	C1orf101;C1orf101	1	Body;Body	Open sea	69.32	3.35	71.98	3.95	-2.66	7.96E-04	1.44E-02
cg26534993	TSSC1	2	Body	Island	52.29	4.13	54.95	3.27	-2.66	1.28E-03	2.06E-02
cg08355702		2	Intergenic	Open sea	64.71	4.55	67.37	3.72	-2.66	3.43E-03	4.11E-02
cg23238834	CCHCR1;CCHCR1;TCF19;TCF19;CCHCR	6	TSS1500;TSS1500;5'UTR;5'UTR;TSS1500	S_Shore	35.09	5.34	37.74	4.96	-2.66	2.20E-03	3.02E-02
cg08182597		16	Intergenic	Open sea	75.37	4.77	78.03	5.57	-2.66	7.08E-04	1.32E-02
cg25992874	CASP2;CASP2	7	3'UTR;Body	Open sea	76.69	3.95	79.35	3.25	-2.66	1.12E-03	1.87E-02
cg00405078		6	Intergenic	Open sea	46.50	2.76	49.16	3.65	-2.66	7.68E-05	2.24E-03
cg27093143	APLP1;APLP1	19	Body;Body	Island	17.63	3.29	20.28	4.13	-2.66	1.67E-04	4.27E-03
cg11720170	KLHL20	1	TSS1500	N_Shore	88.40	3.43	91.05	2.28	-2.66	1.53E-05	5.51E-04
cg18431123	SPINT3	20	TSS1500	Open sea	59.93	4.00	62.58	4.05	-2.66	3.87E-03	4.47E-02
cg03440556	SCD	10	Body	S_Shore	19.12	3.49	21.78	3.58	-2.66	1.60E-03	2.41E-02
cg02319354		5	Intergenic	S_Shore	13.76	2.29	16.41	2.75	-2.66	7.15E-06	2.77E-04
cg14743708	LEMD2;LEMD2	6	5'UTR;Body	N_Shelf	81.58	3.30	84.23	3.02	-2.66	1.22E-03	1.99E-02
cg00083833	NKD1	16	Body	Island	43.60	4.81	46.26	5.27	-2.66	3.15E-03	3.87E-02

cg00860808		2	Intergenic	Island	16.69	2.31	19.34	3.35	-2.66	5.37E-05	1.65E-03
cg24424525	KIAA1217;KIAA1217;PRINS;KIAA1217	10	Body;Body;TSS1500;Body	Open sea	81.61	4.56	84.26	3.76	-2.66	2.90E-03	3.65E-02
cg20417500	RGNEF	5	Body	Open sea	54.13	5.29	56.79	6.58	-2.66	4.51E-03	4.96E-02
cg25528260		7	Intergenic	Open sea	79.46	3.88	82.11	2.54	-2.66	2.03E-03	2.86E-02
cg01206259	GRK5	10	Body	Open sea	75.45	4.39	78.10	3.40	-2.66	2.14E-03	2.97E-02
cg13741070		7	Intergenic	N_Shelf	60.68	3.68	63.33	2.95	-2.66	9.19E-04	1.61E-02
cg17526311	HIPK2;HIPK2	7	Body;Body	Open sea	87.02	4.05	89.68	2.18	-2.66	6.62E-04	1.26E-02
cg17734798	PLXNA4;PLXNA4;PLXNA4	7	5'UTR;5'UTR;5'UTR	Open sea	82.93	4.18	85.58	2.41	-2.66	3.80E-03	4.41E-02
cg18507018	TMEM55A	8	Body	Open sea	79.86	4.72	82.52	3.96	-2.66	1.25E-03	2.02E-02
cg01557951		1	Intergenic	N_Shore	35.46	3.22	38.12	4.36	-2.66	1.80E-03	2.63E-02
cg12097330	BAIAP2;BAIAP2;BAIAP2;BAIAP2	17	Body;Body;Body;Body	N_Shore	88.21	3.17	90.87	2.47	-2.66	2.06E-04	5.06E-03
cg01886524	LOC90246	3	TSS1500	Open sea	64.54	4.43	67.19	4.03	-2.66	1.91E-03	2.74E-02
cg21762655		3	Intergenic	Open sea	74.65	4.26	77.31	3.90	-2.66	2.67E-04	6.22E-03
cg13370852	TMEM201;TMEM201	1	Body;Body	S_Shelf	77.91	3.31	80.57	3.02	-2.66	3.36E-04	7.44E-03
cg00965469	LY6G5C	6	TSS1500	N_Shore	49.26	5.17	51.92	4.62	-2.65	2.71E-03	3.49E-02
cg06783197	PACS2;PACS2	14	Body;Body	S_Shore	58.71	5.24	61.37	5.08	-2.65	1.14E-03	1.89E-02
cg05361458	CBFA2T3;CBFA2T3	16	Body;Body	Island	88.26	3.45	90.91	2.09	-2.65	2.86E-04	6.55E-03
cg21269843	OTP	5	TSS200	Island	22.39	2.93	25.04	2.83	-2.65	2.39E-05	8.20E-04
cg21635588	IPCEF1;IPCEF1;IPCEF1	6	5'UTR;5'UTR;5'UTR	Open sea	78.32	4.13	80.98	4.43	-2.65	4.54E-03	4.97E-02
cg11697427		10	Intergenic	Island	15.58	3.38	18.23	4.32	-2.65	2.10E-03	2.93E-02
cg13718387		3	Intergenic	N_Shelf	63.19	3.65	65.84	3.16	-2.65	1.47E-03	2.27E-02
cg26620021	MIR641;AKT2	19	TSS1500;5'UTR	N_Shore	61.08	4.34	63.73	3.92	-2.65	1.68E-03	2.49E-02
cg21415530	KCNK9	8	TSS1500	Island	26.28	3.69	28.94	4.17	-2.65	1.17E-04	3.18E-03
cg00973737	ADH6;ADH6	4	Body;Body	Open sea	87.61	4.19	90.26	2.96	-2.65	1.04E-03	1.76E-02
cg12057673		6	Intergenic	Open sea	85.51	5.63	88.16	3.82	-2.65	1.55E-03	2.36E-02
cg25521254	RNMTL1	17	Body	Open sea	70.68	3.74	73.33	4.23	-2.65	1.00E-03	1.72E-02
cg00987918	TRIM40	6	3'UTR	Open sea	58.73	4.26	61.38	4.75	-2.65	2.38E-03	3.19E-02
cg10853830	PITPNM1;PITPNM1	11	Body;Body	S_Shelf	83.06	3.70	85.71	2.85	-2.65	1.74E-03	2.57E-02
cg12504828	ABCA9	17	3'UTR	Open sea	69.86	4.27	72.51	4.52	-2.65	2.22E-03	3.04E-02
cg00499822	CXCL12;CXCL12;CXCL12	10	TSS1500;TSS1500;TSS1500	Island	54.58	4.28	57.23	3.87	-2.65	1.60E-03	2.41E-02
cg18280830		14	Intergenic	S_Shore	23.30	3.16	25.95	3.61	-2.65	6.91E-04	1.30E-02
cg01031670	CYB5R3;CYB5R3;CYB5R3	22	3'UTR;3'UTR;3'UTR	S_Shelf	78.48	4.19	81.13	3.98	-2.65	2.92E-03	3.67E-02
cg04021672	C9orf25	9	Body	N_Shore	52.38	4.65	55.03	4.83	-2.65	2.12E-03	2.95E-02
cg20029201	BCL9L	11	TSS1500	S_Shore	25.64	3.78	28.30	3.52	-2.65	8.86E-04	1.56E-02
cg18985994	IQCE;IQCE	7	Body;Body	S_Shelf	52.12	4.82	54.77	4.68	-2.65	3.60E-03	4.25E-02
cg07523553	HCCA2;LOC402778	11	Body;TSS1500	Island	9.04	2.84	11.69	3.94	-2.65	1.60E-03	2.41E-02
cg14522047	CREB3L2	7	Body	Open sea	84.12	3.28	86.77	2.94	-2.65	2.49E-04	5.88E-03
cg22434594		17	Intergenic	N_Shore	48.47	4.13	51.12	3.14	-2.65	2.25E-03	3.07E-02
cg21105175	RANBP3;RANBP3;RANBP3	19	3'UTR;3'UTR;3'UTR	S_Shelf	77.68	3.99	80.33	3.94	-2.65	3.72E-03	4.34E-02
cg26627665		18	Intergenic	Island	51.35	3.98	54.01	2.94	-2.65	3.42E-03	4.10E-02
cg27660536		4	Intergenic	Open sea	56.39	3.79	59.04	3.66	-2.65	8.80E-04	1.56E-02
cg25747192	RASSF1;RASSF1	3	TSS200;TSS200	Island	51.51	4.36	54.16	4.75	-2.65	2.00E-03	2.83E-02
cg02354293		3	Intergenic	Open sea	67.51	3.87	70.16	3.77	-2.65	1.24E-03	2.01E-02
cg15301794	PAX6;PAX6;PAX6	11	Body;Body;Body	S_Shore	40.36	3.86	43.01	4.05	-2.65	3.21E-03	3.93E-02
cg05949660	MICAL1;MICAL1	6	TSS1500;TSS1500	Island	34.44	3.59	37.09	3.30	-2.65	2.96E-03	3.71E-02
cg25130962	GNAS;GNAS;GNAS;GNAS;GNAS;GNAS	20	3'UTR;TSS1500;TSS1500;Body;TSS1500;3'U	Island	31.22	3.82	33.87	4.59	-2.65	3.34E-04	7.40E-03
cg04285869	SLC6A18	5	TSS200	N_Shore	68.16	4.49	70.81	3.90	-2.65	1.95E-03	2.78E-02
cg21974239	MAPK12	22	TSS1500	S_Shore	19.18	4.29	21.83	4.41	-2.65	6.60E-04	1.25E-02
cg23201938		5	Intergenic	Open sea	80.17	3.80	82.82	3.37	-2.65	1.67E-03	2.49E-02
cg19486271	DDB2	11	TSS1500	N_Shore	12.14	4.11	14.79	4.32	-2.65	3.56E-03	4.21E-02
cg07757702		3	Intergenic	S_Shelf	74.89	3.56	77.54	3.85	-2.65	1.47E-04	3.84E-03
cg02431672		1	Intergenic	Island	34.55	3.97	37.20	4.14	-2.65	1.57E-03	2.38E-02
cg19929617	ELOVL4	6	Body	N_Shore	18.80	3.83	21.45	4.24	-2.65	6.10E-04	1.18E-02

cg14182819		20	Intergenic	S_Shore	45.54	3.55	48.18	3.82	-2.65	1.04E-03	1.76E-02
cg17552295		7	Intergenic	Open sea	68.86	5.53	71.51	4.57	-2.65	2.75E-03	3.53E-02
cg10734448	TNXB	6	Body	N_Shelf	78.44	4.28	81.09	3.23	-2.65	1.62E-03	2.44E-02
cg02770054	ATP4A	19	Body	Island	21.79	4.73	24.44	5.41	-2.65	1.89E-03	2.72E-02
cg02393907	ACTR3C	7	5'UTR	Open sea	77.83	4.54	80.47	3.05	-2.65	2.72E-03	3.50E-02
cg05295584	AKNAD1	1	TSS200	Open sea	60.24	4.83	62.89	5.09	-2.65	2.63E-03	3.42E-02
cg10904109	GRM1;GRM1	6	3'UTR;Body	Island	16.29	3.83	18.93	4.16	-2.65	2.89E-03	3.65E-02
cg23849483	SYDE1	19	Body	Island	58.93	3.28	61.57	3.61	-2.65	1.64E-03	2.46E-02
cg13618604		4	Intergenic	Island	25.11	3.96	27.75	5.00	-2.65	3.71E-03	4.33E-02
cg23196781	FGFR1OP2	12	3'UTR	Open sea	88.79	4.44	91.43	2.48	-2.65	1.61E-03	2.42E-02
cg03690956		10	Intergenic	Island	22.10	3.60	24.75	3.86	-2.65	5.96E-04	1.16E-02
cg26297203		2	Intergenic	N_Shelf	61.47	2.95	64.12	3.02	-2.65	3.36E-04	7.44E-03
cg13588388	NACC2	9	5'UTR	Open sea	47.65	4.14	50.29	4.44	-2.65	1.17E-03	1.93E-02
cg09241332	ZIC1	3	TSS1500	N_Shore	15.80	3.55	18.45	4.57	-2.65	2.02E-04	4.97E-03
cg22911898		11	Intergenic	N_Shore	31.24	4.26	33.89	4.18	-2.65	3.54E-03	4.20E-02
cg01195910	RASGEF1A	10	5'UTR	S_Shelf	69.04	3.39	71.68	3.75	-2.65	3.61E-03	4.25E-02
cg07358136		13	Intergenic	Open sea	82.51	3.16	85.16	2.49	-2.65	1.74E-05	6.17E-04
cg15109118	FARS2	6	Body	Open sea	87.37	4.76	90.02	4.31	-2.65	1.38E-03	2.17E-02
cg03610527	HDLBP;HDLBP	2	Body;Body	Open sea	75.58	3.10	78.23	2.98	-2.65	1.07E-04	2.96E-03
cg27267377		8	Intergenic	Open sea	86.88	3.03	89.53	2.32	-2.65	3.25E-05	1.07E-03
cg11995638	FBXO25;FBXO25;FBXO25	8	TSS1500;TSS1500;TSS1500	N_Shore	64.47	3.76	67.11	3.94	-2.65	3.72E-03	4.34E-02
cg13741876		6	Intergenic	Open sea	78.32	4.54	80.97	4.07	-2.65	9.80E-04	1.69E-02
cg22923398	NCRNA00164;NCRNA00164	2	TSS1500;Body	Open sea	43.59	3.64	46.24	3.36	-2.65	2.71E-03	3.49E-02
cg11204987	AGBL4	1	Body	Open sea	70.69	3.30	73.34	3.01	-2.65	9.18E-05	2.61E-03
cg14286660	C7orf50;C7orf50;C7orf50	7	Body;Body;Body	S_Shore	75.56	3.82	78.21	3.29	-2.65	2.48E-03	3.28E-02
cg01792592	MEGF6	1	Body	Open sea	74.96	5.45	77.61	3.87	-2.65	1.16E-03	1.91E-02
cg00541303		1	Intergenic	Open sea	60.91	5.28	63.56	4.91	-2.64	3.05E-03	3.79E-02
cg00510111	HECW2	2	TSS1500	Island	26.94	3.15	29.59	3.47	-2.64	2.12E-04	5.18E-03
cg24718722	FOXI2	10	Body	Island	17.08	2.85	19.72	3.43	-2.64	2.03E-03	2.86E-02
cg00558749		1	Intergenic	Island	17.79	2.94	20.44	4.18	-2.64	2.85E-03	3.61E-02
cg01565314	EXOC3L2	19	TSS200	Island	28.30	3.11	30.94	3.41	-2.64	6.25E-04	1.20E-02
cg05953373	CBFA2T3	16	Body	Open sea	76.66	4.04	79.30	3.73	-2.64	2.74E-03	3.52E-02
cg09484032		12	Intergenic	N_Shelf	22.44	3.41	25.09	4.05	-2.64	9.40E-04	1.64E-02
cg21517792	MTA1	14	TSS1500	S_Shelf	70.42	4.42	73.06	3.75	-2.64	2.33E-03	3.15E-02
cg13331200	CADM2;CADM2;CADM2;CADM2	3	5'UTR;1stExon;5'UTR;1stExon	Island	10.99	2.95	13.63	4.06	-2.64	2.08E-03	2.90E-02
cg20809087	BRUNOL6;BRUNOL6	15	5'UTR;1stExon	Island	19.39	4.64	22.03	4.92	-2.64	1.68E-03	2.50E-02
cg23355997	DULLARD;DULLARD;C17orf81;C17orf8	17	5'UTR;5'UTR;TSS1500;TSS1500;TSS1500;T	Island	50.23	4.10	52.88	3.66	-2.64	3.39E-03	4.08E-02
cg11991735	C7orf50;C7orf50;C7orf50	7	Body;Body;Body	Open sea	55.62	5.24	58.27	5.66	-2.64	4.20E-03	4.72E-02
cg09346546		2	Intergenic	N_Shore	19.17	3.66	21.82	3.81	-2.64	1.30E-03	2.08E-02
cg06285307		3	Intergenic	Open sea	54.10	4.41	56.74	3.63	-2.64	5.76E-04	1.13E-02
cg25685519	NID2	14	TSS1500	Island	21.97	3.10	24.61	4.71	-2.64	1.64E-03	2.46E-02
cg21385746	LOC150568	2	Body	Open sea	85.97	3.17	88.62	2.54	-2.64	2.37E-05	8.13E-04
cg27036936	KIAA1462	10	3'UTR	Open sea	82.39	3.95	85.03	3.90	-2.64	2.15E-04	5.23E-03
cg25046790		12	Intergenic	Open sea	59.19	3.64	61.84	3.33	-2.64	2.05E-04	5.04E-03
cg17928286		1	Intergenic	Open sea	67.72	3.58	70.36	3.66	-2.64	2.81E-03	3.58E-02
cg00545315		10	Intergenic	N_Shelf	70.53	4.00	73.17	3.73	-2.64	2.48E-03	3.28E-02
cg08553963		12	Intergenic	N_Shelf	55.43	3.50	58.08	2.58	-2.64	1.75E-03	2.57E-02
cg07271302	MARCKS	6	TSS1500	N_Shelf	10.87	3.30	13.51	3.79	-2.64	3.49E-05	1.14E-03
cg27514530		8	Intergenic	Open sea	86.81	1.81	89.45	1.78	-2.64	2.09E-09	1.15E-07
cg12015884	C21orf33;C21orf33	21	Body;Body	Island	66.43	4.73	69.07	5.47	-2.64	3.46E-03	4.13E-02
cg13444518		9	Intergenic	S_Shore	11.71	2.26	14.35	3.23	-2.64	5.95E-05	1.81E-03
cg09893576		12	Intergenic	N_Shore	25.59	3.65	28.24	3.35	-2.64	1.88E-04	4.70E-03
cg20682563	TCF7;TCF7;TCF7;TCF7;TCF7;TCF7;TCF7	5	Body;Body;Body;Body;Body;Body	Open sea	40.19	3.78	42.83	3.90	-2.64	4.50E-03	4.95E-02

cg23715045	VSIG2	11	Body	Open sea	44.75	3.10	47.39	3.61	-2.64	2.86E-03	3.62E-02
cg14361409		14	Intergenic	Island	11.83	3.29	14.47	3.97	-2.64	1.84E-04	4.62E-03
cg12690333	C1orf103;C1orf103	1	Body;5'UTR	N_Shelf	75.19	3.73	77.83	3.61	-2.64	4.28E-03	4.78E-02
cg27000386	OGFOD2	12	Body	N_Shore	79.37	4.12	82.01	3.87	-2.64	3.95E-03	4.53E-02
cg15965716	PBX1	1	Body	Open sea	78.87	4.51	81.51	3.70	-2.64	3.10E-04	6.98E-03
cg18389339	DLGAP1;DLGAP1	18	Body;Body	S_Shore	77.85	4.74	80.49	4.99	-2.64	2.05E-03	2.88E-02
cg03798336	CYTIP	2	3'UTR	Open sea	81.74	4.22	84.38	4.25	-2.64	1.35E-04	3.59E-03
cg20985587	CORO6	17	Body	Island	35.56	3.77	38.20	4.02	-2.64	3.25E-03	3.95E-02
cg22680911		3	Intergenic	S_Shore	73.02	2.87	75.66	2.39	-2.64	3.06E-05	1.01E-03
cg27553890	SSH1;SSH1;SSH1	12	TSS200;Body;Body	Open sea	62.71	4.08	65.35	3.71	-2.64	4.11E-03	4.65E-02
cg20643675	MIR1323	19	TSS1500	Open sea	57.89	4.93	60.53	4.63	-2.64	1.45E-03	2.25E-02
cg15699226	EDNRB;EDNRB;EDNRB	13	1stExon;1stExon;Body	Island	10.28	2.76	12.92	3.03	-2.64	1.43E-04	3.76E-03
cg05281623	TMPRSS8	16	TSS1500	S_Shore	61.22	3.55	63.85	3.30	-2.64	3.37E-04	7.46E-03
cg16531181		10	Intergenic	Open sea	86.92	4.67	89.56	2.92	-2.64	7.83E-04	1.43E-02
cg07927266	C1orf97	1	Body	S_Shore	86.02	3.86	88.66	3.54	-2.64	3.00E-04	6.79E-03
cg06139166	ZDHHC1	16	Body	Open sea	76.73	3.19	79.36	3.56	-2.64	4.92E-04	1.00E-02
cg20745248	ATP2B2;MIR885;ATP2B2	3	Body;TSS200;Body	Open sea	74.77	3.88	77.41	3.60	-2.64	3.19E-03	3.91E-02
cg02780849	GNG4;GNG4;GNG4	1	TSS1500;TSS200;TSS200	N_Shore	13.99	3.05	16.63	3.71	-2.64	7.90E-04	1.43E-02
cg05390279		1	Intergenic	Open sea	58.02	3.16	60.66	3.45	-2.64	1.25E-04	3.36E-03
cg12939390	SRBD1	2	Body	Open sea	69.02	4.54	71.66	3.71	-2.64	3.26E-03	3.96E-02
cg20203400	TMEM182	2	TSS200	Open sea	82.57	3.47	85.21	3.48	-2.64	7.58E-04	1.39E-02
cg07280582	PCBP2;PCBP2;PCBP2;PCBP2;PCBP2;PC	12	Body;Body;Body;Body;Body;Body;Body	Open sea	76.30	4.96	78.94	4.42	-2.64	1.04E-03	1.76E-02
cg11746651	KIAA0495	1	TSS1500	S_Shore	74.48	3.80	77.12	3.81	-2.64	1.14E-03	1.89E-02
cg25585712		4	Intergenic	Island	32.78	5.07	35.42	5.40	-2.64	1.17E-03	1.92E-02
cg04215179	C7orf46;C7orf46;C7orf46	7	TSS200;TSS200;TSS200	Island	19.73	2.12	22.37	2.18	-2.64	2.01E-06	8.62E-05
cg23115487		13	Intergenic	Open sea	37.42	5.77	40.06	4.83	-2.64	4.23E-03	4.74E-02
cg00737978	C18orf1;C18orf1;C18orf1;C18orf1;C18	18	5'UTR;Body;Body;5'UTR;1stExon;1stExon	Open sea	51.27	3.55	53.91	3.99	-2.64	1.20E-03	1.96E-02
cg08204714	JPH3	16	Body	S_Shelf	75.26	3.12	77.90	3.00	-2.64	1.89E-05	6.64E-04
cg13666041	ACTN1;ACTN1;ACTN1	14	Body;Body;Body	N_Shore	77.80	4.79	80.44	3.61	-2.64	1.48E-03	2.29E-02
cg24127115		3	Intergenic	Open sea	87.35	3.67	89.99	1.72	-2.64	4.27E-04	8.98E-03
cg03757805	C13orf29	13	Body	Open sea	72.56	4.32	75.20	3.36	-2.64	2.68E-03	3.46E-02
cg13020386		3	Intergenic	Open sea	80.61	5.20	83.25	5.31	-2.64	3.53E-03	4.19E-02
cg02900183	CIB3	19	TSS200	Open sea	76.49	4.83	79.12	4.65	-2.64	3.29E-03	3.99E-02
cg15163719	TNXB	6	Body	N_Shelf	71.00	4.58	73.63	4.48	-2.64	1.33E-03	2.12E-02
cg04215950	CAMTA1	1	Body	S_Shelf	67.36	4.00	70.00	4.35	-2.64	1.97E-04	4.89E-03
cg04304033	ISYNA1;ISYNA1;ISYNA1	19	Body;Body;Body	Island	86.35	3.15	88.99	2.50	-2.64	8.00E-05	2.32E-03
cg05938633	NXN	17	Body	Open sea	80.36	5.77	83.00	4.53	-2.64	1.00E-03	1.72E-02
cg03812837	PARD6B	20	TSS1500	N_Shore	66.37	5.18	69.00	4.76	-2.64	2.73E-03	3.50E-02
cg24505892		10	Intergenic	Island	17.54	2.83	20.17	3.78	-2.64	2.11E-04	5.16E-03
cg02056098	SCN9A	2	TSS1500	S_Shore	77.08	4.64	79.71	4.62	-2.64	1.51E-03	2.32E-02
cg03137131	ZFYVE28	4	Body	Island	44.33	3.90	46.97	4.15	-2.64	2.12E-03	2.94E-02
cg20641095	STAT5B	17	Body	Open sea	19.37	3.72	22.01	3.00	-2.64	2.11E-03	2.93E-02
cg21005459	CD2AP	6	Body	S_Shelf	62.16	3.25	64.80	3.27	-2.64	2.38E-04	5.68E-03
cg04896949	RAB42	1	TSS1500	N_Shore	53.12	4.91	55.76	4.78	-2.64	7.27E-04	1.35E-02
cg03930921	KIRREL3;KIRREL3	11	Body;Body	Open sea	72.96	4.66	75.60	3.93	-2.64	1.34E-03	2.12E-02
cg07829379	LHX1	17	TSS1500	Island	20.12	3.22	22.75	3.16	-2.64	2.62E-05	8.87E-04
cg04160694	DBN1;DBN1	5	Body;Body	S_Shelf	80.71	2.61	83.35	2.58	-2.64	1.31E-05	4.81E-04
cg15952840		8	Intergenic	Open sea	61.33	5.20	63.97	4.91	-2.64	3.12E-03	3.85E-02
cg05486616		7	Intergenic	Open sea	70.11	3.73	72.75	3.52	-2.64	1.39E-03	2.18E-02
cg13948082	CYP26B1	2	Body	Island	14.07	3.61	16.71	4.27	-2.64	3.48E-03	4.15E-02
cg02342415	IQSEC1;IQSEC1	3	3'UTR;3'UTR	Island	75.35	4.09	77.99	2.80	-2.64	2.56E-03	3.35E-02
cg03822873	AHCYL2;AHCYL2	7	Body;Body	Open sea	75.16	4.94	77.80	3.78	-2.64	4.40E-03	4.87E-02
cg09059945	MEST	7	TSS200	Island	10.48	3.00	13.11	3.84	-2.63	5.87E-04	1.15E-02

cg03479694	DLX1;DLX1	2	3'UTR;3'UTR	Island	26.17	3.43	28.81	4.01	-2.63	4.03E-04	8.57E-03
cg06687848		10	Intergenic	N_Shore	24.85	3.46	27.49	4.12	-2.63	8.88E-05	2.54E-03
cg25821304	RNF135;RNF135	17	TSS200;TSS200	N_Shore	14.69	3.20	17.33	4.60	-2.63	3.32E-03	4.01E-02
cg05934592		14	Intergenic	Open sea	13.62	2.62	16.25	3.03	-2.63	1.24E-04	3.35E-03
cg24356700	SRRT;SRRT;SRRT;SRRT	7	Body;Body;Body;Body	N_Shore	64.54	4.07	67.17	3.74	-2.63	2.23E-03	3.05E-02
cg11722990	HS6ST3	13	Body	Open sea	83.79	3.54	86.42	2.74	-2.63	1.79E-04	4.52E-03
cg27123256	BCL11B;BCL11B	14	Body;Body	Open sea	60.02	3.89	62.65	3.47	-2.63	1.94E-03	2.77E-02
cg27257955	SLC44A4	6	Body	S_Shelf	72.95	3.31	75.59	3.75	-2.63	2.90E-04	6.63E-03
cg03703356	CKB	14	TSS200	Island	18.78	2.72	21.42	4.09	-2.63	1.14E-04	3.13E-03
cg27064063	DCBLD1	6	Body	Island	43.88	4.38	46.52	3.73	-2.63	1.84E-03	2.66E-02
cg23311108		5	Intergenic	Open sea	26.04	4.46	28.67	4.55	-2.63	3.83E-03	4.43E-02
cg03986620	MMP15	16	Body	Island	84.04	3.71	86.67	2.35	-2.63	3.08E-04	6.94E-03
cg02776659	C21orf57	21	Body	Island	15.09	4.08	17.72	4.13	-2.63	1.19E-04	3.24E-03
cg23658354	PTPRO;PTPRO;PTPRO;PTPRO;PTPRO;F	12	TSS1500;TSS1500;TSS1500;TSS1500;Body;	Open sea	78.11	5.59	80.74	4.63	-2.63	1.77E-03	2.59E-02
cg22577701	TRIM15	6	Body	N_Shelf	33.10	4.36	35.73	3.80	-2.63	4.57E-04	9.45E-03
cg06311920	COTL1	16	3'UTR	Open sea	55.87	3.77	58.50	3.96	-2.63	2.62E-03	3.41E-02
cg27326226	GJB2	13	TSS1500	Island	11.85	3.30	14.49	3.76	-2.63	1.33E-04	3.54E-03
cg01333824	TCF25	16	Body	S_Shelf	80.76	4.11	83.39	3.44	-2.63	2.13E-03	2.96E-02
cg16254946	GLIS1	1	Body	Open sea	55.52	3.34	58.15	3.51	-2.63	5.76E-04	1.13E-02
cg25627689		19	Intergenic	S_Shelf	85.50	3.57	88.13	2.55	-2.63	4.71E-04	9.68E-03
cg01419164	EBF3	10	Body	Open sea	82.96	3.44	85.59	3.24	-2.63	3.53E-04	7.73E-03
cg12207450	NR2F1;NR2F1	5	1stExon;5'UTR	N_Shore	13.04	1.99	15.67	2.59	-2.63	1.41E-05	5.12E-04
cg23970523	FKBP8	19	5'UTR	Island	20.34	3.42	22.97	3.23	-2.63	3.40E-05	1.11E-03
cg24421839	UBE2QP1	15	Body	N_Shelf	78.57	4.55	81.20	4.34	-2.63	3.08E-03	3.81E-02
cg26177243	ARHGEF10	8	Body	Island	89.00	6.31	91.63	2.88	-2.63	2.45E-03	3.26E-02
cg13234275	PHRF1	11	Body	N_Shelf	73.64	3.50	76.27	3.07	-2.63	1.01E-03	1.73E-02
cg04680209	MYT1L	2	Body	Island	73.16	3.12	75.79	2.53	-2.63	5.04E-05	1.56E-03
cg24470929	LAPTM4B	8	Body	S_Shelf	68.24	3.98	70.87	3.22	-2.63	3.11E-03	3.84E-02
cg22868624	RCN3	19	Body	S_Shelf	84.06	4.53	86.69	4.06	-2.63	6.34E-04	1.22E-02
cg27056880	ERBB2IP;ERBB2IP	5	3'UTR;3'UTR	Open sea	87.99	5.14	90.62	3.70	-2.63	3.93E-03	4.51E-02
cg22567473	HERC1	15	Body	Open sea	19.41	4.39	22.04	4.37	-2.63	3.10E-04	6.98E-03
cg06692929	CD7	17	Body	N_Shelf	48.33	5.06	50.96	5.76	-2.63	3.31E-04	7.35E-03
cg21993547	NOTCH4	6	Body	Open sea	72.40	4.09	75.02	3.53	-2.63	1.98E-03	2.80E-02
cg07953845		7	Intergenic	Open sea	80.43	3.08	83.06	3.11	-2.63	3.24E-05	1.07E-03
cg09085792	TP63;TP63;TP63;TP63;MIR944;TP63;T	3	Body;Body;Body;Body;TSS1500;Body;Body	Open sea	79.10	3.54	81.73	3.46	-2.63	9.24E-04	1.62E-02
cg00293599	SPDEF	6	3'UTR	Open sea	77.41	4.15	80.04	3.99	-2.63	2.74E-03	3.51E-02
cg18313182	PNPLA2;PNPLA2	11	1stExon;5'UTR	N_Shore	39.69	3.89	42.31	4.48	-2.63	2.99E-03	3.74E-02
cg20802255		2	Intergenic	N_Shore	5.77	1.47	8.40	1.89	-2.63	4.44E-10	2.51E-08
cg17654335		3	Intergenic	Island	44.45	3.70	47.07	3.49	-2.63	2.93E-03	3.68E-02
cg10802480	FLJ42875;PRDM16;FLJ42875;PRDM16	1	Body;TSS1500;Body;TSS1500	Island	12.33	2.68	14.95	4.38	-2.63	1.22E-03	1.98E-02
cg06604447	RAGE	14	Body	Open sea	47.71	2.37	50.34	2.60	-2.63	9.70E-06	3.66E-04
cg08120263	FOXG1	14	TSS1500	N_Shore	22.85	3.82	25.48	4.12	-2.63	1.14E-03	1.89E-02
cg06806891		3	Intergenic	N_Shelf	77.12	4.17	79.75	3.14	-2.63	4.15E-03	4.68E-02
cg16621856		12	Intergenic	S_Shelf	74.95	4.00	77.57	3.15	-2.63	3.66E-03	4.29E-02
cg00881585		4	Intergenic	N_Shore	16.31	2.96	18.94	3.41	-2.63	2.50E-04	5.92E-03
cg12328429	ME3;ME3;ME3	11	Body;Body;Body	Open sea	85.22	3.66	87.84	2.90	-2.63	1.67E-03	2.49E-02
cg19328640		20	Intergenic	S_Shelf	85.36	3.44	87.98	3.21	-2.63	2.52E-05	8.58E-04
cg04606367		7	Intergenic	Island	15.27	2.96	17.90	3.11	-2.63	1.38E-05	5.03E-04
cg27402043		17	Intergenic	Open sea	20.87	3.26	23.50	3.65	-2.63	5.18E-05	1.60E-03
cg14331163	RORB	9	TSS1500	N_Shore	17.62	3.89	20.24	4.77	-2.63	8.45E-04	1.51E-02
cg08713073	PCDHB18	5	Body	N_Shore	12.05	3.20	14.68	4.52	-2.63	4.13E-03	4.66E-02
cg03394422	RPS6KL1	14	TSS200	N_Shore	88.45	3.57	91.07	1.94	-2.63	1.22E-04	3.29E-03
cg07232945	ADORA1;ADORA1	1	TSS1500;TSS1500	N_Shore	10.36	2.49	12.99	2.85	-2.63	1.97E-05	6.89E-04

cg06458238	CASKIN2;CASKIN2	17	Body;TSS200	Open sea	72.46	4.19	75.08	3.87	-2.63	4.29E-03	4.79E-02
cg12052497	LPIN2	18	TSS200	N_Shore	71.97	3.38	74.59	4.01	-2.63	1.11E-04	3.04E-03
cg21067775	EPS15L1	19	Body	Island	76.62	4.00	79.25	3.33	-2.63	2.08E-03	2.91E-02
cg11160784	RGL2;RGL2	6	Body;Body	N_Shore	72.07	3.76	74.70	3.96	-2.63	1.67E-03	2.48E-02
cg23622026	ANKRD5;ANKRD5	20	3'UTR;3'UTR	Open sea	59.30	4.35	61.92	4.47	-2.63	4.49E-03	4.94E-02
cg04731624		5	Intergenic	Open sea	56.15	5.39	58.77	5.10	-2.63	3.69E-03	4.32E-02
cg22129220	FAM65B	6	5'UTR	N_Shore	65.24	5.15	67.86	6.06	-2.63	3.39E-03	4.08E-02
cg03744043		18	Intergenic	N_Shore	37.97	4.29	40.59	4.83	-2.63	3.26E-03	3.96E-02
cg00901741	CSN1S2B	4	Body	Open sea	88.42	3.85	91.04	3.13	-2.63	4.07E-04	8.64E-03
cg18651470		6	Intergenic	N_Shore	13.87	2.87	16.49	3.95	-2.63	9.94E-04	1.70E-02
cg19371339	LCE2B	1	TSS1500	Open sea	65.25	5.60	67.87	5.91	-2.63	3.42E-03	4.10E-02
cg19717061	ZBTB20;ZBTB20;ZBTB20;ZBTB20;ZBTB	3	5'UTR;5'UTR;5'UTR;5'UTR;5'UTR;5'UTR	Open sea	69.44	5.45	72.06	4.76	-2.62	2.28E-03	3.09E-02
cg12682870		6	Intergenic	Open sea	37.37	4.79	40.00	3.87	-2.62	1.36E-04	3.60E-03
cg12888113	MASP2;MASP2	1	Body;Body	Open sea	42.93	2.81	45.55	3.06	-2.62	1.18E-03	1.94E-02
cg00785738	CD72	9	Body	Island	23.79	5.28	26.41	6.79	-2.62	1.43E-03	2.22E-02
cg11080552	RERE;RERE	1	Body;Body	Open sea	80.55	3.12	83.18	3.28	-2.62	7.47E-04	1.38E-02
cg09395844	LDLRAD3	11	Body	Open sea	73.83	3.93	76.45	4.29	-2.62	1.58E-03	2.39E-02
cg26221796	CDC42EP5	19	5'UTR	N_Shore	65.11	4.94	67.73	3.96	-2.62	2.95E-03	3.70E-02
cg16373984	C14orf143	14	Body	Open sea	80.11	4.05	82.73	4.14	-2.62	1.24E-03	2.01E-02
cg18739800	HIST1H2BN;HIST1H2AK	6	TSS1500;1stExon	Island	11.32	2.59	13.95	4.50	-2.62	2.03E-03	2.85E-02
cg11464053	MUPCDH;MUPCDH	11	Body;Body	S_Shore	83.86	4.39	86.48	2.96	-2.62	1.49E-03	2.29E-02
cg21898424	VMO1;VMO1;VMO1;VMO1;VMO1;VM	17	1stExon;5'UTR;1stExon;5'UTR;5'UTR;1stEx	S_Shore	42.30	4.56	44.93	4.91	-2.62	2.77E-03	3.54E-02
cg16522260		11	Intergenic	Open sea	67.80	4.84	70.42	4.62	-2.62	4.13E-03	4.66E-02
cg09649382	ECT2L	6	TSS1500	Open sea	76.36	3.21	78.98	3.50	-2.62	8.95E-04	1.58E-02
cg07583420	INS-IGF2;IGF2;IGF2;IGF2	11	Body;Body;5'UTR;5'UTR	N_Shore	4.39	1.39	7.01	2.97	-2.62	6.44E-07	2.96E-05
cg09734761		16	Intergenic	Open sea	61.24	4.85	63.87	4.67	-2.62	1.37E-03	2.15E-02
cg03363256	PAG1	8	5'UTR	N_Shore	9.18	2.44	11.81	3.19	-2.62	5.05E-06	2.02E-04
cg12856782		20	Intergenic	N_Shelf	78.07	3.89	80.69	3.74	-2.62	2.76E-04	6.37E-03
cg20033239	LOC84856	10	Body	Island	85.28	3.91	87.91	3.45	-2.62	7.26E-04	1.35E-02
cg03782220		12	Intergenic	S_Shelf	74.16	4.11	76.78	3.78	-2.62	2.85E-03	3.61E-02
cg19991046	DENND3	8	Body	Island	61.35	3.69	63.97	3.35	-2.62	1.16E-03	1.91E-02
cg18175611		14	Intergenic	N_Shelf	72.08	4.44	74.70	4.18	-2.62	4.53E-03	4.96E-02
cg19763699	STAM	10	3'UTR	Open sea	84.77	4.74	87.39	4.01	-2.62	1.95E-03	2.78E-02
cg17569124	HOXA5	7	TSS1500	Island	14.81	2.84	17.43	3.63	-2.62	1.43E-03	2.23E-02
cg15076820	KIFC1	6	Body	N_Shore	79.31	4.58	81.93	5.50	-2.62	3.71E-03	4.34E-02
cg02679028	LY6D	8	TSS1500	Open sea	66.21	4.52	68.83	4.34	-2.62	2.57E-03	3.37E-02
cg23234674	SNORA74B;ATP6V0E1	5	Body;3'UTR	Open sea	78.37	3.50	81.00	3.04	-2.62	6.06E-04	1.17E-02
cg11862081	PC;LRFN4;PC;LRFN4;PC	11	Body;5'UTR;Body;1stExon;Body	Island	71.49	4.46	74.11	4.32	-2.62	2.22E-03	3.04E-02
cg06745944	DNAH10	12	TSS1500	N_Shore	16.91	5.80	19.53	5.32	-2.62	3.78E-03	4.39E-02
cg11109721	GPX3	5	3'UTR	Open sea	77.54	3.80	80.17	3.52	-2.62	6.68E-04	1.26E-02
cg04202736	PLA2G4D	15	Body	Island	87.54	4.40	90.16	3.91	-2.62	2.12E-03	2.95E-02
cg06539006	CPO	2	Body	Open sea	42.41	6.18	45.03	4.65	-2.62	2.54E-03	3.33E-02
cg02880444	RAB11FIP4	17	3'UTR	Open sea	41.36	3.61	43.99	3.53	-2.62	7.93E-05	2.30E-03
cg04942564	DNAJC5	20	Body	N_Shore	70.26	3.15	72.88	3.45	-2.62	4.85E-04	9.89E-03
cg03624576		19	Intergenic	Island	23.92	2.67	26.54	2.92	-2.62	1.76E-04	4.46E-03
cg17588904	HIPK1;HIPK1;HIPK1	1	Body;Body;TSS1500	Open sea	57.88	3.67	60.50	3.82	-2.62	5.98E-04	1.16E-02
cg20103938	ELF2;ELF2	4	Body;Body	Island	16.95	3.93	19.57	3.99	-2.62	3.23E-03	3.94E-02
cg13664635	C9orf3	9	Body	Open sea	79.50	2.81	82.12	2.52	-2.62	7.24E-06	2.81E-04
cg10199913	HIST1H3C	6	1stExon	Island	20.53	3.10	23.15	2.85	-2.62	4.47E-04	9.29E-03
cg04674201		6	Intergenic	Open sea	39.26	5.11	41.88	5.69	-2.62	1.91E-03	2.74E-02
cg13564742	FOXE1	9	TSS1500	N_Shore	16.62	3.04	19.25	3.64	-2.62	1.31E-03	2.09E-02
cg01820007	NDUFS4	5	Body	S_Shore	85.09	4.09	87.71	2.68	-2.62	3.98E-03	4.55E-02
cg00558645		1	Intergenic	Open sea	86.86	3.35	89.48	2.57	-2.62	1.04E-05	3.91E-04

cg03241244	KDM2B;KDM2B	12	Body;Body	Island	43.71	4.70	46.33	5.18	-2.62	1.06E-03	1.79E-02
cg04610897	CD81	11	3'UTR	S_Shore	78.65	3.45	81.27	3.08	-2.62	4.41E-04	9.19E-03
cg19878516	PANX2;PANX2;PANX2	22	TSS1500;TSS1500;TSS1500	Island	12.43	3.03	15.05	2.92	-2.62	4.93E-06	1.98E-04
cg09824402	NTNG1;NTNG1;NTNG1	1	TSS1500;TSS1500;TSS1500	N_Shore	80.77	4.15	83.38	4.47	-2.62	8.26E-04	1.48E-02
cg23774453	MTMR10;MTMR15	15	3'UTR;3'UTR	Open sea	84.67	3.97	87.29	3.29	-2.62	2.84E-03	3.61E-02
cg22959742	FRMD4A	10	Body	Open sea	72.07	4.22	74.69	3.70	-2.62	1.88E-03	2.71E-02
cg25889918		2	Intergenic	Open sea	76.17	4.60	78.79	4.74	-2.62	8.19E-04	1.47E-02
cg09432376	APOL6	22	TSS200	Open sea	25.62	4.48	28.24	4.73	-2.62	9.77E-04	1.68E-02
cg19114999		3	Intergenic	Open sea	72.90	5.00	75.52	4.56	-2.62	3.99E-03	4.56E-02
cg18879950		6	Intergenic	Open sea	74.70	3.41	77.32	3.43	-2.62	2.02E-04	4.97E-03
cg10778337	CAV3;CAV3	3	Body;Body	Open sea	74.42	4.35	77.04	4.49	-2.62	1.30E-03	2.08E-02
cg10768996	TMCC2	1	Body	N_Shelf	74.34	3.41	76.96	3.63	-2.62	1.59E-03	2.40E-02
cg24912473	SGTA	19	3'UTR	Island	87.78	4.92	90.40	3.78	-2.62	3.19E-03	3.91E-02
cg19326217		4	Intergenic	Open sea	83.48	4.30	86.10	4.88	-2.62	9.18E-04	1.61E-02
cg24355809	NXN	17	Body	S_Shore	76.07	3.75	78.68	3.26	-2.62	5.00E-04	1.01E-02
cg14692854		2	Intergenic	Open sea	34.77	5.09	37.39	5.01	-2.62	1.39E-03	2.18E-02
cg19335617	C7orf58;C7orf58	7	Body;Body	Open sea	85.15	3.32	87.76	3.04	-2.62	8.20E-04	1.47E-02
cg15998779		3	Intergenic	Island	30.57	4.80	33.19	3.74	-2.62	1.76E-03	2.59E-02
cg16857548	LPIN2	18	TSS200	N_Shore	61.73	3.76	64.35	4.08	-2.62	2.53E-04	5.95E-03
cg24415793	SLC4A8;SLC4A8	12	TSS200;TSS200	N_Shore	19.11	4.46	21.73	4.89	-2.62	1.07E-03	1.79E-02
cg13077784	NAV1	1	Body	N_Shelf	66.28	4.00	68.90	3.83	-2.62	1.74E-03	2.57E-02
cg04398978	CBX4	17	TSS1500	Island	28.85	3.58	31.46	4.54	-2.62	3.45E-03	4.12E-02
cg06587495	ENOX1	13	5'UTR	Open sea	85.28	5.47	87.90	3.87	-2.62	2.49E-03	3.29E-02
cg04857172	SRL	16	Body	Open sea	77.50	3.98	80.12	3.52	-2.62	9.59E-04	1.66E-02
cg10441789		17	Intergenic	N_Shelf	12.15	2.85	14.76	3.47	-2.62	2.63E-04	6.13E-03
cg16428857	WDR46;WDR46	6	Body;Body	S_Shelf	78.74	3.78	81.35	3.58	-2.62	3.27E-03	3.97E-02
cg21163444	ZNF385A;ZNF385A;ZNF385A	12	Body;Body;Body	S_Shore	55.72	3.87	58.34	3.96	-2.62	3.88E-03	4.47E-02
cg08332992		10	Intergenic	N_Shelf	85.50	3.32	88.12	3.36	-2.62	5.17E-04	1.04E-02
cg19534805	KHSRP	19	Body	S_Shelf	85.05	4.01	87.67	3.48	-2.62	3.77E-03	4.38E-02
cg17561417	FBLN2;FBLN2	3	TSS200;TSS200	Island	23.41	4.16	26.03	4.60	-2.62	2.72E-03	3.50E-02
cg26836717		1	Intergenic	Open sea	83.81	2.85	86.43	2.53	-2.62	6.08E-05	1.84E-03
cg03556313		11	Intergenic	S_Shore	13.94	2.52	16.56	4.58	-2.62	2.59E-03	3.39E-02
cg01877936		6	Intergenic	N_Shore	21.17	4.53	23.79	3.38	-2.62	2.22E-04	5.38E-03
cg24193797	COX4I2	20	TSS1500	N_Shore	51.81	2.80	54.43	2.94	-2.62	2.84E-04	6.53E-03
cg20318845	SIX2	2	TSS1500	Island	23.35	3.38	25.97	4.04	-2.62	4.38E-04	9.14E-03
cg08691775	NDRG1;NDRG1	8	TSS1500;TSS1500	S_Shore	60.93	6.75	63.55	6.57	-2.62	3.68E-03	4.31E-02
cg03630273	LIN7B	19	Body	Island	65.56	4.02	68.18	4.16	-2.62	3.50E-03	4.16E-02
cg24903183	SNX31	8	TSS200	Island	16.36	3.99	18.97	3.43	-2.62	1.64E-04	4.21E-03
cg27658698	RNF44	5	3'UTR	Open sea	76.79	4.04	79.41	3.27	-2.62	1.05E-03	1.78E-02
cg19166242		1	Intergenic	Open sea	70.21	3.92	72.82	2.91	-2.62	3.52E-03	4.18E-02
cg12530503	MIR9-3	15	TSS200	Island	29.38	2.86	31.99	3.42	-2.62	4.44E-04	9.24E-03
cg10617763	MLF2;MLF2	12	Body;Body	S_Shore	81.99	5.15	84.61	2.24	-2.62	4.08E-04	8.65E-03
cg02568274	HOXC9	12	TSS1500	Island	22.05	2.39	24.67	3.13	-2.62	4.07E-05	1.30E-03
cg18705279	HSF4;HSF4;FBXL8	16	TSS200;TSS200;Body	Island	18.37	4.10	20.98	4.38	-2.62	3.02E-03	3.76E-02
cg14395791	COBRA1	9	Body	N_Shore	85.67	3.66	88.28	2.80	-2.62	1.71E-04	4.35E-03
cg21625881	GNAS;GNAS;GNAS	20	1stExon;1stExon;3'UTR	Island	49.65	3.03	52.26	3.13	-2.62	2.85E-04	6.54E-03
cg15244049	GCM2	6	TSS1500	S_Shore	25.04	4.32	27.65	4.46	-2.62	2.27E-03	3.08E-02
cg26575057		6	Intergenic	S_Shore	41.87	4.22	44.49	4.99	-2.61	1.81E-03	2.64E-02
cg04065297		2	Intergenic	Open sea	83.61	4.29	86.22	4.56	-2.61	2.10E-03	2.92E-02
cg07380021	RASSF5;RASSF5;RASSF5	1	Body;Body;Body	Open sea	49.57	3.72	52.18	4.68	-2.61	1.36E-03	2.15E-02
cg11867016		4	Intergenic	Open sea	39.49	4.91	42.11	5.17	-2.61	1.08E-03	1.81E-02
cg25072766	CARS;CARS;CARS;CARS	11	Body;Body;5'UTR;Body	Open sea	75.59	3.96	78.21	4.08	-2.61	2.23E-03	3.05E-02
cg13573582	RNF220	1	Body	S_Shelf	78.46	3.96	81.07	3.60	-2.61	2.76E-03	3.54E-02

cg25824217	HLA-DPA1	6	Body	Open sea	63.62	7.06	66.24	6.00	-2.61	3.44E-03	4.12E-02
cg07114422	SKI	1	Body	Open sea	21.91	3.44	24.53	3.91	-2.61	2.56E-03	3.36E-02
cg12253851	MAD1L1;MAD1L1;MAD1L1	7	Body;Body;Body	N_Shelf	83.70	3.37	86.32	3.20	-2.61	3.45E-04	7.59E-03
cg06929843		16	Intergenic	S_Shelf	78.10	3.71	80.71	3.76	-2.61	6.58E-04	1.25E-02
cg16808373	BSX	11	Body	S_Shore	19.78	2.48	22.39	3.67	-2.61	2.69E-04	6.25E-03
cg19530885	SCGB3A2	5	TSS200	Open sea	78.86	3.98	81.48	3.18	-2.61	2.88E-03	3.64E-02
cg17182464	PZP	12	Body	Open sea	78.75	3.01	81.36	2.85	-2.61	3.67E-04	7.97E-03
cg00985817		4	Intergenic	Open sea	54.52	6.05	57.13	5.49	-2.61	2.69E-03	3.48E-02
cg07592830	NEK7	1	Body	Open sea	84.31	4.99	86.92	3.90	-2.61	1.56E-03	2.37E-02
cg14287027	CNNM2;CNNM2	10	Body;Body	Open sea	79.90	4.14	82.51	3.36	-2.61	2.97E-03	3.72E-02
cg02915437	BFSP2	3	Body	Open sea	11.51	3.13	14.12	3.81	-2.61	6.28E-04	1.21E-02
cg24145020	INSL5	1	TSS1500	Open sea	72.68	3.99	75.30	3.26	-2.61	1.08E-03	1.81E-02
cg14590843	GDNF	5	5'UTR	Island	16.36	3.01	18.98	2.66	-2.61	1.22E-04	3.29E-03
cg23297230	WDR25;WDR25	14	Body;Body	Open sea	84.52	2.85	87.13	2.05	-2.61	6.13E-06	2.41E-04
cg09051513	PNPLA2	11	Body	Island	90.57	4.06	93.18	2.42	-2.61	4.31E-03	4.80E-02
cg16337108	GCNT2;GCNT2;GCNT2	6	Body;Body;TSS1500	Open sea	81.87	4.67	84.48	3.67	-2.61	4.39E-03	4.86E-02
cg10385188	FAM98B;FAM98B	15	Body;Body	Open sea	85.27	4.25	87.88	3.49	-2.61	7.05E-04	1.32E-02
cg24332770	LCE2B	1	TSS1500	Open sea	55.44	4.98	58.05	4.81	-2.61	3.65E-03	4.29E-02
cg00492979	SH3PXD2A	10	Body	N_Shore	59.17	4.32	61.78	4.40	-2.61	4.27E-03	4.77E-02
cg25425705		3	Intergenic	Open sea	35.25	3.17	37.86	2.90	-2.61	2.42E-04	5.76E-03
cg25365370		2	Intergenic	Open sea	73.76	3.44	76.37	3.41	-2.61	1.76E-03	2.58E-02
cg06546787	DEFB112	6	TSS1500	Open sea	68.32	5.50	70.93	5.72	-2.61	9.82E-04	1.69E-02
cg04858631	DDIT4	10	3'UTR	S_Shore	14.57	2.26	17.18	2.70	-2.61	3.83E-06	1.57E-04
cg11552585	NCLN	19	Body	Island	83.49	3.00	86.10	3.34	-2.61	6.11E-05	1.85E-03
cg05591235	ACOT7;ACOT7;ACOT7;ACOT7	1	Body;Body;Body;Body	Open sea	88.40	2.97	91.01	1.68	-2.61	5.88E-06	2.32E-04
cg00307214	GALNTL1;GALNTL1	14	TSS1500;TSS1500	Island	19.50	4.45	22.11	4.57	-2.61	1.10E-03	1.83E-02
cg25957306	BAZ2A	12	Body	N_Shelf	77.03	3.91	79.64	2.88	-2.61	1.27E-03	2.05E-02
cg03112665		16	Intergenic	S_Shore	28.00	3.81	30.61	4.28	-2.61	4.22E-03	4.73E-02
cg05322822	INPP4A;INPP4A;INPP4A;INPP4A	2	TSS1500;TSS1500;TSS1500;TSS1500	N_Shore	76.84	4.84	79.45	4.93	-2.61	3.22E-03	3.93E-02
cg23499789	ZNF468;ZNF468	19	Body;Body	Open sea	75.72	2.91	78.33	3.60	-2.61	6.48E-04	1.24E-02
cg23945382	MBD2;MBD2;SNORA37	18	Body;Body;TSS200	N_Shore	50.74	3.49	53.34	3.88	-2.61	2.18E-03	3.00E-02
cg03510732	TCF7L2;TCF7L2;TCF7L2;TCF7L2;TCF7L	10	Body;Body;Body;Body;Body;Body	Open sea	32.58	3.09	35.19	3.31	-2.61	9.54E-04	1.65E-02
cg04737087	ANKRD53;ANKRD53	2	TSS1500;TSS1500	N_Shore	28.37	3.88	30.98	4.65	-2.61	1.86E-03	2.69E-02
cg24003850		5	Intergenic	Open sea	88.57	2.52	91.18	1.88	-2.61	1.06E-07	5.27E-06
cg16915316	CTDSP2	12	Body	N_Shore	51.48	4.93	54.09	4.70	-2.61	2.55E-03	3.35E-02
cg20351875	MIR548C;RASSF3	12	TSS1500;Body	Open sea	80.29	3.87	82.90	3.30	-2.61	7.60E-04	1.39E-02
cg21035142	CRYGN	7	Body	Island	26.75	4.19	29.36	3.34	-2.61	9.42E-04	1.64E-02
cg26849637	PPP1R14C	6	Body	Open sea	73.66	3.46	76.27	3.55	-2.61	8.48E-05	2.44E-03
cg10515131	BCL11B;BCL11B	14	Body;Body	N_Shelf	21.24	4.71	23.84	4.70	-2.61	2.51E-03	3.31E-02
cg07525420	EBF3	10	Body	Island	41.17	3.37	43.78	3.81	-2.61	6.10E-04	1.18E-02
cg01215215	11-Sep	4	Body	Open sea	80.85	2.83	83.46	2.65	-2.61	2.89E-05	9.66E-04
cg01321673	IL17REL	22	Body	Island	37.00	3.47	39.61	4.39	-2.61	1.84E-03	2.66E-02
cg09595163	WNT5A	3	Body	Island	32.19	2.94	34.79	2.79	-2.61	1.22E-04	3.29E-03
cg06895946	UBE2I;UBE2I;UBE2I;UBE2I	16	Body;Body;Body;Body	N_Shore	82.59	3.83	85.20	3.01	-2.61	3.65E-04	7.93E-03
cg19190728	ZEB2;ZEB2;ZEB2	2	Body;5'UTR;5'UTR	S_Shore	12.49	2.55	15.09	3.74	-2.61	5.05E-06	2.02E-04
cg20672708	TM9SF4	20	TSS1500	Open sea	54.47	4.87	57.08	4.12	-2.61	2.73E-03	3.51E-02
cg05596552	NAALADL1	11	TSS1500	Open sea	75.42	3.28	78.03	3.58	-2.61	1.86E-05	6.54E-04
cg18209489	MTSS1	8	Body	Open sea	49.82	3.72	52.43	4.18	-2.61	1.66E-03	2.47E-02
cg09025625	SHC1;SHC1;SHC1;SHC1	1	Body;1stExon;Body;1stExon	N_Shelf	35.99	4.04	38.59	3.61	-2.61	1.39E-03	2.18E-02
cg08941873	DTWD2	5	Body	N_Shore	60.86	3.96	63.47	3.62	-2.61	3.64E-03	4.28E-02
cg01960300	PDZK1	1	TSS1500	Open sea	75.66	3.70	78.27	4.07	-2.61	1.38E-03	2.17E-02
cg25039706	BRMS1;BRMS1	11	Body;Body	N_Shelf	75.91	3.43	78.52	3.02	-2.61	1.05E-03	1.78E-02
cg12640469	GPX7	1	TSS1500	N_Shore	49.06	5.44	51.67	5.08	-2.61	1.65E-03	2.47E-02

cg24483502	TRAPPC9;TRAPPC9	8	Body;Body	Open sea	83.90	3.52	86.50	2.53	-2.61	5.17E-04	1.04E-02
cg00474713	ATP2A1;ATP2A1	16	TSS1500;TSS1500	N_Shelf	78.79	3.24	81.39	2.51	-2.61	9.59E-04	1.66E-02
cg06542022		7	Intergenic	Island	24.43	3.51	27.04	3.95	-2.61	4.63E-04	9.55E-03
cg05597624	RNF220	1	5'UTR	S_Shore	15.78	2.91	18.38	3.15	-2.61	1.71E-05	6.08E-04
cg09226319		4	Intergenic	N_Shelf	77.17	4.94	79.78	4.41	-2.60	1.68E-03	2.49E-02
cg19331876	TTC3;TTC3	21	3'UTR;3'UTR	Open sea	94.24	1.46	96.85	0.84	-2.60	8.17E-17	5.51E-15
cg25880980	BAZ2A;RBMS2	12	3'UTR;3'UTR	Open sea	65.60	3.58	68.21	2.96	-2.60	1.53E-03	2.34E-02
cg09486406		2	Intergenic	Island	13.21	2.32	15.81	3.36	-2.60	5.75E-05	1.75E-03
cg01587682	PAX6;PAX6;PAX6	11	Body;Body;Body	Island	20.79	3.45	23.40	3.14	-2.60	2.14E-04	5.21E-03
cg04246763	LOC339524	1	Body	Island	23.96	3.91	26.56	4.32	-2.60	3.90E-04	8.36E-03
cg14344122	DNM1;DNM1	9	Body;Body	Island	17.19	3.91	19.79	4.48	-2.60	3.11E-03	3.84E-02
cg03316000		16	Intergenic	Open sea	77.96	4.53	80.57	3.08	-2.60	8.19E-04	1.47E-02
cg17778556	WDFY1	2	Body	Open sea	53.79	3.65	56.39	4.06	-2.60	4.03E-03	4.59E-02
cg11871064	ANKFN1	17	1stExon	Open sea	75.99	4.67	78.59	3.78	-2.60	1.40E-03	2.19E-02
cg21172011		21	Intergenic	Open sea	39.11	5.73	41.71	6.14	-2.60	3.10E-03	3.83E-02
cg23682609	BCAS1	20	TSS200	Open sea	64.76	3.93	67.36	4.23	-2.60	1.26E-03	2.04E-02
cg14846244		12	Intergenic	N_Shelf	72.86	3.70	75.46	2.54	-2.60	1.93E-03	2.76E-02
cg04081402	TCN2	22	Body	Open sea	79.66	2.92	82.27	3.63	-2.60	1.20E-03	1.96E-02
cg00808175	GNB3	12	TSS1500	Open sea	83.75	3.87	86.35	2.93	-2.60	8.14E-04	1.47E-02
cg18767287	MYLK;MYLK;MYLK;MYLK	3	Body;Body;Body;Body	Open sea	69.00	4.05	71.60	3.23	-2.60	4.14E-03	4.67E-02
cg17476752		19	Intergenic	Open sea	49.53	2.81	52.13	2.91	-2.60	3.37E-04	7.46E-03
cg01080600	AGAP1;AGAP1	2	Body;Body	N_Shore	86.08	4.15	88.68	3.17	-2.60	4.40E-03	4.87E-02
cg13546538	CTNNAL1	9	Body	Open sea	39.22	3.21	41.82	2.84	-2.60	6.53E-04	1.24E-02
cg09274658	PFKL;PFKL	21	Body;Body	N_Shelf	78.04	3.27	80.65	3.70	-2.60	1.22E-03	1.99E-02
cg17675712		2	Intergenic	Open sea	83.78	3.99	86.39	3.40	-2.60	3.63E-03	4.27E-02
cg23926866		1	Intergenic	Open sea	45.06	3.07	47.67	3.18	-2.60	5.27E-04	1.05E-02
cg27441114	VPS18	15	Body	S_Shelf	83.56	3.73	86.16	2.46	-2.60	2.16E-03	2.98E-02
cg14254767	TJP2;TJP2;TJP2;TJP2;TJP2;TJP2	9	TSS200;Body;5'UTR;Body;Body;TSS200	Open sea	58.55	4.08	61.16	3.89	-2.60	1.24E-03	2.01E-02
cg02747151	ATP10A	15	Body	Island	80.46	3.64	83.06	3.52	-2.60	5.85E-04	1.14E-02
cg24060173	CDK18;CDK18;CDK18	1	5'UTR;5'UTR;5'UTR	Open sea	61.48	4.20	64.08	3.96	-2.60	2.87E-03	3.63E-02
cg06868100	PRR15	7	3'UTR	S_Shore	14.60	3.99	17.20	4.04	-2.60	1.84E-03	2.67E-02
cg08573355	TAPBP;TAPBP;TAPBP;ZBTB22;ZBTB22	6	TSS1500;TSS1500;TSS1500;3'UTR;3'UTR	N_Shore	12.87	3.45	15.47	3.67	-2.60	3.18E-04	7.12E-03
cg08443563		2	Intergenic	Open sea	23.93	5.29	26.53	6.00	-2.60	2.48E-03	3.28E-02
cg16300838	TUBGCP2	10	Body	Open sea	82.31	3.64	84.91	3.50	-2.60	4.68E-04	9.62E-03
cg20744362	C22orf34	22	Body	Open sea	74.06	4.99	76.66	4.44	-2.60	4.43E-03	4.89E-02
cg08802910	FAM53A	4	3'UTR	N_Shelf	81.12	4.66	83.72	4.32	-2.60	2.46E-03	3.27E-02
cg01459389	TAC4;TAC4;TAC4;TAC4;TAC4	17	Body;Body;Body;Body;Body	Open sea	73.75	3.94	76.35	2.96	-2.60	1.70E-03	2.52E-02
cg19679112		17	Intergenic	N_Shore	38.37	3.67	40.97	3.76	-2.60	1.27E-03	2.04E-02
cg13378013		10	Intergenic	Open sea	31.36	6.51	33.96	6.28	-2.60	1.65E-03	2.47E-02
cg00053393	MGC45800	4	Body	Island	26.89	3.33	29.49	3.87	-2.60	5.51E-04	1.09E-02
cg06664900		16	Intergenic	N_Shelf	64.40	3.99	67.00	3.55	-2.60	4.50E-03	4.94E-02
cg19682537		1	Intergenic	S_Shelf	52.18	3.99	54.78	4.16	-2.60	2.96E-03	3.71E-02
cg19516515	RUSC1;RUSC1;C1orf104	1	TSS200;TSS200;Body	Island	30.34	4.38	32.94	4.24	-2.60	4.40E-03	4.87E-02
cg02991338	FOXG1	14	TSS1500	N_Shore	15.40	2.73	18.00	3.43	-2.60	1.39E-04	3.67E-03
cg16534399		16	Intergenic	Open sea	55.36	4.12	57.96	4.76	-2.60	1.73E-04	4.41E-03
cg04020999	TCHP;TCHP	12	Body;Body	Open sea	88.42	2.90	91.01	2.17	-2.60	9.67E-05	2.72E-03
cg18220058	FAM136A	2	3'UTR	S_Shelf	84.40	3.87	87.00	4.16	-2.60	7.34E-04	1.36E-02
cg03531810	LY6D	8	Body	Open sea	81.02	3.52	83.62	3.07	-2.60	3.32E-04	7.37E-03
cg05620288	HS6ST1	2	TSS1500	Island	31.45	4.05	34.04	3.96	-2.60	3.87E-04	8.31E-03
cg08713568		1	Intergenic	S_Shelf	31.29	3.40	33.89	4.42	-2.60	2.50E-03	3.30E-02
cg06673956	GPR116;GPR116	6	5'UTR;5'UTR	Open sea	54.85	4.02	57.44	3.81	-2.60	3.64E-03	4.28E-02
cg04831900		1	Intergenic	S_Shelf	84.47	3.66	87.07	2.41	-2.60	8.46E-04	1.51E-02
cg06299833	NKX1-2	10	Body	Island	11.66	2.23	14.26	3.10	-2.60	1.20E-05	4.42E-04

cg03121538	DOC2A	16	Body	Open sea	85.43	3.82	88.03	3.29	-2.60	4.15E-04	8.77E-03
cg17342216		3	Intergenic	Open sea	77.58	4.87	80.18	5.18	-2.60	1.72E-03	2.54E-02
cg20269253		2	Intergenic	N_Shelf	83.44	3.87	86.04	2.75	-2.60	3.40E-04	7.50E-03
cg09057050	CMTM3;CMTM3;CMTM3;CMTM3	16	Body;Body;Body;Body	Island	15.79	3.04	18.39	4.12	-2.60	1.19E-04	3.23E-03
cg00592781		12	Intergenic	Island	16.70	2.76	19.30	3.16	-2.60	3.75E-04	8.10E-03
cg03204140	TNXB	6	Body	N_Shore	78.58	3.61	81.18	3.53	-2.60	6.42E-04	1.23E-02
cg20431181	ORC3L;ORC3L	6	Body;Body	Open sea	85.03	3.64	87.63	3.11	-2.60	2.63E-03	3.42E-02
cg14426174	SERPINB7	18	TSS1500	Open sea	83.54	4.36	86.13	3.59	-2.60	8.97E-04	1.58E-02
cg03484717		2	Intergenic	Open sea	54.98	3.33	57.58	3.71	-2.60	1.55E-03	2.36E-02
cg27533288	VAX1;VAX1	10	Body;Body	Island	12.79	2.96	15.38	3.29	-2.60	2.33E-04	5.58E-03
cg05645505	EPHB3	3	Body	N_Shore	76.79	4.26	79.38	3.24	-2.60	4.53E-03	4.97E-02
cg07348300	ZC3H12A	1	3'UTR	Open sea	59.00	4.23	61.59	4.11	-2.60	3.43E-03	4.11E-02
cg14266348		19	Intergenic	S_Shore	59.73	3.99	62.32	4.01	-2.60	2.44E-03	3.25E-02
cg13941669	NIPAL4	5	TSS200	Island	19.57	3.85	22.17	3.73	-2.60	1.77E-04	4.49E-03
cg10550074	C6orf222	6	TSS200	Open sea	74.21	4.63	76.81	4.80	-2.60	2.75E-03	3.52E-02
cg02312525	MLC1	22	TSS1500	N_Shelf	55.80	3.51	58.40	2.70	-2.60	8.15E-04	1.47E-02
cg07089275	SSTR5;LOC146336;SSTR5	16	3'UTR;TSS1500;1stExon	Island	71.95	5.45	74.54	5.07	-2.60	1.28E-03	2.06E-02
cg07014523	ZFP64	20	Body	Island	12.26	2.65	14.85	3.22	-2.60	5.86E-06	2.31E-04
cg09893871	PRR18	6	1stExon	Island	35.17	5.72	37.77	6.29	-2.60	1.90E-03	2.73E-02
cg04841245	HDAC4	2	Body	Open sea	88.48	3.71	91.07	2.51	-2.60	1.11E-03	1.85E-02
cg15077922	TNIP2;TNIP2	4	Body;Body	Open sea	81.94	3.36	84.53	3.06	-2.59	4.79E-04	9.79E-03
cg13909582	DMRTA2	1	Body	Island	27.73	3.34	30.32	3.52	-2.59	1.20E-03	1.96E-02
cg01597641		7	Intergenic	Island	13.40	2.75	15.99	2.74	-2.59	4.07E-05	1.30E-03
cg00529958	ZIC5	13	TSS200	Island	12.13	2.34	14.73	3.12	-2.59	2.20E-05	7.62E-04
cg19464917	ISL2	15	TSS1500	Island	10.60	2.03	13.20	3.00	-2.59	3.49E-06	1.44E-04
cg18115757		14	Intergenic	N_Shelf	45.43	3.62	48.02	3.50	-2.59	3.28E-04	7.30E-03
cg05869617		6	Intergenic	Open sea	17.12	3.59	19.72	4.60	-2.59	9.21E-04	1.61E-02
cg10451772	PODXL2	3	TSS200	Island	24.49	3.39	27.08	2.80	-2.59	3.09E-05	1.02E-03
cg17315964	FAM193B;FAM193B	5	Body;Body	N_Shore	85.15	3.53	87.75	2.45	-2.59	1.36E-03	2.15E-02
cg14815361		5	Intergenic	N_Shelf	66.69	4.36	69.28	3.86	-2.59	1.81E-03	2.63E-02
cg21182103		9	Intergenic	N_Shelf	64.96	4.38	67.55	3.26	-2.59	1.88E-03	2.70E-02
cg25408698	HRNBP3	17	Body	N_Shore	79.46	4.33	82.05	3.67	-2.59	4.24E-03	4.75E-02
cg19986126	MIR1257	20	TSS1500	Open sea	63.25	3.92	65.85	5.27	-2.59	1.75E-03	2.57E-02
cg26422488	MYO10;MYO10	5	1stExon;5'UTR	Island	14.82	3.85	17.41	4.40	-2.59	1.86E-03	2.69E-02
cg15812753	HOXD3	2	5'UTR	Island	20.69	2.94	23.28	3.06	-2.59	1.18E-05	4.36E-04
cg15009328	ABCG5	2	Body	N_Shore	73.09	3.84	75.68	3.99	-2.59	3.16E-03	3.88E-02
cg23619577	RAVER2	1	3'UTR	Open sea	73.98	4.18	76.58	3.88	-2.59	2.42E-03	3.23E-02
cg22953267	ABCF1;ABCF1	6	Body;Body	S_Shelf	75.15	3.61	77.74	4.12	-2.59	6.27E-04	1.21E-02
cg19541697	ASXL2	2	Body	Open sea	14.44	3.31	17.03	3.89	-2.59	2.48E-03	3.28E-02
cg23540210	CCDC39	3	3'UTR	Open sea	85.97	4.18	88.56	4.32	-2.59	1.01E-03	1.73E-02
cg10022722	SYNGR3	16	Body	Island	19.89	3.34	22.48	3.92	-2.59	1.75E-03	2.57E-02
cg24335981	GIMAP1	7	TSS1500	Open sea	50.20	4.55	52.79	4.75	-2.59	8.54E-04	1.52E-02
cg26833652	MYBPC1;MYBPC1;MYBPC1;MYBPC1	12	Body;Body;Body;Body	Island	15.67	4.05	18.26	4.18	-2.59	3.31E-03	4.01E-02
cg04070976		17	Intergenic	Open sea	67.98	4.28	70.58	4.39	-2.59	7.21E-04	1.34E-02
cg04430582	MIR195;MIR497	17	TSS1500;TSS1500	N_Shelf	78.98	4.05	81.57	3.29	-2.59	1.63E-03	2.44E-02
cg24526596	DLGAP2	8	5'UTR	Open sea	59.07	4.67	61.67	4.46	-2.59	3.46E-03	4.13E-02
cg09275704		16	Intergenic	Island	18.16	3.21	20.75	3.22	-2.59	9.05E-04	1.59E-02
cg21749091	MBTD1;UTP18	17	TSS1500;Body	Island	13.21	2.20	15.80	3.13	-2.59	1.67E-05	5.95E-04
cg26605683	STC2	5	Body	N_Shelf	50.03	4.42	52.62	3.82	-2.59	9.37E-04	1.63E-02
cg01138020	SPNS3	17	TSS200	Open sea	54.16	4.25	56.75	4.38	-2.59	1.24E-03	2.01E-02
cg10986236		3	Intergenic	Open sea	84.37	4.56	86.96	3.46	-2.59	7.59E-04	1.39E-02
cg13403719		15	Intergenic	Open sea	82.31	4.42	84.90	3.49	-2.59	1.78E-03	2.60E-02
cg27491557		6	Intergenic	S_Shelf	75.49	4.31	78.08	3.33	-2.59	4.37E-03	4.84E-02

cg03969725	UBE2C;UBE2C;UBE2C;UBE2C;UBE2C;U	20	5'UTR;1stExon;5'UTR;Body;Body;Body;Bo	S_Shore	43.14	4.97	45.73	4.82	-2.59	1.36E-03	2.15E-02
cg15178540	WNT16;WNT16	7	Body;Body	Island	13.72	3.42	16.31	3.42	-2.59	7.17E-04	1.33E-02
cg14282207	NEDD1;NEDD1;NEDD1;NEDD1	12	Body;Body;Body;Body	Open sea	87.76	3.70	90.35	3.05	-2.59	7.30E-05	2.15E-03
cg10117719	C1orf216	1	3'UTR	N_Shelf	62.26	2.87	64.85	4.10	-2.59	3.21E-05	1.06E-03
cg25354617	HTT	4	3'UTR	Open sea	84.42	2.52	87.01	2.56	-2.59	2.44E-06	1.03E-04
cg00036732	LZTS2	10	3'UTR	S_Shore	71.35	4.41	73.94	3.52	-2.59	3.78E-03	4.39E-02
cg03463778	LOC642826	10	TSS1500	N_Shore	38.06	3.98	40.65	4.20	-2.59	2.08E-03	2.91E-02
cg16297569	GFI1;GFI1	1	TSS1500;TSS200	Island	21.97	4.30	24.56	5.17	-2.59	2.22E-03	3.04E-02
cg01419967	PLEKHA7	11	Body	Open sea	75.48	3.65	78.07	3.75	-2.59	1.19E-03	1.95E-02
cg05467160	EVX2	2	Body	Island	14.97	2.53	17.56	3.80	-2.59	5.98E-04	1.16E-02
cg17616554	CFTR	7	TSS1500	Open sea	12.97	2.94	15.56	3.83	-2.59	1.85E-04	4.64E-03
cg02243386	XIRP1	3	TSS1500	Open sea	64.18	4.07	66.77	4.83	-2.59	1.54E-03	2.35E-02
cg26684679		12	Intergenic	S_Shelf	62.53	4.65	65.12	4.21	-2.59	2.19E-03	3.01E-02
cg01432120		2	Intergenic	Island	51.93	2.79	54.51	3.18	-2.59	1.31E-03	2.09E-02
cg22118465	TNNC1	3	Body	N_Shelf	81.77	3.17	84.36	2.47	-2.59	7.86E-05	2.29E-03
cg21212076	FAM166B;FAM166B	9	TSS200;TSS200	Open sea	68.78	4.63	71.36	3.52	-2.59	1.60E-03	2.41E-02
cg02168159	SKIV2L	6	Body	S_Shelf	82.14	3.43	84.73	2.33	-2.59	2.42E-04	5.75E-03
cg27105390		19	Intergenic	Island	27.82	4.03	30.40	4.83	-2.59	3.97E-03	4.54E-02
cg11997734	COL18A1;COL18A1;COL18A1	21	Body;Body;Body	N_Shore	83.38	3.95	85.97	3.42	-2.59	3.13E-03	3.86E-02
cg25921609	MYH10	17	Body	N_Shore	83.60	2.73	86.19	2.74	-2.59	2.06E-05	7.18E-04
cg02851548		1	Intergenic	Open sea	75.27	4.48	77.86	4.76	-2.59	7.99E-04	1.45E-02
cg00516481	PDE9A;PDE9A;PDE9A;PDE9A;PDE9A;P	21	TSS1500;TSS1500;TSS1500;TSS1500;TSS15	Island	26.71	5.06	29.29	5.02	-2.59	1.28E-03	2.05E-02
cg09095525	ZC3H7A	16	Body	Open sea	75.39	4.91	77.98	4.31	-2.59	1.46E-03	2.26E-02
cg08936411	GRID2	4	Body	Open sea	73.41	4.28	76.00	4.39	-2.59	3.53E-03	4.19E-02
cg07999415	NUMA1	11	Body	Open sea	86.18	3.15	88.76	3.14	-2.59	4.44E-04	9.24E-03
cg17956079	PLEKHB1;PLEKHB1;PLEKHB1;PLEKHB1	11	TSS200;TSS1500;TSS200;Body;Body	Open sea	14.95	3.12	17.54	3.70	-2.59	3.54E-04	7.76E-03
cg07946961	C19orf34;CSNK1G2	19	TSS1500;5'UTR	N_Shore	84.72	3.65	87.31	2.38	-2.59	2.17E-04	5.27E-03
cg19013522	TTYH3	7	Body	N_Shore	83.87	3.51	86.46	2.52	-2.59	1.01E-03	1.72E-02
cg11811705		1	Intergenic	Island	14.15	2.77	16.74	3.27	-2.59	1.66E-05	5.92E-04
cg07406498		5	Intergenic	Open sea	69.42	4.21	72.00	3.47	-2.59	3.01E-03	3.75E-02
cg16686900	C22orf43;C22orf43	22	5'UTR;1stExon	Open sea	56.46	5.06	59.04	4.15	-2.59	9.77E-04	1.68E-02
cg01824410		6	Intergenic	Open sea	10.08	2.87	12.67	4.27	-2.59	1.03E-03	1.75E-02
cg23938220	APC;APC;APC	5	TSS200;TSS200;5'UTR	Open sea	36.07	5.07	38.66	5.98	-2.59	2.21E-03	3.03E-02
cg08742288	FCHSD1	5	TSS200	Island	11.87	4.28	14.46	5.89	-2.59	3.00E-03	3.74E-02
cg21511748		11	Intergenic	N_Shore	60.03	3.21	62.62	3.77	-2.59	1.48E-03	2.28E-02
cg00245057		8	Intergenic	Open sea	80.95	3.89	83.53	4.15	-2.59	1.21E-03	1.97E-02
cg03341413	LBXCOR1	15	Body	N_Shore	21.40	4.18	23.98	4.63	-2.59	3.33E-03	4.03E-02
cg16857801	LOC440040	11	TSS200	Open sea	13.99	2.93	16.58	3.68	-2.59	1.15E-03	1.90E-02
cg27649348		5	Intergenic	N_Shore	22.26	2.73	24.84	3.21	-2.59	8.97E-04	1.58E-02
cg09853702		12	Intergenic	Open sea	76.77	4.50	79.36	3.72	-2.59	3.17E-04	7.12E-03
cg06971353	ADNP2;ADNP2	18	1stExon;5'UTR	Open sea	16.25	3.46	18.84	4.16	-2.59	2.23E-03	3.05E-02
cg17780624	DLX1;DLX1	2	TSS1500;TSS1500	Island	15.12	3.10	17.70	3.04	-2.58	3.23E-05	1.06E-03
cg08602528	GLIS2	16	Body	Open sea	81.81	2.84	84.39	2.38	-2.58	1.28E-04	3.44E-03
cg14315444		17	Intergenic	Island	41.02	4.28	43.60	3.96	-2.58	1.38E-03	2.17E-02
cg22033281		11	Intergenic	Open sea	66.03	4.25	68.61	3.70	-2.58	3.94E-03	4.52E-02
cg05363616	TBX15	1	5'UTR	N_Shore	23.86	3.60	26.44	3.83	-2.58	1.92E-03	2.75E-02
cg11151942		7	Intergenic	S_Shore	70.07	4.70	72.66	3.33	-2.58	2.70E-03	3.48E-02
cg17414733	ZNF429	19	Body	Island	34.46	3.17	37.04	3.04	-2.58	9.87E-04	1.69E-02
cg18864739	CDH6	5	5'UTR	Open sea	80.20	5.50	82.78	4.95	-2.58	2.16E-03	2.98E-02
cg02274263	SNORD115-2	15	Body	Open sea	80.35	3.74	82.93	3.13	-2.58	1.67E-03	2.49E-02
cg18315248	FSTL5;FSTL5;FSTL5	4	Body;Body;Body	Open sea	85.52	4.21	88.10	2.93	-2.58	1.59E-03	2.40E-02
cg23110005	PTHLH;PTHLH;PTHLH;PTHLH	12	TSS1500;5'UTR;5'UTR;TSS1500	S_Shore	32.74	4.29	35.32	4.43	-2.58	2.04E-03	2.86E-02
cg02508328		12	Intergenic	N_Shelf	87.91	4.37	90.50	4.30	-2.58	3.47E-04	7.62E-03

cg08580056	DIAPH3;DIAPH3	13	Body;Body	Open sea	88.37	4.41	90.95	3.22	-2.58	6.78E-05	2.02E-03
cg14789659	ARID5B	10	Body	Open sea	11.56	2.20	14.14	2.57	-2.58	4.59E-06	1.85E-04
cg11205797		10	Intergenic	Open sea	83.64	4.78	86.22	4.14	-2.58	4.09E-03	4.63E-02
cg17786697	PRDM6	5	Body	N_Shore	30.13	3.29	32.72	3.85	-2.58	1.81E-03	2.64E-02
cg19678828	CYYR1;CYYR1	21	5'UTR;1stExon	Island	14.04	2.70	16.62	3.98	-2.58	6.80E-04	1.28E-02
cg17065810	C11orf54	11	Body	Open sea	73.24	2.71	75.82	2.83	-2.58	1.22E-04	3.30E-03
cg07961015		5	Intergenic	Island	15.47	3.06	18.05	3.13	-2.58	7.20E-04	1.34E-02
cg07500806	FMN1	15	Body	Open sea	66.44	4.39	69.02	3.92	-2.58	1.30E-03	2.08E-02
cg21507636		7	Intergenic	Open sea	69.92	3.44	72.51	4.04	-2.58	8.81E-04	1.56E-02
cg00187129	TK2	16	Body	N_Shelf	78.56	4.26	81.14	3.52	-2.58	1.79E-03	2.61E-02
cg16358099	MYOF;MYOF	10	Body;Body	Open sea	23.24	3.50	25.82	3.78	-2.58	2.40E-03	3.21E-02
cg01957150		6	Intergenic	Island	87.54	3.94	90.12	1.92	-2.58	5.39E-04	1.07E-02
cg11306767	GLRX;GLRX	5	Body;Body	Open sea	61.33	4.50	63.91	4.05	-2.58	2.84E-03	3.60E-02
cg26803969		2	Intergenic	Open sea	65.24	3.49	67.82	3.13	-2.58	3.38E-03	4.06E-02
cg06609094		4	Intergenic	N_Shelf	14.35	2.33	16.93	3.32	-2.58	1.14E-05	4.22E-04
cg14146751	GNA14	9	Body	N_Shore	71.51	4.04	74.10	3.38	-2.58	2.42E-03	3.23E-02
cg26390050	ZC3H3	8	Body	N_Shore	73.08	4.61	75.66	3.53	-2.58	3.59E-03	4.24E-02
cg04304255	PCDH9;PCDH9	13	Body;Body	Open sea	83.35	4.46	85.93	4.66	-2.58	1.04E-03	1.76E-02
cg18251191		19	Intergenic	N_Shore	70.00	4.89	72.58	3.89	-2.58	1.21E-03	1.98E-02
cg11131902	HRH2	5	TSS1500	N_Shore	19.71	3.43	22.29	4.38	-2.58	1.12E-03	1.87E-02
cg15388309	L3MBTL;L3MBTL	20	TSS1500;TSS1500	N_Shore	53.30	2.93	55.88	2.62	-2.58	1.61E-04	4.14E-03
cg13013900	XAF1;XAF1;XAF1;XAF1	17	5'UTR;5'UTR;1stExon;1stExon	Open sea	8.12	2.66	10.70	3.77	-2.58	8.39E-04	1.50E-02
cg01046511	KDM6B	17	TSS1500	N_Shelf	83.80	3.00	86.38	2.49	-2.58	1.14E-05	4.22E-04
cg19291926	CDC6	17	TSS1500	N_Shore	20.33	2.86	22.92	3.29	-2.58	3.97E-05	1.27E-03
cg15534561		5	Intergenic	Open sea	11.95	4.24	14.53	3.67	-2.58	1.19E-03	1.95E-02
cg24324942	ZNF10	12	Body	Open sea	72.23	3.28	74.81	3.08	-2.58	1.48E-04	3.88E-03
cg09473180		2	Intergenic	Open sea	90.13	3.45	92.72	2.03	-2.58	7.18E-05	2.12E-03
cg12244275		2	Intergenic	S_Shelf	71.04	6.25	73.62	5.68	-2.58	3.60E-03	4.25E-02
cg25249068	BFSP2	3	1stExon	Open sea	69.43	4.44	72.01	3.98	-2.58	1.53E-03	2.33E-02
cg08833577	VAX1;VAX1	10	TSS1500;TSS1500	Island	14.24	3.34	16.82	3.42	-2.58	3.88E-04	8.32E-03
cg05323725	LHX1;LHX1	17	1stExon;5'UTR	Island	14.44	3.08	17.02	3.13	-2.58	3.06E-05	1.02E-03
cg09003373	MESTIT1;MEST;MEST;MEST	7	Body;TSS1500;TSS200;5'UTR	Island	37.10	3.47	39.68	3.71	-2.58	1.37E-04	3.63E-03
cg17570188	SLC45A2;SLC45A2	5	TSS1500;TSS1500	Open sea	76.87	2.47	79.45	2.29	-2.58	4.79E-06	1.92E-04
cg18516557		2	Intergenic	N_Shore	30.25	3.90	32.83	4.36	-2.58	4.17E-03	4.69E-02
cg19442495	CHST2	3	TSS1500	Island	9.28	2.07	11.86	2.22	-2.58	2.09E-08	1.09E-06
cg18954900	ELK3	12	5'UTR	Open sea	71.53	4.15	74.11	4.10	-2.58	4.06E-03	4.61E-02
cg23003832	ZDHHC12	9	TSS1500	S_Shore	15.86	4.09	18.44	4.20	-2.58	1.66E-04	4.25E-03
cg15328072		2	Intergenic	Open sea	78.03	4.58	80.61	4.87	-2.58	1.31E-03	2.08E-02
cg06179485	TMEM54	1	TSS1500	S_Shore	20.87	4.20	23.45	4.83	-2.58	4.11E-03	4.65E-02
cg12610079	C1orf110	1	TSS1500	Open sea	79.35	3.96	81.93	3.62	-2.58	6.44E-04	1.23E-02
cg02060584	ANK2;ANK2;ANK2	4	TSS200;Body;TSS200	Open sea	32.95	5.08	35.53	6.22	-2.58	1.89E-03	2.72E-02
cg19127724	PANK2;MIR103-2;PANK2;PANK2	20	Body;TSS200;Body;Body	Open sea	83.50	3.88	86.08	3.73	-2.58	1.75E-03	2.57E-02
cg01576496	PPP1R12A;PPP1R12A;PPP1R12A	12	Body;Body;5'UTR	Open sea	87.79	3.21	90.37	2.69	-2.58	5.19E-05	1.60E-03
cg26946288	PLSCR3	17	Body	Island	86.62	3.63	89.20	2.64	-2.58	1.24E-03	2.01E-02
cg00275090		5	Intergenic	N_Shelf	76.69	3.93	79.27	4.30	-2.58	1.83E-03	2.66E-02
cg11504740	GPR152	11	1stExon	Open sea	65.24	3.99	67.82	3.56	-2.58	2.00E-03	2.83E-02
cg03159127	RPL7L1	6	TSS200	Open sea	43.46	3.81	46.04	3.64	-2.58	1.13E-03	1.87E-02
cg26764846	GJB6;GJB6;GJB6;GJB6	13	5'UTR;5'UTR;5'UTR;5'UTR	Open sea	74.28	3.77	76.86	3.10	-2.58	4.26E-03	4.76E-02
cg04130455		4	Intergenic	Island	88.18	4.33	90.76	3.48	-2.58	7.66E-04	1.40E-02
cg20460388	IRAK4;IRAK4;IRAK4;IRAK4;IRAK4	12	3'UTR;3'UTR;3'UTR;3'UTR;3'UTR	Open sea	61.91	4.26	64.49	3.91	-2.58	3.82E-03	4.42E-02
cg16245345	CNOT6	5	5'UTR	S_Shore	32.25	3.91	34.83	4.57	-2.58	2.32E-03	3.14E-02
cg05571448	PPP3CA;PPP3CA;PPP3CA	4	Body;Body;Body	Open sea	81.81	5.40	84.39	5.24	-2.58	8.86E-04	1.56E-02
cg02471153	KCNS2	8	Body	Island	25.83	3.69	28.41	3.51	-2.58	3.00E-03	3.75E-02

cg20684375		8	Intergenic	Open sea	73.44	4.19	76.02	4.16	-2.58	3.91E-04	8.37E-03
cg02144258	ACCN4;ACCN4	2	Body;Body	S_Shelf	48.64	4.02	51.22	4.25	-2.58	1.45E-03	2.25E-02
cg24586444	DCBLD1	6	Body	S_Shelf	69.72	4.47	72.29	3.70	-2.58	3.63E-03	4.27E-02
cg20518572		2	Intergenic	Open sea	15.49	4.20	18.06	4.88	-2.58	2.02E-03	2.85E-02
cg19797376	TAL1	1	TSS200	N_Shore	28.24	3.88	30.82	3.61	-2.58	1.05E-03	1.78E-02
cg07894004	NTSR2	2	TSS1500	S_Shore	81.61	4.20	84.18	4.03	-2.58	7.32E-04	1.36E-02
cg04972513	FAM171A2	17	Body	S_Shore	65.27	4.34	67.85	4.55	-2.58	1.56E-03	2.37E-02
cg08046604	ALDH8A1;ALDH8A1	6	3'UTR;3'UTR	Open sea	83.96	4.03	86.54	3.33	-2.58	9.46E-04	1.64E-02
cg13568659		6	Intergenic	Open sea	81.27	4.48	83.85	4.84	-2.58	1.22E-03	1.99E-02
cg06964353	NADK	1	3'UTR	N_Shelf	83.75	3.94	86.32	3.59	-2.58	1.65E-03	2.47E-02
cg05562851	UNC119B	12	Body	S_Shelf	11.47	2.55	14.05	3.76	-2.58	9.30E-04	1.62E-02
cg12930124	DHH	12	Body	Island	17.00	3.15	19.57	3.19	-2.58	1.26E-03	2.03E-02
cg17685019	FSHB;FSHB	11	TSS1500;TSS1500	Open sea	38.82	3.85	41.40	3.34	-2.58	4.79E-04	9.79E-03
cg18082112	C3orf70	3	Body	N_Shore	15.80	3.29	18.37	3.98	-2.58	2.88E-03	3.64E-02
cg04453371		10	Intergenic	Open sea	55.75	3.75	58.33	3.83	-2.58	1.52E-03	2.32E-02
cg06876354	RALB	2	5'UTR	Open sea	73.82	3.75	76.39	3.24	-2.58	8.77E-04	1.55E-02
cg05349245	C4orf49	4	TSS200	S_Shore	41.92	4.03	44.50	4.62	-2.58	3.75E-03	4.36E-02
cg26469982	RPTOR;RPTOR	17	Body;Body	S_Shore	86.10	3.39	88.68	2.59	-2.58	1.09E-03	1.82E-02
cg22649187		11	Intergenic	Open sea	66.53	3.68	69.11	3.50	-2.58	2.18E-03	3.01E-02
cg01811583		14	Intergenic	Open sea	75.95	3.42	78.53	3.06	-2.58	4.84E-05	1.51E-03
cg02211735	OR52A4	11	1stExon	Open sea	40.02	6.85	42.60	5.04	-2.58	2.92E-03	3.67E-02
cg26941291		5	Intergenic	N_Shore	76.07	3.55	78.65	3.08	-2.58	4.74E-04	9.73E-03
cg25835226		10	Intergenic	Island	31.89	3.74	34.47	3.34	-2.58	4.53E-04	9.39E-03
cg03190911	TAPBP;TAPBP;TAPBP	6	Body;Body;Body	Open sea	80.44	3.08	83.01	2.72	-2.58	6.78E-04	1.28E-02
cg10620429	MEF2A;MEF2A;MEF2A;MEF2A	15	3'UTR;3'UTR;3'UTR;3'UTR	Open sea	85.93	4.47	88.51	4.00	-2.58	3.31E-03	4.01E-02
cg07299819	NPR1	1	TSS200	Island	19.72	2.95	22.29	3.04	-2.57	5.59E-05	1.71E-03
cg12344249	FAM134B;FAM134B	5	Body;Body	Open sea	81.05	4.38	83.62	4.55	-2.57	3.15E-03	3.87E-02
cg23944005	HIST1H3G	6	1stExon	Island	38.22	2.76	40.79	2.83	-2.57	1.40E-04	3.69E-03
cg26234658		7	Intergenic	Open sea	77.21	4.28	79.78	3.55	-2.57	1.99E-03	2.82E-02
cg11952914	RERE;RERE	1	5'UTR;5'UTR	Open sea	86.02	4.84	88.60	5.35	-2.57	3.02E-03	3.76E-02
cg16844941		8	Intergenic	Island	17.22	3.04	19.79	3.42	-2.57	1.34E-04	3.56E-03
cg11588584	CDK11B;CDK11B;CDK11B;CDK11B;CDI	1	Body;Body;Body;Body;Body;5'UTR;5'UTR	S_Shelf	84.57	4.01	87.14	2.98	-2.57	1.83E-03	2.66E-02
cg27188358	PPAP2C;PPAP2C;PPAP2C	19	3'UTR;3'UTR;3'UTR	Open sea	80.25	3.88	82.82	3.50	-2.57	1.85E-03	2.68E-02
cg02825977	CYP26C1	10	Body	Island	10.81	2.46	13.39	2.94	-2.57	4.30E-05	1.36E-03
cg10282807	ACRBP;ACRBP	12	1stExon;5'UTR	Island	23.89	4.56	26.46	4.22	-2.57	4.55E-03	4.98E-02
cg16636767	FAR1	11	5'UTR	S_Shelf	79.52	4.58	82.10	3.55	-2.57	2.80E-03	3.57E-02
cg09387382		17	Intergenic	S_Shore	41.44	4.93	44.01	5.03	-2.57	2.64E-03	3.43E-02
cg22216017	SGCE;SGCE;PEG10;SGCE;PEG10	7	Body;Body;TSS1500;Body;TSS1500	Island	12.07	2.49	14.64	2.41	-2.57	3.40E-06	1.40E-04
cg21797131	ARVCF	22	Body	N_Shelf	83.23	3.29	85.80	2.55	-2.57	2.34E-04	5.60E-03
cg09274104	ACOT6	14	TSS1500	S_Shelf	66.95	2.44	69.52	2.85	-2.57	7.88E-05	2.29E-03
cg11935248	CDYL;CDYL;CDYL;CDYL	6	Body;Body;Body;Body	Open sea	19.62	7.31	22.19	7.86	-2.57	1.49E-03	2.30E-02
cg12485386	PPP1R12C	19	Body	N_Shore	80.09	3.16	82.66	2.57	-2.57	4.01E-05	1.28E-03
cg05800983	GRM4	6	TSS1500	Open sea	79.76	4.90	82.34	4.64	-2.57	2.09E-03	2.91E-02
cg24917065	SLC25A37	8	Body	Open sea	74.62	4.04	77.19	3.64	-2.57	4.16E-03	4.68E-02
cg14531782	PDE2A;PDE2A	11	1stExon;5'UTR	Open sea	16.42	3.10	18.99	3.42	-2.57	2.55E-05	8.65E-04
cg20340508	OLIG1	21	TSS200	Island	10.72	3.03	13.29	3.72	-2.57	4.64E-05	1.46E-03
cg03995361	VIPR1	3	Body	N_Shore	81.00	3.23	83.58	2.24	-2.57	3.05E-04	6.88E-03
cg25703864	SMOC2;SMOC2	6	Body;Body	S_Shore	71.00	3.49	73.57	3.54	-2.57	2.61E-03	3.40E-02
cg05991454		4	Intergenic	Island	20.04	3.34	22.61	2.76	-2.57	2.53E-05	8.60E-04
cg18146843	FAM159A	1	1stExon	Island	22.28	3.12	24.85	4.67	-2.57	4.55E-03	4.98E-02
cg03325664		8	Intergenic	N_Shelf	50.65	5.22	53.22	5.06	-2.57	3.39E-03	4.08E-02
cg04852949	COL16A1	1	TSS1500	S_Shore	19.47	3.48	22.04	4.57	-2.57	3.98E-03	4.55E-02
cg27514777		16	Intergenic	N_Shore	73.57	4.18	76.14	4.35	-2.57	1.61E-03	2.42E-02

cg25072666	NPLOC4	17	Body	Open sea	9.73	2.63	12.30	3.19	-2.57	9.93E-04	1.70E-02
cg15880704	RBM20	10	Body	Open sea	59.64	3.50	62.21	3.06	-2.57	6.02E-04	1.17E-02
cg18141744	ANO1	11	TSS1500	N_Shore	14.96	2.66	17.53	3.86	-2.57	1.88E-04	4.70E-03
cg25651783	PKLR;PKLR	1	Body;Body	Island	35.66	4.34	38.23	4.77	-2.57	4.59E-04	9.49E-03
cg21966764	OR52B2	11	TSS1500	Open sea	75.58	3.85	78.15	3.63	-2.57	4.07E-05	1.30E-03
cg17882033	AKD1;AKD1	6	Body;Body	Open sea	54.20	3.28	56.77	2.99	-2.57	2.01E-04	4.95E-03
cg06320418	METRN	16	Body	N_Shore	77.09	3.30	79.66	2.50	-2.57	8.87E-05	2.53E-03
cg23760687		13	Intergenic	S_Shore	14.28	2.76	16.85	2.78	-2.57	5.01E-05	1.56E-03
cg04415297		1	Intergenic	Open sea	70.53	3.75	73.10	4.19	-2.57	1.40E-04	3.70E-03
cg13508904	SPTAN1;SPTAN1	9	5'UTR;5'UTR	S_Shore	32.47	3.94	35.04	5.06	-2.57	4.56E-03	4.99E-02
cg24921808	PALM;PALM	19	Body;Body	Island	33.96	2.78	36.53	3.54	-2.57	1.08E-03	1.81E-02
cg00199759		8	Intergenic	N_Shore	54.17	4.76	56.74	2.56	-2.57	4.27E-03	4.77E-02
cg13728103	CYB5D1	17	3'UTR	S_Shelf	88.11	2.99	90.68	1.86	-2.57	5.95E-05	1.81E-03
cg19457763	ASAM	11	TSS1500	Island	25.46	3.47	28.03	3.09	-2.57	2.01E-04	4.95E-03
cg22214800		11	Intergenic	Open sea	68.29	4.90	70.86	4.56	-2.57	3.48E-03	4.15E-02
cg23627083	GORASP2	2	Body	S_Shore	75.12	4.43	77.69	4.07	-2.57	3.59E-03	4.24E-02
cg06602009	ZNF587	19	TSS1500	N_Shore	83.58	4.00	86.15	3.89	-2.57	2.58E-03	3.38E-02
cg23983315	MLXIPL;MLXIPL;MLXIPL;MLXIPL	7	Body;Body;Body;Body	Island	48.82	3.24	51.39	3.36	-2.57	1.94E-03	2.77E-02
cg04607412	PRR5;PRR5;PRR5;PRR5	22	5'UTR;5'UTR;5'UTR;5'UTR	Open sea	20.40	3.83	22.96	3.39	-2.57	6.30E-04	1.21E-02
cg27272363		8	Intergenic	Open sea	56.97	4.95	59.54	5.18	-2.57	6.68E-04	1.27E-02
cg17892629	TMEM168	7	Body	Open sea	74.84	5.04	77.40	5.33	-2.57	2.06E-03	2.89E-02
cg05696877	IFI44L	1	5'UTR	Open sea	81.17	3.85	83.73	3.42	-2.57	1.04E-03	1.76E-02
cg19548862	FAM71D	14	3'UTR	Open sea	81.72	3.61	84.28	3.50	-2.57	2.89E-03	3.65E-02
cg20138067	BTG2	1	TSS1500	N_Shore	73.65	3.83	76.21	3.68	-2.57	1.95E-03	2.78E-02
cg10580056	ACTC1	15	Body	Open sea	21.99	3.66	24.56	3.72	-2.57	2.27E-03	3.09E-02
cg01422881	RNF220	1	5'UTR	Island	30.14	2.74	32.71	3.23	-2.57	1.41E-04	3.72E-03
cg08880369	IFT140;TMEM204	16	Body;Body	S_Shore	73.73	4.19	76.29	4.54	-2.57	4.49E-03	4.94E-02
cg26996201	PAK1;PAK1	11	5'UTR;5'UTR	Island	48.97	2.83	51.54	3.20	-2.57	1.10E-04	3.03E-03
cg21243631	NPC1	18	Body	Open sea	34.87	7.79	37.44	6.44	-2.57	1.56E-03	2.37E-02
cg27291539		1	Intergenic	Open sea	84.25	3.47	86.82	2.73	-2.57	3.13E-04	7.04E-03
cg20073553	NKX3-2	4	Body	Island	18.14	3.97	20.70	4.22	-2.57	9.92E-04	1.70E-02
cg24399529	MIR548H4;TMEM84	15	Body;TSS200	Open sea	61.02	5.02	63.59	4.58	-2.57	5.35E-04	1.07E-02
cg23046934	NFATC1;NFATC1;NFATC1	18	TSS1500;TSS1500;TSS1500	Island	21.89	4.20	24.46	5.21	-2.57	2.06E-03	2.89E-02
cg05372311		11	Intergenic	Open sea	60.31	2.85	62.87	2.79	-2.57	2.66E-04	6.19E-03
cg07003360	GDAP1L1	20	TSS200	N_Shore	12.42	2.65	14.99	3.45	-2.57	2.21E-04	5.36E-03
cg15890469	PGF	14	3'UTR	Open sea	70.10	3.71	72.66	3.80	-2.56	2.16E-04	5.25E-03
cg09512236	KCNMA1;KCNMA1;KCNMA1;KCNMA1	10	TSS1500;TSS1500;TSS1500;TSS1500	Island	22.43	3.65	25.00	3.95	-2.56	3.54E-04	7.74E-03
cg17782317		6	Intergenic	S_Shelf	84.44	4.50	87.01	3.75	-2.56	1.20E-03	1.97E-02
cg08009711	C2CD2;C2CD2	21	Body;5'UTR	Open sea	25.39	3.92	27.96	4.00	-2.56	3.67E-03	4.30E-02
cg10938899	GTF2F2	13	Body	Open sea	87.21	4.79	89.78	4.02	-2.56	1.76E-03	2.59E-02
cg08903181		12	Intergenic	Open sea	78.13	4.25	80.69	2.94	-2.56	9.83E-04	1.69E-02
cg24166864	WIF1	12	TSS200	Island	14.30	3.30	16.86	3.85	-2.56	3.19E-04	7.15E-03
cg07789132		1	Intergenic	Island	15.99	2.68	18.55	2.45	-2.56	3.18E-05	1.05E-03
cg03746482		10	Intergenic	Island	27.64	2.61	30.21	2.53	-2.56	9.61E-05	2.71E-03
cg26839652		2	Intergenic	Open sea	77.64	5.18	80.20	5.14	-2.56	2.54E-03	3.34E-02
cg02525597	CIITA	16	Body	S_Shelf	78.04	4.30	80.60	4.59	-2.56	3.77E-03	4.38E-02
cg09326204	CASR	3	5'UTR	S_Shelf	24.45	3.79	27.02	3.44	-2.56	8.60E-04	1.53E-02
cg06117072	TFAP2B	6	Body	Island	19.04	3.26	21.60	4.00	-2.56	4.02E-03	4.58E-02
cg16930880		2	Intergenic	Open sea	70.23	4.36	72.79	3.77	-2.56	2.11E-03	2.94E-02
cg16906086		7	Intergenic	N_Shore	8.63	1.75	11.19	3.28	-2.56	6.03E-07	2.79E-05
cg04853906	MT3	16	TSS1500	N_Shore	25.17	4.03	27.73	4.52	-2.56	1.36E-03	2.15E-02
cg04205655	CTRL;CTRL	16	5'UTR;1stExon	N_Shelf	76.08	3.43	78.65	3.08	-2.56	1.63E-03	2.44E-02
cg24065504	ANKRD22	10	TSS1500	Open sea	80.82	4.30	83.38	3.47	-2.56	8.30E-04	1.49E-02

cg23614835	EPYC	12	Body	Open sea	81.97	4.95	84.54	4.95	-2.56	3.48E-03	4.15E-02
cg14446166		7	Intergenic	N_Shelf	55.99	3.74	58.55	3.65	-2.56	1.13E-03	1.87E-02
cg25207147	PDE11A	2	TSS200	N_Shelf	85.61	4.06	88.17	2.81	-2.56	3.41E-03	4.09E-02
cg11747279		17	Intergenic	Open sea	35.24	4.37	37.81	3.80	-2.56	2.76E-03	3.54E-02
cg09686800	IPMK	10	Body	Open sea	67.15	3.42	69.71	2.25	-2.56	2.33E-04	5.58E-03
cg19401340	PPM1E	17	TSS200	Island	11.23	3.06	13.80	3.91	-2.56	9.59E-04	1.66E-02
cg26009486	NFATC4;NFATC4	14	Body;Body	Island	20.28	4.89	22.84	5.14	-2.56	3.42E-03	4.10E-02
cg11545457		4	Intergenic	N_Shelf	68.46	3.52	71.02	4.03	-2.56	3.39E-03	4.08E-02
cg02900740	TSSC4	11	3'UTR	S_Shelf	83.27	3.81	85.83	3.74	-2.56	2.61E-03	3.40E-02
cg01109017	DENND1A	9	Body	N_Shore	67.17	3.90	69.74	4.25	-2.56	2.15E-03	2.97E-02
cg20491785	BRF1;BRF1	14	Body;5'UTR	Open sea	85.12	3.59	87.68	2.83	-2.56	2.87E-03	3.63E-02
cg18959422	MYBPH	1	TSS1500	Open sea	73.72	4.68	76.28	5.62	-2.56	3.55E-04	7.76E-03
cg20384613	CDC14A;CDC14A;CDC14A	1	3'UTR;Body;Body	Open sea	89.40	3.43	91.97	2.08	-2.56	4.90E-05	1.53E-03
cg08246316	SRCAP	16	3'UTR	Open sea	64.66	3.89	67.22	4.02	-2.56	2.53E-03	3.33E-02
cg13209762		1	Intergenic	Open sea	53.92	4.69	56.48	4.16	-2.56	2.52E-03	3.32E-02
cg02647866		6	Intergenic	S_Shore	80.13	3.99	82.69	3.73	-2.56	3.88E-04	8.32E-03
cg19787727		14	Intergenic	N_Shore	14.33	2.73	16.89	3.33	-2.56	1.80E-04	4.54E-03
cg06551822	MVP;MVP	16	Body;Body	S_Shelf	72.75	4.67	75.32	3.55	-2.56	3.82E-03	4.43E-02
cg02320501	SSH2	17	Body	Island	11.67	4.27	14.23	6.00	-2.56	4.26E-03	4.76E-02
cg22260625	PLXNB1;PLXNB1	3	3'UTR;3'UTR	Open sea	60.42	3.78	62.98	3.46	-2.56	1.12E-03	1.87E-02
cg26778584		3	Intergenic	S_Shelf	86.24	4.00	88.80	3.12	-2.56	1.16E-03	1.91E-02
cg09926380	LOC401052;LOC401052	3	5'UTR;1stExon	Island	43.67	2.38	46.23	2.74	-2.56	2.30E-04	5.53E-03
cg26891410	TAL1	1	TSS1500	N_Shore	24.35	3.80	26.91	4.11	-2.56	1.68E-03	2.50E-02
cg23246821	TM4SF1	3	TSS200	Open sea	12.15	4.12	14.71	6.52	-2.56	1.74E-03	2.56E-02
cg16688483	GRHL2	8	Body	Island	22.12	3.83	24.68	3.98	-2.56	3.09E-03	3.83E-02
cg12325455	TRIOBP	22	TSS200	Open sea	47.64	4.39	50.20	4.86	-2.56	1.31E-03	2.09E-02
cg17635114	NAP1L5;HERC3	4	1stExon;Body	Open sea	41.23	3.12	43.79	3.00	-2.56	1.12E-03	1.87E-02
cg20023259	PITX3	10	5'UTR	Island	21.68	3.36	24.24	4.94	-2.56	1.31E-04	3.50E-03
cg09357740		14	Intergenic	Open sea	23.44	5.41	26.00	5.04	-2.56	2.47E-03	3.27E-02
cg01258488	PTPRR;PTPRR	12	Body;Body	Open sea	87.02	2.73	89.58	2.39	-2.56	2.64E-05	8.93E-04
cg05746227		5	Intergenic	Island	14.23	2.06	16.79	3.15	-2.56	2.18E-05	7.56E-04
cg23165310	SOX2OT	3	Body	Open sea	20.13	3.22	22.69	3.19	-2.56	2.56E-04	6.01E-03
cg14976391	MCAM	11	Body	Island	56.86	3.03	59.42	3.40	-2.56	8.93E-04	1.57E-02
cg06641153	ST8SIA3	18	Body	S_Shore	20.06	3.19	22.61	4.01	-2.56	7.02E-04	1.31E-02
cg25780671	OTP	5	Body	Island	19.14	3.46	21.70	3.53	-2.56	1.54E-03	2.35E-02
cg16094155		13	Intergenic	N_Shelf	81.12	2.33	83.68	2.30	-2.56	1.16E-05	4.31E-04
cg17753212	XAF1;XAF1	17	Body;Body	Open sea	7.95	2.86	10.51	4.21	-2.56	3.67E-03	4.30E-02
cg26200378	KIAA0652;KIAA0652;KIAA0652;KIAA0(	11	Body;Body;Body;5'UTR;5'UTR	S_Shelf	77.11	4.17	79.66	3.21	-2.56	2.39E-03	3.20E-02
cg24494707	SKAP2	7	TSS1500	S_Shore	81.10	3.49	83.66	2.74	-2.56	2.29E-04	5.51E-03
cg24283836	COL4A2	13	Body	Open sea	75.15	3.64	77.71	2.78	-2.56	2.00E-03	2.83E-02
cg12461141	TRIM22	11	TSS1500	Open sea	66.80	5.28	69.36	5.75	-2.56	4.15E-04	8.77E-03
cg20031632	PRR4;TAS2R46;PRH1	12	5'UTR;TSS200;5'UTR	Open sea	87.19	4.87	89.75	3.92	-2.56	3.36E-03	4.05E-02
cg23924241		5	Intergenic	Open sea	81.34	4.04	83.89	3.68	-2.56	1.46E-03	2.26E-02
cg23924737	MRPS23	17	TSS1500	S_Shore	74.71	5.74	77.27	6.00	-2.56	1.29E-03	2.06E-02
cg14832317		4	Intergenic	Island	23.23	2.58	25.79	3.35	-2.56	9.12E-05	2.59E-03
cg20640261	MSH5;MSH5;MSH5;MSH5	6	TSS1500;TSS1500;TSS1500;TSS1500	N_Shore	33.02	3.77	35.58	4.08	-2.56	2.10E-03	2.93E-02
cg21593030		11	Intergenic	Open sea	19.71	4.44	22.26	5.25	-2.56	2.00E-03	2.83E-02
cg00061860		15	Intergenic	Open sea	81.61	4.32	84.16	3.50	-2.56	3.02E-04	6.85E-03
cg05127995		4	Intergenic	S_Shore	73.65	3.25	76.20	2.63	-2.56	1.95E-04	4.84E-03
cg15784521	LRRC37A3	17	Body	Open sea	71.36	3.26	73.91	4.27	-2.56	7.23E-04	1.34E-02
cg12152002	OTX2OS1	14	TSS1500	S_Shore	13.65	2.60	16.21	3.48	-2.56	2.97E-04	6.75E-03
cg08607108	MAD1L1;MAD1L1;MAD1L1	7	Body;Body;Body	S_Shelf	77.99	3.69	80.55	3.99	-2.56	6.19E-04	1.19E-02
cg07888957	TBCD	17	Body	Open sea	55.46	4.06	58.02	3.30	-2.56	1.07E-03	1.80E-02

cg22940954	RUFY4	2	Body	Open sea	59.77	4.17	62.33	3.38	-2.56	1.87E-03	2.70E-02
cg26757711	RFX2;RFX2	19	Body;Body	N_Shelf	31.52	3.35	34.08	4.38	-2.56	4.09E-03	4.63E-02
cg14112569	ITGB5	3	Body	N_Shore	76.16	5.10	78.72	4.49	-2.56	2.76E-03	3.54E-02
cg10038618	TP73;WDR8	1	TSS1500;TSS1500	Island	24.94	3.61	27.50	3.77	-2.56	6.30E-04	1.21E-02
cg22647566	FAM160B1;FAM160B1	10	Body;Body	Open sea	87.13	4.60	89.69	3.51	-2.56	1.66E-03	2.47E-02
cg18872418		11	Intergenic	S_Shore	86.48	3.93	89.04	2.79	-2.55	2.48E-04	5.88E-03
cg13324311		7	Intergenic	Island	86.89	3.41	89.44	2.09	-2.55	1.40E-06	6.13E-05
cg00016968	RHOC;RHOC;RHOC	1	TSS1500;TSS1500;TSS1500	S_Shore	71.66	3.84	74.21	3.23	-2.55	1.45E-03	2.25E-02
cg18583702		2	Intergenic	S_Shore	67.13	4.27	69.68	3.72	-2.55	3.09E-03	3.82E-02
cg12560320	LOC645851;FLJ25006	17	Body;3'UTR	Open sea	86.87	3.18	89.42	2.49	-2.55	2.60E-04	6.08E-03
cg19507206		10	Intergenic	Island	10.44	2.90	13.00	4.28	-2.55	8.21E-04	1.48E-02
cg11420883	PAPOLB;RADIL;PAPOLB	7	5'UTR;Body;1stExon	Island	22.26	3.42	24.81	3.44	-2.55	3.44E-03	4.12E-02
cg03173974	TMEM14C;TMEM14C	6	TSS1500;TSS1500	N_Shore	87.48	3.84	90.03	2.66	-2.55	1.24E-04	3.35E-03
cg05901447	SLC34A1;SLC34A1	5	TSS1500;TSS1500	Open sea	65.25	4.62	67.81	3.54	-2.55	3.20E-03	3.91E-02
cg14037463	TNXB	6	Body	Open sea	76.64	5.19	79.19	3.94	-2.55	2.44E-04	5.78E-03
cg04532707		8	Intergenic	N_Shore	81.75	4.17	84.30	3.58	-2.55	2.18E-03	3.01E-02
cg16923485	SLCO1A2;SLCO1A2	12	Body;Body	Open sea	69.08	5.09	71.64	5.50	-2.55	9.32E-04	1.63E-02
cg27248887	EEF1B2;EEF1B2;SNORD51;NDUFS1;EE	2	Body;Body;TSS1500;TSS1500;Body	S_Shore	31.80	3.80	34.35	4.15	-2.55	2.77E-03	3.54E-02
cg24751773		2	Intergenic	Open sea	17.90	4.06	20.45	3.41	-2.55	2.66E-03	3.44E-02
cg17893108	C7orf50;C7orf50;C7orf50	7	Body;Body;Body	N_Shore	58.05	4.96	60.60	3.88	-2.55	4.47E-03	4.92E-02
cg12708109		1	Intergenic	Open sea	65.20	5.12	67.75	5.21	-2.55	3.68E-03	4.31E-02
cg23021638	PPP2R3A;PPP2R3A	3	Body;Body	Open sea	84.22	3.73	86.78	4.19	-2.55	5.39E-04	1.07E-02
cg18780257	FGF12;FGF12;FGF12	3	5'UTR;1stExon;Body	Island	10.53	2.20	13.08	4.07	-2.55	8.54E-05	2.45E-03
cg05475934	APBB1IP;APBB1IP	10	1stExon;5'UTR	Island	6.25	1.76	8.80	2.89	-2.55	3.03E-06	1.26E-04
cg22826239	IGF2BP3	7	TSS200	S_Shore	17.06	3.32	19.61	3.92	-2.55	1.43E-03	2.23E-02
cg07984358	KIAA1217;KIAA1217;KIAA1217	10	Body;Body;Body	Open sea	52.78	4.35	55.33	4.79	-2.55	3.56E-03	4.22E-02
cg12261545		6	Intergenic	Open sea	85.80	3.91	88.35	3.37	-2.55	2.46E-03	3.27E-02
cg08795320		16	Intergenic	Island	43.86	4.36	46.42	3.86	-2.55	4.27E-03	4.77E-02
cg05394456	MPG;MPG;MPG	16	5'UTR;Body;5'UTR	S_Shore	32.21	3.81	34.76	4.25	-2.55	3.44E-03	4.11E-02
cg00044796		1	Intergenic	Island	44.44	5.28	46.99	6.37	-2.55	3.91E-03	4.49E-02
cg23493751		3	Intergenic	Open sea	54.18	2.51	56.73	2.50	-2.55	2.06E-05	7.16E-04
cg11011512	STX2;STX2	12	Body;Body	Island	52.85	4.62	55.40	4.93	-2.55	3.04E-04	6.88E-03
cg00981350	MVD;CYBA	16	3'UTR;TSS1500	S_Shore	79.43	3.41	81.98	3.65	-2.55	5.99E-04	1.17E-02
cg19221620		2	Intergenic	N_Shore	14.47	2.26	17.02	3.27	-2.55	4.19E-05	1.33E-03
cg23660064	SUSD4	1	3'UTR	Open sea	80.96	3.49	83.51	3.54	-2.55	1.84E-03	2.66E-02
cg01983682		2	Intergenic	Island	32.06	3.17	34.61	4.28	-2.55	3.00E-03	3.75E-02
cg02152475	PAX5	9	Body	N_Shore	22.35	3.47	24.90	4.08	-2.55	1.27E-03	2.05E-02
cg16852440		2	Intergenic	Open sea	45.88	3.44	48.43	3.97	-2.55	1.76E-03	2.59E-02
cg07586345	SGTA	19	5'UTR	Island	88.12	3.42	90.67	2.49	-2.55	1.01E-04	2.83E-03
cg10982364		2	Intergenic	Island	14.80	2.86	17.35	3.78	-2.55	1.52E-04	3.95E-03
cg02336104	FOXP1;FOXP1	3	5'UTR;5'UTR	Open sea	70.34	3.70	72.89	3.02	-2.55	3.85E-03	4.45E-02
cg04371126	MMP15	16	Body	Island	80.10	4.09	82.65	3.98	-2.55	8.14E-04	1.47E-02
cg03870234	FBRSL1	12	Body	Open sea	82.29	4.53	84.84	7.67	-2.55	3.16E-03	3.88E-02
cg13570861		9	Intergenic	N_Shore	67.85	2.66	70.40	2.79	-2.55	4.82E-05	1.51E-03
cg19657351	GCNT2	6	TSS200	Open sea	17.02	4.11	19.57	3.71	-2.55	1.66E-03	2.47E-02
cg00721530		15	Intergenic	N_Shore	26.54	2.92	29.09	2.87	-2.55	5.19E-05	1.60E-03
cg25287153	NEXN	1	3'UTR	Open sea	79.71	5.23	82.26	4.22	-2.55	1.15E-03	1.90E-02
cg02072484	SEMA5A	5	5'UTR	Open sea	79.88	4.04	82.43	3.18	-2.55	1.39E-03	2.18E-02
cg23522981	ANO7	2	Body	N_Shore	72.82	3.92	75.37	4.28	-2.55	4.30E-04	9.02E-03
cg01049933		8	Intergenic	N_Shore	63.27	4.32	65.81	3.31	-2.55	2.05E-03	2.87E-02
cg20550458	SNORA52;RPLP2	11	Body;Body	S_Shore	52.59	3.75	55.14	3.83	-2.55	1.82E-03	2.65E-02
cg19565262	NAT8	2	TSS1500	Open sea	11.29	2.50	13.83	3.59	-2.55	1.31E-03	2.09E-02
cg25428553		2	Intergenic	Island	13.95	3.70	16.50	3.77	-2.55	3.47E-03	4.14E-02

cg16082644		11	Intergenic	N_Shelf	16.27	3.95	18.82	3.93	-2.55	3.82E-03	4.42E-02
cg03043928		11	Intergenic	Open sea	54.52	3.08	57.07	3.28	-2.55	6.11E-04	1.18E-02
cg05962055	TBC1D9	4	Body	Open sea	57.75	6.22	60.30	6.46	-2.55	3.83E-03	4.43E-02
cg13540795	ZNF432	19	5'UTR	N_Shore	6.35	2.81	8.90	3.77	-2.55	2.72E-04	6.30E-03
cg14365070		17	Intergenic	Island	31.52	2.92	34.07	3.12	-2.55	2.61E-04	6.11E-03
cg10011855		1	Intergenic	S_Shore	40.63	3.36	43.18	3.98	-2.55	4.34E-03	4.83E-02
cg14654468	SLC16A3;SLC16A3;SLC16A3	17	TSS1500;5'UTR;1stExon	Island	44.74	3.89	47.29	5.05	-2.55	3.49E-05	1.14E-03
cg14559356		10	Intergenic	Island	12.00	2.63	14.54	3.70	-2.55	7.12E-04	1.33E-02
cg06492076		2	Intergenic	Open sea	85.76	3.85	88.31	2.35	-2.55	6.20E-04	1.20E-02
cg27495728		7	Intergenic	N_Shelf	13.34	3.20	15.88	3.83	-2.55	1.09E-03	1.83E-02
cg03833604	HOXC10	12	TSS1500	N_Shore	16.07	2.84	18.62	3.46	-2.55	2.26E-04	5.45E-03
cg26233331	S100P;S100P	4	1stExon;5'UTR	Open sea	72.57	4.04	75.11	3.24	-2.55	4.10E-03	4.64E-02
cg08534653	PXDN	2	Body	Island	15.05	2.59	17.60	2.61	-2.55	4.28E-05	1.36E-03
cg01540165	C11orf36;MRGPRG	11	TSS200;1stExon	Island	65.81	5.12	68.36	4.08	-2.55	4.02E-03	4.58E-02
cg07434518	CLDN11	3	TSS1500	Island	14.78	2.95	17.33	4.12	-2.55	1.14E-03	1.89E-02
cg16642791	IRX5	16	Body	Island	18.31	3.50	20.85	3.19	-2.55	6.01E-04	1.17E-02
cg14621217	B3GNTL1	17	Body	Open sea	47.21	5.36	49.76	7.00	-2.55	2.67E-03	3.46E-02
cg05655022	C1orf127;C1orf127	1	TSS1500;Body	Open sea	76.99	3.85	79.54	3.27	-2.55	2.33E-03	3.14E-02
cg26558689	ARMC2	6	Body	Open sea	79.39	2.93	81.93	2.39	-2.55	7.37E-05	2.16E-03
cg06183244		6	Intergenic	Open sea	82.74	4.13	85.29	4.49	-2.55	1.95E-04	4.85E-03
cg22827063		2	Intergenic	Island	14.51	2.89	17.05	3.10	-2.55	1.35E-04	3.59E-03
cg11361344		2	Intergenic	Island	25.98	4.33	28.52	3.67	-2.55	2.83E-03	3.60E-02
cg07593839	NRN1	6	TSS200	Island	19.91	2.82	22.46	3.78	-2.55	1.44E-04	3.77E-03
cg21763793	FNDC3A;FNDC3A	13	Body;Body	Open sea	85.46	4.45	88.00	3.43	-2.55	9.14E-04	1.60E-02
cg12668551		8	Intergenic	Open sea	38.14	5.22	40.69	5.21	-2.55	1.53E-03	2.34E-02
cg05705605		1	Intergenic	Open sea	82.29	3.63	84.83	3.05	-2.55	3.99E-04	8.50E-03
cg27173148	LOC100130872;LOC100130872-SPON2	4	TSS1500;TSS1500	Island	85.79	3.99	88.34	3.70	-2.55	2.60E-04	6.08E-03
cg15391425	PRDM16;PRDM16	1	Body;Body	S_Shore	85.05	3.48	87.60	2.83	-2.55	7.37E-06	2.85E-04
cg18640444		4	Intergenic	Open sea	65.72	4.89	68.26	3.99	-2.55	1.17E-03	1.93E-02
cg18643093	ANXA6;ANXA6	5	Body;Body	Open sea	53.53	5.61	56.07	7.20	-2.54	3.42E-03	4.10E-02
cg21707089		8	Intergenic	N_Shelf	48.03	3.49	50.57	3.85	-2.54	3.38E-04	7.47E-03
cg09036612	ST8SIA3	18	TSS200	N_Shore	8.82	1.88	11.37	3.56	-2.54	1.03E-06	4.62E-05
cg05527530	LHX9	1	Body	S_Shore	17.49	2.91	20.03	3.23	-2.54	5.48E-04	1.09E-02
cg13780516	GGTA1	9	TSS1500	Island	24.68	4.80	27.22	5.27	-2.54	3.40E-03	4.08E-02
cg14505667	DNM1;DNM1	9	Body;Body	S_Shore	68.98	3.65	71.52	2.97	-2.54	3.35E-04	7.42E-03
cg02333730		17	Intergenic	Open sea	72.60	3.95	75.15	4.04	-2.54	2.92E-03	3.68E-02
cg20076301		8	Intergenic	N_Shore	78.56	3.67	81.10	3.57	-2.54	1.25E-03	2.02E-02
cg15540723		17	Intergenic	Island	51.12	3.81	53.67	3.74	-2.54	7.15E-04	1.33E-02
cg18473196	MAMSTR	19	5'UTR	N_Shore	71.64	2.73	74.19	2.56	-2.54	1.48E-04	3.86E-03
cg08140114	HECA	6	Body	Open sea	66.02	4.18	68.57	4.47	-2.54	1.41E-03	2.20E-02
cg09683623	LOC201651	3	Body	Open sea	88.29	4.74	90.84	3.71	-2.54	3.34E-03	4.03E-02
cg11517728	CYFIP1;CYFIP1;CYFIP1	15	Body;5'UTR;1stExon	Open sea	84.84	4.28	87.39	2.89	-2.54	9.19E-05	2.61E-03
cg25123521	MYOZ3;MYOZ3;MYOZ3	5	1stExon;5'UTR;5'UTR	Open sea	81.70	3.14	84.24	2.82	-2.54	1.16E-04	3.16E-03
cg17803713	SH3RF3	2	Body	Open sea	63.55	4.08	66.10	3.78	-2.54	9.71E-04	1.67E-02
cg13740508	C4orf22	4	Body	N_Shore	81.15	3.82	83.69	2.75	-2.54	1.90E-03	2.73E-02
cg15117372	SLC26A8;SLC26A8;SLC26A8	6	5'UTR;5'UTR;1stExon	N_Shelf	44.18	3.45	46.73	3.94	-2.54	1.60E-03	2.42E-02
cg22732253	OTX1	2	Body	N_Shore	34.02	3.13	36.57	4.04	-2.54	1.63E-03	2.45E-02
cg12819676		2	Intergenic	Open sea	83.80	3.85	86.35	3.28	-2.54	2.26E-04	5.45E-03
cg25512179		19	Intergenic	Island	85.66	2.57	88.20	3.40	-2.54	1.38E-04	3.66E-03
cg25650579	FAM133B;FAM133B	7	TSS1500;TSS1500	S_Shore	81.87	4.01	84.41	4.28	-2.54	2.16E-03	2.98E-02
cg02744113	EMX1	2	Body	Island	15.91	3.28	18.45	3.98	-2.54	7.28E-04	1.35E-02
cg23870587	TTC7B	14	Body	Open sea	81.25	3.65	83.79	3.05	-2.54	9.34E-04	1.63E-02
cg16420801	SLC22A17;SLC22A17	14	Body;Body	Island	75.43	4.31	77.97	4.41	-2.54	3.87E-03	4.46E-02

cg01898698	POU3F3	2	1stExon	Island	23.27	3.77	25.81	4.41	-2.54	3.26E-03	3.96E-02
cg17807863	TNIP1	5	Body	Open sea	82.01	3.32	84.55	3.12	-2.54	1.28E-04	3.43E-03
cg01033299	ITGB7	12	TSS200	Open sea	64.48	4.49	67.02	4.47	-2.54	9.44E-04	1.64E-02
cg23851532	ALDH3B2;ALDH3B2	11	3'UTR;3'UTR	N_Shelf	85.24	4.01	87.78	3.22	-2.54	5.06E-04	1.02E-02
cg24936204	COL11A2;COL11A2;COL11A2;COL11A	6	Body;Body;Body;Body	N_Shelf	77.23	3.76	79.78	3.80	-2.54	2.72E-03	3.50E-02
cg04695027	CATSPER1;CATSPER1	11	1stExon;5'UTR	S_Shelf	30.64	4.78	33.18	4.60	-2.54	1.93E-03	2.76E-02
cg13233545		3	Intergenic	Open sea	82.33	3.09	84.87	3.33	-2.54	2.14E-04	5.21E-03
cg25109009	NQO1;NQO1;NQO1	16	Body;Body;Body	Island	20.67	5.28	23.21	6.55	-2.54	2.61E-03	3.40E-02
cg13581437	CCNB2	15	Body	S_Shore	11.06	3.05	13.60	3.67	-2.54	6.44E-06	2.52E-04
cg01734112	C2orf39	2	TSS200	Island	21.35	4.35	23.89	4.50	-2.54	2.25E-03	3.07E-02
cg13376199		8	Intergenic	N_Shore	44.52	4.80	47.06	3.80	-2.54	4.02E-03	4.58E-02
cg09417809		12	Intergenic	S_Shore	62.46	4.42	65.00	4.55	-2.54	2.00E-03	2.83E-02
cg02744524		3	Intergenic	N_Shore	23.25	4.08	25.79	4.58	-2.54	1.46E-03	2.26E-02
cg11827910	PAX6;PAX6;PAX6	11	Body;Body;Body	Island	17.44	3.27	19.98	3.81	-2.54	8.41E-04	1.50E-02
cg11616560	MLLT6	17	Body	Open sea	75.55	3.72	78.09	3.83	-2.54	2.48E-03	3.28E-02
cg18096962		5	Intergenic	Island	18.68	2.31	21.22	2.60	-2.54	2.50E-05	8.52E-04
cg23547048	PCGF3	4	TSS1500	Island	83.98	2.83	86.52	1.89	-2.54	1.99E-05	6.94E-04
cg04830812	PHB2;PHB2	12	3'UTR;3'UTR	Open sea	38.66	3.43	41.20	3.03	-2.54	1.60E-03	2.41E-02
cg18795232	KDM2B;KDM2B	12	Body;Body	N_Shore	54.74	3.50	57.28	3.51	-2.54	1.45E-03	2.25E-02
cg12298745		18	Intergenic	Island	13.50	2.93	16.04	3.22	-2.54	1.96E-03	2.78E-02
cg09287359	LGALS7B	19	TSS1500	N_Shore	65.53	4.03	68.07	3.23	-2.54	9.57E-04	1.66E-02
cg24127506		14	Intergenic	N_Shore	77.40	4.09	79.94	3.85	-2.54	2.58E-03	3.38E-02
cg22211154	NRG4	15	TSS200	Open sea	35.96	5.14	38.50	4.91	-2.54	4.37E-03	4.84E-02
cg21045915		14	Intergenic	Open sea	87.92	3.68	90.46	2.48	-2.54	2.75E-04	6.36E-03
cg17667294	ACER1	19	TSS200	Open sea	85.38	4.40	87.92	3.37	-2.54	2.10E-03	2.93E-02
cg26729217		11	Intergenic	Open sea	7.41	2.82	9.95	3.65	-2.54	5.23E-04	1.05E-02
cg02495569	NEURL1B	5	Body	N_Shore	86.85	4.49	89.39	3.46	-2.54	4.47E-03	4.93E-02
cg10495997	ARAP1	11	5'UTR	S_Shore	70.49	3.75	73.03	3.05	-2.54	1.11E-03	1.85E-02
cg07274716	PITX1	5	3'UTR	Island	19.16	3.44	21.70	3.84	-2.54	1.49E-03	2.29E-02
cg00395990	PDZD3;PDZD3	11	TSS1500;TSS1500	Open sea	78.44	3.66	80.98	2.37	-2.54	1.33E-03	2.11E-02
cg05346902	MEIS3;MEIS3	19	Body;Body	Island	58.39	3.97	60.93	3.66	-2.54	1.91E-03	2.73E-02
cg21236090		5	Intergenic	Open sea	62.49	3.07	65.02	3.36	-2.54	2.46E-04	5.83E-03
cg13279713		11	Intergenic	N_Shelf	67.20	3.09	69.74	2.86	-2.54	6.75E-04	1.28E-02
cg11976164	MIR2054	4	TSS1500	Open sea	85.50	3.65	88.04	3.02	-2.54	3.43E-04	7.55E-03
cg24092295		17	Intergenic	N_Shelf	63.86	4.53	66.40	3.82	-2.54	4.11E-03	4.65E-02
cg26302157	LCE1F	1	TSS1500	Open sea	69.38	4.60	71.92	4.14	-2.54	2.25E-03	3.07E-02
cg02087356		16	Intergenic	Open sea	65.67	3.49	68.21	3.06	-2.54	1.53E-03	2.34E-02
cg09184832		6	Intergenic	Island	15.33	2.75	17.86	4.05	-2.54	1.96E-03	2.79E-02
cg25129716	GIMAP6;GIMAP6	7	TSS1500;TSS1500	Open sea	78.97	3.28	81.50	3.07	-2.54	2.24E-04	5.41E-03
cg11806488		19	Intergenic	Island	18.40	3.34	20.94	2.70	-2.54	1.71E-04	4.37E-03
cg12145394	RMND5B	5	Body	Open sea	40.76	3.12	43.29	3.22	-2.54	1.28E-03	2.05E-02
cg18617808	RNF216;RNF216	7	Body;Body	Open sea	45.03	4.37	47.56	2.87	-2.54	3.56E-03	4.21E-02
cg01386978	PARK2;PARK2;PARK2	6	Body;Body;Body	S_Shore	81.59	3.64	84.12	3.15	-2.54	1.08E-03	1.82E-02
cg12489915	SPAG9;SPAG9	17	Body;Body	N_Shelf	89.36	2.90	91.89	2.12	-2.54	4.15E-05	1.32E-03
cg00178562		1	Intergenic	Open sea	81.93	3.03	84.47	2.30	-2.54	1.45E-04	3.80E-03
cg23118721		8	Intergenic	Open sea	64.66	4.32	67.19	4.14	-2.54	2.17E-03	2.99E-02
cg16439608		2	Intergenic	Open sea	82.80	4.92	85.34	3.46	-2.54	2.66E-03	3.45E-02
cg15501415		1	Intergenic	N_Shelf	76.51	3.07	79.04	2.56	-2.54	1.63E-04	4.18E-03
cg00119053		6	Intergenic	N_Shore	79.69	4.22	82.23	3.89	-2.54	3.88E-03	4.47E-02
cg01570589	PLEKHO1	1	TSS1500	Island	32.48	5.12	35.01	5.26	-2.54	2.56E-03	3.35E-02
cg23201059		8	Intergenic	Open sea	80.04	5.13	82.58	4.85	-2.54	8.12E-04	1.46E-02
cg04238199	C5orf49	5	3'UTR	Open sea	56.11	4.57	58.65	4.10	-2.54	2.91E-03	3.67E-02
cg06614508	ABR;ABR;ABR	17	Body;Body;Body	S_Shore	84.00	4.21	86.54	3.82	-2.54	3.86E-03	4.45E-02

cg17297613	SH3RF3	2	Body	Open sea	78.81	3.78	81.34	4.45	-2.54	2.74E-04	6.33E-03
cg18063017	LHX4	1	Body	Island	18.71	3.30	21.25	4.45	-2.54	1.41E-03	2.20E-02
cg26172051	LPP	3	5'UTR	Open sea	85.63	3.36	88.16	2.37	-2.54	8.30E-05	2.39E-03
cg12671030		2	Intergenic	Island	40.20	4.81	42.74	5.97	-2.54	2.72E-03	3.50E-02
cg11938367	IQCG;IQCG	3	Body;Body	Open sea	78.68	4.17	81.22	4.54	-2.53	3.82E-04	8.21E-03
cg03308652		3	Intergenic	Island	29.73	3.76	32.27	4.38	-2.53	1.34E-03	2.12E-02
cg03606725		16	Intergenic	Open sea	82.43	4.64	84.96	4.72	-2.53	3.03E-03	3.77E-02
cg23159236	GNAS;GNAS;GNAS;GNAS	20	3'UTR;TSS200;3'UTR;Body	Island	26.11	3.45	28.64	4.45	-2.53	2.43E-03	3.24E-02
cg24430580	PITX2;PITX2;PITX2;PITX2	4	1stExon;5'UTR;Body;Body	Island	17.64	2.74	20.17	3.65	-2.53	2.67E-04	6.22E-03
cg07344172	KIF21B	1	Body	Open sea	85.35	3.11	87.89	3.46	-2.53	2.13E-03	2.95E-02
cg08018049	PPP1R10	6	Body	Open sea	81.91	3.58	84.45	2.77	-2.53	6.55E-04	1.25E-02
cg02154436	FAM38A	16	Body	Open sea	83.32	4.71	85.85	3.84	-2.53	8.54E-04	1.52E-02
cg08701208		3	Intergenic	Open sea	84.17	4.03	86.70	3.51	-2.53	3.71E-04	8.04E-03
cg19047660		17	Intergenic	N_Shore	22.59	3.77	25.12	3.80	-2.53	2.41E-03	3.22E-02
cg10066534	TCF21;TCF21	6	TSS200;TSS200	N_Shore	46.94	4.14	49.47	3.81	-2.53	1.07E-03	1.81E-02
cg06223736	HDAC4	2	Body	Open sea	82.37	3.63	84.90	2.58	-2.53	4.21E-03	4.72E-02
cg17724789	PBX1	1	Body	Open sea	83.47	3.43	86.00	3.12	-2.53	2.50E-04	5.91E-03
cg27522078		1	Intergenic	Open sea	31.64	3.74	34.18	5.12	-2.53	4.14E-03	4.67E-02
cg16264705	ATP5G2;ATP5G2	12	TSS1500;TSS1500	Island	55.57	3.76	58.10	4.38	-2.53	5.87E-04	1.15E-02
cg07021696	RCCD1;RCCD1	15	Body;Body	Island	71.51	4.34	74.04	4.42	-2.53	3.20E-03	3.91E-02
cg15225091	SLC18A2	10	Body	S_Shore	23.92	4.18	26.45	4.56	-2.53	4.18E-03	4.70E-02
cg18181034		7	Intergenic	Island	22.15	3.24	24.68	3.12	-2.53	2.64E-04	6.15E-03
cg11435225	SFI1;SFI1	22	3'UTR;3'UTR	Open sea	78.78	3.57	81.31	3.60	-2.53	1.69E-03	2.51E-02
cg07799386	PRDM6	5	Body	Island	21.38	3.05	23.91	3.69	-2.53	2.83E-04	6.50E-03
cg10121146	MFSD4	1	3'UTR	Open sea	83.66	3.35	86.20	2.71	-2.53	8.04E-04	1.45E-02
cg12508693	BDNFOS	11	Body	Open sea	73.35	4.47	75.88	3.48	-2.53	4.34E-03	4.83E-02
cg11756387	PTP4A2;PTP4A2	1	5'UTR;5'UTR	N_Shelf	78.67	4.60	81.20	3.80	-2.53	1.01E-03	1.72E-02
cg00732845		7	Intergenic	N_Shore	25.76	4.94	28.29	4.74	-2.53	2.79E-03	3.56E-02
cg00554871		3	Intergenic	Open sea	85.50	3.78	88.03	3.08	-2.53	4.58E-05	1.44E-03
cg03238216		4	Intergenic	Open sea	66.26	4.60	68.79	3.63	-2.53	8.96E-04	1.58E-02
cg23626862		22	Intergenic	N_Shelf	79.14	4.37	81.68	4.60	-2.53	3.39E-03	4.08E-02
cg21570493		1	Intergenic	N_Shelf	79.09	3.20	81.62	3.08	-2.53	7.66E-04	1.40E-02
cg21685808	CLIC5;CLIC5	6	Body;Body	N_Shore	26.19	3.78	28.72	4.10	-2.53	4.36E-04	9.11E-03
cg25052847		2	Intergenic	Open sea	68.93	6.35	71.46	5.18	-2.53	4.57E-03	4.99E-02
cg19256236	KCNH3	12	TSS1500	Island	21.57	3.18	24.10	3.40	-2.53	8.88E-05	2.54E-03
cg18856043	SLC44A4	6	Body	Open sea	57.91	4.23	60.44	3.63	-2.53	3.09E-03	3.83E-02
cg07511409	CCNJL	5	Body	Open sea	78.14	3.85	80.67	3.79	-2.53	9.31E-04	1.62E-02
cg04175877	ERBB2IP;ERBB2IP	5	Body;Body	Open sea	80.74	4.39	83.27	4.28	-2.53	1.36E-03	2.15E-02
cg20770175	COL3A1	2	Body	Open sea	73.36	5.85	75.89	4.97	-2.53	3.39E-03	4.08E-02
cg25567448	NEURL3	2	TSS1500	S_Shore	75.55	3.79	78.08	3.88	-2.53	3.35E-03	4.04E-02
cg18799510	GRIN3A	9	1stExon	N_Shore	35.11	5.03	37.64	5.57	-2.53	9.67E-04	1.67E-02
cg20992180	FOLR1;FOLR1;FOLR1;FOLR1	11	5'UTR;TSS1500;5'UTR;5'UTR	Open sea	68.02	3.86	70.55	4.44	-2.53	3.37E-03	4.06E-02
cg08358233		10	Intergenic	Open sea	67.84	4.33	70.37	4.45	-2.53	4.42E-03	4.88E-02
cg24380489	DYSFIP1	17	TSS200	S_Shore	86.48	3.55	89.01	2.98	-2.53	2.53E-04	5.97E-03
cg10363926	SH3RF3	2	Body	Open sea	53.00	4.44	55.53	4.39	-2.53	8.53E-04	1.52E-02
cg24555639	HEXDC	17	Body	Island	87.39	3.96	89.92	2.76	-2.53	2.84E-03	3.60E-02
cg16770368	DCHS1	11	5'UTR	N_Shelf	58.84	3.30	61.36	3.34	-2.53	4.32E-04	9.05E-03
cg27077673	PVR;PVR;PVR;PVR	19	Body;Body;Body;Body	S_Shelf	84.06	3.38	86.59	2.86	-2.53	5.50E-04	1.09E-02
cg17183752	MEGF6	1	Body	N_Shelf	74.67	5.01	77.20	5.07	-2.53	2.65E-03	3.44E-02
cg22655696	EBF3	10	Body	Island	20.26	3.42	22.78	4.18	-2.53	4.68E-04	9.63E-03
cg22810684	C17orf62;C17orf62;C17orf62	17	3'UTR;3'UTR;3'UTR	Island	46.45	2.98	48.98	3.48	-2.53	2.94E-03	3.69E-02
cg24634593	ZBTB38	3	5'UTR	S_Shore	10.51	3.00	13.04	3.29	-2.53	2.13E-05	7.39E-04
cg14010394	FAM166A	9	Body	S_Shelf	79.92	4.00	82.44	2.83	-2.53	1.78E-03	2.60E-02

cg14238120	CELA3A	1	TSS200	Open sea	72.45	3.70	74.98	2.68	-2.53	5.72E-04	1.12E-02
cg04651601	OTOA;OTOA	16	Body;TSS1500	Open sea	79.39	3.99	81.92	2.79	-2.53	1.34E-03	2.12E-02
cg07856962		21	Intergenic	Island	78.19	5.24	80.72	4.38	-2.53	3.39E-03	4.08E-02
cg25824760	ANKRD30B	18	TSS200	Island	25.08	2.60	27.61	2.31	-2.53	2.43E-05	8.30E-04
cg00740721	PIGH	14	TSS1500	S_Shore	85.37	3.89	87.90	3.57	-2.53	3.23E-04	7.22E-03
cg01320048	VWA2	10	5'UTR	Island	29.00	3.50	31.53	3.66	-2.53	2.20E-03	3.02E-02
cg24552697	ARHGAP29	1	5'UTR	N_Shelf	75.72	4.71	78.25	4.99	-2.53	2.35E-03	3.16E-02
cg23502378	ZDHHC14;ZDHHC14	6	Body;Body	Open sea	70.98	3.70	73.51	2.82	-2.53	2.75E-03	3.53E-02
cg09403190		7	Intergenic	Open sea	61.02	3.57	63.54	3.31	-2.53	3.22E-03	3.93E-02
cg19688616	LRTOMT;C11orf51;LRTOMT;LRTOMT	11	3'UTR;Body;3'UTR;3'UTR	N_Shore	71.09	4.31	73.61	3.38	-2.53	4.29E-03	4.78E-02
cg20634266		1	Intergenic	Island	26.07	3.15	28.60	3.00	-2.53	6.42E-04	1.23E-02
cg23170410		6	Intergenic	Open sea	59.48	6.28	62.01	6.90	-2.53	1.17E-03	1.93E-02
cg16558925	ARL10	5	Body	S_Shelf	82.85	3.41	85.37	3.09	-2.53	1.85E-04	4.65E-03
cg07830156		1	Intergenic	Open sea	65.89	5.73	68.42	5.10	-2.53	7.53E-04	1.38E-02
cg16021159	PRKAA2	1	Body	Open sea	88.01	1.98	90.54	1.19	-2.53	8.52E-10	4.76E-08
cg19423978	PIP5K1C	19	Body	S_Shore	59.73	4.84	62.26	5.39	-2.53	1.08E-03	1.81E-02
cg10518527		10	Intergenic	Open sea	83.80	3.95	86.32	3.25	-2.53	1.53E-03	2.34E-02
cg07316846		2	Intergenic	S_Shore	7.36	2.63	9.88	4.22	-2.53	2.45E-03	3.26E-02
cg08578136	ZDBF2	2	TSS200	Island	6.55	1.73	9.08	2.15	-2.53	2.51E-08	1.31E-06
cg06114363	ZNF683;ZNF683	1	TSS200;TSS200	Open sea	54.36	4.20	56.89	3.82	-2.52	1.25E-03	2.02E-02
cg15936446		5	Intergenic	Island	22.64	3.46	25.17	3.37	-2.52	1.07E-04	2.96E-03
cg06194809		7	Intergenic	Open sea	34.08	5.49	36.60	5.07	-2.52	2.14E-03	2.97E-02
cg05900530	WDR49	3	TSS200	Open sea	80.58	4.93	83.10	4.25	-2.52	4.42E-03	4.89E-02
cg08105834	SDK1;SDK1	7	Body;Body	Open sea	76.80	3.63	79.32	4.53	-2.52	4.06E-03	4.61E-02
cg26342147	EMX2OS	10	Body	Island	27.33	3.25	29.85	3.14	-2.52	4.37E-04	9.13E-03
cg12070646	VAX2	2	Body	Open sea	78.92	3.66	81.45	2.91	-2.52	2.34E-03	3.15E-02
cg20439473	VEZF1	17	Body	N_Shelf	89.83	2.43	92.35	1.79	-2.52	7.81E-07	3.56E-05
cg23207676	SPTBN4	19	Body	Island	21.18	4.88	23.70	4.86	-2.52	3.67E-03	4.30E-02
cg10662603		3	Intergenic	Open sea	82.52	4.07	85.04	3.78	-2.52	1.05E-03	1.77E-02
cg15127656	ABCC5	3	Body	Open sea	86.37	3.72	88.90	2.55	-2.52	3.91E-04	8.37E-03
cg27150870	ANKRD11	16	5'UTR	Open sea	75.62	4.36	78.14	4.16	-2.52	1.58E-03	2.39E-02
cg04728789	CSNK1E;CSNK1E	22	5'UTR;5'UTR	N_Shore	59.49	4.72	62.01	4.84	-2.52	3.09E-03	3.82E-02
cg19197419	UBE2C;UBE2C;UBE2C;UBE2C;UBE2C;U	20	1stExon;5'UTR;TSS200;Body;Body;Body;Bo	Island	30.25	5.23	32.77	6.96	-2.52	1.46E-03	2.26E-02
cg10039047	B3GNTL1	17	Body	Island	63.24	4.02	65.76	3.89	-2.52	4.25E-03	4.75E-02
cg09575280	RAET1L	6	TSS1500	Open sea	9.02	2.79	11.54	5.51	-2.52	3.85E-03	4.45E-02
cg21417130		1	Intergenic	Open sea	73.22	3.14	75.75	3.06	-2.52	2.69E-04	6.25E-03
cg17533142	SUCLA2	13	Body	N_Shelf	84.82	4.09	87.34	3.53	-2.52	2.61E-03	3.40E-02
cg25707924		3	Intergenic	Island	20.95	3.04	23.48	3.28	-2.52	1.28E-04	3.43E-03
cg24750513	BIN1;BIN1;BIN1;BIN1;BIN1;BIN1;BIN1	2	Body;Body;Body;Body;Body;Body;Body;Bo	S_Shore	76.42	3.71	78.95	3.08	-2.52	4.02E-03	4.58E-02
cg16975095		3	Intergenic	Open sea	45.67	3.71	48.19	3.52	-2.52	3.17E-03	3.88E-02
cg23713998	PRKD1	14	Body	Open sea	80.24	4.30	82.76	4.85	-2.52	9.34E-04	1.63E-02
cg06143244	RRN3P3	16	Body	Open sea	71.80	3.51	74.32	3.61	-2.52	2.63E-03	3.42E-02
cg17337091		3	Intergenic	Open sea	80.96	3.96	83.49	2.95	-2.52	1.95E-03	2.78E-02
cg12986021	DIRAS3	1	TSS200	N_Shore	41.22	4.20	43.74	4.11	-2.52	2.69E-03	3.48E-02
cg24678581	FGF18	5	Body	N_Shore	75.74	4.88	78.26	4.16	-2.52	4.16E-03	4.69E-02
cg15451548		1	Intergenic	S_Shore	50.06	3.63	52.58	3.51	-2.52	1.26E-03	2.04E-02
cg00286188	LTBP1;LTBP1;LTBP1;LTBP1;LTBP1	2	Body;Body;Body;Body;Body	Open sea	77.04	3.90	79.56	4.21	-2.52	1.59E-04	4.12E-03
cg19708803		6	Intergenic	S_Shelf	22.09	3.55	24.61	3.28	-2.52	1.19E-03	1.95E-02
cg04503968	PRDM6	5	TSS1500	N_Shore	28.65	4.00	31.18	3.89	-2.52	8.51E-04	1.52E-02
cg01645401	KLF5	13	Body	Open sea	88.65	3.54	91.17	2.78	-2.52	1.28E-03	2.06E-02
cg00867889	OC90	8	Body	Open sea	81.06	3.52	83.58	3.76	-2.52	2.26E-04	5.45E-03
cg07112337	CNGB1	16	3'UTR	Island	59.01	4.46	61.54	4.79	-2.52	2.27E-03	3.08E-02
cg01179328	RFPL3	22	TSS200	Open sea	76.58	3.69	79.10	3.95	-2.52	2.93E-03	3.69E-02

cg01592257		12	Intergenic	Open sea	83.11	4.93	85.64	3.20	-2.52	3.29E-03	3.99E-02
cg21395191	PANX2;PANX2;PANX2	22	TSS1500;TSS1500;TSS1500	Island	13.71	3.90	16.23	4.04	-2.52	1.24E-04	3.34E-03
cg07958575		7	Intergenic	Open sea	66.87	4.21	69.39	3.91	-2.52	6.31E-04	1.21E-02
cg03787988	MRVI1;MRVI1;MRVI1;MRVI1	11	Body;Body;Body;Body	Open sea	59.25	3.85	61.78	4.30	-2.52	1.83E-03	2.66E-02
cg13776560		14	Intergenic	S_Shore	12.76	1.79	15.29	2.03	-2.52	3.14E-08	1.62E-06
cg22971594	JUB;JUB	14	Body;Body	Open sea	67.69	3.82	70.21	4.22	-2.52	2.15E-03	2.97E-02
cg08143133	RFPL2;RFPL2;RFPL2	22	5'UTR;5'UTR;TSS200	Open sea	17.89	2.50	20.41	2.24	-2.52	1.06E-05	3.98E-04
cg03372076		4	Intergenic	Open sea	80.20	3.98	82.72	3.02	-2.52	1.51E-03	2.31E-02
cg11211384		6	Intergenic	Open sea	85.23	4.21	87.75	2.79	-2.52	1.15E-03	1.90E-02
cg26438284	CD81	11	Body	N_Shore	68.26	3.28	70.78	3.03	-2.52	6.65E-04	1.26E-02
cg10242496	SLC35D3	6	TSS1500	Island	22.92	3.64	25.44	3.53	-2.52	1.75E-04	4.45E-03
cg01417625	LAMC2;LAMC2	1	Body;Body	Open sea	72.31	4.30	74.83	3.79	-2.52	2.86E-03	3.62E-02
cg23130651	COMMD3	10	TSS1500	N_Shore	86.61	4.26	89.13	4.13	-2.52	3.88E-03	4.47E-02
cg24132554		14	Intergenic	N_Shelf	89.80	3.24	92.32	1.50	-2.52	3.39E-05	1.11E-03
cg19713638	LRCH1;LRCH1;LRCH1	13	Body;Body;Body	Open sea	88.33	3.14	90.85	2.43	-2.52	3.96E-04	8.44E-03
cg00908004	PCID2;PCID2;PCID2	13	Body;Body;Body	Open sea	82.36	4.17	84.87	3.57	-2.52	1.55E-03	2.35E-02
cg13064679	TNXB	6	Body	S_Shelf	66.53	4.79	69.05	4.61	-2.52	1.62E-03	2.44E-02
cg14266287	ASB1	2	TSS1500	N_Shore	65.40	4.52	67.92	4.13	-2.52	4.31E-03	4.80E-02
cg17883035	BCL3	19	Body	Island	67.40	4.23	69.92	4.37	-2.52	3.02E-03	3.76E-02
cg19887700		12	Intergenic	Island	9.56	2.75	12.08	3.61	-2.52	2.32E-06	9.84E-05
cg08095204	FGF17	8	TSS1500	Open sea	21.00	2.38	23.52	2.85	-2.52	9.73E-05	2.74E-03
cg21120063	C2orf82	2	TSS200	Open sea	76.07	3.30	78.59	3.69	-2.52	1.46E-03	2.25E-02
cg15965155		5	Intergenic	N_Shelf	56.18	3.13	58.70	3.15	-2.52	1.50E-03	2.30E-02
cg24069262	U2AF2;U2AF2	19	Body;Body	S_Shelf	84.60	3.98	87.12	3.15	-2.52	1.43E-03	2.22E-02
cg00432789	RD3;RD3	1	3'UTR;3'UTR	N_Shore	80.36	3.75	82.88	2.91	-2.52	3.55E-03	4.21E-02
cg01083223		6	Intergenic	Open sea	87.56	4.41	90.07	3.11	-2.52	1.56E-03	2.37E-02
cg22444124	ITPKB	1	Body	Open sea	25.71	4.69	28.23	5.19	-2.52	1.72E-03	2.54E-02
cg24611608	EYA1	8	TSS200	Open sea	20.62	3.70	23.14	3.98	-2.52	3.91E-04	8.37E-03
cg11609780	LOC388796;LOC388796;SNORA71C	20	Body;Body;TSS1500	N_Shelf	79.56	5.71	82.08	5.21	-2.52	3.54E-03	4.20E-02
cg09701702		6	Intergenic	Island	9.25	2.35	11.77	2.83	-2.52	7.88E-07	3.59E-05
cg08759960	LY6G5C	6	TSS1500	Island	14.35	4.24	16.87	4.77	-2.52	2.26E-03	3.07E-02
cg06838581	C7orf27	7	Body	Island	87.94	3.70	90.45	2.03	-2.52	8.06E-04	1.46E-02
cg19915762	PLCB3	11	Body	N_Shelf	61.67	3.21	64.19	4.14	-2.52	4.91E-04	9.98E-03
cg14501119		14	Intergenic	N_Shore	87.26	3.34	89.78	2.11	-2.52	3.99E-04	8.50E-03
cg25084760	PCCA;PCCA	13	Body;Body	Open sea	75.64	4.77	78.16	4.10	-2.52	3.72E-03	4.34E-02
cg21427956	C20orf160	20	3'UTR	S_Shore	24.85	3.62	27.36	3.13	-2.52	1.37E-03	2.16E-02
cg04227278	NDRG1;NDRG1	8	5'UTR;5'UTR	Open sea	76.05	3.86	78.56	3.30	-2.52	1.45E-04	3.81E-03
cg18949786	NXF1;NXF1;TMEM223	11	3'UTR;3'UTR;TSS1500	S_Shore	23.44	3.98	25.95	4.59	-2.52	8.86E-04	1.56E-02
cg23328335	TNXB	6	Body	Open sea	56.61	4.19	59.13	4.45	-2.52	3.09E-04	6.97E-03
cg21301514	KIF26B	1	Body	Open sea	52.18	4.17	54.70	4.11	-2.52	7.81E-04	1.42E-02
cg04228935	RUNX1	21	Body	Island	32.07	5.05	34.58	4.75	-2.52	4.43E-03	4.90E-02
cg25024317	SLC4A3;SLC4A3	2	TSS1500;TSS1500	N_Shore	79.65	3.15	82.16	3.49	-2.52	1.31E-03	2.09E-02
cg25198340	IL17RD	3	Body	Open sea	80.23	3.80	82.75	3.55	-2.52	2.49E-03	3.30E-02
cg06213763		1	Intergenic	Open sea	59.40	4.81	61.91	5.00	-2.52	1.27E-03	2.04E-02
cg24718756	03-Mar	5	5'UTR	Open sea	62.99	5.63	65.50	5.73	-2.52	9.61E-04	1.66E-02
cg17941572		1	Intergenic	Island	23.91	3.77	26.43	3.79	-2.51	3.16E-03	3.88E-02
cg15357945	PRG2	11	TSS1500	Open sea	83.74	3.51	86.25	2.39	-2.51	8.72E-05	2.50E-03
cg25927375	KIRREL3	11	Body	Open sea	54.43	3.37	56.95	2.82	-2.51	1.77E-03	2.60E-02
cg00216570	TFEB;TFEB	6	3'UTR;3'UTR	S_Shore	68.46	3.83	70.98	3.83	-2.51	4.34E-03	4.82E-02
cg26584528	RASGRP2;RASGRP2;RASGRP2	11	TSS1500;5'UTR;5'UTR	Island	22.61	2.90	25.13	3.24	-2.51	7.50E-05	2.20E-03
cg03404726	DUPD1	10	TSS1500	Open sea	81.30	3.69	83.82	2.48	-2.51	7.38E-04	1.36E-02
cg22736037	GIMAP4	7	Body	Open sea	82.56	3.27	85.07	3.21	-2.51	3.74E-04	8.08E-03
cg26701043	BAIAP3	16	Body	N_Shore	62.91	3.57	65.42	3.96	-2.51	2.93E-04	6.67E-03

cg15574642	LRRC59	17	Body	N_Shore	14.42	2.25	16.94	3.36	-2.51	1.99E-04	4.93E-03
cg07678904	FSD1	19	TSS200	Island	13.15	3.26	15.67	3.84	-2.51	1.85E-04	4.65E-03
cg10040329	RFX4	12	Body	Island	12.68	2.95	15.20	3.11	-2.51	3.56E-04	7.79E-03
cg08884932	UBE2J2;UBE2J2;LOC100128842;UBE2J	1	Body;Body;Body;Body;Body	Open sea	57.42	3.37	59.94	2.94	-2.51	5.98E-04	1.16E-02
cg01119831	MAD1L1;MAD1L1;MAD1L1	7	Body;Body;Body	S_Shelf	69.93	4.18	72.45	3.81	-2.51	4.43E-03	4.89E-02
cg12516686		12	Intergenic	Open sea	54.80	7.69	57.31	5.04	-2.51	4.14E-03	4.67E-02
cg08643131	MUM1;MUM1	19	TSS200;5'UTR	S_Shore	15.06	2.97	17.57	4.01	-2.51	3.65E-03	4.29E-02
cg20116979	NCRNA00171	6	Body	Open sea	84.75	3.24	87.26	3.69	-2.51	2.75E-04	6.35E-03
cg25397922		4	Intergenic	Island	17.09	3.06	19.60	3.13	-2.51	5.89E-05	1.79E-03
cg27062060	SLC38A10;SLC38A10	17	Body;Body	N_Shore	71.04	4.43	73.55	4.87	-2.51	2.06E-03	2.89E-02
cg10330057		1	Intergenic	Open sea	53.26	3.59	55.78	3.91	-2.51	2.34E-03	3.15E-02
cg11594770		3	Intergenic	Open sea	16.96	3.04	19.48	3.47	-2.51	3.50E-05	1.14E-03
cg20209290	MAK;MAK	6	1stExon;5'UTR	Open sea	81.16	5.43	83.67	4.57	-2.51	4.12E-03	4.65E-02
cg16083168	UVRAG	11	Body	Open sea	85.48	4.52	88.00	4.56	-2.51	3.90E-03	4.48E-02
cg10204259	VPS13D;VPS13D	1	Body;Body	Open sea	80.48	3.79	83.00	3.53	-2.51	3.80E-03	4.40E-02
cg14952802	PLEKHB2;PLEKHB2	2	TSS1500;TSS1500	N_Shore	71.05	3.30	73.57	2.76	-2.51	1.15E-03	1.91E-02
cg12544191		5	Intergenic	Island	19.04	2.53	21.55	3.04	-2.51	1.48E-04	3.86E-03
cg24575128	NISCH	3	Body	Open sea	63.23	4.37	65.74	3.90	-2.51	2.62E-03	3.41E-02
cg22896354		17	Intergenic	Island	12.15	2.90	14.66	4.06	-2.51	3.08E-03	3.81E-02
cg14058848	TTC16;PTRH1	9	Body;TSS1500	S_Shore	58.39	3.91	60.90	4.51	-2.51	1.41E-03	2.21E-02
cg08734090	C10orf105;C10orf105;C10orf105;CDH	10	1stExon;5'UTR;5'UTR;Body	Open sea	78.50	3.65	81.01	3.33	-2.51	1.46E-04	3.83E-03
cg09642525		16	Intergenic	Island	39.58	2.59	42.09	2.99	-2.51	9.46E-04	1.64E-02
cg21851672	NR5A2;NR5A2	1	Body;Body	N_Shore	48.98	3.08	51.49	3.44	-2.51	7.60E-04	1.39E-02
cg03949978	LRRC14	8	3'UTR	N_Shore	69.21	4.29	71.72	3.77	-2.51	4.46E-03	4.92E-02
cg02486364		7	Intergenic	Open sea	80.69	3.92	83.20	4.15	-2.51	3.14E-03	3.86E-02
cg11681959	KCNK15	20	TSS200	Island	24.13	5.28	26.64	5.54	-2.51	7.67E-04	1.40E-02
cg12733254		1	Intergenic	N_Shelf	81.91	3.92	84.42	3.29	-2.51	2.34E-03	3.16E-02
cg26284483	C17orf79	17	Body	N_Shore	74.77	3.64	77.28	3.34	-2.51	3.33E-04	7.40E-03
cg02257405	ACSL6;ACSL6	5	Body;Body	Island	52.91	3.56	55.42	3.96	-2.51	1.59E-03	2.40E-02
cg08395122	PCDHGA4;PCDHGA9;PCDHGA1;PCDH	5	Body;Body;Body;Body;Body;Body;1stExon	Island	33.38	3.26	35.89	2.71	-2.51	6.20E-04	1.20E-02
cg00992850		7	Intergenic	N_Shore	20.24	3.96	22.75	3.36	-2.51	3.07E-03	3.80E-02
cg16572910		1	Intergenic	Open sea	69.55	3.84	72.06	3.05	-2.51	4.84E-04	9.86E-03
cg07852825	GHSR;GHSR	3	1stExon;1stExon	Island	28.54	3.16	31.05	3.82	-2.51	3.23E-03	3.94E-02
cg14081465	ASTN2;ASTN2;ASTN2	9	Body;Body;Body	Open sea	29.28	4.56	31.79	4.12	-2.51	4.04E-03	4.60E-02
cg23889391	ENPP5;ENPP5	6	1stExon;5'UTR	Open sea	19.10	4.37	21.61	5.17	-2.51	4.73E-04	9.70E-03
cg08146495	C1orf14	1	Body	Open sea	24.39	3.00	26.90	3.47	-2.51	1.66E-03	2.47E-02
cg18103730	GALC;GALC	14	Body;Body	N_Shore	21.03	4.28	23.54	4.68	-2.51	9.79E-04	1.68E-02
cg02600430	ZIC5;ZIC5	13	1stExon;5'UTR	Island	28.98	3.67	31.49	3.82	-2.51	2.32E-03	3.14E-02
cg21141346	EHMT2;EHMT2	6	Body;Body	N_Shelf	83.79	3.14	86.30	2.87	-2.51	1.77E-04	4.49E-03
cg00086429	NRP2;NRP2;NRP2;NRP2;NRP2;NRP2	2	Body;Body;Body;Body;Body;Body	Open sea	67.01	2.70	69.51	2.91	-2.51	8.87E-05	2.53E-03
cg24620635	GNLY;GNLY	2	Body;5'UTR	Open sea	81.73	3.35	84.24	2.97	-2.51	1.12E-03	1.86E-02
cg02573703	SOX2OT;SOX2	3	Body;TSS1500	N_Shore	25.98	2.87	28.49	3.38	-2.51	8.88E-05	2.54E-03
cg08454922		6	Intergenic	Open sea	73.35	4.38	75.86	4.39	-2.51	2.54E-03	3.34E-02
cg20382867		2	Intergenic	Open sea	53.35	5.11	55.86	5.04	-2.51	2.65E-03	3.44E-02
cg21235151		2	Intergenic	Island	18.48	3.34	20.98	4.44	-2.51	4.57E-03	4.99E-02
cg22337509	SOX13	1	Body	Open sea	87.04	2.30	89.55	1.52	-2.51	3.67E-07	1.74E-05
cg26818735	TWIST1	7	1stExon	Island	10.15	2.98	12.66	3.59	-2.51	2.77E-04	6.39E-03
cg11676931		1	Intergenic	Open sea	75.33	4.55	77.83	3.83	-2.51	4.25E-03	4.76E-02
cg15150034		1	Intergenic	S_Shelf	35.09	2.77	37.59	2.67	-2.51	3.42E-04	7.53E-03
cg16904430	CLDN10;CLDN10;CLDN10	13	Body;Body;Body	Open sea	76.33	5.01	78.84	4.37	-2.51	3.40E-03	4.08E-02
cg24226219	RASA3	13	Body	Open sea	77.61	3.76	80.12	3.21	-2.51	2.09E-04	5.11E-03
cg20128319	NEURL3	2	TSS1500	S_Shore	54.02	4.20	56.53	3.89	-2.51	1.73E-03	2.56E-02
cg09954161	NCRNA00171	6	Body	Open sea	61.57	3.26	64.08	3.22	-2.51	2.73E-04	6.31E-03

cg14155035	ATF6B;ATF6B;FKBPL	6	TSS1500;TSS1500;Body	N_Shore	64.93	4.07	67.44	4.12	-2.51	4.28E-03	4.78E-02
cg09179084	TRIM36;TRIM36;TRIM36	5	TSS200;TSS200;TSS200	S_Shore	30.00	3.71	32.50	4.66	-2.51	1.39E-03	2.18E-02
cg15695952	PGAP2;PGAP2;PGAP2;PGAP2;PGAP2;I	11	TSS1500;Body;Body;Body;Body;Body	N_Shore	60.60	2.62	63.11	2.24	-2.51	9.66E-05	2.72E-03
cg06197731		1	Intergenic	Open sea	85.30	2.78	87.80	2.51	-2.51	2.72E-05	9.16E-04
cg15641024	RTN4RL2	11	Body	Island	53.81	3.41	56.32	3.57	-2.51	6.66E-04	1.26E-02
cg22450662	C1orf14	1	Body	Open sea	82.57	4.66	85.08	4.05	-2.50	2.03E-03	2.86E-02
cg26648579		16	Intergenic	Island	78.47	3.83	80.98	3.64	-2.50	1.83E-03	2.65E-02
cg15452970		1	Intergenic	Island	31.33	2.51	33.84	2.47	-2.50	6.02E-05	1.82E-03
cg25368728	SLC44A4	6	Body	S_Shelf	81.44	3.67	83.94	3.47	-2.50	2.19E-03	3.01E-02
cg25643118	ELOVL4	6	TSS200	Island	13.94	3.24	16.44	3.47	-2.50	1.71E-05	6.08E-04
cg03538787		19	Intergenic	Open sea	30.30	2.63	32.81	3.24	-2.50	9.76E-04	1.68E-02
cg09106624	HOXC13	12	TSS200	N_Shore	13.03	2.07	15.54	2.67	-2.50	1.74E-05	6.17E-04
cg15543736	SBF2	11	Body	N_Shelf	80.97	3.25	83.47	3.62	-2.50	4.30E-04	9.03E-03
cg18355533	MIR637;DAPK3	19	TSS200;Body	S_Shore	80.77	4.51	83.27	4.16	-2.50	1.64E-03	2.45E-02
cg12670310	DGCR8	22	5'UTR	N_Shelf	74.62	3.68	77.13	3.23	-2.50	3.90E-03	4.49E-02
cg04086977		10	Intergenic	N_Shore	75.85	4.11	78.35	3.48	-2.50	1.23E-03	2.00E-02
cg09570676	PI15	8	Body	Open sea	48.05	4.16	50.55	3.11	-2.50	3.07E-03	3.81E-02
cg20697519	SLAMF8	1	TSS1500	Open sea	79.32	3.63	81.82	3.34	-2.50	4.98E-04	1.01E-02
cg17950186	MICA	6	Body	S_Shelf	80.27	3.81	82.78	2.61	-2.50	3.35E-03	4.04E-02
cg17796348	STATH;STATH	4	5'UTR;5'UTR	Open sea	81.64	4.73	84.15	4.62	-2.50	1.97E-03	2.80E-02
cg09126870		12	Intergenic	Island	19.59	3.50	22.10	3.39	-2.50	1.52E-03	2.32E-02
cg14689979	NPY6R	5	Body	Open sea	88.81	3.86	91.32	3.74	-2.50	3.16E-04	7.09E-03
cg04847273	GABRD	1	Body	N_Shore	62.87	4.17	65.38	3.32	-2.50	2.16E-03	2.98E-02
cg21545313	MECOM	3	TSS1500	S_Shore	33.50	3.36	36.00	3.63	-2.50	9.01E-04	1.59E-02
cg18016181	FOXD4	9	1stExon	Island	14.78	2.50	17.28	4.09	-2.50	2.30E-04	5.53E-03
cg14374337		14	Intergenic	N_Shore	71.01	4.80	73.51	4.50	-2.50	4.20E-03	4.71E-02
cg07781162	CYGB	17	TSS200	Island	9.96	1.92	12.46	3.26	-2.50	1.87E-05	6.60E-04
cg03520624	SHANK2	11	Body	Open sea	84.25	2.91	86.75	2.85	-2.50	4.02E-04	8.55E-03
cg11349631	NXN	17	Body	S_Shore	86.80	5.39	89.30	4.44	-2.50	2.62E-03	3.41E-02
cg20316713	YIF1A	11	Body	N_Shelf	77.43	4.18	79.94	3.43	-2.50	2.39E-03	3.21E-02
cg17566710	FAM193A	4	Body	Open sea	85.70	3.95	88.21	3.33	-2.50	2.17E-03	2.99E-02
cg02299360	BTG2	1	Body	Island	35.12	6.43	37.62	8.13	-2.50	1.25E-03	2.02E-02
cg12999109	ACER1;ACER1	19	5'UTR;1stExon	Open sea	87.15	3.89	89.65	2.86	-2.50	1.09E-03	1.82E-02
cg06970472	APBB2;APBB2;APBB2	4	Body;Body;Body	Open sea	37.36	3.77	39.86	3.68	-2.50	1.61E-04	4.16E-03
cg01628067	SKI	1	Body	Island	26.83	4.74	29.33	3.90	-2.50	9.90E-04	1.70E-02
cg08690031	CDCA7;CDCA7	2	TSS1500;TSS1500	N_Shore	9.23	2.13	11.73	3.32	-2.50	1.26E-05	4.64E-04
cg08877148	PRICKLE2	3	Body	Open sea	85.44	4.79	87.94	3.17	-2.50	4.11E-03	4.65E-02
cg20105087	PLTP;PLTP	20	TSS1500;TSS1500	S_Shore	61.83	4.08	64.33	4.12	-2.50	1.33E-03	2.12E-02
cg16198450	FABP6;FABP6;FABP6	5	TSS1500;Body;Body	Open sea	76.17	4.31	78.67	4.43	-2.50	3.99E-03	4.56E-02
cg14871601		7	Intergenic	Island	15.33	3.06	17.83	3.40	-2.50	1.58E-04	4.08E-03
cg23653667	ALG14	1	Body	Open sea	77.82	3.78	80.32	2.97	-2.50	1.07E-03	1.79E-02
cg13553111		15	Intergenic	N_Shore	83.72	3.52	86.22	2.78	-2.50	9.78E-04	1.68E-02
cg17076443	FYN	6	5'UTR	Open sea	49.35	3.25	51.85	2.99	-2.50	4.40E-04	9.18E-03
cg12046758		17	Intergenic	Island	24.09	3.40	26.59	4.14	-2.50	3.96E-04	8.45E-03
cg11719362		7	Intergenic	N_Shore	16.09	2.82	18.59	4.03	-2.50	1.47E-03	2.27E-02
cg16275882		7	Intergenic	S_Shore	18.05	3.14	20.55	3.36	-2.50	1.26E-04	3.38E-03
cg14050954	ACAP1	17	TSS1500	Open sea	68.15	4.68	70.65	4.74	-2.50	7.87E-04	1.43E-02
cg08480461	MACF1	1	Body	Open sea	58.58	4.35	61.08	3.95	-2.50	2.30E-03	3.11E-02
cg06993255		8	Intergenic	S_Shore	85.67	3.89	88.17	2.94	-2.50	8.07E-04	1.46E-02
cg06803253	GNAT1;GNAT1	3	Body;Body	N_Shore	85.89	3.52	88.39	2.45	-2.50	2.52E-03	3.32E-02
cg03249630	ANKRD22	10	TSS200	Open sea	85.17	4.86	87.67	2.72	-2.50	2.28E-03	3.09E-02
cg12105240	TMEM175	4	5'UTR	N_Shelf	65.28	3.60	67.78	3.32	-2.50	1.12E-03	1.86E-02
cg13444243		9	Intergenic	S_Shore	76.87	3.09	79.37	3.96	-2.50	2.63E-04	6.14E-03

cg04519775		2	Intergenic	N_Shelf	60.41	4.43	62.91	3.87	-2.50	4.94E-04	1.00E-02
cg06430220	ZNF787	19	Body	N_Shore	78.69	4.70	81.19	5.00	-2.50	1.44E-03	2.24E-02
cg07687820		3	Intergenic	S_Shelf	65.20	3.47	67.70	4.01	-2.50	3.23E-03	3.94E-02
cg08867767	GJA9	1	5'UTR	S_Shelf	65.70	3.55	68.20	3.95	-2.50	2.52E-03	3.32E-02
cg20943039		15	Intergenic	Open sea	20.43	3.43	22.93	3.52	-2.50	3.94E-04	8.42E-03
cg14093663	PIP5K1C	19	Body	N_Shore	86.28	3.77	88.78	2.03	-2.50	4.05E-03	4.60E-02
cg25624359	MEGF6	1	Body	S_Shore	78.81	3.27	81.31	3.17	-2.50	2.56E-03	3.36E-02
cg07911361	PRR5;PRR5;PRR5;PRR5-ARHGAP8;PRF	22	Body;Body;Body;Body;Body;Body	S_Shore	52.68	2.99	55.17	3.32	-2.50	1.66E-03	2.48E-02
cg10919889	NRXN1;NRXN1	2	Body;Body	Open sea	81.46	2.83	83.96	2.49	-2.50	6.82E-06	2.66E-04
cg14039779	DUSP3;C17orf105	17	TSS1500;TSS200	S_Shore	74.81	4.03	77.31	3.12	-2.50	1.20E-04	3.26E-03
cg03759094	PIGQ;PIGQ	16	Body;Body	N_Shelf	66.55	2.94	69.05	2.60	-2.50	3.78E-04	8.15E-03
cg16541653	ADAM21P1	14	Body	Open sea	74.08	3.54	76.58	3.62	-2.50	4.01E-03	4.58E-02
cg15099666		7	Intergenic	Open sea	15.37	3.49	17.86	3.00	-2.50	2.89E-04	6.61E-03
cg20460554	RSPH1	21	TSS1500	Island	62.82	4.12	65.32	4.02	-2.50	4.06E-03	4.61E-02
cg17024482	ANKFN1	17	TSS200	Open sea	66.01	4.17	68.50	4.88	-2.50	4.14E-03	4.67E-02
cg16561266	LOC146880;LOC146880	17	Body;TSS200	Island	25.71	5.52	28.21	5.66	-2.50	3.24E-03	3.95E-02
cg17196739	C3orf25	3	3'UTR	Open sea	85.30	3.07	87.79	2.38	-2.50	4.98E-04	1.01E-02
cg10568796	PI4KA	22	5'UTR	Open sea	81.04	3.69	83.54	3.51	-2.50	1.84E-03	2.67E-02
cg23757136		6	Intergenic	Open sea	85.29	4.56	87.79	3.67	-2.50	3.67E-03	4.30E-02
cg17135191	MMP17	12	Body	S_Shore	78.20	4.25	80.70	4.03	-2.50	1.58E-03	2.39E-02
cg02618733	ATP8B3	19	Body	Island	18.22	4.04	20.71	3.59	-2.50	4.47E-03	4.93E-02
cg08903261		1	Intergenic	S_Shelf	69.62	4.06	72.12	3.55	-2.50	2.72E-03	3.50E-02
cg24427724	VWF	12	TSS1500	Open sea	49.98	4.17	52.48	4.09	-2.50	2.78E-03	3.55E-02
cg21914362	WHSC1;WHSC1;WHSC1;SCARNA22;W	4	Body;Body;Body;TSS1500;Body	Open sea	81.03	3.89	83.52	3.14	-2.50	4.64E-04	9.57E-03
cg07857119		4	Intergenic	Island	83.72	3.38	86.22	3.42	-2.50	5.56E-04	1.10E-02
cg23105697	HOXD10	2	Body	S_Shore	27.22	3.82	29.71	3.97	-2.50	5.57E-04	1.10E-02
cg17361203	DSCAML1	11	Body	Island	12.58	2.79	15.08	3.64	-2.50	1.14E-03	1.89E-02
cg15916646	HOXA11AS;HOXA11	7	Body;TSS1500	Island	28.71	3.20	31.21	3.91	-2.50	6.47E-04	1.24E-02
cg02739040		13	Intergenic	N_Shore	15.84	3.49	18.34	3.84	-2.50	6.62E-04	1.26E-02
cg15661311	RNH1;RNH1;RNH1;RNH1;RNH1;RNH1	11	5'UTR;5'UTR;5'UTR;5'UTR;5'UTR;5'UTR;5'U	Island	26.85	3.88	29.35	4.80	-2.49	2.86E-03	3.62E-02
cg24772240	IGFBP3;IGFBP3	7	TSS1500;TSS1500	S_Shore	33.94	3.60	36.43	5.25	-2.49	3.87E-03	4.46E-02
cg03780132		5	Intergenic	Island	19.33	3.45	21.82	4.24	-2.49	5.53E-04	1.09E-02
cg03464655	SLC38A11;SLC38A11	2	1stExon;5'UTR	Open sea	12.35	3.30	14.84	3.55	-2.49	2.31E-03	3.13E-02
cg27200267	WNT5B	12	TSS200	Open sea	61.92	3.59	64.42	4.03	-2.49	1.41E-03	2.20E-02
cg03213216	RTN2;FLJ40125;RTN2	19	TSS1500;1stExon;TSS1500	N_Shore	26.19	3.51	28.69	3.79	-2.49	5.03E-04	1.02E-02
cg01423820	VASH1;VASH1	14	1stExon;5'UTR	Island	28.06	4.11	30.55	5.01	-2.49	1.24E-03	2.01E-02
cg01897323	TXNDC6	3	TSS1500	S_Shore	30.04	3.77	32.54	4.37	-2.49	5.87E-04	1.15E-02
cg18818644		6	Intergenic	Open sea	76.55	4.04	79.04	4.76	-2.49	3.03E-04	6.86E-03
cg14496983	RNASE4;ANG	14	TSS200;TSS200	N_Shore	19.27	2.61	21.77	2.87	-2.49	5.20E-05	1.61E-03
cg02470045		19	Intergenic	Open sea	11.43	3.83	13.92	4.38	-2.49	2.88E-03	3.64E-02
cg23961466		5	Intergenic	Island	21.99	3.37	24.48	3.89	-2.49	3.77E-03	4.38E-02
cg01298731	SFRP2	4	TSS1500	N_Shore	79.51	4.30	82.00	3.21	-2.49	3.40E-03	4.08E-02
cg11558212		13	Intergenic	Open sea	33.35	4.38	35.84	4.62	-2.49	2.38E-03	3.20E-02
cg27228601	EPC2	2	Body	Open sea	86.66	3.69	89.15	3.76	-2.49	3.50E-04	7.68E-03
cg13430755		9	Intergenic	Open sea	16.23	3.26	18.73	2.74	-2.49	6.14E-04	1.19E-02
cg24479522	LTB;LTB	6	Body;Body	S_Shore	48.56	6.04	51.05	6.01	-2.49	1.87E-03	2.70E-02
cg09565326	ALG10	12	3'UTR	Open sea	82.98	4.06	85.47	3.41	-2.49	3.67E-03	4.30E-02
cg19510699	SDCBP2	20	Body	S_Shelf	52.73	4.55	55.22	3.51	-2.49	1.52E-03	2.33E-02
cg12572625	PCDHB1;PCDHB1	5	1stExon;5'UTR	Open sea	11.55	2.45	14.04	3.50	-2.49	8.14E-04	1.47E-02
cg01490102	GPR177;GPR177	1	TSS1500;TSS1500	S_Shore	14.02	3.51	16.51	4.79	-2.49	5.42E-04	1.08E-02
cg08374472	ZCCHC14	16	Body	Island	67.15	3.71	69.64	4.63	-2.49	3.04E-03	3.78E-02
cg22082347	UBE2C;UBE2C;UBE2C;UBE2C;UBE2C;U	20	1stExon;5'UTR;TSS200;Body;Body;Body;Bo	Island	11.96	2.47	14.45	3.84	-2.49	2.37E-05	8.13E-04
cg12612445	C21orf67	21	Body	S_Shore	68.89	3.55	71.38	3.33	-2.49	1.81E-03	2.63E-02

cg09023965	EXOC6B	2	Body	Open sea	85.37	5.32	87.86	3.44	-2.49	1.89E-03	2.71E-02
cg25995452	GALNTL6	4	Body	Open sea	69.09	3.22	71.58	2.91	-2.49	5.64E-04	1.11E-02
cg26658897	LHFPL2	5	5'UTR	Open sea	76.76	3.79	79.26	3.57	-2.49	2.59E-03	3.38E-02
cg04560503	TRRAP	7	Body	Open sea	84.44	1.83	86.93	1.86	-2.49	4.90E-09	2.65E-07
cg09883849		7	Intergenic	Open sea	57.66	2.38	60.15	1.89	-2.49	4.23E-05	1.34E-03
cg00703061		18	Intergenic	S_Shelf	82.79	3.81	85.28	3.01	-2.49	1.49E-03	2.29E-02
cg19593229	ZNF92;ZNF92	7	5'UTR;Body	S_Shore	46.57	2.74	49.06	2.22	-2.49	7.89E-05	2.29E-03
cg01644518	HIPK4	19	Body	Island	90.42	3.45	92.91	1.79	-2.49	8.45E-06	3.23E-04
cg14024502	MAP3K6	1	TSS1500	S_Shore	68.52	3.43	71.01	3.01	-2.49	1.82E-03	2.65E-02
cg23541273	PPM1G;PPM1G	2	Body;Body	N_Shore	18.14	3.24	20.63	3.28	-2.49	3.61E-04	7.86E-03
cg01882222		5	Intergenic	N_Shelf	62.72	3.46	65.21	3.05	-2.49	7.76E-04	1.42E-02
cg11027717		11	Intergenic	Open sea	13.79	3.24	16.28	3.51	-2.49	3.01E-03	3.75E-02
cg04428290	GALNS	16	Body	Island	86.91	3.83	89.40	2.84	-2.49	1.55E-03	2.36E-02
cg26034769	NEDD4L;NEDD4L;NEDD4L;NEDD4L	18	1stExon;5'UTR;Body;Body	S_Shelf	45.77	3.95	48.26	3.77	-2.49	2.71E-03	3.50E-02
cg26489818		5	Intergenic	N_Shelf	56.52	3.54	59.01	3.14	-2.49	9.31E-04	1.62E-02
cg13580008	OSTBETA	15	5'UTR	Open sea	45.77	5.23	48.26	6.10	-2.49	3.72E-03	4.34E-02
cg19876216	HSP90AB2P	4	Body	Open sea	77.89	3.55	80.38	2.79	-2.49	3.93E-04	8.40E-03
cg27464378		16	Intergenic	S_Shore	80.56	3.46	83.05	2.39	-2.49	2.40E-04	5.73E-03
cg24846791	TRIM31	6	Body	S_Shelf	71.19	2.53	73.68	2.53	-2.49	9.10E-05	2.59E-03
cg08255724	C6orf227	6	Body	N_Shore	72.60	3.68	75.09	3.00	-2.49	4.73E-04	9.70E-03
cg02817981	MOBKL3;MOBKL3;MOBKL3	2	TSS1500;TSS1500;TSS1500	N_Shore	36.28	4.67	38.77	5.13	-2.49	2.42E-03	3.23E-02
cg15229943		10	Intergenic	Island	23.36	3.21	25.84	3.15	-2.49	1.06E-04	2.93E-03
cg15531814	KLK2;KLK2	19	TSS1500;TSS1500	Open sea	54.21	2.81	56.70	3.86	-2.49	2.92E-04	6.66E-03
cg07108754		15	Intergenic	Open sea	85.44	3.30	87.93	3.15	-2.49	2.30E-04	5.53E-03
cg26771998	BMS1P4	10	TSS200	Island	24.27	3.11	26.76	3.43	-2.49	8.19E-04	1.47E-02
cg27045723		16	Intergenic	Island	82.28	4.35	84.77	3.11	-2.49	4.37E-03	4.84E-02
cg15934607	RASA3	13	Body	N_Shore	82.90	3.19	85.39	2.77	-2.49	1.09E-03	1.83E-02
cg12337988		3	Intergenic	N_Shelf	86.13	3.86	88.62	4.24	-2.49	5.20E-04	1.04E-02
cg22022797	SAFB2	19	Body	Island	84.52	1.98	87.01	2.06	-2.49	4.43E-07	2.08E-05
cg22881141	TRMU	22	TSS1500	N_Shore	74.78	5.02	77.27	4.93	-2.49	3.92E-03	4.50E-02
cg27579609	HTR6	1	Body	N_Shelf	14.10	2.75	16.59	3.96	-2.49	1.45E-03	2.25E-02
cg26815395		1	Intergenic	Open sea	21.72	4.04	24.21	4.61	-2.49	2.01E-03	2.84E-02
cg22143809		7	Intergenic	N_Shelf	52.12	3.27	54.61	3.94	-2.49	3.96E-04	8.45E-03
cg13742513	SLC25A41	19	TSS200	Open sea	72.47	5.34	74.96	3.98	-2.49	3.96E-03	4.54E-02
cg00164894	USP24	1	Body	Open sea	81.04	4.20	83.52	3.64	-2.49	3.91E-03	4.50E-02
cg25551262	CLSPN	1	Body	N_Shelf	85.88	4.24	88.37	2.83	-2.49	4.06E-03	4.61E-02
cg08135437	PSMD14	2	Body	Open sea	81.84	4.40	84.33	4.32	-2.49	1.17E-03	1.92E-02
cg16973098		13	Intergenic	N_Shore	19.61	2.65	22.10	2.76	-2.49	3.92E-04	8.39E-03
cg11807529	ARHGEF10L;ARHGEF10L	1	Body;Body	Open sea	73.61	3.52	76.10	3.71	-2.49	3.10E-03	3.83E-02
cg15149095	SYT14;SYT14;SYT14;SYT14;SYT14;SYT1	1	Body;Body;Body;Body;Body;Body	Island	17.88	2.80	20.37	3.75	-2.49	2.66E-04	6.19E-03
cg19272754	PC;PC;PC	11	Body;Body;Body	Island	83.38	3.54	85.87	2.92	-2.49	2.49E-03	3.29E-02
cg23074992	GSC	14	TSS1500	N_Shore	49.23	4.20	51.72	4.34	-2.49	3.01E-03	3.75E-02
cg22864617		2	Intergenic	Open sea	80.84	3.45	83.33	3.43	-2.49	8.86E-04	1.56E-02
cg11220060	KLF1	19	Body	S_Shore	48.53	4.92	51.02	4.60	-2.49	4.17E-03	4.70E-02
cg02633409	KDM4B	19	Body	S_Shelf	86.90	3.63	89.39	2.66	-2.49	4.69E-04	9.65E-03
cg00155622	RTF1	15	Body	S_Shore	16.65	2.83	19.13	3.88	-2.49	2.00E-04	4.94E-03
cg14799446	FKRP;FKRP;STRN4;STRN4	19	5'UTR;5'UTR;TSS1500;TSS1500	S_Shore	74.59	4.20	77.08	4.46	-2.49	3.70E-03	4.33E-02
cg18050892	TAP2;TAP2	6	3'UTR;Body	Open sea	87.95	3.71	90.43	2.75	-2.48	1.07E-03	1.80E-02
cg21778348	HOXA7	7	Body	Island	12.17	1.92	14.65	2.03	-2.48	2.52E-07	1.22E-05
cg06824720	TNXB;TNXB	6	Body;TSS1500	Open sea	80.46	3.40	82.95	2.52	-2.48	1.24E-03	2.01E-02
cg13389958	EVX1	7	TSS1500	N_Shore	30.11	4.44	32.59	4.22	-2.48	2.76E-03	3.54E-02
cg12845808	PCDH12;PCDH12	5	1stExon;5'UTR	Open sea	74.99	3.46	77.47	2.76	-2.48	1.49E-03	2.30E-02
cg03962846	POU6F2;POU6F2	7	Body;Body	Open sea	86.10	4.19	88.59	3.54	-2.48	8.10E-04	1.46E-02

cg26334832	IFT52	20	5'UTR	S_Shore	33.33	5.60	35.82	5.88	-2.48	2.05E-03	2.88E-02
cg07315493	IFT140	16	Body	S_Shelf	73.28	3.62	75.76	2.86	-2.48	3.36E-03	4.05E-02
cg15203271	LOC100130872;LOC100130872-SPON2	4	TSS1500;TSS1500	Island	72.02	5.04	74.51	4.96	-2.48	2.85E-03	3.61E-02
cg04112704	DICER1;DICER1	14	5'UTR;TSS1500	Open sea	74.27	4.31	76.75	3.88	-2.48	3.76E-03	4.37E-02
cg16098340	MIR548C;RASSF3	12	TSS1500;Body	Open sea	80.66	4.17	83.14	3.81	-2.48	4.00E-03	4.56E-02
cg00082219	FEV	2	3'UTR	Island	32.45	4.75	34.94	5.00	-2.48	2.28E-03	3.10E-02
cg12486498	C1orf198;C1orf198;C1orf198	1	3'UTR;3'UTR;3'UTR	Open sea	80.13	3.69	82.62	2.54	-2.48	9.71E-04	1.67E-02
cg03060036		17	Intergenic	S_Shelf	80.97	3.97	83.45	4.34	-2.48	3.30E-04	7.33E-03
cg08034663	NCOR2;NCOR2	12	Body;Body	Open sea	88.70	2.85	91.18	2.07	-2.48	4.01E-05	1.29E-03
cg06196145	PNPLA2	11	TSS200	N_Shore	13.83	2.80	16.32	3.69	-2.48	9.50E-04	1.65E-02
cg08376992	DPEP2	16	TSS200	Open sea	29.56	4.01	32.04	4.46	-2.48	1.45E-03	2.25E-02
cg05836145	SP8;SP8	7	TSS1500;TSS1500	S_Shore	33.35	2.86	35.84	3.73	-2.48	2.84E-03	3.61E-02
cg25283159	RAVER1	19	Body	N_Shelf	83.26	3.78	85.74	3.44	-2.48	2.85E-03	3.61E-02
cg08772789	SEPT9;SEPT9;SEPT9	17	5'UTR;Body;Body	Open sea	64.06	4.44	66.55	3.71	-2.48	3.33E-03	4.03E-02
cg07984515		12	Intergenic	Open sea	44.56	5.17	47.04	5.64	-2.48	2.42E-03	3.23E-02
cg04264495	SUPT3H;SUPT3H	6	Body;Body	Open sea	88.12	4.95	90.60	2.77	-2.48	8.84E-04	1.56E-02
cg16983723		3	Intergenic	Open sea	61.84	4.36	64.32	4.28	-2.48	3.89E-03	4.48E-02
cg17513592		2	Intergenic	N_Shelf	67.03	4.76	69.51	3.67	-2.48	3.40E-03	4.08E-02
cg06821992		5	Intergenic	Open sea	50.41	5.58	52.89	6.22	-2.48	1.65E-03	2.46E-02
cg19307545	PARD3	10	Body	Open sea	39.03	4.21	41.51	4.19	-2.48	2.18E-03	3.00E-02
cg04766618	SMTNL1	11	Body	Open sea	85.98	3.35	88.46	2.69	-2.48	2.41E-03	3.22E-02
cg15571896	DST;DST;DST;DST	6	Body;Body;Body;Body	Open sea	85.65	4.36	88.13	4.11	-2.48	1.46E-05	5.30E-04
cg16301975		6	Intergenic	S_Shelf	84.40	4.83	86.88	3.05	-2.48	4.08E-03	4.63E-02
cg01390564	PRSS36	16	TSS1500	S_Shelf	57.81	3.34	60.29	2.62	-2.48	4.62E-04	9.53E-03
cg10328047	PLEC1;PLEC1;PLEC1;PLEC1	8	Body;1stExon;Body;Body	Island	87.19	3.47	89.67	2.79	-2.48	1.07E-04	2.96E-03
cg20935841	MED24;MED24;SNORD124	17	Body;Body;TSS1500	Open sea	68.51	3.77	70.99	3.20	-2.48	2.82E-03	3.59E-02
cg27278640		8	Intergenic	Open sea	84.23	3.72	86.71	2.35	-2.48	5.44E-04	1.08E-02
cg20582800	RSPH9	6	TSS200	Island	40.73	2.99	43.21	2.64	-2.48	1.74E-04	4.42E-03
cg00081465		18	Intergenic	N_Shelf	58.85	4.42	61.33	3.54	-2.48	2.11E-03	2.94E-02
cg22700148	CACNG1	17	Body	S_Shore	64.46	4.17	66.94	3.89	-2.48	1.20E-03	1.97E-02
cg22517356	TMEM88B	1	TSS1500	N_Shore	48.11	3.42	50.59	4.39	-2.48	4.93E-04	1.00E-02
cg10367023		2	Intergenic	Island	15.77	2.13	18.25	2.80	-2.48	1.14E-05	4.23E-04
cg16691568	OR7D4	19	TSS1500	Open sea	56.55	3.23	59.03	3.54	-2.48	6.12E-04	1.18E-02
cg02279625	SH2D7	15	TSS1500	Open sea	68.23	4.12	70.71	3.87	-2.48	3.53E-03	4.19E-02
cg08519191		5	Intergenic	Open sea	68.69	3.09	71.17	3.85	-2.48	2.76E-03	3.54E-02
cg24662154	EXT2;EXT2	11	5'UTR;5'UTR	S_Shore	18.19	2.80	20.67	3.32	-2.48	1.60E-04	4.13E-03
cg00032490		12	Intergenic	Open sea	80.47	3.91	82.95	3.13	-2.48	4.59E-04	9.48E-03
cg24646414	GATA4	8	5'UTR	Island	51.85	4.64	54.33	4.46	-2.48	2.76E-03	3.53E-02
cg15944824		17	Intergenic	N_Shelf	58.88	3.06	61.36	2.85	-2.48	3.06E-04	6.90E-03
cg22012333	RPS25;TRAPPC4;RPS25	11	1stExon;TSS1500;5'UTR	Island	25.17	3.27	27.65	2.84	-2.48	9.38E-05	2.66E-03
cg17606491	CHST15	10	TSS200	S_Shelf	63.24	4.38	65.72	4.65	-2.48	8.20E-04	1.47E-02
cg00323915	GIMAP4	7	5'UTR	Open sea	62.01	5.20	64.49	4.45	-2.48	2.92E-03	3.67E-02
cg01088658	ENAM	4	3'UTR	Open sea	83.62	4.34	86.10	3.18	-2.48	3.80E-03	4.41E-02
cg12840502	CABP7	22	Body	Island	10.97	2.47	13.45	3.95	-2.48	8.24E-05	2.38E-03
cg01483824	GRIN2D	19	Body	Island	88.44	4.09	90.92	3.09	-2.48	2.58E-03	3.38E-02
cg11324957		15	Intergenic	Island	41.75	4.17	44.23	4.21	-2.48	1.77E-03	2.59E-02
cg17983632		6	Intergenic	S_Shore	81.07	2.65	83.55	3.25	-2.48	1.72E-04	4.38E-03
cg14630933	TNXB	6	5'UTR	Open sea	20.41	4.83	22.88	4.97	-2.48	3.16E-03	3.88E-02
cg00066184	IGSF11;IGSF11	3	Body;Body	Open sea	60.63	3.84	63.11	4.03	-2.48	2.13E-03	2.96E-02
cg02859934	NAGPA	16	Body	N_Shore	19.75	3.40	22.23	2.95	-2.48	1.94E-03	2.77E-02
cg06700330	PLXDC1	17	Body	N_Shore	55.75	3.66	58.23	3.51	-2.48	3.42E-03	4.10E-02
cg24057204	GRID1	10	Body	Open sea	71.20	5.15	73.67	5.08	-2.48	1.37E-03	2.15E-02
cg09782889		6	Intergenic	S_Shore	16.71	3.78	19.19	5.45	-2.48	2.18E-03	3.00E-02

cg10021043	CNRIP1;CNRIP1	2	TSS200;TSS200	S_Shore	26.26	5.70	28.74	5.62	-2.48	3.06E-03	3.79E-02
cg18669135		2	Intergenic	S_Shore	88.83	1.88	91.31	1.14	-2.48	3.27E-10	1.86E-08
cg14528596	CHCHD6	3	Body	Open sea	85.91	3.47	88.38	2.76	-2.48	1.36E-03	2.15E-02
cg15795515		8	Intergenic	Open sea	75.52	5.33	77.99	6.60	-2.48	1.89E-03	2.72E-02
cg21629895	DNMT3A;DNMT3A;DNMT3A	2	TSS1500;TSS1500;TSS1500	S_Shore	79.16	3.34	81.64	3.26	-2.48	1.11E-03	1.85E-02
cg02751838	LDHD;LDHD	16	Body;Body	N_Shore	77.84	3.33	80.32	3.09	-2.48	2.22E-03	3.04E-02
cg08596486		8	Intergenic	Open sea	71.46	3.31	73.94	2.72	-2.48	1.06E-04	2.95E-03
cg06445981	MIR518B	19	Body	Open sea	82.84	4.11	85.31	4.92	-2.48	4.27E-03	4.77E-02
cg02213234	PKD1L3	16	TSS1500	Open sea	59.20	5.18	61.68	5.37	-2.48	1.47E-03	2.27E-02
cg00116430		1	Intergenic	Open sea	66.90	3.46	69.37	3.19	-2.48	4.84E-04	9.87E-03
cg02800334	ANXA13;ANXA13	8	TSS1500;TSS1500	Open sea	85.33	4.45	87.80	3.77	-2.48	1.41E-03	2.21E-02
cg17805072	GPRIN1	5	5'UTR	N_Shelf	68.88	4.43	71.35	4.51	-2.48	2.76E-03	3.53E-02
cg23553659	ZC3H13	13	TSS200	Island	4.77	1.47	7.25	4.25	-2.47	4.54E-05	1.43E-03
cg20999565	ZFYVE26	14	Body	Open sea	28.52	2.80	30.99	3.43	-2.47	6.45E-04	1.23E-02
cg02431260	ESAM	11	Body	Island	22.38	2.88	24.86	2.87	-2.47	1.46E-03	2.26E-02
cg15059851	IVL	1	TSS1500	Open sea	68.52	5.02	71.00	3.49	-2.47	4.09E-04	8.67E-03
cg17086579	PITPNM1;PITPNM1	11	Body;Body	S_Shelf	85.47	3.92	87.94	2.20	-2.47	2.75E-03	3.52E-02
cg04961582		14	Intergenic	Island	22.26	3.59	24.73	3.40	-2.47	1.22E-03	1.98E-02
cg20971045		5	Intergenic	Open sea	43.87	4.72	46.35	5.33	-2.47	2.58E-03	3.38E-02
cg25438746		2	Intergenic	Open sea	67.90	3.41	70.38	4.20	-2.47	2.11E-03	2.93E-02
cg01801040	ZSWIM7;ZSWIM7	17	Body;Body	N_Shelf	71.80	4.01	74.27	4.74	-2.47	4.46E-03	4.92E-02
cg10996463	AGER;AGER	6	Body;Body	Open sea	77.58	3.33	80.05	2.87	-2.47	6.34E-04	1.22E-02
cg17953300	SIPA1;SIPA1	11	3'UTR;3'UTR	N_Shore	76.04	3.00	78.52	3.08	-2.47	1.94E-04	4.82E-03
cg22356726		10	Intergenic	Island	9.48	3.49	11.95	5.15	-2.47	2.51E-03	3.32E-02
cg24965791	CLDN10;CLDN10	13	TSS1500;TSS1500	N_Shore	77.09	3.95	79.56	3.91	-2.47	2.21E-03	3.03E-02
cg17153794	MACROD1	11	Body	N_Shelf	66.94	3.64	69.41	4.08	-2.47	1.43E-04	3.76E-03
cg11698099	PARD6A;ACD;ACD;PARD6A;ACD	16	Body;TSS1500;TSS1500;Body;TSS1500	Island	32.74	3.88	35.22	4.22	-2.47	3.92E-03	4.51E-02
cg05256504		7	Intergenic	S_Shore	63.04	4.16	65.51	4.00	-2.47	2.92E-03	3.68E-02
cg14921485		6	Intergenic	N_Shelf	85.17	4.86	87.65	4.74	-2.47	1.71E-03	2.53E-02
cg11309237		5	Intergenic	Open sea	55.65	4.25	58.12	3.73	-2.47	1.67E-03	2.49E-02
cg07786776		17	Intergenic	Island	25.50	4.89	27.98	5.15	-2.47	8.78E-04	1.55E-02
cg20713492	AQP10	1	TSS200	Open sea	46.05	4.17	48.52	3.91	-2.47	1.80E-03	2.62E-02
cg21766141	SOX5	12	5'UTR	Open sea	77.95	3.43	80.43	3.30	-2.47	1.25E-03	2.02E-02
cg13393009	CHRNB2	1	Body	S_Shore	30.05	2.60	32.52	3.50	-2.47	1.31E-03	2.09E-02
cg10425861		15	Intergenic	N_Shore	16.78	3.11	19.25	2.82	-2.47	5.04E-04	1.02E-02
cg00345443		3	Intergenic	Island	12.76	3.12	15.23	3.80	-2.47	1.97E-03	2.80E-02
cg09741851		10	Intergenic	Open sea	85.42	2.69	87.89	2.63	-2.47	3.98E-05	1.28E-03
cg25224369		3	Intergenic	S_Shore	82.68	3.52	85.15	2.45	-2.47	1.88E-04	4.70E-03
cg25575520		5	Intergenic	S_Shelf	85.95	4.26	88.42	3.73	-2.47	1.31E-03	2.09E-02
cg10867456		4	Intergenic	Open sea	74.33	3.20	76.80	4.25	-2.47	2.97E-03	3.71E-02
cg10157715		17	Intergenic	Island	11.71	2.23	14.18	2.44	-2.47	6.80E-06	2.65E-04
cg17960769	C1orf113;C1orf113;C1orf113	1	5'UTR;1stExon;Body	Island	14.88	3.27	17.35	4.28	-2.47	1.50E-03	2.30E-02
cg04678141	SVIL;SVIL	10	TSS200;5'UTR	Open sea	82.76	4.52	85.23	3.41	-2.47	1.25E-04	3.37E-03
cg12830180		18	Intergenic	S_Shore	84.65	3.76	87.12	2.62	-2.47	2.80E-04	6.44E-03
cg24375399	PLEKHH3	17	Body	N_Shelf	34.98	4.40	37.45	3.83	-2.47	3.56E-03	4.21E-02
cg00339687		1	Intergenic	Open sea	44.74	2.99	47.21	5.39	-2.47	3.04E-03	3.78E-02
cg03804556	SGCG	13	TSS1500	Open sea	39.67	3.25	42.14	4.31	-2.47	2.14E-03	2.97E-02
cg21752211		7	Intergenic	N_Shore	23.28	4.07	25.75	4.58	-2.47	1.58E-03	2.39E-02
cg05496514		5	Intergenic	Island	27.95	2.76	30.42	3.09	-2.47	8.07E-05	2.34E-03
cg22798925	GNAS;GNAS;GNAS;GNAS	20	3'UTR;TSS200;3'UTR;Body	Island	43.82	2.75	46.29	3.03	-2.47	1.57E-04	4.07E-03
cg15724876	TGFBR2;TGFBR2	3	Body;Body	Open sea	87.17	3.26	89.64	2.07	-2.47	1.24E-04	3.35E-03
cg24540281	TGFBRAP1;TGFBRAP1	2	Body;Body	Open sea	85.81	3.75	88.28	3.45	-2.47	5.37E-04	1.07E-02
cg05080966	DGAT1	8	Body	N_Shore	12.39	3.51	14.86	3.72	-2.47	4.16E-03	4.69E-02

cg13734137	RGS3;RGS3;RGS3	9	Body;Body;Body	Open sea	74.36	3.45	76.83	3.94	-2.47	2.57E-03	3.36E-02
cg22867154	LOC729234	2	Body	S_Shore	71.01	3.52	73.48	3.64	-2.47	2.69E-03	3.48E-02
cg13635014		9	Intergenic	N_Shore	89.30	3.14	91.77	2.36	-2.47	2.21E-06	9.41E-05
cg26443127		10	Intergenic	Open sea	64.77	4.20	67.24	3.38	-2.47	1.32E-03	2.10E-02
cg27142125	EXPH5;EXPH5;EXPH5	11	Body;5'UTR;Body	Open sea	66.77	3.42	69.24	3.98	-2.47	2.63E-03	3.42E-02
cg20871346		6	Intergenic	Open sea	86.11	3.53	88.58	3.46	-2.47	4.53E-04	9.40E-03
cg16003913	MPG;MPG;MPG;MPG	16	5'UTR;TSS1500;1stExon;TSS1500	N_Shore	49.63	4.17	52.10	3.69	-2.47	2.85E-03	3.61E-02
cg11846226	TNFAIP8	5	Body	Open sea	61.84	2.58	64.31	2.90	-2.47	1.75E-05	6.22E-04
cg01675895	AFM	4	TSS1500	Open sea	89.45	3.19	91.92	1.74	-2.47	1.26E-05	4.65E-04
cg09326440	HLA-E	6	Body	Island	61.72	4.38	64.18	5.44	-2.47	2.44E-03	3.25E-02
cg02085294	SLC1A4;SLC1A4	2	Body;5'UTR	S_Shelf	84.22	3.32	86.69	3.75	-2.47	1.27E-03	2.04E-02
cg26366091	CHI3L2;CHI3L2	1	TSS200;TSS200	Open sea	37.54	5.04	40.00	4.48	-2.47	3.96E-03	4.53E-02
cg07421737	TCIRG1;TCIRG1	11	Body;Body	Open sea	85.81	2.77	88.28	2.10	-2.47	7.34E-06	2.84E-04
cg02902261	TBL2	7	Body	N_Shore	84.48	4.00	86.95	3.62	-2.47	6.16E-04	1.19E-02
cg25040850	ELAC1	18	Body	Open sea	81.16	3.89	83.63	3.51	-2.47	1.41E-03	2.21E-02
cg02994291	MAPK8IP3;MAPK8IP3	16	Body;Body	Open sea	86.45	3.38	88.92	2.11	-2.47	4.02E-04	8.55E-03
cg11320438	SIGLEC11;SIGLEC11	19	Body;Body	Open sea	60.56	4.37	63.03	3.67	-2.47	2.80E-03	3.57E-02
cg10071442	EHMT2;EHMT2	6	Body;Body	S_Shelf	62.36	4.33	64.83	4.18	-2.47	7.38E-04	1.36E-02
cg11731793		10	Intergenic	Open sea	82.94	3.31	85.41	3.30	-2.47	5.07E-04	1.02E-02
cg20486569	CLDN5;CLDN5;CLDN5	22	1stExon;5'UTR;5'UTR	Island	19.61	3.65	22.07	3.72	-2.47	6.91E-05	2.05E-03
cg26435572	HOXA11AS;HOXA11	7	TSS1500;Body	N_Shore	12.42	2.43	14.88	3.15	-2.47	4.17E-05	1.33E-03
cg18975055	ARRB1;ARRB1	11	3'UTR;3'UTR	Open sea	60.40	3.19	62.87	2.82	-2.47	1.18E-03	1.94E-02
cg06432655	CLIP3	19	5'UTR	Island	34.45	3.84	36.92	3.80	-2.47	2.27E-03	3.09E-02
cg22255288	RPTOR;RPTOR	17	Body;Body	N_Shelf	80.11	3.87	82.57	3.16	-2.47	2.07E-03	2.90E-02
cg06937549	HNRNPH1	5	Body	N_Shelf	15.79	3.18	18.25	3.34	-2.47	2.22E-03	3.04E-02
cg19782190	CDC42BPB	14	Body	Open sea	79.27	4.14	81.73	3.48	-2.47	3.95E-03	4.53E-02
cg02768912	SKI	1	Body	S_Shelf	87.26	3.59	89.73	3.28	-2.47	2.13E-03	2.96E-02
cg01849970	FCHSD1	5	TSS1500	S_Shore	14.03	3.40	16.50	5.09	-2.47	1.23E-03	2.00E-02
cg14556762		1	Intergenic	Open sea	67.39	5.56	69.86	6.12	-2.47	3.17E-03	3.89E-02
cg06356912	VILL	3	Body	N_Shore	33.45	5.04	35.92	5.45	-2.47	2.48E-03	3.28E-02
cg26847834		5	Intergenic	Open sea	72.75	4.55	75.22	4.76	-2.47	3.57E-03	4.22E-02
cg25605360	KIAA0146	8	Body	Open sea	77.29	2.89	79.76	2.86	-2.47	8.18E-04	1.47E-02
cg08299809	HPCAL1;HPCAL1	2	5'UTR;5'UTR	Open sea	82.83	3.43	85.30	2.51	-2.47	2.16E-03	2.98E-02
cg21574064	ADAMTS10	19	Body	S_Shore	84.82	3.60	87.29	3.45	-2.46	4.54E-04	9.41E-03
cg07529884		9	Intergenic	Island	14.79	3.31	17.26	3.64	-2.46	1.18E-03	1.94E-02
cg15013982	LRRC56	11	Body	S_Shelf	79.38	3.28	81.85	2.62	-2.46	9.34E-05	2.65E-03
cg05779441		1	Intergenic	Open sea	40.16	4.11	42.62	4.56	-2.46	5.63E-04	1.11E-02
cg12893428	SDHAP1	3	TSS1500	S_Shore	87.69	4.06	90.15	2.93	-2.46	1.84E-03	2.67E-02
cg09779044	CKAP5;CKAP5	11	5'UTR;5'UTR	N_Shelf	84.01	2.23	86.47	1.67	-2.46	4.41E-07	2.07E-05
cg00438284	THBS2	6	Body	S_Shelf	70.23	4.14	72.69	4.21	-2.46	1.51E-03	2.32E-02
cg04387952	NOTCH1	9	Body	N_Shore	80.23	3.85	82.69	3.77	-2.46	1.41E-03	2.20E-02
cg22664798	C10orf93	10	Body	Open sea	74.91	3.51	77.37	4.41	-2.46	1.21E-03	1.97E-02
cg18639129	C3orf21	3	Body	Open sea	76.17	3.73	78.63	3.06	-2.46	1.22E-03	1.99E-02
cg18224077	LOC399959	11	Body	Open sea	37.97	3.54	40.43	4.37	-2.46	1.12E-03	1.86E-02
cg12009856	ACBD7	10	TSS1500	S_Shore	73.17	5.08	75.63	4.25	-2.46	9.39E-04	1.63E-02
cg13535626	ZNF876P	4	TSS200	N_Shore	9.34	2.40	11.80	2.97	-2.46	4.94E-05	1.54E-03
cg09088448		6	Intergenic	Open sea	82.16	4.56	84.62	3.35	-2.46	1.57E-03	2.38E-02
cg19561908		19	Intergenic	Island	84.71	3.72	87.17	3.09	-2.46	1.51E-03	2.31E-02
cg22867719	IFFO2	1	Body	Open sea	18.54	3.62	21.00	3.68	-2.46	2.23E-03	3.05E-02
cg12765028		4	Intergenic	Island	30.59	2.70	33.05	2.83	-2.46	1.19E-04	3.23E-03
cg03952724	RBPMS;RBPMS;RBPMS;RBPMS	8	Body;Body;Body;Body	Open sea	84.75	3.84	87.21	3.58	-2.46	2.25E-03	3.07E-02
cg08726248	DRD4	11	TSS1500	Island	16.32	4.31	18.78	4.78	-2.46	2.59E-03	3.39E-02
cg01366354		16	Intergenic	N_Shelf	67.80	3.35	70.26	3.65	-2.46	1.52E-03	2.33E-02

cg12422351		1	Intergenic	Open sea	65.74	5.14	68.20	5.00	-2.46	3.32E-03	4.02E-02
cg23817343	CSNK2B	6	Body	S_Shelf	74.94	2.80	77.40	2.89	-2.46	1.15E-05	4.26E-04
cg23779993	LRP5	11	Body	Open sea	79.27	4.37	81.73	3.70	-2.46	6.44E-04	1.23E-02
cg26011364	RFX8	2	TSS200	S_Shore	24.77	4.61	27.23	4.39	-2.46	5.36E-04	1.07E-02
cg00955904	STX6	1	TSS1500	S_Shore	62.09	3.49	64.55	3.14	-2.46	1.01E-03	1.73E-02
cg22781942	C12orf75	12	Body	Open sea	85.38	3.76	87.85	2.46	-2.46	1.21E-03	1.97E-02
cg21152628	TMEM132A;SLC15A3;SLC15A3;TMEM	11	3'UTR;3'UTR;Body;3'UTR	S_Shelf	74.90	3.61	77.36	3.10	-2.46	2.66E-03	3.45E-02
cg26069745	HOXA2;HOXA2	7	1stExon;5'UTR	N_Shore	22.18	3.25	24.64	3.05	-2.46	4.94E-04	1.00E-02
cg04745673		12	Intergenic	Island	6.18	2.28	8.64	2.03	-2.46	3.56E-06	1.46E-04
cg11667020	NKX2-4;NKX2-4	20	5'UTR;1stExon	Island	7.47	2.34	9.93	3.17	-2.46	9.85E-05	2.76E-03
cg14081667		21	Intergenic	Island	56.03	4.87	58.49	4.83	-2.46	4.57E-03	4.99E-02
cg15979173	BARHL2	1	1stExon	Island	28.89	3.38	31.35	4.19	-2.46	3.69E-03	4.31E-02
cg00077898	LOC285768;LOC285768	6	Body;Body	Open sea	92.77	2.96	95.23	1.38	-2.46	1.26E-07	6.25E-06
cg00253339	MVP;MVP	16	Body;Body	Island	84.17	4.48	86.63	3.79	-2.46	4.08E-03	4.63E-02
cg24007445		3	Intergenic	Open sea	88.31	4.69	90.77	3.08	-2.46	3.29E-03	3.99E-02
cg27624769	TRPS1	8	5'UTR	N_Shelf	74.38	3.78	76.84	3.34	-2.46	1.66E-03	2.48E-02
cg08012156	PMS2L5;PMS2L5;STAG3L2;PMS2L5	7	TSS1500;TSS1500;5'UTR;TSS1500	N_Shore	57.74	3.58	60.20	3.47	-2.46	3.50E-03	4.16E-02
cg05626073	AP2A2	11	Body	N_Shelf	67.31	5.08	69.77	4.53	-2.46	1.70E-03	2.52E-02
cg07101029	TSC2;TSC2;TSC2	16	Body;Body;Body	N_Shore	84.53	3.74	86.99	2.60	-2.46	1.71E-03	2.53E-02
cg25798341	TAP2	6	Body	Open sea	88.48	2.94	90.94	2.57	-2.46	3.15E-05	1.04E-03
cg08822360		10	Intergenic	Island	9.70	3.12	12.16	4.13	-2.46	1.60E-03	2.41E-02
cg16094352	REC8;REC8	14	Body;Body	S_Shore	79.80	3.91	82.26	4.51	-2.46	3.57E-03	4.22E-02
cg15705054	PBXIP1	1	5'UTR	Open sea	29.46	2.98	31.92	4.34	-2.46	1.17E-04	3.20E-03
cg24353651	PLSCR5	3	TSS1500	Open sea	54.30	2.13	56.76	2.61	-2.46	6.84E-05	2.04E-03
cg21962835	PBX1	1	Body	Open sea	82.65	3.97	85.11	3.39	-2.46	1.75E-04	4.44E-03
cg08507178	PIM3	22	3'UTR	S_Shore	18.88	3.02	21.34	3.62	-2.46	3.91E-03	4.50E-02
cg12658012		1	Intergenic	Open sea	72.93	3.77	75.39	3.05	-2.46	2.97E-03	3.72E-02
cg12659883		1	Intergenic	Island	18.10	3.33	20.56	3.81	-2.46	9.06E-04	1.59E-02
cg11505855		7	Intergenic	Open sea	89.47	3.11	91.93	1.87	-2.46	1.33E-04	3.54E-03
cg08275454	SLC44A2	19	Body	N_Shelf	86.60	3.39	89.06	2.57	-2.46	8.10E-05	2.35E-03
cg14575656		6	Intergenic	Open sea	41.29	3.83	43.75	4.55	-2.46	3.45E-03	4.12E-02
cg14098681	FLJ45983;GATA3;GATA3;GATA3;GATA	10	TSS1500;5'UTR;1stExon;5'UTR;1stExon;TS!	Island	12.18	3.15	14.64	3.73	-2.46	2.30E-05	7.90E-04
cg01481989	FRZB	2	1stExon	Island	32.05	5.22	34.51	6.17	-2.46	4.48E-03	4.93E-02
cg20863756	C12orf53;C12orf53	12	5'UTR;1stExon	Island	21.46	3.34	23.92	3.41	-2.46	5.54E-05	1.70E-03
cg06621132		4	Intergenic	Open sea	17.94	2.24	20.39	3.23	-2.46	3.82E-04	8.22E-03
cg05411056		13	Intergenic	S_Shelf	89.14	2.89	91.60	2.02	-2.46	1.30E-06	5.75E-05
cg20862447		2	Intergenic	Open sea	87.93	2.79	90.39	2.19	-2.46	4.67E-05	1.47E-03
cg27047255	PIK3C2B	1	Body	Open sea	85.07	3.13	87.53	2.36	-2.46	7.37E-05	2.16E-03
cg26678970		13	Intergenic	S_Shore	18.34	3.11	20.80	3.31	-2.46	1.64E-04	4.22E-03
cg15210307	KHDRBS1	1	Body	Open sea	82.47	4.69	84.93	3.52	-2.46	3.53E-03	4.19E-02
cg02596107	DAND5	19	TSS1500	Open sea	87.09	3.60	89.55	2.56	-2.46	1.22E-03	1.99E-02
cg02364831		16	Intergenic	Open sea	20.39	3.36	22.84	3.33	-2.46	2.84E-03	3.60E-02
cg16142218	CHMP7;CHMP7	8	1stExon;5'UTR	N_Shelf	66.42	4.22	68.87	4.04	-2.46	3.55E-04	7.77E-03
cg19780571	CPN1	10	Body	S_Shelf	85.23	4.13	87.69	3.86	-2.46	2.61E-03	3.40E-02
cg05750078	ADARB2	10	Body	Open sea	83.54	4.24	85.99	3.80	-2.46	9.16E-04	1.61E-02
cg04311964	LYPD2;LYPD2	8	1stExon;5'UTR	Open sea	78.61	3.41	81.07	3.70	-2.46	8.94E-05	2.55E-03
cg00338002	C1orf38;C1orf38;C1orf38	1	TSS1500;TSS1500;TSS1500	N_Shore	48.59	4.99	51.05	5.18	-2.46	3.41E-03	4.09E-02
cg22629987	QPCT	2	TSS200	Island	14.14	2.72	16.59	4.04	-2.46	1.72E-03	2.53E-02
cg15440402	KTN1;KTN1;KTN1;KTN1	14	5'UTR;5'UTR;5'UTR;5'UTR	Open sea	84.26	4.13	86.71	3.29	-2.46	3.87E-03	4.46E-02
cg00358023		7	Intergenic	S_Shore	79.31	4.02	81.76	3.10	-2.46	2.16E-03	2.98E-02
cg22489321		13	Intergenic	S_Shore	19.01	3.13	21.47	2.72	-2.45	2.86E-04	6.56E-03
cg08166750	EPS8L1;EPS8L1	19	Body;Body	Island	10.62	2.42	13.07	4.31	-2.45	1.94E-04	4.82E-03
cg22649800	OGDH;OGDH	7	3'UTR;3'UTR	Open sea	76.72	4.15	79.17	3.72	-2.45	3.78E-03	4.39E-02

cg02712165	C17orf76;C17orf76	17	TSS1500;TSS1500	S_Shore	82.31	3.72	84.77	4.14	-2.45	3.91E-03	4.50E-02
cg13771376	DDX31;GTF3C4;DDX31	9	TSS1500;Body;TSS1500	S_Shore	85.85	3.68	88.30	3.03	-2.45	9.82E-04	1.69E-02
cg05530568	EBF1;EBF1	5	1stExon;5'UTR	N_Shore	14.75	3.57	17.20	3.74	-2.45	1.77E-03	2.60E-02
cg20050761	MEST;MEST;MEST;MESTIT1	7	5'UTR;TSS200;5'UTR;TSS1500	Island	43.73	2.78	46.19	3.56	-2.45	1.40E-03	2.20E-02
cg21627409	PCDHGA4;PCDHGA11;PCDHGA11;PCD	5	Body;Body;Body;Body;Body;Body;Body;TS	N_Shore	8.96	2.58	11.42	3.47	-2.45	3.16E-04	7.09E-03
cg21610164	HIST1H2BI	6	1stExon	Island	19.89	3.05	22.35	3.05	-2.45	8.05E-05	2.33E-03
cg15229773	PATE2	11	TSS1500	Open sea	67.03	4.02	69.48	3.49	-2.45	1.49E-03	2.29E-02
cg10791342	PLTP;PLTP	20	Body;Body	N_Shelf	79.79	3.71	82.25	3.09	-2.45	2.55E-03	3.35E-02
cg13951491	HPDL	1	1stExon	Island	37.84	3.22	40.29	3.39	-2.45	1.28E-04	3.42E-03
cg18530748	SULT1A1;SULT1A1	16	5'UTR;1stExon	Island	10.61	4.06	13.06	4.27	-2.45	9.18E-04	1.61E-02
cg09861373	RAP1GAP;RAP1GAP	1	5'UTR;5'UTR	Island	9.10	2.94	11.55	3.80	-2.45	1.30E-04	3.47E-03
cg12136772	SMPD3	16	5'UTR	Island	27.08	4.14	29.53	4.76	-2.45	3.43E-03	4.11E-02
cg11283429		13	Intergenic	N_Shore	20.28	3.13	22.73	4.12	-2.45	3.62E-03	4.26E-02
cg09560096	BCS1L;BCS1L	2	Body;Body	S_Shelf	75.01	3.57	77.46	3.43	-2.45	8.92E-04	1.57E-02
cg21785710	C2orf86;C2orf86	2	Body;Body	Open sea	81.12	3.68	83.57	3.41	-2.45	1.85E-03	2.68E-02
cg08288074		11	Intergenic	Open sea	48.55	4.23	51.00	4.53	-2.45	2.98E-03	3.72E-02
cg25303023		6	Intergenic	Open sea	80.84	2.56	83.29	3.05	-2.45	1.71E-05	6.07E-04
cg26204328	C1QTNF9B;PCOTH;PCOTH	13	Body;Body;5'UTR	S_Shore	84.80	3.62	87.25	2.47	-2.45	4.68E-04	9.63E-03
cg00624589		16	Intergenic	N_Shelf	89.77	3.58	92.22	2.47	-2.45	9.26E-04	1.62E-02
cg16203491		13	Intergenic	Island	10.26	2.45	12.71	3.27	-2.45	2.89E-04	6.61E-03
cg21244038	RASGRP3;RASGRP3;RASGRP3	2	5'UTR;5'UTR;TSS1500	Open sea	82.47	5.46	84.92	3.60	-2.45	3.61E-03	4.26E-02
cg10306963		16	Intergenic	Open sea	80.05	5.19	82.50	4.04	-2.45	4.04E-03	4.60E-02
cg22803285	TMEM211	22	TSS1500	Open sea	39.98	3.39	42.43	3.66	-2.45	1.62E-03	2.43E-02
cg01255465	BAT3;BAT3;BAT3;BAT3	6	5'UTR;5'UTR;5'UTR;5'UTR	N_Shore	18.87	3.85	21.32	3.80	-2.45	2.29E-03	3.11E-02
cg24441324	ADAMTSL4;ADAMTSL4	1	5'UTR;5'UTR	S_Shore	71.77	3.66	74.22	3.22	-2.45	4.57E-03	5.00E-02
cg03761952	DSG4;DSG4;DSG4;DSG4	18	1stExon;1stExon;5'UTR;5'UTR	Open sea	87.72	4.67	90.17	2.40	-2.45	3.61E-03	4.26E-02
cg00449812		16	Intergenic	N_Shore	60.82	3.20	63.27	3.33	-2.45	3.10E-03	3.83E-02
cg17631454		4	Intergenic	Open sea	80.75	4.20	83.20	2.90	-2.45	3.01E-03	3.75E-02
cg14645841		12	Intergenic	Open sea	74.00	3.58	76.45	4.35	-2.45	3.11E-03	3.84E-02
cg26919723		8	Intergenic	Open sea	77.84	3.93	80.29	3.93	-2.45	1.34E-04	3.56E-03
cg23151860	RNF6;RNF6;RNF6	13	Body;Body;Body	N_Shelf	87.82	3.43	90.27	2.44	-2.45	2.03E-04	5.00E-03
cg07392324		7	Intergenic	S_Shore	37.18	6.17	39.62	5.35	-2.45	2.14E-03	2.97E-02
cg11018337	FLJ45983;GATA3;GATA3;FLJ45983	10	TSS200;TSS1500;TSS1500;TSS200	Island	5.89	2.45	8.34	4.34	-2.45	8.82E-04	1.56E-02
cg04059146	MMP7	11	TSS1500	Open sea	83.63	3.28	86.08	3.83	-2.45	4.85E-04	9.89E-03
cg15006973	GJA4	1	5'UTR	S_Shore	66.32	3.30	68.77	3.05	-2.45	2.59E-03	3.39E-02
cg01265653		11	Intergenic	Open sea	72.26	3.35	74.71	3.08	-2.45	5.87E-04	1.15E-02
cg14319545		11	Intergenic	Open sea	42.96	2.09	45.41	2.43	-2.45	6.58E-05	1.97E-03
cg26645237		5	Intergenic	Open sea	72.55	3.93	74.99	4.59	-2.45	9.83E-04	1.69E-02
cg19255053	FAM129A	1	TSS1500	S_Shore	40.86	3.47	43.31	4.13	-2.45	4.09E-03	4.64E-02
cg03820795	SPNS3	17	TSS200	Open sea	52.45	4.40	54.90	4.79	-2.45	7.78E-04	1.42E-02
cg08271229	SKI	1	Body	S_Shore	51.05	3.94	53.50	4.50	-2.45	2.22E-03	3.04E-02
cg14615784	GPR150	5	TSS1500	Island	9.50	2.43	11.95	2.58	-2.45	1.43E-06	6.25E-05
cg26065223		6	Intergenic	Island	16.34	3.05	18.78	3.89	-2.45	4.42E-04	9.22E-03
cg16303478		10	Intergenic	Open sea	81.31	4.08	83.76	3.47	-2.45	1.15E-04	3.15E-03
cg24576425	GALNT5;GALNT5	2	1stExon;5'UTR	Open sea	12.36	2.22	14.81	3.95	-2.45	1.42E-03	2.22E-02
cg00886909	CFI	4	TSS1500	Open sea	89.82	2.31	92.26	1.54	-2.45	2.45E-06	1.03E-04
cg06416026	PPFIBP2	11	3'UTR	Open sea	86.60	2.66	89.05	1.62	-2.45	2.03E-05	7.08E-04
cg12716083	ABCF1;ABCF1	6	3'UTR;3'UTR	Open sea	68.55	4.08	70.99	3.10	-2.45	3.37E-03	4.06E-02
cg18712755	BAT2	6	Body	S_Shore	87.05	2.82	89.50	2.82	-2.45	8.10E-05	2.34E-03
cg08351100	FLJ32063	2	TSS1500	N_Shore	17.66	3.29	20.10	3.67	-2.45	4.72E-04	9.69E-03
cg00591225	WWC2	4	Body	Open sea	74.65	3.27	77.09	2.57	-2.45	2.45E-04	5.81E-03
cg05711886	MEG3;MEG3;MEG3	14	Body;Body;Body	Island	48.03	2.61	50.48	2.96	-2.45	8.54E-04	1.52E-02
cg16506290	STAU2;STAU2;STAU2;STAU2;STAU2;ST	8	Body;Body;Body;Body;Body;Body;5'UTR	Open sea	87.31	4.02	89.75	3.91	-2.45	6.42E-04	1.23E-02

cg02955100	EGFL8	6	5'UTR	N_Shore	76.91	2.86	79.36	2.28	-2.45	3.07E-04	6.92E-03
cg00379713	SLC37A2;SLC37A2	11	Body;Body	Open sea	68.69	4.17	71.13	4.12	-2.45	2.36E-03	3.17E-02
cg14077965	FERMT2;FERMT2;FERMT2	14	TSS1500;TSS1500;TSS1500	S_Shore	15.71	3.41	18.16	3.59	-2.45	4.16E-03	4.69E-02
cg00049047	GDNF	5	5'UTR	Island	12.95	3.04	15.40	3.77	-2.45	6.77E-04	1.28E-02
cg01582980	SP8;SP8	7	5'UTR;Body	Island	13.21	2.99	15.66	3.78	-2.45	1.23E-03	2.00E-02
cg08592305	MAST1	19	Body	Island	18.72	4.12	21.16	3.30	-2.45	3.83E-04	8.24E-03
cg11260097		7	Intergenic	Island	22.83	3.21	25.27	3.97	-2.45	4.28E-03	4.78E-02
cg06731730	NOB1	16	TSS200	Island	22.78	1.99	25.23	2.02	-2.45	1.29E-06	5.70E-05
cg27267227	SEZ6L2;SEZ6L2;SEZ6L2;SEZ6L2	16	Body;Body;Body;Body	Island	10.38	2.06	12.82	3.12	-2.45	5.96E-06	2.35E-04
cg26678383		1	Intergenic	Open sea	78.93	4.29	81.38	3.12	-2.44	1.17E-03	1.93E-02
cg07712598		2	Intergenic	Open sea	82.98	2.50	85.42	2.06	-2.44	2.23E-05	7.68E-04
cg02693150	C8orf38	8	Body	S_Shelf	80.35	3.44	82.80	3.16	-2.44	1.41E-03	2.21E-02
cg25843866		6	Intergenic	S_Shore	31.88	5.49	34.32	4.51	-2.44	3.62E-03	4.26E-02
cg08911275	HOXD13	2	TSS200	Island	13.13	3.31	15.57	3.66	-2.44	1.38E-04	3.65E-03
cg19014730	FKBP5;FKBP5;FKBP5;FKBP5	6	5'UTR;5'UTR;5'UTR;5'UTR	Open sea	65.97	4.67	68.42	5.41	-2.44	1.84E-03	2.67E-02
cg19570940		5	Intergenic	Open sea	10.28	2.16	12.73	3.13	-2.44	2.23E-05	7.70E-04
cg08816455	TRIM24;TRIM24	7	Body;Body	Open sea	63.93	2.98	66.38	3.04	-2.44	1.08E-03	1.81E-02
cg15969149		10	Intergenic	Open sea	73.32	4.16	75.76	3.26	-2.44	1.69E-03	2.51E-02
[truncated: 736,353 more chars]
